# Network analysis of human glaucomatous optic nerve head astrocytes

**DOI:** 10.1186/1755-8794-2-24

**Published:** 2009-05-09

**Authors:** Tatiana Nikolskaya, Yuri Nikolsky, Tatiana Serebryiskaya, Svetlana Zvereva, Eugene Sviridov, Zoltan Dezso, Eugene Rahkmatulin, Richard J Brennan, Nick Yankovsky, Sanjoy K Bhattacharya, Olga Agapova, M Rosario Hernandez, Valery I Shestopalov

**Affiliations:** 1 Vavilov Institute of General Genetics, Russian Academy of Sciences, 3 Gubkina Str, Moscow, Russia; 2 GeneGo Inc, 500 Renaissance Drive, Suite 106, St. Joseph, MI, 49085, USA; 3 Current address: Bascom Palmer Eye Institute Department of Ophthalmology, University of Miami Miller School of Medicine; 1638 NW 10th Avenue, Miami, FL 33136, USA; 4 Department of Molecular Biology and Biochemistry, University of Miami Miller School of Medicine, 1638 NW 10th Avenue, Miami, FL 33136, USA; 5 Department of Ophthalmology and Visual Sciences Washington University School of Medicine 660 South Euclid Ave, St Louis, MO 63110, USA; 6 Department of Ophthalmology, Feinberg School of Medicine, Northwestern University Chicago, IL 60611, USA; 7 Department of Cell Biology and Anatomy, University of Miami Miller School of Medicine, 1638 NW 10th Avenue, Miami, FL 33136, USA

## Abstract

**Background:**

Astrocyte activation is a characteristic response to injury in the central nervous system, and can be either neurotoxic or neuroprotective, while the regulation of both roles remains elusive.

**Methods:**

To decipher the regulatory elements controlling astrocyte-mediated neurotoxicity in glaucoma, we conducted a systems-level functional analysis of gene expression, proteomic and genetic data associated with reactive optic nerve head astrocytes (ONHAs).

**Results:**

Our reconstruction of the molecular interactions affected by glaucoma revealed multi-domain biological networks controlling activation of ONHAs at the level of intercellular stimuli, intracellular signaling and core effectors. The analysis revealed that synergistic action of the transcription factors AP-1, vitamin D receptor and Nuclear Factor-kappaB in cross-activation of multiple pathways, including inflammatory cytokines, complement, clusterin, ephrins, and multiple metabolic pathways. We found that the products of over two thirds of genes linked to glaucoma by genetic analysis can be functionally interconnected into one epistatic network via experimentally-validated interactions. Finally, we built and analyzed an integrative disease pathology network from a combined set of genes revealed in genetic studies, genes differentially expressed in glaucoma and closely connected genes/proteins in the interactome.

**Conclusion:**

Our results suggest several key biological network modules that are involved in regulating neurotoxicity of reactive astrocytes in glaucoma, and comprise potential targets for cell-based therapy.

## Background

Astrocyte activation is a hallmark of various CNS injuries and pathologies, including stroke, trauma, tumor, infection, and neurodegenerative diseases [1-3]. Upon activation, astrocytes display altered metabolism and the ability to preserve CNS homeostasis and support neuronal function. Reactive astrocytes were shown to reduce damage during the acute phase of CNS insults [4]. In contrast, progressive degenerative diseases, such as glaucoma, feature chronic astrocyte activation that exacerbates damage to neurons and impairs regeneration of their axons [5,6]. Importantly, a prominent astrocyte reactivation in primary open angle glaucoma (POAG) is localized to the optic nerve head, which is also the site of primary damage to the retinal ganglion cells (RGCs) [7].

In common with many other complex, age-related diseases, neurodegeneration in POAG is associated with a homeostatic imbalance resulting from environmental factors and multiple genetic components interconnected within complex epistatic networks [8]. Such imbalance is manifested at three interconnected functional levels: intercellular stimuli, intracellular signal transduction, and core effectors (i.e. endogenous metabolism, structural complexes, etc). Disease causes alterations at all three levels. These can be measured by high-content screens that include differential gene expression, proteomics and metabolomics, as well as in genetic linkage studies that connect genes or protein variants to disease onset [9,10]. Recent progress in systems biology has allowed a quantification, cross-comparison and functional interpretation of heterogeneous datasets within the framework of human biological pathways, networks and processes, which are assembled from a knowledgebase of functional biological interactions [10,11]. This systems level approach requires an understanding of connectivity between the genes and proteins affected in a given disease. Connectivity is defined by binary protein interactions with genes, proteins and biologically active compounds [12]. The biological networks are scale-free but converge in regulatory nodes and modules, such as major transcription factors and receptors [13,14]. Identification of such key topological elements [15,16] on the networks derived from disease-related data may reveal potential therapeutic targets. This approach is particularly powerful for diseases of complex etiology, such as glaucoma.

Regulation of astrocyte activation, which is associated with increased neurotoxicity, involves differential activation of key cellular network modules [17]. To perform *in silico *reconstructions of the cellular pathways affected during the development of glaucoma, however, data derived specifically from astrocytes must be used, rather than data derived from whole-tissue (retina or optic nerve) samples. It is feasible to suggest that the cell-specific data from interacting cell types, such as astrocytes and retinal ganglion cells, will allow us to analyze differences in trans-cellular crosstalk that are implicated in glaucoma. Here, we performed functional analysis of the signaling and effector networks [18] potentially implicated in the pathophysiology of glaucoma, using both small experimental and high-content differential data obtained from whole optic nerves and from reactive ONHAs isolated from the optic nerves of glaucomatous patients. We reasoned that the bulk of changes to the ONHA transcriptome would be disease-related, and subjected the data to meta-analysis at the level of affected biological processes, and at the level of biological network topology, using the MetaCore Analytical Suite [19,20]. This suite of analytical tools, linked to a comprehensive interaction and biological function database, is available as a stand-alone application and can be linked via a "plugin" to other pathway tools such as Cytoscape [21]. Biological networks were built starting from either gene expression data alone, or a combination of high content data sets reflecting multiple levels of information flow in the cell. Using a two-step procedure, we narrowed the set of differentially expressed genes, and merged it with genes associated in the literature with glaucoma, thus filling gaps in the fragmented, literature-derived genetic data. The final direct interaction network displayed a synergy between these two minimally-overlapping datasets, allowing broad characterization of pathological changes in reactive astrocytes, and defining network modules potentially implicated in the shift to neurotoxicity. Modules regulating such shift represent therapeutically valuable targets that can be further validated experimentally.

## Results

### Functional characterization of gene expression data

**Figure 1:**
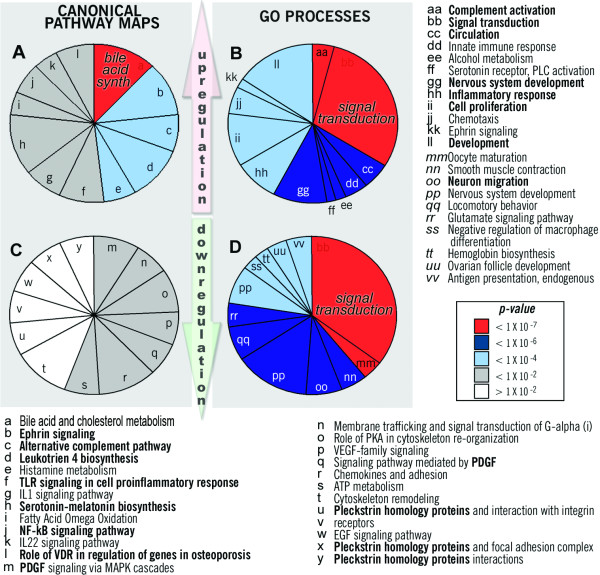
**The enrichment analysis of differential gene expression (microarray) data**. The distributions for the top twelve canonical maps and GO processes represented by the up-regulated (**A, B**) and down-regulated (**C, D**) genes in the combined data set. Individual sector areas reflect relative gene representation of each process/map among the top 12 shown. Processes and pathway maps were prioritized by p-value; the p-value range for maps and GO processes is color-coded. Bolded processes scored in the top 12 in both data sets.

Primary astrocytes were purified from the cadaver eye optic nerve tissue (see Additional File 1, Supplement Table S1 for clinical information of donor eyes) by two-step immunopanning procedure and expansion *in vitro *prior to analysis, as described [22,23]. A detailed protocol is provided in Methods. Astrocytes from both normal (ages *59 *± *13*) and glaucomatous (ages *69 *± *13*) eyes with mild, moderate or advanced (1 donor) axon loss were isolated, maintained and processed for analysis in a strictly similar manner so that cultivation-induced differences on gene transcription between cell lines would be minimized. Comparison of the two profiles (normal vs. glaucoma) will, therefore, cancel much of the cultivation-induced changes, allowing disease-imprinted ones to be identified. Thus, the gene expression data analysis performed here revealed the changes in the disease-imprinted ONHAs, an experimental strategy that is often implemented by other investigators working with human diseases [24,25]. Our analysis showed very significant correlation between transcriptomic changes observed in human and animal glaucoma; however, we have to admit that the influence of other factors on gene expression profile of primary cells cannot be fully excluded. Two separate sets of experimental data, were obtained from a total of seven normal and eight glaucomatous human donor eyes. A detailed description of the microarray analysis is given in Methods section, and in a previous publication [22]. We combined the data from donor Groups #1 and #2, which contained tissues from four glaucomatous and three or four normal eyes each (see details in Methods) into a non-redundant data set comprised of 461 up-regulated genes and 301 down-regulated genes (shown as AFFI IDs, see Additional File 2), and analyzed it along with individual data sets from each experimental series separately. There was a high abundance of genes with significant changes in the expression level between normal and glaucomatous ONHAs. We limited our analysis to those genes with the most significant changes in expression exceeding 2.5-fold differences in RNA abundance. Firstly, we ranked canonical pathways and gene ontology biological processes by the statistical significance of the overlap between their gene content and genes highly overrepresented in human glaucomatous astrocytes compared to normal astrocytes. In the enrichment analysis, we used the over-representation of the DE genes as a parameter to score the Gene Ontology (GO) functional folders and Metacore canonical pathway maps. The results were ranked by increasing the p-value (Figure 1; for the enrichment data on individual datasets see Additional File 1, Supplement Table S2).

### Activated processes and pathways

A major feature of astroglial reactivation following various CNS injuries is a profound activation of pro-inflammatory and stress response pathways [4,26-28]. Consistently, upregulated genes outnumbered downregulated ones by approximately 50%. Enrichment analysis of the combined DE data set from this study showed that highest scoring GO terms for up-regulated genes included complement activation, signal transduction, regulation of circulation, inflammatory and innate immune response, nervous system development and cell adhesion (Figure 1). Metabolic pathways were well-represented among up-regulated genes, particularly, those encoding three enzymatic functions (defines as EC numbers): EC 1.1.1.1 – alcohol dehydrogenases; EC 1.2.1.3 – aldehyde dehydrogenases; EC1.14.14.1 – oxidases, cytochromes P450s (Table 1). Analysis of the data specific for individual donor groups added cell-cell signaling and lipid metabolism (see Additional File 1, Supplement Table S2). The most over-represented GO process, complement activation, scored consistently high in all data sets examined with nine out of thirty five genes activated (p-value 2.5e-8). A network built using the "Shortest Path" (SP) algorithm (see Additional File 1, Supplement Table S3 and in [19]) to connect the genes in this process that changed, revealed highly-coordinated activation of several complement factors and regulatory genes, including complement factor H and clusterin (see Additional File 3, Supplement Figure S1). Next, we analyzed up-regulated genes associated with the GO process nervous system development (33 genes; p-value 4.1e-9). We used these genes as seed nodes to build process-specific networks using the SP algorithm: the network featured activation of ephrins, WNT regulatory proteins, pleiotropin, midkine, and GRO-I (see Additional File 3, Supplement Figure S2).

**Table 1:** Metabolic pathways activation revealed by gene expression data from glaucomatous ONHAs

					**Metabolic pathway maps**						
					
**Enzyme EC #**	**Gene**	**gene ID**	**fold change** (combined set)	**common protein name**	**BAB**	**RB**	**CM**	**VDP**	***LB***	**SMB**	**AT**
1.1.1.1.	*ADH1A*	**124**	**11.7**	Alcohol dehydrogenase 1A	**+**		**+**		**+**		

	*ADH1B*	**125**	**9.7**	Alcohol dehydrogenase 1B	**+**		**+**		**+**		

	*ADh1C*	**126**	**5.6**	Alcohol dehydrogenase 1C	**+**		**+**		**+**		

1.2.1.3.	*ALDH1A2*	**8854**	**2.2**	Aldehyde dehydrogenase(NAD+)	**+**				**+**	**+**	

	*ALDH1A3*	**220**	**5.1**	Aldehyde dehydrogenase 1A3					**+**	**+**	

	*ALDH3A2*	**224**	**2.6**	Aldehyde dehydrogenase 3A2	**+**				**+**	**+**	

1.2.1.5	*ALDH1A3*	**220**	**5.1**	retylaldehyde dehydrogenase 1A3		**+**					

1.3.1.20	*AKR1C1*	**1645**	**20.1**	aldo-keto reductase 1C1	**+**						

1.14.14.1	*CYP2D6*	**1565**	**2.3**	cytochrome P450 family 2D6		**+**	**+**		**+**		

	*CYP2C9*	**1559**	**2.3**	cytochrome P450 2C9		**+**	**+**		**+**		**+**

	*CYP1A1*	**1543**	**2**	cytochrome P450 family IA		**+**	**+**		**+**		**+**

	*CYP3A4*	**1576**	**1.8**	cytochrome P450 family IIIA4		**+**	**+**			**+**	**+**

	*CYP7A1*	**1581**	**2.9**	cytochrome P450 family 7A1	**+**						

	*CYP27A1*	**1593**	**2.4**	cytochrome P450C 27/25	**+**			**+**			

	*CYP27B1*	**1594**	**1.6**	calcidiol 1-monooxygenase				**+**			

2.1.1.-	*LCMT2*	**9836**	**2.3**	leucine carboxyl methyltransferase 2						**+**	

6.3.2.-	*MID1*	**4281**	**3.5**	midkine1						**+**	

Abbreviations: **BAB ***Bile acid biosynthesis*; **RB **– *Retinol biosynthesis; ***CM **– *Catecholamine metabolism; ***VDP **– *Vitamin D3 c23 and c24 pathways; ***LB **– *Leukotrien 4 biosynthesis; ***SMB **– *Serotonin-melatonin biosynthesis; ***AT **– *Androstenedion testosterone metabolism*

Significantly, mapping the combined activation data set onto canonical pathway maps revealed individual perturbed pathways within the larger GO categories. Affected pro-inflammatory pathways (maps) included, Complement activation, Signaling via ephrins, TOLL-like (TLR) receptors and IL-1. Nuclear Factor-kappaB (NF-kB) was among other top changed stress-response factors (Figure 1). Analysis of the mapped data suggested that increased activity of ephrin receptors in reactive ONHAs transduced signals through either PI3K (PIK3CG), intersectin (ITSN1) or Tiam1 (TIAM1) to RAC1 and CDC42, and through JNK3/MAPK10 to paxillin, and affected pathways related to cell migration and neurogenesis (see Additional File 3, Supplement Figure S3). The established role of these molecules in regulation of cell movement may explain the molecular control of de-differentiation and increased motility of ONHAs, which are characteristics of reactive astrocytes in general [4,28,29], and of glaucomatous ones in particular [1,3]. Activation of ephrin A4 receptor (EFNA4), which has been shown to inhibit axonal regeneration via repulsive signaling [30,31], facilitated neuronal death in optic nerve and spinal cord injury models [32]. In contrast, the up-regulation of IL-6, ephrins B1 and B2 in glaucomatous optic nerves was interpreted as neuroprotective [33,34]. Similar analysis of another top scoring map representing VDR-mediated signaling revealed a cross-talk between AP-1 subunit c-Fos (FOS), androgen receptor (AR), p57 (CDKN1C), osteopontin (SPP1) and cytochromes CYP27B1, CYP3A4 in glaucomatous ONHAs (See Additional File 3, Supplement Figure S4). Among metabolic maps, biosynthetic pathways for bile acid and cholesterol, serotonin-melatonin, leukotriene 4 and histamine biosynthesis were enriched (Table 2, also see Additional File 3, Supplement Figure S5) indicating alterations in lipid membrane biogenesis.

**Table 2:** Distribution of Gene Ontology (GO) processes for the largest Direct Interactions (DI) network of genes up-regulated in the combined data set

**GO process**	**number of DE genes**	**# genes in a pathway**	**p-value**
***upregulation, 196 nodes***			

Cellular physiological process	16	114	2.83e-11

Inflammatory response	23	269	4.26e-11

Regulation of transcription, DNA-dependent	51	1401	7.77e-09

Cell-cell signaling	23	351	7.89e-09

Llipid metabolism	17	197	1.44e-08

Alcohol metabolism	5	7	1.92e-08

Transcription	45	1182	1.97e-08

Positive regulation of cell proliferation	17	215	5.28e-08

Signal transduction	50	1493	1.65e-07

Cytokine and chemokine signaling	5	96	2.07e-07

***downregulation, 50 nodes***			

Cell-cell signaling	17	351	2.76E-13

Regulation of sodium ion transport	4	4	4.39E-10

Norepinephrine-epinephrine regulation of blood pressure	4	4	4.39E-10

Signal transduction	26	1493	7.02E-10

immune response	15	460	2.20E-09

Positive regulation of bone mineralization	4	6	6.54E-09

Transmembrane receptor protein tyrosine kinase activation	4	6	6.54E-09

Heat generation	4	6	6.54E-09

Negative regulation of smooth muscle contraction	4	6	6.54E-09

Norepinephrine-epinephrine vasodilation during regulation of blood pressure	4	6	6.54E-09

Overall, the enrichment analysis of transcriptional changes in glaucomatous ONHAs revealed significantly more activated than down-regulated pathways, which is consistent with the "re-activation" status of these cells. Transcriptional activation orchestrated by the transcription factors AP-1/c-FOS, VDR, and NF-kB, was modulated by increased activity of TLR, AR and ephrin receptors. Pro-inflammatory processes in these cells were mediated by an increased activity of TOLL-like receptors, NF-kB, MAPKs, JNK3 and complement system. While increased signaling via VDR, AP-1, IL-6 and AR in the optic nerve can be broadly interpreted as a cytoprotective, activation of ephrin A4 receptor and multiple pro-inflammatory pathways represent potentially cytotoxic events, which are conducive to oxidative stress and possibly harmful to CNS neurons and their axons. Significantly, enrichment analysis of activated genes across metabolic maps revealed alterations in cholesterol metabolism that correlated with increased sensitization of neurons to glutamate in glia-neuron co-cultures [35]. Similar changes in cholesterol metabolism were also detected in transcriptomic analysis of whole optic nerve head tissue in rat experimental glaucoma [36]. We compared published transcriptomic and biochemical studies in retina and optic nerves from different experimental glaucoma models. Importantly, the published data showed multiple similarities with our data from human ONHAs, particularly in the activation of innate and adaptive immune responses. Consistent with our results, animal models of glaucoma showed robust activation of pathways mediated by AP-1/c-Fos/c-Jun [5,37,38], NF-*κ*B [39], complement [36,40-42], androgen receptor [39,43] and ephrins [44]. Oxidative stress response and signaling via NF-*κ*B, AP-1 and TLRs have also been detected in transcriptomic studies of hydrostatic pressure-induced human ONHAs, and in cytokine-activated murine astrocytes [45,46]. Gene expression analysis of EGFR-stimulated rat ONHAs revealed additional commonalities in the activation of genes related to cell migration, ECM reorganization and immune response [47]. Major differences in the transcriptomic data derived from rodent astrocytes included a lack of activation of VDR-mediated signaling pathway. This effect may be specific to glaucomatous human ONHAs. These comparisons lead us to suggest that the signature profile of the glaucomatous process in reactive astrocytes includes chronic activation of AP-1, NF-kB, complement, AR, TLRs and cell adhesion pathways.

### Down-regulated pathways and processes

Analysis of down-regulated genes revealed a fewer number of significantly affected pathways. Perturbed pathways included PDGF signaling via MAPK cascades, membrane trafficking and signal transduction via G-*α*I (Figure 1, also see Additional File 3, Supplement Figure S6). We identified several levels of inhibitory regulation in these processes, including structural proteins, signaling complexes, cellular matrix proteases and intracellular signal transduction proteins. Down-regulation of many integrins (a total of twelve in the two donor groups, see Additional File 1 Supplement Table S4), evidenced profound changes in the adhesion state and cell signaling in glaucoma. Interactome analysis of the mapped data was performed to analyze connectivity between down-regulated pathways using the MetaCore network building tools and gene content from maps (69 genes found on the 12 most significantly down-regulated maps). Analysis of global connectivity in the networks using the Direct Interactions (DI) algorithm (see Additional File 1 Supplement Table S3) revealed integrins, TGF-*β*, XIAP, kinase families PKC and PI3K as critical hubs interconnecting a compact network (see Additional File 3, Supplement Figure S7A). Concerted inhibition of adhesion complexes (see Additional File 3, Supplement Figure S7B) is a likely explanation for the increased ONHA motility observed in glaucoma, and may contribute to a change in the physiological role of astroglia upon activation [3,48]. In good agreement with these findings, studies in an animal model of glaucoma also showed significant changes in the expression of ECM components [36], and a toxic decrease of PKC activity in retinal neurons [49]. However, results of individual studies on TGF-*β*, and PDGF activation in glaucoma [50] varied significantly, likely due to differences in the design, species and cellular populations examined.

### Networks for genes differentially regulated in glaucomatous ONHAs

**Figure 2:**
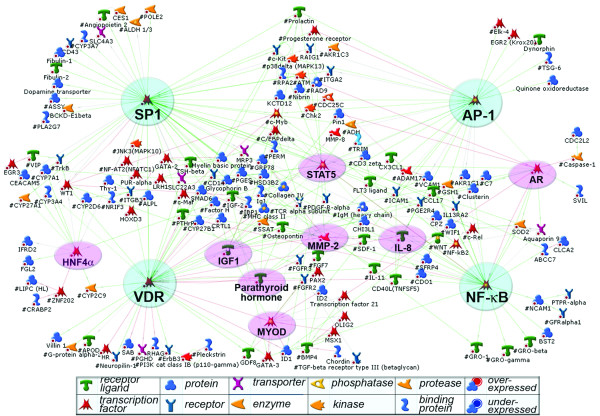
**A direct interactions (DI) "global" network for the genes up-regulated in glaucomatous ONHAs**. The network was generated using a subset of over-expressed (fold change >2.5) genes from the combined microarray data set as an input. The top four interconnected (>20 edges) hubs are marked with cyan circles, less connected (10–20 edges) with pink ovals. Green arrows, activation, red arrow, inhibition; short legend is shown below, for a complete legend see Additional File 1 Supplement Table S3.

We subsequently generated and analyzed merged signaling/metabolic networks from a combined set of 461 up-regulated and 301 down-regulated genes (see Additional File 2). A direct interaction (DI) network (see Methods for algorithm details) was built using the most stringent algorithm that connected only those differentially expressed genes that are functionally proximal and interact directly [51]. The largest DI network interconnecting up-regulated genes contained 196 nodes (Figure 2), while the largest network of down-regulated genes connected only 50 nodes (see high resolution images in Additional File 3, Supplement Figures S8 and S9). Both networks were classified according to their distribution of GO functional processes (Table 2). The likelihood of finding a DI cluster of 196 nodes from a random set of 461 genes is very low (p < 0.0001), reflecting the non-randomness of multiple activated pathways revealed in ONHAs by network analysis (see Methods for p-value calculation).

### Networks for genes with increased expression

Networks can be characterized by a variety of parameters depicting network topology [52] – distribution of nodes and hubs (top 25% of nodes by the number of interactions), transcription factors, surface receptors etc. [53]. Typically, hubs on human networks are transcription factors or membrane receptors, each forming a cluster (module) of direct targets and upstream regulators. Twelve major network hubs constitute the core of the DI network built from a subset of genes activated in glaucoma. The top hubs scored by combination of p-value and connectivity included SP1, VDR, NF-*κ*B, AP-1/FOS and AR (Table 3), most of which represent redox-sensitive transcription factors. Major membrane receptors affected in glaucoma included ICAM1 (up-regulated), PDGF-R*α *and CCR1 (down-regulated) (Table 4). The gene content for the five major up-regulated network modules is listed in Table 5.

**Table 3:** Top hubs differentially activated in the DI network

**network hubs**	**hub type**	**edges**	**in**	**out**
***up-regulated hubs***				

SP1	TF	58	7	53

VDR	TF	40	7	33

NF-kB	TF	32	5	27

AP1	TF	21	2	19

AR	TF	18	9	8

c-Fos	TF	19	10	9

IGF-1	growth factor	17	7	10

c-Myb	TF	14	7	7

STAT5B	TF	13	9	4

HNF4-alpha	TF	12	3	9

E2A	TF	12	4	8

IL8	cytokine	11	10	1

MMP2	protease	10	8	2

***down-regulated hubs***				

Interferon-gamma	growth factor	19	3	16

STAT1	TF	17	6	11

c-Myb	TF	6	0	6

PAI1	ligand	5	3	2

**Table 4:** Top differentially regulated membrane receptors

**membrane receptors**	**# edges**	**in**	**out**
***up-regulated receptors***			

ICAM1	8	7	1

c-Kit	8	3	5

FGFR2	5	5	0

ITGB3	5	5	0

CD14	2	2	0

ErbB3	2	1	1

IL13RA2	2	2	0

ITGA2	2	2	0

RAIG1	2	2	0

TCRalpha subunit	2	2	0

***down-regulated receptors***			

PDGF-R-alpha	5	5	0

CCR1	4	2	2

Galpha(q)-specific peptide GPCRs	4	4	0

Substance P receptor	3	2	1

Angiotensin II receptor, type-1	2	0	2

Beta-2 adrenergic receptor	2	1	1

Prostacyclin receptor	2	1	1

**Table 5:** Regulation of activated genes by transcription factors in the DI network

	**SP1**		**VDR**	**AP-1**	**NF-*κ*B**	**AR**
**Upstream regulation**	*MAPK13*		MYB*	*AR*	*AR*	*CASP1*
	*NFATC1*		*HR*	*EGR2*	*BST2*	*PIN1*
	*PRL*		*IGF1*	*ELK4*	STAT5*	*NFKB2*
	*PGR*		*PTH*	MAPK13*		*REL*
	*SAT*		*PRL*	*PTH*		*RAD9A*
	*STAT5*		*SP1*	*SP1*		*SVIL*
	*WT1*		*WT1*	STAT5*		*TCF21*
				VDR*		VDR*

**Down-stream targets**	*ADH1A*	*GPRC5A*	ALPL*	ALPL*	*AR*	*SOD2*
	*ADH1B*	*ICAM1*	*APOD*	ADH1A*	*AKR1C1*	*AP-I/JUND*
	*ADH1C*	*ID1*	*AR*	ADH1B*	*AQP9*	*AQP9*
	*ALDH1A1*	*IGF2*	CD14*	ADH1C*	CEBPD *	*IGF1*
	*ALDH1A2*	*ITGA2*	*CRABP2*	*AKR1C1*	*CCL17*	*REL*
	*ALDH1A3*	*ITGB3*	*CD40L*	CYP27B1*	*CD40L*	SAT *
	*ALDH3A2*	*KIT*	*CYP3A4*	ICAM1*	*CLU*	
	*ALPL*	*MMP2*	CYP27B1*	*CLU*	*CDO1*	
	*ANGPT2*	*MPO*	*CYP2C9*	*CRYZ*	*CDN1A*	
	*ASS*	MYB *	*IGF1*	*EGR2*	CYP27B1*	
	*ATM*	*OPRM1*	IGF2*	*ELK4*	*GATA3*	
	*BCKDHB*	*PLAT*	*GDF8*	*GCLC*	CXCL1*	
	*CEBPD*	*POLE2*	*GATA3*	*GPRC5A*	*CXCL3*	
	*CD14*	*PTGES*	*HLA-DQA2*	*ID2*	ICAM1*	
	*CD43*	*PTHLH*	*ID1*	*IL13RA2*	*GCLC*	
	*CES1*	*PURA*	*IL11*	*IL8*	*IL13RA2*	
	*CHI3L1*	*SLC4A3*	*MAF*	ITGA2*	***IL8***	
	*CXCL1*	*SLC6A3*	***IL8***	*MMP8*	*HLA-DQA2*	
	***CYP27B1***	*SOD2*	***MYB***	MYB *	MYB *	
	*CYP3A7*	*SPP1*	*MYOD*	*PDYN*	*NCAM1*	
	*FBLN1*	*STAT5*	*NRP1*	PGR*	SPP1*	
	*FBLN2*	*VDR*	*PTGDS*	PRL*	PGES*	
	*FOS*		PTH*	*TNFAIP6*	SOD2*	
	*HNF4A*		PTHLH*	*VCAM1*	*VCAM1*	
	*GYPB*		*RHAG*		*WNT2B*	
			*SH3BP5*		*CXCL2*	
			*SLC22A3*			
			SPP1*			
			*VIL1*			

*Co-regulated by SP1

Co-regulated by NF-*κ*B & AP-1 & VDR

Co-regulated by NF-*κ*B & AP-1

**Figure 3:**
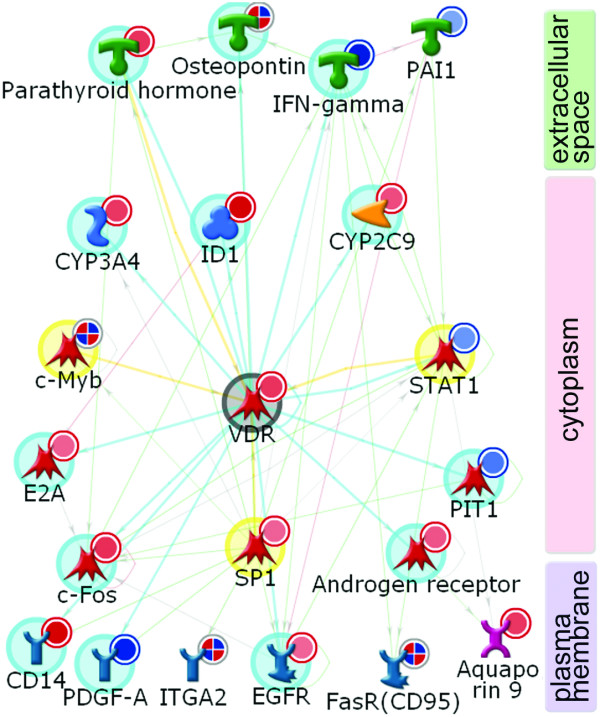
**A network for the VDR-regulated genes affected in glaucoma**. The gene expression data for the combined data set (2.5 fold change) is mapped on the network. The network was generated using the analyze network (AN) algorithm. Network objects are vertically stratified according to their cellular localization, which is indicated on the right. VDR edges highlight color code: yellow, the upstream regulators; cyan, the downstream targets.

Transcription factor SP1 forms the largest module with 55 edges, including 48 downstream target genes (Table 3). This is not unexpected, as SP1 is one of the most prolific general transcription factors in human cells, and co-activates a variety of genes in many different conditions [54]. The second largest cluster was formed by the vitamin D receptor (VDR), 40 edges, 33 targets activated. VDR is a key nuclear receptor controlling transcriptional activation of multiple genes in response to inflammation, oxidative stress and cancer in CNS [55]. Unlike SP-1, activation of VDR is a less common event, perhaps more specific for glaucomatous astrocytes. Other CNS pathologies affecting glia and showing VDR over-expression include astrocytomas, gliomas [56] and reactive astrocytosis in mice [45]. The neuroprotective and immunomodulatory effects of VDR activation have been shown to facilitate recovery after oxidative stress [57], achieved through tight control of oxidative stress responsive genes and inhibition of iNOS (NOS2A). In the local differentially-expressed gene network for glaucomatous ONHAs, VDR activation is triggered by an increase in activity of the upstream regulators MYB (c-Myb), SP-1 and parathyroid hormone (Figure 3). AP-1 (21 edges, 19 activated targets) is a major transcription factor complex that has been implicated in regulation of cell proliferation, differentiation, survival and death in response to physiological stimuli and environmental factors [58]. Simultaneous up-regulation of the AP-1 subunits c-Jun (JUN), JunD (JUND) and c-Fos (FOS) (the subunits are merged into an "AP-1" complex to simplify Figure 2) in glaucomatous ONHAs have translated into strong activation of a subset of AP-1 regulated targets shown in Table 5. Co-activation of transcription factors AP-1 and NF-*κ*B in glaucomatous ONHAs is consistent with their parallel regulation via TLR receptors and MyD88 [59-62], and a synergistic role in promoting cell response and survival to various stresses [63]. Importantly, the gene encoding GATA-3, a transcriptional regulator of TLR2, was elevated in glaucomatous ONHAs. The activation of AP-1/c-Fos/c-Jun complex has been well characterized recently in primate model of glaucoma [5].

**Figure 4:**
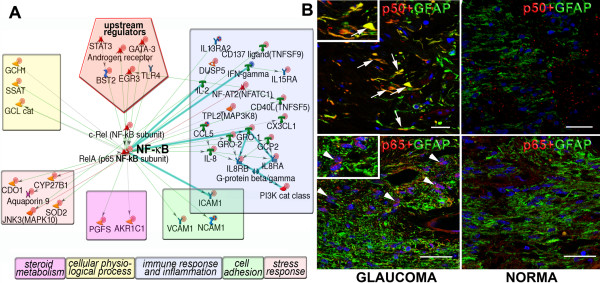
**Activation of the NF-*κ*B module in the ONHA-specific disease network**. **A**. An AN network for upregulated genes connected to the NF-*κ*B regulated cluster. The network objects regulated by NF-*κ*B are grouped according to the GO processes, and color-coded as shown in the boxes below. The color density of red indicator circles reflects the degree of up-regulation. See Figure 2 and see Additional File 1, Supplement Table S3 for full legend. **B**. Immunohistochemistry data indicated increased accumulation of both p50 (arrows) and nuclear translocation of phospho-p65 (arrowhead) subunits of the NF-kB heterodimer in glaucomatous ON tissue. Boxed inserts in left upper corners represent high-maginification images of cells showing activated NF-kB subunits. Astrocytes are stained for GFAP (green), nuclei for DAPI (blue), size bars= 50 *μ*m.

We compared relative connectivity (experimental vs. expected) of the top five transcription factors and within the list of activated genes (2.5 fold) using the MetaCore Interactome characterization tool ([64] see also description in -Methods). Although NF-kB forms only the third largest module (32 edges, 27 activated targets) in the network, interactome analysis showed it as the most over-connected one within activated data set (see Additional File 1, Supplement Table S5). Combined with a well-defined role in regulation of inflammation [2,63-66], responses to stressors (cytokines, complement factors [67] and reactive oxygen species [64,68,69]), this result may suggest that NF-kB is the key regulator of neurotoxicity in reactive ONHAs in human glaucoma. Four NF-*κ*B subunits, c-REL, RELA, NFKB2 and NFKBIA, showed activation at transcriptional level (see Additional File 2). Our data showed elevated expression of several upstream regulators of NF-*κ*B (AR, EGR3, GATA3 and SATA3/BST2), as well as downstream targets in glaucomatous ONHAs (Figure 4A). Transcriptional changes in other well known activators of NF-*κ*B and AP-1, such as TNF*α*, IL-1*β*, and TLRs were below the cut-off level of our analysis, and examination of post-translational activation of those was outside the scope of this study. Alternatively, we performed immunohistochemical analysis of optic nerve slices from human donor tissue (see Additional File 1, Supplement Table S1), which validated the activation of p50/NFKB1 and p65/c-REL subunits in glaucoma (Figure 4B). Transcriptional activation of the androgen receptor (AR) module indicated that glaucomatous ONHAs convert 5*α*-dihydrotestosterone (5*α*-DHT) into 5*α*-androstane-3*α*, 17*β*-diol (3*α*-diol) more efficiently than normal astrocytes, due to over-expression of the gene AKR1C6 encoding the enzyme 3*α*-hydroxysteroid dehydrogenase (3*α*-HSD) [43]. 3*α*-diol can regulate various cellular functions, and is connected to the DI network via AR. The DI sub-network for AR does not include 3*α*-HSD (see Additional File 3, Supplement Figure S10), which is markedly up-regulated in glaucomatous ONHA (see Additional File 2) and has an indirect connection to AR through 3*α*-diol. NF-*κ*B along with its co-regulators, supervillin (SVIL) and RAD9A, facilitates transcriptional activation of AR in good agreement with published data [65]. Also consistent with the literature [66] AR activated downstream targets including AQP9, mitochondrial superoxide dismutase (SOD2), IGF1 and AP-1 (JUND subunit). Given that activation of AQP9 and SOD2 is characteristic of activated astrocytes [67], we suggested that 3*α*-diol plays a protective role in glaucomatous ONHA by increasing the SOD2 expression via AR [68,69].

**Figure 5:**
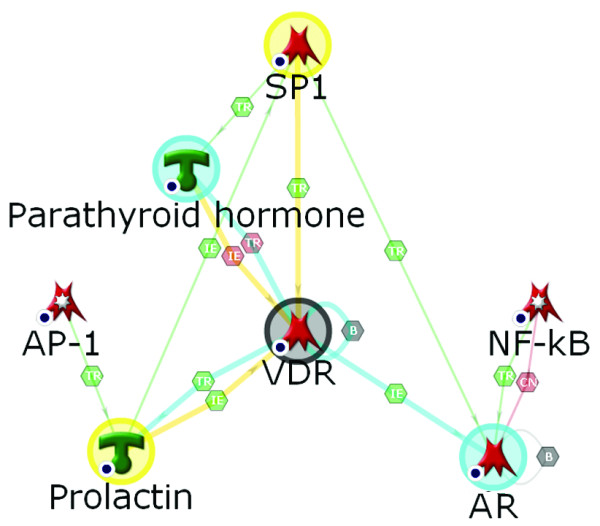
**A "core" network showing interactions between the major hubs of the "global" differential network**. Transcription factors in glaucomatous ONHAs are involved in complex co-regulation events between each other, and the signaling peptides parathyroid hormone and prolactin. Hexagonal symbols on edges define the type of interaction in the link, see Figure 2; Additional Files 1 and 2, Supplement Table S3 for complete legend.

We analyzed cross-activation within regulatory networks formed by top five transcription factors. For instance, MYB and CYP27B1 were co-activated by SP1, AP-1, NF-*κ*B and VDR, IL8 was activated by AP-1, NF-*κ*B and VDR. Six genes were co-activated by both AP-1 and NF-*κ*B, four genes – by NF-*κ*B and VDR, and two genes -by NF-*κ*B and AR (Table 5). Network analysis suggested that major transcription factors activated in glaucomatous ONHAs were involved in complex co-regulation events with each other and the signaling peptides parathyroid hormone and prolactin (Figure 5). Prolactin, which has been implicated in neuroinflammatory response [70], showed differential activation in the microarray data and the prolactin-inducible protein was revealed by proteomics (see Additional File 1, Supplement Table S6).

**Figure 6:**
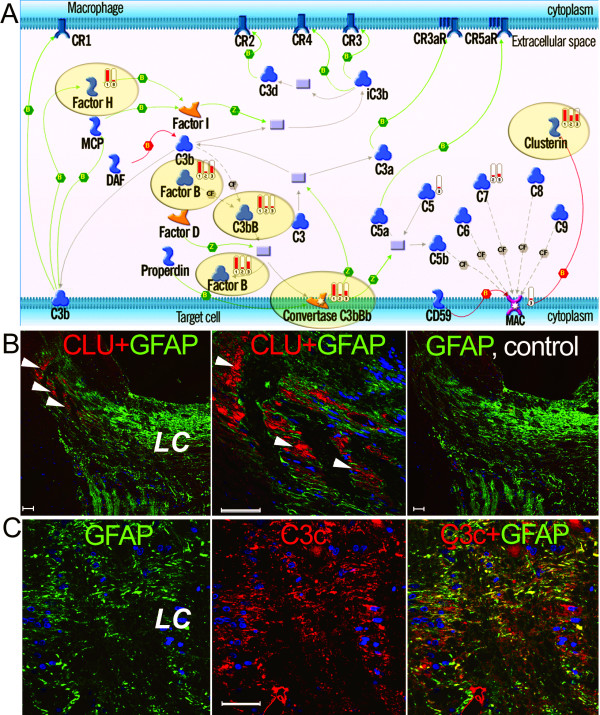
**Complement pathway activation in glaucomatous ONHAs**. **A**. The relevant proteomic data (solid red indicator 1) and the differential gene expression data (indicators 2 and 3), mapped on the canonical pathway originally characterized in macrophage, show cross-verification of the complement pathway activation in glaucomatous ONHAs. Pathway steps confirmed by both data types are highlighted with ovals. Differential gene expression data are shown for Group 1 and 2 separately. **B, C **Immunostaining (red) for clusterin (CLU, arrowheads) and complement C3c (C3c) showing increased accumulation of both proteins in the lamina cribrosa (LC) region of glaucomatous vs. normal human ONH. Control staining for CLU was performed with primary antibodies omitted (B, right panel). DAPI-stained nuclei, blue; size bar = 50 *μ*m

Consistent with the results of GO enrichment analysis, we observed robust activation of a network for innate immunity in glaucomatous ONHAs. Given a non-linear correlation between gene expression and protein translation, and post-translational activation of complement system, we tested ONH samples for accumulation of relevant proteins. Our immunohistochemistry and shotgun proteomics data fully supported the transcriptomic data, showing accumulation of clusterin, complement factors H and B, and complement protein C3c, a by-product of activation of the key complement protein C3 in ONH of eyes with glaucoma (Figures 6A–C). Activation of clusterin and complement proteins in human ONHAs is in good agreement with recent observations in rat, where it contributed to retinal ganglion cell (RGC) death induced by chronic ocular hypertension [42]. Similar activation has been detected in brain neurons challenged by hypoxic-ischemic injury [71,72]. On the DI network for glaucoma, clusterin is connected to two major hubs, AP-1 and NF-*κ*B (Figure 2; also see Additional File 3, Supplement Figure S8). Upstream regulators of clusterin included c-Fos, AP-2A, beta-endorphin and APP, while C7, C8 and C9 complement proteins were major downstream targets activated in glaucomatous ONHAs (see Additional File 3, Supplement Figure S4). Complement activation has been implicated in CNS injury and inflammation, brain ischemia, Alzheimer's and Parkinson's diseases, and more recently, in age-related macular degeneration (AMD) [26,73,74]. Clusterin, a major complement regulator, is also a molecular chaperone [75], and is essential for cell homeostasis. Excessive accumulation of clusterin in glial cells, however, correlated with neurotoxicity in brain ischemia [26]; it promoted microglia activation [76] and to contributed to RGC loss in a retinal hypoxia-ischemia model [77]. Our analysis strongly suggests that, similarly to the above-mentioned pathologies, the inflammatory damage to optic nerve in POAG is facilitated by excessive production of complement proteins and clusterin by the reactive ONHAs.

### Networks for genes with decreased expression

Fifty out of a total of 301 genes down-regulated in the combined data set (2.5 fold threshold) were connected in MetaCore into a DI network (see Additional File 3, Supplement Figure S9) with a p-value < 0.1. This p-value is relatively high due to a smaller percentage of functionally interconnected genes in the subset of genes with decreased expression. The major hubs in the network are interferon gamma (IFN-*γ*, 19 edges), and transcription factor STAT1 (17 edges) (Table 3). STAT1 and INF-*γ *are the core hubs in the down-regulation network that are closely connected to the down-regulated modules c-Myb, PAI1, PDGF receptor, GPCR G alpha (q)-specific peptide, Substance P receptor, ATGTR1, TACR1 and CCR1 (Table 4). This network features a number of signaling protein ligands, including thrombopoetin (CCL5), lactoferrin, substance P, IFN-*γ*, neuromedin U, activin beta A, protein C, CCL8, and is consistent with a decrease in STAT1 and STAT4, a transcriptional regulators for most of these genes. STAT1 activation is pro-apoptotic [78], it is involved in the regulation of vital processes such as cell cycle, survival [79], and is implicated in ischemic response and inflammation. Down-regulation of STAT1 is in agreement with the suppression of its key regulators IFN-*γ*, EGFR and PDGF [80], and contributes to activation of inflammation-associated genes IRF-1, iNOS, and TNF-*α*. Decreased IFN-*γ *and STAT1 signaling, therefore, contributes to the survival of activated ONHAs in glaucoma.

### Integration of proteomics data

The proteomic data were generated in an independent series of experiments by shotgun MS/MS analysis of human ONH tissue dissected from eight normal and eight glaucomatous donor eyes [81]. From a combined set of 250 identified proteins, we selected two condition-specific subsets of proteins. One was comprised of 32 proteins that were detected only in normal tissue; the second subset contained 68 proteins that were only detected in glaucomatous eyes. Only those proteins which were identified at least twice in one sample group and not identified in the other, were considered differentially accumulated. A set of protein IDs was subsequently filtered using a reference list of proteins with known expression in neurons (such as 14-3-3 and HSP families), astrocytes, lamina cribrosa and blood endothelial cells, which contribute to the bulk of the optic nerve head tissue. Thirty five out of 49 glaucoma-enriched and 12 out of 32 normal-enriched proteins matched the objects on MetaCore maps and networks (see Additional File 1, Supplement Table S6) and their corresponding genes were expressed in ONHAs. The top scored GO processes in the glaucoma-enriched proteomic data included activation of the complement pathway of the innate immunity, Stress response via heat-shock protein HSP70 and chromatin condensation (Table 6). Significantly, MS/MS analysis detected complement factors B and H only in the glaucomatous ONHAs, thus providing additional validation of the microarray and immunohistochemistry data (Figure 6) that indicated robust activation of the complement pathway in glaucoma.

**Table 6:** Distribution of GO processes in glaucomatous ONHAs revealed by proteomics

**Top 10 GO processes (for 35 proteins)**	**p-value**	**maps (for 20 mapped proteins)**	**p-value**
Complement activation. Alternative pathway	3.60E-06	CDC42 in cellular processes	1.10E-03

Response to heat	1.10E-05	Alternative complement pathway	1.70E-03

Nucleosome assembly	3.80E-05	Putative ubiquinin pathway	3.10E-03

Complement activation	4.10E-05	Role of ASK1 under oxydative stress	3.20E-03

Glycogen catabolism	5.40E-05	Role of IAP proteins in apoptosis	4.10E-03

DNA DSB repair via homologous recombination	1.00E-04	Glucocorticoid receptor signaling	5.10E-03

Chromosome organization	1.40E-04	Role of Akt in hypoxia	7.80E-03

Carbohydrate metabolism	1.50E-04	Parkin disorder under Parkinson's disease	8.10E-03

Innate immune response	2.20E-04	Role of Parkin in the ubiquitin-proteosomal pathway	8.10E-03

Small GTPase mediated signal transduction	3.10E-04	Urea cycle	3.40E-02

The combined transcriptomic and proteomic dataset that included both up-and down-regulated molecular entities (>2.5 fold change) was used to build a DI network in order to detect modules and pathways potentially overlooked by "unidirectional" analysis. The resultant network of 488 nodes (See Additional File 3, Supplement Figure S11) was interlinked by more then 30 major hubs, where the top-scoring five included SP1, HNF-4*α*, AR, NF-*κ*B and AP-1/cFos (DE up/down, Table 7). It is noteworthy that on this combined network, the VDR hub is scored lower (less connectivity) than on any of the "unidirectional" networks. In contrast, HNF-4*α *hub showed a higher score, suggesting an essential role of this transcription factor in cellular regulation of the reactive ONHAs.

**Table 7:** Comparison of the hub structure in the DI networks built from the three data sets and the combined set

DI networks			
DE set	DE up/down	G-set	G/DE combined

SP1*	.SP1*	TNF*α*	SP1*

VDR*	HNF-4α*	c-Jun*	c-Jun*

NF-kB*	AR*	ET-1	P53*

AP-1*	NF-kB*	STAT3*	VDR*

AR*	AP-1*	IL-1*β*	STAT1*

c-Fos*	STAT1*	AP-1*	AP-1*

STAT5*	c-Fos*	c-Fos*	TNF*α*

HNF-4α*	c-Rel*	EGFR	IL-1*β*

c-Myb*	VDR*	P53*	EGFR

IGF-1*	c-Myb*	iNOS	IFN*γ*

*transcriptional factors

**bolded**, top scored hubs shared by most networks

### Integration of genetic data on the networks

In order to identify a subset of genetically (epistatically) inter-connected genes encoding physically-interacting proteins implicated in glaucoma, we built networks using the manually-annotated list of POAG-linked genes as the seed nodes. The list of 70 genes, the "G-set", was collected mostly from epidemiological, genetic and partly from biochemical studies, i.e. the G-set potentially represented epistatic and/or mechanistic associations between genes, proteins and the onset of POAG (see Additional File 1, Supplement Table S7). However, it is noteworthy that causative relationship with POAG for most of these genes has not been established and their link with POAG is only putative. Published gene expression – POAG associations were not included in the G-list. Epistatic, "causative" associations are inherently different from the physical protein interactions that populate the MetaCore database, which define a "mechanistic interactome". Since gene expression – POAG associations were excluded from the G-set, we did not expect *a priori *any considerable overlap between the G-set and the list of differentially-expressed genes (DE-set). Indeed, only nine genes from the G-set were differentially expressed in glaucomatous ONHAs. The top-scoring GO processes for the G-set included response to wound, cell proliferation, visual perception, blood pressure regulation and inflammatory response while the top canonical pathways maps were ECM remodeling, the Role of VDR in regulation and IL1 signaling pathway (Table 8). Some processes (bolded in Table 8) overlapped with those that were enriched in the DE-set. The direct interaction network generated from the G-set connected 47 out of 70 genes into a statistically significant network (p-value < 0.0001) (See Additional File 3, Supplement Figure S12). Signaling proteins made up 17 of the 47 nodes on the network, with TNF*α*, AP-1, endothelin-1 (ET-1), IL-1*β*, and APP being the major hubs. Major transcription factors included AP-1, P53 and STAT3 (Table 9). As expected, half of the G-network hubs were receptors and ligands, with TNF*α *and ET-1 being the top ones, in contrast to the transcriptional factor-regulated DE networks (Table 7).

**Table 8:** Distribution of GO processes and pathways maps for the "literature" G-set gene list

**Top GO processes**	**p-value**	**Pathway maps**	**p-value**
Response to wounding	7.09E-07	**ECM remodeling**	2.00E-12

**Cell proliferation**	3.63E-06	**VDR in regulation of differentiation**	1.10E-06

Visual perception	2.08E-05	Heme metabolism	3.90E-06

**Blood pressure regulation**	2.34E-05	**IL1 signaling pathway**	5.70E-06

Heart development	2.92E-05	**MIF in innate immune response**	2.30E-05

**Inflammatory response**	7.58E-05	Integrin outside-out signaling	1.10E-04

Peptide transport	8.03E-05	**MAPK cascade. Nuclear function of p38-MAPK**	1.20E-04

Cytosol to ER transport	8.03E-05	HGF signaling pathway	1.50E-04

**Positive regulation of I-kB/NF-kB cascade**	9.90E-05	**JNK pathway**	1.80E-04

Skeletal development	1.11E-04	Plasmin signaling	2.30E-04

**Bolded**, *processes and maps similar to those revealed with the gene expression data*

**Table 9:** The basic network statistics ("top tens") for the combined G/DE/DI network

**Hubs**	**TFs**	**Signaling peptides**	**GO processes**
SP1	ANDR	Amphiregulin	Regulation of transcription from RNA Pol II promoter

c-Jun	AP-1	APOE	Skeletal development

P53	Bcl-6	BDNF	BMP signaling pathway

VDR	c-Fos	BMP2	Transcription

STAT1	c-Jun	BMP4	Cartilage development

AP-1	c-Myb	BMP5	Cell differentiation

TNF*α*	c-Rel	BMP7	Regulation of transcription, DNA-dependent

IL-1*β*	C/EBPbeta	COL1A1	Positive regulation of progression through mitotic cell cycle

EGFR	E2A	COL4A4	Development

IFN*γ*	ESR1	Collagen III	Mammalian eye development

Despite little overlap between the G-set and DE-set, more similarities were revealed at the network topology level by a functional proximity search for differentially expressed genes within the interactome "neighborhood" of the G-set. This approach establishes relationships between datasets of different types (meta-analysis) using the parameter of functional proximity, which is defined as one-step physical interactions between the gene products from different sets [82]. We built a set of networks using the G-set gene content as input nodes and the Analyze Networks (AN) algorithm. We then mapped the expression data onto the AN networks. The AN algorithm prioritizes sub-networks based on relative enrichment with experimental (gene expression) data and canonical pathways (See Materials & Methods). This helped us to identify the proximal DE-set network objects and to fill the gaps in the fragmented disease association data (see Additional File 3, Supplement Figure S13). After analyzing the 50 top scored AN networks built from the G-set, we identified 37 up-regulated and 20 down-regulated genes (see Additional File 1, Supplement Table S8). The majority (32 out of 57) of genes identified in the interactome neighborhood of the genetic network were also present on the global DI network for activated genes and proteins (see Additional File 3, Supplement Figures S14 a, b).

We then merged the G-set with the 57 DE-set genes from close interactome neighborhood to identify possible connectivity between the networks originating from genetic and gene expression data. The combined data set of 119 genes included only nine genes common to both data sets. Differential activation of another four genes from the G-set was suggested by the proteomic data. GO distribution showed enrichment in the processes: regulation of transcription, BMP signaling pathway, cell differentiation and development, in the combined G- and DE-sets (Table 7). The stringent DI algorithm connected 102 out of 119 nodes into one combined G/DE network (see Additional File 3, Supplement Figures S14 a, b), with a p-value < 0.0001 (see Additional File 1, Supplement Table S9). It narrowed the interaction gaps between the literature-derived G-set data. An important feature preserved in both DE and G/DE networks is the synergy between the major transcription factors AP-1, SP1, VDR, AR and NF-*κ*B. As the G-set data are not cell type- or tissue-specific, the conservative hub structure of the disease network may represent a core feature of the glaucomatous process across all eye tissues. Some major hubs present only in the G/DE network included P53, STAT1, TNF*α*, IL-1*β*, EGFR, and IFN-*γ *(Table 7), from which STAT1 and P53 hubs increased their relative scoring, suggesting that their potential role in astrocyte activation in glaucoma should be further investigated.

## Discussion

The ultimate goal of our functional analysis of glaucoma-related high content data was reconstruction of the condition-specific model at three levels: i) stimuli, i.e. the endogenous and exogenous (environmental) signals, which trigger the disease condition; ii) intracellular signaling activated in the disease; and iii) effectors – the cellular processes, structural complexes and metabolic pathways altered by aberrant signaling in the disease. Changes at the effector level should be consistent with the observed disease manifestations in molecular profile and phenotype. While complex exogenous signals are known to include genetics, age, mechanical damage, etc., our network analysis has revealed molecular modules that are activated intracellularly, and suggests plausible disease effectors in glaucomatous ONHAs. This analysis suggests that intracellular signaling in reactive ONHAs in response to glaucomatous conditions is mediated by a synergy of key transcription factors VDR, NF-*κ*B, AP-1 and AR. These changes are paralleled by decreased signaling via integrins, PDGF and STAT1, which might be insulting to the retinal ganglion cells, since these molecules control adherence, while the motility of glaucomatous astrocytes is likely to be disruptive to their metabolic support functions [2,3,48]. Integration of the gene expression and proteomic data for ONHAs confirmed substantial activation of the innate immune response and suggests that this is one of the key effector events in activated astrocytes, which has been also detected in recent gene expression studies [42,83]. At the level of metabolism, our analysis revealed coordinated activation of endogenous alcohol and aldehyde dehydrogenases, which is consistent with increased metabolic activity and oxidative stress response [69,84]. Similar activation of these pathways has been shown in the brain of Alzheimer's sufferers, and is likely to play a protective role in detoxification of reactive intermediates of lipid peroxidation [85]. Significant activation of phospholipase A2 and it's receptor PLA2R1, which are involved in a complex network linking receptor agonists, oxidative agents, and proinflammatory cytokines to the release of arachidonic acid [86], represents a pro-inflammatory effect of glial activation also observed in brain pathologies [87].

Our network analysis demonstrated activation of potentially neurotoxic and neuroprotective programs in ONHAs in response to glaucomatous ON injury. We found that NF-*κ*B is significantly over-connected on the DI and AN networks, showing links to both programs. In contrast to the well known pro-survival role of this transcription factor, recent studies have demonstrated a profound negative impact of NF-*κ*B activation on neuronal survival in various CNS pathologies [88-91]. Our analysis suggests that the activation of a NF-*κ*B-controlled transcriptional program in glaucomatous ONHAs facilitates pro-inflammatory events and is, therefore, potentially neurotoxic. It has been demonstrated that in the glia reactivated with LPS, amyloid-beta, elevated hydrostatic pressure, cytokines or double-stranded RNA, NF-*κ*B induction correlated with neurotoxic levels of NOS-2, MMPs, JNKs (MAPK8-10), complement, clusterin and cytokines [6,45,92-94]. Activation of several NF-*κ*B subunits demonstrated at the levels of gene transcripts, proteins and over-connectivity with upstream regulators and downstream targets on the disease network, supports the notion that NF-*κ*B is key convergence hub in this pathology. In contrast to systemic inhibition of this vital transcription factor that would have a negative impact on neuronal survival, as shown in recent studies utilizing sulfasalazine inhibitor [95] or a knockout mouse model [96], our experimental data strongly endorse astroglial NF-*κ*B as a potential target for a cell-level inhibition in glaucoma therapy.

## Conclusion

Direct networking of gene products via protein interactions can be used to identifying modules of epistatic interaction between disease related genes, as described for yeast metabolism [97]. As we showed previously [10], major topological features of differential networks represent molecules affected by the disease process, and represent potential targets for therapeutic intervention. In this work, we took advantage of the functional analysis capabilities and database of molecular interactions in MetaCore and cross-referenced two experimental datasets, proteomic and genetic, which are otherwise poorly comparable. Using a two-step procedure, we narrowed the set of differentially expressed genes to fifty seven genes (DE-set) most relevant to the G-set of genes associated in the literature with glaucoma, effectively filling the gaps in the fragmented literature-derived genetic data. Our analysis of glaucomatous astrocytes indicated that the synergistic activation of the major hubs SP-1, VDR, NF-*κ*B and AP-1 in response to oxidative stress and mild ischemia associated with glaucoma, orchestrates genome-wide changes in the transcriptional profile and chronic activation of ONHAs. In contrast to the pro-survival effects of VDR, AP-1and AR, the harmful effects of chronic activation of major downstream effectors such as NF-*κ*B, clusterin, complement and ephrin A4 receptor are likely to be responsible for tipping the balance in the optic nerve microenvironment towards neurotoxicity and inhibition of regeneration. Combined, these glial factors could actively contribute to the retinal ganglion cell death in glaucoma by exacerbating inflammatory damage to challenged axons. Our results imply that the suppression of the major downstream effectors may represent feasible strategy for glia-targeted therapy of glaucoma, ensuing further experimental validation of these targets.

## Methods

### Optic nerve Astrocytes

Cultures of human ONHA were generated as previously described [23]. Briefly, ONH were dissected and processed within 24 h of death. Four explants from each lamina cribrosa were dissected and placed into 25-cm^2 ^Primaria tissue culture flasks (Falcon, Lincoln Park, NJ). Explants were maintained in DMEM-F12 supplemented with 10% FBS (Biowhittaker, Walkerswille, MD) and PSFM (10,000 U/ml penicillin, 10,000 *μ*g/ml streptomycin and 25 *μ*g/ml amphotericin B; Gibco/BRL, Gaithersburg, MD). Cells were kept in a 37°C, 5% CO_2 _incubator. After 2–4 weeks, primary cultures were purified by using modified two-step immunopanning procedure described by Mi and Barres [98]. Purified cells were expanded after characterization by immunostaining for astrocyte markers GFAP and NCAM as described [98,99]. Second passage cell cultures were stored in RPMI 1640 with 10% DMSO in liquid nitrogen until use. For each set of experiments, cells were thawed and cultured for one more passage so that sufficient cells from the same batch were available in each set of experiments. Astrocytes from both normal and glaucomatous eyes were cultivated similarly in parallel experiments. This ensured that differential transcriptomic data obtained from them have been normalized for cultivation-induced changes, and showed only disease-imprinted profile changes as reported by others [24,25].

Seventeen pairs of normal human eyes from donors (age 58 ± 12) with no history of chronic CNS or eye disease were obtained from the National Disease Research Interchange (NDRI) and from Mid-America Eye Bank (St. Louis, MO) within 2–4 h of death (see Additional File 1, Supplement Table S1). A total of eight eyes from six donors with documented primary open angle glaucoma (POAG) (age 73 ± 9) were obtained for the analysis. Seven normal eyes were used to generate astrocytes for microarray analysis, the rest were used for real time RT-PCR. ONH were dissected and processed within 24 h of death to generate astrocyte cultures. To test whether the normal eyes in this study may have had hidden optic nerve disease, and to assess damage in samples from POAG. Samples of the myelinated nerves were fixed in 4% paraformaldehyde, post-fixed in osmium, embedded in epoxy resin, and stained with paraphenylendiamine to detect axon degeneration [100,101].

### Human ONHA microarray data

The microarray data for two separate donor groups, Group 1 and Group 2 (four or three normal and four eyes with glaucoma in each group, a total of fifteen human optic nerve samples) were obtained from primary human ONHAs, type 1B, derived from glaucomatous and from normal control eyes as described previously [22]. The microarray analysis of Groups #1 and #2, which was separated by a two year time interval possessed significant difference in the mean threshold levels of signal intensities and, therefore, corresponding data were analyzed separately. In total, we analyzed three differential expression (DE) data files: donor Group 1, revealed 322 up-regulated and 152 down-regulated Affymetrix gene IDs (that correspond to 298 and 130 non-redundant genes by HUGO nomenclature, respectively); donor Group 2, (357/335 up- and 271/261 down-regulated AFFI/HUGO IDs); and combined dataset comprised of 461 up-regulated and 301 down-regulated AFFI IDs/genes (see Additional File 2). The latter set was obtained by pair-wise comparison of all disease vs. all normal samples. All original data were deposited in GEO (accession #GSE2378). A portion of these data (referred to as donor Group 1) has been published previously [22]. The raw normalized data were statistically analyzed as described below and used to obtain the gene-specific ratios of differential expression. In total, we analyzed three data files composed of genes with fold change exceeding 2.5: donor Group 1, revealed 322 up-regulated and 152 down-regulated Affymetrix gene IDs (that correspond to 298 and 130 non-redundant genes by HUGO nomenclature, respectively); donor Group 2, (357/335 up- and 271/261 down-regulated AFFI/HUGO IDs). In addition, 484 up-regulated and 323 down-regulated genes comprised the combined non-redundant set of genes with known functions, in which 461 and 301, respectively, were recognized as "network objects" in the MetaCore database (see Additional File 2).

### Human ONHA proteomic data

Proteomic data was generated by shotgun MS/MS analysis of human ONH tissue from eight normal and eight glaucomatous donor eyes as described previously[81]. From the combined set of 248 proteins identified by this analysis we selected two subsets: proteins detected only in normal tissue (31 IDs) and those found only in glaucomatous eyes (67 IDs). Shotgun MS-identification of proteins has very limited ability in ruling out the absence of a protein, so for further analysis we only selected those proteins that were identified only in glaucomatous tissue. To focus on the data relevant to astrocytes, the selected protein IDs were filtered against proteins with known association with neuronal axons, which formed the bulk of the optic nerve head tissue. Of the remaining 50 IDs, 35 had links to MetaCore maps and networks and were used in our analysis. The expression of all 35 proteins in astrocytes was confirmed by microarray data.

### Statistical analysis of gene expression data

Affymetrix microarray data contain intensity values and absent/present flags for each probe set. There were a total of sixteen sets of data used in this study, eight with glaucoma and eight with normal expression. The calculations and data analyses were done with R statistical software . First, we tested whether or not the gene expression values from the glaucoma set were different overall from those of the normal set. This was done by comparing two statistical models:

y_i _= *μ*e_i_, i = 1,..., number of genes, grand mean, e_i _– error, and

y_ij _= *α*_i _+ e_ij_, i = 1,2, j = 1,..., number of genes, *α*_i _– group mean, e_i _– error.

The first model ignores the presence of two different groups. In the second model, we differentiate between the means of the glaucoma and normal groups.

Next, we analyzed the expression of every gene in the glaucoma set with respect to the normal set. Since we had data from eight independent experiments, we could not obtain accurate results from pairing each set of glaucoma data with a unique set of normal data. Every possible glaucoma-normal combination was therefore considered for every gene. The number of such combinations is n*m, where n is the number of glaucoma values, and m is the number of normal values. When no expression values are missing for a specific gene in any of the experiments, there are 16 combinations for every of the two donor groups. The ratios of glaucoma to normal expression were then calculated. To allow the construction of a linear model from the data, base-ten logarithms were taken for each ratio.

The table of results consists of eight columns (see Additional File 1, Supplement Table S10). The first column is the gene; the second is the mean of the log ratios. To help visualize the results, the third column contains the ratio itself, such that the values for all over-expressed genes are glaucoma/normal ratios, and the values for under-expressed genes are negative normal/glaucoma ratios. The fourth column shows the standard error (se) for the mean log ratio of a gene, which is defined by , where  is the sample variance, and n is the size of the sample.

The next two columns present the bounds of the 95% confidence interval for every mean log ratio. Confidence intervals were calculated with Bonferroni adjustments, since we wanted to maintain an overall confidence of 95% while considering all genes at once. Each confidence interval is defined by



Due to the very large number of degrees of freedom (189375), values from the standard normal distribution could also have been used instead of those from the Student t distribution. The seventh column has the "TRUE" value if the 95% confidence interval for that log ratio contains 0 and "FALSE" if it does not. That way it is easy to see which genes have glaucomatous expression levels different from the normal tissue expression level of the same genes.

Another way to interpret the results is to use p-values, i.e. the smallest levels of significance for which one can consider the corresponding log ratio means is to be different from zero. If a given p-value is smaller than the chosen level of significance for the test, then it can be concluded that the gene is expressed. A p-value is calculated by P(Z < (-_gene_/se)) + P(Z > (_gene_/se)), Z ~ N(0, 1). A Bonferroni adjustment is then applied to this result to ensure small enough overall error. Tables were created from the results with genes that have expression ratios greater than a chosen ratio or smaller than the negative chosen ratio and p-values smaller than 0.05. Another pair of columns contains the average glaucoma and normal expression levels for every gene, the standard error for these values and the 95% confidence intervals to identify the ranges for the true averages. A present flag was given to every gene that is marked as present in at least three out of four experiments. Otherwise the flag is set to "Absent".

### Functional analysis of the data

The functional analysis workflow consists of series of qualitative and quantitative procedures for parsing large datasets into smaller, functionally-meaningful subsets, such as linear signaling and metabolic pathways, and cellular and molecular processes. The overlap between genes/proteins within a functional category and the components of a high-content dataset identified as meaningfully altered can be given a p-value based on the likelihood of this overlap happening by random chance (see below). Multiple functional categories can be scored for each dataset, a procedure referred to as enrichment analysis[102]. The distribution of categories reflects their relative relevance to the condition within the dataset. We used the content of gene ontologies from OnthoExpress [35] in MetaCore™ (GeneGo Inc, MI, ) for enrichment analysis of genes differentially expressed in reactive vs. normal ONHAs.

### Interactome analysis for relative connectivity

The goal of the analysis was to identify hub proteins, particularly transcription factors, with a significantly large number of interactions within the set of interest. This type of meta-analysis a part of Interactome tool of the MetaCore and allows calculation of relative connectivity for a hub as a measure of interactions enrichment in experimental data vs. expected for a random data set of a similar size. This characteristics serves as better indication of relevance of a hub gene/protein for the condition-induced changes within a dataset as compared to the absolute number of interactions, where highly prolific hubs (SP-1, P53, etc.) dominate routinely. Relative connectivity of major transcription factors was measured as described by Wood and co-authors [64]. We calculated the interactions of proteins within a set of interest and compared that with the number of connection both in the global protein "interactome"and in the experimental subset. We assigned statistical significance by using the cumulative hypergeometric distribution as follows:  where



N – the number of proteins (protein-based network objects) in our global "interactome" extracted from Metacore

n – number of proteins derived from the sets of genes of interest

D – the degree of a given protein in the global "interactome" database

k – the degree of a given protein within the set of interest

The p-value calculated above gives the probability of observing *k *or more interactions of a given protein (with degree D in global network) by random chance within the set of interest (of size (*n*)).

The probability of observing "under-connected" connected proteins can be calculated by *1-p(k)*.

The input lists of genes were converted to protein-based network objects which have been used in our analysis. The resulting network objects sets were divided by subsets based on the molecular function (receptors, ligands, etc.).

### Identification of differentially expressed genes in close proximity to the genetic network

Data of different origin can be connected on the same networks. For instance, genes implicated in the onset of the disease can be linked with differentially expressed genes via physical interactions between their corresponding protein products. The network analysis process does not require clustering or statistical analysis other than when defining the probability (p-value) of the assembly of a network of a certain size out of randomly selected relevant nodes (described in the Methods). Each network is, therefore, unique for the data set at the level of specific proteins, subunits and binary interactions. The Analyze Networks (AN) algorithm (see Additional File 1, Supplement Table S3) connects every node with all others by unidirectional shortest paths using the G-set as the input nodes. This algorithm builds sets of the overlapping small networks consecutively covering all root nodes from the input list. The essential difference between DI and AN networks is that the latter allow non-root objects to form connectors between root nodes. In each of the top-scoring 50 networks, 40 – 55% of the objects were root nodes from the G-set. Next, we mapped the differential expression data (DE-set) onto AN networks using the filter experiments tool in MetaCore, which mainly maps the non-root nodes. Between one and seven differentially expressed "close neighbor" genes were typically located on each of AN networks (see example in See Additional File 3, Supplement Figure S13). In our data, the majority (32 out of 57) of the interactome neighborhood genes were also present on the global DI network (See Additional File 3, Supplement Figure S14). Since the global DI network included only 277 out of 807 input genes (34%), one would expect only 19 out of 57 genes from the vicinity list to overlap with the DI network list with random distribution. The 56% overlap between two gene expression lists (p-value of such an event is 0.005) indicates a strong correlation between the genetic network and the gene expression network for glaucoma.

### Quantitative RT-PCR

To validate the microarray data, total RNA was extracted from ONHAs that were obtained from normal and glaucomatous eyes using Qiagen RNAeasy mini kits (Qiagen, Valencia, CA). RNA was then purified and quantified by measuring absorbance at 260 nm and treated with RNase free DNase (Ambion). Single strand cDNA was prepared from 2 *μ*g total RNA using SuperScript II reverse transcriptase (Invitrogen). 5 *μ*l of 1:20 -1:80 diluted cDNA were used for reaction with 2× Bio-Rad SYBRGreen SuperMix (Bio-Rad Laboratories Inc, Hercules, CA) in 25 *μ*l, and quantitative PCR was performed by monitoring in real time the increase of fluorescence of SYBR Green using the MyiQ (Bio-Rad Laboratories Inc). Custom primers were designed using the Primer Express program (PE Applied Biosystems) for 22 transcripts representing major network hubs as well as randomly picked DE genes. At least one primer crossed the exon-exon boundary to prevent genomic DNA amplification. Primer quality (lack of primer-dimer amplification) was confirmed by melting curve analysis. Sequences of primers are available upon request. Relative quantification of gene expression was performed using the standard curve method (User Bulletin 2 of the ABI Prism 7700 Sequence Detection System, PE Applied Biosystems, Forster City, CA). Serial dilutions (1:4: 1:16, 1:64, 1:256) of mixtures of all samples were used for standard curves. For each cell culture the relative amount of mRNA for a target gene was normalized to the relative amount of reference gene RNA (18S RNA). Then, the average of normalized relative expression levels for all glaucoma and average for all normal samples for each gene was calculated. The higher average (glaucoma or normal) was divided by the lower average (normal or glaucoma) to determine fold change for each gene. The fold increase in glaucoma (glaucoma higher than normal) was expressed by positive numbers and fold decrease (normal higher than glaucoma) by negative numbers (see Additional File 1, Supplement Table S11). Quantitative PCR data for validation of the expression of the three 3*α*HSD isoforms used isoform-specific probes and primers previously described [43]. The *3αHSD *probes have full homology to the *AKR1C3 *gene sequence and recognized mostly *AKR1C3*. The sequence of the *AKR1C1 *probe has full homology to the *AKR1C1 *and *AKR1C2 *gene isoforms and recognizes both of them.

### Network visualization and analysis

The set of genes differentially expressed at the cut-off value of 2.5-fold was uploaded into the MetaCore Analytical suite (GeneGo, Inc. St. Joseph, MI). MetaCore is a web-based computational platform primarily designed for the analysis of high-content experimental data in the context of human metabolic and regulatory networks and pathways. MetaCore includes a manually-curated database of human protein interactions, metabolism and bioactive compounds. Analysis was conducted in accordance with the application manual and has been described previously [102,103].

### Scoring and prioritization of networks according to the relevance of input data

In most cases, high-content experimental datasets are very large and may include data on thousands of genes. In such cases, the issue of prioritizing networks and network modules is important. Prioritization can be based on different parameters, but generally follows the procedure described below. The data set is divided into two random overlapping subsets, in which the size of the intersection between them represents a random variable with a hypergeometric distribution. We applied this for numerical scoring and prioritization of the node-centered networks. Let us consider a general data set size of *N *with *R *marked objects/events (for example, the nodes with expression data). The probability of a random sub-set size of *n *to include *r *marked events/objects is described by the distribution



The mean of this distribution is equal to:



Where *q *= *R*/*N *defines the ratio of marked objects.

The dispersion of this distribution is described as:



It is essential that these equations are invariant in terms of exchange of *n *for *R *which means that the "subset" and "marked" are equivalent and symmetrical sets. Importantly, in the cases of *r *> *n*, *r *> *R *or *r *<*R *+ *n *- *N*, *P*(*r*, *n*, *R*, *N*) = 0

We used the following z-score equation for comparison and prioritization of node-specific SP sub-networks



Where *N *is the total number of nodes after filtration, *R *is the number of nodes in the input list or the nodes associated with experimental data, *n *is the number of nodes in the network,*r *is the number of the network's nodes associated with experimental data or included in the input list, and *μ *and *σ *are the mean and dispersion of the hypergeometric distribution described above.

### P-value and evaluation of statistical significance of networks

The p-values throughout MetaCore – for maps, networks and processes – are all calculated by using the same basic formula: a hypergeometric distribution in which the p-value essentially represents the probability of a particular mapping arising by chance, given the numbers of genes in the set of all genes on a particular map/network/process and genes in an experiment (see the formula below). For a network of a certain size we can evaluate its statistical significance based on the probability of its assembly from a random set of nodes the same size as the input list (see Additional File 1, Supplement Table S12). We can also evaluate the network's relevance to biological processes or any other subset of nodes. Let us consider a complete set of nodes in the network that are divided into two overlapping subsets. These subsets represent the nodes that are linked to a certain pre-defined node list. In the general case, these subsets are different but overlapping, and we assume that the intersection is large enough and non-random. We do not consider a situation where the intersection is small but non-random. The null hypothesis states that the subsets are independent and, therefore, the size of the intersection satisfies a hypergeometric distribution. The alternative hypothesis states that there is positive correlation between the subsets. Based on these assumptions, we can calculate a p-value as the probability of intersection of two random subsets from the same set.



Where: N-total number of nodes in MetaCore Database, R – number of network's objects corresponding to the genes and proteins in the list; n- total number of nodes in each map or network generated from the list; r- number of nodes with data in each map or small network generated from the list.

### Statistical test for network non-randomness

If we consider a list of nodes corresponding to experimentally altered genes and proteins, this pre-selected list is then used for building the networks using one of the algorithms. The statistical significance of the resulting networks can be defined using the DI algorithm, which displays only the nodes from the input list connected to each other in one step. We can now evaluate the probability of random generation of an interaction cluster of equal or larger size than the number of nodes in the DI network. After defining the list of nodes, a random subset is selected from this list, the network is built with the same algorithm and settings, and the size of the largest network cluster is calculated. The procedure is repeated many times (e.g. 1000 or 10,000 trials) and statistics are accumulated on the number of times the largest cluster of a certain size is generated. The ratio of the number of random clusters the same as the original DI network or larger compared to the total number of trials is a parameter describing the significance of the input list. For example, we calculated these parameters in 10,000 trials using the gene list with unique identifiers from Affymetrix U95 human array as the initial set.

Among the total gene set recognized by MetaCore on the Affymetrix U95A array were 5117 network nodes; random samples of 50 to 500 nodes were selected as an input for network building using the DI algorithm with the same settings as for the original network. The mean cluster size and p-values are presented in see Additional File 1, Supplement Table S12. The calculated p-value of the large DI cluster of 196 out of 484 random nodes was < 0.0001. Calculation was based on statistical probability that 196 genes form a network by random chance.

### Immunohistochemistry

Sagittal sections of four human normal, and four glaucomatous optic nerve heads, 6 *μ*m thick, were used for immunodetection of glial fibrillary acid protein (GFAP), clusterin and complement protein C3c. Human anti-clusterin antibodies were purchased from Upstate (Lake Placid, NY) and applied at a dilution of 1:200; human anti-C3c sheep polyclonal antibodies (Abcam, Cambridge, UK) were used at a dilution of 1:250. For double immunofluorescence staining of reactive astrocytes we used rabbit polyclonal antibody against human GFAP (Sigma, St. Louis, MO) diluted 1:150. The distribution of the primary antibodies was visualized using anti-mouse IgG or anti-sheep IgG secondary antibody conjugated to the Alexa 568 fluorochrome (Molecular Probes, Eugene, OR). To visualize cell nuclei, DAPI stain (Molecular Probes) was applied at the dilution 1:10,000 in PBS buffer (Sigma). For negative controls, the primary antibody was replaced with the appropriate non-immune serum. To control for cross-reactivity in double immunofluorescence, sections were incubated with secondary antibody only. Sections of normal and glaucomatous eyes were stained simultaneously to control for individual variations in immunostaining. Stained slices were then analyzed by confocal microscopy using a Zeiss LSM 510 microscope equipped with argon, HeNe and UV lasers for multiple fluorophore excitation.

## Abbreviations

POAG: primary open angle glaucoma; ONHA: optic nerve head astrocyte; RGC: retinal ganglion cell; NF-*κ*B: nuclear factor kappaB; CNS: central nervous system; HT: high throughput; TF: transcription factor; SP: Shortest Path algorithm: DI: Direct Interactions algorithm; AN: Analyze Network algorithm

## Competing interests

The authors declare that they have no competing interests.

## Authors' contributions

TN and YN have equally contributed to this work: they coordinated and contributed to designing the diverse data integration algorithms, performed the major body of the network analysis and drafted the manuscript. TS and SZ performed the annotation of literature lists for the glaucoma gene associations and the network analysis. ZD, ER, ES performed the functional analysis of meta-data, integration of microarray and proteomics data. RB and NY performed the glaucoma genetic data sorting and integration, discussion of the results of meta-analysis, participated in refining arguments and helped draft the manuscript. SKB has collected and processed human optic nerve samples for protein extraction, generated proteomics data, analyzed it and helped draft the manuscript. OA has performed RNA extraction from human ON samples, processed them for microarray analysis and performed PCR validation of the results. MRH has coordinated glaucomatous and control human eyes collection and participated in microarray data collection and analysis, she also helped draft the manuscript. VIS conceived of the study, participated in its design, coordination of the network analysis and drafted the manuscript. All authors have read and approved the final version of the manuscript.

## Pre-publication history

The pre-publication history for this paper can be accessed here:



## Supplementary Material

Additional file 1 **Supplement Table S1**. Clinical information of donor eyes used to generate cultures of ONH astrocytes. **Supplement Table S2 **Distributions for the top 12 canonical pathway maps and GO functional folders for a subset of genes up-regulated in glaucomatous astrocytes, fold change >2.5. **Supplement Table S3**. Description of the MetaCore™ network building algorithms and the Metacore network legend. **Supplement Table S4**. Down-regulation of integrins in the data sets from the two groups of patients. **Supplement Table S5**. Interactome analysis of connectivity for the top transcription factors on the network for upregulated genes. **Supplement Table S6**. Differential proteomics data for human glaucomatous optic nerves utilized in this study. **Supplement Table S7**. A list of literature-derived genes and proteins linked by genetic and other non-expression methods, and implicated in glaucoma ("G-set"). **Supplement Table S8**. Proximity dataset revealed using the AN networks generated from the G-set ("literature" genes). **Supplement Table S9**. Major parameters used for calculation of p-value for networks. **Supplement Table S10**. Statistical analysis of gene expression data. **Supplement Table S11**. Cluster size and corresponding p-value calculation for networks generated from 500 random nodes. **Supplement Table S12**. Validation of genes encoding major activated hubs in glaucomatous ONHAs by quantitative RT-PCR. Click here for file

Additional file 2 **Original microarray gene expression data, listing AFFY and HUGO IDs for the genes that changed in excess of 2.5 fold in glaucomatous vs. normal donor eyes.** Click here for file

Additional file 3 **Supplement Figure S1**. The shortest paths (SP) network built using up-regulated genes from GO process "complement activation" with mapped experimental data. **Supplement Figure S2**. The shortest paths (SP) network built from the gene list derived from the up-regulated GO process "nervous system development". **Supplement Figure S3**. Canonical map for "ephrin signaling" that scored the highest for differential activation during the experimental data mapping and analysis. **Supplement Figure S4**. Canonical map of VDR signaling with mapped experimental data. **Supplement Figure S5**. The highest scored map "Bile acids biosynthesis", a part of the process for cholesterol metabolism, with mapped experimental data. **Supplement Figure S6**. PDGF signaling was the highest scored (lowest p-values) pathway map among down-regulated pathways. **Supplement Figure S7**. A DI network built from the subset of downregulated genes extracted from the top 12 scored pathway maps. **Supplement Figure S8**. A DI network for the combined differential gene expression data set in glaucoma. **Supplement Figure S9**. A DI network for the downregulated genes from the group 1 and group 2 combined data set. **Supplement Figure S10**. The highest scored AN network for the Androgen receptor (AR) cluster built using the combined data set as an input list. **Supplement Figure S11**. The DI network for the combined transcriptomic and proteomic data set. **Supplement Figure S12**. The DI network built using 70 "literature" genes (G-set). **Supplement Figure S13**. A network illustrating the proximity analysis using the AN algorithm. **Supplement Figure S14a**. The "disease" DI network for the combined G/DE proximity data set implicated in glaucoma. **Supplement Figure S14b**. The "disease" DI network with the mapped gene expression data. Click here for file

## References

[B1] Hernandez MR, Pena JD (1997). The optic nerve head in glaucomatous optic neuropathy. Arch Ophthalmol.

[B2] Swanson RA, Ying W, Kauppinen TM (2004). Astrocyte influences on ischemic neuronal death. Curr Mol Med.

[B3] Varela HJ, Hernandez MR (1997). Astrocyte responses in human optic nerve head with primary open-angle glaucoma. J Glaucoma.

[B4] Sofroniew MV (2005). Reactive astrocytes in neural repair and protection. Neuroscientist.

[B5] Hashimoto K, Parker A, Malone P, Gabelt BT, Rasmussen C, Kaufman PS, Hernandez MR (2005). Long-term activation of c-Fos and c-Jun in optic nerve head astrocytes in experimental ocular hypertension in monkeys and after exposure to elevated pressure in vitro. Brain Res.

[B6] Liu B, Neufeld AH (2000). Expression of nitric oxide synthase-2 (NOS-2) in reactive astrocytes of the human glaucomatous optic nerve head. Glia.

[B7] Quigley HA (1999). Neuronal death in glaucoma. Prog Retin Eye Res.

[B8] Thornton-Wells TA, Moore JH, Haines JL (2004). Genetics, statistics and human disease: analytical retooling for complexity. Trends Genet.

[B9] Jenssen TK, Laegreid A, Komorowski J, Hovog E (2001). A literature network of human genes for high-throughput analysis of gene expression. Nature Genetics.

[B10] Nikolsky Y, Nikolskaya T, Bugrim A (2005). Biological networks and analysis of experimental data in drug discovery. Drug Discov Today.

[B11] Sharan R, Ideker T (2006). Modeling cellular machinery through biological network comparison. Nat Biotechnol.

[B12] Barabasi A-L, Oltvai ZN (2004). Network biology: understanding the cell's functional organization. Nature Reviews Genetics.

[B13] Jeong H, Tombor B, Albert R, Oltvai ZN, Barabasi AL (2000). The large-scale organization of metabolic networks. Nature.

[B14] Ravasz E, Somera AL, Mongru DA, Oltvai ZN, Barabasi AL (2002). Hierarchical organization of modularity in metabolic networks. Science.

[B15] Milo R, Shen-Orr S, Itzkovitz S, Kashtan N, Chklovskii D, Alon U (2002). Network motifs: simple building blocks of complex networks. Science.

[B16] Bortoluzzi S, Romualdi C, Bisognin A, Danieli GA (2003). Disease genes and intracellular protein networks. Physiol Genomics.

[B17] Kitano H (2002). Computational systems biology. Nature.

[B18] Csete ME, Doyle JC (2002). Reverse engineering of biological complexity. Science.

[B19] Ekins S, Bugrim A, Brovold L, Kirillov E, Nikolsky Y, Rakhmatulin E, Sorokina S, Ryabov A, Serebryiskaya T, Melnikov A (2006). Algorithms for network analysis in systems-ADME/Tox using the MetaCore and MetaDrug platforms. Xenobiotica.

[B20] Ekins S, Kirillov E, Rakhmatulin EA, Nikolskaya T (2005). A novel method for visualizing nuclear hormone receptor networks relevant to drug metabolism. Drug Metab Dispos.

[B21] Shannon P, Markiel A, Ozier O, Baliga NS, Wang JT, Ramage D, Amin N, Schwikowski B, Ideker T (2003). Cytoscape: a software environment for integrated models of biomolecular interaction networks. Genome Res.

[B22] Hernandez MR, Agapova OA, Yang P, Salvador-Silva M, Ricard CS, Aoi S (2002). Differential gene expression in astrocytes from human normal and glaucomatous optic nerve head analyzed by cDNA microarray. Glia.

[B23] Kobayashi S, Vidal I, Pena JD, Hernandez MR (1997). Expression of neural cell adhesion molecule (NCAM) characterizes a subpopulation of type 1 astrocytes in human optic nerve head. Glia.

[B24] Mense SM, Sengupta A, Zhou M, Lan C, Bentsman G, Volsky DJ, Zhang L (2006). Gene expression profiling reveals the profound upregulation of hypoxia-responsive genes in primary human astrocytes. Physiol Genomics.

[B25] Rozsa FW, Scott KM, Pawar H, Samples JR, Wirtz MK, Richards JE (2007). Differential expression profile prioritization of positional candidate glaucoma genes: the GLC1C locus. Arch Ophthalmol.

[B26] Van Beek J, Chan P, Bernaudin M, Petit E, MacKenzie ET, Fontaine M (2000). Glial responses, clusterin, and complement in permanent focal cerebral ischemia in the mouse. Glia.

[B27] Falsig J, Latta M, Leist M (2004). Defined inflammatory states in astrocyte cultures: correlation with susceptibility towards CD95-driven apoptosis. J Neurochem.

[B28] Ridet JL, Malhotra SK, Privat A, Gage FH (1997). Reactive astrocytes: cellular and molecular cues to biological function. Trends Neurosci.

[B29] Lang B, Liu HL, Liu R, Feng GD, Jiao XY, Ju G (2004). Astrocytes in injured adult rat spinal cord may acquire the potential of neural stem cells. Neuroscience.

[B30] Goldshmit Y, Galea MP, Wise G, Bartlett PF, Turnley AM (2004). Axonal regeneration and lack of astrocytic gliosis in EphA4-deficient mice. J Neurosci.

[B31] Sobel RA (2005). Ephrin A receptors and ligands in lesions and normal-appearing white matter in multiple sclerosis. Brain Pathol.

[B32] Sandvig A, Berry M, Barrett LB, Butt A, Logan A (2004). Myelin-, reactive glia-, and scar-derived CNS axon growth inhibitors: expression, receptor signaling, and correlation with axon regeneration. Glia.

[B33] Sappington RM, Chan M, Calkins DJ (2006). Interleukin-6 protects retinal ganglion cells from pressure-induced death. Invest Ophthalmol Vis Sci.

[B34] Schmidt JF, Agapova OA, Yang P, Kaufman PL, Hernandez MR (2007). Expression of ephrinB1 and its receptor in glaucomatous optic neuropathy. Br J Ophthalmol.

[B35] Chou YC, Lin SB, Tsai LH, Tsai HI, Lin CM (2003). Cholesterol deficiency increases the vulnerability of hippocampal glia in primary culture to glutamate-induced excitotoxicity. Neurochem Int.

[B36] Johnson EC, Jia L, Cepurna WO, Doser TA, Morrison JC (2007). Global changes in optic nerve head gene expression after exposure to elevated intraocular pressure in a rat glaucoma model. Invest Ophthalmol Vis Sci.

[B37] Wang X, Ng YK, Tay SS (2005). Factors contributing to neuronal degeneration in retinas of experimental glaucomatous rats. J Neurosci Res.

[B38] Levkovitch-Verbin H, Quigley HA, Martin KR, Harizman N, Valenta DF, Pease ME, Melamed S (2005). The transcription factor c-jun is activated in retinal ganglion cells in experimental rat glaucoma. Exp Eye Res.

[B39] Agapova OA, Kaufman PL, Hernandez MR (2006). Androgen receptor and NFkB expression in human normal and glaucomatous optic nerve head astrocytes in vitro and in experimental glaucoma. Exp Eye Res.

[B40] Ahmed F, Brown KM, Stephan DA, Morrison JC, Johnson EC, Tomarev SI (2004). Microarray analysis of changes in mRNA levels in the rat retina after experimental elevation of intraocular pressure. Invest Ophthalmol Vis Sci.

[B41] Steele MR, Inman DM, Calkins DJ, Horner PJ, Vetter ML (2006). Microarray analysis of retinal gene expression in the DBA/2J model of glaucoma. Invest Ophthalmol Vis Sci.

[B42] Kuehn MH, Kim CY, Ostojic J, Bellin M, Alward WL, Stone EM, Sakaguchi DS, Grozdanic SD, Kwon YH (2006). Retinal synthesis and deposition of complement components induced by ocular hypertension. Exp Eye Res.

[B43] Agapova OA, Yang P, Wang WH, Lane DA, Clark AF, Weinstein BI, Hernandez MR (2003). Altered expression of 3 alpha-hydroxysteroid dehydrogenases in human glaucomatous optic nerve head astrocytes. Neurobiol Dis.

[B44] Schmidt J, Agapova OA, Yang P, Kaufman PL, Hernandez MR (2007). Expression of EphrinB1 and its Receptor in Glaucomatous Optic Neuropathy. Br J Ophthalmol.

[B45] Falsig J, Porzgen P, Lund S, Schrattenholz A, Leist M (2006). The inflammatory transcriptome of reactive murine astrocytes and implications for their innate immune function. J Neurochem.

[B46] Yang P, Agapova O, Parker A, Shannon W, Pecen P, Duncan J, Salvador-Silva M, Hernandez MR (2004). DNA microarray analysis of gene expression in human optic nerve head astrocytes in response to hydrostatic pressure. Physiol Genomics.

[B47] Liu B, Chen H, Johns TG, Neufeld AH (2006). Epidermal growth factor receptor activation: an upstream signal for transition of quiescent astrocytes into reactive astrocytes after neural injury. J Neurosci.

[B48] Hernandez MR (2000). The optic nerve head in glaucoma: role of astrocytes in tissue remodeling. Prog Retin Eye Res.

[B49] Liang H, Baudouin C, Behar-Cohen F, Crisanti P, Omri B (2007). Protein kinase C-zeta mediates retinal degeneration in response to TNF. J Neuroimmunol.

[B50] Kirwan RP, Leonard MO, Murphy M, Clark AF, O'Brien CJ (2005). Transforming growth factor-beta-regulated gene transcription and protein expression in human GFAP-negative lamina cribrosa cells. Glia.

[B51] Yu H, Zhu X, Greenbaum D, Karro J, Gerstein M (2004). TopNet: a tool for comparing biological sub-networks, correlating protein properties with topological statistics. Nucleic Acids Res.

[B52] Lukashin AV, Lukashev ME, Fuchs R (2003). Topology of gene expression networks as revealed by data mining and modeling. Bioinformatics.

[B53] Kirillov E (2006). MetaCore: An Integrated Software Platform and Statistical Evaluation Method for Network Analysis and Mapping of Human High Throughput Data. Appl Bioinform.

[B54] Rulten SL, Ripley TL, Hunt CL, Stephens DN, Mayne LV (2006). Sp1 and NFkappaB pathways are regulated in brain in response to acute and chronic ethanol. Genes Brain Behav.

[B55] Garcion E, Wion-Barbot N, Montero-Menei CN, Berger F, Wion D (2002). New clues about vitamin D functions in the nervous system. Trends Endocrinol Metab.

[B56] Magrassi L, Bono F, Milanesi G, Butti G (1992). Vitamin D receptor expression in human brain tumors. J Neurosurg Sci.

[B57] Wiseman H (1993). Vitamin D is a membrane antioxidant. Ability to inhibit iron-dependent lipid peroxidation in liposomes compared to cholesterol, ergosterol and tamoxifen and relevance to anticancer action. FEBS Lett.

[B58] Shaulian E, Karin M (2002). AP-1 as a regulator of cell life and death. Nat Cell Biol.

[B59] Jones BW, Means TK, Heldwein KA, Keen MA, Hill PJ, Belisle JT, Fenton MJ (2001). Different Toll-like receptor agonists induce distinct macrophage responses. J Leukoc Biol.

[B60] Kawai T, Akira S (2006). TLR signaling. Cell Death Differ.

[B61] Takeuchi O, Akira S (2001). Toll-like receptors; their physiological role and signal transduction system. Int Immunopharmacol.

[B62] Rivieccio MA, John GR, Song X, Suh HS, Zhao Y, Lee SC, Brosnan CF (2005). The cytokine IL-1beta activates IFN response factor 3 in human fetal astrocytes in culture. J Immunol.

[B63] Dhandapani KM, Hadman M, De Sevilla L, Wade MF, Mahesh VB, Brann DW (2003). Astrocyte protection of neurons: role of transforming growth factor-beta signaling via a c-Jun-AP-1 protective pathway. J Biol Chem.

[B64] Wood LD, Parsons DW, Jones S, Lin J, Sjoblom T, Leary RJ, Shen D, Boca SM, Barber T, Ptak J (2007). The genomic landscapes of human breast and colorectal cancers. Science.

[B65] Delfino FJ, Boustead JN, Fix C, Walker WH (2003). NF-kappaB and TNF-alpha stimulate androgen receptor expression in Sertoli cells. Mol Cell Endocrinol.

[B66] Tam NN, Gao Y, Leung YK, Ho SM (2003). Androgenic regulation of oxidative stress in the rat prostate: involvement of NAD(P)H oxidases and antioxidant defense machinery during prostatic involution and regrowth. Am J Pathol.

[B67] Shibata N, Asayama K, Hirano A, Kobayashi M (1996). Immunohistochemical study on superoxide dismutases in spinal cords from autopsied patients with amyotrophic lateral sclerosis. Dev Neurosci.

[B68] Agapova OA, Malone PE, Hernandez MR (2006). A neuroactive steroid 5alpha-androstane-3alpha,17beta-diol regulates androgen receptor level in astrocytes. J Neurochem.

[B69] Malone PE, Hernandez MR (2007). 4-Hydroxynonenal, a product of oxidative stress, leads to an antioxidant response in optic nerve head astrocytes. Exp Eye Res.

[B70] Funk JL, Trout CR, Wei H, Stafford G, Reichlin S (2001). Parathyroid hormone-related protein (PTHrP) induction in reactive astrocytes following brain injury: a possible mediator of CNS inflammation. Brain Res.

[B71] Cowell RM, Plane JM, Silverstein FS (2003). Complement activation contributes to hypoxic-ischemic brain injury in neonatal rats. J Neurosci.

[B72] Wiggins AK, Shen PJ, Gundlach AL (2003). Delayed, but prolonged increases in astrocytic clusterin (ApoJ) mRNA expression following acute cortical spreading depression in the rat: evidence for a role of clusterin in ischemic tolerance. Brain Res Mol Brain Res.

[B73] Edwards AO, Ritter R, Abel KJ, Manning A, Panhuysen C, Farrer LA (2005). Complement factor H polymorphism and age-related macular degeneration. Science.

[B74] Tornqvist E, Liu L, Aldskogius H, Holst HV, Svensson M (1996). Complement and clusterin in the injured nervous system. Neurobiol Aging.

[B75] Zenkel M, Kruse FE, Junemann AG, Naumann GO, Schlotzer-Schrehardt U (2006). Clusterin deficiency in eyes with pseudoexfoliation syndrome may be implicated in the aggregation and deposition of pseudoexfoliative material. Invest Ophthalmol Vis Sci.

[B76] Xie Z, Harris-White ME, Wals PA, Frautschy SA, Finch CE, Morgan TE (2005). Apolipoprotein J (clusterin) activates rodent microglia in vivo and in vitro. J Neurochem.

[B77] Han BH, DeMattos RB, Dugan LL, Kim-Han JS, Brendza RP, Fryer JD, Kierson M, Cirrito J, Quick K, Harmony JA (2001). Clusterin contributes to caspase-3-independent brain injury following neonatal hypoxia-ischemia. Nat Med.

[B78] Stephanou A (2004). Role of STAT-1 and STAT-3 in ischaemia/reperfusion injury. J Cell Mol Med.

[B79] Calo V, Migliavacca M, Bazan V, Macaluso M, Buscemi M, Gebbia N, Russo A (2003). STAT proteins: from normal control of cellular events to tumorigenesis. J Cell Physiol.

[B80] Lee JH, Park EJ, Kim OS, Kim HY, Joe EH, Jou I (2005). Double-stranded RNA-activated protein kinase is required for the LPS-induced activation of STAT1 inflammatory signaling in rat brain glial cells. Glia.

[B81] Bhattacharya SK, Crabb JS, Bonilha VL, Gu X, Takahara H, Crabb JW (2006). Proteomics implicates peptidyl arginine deiminase 2 and optic nerve citrullination in glaucoma pathogenesis. Invest Ophthalmol Vis Sci.

[B82] Ekins SNY, Bugrim A, Kirillov E, Nikolskaya T (2007). Pathway mapping tools for analysis of high content data. High Content Screening Handbook.

[B83] Stasi K, Nagel D, Yang X, Wang RF, Ren L, Podos SM, Mittag T, Danias J (2006). Complement component 1Q (C1Q) upregulation in retina of murine, primate, and human glaucomatous eyes. Invest Ophthalmol Vis Sci.

[B84] Tezel G, Yang X, Cai J (2005). Proteomic identification of oxidatively modified retinal proteins in a chronic pressure-induced rat model of glaucoma. Invest Ophthalmol Vis Sci.

[B85] Picklo MJ, Olson SJ, Markesbery WR, Montine TJ (2001). Expression and activities of aldo-keto oxidoreductases in Alzheimer disease. J Neuropathol Exp Neurol.

[B86] Arai K, Nishiyama N, Matsuki N, Ikegaya Y (2001). Neuroprotective effects of lipoxygenase inhibitors against ischemic injury in rat hippocampal slice cultures. Brain Res.

[B87] Sun GY, Xu J, Jensen MD, Yu S, Wood WG, Gonzalez FA, Simonyi A, Sun AY, Weisman GA (2005). Phospholipase A2 in astrocytes: responses to oxidative stress, inflammation, and G protein-coupled receptor agonists. Mol Neurobiol.

[B88] Herrmann O, Baumann B, de Lorenzi R, Muhammad S, Zhang W, Kleesiek J, Malfertheiner M, Kohrmann M, Potrovita I, Maegele I (2005). IKK mediates ischemia-induced neuronal death. Nat Med.

[B89] Kitaoka Y, Kumai T, Kitaoka Y, Lam TT, Munemasa Y, Isenoumi K, Motoki M, Kuribayashi K, Kogo J, Kobayashi S, Ueno S (2004). Nuclear factor-kappa B p65 in NMDA-induced retinal neurotoxicity. Brain Res Mol Brain Res.

[B90] Pizzi M, Sarnico I, Boroni F, Benetti A, Benarese M, Spano PF (2005). Inhibition of IkappaBalpha phosphorylation prevents glutamate-induced NF-kappaB activation and neuronal cell death. Acta Neurochir Suppl.

[B91] Schneider A, Martin-Villalba A, Weih F, Vogel J, Wirth T, Schwaninger M (1999). NF-kappaB is activated and promotes cell death in focal cerebral ischemia. Nat Med.

[B92] Davies AM (2003). Regulation of neuronal survival and death by extracellular signals during development. Embo J.

[B93] Huang Y, Krein PM, Muruve DA, Winston BW (2002). Complement factor B gene regulation: synergistic effects of TNF-alpha and IFN-gamma in macrophages. J Immunol.

[B94] Molina-Holgado E, Arevalo-Martin A, Castrillo A, Bosca L, Vela JM, Guaza C (2002). Interleukin-4 and interleukin-10 modulate nuclear factor kappaB activity and nitric oxide synthase-2 expression in Theiler's virus-infected brain astrocytes. J Neurochem.

[B95] Choi JS, Kim JA, Kim DH, Chun MH, Gwag BJ, Yoon SK, Joo CK (2000). Failure to activate NF-kappaB promotes apoptosis of retinal ganglion cells following optic nerve transection. Brain Res.

[B96] Takahashi Y, Katai N, Murata T, Taniguchi SI, Hayashi T (2007). Development of spontaneous optic neuropathy in NF-kappaBetap50-deficient mice: requirement for NF-kappaBetap50 in ganglion cell survival. Neuropathol Appl Neurobiol.

[B97] Segre D, Deluna A, Church GM, Kishony R (2005). Modular epistasis in yeast metabolism. Nat Genet.

[B98] Mi H, Barres BA (1999). Purification and characterization of astrocyte precursor cells in the developing rat optic nerve. J Neurosci.

[B99] Yang P, Hernandez MR (2003). Purification of astrocytes from adult human optic nerve heads by immunopanning. Brain Res Brain Res Protoc.

[B100] Pena JD, Netland PA, Vidal I, Dorr DA, Rasky A, Hernandez MR (1998). Elastosis of the lamina cribrosa in glaucomatous optic neuropathy. Exp Eye Res.

[B101] Yucel YH, Kalichman MW, Mizisin AP, Powell HC, Weinreb RN (1999). Histomorphometric analysis of optic nerve changes in experimental glaucoma. J Glaucoma.

[B102] Ekins S, Nikolsky Y, Nikolskaya T (2005). Techniques: application of systems biology to absorption, distribution, metabolism, excretion and toxicity. Trends Pharmacol Sci.

[B103] Nikolsky Y, Ekins S, Nikolskaya T, Bugrim A (2005). A novel method for generation of signature networks as biomarkers from complex high throughput data. Toxicol Lett.

