# Supplementary material for: Network analysis of human glaucomatous optic nerve head astrocytes
Source: BMC Med Genomics. 2009 May 9;2:24. doi: 10.1186/1755-8794-2-24 (PMC2705386; doi:10.1186/1755-8794-2-24)
Supplement: Additional file 1 — Supplement Table S1. Clinical information of donor eyes used to generate cultures of ONH astrocytes. Supplement Table S2 Distributions for the top 12 canonical pathway maps and GO functional folders for a subset of genes up-regulated in glaucomatous astrocytes, fold change >2.5. Supplement Table S3. Description of the MetaCore™ network building algorithms and the Metacore network legend. Supplement Table S4. Down-regulation of integrins in the data sets from the two groups of patients. Supplement Table S5. Interactome analysis of connectivity for the top transcription factors on the network for upregulated genes. Supplement Table S6. Differential proteomics data for human glaucomatous optic nerves utilized in this study. Supplement Table S7. A list of literature-derived genes and proteins linked by genetic and other non-expression methods, and implicated in glaucoma ("G-set"). Supplement Table S8. Proximity dataset revealed using the AN networks generated from the G-set ("literature" genes). Supplement Table S9. Major parameters used for calculation of p-value for networks. Supplement Table S10. Statistical analysis of gene expression data. Supplement Table S11. Cluster size and corresponding p-value calculation for networks generated from 500 random nodes. Supplement Table S12. Validation of genes encoding major activated hubs in glaucomatous ONHAs by quantitative RT-PCR. [file 1755-8794-2-24-S1.pdf]

Supplemental Table 1. Clinical information of donor eyes used to generate cultures of ONH astrocytes

Donor ID	Gen der	Ag e	Medical history	COD	TOD	TOE	Severity of glaucoma	Glaucoma treatment	Experiments
**Caucasian American with POAG**									
**00-6L**	F	56	CVA, SAH	SAH	5:21	13:45	Mild	Latanoprost	MA, RT-PCR
**00-6R**	F	56	CVA, SAH	SAH	5:21	13:45	Mild	Latanoprost	MA, RT-PCR
**00-7L**	M	83	CAD, HTN	CHF	6:00	12:00	Moderate	Xalatan	MA, RT-PCR
**00-7R**	M	83	CAD, HTN	CHF	6:00	12:00	Moderate	Xalatan	MA, RT-PCR
**02-1R**	M	69	CHF	ARF	6:59	11:00	Moderate	NA	MA, RT-PCR
02-7L	M	71	HTN	CVA	23:50	3:55	Moderate	NA	RT-PCR
**02-10R**	F	80	Pneumonia	ARF	6:00	12:00	Advanced	NA	MA, RT-PCR
**04-1L**	F	79	COPD, CHF	CHF	21:15	7:35	Mild	NA	MA, RT-PCR
**04-5L**	F	72	CHF, MI	CHF, RF	5:22	10:35	Moderate	NA	MA, RT-PCR
04-16L	M	80	CHF, MI	CHF	23:30	2:30	Advanced	Alphagan, Cosopt	RT-PCR
05-2R	M	70	Prostate cancer	Cardiac arrest	1:18	4:15	Mild	Xalatan	RT-PCR
05-12R	F	77	Pancreatic cancer	Pancreatic cancer	21:22	23:55	Mild	Xalatan, Timolol Alphagan,	RT-PCR
**Donor eyes from Caucasian American without glaucoma**									
**99-9L**	F	46	Heart disease	Myocardial infarction	0:37	5:45	No signs	NA	MA, RT-PCR
**99-9R**	F	46	Heart disease	Myocardial infarction	0:37	5:45	No signs	NA	MA, RT-PCR
**99-6L**	M	42	None	Head trauma	21-06	23-30	No signs	NA	MA, RT-PCR
**03-11R**	F	68	Heart isease, bronchiectasis	Sepsis	16:20	20:30	No signs	NA	MA, RT-PCR
**03-13L**	F	72	Heart disease, HTN	Myocardial infarction	20:50	1:52	No signs	NA	MA, RT-PCR
03-1L	M	49	Hypotension	Metastatic cancer	4:25	8:35	No signs	NA	MA, RT-PCR
04-19L	M	50	Heart disease, HTN	Aspiration pneumonia	13:56	18:00	No signs	NA	RT-PCR
04-2R	M	65	Heart disease, HTN	Cardiac arrest	1:01	5:15	No signs	NA	RT-PCR
**04-2L**	M	65	Heart disease, HTN	Cardiac arrest	1:01	5:15	No signs	NA	RT-PCR
04-4L	M	42	Liver carcinoma	Hypotensio n, cancer	6:21	9:30	No signs	NA	RT-PCR
04-6L	M	57	HTN, throat cancer	Cardiac arrest	3:57	9:55	No signs	NA	RT-PCR
**LC46**	M	46	CVA	CVA	17:42	2:45	No signs	NA	RT-PCR
02-8R	F	73	Heart disease, renal insufficiency	CHF	0:40	9:25	No signs	NA	MA, RT-PCR
03-17L	M	56	Lung cancer	Cardiac arrest	21:18	2:30	No signs	NA	RT-PCR
04-22R	F	77	Renal failure, heart disease	Renal disease	19:30	22:03	No signs	NA	RT-PCR
04-23R	F	78	Subarachnoid hemorrhage	CVA	16:31	20:50	No signs	NA	RT-PCR
04-7R	M	58	HTN	Cardiopulm onary arrest	17:24	1:20	No signs	NA	RT-PCR

#Abbreviations and comments:

Severity of glaucoma was based on evaluation of the myelinated optic nerve and clinical ophthalmic history. COD: cause of death; TOD: time of death; TOE: time of enucleation. ARF: acute respiratory failure; CAD: coronary artery disease; CHF: chronic heart failure; COPD: chronic obstructive pulmonary disease; CVA: cerebral-vascular accident; GC: gastric cancer; HTN: hypertension. MI: Myocardial infarction, RF: renal failure, SAH: spontaneous aneurysm hemorrhage. None*: No treatment indicated in the charts. Eye drops**: indicated in the chart. NA: Data not available. MA: microarray, RT-PCR real time RT-PCR. Donors in **bold** were used for microarray analysis.

Evaluation of Nerve Damage

To evaluate optic nerve damage, cross sections were taken from the myelinated optic nerves fixed in 4% paraformaldehyde, osmicated, embedded in plastic and stained with *p*-phenylenediamine (50-51). Digital images were taken with at 2X magnification, so that the entire circumference of the nerve was within the lens field. Images were imported into Optimas software, where the total circumference area as well as the areas with axonal degeneration was measured. Results were expressed as a ratio between area of axon degeneration and total area. “Mild” axon loss was defined as a loss of up to one third of myelinated axon area, “moderate” axon loss when there was loss between one and two thirds of myelinated axon area, and “marked” axon loss when the loss in axon area surpassed two thirds of the total myelinated area.

Supplemental Table 2

A. Distributions for the top 12 canonical pathway maps and GO functional folders for a subset of genes up-regulated in glaucomatous astrocytes, fold change >2.5. The maps and GO folders are prioritized according to the score, which is inversely proportional to p-value.

Pathway maps *(128 mapped IDs)*	***hits/map***	***p-value***	***GO processes***	***hits/proces***	***p-value***
**group 1** (124 mapped IDs)			**group 1** (264 linked to processes)		
Bile acid biosynthesis	7 (32)	2.65E-06	Signal transduction	70/1493	1.42E-11
Serotonin-melatonin biosynthesis	9 (60)	2.71E-06	Cellular physiological response	16/114	1.34E-09
Leukotrien 4 biosynthesis	8 (158)	2.77E-06	Cell-cell signaling	26/351	1.50E-08
Catecholamine metabolism	13 (173)	5.82E-05	Circulation	13/76	4.10E-09
Ephrins signaling	6 (43)	7.22E-05	Complement activation	9/_35	2.48E-08
Role of AP-1 in regulation of cellular metabolism	6 (44)	2.02E-04	Alcohol metabolism	5/_7	7.09E-08
P53 signaling pathway	6 (48)	2.30E-04	Inflammatory response	21/269	1.61E-07
TLR signaling in cell proinflammatory response	5 (32)	3.73E-04	Serotonin receptor, PLC activating pat	4/_4	1.73E-07
IL6 signaling pathway	6 (57)	4.05E-04	Cell proliferation	25/377	2.45E-07
MIF in innate immunity response	4 (23)	9.50E-04	Positive regulation of NF-kB cascade	12/_89	2.53E-07
Alternative complement pathway	4 (23)	1.04E-03	cytokine production	6/_19	1.52E-06
ECM remodeling	6 (60)	2.14E-03	Nervous system development	21/344	8.47E-06
**group 2** (128 mapped IDs)			**group 2** (291 linked to processes**)**		
Regulation of RAC1 activity	21 (188)	4.85E-09	Immune response	33/460	4.29E-09
TCR and CD28 co-stimulation in activation of NF-kB	21 (212)	4.12E-08	Development	40/695	4.72E-08
Immunological synapse formation	23 (260)	7.60E-08	Lipid metabolism	19/197	1.08E-07
CD28 signaling	21 (224)	2.6E-07	Complement activation	8/_35	8.64E-07
NF-kB signaling pathway	19 (196)	1.07E-07	Cell adhesion	30/502	1.19E-06
Rap1A regulation pathway	19 (197)	5.98E-05	Regulation of cell migration	9 (49)	1.28E-06
Erk Interactions: Inhibition of Erk	21 (253)	6.99E-05	Germ cell migration	5 (10)	1.32E-06
Role of VDR in regulation of genes in osteoporosi	8 (58)	9.65E-04	Positive regulation of cell proliferation	18 (215)	1.89E-06
Bile acid biosynthesis	6 (32)	2.81E-03	Cell-cell signaling	23 (351)	4.97E-06
Androstenedione and testosterone biosynthesis	4 (19)	1.71E-03	Inflammatory response	19 (269)	1.18E-05
MAPK cascade. Nuclear function of p38-MAPK	2 (3)	6.32E-03	Signal transduction	58 (1493)	2.40E-05
Leukotrien 4 biosynthesis and metabolism	5 (60)	1.17E-02	Canalicular bile acid transport	3 (30)	1.15E-05
		**Merged non-redundant set**			
(168 mapped IDs)			(320 linked to processes)		
Bile acid biosynthesis	10(32)	7.82E-09	Complement activation	11 (35)	1.15E-09
Ephrin signaling	9 (58)	2.54E-05	Signal transduction	78 (1493)	6.03E-09
Alternative complement pathway	6 (23)	2.75E-05	Circulation	14 (76)	1.33E-08
Leukotrien 4 biosynthesis	9 (60)	3.36E-05	Innate immune response	12(60)	5.63E-08
Histamine metabolism	5 (18)	9.55E-05	Alcohol metabolism	5 (97)	2.85E-07
TLR signaling in cell proinflammatory response	7 (48)	3.70E-04	Serotonin receptor, PLC activating pat	4 (4)	5.30E-07
IL1 signaling pathway	6 (43)	1.05E-03	Nervous system development	27 (344)	7.30E-07
Serotonin-melatonin biosynthesis	10 (119)	1.57E-03	Inflammatory response	23 (269)	1.15E-06
Fatty Acid Omega Oxidation	4 (20)	1.89E-03	Cell proliferation	28 (377)	1.37E-06
NF-kB signaling pathway	5 (35)	2.47E-03	Chemotaxis	15 (125)	1.40E-06
IL22 signaling pathway	4 (24)	3.81E-03	Ephrin receptor signaling pathway	5 (9)	1.63E-06
Role of VDR in regulation of genes in osteoporosi	6 (58)	4.93E-03	Development	41 (695)	2.15E-06
		**Combined (redundant) set**			
**(**223 mapped**)**			(456 linked to GO processes)		
Bile acid metabolism	11 (32)	2.10E-08	Signal transduction	112 (1493)	7.68E-13

Leukotriene 4 biosynthesis and metabolism	13 (60)	4.87E-07	Cell-cell signaling	41(351)	1.59E-10
Immunological synapse formation	27 (260)	4.78E-06	Development	60 (695)	2.19E-09
Regulation of RAC1 activity	22 (188)	5.42E-06	Lipid metabolism	27/197	7.85E-09
TCR and CD28 cooperation in activation of NF-kE	23 (212)	1.19E-05	Immune response	44/460	1.83E-08
NF-kB signaling pathway	21 (196)	3.41E-05	Circulation	16/76	2.12E-08
Ephrins signaling	10 (58)	8.36E-05	Cell adhesion	46/502	3.20E-08
Role of VDR in regulation of genes in osteoporosi	10 (58)	8.37E-05	Inflammatory response	31/269	4.01E-08
MAPK cascade.Nuclear function of p38-MAPK	9 (49)	1.14E-04	Cytokine production	8 (19)	2.24E-07
Histamine metabolism	5 (18)	5.40E-04	Cellular physiological process	18/114	2.97E-07
TLR signaling in cell proinflammatory response	8 (48)	5.44E-04	Complement activation	10 (35)	4.71E-07
Androstenedione and testosterone biosiynthesis	5 (19)	7.09E-04	Nervous system development	33/344	1.10E-06

B. Distributions for the top 12 canonical pathway maps and GO functional folders for a subset of genes down-regulated in glaucomatous astrocytes, fold change >2.5. The maps and GO folders are prioritized according to the score, which is inversely proportional to p-value.

**Pathway maps**	hits/map	**p-value**	**GO processes**	hits/proces	**p-value**
**Group 1** (59 mapped IDs)			**Group 1** (130 linked to GO processes)		
Chemokines and adhesion	8 (173)	2.43E-04	signal transduction	46 (1493)	7.42E-12
PH proteins participation in RTKs adaptor comple	5 (64)	3.50E-04	Regulation of smooth muscle contracti	6 (15)	5.87E-09
PDGF signaling via STATs and NF-kB	4 (40)	5.49E-04	Neuron migration	8 (50)	4.78E-08
PLAU signaling	4 (47)	1.02E-03	transmembrane receptor PTK signalin	10 (96)	6.69E-08
IL10 signaling pathway	3 (23)	1.30E-03	oocyte maturation	4 (6)	1.78E-07
Putative erythropoetin signaling pathway	4 (52)	1.49E-03	cell proliferation	17 (377)	4.85E-07
Cytoskeleton remodeling	7 (177)	1.50E-03	axon guidance	8 (75)	1.20E-06
Role of PDGFs in cell migration	3 (25)	1.66E-03	cell adhesion	19 (502)	1.41E-06
Signaling pathway mediated by PDGF	4 (56)	1.96E-03	regulation of progression through cell	16 (373)	2.07E-06
PH proteins and focal adhesion complex	4 (62)	2.85E-03	organ morphogenesis	12 (240)	9.15E-06
PDGF signaling via MAPK cascades	4 (63)	3.02E-03	regulation of angiogenesis	4 (14)	1.11E-05
Regulation of Apoptosis by Mitochondrial Proteins	3 (31)	3.20E-03	muscle development	8 (108)	1.87E-05
PH protein interactions	4 (64)	3.38E-03	cellular physiological process	8 (114)	2.78E-05
**Group 2** (81 mapped IDs)			**Group 2** (213 linked to GO processes)		
HETE and HPTE biosyntesis	4 (42)	5.18E-03	Synaptic transmission	26 (259)	8.71E-13
Role of VDR in regulation of genes involved in ost	4 (58)	1.60E-02	Nervous system development	27 (344)	9.91E-11
A2B receptor: action via G-alpha	4 (60)	1.79E-02	Positive regulation of heart contraction	6 (8)	8.27E-10
Leukotrien 4 biosynthesis and metabolism	4 (60)	1.79E-02	Increased strength of heart contractior	6 (8)	8.27E-10
Androstenedione and testosterone biosynthesis	2 (17)	3.70E-02	Blood pressure regulation	12 (66)	1.67E-09
Catecholamin metabolism	3 (44)	3.21E-02	smooth muscle contraction	9 (32)	3.11E-09
Transcription regulation of granulocyte developme	3 (45)	3.92E-02	Elevation of cytosolic calcium	12 (74)	6.56E-09
Tyrosine metabolism	2 (19)	3.95E-02	ion transport	27 (429)	1.26E-08
EphB receptors in dendritic spine morphogenesis	4 (81)	4.67E-02	signal transduction	57 (1493)	1.59E-08
Role of PKA in cytoskeleton reorganization	4 (82)	4.85E-02	circulation	11 (76)	9.61E-08
Integrin-mediated cell adhesion	4 (93)	5.88E-02	regulation of sodium ion transport	4 (4)	9.91E-08
PH protein interactions	3 (64)	9.17E-02	muscle development	12 (108)	4.88E-07
		**Merged non-redundant set**			
(43 mapped IDs**)**			(103 linked to GO processes)		
Membrane trafficking and signal transduction of G	3 (37)	3.50E-04	signal transduction	36/1493	1.02E-08
PDGF signaling via MAPK cascades	4 (63)	1.20E-03	oocyte maturation	4 (6)	8.63E-08
Role of PKA in cytoskeleton re-organization	4 (82)	3.17E-03	smooth muscle contraction	5 (15)	1.40E-07

VEGF-family signaling	3 (45)	4.46E-03	neuron migration	7 (50)	2.59E-07
Signaling Pathway mediated by PDGF	3 (56)	8.21E-03	nervous system development	15 (344)	3.58E-07
Chemokines and adhesion	5 (173)	9.18E-03	locomotory behavior	7 (54)	4.46E-07
ATP metabolism	3 (59)	9.48E-03	glutamate signaling pathway	5 (20)	6.96E-07
Cytoskeleton remodeling	5 (177)	1.01E-02	CNS development	8 (106)	4.34E-06
PH proteins and focal adhesion complex	3 (64)	1.09E-02	negative regulation of macrophage dif	3 (5)	6.67E-06
PH proteins and interaction with integrin receptors	3 (62)	1.09E-02	hemoglobin biosynthesis	3 (5)	6.67E-06
EGF signaling pathway	3 (64)	1.18E-02	ovarian follicle development	4 (15)	7.38E-06
Pleckstrin homology proteins interactions.	3 (64)	1.18E-02	antigen presentation, endogenous ant	5 (33)	9.71E-06

Combined (redundant) set

(130 mapped IDs)			(324 linked to GO processes)		
A2B receptor: action via G-alpha	6 (60)	3.71E-03	Signal transduction	93/1493	1.03E-15
Chemokines and adhesion	11 (173)	4.08E-03	Nervous system development	35/344	7.96E-12
Role of PKA in cytoskeleton reorganzation	7 (82)	4.08E-03	Synaptic transmission	30/259	1.04E-11
PH proteins in RTKs adaptor complexes	6 (64)	4.31E-03	Regulation of smooth muscle contracti	9 (15)	2.58E-11
Putative Erythropoietin Signaling Pathway	5 (52)	5.11E-03	Locomotory behavior	13/54	1.09E-09
Cytoskeleton remodeling	10 (177)	9.32E-03	muscle development	17/108	3.38E-09
ATP metabolism	5 (59)	1.24E-02	Positive regulation of heart contraction	6 (8)	9.00E-09
ECM remodeling	5 (60)	1.56E-02	Increased strength of heart contraction	6 (8)	9.00E-09
PDGF signaling via STATs and NF-kB	4 (40)	1.56E-02	Neuron migration	11 (50)	5.86E-08
IL10 signaling pathway	3 (23)	1.71E-02	Elevation of cytosolic calcium	13/74	6.35E-08
PH proteins and focal adhesion complex formatio	5 (62)	1.90E-02	Blood pressure regulation	12 (66)	1.38E-07
PDGF signaling via MAPK cascades	5 (63)	2.02E-02	Regulation of heart contraction	11 (56)	2.02E-07

	**Color code**
(bright green)	Present in at least 3 files
(light green)	Present in at least 2 files
(cyan)	unique

Supplemental Table 3. Algorithms and full legend for networks

Algorithm	How is it implicated	Relative Stringency	Potential applications
Analyze Network (**AN**)	Generates sub-networks highly saturated with selected objects. Sub-networks are ranked by a P-value and G-Score and interpreted in terms of Gene Ontology	+ (adjustable)	to detect subnetworks most relevant to a set of objects
Shortest Path (**SP**)	Uses Dijkstra's shortest paths algorithm to find the shortest directed paths between the selected objects	++	to determine potential pathways linking upstream and downstream objects
Direct Interactions (**DI**)	Draws direct interactions between selected objects. No additional objects are added to the network	+++	to detect interconnected clusters among selected objects
Auto Expand (**AE**)	Draws sub-networks around selected objects, stopping the expansion when the sub-networks intersect	+	to find an extended network environment for a set of selected objects
Analyze Transcription Regulation (**ATR)**	Generates sub-networks centered on transcription factors. Sub-networks are ranked by a P-value and interpreted in terms of Gene Ontology	+ (adjustable)	to detect common transcriptional regulation cascades for a set of objects
Self Regulations (**SR**)	Finds the shortest directed paths containing transcription factors between the selected objects	++	To detect mutual transcriptional regulation for a set of objects
Expand by One Interaction (**EOI**)	Finds the shortest directed paths containing transcription factors between the selected objects	+	To find immediate network environment for an object

Networks can be generated using the following types of input data:

- Gene/protein/object lists including experimental and user-created lists
- Map- and GO process-specific gene/protein lists generated in MetaCore;
- Disease-focused gene lists
- Pre-built networks for major cellular processes

NETWORK LEGEND

Network Objects

Click on any object in the network to obtain class info

Enzymes			
	Generic enzyme		
KINASE		PHOSPHATASE	
	Generic kinase		Generic phosphatase
	Generic protein kinase		Generic protein phosphatase
	Generic lipid kinase		Generic lipid phosphatase
PHOSPHOLIPASE			
	Generic phospholipase		
PROTEASE		GTPase	
	Generic protease		G-alpha
	Generic metalloprotease		RAS - superfamily

Generic classes	
	Receptor ligand
	Transcription factor
	Protein
	Cell membrane glycoprotein
	Anchoring phospholipid
	Molecule
	Inorganic ion

Channels / Transporters	
	Generic channel
	Ligand-gated ion channel
	Voltage-gated ion channel
	Transporter

Receptors	
	Generic
	GPCR
	Receptors with kinase activity
	Nuclear receptor

G protein adaptor / regulators	
	G beta/gamma
	Generic (RGS, GDI, GAP, GAF, GRF, ARF)
	Heterotrimeric G-protein

Adaptors / regulators	
	Generic binding protein
	Adaptor

Groups of objects	
	**Group of related object(s)**<br>Group of objects with common properties
	**Protein complex**<br>Group of proteins physically connected in a complex
Group 1	**User created group**<br>Group of collapsed objects chosen by user

Object highlighting

Nodes and root nodes

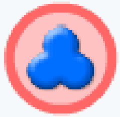

Found object

Object selected on the search pane

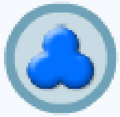

Manually selected node(s)

Object(s) selected by ctrl + click on it or by click + drag rectangle around it

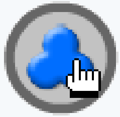

Highlight by mouse over

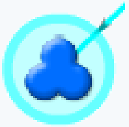

Highlight upstream objects

When the mouse is over an object (node on a network), the closest interacting nodes are highlighted in CYAN if the direction of interaction is **from** the initial object

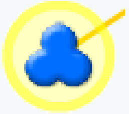

Highlight downstream objects

When the mouse is over an object (node on a network), the closest interacting nodes are highlighted in yellow if the direction of interaction is **towards** the initial object

Root nodes

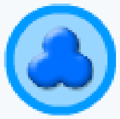

Root node(s) for network expansion (building)

Object(s) from a user-specified uploaded list or from experiments

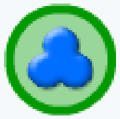

Initial object(s)

Object(s) chosen to build the pathways **from**

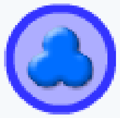

Intermediate object(s)

Object(s) situated along the pathway

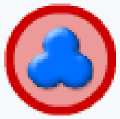	**Terminal object(s)**<br>Object(s) the pathways terminate **on**
	**Possible combinations of three above marks (except the first one)**<br>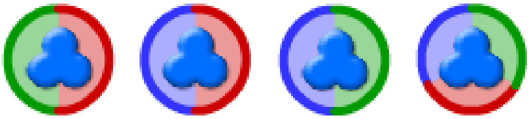

Expression data	
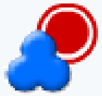	**Overexpressed gene(s)**<br>Genes with higher conditional expression level compared to the experimental "control"
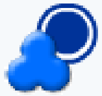	**Underexpressed gene(s)**<br>Genes with lower conditional expression level compare to the experimental "control"
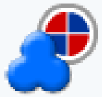	**Mixed-expressed gene(s)**<br>Genes with conditional expression level statistically different from the experimental "control", with the "sign" of expression varying in different experiments

Other marks	
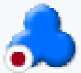	**Red circle**<br>The links terminated due to a restriction of the number of steps in network expansion.<br>Network may be expanded from such nodes
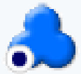	**Blue circle**<br>The links terminated due to network truncation.<br>Network may be expanded from such nodes

Interactions between objects

Click on any hexagon in the networks for interaction annotation

Functional interactions	
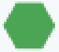	**Positive effect**
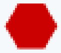	**Negative effect**
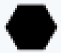	**Unspecified effect**

Mechanisms	
**Direct interactions**	
A	**Allosteric regulation**<br>Compound binds to the allosteric site of enzyme in a non-covalent manner and alters it's activity
B	**Binding**<br>Compound binds the enzyme or receptor
C	**Cleavage**<br>Cleavage of a protein at a specific site yielding distinctive peptide fragments.<br>Proteolytic cleavage can be carried out by both enzymes and compounds
CM	**Covalent modifications**<br>Protein activity regulation by covalent binding of a small chemical group to the aminoacids of an active site.
+P	**Phosphorylation**<br>Protein activity is altered via addition of a phosphate group
-P	**Dephosphorylation**<br>Protein activity is altered via removal of a phosphate group
T	**Transformation**

Tn	**Translocation**
Z	**Catalysis**
Tr	**Transcription regulation**
S	**Substrate**<br>Enzyme may attenuate the compound's regulatory effect by decreasing its level
P	**Product**
**Indirect interactions**	
IE	**Influence on expression**<br>Compounds change the expression level of target genes indirectly, for instance by binding to upstream receptors
?	**Unspecified interactions**<br>Mechanism is unknown
**Logical relations**	
CR	**Class relation**<br>Object belongs to a generic group of related objects
CS	**Complex subunit**<br>Active protein is a subunit of a protein complex

Connectors

Connectors	
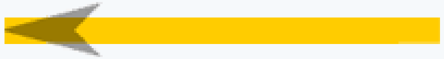	**Incoming interaction**<br>When the mouse is over an object, yellow link indicates direction **to** the object
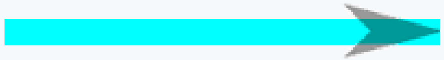	**Outgoing interaction**<br>Cyan link indicates direction **from** the object
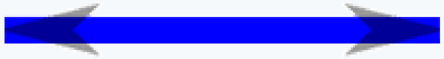	**Bidirectional interaction**<br>Blue link indicates **BI-DIRECTIONAL** interaction
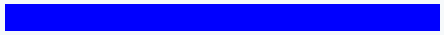	**Non-directional link**<br>Blue link also indicates an interaction for which the direction is not specified
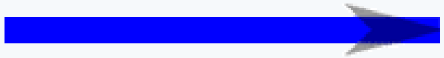	**Traced link**<br>The link is always highlighted in blue if both linked objects are selected in "Trace" mode

Custom marked links (user's choice)

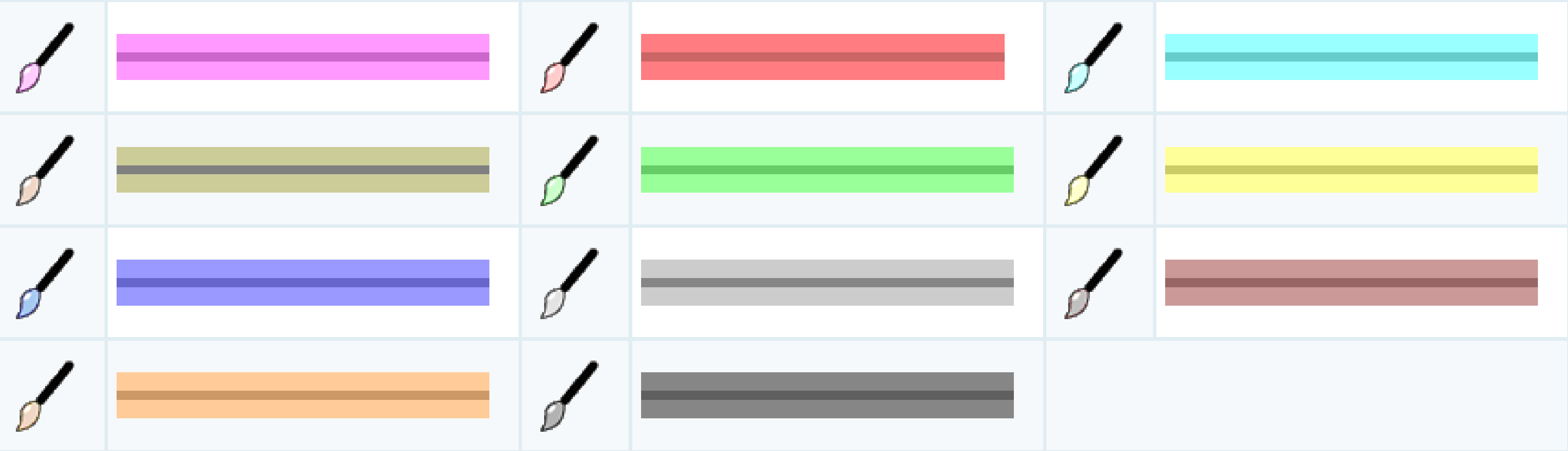

Supplement Table 4. Down-regulation of integrins in the data sets from the two groups of donors.

Gene ID	*Group 1*	*Group2*
ITGA1	-2.1	-
ITGA2	-	-2.1
ITGA2B	-	-1.8
ITGA3	-2.9	-1.7
ITGA4	-13.4	-3.7
ITGA6	-9.3	-
ITGA7	-2.4	-2.1
ITGA9	-	-2.1
ITGAV	-	-2.2
ITGB5	-2.1	-1.6
ITGB7	-	-1.9
ITGA2	-	-2.6

Genes changed in both groups are bolded

Supplement Table 5. Over-connected transcription factors, upregulated genes 2.5-fold

node	*r*	*R*	*mean*	*p-value*	*over-connection ratio (r/mean)*
**NF-kB2 (p100)**	3	13	0.1916	0.00081	15.65765293
**c-Rel (NF-kB subunit)**	10	80	1.1791	2.8E-07	8.481228669
**NF-kB2 (p52)**	4	40	0.5895	0.00278	6.784982935
**JunD**	4	65	0.958	0.01549	4.175374114
**c-Fos**	11	186	2.7413	0.0001	4.012624316
**VDR**	8	133	1.9602	0.00081	4.081192743
**Androgen receptor**	16	507	7.4724	0.00367	2.141217494

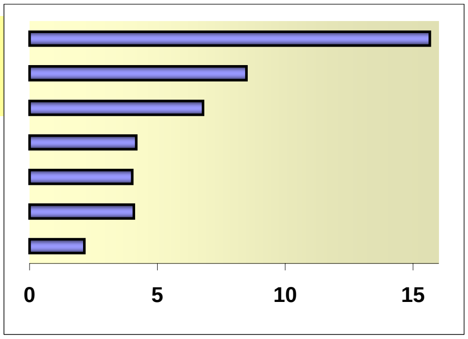

Supplemental Table 6. Differential proteomics data from human glaucomatous optic nerves.

Protein ID#	Common name	Matches	Freq.
Proteins specific to ONHs with glaucoma			
P02511*	Alpha crystallin B chain	5	7
Q9Y2J8*	Protein-arginine deiminase type II	3	4
P01842	Ig lambda chain C regions	2	4
P13591*	**NCAM 1, 140 kDa isoform precurs**	2	4
P68104*	Elongation factor 1-alpha 1	2	4
P21333*	Filamin A	5	2
P33778*	Histone H2B.f	4	2
P50395*	Rab GDP dissociation inhibitor beta	4	2
P31946*	14-3-3 protein beta/alpha	3	2
Q14697*	Neutral alpha-glucosidase AB precursor	3	2
P12273*	**Prolactin-inducible protein precursor**	2	2
P17174*	Aspartate aminotransferase, cytoplasmic	2	2
P34932*	Heat shock 70 kDa protein 4	2	2
P53674*	Beta crystallin B1	2	2
Q13938*	Calcyphosine	2	2
Q16378	Proline-rich protein 4 precursor	2	2
Q9BPU6*	Dihydropyrimidinase related protein-5	2	2
P48666*	Keratin, type II cytoskeletal 6C	9	1
P04745	Alpha-amylase	7	1
Q9NP55	Protein Plunc precursor	5	1
P00751*	**Complement factor B precursor**	4	1
P13646*	Keratin, type I cytoskeletal 13	4	1
P01877	Ig alpha-2 chain C region	3	1
P08603*	**Complement factor H precursor**	3	1
P11217*	Glycogen phosphorylase, muscle form	3	1
P17317	Histone H2A.z	3	1
P34931*	Heat shock 70 kDa protein 1-HOM	3	1
Q9NZT1*	Calmodulin-like protein 5	3	1
Q9Y281	Cofilin, muscle isoform	3	1
Q9Y490	Talin 1	3	1
O75891*	10-formyltetrahydrofolate dehydrogenase	2	1
P00491	Purine nucleoside phosphorylase	2	1
P00568*	Adenylate kinase isoenzyme 1	2	1
P01833	Polymeric-immunoglobulin receptor precursor	2	1
P02489*	Alpha crystallin A chain	2	1
P02814*	Proline-rich protein 3 precursor	2	1
P23527*	Histone H2B.n	2	1
P30044	Peroxiredoxin 5	2	1
P31944	Caspase-14 precursor	2	1
P35558*	Phosphoenolpyruvate carboxykinase	2	1
P46940*	Ras GTPase-activating-like protein IQGAP1	2	1
P47929*	Galectin-7	2	1
P51148*	Ras-related protein Rab-5C	2	1
P55786*	Puromycin-sensitive aminopeptidase	2	1
P62158*	Calmodulin	2	1
P81605	Dermicidin precursor	2	1
Q16778*	Histone H2B.q	2	1
Q9BXN1	Asporin precursor	2	1
Q9Y4W6	AFG3-like protein 2	2	1
Proteins specific to normal ONHs			
P02765*	Alpha-2-HS-glycoprotein precursor		
P55087*	Aquaporin 4		
O43852	Calumenin precursor		
P09972*	Fructose-bisphosphate aldolase C		
P09972*	Glutathione S-transferase Mu 2		

P02788 *	Lactotransferrin precursor
P13590*	NCAM 1, 180 kDa isoform precursor
P36955*	Pigment epithelium-derived factor precursor
P21980*	Protein-glutamine gamma-glutamyltransferase
P09493*	Tropomyosin 1 alpha chain
P67936*	Tropomyosin 4 alpha chain
P26640	Valyl-tRNA synthetase

**Proteins that appear on the Metacore maps and networks revealed by the DE data*
***Bolded** proteins that validate the gene expression data*

Supplemental Table 7. A list of literature-derived genes and proteins linked by genetic and other non-expression methods, and implicated in glaucoma (**"G-set")**.

MetaCore symbol	*Gene symbol*	*Entrez Gene ID*	*Gene product*	*PMID Reference #*
**AGTR2**	AGTR2	186	angiotensin II receptor	15914614
**APOE**	APOE	348	Chylomicron (VLDL)	15525904 ; 15031182; 15031162; 14769603; 12379839
**APP**	APP	351	apolipoprotein precusor.	12957857; 15838725; 11923249
**BDNF**	BDNF	627	brain derived nerve growth factor	15579199; 15105791
**BMP4**	BMP4	652	bone morphogenetic protein 4	11722794
**CA12**	CA12	771	Carbonic anhydrase 12	12676895
**CASP3**	CASP3	836	caspase 3	12957857; 11923249
**CASP8**	CASP8	841	caspase 8	12957857; 11923249
**CASP9**	CASP9	842	caspase 8	12789547
**CDH1**	E-cadherin	999	E-cadherin	16276119
**CDKN1A**	CDKN1A	1026	cyclin-dependent kinase inhibitor 1A	14738489
**c-FOS**	FOS	2353	FOS, FOSB, FOSL1, and FOSL2	14747662; 12021818; 16081055
**c-JUN**	JUN	3725	similar to avian sarcoma virus gene17	16081055; 16273539
**collagen type I**	COL1A1	1277	collagen type I	15161848; 8500565; 15623771; 10223659; 8983948; 8217094; 8500565
**Connexin 43**	GJA1	2697	Gap junction protein, alpha 1	10670473; 12868015
**COX1**	PTGS1	5742	cyclohexogenase-1	11391707
**COX-2**	PTGS2	5743	cyclohexogenase-2	14738509; 14738509 ; 11391707
**CSPG4**	CSPG4	1464	chondroitin sulfate proteoglycan	11820702
**CYP1B1**	CYP1B1	1545	cytochrome P450 superfamily of enzymes.	15558492; 15475877; 15723004; 14640115; 15342693; 15342693; 14507861; 9097971
**DSPG3**	DSPG3	1833	Dermatan sulfate proteoglycan3	9479524; 8983948; 8157116
**EGFR**	EGFR	1956	epidermal growth factor receptor	16837601; 15328014; 15042583
**eIF5A**	EIF5A	1984	eukaryotic translation initiation factor 5A	15452064
**ELN**	ELN	2006	elastin	11527944; 8045433; 8112998; 1911659; 2405683 ; 2653045
**EDNRA**	EDNRA	1909	Endothelin-1 receptor precursor	15988412
**eNOS**	NOS3	4846	endothelial nitric oxide synthase 3	9109759; 9493554
**E-selectin**	SELE	6401	endothelial leukocyte adhesion molecule-1	12714632 ; 11231628
**ET1**	EDN1	1906	endothelin 1	15161847 ; 14578413; 12356828; 12147606
**FBN1**	FBN1	2200	fibrillin 1	10870517 ; 7826283
**FN1**	FN1	2335	fibronectin 1	10070530; 1434040; 2654504; 3891665; 6771728
**FOXC1**	FOXC1	2296	forkhead family transcription factor C1	12036988; 11880716; 11170889; 11007653; 9620769; 10767326
**GFAP**	GFAP	2670	intermediate filament protein	15185390; 15183107 ;
**HSPB1 = HSP27**	HSPB1	3315	heat shock 27kDa protein 1	11599002 ; 11391707; 11158802; 10804196

**GSTM1**	GSTM1	2944	thione S-transferase Mu 1	11040079
**IGF2**	IGF2	3481	Insulin-like growth factor II precursor	14614750
**IL1B**	IL1B	3553	interleukin 1 beta	11747366; 11231628
**IL1RN**	IL1RN	3557	interleukin 1 receptor	12913327
**iNOS**	NOS2A	4843	nitric oxide synthase 2, inducible	11176986; 10719359; 10449799; 9109759
**LMX1B**	LMX1B	4010	LIM homeobox transcription factor 1, beta	15558492; 10660670; 9618165
**LOXL1**	LOXL1		lysyl oxidase–like protein 1	17690267; 18385788
**MMP1**	MMP1	4312	matrix metalloproteinase 1	12650974; 11483084; 11241738
**MMP14**	MMP14	4323	matrix metalloproteinase 14	12650974; 11241738
**MMP9**	MMP9	4318	matrix metalloproteinase 9 .	12650974; 12354772
**MTHFR**	MTHFR	4524	Methylenetetrahydrofolate reductase	15808177
**MYOC (TIGR)**	MYOC	4653	myocilin	9300658; 9005853; 12912696
**NCAM1**	NCAM1	4684	neural cell adhesion molecule 1	11000479; 10640677
**NT-3**	NTF3	4908	neurotrophin 3	15579199 ; 15105791
**OLFM3**	OLFM3	118427	olfactomedin (optimedin)	12019210; 15123989
**OPA1**	OPA1	4976	optic atrophy type 1 protein	12073024; 11810296
**OPTN**	OPTN	10133	optineurin	15557444 ; 15326130; 14764620; 14740994; 11834836; 14627677; 12939304
**PALB**	TTR	7276	transthyretin (prealbumin, amyloidosis type I)	12617705; 12557757
**PAX6**	PAX6	5080	PAX6 homeo box protein	12351165
**PITX2**	PITX2	5308	RIEG/PITX homeobox protein	15558492 ; 11880716;
**PKC-MU**	PRKD1	5587	protein kinase C mu (PKCm)	11687525
**PLC-beta**	PLCB1	23236	phospholipase C-beta(1)	11923256
**RELA**	RelA/p65	5970	RelA/p65 protein	16310187
**SBF2**	SBF2	81846		15304601
**SLC4A4**	SLC4A4	8671	Sodium bicarbonate cotransporter A4	15471865; 11274232
**SNCG**	SNCG	6623	gamma synuclein	16392033
**STAT3**	STAT3	6774	STAT protein	15183107; 14725620
**TAP1**	TAP1 (PSF1)	6890	Antigen peptide transporter 1	15887980
**TGFB2**	TGFB2	7042	transforming growth factor, beta 2	15165125; 15085971
**TIMP1**	TIMP1	7076	tissue inhibitor of the matrix metalloproteinases	12650974; 11241738; 7587299
**TNC**	TNC	3371	tenascin C (hexabrachion)	9986739; 7556496
**TNFA**	TNF	7124	tumor necrosis factor alpha	15557444; 15505055; 14615639; 12579167; 11431443
**TNF-R1**	TNFRSF1A	7132	TNF-receptor 1	11391707; 10975909; 14697498; 11431443; 10975909; 10815159
**TP53**	TP53	7157	Tumor protein p53	15060108
**TYR**	TYR	7299	tyrosinase (oculocutaneous albinism IA)	12624268
**TYRP1**	TYRP1	7306	tyrosinase-related protein 1	11743578; 12011806
**VIM**	VIM	7431	vimentin	11599002; 15505052
**WDR36**	WDR36	134430	WDR36 (WD40-repeat 36)	15677485

Table 8. Proximity dataset revealed using the AN networks generated from the G-set ("literature" genes)

Literature Genes overlapping with the DE set

Gene	*LL IDs*	*Protein*	*fold change*
APP	351	Amyloid beta A4 protein	2.5
BMP4	652	Bone morphogenetic protein 4 precursor	2.5
c-Fos	2353	Proto-oncogene protein c-fos	3
Cox-2	5743	Prostaglandin-endoperoxide synthase (PTGS), cyclooxygenase-2	-3.8
EGFR	1956	Epidermal growth factor receptor precursor	2.5
ELN	2006	Elastin	-2.6
MMP-1 -	4312	matrix metallopeptidase 1	-8.4
NCAM1	4684	neural cell adhesion molecule 1	2.7
CSPG4(NG2)	1464	Chondroitin sulfate proteoglycan 4 precursor	-3.9

Up-regulated close neighbor DE (non-literature) genes

Gene	*LL IDs*	*Protein*	*fold change*
ADAM17	6868	ADAM metallopeptidase domain 17	3.5
AR	367	androgen receptor	3
BRCA1	672	breast and ovarian cancer susceptibility protein 1	2.5
CASP1	834	Caspase-1	3.7
CEBPD	1052	CCAAT/enhancer binding protein delta	2.8
CLU	1191	apolipoprotein J, clusterin	6.3
MYB	4602	Myb proto-oncogene protein	2.7
COL4A4	1286	alpha 4 type IV collagen	2.7
CX3CL1	6376	chemokine (C-X3-C motif) ligand 1	2.9
DAPK1	1612	Death-associated protein kinase 1	4.4
HNF4A	3172	hepatic nuclear factor 4 alpha	2.5
HSPA5	3309	heat shock 70kD protein 5	3.2
ID1	3397	ibitor of differentiation 1	4.2
IGF1	3479	insulin-like growth factor 1 (somatomedin C)	3.3
MAPK10	5602	JNK3 alpha protein kinase	3
Jun-D	3727	Transcription factor JunD	3.3
MAPK 13	5603	Mitogen-activated protein kinase 13, p38delta	
MAPKAPK3	7867	MAP kinase-activated protein kinase 3	2.7
MMP-2	4313	matrix metallopeptidase 2	2.5
MSX1	4487	Homeobox protein MSX-1	3.5
MYOD1	4654	myoblast determination protein 1	3.5
NTRK2	4915	BDNF/NT-3 growth factors receptor	3.7
REL	5966	c-Rel proto-oncogene protein	2.7

PAX2	5076	paired box gene 2	4.3
MPO	4353	Myeloperoxidase	3.4
PIN1	5300	Peptidyl-prolyl cis-trans isomerase NIMA-interacting 1	3.2
PLEK	5341	Pleckstrin	2.5
SP1	6667	Sp1 transcription factor	4
SAT	6303	spermidine/spermine N1-acetyltransferase	2.6
STAT5B	6777	signal transducer and activator of transcription 5B	2.6
STAT5A	6776	signal transducer and activator of transcription 5A	2.9
TCF3	6929	Transcription factor E2-alpha	3.6
TFCP2	7024	transcription factor CP2	3.2
VDR	7421	vitamin D (1,25- dihydroxyvitamin D3) receptor	2.7
WT1	7490	Wilms tumor 1	3.2
IL-1RI	3554	Interleukin-1 receptor type I precursor	2.5
G-protein alpha-I	2771	Guanine nucleotide-binding protein G(i), alpha-2 subunit	3.5

Down-regulated close neighbor DE (non-literature) genes

Gene	LL IDs	Protein	fold change
Bcl-6	604	B-cell lymphoma 6 protein	-2.7
CTSG	1511	Cathepsin G	-2.7
CDH1	999	cadherin 1, E-cadherin (epithelial)	-3.7
ESR1	2099	estrogen receptor 1	-4.4
HBEGF	1839	Heparin-binding EGF-like growth factor	-2.8
IFNG	3458	Interferon gamma	-3.8
LCN2	3934	Neutrophil gelatinase-associated lipocalin	-3.6
MEF2C	4208	Myocyte-specific enhancer factor 2C	-2.5
NHLH2	4808	nescient helix loop helix 2	-3
SERPINE1	5054	Plasminogen activator inhibitor 1 precursor	-2.5
PDGFRA	5156	platelet-derived growth factor receptor alpha	-2.8
POU1F1	5449	Pituitary-specific positive transcription factor 1	-3.3
PKC-epsilon	5581	Protein kinase C epsilon type	-2.5
PTPRZ1	5803	Receptor-type tyrosine-protein phosphatase zeta	-11.8
SLC4A4	8671	sodium bicarbonate cotransporter 1	-2.7
STAT1	6772	signal transducer and activator of transcription 1	-2.9
STAT4	6775	Signal transducer and activator of transcription 4	-3.7
TEK	7010	TEK tyrosine kinase, endothelial	-3
ZIC1	7545	Zinc finger protein ZIC 1	-2.7
ZIC1	7545	Zinc finger protein ZIC 1	-2.7

the underlined gene IDs overlapping with the global DI network

Literature Genes without gene expression data

Gene
14-3-3 sigma
BDNF
BMP Receptor1
BMP Receptor2
BMP-2
Ceruloplasmin
c-Jun
Collagen III
CTGF
CYP1B1
ELF5
Endothelin-1
eNOS
Fibrillin1
Fibronectin
FTH1
HSP-27
IL-1 beta
IL1RN
IL-6
iNOS
LMX1B
LMX1B
MGST
MMP-14
MMP-9
Myocilin
optineurin
p21
p53
PAX6
Pitx2
PPAP
STAT3
TAP1/PSF1
Tenascin-C
TGF-beta2
Thrombospond
TIMP1

Supplement Table 9

Major parameters used for calculation of p-value for networks				
number of nodes	DI network largest cluster	average largest cluster (random)	standard deviation	p-value
461	196	84	30.6	<0.0001
70	47	3	1.37	<0.0001
141	127	7	3.5	<0.0001
301	50	30	16.9	<0.04

Supplement Table 10. Statistical analysis of gene expression data

gene	mean	mean (ratios)	std.error	bonf.lo	bonf.hi	includes.ze	p.value (adjusted)
40887_g_at	-1.7683	-58.654319	0.414136	-3.67895	0.14235	TRUE	2.47E-01
38965_at	-1.60031	-39.839144	0.159062	-2.33416	-0.86646	FALSE	1.04E-19
37407_s_at	-1.56161	-36.442654	0.091692	-1.98464	-1.13858	FALSE	6.10E-61
32582_at	-1.43469	-27.207585	0.124001	-2.00678	-0.8626	FALSE	7.39E-27
39207_r_at	-1.42386	-26.5375	0.144156	-2.08894	-0.75878	FALSE	6.60E-19
774_g_at	-1.36857	-23.365227	0.078438	-1.73045	-1.00669	FALSE	4.52E-64
767_at	-1.30321	-20.100645	0.112485	-1.82217	-0.78425	FALSE	6.15E-27
38749_at	-1.17735	-15.043538	0.094857	-1.61498	-0.73972	FALSE	2.84E-31
39206_s_at	-1.16927	-14.766243	0.156379	-1.89074	-0.4478	FALSE	9.59E-10
34476_r_at	-1.16141	-14.501402	0.09942	-1.62009	-0.70272	FALSE	1.99E-27
affx-thrx-5_at	-1.15571	-14.312319	0.315386	-2.61077	0.29935	TRUE	1.00E+00
35731_at	-1.12583	-13.360724	0.085625	-1.52087	-0.7308	FALSE	2.19E-35
35712_at	-1.09627	-12.481593	0.108614	-1.59738	-0.59517	FALSE	7.47E-20
914_g_at	-1.01512	-10.354282	0.127083	-1.60143	-0.42881	FALSE	1.73E-11
33232_at	-1.01102	-10.256992	0.123108	-1.57899	-0.44304	FALSE	2.74E-12
33411_g_at	-0.96594	-9.245704	0.090795	-1.38483	-0.54705	FALSE	2.49E-22
38004_at	-0.88491	-7.672025	0.096329	-1.32933	-0.44048	FALSE	5.13E-16
36043_at	-0.87532	-7.50447	0.042155	-1.0698	-0.68083	FALSE	1.16E-91
35324_at	-0.87295	-7.463628	0.186808	-1.7348	-0.01109	FALSE	3.75E-02
34235_at	-0.86201	-7.277966	0.089422	-1.27457	-0.44945	FALSE	6.85E-18
2061_at	-0.86091	-7.259555	0.137437	-1.49499	-0.22683	FALSE	4.74E-06
40899_at	-0.85714	-7.196809	0.152454	-1.5605	-0.15377	FALSE	2.38E-04
41714_at	-0.85546	-7.169023	0.072659	-1.19068	-0.52024	FALSE	6.75E-28
159_at	-0.85478	-7.157807	0.101647	-1.32373	-0.38582	FALSE	5.21E-13
36370_at	-0.84976	-7.075547	0.179778	-1.67918	-0.02033	FALSE	2.88E-02
40350_at	-0.8403	-6.92309	0.076914	-1.19515	-0.48545	FALSE	1.10E-23
1776_at	-0.82116	-6.624605	0.098688	-1.27647	-0.36586	FALSE	1.10E-12
1545_g_at	-0.80304	-6.353895	0.088163	-1.20979	-0.39629	FALSE	1.05E-15
affx-thrx-m_at	-0.79	-6.16595	0.287899	-2.11825	0.53825	TRUE	1.00E+00
36258_at	-0.78065	-6.034621	0.107441	-1.27633	-0.28496	FALSE	4.68E-09
33143_s_at	-0.76872	-5.871107	0.076374	-1.12108	-0.41636	FALSE	9.95E-20
affx-phex-5_at	-0.76451	-5.814468	0.249786	-1.91692	0.3879	TRUE	1.00E+00
36650_at	-0.76327	-5.79789	0.138663	-1.403	-0.12354	FALSE	4.67E-04
35889_at	-0.74931	-5.614486	0.114679	-1.2784	-0.22023	FALSE	8.08E-07
affx-trpnx-m_at	-0.73811	-5.471545	0.185938	-1.59595	0.11973	TRUE	9.09E-01
2073_s_at	-0.73289	-5.406174	0.077704	-1.09139	-0.3744	FALSE	5.09E-17
38289_r_at	-0.73264	-5.403063	0.065925	-1.03679	-0.42849	FALSE	1.36E-24
33410_at	-0.73185	-5.393243	0.075722	-1.0812	-0.38251	FALSE	5.36E-18
1758_r_at	-0.72884	-5.355993	0.173808	-1.53072	0.07303	TRUE	3.47E-01
1806_at	-0.71311	-5.165472	0.127387	-1.30082	-0.1254	FALSE	2.74E-04
201_s_at	-0.7124	-5.157034	0.181538	-1.54995	0.12514	TRUE	1.00E+00
35083_at	-0.71207	-5.153117	0.082838	-1.09425	-0.32989	FALSE	1.04E-13
36966_at	-0.70728	-5.096594	0.12723	-1.29427	-0.1203	FALSE	3.42E-04
39710_at	-0.69161	-4.915979	0.046287	-0.90515	-0.47806	FALSE	2.23E-46
39618_at	-0.68989	-4.896548	0.313465	-2.13609	0.75631	TRUE	1.00E+00
39528_at	-0.6889	-4.885399	0.082501	-1.06953	-0.30828	FALSE	8.60E-13
32711_g_at	-0.6858	-4.850651	0.120052	-1.23968	-0.13193	FALSE	1.41E-04
affx-phex-m_at	-0.68567	-4.849199	0.224102	-1.71959	0.34824	TRUE	1.00E+00
2070_i_at	-0.68502	-4.841947	0.230844	-1.75004	0.38	TRUE	1.00E+00
32328_at	-0.67218	-4.700889	0.106246	-1.16236	-0.182	FALSE	3.16E-06

902_at	-0.67206	-4.69959	0.098217	-1.12519	-0.21892	FALSE	9.82E-08
31843_at	-0.67125	-4.690833	0.137078	-1.30368	-0.03883	FALSE	1.23E-02
1404_r_at	-0.67016	-4.679075	0.142639	-1.32824	-0.01208	FALSE	3.31E-02
35558_at	-0.65619	-4.530958	0.086196	-1.05387	-0.25852	FALSE	3.39E-10
35989_at	-0.65455	-4.51388	0.162062	-1.40224	0.09314	TRUE	6.78E-01
affx-trpnx-5_at	-0.6464	-4.429962	0.281101	-1.94328	0.65048	TRUE	1.00E+00
35679_s_at	-0.63872	-4.352312	0.056266	-0.89831	-0.37913	FALSE	9.18E-26
41266_at	-0.63713	-4.336407	0.04843	-0.86057	-0.4137	FALSE	1.99E-35
1597_at	-0.6365	-4.330121	0.063356	-0.92879	-0.3442	FALSE	1.20E-19
35049_g_at	-0.63581	-4.323247	0.165591	-1.39978	0.12816	TRUE	1.00E+00
32847_at	-0.63498	-4.314992	0.077375	-0.99196	-0.27801	FALSE	2.87E-12
39690_at	-0.63259	-4.291311	0.063977	-0.92776	-0.33743	FALSE	5.94E-19
40075_at	-0.63089	-4.274546	0.044472	-0.83607	-0.42572	FALSE	1.41E-41
194_at	-0.62805	-4.246685	0.103914	-1.10747	-0.14863	FALSE	1.90E-05
40488_at	-0.624	-4.207266	0.078891	-0.98797	-0.26003	FALSE	3.26E-11
38583_at	-0.62142	-4.182346	0.205802	-1.57091	0.32806	TRUE	1.00E+00
34517_at	-0.6214	-4.182154	0.104021	-1.10131	-0.14149	FALSE	2.93E-05
35703_at	-0.61783	-4.147916	0.03258	-0.76814	-0.46752	FALSE	4.30E-76
39878_at	-0.61654	-4.135614	0.143447	-1.27834	0.04527	TRUE	2.18E-01
110_at	-0.61042	-4.077744	0.072264	-0.94381	-0.27702	FALSE	3.77E-13
33359_at	-0.60987	-4.072584	0.057196	-0.87375	-0.34599	FALSE	1.92E-22
1099_s_at	-0.60945	-4.068647	0.078019	-0.9694	-0.2495	FALSE	7.13E-11
35616_at	-0.60919	-4.066212	0.102553	-1.08233	-0.13606	FALSE	3.59E-05
41401_at	-0.59326	-3.919765	0.062942	-0.88365	-0.30287	FALSE	5.40E-17
39901_at	-0.59255	-3.913362	0.06089	-0.87347	-0.31164	FALSE	2.79E-18
483_g_at	-0.58699	-3.863581	0.072605	-0.92197	-0.25202	FALSE	7.87E-12
affx-lysx-5_at	-0.58697	-3.863403	0.173618	-1.38797	0.21403	TRUE	1.00E+00
31733_at	-0.58099	-3.81057	0.068953	-0.89911	-0.26287	FALSE	4.52E-13
38428_at	-0.58073	-3.80829	0.353775	-2.2129	1.05144	TRUE	1.00E+00
40097_at	-0.57813	-3.785559	0.199231	-1.49729	0.34104	TRUE	1.00E+00
161_at	-0.57777	-3.782422	0.1253	-1.15585	0.00032	TRUE	5.06E-02
35832_at	-0.57707	-3.776331	0.075599	-0.92586	-0.22829	FALSE	2.89E-10
32466_at	-0.57663	-3.772507	0.166078	-1.34285	0.18958	TRUE	1.00E+00
36877_at	-0.57365	-3.746709	0.085732	-0.96918	-0.17813	FALSE	2.79E-07
906_at	-0.57231	-3.735167	0.042262	-0.76729	-0.37733	FALSE	1.12E-37
236_at	-0.56884	-3.705442	0.052864	-0.81274	-0.32495	FALSE	6.68E-23
36156_at	-0.56833	-3.701093	0.058015	-0.83599	-0.30067	FALSE	1.48E-18
37185_at	-0.5654	-3.676207	0.080481	-0.93671	-0.19409	FALSE	2.70E-08
35607_at	-0.55836	-3.617096	0.136267	-1.18704	0.07032	TRUE	5.27E-01
36410_f_at	-0.55803	-3.614348	0.096816	-1.00471	-0.11136	FALSE	1.04E-04
31848_at	-0.55509	-3.589963	0.092991	-0.98411	-0.12606	FALSE	3.01E-05
39052_at	-0.55499	-3.589137	0.227909	-1.60647	0.49649	TRUE	1.00E+00
31656_at	-0.55396	-3.580635	0.113323	-1.07679	-0.03113	FALSE	1.28E-02
32331_at	-0.55105	-3.556723	0.133932	-1.16895	0.06686	TRUE	4.90E-01
901_g_at	-0.54346	-3.495103	0.097846	-0.99488	-0.09204	FALSE	3.52E-04
31962_at	-0.5426	-3.488189	0.138993	-1.18386	0.09866	TRUE	1.00E+00
35172_at	-0.53929	-3.461705	0.056191	-0.79854	-0.28005	FALSE	1.04E-17
41299_f_at	-0.53353	-3.416095	0.073878	-0.87438	-0.19269	FALSE	6.48E-09
41355_at	-0.53289	-3.411065	0.051729	-0.77155	-0.29424	FALSE	8.75E-21
39407_at	-0.53204	-3.404395	0.038277	-0.70864	-0.35545	FALSE	8.01E-40
31765_at	-0.53126	-3.398287	0.124704	-1.10659	0.04408	TRUE	2.58E-01

1732_at	-0.53089	-3.395393	0.097643	-0.98138	-0.08041	FALSE	6.84E-04
39575_at	-0.52825	-3.374815	0.107916	-1.02613	-0.03037	FALSE	1.24E-02
758_at	-0.5244	-3.34503	0.058112	-0.7925	-0.25629	FALSE	2.29E-15
39898_at	-0.52276	-3.332422	0.150034	-1.21496	0.16943	TRUE	1.00E+00
35433_s_at	-0.52066	-3.316347	0.067217	-0.83077	-0.21054	FALSE	1.20E-10
34479_at	-0.51788	-3.295187	0.079393	-0.88417	-0.1516	FALSE	8.70E-07
31311_at	-0.51715	-3.289652	0.045001	-0.72477	-0.30954	FALSE	1.83E-26
34203_at	-0.51417	-3.267157	0.095697	-0.95568	-0.07266	FALSE	9.78E-04
31636_s_at	-0.51394	-3.265427	0.081675	-0.89076	-0.13712	FALSE	3.95E-06
31732_at	-0.51372	-3.263773	0.116122	-1.04946	0.02202	TRUE	1.22E-01
37459_at	-0.51161	-3.247955	0.035539	-0.67558	-0.34765	FALSE	6.94E-43
34012_at	-0.50978	-3.234298	0.179029	-1.33575	0.31618	TRUE	1.00E+00
1098_at	-0.50846	-3.224482	0.094444	-0.94419	-0.07273	FALSE	9.21E-04
38562_g_at	-0.50803	-3.221291	0.0922	-0.9334	-0.08266	FALSE	4.53E-04
41020_at	-0.50476	-3.197128	0.130451	-1.10661	0.09709	TRUE	1.00E+00
32919_at	-0.50443	-3.194699	0.055061	-0.75846	-0.25041	FALSE	6.46E-16
1286_s_at	-0.50404	-3.191832	0.076254	-0.85585	-0.15224	FALSE	4.85E-07
931_at	-0.50238	-3.179655	0.097324	-0.9514	-0.05337	FALSE	3.09E-03
33206_at	-0.49486	-3.125072	0.058246	-0.76358	-0.22614	FALSE	2.48E-13
36425_at	-0.4929	-3.111	0.064737	-0.79157	-0.19423	FALSE	3.36E-10
1679_at	-0.49289	-3.110928	0.087419	-0.89621	-0.08957	FALSE	2.17E-04
32687_s_at	-0.49163	-3.101916	0.111765	-1.00727	0.02401	TRUE	1.37E-01
34650_at	-0.4906	-3.094568	0.082638	-0.87186	-0.10934	FALSE	3.67E-05
38430_at	-0.48994	-3.089869	0.191405	-1.373	0.39313	TRUE	1.00E+00
33439_at	-0.48861	-3.08042	0.125859	-1.06927	0.09205	TRUE	1.00E+00
1866_g_at	-0.48639	-3.064714	0.088509	-0.89473	-0.07805	FALSE	4.92E-04
1596_g_at	-0.48598	-3.061822	0.06055	-0.76534	-0.20663	FALSE	1.27E-11
34040_s_at	-0.48438	-3.050563	0.086835	-0.885	-0.08376	FALSE	3.07E-04
39063_at	-0.48175	-3.032145	0.143524	-1.14392	0.18041	TRUE	1.00E+00
affx-thrx-3_at	-0.48171	-3.031866	0.132846	-1.0946	0.13119	TRUE	1.00E+00
160040_at	-0.4807	-3.024823	0.133592	-1.09704	0.13564	TRUE	1.00E+00
36144_at	-0.48017	-3.021134	0.122695	-1.04623	0.0859	TRUE	1.00E+00
33925_at	-0.47902	-3.013145	0.127547	-1.06747	0.10943	TRUE	1.00E+00
1934_s_at	-0.47895	-3.012659	0.055674	-0.73581	-0.2221	FALSE	9.82E-14
40062_s_at	-0.47875	-3.011272	0.064161	-0.77476	-0.18274	FALSE	1.08E-09
31714_at	-0.47737	-3.001719	0.080319	-0.84793	-0.10681	FALSE	3.52E-05
31811_r_at	-0.47639	-2.994953	0.165769	-1.24118	0.2884	TRUE	1.00E+00
36157_at	-0.47635	-2.994677	0.099722	-0.93643	-0.01628	FALSE	2.25E-02
40168_at	-0.47619	-2.993574	0.075204	-0.82316	-0.12923	FALSE	3.06E-06
36246_at	-0.47385	-2.977488	0.078622	-0.83657	-0.11112	FALSE	2.11E-05
37583_at	-0.4716	-2.962102	0.129617	-1.0696	0.1264	TRUE	1.00E+00
33338_at	-0.47138	-2.960602	0.018551	-0.55696	-0.38579	FALSE	2.51E-138
36331_at	-0.47035	-2.953589	0.061517	-0.75416	-0.18654	FALSE	2.62E-10
1490_at	-0.46558	-2.921326	0.061345	-0.7486	-0.18257	FALSE	4.05E-10
34390_at	-0.46343	-2.906899	0.026919	-0.58763	-0.33924	FALSE	2.56E-62
885_g_at	-0.46261	-2.901416	0.045588	-0.67293	-0.25228	FALSE	4.29E-20
34477_at	-0.46198	-2.89721	0.112461	-0.98083	0.05687	TRUE	5.04E-01
affx-humisgf3a/m	-0.45907	-2.877862	0.027585	-0.58634	-0.3318	FALSE	4.38E-58
1279_s_at	-0.45805	-2.871111	0.074815	-0.80322	-0.11289	FALSE	1.16E-05
41654_at	-0.45791	-2.870186	0.061386	-0.74112	-0.1747	FALSE	1.10E-09
33496_at	-0.45563	-2.855157	0.131242	-1.06113	0.14987	TRUE	1.00E+00

37562_at	-0.45557	-2.854763	0.048496	-0.67931	-0.23183	FALSE	7.29E-17
40691_at	-0.45334	-2.840142	0.096188	-0.89712	-0.00957	FALSE	3.08E-02
1808_s_at	-0.452	-2.831392	0.060228	-0.72987	-0.17413	FALSE	7.77E-10
1128_s_at	-0.44812	-2.806209	0.078011	-0.80803	-0.08821	FALSE	1.17E-04
35285_at	-0.44804	-2.805692	0.032365	-0.59736	-0.29872	FALSE	1.77E-39
40525_at	-0.44784	-2.8044	0.06041	-0.72654	-0.16913	FALSE	1.56E-09
35885_at	-0.44771	-2.803561	0.129852	-1.04679	0.15137	TRUE	1.00E+00
31856_at	-0.44766	-2.803238	0.035299	-0.61052	-0.28481	FALSE	9.39E-33
41685_at	-0.4467	-2.797049	0.099145	-0.90411	0.01072	TRUE	8.36E-02
41582_at	-0.44606	-2.79293	0.098304	-0.89959	0.00747	TRUE	7.18E-02
40895_g_at	-0.44555	-2.789652	0.051388	-0.68263	-0.20847	FALSE	5.44E-14
32242_at	-0.44548	-2.789202	0.050546	-0.67868	-0.21228	FALSE	1.53E-14
32312_at	-0.44484	-2.785095	0.15087	-1.14089	0.25122	TRUE	1.00E+00
38267_at	-0.44327	-2.775045	0.05768	-0.70938	-0.17716	FALSE	1.93E-10
34870_at	-0.4412	-2.761849	0.083949	-0.82851	-0.0539	FALSE	1.86E-03
40560_at	-0.44014	-2.755117	0.058255	-0.7089	-0.17137	FALSE	5.28E-10
672_at	-0.43991	-2.753658	0.024749	-0.55409	-0.32573	FALSE	1.39E-66
36389_at	-0.4388	-2.746629	0.167844	-1.21317	0.33556	TRUE	1.00E+00
39680_at	-0.43871	-2.74606	0.091204	-0.85949	-0.01793	FALSE	1.90E-02
37279_at	-0.43776	-2.74006	0.08604	-0.83471	-0.04081	FALSE	4.57E-03
33382_at	-0.43616	-2.729983	0.099117	-0.89344	0.02112	TRUE	1.36E-01
36009_at	-0.43362	-2.714063	0.045949	-0.64561	-0.22164	FALSE	4.84E-17
33910_at	-0.43272	-2.708445	0.13211	-1.04222	0.17678	TRUE	1.00E+00
31666_f_at	-0.43147	-2.700661	0.086683	-0.83139	-0.03155	FALSE	8.13E-03
41455_at	-0.42833	-2.681205	0.122115	-0.99172	0.13506	TRUE	1.00E+00
32950_at	-0.42808	-2.679662	0.095676	-0.86949	0.01333	TRUE	9.68E-02
907_at	-0.42798	-2.679045	0.051571	-0.66591	-0.19005	FALSE	1.33E-12
34905_at	-0.42683	-2.67196	0.077999	-0.78668	-0.06697	FALSE	5.61E-04
32859_at	-0.42561	-2.664465	0.036023	-0.5918	-0.25942	FALSE	4.11E-28
31318_at	-0.42519	-2.661889	0.069746	-0.74697	-0.10342	FALSE	1.37E-05
36395_at	-0.42439	-2.65699	0.101983	-0.89489	0.04612	TRUE	3.99E-01
affx-humisgf3a/m	-0.42421	-2.655889	0.039058	-0.60441	-0.24402	FALSE	2.23E-23
679_at	-0.42402	-2.654728	0.075356	-0.77168	-0.07636	FALSE	2.32E-04
32568_at	-0.42357	-2.651979	0.102356	-0.8958	0.04866	TRUE	4.42E-01
32291_at	-0.42325	-2.650025	0.138987	-1.06448	0.21797	TRUE	1.00E+00
343_s_at	-0.4227	-2.646671	0.067934	-0.73612	-0.10928	FALSE	6.19E-06
33204_at	-0.42165	-2.64028	0.112099	-0.93883	0.09553	TRUE	1.00E+00
36808_at	-0.42102	-2.636453	0.091971	-0.84534	0.0033	TRUE	5.93E-02
35714_at	-0.42043	-2.632874	0.113007	-0.94179	0.10094	TRUE	1.00E+00
34566_at	-0.4197	-2.628452	0.099936	-0.88076	0.04137	TRUE	3.37E-01
884_at	-0.41896	-2.623977	0.02857	-0.55077	-0.28715	FALSE	1.38E-44
35220_at	-0.41876	-2.622769	0.157557	-1.14566	0.30814	TRUE	1.00E+00
1687_s_at	-0.41557	-2.603574	0.064365	-0.71252	-0.11861	FALSE	1.35E-06
31986_at	-0.41331	-2.590061	0.171706	-1.20549	0.37887	TRUE	1.00E+00
34050_at	-0.41314	-2.589047	0.079457	-0.77972	-0.04656	FALSE	2.52E-03
31314_at	-0.41305	-2.588511	0.068785	-0.73039	-0.0957	FALSE	2.42E-05
34718_at	-0.41214	-2.583093	0.060035	-0.68912	-0.13516	FALSE	8.40E-08
34972_s_at	-0.4114	-2.578695	0.075321	-0.7589	-0.0639	FALSE	5.95E-04
31682_s_at	-0.41125	-2.577805	0.070803	-0.73791	-0.0846	FALSE	7.96E-05
41294_at	-0.41112	-2.577033	0.08193	-0.78911	-0.03313	FALSE	6.59E-03
35041_at	-0.40902	-2.564602	0.085892	-0.80529	-0.01275	FALSE	2.42E-02

707_s_at	-0.40779	-2.557349	0.036068	-0.57419	-0.24138	FALSE	1.55E-25
32689_s_at	-0.40755	-2.555936	0.088663	-0.8166	0.0015	TRUE	5.42E-02
35871_s_at	-0.40668	-2.550821	0.087156	-0.80879	-0.00458	FALSE	3.87E-02
39069_at	-0.40662	-2.550469	0.03868	-0.58507	-0.22816	FALSE	9.57E-22
37765_at	-0.40627	-2.548414	0.038681	-0.58473	-0.22781	FALSE	1.06E-21
41157_at	-0.40605	-2.547123	0.091907	-0.83008	0.01797	TRUE	1.26E-01
38355_at	-0.406	-2.54683	0.271376	-1.65802	0.84601	TRUE	1.00E+00
40635_at	-0.40592	-2.546361	0.110421	-0.91536	0.10351	TRUE	1.00E+00
35654_at	-0.40542	-2.543431	0.130529	-1.00762	0.19679	TRUE	1.00E+00
1553_r_at	-0.40439	-2.537406	0.114261	-0.93154	0.12277	TRUE	1.00E+00
342_at	-0.40348	-2.532095	0.040554	-0.59059	-0.21638	FALSE	3.21E-19
1826_at	-0.40289	-2.528657	0.070767	-0.72938	-0.0764	FALSE	1.57E-04
31766_s_at	-0.40139	-2.519939	0.085599	-0.7963	-0.00647	FALSE	3.46E-02
34044_at	-0.40036	-2.513969	0.166207	-1.16717	0.36646	TRUE	1.00E+00
38112_g_at	-0.39926	-2.50761	0.052337	-0.64072	-0.1578	FALSE	3.00E-10
1977_s_at	-0.39913	-2.50686	0.097134	-0.84727	0.049	TRUE	5.01E-01
33257_at	-0.39905	-2.506398	0.101383	-0.86679	0.06869	TRUE	1.00E+00
40512_at	-0.39672	-2.492987	0.07091	-0.72387	-0.06956	FALSE	2.79E-04
33542_at	-0.3966	-2.492298	0.131851	-1.00491	0.2117	TRUE	1.00E+00
1964_g_at	-0.39632	-2.490692	0.064747	-0.69504	-0.09761	FALSE	1.17E-05
32231_at	-0.3963	-2.490577	0.080976	-0.76989	-0.02271	FALSE	1.25E-02
38268_at	-0.39574	-2.487368	0.052121	-0.6362	-0.15527	FALSE	3.96E-10
33878_at	-0.39508	-2.483591	0.020994	-0.49194	-0.29822	FALSE	6.73E-75
35865_at	-0.39417	-2.478392	0.111602	-0.90906	0.12071	TRUE	1.00E+00
35674_at	-0.39415	-2.478278	0.093504	-0.82554	0.03724	TRUE	3.15E-01
1260_s_at	-0.39348	-2.474458	0.071116	-0.72158	-0.06538	FALSE	3.98E-04
40194_at	-0.39346	-2.474344	0.15414	-1.1046	0.31768	TRUE	1.00E+00
41087_at	-0.39241	-2.468369	0.062435	-0.68046	-0.10436	FALSE	4.14E-06
2009_at	-0.39151	-2.463259	0.030276	-0.53119	-0.25183	FALSE	3.77E-34
32149_at	-0.39131	-2.462124	0.076989	-0.74651	-0.03612	FALSE	4.70E-03
40848_g_at	-0.38956	-2.452223	0.025653	-0.50791	-0.2712	FALSE	5.57E-48
41093_at	-0.38948	-2.451772	0.101684	-0.85861	0.07965	TRUE	1.00E+00
1610_s_at	-0.38924	-2.450417	0.092082	-0.81407	0.03558	TRUE	2.99E-01
33765_at	-0.38916	-2.449966	0.124377	-0.96298	0.18467	TRUE	1.00E+00
33339_g_at	-0.38869	-2.447316	0.025157	-0.50476	-0.27263	FALSE	9.38E-50
35879_at	-0.38816	-2.444331	0.107606	-0.88461	0.10828	TRUE	1.00E+00
33756_at	-0.3881	-2.443993	0.083424	-0.77298	-0.00321	FALSE	4.15E-02
41323_at	-0.38807	-2.443824	0.122912	-0.95513	0.179	TRUE	1.00E+00
220_r_at	-0.38646	-2.434782	0.097547	-0.8365	0.06359	TRUE	9.39E-01
affx-trpnx-3_at	-0.38636	-2.434221	0.145762	-1.05885	0.28612	TRUE	1.00E+00
36892_at	-0.38524	-2.427951	0.055038	-0.63916	-0.13131	FALSE	3.24E-08
41723_s_at	-0.38522	-2.42784	0.133749	-1.00228	0.23184	TRUE	1.00E+00
37669_s_at	-0.38519	-2.427672	0.052586	-0.62779	-0.14258	FALSE	3.02E-09
32182_at	-0.38516	-2.427504	0.058296	-0.65411	-0.11621	FALSE	4.95E-07
35539_at	-0.38422	-2.422256	0.131868	-0.9926	0.22416	TRUE	1.00E+00
31549_at	-0.38401	-2.421085	0.047399	-0.60269	-0.16533	FALSE	6.84E-12
31607_at	-0.38341	-2.417742	0.097762	-0.83444	0.06762	TRUE	1.00E+00
696_at	-0.38298	-2.41535	0.082365	-0.76298	-0.00298	FALSE	4.19E-02
1862_at	-0.38231	-2.411626	0.081851	-0.75994	-0.00468	FALSE	3.79E-02
39326_at	-0.38201	-2.409961	0.119226	-0.93207	0.16805	TRUE	1.00E+00
37115_at	-0.38136	-2.406357	0.043975	-0.58424	-0.17847	FALSE	5.35E-14

32319_at	-0.38055	-2.401873	0.079087	-0.74542	-0.01567	FALSE	1.89E-02
32017_at	-0.38041	-2.401099	0.116151	-0.91628	0.15546	TRUE	1.00E+00
607_s_at	-0.37962	-2.396735	0.064654	-0.67791	-0.08133	FALSE	5.45E-05
41779_at	-0.37956	-2.396404	0.030515	-0.52034	-0.23877	FALSE	2.04E-31
34209_at	-0.37943	-2.395687	0.063046	-0.6703	-0.08856	FALSE	2.23E-05
40653_at	-0.37871	-2.391718	0.043023	-0.5772	-0.18022	FALSE	1.69E-14
1376_at	-0.378	-2.387811	0.041786	-0.57078	-0.18521	FALSE	1.87E-15
37145_at	-0.37704	-2.382539	0.058509	-0.64698	-0.1071	FALSE	1.47E-06
1958_at	-0.37404	-2.366138	0.148318	-1.05832	0.31023	TRUE	1.00E+00
33116_f_at	-0.37295	-2.360206	0.250343	-1.52793	0.78203	TRUE	1.00E+00
37921_at	-0.37238	-2.357111	0.075975	-0.7229	-0.02186	FALSE	1.20E-02
36379_at	-0.37224	-2.356351	0.051787	-0.61116	-0.13331	FALSE	8.31E-09
200_at	-0.37167	-2.35326	0.156785	-1.09501	0.35167	TRUE	1.00E+00
41285_at	-0.3705	-2.346929	0.039894	-0.55455	-0.18644	FALSE	2.00E-16
33108_i_at	-0.36948	-2.341424	0.083318	-0.75387	0.01492	TRUE	1.16E-01
37789_at	-0.36933	-2.340615	0.125338	-0.94759	0.20893	TRUE	1.00E+00
36386_at	-0.369	-2.338837	0.077736	-0.72764	-0.01035	FALSE	2.61E-02
38133_at	-0.36883	-2.337922	0.131286	-0.97452	0.23687	TRUE	1.00E+00
41687_r_at	-0.3688	-2.33776	0.129279	-0.96524	0.22764	TRUE	1.00E+00
33060_g_at	-0.36848	-2.336039	0.045445	-0.57814	-0.15881	FALSE	6.49E-12
31474_r_at	-0.36829	-2.335017	0.096596	-0.81394	0.07737	TRUE	1.00E+00
1595_at	-0.36777	-2.332223	0.060427	-0.64655	-0.08898	FALSE	1.46E-05
39250_at	-0.36771	-2.3319	0.036303	-0.5352	-0.20022	FALSE	5.20E-20
38700_at	-0.36682	-2.327127	0.044738	-0.57322	-0.16042	FALSE	3.05E-12
35030_i_at	-0.36667	-2.326323	0.070777	-0.6932	-0.04013	FALSE	2.79E-03
affx-biodn-5_st	-0.36548	-2.319957	0.079768	-0.7335	0.00253	TRUE	5.82E-02
41794_at	-0.36499	-2.317341	0.123101	-0.93293	0.20294	TRUE	1.00E+00
38140_at	-0.36439	-2.314142	0.120808	-0.92175	0.19296	TRUE	1.00E+00
668_s_at	-0.36389	-2.311479	0.091853	-0.78766	0.05988	TRUE	9.40E-01
36384_at	-0.36377	-2.310841	0.063493	-0.6567	-0.07084	FALSE	1.27E-04
37127_at	-0.36374	-2.310681	0.103034	-0.8391	0.11162	TRUE	1.00E+00
40847_at	-0.36368	-2.310362	0.030612	-0.50491	-0.22245	FALSE	1.89E-28
32139_at	-0.36361	-2.309989	0.03468	-0.52361	-0.20361	FALSE	1.28E-21
31437_r_at	-0.36335	-2.308607	0.090532	-0.78103	0.05432	TRUE	7.55E-01
38547_at	-0.36255	-2.304358	0.080539	-0.73412	0.00903	TRUE	8.52E-02
33483_at	-0.36179	-2.300329	0.180104	-1.19271	0.46914	TRUE	1.00E+00
35281_at	-0.36094	-2.295831	0.110904	-0.8726	0.15073	TRUE	1.00E+00
1956_s_at	-0.3609	-2.29562	0.107325	-0.85605	0.13425	TRUE	1.00E+00
2053_at	-0.36008	-2.29129	0.022042	-0.46177	-0.25839	FALSE	6.87E-56
36629_at	-0.36006	-2.291184	0.037504	-0.53309	-0.18703	FALSE	1.00E-17
37711_at	-0.3597	-2.289286	0.095612	-0.80081	0.08142	TRUE	1.00E+00
33526_at	-0.35941	-2.287758	0.052631	-0.60223	-0.11659	FALSE	1.08E-07
37109_at	-0.3587	-2.284021	0.089113	-0.76983	0.05243	TRUE	7.19E-01
33589_at	-0.35795	-2.28008	0.060661	-0.63781	-0.07808	FALSE	4.57E-05
36470_s_at	-0.35715	-2.275883	0.192963	-1.2474	0.5331	TRUE	1.00E+00
39914_r_at	-0.35664	-2.273212	0.055229	-0.61145	-0.10184	FALSE	1.34E-06
34114_at	-0.35658	-2.272898	0.110793	-0.86774	0.15457	TRUE	1.00E+00
39330_s_at	-0.35618	-2.270806	0.036145	-0.52294	-0.18942	FALSE	8.30E-19
34494_at	-0.35612	-2.270492	0.099001	-0.81287	0.10063	TRUE	1.00E+00
37178_at	-0.35575	-2.268559	0.059646	-0.63093	-0.08056	FALSE	3.10E-05
39453_at	-0.35573	-2.268454	0.132595	-0.96747	0.25601	TRUE	1.00E+00

38147_at	-0.35561	-2.267827	0.062534	-0.64411	-0.06711	FALSE	1.64E-04
35027_at	-0.35538	-2.266627	0.073163	-0.69292	-0.01784	FALSE	1.50E-02
32243_g_at	-0.35499	-2.264592	0.039141	-0.53558	-0.17441	FALSE	1.51E-15
35627_at	-0.35454	-2.262247	0.033764	-0.51032	-0.19877	FALSE	1.08E-21
38731_at	-0.35424	-2.260685	0.081334	-0.72948	0.021	TRUE	1.68E-01
32778_at	-0.35422	-2.260581	0.089414	-0.76674	0.05829	TRUE	9.40E-01
40624_at	-0.35402	-2.25954	0.074943	-0.69978	-0.00827	FALSE	2.92E-02
33231_at	-0.35394	-2.259124	0.053616	-0.6013	-0.10657	FALSE	5.15E-07
32200_at	-0.35303	-2.254395	0.053357	-0.5992	-0.10687	FALSE	4.64E-07
35668_at	-0.35275	-2.252942	0.16184	-1.09942	0.39391	TRUE	1.00E+00
34523_at	-0.35146	-2.24626	0.053079	-0.59634	-0.10658	FALSE	4.49E-07
38905_at	-0.35144	-2.246156	0.086155	-0.74892	0.04604	TRUE	5.71E-01
38078_at	-0.35088	-2.243262	0.029342	-0.48625	-0.21551	FALSE	7.41E-29
40734_r_at	-0.35084	-2.243055	0.158153	-1.08049	0.37881	TRUE	1.00E+00
1109_s_at	-0.3501	-2.239237	0.103799	-0.82899	0.12879	TRUE	1.00E+00
36424_at	-0.34993	-2.23836	0.049495	-0.57828	-0.12158	FALSE	1.96E-08
31834_r_at	-0.34974	-2.237381	0.106955	-0.84318	0.14371	TRUE	1.00E+00
41112_at	-0.34863	-2.23167	0.124059	-0.92098	0.22373	TRUE	1.00E+00
33803_at	-0.34719	-2.224283	0.100348	-0.81015	0.11577	TRUE	1.00E+00
35028_at	-0.34628	-2.219627	0.074702	-0.69092	-0.00164	FALSE	4.50E-02
40943_at	-0.34618	-2.219116	0.046285	-0.55973	-0.13264	FALSE	9.43E-10
36341_s_at	-0.34564	-2.216358	0.113433	-0.86897	0.1777	TRUE	1.00E+00
770_at	-0.34435	-2.209785	0.121439	-0.90462	0.21592	TRUE	1.00E+00
1530_g_at	-0.34386	-2.207293	0.071135	-0.67205	-0.01568	FALSE	1.69E-02
31606_at	-0.34368	-2.206378	0.091236	-0.7646	0.07724	TRUE	1.00E+00
31375_at	-0.34273	-2.201557	0.107882	-0.84045	0.155	TRUE	1.00E+00
1743_s_at	-0.34243	-2.200037	0.115093	-0.87342	0.18856	TRUE	1.00E+00
34431_at	-0.34149	-2.19528	0.061464	-0.62507	-0.05792	FALSE	3.48E-04
36265_at	-0.34133	-2.194472	0.106002	-0.83038	0.14772	TRUE	1.00E+00
37416_at	-0.34119	-2.193764	0.050497	-0.57416	-0.10822	FALSE	1.78E-07
32072_at	-0.34097	-2.192653	0.055098	-0.59517	-0.08677	FALSE	7.67E-06
32270_g_at	-0.34095	-2.192552	0.104629	-0.82366	0.14177	TRUE	1.00E+00
31645_at	-0.34066	-2.191089	0.152611	-1.04474	0.36343	TRUE	1.00E+00
34082_at	-0.34031	-2.189324	0.042253	-0.53524	-0.14537	FALSE	1.01E-11
186_at	-0.3398	-2.186754	0.076855	-0.69438	0.01478	TRUE	1.24E-01
36383_at	-0.33941	-2.184792	0.095641	-0.78066	0.10183	TRUE	1.00E+00
40717_at	-0.33929	-2.184188	0.111911	-0.8556	0.17702	TRUE	1.00E+00
34616_at	-0.33848	-2.180118	0.113056	-0.86007	0.18311	TRUE	1.00E+00
32899_s_at	-0.33834	-2.179415	0.120853	-0.8959	0.21923	TRUE	1.00E+00
37817_at	-0.33821	-2.178763	0.052791	-0.58177	-0.09466	FALSE	1.88E-06
987_g_at	-0.33804	-2.17791	0.064906	-0.63749	-0.03859	FALSE	2.41E-03
39781_at	-0.33769	-2.176156	0.044361	-0.54236	-0.13303	FALSE	3.40E-10
40926_at	-0.33757	-2.175555	0.058053	-0.6054	-0.06974	FALSE	7.66E-05
32148_at	-0.33719	-2.173652	0.040202	-0.52267	-0.15172	FALSE	6.27E-13
40666_at	-0.33715	-2.173452	0.092793	-0.76526	0.09095	TRUE	1.00E+00
37868_s_at	-0.33664	-2.170901	0.111592	-0.85148	0.1782	TRUE	1.00E+00
35382_at	-0.33562	-2.165808	0.071553	-0.66573	-0.00551	FALSE	3.44E-02
36801_at	-0.33511	-2.163266	0.075585	-0.68383	0.01361	TRUE	1.17E-01
40547_at	-0.33457	-2.160578	0.103493	-0.81204	0.14291	TRUE	1.00E+00
33812_at	-0.33456	-2.160529	0.035966	-0.50049	-0.16862	FALSE	1.74E-16
34973_at	-0.33425	-2.158987	0.12293	-0.9014	0.2329	TRUE	1.00E+00

40964_at	-0.33403	-2.157893	0.064837	-0.63316	-0.0349	FALSE	3.26E-03
2058_s_at	-0.33373	-2.156403	0.035253	-0.49637	-0.17109	FALSE	3.64E-17
35857_at	-0.33336	-2.154567	0.107568	-0.82964	0.16291	TRUE	1.00E+00
36014_at	-0.33307	-2.153129	0.0671	-0.64264	-0.0235	FALSE	8.73E-03
636_at	-0.33281	-2.15184	0.071213	-0.66135	-0.00426	FALSE	3.74E-02
38949_at	-0.33266	-2.151097	0.107032	-0.82646	0.16114	TRUE	1.00E+00
1123_at	-0.3325	-2.150305	0.063772	-0.62671	-0.03828	FALSE	2.34E-03
33508_at	-0.33243	-2.149958	0.084292	-0.72132	0.05646	TRUE	1.00E+00
33704_at	-0.33209	-2.148276	0.070443	-0.65708	-0.00709	FALSE	3.06E-02
33014_at	-0.33167	-2.146199	0.07173	-0.6626	-0.00074	FALSE	4.76E-02
38168_at	-0.33165	-2.1461	0.070079	-0.65496	-0.00833	FALSE	2.80E-02
37478_at	-0.33119	-2.143828	0.039024	-0.51123	-0.15115	FALSE	2.68E-13
1618_at	-0.33084	-2.142101	0.115707	-0.86466	0.20299	TRUE	1.00E+00
36116_at	-0.33035	-2.139686	0.051327	-0.56715	-0.09354	FALSE	1.55E-06
37182_at	-0.33016	-2.13875	0.100471	-0.79369	0.13337	TRUE	1.00E+00
33923_s_at	-0.33009	-2.138405	0.138965	-0.97122	0.31103	TRUE	1.00E+00
1770_at	-0.32971	-2.136535	0.121422	-0.8899	0.23048	TRUE	1.00E+00
31830_s_at	-0.32898	-2.132947	0.042767	-0.52629	-0.13167	FALSE	1.82E-10
40763_at	-0.32878	-2.131965	0.139516	-0.97245	0.31489	TRUE	1.00E+00
33786_r_at	-0.3286	-2.131081	0.155614	-1.04654	0.38934	TRUE	1.00E+00
36730_at	-0.32816	-2.128923	0.097902	-0.77984	0.12352	TRUE	1.00E+00
36546_r_at	-0.32783	-2.127306	0.194826	-1.22668	0.57102	TRUE	1.00E+00
31451_at	-0.32752	-2.125788	0.072572	-0.66234	0.0073	TRUE	8.07E-02
39106_at	-0.32725	-2.124467	0.074742	-0.67208	0.01757	TRUE	1.51E-01
35792_at	-0.32717	-2.124076	0.030733	-0.46896	-0.18538	FALSE	2.31E-22
41109_at	-0.32678	-2.122169	0.117325	-0.86808	0.21451	TRUE	1.00E+00
38551_at	-0.32639	-2.120264	0.048989	-0.5524	-0.10037	FALSE	3.40E-07
38278_at	-0.32623	-2.119483	0.068387	-0.64174	-0.01072	FALSE	2.32E-02
31363_at	-0.3259	-2.117873	0.052257	-0.56699	-0.0848	FALSE	5.65E-06
40357_at	-0.32588	-2.117776	0.055109	-0.58013	-0.07163	FALSE	4.23E-05
38057_at	-0.32573	-2.117045	0.114024	-0.85179	0.20032	TRUE	1.00E+00
34331_at	-0.32535	-2.115193	0.044424	-0.5303	-0.1204	FALSE	3.04E-09
32483_at	-0.32528	-2.114852	0.076559	-0.6785	0.02793	TRUE	2.71E-01
32873_at	-0.32473	-2.112175	0.084835	-0.71612	0.06666	TRUE	1.00E+00
32035_at	-0.32472	-2.112127	0.061385	-0.60792	-0.04151	FALSE	1.55E-03
36321_at	-0.32391	-2.108191	0.150393	-1.01776	0.36995	TRUE	1.00E+00
37627_g_at	-0.32387	-2.107997	0.07717	-0.6799	0.03216	TRUE	3.42E-01
1658_g_at	-0.32382	-2.107754	0.090507	-0.74138	0.09375	TRUE	1.00E+00
38238_at	-0.32377	-2.107512	0.073946	-0.66492	0.01739	TRUE	1.51E-01
37845_at	-0.32351	-2.10625	0.103248	-0.79985	0.15284	TRUE	1.00E+00
37484_at	-0.32316	-2.104554	0.033524	-0.47783	-0.16849	FALSE	6.88E-18
37435_s_at	-0.32287	-2.103149	0.094792	-0.7602	0.11446	TRUE	1.00E+00
1529_at	-0.3226	-2.101842	0.048838	-0.54792	-0.09728	FALSE	5.01E-07
36008_at	-0.32259	-2.101793	0.08866	-0.73163	0.08645	TRUE	1.00E+00
40918_at	-0.32241	-2.100922	0.091228	-0.7433	0.09848	TRUE	1.00E+00
40093_at	-0.32201	-2.098988	0.049037	-0.54825	-0.09578	FALSE	6.49E-07
31967_at	-0.32152	-2.096621	0.097589	-0.77175	0.12872	TRUE	1.00E+00
35877_at	-0.31981	-2.088382	0.097797	-0.771	0.13138	TRUE	1.00E+00
affx-phex-3_at	-0.31925	-2.085691	0.098828	-0.7752	0.1367	TRUE	1.00E+00
39652_at	-0.31906	-2.084779	0.140097	-0.96541	0.32729	TRUE	1.00E+00
35082_at	-0.31833	-2.081278	0.071445	-0.64794	0.01129	TRUE	1.06E-01

38975_at	-0.31829	-2.081086	0.043823	-0.52047	-0.11611	FALSE	4.77E-09
35966_at	-0.31798	-2.079601	0.054335	-0.56865	-0.0673	FALSE	6.13E-05
37068_at	-0.31782	-2.078835	0.137075	-0.95023	0.31458	TRUE	1.00E+00
624_at	-0.31742	-2.076921	0.058975	-0.5895	-0.04533	FALSE	9.29E-04
38536_at	-0.31673	-2.073624	0.131516	-0.92348	0.29003	TRUE	1.00E+00
34403_at	-0.31646	-2.072335	0.038167	-0.49255	-0.14037	FALSE	1.41E-12
affx-humisgf3a/m	-0.31615	-2.070856	0.040865	-0.50468	-0.12762	FALSE	1.29E-10
38298_at	-0.31613	-2.070761	0.045584	-0.52644	-0.10583	FALSE	5.12E-08
755_at	-0.31605	-2.07038	0.055015	-0.56987	-0.06224	FALSE	1.16E-04
36807_at	-0.31602	-2.070237	0.077963	-0.67571	0.04366	TRUE	6.37E-01
37514_s_at	-0.31591	-2.069712	0.057581	-0.58157	-0.05026	FALSE	5.18E-04
785_at	-0.31576	-2.068998	0.035796	-0.4809	-0.15061	FALSE	1.43E-14
34071_at	-0.31553	-2.067902	0.050388	-0.548	-0.08306	FALSE	4.80E-06
37850_at	-0.31547	-2.067617	0.036091	-0.48197	-0.14896	FALSE	2.92E-14
40799_at	-0.31539	-2.067236	0.068755	-0.6326	0.00182	TRUE	5.67E-02
39371_at	-0.31504	-2.06557	0.054018	-0.56425	-0.06582	FALSE	6.91E-05
1788_s_at	-0.31482	-2.064524	0.128951	-0.90975	0.2801	TRUE	1.00E+00
32022_at	-0.31458	-2.063384	0.099537	-0.7738	0.14465	TRUE	1.00E+00
37608_g_at	-0.3145	-2.063004	0.065463	-0.61652	-0.01248	FALSE	1.96E-02
41270_at	-0.31396	-2.06044	0.052387	-0.55566	-0.07227	FALSE	2.60E-05
39188_at	-0.31379	-2.059634	0.084122	-0.70189	0.07432	TRUE	1.00E+00
33128_s_at	-0.31375	-2.059444	0.036549	-0.48237	-0.14513	FALSE	1.15E-13
39662_s_at	-0.31343	-2.057927	0.059639	-0.58858	-0.03828	FALSE	1.86E-03
677_s_at	-0.31342	-2.05788	0.078753	-0.67675	0.04992	TRUE	8.71E-01
39467_f_at	-0.31287	-2.055275	0.056655	-0.57425	-0.05149	FALSE	4.22E-04
40103_at	-0.31279	-2.054897	0.032032	-0.46058	-0.16501	FALSE	2.01E-18
35188_at	-0.31264	-2.054187	0.052817	-0.55632	-0.06897	FALSE	4.08E-05
40871_at	-0.31254	-2.053714	0.097776	-0.76364	0.13856	TRUE	1.00E+00
917_g_at	-0.31208	-2.05154	0.132112	-0.92159	0.29743	TRUE	1.00E+00
35438_at	-0.31161	-2.049321	0.039714	-0.49484	-0.12839	FALSE	5.40E-11
32506_at	-0.31152	-2.048896	0.047024	-0.52847	-0.09457	FALSE	4.39E-07
37110_at	-0.31152	-2.048896	0.085719	-0.70699	0.08396	TRUE	1.00E+00
39125_at	-0.31139	-2.048283	0.079613	-0.67869	0.05591	TRUE	1.00E+00
36547_r_at	-0.31129	-2.047812	0.0345	-0.47045	-0.15212	FALSE	2.31E-15
31782_at	-0.31128	-2.047764	0.086368	-0.70974	0.08719	TRUE	1.00E+00
36328_at	-0.31108	-2.046822	0.065369	-0.61267	-0.0095	FALSE	2.46E-02
36025_at	-0.31108	-2.046822	0.085803	-0.70694	0.08478	TRUE	1.00E+00
38671_at	-0.31082	-2.045597	0.036672	-0.48001	-0.14163	FALSE	2.95E-13
36364_at	-0.31057	-2.044419	0.064342	-0.60742	-0.01372	FALSE	1.75E-02
41090_s_at	-0.31029	-2.043102	0.10412	-0.79066	0.17008	TRUE	1.00E+00
1190_at	-0.30958	-2.039764	0.084728	-0.70048	0.08132	TRUE	1.00E+00
36094_at	-0.30956	-2.03967	0.041554	-0.50127	-0.11785	FALSE	1.18E-09
32988_at	-0.30892	-2.036667	0.047127	-0.52634	-0.0915	FALSE	7.02E-07
40317_at	-0.30853	-2.034839	0.047029	-0.5255	-0.09155	FALSE	6.78E-07
37579_at	-0.30852	-2.034792	0.085062	-0.70096	0.08393	TRUE	1.00E+00
39614_at	-0.30829	-2.033715	0.034568	-0.46777	-0.14881	FALSE	5.97E-15
36401_at	-0.30756	-2.030299	0.051624	-0.54574	-0.06939	FALSE	3.23E-05
39224_at	-0.30737	-2.029411	0.098877	-0.76355	0.14881	TRUE	1.00E+00
1491_at	-0.30735	-2.029317	0.068926	-0.62534	0.01064	TRUE	1.04E-01
40193_at	-0.30734	-2.029271	0.035907	-0.47301	-0.14168	FALSE	1.43E-13
39527_at	-0.30717	-2.028477	0.122963	-0.87447	0.26014	TRUE	1.00E+00

36705_at	-0.30652	-2.025443	0.048666	-0.53104	-0.08199	FALSE	3.80E-06
32283_at	-0.30597	-2.022879	0.106736	-0.7984	0.18647	TRUE	1.00E+00
40380_at	-0.30596	-2.022833	0.136166	-0.93418	0.32225	TRUE	1.00E+00
41802_at	-0.30566	-2.021436	0.05592	-0.56365	-0.04767	FALSE	5.81E-04
38498_at	-0.30516	-2.01911	0.059653	-0.58037	-0.02994	FALSE	3.95E-03
37781_at	-0.30474	-2.017158	0.083939	-0.692	0.08253	TRUE	1.00E+00
41599_at	-0.30415	-2.01442	0.08672	-0.70424	0.09595	TRUE	1.00E+00
38958_at	-0.30391	-2.013307	0.094931	-0.74188	0.13406	TRUE	1.00E+00
38197_at	-0.3039	-2.013261	0.07793	-0.66344	0.05564	TRUE	1.00E+00
1575_at	-0.30363	-2.012009	0.04063	-0.49108	-0.11618	FALSE	9.89E-10
34553_at	-0.30262	-2.007336	0.093726	-0.73503	0.1298	TRUE	1.00E+00
34599_at	-0.3024	-2.006319	0.083525	-0.68775	0.08295	TRUE	1.00E+00
1634_s_at	-0.30214	-2.005118	0.086148	-0.69959	0.09531	TRUE	1.00E+00
36184_at	-0.30079	-1.998895	0.030721	-0.44253	-0.15906	FALSE	1.55E-18
34538_at	-0.30055	-1.997791	0.095953	-0.74324	0.14214	TRUE	1.00E+00
38293_s_at	-0.29983	-1.994481	0.107585	-0.79618	0.19652	TRUE	1.00E+00
34416_at	-0.29983	-1.994481	0.060079	-0.57701	-0.02265	FALSE	7.60E-03
31928_at	-0.2996	-1.993425	0.097299	-0.74849	0.1493	TRUE	1.00E+00
37120_at	-0.29958	-1.993334	0.096323	-0.74397	0.14481	TRUE	1.00E+00
39961_at	-0.29918	-1.991499	0.118881	-0.84765	0.24928	TRUE	1.00E+00
37996_s_at	-0.29893	-1.990353	0.04521	-0.50751	-0.09035	FALSE	4.79E-07
34026_at	-0.29881	-1.989803	0.065105	-0.59918	0.00156	TRUE	5.60E-02
34061_at	-0.29872	-1.98939	0.052769	-0.54217	-0.05526	FALSE	1.90E-04
33972_r_at	-0.29827	-1.98733	0.110813	-0.80951	0.21298	TRUE	1.00E+00
31713_s_at	-0.29824	-1.987193	0.041082	-0.48778	-0.1087	FALSE	4.90E-09
37712_g_at	-0.29729	-1.982851	0.005948	-0.32473	-0.26985	FALSE	0.00E+00
36440_at	-0.29714	-1.982166	0.072388	-0.63111	0.03683	TRUE	5.11E-01
38111_at	-0.29684	-1.980797	0.041524	-0.48841	-0.10526	FALSE	1.11E-08
41011_i_at	-0.29637	-1.978655	0.130852	-0.90007	0.30733	TRUE	1.00E+00
1972_s_at	-0.29596	-1.976788	0.042784	-0.49334	-0.09857	FALSE	5.81E-08
1485_at	-0.29578	-1.975968	0.165975	-1.06152	0.46996	TRUE	1.00E+00
33053_at	-0.29563	-1.975286	0.054573	-0.54741	-0.04385	FALSE	7.64E-04
34137_at	-0.2953	-1.973786	0.108019	-0.79365	0.20305	TRUE	1.00E+00
35895_at	-0.29458	-1.970516	0.100454	-0.75804	0.16887	TRUE	1.00E+00
39049_at	-0.29443	-1.969836	0.053712	-0.54223	-0.04662	FALSE	5.32E-04
411_i_at	-0.29395	-1.96766	0.047181	-0.51162	-0.07628	FALSE	5.88E-06
39874_at	-0.29392	-1.967524	0.118692	-0.84152	0.25368	TRUE	1.00E+00
2054_g_at	-0.29377	-1.966844	0.058533	-0.56382	-0.02372	FALSE	6.56E-03
39084_at	-0.29359	-1.966029	0.110709	-0.80435	0.21718	TRUE	1.00E+00
482_at	-0.29314	-1.963993	0.056986	-0.55605	-0.03024	FALSE	3.39E-03
39148_s_at	-0.29307	-1.963677	0.111831	-0.80902	0.22287	TRUE	1.00E+00
33629_at	-0.29283	-1.962592	0.034896	-0.45383	-0.13184	FALSE	6.06E-13
1983_at	-0.29283	-1.962592	0.046396	-0.50688	-0.07878	FALSE	3.49E-06
37401_g_at	-0.2924	-1.96065	0.085116	-0.68509	0.10029	TRUE	1.00E+00
34919_at	-0.29237	-1.960514	0.062739	-0.58182	-0.00292	FALSE	3.99E-02
38750_at	-0.29224	-1.959927	0.030873	-0.43467	-0.1498	FALSE	3.68E-17
40318_at	-0.29205	-1.95907	0.081878	-0.6698	0.08571	TRUE	1.00E+00
33171_s_at	-0.29162	-1.957131	0.100257	-0.75417	0.17092	TRUE	1.00E+00
1385_at	-0.29142	-1.95623	0.058037	-0.55917	-0.02366	FALSE	6.48E-03
32114_s_at	-0.29124	-1.95542	0.049733	-0.52068	-0.06179	FALSE	5.98E-05
31953_f_at	-0.29123	-1.955375	0.070041	-0.61437	0.03191	TRUE	4.05E-01

33044_f_at	-0.29096	-1.954159	0.083962	-0.67833	0.0964	TRUE	1.00E+00
108_g_at	-0.29074	-1.95317	0.075952	-0.64115	0.05967	TRUE	1.00E+00
36596_r_at	-0.29021	-1.950788	0.102663	-0.76385	0.18344	TRUE	1.00E+00
1657_at	-0.28955	-1.947825	0.108703	-0.79106	0.21196	TRUE	1.00E+00
33990_at	-0.28953	-1.947736	0.095685	-0.73098	0.15192	TRUE	1.00E+00
41210_at	-0.28941	-1.947197	0.083639	-0.67528	0.09647	TRUE	1.00E+00
37530_s_at	-0.28904	-1.945539	0.052342	-0.53053	-0.04756	FALSE	4.23E-04
31570_at	-0.28894	-1.945091	0.067239	-0.59916	0.02127	TRUE	2.18E-01
34910_s_at	-0.28874	-1.944196	0.043928	-0.4914	-0.08608	FALSE	6.22E-07
372_f_at	-0.28862	-1.943659	0.076348	-0.64086	0.06362	TRUE	1.00E+00
41867_at	-0.28811	-1.941378	0.030423	-0.42847	-0.14775	FALSE	3.53E-17
33547_i_at	-0.28803	-1.94102	0.130831	-0.89163	0.31557	TRUE	1.00E+00
41847_at	-0.28742	-1.938296	0.1179	-0.83136	0.25652	TRUE	1.00E+00
39754_at	-0.28735	-1.937983	0.019999	-0.37962	-0.19508	FALSE	1.04E-42
1015_s_at	-0.28723	-1.937448	0.058193	-0.55571	-0.01876	FALSE	1.01E-02
38848_at	-0.2872	-1.937314	0.093897	-0.7204	0.146	TRUE	1.00E+00
40374_at	-0.28658	-1.93455	0.033922	-0.44308	-0.13008	FALSE	3.74E-13
36788_at	-0.28655	-1.934417	0.095976	-0.72934	0.15624	TRUE	1.00E+00
39886_at	-0.28633	-1.933437	0.113937	-0.81199	0.23933	TRUE	1.00E+00
31831_at	-0.28583	-1.931212	0.064625	-0.58398	0.01232	TRUE	1.23E-01
33241_at	-0.28558	-1.930101	0.098439	-0.73974	0.16858	TRUE	1.00E+00
37137_at	-0.28557	-1.930056	0.094159	-0.71998	0.14885	TRUE	1.00E+00
40209_at	-0.28553	-1.929879	0.134592	-0.90648	0.33542	TRUE	1.00E+00
41023_at	-0.28531	-1.928901	0.065584	-0.58789	0.01727	TRUE	1.72E-01
37423_at	-0.28505	-1.927747	0.039323	-0.46647	-0.10362	FALSE	5.31E-09
36346_at	-0.28453	-1.92544	0.067654	-0.59665	0.0276	TRUE	3.29E-01
34439_at	-0.28431	-1.924465	0.133083	-0.8983	0.32968	TRUE	1.00E+00
31471_at	-0.28402	-1.92318	0.049878	-0.51413	-0.0539	FALSE	1.56E-04
32537_at	-0.28387	-1.922516	0.095722	-0.72549	0.15775	TRUE	1.00E+00
32012_at	-0.28374	-1.921941	0.087348	-0.68672	0.11925	TRUE	1.00E+00
41610_at	-0.28356	-1.921144	0.033246	-0.43694	-0.13017	FALSE	1.86E-13
39974_at	-0.283	-1.918669	0.13665	-0.91345	0.34744	TRUE	1.00E+00
31496_g_at	-0.2827	-1.917344	0.059653	-0.55792	-0.00749	FALSE	2.71E-02
37149_s_at	-0.28266	-1.917167	0.084576	-0.67286	0.10754	TRUE	1.00E+00
40747_at	-0.28263	-1.917035	0.060649	-0.56244	-0.00282	FALSE	3.99E-02
31560_at	-0.28261	-1.916947	0.073013	-0.61946	0.05424	TRUE	1.00E+00
39890_at	-0.28252	-1.916549	0.070226	-0.60652	0.04147	TRUE	7.25E-01
31421_at	-0.28197	-1.914124	0.071708	-0.6128	0.04886	TRUE	1.00E+00
40356_at	-0.28125	-1.910953	0.102812	-0.75558	0.19308	TRUE	1.00E+00
32470_at	-0.28075	-1.908754	0.080242	-0.65095	0.08946	TRUE	1.00E+00
37117_at	-0.2803	-1.906777	0.105583	-0.76741	0.20682	TRUE	1.00E+00
38587_at	-0.28022	-1.906426	0.131905	-0.88878	0.32833	TRUE	1.00E+00
31345_at	-0.27997	-1.905329	0.077259	-0.63641	0.07647	TRUE	1.00E+00
36979_at	-0.27976	-1.904408	0.038558	-0.45765	-0.10187	FALSE	5.05E-09
39186_s_at	-0.27923	-1.902085	0.074856	-0.62459	0.06612	TRUE	1.00E+00
40010_s_at	-0.27912	-1.901604	0.078468	-0.64114	0.08289	TRUE	1.00E+00
37071_at	-0.27895	-1.900859	0.057995	-0.54652	-0.01139	FALSE	1.91E-02
32947_at	-0.27882	-1.900291	0.042563	-0.47519	-0.08246	FALSE	7.22E-07
882_at	-0.27841	-1.898497	0.09812	-0.7311	0.17428	TRUE	1.00E+00
36204_at	-0.27823	-1.897711	0.039374	-0.45988	-0.09657	FALSE	2.01E-08
32105_f_at	-0.27811	-1.897186	0.055482	-0.53408	-0.02214	FALSE	6.78E-03

36450_at	-0.27775	-1.895614	0.110399	-0.78709	0.23158	TRUE	1.00E+00
41098_at	-0.27761	-1.895003	0.048147	-0.49974	-0.05548	FALSE	1.03E-04
35901_at	-0.27751	-1.894567	0.12194	-0.84009	0.28507	TRUE	1.00E+00
649_s_at	-0.27747	-1.894393	0.087707	-0.68211	0.12717	TRUE	1.00E+00
33212_at	-0.27724	-1.89339	0.041584	-0.46909	-0.08539	FALSE	3.30E-07
36713_at	-0.27721	-1.893259	0.103933	-0.75671	0.2023	TRUE	1.00E+00
37326_at	-0.27695	-1.892126	0.046183	-0.49002	-0.06388	FALSE	2.54E-05
37351_at	-0.27678	-1.891385	0.04454	-0.48227	-0.07129	FALSE	6.51E-06
38022_s_at	-0.27643	-1.889862	0.06855	-0.59269	0.03983	TRUE	6.96E-01
32796_f_at	-0.27642	-1.889818	0.063803	-0.57078	0.01794	TRUE	1.86E-01
34619_at	-0.27637	-1.889601	0.085156	-0.66924	0.1165	TRUE	1.00E+00
35766_at	-0.2763	-1.889296	0.053163	-0.52158	-0.03103	FALSE	2.55E-03
1402_at	-0.27613	-1.888557	0.068573	-0.5925	0.04023	TRUE	7.14E-01
32298_at	-0.27608	-1.888339	0.14601	-0.94971	0.39755	TRUE	1.00E+00
35024_at	-0.27608	-1.888339	0.085032	-0.66838	0.11622	TRUE	1.00E+00
1763_at	-0.27583	-1.887252	0.070946	-0.60314	0.05149	TRUE	1.00E+00
37710_at	-0.27578	-1.887035	0.043564	-0.47676	-0.07479	FALSE	3.09E-06
40472_at	-0.27567	-1.886557	0.045888	-0.48738	-0.06396	FALSE	2.38E-05
32828_at	-0.27546	-1.885645	0.048026	-0.49704	-0.05389	FALSE	1.23E-04
37951_at	-0.27545	-1.885602	0.020699	-0.37095	-0.17996	FALSE	2.64E-36
1646_at	-0.27539	-1.885341	0.04994	-0.50579	-0.04499	FALSE	4.42E-04
31580_at	-0.27519	-1.884473	0.065444	-0.57713	0.02674	TRUE	3.30E-01
219_i_at	-0.27514	-1.884256	0.132528	-0.88657	0.3363	TRUE	1.00E+00
120_at	-0.27512	-1.88417	0.030353	-0.41515	-0.13508	FALSE	1.59E-15
37243_at	-0.2751	-1.884083	0.042078	-0.46923	-0.08097	FALSE	7.87E-07
35057_at	-0.27456	-1.881742	0.092348	-0.70062	0.15149	TRUE	1.00E+00
39209_r_at	-0.27452	-1.881568	0.110605	-0.78481	0.23577	TRUE	1.00E+00
32093_at	-0.27448	-1.881395	0.08646	-0.67337	0.12441	TRUE	1.00E+00
136_at	-0.27438	-1.880962	0.058099	-0.54242	-0.00633	FALSE	2.94E-02
36016_at	-0.27425	-1.880399	0.080392	-0.64515	0.09664	TRUE	1.00E+00
1690_at	-0.27384	-1.878625	0.128736	-0.86777	0.3201	TRUE	1.00E+00
311_s_at	-0.27371	-1.878062	0.042668	-0.47056	-0.07685	FALSE	1.78E-06
31563_at	-0.27365	-1.877803	0.141904	-0.92833	0.38104	TRUE	1.00E+00
34081_at	-0.27359	-1.877543	0.084739	-0.66454	0.11737	TRUE	1.00E+00
35969_at	-0.27357	-1.877457	0.067787	-0.58631	0.03917	TRUE	6.87E-01
40164_at	-0.27293	-1.874692	0.053218	-0.51845	-0.02741	FALSE	3.69E-03
41608_at	-0.27271	-1.873743	0.118717	-0.82042	0.275	TRUE	1.00E+00
39871_at	-0.27253	-1.872966	0.082135	-0.65147	0.10641	TRUE	1.00E+00
35033_at	-0.2721	-1.871113	0.04524	-0.48082	-0.06338	FALSE	2.28E-05
40373_at	-0.2721	-1.871113	0.138611	-0.91159	0.3674	TRUE	1.00E+00
34456_s_at	-0.27205	-1.870898	0.042501	-0.46814	-0.07597	FALSE	1.95E-06
39175_at	-0.27188	-1.870165	0.020937	-0.36847	-0.17528	FALSE	1.87E-34
33235_at	-0.27186	-1.870079	0.05065	-0.50553	-0.03818	FALSE	1.01E-03
160035_at	-0.27172	-1.869476	0.13765	-0.90678	0.36334	TRUE	1.00E+00
40153_at	-0.27128	-1.867583	0.07239	-0.60526	0.0627	TRUE	1.00E+00
1256_at	-0.2711	-1.866809	0.071098	-0.59911	0.05692	TRUE	1.00E+00
37210_at	-0.27097	-1.866251	0.030083	-0.40976	-0.13218	FALSE	2.67E-15
33593_at	-0.27086	-1.865778	0.075894	-0.62101	0.07928	TRUE	1.00E+00
33402_at	-0.27074	-1.865263	0.054344	-0.52146	-0.02002	FALSE	7.95E-03
38388_at	-0.27061	-1.864704	0.111191	-0.7836	0.24238	TRUE	1.00E+00
36221_at	-0.27045	-1.864018	0.031221	-0.41449	-0.12641	FALSE	5.84E-14

1085_s_at	-0.27043	-1.863932	0.05305	-0.51518	-0.02568	FALSE	4.34E-03
676_g_at	-0.27041	-1.863846	0.066131	-0.57551	0.03469	TRUE	5.47E-01
33642_s_at	-0.27032	-1.86346	0.091667	-0.69323	0.15259	TRUE	1.00E+00
32750_r_at	-0.27024	-1.863116	0.045731	-0.48123	-0.05926	FALSE	4.33E-05
31588_at	-0.27024	-1.863116	0.08655	-0.66955	0.12907	TRUE	1.00E+00
35946_at	-0.26984	-1.861401	0.084592	-0.66011	0.12043	TRUE	1.00E+00
36477_at	-0.26958	-1.860287	0.101735	-0.73894	0.19978	TRUE	1.00E+00
39824_at	-0.26884	-1.85712	0.078809	-0.63243	0.09475	TRUE	1.00E+00
41356_at	-0.26882	-1.857035	0.067762	-0.58145	0.0438	TRUE	9.18E-01
38861_at	-0.26863	-1.856222	0.150695	-0.96387	0.42662	TRUE	1.00E+00
34342_s_at	-0.26861	-1.856137	0.098386	-0.72252	0.18531	TRUE	1.00E+00
34980_at	-0.26853	-1.855795	0.076317	-0.62062	0.08357	TRUE	1.00E+00
39102_at	-0.26846	-1.855496	0.088534	-0.67692	0.14	TRUE	1.00E+00
40378_at	-0.26845	-1.855453	0.077916	-0.62792	0.09102	TRUE	1.00E+00
34017_s_at	-0.26805	-1.853745	0.093075	-0.69746	0.16136	TRUE	1.00E+00
38006_at	-0.2678	-1.852678	0.116422	-0.80492	0.26933	TRUE	1.00E+00
33088_at	-0.26761	-1.851868	0.029976	-0.40591	-0.12931	FALSE	5.50E-15
36484_at	-0.26736	-1.850802	0.117237	-0.80825	0.27352	TRUE	1.00E+00
35569_at	-0.26734	-1.850717	0.058112	-0.53545	0.00076	TRUE	5.32E-02
33779_at	-0.26723	-1.850248	0.130974	-0.87149	0.33703	TRUE	1.00E+00
37657_at	-0.26693	-1.848971	0.042974	-0.4652	-0.06867	FALSE	6.62E-06
33259_at	-0.2668	-1.848417	0.034345	-0.42525	-0.10834	FALSE	1.01E-10
31885_at	-0.26676	-1.848247	0.076199	-0.61831	0.08479	TRUE	1.00E+00
34450_at	-0.26662	-1.847651	0.096069	-0.70984	0.1766	TRUE	1.00E+00
1929_at	-0.26607	-1.845313	0.061978	-0.55201	0.01988	TRUE	2.23E-01
35828_at	-0.26592	-1.844676	0.028706	-0.39836	-0.13349	FALSE	2.49E-16
33217_at	-0.26575	-1.843954	0.074572	-0.6098	0.07829	TRUE	1.00E+00
500_at	-0.26564	-1.843487	0.095071	-0.70426	0.17298	TRUE	1.00E+00
33452_at	-0.26546	-1.842723	0.048522	-0.48932	-0.0416	FALSE	5.65E-04
41516_at	-0.26546	-1.842723	0.057808	-0.53216	0.00124	TRUE	5.54E-02
37499_at	-0.26513	-1.841323	0.104618	-0.74779	0.21754	TRUE	1.00E+00
1478_at	-0.2651	-1.841196	0.14018	-0.91183	0.38163	TRUE	1.00E+00
35775_at	-0.26487	-1.840221	0.056244	-0.52436	-0.00539	FALSE	3.14E-02
34438_at	-0.26472	-1.839586	0.04202	-0.45859	-0.07086	FALSE	3.76E-06
36670_at	-0.26465	-1.839289	0.015768	-0.3374	-0.19191	FALSE	3.99E-59
34467_g_at	-0.2645	-1.838654	0.09818	-0.71746	0.18847	TRUE	1.00E+00
114_r_at	-0.26423	-1.837511	0.138958	-0.90532	0.37687	TRUE	1.00E+00
33979_at	-0.26392	-1.8362	0.071389	-0.59328	0.06543	TRUE	1.00E+00
33177_at	-0.26369	-1.835228	0.074752	-0.60856	0.08119	TRUE	1.00E+00
41559_at	-0.26365	-1.835059	0.06106	-0.54535	0.01805	TRUE	1.99E-01
32103_at	-0.26357	-1.834721	0.015602	-0.33554	-0.19159	FALSE	6.33E-60
31328_at	-0.26352	-1.83451	0.070029	-0.58661	0.05957	TRUE	1.00E+00
35332_at	-0.26348	-1.834341	0.081217	-0.63818	0.11122	TRUE	1.00E+00
32003_at	-0.26337	-1.833876	0.057293	-0.5277	0.00096	TRUE	5.41E-02
35917_at	-0.26312	-1.832821	0.017256	-0.34273	-0.18351	FALSE	2.12E-48
1582_at	-0.26282	-1.831555	0.046964	-0.4795	-0.04615	FALSE	2.77E-04
39925_at	-0.26249	-1.830164	0.073176	-0.60009	0.07511	TRUE	1.00E+00
34155_s_at	-0.26225	-1.829153	0.119228	-0.81232	0.28782	TRUE	1.00E+00
1753_s_at	-0.26219	-1.8289	0.084094	-0.65016	0.12579	TRUE	1.00E+00
1202_g_at	-0.26199	-1.828058	0.049652	-0.49106	-0.03292	FALSE	1.66E-03
34872_at	-0.26189	-1.827637	0.070818	-0.58861	0.06484	TRUE	1.00E+00

39473_r_at	-0.26158	-1.826333	0.082545	-0.6424	0.11925	TRUE	1.00E+00
36644_at	-0.26141	-1.825618	0.032339	-0.4106	-0.11221	FALSE	7.95E-12
38946_at	-0.2614	-1.825576	0.07227	-0.59482	0.07202	TRUE	1.00E+00
1335_at	-0.26109	-1.824274	0.059217	-0.53429	0.01212	TRUE	1.31E-01
573_at	-0.26105	-1.824106	0.039561	-0.44357	-0.07854	FALSE	5.23E-07
1292_at	-0.26099	-1.823854	0.11407	-0.78726	0.26528	TRUE	1.00E+00
39221_at	-0.26056	-1.822049	0.078129	-0.62101	0.0999	TRUE	1.00E+00
33764_at	-0.26039	-1.821336	0.128633	-0.85385	0.33306	TRUE	1.00E+00
34969_s_at	-0.26027	-1.820833	0.065322	-0.56164	0.0411	TRUE	8.54E-01
38209_at	-0.26017	-1.820413	0.035676	-0.42476	-0.09558	FALSE	3.84E-09
36908_at	-0.26006	-1.819952	0.104488	-0.74212	0.22201	TRUE	1.00E+00
32538_at	-0.25982	-1.818947	0.062955	-0.55027	0.03063	TRUE	4.64E-01
1298_at	-0.25963	-1.818151	0.123858	-0.83106	0.3118	TRUE	1.00E+00
37457_at	-0.25957	-1.8179	0.046167	-0.47256	-0.04657	FALSE	2.38E-04
38964_r_at	-0.25955	-1.817816	0.057472	-0.5247	0.0056	TRUE	7.95E-02
32055_g_at	-0.25914	-1.816101	0.054488	-0.51052	-0.00775	FALSE	2.49E-02
41172_at	-0.25897	-1.81539	0.044921	-0.46622	-0.05172	FALSE	1.03E-04
35453_at	-0.25895	-1.815307	0.034496	-0.4181	-0.0998	FALSE	7.66E-10
37780_at	-0.25855	-1.813635	0.104525	-0.74078	0.22369	TRUE	1.00E+00
38567_at	-0.2581	-1.811757	0.060735	-0.53831	0.02211	TRUE	2.70E-01
34223_at	-0.25739	-1.808798	0.072092	-0.58999	0.07521	TRUE	1.00E+00
40753_at	-0.25727	-1.808298	0.066873	-0.56579	0.05125	TRUE	1.00E+00
38598_at	-0.2571	-1.80759	0.105845	-0.74543	0.23122	TRUE	1.00E+00
37057_s_at	-0.25688	-1.806675	0.029816	-0.39444	-0.11932	FALSE	8.81E-14
37558_at	-0.25687	-1.806633	0.063516	-0.54991	0.03616	TRUE	6.63E-01
619_s_at	-0.25677	-1.806217	0.129453	-0.85401	0.34048	TRUE	1.00E+00
807_at	-0.2566	-1.80551	0.051318	-0.49336	-0.01984	FALSE	7.23E-03
41520_at	-0.25641	-1.804721	0.082992	-0.63931	0.12648	TRUE	1.00E+00
31721_at	-0.25635	-1.804471	0.094281	-0.69132	0.17863	TRUE	1.00E+00
39314_at	-0.25596	-1.802852	0.052168	-0.49664	-0.01528	FALSE	1.17E-02
160025_at	-0.25589	-1.802561	0.107161	-0.75028	0.23851	TRUE	1.00E+00
31508_at	-0.25569	-1.801731	0.089593	-0.66903	0.15766	TRUE	1.00E+00
39491_s_at	-0.25546	-1.800777	0.072613	-0.59047	0.07954	TRUE	1.00E+00
36293_at	-0.25521	-1.799741	0.078266	-0.6163	0.10588	TRUE	1.00E+00
39178_at	-0.2551	-1.799285	0.053058	-0.49988	-0.01031	FALSE	1.93E-02
1025_g_at	-0.25441	-1.796429	0.051611	-0.49252	-0.0163	FALSE	1.04E-02
37956_at	-0.25425	-1.795767	0.084215	-0.64279	0.13428	TRUE	1.00E+00
37612_at	-0.25359	-1.79304	0.152478	-0.95706	0.44988	TRUE	1.00E+00
37332_r_at	-0.25359	-1.79304	0.066157	-0.55881	0.05163	TRUE	1.00E+00
31762_at	-0.25358	-1.792999	0.106669	-0.74571	0.23855	TRUE	1.00E+00
34721_at	-0.25347	-1.792545	0.087967	-0.65931	0.15237	TRUE	1.00E+00
40030_at	-0.25345	-1.792462	0.100418	-0.71674	0.20984	TRUE	1.00E+00
32176_at	-0.2528	-1.789781	0.154511	-0.96565	0.46005	TRUE	1.00E+00
1165_at	-0.25246	-1.788381	0.118721	-0.80019	0.29527	TRUE	1.00E+00
34309_at	-0.25246	-1.788381	0.060715	-0.53257	0.02766	TRUE	4.05E-01
32616_at	-0.2517	-1.785254	0.037219	-0.42341	-0.07999	FALSE	1.71E-07
37144_at	-0.25153	-1.784555	0.044164	-0.45529	-0.04778	FALSE	1.55E-04
32476_at	-0.25118	-1.783118	0.0491	-0.47771	-0.02465	FALSE	3.95E-03
39849_at	-0.25115	-1.782994	0.07709	-0.60681	0.10451	TRUE	1.00E+00
38037_at	-0.25105	-1.782584	0.127206	-0.83792	0.33583	TRUE	1.00E+00
41796_at	-0.25061	-1.780779	0.048	-0.47207	-0.02916	FALSE	2.24E-03

38730_at	-0.25056	-1.780574	0.011612	-0.30413	-0.19698	FALSE	3.77E-99
33544_at	-0.25047	-1.780205	0.108568	-0.75136	0.25042	TRUE	1.00E+00
31347_at	-0.25031	-1.779549	0.04348	-0.45091	-0.04972	FALSE	1.08E-04
34711_at	-0.2503	-1.779508	0.062132	-0.53695	0.03634	TRUE	7.08E-01
708_at	-0.2501	-1.778689	0.082484	-0.63064	0.13045	TRUE	1.00E+00
36559_g_at	-0.24987	-1.777747	0.162705	-1.00053	0.50078	TRUE	1.00E+00
31513_at	-0.24877	-1.77325	0.051367	-0.48576	-0.01179	FALSE	1.61E-02
35701_at	-0.24865	-1.77276	0.039654	-0.4316	-0.0657	FALSE	4.55E-06
1705_s_at	-0.24814	-1.77068	0.119609	-0.79997	0.30369	TRUE	1.00E+00
32673_at	-0.24799	-1.770068	0.027418	-0.37448	-0.1215	FALSE	1.89E-15
31459_i_at	-0.24782	-1.769375	0.085931	-0.64427	0.14863	TRUE	1.00E+00
39612_at	-0.24766	-1.768724	0.064418	-0.54485	0.04954	TRUE	1.00E+00
41538_r_at	-0.24753	-1.768194	0.030152	-0.38664	-0.10842	FALSE	2.81E-12
35597_at	-0.24736	-1.767502	0.094777	-0.68462	0.1899	TRUE	1.00E+00
33608_at	-0.24724	-1.767014	0.076868	-0.60188	0.1074	TRUE	1.00E+00
34624_at	-0.24719	-1.766811	0.094947	-0.68523	0.19086	TRUE	1.00E+00
39892_at	-0.24703	-1.76616	0.07111	-0.57511	0.08104	TRUE	1.00E+00
41605_at	-0.2469	-1.765631	0.021272	-0.34504	-0.14876	FALSE	4.80E-27
32387_at	-0.24677	-1.765103	0.049675	-0.47595	-0.01759	FALSE	8.55E-03
35375_at	-0.24667	-1.764696	0.084851	-0.63814	0.14479	TRUE	1.00E+00
31859_at	-0.2463	-1.763194	0.106528	-0.73777	0.24518	TRUE	1.00E+00
32900_at	-0.24615	-1.762585	0.070874	-0.57313	0.08083	TRUE	1.00E+00
36192_at	-0.24609	-1.762341	0.025203	-0.36236	-0.12981	FALSE	2.02E-18
38213_at	-0.24587	-1.761449	0.068612	-0.56242	0.07068	TRUE	1.00E+00
33576_at	-0.24517	-1.758612	0.071862	-0.57671	0.08637	TRUE	1.00E+00
37440_at	-0.24492	-1.7576	0.077692	-0.60336	0.11352	TRUE	1.00E+00
31433_at	-0.2446	-1.756305	0.082442	-0.62495	0.13576	TRUE	1.00E+00
40694_at	-0.24457	-1.756184	0.059252	-0.51793	0.02879	TRUE	4.63E-01
39082_at	-0.24455	-1.756103	0.023178	-0.35148	-0.13762	FALSE	6.35E-22
40113_at	-0.24452	-1.755982	0.03959	-0.42717	-0.06187	FALSE	8.28E-06
39215_at	-0.24447	-1.75578	0.061027	-0.52603	0.03708	TRUE	7.80E-01
31650_g_at	-0.24427	-1.754971	0.064531	-0.54199	0.05345	TRUE	1.00E+00
40060_r_at	-0.2438	-1.753073	0.040643	-0.43132	-0.05629	FALSE	2.51E-05
867_s_at	-0.24364	-1.752427	0.049538	-0.47219	-0.01509	FALSE	1.10E-02
40865_at	-0.24362	-1.752347	0.020313	-0.33733	-0.1499	FALSE	4.88E-29
32905_s_at	-0.24338	-1.751378	0.099656	-0.70315	0.21639	TRUE	1.00E+00
36591_at	-0.24331	-1.751096	0.032201	-0.39187	-0.09475	FALSE	5.24E-10
39300_at	-0.2433	-1.751056	0.134425	-0.86348	0.37688	TRUE	1.00E+00
1880_at	-0.24322	-1.750733	0.09036	-0.6601	0.17367	TRUE	1.00E+00
31910_at	-0.24301	-1.749887	0.138402	-0.88154	0.39552	TRUE	1.00E+00
979_g_at	-0.24292	-1.749524	0.087855	-0.64825	0.1624	TRUE	1.00E+00
41330_s_at	-0.24251	-1.747874	0.073168	-0.58008	0.09506	TRUE	1.00E+00
1269_at	-0.2425	-1.747833	0.04946	-0.47068	-0.01431	FALSE	1.19E-02
33127_at	-0.24198	-1.745742	0.042343	-0.43733	-0.04663	FALSE	1.39E-04
31820_at	-0.24171	-1.744657	0.098994	-0.69843	0.21501	TRUE	1.00E+00
32485_at	-0.24168	-1.744536	0.078962	-0.60598	0.12262	TRUE	1.00E+00
32566_at	-0.24159	-1.744175	0.023706	-0.35096	-0.13222	FALSE	2.74E-20
35953_at	-0.24158	-1.744135	0.05027	-0.47351	-0.00966	FALSE	1.95E-02
36739_at	-0.24157	-1.744094	0.1562	-0.96221	0.47907	TRUE	1.00E+00
38692_at	-0.24145	-1.743613	0.02966	-0.37829	-0.10461	FALSE	4.96E-12
36271_at	-0.24136	-1.743251	0.095068	-0.67997	0.19724	TRUE	1.00E+00

36919_r_at	-0.2412	-1.742609	0.17736	-1.05947	0.57706	TRUE	1.00E+00
38644_at	-0.24113	-1.742328	0.034683	-0.40114	-0.08112	FALSE	4.53E-08
1598_g_at	-0.24107	-1.742088	0.029004	-0.37488	-0.10725	FALSE	1.19E-12
37546_r_at	-0.24015	-1.738401	0.082467	-0.62062	0.14032	TRUE	1.00E+00
41064_at	-0.24015	-1.738401	0.074997	-0.58616	0.10586	TRUE	1.00E+00
31653_at	-0.2401	-1.738201	0.091469	-0.66211	0.1819	TRUE	1.00E+00
41755_at	-0.24007	-1.738081	0.079105	-0.60503	0.12489	TRUE	1.00E+00
31621_s_at	-0.2397	-1.736601	0.088231	-0.64676	0.16736	TRUE	1.00E+00
33755_at	-0.23941	-1.735442	0.062759	-0.52896	0.05014	TRUE	1.00E+00
40219_at	-0.23877	-1.732886	0.043298	-0.43853	-0.03901	FALSE	4.41E-04
39484_at	-0.23847	-1.731689	0.046083	-0.45108	-0.02586	FALSE	2.88E-03
34083_at	-0.2384	-1.73141	0.047249	-0.45639	-0.02042	FALSE	5.70E-03
35052_r_at	-0.23795	-1.729617	0.126195	-0.82016	0.34426	TRUE	1.00E+00
39616_at	-0.23779	-1.72898	0.092019	-0.66232	0.18675	TRUE	1.00E+00
40362_at	-0.23741	-1.727468	0.102745	-0.71143	0.23661	TRUE	1.00E+00
38125_at	-0.23736	-1.727269	0.017596	-0.31854	-0.15618	FALSE	2.28E-37
36707_s_at	-0.23718	-1.726553	0.058895	-0.5089	0.03453	TRUE	7.12E-01
32970_f_at	-0.23681	-1.725083	0.046674	-0.45214	-0.02147	FALSE	4.93E-03
34105_f_at	-0.23674	-1.724805	0.041286	-0.42721	-0.04626	FALSE	1.24E-04
152_f_at	-0.23662	-1.724328	0.076429	-0.58924	0.11599	TRUE	1.00E+00
328_at	-0.23652	-1.723931	0.094423	-0.67215	0.19911	TRUE	1.00E+00
35280_at	-0.23649	-1.723812	0.127557	-0.82499	0.352	TRUE	1.00E+00
38145_at	-0.2361	-1.722265	0.14618	-0.91052	0.43831	TRUE	1.00E+00
33691_at	-0.23604	-1.722027	0.081295	-0.6111	0.13902	TRUE	1.00E+00
37658_at	-0.23569	-1.72064	0.039573	-0.41826	-0.05312	FALSE	3.27E-05
33714_at	-0.23563	-1.720402	0.1175	-0.77772	0.30647	TRUE	1.00E+00
38181_at	-0.23556	-1.720125	0.048847	-0.46092	-0.0102	FALSE	1.79E-02
39700_at	-0.23553	-1.720006	0.01571	-0.30801	-0.16305	FALSE	1.04E-46
32821_at	-0.23498	-1.717829	0.116969	-0.77462	0.30467	TRUE	1.00E+00
35636_at	-0.23491	-1.717552	0.081744	-0.61204	0.14222	TRUE	1.00E+00
34630_s_at	-0.23484	-1.717276	0.104906	-0.71884	0.24915	TRUE	1.00E+00
33100_at	-0.23438	-1.715458	0.139399	-0.87751	0.40875	TRUE	1.00E+00
35014_at	-0.23426	-1.714984	0.102234	-0.70593	0.2374	TRUE	1.00E+00
37472_at	-0.23403	-1.714076	0.029475	-0.37002	-0.09805	FALSE	2.55E-11
39177_r_at	-0.23378	-1.713089	0.048214	-0.45622	-0.01134	FALSE	1.57E-02
41653_at	-0.23372	-1.712853	0.068162	-0.54819	0.08075	TRUE	1.00E+00
31651_at	-0.23355	-1.712182	0.156707	-0.95654	0.48943	TRUE	1.00E+00
900_at	-0.23347	-1.711867	0.126045	-0.81499	0.34805	TRUE	1.00E+00
35728_at	-0.23318	-1.710724	0.087372	-0.63628	0.16992	TRUE	1.00E+00
36921_at	-0.23307	-1.710291	0.044275	-0.43734	-0.02881	FALSE	1.78E-03
1966_i_at	-0.23288	-1.709543	0.049574	-0.4616	-0.00417	FALSE	3.32E-02
32450_at	-0.23286	-1.709464	0.068969	-0.55105	0.08534	TRUE	1.00E+00
35051_at	-0.23284	-1.709385	0.081005	-0.60656	0.14089	TRUE	1.00E+00
affx-murfas_at	-0.2327	-1.708834	0.065572	-0.53522	0.06982	TRUE	1.00E+00
1029_s_at	-0.23257	-1.708323	0.126374	-0.81561	0.35046	TRUE	1.00E+00
34412_s_at	-0.23251	-1.708087	0.037911	-0.40741	-0.0576	FALSE	1.09E-05
36270_at	-0.23249	-1.708008	0.07964	-0.59991	0.13494	TRUE	1.00E+00
35476_at	-0.23242	-1.707733	0.133506	-0.84836	0.38352	TRUE	1.00E+00
35424_g_at	-0.23216	-1.706711	0.070397	-0.55694	0.09263	TRUE	1.00E+00
31786_at	-0.23215	-1.706672	0.072752	-0.56779	0.1035	TRUE	1.00E+00
39982_r_at	-0.23195	-1.705886	0.103848	-0.71106	0.24716	TRUE	1.00E+00

37447_at	-0.23194	-1.705847	0.041675	-0.42421	-0.03967	FALSE	3.30E-04
1285_at	-0.23181	-1.705336	0.133707	-0.84868	0.38506	TRUE	1.00E+00
395_at	-0.23178	-1.705218	0.091123	-0.65219	0.18862	TRUE	1.00E+00
33222_at	-0.23176	-1.70514	0.032408	-0.38127	-0.08224	FALSE	1.09E-08
1056_s_at	-0.23132	-1.703413	0.099035	-0.68822	0.22559	TRUE	1.00E+00
31755_at	-0.23111	-1.70259	0.09339	-0.66197	0.19975	TRUE	1.00E+00
36503_at	-0.23054	-1.700357	0.067547	-0.54217	0.0811	TRUE	1.00E+00
32773_at	-0.23027	-1.6993	0.110636	-0.7407	0.28016	TRUE	1.00E+00
36350_at	-0.23014	-1.698791	0.104444	-0.71199	0.25172	TRUE	1.00E+00
1821_at	-0.2301	-1.698635	0.084893	-0.62176	0.16156	TRUE	1.00E+00
38954_at	-0.22988	-1.697774	0.060453	-0.50879	0.04903	TRUE	1.00E+00
1451_s_at	-0.22985	-1.697657	0.089108	-0.64095	0.18126	TRUE	1.00E+00
31407_at	-0.22951	-1.696329	0.128327	-0.82155	0.36254	TRUE	1.00E+00
33957_at	-0.22918	-1.69504	0.124361	-0.80293	0.34458	TRUE	1.00E+00
33889_s_at	-0.22915	-1.694923	0.113737	-0.75389	0.29559	TRUE	1.00E+00
35852_at	-0.2291	-1.694728	0.047203	-0.44688	-0.01133	FALSE	1.53E-02
31366_at	-0.2291	-1.694728	0.050868	-0.46378	0.00559	TRUE	8.43E-02
31737_at	-0.22903	-1.694455	0.031376	-0.37378	-0.08427	FALSE	3.65E-09
34641_at	-0.22897	-1.694221	0.098739	-0.68451	0.22657	TRUE	1.00E+00
1704_at	-0.22888	-1.69387	0.084958	-0.62085	0.16308	TRUE	1.00E+00
753_at	-0.22856	-1.692622	0.046238	-0.44188	-0.01524	FALSE	9.71E-03
38904_at	-0.22855	-1.692583	0.082751	-0.61033	0.15323	TRUE	1.00E+00
552_at	-0.22826	-1.691453	0.014135	-0.29347	-0.16305	FALSE	1.46E-54
37986_at	-0.22819	-1.691181	0.051638	-0.46643	0.01004	TRUE	1.25E-01
39089_at	-0.22804	-1.690597	0.036361	-0.39579	-0.06028	FALSE	4.52E-06
33563_s_at	-0.22768	-1.689196	0.108856	-0.7299	0.27454	TRUE	1.00E+00
1065_at	-0.22759	-1.688846	0.070407	-0.55242	0.09724	TRUE	1.00E+00
32332_at	-0.22738	-1.688029	0.059262	-0.50079	0.04603	TRUE	1.00E+00
41676_at	-0.22735	-1.687913	0.051373	-0.46436	0.00966	TRUE	1.21E-01
39574_at	-0.22724	-1.687485	0.041165	-0.41715	-0.03732	FALSE	4.28E-04
31740_s_at	-0.22721	-1.687369	0.095315	-0.66695	0.21254	TRUE	1.00E+00
31781_at	-0.22717	-1.687213	0.09416	-0.66159	0.20724	TRUE	1.00E+00
2004_at	-0.22709	-1.686903	0.051017	-0.46246	0.00828	TRUE	1.08E-01
37259_at	-0.22707	-1.686825	0.055622	-0.48369	0.02954	TRUE	5.63E-01
32380_at	-0.22703	-1.68667	0.059524	-0.50165	0.04759	TRUE	1.00E+00
40284_at	-0.22694	-1.68632	0.078726	-0.59015	0.13627	TRUE	1.00E+00
41069_at	-0.2269	-1.686165	0.067026	-0.53613	0.08232	TRUE	1.00E+00
35251_at	-0.22688	-1.686087	0.051677	-0.46529	0.01154	TRUE	1.43E-01
35625_at	-0.22683	-1.685893	0.04011	-0.41188	-0.04177	FALSE	1.97E-04
37204_at	-0.22679	-1.685738	0.043148	-0.42585	-0.02772	FALSE	1.86E-03
33049_at	-0.22679	-1.685738	0.060927	-0.50788	0.0543	TRUE	1.00E+00
34088_at	-0.22674	-1.685544	0.023252	-0.33402	-0.11947	FALSE	2.29E-18
1536_at	-0.22667	-1.685272	0.188672	-1.09713	0.64378	TRUE	1.00E+00
34672_at	-0.22655	-1.684806	0.042245	-0.42145	-0.03165	FALSE	1.03E-03
33369_at	-0.22654	-1.684768	0.073435	-0.56534	0.11225	TRUE	1.00E+00
34162_at	-0.22647	-1.684496	0.043421	-0.4268	-0.02615	FALSE	2.31E-03
31452_at	-0.22628	-1.683759	0.101429	-0.69423	0.24168	TRUE	1.00E+00
33615_at	-0.22619	-1.68341	0.097659	-0.67675	0.22437	TRUE	1.00E+00
35181_at	-0.22606	-1.682907	0.044658	-0.43209	-0.02003	FALSE	5.24E-03
39543_at	-0.22594	-1.682442	0.030795	-0.36802	-0.08387	FALSE	2.76E-09
40566_at	-0.22591	-1.682325	0.017497	-0.30663	-0.14518	FALSE	4.90E-34

31768_at	-0.22584	-1.682054	0.125657	-0.80556	0.35389	TRUE	1.00E+00
37800_r_at	-0.22542	-1.680428	0.097543	-0.67544	0.22461	TRUE	1.00E+00
35112_at	-0.22505	-1.678997	0.107243	-0.71982	0.26973	TRUE	1.00E+00
31430_at	-0.2249	-1.678418	0.092978	-0.65387	0.20406	TRUE	1.00E+00
32779_s_at	-0.22477	-1.677915	0.04209	-0.41896	-0.03059	FALSE	1.17E-03
31718_at	-0.22477	-1.677915	0.061415	-0.50812	0.05857	TRUE	1.00E+00
33759_at	-0.22474	-1.677799	0.0479	-0.44574	-0.00375	FALSE	3.42E-02
37920_at	-0.22455	-1.677065	0.042826	-0.42213	-0.02697	FALSE	1.99E-03
37328_at	-0.22427	-1.675985	0.174699	-1.03026	0.58172	TRUE	1.00E+00
35377_at	-0.22416	-1.67556	0.095252	-0.66361	0.2153	TRUE	1.00E+00
35054_at	-0.22384	-1.674326	0.099653	-0.6836	0.23592	TRUE	1.00E+00
35146_at	-0.22362	-1.673478	0.034238	-0.38158	-0.06566	FALSE	8.23E-07
32441_at	-0.2236	-1.673401	0.044483	-0.42883	-0.01838	FALSE	6.30E-03
35962_at	-0.22355	-1.673208	0.058429	-0.49311	0.04602	TRUE	1.00E+00
548_s_at	-0.22348	-1.672939	0.090006	-0.63873	0.19177	TRUE	1.00E+00
36288_at	-0.2233	-1.672245	0.036947	-0.39376	-0.05284	FALSE	1.90E-05
39757_at	-0.22328	-1.672168	0.036833	-0.39322	-0.05335	FALSE	1.70E-05
41144_g_at	-0.22318	-1.671783	0.029729	-0.36033	-0.08602	FALSE	7.64E-10
41517_g_at	-0.22296	-1.670937	0.03194	-0.37032	-0.0756	FALSE	3.71E-08
39504_at	-0.22281	-1.67036	0.062019	-0.50894	0.06332	TRUE	1.00E+00
1203_at	-0.22273	-1.670052	0.101738	-0.6921	0.24665	TRUE	1.00E+00
1201_at	-0.22262	-1.669629	0.060283	-0.50074	0.0555	TRUE	1.00E+00
31649_at	-0.22247	-1.669053	0.091913	-0.64652	0.20158	TRUE	1.00E+00
34068_f_at	-0.22228	-1.668322	0.083557	-0.60777	0.16322	TRUE	1.00E+00
36989_at	-0.22214	-1.667785	0.021825	-0.32284	-0.12145	FALSE	3.13E-20
40011_s_at	-0.22176	-1.666326	0.06395	-0.5168	0.07328	TRUE	1.00E+00
34248_at	-0.22175	-1.666288	0.071726	-0.55266	0.10917	TRUE	1.00E+00
36848_r_at	-0.2215	-1.665329	0.072485	-0.55591	0.11292	TRUE	1.00E+00
717_at	-0.22084	-1.6628	0.042761	-0.41812	-0.02356	FALSE	3.04E-03
36430_at	-0.22083	-1.662762	0.087045	-0.62242	0.18076	TRUE	1.00E+00
37112_at	-0.2206	-1.661881	0.056789	-0.4826	0.0414	TRUE	1.00E+00
37858_at	-0.22035	-1.660925	0.081537	-0.59653	0.15582	TRUE	1.00E+00
38322_at	-0.22025	-1.660543	0.104656	-0.70309	0.26259	TRUE	1.00E+00
339_at	-0.22018	-1.660275	0.028599	-0.35213	-0.08824	FALSE	1.73E-10
37639_at	-0.21978	-1.658746	0.052966	-0.46414	0.02459	TRUE	4.21E-01
38408_at	-0.21963	-1.658174	0.115426	-0.75215	0.3129	TRUE	1.00E+00
34593_g_at	-0.21949	-1.657639	0.052837	-0.46326	0.02428	TRUE	4.12E-01
31371_at	-0.21949	-1.657639	0.08579	-0.61529	0.17631	TRUE	1.00E+00
34461_at	-0.21945	-1.657487	0.121405	-0.77957	0.34066	TRUE	1.00E+00
1397_at	-0.21927	-1.6568	0.105584	-0.70639	0.26785	TRUE	1.00E+00
32886_at	-0.21917	-1.656418	0.0632	-0.51075	0.07241	TRUE	1.00E+00
2030_at	-0.21911	-1.656189	0.066949	-0.52799	0.08976	TRUE	1.00E+00
38432_at	-0.21853	-1.653979	0.033599	-0.37354	-0.06352	FALSE	9.87E-07
34937_at	-0.21834	-1.653256	0.066015	-0.52291	0.08622	TRUE	1.00E+00
33437_at	-0.21805	-1.652152	0.03978	-0.40158	-0.03452	FALSE	5.33E-04
36669_at	-0.218	-1.651962	0.048013	-0.43952	0.00351	TRUE	7.08E-02
2082_s_at	-0.21795	-1.651772	0.105343	-0.70396	0.26806	TRUE	1.00E+00
33511_at	-0.21795	-1.651772	0.091047	-0.638	0.2021	TRUE	1.00E+00
37074_at	-0.21787	-1.651467	0.03706	-0.38885	-0.04689	FALSE	5.22E-05
32347_at	-0.21785	-1.651391	0.077993	-0.57768	0.14198	TRUE	1.00E+00
35837_at	-0.21766	-1.650669	0.062195	-0.5046	0.06928	TRUE	1.00E+00

40603_at	-0.21765	-1.650631	0.083969	-0.60505	0.16974	TRUE	1.00E+00
553_g_at	-0.21757	-1.650327	0.005812	-0.24438	-0.19075	FALSE	1.30E-302
41383_at	-0.2174	-1.649681	0.053963	-0.46636	0.03157	TRUE	7.08E-01
1730_s_at	-0.21738	-1.649605	0.025587	-0.33543	-0.09934	FALSE	2.48E-13
35755_at	-0.21712	-1.648618	0.031456	-0.36225	-0.072	FALSE	6.45E-08
39526_at	-0.21697	-1.648049	0.061448	-0.50046	0.06653	TRUE	1.00E+00
36467_g_at	-0.21661	-1.646683	0.051342	-0.45348	0.02026	TRUE	3.10E-01
34051_at	-0.21655	-1.646456	0.121805	-0.77851	0.3454	TRUE	1.00E+00
41668_r_at	-0.21647	-1.646152	0.133628	-0.83297	0.40004	TRUE	1.00E+00
39476_at	-0.21642	-1.645963	0.063558	-0.50965	0.07681	TRUE	1.00E+00
40361_at	-0.21599	-1.644334	0.035469	-0.37963	-0.05235	FALSE	1.43E-05
37889_at	-0.21593	-1.644107	0.117862	-0.7597	0.32783	TRUE	1.00E+00
39193_at	-0.21593	-1.644107	0.024709	-0.32993	-0.10194	FALSE	2.97E-14
39758_f_at	-0.21582	-1.64369	0.046784	-0.43167	0.00002	TRUE	5.00E-02
33586_at	-0.2158	-1.643615	0.119467	-0.76697	0.33537	TRUE	1.00E+00
32612_at	-0.21569	-1.643198	0.029006	-0.34951	-0.08186	FALSE	1.31E-09
36762_at	-0.21568	-1.643161	0.061121	-0.49767	0.06631	TRUE	1.00E+00
36139_at	-0.21547	-1.642366	0.034349	-0.37395	-0.057	FALSE	4.47E-06
1072_g_at	-0.21539	-1.642064	0.0493	-0.44284	0.01206	TRUE	1.58E-01
37180_at	-0.21517	-1.641232	0.061874	-0.50063	0.07029	TRUE	1.00E+00
35103_i_at	-0.21507	-1.640854	0.058743	-0.48609	0.05594	TRUE	1.00E+00
37997_r_at	-0.21482	-1.63991	0.073734	-0.555	0.12535	TRUE	1.00E+00
39644_at	-0.2148	-1.639834	0.086458	-0.61368	0.18409	TRUE	1.00E+00
31565_at	-0.21478	-1.639759	0.034714	-0.37494	-0.05463	FALSE	7.73E-06
2081_s_at	-0.21471	-1.639495	0.103617	-0.69275	0.26334	TRUE	1.00E+00
40059_r_at	-0.21456	-1.638928	0.064837	-0.51369	0.08458	TRUE	1.00E+00
40127_at	-0.21445	-1.638513	0.026097	-0.33485	-0.09405	FALSE	2.62E-12
40107_at	-0.21432	-1.638023	0.030746	-0.35616	-0.07247	FALSE	3.98E-08
38553_r_at	-0.21419	-1.637533	0.047656	-0.43406	0.00567	TRUE	8.80E-02
32686_at	-0.21345	-1.634745	0.073866	-0.55424	0.12734	TRUE	1.00E+00
36114_r_at	-0.21327	-1.634068	0.128176	-0.80462	0.37808	TRUE	1.00E+00
33871_s_at	-0.21315	-1.633616	0.056224	-0.47254	0.04625	TRUE	1.00E+00
38997_at	-0.21311	-1.633466	0.038444	-0.39048	-0.03574	FALSE	3.75E-04
34885_at	-0.21295	-1.632864	0.035444	-0.37648	-0.04943	FALSE	2.37E-05
1130_at	-0.21286	-1.632526	0.025186	-0.32906	-0.09666	FALSE	3.63E-13
31364_i_at	-0.21278	-1.632225	0.070086	-0.53613	0.11057	TRUE	1.00E+00
34632_r_at	-0.21227	-1.630309	0.094741	-0.64937	0.22483	TRUE	1.00E+00
38446_at	-0.21175	-1.628358	0.145079	-0.88108	0.45759	TRUE	1.00E+00
34134_at	-0.21159	-1.627759	0.028007	-0.3408	-0.08238	FALSE	5.29E-10
33963_at	-0.21154	-1.627571	0.05964	-0.48669	0.06362	TRUE	1.00E+00
40043_at	-0.21152	-1.627496	0.060226	-0.48938	0.06633	TRUE	1.00E+00
936_s_at	-0.21151	-1.627459	0.072927	-0.54797	0.12494	TRUE	1.00E+00
1534_at	-0.21129	-1.626635	0.124508	-0.78572	0.36314	TRUE	1.00E+00
32868_at	-0.21106	-1.625773	0.044649	-0.41705	-0.00506	FALSE	2.88E-02
36096_at	-0.21102	-1.625624	0.075027	-0.55716	0.13513	TRUE	1.00E+00
467_at	-0.21095	-1.625362	0.035565	-0.37504	-0.04687	FALSE	3.79E-05
33007_at	-0.21074	-1.624576	0.086571	-0.61014	0.18867	TRUE	1.00E+00
35096_at	-0.21073	-1.624538	0.065573	-0.51326	0.09179	TRUE	1.00E+00
31965_at	-0.21068	-1.624351	0.059048	-0.4831	0.06174	TRUE	1.00E+00
34568_at	-0.21066	-1.624277	0.053272	-0.45643	0.03512	TRUE	9.69E-01
33929_at	-0.21062	-1.624127	0.032182	-0.35909	-0.06214	FALSE	7.53E-07

37754_at	-0.21059	-1.624015	0.051545	-0.44839	0.02722	TRUE	5.55E-01
33901_at	-0.21046	-1.623529	0.028326	-0.34114	-0.07978	FALSE	1.37E-09
31751_f_at	-0.21037	-1.623192	0.050727	-0.4444	0.02367	TRUE	4.25E-01
33172_at	-0.21018	-1.622482	0.036798	-0.37995	-0.04041	FALSE	1.41E-04
36743_at	-0.21008	-1.622109	0.056286	-0.46976	0.0496	TRUE	1.00E+00
34100_at	-0.21005	-1.621997	0.055427	-0.46577	0.04566	TRUE	1.00E+00
40395_at	-0.21002	-1.621885	0.046108	-0.42274	0.0027	TRUE	6.62E-02
762_f_at	-0.20993	-1.621549	0.046131	-0.42276	0.0029	TRUE	6.75E-02
35396_at	-0.20943	-1.619683	0.172588	-1.00568	0.58682	TRUE	1.00E+00
37342_s_at	-0.20914	-1.618602	0.057211	-0.47309	0.05481	TRUE	1.00E+00
32997_at	-0.20913	-1.618564	0.107013	-0.70284	0.28459	TRUE	1.00E+00
407_at	-0.20897	-1.617968	0.109354	-0.71349	0.29554	TRUE	1.00E+00
640_at	-0.20895	-1.617894	0.090102	-0.62464	0.20675	TRUE	1.00E+00
31771_at	-0.20875	-1.617149	0.112532	-0.72793	0.31042	TRUE	1.00E+00
34752_at	-0.20843	-1.615958	0.024079	-0.31952	-0.09734	FALSE	6.16E-14
40663_at	-0.20832	-1.615548	0.081857	-0.58598	0.16933	TRUE	1.00E+00
1411_at	-0.20823	-1.615214	0.027983	-0.33733	-0.07913	FALSE	1.26E-09
35919_at	-0.20813	-1.614842	0.071809	-0.53943	0.12317	TRUE	1.00E+00
714_at	-0.20811	-1.614768	0.064177	-0.50419	0.08798	TRUE	1.00E+00
32154_at	-0.208	-1.614359	0.155768	-0.92665	0.51065	TRUE	1.00E+00
38307_at	-0.20782	-1.61369	0.047836	-0.42851	0.01288	TRUE	1.76E-01
1242_at	-0.20778	-1.613541	0.027114	-0.33288	-0.08269	FALSE	2.28E-10
32891_at	-0.20775	-1.61343	0.034106	-0.3651	-0.05039	FALSE	1.42E-05
37022_at	-0.20773	-1.613355	0.029499	-0.34383	-0.07164	FALSE	2.39E-08
1830_s_at	-0.20766	-1.613095	0.027779	-0.33582	-0.0795	FALSE	9.71E-10
39633_at	-0.20764	-1.613021	0.083301	-0.59196	0.17667	TRUE	1.00E+00
1783_at	-0.20754	-1.61265	0.096329	-0.65197	0.23688	TRUE	1.00E+00
35647_at	-0.20753	-1.612612	0.125141	-0.78488	0.36982	TRUE	1.00E+00
157_at	-0.20751	-1.612538	0.046858	-0.42369	0.00867	TRUE	1.20E-01
34663_at	-0.2075	-1.612501	0.03926	-0.38863	-0.02637	FALSE	1.59E-03
33800_at	-0.2074	-1.61213	0.03059	-0.34853	-0.06627	FALSE	1.52E-07
41056_at	-0.20734	-1.611907	0.036731	-0.3768	-0.03788	FALSE	2.09E-04
36911_at	-0.20716	-1.611239	0.054302	-0.45768	0.04337	TRUE	1.00E+00
41114_at	-0.20695	-1.61046	0.118753	-0.75483	0.34093	TRUE	1.00E+00
38230_at	-0.20688	-1.610201	0.053525	-0.45382	0.04006	TRUE	1.00E+00
2059_s_at	-0.20688	-1.610201	0.08132	-0.58205	0.1683	TRUE	1.00E+00
36390_at	-0.2067	-1.609533	0.056619	-0.46791	0.05452	TRUE	1.00E+00
36329_at	-0.20653	-1.608904	0.131686	-0.81408	0.40101	TRUE	1.00E+00
33061_at	-0.20619	-1.607644	0.080874	-0.57931	0.16693	TRUE	1.00E+00
39558_s_at	-0.20614	-1.607459	0.083916	-0.59329	0.18101	TRUE	1.00E+00
37173_at	-0.20601	-1.606978	0.168269	-0.98233	0.57031	TRUE	1.00E+00
39622_at	-0.20584	-1.606349	0.063162	-0.49724	0.08557	TRUE	1.00E+00
40529_at	-0.20558	-1.605388	0.067909	-0.51888	0.10772	TRUE	1.00E+00
33559_at	-0.20542	-1.604797	0.03019	-0.3447	-0.06613	FALSE	1.28E-07
36222_at	-0.2051	-1.603615	0.042406	-0.40074	-0.00946	FALSE	1.67E-02
33254_at	-0.20506	-1.603467	0.050508	-0.43808	0.02796	TRUE	6.20E-01
33210_at	-0.20497	-1.603135	0.111534	-0.71955	0.3096	TRUE	1.00E+00
1756_f_at	-0.20495	-1.603061	0.116356	-0.74177	0.33187	TRUE	1.00E+00
35942_at	-0.20463	-1.60188	0.085282	-0.59809	0.18882	TRUE	1.00E+00
35575_f_at	-0.20459	-1.601733	0.068191	-0.5192	0.11001	TRUE	1.00E+00
34421_g_at	-0.20441	-1.601069	0.03069	-0.346	-0.06282	FALSE	3.45E-07

313_at	-0.2044	-1.601032	0.114685	-0.73351	0.32471	TRUE	1.00E+00
39994_at	-0.20439	-1.600995	0.066771	-0.51244	0.10366	TRUE	1.00E+00
31985_at	-0.20434	-1.600811	0.112453	-0.72315	0.31447	TRUE	1.00E+00
37859_r_at	-0.20414	-1.600074	0.037573	-0.37749	-0.03079	FALSE	6.99E-04
36536_at	-0.20405	-1.599742	0.024884	-0.31886	-0.08924	FALSE	3.04E-12
37164_at	-0.20389	-1.599153	0.087559	-0.60785	0.20007	TRUE	1.00E+00
34794_r_at	-0.20371	-1.59849	0.030124	-0.34269	-0.06473	FALSE	1.71E-07
35721_at	-0.20368	-1.59838	0.099924	-0.66469	0.25733	TRUE	1.00E+00
1197_at	-0.20337	-1.597239	0.09963	-0.66302	0.25628	TRUE	1.00E+00
38467_at	-0.20328	-1.596908	0.050594	-0.4367	0.03013	TRUE	7.41E-01
39255_at	-0.20322	-1.596688	0.096463	-0.64826	0.24182	TRUE	1.00E+00
37851_at	-0.20317	-1.596504	0.080432	-0.57425	0.16791	TRUE	1.00E+00
733_at	-0.20305	-1.596063	0.090033	-0.61842	0.21233	TRUE	1.00E+00
40201_at	-0.20303	-1.595989	0.064305	-0.4997	0.09365	TRUE	1.00E+00
39822_s_at	-0.20284	-1.595291	0.037051	-0.37378	-0.0319	FALSE	5.53E-04
40702_at	-0.20284	-1.595291	0.102184	-0.67428	0.26859	TRUE	1.00E+00
35839_at	-0.20278	-1.595071	0.0613	-0.48559	0.08004	TRUE	1.00E+00
40226_at	-0.20277	-1.595034	0.060938	-0.48392	0.07837	TRUE	1.00E+00
1441_s_at	-0.20267	-1.594667	0.029326	-0.33797	-0.06737	FALSE	6.08E-08
2026_at	-0.20261	-1.594447	0.059261	-0.47601	0.0708	TRUE	1.00E+00
39048_at	-0.2026	-1.59441	0.078652	-0.56547	0.16027	TRUE	1.00E+00
41700_at	-0.20256	-1.594263	0.044052	-0.40579	0.00068	TRUE	5.38E-02
39646_at	-0.20255	-1.594226	0.100605	-0.6667	0.2616	TRUE	1.00E+00
691_g_at	-0.2025	-1.594043	0.023825	-0.31241	-0.09258	FALSE	2.41E-13
39698_at	-0.20246	-1.593896	0.031047	-0.3457	-0.05922	FALSE	8.82E-07
39732_at	-0.20244	-1.593823	0.076735	-0.55646	0.15159	TRUE	1.00E+00
37741_at	-0.20223	-1.593052	0.043038	-0.40079	-0.00367	FALSE	3.30E-02
113_i_at	-0.20216	-1.592795	0.110872	-0.71368	0.30935	TRUE	1.00E+00
40014_at	-0.20189	-1.591805	0.063287	-0.49387	0.09009	TRUE	1.00E+00
38021_at	-0.20186	-1.591696	0.030453	-0.34236	-0.06136	FALSE	4.28E-07
38374_at	-0.20166	-1.590963	0.038884	-0.38105	-0.02227	FALSE	2.71E-03
35017_f_at	-0.20134	-1.589791	0.04982	-0.43118	0.02851	TRUE	6.71E-01
34163_g_at	-0.20125	-1.589461	0.046407	-0.41536	0.01285	TRUE	1.83E-01
31845_at	-0.20113	-1.589022	0.026681	-0.32423	-0.07804	FALSE	6.01E-10
34317_g_at	-0.20109	-1.588876	0.048347	-0.42414	0.02197	TRUE	4.03E-01
31319_at	-0.20094	-1.588327	0.076116	-0.55211	0.15022	TRUE	1.00E+00
40078_at	-0.20093	-1.588291	0.032804	-0.35228	-0.04959	FALSE	1.14E-05
1790_s_at	-0.20087	-1.588071	0.057742	-0.46727	0.06552	TRUE	1.00E+00
35855_s_at	-0.2008	-1.587815	0.053347	-0.44693	0.04532	TRUE	1.00E+00
41631_f_at	-0.20055	-1.586902	0.066707	-0.50831	0.10721	TRUE	1.00E+00
37477_at	-0.20048	-1.586646	0.075102	-0.54697	0.14601	TRUE	1.00E+00
36061_at	-0.20047	-1.586609	0.04347	-0.40102	0.00009	TRUE	5.04E-02
37290_at	-0.20042	-1.586427	0.071265	-0.52921	0.12837	TRUE	1.00E+00
36767_at	-0.20029	-1.585952	0.030948	-0.34308	-0.05751	FALSE	1.22E-06
32292_at	-0.20028	-1.585915	0.045515	-0.41027	0.00971	TRUE	1.36E-01
1107_s_at	-0.20027	-1.585879	0.019093	-0.28836	-0.11219	FALSE	1.22E-21
31332_at	-0.20019	-1.585587	0.135863	-0.82701	0.42662	TRUE	1.00E+00
41164_at	-0.20012	-1.585331	0.06975	-0.52192	0.12167	TRUE	1.00E+00
34698_at	-0.2	-1.584893	0.114207	-0.7269	0.32691	TRUE	1.00E+00
41486_at	-0.19995	-1.584711	0.066581	-0.50713	0.10722	TRUE	1.00E+00
33552_at	-0.19989	-1.584492	0.117604	-0.74247	0.34269	TRUE	1.00E+00

32452_at	-0.19979	-1.584127	0.073117	-0.53712	0.13754	TRUE	1.00E+00
36985_at	-0.19951	-1.583106	0.050447	-0.43225	0.03324	TRUE	9.67E-01
32037_r_at	-0.19888	-1.580811	0.069211	-0.51819	0.12043	TRUE	1.00E+00
40178_at	-0.19877	-1.580411	0.099412	-0.65741	0.25988	TRUE	1.00E+00
39535_at	-0.19872	-1.580229	0.034707	-0.35885	-0.0386	FALSE	1.30E-04
39312_r_at	-0.1986	-1.579792	0.075273	-0.54588	0.14867	TRUE	1.00E+00
35533_f_at	-0.19854	-1.579574	0.066385	-0.50481	0.10773	TRUE	1.00E+00
35669_at	-0.19852	-1.579501	0.050239	-0.4303	0.03327	TRUE	9.81E-01
309_f_at	-0.19851	-1.579465	0.05869	-0.46928	0.07226	TRUE	1.00E+00
40204_at	-0.19842	-1.579138	0.068608	-0.51494	0.11811	TRUE	1.00E+00
36273_at	-0.19839	-1.579029	0.057917	-0.46559	0.06882	TRUE	1.00E+00
37589_at	-0.19835	-1.578883	0.038387	-0.37545	-0.02125	FALSE	3.00E-03
40392_at	-0.19831	-1.578738	0.076163	-0.54969	0.15307	TRUE	1.00E+00
41511_at	-0.19831	-1.578738	0.031315	-0.34278	-0.05383	FALSE	3.04E-06
37529_at	-0.19828	-1.578629	0.03174	-0.34472	-0.05185	FALSE	5.28E-06
32492_g_at	-0.19828	-1.578629	0.094727	-0.63531	0.23874	TRUE	1.00E+00
36665_at	-0.19821	-1.578374	0.063648	-0.49186	0.09544	TRUE	1.00E+00
31735_at	-0.19818	-1.578265	0.097982	-0.65023	0.25387	TRUE	1.00E+00
33441_at	-0.1979	-1.577248	0.02772	-0.32579	-0.07002	FALSE	1.18E-08
32199_at	-0.19764	-1.576304	0.141975	-0.85265	0.45738	TRUE	1.00E+00
33404_at	-0.19746	-1.575651	0.037466	-0.37032	-0.02461	FALSE	1.72E-03
37236_at	-0.19744	-1.575578	0.132536	-0.8089	0.41403	TRUE	1.00E+00
31620_at	-0.19743	-1.575542	0.068824	-0.51495	0.1201	TRUE	1.00E+00
712_s_at	-0.19731	-1.575107	0.040713	-0.38515	-0.00948	FALSE	1.59E-02
35617_at	-0.19719	-1.574672	0.028639	-0.32932	-0.06506	FALSE	7.28E-08
38166_r_at	-0.1968	-1.573258	0.03988	-0.38079	-0.01281	FALSE	1.01E-02
37827_r_at	-0.19676	-1.573113	0.060677	-0.4767	0.08318	TRUE	1.00E+00
33298_at	-0.19665	-1.572715	0.112752	-0.71684	0.32355	TRUE	1.00E+00
36106_at	-0.19655	-1.572353	0.040791	-0.38474	-0.00836	FALSE	1.83E-02
32224_at	-0.19645	-1.571991	0.024338	-0.30874	-0.08416	FALSE	8.76E-12
34376_at	-0.19638	-1.571737	0.025554	-0.31428	-0.07848	FALSE	1.93E-10
38010_at	-0.19638	-1.571737	0.092662	-0.62388	0.23113	TRUE	1.00E+00
39192_at	-0.19636	-1.571665	0.063951	-0.49141	0.09868	TRUE	1.00E+00
32303_at	-0.19633	-1.571557	0.082606	-0.57744	0.18478	TRUE	1.00E+00
41163_at	-0.19624	-1.571231	0.023715	-0.30565	-0.08683	FALSE	1.62E-12
36999_at	-0.19623	-1.571195	0.056427	-0.45656	0.0641	TRUE	1.00E+00
38007_at	-0.1962	-1.571086	0.031816	-0.34298	-0.04942	FALSE	8.80E-06
792_s_at	-0.1961	-1.570724	0.070602	-0.52183	0.12963	TRUE	1.00E+00
35392_g_at	-0.19605	-1.570544	0.01946	-0.28583	-0.10626	FALSE	9.07E-20
1735_g_at	-0.196	-1.570363	0.06331	-0.48809	0.09609	TRUE	1.00E+00
761_g_at	-0.19599	-1.570327	0.029051	-0.33002	-0.06196	FALSE	1.91E-07
38917_at	-0.19591	-1.570037	0.130821	-0.79946	0.40765	TRUE	1.00E+00
38182_at	-0.19589	-1.569965	0.074834	-0.54114	0.14937	TRUE	1.00E+00
34913_at	-0.19551	-1.568592	0.039907	-0.37963	-0.0114	FALSE	1.21E-02
773_at	-0.19551	-1.568592	0.101811	-0.66523	0.2742	TRUE	1.00E+00
33084_at	-0.19544	-1.568339	0.069555	-0.51634	0.12546	TRUE	1.00E+00
39332_at	-0.19543	-1.568303	0.034979	-0.35681	-0.03406	FALSE	2.91E-04
35509_at	-0.19539	-1.568159	0.052847	-0.43921	0.04842	TRUE	1.00E+00
38397_at	-0.19522	-1.567545	0.015346	-0.26602	-0.12442	FALSE	5.69E-33
35506_s_at	-0.19504	-1.566895	0.172657	-0.9916	0.60153	TRUE	1.00E+00
32140_at	-0.19466	-1.565525	0.106577	-0.68637	0.29704	TRUE	1.00E+00

40645_at	-0.19461	-1.565345	0.019117	-0.28281	-0.10641	FALSE	3.08E-20
38996_at	-0.19452	-1.56502	0.019299	-0.28356	-0.10549	FALSE	8.61E-20
38463_s_at	-0.19442	-1.56466	0.028271	-0.32485	-0.06399	FALSE	7.71E-08
33965_at	-0.19431	-1.564264	0.087073	-0.59603	0.20741	TRUE	1.00E+00
40306_at	-0.19416	-1.563724	0.120438	-0.74981	0.36149	TRUE	1.00E+00
31414_at	-0.19393	-1.562896	0.018982	-0.28151	-0.10636	FALSE	2.11E-20
35869_at	-0.19384	-1.562572	0.100859	-0.65917	0.27148	TRUE	1.00E+00
1867_at	-0.19357	-1.561601	0.048872	-0.41904	0.0319	TRUE	9.43E-01
31554_at	-0.19352	-1.561421	0.021318	-0.29187	-0.09517	FALSE	1.40E-15
31458_at	-0.19327	-1.560522	0.027928	-0.32211	-0.06442	FALSE	5.70E-08
39103_s_at	-0.19324	-1.560415	0.024116	-0.3045	-0.08198	FALSE	1.41E-11
40762_g_at	-0.19315	-1.560091	0.066341	-0.49922	0.11292	TRUE	1.00E+00
31438_s_at	-0.19305	-1.559732	0.058161	-0.46138	0.07528	TRUE	1.00E+00
38331_at	-0.193	-1.559553	0.014378	-0.25934	-0.12667	FALSE	5.59E-37
38259_at	-0.19294	-1.559337	0.088899	-0.60309	0.2172	TRUE	1.00E+00
36397_at	-0.1928	-1.558834	0.117124	-0.73316	0.34756	TRUE	1.00E+00
34027_f_at	-0.19272	-1.558547	0.074757	-0.53762	0.15217	TRUE	1.00E+00
33173_g_at	-0.19266	-1.558332	0.041089	-0.38223	-0.0031	FALSE	3.47E-02
35191_at	-0.19265	-1.558296	0.025591	-0.31071	-0.07458	FALSE	6.50E-10
693_g_at	-0.19256	-1.557973	0.040665	-0.38017	-0.00494	FALSE	2.76E-02
37771_at	-0.1923	-1.557041	0.050149	-0.42366	0.03907	TRUE	1.00E+00
41848_f_at	-0.19208	-1.556252	0.056942	-0.45478	0.07063	TRUE	1.00E+00
34011_at	-0.19201	-1.556001	0.054967	-0.44561	0.06158	TRUE	1.00E+00
38382_at	-0.192	-1.555966	0.053003	-0.43654	0.05253	TRUE	1.00E+00
1538_s_at	-0.19199	-1.55593	0.094723	-0.629	0.24503	TRUE	1.00E+00
33630_s_at	-0.19195	-1.555787	0.073956	-0.53315	0.14925	TRUE	1.00E+00
33330_at	-0.19175	-1.55507	0.038565	-0.36967	-0.01382	FALSE	8.36E-03
34373_at	-0.19167	-1.554784	0.04347	-0.39222	0.00888	TRUE	1.31E-01
31956_f_at	-0.19157	-1.554426	0.051697	-0.43008	0.04694	TRUE	1.00E+00
35250_at	-0.19156	-1.55439	0.060463	-0.47051	0.08739	TRUE	1.00E+00
754_s_at	-0.19148	-1.554104	0.064856	-0.4907	0.10774	TRUE	1.00E+00
39932_at	-0.19128	-1.553388	0.023506	-0.29973	-0.08283	FALSE	5.10E-12
862_at	-0.19117	-1.552995	0.051547	-0.42899	0.04664	TRUE	1.00E+00
33050_at	-0.19104	-1.55253	0.097558	-0.64114	0.25905	TRUE	1.00E+00
34535_at	-0.19094	-1.552173	0.125881	-0.77171	0.38982	TRUE	1.00E+00
36468_at	-0.19067	-1.551208	0.106227	-0.68076	0.29941	TRUE	1.00E+00
34795_at	-0.19041	-1.550279	0.044706	-0.39666	0.01584	TRUE	2.59E-01
40582_at	-0.19034	-1.55003	0.065084	-0.4906	0.10993	TRUE	1.00E+00
37869_at	-0.19002	-1.548888	0.014773	-0.25818	-0.12186	FALSE	9.21E-34
33968_at	-0.18992	-1.548531	0.083601	-0.57562	0.19578	TRUE	1.00E+00
31441_at	-0.18921	-1.546002	0.093726	-0.62162	0.24321	TRUE	1.00E+00
32737_at	-0.18906	-1.545468	0.089842	-0.60355	0.22543	TRUE	1.00E+00
31946_s_at	-0.18904	-1.545397	0.087596	-0.59317	0.2151	TRUE	1.00E+00
34818_at	-0.18903	-1.545361	0.037423	-0.36168	-0.01637	FALSE	5.55E-03
39827_at	-0.18897	-1.545148	0.040693	-0.37671	-0.00123	FALSE	4.32E-02
32716_at	-0.18889	-1.544863	0.024174	-0.30042	-0.07737	FALSE	6.99E-11
41050_at	-0.18875	-1.544365	0.03033	-0.32868	-0.04882	FALSE	6.15E-06
160039_at	-0.18875	-1.544365	0.065591	-0.49135	0.11386	TRUE	1.00E+00
686_s_at	-0.1887	-1.544187	0.044319	-0.39317	0.01577	TRUE	2.61E-01
40583_at	-0.18869	-1.544152	0.031326	-0.33321	-0.04416	FALSE	2.16E-05
41345_at	-0.18864	-1.543974	0.092399	-0.61493	0.23765	TRUE	1.00E+00

35101_at	-0.18838	-1.54305	0.054038	-0.43769	0.06093	TRUE	1.00E+00
40781_at	-0.18827	-1.542659	0.033632	-0.34344	-0.03311	FALSE	2.74E-04
41334_r_at	-0.18827	-1.542659	0.078691	-0.55132	0.17478	TRUE	1.00E+00
41439_at	-0.18822	-1.542482	0.020866	-0.28448	-0.09195	FALSE	2.37E-15
31409_at	-0.18809	-1.54202	0.116432	-0.72526	0.34908	TRUE	1.00E+00
36929_at	-0.18794	-1.541487	0.092122	-0.61295	0.23708	TRUE	1.00E+00
38873_at	-0.18787	-1.541239	0.096024	-0.63088	0.25514	TRUE	1.00E+00
34092_at	-0.18786	-1.541204	0.09137	-0.6094	0.23368	TRUE	1.00E+00
35087_at	-0.1878	-1.540991	0.078934	-0.55197	0.17637	TRUE	1.00E+00
34475_at	-0.18762	-1.540352	0.058821	-0.45899	0.08375	TRUE	1.00E+00
32422_at	-0.18741	-1.539607	0.038002	-0.36274	-0.01209	FALSE	1.03E-02
34918_at	-0.18741	-1.539607	0.044844	-0.3943	0.01948	TRUE	3.69E-01
36809_at	-0.18737	-1.539466	0.159415	-0.92285	0.5481	TRUE	1.00E+00
37419_g_at	-0.1872	-1.538863	0.04779	-0.40768	0.03329	TRUE	1.00E+00
40323_at	-0.18713	-1.538615	0.128354	-0.7793	0.40504	TRUE	1.00E+00
38494_at	-0.18709	-1.538473	0.125034	-0.76394	0.38976	TRUE	1.00E+00
32749_s_at	-0.18706	-1.538367	0.027706	-0.31488	-0.05924	FALSE	1.85E-07
430_at	-0.18704	-1.538296	0.038821	-0.36614	-0.00793	FALSE	1.83E-02
41747_s_at	-0.18693	-1.537907	0.037107	-0.35812	-0.01573	FALSE	5.95E-03
36239_at	-0.18687	-1.537694	0.095642	-0.62812	0.25439	TRUE	1.00E+00
33761_s_at	-0.18675	-1.537269	0.019382	-0.27617	-0.09733	FALSE	7.15E-18
35976_at	-0.18662	-1.536809	0.065467	-0.48866	0.11542	TRUE	1.00E+00
37714_at	-0.18619	-1.535289	0.052909	-0.43029	0.05791	TRUE	1.00E+00
1077_at	-0.18619	-1.535289	0.119402	-0.73706	0.36468	TRUE	1.00E+00
36789_f_at	-0.18615	-1.535147	0.096043	-0.62925	0.25696	TRUE	1.00E+00
40511_at	-0.18611	-1.535006	0.074966	-0.53197	0.15975	TRUE	1.00E+00
864_at	-0.1858	-1.53391	0.08676	-0.58607	0.21448	TRUE	1.00E+00
1624_at	-0.18578	-1.53384	0.016872	-0.26362	-0.10794	FALSE	4.28E-24
34244_r_at	-0.18575	-1.533734	0.151073	-0.88274	0.51124	TRUE	1.00E+00
34924_at	-0.18564	-1.533345	0.066984	-0.49468	0.12339	TRUE	1.00E+00
37053_at	-0.18555	-1.533028	0.040136	-0.37072	-0.00038	FALSE	4.77E-02
37037_at	-0.18526	-1.532004	0.032534	-0.33536	-0.03516	FALSE	1.56E-04
35204_at	-0.1849	-1.530735	0.030091	-0.32372	-0.04607	FALSE	1.01E-05
39067_at	-0.1848	-1.530383	0.02639	-0.30655	-0.06305	FALSE	3.17E-08
2055_s_at	-0.18477	-1.530277	0.106076	-0.67416	0.30462	TRUE	1.00E+00
41200_at	-0.18441	-1.529009	0.018227	-0.2685	-0.10032	FALSE	5.81E-20
40989_at	-0.18436	-1.528833	0.042368	-0.37983	0.01111	TRUE	1.71E-01
36555_at	-0.18418	-1.528199	0.073105	-0.52146	0.15309	TRUE	1.00E+00
35592_at	-0.18418	-1.528199	0.078069	-0.54435	0.176	TRUE	1.00E+00
31980_at	-0.18409	-1.527883	0.035085	-0.34596	-0.02222	FALSE	1.95E-03
32642_at	-0.18402	-1.527636	0.120597	-0.74041	0.37236	TRUE	1.00E+00
32019_at	-0.18388	-1.527144	0.050992	-0.41913	0.05138	TRUE	1.00E+00
37643_at	-0.1837	-1.526511	0.028449	-0.31495	-0.05245	FALSE	1.35E-06
33780_at	-0.18359	-1.526125	0.126252	-0.76607	0.39889	TRUE	1.00E+00
39352_at	-0.18359	-1.526125	0.055565	-0.43994	0.07277	TRUE	1.00E+00
33787_at	-0.18358	-1.526089	0.037362	-0.35596	-0.01121	FALSE	1.13E-02
39480_s_at	-0.18342	-1.525527	0.070637	-0.50931	0.14247	TRUE	1.00E+00
39891_at	-0.18337	-1.525352	0.082909	-0.56588	0.19913	TRUE	1.00E+00
36181_at	-0.18333	-1.525211	0.02778	-0.31149	-0.05516	FALSE	5.22E-07
33097_at	-0.18327	-1.525001	0.024118	-0.29454	-0.07199	FALSE	3.78E-10
37366_at	-0.18326	-1.524965	0.038899	-0.36272	-0.0038	FALSE	3.11E-02

39253_s_at	-0.18303	-1.524158	0.029074	-0.31717	-0.0489	FALSE	3.87E-06
36289_f_at	-0.18283	-1.523456	0.021117	-0.28025	-0.0854	FALSE	6.06E-14
32771_at	-0.18279	-1.523316	0.05558	-0.43921	0.07363	TRUE	1.00E+00
39530_at	-0.18268	-1.52293	0.039979	-0.36713	0.00177	TRUE	6.18E-02
40450_at	-0.18242	-1.522019	0.101958	-0.65282	0.28797	TRUE	1.00E+00
643_at	-0.18238	-1.521879	0.06153	-0.46625	0.1015	TRUE	1.00E+00
33509_at	-0.18232	-1.521668	0.089739	-0.59634	0.2317	TRUE	1.00E+00
32532_at	-0.1822	-1.521248	0.071493	-0.51204	0.14763	TRUE	1.00E+00
39840_at	-0.18208	-1.520828	0.032481	-0.33194	-0.03223	FALSE	2.62E-04
836_at	-0.18202	-1.520618	0.141284	-0.83384	0.46981	TRUE	1.00E+00
1489_s_at	-0.18187	-1.520092	0.016205	-0.25663	-0.1071	FALSE	3.97E-25
37874_at	-0.18164	-1.519288	0.062898	-0.47183	0.10854	TRUE	1.00E+00
32208_at	-0.18139	-1.518413	0.039706	-0.36457	0.0018	TRUE	6.21E-02
37572_at	-0.18122	-1.517819	0.046575	-0.3961	0.03366	TRUE	1.00E+00
37452_at	-0.18093	-1.516806	0.075719	-0.53027	0.1684	TRUE	1.00E+00
40312_at	-0.18085	-1.516526	0.154641	-0.8943	0.5326	TRUE	1.00E+00
1774_at	-0.1808	-1.516352	0.04596	-0.39284	0.03124	TRUE	1.00E+00
40536_f_at	-0.18056	-1.515514	0.035026	-0.34216	-0.01897	FALSE	3.20E-03
1662_r_at	-0.18052	-1.515375	0.095996	-0.62341	0.26237	TRUE	1.00E+00
36704_at	-0.18046	-1.515165	0.11515	-0.71171	0.3508	TRUE	1.00E+00
32884_at	-0.18027	-1.514503	0.075801	-0.52998	0.16944	TRUE	1.00E+00
978_at	-0.18003	-1.513666	0.047622	-0.39974	0.03967	TRUE	1.00E+00
33612_at	-0.17992	-1.513282	0.140926	-0.83009	0.47026	TRUE	1.00E+00
40304_at	-0.17992	-1.513282	0.068069	-0.49396	0.13413	TRUE	1.00E+00
33594_at	-0.17992	-1.513282	0.051576	-0.41786	0.05803	TRUE	1.00E+00
34565_at	-0.17977	-1.51276	0.096214	-0.62366	0.26412	TRUE	1.00E+00
34534_at	-0.17976	-1.512725	0.064401	-0.47688	0.11736	TRUE	1.00E+00
41805_g_at	-0.17967	-1.512412	0.086907	-0.58062	0.22129	TRUE	1.00E+00
33465_at	-0.17957	-1.512063	0.040923	-0.36837	0.00924	TRUE	1.45E-01
38608_at	-0.17951	-1.511855	0.059881	-0.45578	0.09676	TRUE	1.00E+00
37687_i_at	-0.1793	-1.511124	0.075196	-0.52623	0.16762	TRUE	1.00E+00
32410_at	-0.17905	-1.510254	0.058362	-0.44831	0.09021	TRUE	1.00E+00
35425_at	-0.17903	-1.510184	0.065014	-0.47898	0.12092	TRUE	1.00E+00
2021_s_at	-0.17898	-1.510011	0.04685	-0.39513	0.03716	TRUE	1.00E+00
33470_at	-0.17898	-1.510011	0.029021	-0.31287	-0.04509	FALSE	8.77E-06
39413_at	-0.1788	-1.509385	0.128566	-0.77195	0.41435	TRUE	1.00E+00
39435_at	-0.17864	-1.508829	0.034238	-0.33661	-0.02068	FALSE	2.29E-03
31527_at	-0.17845	-1.508169	0.056879	-0.44087	0.08397	TRUE	1.00E+00
40703_at	-0.17845	-1.508169	0.028923	-0.31189	-0.04501	FALSE	8.64E-06
37397_at	-0.17842	-1.508065	0.050066	-0.4094	0.05257	TRUE	1.00E+00
38918_at	-0.17835	-1.507822	0.026483	-0.30054	-0.05617	FALSE	2.07E-07
34077_at	-0.17822	-1.50737	0.051394	-0.41533	0.0589	TRUE	1.00E+00
486_at	-0.1782	-1.507301	0.097525	-0.62814	0.27174	TRUE	1.00E+00
38088_r_at	-0.17805	-1.506781	0.080334	-0.54868	0.19257	TRUE	1.00E+00
37439_at	-0.17804	-1.506746	0.042673	-0.37492	0.01884	TRUE	3.81E-01
842_at	-0.17803	-1.506711	0.021291	-0.27626	-0.07981	FALSE	7.79E-13
39410_at	-0.17799	-1.506572	0.03403	-0.335	-0.02099	FALSE	2.13E-03
31674_s_at	-0.17769	-1.505532	0.083488	-0.56287	0.20749	TRUE	1.00E+00
1407_g_at	-0.17768	-1.505497	0.050207	-0.40932	0.05395	TRUE	1.00E+00
34683_at	-0.17768	-1.505497	0.131479	-0.78427	0.42891	TRUE	1.00E+00
34914_at	-0.17756	-1.505081	0.09732	-0.62655	0.27144	TRUE	1.00E+00

40996_s_at	-0.17755	-1.505047	0.134719	-0.79909	0.44399	TRUE	1.00E+00
33674_at	-0.17749	-1.504839	0.047972	-0.39881	0.04384	TRUE	1.00E+00
40170_at	-0.17748	-1.504804	0.072331	-0.51119	0.15622	TRUE	1.00E+00
31623_f_at	-0.17743	-1.504631	0.105385	-0.66363	0.30878	TRUE	1.00E+00
38546_at	-0.17722	-1.503904	0.043402	-0.37746	0.02302	TRUE	5.61E-01
36838_at	-0.17714	-1.503627	0.097791	-0.6283	0.27403	TRUE	1.00E+00
39513_r_at	-0.17712	-1.503557	0.078275	-0.53825	0.184	TRUE	1.00E+00
38215_at	-0.17709	-1.503453	0.06002	-0.454	0.09982	TRUE	1.00E+00
35475_at	-0.17701	-1.503177	0.095063	-0.61559	0.26158	TRUE	1.00E+00
32529_at	-0.17657	-1.501654	0.048807	-0.40174	0.04861	TRUE	1.00E+00
38698_at	-0.1764	-1.501067	0.021254	-0.27445	-0.07834	FALSE	1.32E-12
32890_at	-0.17623	-1.500479	0.036475	-0.34451	-0.00795	FALSE	1.71E-02
37287_at	-0.17617	-1.500272	0.067912	-0.48948	0.13715	TRUE	1.00E+00
40687_at	-0.176	-1.499685	0.079225	-0.54151	0.18951	TRUE	1.00E+00
41813_at	-0.17599	-1.49965	0.086575	-0.57542	0.22343	TRUE	1.00E+00
32926_at	-0.17593	-1.499443	0.048411	-0.39928	0.04742	TRUE	1.00E+00
805_at	-0.17582	-1.499063	0.048426	-0.39924	0.0476	TRUE	1.00E+00
38559_at	-0.17579	-1.49896	0.033899	-0.33218	-0.01939	FALSE	2.72E-03
233_s_at	-0.17578	-1.498925	0.111844	-0.69178	0.34022	TRUE	1.00E+00
35525_at	-0.17558	-1.498235	0.07832	-0.53692	0.18576	TRUE	1.00E+00
32515_s_at	-0.17555	-1.498132	0.028059	-0.305	-0.04609	FALSE	4.98E-06
36238_at	-0.17541	-1.497649	0.060844	-0.45612	0.1053	TRUE	1.00E+00
31622_f_at	-0.1754	-1.497614	0.040518	-0.36233	0.01153	TRUE	1.89E-01
33648_at	-0.17537	-1.497511	0.036681	-0.3446	-0.00614	FALSE	2.20E-02
affx-humisgf3a/m	-0.17531	-1.497304	0.020453	-0.26967	-0.08095	FALSE	1.29E-13
1576_g_at	-0.1753	-1.49727	0.048324	-0.39825	0.04765	TRUE	1.00E+00
2072_at	-0.17527	-1.497166	0.088386	-0.58305	0.2325	TRUE	1.00E+00
40693_at	-0.17526	-1.497132	0.044975	-0.38276	0.03223	TRUE	1.00E+00
41624_r_at	-0.17514	-1.496718	0.04481	-0.38187	0.03159	TRUE	1.00E+00
41447_at	-0.1751	-1.49658	0.035801	-0.34027	-0.00993	FALSE	1.27E-02
34941_at	-0.17509	-1.496546	0.121161	-0.73408	0.3839	TRUE	1.00E+00
35175_f_at	-0.17505	-1.496408	0.020204	-0.26826	-0.08184	FALSE	5.74E-14
37330_at	-0.17492	-1.49596	0.026952	-0.29927	-0.05058	FALSE	1.08E-06
32704_at	-0.17481	-1.495581	0.038673	-0.35323	0.00361	TRUE	7.80E-02
31426_at	-0.17481	-1.495581	0.036517	-0.34328	-0.00633	FALSE	2.14E-02
1535_at	-0.17463	-1.494961	0.03439	-0.3333	-0.01597	FALSE	4.82E-03
1149_at	-0.1746	-1.494858	0.048367	-0.39775	0.04854	TRUE	1.00E+00
37767_at	-0.17454	-1.494652	0.028794	-0.30738	-0.04169	FALSE	1.70E-05
36712_at	-0.17453	-1.494617	0.123439	-0.74403	0.39497	TRUE	1.00E+00
1282_s_at	-0.17435	-1.493998	0.078481	-0.53643	0.18773	TRUE	1.00E+00
32326_at	-0.17431	-1.49386	0.087178	-0.57651	0.22789	TRUE	1.00E+00
41688_at	-0.17424	-1.49362	0.058929	-0.44612	0.09763	TRUE	1.00E+00
39136_at	-0.17418	-1.493413	0.027798	-0.30243	-0.04593	FALSE	4.68E-06
32399_at	-0.17389	-1.492416	0.05302	-0.41851	0.07072	TRUE	1.00E+00
38521_at	-0.17374	-1.491901	0.023086	-0.28025	-0.06723	FALSE	6.62E-10
36708_at	-0.17343	-1.490836	0.099601	-0.63295	0.28609	TRUE	1.00E+00
32513_at	-0.17342	-1.490802	0.097195	-0.62184	0.275	TRUE	1.00E+00
affx-muril2_at	-0.17337	-1.49063	0.04902	-0.39953	0.05278	TRUE	1.00E+00
35896_at	-0.17329	-1.490356	0.019417	-0.26288	-0.08371	FALSE	5.64E-15
40862_i_at	-0.17317	-1.489944	0.041385	-0.36411	0.01776	TRUE	3.61E-01
39411_at	-0.1731	-1.489704	0.05255	-0.41555	0.06935	TRUE	1.00E+00

34085_at	-0.17309	-1.48967	0.060238	-0.451	0.10483	TRUE	1.00E+00
32293_at	-0.17308	-1.489635	0.124254	-0.74634	0.40018	TRUE	1.00E+00
32652_g_at	-0.17295	-1.48919	0.046385	-0.38695	0.04105	TRUE	1.00E+00
38108_at	-0.17292	-1.489087	0.043916	-0.37553	0.02969	TRUE	1.00E+00
41346_at	-0.17289	-1.488984	0.038394	-0.35003	0.00424	TRUE	8.45E-02
1620_at	-0.17266	-1.488196	0.039836	-0.35645	0.01113	TRUE	1.85E-01
31423_at	-0.1726	-1.48799	0.029288	-0.30772	-0.03748	FALSE	4.78E-05
33634_at	-0.17258	-1.487921	0.026814	-0.29629	-0.04887	FALSE	1.55E-06
40229_at	-0.1725	-1.487647	0.051211	-0.40876	0.06377	TRUE	1.00E+00
1406_at	-0.17233	-1.487065	0.085131	-0.56509	0.22043	TRUE	1.00E+00
935_at	-0.17224	-1.486757	0.025301	-0.28897	-0.05552	FALSE	1.25E-07
1628_at	-0.17217	-1.486517	0.026699	-0.29535	-0.04899	FALSE	1.43E-06
35344_at	-0.17217	-1.486517	0.041815	-0.36509	0.02075	TRUE	4.84E-01
37224_at	-0.17216	-1.486483	0.035278	-0.33492	-0.0094	FALSE	1.34E-02
34920_at	-0.17212	-1.486346	0.05202	-0.41212	0.06788	TRUE	1.00E+00
33490_at	-0.17195	-1.485765	0.12643	-0.75525	0.41134	TRUE	1.00E+00
41105_s_at	-0.17192	-1.485662	0.051609	-0.41002	0.06619	TRUE	1.00E+00
31821_at	-0.17187	-1.485491	0.071492	-0.5017	0.15797	TRUE	1.00E+00
33176_at	-0.17183	-1.485354	0.036404	-0.33979	-0.00388	FALSE	2.98E-02
1146_at	-0.17182	-1.48532	0.107091	-0.66589	0.32226	TRUE	1.00E+00
41552_g_at	-0.1718	-1.485252	0.051194	-0.40799	0.06439	TRUE	1.00E+00
39201_r_at	-0.17179	-1.485217	0.137944	-0.80821	0.46463	TRUE	1.00E+00
35952_at	-0.17151	-1.48426	0.07369	-0.51149	0.16846	TRUE	1.00E+00
103_at	-0.1713	-1.483543	0.04588	-0.38297	0.04037	TRUE	1.00E+00
39498_at	-0.17124	-1.483338	0.056911	-0.4338	0.09133	TRUE	1.00E+00
34067_at	-0.17109	-1.482825	0.020093	-0.26379	-0.07838	FALSE	2.11E-13
36950_at	-0.17085	-1.482006	0.018691	-0.25708	-0.08462	FALSE	7.81E-16
32634_s_at	-0.17082	-1.481904	0.07458	-0.5149	0.17326	TRUE	1.00E+00
33201_at	-0.17079	-1.481801	0.047293	-0.38898	0.0474	TRUE	1.00E+00
39463_at	-0.17059	-1.481119	0.116839	-0.70963	0.36846	TRUE	1.00E+00
38001_at	-0.17058	-1.481085	0.048351	-0.39365	0.05249	TRUE	1.00E+00
35015_at	-0.17055	-1.480983	0.08795	-0.57632	0.23521	TRUE	1.00E+00
34246_at	-0.17025	-1.47996	0.028748	-0.30288	-0.03762	FALSE	4.01E-05
330_s_at	-0.17012	-1.479517	0.021488	-0.26926	-0.07099	FALSE	3.06E-11
31325_at	-0.17011	-1.479483	0.034446	-0.32903	-0.01119	FALSE	9.94E-03
40897_at	-0.17007	-1.479347	0.123626	-0.74043	0.40029	TRUE	1.00E+00
35065_at	-0.17003	-1.479211	0.049465	-0.39825	0.05818	TRUE	1.00E+00
33156_at	-0.16966	-1.477951	0.0499	-0.39988	0.06055	TRUE	1.00E+00
40953_at	-0.16964	-1.477883	0.014845	-0.23813	-0.10115	FALSE	3.87E-26
41344_s_at	-0.16963	-1.477849	0.02918	-0.30426	-0.03501	FALSE	7.73E-05
34495_r_at	-0.16963	-1.477849	0.064399	-0.46674	0.12748	TRUE	1.00E+00
35541_r_at	-0.16958	-1.477679	0.060513	-0.44876	0.1096	TRUE	1.00E+00
36142_at	-0.16954	-1.477543	0.028818	-0.3025	-0.03659	FALSE	5.08E-05
33521_at	-0.16937	-1.476964	0.068939	-0.48742	0.14868	TRUE	1.00E+00
1068_g_at	-0.16912	-1.476114	0.062232	-0.45624	0.11799	TRUE	1.00E+00
41016_at	-0.16912	-1.476114	0.054803	-0.42195	0.08372	TRUE	1.00E+00
41524_at	-0.16909	-1.476012	0.02132	-0.26745	-0.07073	FALSE	2.74E-11
32468_f_at	-0.16898	-1.475639	0.048503	-0.39275	0.05479	TRUE	1.00E+00
39445_at	-0.16891	-1.475401	0.020316	-0.26264	-0.07518	FALSE	1.17E-12
40339_at	-0.16888	-1.475299	0.044057	-0.37214	0.03438	TRUE	1.00E+00
41736_g_at	-0.16862	-1.474416	0.123531	-0.73854	0.4013	TRUE	1.00E+00

39304_g_at	-0.16861	-1.474382	0.021511	-0.26785	-0.06937	FALSE	5.77E-11
40721_g_at	-0.16859	-1.474314	0.095661	-0.60993	0.27275	TRUE	1.00E+00
2017_s_at	-0.16847	-1.473907	0.031823	-0.31529	-0.02165	FALSE	1.51E-03
32844_at	-0.16831	-1.473364	0.024532	-0.28149	-0.05512	FALSE	8.66E-08
1844_s_at	-0.16824	-1.473126	0.022404	-0.27161	-0.06488	FALSE	7.50E-10
38288_at	-0.16774	-1.471431	0.056817	-0.42987	0.09439	TRUE	1.00E+00
39837_s_at	-0.16769	-1.471262	0.069101	-0.4865	0.15111	TRUE	1.00E+00
33686_at	-0.16766	-1.47116	0.088057	-0.57391	0.2386	TRUE	1.00E+00
404_at	-0.16759	-1.470923	0.043537	-0.36846	0.03327	TRUE	1.00E+00
32896_at	-0.16757	-1.470855	0.139472	-0.81103	0.4759	TRUE	1.00E+00
41288_at	-0.1674	-1.47028	0.021256	-0.26547	-0.06934	FALSE	4.28E-11
39107_at	-0.16739	-1.470246	0.061319	-0.45029	0.11551	TRUE	1.00E+00
1333_f_at	-0.16731	-1.469975	0.049055	-0.39363	0.05901	TRUE	1.00E+00
37017_at	-0.16731	-1.469975	0.059585	-0.44221	0.10759	TRUE	1.00E+00
37064_at	-0.16725	-1.469772	0.123528	-0.73716	0.40265	TRUE	1.00E+00
33450_at	-0.16723	-1.469704	0.048804	-0.39239	0.05793	TRUE	1.00E+00
32355_at	-0.16715	-1.469434	0.039198	-0.34799	0.01369	TRUE	2.53E-01
33370_r_at	-0.16713	-1.469366	0.068791	-0.4845	0.15025	TRUE	1.00E+00
1946_at	-0.1671	-1.469265	0.088256	-0.57428	0.24007	TRUE	1.00E+00
36598_s_at	-0.16699	-1.468892	0.035722	-0.3318	-0.00219	FALSE	3.71E-02
32008_at	-0.16687	-1.468487	0.109375	-0.67148	0.33774	TRUE	1.00E+00
31809_at	-0.1668	-1.46825	0.169191	-0.94737	0.61378	TRUE	1.00E+00
33588_at	-0.16643	-1.467	0.119583	-0.71814	0.38527	TRUE	1.00E+00
32021_at	-0.16643	-1.467	0.05094	-0.40145	0.06858	TRUE	1.00E+00
41823_at	-0.16643	-1.467	0.023888	-0.27664	-0.05622	FALSE	4.09E-08
35451_s_at	-0.16622	-1.46629	0.040904	-0.35494	0.02249	TRUE	6.10E-01
32109_at	-0.16622	-1.46629	0.037369	-0.33863	0.00618	TRUE	1.09E-01
39661_s_at	-0.16597	-1.465447	0.028557	-0.29772	-0.03422	FALSE	7.80E-05
32602_at	-0.16593	-1.465312	0.026843	-0.28977	-0.04209	FALSE	8.01E-06
39115_at	-0.1658	-1.464873	0.064672	-0.46417	0.13258	TRUE	1.00E+00
39579_at	-0.16553	-1.463963	0.071159	-0.49383	0.16276	TRUE	1.00E+00
33356_at	-0.16538	-1.463457	0.079403	-0.53171	0.20096	TRUE	1.00E+00
36299_at	-0.16534	-1.463322	0.060238	-0.44325	0.11258	TRUE	1.00E+00
31307_at	-0.16533	-1.463289	0.05909	-0.43795	0.10728	TRUE	1.00E+00
1438_at	-0.16496	-1.462043	0.060157	-0.4425	0.11258	TRUE	1.00E+00
41781_at	-0.16484	-1.461639	0.096903	-0.61191	0.28224	TRUE	1.00E+00
34984_at	-0.16477	-1.461403	0.109911	-0.67186	0.34231	TRUE	1.00E+00
1747_at	-0.16476	-1.461369	0.044122	-0.36832	0.0388	TRUE	1.00E+00
39271_at	-0.16474	-1.461302	0.108866	-0.667	0.33752	TRUE	1.00E+00
33947_at	-0.16451	-1.460528	0.041258	-0.35486	0.02583	TRUE	8.43E-01
35055_at	-0.1644	-1.460158	0.040782	-0.35255	0.02376	TRUE	7.01E-01
31938_g_at	-0.16438	-1.460091	0.11829	-0.71012	0.38136	TRUE	1.00E+00
38515_at	-0.16435	-1.45999	0.104298	-0.64554	0.31684	TRUE	1.00E+00
32514_s_at	-0.16435	-1.45999	0.074852	-0.50969	0.18099	TRUE	1.00E+00
33174_s_at	-0.16425	-1.459654	0.083805	-0.55089	0.2224	TRUE	1.00E+00
35961_at	-0.16412	-1.459217	0.090339	-0.58091	0.25266	TRUE	1.00E+00
36990_at	-0.16397	-1.458713	0.034372	-0.32255	-0.00539	FALSE	2.32E-02
39447_f_at	-0.16395	-1.458646	0.068303	-0.47907	0.15117	TRUE	1.00E+00
36505_at	-0.16394	-1.458613	0.131629	-0.77122	0.44334	TRUE	1.00E+00
587_at	-0.16383	-1.458243	0.100965	-0.62965	0.30198	TRUE	1.00E+00
743_at	-0.16366	-1.457673	0.051462	-0.40108	0.07377	TRUE	1.00E+00

37473_at	-0.16365	-1.457639	0.030007	-0.30209	-0.02521	FALSE	6.23E-04
33650_at	-0.16349	-1.457102	0.071055	-0.49131	0.16433	TRUE	1.00E+00
32020_at	-0.16338	-1.456733	0.057092	-0.42678	0.10001	TRUE	1.00E+00
39948_at	-0.16336	-1.456666	0.070331	-0.48784	0.16112	TRUE	1.00E+00
1645_at	-0.16331	-1.456498	0.066501	-0.47012	0.1435	TRUE	1.00E+00
31994_at	-0.16319	-1.456096	0.025588	-0.28125	-0.04514	FALSE	2.27E-06
40513_at	-0.16314	-1.455928	0.050464	-0.39596	0.06968	TRUE	1.00E+00
31723_at	-0.16313	-1.455895	0.06619	-0.4685	0.14225	TRUE	1.00E+00
32760_at	-0.1631	-1.455794	0.091238	-0.58403	0.25783	TRUE	1.00E+00
32371_at	-0.16287	-1.455023	0.059186	-0.43593	0.11019	TRUE	1.00E+00
37217_at	-0.1628	-1.454789	0.051905	-0.40227	0.07666	TRUE	1.00E+00
37186_s_at	-0.16278	-1.454722	0.076797	-0.51708	0.19153	TRUE	1.00E+00
2016_s_at	-0.16272	-1.454521	0.035977	-0.3287	0.00327	TRUE	7.71E-02
41134_at	-0.16245	-1.453617	0.049495	-0.3908	0.0659	TRUE	1.00E+00
38460_at	-0.16231	-1.453149	0.10705	-0.65619	0.33158	TRUE	1.00E+00
32062_at	-0.1623	-1.453115	0.065724	-0.46553	0.14092	TRUE	1.00E+00
36850_at	-0.16228	-1.453048	0.077205	-0.51847	0.19392	TRUE	1.00E+00
107_at	-0.16223	-1.452881	0.078123	-0.52266	0.19819	TRUE	1.00E+00
32491_at	-0.16222	-1.452847	0.08416	-0.5505	0.22606	TRUE	1.00E+00
32500_at	-0.16217	-1.45268	0.060776	-0.44257	0.11823	TRUE	1.00E+00
40503_at	-0.16208	-1.452379	0.031796	-0.30877	-0.01539	FALSE	4.34E-03
34557_at	-0.16198	-1.452045	0.049266	-0.38927	0.06531	TRUE	1.00E+00
34111_s_at	-0.16182	-1.45151	0.084822	-0.55315	0.22951	TRUE	1.00E+00
37552_at	-0.16181	-1.451476	0.030149	-0.30091	-0.02272	FALSE	1.01E-03
33784_at	-0.1617	-1.451109	0.021156	-0.2593	-0.06409	FALSE	2.68E-10
33816_at	-0.1617	-1.451109	0.027837	-0.29013	-0.03327	FALSE	7.95E-05
38554_at	-0.16166	-1.450975	0.036295	-0.32911	0.00579	TRUE	1.06E-01
31556_at	-0.16152	-1.450508	0.041761	-0.35419	0.03115	TRUE	1.00E+00
33041_at	-0.16146	-1.450307	0.019118	-0.24966	-0.07326	FALSE	3.82E-13
41675_at	-0.1614	-1.450107	0.039653	-0.34434	0.02154	TRUE	5.93E-01
35510_at	-0.16129	-1.44974	0.04748	-0.38035	0.05776	TRUE	1.00E+00
31586_f_at	-0.16122	-1.449506	0.09858	-0.61603	0.29359	TRUE	1.00E+00
36006_at	-0.16111	-1.449139	0.060597	-0.44068	0.11845	TRUE	1.00E+00
35048_at	-0.16105	-1.448939	0.097192	-0.60945	0.28736	TRUE	1.00E+00
35242_at	-0.16092	-1.448505	0.048524	-0.38479	0.06295	TRUE	1.00E+00
40675_r_at	-0.16086	-1.448305	0.042833	-0.35847	0.03675	TRUE	1.00E+00
210_at	-0.16081	-1.448138	0.039186	-0.3416	0.01998	TRUE	5.13E-01
40335_at	-0.16079	-1.448071	0.12148	-0.72125	0.39967	TRUE	1.00E+00
249_at	-0.16079	-1.448071	0.043888	-0.36327	0.0417	TRUE	1.00E+00
34509_at	-0.1607	-1.447771	0.121684	-0.7221	0.4007	TRUE	1.00E+00
34643_at	-0.16069	-1.447738	0.059509	-0.43524	0.11386	TRUE	1.00E+00
33574_at	-0.16065	-1.447605	0.114122	-0.68716	0.36586	TRUE	1.00E+00
1412_g_at	-0.1603	-1.446439	0.077906	-0.51973	0.19913	TRUE	1.00E+00
37176_at	-0.16028	-1.446372	0.045256	-0.36907	0.04852	TRUE	1.00E+00
40175_at	-0.16019	-1.446072	0.038	-0.3355	0.01513	TRUE	3.15E-01
32447_at	-0.16017	-1.446006	0.050437	-0.39287	0.07252	TRUE	1.00E+00
31611_s_at	-0.16013	-1.445873	0.034358	-0.31864	-0.00161	FALSE	3.98E-02
38779_r_at	-0.1601	-1.445773	0.06886	-0.47779	0.1576	TRUE	1.00E+00
37069_at	-0.16008	-1.445706	0.108767	-0.66188	0.34173	TRUE	1.00E+00
35011_at	-0.15996	-1.445307	0.105901	-0.64855	0.32862	TRUE	1.00E+00
37412_at	-0.15995	-1.445273	0.067317	-0.47052	0.15062	TRUE	1.00E+00

32156_at	-0.15994	-1.44524	0.030048	-0.29857	-0.02131	FALSE	1.29E-03
39406_at	-0.15988	-1.44504	0.035015	-0.32143	0.00166	TRUE	6.27E-02
40040_at	-0.15982	-1.444841	0.039005	-0.33978	0.02013	TRUE	5.27E-01
36568_at	-0.15979	-1.444741	0.079773	-0.52784	0.20825	TRUE	1.00E+00
34851_at	-0.15966	-1.444309	0.071199	-0.48814	0.16882	TRUE	1.00E+00
31688_at	-0.15958	-1.444043	0.044302	-0.36397	0.04481	TRUE	1.00E+00
35864_at	-0.15956	-1.443976	0.019524	-0.24964	-0.06949	FALSE	3.81E-12
33469_r_at	-0.15954	-1.44391	0.038916	-0.33908	0.02	TRUE	5.22E-01
34318_at	-0.1595	-1.443777	0.031159	-0.30326	-0.01575	FALSE	3.88E-03
34585_at	-0.15939	-1.443411	0.087605	-0.56356	0.24479	TRUE	1.00E+00
39131_at	-0.15934	-1.443245	0.018857	-0.24633	-0.07234	FALSE	3.69E-13
36082_at	-0.15931	-1.443145	0.104112	-0.63964	0.32101	TRUE	1.00E+00
32795_at	-0.15924	-1.442913	0.064915	-0.45873	0.14025	TRUE	1.00E+00
37277_at	-0.15918	-1.442713	0.023295	-0.26665	-0.05171	FALSE	1.05E-07
37957_at	-0.15917	-1.44268	0.027467	-0.28589	-0.03245	FALSE	8.62E-05
40157_s_at	-0.15912	-1.442514	0.066108	-0.46412	0.14587	TRUE	1.00E+00
37151_at	-0.1591	-1.442447	0.029885	-0.29698	-0.02122	FALSE	1.28E-03
38067_at	-0.15906	-1.442315	0.026657	-0.28204	-0.03607	FALSE	3.05E-05
38888_at	-0.15905	-1.442281	0.065336	-0.46048	0.14238	TRUE	1.00E+00
33286_at	-0.15901	-1.442149	0.063825	-0.45348	0.13545	TRUE	1.00E+00
115_at	-0.159	-1.442115	0.053514	-0.40589	0.08789	TRUE	1.00E+00
35522_at	-0.15885	-1.441617	0.046141	-0.37172	0.05403	TRUE	1.00E+00
33460_at	-0.15877	-1.441352	0.059621	-0.43383	0.1163	TRUE	1.00E+00
38740_at	-0.15875	-1.441285	0.025014	-0.27416	-0.04335	FALSE	2.78E-06
38338_at	-0.15873	-1.441219	0.015603	-0.23071	-0.08674	FALSE	3.31E-20
34965_at	-0.15857	-1.440688	0.091464	-0.58055	0.26341	TRUE	1.00E+00
33085_at	-0.15854	-1.440589	0.060222	-0.43638	0.1193	TRUE	1.00E+00
41814_at	-0.15852	-1.440522	0.039831	-0.34229	0.02524	TRUE	8.71E-01
1745_at	-0.15823	-1.439561	0.086508	-0.55734	0.24088	TRUE	1.00E+00
31989_s_at	-0.15819	-1.439428	0.044436	-0.3632	0.04682	TRUE	1.00E+00
37375_at	-0.15819	-1.439428	0.040248	-0.34388	0.0275	TRUE	1.00E+00
41528_at	-0.15816	-1.439329	0.037591	-0.33159	0.01527	TRUE	3.26E-01
35904_at	-0.15804	-1.438931	0.097024	-0.60567	0.28959	TRUE	1.00E+00
37261_g_at	-0.15804	-1.438931	0.069322	-0.47787	0.16179	TRUE	1.00E+00
39683_at	-0.15801	-1.438832	0.083343	-0.54252	0.2265	TRUE	1.00E+00
31478_at	-0.15778	-1.43807	0.024503	-0.27083	-0.04474	FALSE	1.52E-06
32490_at	-0.15767	-1.437706	0.017614	-0.23893	-0.0764	FALSE	4.44E-15
39753_at	-0.15764	-1.437606	0.031383	-0.30243	-0.01286	FALSE	6.41E-03
33695_at	-0.15759	-1.437441	0.098287	-0.61104	0.29587	TRUE	1.00E+00
41096_at	-0.15756	-1.437342	0.079402	-0.52388	0.20877	TRUE	1.00E+00
1061_at	-0.15752	-1.437209	0.047251	-0.37552	0.06047	TRUE	1.00E+00
40939_at	-0.15751	-1.437176	0.079961	-0.52641	0.2114	TRUE	1.00E+00
32313_at	-0.15748	-1.437077	0.024365	-0.2699	-0.04507	FALSE	1.29E-06
31385_at	-0.15742	-1.436878	0.036298	-0.32488	0.01005	TRUE	1.83E-01
39887_at	-0.15734	-1.436614	0.076027	-0.5081	0.19342	TRUE	1.00E+00
32007_at	-0.15724	-1.436283	0.081741	-0.53436	0.21988	TRUE	1.00E+00
34583_at	-0.15724	-1.436283	0.078374	-0.51882	0.20435	TRUE	1.00E+00
40728_at	-0.15711	-1.435853	0.072246	-0.49042	0.17621	TRUE	1.00E+00
35691_r_at	-0.15702	-1.435556	0.083811	-0.54369	0.22964	TRUE	1.00E+00
36528_at	-0.15689	-1.435126	0.041891	-0.35015	0.03638	TRUE	1.00E+00
40163_r_at	-0.15686	-1.435027	0.024734	-0.27097	-0.04274	FALSE	2.87E-06

40483_at	-0.15685	-1.434994	0.066354	-0.46298	0.14928	TRUE	1.00E+00
35108_at	-0.15679	-1.434795	0.023366	-0.26459	-0.04899	FALSE	2.45E-07
35675_at	-0.15676	-1.434696	0.01622	-0.23159	-0.08192	FALSE	5.39E-18
39508_at	-0.15674	-1.43463	0.062025	-0.4429	0.12942	TRUE	1.00E+00
35420_r_at	-0.15668	-1.434432	0.062866	-0.44672	0.13336	TRUE	1.00E+00
41392_at	-0.15666	-1.434366	0.093069	-0.58604	0.27273	TRUE	1.00E+00
37136_at	-0.1564	-1.433508	0.049488	-0.38472	0.07191	TRUE	1.00E+00
40165_at	-0.15633	-1.433277	0.022142	-0.25848	-0.05418	FALSE	2.09E-08
35559_at	-0.15632	-1.433244	0.072409	-0.49039	0.17774	TRUE	1.00E+00
32186_at	-0.15625	-1.433013	0.020815	-0.25229	-0.06022	FALSE	7.67E-10
1871_g_at	-0.1561	-1.432518	0.071239	-0.48477	0.17257	TRUE	1.00E+00
33525_at	-0.15605	-1.432353	0.091304	-0.57729	0.26519	TRUE	1.00E+00
35698_at	-0.15603	-1.432287	0.073895	-0.49695	0.18489	TRUE	1.00E+00
41284_at	-0.15601	-1.432221	0.093704	-0.58832	0.27631	TRUE	1.00E+00
32600_at	-0.15578	-1.431463	0.018188	-0.23969	-0.07187	FALSE	1.36E-13
35386_at	-0.1556	-1.430869	0.119349	-0.70623	0.39503	TRUE	1.00E+00
37737_at	-0.15544	-1.430342	0.028935	-0.28893	-0.02194	FALSE	9.84E-04
39198_s_at	-0.1553	-1.429881	0.066001	-0.4598	0.1492	TRUE	1.00E+00
32759_at	-0.15521	-1.429585	0.061219	-0.43765	0.12723	TRUE	1.00E+00
affx-muril10_at	-0.15521	-1.429585	0.103789	-0.63405	0.32363	TRUE	1.00E+00
511_s_at	-0.15519	-1.429519	0.062488	-0.44348	0.1331	TRUE	1.00E+00
1921_at	-0.15511	-1.429256	0.068723	-0.47217	0.16195	TRUE	1.00E+00
33362_at	-0.15491	-1.428598	0.033321	-0.30864	-0.00119	FALSE	4.21E-02
1276_g_at	-0.15488	-1.428499	0.027556	-0.28201	-0.02774	FALSE	2.41E-04
41061_at	-0.15486	-1.428433	0.059814	-0.43082	0.1211	TRUE	1.00E+00
1408_at	-0.15484	-1.428368	0.117549	-0.69716	0.38748	TRUE	1.00E+00
41726_at	-0.15459	-1.427546	0.022336	-0.25764	-0.05154	FALSE	5.66E-08
37114_at	-0.15458	-1.427513	0.025555	-0.27248	-0.03668	FALSE	1.84E-05
33124_at	-0.15457	-1.42748	0.045629	-0.36508	0.05594	TRUE	1.00E+00
40470_at	-0.15448	-1.427184	0.051294	-0.39112	0.08217	TRUE	1.00E+00
37958_at	-0.15439	-1.426888	0.046495	-0.3689	0.06012	TRUE	1.00E+00
35036_at	-0.15428	-1.426527	0.067512	-0.46576	0.15719	TRUE	1.00E+00
36483_at	-0.15413	-1.426034	0.102625	-0.6276	0.31933	TRUE	1.00E+00
2024_s_at	-0.15412	-1.426002	0.066308	-0.46003	0.1518	TRUE	1.00E+00
37159_at	-0.15406	-1.425805	0.020807	-0.25006	-0.05807	FALSE	1.66E-09
938_at	-0.15402	-1.425673	0.067182	-0.46397	0.15593	TRUE	1.00E+00
1567_at	-0.154	-1.425608	0.058766	-0.42513	0.11712	TRUE	1.00E+00
40243_at	-0.15385	-1.425115	0.071427	-0.48338	0.17569	TRUE	1.00E+00
33777_at	-0.15361	-1.424328	0.046034	-0.366	0.05877	TRUE	1.00E+00
35829_at	-0.15351	-1.424	0.115569	-0.6867	0.37968	TRUE	1.00E+00
40454_at	-0.15342	-1.423705	0.021061	-0.25058	-0.05625	FALSE	4.08E-09
32481_at	-0.15338	-1.423574	0.04775	-0.37367	0.06692	TRUE	1.00E+00
32474_at	-0.15329	-1.423279	0.031048	-0.29654	-0.01005	FALSE	1.00E-02
37812_at	-0.15328	-1.423246	0.03008	-0.29206	-0.0145	FALSE	4.38E-03
33623_g_at	-0.1532	-1.422984	0.066287	-0.45903	0.15262	TRUE	1.00E+00
31530_at	-0.15312	-1.422722	0.068339	-0.4684	0.16217	TRUE	1.00E+00
32666_at	-0.15306	-1.422525	0.071097	-0.48107	0.17495	TRUE	1.00E+00
39716_at	-0.15295	-1.422165	0.106494	-0.64427	0.33837	TRUE	1.00E+00
31983_at	-0.15273	-1.421445	0.091162	-0.57332	0.26785	TRUE	1.00E+00
31639_f_at	-0.1526	-1.421019	0.0747	-0.49723	0.19204	TRUE	1.00E+00
35733_at	-0.15244	-1.420496	0.029682	-0.28938	-0.01551	FALSE	3.54E-03

39315_at	-0.15241	-1.420398	0.041915	-0.34579	0.04097	TRUE	1.00E+00
550_at	-0.15237	-1.420267	0.090873	-0.57162	0.26688	TRUE	1.00E+00
32860_g_at	-0.1523	-1.420038	0.019494	-0.24223	-0.06236	FALSE	7.09E-11
32667_at	-0.15226	-1.419907	0.043507	-0.35298	0.04846	TRUE	1.00E+00
31374_at	-0.1522	-1.419711	0.051272	-0.38875	0.08434	TRUE	1.00E+00
32675_at	-0.15202	-1.419123	0.027194	-0.27748	-0.02656	FALSE	2.86E-04
1445_at	-0.15201	-1.41909	0.031639	-0.29798	-0.00604	FALSE	1.96E-02
296_at	-0.15178	-1.418339	0.012965	-0.2116	-0.09196	FALSE	1.49E-27
34524_at	-0.15178	-1.418339	0.040536	-0.3388	0.03524	TRUE	1.00E+00
36449_s_at	-0.15163	-1.417849	0.074743	-0.49647	0.1932	TRUE	1.00E+00
34738_at	-0.1515	-1.417425	0.07461	-0.49572	0.19272	TRUE	1.00E+00
40776_at	-0.15149	-1.417392	0.042876	-0.3493	0.04632	TRUE	1.00E+00
34604_at	-0.15134	-1.416903	0.029464	-0.28728	-0.0154	FALSE	3.54E-03
35262_at	-0.15124	-1.416576	0.016506	-0.2274	-0.07509	FALSE	6.38E-16
32472_at	-0.15123	-1.416544	0.076009	-0.50191	0.19944	TRUE	1.00E+00
602_s_at	-0.15107	-1.416022	0.099682	-0.61096	0.30883	TRUE	1.00E+00
35106_at	-0.151	-1.415794	0.064758	-0.44977	0.14776	TRUE	1.00E+00
37768_at	-0.15083	-1.41524	0.014929	-0.21971	-0.08196	FALSE	6.74E-20
31531_g_at	-0.15076	-1.415012	0.062179	-0.43762	0.13611	TRUE	1.00E+00
39592_r_at	-0.15072	-1.414881	0.094194	-0.58529	0.28385	TRUE	1.00E+00
41150_r_at	-0.15066	-1.414686	0.225546	-1.19123	0.88992	TRUE	1.00E+00
961_at	-0.15064	-1.414621	0.100757	-0.61549	0.31421	TRUE	1.00E+00
36751_at	-0.15046	-1.414034	0.051346	-0.38735	0.08643	TRUE	1.00E+00
32102_at	-0.15045	-1.414002	0.024141	-0.26183	-0.03907	FALSE	5.81E-06
38766_at	-0.15027	-1.413416	0.051886	-0.38965	0.08911	TRUE	1.00E+00
40039_g_at	-0.15025	-1.413351	0.03365	-0.3055	0.005	TRUE	1.01E-01
39477_s_at	-0.15012	-1.412928	0.044178	-0.35394	0.0537	TRUE	1.00E+00
33681_at	-0.15007	-1.412765	0.039919	-0.33424	0.0341	TRUE	1.00E+00
33243_at	-0.15003	-1.412635	0.040241	-0.33569	0.03563	TRUE	1.00E+00
33606_g_at	-0.15	-1.412538	0.045775	-0.36118	0.06119	TRUE	1.00E+00
36630_at	-0.14989	-1.41218	0.022322	-0.25288	-0.04691	FALSE	2.37E-07
36742_at	-0.14985	-1.41205	0.088352	-0.55747	0.25777	TRUE	1.00E+00
38444_at	-0.14963	-1.411335	0.051809	-0.38866	0.08939	TRUE	1.00E+00
437_at	-0.1495	-1.410912	0.138246	-0.78731	0.48831	TRUE	1.00E+00
36779_at	-0.1495	-1.410912	0.033937	-0.30607	0.00707	TRUE	1.33E-01
33213_g_at	-0.14924	-1.410068	0.033189	-0.30236	0.00388	TRUE	8.71E-02
31929_at	-0.14923	-1.410035	0.051112	-0.38504	0.08658	TRUE	1.00E+00
32289_at	-0.14923	-1.410035	0.098955	-0.60576	0.30731	TRUE	1.00E+00
40265_s_at	-0.14923	-1.410035	0.038162	-0.32529	0.02684	TRUE	1.00E+00
38190_r_at	-0.14909	-1.409581	0.073556	-0.48845	0.19026	TRUE	1.00E+00
37135_f_at	-0.14881	-1.408672	0.07582	-0.49861	0.20099	TRUE	1.00E+00
41446_f_at	-0.14872	-1.40838	0.066077	-0.45358	0.15613	TRUE	1.00E+00
38179_at	-0.14832	-1.407084	0.091896	-0.57229	0.27565	TRUE	1.00E+00
35944_at	-0.14832	-1.407084	0.04755	-0.36769	0.07106	TRUE	1.00E+00
38884_at	-0.14826	-1.40689	0.047584	-0.36779	0.07128	TRUE	1.00E+00
40429_r_at	-0.1482	-1.406695	0.086448	-0.54704	0.25063	TRUE	1.00E+00
37592_at	-0.14814	-1.406501	0.098021	-0.60037	0.30409	TRUE	1.00E+00
37846_at	-0.14794	-1.405853	0.048539	-0.37188	0.076	TRUE	1.00E+00
39339_at	-0.14782	-1.405465	0.014217	-0.21341	-0.08223	FALSE	3.22E-21
38243_at	-0.1478	-1.4054	0.04862	-0.37211	0.07651	TRUE	1.00E+00
33635_at	-0.14775	-1.405238	0.047221	-0.36561	0.07011	TRUE	1.00E+00

37292_at	-0.14771	-1.405109	0.030474	-0.2883	-0.00711	FALSE	1.58E-02
35361_at	-0.14764	-1.404882	0.028232	-0.27789	-0.01739	FALSE	2.14E-03
40913_at	-0.1475	-1.40443	0.048171	-0.36974	0.07474	TRUE	1.00E+00
37177_at	-0.14738	-1.404042	0.032064	-0.29531	0.00055	TRUE	5.43E-02
38561_at	-0.14732	-1.403848	0.05369	-0.39503	0.10038	TRUE	1.00E+00
33841_at	-0.14718	-1.403395	0.025318	-0.26398	-0.03037	FALSE	7.75E-05
34823_at	-0.14715	-1.403298	0.119523	-0.69857	0.40428	TRUE	1.00E+00
37310_at	-0.14702	-1.402878	0.02483	-0.26158	-0.03246	FALSE	4.04E-05
1740_g_at	-0.14699	-1.402781	0.067108	-0.4566	0.16262	TRUE	1.00E+00
36391_at	-0.14691	-1.402523	0.029575	-0.28336	-0.01047	FALSE	8.56E-03
40535_i_at	-0.14689	-1.402458	0.083614	-0.53265	0.23887	TRUE	1.00E+00
151_s_at	-0.14681	-1.4022	0.047114	-0.36418	0.07055	TRUE	1.00E+00
38788_at	-0.14679	-1.402136	0.022084	-0.24867	-0.0449	FALSE	3.78E-07
37601_at	-0.14648	-1.401135	0.09998	-0.60775	0.31478	TRUE	1.00E+00
36041_at	-0.14647	-1.401103	0.135741	-0.77273	0.47978	TRUE	1.00E+00
33352_at	-0.14647	-1.401103	0.046088	-0.3591	0.06616	TRUE	1.00E+00
35415_at	-0.14626	-1.400425	0.067333	-0.45691	0.16439	TRUE	1.00E+00
998_s_at	-0.14625	-1.400393	0.072673	-0.48153	0.18903	TRUE	1.00E+00
40936_at	-0.14624	-1.400361	0.026886	-0.27028	-0.0222	FALSE	6.75E-04
37234_at	-0.14614	-1.400039	0.02988	-0.284	-0.00829	FALSE	1.27E-02
37483_at	-0.14602	-1.399652	0.06079	-0.42648	0.13444	TRUE	1.00E+00
1218_at	-0.14598	-1.399523	0.019726	-0.23699	-0.05498	FALSE	1.71E-09
34612_at	-0.14598	-1.399523	0.069708	-0.46759	0.17562	TRUE	1.00E+00
37709_at	-0.14591	-1.399297	0.027411	-0.27237	-0.01944	FALSE	1.29E-03
32071_at	-0.14589	-1.399233	0.016467	-0.22186	-0.06992	FALSE	1.01E-14
32855_at	-0.14588	-1.399201	0.064949	-0.44553	0.15376	TRUE	1.00E+00
1831_at	-0.14582	-1.399007	0.134182	-0.76488	0.47324	TRUE	1.00E+00
166_at	-0.14582	-1.399007	0.041724	-0.33831	0.04668	TRUE	1.00E+00
34422_r_at	-0.14581	-1.398975	0.017464	-0.22638	-0.06524	FALSE	8.70E-13
414_at	-0.14576	-1.398814	0.065236	-0.44673	0.15521	TRUE	1.00E+00
1048_at	-0.14559	-1.398267	0.037563	-0.31889	0.02771	TRUE	1.00E+00
37088_at	-0.14557	-1.398202	0.075251	-0.49275	0.20161	TRUE	1.00E+00
40299_at	-0.1455	-1.397977	0.030355	-0.28554	-0.00545	FALSE	2.07E-02
898_s_at	-0.14543	-1.397752	0.019072	-0.23342	-0.05744	FALSE	3.08E-10
39577_at	-0.14539	-1.397623	0.09032	-0.56209	0.27131	TRUE	1.00E+00
36805_s_at	-0.14536	-1.397526	0.048158	-0.36754	0.07682	TRUE	1.00E+00
866_at	-0.14536	-1.397526	0.069018	-0.46377	0.17306	TRUE	1.00E+00
102_at	-0.14529	-1.397301	0.032542	-0.29542	0.00485	TRUE	1.01E-01
31404_at	-0.14524	-1.39714	0.023689	-0.25453	-0.03595	FALSE	1.10E-05
34959_at	-0.14522	-1.397076	0.090565	-0.56305	0.27261	TRUE	1.00E+00
39604_at	-0.14513	-1.396786	0.125678	-0.72495	0.4347	TRUE	1.00E+00
34008_at	-0.14511	-1.396722	0.058401	-0.41455	0.12432	TRUE	1.00E+00
37866_at	-0.1451	-1.39669	0.041531	-0.33671	0.0465	TRUE	1.00E+00
33112_at	-0.1451	-1.39669	0.070324	-0.46954	0.17935	TRUE	1.00E+00
39576_at	-0.14507	-1.396593	0.117075	-0.68521	0.39507	TRUE	1.00E+00
33178_at	-0.14507	-1.396593	0.058417	-0.41458	0.12444	TRUE	1.00E+00
38845_at	-0.14501	-1.396401	0.120141	-0.69929	0.40927	TRUE	1.00E+00
34010_at	-0.14497	-1.396272	0.036554	-0.31362	0.02367	TRUE	9.23E-01
41497_at	-0.14481	-1.395758	0.028333	-0.27552	-0.01409	FALSE	4.05E-03
34949_at	-0.14478	-1.395661	0.03623	-0.31193	0.02237	TRUE	8.13E-01
40366_at	-0.14478	-1.395661	0.096951	-0.59207	0.30251	TRUE	1.00E+00

35897_r_at	-0.14469	-1.395372	0.040214	-0.33022	0.04084	TRUE	1.00E+00
affx-yel021w/ura3	-0.14464	-1.395211	0.080846	-0.51763	0.22834	TRUE	1.00E+00
41351_at	-0.14456	-1.394954	0.035436	-0.30805	0.01893	TRUE	5.70E-01
39608_at	-0.14454	-1.39489	0.093778	-0.57719	0.28811	TRUE	1.00E+00
38138_at	-0.14451	-1.394794	0.025648	-0.26284	-0.02619	FALSE	2.22E-04
39171_at	-0.1445	-1.394762	0.042204	-0.33922	0.05021	TRUE	1.00E+00
31979_at	-0.1444	-1.394441	0.018019	-0.22754	-0.06127	FALSE	1.40E-11
846_s_at	-0.14437	-1.394344	0.092368	-0.57052	0.28178	TRUE	1.00E+00
36356_at	-0.14428	-1.394055	0.045114	-0.35242	0.06385	TRUE	1.00E+00
35512_at	-0.14417	-1.393702	0.055646	-0.4009	0.11255	TRUE	1.00E+00
34045_at	-0.1441	-1.393478	0.091951	-0.56833	0.28012	TRUE	1.00E+00
41525_at	-0.14405	-1.393317	0.018158	-0.22783	-0.06028	FALSE	2.69E-11
849_g_at	-0.14401	-1.393189	0.069597	-0.4651	0.17708	TRUE	1.00E+00
36531_r_at	-0.14397	-1.393061	0.04448	-0.34919	0.06124	TRUE	1.00E+00
32736_at	-0.14386	-1.392708	0.067016	-0.45305	0.16532	TRUE	1.00E+00
32141_at	-0.14385	-1.392676	0.041889	-0.33711	0.04941	TRUE	1.00E+00
39213_at	-0.14383	-1.392612	0.220116	-1.15935	0.8717	TRUE	1.00E+00
37158_at	-0.14372	-1.392259	0.022059	-0.24549	-0.04195	FALSE	9.16E-07
40650_r_at	-0.14368	-1.392131	0.061791	-0.42876	0.1414	TRUE	1.00E+00
39460_g_at	-0.14366	-1.392067	0.059792	-0.41952	0.13219	TRUE	1.00E+00
35598_at	-0.14363	-1.39197	0.059887	-0.41993	0.13266	TRUE	1.00E+00
37492_at	-0.14352	-1.391618	0.039411	-0.32535	0.0383	TRUE	1.00E+00
31875_at	-0.1433	-1.390913	0.052428	-0.38518	0.09858	TRUE	1.00E+00
38513_at	-0.14328	-1.390849	0.059596	-0.41823	0.13167	TRUE	1.00E+00
36063_at	-0.14326	-1.390785	0.063143	-0.43458	0.14805	TRUE	1.00E+00
37166_at	-0.14323	-1.390689	0.046532	-0.35791	0.07145	TRUE	1.00E+00
363_at	-0.1432	-1.390593	0.031219	-0.28723	0.00083	TRUE	5.67E-02
35660_at	-0.14319	-1.390561	0.020589	-0.23818	-0.0482	FALSE	4.46E-08
41148_at	-0.14319	-1.390561	0.138954	-0.78426	0.49789	TRUE	1.00E+00
1355_g_at	-0.14315	-1.390433	0.075579	-0.49184	0.20553	TRUE	1.00E+00
34541_at	-0.14293	-1.389729	0.036675	-0.31213	0.02628	TRUE	1.00E+00
32922_at	-0.14279	-1.389281	0.084762	-0.53385	0.24827	TRUE	1.00E+00
40589_at	-0.14276	-1.389185	0.038155	-0.31879	0.03327	TRUE	1.00E+00
35182_f_at	-0.14274	-1.389121	0.031609	-0.28857	0.00309	TRUE	7.96E-02
40540_at	-0.14265	-1.388833	0.053565	-0.38977	0.10448	TRUE	1.00E+00
32364_at	-0.14263	-1.388769	0.110243	-0.65124	0.36599	TRUE	1.00E+00
32949_at	-0.14262	-1.388737	0.061333	-0.42559	0.14034	TRUE	1.00E+00
34449_at	-0.14262	-1.388737	0.009992	-0.18872	-0.09652	FALSE	4.03E-42
797_at	-0.1425	-1.388353	0.082183	-0.52166	0.23666	TRUE	1.00E+00
31778_at	-0.14249	-1.388321	0.044682	-0.34863	0.06366	TRUE	1.00E+00
32285_g_at	-0.14247	-1.388257	0.059331	-0.4162	0.13126	TRUE	1.00E+00
36937_s_at	-0.14246	-1.388225	0.028788	-0.27528	-0.00965	FALSE	9.44E-03
35349_at	-0.14224	-1.387522	0.042154	-0.33672	0.05224	TRUE	1.00E+00
1998_i_at	-0.1422	-1.387395	0.037397	-0.31473	0.03034	TRUE	1.00E+00
222_at	-0.14208	-1.387011	0.019439	-0.23176	-0.0524	FALSE	3.40E-09
32127_at	-0.14201	-1.386788	0.023198	-0.24903	-0.03498	FALSE	1.17E-05
32023_at	-0.14198	-1.386692	0.074792	-0.48704	0.20308	TRUE	1.00E+00
32649_at	-0.14187	-1.386341	0.097444	-0.59144	0.30769	TRUE	1.00E+00
31501_at	-0.14185	-1.386277	0.121632	-0.70301	0.41931	TRUE	1.00E+00
2089_s_at	-0.14174	-1.385926	0.088417	-0.54966	0.26618	TRUE	1.00E+00
32553_at	-0.14172	-1.385862	0.118247	-0.68726	0.40382	TRUE	1.00E+00

37378_r_at	-0.14167	-1.385702	0.029933	-0.27976	-0.00357	FALSE	2.80E-02
40309_at	-0.14163	-1.385575	0.088446	-0.54968	0.26642	TRUE	1.00E+00
36340_at	-0.14163	-1.385575	0.037552	-0.31488	0.03163	TRUE	1.00E+00
38803_at	-0.14162	-1.385543	0.099912	-0.60257	0.31933	TRUE	1.00E+00
39256_at	-0.1416	-1.385479	0.087144	-0.54365	0.26044	TRUE	1.00E+00
35373_at	-0.14156	-1.385352	0.065534	-0.44391	0.16078	TRUE	1.00E+00
39364_s_at	-0.14152	-1.385224	0.045222	-0.35016	0.06711	TRUE	1.00E+00
37909_at	-0.14144	-1.384969	0.105374	-0.62759	0.34471	TRUE	1.00E+00
40294_at	-0.14135	-1.384682	0.02091	-0.23782	-0.04488	FALSE	1.75E-07
40695_at	-0.14124	-1.384331	0.01623	-0.21612	-0.06636	FALSE	4.10E-14
31379_at	-0.14102	-1.38363	0.046592	-0.35597	0.07394	TRUE	1.00E+00
36776_at	-0.14045	-1.381815	0.039636	-0.32331	0.04241	TRUE	1.00E+00
34757_at	-0.1402	-1.38102	0.028378	-0.27112	-0.00928	FALSE	9.84E-03
34231_at	-0.14018	-1.380957	0.021796	-0.24074	-0.03962	FALSE	1.60E-06
39854_r_at	-0.14017	-1.380925	0.054119	-0.38985	0.10951	TRUE	1.00E+00
417_at	-0.14014	-1.380829	0.070336	-0.46464	0.18436	TRUE	1.00E+00
36692_at	-0.14013	-1.380798	0.016973	-0.21844	-0.06182	FALSE	1.90E-12
40910_at	-0.14009	-1.38067	0.028096	-0.26971	-0.01047	FALSE	7.77E-03
35570_at	-0.14009	-1.38067	0.080789	-0.51282	0.23264	TRUE	1.00E+00
35436_at	-0.14007	-1.380607	0.014216	-0.20566	-0.07448	FALSE	8.42E-19
35260_at	-0.13984	-1.379876	0.033138	-0.29273	0.01304	TRUE	3.08E-01
31369_at	-0.13983	-1.379844	0.04951	-0.36825	0.08859	TRUE	1.00E+00
33970_at	-0.13971	-1.379463	0.111268	-0.65306	0.37363	TRUE	1.00E+00
38049_g_at	-0.1397	-1.379431	0.033316	-0.29341	0.01401	TRUE	3.47E-01
40455_at	-0.13964	-1.37924	0.012984	-0.19954	-0.07973	FALSE	7.14E-23
322_at	-0.13955	-1.378955	0.124183	-0.71248	0.43338	TRUE	1.00E+00
34047_at	-0.13949	-1.378764	0.05094	-0.37451	0.09553	TRUE	1.00E+00
37085_g_at	-0.13929	-1.378129	0.077805	-0.49825	0.21967	TRUE	1.00E+00
34576_at	-0.13923	-1.377939	0.042699	-0.33623	0.05776	TRUE	1.00E+00
36017_at	-0.1392	-1.377844	0.033682	-0.2946	0.01619	TRUE	4.52E-01
610_at	-0.13917	-1.377749	0.026735	-0.26252	-0.01583	FALSE	2.44E-03
1427_g_at	-0.13915	-1.377685	0.099187	-0.59676	0.31846	TRUE	1.00E+00
36958_at	-0.13913	-1.377622	0.024926	-0.25412	-0.02413	FALSE	3.01E-04
37591_at	-0.13888	-1.376829	0.076911	-0.49371	0.21596	TRUE	1.00E+00
37184_at	-0.13885	-1.376734	0.026996	-0.2634	-0.01431	FALSE	3.40E-03
35035_at	-0.13881	-1.376607	0.095558	-0.57967	0.30206	TRUE	1.00E+00
31544_at	-0.13876	-1.376449	0.105355	-0.62483	0.3473	TRUE	1.00E+00
37652_at	-0.13871	-1.37629	0.029452	-0.27459	-0.00283	FALSE	3.13E-02
40009_at	-0.13865	-1.3761	0.041148	-0.32849	0.05119	TRUE	1.00E+00
36362_at	-0.13859	-1.37591	0.095955	-0.58129	0.30411	TRUE	1.00E+00
32294_g_at	-0.13858	-1.375878	0.080958	-0.51209	0.23493	TRUE	1.00E+00
40686_at	-0.13852	-1.375688	0.029718	-0.27562	-0.00141	FALSE	3.97E-02
41619_at	-0.13851	-1.375656	0.031767	-0.28507	0.00805	TRUE	1.64E-01
523_s_at	-0.13848	-1.375561	0.084333	-0.52756	0.25059	TRUE	1.00E+00
33115_at	-0.13844	-1.375435	0.098685	-0.59373	0.31685	TRUE	1.00E+00
39321_at	-0.13843	-1.375403	0.04309	-0.33723	0.06037	TRUE	1.00E+00
33405_at	-0.13838	-1.375245	0.031387	-0.28319	0.00642	TRUE	1.31E-01
38163_at	-0.13833	-1.375086	0.07035	-0.4629	0.18623	TRUE	1.00E+00
1918_at	-0.13822	-1.374738	0.079564	-0.5053	0.22885	TRUE	1.00E+00
36248_at	-0.13814	-1.374485	0.025778	-0.25707	-0.01922	FALSE	1.06E-03
35338_at	-0.1381	-1.374358	0.032434	-0.28774	0.01153	TRUE	2.60E-01

461_at	-0.1381	-1.374358	0.057057	-0.40134	0.12514	TRUE	1.00E+00
35206_at	-0.13802	-1.374105	0.032229	-0.28671	0.01067	TRUE	2.33E-01
2050_s_at	-0.13781	-1.373441	0.036301	-0.30529	0.02967	TRUE	1.00E+00
35200_at	-0.13767	-1.372998	0.051446	-0.37502	0.09968	TRUE	1.00E+00
39122_at	-0.13763	-1.372872	0.020407	-0.23178	-0.04348	FALSE	1.94E-07
37513_at	-0.13756	-1.372651	0.096643	-0.58343	0.30831	TRUE	1.00E+00
41293_at	-0.13749	-1.372429	0.049031	-0.36369	0.08872	TRUE	1.00E+00
35862_at	-0.13746	-1.372335	0.102558	-0.61062	0.33571	TRUE	1.00E+00
33676_at	-0.13738	-1.372082	0.03136	-0.28206	0.0073	TRUE	1.49E-01
33697_at	-0.13736	-1.372019	0.049219	-0.36443	0.08972	TRUE	1.00E+00
39689_at	-0.13734	-1.371955	0.034327	-0.29571	0.02103	TRUE	7.97E-01
955_at	-0.13732	-1.371892	0.019337	-0.22653	-0.04811	FALSE	1.56E-08
32049_f_at	-0.13724	-1.37164	0.132361	-0.74789	0.47342	TRUE	1.00E+00
37831_at	-0.13723	-1.371608	0.031088	-0.28066	0.00619	TRUE	1.28E-01
34847_s_at	-0.13719	-1.371482	0.022542	-0.24119	-0.03319	FALSE	1.46E-05
38206_at	-0.13712	-1.371261	0.040279	-0.32295	0.04871	TRUE	1.00E+00
33707_at	-0.13703	-1.370976	0.04796	-0.3583	0.08424	TRUE	1.00E+00
41564_at	-0.13696	-1.370756	0.083559	-0.52247	0.24854	TRUE	1.00E+00
38647_at	-0.13695	-1.370724	0.033166	-0.28997	0.01606	TRUE	4.59E-01
32207_at	-0.13691	-1.370598	0.029987	-0.27526	0.00144	TRUE	6.29E-02
36199_at	-0.13685	-1.370408	0.039542	-0.31928	0.04558	TRUE	1.00E+00
39631_at	-0.13685	-1.370408	0.050757	-0.37102	0.09732	TRUE	1.00E+00
37377_i_at	-0.13685	-1.370408	0.036525	-0.30536	0.03167	TRUE	1.00E+00
287_at	-0.13683	-1.370345	0.039104	-0.31725	0.04358	TRUE	1.00E+00
32040_i_at	-0.13682	-1.370314	0.062923	-0.42712	0.15348	TRUE	1.00E+00
34432_at	-0.13678	-1.370187	0.047367	-0.35531	0.08175	TRUE	1.00E+00
37644_s_at	-0.13669	-1.369904	0.048357	-0.35979	0.08641	TRUE	1.00E+00
991_g_at	-0.13652	-1.369367	0.031862	-0.28352	0.01048	TRUE	2.31E-01
1622_at	-0.13649	-1.369273	0.054996	-0.39022	0.11724	TRUE	1.00E+00
32229_at	-0.13634	-1.3688	0.025785	-0.25531	-0.01738	FALSE	1.56E-03
37791_at	-0.13632	-1.368737	0.087815	-0.54146	0.26882	TRUE	1.00E+00
40805_at	-0.13631	-1.368705	0.04774	-0.35656	0.08395	TRUE	1.00E+00
36213_at	-0.13626	-1.368548	0.130823	-0.73982	0.46731	TRUE	1.00E+00
35805_at	-0.13624	-1.368485	0.021278	-0.2344	-0.03807	FALSE	1.93E-06
37488_at	-0.13614	-1.36817	0.017136	-0.2152	-0.05708	FALSE	2.45E-11
35694_at	-0.13614	-1.36817	0.030334	-0.27609	0.00381	TRUE	9.07E-02
2022_at	-0.13614	-1.36817	0.102607	-0.60952	0.33725	TRUE	1.00E+00
41038_at	-0.13593	-1.367508	0.065906	-0.44	0.16813	TRUE	1.00E+00
38870_at	-0.13593	-1.367508	0.083358	-0.52051	0.24865	TRUE	1.00E+00
37835_at	-0.13592	-1.367477	0.021086	-0.2332	-0.03864	FALSE	1.45E-06
40159_r_at	-0.1359	-1.367414	0.029827	-0.27352	0.00171	TRUE	6.57E-02
41866_s_at	-0.13584	-1.367225	0.080572	-0.50757	0.23589	TRUE	1.00E+00
848_at	-0.13566	-1.366658	0.054402	-0.38665	0.11533	TRUE	1.00E+00
1153_f_at	-0.13559	-1.366438	0.020739	-0.23127	-0.03991	FALSE	7.87E-07
31316_at	-0.13545	-1.365998	0.089261	-0.54726	0.27636	TRUE	1.00E+00
33842_at	-0.13542	-1.365903	0.02838	-0.26635	-0.00448	FALSE	2.31E-02
31450_s_at	-0.13536	-1.365715	0.055602	-0.39188	0.12117	TRUE	1.00E+00
1851_s_at	-0.13531	-1.365558	0.084778	-0.52644	0.25583	TRUE	1.00E+00
34627_at	-0.13524	-1.365337	0.048821	-0.36048	0.09	TRUE	1.00E+00
31488_s_at	-0.13513	-1.364992	0.052266	-0.37627	0.106	TRUE	1.00E+00
41541_at	-0.13512	-1.36496	0.079695	-0.50281	0.23256	TRUE	1.00E+00

31352_at	-0.1351	-1.364897	0.037459	-0.30792	0.03772	TRUE	1.00E+00
790_at	-0.13507	-1.364803	0.033282	-0.28862	0.01848	TRUE	6.24E-01
40987_at	-0.13488	-1.364206	0.108112	-0.63367	0.36391	TRUE	1.00E+00
32134_at	-0.13487	-1.364175	0.047971	-0.35619	0.08645	TRUE	1.00E+00
37099_at	-0.13485	-1.364112	0.079616	-0.50217	0.23246	TRUE	1.00E+00
34032_at	-0.13474	-1.363766	0.090476	-0.55216	0.28268	TRUE	1.00E+00
878_s_at	-0.13473	-1.363735	0.027104	-0.25978	-0.00969	FALSE	8.41E-03
40863_r_at	-0.13466	-1.363515	0.02781	-0.26297	-0.00636	FALSE	1.62E-02
111_at	-0.13465	-1.363484	0.013275	-0.1959	-0.07341	FALSE	4.49E-20
38570_at	-0.13464	-1.363452	0.039145	-0.31524	0.04596	TRUE	1.00E+00
36724_s_at	-0.13463	-1.363421	0.064634	-0.43282	0.16356	TRUE	1.00E+00
34617_at	-0.13459	-1.363295	0.16864	-0.91262	0.64344	TRUE	1.00E+00
336_at	-0.13457	-1.363233	0.043327	-0.33446	0.06533	TRUE	1.00E+00
33834_at	-0.13455	-1.36317	0.051271	-0.37109	0.10199	TRUE	1.00E+00
31601_s_at	-0.13454	-1.363139	0.094881	-0.57228	0.3032	TRUE	1.00E+00
40689_at	-0.13439	-1.362668	0.036535	-0.30295	0.03417	TRUE	1.00E+00
33768_at	-0.13428	-1.362323	0.017317	-0.21417	-0.05439	FALSE	1.12E-10
956_at	-0.13425	-1.362229	0.027312	-0.26026	-0.00825	FALSE	1.12E-02
39910_at	-0.1342	-1.362072	0.066955	-0.4431	0.1747	TRUE	1.00E+00
1343_s_at	-0.13418	-1.362009	0.106282	-0.62452	0.35616	TRUE	1.00E+00
35584_s_at	-0.13417	-1.361978	0.044213	-0.33815	0.06981	TRUE	1.00E+00
34258_at	-0.13415	-1.361915	0.018676	-0.22031	-0.04799	FALSE	8.60E-09
32029_at	-0.1341	-1.361758	0.024438	-0.24684	-0.02135	FALSE	5.15E-04
37979_at	-0.13394	-1.361257	0.050524	-0.36704	0.09916	TRUE	1.00E+00
533_g_at	-0.13377	-1.360724	0.068481	-0.44971	0.18218	TRUE	1.00E+00
40302_at	-0.13374	-1.36063	0.032786	-0.285	0.01752	TRUE	5.71E-01
39474_s_at	-0.13367	-1.360411	0.053634	-0.38111	0.11378	TRUE	1.00E+00
31759_at	-0.13366	-1.360379	0.071185	-0.46208	0.19476	TRUE	1.00E+00
32024_at	-0.13366	-1.360379	0.122126	-0.6971	0.42978	TRUE	1.00E+00
36731_g_at	-0.13351	-1.359909	0.011926	-0.18853	-0.07848	FALSE	5.49E-25
32473_at	-0.13339	-1.359534	0.128339	-0.72549	0.45871	TRUE	1.00E+00
1514_g_at	-0.1333	-1.359252	0.085	-0.52546	0.25885	TRUE	1.00E+00
31598_s_at	-0.13328	-1.359189	0.103961	-0.61291	0.34636	TRUE	1.00E+00
601_s_at	-0.13326	-1.359127	0.105288	-0.61901	0.35249	TRUE	1.00E+00
39808_at	-0.13319	-1.358908	0.048545	-0.35716	0.09078	TRUE	1.00E+00
695_at	-0.13311	-1.358658	0.058303	-0.4021	0.13588	TRUE	1.00E+00
34859_at	-0.13308	-1.358564	0.020007	-0.22538	-0.04077	FALSE	3.66E-07
41804_at	-0.13307	-1.358532	0.02868	-0.26539	-0.00075	FALSE	4.40E-02
39880_at	-0.13293	-1.358095	0.047889	-0.35387	0.08801	TRUE	1.00E+00
32094_at	-0.13291	-1.358032	0.009783	-0.17805	-0.08778	FALSE	6.15E-38
39589_at	-0.13273	-1.357469	0.131002	-0.73711	0.47166	TRUE	1.00E+00
32930_f_at	-0.13262	-1.357125	0.110556	-0.64268	0.37744	TRUE	1.00E+00
35434_at	-0.13252	-1.356813	0.016741	-0.20975	-0.05528	FALSE	3.10E-11
32423_at	-0.13252	-1.356813	0.041746	-0.32512	0.06008	TRUE	1.00E+00
35546_at	-0.1325	-1.356751	0.060155	-0.41003	0.14503	TRUE	1.00E+00
38897_at	-0.13246	-1.356626	0.030271	-0.27211	0.0072	TRUE	1.53E-01
32101_at	-0.13244	-1.356563	0.054721	-0.3849	0.12002	TRUE	1.00E+00
40190_at	-0.13242	-1.356501	0.128233	-0.72403	0.45919	TRUE	1.00E+00
33021_at	-0.13241	-1.356469	0.064938	-0.432	0.16719	TRUE	1.00E+00
34639_at	-0.13239	-1.356407	0.023679	-0.24163	-0.02314	FALSE	2.85E-04
35678_at	-0.13212	-1.355564	0.136838	-0.76344	0.49919	TRUE	1.00E+00

40888_f_at	-0.13205	-1.355345	0.061628	-0.41638	0.15227	TRUE	1.00E+00
33391_r_at	-0.13202	-1.355252	0.069209	-0.45132	0.18729	TRUE	1.00E+00
1027_at	-0.13201	-1.355221	0.041566	-0.32378	0.05975	TRUE	1.00E+00
40835_at	-0.132	-1.355189	0.040459	-0.31867	0.05466	TRUE	1.00E+00
34501_at	-0.13194	-1.355002	0.051652	-0.37024	0.10636	TRUE	1.00E+00
33529_at	-0.13193	-1.354971	0.072582	-0.46679	0.20293	TRUE	1.00E+00
1517_at	-0.1319	-1.354877	0.03502	-0.29346	0.02967	TRUE	1.00E+00
37795_at	-0.13188	-1.354815	0.058859	-0.40342	0.13967	TRUE	1.00E+00
35429_at	-0.13186	-1.354753	0.067788	-0.4446	0.18089	TRUE	1.00E+00
35979_at	-0.13176	-1.354441	0.022203	-0.2342	-0.02933	FALSE	3.72E-05
39636_at	-0.13173	-1.354347	0.031656	-0.27777	0.01432	TRUE	4.00E-01
41539_at	-0.1317	-1.354254	0.064684	-0.43012	0.16673	TRUE	1.00E+00
705_at	-0.13169	-1.354222	0.060501	-0.41082	0.14744	TRUE	1.00E+00
310_s_at	-0.13168	-1.354191	0.040392	-0.31803	0.05468	TRUE	1.00E+00
34215_at	-0.13149	-1.353599	0.054722	-0.38395	0.12098	TRUE	1.00E+00
31930_f_at	-0.13138	-1.353256	0.043268	-0.331	0.06824	TRUE	1.00E+00
36291_at	-0.1313	-1.353007	0.088151	-0.53799	0.27539	TRUE	1.00E+00
37525_at	-0.1312	-1.352695	0.053592	-0.37845	0.11605	TRUE	1.00E+00
1416_g_at	-0.13107	-1.352291	0.05217	-0.37176	0.10962	TRUE	1.00E+00
34153_at	-0.13091	-1.351792	0.080964	-0.50445	0.24262	TRUE	1.00E+00
34320_at	-0.13086	-1.351637	0.040444	-0.31745	0.05573	TRUE	1.00E+00
39241_at	-0.13085	-1.351606	0.037283	-0.30286	0.04116	TRUE	1.00E+00
31327_at	-0.13073	-1.351232	0.096762	-0.57715	0.31569	TRUE	1.00E+00
40850_at	-0.13048	-1.350455	0.098135	-0.58323	0.32227	TRUE	1.00E+00
39214_at	-0.13042	-1.350268	0.082448	-0.5108	0.24996	TRUE	1.00E+00
34470_at	-0.13041	-1.350237	0.112711	-0.65041	0.38959	TRUE	1.00E+00
1590_s_at	-0.13026	-1.349771	0.039609	-0.313	0.05248	TRUE	1.00E+00
39709_at	-0.1302	-1.349584	0.021274	-0.22835	-0.03205	FALSE	1.18E-05
40084_at	-0.13019	-1.349553	0.033356	-0.28409	0.0237	TRUE	1.00E+00
31897_at	-0.13018	-1.349522	0.064626	-0.42834	0.16798	TRUE	1.00E+00
36694_at	-0.13012	-1.349336	0.095771	-0.57197	0.31173	TRUE	1.00E+00
32939_g_at	-0.13008	-1.349211	0.053216	-0.3756	0.11543	TRUE	1.00E+00
35544_at	-0.13005	-1.349118	0.124715	-0.70543	0.44533	TRUE	1.00E+00
33292_at	-0.12997	-1.34887	0.0928	-0.55811	0.29817	TRUE	1.00E+00
41079_at	-0.12994	-1.348777	0.045394	-0.33937	0.07948	TRUE	1.00E+00
36452_at	-0.12993	-1.348745	0.065321	-0.43129	0.17144	TRUE	1.00E+00
40539_at	-0.12989	-1.348621	0.018147	-0.21361	-0.04616	FALSE	1.04E-08
38514_at	-0.12984	-1.348466	0.076834	-0.48433	0.22464	TRUE	1.00E+00
34095_f_at	-0.12984	-1.348466	0.077331	-0.48661	0.22693	TRUE	1.00E+00
32013_at	-0.12976	-1.348218	0.116166	-0.66571	0.40618	TRUE	1.00E+00
40266_at	-0.12974	-1.348156	0.050154	-0.36113	0.10165	TRUE	1.00E+00
37598_at	-0.12973	-1.348124	0.053513	-0.37662	0.11715	TRUE	1.00E+00
37773_at	-0.12973	-1.348124	0.137527	-0.76422	0.50476	TRUE	1.00E+00
32604_s_at	-0.12938	-1.347038	0.031932	-0.2767	0.01795	TRUE	6.42E-01
31818_at	-0.12926	-1.346666	0.067985	-0.44292	0.18439	TRUE	1.00E+00
33789_at	-0.12925	-1.346635	0.02932	-0.26452	0.00602	TRUE	1.32E-01
35411_at	-0.12922	-1.346542	0.040994	-0.31835	0.05991	TRUE	1.00E+00
31589_at	-0.1291	-1.34617	0.077473	-0.48652	0.22833	TRUE	1.00E+00
36558_at	-0.12898	-1.345798	0.020041	-0.22145	-0.03652	FALSE	1.55E-06
32391_g_at	-0.12895	-1.345705	0.097164	-0.57722	0.31932	TRUE	1.00E+00
34165_at	-0.12892	-1.345612	0.026105	-0.24936	-0.00848	FALSE	9.94E-03

41319_at	-0.12892	-1.345612	0.05312	-0.37399	0.11616	TRUE	1.00E+00
34966_at	-0.12889	-1.34552	0.035977	-0.29487	0.0371	TRUE	1.00E+00
40507_at	-0.12887	-1.345458	0.034269	-0.28697	0.02923	TRUE	1.00E+00
40681_at	-0.12881	-1.345272	0.040529	-0.31579	0.05818	TRUE	1.00E+00
37930_at	-0.1288	-1.345241	0.042345	-0.32416	0.06656	TRUE	1.00E+00
31615_i_at	-0.12872	-1.344993	0.059887	-0.40502	0.14758	TRUE	1.00E+00
32688_at	-0.12872	-1.344993	0.146655	-0.80532	0.54789	TRUE	1.00E+00
32633_at	-0.12866	-1.344807	0.059718	-0.40417	0.14686	TRUE	1.00E+00
1010_at	-0.12861	-1.344652	0.116686	-0.66695	0.40973	TRUE	1.00E+00
40567_at	-0.12856	-1.344498	0.024367	-0.24098	-0.01614	FALSE	1.67E-03
284_at	-0.12855	-1.344467	0.056851	-0.39083	0.13374	TRUE	1.00E+00
36703_at	-0.12842	-1.344064	0.053929	-0.37722	0.12039	TRUE	1.00E+00
34173_s_at	-0.12833	-1.343786	0.111439	-0.64246	0.38581	TRUE	1.00E+00
33937_at	-0.12827	-1.3436	0.09419	-0.56282	0.30629	TRUE	1.00E+00
36464_at	-0.1282	-1.343383	0.031714	-0.27452	0.01811	TRUE	6.68E-01
34117_at	-0.12809	-1.343043	0.136175	-0.75635	0.50017	TRUE	1.00E+00
35526_at	-0.128	-1.342765	0.020448	-0.22233	-0.03366	FALSE	4.87E-06
39472_s_at	-0.12798	-1.342703	0.031952	-0.27539	0.01944	TRUE	7.82E-01
40042_r_at	-0.12797	-1.342672	0.03753	-0.30112	0.04518	TRUE	1.00E+00
31351_at	-0.12796	-1.342641	0.050349	-0.36024	0.10433	TRUE	1.00E+00
35414_s_at	-0.12791	-1.342487	0.017545	-0.20885	-0.04696	FALSE	3.91E-09
35004_at	-0.12778	-1.342085	0.046168	-0.34078	0.08522	TRUE	1.00E+00
31746_at	-0.12774	-1.341961	0.107765	-0.62492	0.36944	TRUE	1.00E+00
33604_at	-0.12772	-1.3419	0.067602	-0.43961	0.18416	TRUE	1.00E+00
1486_at	-0.12766	-1.341714	0.045713	-0.33856	0.08324	TRUE	1.00E+00
31455_r_at	-0.12764	-1.341652	0.208587	-1.08998	0.83469	TRUE	1.00E+00
1720_at	-0.12759	-1.341498	0.105103	-0.61249	0.35731	TRUE	1.00E+00
160026_at	-0.12753	-1.341313	0.053848	-0.37597	0.1209	TRUE	1.00E+00
39768_at	-0.12741	-1.340942	0.066029	-0.43204	0.17722	TRUE	1.00E+00
38012_at	-0.12732	-1.340664	0.047901	-0.34831	0.09368	TRUE	1.00E+00
38487_at	-0.12726	-1.340479	0.021783	-0.22776	-0.02677	FALSE	6.50E-05
38002_s_at	-0.12723	-1.340386	0.052865	-0.37113	0.11667	TRUE	1.00E+00
34229_s_at	-0.12718	-1.340232	0.046908	-0.34359	0.08924	TRUE	1.00E+00
40154_at	-0.12711	-1.340016	0.097179	-0.57545	0.32123	TRUE	1.00E+00
35643_at	-0.12699	-1.339646	0.046546	-0.34173	0.08775	TRUE	1.00E+00
35693_at	-0.12696	-1.339553	0.039871	-0.31091	0.05698	TRUE	1.00E+00
160029_at	-0.12691	-1.339399	0.057377	-0.39163	0.1378	TRUE	1.00E+00
33835_at	-0.12691	-1.339399	0.02232	-0.22988	-0.02393	FALSE	1.64E-04
33717_at	-0.12687	-1.339276	0.099178	-0.58444	0.3307	TRUE	1.00E+00
35064_at	-0.12676	-1.338937	0.100333	-0.58965	0.33614	TRUE	1.00E+00
575_s_at	-0.12661	-1.338474	0.131756	-0.73448	0.48126	TRUE	1.00E+00
37035_at	-0.12661	-1.338474	0.019252	-0.21543	-0.03778	FALSE	6.09E-07
41091_at	-0.12653	-1.338228	0.033832	-0.28262	0.02955	TRUE	1.00E+00
37233_at	-0.12649	-1.338104	0.152699	-0.83098	0.578	TRUE	1.00E+00
1288_s_at	-0.12624	-1.337334	0.075895	-0.47638	0.22391	TRUE	1.00E+00
36367_at	-0.12622	-1.337273	0.107447	-0.62193	0.36949	TRUE	1.00E+00
35529_at	-0.1262	-1.337211	0.045	-0.33381	0.08141	TRUE	1.00E+00
38349_at	-0.12602	-1.336657	0.031345	-0.27063	0.01859	TRUE	7.33E-01
32090_at	-0.12599	-1.336565	0.116157	-0.66189	0.40991	TRUE	1.00E+00
33347_at	-0.12583	-1.336072	0.034512	-0.28505	0.0334	TRUE	1.00E+00
41409_at	-0.12582	-1.336042	0.048917	-0.3515	0.09986	TRUE	1.00E+00

35223_at	-0.12571	-1.335703	0.026308	-0.24708	-0.00433	FALSE	2.23E-02
39247_at	-0.12568	-1.335611	0.149214	-0.8141	0.56273	TRUE	1.00E+00
378_s_at	-0.12567	-1.33558	0.056107	-0.38452	0.13319	TRUE	1.00E+00
38196_at	-0.12566	-1.33555	0.053451	-0.37226	0.12094	TRUE	1.00E+00
33794_g_at	-0.12562	-1.335427	0.023372	-0.23345	-0.01779	FALSE	9.67E-04
40785_g_at	-0.12538	-1.334689	0.03213	-0.27362	0.02285	TRUE	1.00E+00
34035_at	-0.12538	-1.334689	0.04189	-0.31864	0.06789	TRUE	1.00E+00
39439_at	-0.12537	-1.334658	0.090356	-0.54224	0.29149	TRUE	1.00E+00
32030_at	-0.12532	-1.334504	0.025267	-0.24189	-0.00875	FALSE	8.90E-03
32321_at	-0.12521	-1.334166	0.033916	-0.28169	0.03126	TRUE	1.00E+00
35218_at	-0.12516	-1.334013	0.02552	-0.2429	-0.00742	FALSE	1.18E-02
32643_at	-0.12514	-1.333951	0.014427	-0.1917	-0.05858	FALSE	5.25E-14
40058_s_at	-0.12514	-1.333951	0.024707	-0.23912	-0.01115	FALSE	5.16E-03
1607_at	-0.12497	-1.333429	0.033008	-0.27725	0.02732	TRUE	1.00E+00
37988_at	-0.12494	-1.333337	0.030044	-0.26355	0.01368	TRUE	4.05E-01
34916_s_at	-0.12491	-1.333245	0.027635	-0.2524	0.00259	TRUE	7.81E-02
41570_at	-0.1249	-1.333214	0.118611	-0.67212	0.42232	TRUE	1.00E+00
31803_at	-0.12486	-1.333092	0.034247	-0.28286	0.03315	TRUE	1.00E+00
32122_at	-0.12484	-1.33303	0.031019	-0.26795	0.01827	TRUE	7.21E-01
224_at	-0.12483	-1.333	0.038452	-0.30223	0.05257	TRUE	1.00E+00
32159_at	-0.12482	-1.332969	0.029636	-0.26155	0.01191	TRUE	3.20E-01
36678_at	-0.12463	-1.332386	0.018715	-0.21098	-0.03829	FALSE	3.47E-07
41169_at	-0.12463	-1.332386	0.063225	-0.41632	0.16706	TRUE	1.00E+00
34618_at	-0.12446	-1.331864	0.047859	-0.34526	0.09634	TRUE	1.00E+00
925_at	-0.12443	-1.331772	0.059068	-0.39695	0.14808	TRUE	1.00E+00
32325_at	-0.12442	-1.331742	0.10613	-0.61406	0.36522	TRUE	1.00E+00
32164_at	-0.12436	-1.331558	0.044164	-0.32811	0.0794	TRUE	1.00E+00
32402_s_at	-0.12402	-1.330516	0.032272	-0.2729	0.02487	TRUE	1.00E+00
611_at	-0.12389	-1.330117	0.054152	-0.37372	0.12594	TRUE	1.00E+00
1876_at	-0.12373	-1.329628	0.030409	-0.26402	0.01657	TRUE	5.97E-01
40301_at	-0.12372	-1.329597	0.102222	-0.59533	0.34788	TRUE	1.00E+00
167_at	-0.12369	-1.329505	0.041186	-0.31371	0.06632	TRUE	1.00E+00
33846_at	-0.12369	-1.329505	0.054511	-0.37518	0.1278	TRUE	1.00E+00
41024_f_at	-0.12368	-1.329474	0.027646	-0.25123	0.00387	TRUE	9.72E-02
33571_at	-0.12346	-1.328801	0.069519	-0.44419	0.19727	TRUE	1.00E+00
34079_at	-0.12344	-1.32874	0.040534	-0.31044	0.06357	TRUE	1.00E+00
33379_at	-0.12336	-1.328495	0.110649	-0.63385	0.38713	TRUE	1.00E+00
40123_at	-0.12328	-1.328251	0.018216	-0.20732	-0.03924	FALSE	1.65E-07
36435_at	-0.12317	-1.327914	0.033037	-0.27559	0.02925	TRUE	1.00E+00
1021_at	-0.12311	-1.327731	0.05232	-0.36449	0.11828	TRUE	1.00E+00
35604_at	-0.12308	-1.327639	0.040025	-0.30774	0.06158	TRUE	1.00E+00
36294_at	-0.12304	-1.327517	0.042423	-0.31877	0.07268	TRUE	1.00E+00
32546_at	-0.123	-1.327394	0.031476	-0.26821	0.02222	TRUE	1.00E+00
1825_at	-0.12299	-1.327364	0.018135	-0.20666	-0.03933	FALSE	1.50E-07
34571_at	-0.12297	-1.327303	0.036874	-0.29309	0.04715	TRUE	1.00E+00
750_at	-0.12286	-1.326967	0.108515	-0.62351	0.37778	TRUE	1.00E+00
38316_at	-0.12269	-1.326447	0.038648	-0.301	0.05562	TRUE	1.00E+00
39220_at	-0.12266	-1.326356	0.051222	-0.35898	0.11366	TRUE	1.00E+00
35591_at	-0.12266	-1.326356	0.109128	-0.62613	0.38082	TRUE	1.00E+00
1707_g_at	-0.12263	-1.326264	0.039023	-0.30267	0.05741	TRUE	1.00E+00
34789_at	-0.12262	-1.326234	0.01058	-0.17143	-0.07381	FALSE	5.86E-27

34036_at	-0.12256	-1.32605	0.102364	-0.59483	0.3497	TRUE	1.00E+00
1559_at	-0.12246	-1.325745	0.050751	-0.3566	0.11168	TRUE	1.00E+00
41783_at	-0.12246	-1.325745	0.040195	-0.3079	0.06298	TRUE	1.00E+00
36653_g_at	-0.1224	-1.325562	0.022664	-0.22696	-0.01784	FALSE	8.39E-04
41292_at	-0.12228	-1.325196	0.043928	-0.32495	0.08038	TRUE	1.00E+00
39358_at	-0.12226	-1.325135	0.017316	-0.20216	-0.04237	FALSE	2.09E-08
31806_at	-0.12225	-1.325104	0.094808	-0.55965	0.31516	TRUE	1.00E+00
39114_at	-0.12214	-1.324769	0.065979	-0.42654	0.18226	TRUE	1.00E+00
40132_g_at	-0.12206	-1.324525	0.020208	-0.21529	-0.02882	FALSE	1.95E-05
39282_at	-0.12202	-1.324403	0.015789	-0.19487	-0.04918	FALSE	1.37E-10
33395_at	-0.12199	-1.324311	0.035141	-0.28412	0.04013	TRUE	1.00E+00
41587_g_at	-0.12198	-1.324281	0.118812	-0.67013	0.42617	TRUE	1.00E+00
163_at	-0.12195	-1.324189	0.025289	-0.23863	-0.00528	FALSE	1.79E-02
33808_at	-0.12194	-1.324159	0.0257	-0.24051	-0.00337	FALSE	2.64E-02
41404_at	-0.12191	-1.324067	0.040182	-0.30729	0.06347	TRUE	1.00E+00
38625_g_at	-0.12187	-1.323945	0.011269	-0.17386	-0.06988	FALSE	3.72E-23
39973_at	-0.12185	-1.323884	0.04231	-0.31705	0.07335	TRUE	1.00E+00
32982_at	-0.12185	-1.323884	0.105825	-0.61008	0.36638	TRUE	1.00E+00
39770_at	-0.1218	-1.323732	0.024281	-0.23382	-0.00977	FALSE	6.66E-03
33799_at	-0.12177	-1.32364	0.010153	-0.16862	-0.07493	FALSE	4.85E-29
37809_at	-0.12176	-1.32361	0.041018	-0.311	0.06748	TRUE	1.00E+00
34780_at	-0.12169	-1.323397	0.020207	-0.21492	-0.02846	FALSE	2.18E-05
37129_at	-0.12165	-1.323275	0.010203	-0.16872	-0.07458	FALSE	1.13E-28
36030_at	-0.12163	-1.323214	0.017314	-0.2015	-0.04175	FALSE	2.71E-08
39169_at	-0.12119	-1.321874	0.014041	-0.18597	-0.05641	FALSE	7.67E-14
34046_at	-0.12103	-1.321387	0.015212	-0.19121	-0.05085	FALSE	2.24E-11
34287_at	-0.12098	-1.321235	0.025008	-0.23636	-0.0056	FALSE	1.66E-02
348_at	-0.12069	-1.320353	0.100972	-0.58653	0.34515	TRUE	1.00E+00
41845_at	-0.12066	-1.320262	0.065241	-0.42166	0.18033	TRUE	1.00E+00
37018_at	-0.12066	-1.320262	0.124629	-0.69565	0.45433	TRUE	1.00E+00
293_at	-0.12058	-1.320018	0.020526	-0.21528	-0.02588	FALSE	5.35E-05
41065_at	-0.1205	-1.319775	0.08875	-0.52996	0.28896	TRUE	1.00E+00
34775_at	-0.12048	-1.319715	0.092116	-0.54547	0.3045	TRUE	1.00E+00
41386_i_at	-0.12048	-1.319715	0.015667	-0.19276	-0.0482	FALSE	1.86E-10
41129_at	-0.12044	-1.319593	0.015579	-0.19232	-0.04857	FALSE	1.35E-10
41742_s_at	-0.1204	-1.319471	0.034163	-0.27802	0.03721	TRUE	1.00E+00
241_g_at	-0.12026	-1.319046	0.016529	-0.19652	-0.044	FALSE	4.35E-09
1950_s_at	-0.12021	-1.318894	0.035164	-0.28245	0.04202	TRUE	1.00E+00
557_s_at	-0.12017	-1.318773	0.055667	-0.377	0.13665	TRUE	1.00E+00
38429_at	-0.12013	-1.318651	0.036457	-0.28833	0.04807	TRUE	1.00E+00
39567_at	-0.12006	-1.318439	0.046722	-0.33561	0.0955	TRUE	1.00E+00
37961_at	-0.11991	-1.317984	0.023735	-0.22941	-0.01041	FALSE	5.52E-03
33658_at	-0.11984	-1.317771	0.106627	-0.61178	0.37209	TRUE	1.00E+00
39254_at	-0.11983	-1.317741	0.018412	-0.20478	-0.03489	FALSE	9.58E-07
39904_at	-0.11982	-1.31771	0.056337	-0.37974	0.14009	TRUE	1.00E+00
33413_at	-0.11978	-1.317589	0.035142	-0.28191	0.04235	TRUE	1.00E+00
39459_at	-0.11973	-1.317437	0.054717	-0.37217	0.13271	TRUE	1.00E+00
31777_at	-0.11972	-1.317407	0.057514	-0.38507	0.14562	TRUE	1.00E+00
37898_r_at	-0.11962	-1.317104	0.051478	-0.35711	0.11788	TRUE	1.00E+00
33566_at	-0.11955	-1.316892	0.022923	-0.22531	-0.01379	FALSE	2.32E-03
37108_at	-0.11946	-1.316619	0.074753	-0.46434	0.22542	TRUE	1.00E+00

37294_at	-0.11946	-1.316619	0.029639	-0.2562	0.01729	TRUE	7.03E-01
39920_r_at	-0.11944	-1.316558	0.044742	-0.32586	0.08698	TRUE	1.00E+00
39375_g_at	-0.11928	-1.316073	0.059454	-0.39358	0.15501	TRUE	1.00E+00
37519_at	-0.11926	-1.316012	0.066193	-0.42464	0.18613	TRUE	1.00E+00
39486_s_at	-0.11923	-1.315922	0.031389	-0.26404	0.02559	TRUE	1.00E+00
1428_at	-0.11915	-1.315679	0.097633	-0.56959	0.33129	TRUE	1.00E+00
36447_at	-0.11912	-1.315588	0.021216	-0.217	-0.02123	FALSE	2.49E-04
295_s_at	-0.11888	-1.314861	0.059874	-0.39512	0.15735	TRUE	1.00E+00
39976_at	-0.11883	-1.31471	0.028638	-0.25095	0.0133	TRUE	4.21E-01
1879_at	-0.11881	-1.31465	0.037619	-0.29236	0.05475	TRUE	1.00E+00
35707_at	-0.11878	-1.314559	0.054787	-0.37155	0.13398	TRUE	1.00E+00
160022_at	-0.11875	-1.314468	0.069108	-0.43758	0.20008	TRUE	1.00E+00
1976_s_at	-0.11872	-1.314377	0.063754	-0.41285	0.17542	TRUE	1.00E+00
968_i_at	-0.1187	-1.314317	0.026356	-0.24029	0.0029	TRUE	8.44E-02
36307_at	-0.11863	-1.314105	0.020077	-0.21126	-0.02601	FALSE	4.34E-05
40359_at	-0.11844	-1.31353	0.022297	-0.22131	-0.01557	FALSE	1.37E-03
33631_at	-0.1184	-1.313409	0.02574	-0.23716	0.00035	TRUE	5.34E-02
33017_at	-0.11836	-1.313288	0.051677	-0.35678	0.12005	TRUE	1.00E+00
33056_at	-0.11835	-1.313258	0.164341	-0.87655	0.63986	TRUE	1.00E+00
35313_at	-0.11833	-1.313197	0.026246	-0.23942	0.00275	TRUE	8.24E-02
40476_s_at	-0.11821	-1.312835	0.028223	-0.24842	0.012	TRUE	3.55E-01
385_at	-0.11813	-1.312593	0.013474	-0.18029	-0.05596	FALSE	2.31E-14
34030_at	-0.11811	-1.312532	0.069437	-0.43846	0.20225	TRUE	1.00E+00
39170_at	-0.11809	-1.312472	0.020961	-0.2148	-0.02139	FALSE	2.22E-04
36231_at	-0.11804	-1.312321	0.043953	-0.32082	0.08474	TRUE	1.00E+00
39365_i_at	-0.11803	-1.312291	0.081204	-0.49267	0.25661	TRUE	1.00E+00
1246_at	-0.11788	-1.311837	0.069578	-0.43888	0.20313	TRUE	1.00E+00
40907_at	-0.11776	-1.311475	0.025721	-0.23642	0.00091	TRUE	5.92E-02
37933_at	-0.11774	-1.311415	0.078307	-0.47901	0.24353	TRUE	1.00E+00
34603_at	-0.11765	-1.311143	0.105802	-0.60578	0.37047	TRUE	1.00E+00
39938_g_at	-0.11762	-1.311052	0.105492	-0.60432	0.36908	TRUE	1.00E+00
35295_g_at	-0.11761	-1.311022	0.021924	-0.21876	-0.01646	FALSE	1.03E-03
1562_g_at	-0.11759	-1.310962	0.051644	-0.35585	0.12067	TRUE	1.00E+00
1734_at	-0.11758	-1.310932	0.069523	-0.43833	0.20317	TRUE	1.00E+00
35002_g_at	-0.11758	-1.310932	0.084167	-0.50589	0.27073	TRUE	1.00E+00
38650_at	-0.11757	-1.310901	0.038074	-0.29323	0.05809	TRUE	1.00E+00
31396_r_at	-0.11754	-1.310811	0.161741	-0.86375	0.62866	TRUE	1.00E+00
40729_s_at	-0.11754	-1.310811	0.0204	-0.21166	-0.02343	FALSE	1.05E-04
37257_at	-0.11748	-1.31063	0.04949	-0.34581	0.11084	TRUE	1.00E+00
38092_at	-0.11746	-1.310569	0.046113	-0.33021	0.09528	TRUE	1.00E+00
37361_at	-0.11745	-1.310539	0.040099	-0.30245	0.06755	TRUE	1.00E+00
32528_at	-0.11736	-1.310268	0.01127	-0.16936	-0.06537	FALSE	2.71E-21
150_at	-0.11727	-1.309996	0.047816	-0.33788	0.10333	TRUE	1.00E+00
1019_g_at	-0.11722	-1.309845	0.035585	-0.2814	0.04695	TRUE	1.00E+00
35019_at	-0.11721	-1.309815	0.040819	-0.30553	0.07111	TRUE	1.00E+00
33584_at	-0.11719	-1.309755	0.139444	-0.76053	0.52615	TRUE	1.00E+00
35422_at	-0.11718	-1.309725	0.083199	-0.50102	0.26667	TRUE	1.00E+00
32459_at	-0.1171	-1.309483	0.034819	-0.27774	0.04354	TRUE	1.00E+00
32729_at	-0.11709	-1.309453	0.047765	-0.33745	0.10328	TRUE	1.00E+00
32968_s_at	-0.11708	-1.309423	0.048688	-0.3417	0.10755	TRUE	1.00E+00
554_at	-0.117	-1.309182	0.024926	-0.232	-0.002	FALSE	3.38E-02

1214_s_at	-0.11696	-1.309061	0.072607	-0.45195	0.21802	TRUE	1.00E+00
36519_at	-0.11692	-1.308941	0.030489	-0.25759	0.02374	TRUE	1.00E+00
34218_at	-0.11687	-1.30879	0.034873	-0.27776	0.04401	TRUE	1.00E+00
38755_at	-0.11677	-1.308489	0.02212	-0.21882	-0.01472	FALSE	1.64E-03
31819_at	-0.11664	-1.308097	0.041475	-0.30799	0.07471	TRUE	1.00E+00
41590_at	-0.11661	-1.308007	0.043058	-0.31526	0.08204	TRUE	1.00E+00
36803_at	-0.11654	-1.307796	0.075075	-0.46291	0.22982	TRUE	1.00E+00
445_at	-0.11651	-1.307706	0.076178	-0.46796	0.23495	TRUE	1.00E+00
34485_r_at	-0.1165	-1.307676	0.045298	-0.32549	0.09248	TRUE	1.00E+00
1822_at	-0.11648	-1.307615	0.077074	-0.47207	0.23911	TRUE	1.00E+00
36165_at	-0.11641	-1.307405	0.012318	-0.17324	-0.05958	FALSE	4.26E-17
40227_at	-0.11638	-1.307314	0.015253	-0.18675	-0.04601	FALSE	2.96E-10
36993_at	-0.11638	-1.307314	0.024986	-0.23165	-0.0011	FALSE	4.04E-02
35489_at	-0.11637	-1.307284	0.038506	-0.29402	0.06128	TRUE	1.00E+00
40817_at	-0.11631	-1.307104	0.055677	-0.37318	0.14056	TRUE	1.00E+00
251_at	-0.11627	-1.306983	0.026569	-0.23886	0.00631	TRUE	1.52E-01
34769_at	-0.11621	-1.306803	0.019784	-0.20749	-0.02494	FALSE	5.37E-05
37363_at	-0.11621	-1.306803	0.084459	-0.50587	0.27344	TRUE	1.00E+00
2048_s_at	-0.11618	-1.306712	0.024162	-0.22765	-0.0047	FALSE	1.92E-02
31685_at	-0.11616	-1.306652	0.038017	-0.29156	0.05924	TRUE	1.00E+00
35090_g_at	-0.1161	-1.306472	0.059331	-0.38983	0.15763	TRUE	1.00E+00
38309_r_at	-0.11609	-1.306442	0.033524	-0.27076	0.03858	TRUE	1.00E+00
34786_at	-0.11604	-1.306291	0.061902	-0.40163	0.16955	TRUE	1.00E+00
37875_at	-0.116	-1.306171	0.051587	-0.354	0.122	TRUE	1.00E+00
37448_s_at	-0.1159	-1.30587	0.015542	-0.1876	-0.04419	FALSE	1.12E-09
41358_at	-0.11575	-1.305419	0.060696	-0.39577	0.16428	TRUE	1.00E+00
38255_at	-0.11568	-1.305209	0.065957	-0.41998	0.18862	TRUE	1.00E+00
32923_r_at	-0.11552	-1.304728	0.025376	-0.2326	0.00155	TRUE	6.69E-02
41054_at	-0.11547	-1.304578	0.03814	-0.29143	0.06049	TRUE	1.00E+00
35518_at	-0.11546	-1.304548	0.059283	-0.38896	0.15805	TRUE	1.00E+00
35274_at	-0.11542	-1.304428	0.032942	-0.2674	0.03656	TRUE	1.00E+00
31372_at	-0.11536	-1.304247	0.061114	-0.39731	0.16659	TRUE	1.00E+00
31675_s_at	-0.11535	-1.304217	0.025934	-0.235	0.00429	TRUE	1.09E-01
116_at	-0.11526	-1.303947	0.046714	-0.33078	0.10026	TRUE	1.00E+00
38423_at	-0.11518	-1.303707	0.033145	-0.2681	0.03774	TRUE	1.00E+00
32479_at	-0.11517	-1.303677	0.070626	-0.44101	0.21067	TRUE	1.00E+00
38871_at	-0.11514	-1.303587	0.047576	-0.33463	0.10435	TRUE	1.00E+00
1163_at	-0.11513	-1.303557	0.029143	-0.24958	0.01933	TRUE	9.85E-01
32471_at	-0.11511	-1.303497	0.095563	-0.556	0.32578	TRUE	1.00E+00
33076_at	-0.115	-1.303167	0.191054	-0.99644	0.76645	TRUE	1.00E+00
36471_f_at	-0.11477	-1.302477	0.064006	-0.41007	0.18053	TRUE	1.00E+00
31462_f_at	-0.11477	-1.302477	0.056038	-0.3733	0.14377	TRUE	1.00E+00
34748_at	-0.11469	-1.302237	0.014779	-0.18288	-0.04651	FALSE	1.07E-10
1328_at	-0.11468	-1.302207	0.0363	-0.28216	0.0528	TRUE	1.00E+00
40007_at	-0.11463	-1.302057	0.055685	-0.37154	0.14228	TRUE	1.00E+00
40713_at	-0.1146	-1.301967	0.043763	-0.3165	0.0873	TRUE	1.00E+00
40682_at	-0.11442	-1.301428	0.154984	-0.82945	0.60061	TRUE	1.00E+00
31959_at	-0.11437	-1.301278	0.129408	-0.7114	0.48266	TRUE	1.00E+00
32977_at	-0.11434	-1.301188	0.024244	-0.22619	-0.00248	FALSE	3.04E-02
35328_at	-0.11432	-1.301128	0.024791	-0.22869	0.00006	TRUE	5.05E-02
41561_s_at	-0.11426	-1.300948	0.025423	-0.23155	0.00303	TRUE	8.81E-02

32822_at	-0.11419	-1.300739	0.028711	-0.24665	0.01827	TRUE	8.80E-01
37206_at	-0.11418	-1.300709	0.06905	-0.43275	0.20439	TRUE	1.00E+00
39893_at	-0.11415	-1.300619	0.07243	-0.44832	0.22001	TRUE	1.00E+00
37689_s_at	-0.11409	-1.300439	0.092299	-0.53992	0.31174	TRUE	1.00E+00
41195_at	-0.11404	-1.300289	0.03488	-0.27496	0.04688	TRUE	1.00E+00
affx-hsac07/x003	-0.11402	-1.300229	0.152428	-0.81726	0.58922	TRUE	1.00E+00
34577_at	-0.11398	-1.30011	0.040732	-0.3019	0.07394	TRUE	1.00E+00
31647_at	-0.11376	-1.299451	0.123726	-0.68458	0.45706	TRUE	1.00E+00
34921_at	-0.11362	-1.299032	0.04417	-0.31741	0.09016	TRUE	1.00E+00
33117_r_at	-0.11362	-1.299032	0.022426	-0.21708	-0.01016	FALSE	5.12E-03
1458_at	-0.11354	-1.298793	0.039263	-0.29468	0.0676	TRUE	1.00E+00
36971_at	-0.11352	-1.298733	0.013786	-0.17713	-0.04992	FALSE	2.28E-12
37931_at	-0.1135	-1.298674	0.025498	-0.23114	0.00413	TRUE	1.08E-01
31667_r_at	-0.11343	-1.298464	0.099895	-0.5743	0.34745	TRUE	1.00E+00
39975_at	-0.11338	-1.298315	0.032326	-0.26252	0.03576	TRUE	1.00E+00
40700_at	-0.11332	-1.298135	0.043271	-0.31296	0.08631	TRUE	1.00E+00
33260_at	-0.1133	-1.298076	0.034273	-0.27142	0.04482	TRUE	1.00E+00
32641_at	-0.11329	-1.298046	0.033589	-0.26826	0.04168	TRUE	1.00E+00
1306_at	-0.11327	-1.297986	0.014011	-0.17791	-0.04863	FALSE	7.90E-12
39384_at	-0.11326	-1.297956	0.13476	-0.73499	0.50847	TRUE	1.00E+00
32124_at	-0.11311	-1.297508	0.01645	-0.189	-0.03722	FALSE	7.77E-08
40198_at	-0.11307	-1.297388	0.020716	-0.20864	-0.01749	FALSE	6.08E-04
36101_s_at	-0.11302	-1.297239	0.039542	-0.29546	0.06941	TRUE	1.00E+00
33432_at	-0.11295	-1.29703	0.03525	-0.27558	0.04968	TRUE	1.00E+00
38969_at	-0.11294	-1.297	0.040347	-0.29909	0.07321	TRUE	1.00E+00
40224_s_at	-0.11279	-1.296552	0.041118	-0.30249	0.07691	TRUE	1.00E+00
33130_at	-0.11278	-1.296522	0.067305	-0.4233	0.19774	TRUE	1.00E+00
1174_at	-0.11271	-1.296313	0.052384	-0.35438	0.12897	TRUE	1.00E+00
31795_at	-0.11268	-1.296224	0.03589	-0.27827	0.0529	TRUE	1.00E+00
32991_f_at	-0.11268	-1.296224	0.052533	-0.35504	0.12969	TRUE	1.00E+00
31750_at	-0.11267	-1.296194	0.099121	-0.56998	0.34463	TRUE	1.00E+00
37760_at	-0.11264	-1.296104	0.034888	-0.27359	0.04832	TRUE	1.00E+00
36530_g_at	-0.11262	-1.296045	0.067624	-0.42461	0.19937	TRUE	1.00E+00
1150_at	-0.11261	-1.296015	0.038333	-0.28946	0.06424	TRUE	1.00E+00
38610_s_at	-0.11259	-1.295955	0.026253	-0.23372	0.00853	TRUE	2.27E-01
40268_at	-0.11259	-1.295955	0.029318	-0.24785	0.02267	TRUE	1.00E+00
31335_at	-0.11256	-1.295866	0.056958	-0.37534	0.15022	TRUE	1.00E+00
35536_at	-0.11255	-1.295836	0.032543	-0.26269	0.03759	TRUE	1.00E+00
1089_i_at	-0.11253	-1.295776	0.181359	-0.94924	0.72419	TRUE	1.00E+00
31538_at	-0.11247	-1.295597	0.041903	-0.30579	0.08086	TRUE	1.00E+00
39303_at	-0.11243	-1.295478	0.123598	-0.68266	0.4578	TRUE	1.00E+00
37648_at	-0.11227	-1.295001	0.0215	-0.21146	-0.01308	FALSE	2.23E-03
40237_at	-0.11216	-1.294673	0.015619	-0.18422	-0.0401	FALSE	8.72E-09
37614_g_at	-0.11214	-1.294613	0.040074	-0.29703	0.07274	TRUE	1.00E+00
256_s_at	-0.1121	-1.294494	0.060332	-0.39045	0.16624	TRUE	1.00E+00
33628_g_at	-0.11209	-1.294464	0.070704	-0.43829	0.21411	TRUE	1.00E+00
35043_at	-0.11206	-1.294375	0.025993	-0.23198	0.00786	TRUE	2.05E-01
38715_at	-0.11205	-1.294345	0.103644	-0.59022	0.36612	TRUE	1.00E+00
1803_at	-0.11203	-1.294285	0.132894	-0.72514	0.50109	TRUE	1.00E+00
39998_at	-0.11184	-1.293719	0.034596	-0.27145	0.04777	TRUE	1.00E+00
38113_at	-0.11181	-1.29363	0.064499	-0.40938	0.18576	TRUE	1.00E+00

1855_at	-0.11175	-1.293451	0.038346	-0.28866	0.06516	TRUE	1.00E+00
37952_at	-0.11174	-1.293421	0.063632	-0.40531	0.18184	TRUE	1.00E+00
36302_f_at	-0.11171	-1.293332	0.055973	-0.36995	0.14652	TRUE	1.00E+00
33499_s_at	-0.11166	-1.293183	0.022158	-0.21389	-0.00944	FALSE	5.89E-03
36798_g_at	-0.11165	-1.293153	0.020166	-0.20469	-0.01862	FALSE	3.89E-04
1274_s_at	-0.11163	-1.293094	0.023539	-0.22023	-0.00303	FALSE	2.67E-02
33423_g_at	-0.11161	-1.293034	0.049123	-0.33824	0.11503	TRUE	1.00E+00
432_s_at	-0.11146	-1.292588	0.127166	-0.69815	0.47523	TRUE	1.00E+00
31995_g_at	-0.1114	-1.292409	0.058592	-0.38172	0.15892	TRUE	1.00E+00
31990_at	-0.11138	-1.29235	0.035342	-0.27444	0.05167	TRUE	1.00E+00
39937_at	-0.11132	-1.292171	0.06465	-0.40959	0.18695	TRUE	1.00E+00
38254_at	-0.11126	-1.291993	0.023597	-0.22013	-0.00239	FALSE	3.05E-02
38235_at	-0.1112	-1.291814	0.042714	-0.30826	0.08586	TRUE	1.00E+00
38234_at	-0.11111	-1.291546	0.023796	-0.22089	-0.00132	FALSE	3.82E-02
1795_g_at	-0.11108	-1.291457	0.033079	-0.2637	0.04153	TRUE	1.00E+00
40003_at	-0.11108	-1.291457	0.117581	-0.65355	0.43139	TRUE	1.00E+00
36641_at	-0.11106	-1.291398	0.013953	-0.17544	-0.04669	FALSE	2.18E-11
40771_at	-0.11104	-1.291338	0.017985	-0.19402	-0.02807	FALSE	8.39E-06
38597_f_at	-0.11104	-1.291338	0.038371	-0.28807	0.06599	TRUE	1.00E+00
40405_at	-0.11102	-1.291279	0.019116	-0.19921	-0.02283	FALSE	8.00E-05
32753_at	-0.1109	-1.290922	0.039444	-0.29288	0.07108	TRUE	1.00E+00
31724_at	-0.11089	-1.290892	0.023928	-0.22128	-0.0005	FALSE	4.52E-02
37175_at	-0.11082	-1.290684	0.033837	-0.26692	0.04529	TRUE	1.00E+00
39226_at	-0.11081	-1.290655	0.059008	-0.38305	0.16143	TRUE	1.00E+00
33733_at	-0.11081	-1.290655	0.055917	-0.36878	0.14717	TRUE	1.00E+00
33467_at	-0.11065	-1.290179	0.05892	-0.38248	0.16119	TRUE	1.00E+00
990_at	-0.11035	-1.289288	0.046827	-0.32639	0.10569	TRUE	1.00E+00
35068_at	-0.11034	-1.289258	0.030353	-0.25037	0.0297	TRUE	1.00E+00
36997_at	-0.11026	-1.289021	0.031257	-0.25447	0.03395	TRUE	1.00E+00
1566_at	-0.11026	-1.289021	0.097778	-0.56136	0.34085	TRUE	1.00E+00
33274_f_at	-0.11024	-1.288962	0.056931	-0.3729	0.15242	TRUE	1.00E+00
32840_at	-0.11023	-1.288932	0.092033	-0.53484	0.31437	TRUE	1.00E+00
41204_s_at	-0.1102	-1.288843	0.042233	-0.30505	0.08464	TRUE	1.00E+00
36332_at	-0.11019	-1.288813	0.021669	-0.21016	-0.01022	FALSE	4.64E-03
33492_at	-0.11008	-1.288487	0.042444	-0.3059	0.08574	TRUE	1.00E+00
35789_at	-0.11003	-1.288339	0.0374	-0.28258	0.06251	TRUE	1.00E+00
1210_s_at	-0.11001	-1.288279	0.040973	-0.29904	0.07902	TRUE	1.00E+00
34446_at	-0.10999	-1.28822	0.027164	-0.23532	0.01533	TRUE	6.49E-01
31858_at	-0.10999	-1.28822	0.027745	-0.23799	0.01802	TRUE	9.30E-01
38452_at	-0.10998	-1.28819	0.042767	-0.30728	0.08733	TRUE	1.00E+00
35456_r_at	-0.10997	-1.288161	0.074099	-0.45183	0.23189	TRUE	1.00E+00
31726_at	-0.10993	-1.288042	0.097874	-0.56148	0.34162	TRUE	1.00E+00
37436_at	-0.10982	-1.287716	0.090999	-0.52966	0.31001	TRUE	1.00E+00
38414_at	-0.10981	-1.287686	0.163467	-0.86398	0.64435	TRUE	1.00E+00
33545_at	-0.10978	-1.287597	0.033233	-0.2631	0.04355	TRUE	1.00E+00
41311_f_at	-0.10972	-1.287419	0.089833	-0.52417	0.30473	TRUE	1.00E+00
34489_f_at	-0.10962	-1.287123	0.033534	-0.26433	0.04509	TRUE	1.00E+00
31974_at	-0.10961	-1.287093	0.037352	-0.28194	0.06271	TRUE	1.00E+00
38426_at	-0.10955	-1.286915	0.027513	-0.23648	0.01739	TRUE	8.64E-01
33077_at	-0.10944	-1.286589	0.033071	-0.26202	0.04314	TRUE	1.00E+00
39564_s_at	-0.10938	-1.286412	0.097004	-0.55692	0.33816	TRUE	1.00E+00

160034_s_at	-0.10937	-1.286382	0.056825	-0.37154	0.1528	TRUE	1.00E+00
38420_at	-0.10935	-1.286323	0.022397	-0.21268	-0.00602	FALSE	1.32E-02
32145_at	-0.10934	-1.286293	0.014205	-0.17488	-0.04381	FALSE	1.75E-10
36823_at	-0.10934	-1.286293	0.067991	-0.42302	0.20434	TRUE	1.00E+00
34210_at	-0.10933	-1.286264	0.046607	-0.32436	0.10569	TRUE	1.00E+00
31608_g_at	-0.10933	-1.286264	0.040855	-0.29782	0.07916	TRUE	1.00E+00
40217_s_at	-0.10924	-1.285997	0.127083	-0.69554	0.47707	TRUE	1.00E+00
39833_at	-0.10918	-1.285819	0.070273	-0.43339	0.21503	TRUE	1.00E+00
1039_s_at	-0.10912	-1.285642	0.022493	-0.2129	-0.00535	FALSE	1.55E-02
41320_s_at	-0.10906	-1.285464	0.032349	-0.2583	0.04019	TRUE	1.00E+00
37300_at	-0.10904	-1.285405	0.032704	-0.25993	0.04184	TRUE	1.00E+00
1723_g_at	-0.109	-1.285287	0.045912	-0.32082	0.10282	TRUE	1.00E+00
33306_at	-0.1089	-1.284991	0.013912	-0.17308	-0.04472	FALSE	6.27E-11
39366_at	-0.10886	-1.284872	0.044123	-0.31242	0.0947	TRUE	1.00E+00
40202_at	-0.10882	-1.284754	0.03834	-0.2857	0.06807	TRUE	1.00E+00
38699_at	-0.1088	-1.284695	0.098144	-0.5616	0.344	TRUE	1.00E+00
34433_at	-0.10873	-1.284488	0.046493	-0.32323	0.10577	TRUE	1.00E+00
40024_at	-0.10871	-1.284429	0.036999	-0.27941	0.06199	TRUE	1.00E+00
37250_at	-0.1087	-1.284399	0.024036	-0.21959	0.00219	TRUE	7.72E-02
35278_at	-0.10868	-1.28434	0.026515	-0.23101	0.01365	TRUE	5.24E-01
1650_g_at	-0.10861	-1.284133	0.034972	-0.26996	0.05273	TRUE	1.00E+00
1124_at	-0.10853	-1.283896	0.025917	-0.2281	0.01104	TRUE	3.56E-01
34591_at	-0.10845	-1.28366	0.095542	-0.54924	0.33235	TRUE	1.00E+00
36333_at	-0.10839	-1.283483	0.026864	-0.23232	0.01555	TRUE	6.90E-01
39329_at	-0.10838	-1.283453	0.019449	-0.19811	-0.01865	FALSE	3.17E-04
38314_at	-0.10832	-1.283276	0.048098	-0.33023	0.11358	TRUE	1.00E+00
39899_at	-0.10829	-1.283187	0.02641	-0.23013	0.01356	TRUE	5.21E-01
36233_at	-0.10828	-1.283158	0.063719	-0.40226	0.18569	TRUE	1.00E+00
41166_at	-0.10822	-1.28298	0.064539	-0.40597	0.18954	TRUE	1.00E+00
1223_at	-0.10816	-1.282803	0.106393	-0.59901	0.38269	TRUE	1.00E+00
1937_at	-0.10815	-1.282774	0.037097	-0.27929	0.063	TRUE	1.00E+00
39902_at	-0.10812	-1.282685	0.03552	-0.27199	0.05575	TRUE	1.00E+00
40466_at	-0.10809	-1.282596	0.026532	-0.2305	0.01431	TRUE	5.83E-01
39881_at	-0.10805	-1.282478	0.065173	-0.40874	0.19263	TRUE	1.00E+00
33098_at	-0.10801	-1.28236	0.116074	-0.64353	0.4275	TRUE	1.00E+00
35671_at	-0.10799	-1.282301	0.016215	-0.1828	-0.03318	FALSE	3.46E-07
37557_at	-0.10791	-1.282065	0.026507	-0.2302	0.01439	TRUE	5.91E-01
1762_at	-0.10789	-1.282006	0.057049	-0.37109	0.15532	TRUE	1.00E+00
37262_at	-0.10783	-1.281829	0.042176	-0.30241	0.08675	TRUE	1.00E+00
32880_at	-0.10766	-1.281327	0.043801	-0.30974	0.09442	TRUE	1.00E+00
36493_at	-0.10764	-1.281268	0.035865	-0.2731	0.05783	TRUE	1.00E+00
33727_r_at	-0.10759	-1.281121	0.090227	-0.52386	0.30868	TRUE	1.00E+00
41609_at	-0.10752	-1.280914	0.033647	-0.26276	0.04771	TRUE	1.00E+00
39629_at	-0.10745	-1.280708	0.07389	-0.44835	0.23344	TRUE	1.00E+00
40901_at	-0.10745	-1.280708	0.027226	-0.23306	0.01816	TRUE	1.00E+00
34552_at	-0.10744	-1.280678	0.040537	-0.29446	0.07958	TRUE	1.00E+00
31716_at	-0.10731	-1.280295	0.012417	-0.1646	-0.05002	FALSE	6.95E-14
32226_at	-0.10722	-1.28003	0.037608	-0.28073	0.06629	TRUE	1.00E+00
1237_at	-0.10716	-1.279853	0.050115	-0.33837	0.12405	TRUE	1.00E+00
38865_at	-0.10713	-1.279764	0.046231	-0.32043	0.10616	TRUE	1.00E+00
894_g_at	-0.10704	-1.279499	0.025206	-0.22333	0.00926	TRUE	2.74E-01

32770_at	-0.10699	-1.279352	0.039645	-0.2899	0.07591	TRUE	1.00E+00
1253_at	-0.10694	-1.279205	0.02047	-0.20138	-0.0125	FALSE	2.21E-03
37967_at	-0.10692	-1.279146	0.079488	-0.47364	0.25981	TRUE	1.00E+00
40360_at	-0.10689	-1.279057	0.019137	-0.19518	-0.0186	FALSE	2.94E-04
40232_at	-0.10677	-1.278704	0.100259	-0.56932	0.35578	TRUE	1.00E+00
131_at	-0.10663	-1.278292	0.033085	-0.25926	0.04601	TRUE	1.00E+00
38900_at	-0.10658	-1.278145	0.029231	-0.24144	0.02828	TRUE	1.00E+00
38047_at	-0.10637	-1.277527	0.019717	-0.19734	-0.0154	FALSE	8.67E-04
41743_i_at	-0.10636	-1.277497	0.022149	-0.20855	-0.00418	FALSE	1.98E-02
41004_at	-0.10625	-1.277174	0.042696	-0.30323	0.09074	TRUE	1.00E+00
32420_at	-0.10619	-1.276997	0.038382	-0.28327	0.07089	TRUE	1.00E+00
41753_at	-0.10618	-1.276968	0.026309	-0.22756	0.0152	TRUE	6.86E-01
33345_at	-0.10618	-1.276968	0.050993	-0.34144	0.12908	TRUE	1.00E+00
34202_at	-0.10616	-1.276909	0.028639	-0.23828	0.02597	TRUE	1.00E+00
41593_at	-0.10613	-1.276821	0.054286	-0.35658	0.14433	TRUE	1.00E+00
31391_at	-0.10608	-1.276674	0.030718	-0.2478	0.03564	TRUE	1.00E+00
1794_at	-0.10608	-1.276674	0.041295	-0.2966	0.08444	TRUE	1.00E+00
966_at	-0.10607	-1.276645	0.024709	-0.22007	0.00793	TRUE	2.23E-01
39845_at	-0.10606	-1.276615	0.031118	-0.24962	0.0375	TRUE	1.00E+00
37594_at	-0.10605	-1.276586	0.047534	-0.32535	0.11326	TRUE	1.00E+00
740_at	-0.106	-1.276439	0.022315	-0.20895	-0.00304	FALSE	2.57E-02
35044_i_at	-0.10599	-1.276409	0.041097	-0.2956	0.08361	TRUE	1.00E+00
40948_at	-0.10594	-1.276262	0.025856	-0.22522	0.01335	TRUE	5.28E-01
689_at	-0.10583	-1.275939	0.024709	-0.21983	0.00817	TRUE	2.33E-01
36268_at	-0.10581	-1.275881	0.078671	-0.46877	0.25714	TRUE	1.00E+00
32592_at	-0.10571	-1.275587	0.026209	-0.22663	0.01521	TRUE	6.94E-01
35992_at	-0.1056	-1.275264	0.023894	-0.21584	0.00464	TRUE	1.25E-01
413_at	-0.10558	-1.275205	0.04486	-0.31255	0.10138	TRUE	1.00E+00
35221_at	-0.10558	-1.275205	0.024862	-0.22029	0.00912	TRUE	2.74E-01
35595_at	-0.10555	-1.275117	0.024023	-0.21638	0.00528	TRUE	1.41E-01
36130_f_at	-0.10554	-1.275088	0.016391	-0.18117	-0.02992	FALSE	1.52E-06
36710_at	-0.10552	-1.275029	0.057414	-0.3704	0.15937	TRUE	1.00E+00
37193_at	-0.10546	-1.274853	0.015979	-0.17918	-0.03174	FALSE	5.18E-07
32959_at	-0.10539	-1.274647	0.032869	-0.25703	0.04626	TRUE	1.00E+00
31784_at	-0.10536	-1.274559	0.030804	-0.24747	0.03676	TRUE	1.00E+00
38832_r_at	-0.10535	-1.27453	0.046355	-0.31921	0.10851	TRUE	1.00E+00
31705_at	-0.10535	-1.27453	0.017183	-0.18462	-0.02607	FALSE	1.10E-05
35397_at	-0.10527	-1.274295	0.126174	-0.68739	0.47684	TRUE	1.00E+00
37886_at	-0.10521	-1.274119	0.032483	-0.25507	0.04466	TRUE	1.00E+00
32006_r_at	-0.10507	-1.273708	0.146593	-0.78139	0.57125	TRUE	1.00E+00
31663_at	-0.10506	-1.273679	0.0511	-0.34081	0.1307	TRUE	1.00E+00
36282_at	-0.10504	-1.27362	0.081089	-0.47915	0.26907	TRUE	1.00E+00
36369_at	-0.10504	-1.27362	0.045601	-0.31542	0.10535	TRUE	1.00E+00
31840_at	-0.10503	-1.273591	0.024672	-0.21885	0.0088	TRUE	2.62E-01
33420_g_at	-0.10486	-1.273093	0.017716	-0.18659	-0.02312	FALSE	4.09E-05
33920_at	-0.10482	-1.272975	0.030117	-0.24377	0.03413	TRUE	1.00E+00
40573_s_at	-0.10477	-1.272829	0.154822	-0.81905	0.60952	TRUE	1.00E+00
2035_s_at	-0.10474	-1.272741	0.02299	-0.2108	0.00133	TRUE	6.59E-02
40044_at	-0.10473	-1.272712	0.060318	-0.38301	0.17356	TRUE	1.00E+00
39448_r_at	-0.1047	-1.272624	0.033019	-0.25704	0.04764	TRUE	1.00E+00
36419_at	-0.10457	-1.272243	0.067594	-0.41642	0.20729	TRUE	1.00E+00

34029_at	-0.10451	-1.272067	0.054328	-0.35516	0.14614	TRUE	1.00E+00
182_at	-0.10444	-1.271862	0.049938	-0.33483	0.12596	TRUE	1.00E+00
38232_f_at	-0.10432	-1.271511	0.020848	-0.2005	-0.00813	FALSE	7.10E-03
32936_at	-0.10429	-1.271423	0.032796	-0.2556	0.04702	TRUE	1.00E+00
39813_s_at	-0.10429	-1.271423	0.074711	-0.44898	0.2404	TRUE	1.00E+00
555_at	-0.10426	-1.271335	0.036388	-0.27214	0.06362	TRUE	1.00E+00
317_at	-0.10425	-1.271306	0.014722	-0.17217	-0.03633	FALSE	1.80E-08
39862_at	-0.10424	-1.271276	0.027704	-0.23206	0.02358	TRUE	1.00E+00
34005_at	-0.10421	-1.271189	0.018627	-0.19015	-0.01828	FALSE	2.79E-04
40363_r_at	-0.10419	-1.27113	0.105733	-0.59199	0.38362	TRUE	1.00E+00
34183_at	-0.10418	-1.271101	0.028842	-0.23724	0.02889	TRUE	1.00E+00
35203_at	-0.10418	-1.271101	0.027017	-0.22882	0.02047	TRUE	1.00E+00
2085_s_at	-0.10417	-1.271072	0.008987	-0.14563	-0.0627	FALSE	5.84E-27
40667_at	-0.1041	-1.270867	0.024241	-0.21594	0.00774	TRUE	2.21E-01
639_s_at	-0.10408	-1.270808	0.03844	-0.28143	0.07326	TRUE	1.00E+00
39624_at	-0.10407	-1.270779	0.075677	-0.45321	0.24508	TRUE	1.00E+00
31348_at	-0.10398	-1.270516	0.028336	-0.23471	0.02675	TRUE	1.00E+00
40969_at	-0.10382	-1.270048	0.086917	-0.50483	0.29718	TRUE	1.00E+00
37664_at	-0.10375	-1.269843	0.037533	-0.27691	0.06942	TRUE	1.00E+00
39607_at	-0.10373	-1.269784	0.028408	-0.23479	0.02734	TRUE	1.00E+00
38729_at	-0.10366	-1.26958	0.028383	-0.2346	0.02729	TRUE	1.00E+00
160038_s_at	-0.10363	-1.269492	0.135254	-0.72763	0.52038	TRUE	1.00E+00
1549_s_at	-0.10356	-1.269287	0.062571	-0.39224	0.18512	TRUE	1.00E+00
36656_at	-0.10355	-1.269258	0.06102	-0.38507	0.17797	TRUE	1.00E+00
685_f_at	-0.10353	-1.2692	0.032458	-0.25328	0.04622	TRUE	1.00E+00
32191_at	-0.10339	-1.268791	0.089172	-0.51479	0.30802	TRUE	1.00E+00
687_at	-0.10336	-1.268703	0.034911	-0.26443	0.0577	TRUE	1.00E+00
35997_g_at	-0.10333	-1.268615	0.022619	-0.20769	0.00102	TRUE	6.21E-02
36278_at	-0.10325	-1.268382	0.0319	-0.25043	0.04392	TRUE	1.00E+00
33273_f_at	-0.10324	-1.268353	0.048571	-0.32733	0.12084	TRUE	1.00E+00
751_at	-0.10324	-1.268353	0.017536	-0.18414	-0.02233	FALSE	4.96E-05
38552_f_at	-0.10313	-1.268031	0.03655	-0.27176	0.06549	TRUE	1.00E+00
1194_g_at	-0.10311	-1.267973	0.021422	-0.20194	-0.00428	FALSE	1.87E-02
38896_at	-0.1031	-1.267944	0.069068	-0.42175	0.21555	TRUE	1.00E+00
1196_at	-0.10306	-1.267827	0.038737	-0.28177	0.07566	TRUE	1.00E+00
39210_at	-0.10305	-1.267798	0.042414	-0.29873	0.09263	TRUE	1.00E+00
37126_at	-0.103	-1.267652	0.01446	-0.16972	-0.03629	FALSE	1.33E-08
33624_at	-0.10299	-1.267623	0.021632	-0.20279	-0.00319	FALSE	2.43E-02
31442_at	-0.10294	-1.267477	0.088581	-0.51162	0.30573	TRUE	1.00E+00
41643_at	-0.10287	-1.267272	0.13073	-0.70601	0.50026	TRUE	1.00E+00
33736_at	-0.10286	-1.267243	0.015364	-0.17374	-0.03198	FALSE	2.73E-07
39428_at	-0.10277	-1.266981	0.023826	-0.21269	0.00715	TRUE	2.03E-01
40806_at	-0.10275	-1.266922	0.042141	-0.29717	0.09167	TRUE	1.00E+00
36760_at	-0.10272	-1.266835	0.030122	-0.24169	0.03626	TRUE	1.00E+00
41797_at	-0.10268	-1.266718	0.057232	-0.36673	0.16136	TRUE	1.00E+00
32680_at	-0.10268	-1.266718	0.058159	-0.371	0.16565	TRUE	1.00E+00
38486_at	-0.10253	-1.266281	0.027705	-0.23034	0.02529	TRUE	1.00E+00
34463_at	-0.1025	-1.266193	0.022963	-0.20844	0.00344	TRUE	1.02E-01
34927_at	-0.10235	-1.265756	0.129158	-0.69823	0.49353	TRUE	1.00E+00
35588_at	-0.10229	-1.265581	0.044165	-0.30605	0.10147	TRUE	1.00E+00
35618_at	-0.10229	-1.265581	0.030441	-0.24273	0.03816	TRUE	1.00E+00

37808_at	-0.10225	-1.265465	0.020266	-0.19575	-0.00875	FALSE	5.71E-03
37291_r_at	-0.10225	-1.265465	0.183816	-0.9503	0.74581	TRUE	1.00E+00
709_at	-0.10221	-1.265348	0.048573	-0.32631	0.12189	TRUE	1.00E+00
33555_at	-0.10215	-1.265173	0.068076	-0.41622	0.21192	TRUE	1.00E+00
38541_at	-0.10206	-1.264911	0.037309	-0.27419	0.07007	TRUE	1.00E+00
40718_at	-0.10197	-1.264649	0.017103	-0.18088	-0.02307	FALSE	3.14E-05
39248_at	-0.10196	-1.26462	0.057161	-0.36568	0.16175	TRUE	1.00E+00
36759_at	-0.10195	-1.264591	0.03174	-0.24838	0.04449	TRUE	1.00E+00
31334_at	-0.10189	-1.264416	0.091397	-0.52356	0.31978	TRUE	1.00E+00
41785_at	-0.10186	-1.264329	0.027353	-0.22805	0.02434	TRUE	1.00E+00
33484_at	-0.10185	-1.2643	0.019337	-0.19106	-0.01264	FALSE	1.75E-03
36000_at	-0.10179	-1.264125	0.07287	-0.43798	0.2344	TRUE	1.00E+00
881_at	-0.10167	-1.263776	0.100559	-0.56561	0.36226	TRUE	1.00E+00
32175_at	-0.10163	-1.263659	0.035489	-0.26536	0.06211	TRUE	1.00E+00
37878_at	-0.1016	-1.263572	0.059322	-0.37529	0.17209	TRUE	1.00E+00
36765_at	-0.1016	-1.263572	0.021949	-0.20286	-0.00034	FALSE	4.64E-02
39991_at	-0.10152	-1.263339	0.051871	-0.34083	0.13779	TRUE	1.00E+00
37095_r_at	-0.10148	-1.263223	0.032563	-0.25171	0.04875	TRUE	1.00E+00
39145_at	-0.10146	-1.263165	0.018063	-0.1848	-0.01813	FALSE	2.45E-04
33866_at	-0.10142	-1.263048	0.033537	-0.25615	0.05331	TRUE	1.00E+00
32028_at	-0.10142	-1.263048	0.031852	-0.24837	0.04554	TRUE	1.00E+00
37822_at	-0.10141	-1.263019	0.038188	-0.27759	0.07477	TRUE	1.00E+00
32050_r_at	-0.10141	-1.263019	0.060697	-0.38144	0.17862	TRUE	1.00E+00
35472_at	-0.10133	-1.262787	0.117762	-0.64463	0.44198	TRUE	1.00E+00
1189_at	-0.10133	-1.262787	0.029347	-0.23672	0.03407	TRUE	1.00E+00
35589_at	-0.10132	-1.262758	0.053948	-0.35021	0.14758	TRUE	1.00E+00
32449_at	-0.10122	-1.262467	0.062084	-0.38765	0.1852	TRUE	1.00E+00
1718_at	-0.10119	-1.26238	0.027509	-0.2281	0.02573	TRUE	1.00E+00
41207_at	-0.10118	-1.262351	0.027384	-0.22752	0.02516	TRUE	1.00E+00
39966_at	-0.10114	-1.262234	0.039463	-0.2832	0.08093	TRUE	1.00E+00
1797_at	-0.10113	-1.262205	0.031554	-0.24671	0.04444	TRUE	1.00E+00
33592_at	-0.10113	-1.262205	0.089273	-0.513	0.31074	TRUE	1.00E+00
35128_at	-0.10098	-1.261769	0.113207	-0.62327	0.42131	TRUE	1.00E+00
537_f_at	-0.10095	-1.261682	0.058812	-0.37229	0.17038	TRUE	1.00E+00
38519_at	-0.1009	-1.261537	0.115837	-0.63532	0.43352	TRUE	1.00E+00
34362_at	-0.10083	-1.261334	0.069555	-0.42173	0.22007	TRUE	1.00E+00
33781_s_at	-0.10081	-1.261276	0.015452	-0.1721	-0.02952	FALSE	8.63E-07
41180_i_at	-0.10081	-1.261276	0.077924	-0.46032	0.2587	TRUE	1.00E+00
38516_at	-0.1008	-1.261247	0.018298	-0.18522	-0.01638	FALSE	4.56E-04
33607_at	-0.10076	-1.26113	0.017811	-0.18294	-0.01859	FALSE	1.94E-04
32636_f_at	-0.10073	-1.261043	0.046497	-0.31525	0.11379	TRUE	1.00E+00
401_s_at	-0.1007	-1.260956	0.033604	-0.25574	0.05433	TRUE	1.00E+00
38087_s_at	-0.1007	-1.260956	0.118825	-0.64891	0.44751	TRUE	1.00E+00
41071_at	-0.10069	-1.260927	0.060188	-0.37838	0.17699	TRUE	1.00E+00
32917_at	-0.10066	-1.26084	0.052483	-0.34279	0.14147	TRUE	1.00E+00
286_at	-0.10064	-1.260782	0.062287	-0.38801	0.18673	TRUE	1.00E+00
34143_at	-0.10061	-1.260695	0.0531	-0.34559	0.14437	TRUE	1.00E+00
37008_r_at	-0.10061	-1.260695	0.08813	-0.5072	0.30599	TRUE	1.00E+00
37504_at	-0.10059	-1.260637	0.036627	-0.26958	0.06839	TRUE	1.00E+00
40402_at	-0.1005	-1.260376	0.024254	-0.2124	0.0114	TRUE	4.32E-01
713_at	-0.10044	-1.260202	0.041762	-0.29312	0.09223	TRUE	1.00E+00

32811_at	-0.10036	-1.259969	0.03524	-0.26295	0.06222	TRUE	1.00E+00
33569_at	-0.10032	-1.259853	0.028805	-0.23321	0.03257	TRUE	1.00E+00
2075_s_at	-0.10028	-1.259737	0.023852	-0.21032	0.00977	TRUE	3.31E-01
38500_at	-0.10023	-1.259592	0.029628	-0.23692	0.03646	TRUE	1.00E+00
33822_at	-0.10015	-1.25936	0.035984	-0.26616	0.06587	TRUE	1.00E+00
405_at	-0.1001	-1.259215	0.080079	-0.46955	0.26935	TRUE	1.00E+00
33640_at	-0.10008	-1.259157	0.044269	-0.30432	0.10416	TRUE	1.00E+00
34894_r_at	-0.10003	-1.259012	0.05995	-0.37661	0.17656	TRUE	1.00E+00
38512_r_at	-0.10001	-1.258954	0.084301	-0.48894	0.28893	TRUE	1.00E+00
38445_at	-0.09987	-1.258549	0.014855	-0.16841	-0.03134	FALSE	2.24E-07
41402_at	-0.09979	-1.258317	0.103264	-0.5762	0.37663	TRUE	1.00E+00
36673_at	-0.09978	-1.258288	0.014563	-0.16697	-0.03259	FALSE	9.25E-08
41744_at	-0.09977	-1.258259	0.032787	-0.25104	0.05149	TRUE	1.00E+00
33424_at	-0.09976	-1.25823	0.014921	-0.1686	-0.03092	FALSE	2.90E-07
36377_at	-0.09973	-1.258143	0.094021	-0.53351	0.33404	TRUE	1.00E+00
41566_at	-0.09971	-1.258085	0.044271	-0.30396	0.10454	TRUE	1.00E+00
33671_f_at	-0.0997	-1.258056	0.080276	-0.47006	0.27065	TRUE	1.00E+00
38849_at	-0.0997	-1.258056	0.035713	-0.26446	0.06506	TRUE	1.00E+00
623_s_at	-0.09969	-1.258027	0.015386	-0.17068	-0.02871	FALSE	1.16E-06
31504_at	-0.09967	-1.257969	0.018733	-0.18609	-0.01324	FALSE	1.31E-03
37155_at	-0.09965	-1.257911	0.082957	-0.48238	0.28308	TRUE	1.00E+00
31895_at	-0.09964	-1.257882	0.05013	-0.33092	0.13164	TRUE	1.00E+00
35437_at	-0.09954	-1.257593	0.069171	-0.41867	0.21958	TRUE	1.00E+00
1057_at	-0.09952	-1.257535	0.053689	-0.34722	0.14818	TRUE	1.00E+00
1336_s_at	-0.09945	-1.257332	0.061899	-0.38502	0.18613	TRUE	1.00E+00
34107_at	-0.09944	-1.257303	0.067343	-0.41013	0.21126	TRUE	1.00E+00
489_at	-0.09943	-1.257274	0.023017	-0.20562	0.00676	TRUE	1.97E-01
35365_at	-0.09934	-1.257014	0.011754	-0.15356	-0.04511	FALSE	3.64E-13
33617_s_at	-0.09931	-1.256927	0.02219	-0.20169	0.00306	TRUE	9.62E-02
38934_at	-0.09928	-1.25684	0.104051	-0.57933	0.38077	TRUE	1.00E+00
37945_at	-0.09925	-1.256753	0.017068	-0.178	-0.0205	FALSE	7.66E-05
36569_at	-0.09915	-1.256464	0.060838	-0.37983	0.18153	TRUE	1.00E+00
36959_at	-0.09915	-1.256464	0.021321	-0.19751	-0.00078	FALSE	4.19E-02
35835_at	-0.09909	-1.25629	0.024415	-0.21173	0.01355	TRUE	6.23E-01
36812_at	-0.09908	-1.256261	0.02566	-0.21746	0.01931	TRUE	1.00E+00
31747_g_at	-0.09908	-1.256261	0.102631	-0.57258	0.37442	TRUE	1.00E+00
1480_at	-0.09905	-1.256175	0.040964	-0.28804	0.08994	TRUE	1.00E+00
31500_at	-0.09904	-1.256146	0.046857	-0.31522	0.11714	TRUE	1.00E+00
36255_at	-0.09895	-1.255885	0.123325	-0.66792	0.47002	TRUE	1.00E+00
33645_at	-0.09895	-1.255885	0.053528	-0.3459	0.14801	TRUE	1.00E+00
588_at	-0.09885	-1.255596	0.025759	-0.21769	0.01999	TRUE	1.00E+00
33132_at	-0.09883	-1.255538	0.030743	-0.24067	0.043	TRUE	1.00E+00
affx-biob-5_at	-0.09879	-1.255423	0.044384	-0.30356	0.10598	TRUE	1.00E+00
1802_s_at	-0.09874	-1.255278	0.078261	-0.4598	0.26233	TRUE	1.00E+00
133_at	-0.09871	-1.255192	0.043192	-0.29798	0.10056	TRUE	1.00E+00
41761_at	-0.09856	-1.254758	0.075542	-0.44708	0.24996	TRUE	1.00E+00
913_at	-0.09851	-1.254614	0.093067	-0.52788	0.33087	TRUE	1.00E+00
33012_at	-0.09849	-1.254556	0.077417	-0.45566	0.25868	TRUE	1.00E+00
32598_at	-0.09848	-1.254527	0.124267	-0.67179	0.47484	TRUE	1.00E+00
429_f_at	-0.09848	-1.254527	0.035424	-0.26191	0.06495	TRUE	1.00E+00
33879_at	-0.09847	-1.254498	0.020454	-0.19284	-0.00411	FALSE	1.86E-02

2080_s_at	-0.09847	-1.254498	0.02003	-0.19088	-0.00606	FALSE	1.11E-02
650_s_at	-0.09843	-1.254383	0.063482	-0.39131	0.19445	TRUE	1.00E+00
38573_at	-0.09841	-1.254325	0.023133	-0.20514	0.00831	TRUE	2.65E-01
36651_at	-0.09834	-1.254123	0.014913	-0.16714	-0.02954	FALSE	5.40E-07
affx-humgapdh/m	-0.0983	-1.254007	0.049591	-0.32709	0.1305	TRUE	1.00E+00
37813_at	-0.09826	-1.253892	0.041813	-0.29117	0.09465	TRUE	1.00E+00
34016_s_at	-0.09826	-1.253892	0.025953	-0.21799	0.02148	TRUE	1.00E+00
34230_r_at	-0.09818	-1.253661	0.028629	-0.23026	0.0339	TRUE	1.00E+00
36349_at	-0.09816	-1.253603	0.082423	-0.47843	0.2821	TRUE	1.00E+00
36137_at	-0.09809	-1.253401	0.013389	-0.15987	-0.03632	FALSE	2.99E-09
37625_at	-0.09808	-1.253372	0.076072	-0.44905	0.25289	TRUE	1.00E+00
36215_at	-0.09801	-1.25317	0.025232	-0.21442	0.0184	TRUE	1.00E+00
38835_at	-0.09799	-1.253112	0.0322	-0.24654	0.05057	TRUE	1.00E+00
40038_at	-0.09796	-1.253026	0.119949	-0.65136	0.45543	TRUE	1.00E+00
1507_s_at	-0.09795	-1.252997	0.061307	-0.38079	0.18489	TRUE	1.00E+00
33824_at	-0.09794	-1.252968	0.029199	-0.23265	0.03678	TRUE	1.00E+00
37654_at	-0.09785	-1.252708	0.015219	-0.16806	-0.02764	FALSE	1.61E-06
35505_at	-0.09775	-1.25242	0.060205	-0.37551	0.18002	TRUE	1.00E+00
32511_at	-0.09774	-1.252391	0.023788	-0.20749	0.012	TRUE	5.02E-01
41645_at	-0.09772	-1.252334	0.029613	-0.23434	0.0389	TRUE	1.00E+00
37939_at	-0.09768	-1.252218	0.029132	-0.23208	0.03672	TRUE	1.00E+00
affx-yel002c/wbp	-0.09759	-1.251959	0.112388	-0.61611	0.42092	TRUE	1.00E+00
33735_at	-0.09752	-1.251757	0.082066	-0.47614	0.28109	TRUE	1.00E+00
1771_s_at	-0.0975	-1.251699	0.025966	-0.2173	0.0223	TRUE	1.00E+00
31543_at	-0.09739	-1.251382	0.047448	-0.31629	0.12151	TRUE	1.00E+00
38641_at	-0.09738	-1.251353	0.021764	-0.19779	0.00303	TRUE	9.68E-02
37969_at	-0.09734	-1.251238	0.044607	-0.30314	0.10846	TRUE	1.00E+00
35243_at	-0.0973	-1.251123	0.036188	-0.26426	0.06966	TRUE	1.00E+00
34735_at	-0.09728	-1.251065	0.016492	-0.17337	-0.0212	FALSE	4.62E-05
34455_at	-0.09728	-1.251065	0.026324	-0.21873	0.02416	TRUE	1.00E+00
38169_s_at	-0.09724	-1.25095	0.032423	-0.24682	0.05235	TRUE	1.00E+00
540_at	-0.09719	-1.250806	0.015025	-0.16651	-0.02787	FALSE	1.25E-06
41812_s_at	-0.09719	-1.250806	0.05594	-0.35527	0.1609	TRUE	1.00E+00
39327_at	-0.09717	-1.250749	0.029439	-0.23299	0.03865	TRUE	1.00E+00
41413_at	-0.09716	-1.25072	0.039451	-0.27917	0.08486	TRUE	1.00E+00
39455_r_at	-0.09712	-1.250605	0.072168	-0.43007	0.23584	TRUE	1.00E+00
31350_at	-0.09698	-1.250201	0.036203	-0.26401	0.07004	TRUE	1.00E+00
31482_at	-0.09691	-1.25	0.033593	-0.25189	0.05808	TRUE	1.00E+00
39159_at	-0.09689	-1.249942	0.03475	-0.25721	0.06343	TRUE	1.00E+00
32128_at	-0.09685	-1.249827	0.174554	-0.90217	0.70847	TRUE	1.00E+00
179_at	-0.09677	-1.249597	0.030662	-0.23823	0.04469	TRUE	1.00E+00
39318_at	-0.09674	-1.249511	0.043612	-0.29794	0.10447	TRUE	1.00E+00
32698_at	-0.09671	-1.249424	0.060392	-0.37533	0.18192	TRUE	1.00E+00
37992_s_at	-0.09667	-1.249309	0.019624	-0.18721	-0.00614	FALSE	1.06E-02
41361_at	-0.09667	-1.249309	0.040979	-0.28573	0.0924	TRUE	1.00E+00
35335_at	-0.09666	-1.249281	0.037686	-0.27053	0.0772	TRUE	1.00E+00
33144_at	-0.09663	-1.249194	0.037517	-0.26972	0.07646	TRUE	1.00E+00
35037_at	-0.09663	-1.249194	0.03045	-0.23711	0.04386	TRUE	1.00E+00
31764_at	-0.09655	-1.248964	0.115241	-0.62822	0.43512	TRUE	1.00E+00
33996_at	-0.09644	-1.248648	0.044526	-0.30187	0.10898	TRUE	1.00E+00
32077_s_at	-0.09641	-1.248562	0.064675	-0.39479	0.20198	TRUE	1.00E+00

39267_at	-0.0964	-1.248533	0.02936	-0.23186	0.03905	TRUE	1.00E+00
40742_at	-0.09637	-1.248447	0.041431	-0.28752	0.09477	TRUE	1.00E+00
31358_at	-0.09633	-1.248332	0.161104	-0.83959	0.64694	TRUE	1.00E+00
318_at	-0.09632	-1.248303	0.048662	-0.32082	0.12819	TRUE	1.00E+00
38045_at	-0.09631	-1.248274	0.082507	-0.47696	0.28435	TRUE	1.00E+00
530_at	-0.09628	-1.248188	0.042766	-0.29359	0.10102	TRUE	1.00E+00
33271_r_at	-0.09628	-1.248188	0.063986	-0.39148	0.19893	TRUE	1.00E+00
37087_at	-0.09623	-1.248044	0.020156	-0.18922	-0.00324	FALSE	2.28E-02
38404_at	-0.09621	-1.247987	0.052247	-0.33726	0.14484	TRUE	1.00E+00
31523_f_at	-0.09616	-1.247843	0.056129	-0.35512	0.16279	TRUE	1.00E+00
31447_at	-0.09615	-1.247814	0.078622	-0.45888	0.26658	TRUE	1.00E+00
36300_at	-0.09615	-1.247814	0.070204	-0.42004	0.22775	TRUE	1.00E+00
36455_at	-0.09612	-1.247728	0.051321	-0.3329	0.14065	TRUE	1.00E+00
896_at	-0.09606	-1.247556	0.03495	-0.25731	0.06518	TRUE	1.00E+00
36154_at	-0.09605	-1.247527	0.025565	-0.214	0.02189	TRUE	1.00E+00
33696_at	-0.09605	-1.247527	0.066572	-0.40319	0.21108	TRUE	1.00E+00
40495_at	-0.09605	-1.247527	0.049175	-0.32292	0.13083	TRUE	1.00E+00
31687_f_at	-0.096	-1.247384	0.068198	-0.41063	0.21864	TRUE	1.00E+00
32189_g_at	-0.09587	-1.24701	0.016879	-0.17374	-0.018	FALSE	1.70E-04
34842_at	-0.09577	-1.246723	0.026745	-0.21916	0.02762	TRUE	1.00E+00
41252_s_at	-0.09567	-1.246436	0.090024	-0.51101	0.31966	TRUE	1.00E+00
40095_at	-0.09561	-1.246264	0.092054	-0.5203	0.32909	TRUE	1.00E+00
35001_at	-0.09559	-1.246206	0.061207	-0.37797	0.18679	TRUE	1.00E+00
791_g_at	-0.09557	-1.246149	0.029743	-0.23279	0.04165	TRUE	1.00E+00
40798_s_at	-0.09555	-1.246092	0.04219	-0.2902	0.09909	TRUE	1.00E+00
31827_s_at	-0.09554	-1.246063	0.033931	-0.25209	0.061	TRUE	1.00E+00
36726_at	-0.09552	-1.246006	0.043625	-0.29679	0.10575	TRUE	1.00E+00
35502_at	-0.09551	-1.245977	0.050223	-0.32722	0.13619	TRUE	1.00E+00
35930_at	-0.09551	-1.245977	0.072644	-0.43066	0.23965	TRUE	1.00E+00
1877_g_at	-0.0955	-1.245948	0.022221	-0.19802	0.00702	TRUE	2.18E-01
37355_at	-0.09549	-1.24592	0.050968	-0.33063	0.13966	TRUE	1.00E+00
37779_at	-0.09547	-1.245862	0.023848	-0.2055	0.01456	TRUE	7.89E-01
32281_at	-0.09532	-1.245432	0.036864	-0.2654	0.07476	TRUE	1.00E+00
34493_at	-0.09531	-1.245403	0.0511	-0.33107	0.14045	TRUE	1.00E+00
36123_at	-0.09531	-1.245403	0.021106	-0.19268	0.00207	TRUE	7.97E-02
34108_g_at	-0.09524	-1.245203	0.017294	-0.17503	-0.01545	FALSE	4.61E-04
38808_at	-0.09522	-1.245145	0.028488	-0.22665	0.03621	TRUE	1.00E+00
33999_f_at	-0.09507	-1.244715	0.084806	-0.48633	0.29619	TRUE	1.00E+00
34909_at	-0.09506	-1.244687	0.039557	-0.27755	0.08744	TRUE	1.00E+00
35568_at	-0.09504	-1.244629	0.083591	-0.48069	0.29062	TRUE	1.00E+00
35194_at	-0.09501	-1.244543	0.068473	-0.41092	0.22089	TRUE	1.00E+00
38710_at	-0.09498	-1.244457	0.014907	-0.16376	-0.0262	FALSE	2.36E-06
34507_s_at	-0.09495	-1.244371	0.070026	-0.41802	0.22812	TRUE	1.00E+00
31381_at	-0.09495	-1.244371	0.037779	-0.26924	0.07935	TRUE	1.00E+00
38281_at	-0.09493	-1.244314	0.023897	-0.20518	0.01533	TRUE	8.99E-01
34976_at	-0.09488	-1.244171	0.075877	-0.44495	0.25518	TRUE	1.00E+00
33987_at	-0.09484	-1.244056	0.010912	-0.14519	-0.0445	FALSE	4.51E-14
37077_at	-0.09482	-1.243999	0.088095	-0.50125	0.31161	TRUE	1.00E+00
34838_at	-0.09478	-1.243884	0.112445	-0.61355	0.424	TRUE	1.00E+00
35747_at	-0.09477	-1.243856	0.015195	-0.16487	-0.02466	FALSE	5.64E-06
32190_at	-0.09474	-1.24377	0.05797	-0.36219	0.1727	TRUE	1.00E+00

40643_at	-0.0947	-1.243655	0.0312	-0.23864	0.04925	TRUE	1.00E+00
36659_at	-0.09468	-1.243598	0.032062	-0.2426	0.05323	TRUE	1.00E+00
33193_at	-0.09465	-1.243512	0.019643	-0.18527	-0.00402	FALSE	1.83E-02
35893_s_at	-0.09455	-1.243226	0.090027	-0.50989	0.3208	TRUE	1.00E+00
40215_at	-0.09452	-1.24314	0.04538	-0.30388	0.11485	TRUE	1.00E+00
31642_at	-0.09451	-1.243111	0.064367	-0.39147	0.20245	TRUE	1.00E+00
31822_at	-0.0945	-1.243083	0.036885	-0.26467	0.07567	TRUE	1.00E+00
33331_at	-0.09448	-1.243025	0.106777	-0.5871	0.39814	TRUE	1.00E+00
38153_at	-0.0944	-1.242796	0.030217	-0.23381	0.045	TRUE	1.00E+00
39978_at	-0.09422	-1.242281	0.152175	-0.79629	0.60786	TRUE	1.00E+00
38003_s_at	-0.09407	-1.241852	0.016492	-0.17016	-0.01798	FALSE	1.48E-04
39045_at	-0.09403	-1.241738	0.010551	-0.14271	-0.04535	FALSE	6.36E-15
37576_at	-0.09401	-1.241681	0.121045	-0.65246	0.46444	TRUE	1.00E+00
2031_s_at	-0.09397	-1.241567	0.026011	-0.21397	0.02604	TRUE	1.00E+00
1339_s_at	-0.09393	-1.241452	0.055702	-0.35092	0.16305	TRUE	1.00E+00
616_s_at	-0.09393	-1.241452	0.089564	-0.50714	0.31928	TRUE	1.00E+00
36500_at	-0.09391	-1.241395	0.018497	-0.17925	-0.00857	FALSE	4.84E-03
31939_at	-0.09387	-1.241281	0.058242	-0.36257	0.17484	TRUE	1.00E+00
39363_at	-0.09386	-1.241252	0.017144	-0.17295	-0.01476	FALSE	5.54E-04
34860_g_at	-0.09377	-1.240995	0.014231	-0.15943	-0.02811	FALSE	5.59E-07
36133_at	-0.09369	-1.240766	0.034066	-0.25085	0.06348	TRUE	1.00E+00
38853_at	-0.09365	-1.240652	0.089727	-0.50762	0.32031	TRUE	1.00E+00
41222_at	-0.09363	-1.240595	0.032098	-0.24172	0.05445	TRUE	1.00E+00
886_at	-0.09361	-1.240538	0.044863	-0.30059	0.11337	TRUE	1.00E+00
36714_at	-0.0936	-1.240509	0.116938	-0.63311	0.4459	TRUE	1.00E+00
39609_at	-0.09354	-1.240338	0.04204	-0.28749	0.10042	TRUE	1.00E+00
36542_at	-0.09353	-1.240309	0.013419	-0.15544	-0.03162	FALSE	3.99E-08
38052_at	-0.09349	-1.240195	0.047297	-0.3117	0.12472	TRUE	1.00E+00
34962_at	-0.09343	-1.240024	0.043083	-0.2922	0.10533	TRUE	1.00E+00
929_at	-0.0933	-1.239653	0.029821	-0.23089	0.04428	TRUE	1.00E+00
33500_i_at	-0.0933	-1.239653	0.018819	-0.18012	-0.00647	FALSE	9.01E-03
39228_at	-0.09325	-1.23951	0.026237	-0.2143	0.0278	TRUE	1.00E+00
493_at	-0.09325	-1.23951	0.017839	-0.17555	-0.01095	FALSE	2.17E-03
33818_at	-0.0932	-1.239367	0.028349	-0.22399	0.03759	TRUE	1.00E+00
37062_at	-0.09303	-1.238882	0.134639	-0.7142	0.52814	TRUE	1.00E+00
32217_at	-0.09302	-1.238854	0.024316	-0.2052	0.01917	TRUE	1.00E+00
41097_at	-0.09301	-1.238825	0.018554	-0.17861	-0.00741	FALSE	6.76E-03
40544_g_at	-0.09296	-1.238682	0.025476	-0.2105	0.02457	TRUE	1.00E+00
41575_at	-0.09296	-1.238682	0.041356	-0.28376	0.09784	TRUE	1.00E+00
38525_at	-0.09294	-1.238625	0.070309	-0.41731	0.23144	TRUE	1.00E+00
32271_at	-0.09293	-1.238597	0.017387	-0.17314	-0.01271	FALSE	1.14E-03
32451_at	-0.09292	-1.238568	0.067499	-0.40433	0.21849	TRUE	1.00E+00
36298_at	-0.09283	-1.238312	0.037527	-0.26596	0.08031	TRUE	1.00E+00
affx-hsac07/x003	-0.09282	-1.238283	0.060378	-0.37138	0.18574	TRUE	1.00E+00
34093_at	-0.09279	-1.238198	0.020119	-0.18561	0.00003	TRUE	5.04E-02
37470_at	-0.09266	-1.237827	0.045921	-0.30452	0.1192	TRUE	1.00E+00
39388_at	-0.09263	-1.237742	0.03716	-0.26408	0.07881	TRUE	1.00E+00
35566_f_at	-0.09263	-1.237742	0.020563	-0.1875	0.00224	TRUE	8.40E-02
39043_at	-0.09249	-1.237343	0.039524	-0.27484	0.08986	TRUE	1.00E+00
1799_at	-0.09248	-1.237314	0.030978	-0.2354	0.05044	TRUE	1.00E+00
39521_at	-0.09243	-1.237172	0.025958	-0.21219	0.02733	TRUE	1.00E+00

1193_at	-0.09243	-1.237172	0.057261	-0.35661	0.17175	TRUE	1.00E+00
37744_r_at	-0.0924	-1.237086	0.040006	-0.27697	0.09217	TRUE	1.00E+00
33856_at	-0.09233	-1.236887	0.011932	-0.14739	-0.03728	FALSE	1.27E-10
31499_s_at	-0.0923	-1.236801	0.117977	-0.6366	0.452	TRUE	1.00E+00
41138_at	-0.09229	-1.236773	0.028978	-0.22598	0.04141	TRUE	1.00E+00
739_at	-0.09219	-1.236488	0.079156	-0.45739	0.273	TRUE	1.00E+00
1409_at	-0.09219	-1.236488	0.031692	-0.2384	0.05402	TRUE	1.00E+00
34102_at	-0.09194	-1.235777	0.063566	-0.38521	0.20132	TRUE	1.00E+00
253_g_at	-0.09187	-1.235578	0.054233	-0.34208	0.15833	TRUE	1.00E+00
34533_at	-0.09185	-1.235521	0.014101	-0.15691	-0.0268	FALSE	9.24E-07
36301_at	-0.09179	-1.23535	0.025552	-0.20968	0.0261	TRUE	1.00E+00
36625_at	-0.09176	-1.235265	0.013538	-0.15422	-0.0293	FALSE	1.54E-07
32637_r_at	-0.0917	-1.235094	0.07895	-0.45594	0.27254	TRUE	1.00E+00
35162_s_at	-0.09167	-1.235009	0.04903	-0.31788	0.13453	TRUE	1.00E+00
38916_at	-0.09167	-1.235009	0.040518	-0.2786	0.09527	TRUE	1.00E+00
33863_at	-0.09163	-1.234895	0.024716	-0.20566	0.0224	TRUE	1.00E+00
33860_at	-0.09163	-1.234895	0.029585	-0.22812	0.04486	TRUE	1.00E+00
37551_at	-0.09161	-1.234838	0.016402	-0.16729	-0.01594	FALSE	2.94E-04
36064_at	-0.0915	-1.234525	0.031758	-0.23802	0.05501	TRUE	1.00E+00
38018_g_at	-0.09147	-1.23444	0.061915	-0.37712	0.19418	TRUE	1.00E+00
36512_at	-0.09138	-1.234184	0.074282	-0.43409	0.25132	TRUE	1.00E+00
37946_at	-0.09127	-1.233872	0.036604	-0.26014	0.0776	TRUE	1.00E+00
35271_at	-0.09127	-1.233872	0.02649	-0.21348	0.03095	TRUE	1.00E+00
39278_at	-0.09116	-1.233559	0.038202	-0.26741	0.08508	TRUE	1.00E+00
34122_at	-0.09114	-1.233502	0.043648	-0.29252	0.11023	TRUE	1.00E+00
39594_f_at	-0.09113	-1.233474	0.03724	-0.26294	0.08068	TRUE	1.00E+00
35711_at	-0.09112	-1.233446	0.05099	-0.32637	0.14412	TRUE	1.00E+00
1337_s_at	-0.09112	-1.233446	0.057365	-0.35577	0.17354	TRUE	1.00E+00
34812_at	-0.09112	-1.233446	0.029793	-0.22857	0.04634	TRUE	1.00E+00
37799_at	-0.09106	-1.233275	0.080334	-0.46168	0.27957	TRUE	1.00E+00
34581_s_at	-0.09105	-1.233247	0.059607	-0.36606	0.18395	TRUE	1.00E+00
41237_at	-0.09103	-1.23319	0.045564	-0.30125	0.11918	TRUE	1.00E+00
40347_at	-0.09086	-1.232707	0.03855	-0.26871	0.087	TRUE	1.00E+00
41310_f_at	-0.09082	-1.232594	0.01815	-0.17456	-0.00709	FALSE	7.09E-03
844_at	-0.0908	-1.232537	0.045948	-0.30278	0.12119	TRUE	1.00E+00
33758_f_at	-0.09077	-1.232452	0.04829	-0.31356	0.13202	TRUE	1.00E+00
33638_at	-0.09071	-1.232282	0.053298	-0.33661	0.15518	TRUE	1.00E+00
40873_at	-0.09068	-1.232197	0.03139	-0.23551	0.05414	TRUE	1.00E+00
2006_at	-0.09067	-1.232168	0.02548	-0.20823	0.02688	TRUE	1.00E+00
36499_at	-0.09066	-1.23214	0.045707	-0.30154	0.12021	TRUE	1.00E+00
609_f_at	-0.09061	-1.231998	0.016327	-0.16593	-0.01528	FALSE	3.61E-04
41126_at	-0.09061	-1.231998	0.039295	-0.2719	0.09068	TRUE	1.00E+00
1028_at	-0.09058	-1.231913	0.044351	-0.29519	0.11404	TRUE	1.00E+00
41487_at	-0.09057	-1.231885	0.049184	-0.31749	0.13634	TRUE	1.00E+00
35620_at	-0.09054	-1.231799	0.04018	-0.27592	0.09483	TRUE	1.00E+00
858_at	-0.09053	-1.231771	0.049292	-0.31795	0.13688	TRUE	1.00E+00
36212_at	-0.09049	-1.231658	0.124534	-0.66504	0.48406	TRUE	1.00E+00
32762_i_at	-0.09047	-1.231601	0.025098	-0.20626	0.02532	TRUE	1.00E+00
34869_at	-0.09046	-1.231573	0.039064	-0.27068	0.08977	TRUE	1.00E+00
982_at	-0.09041	-1.231431	0.036677	-0.25963	0.0788	TRUE	1.00E+00
35826_at	-0.09034	-1.231232	0.04185	-0.28342	0.10274	TRUE	1.00E+00

40100_at	-0.09032	-1.231176	0.021505	-0.18954	0.00889	TRUE	3.37E-01
37702_at	-0.09018	-1.230779	0.07704	-0.44561	0.26525	TRUE	1.00E+00
33603_at	-0.09015	-1.230694	0.122097	-0.65346	0.47315	TRUE	1.00E+00
591_s_at	-0.09014	-1.230665	0.064631	-0.38832	0.20804	TRUE	1.00E+00
41141_at	-0.0901	-1.230552	0.048445	-0.3136	0.13341	TRUE	1.00E+00
39958_at	-0.09002	-1.230325	0.022341	-0.19309	0.01305	TRUE	7.06E-01
31602_at	-0.09002	-1.230325	0.043285	-0.28972	0.10969	TRUE	1.00E+00
1807_g_at	-0.08995	-1.230127	0.136395	-0.71921	0.53932	TRUE	1.00E+00
38618_at	-0.08994	-1.230099	0.051844	-0.32913	0.14924	TRUE	1.00E+00
34410_at	-0.0899	-1.229986	0.051963	-0.32963	0.14984	TRUE	1.00E+00
694_at	-0.08979	-1.229674	0.049183	-0.3167	0.13712	TRUE	1.00E+00
32625_at	-0.08972	-1.229476	0.027514	-0.21666	0.03722	TRUE	1.00E+00
35830_at	-0.08972	-1.229476	0.018959	-0.17719	-0.00224	FALSE	2.81E-02
32302_g_at	-0.08971	-1.229448	0.063004	-0.38039	0.20096	TRUE	1.00E+00
32954_at	-0.08967	-1.229334	0.032064	-0.23759	0.05826	TRUE	1.00E+00
39080_at	-0.08966	-1.229306	0.031062	-0.23297	0.05365	TRUE	1.00E+00
affx-lysx-3_at	-0.08961	-1.229164	0.06196	-0.37547	0.19624	TRUE	1.00E+00
38186_g_at	-0.0896	-1.229136	0.063497	-0.38254	0.20335	TRUE	1.00E+00
32179_s_at	-0.08958	-1.22908	0.014201	-0.1551	-0.02406	FALSE	3.57E-06
33185_at	-0.08949	-1.228825	0.075936	-0.43983	0.26084	TRUE	1.00E+00
38984_at	-0.08949	-1.228825	0.033348	-0.24335	0.06436	TRUE	1.00E+00
1894_f_at	-0.08949	-1.228825	0.042492	-0.28553	0.10655	TRUE	1.00E+00
36796_at	-0.08946	-1.22874	0.024169	-0.20096	0.02205	TRUE	1.00E+00
35079_at	-0.08944	-1.228683	0.043068	-0.28813	0.10926	TRUE	1.00E+00
32709_at	-0.08943	-1.228655	0.047685	-0.30942	0.13057	TRUE	1.00E+00
39093_s_at	-0.0893	-1.228287	0.022509	-0.19315	0.01455	TRUE	9.17E-01
40061_at	-0.08927	-1.228203	0.027561	-0.21642	0.03789	TRUE	1.00E+00
38451_at	-0.08922	-1.228061	0.011915	-0.1442	-0.03425	FALSE	8.81E-10
37972_at	-0.08921	-1.228033	0.020761	-0.185	0.00657	TRUE	2.18E-01
35445_at	-0.08921	-1.228033	0.044704	-0.29546	0.11703	TRUE	1.00E+00
471_f_at	-0.08914	-1.227835	0.020093	-0.18184	0.00356	TRUE	1.16E-01
33679_f_at	-0.08912	-1.227778	0.024372	-0.20156	0.02332	TRUE	1.00E+00
37368_at	-0.0891	-1.227722	0.021086	-0.18638	0.00818	TRUE	3.01E-01
31844_at	-0.08908	-1.227665	0.098434	-0.54322	0.36505	TRUE	1.00E+00
1742_at	-0.08908	-1.227665	0.032946	-0.24108	0.06292	TRUE	1.00E+00
40788_at	-0.08901	-1.227467	0.021876	-0.18994	0.01192	TRUE	5.97E-01
39359_s_at	-0.08898	-1.227383	0.017539	-0.1699	-0.00807	FALSE	4.93E-03
720_at	-0.0889	-1.227157	0.018281	-0.17324	-0.00456	FALSE	1.46E-02
1081_at	-0.0889	-1.227157	0.022094	-0.19083	0.01304	TRUE	7.24E-01
38955_at	-0.08873	-1.226676	0.043616	-0.28996	0.11249	TRUE	1.00E+00
35119_at	-0.0887	-1.226592	0.021911	-0.18979	0.0124	TRUE	6.52E-01
35749_at	-0.08868	-1.226535	0.026888	-0.21274	0.03537	TRUE	1.00E+00
36766_at	-0.08866	-1.226479	0.041707	-0.28108	0.10377	TRUE	1.00E+00
35478_at	-0.08864	-1.226422	0.06079	-0.3691	0.19182	TRUE	1.00E+00
39510_r_at	-0.08859	-1.226281	0.025136	-0.20455	0.02738	TRUE	1.00E+00
affx-biodn-5_at	-0.08855	-1.226168	0.067	-0.39766	0.22057	TRUE	1.00E+00
480_at	-0.08854	-1.22614	0.046337	-0.30232	0.12524	TRUE	1.00E+00
37688_f_at	-0.08848	-1.22597	0.183172	-0.93356	0.7566	TRUE	1.00E+00
35023_at	-0.08827	-1.225378	0.067889	-0.40148	0.22494	TRUE	1.00E+00
34556_at	-0.08827	-1.225378	0.078916	-0.45235	0.27582	TRUE	1.00E+00
230_s_at	-0.08825	-1.225321	0.049442	-0.31635	0.13985	TRUE	1.00E+00

40944_at	-0.08821	-1.225208	0.039395	-0.26997	0.09354	TRUE	1.00E+00
39317_at	-0.08817	-1.225096	0.064023	-0.38355	0.2072	TRUE	1.00E+00
35098_at	-0.08812	-1.224955	0.039523	-0.27046	0.09422	TRUE	1.00E+00
39547_at	-0.08807	-1.224814	0.047286	-0.30623	0.13009	TRUE	1.00E+00
39843_at	-0.08807	-1.224814	0.113326	-0.6109	0.43477	TRUE	1.00E+00
35336_at	-0.08793	-1.224419	0.006715	-0.11891	-0.05694	FALSE	4.54E-35
34066_at	-0.08782	-1.224109	0.020223	-0.18112	0.00548	TRUE	1.78E-01
40849_s_at	-0.08777	-1.223968	0.021813	-0.1884	0.01286	TRUE	7.23E-01
31434_at	-0.08773	-1.223855	0.052254	-0.32881	0.15335	TRUE	1.00E+00
32969_r_at	-0.08772	-1.223827	0.028271	-0.21815	0.04271	TRUE	1.00E+00
32718_at	-0.08769	-1.223742	0.032464	-0.23747	0.06208	TRUE	1.00E+00
33491_at	-0.08768	-1.223714	0.165856	-0.85287	0.67751	TRUE	1.00E+00
33397_at	-0.08763	-1.223573	0.008359	-0.1262	-0.04907	FALSE	1.30E-21
38543_at	-0.08759	-1.223461	0.032774	-0.2388	0.06361	TRUE	1.00E+00
32046_at	-0.08758	-1.223432	0.042733	-0.28473	0.10958	TRUE	1.00E+00
36405_s_at	-0.08757	-1.223404	0.050383	-0.32002	0.14487	TRUE	1.00E+00
40787_at	-0.08751	-1.223235	0.020016	-0.17985	0.00484	TRUE	1.56E-01
41825_at	-0.08749	-1.223179	0.033538	-0.24222	0.06724	TRUE	1.00E+00
37475_at	-0.08749	-1.223179	0.020898	-0.18391	0.00892	TRUE	3.57E-01
40430_at	-0.08743	-1.22301	0.047819	-0.30805	0.13318	TRUE	1.00E+00
1353_g_at	-0.08743	-1.22301	0.0196	-0.17786	0.003	TRUE	1.03E-01
1403_s_at	-0.0874	-1.222925	0.039384	-0.2691	0.0943	TRUE	1.00E+00
41469_at	-0.08716	-1.22225	0.035825	-0.25245	0.07812	TRUE	1.00E+00
41143_at	-0.08714	-1.222194	0.029208	-0.22189	0.04762	TRUE	1.00E+00
518_at	-0.08713	-1.222165	0.015713	-0.15962	-0.01463	FALSE	3.71E-04
244_at	-0.08711	-1.222109	0.034046	-0.24419	0.06996	TRUE	1.00E+00
1273_r_at	-0.08703	-1.221884	0.030459	-0.22755	0.0535	TRUE	1.00E+00
41689_at	-0.08703	-1.221884	0.027838	-0.21546	0.04141	TRUE	1.00E+00
39104_at	-0.087	-1.2218	0.06969	-0.40852	0.23453	TRUE	1.00E+00
34413_at	-0.08691	-1.221546	0.009852	-0.13236	-0.04146	FALSE	1.42E-14
36334_at	-0.08688	-1.221462	0.034391	-0.24555	0.07179	TRUE	1.00E+00
35316_at	-0.08687	-1.221434	0.016083	-0.16108	-0.01267	FALSE	8.34E-04
39229_at	-0.08687	-1.221434	0.036519	-0.25535	0.08161	TRUE	1.00E+00
36790_at	-0.08686	-1.221406	0.0248	-0.20128	0.02755	TRUE	1.00E+00
37122_at	-0.08683	-1.221321	0.030271	-0.22649	0.05282	TRUE	1.00E+00
2032_s_at	-0.08681	-1.221265	0.029203	-0.22154	0.04792	TRUE	1.00E+00
37125_f_at	-0.0868	-1.221237	0.081265	-0.46173	0.28812	TRUE	1.00E+00
32286_at	-0.08676	-1.221125	0.019659	-0.17746	0.00394	TRUE	1.29E-01
33833_at	-0.08676	-1.221125	0.023096	-0.19331	0.0198	TRUE	1.00E+00
33874_at	-0.08675	-1.221097	0.048017	-0.30828	0.13479	TRUE	1.00E+00
32984_s_at	-0.08675	-1.221097	0.051808	-0.32577	0.15228	TRUE	1.00E+00
243_g_at	-0.08665	-1.220815	0.020284	-0.18023	0.00693	TRUE	2.45E-01
824_at	-0.08659	-1.220647	0.040051	-0.27137	0.09818	TRUE	1.00E+00
33804_at	-0.08659	-1.220647	0.070731	-0.41291	0.23973	TRUE	1.00E+00
1786_at	-0.08659	-1.220647	0.045919	-0.29844	0.12526	TRUE	1.00E+00
31424_at	-0.08652	-1.22045	0.02765	-0.21409	0.04104	TRUE	1.00E+00
34151_at	-0.08645	-1.220253	0.023169	-0.19334	0.02044	TRUE	1.00E+00
36338_at	-0.08637	-1.220029	0.016719	-0.1635	-0.00923	FALSE	3.02E-03
41592_at	-0.08635	-1.219972	0.084303	-0.47529	0.30259	TRUE	1.00E+00
1413_at	-0.08634	-1.219944	0.066044	-0.39104	0.21836	TRUE	1.00E+00
1275_at	-0.08628	-1.219776	0.018614	-0.17216	-0.0004	FALSE	4.50E-02

34204_at	-0.08625	-1.219692	0.049548	-0.31484	0.14235	TRUE	1.00E+00
40256_at	-0.08624	-1.219663	0.038002	-0.26156	0.08909	TRUE	1.00E+00
41852_at	-0.08617	-1.219467	0.025708	-0.20477	0.03244	TRUE	1.00E+00
36902_at	-0.08612	-1.219326	0.01363	-0.14901	-0.02324	FALSE	3.33E-06
1158_s_at	-0.08611	-1.219298	0.049181	-0.31301	0.14079	TRUE	1.00E+00
1579_at	-0.0861	-1.21927	0.033139	-0.23899	0.06679	TRUE	1.00E+00
34574_at	-0.08609	-1.219242	0.031953	-0.23351	0.06132	TRUE	1.00E+00
32875_at	-0.08606	-1.219158	0.016938	-0.16421	-0.00792	FALSE	4.74E-03
40468_at	-0.08605	-1.21913	0.035726	-0.25088	0.07878	TRUE	1.00E+00
105_at	-0.08602	-1.219046	0.083311	-0.47039	0.29834	TRUE	1.00E+00
38944_at	-0.08597	-1.218905	0.032808	-0.23733	0.06539	TRUE	1.00E+00
355_s_at	-0.08596	-1.218877	0.032024	-0.23371	0.06179	TRUE	1.00E+00
38714_at	-0.08588	-1.218653	0.037239	-0.25769	0.08592	TRUE	1.00E+00
38555_at	-0.08586	-1.218597	0.064951	-0.38551	0.2138	TRUE	1.00E+00
701_s_at	-0.08585	-1.218569	0.02826	-0.21623	0.04453	TRUE	1.00E+00
364_s_at	-0.08584	-1.218541	0.051923	-0.32539	0.1537	TRUE	1.00E+00
794_at	-0.08581	-1.218456	0.058202	-0.35433	0.18271	TRUE	1.00E+00
38995_at	-0.08579	-1.2184	0.014079	-0.15075	-0.02084	FALSE	1.39E-05
34664_at	-0.08576	-1.218316	0.066851	-0.39418	0.22267	TRUE	1.00E+00
39076_s_at	-0.08565	-1.218008	0.034564	-0.24512	0.07381	TRUE	1.00E+00
1904_at	-0.08563	-1.217952	0.022995	-0.19172	0.02046	TRUE	1.00E+00
2020_at	-0.08562	-1.217923	0.027862	-0.21416	0.04293	TRUE	1.00E+00
1398_g_at	-0.08561	-1.217895	0.024521	-0.19874	0.02752	TRUE	1.00E+00
35884_at	-0.08557	-1.217783	0.049512	-0.31399	0.14286	TRUE	1.00E+00
399_at	-0.08547	-1.217503	0.039022	-0.2655	0.09457	TRUE	1.00E+00
38899_s_at	-0.08546	-1.217475	0.015774	-0.15824	-0.01268	FALSE	7.63E-04
38518_at	-0.08545	-1.217447	0.041667	-0.27768	0.10679	TRUE	1.00E+00
1768_s_at	-0.08536	-1.217195	0.01796	-0.16822	-0.0025	FALSE	2.54E-02
181_g_at	-0.08535	-1.217167	0.046234	-0.29865	0.12796	TRUE	1.00E+00
32372_at	-0.08533	-1.21711	0.024682	-0.19921	0.02854	TRUE	1.00E+00
39291_at	-0.0853	-1.217026	0.078044	-0.44536	0.27476	TRUE	1.00E+00
1572_s_at	-0.08527	-1.216942	0.134617	-0.70634	0.5358	TRUE	1.00E+00
36256_at	-0.0852	-1.216746	0.060288	-0.36334	0.19294	TRUE	1.00E+00
40855_at	-0.08516	-1.216634	0.052248	-0.3262	0.15589	TRUE	1.00E+00
31446_s_at	-0.08513	-1.21655	0.041929	-0.27857	0.10832	TRUE	1.00E+00
37784_at	-0.08511	-1.216494	0.018147	-0.16883	-0.00139	FALSE	3.45E-02
31629_at	-0.0851	-1.216466	0.041717	-0.27757	0.10736	TRUE	1.00E+00
40137_at	-0.08501	-1.216214	0.008882	-0.12599	-0.04403	FALSE	1.34E-17
35428_g_at	-0.08498	-1.21613	0.112978	-0.60621	0.43626	TRUE	1.00E+00
1235_at	-0.08497	-1.216102	0.01379	-0.14859	-0.02134	FALSE	9.11E-06
37380_at	-0.08476	-1.215514	0.061606	-0.36898	0.19946	TRUE	1.00E+00
36785_at	-0.08474	-1.215458	0.028798	-0.2176	0.04813	TRUE	1.00E+00
37505_at	-0.08472	-1.215402	0.018513	-0.17013	0.00069	TRUE	5.97E-02
1813_at	-0.08469	-1.215318	0.036239	-0.25188	0.0825	TRUE	1.00E+00
33854_at	-0.08461	-1.215094	0.015585	-0.15651	-0.01271	FALSE	7.16E-04
33091_at	-0.0845	-1.214787	0.023941	-0.19496	0.02595	TRUE	1.00E+00
39855_at	-0.08447	-1.214703	0.019061	-0.1724	0.00347	TRUE	1.18E-01
1612_s_at	-0.08446	-1.214675	0.01426	-0.15025	-0.01867	FALSE	4.00E-05
38939_r_at	-0.08444	-1.214619	0.120401	-0.63992	0.47104	TRUE	1.00E+00
34371_at	-0.08442	-1.214563	0.029426	-0.22018	0.05133	TRUE	1.00E+00
35550_at	-0.0844	-1.214507	0.076393	-0.43685	0.26804	TRUE	1.00E+00

35634_at	-0.08435	-1.214367	0.017983	-0.16731	-0.00138	FALSE	3.44E-02
36062_at	-0.08434	-1.214339	0.012799	-0.14339	-0.0253	FALSE	5.55E-07
34522_at	-0.08434	-1.214339	0.092571	-0.51143	0.34274	TRUE	1.00E+00
32562_at	-0.08422	-1.214004	0.053999	-0.33334	0.16491	TRUE	1.00E+00
38321_r_at	-0.08414	-1.21378	0.009147	-0.12634	-0.04194	FALSE	4.57E-16
39031_at	-0.08407	-1.213584	0.049455	-0.31223	0.14409	TRUE	1.00E+00
33047_at	-0.08405	-1.213529	0.069395	-0.40421	0.2361	TRUE	1.00E+00
41644_at	-0.08401	-1.213417	0.055812	-0.3415	0.17349	TRUE	1.00E+00
36638_at	-0.08397	-1.213305	0.023508	-0.19243	0.02448	TRUE	1.00E+00
34187_at	-0.08397	-1.213305	0.027379	-0.21028	0.04235	TRUE	1.00E+00
34453_at	-0.08395	-1.213249	0.109442	-0.58887	0.42097	TRUE	1.00E+00
37786_at	-0.08394	-1.213221	0.045274	-0.29282	0.12494	TRUE	1.00E+00
35393_at	-0.08392	-1.213165	0.048231	-0.30644	0.1386	TRUE	1.00E+00
519_g_at	-0.08388	-1.213054	0.013783	-0.14747	-0.02029	FALSE	1.46E-05
38406_f_at	-0.08388	-1.213054	0.113667	-0.60829	0.44053	TRUE	1.00E+00
32715_at	-0.08383	-1.212914	0.056871	-0.34621	0.17855	TRUE	1.00E+00
36826_at	-0.08382	-1.212886	0.009943	-0.12969	-0.03794	FALSE	4.39E-13
38339_at	-0.08381	-1.212858	0.065303	-0.38509	0.21748	TRUE	1.00E+00
31387_at	-0.08379	-1.212802	0.097842	-0.53519	0.36762	TRUE	1.00E+00
760_at	-0.08376	-1.212718	0.021727	-0.184	0.01647	TRUE	1.00E+00
32390_at	-0.08375	-1.212691	0.078956	-0.44802	0.28052	TRUE	1.00E+00
41518_at	-0.08372	-1.212607	0.037574	-0.25707	0.08963	TRUE	1.00E+00
35655_at	-0.08369	-1.212523	0.053555	-0.33077	0.16339	TRUE	1.00E+00
31711_at	-0.08361	-1.2123	0.030364	-0.22369	0.05648	TRUE	1.00E+00
41485_at	-0.08355	-1.212132	0.015076	-0.1531	-0.01399	FALSE	3.78E-04
33843_g_at	-0.08354	-1.212104	0.078082	-0.44378	0.27669	TRUE	1.00E+00
1100_at	-0.08352	-1.212049	0.034097	-0.24083	0.07378	TRUE	1.00E+00
31490_at	-0.0835	-1.211993	0.038013	-0.25887	0.09188	TRUE	1.00E+00
31596_f_at	-0.0835	-1.211993	0.024598	-0.19698	0.02999	TRUE	1.00E+00
38604_at	-0.08348	-1.211937	0.046624	-0.29858	0.13162	TRUE	1.00E+00
32116_at	-0.08347	-1.211909	0.037965	-0.25863	0.09168	TRUE	1.00E+00
1944_f_at	-0.08344	-1.211825	0.013122	-0.14398	-0.0229	FALSE	2.57E-06
37346_at	-0.0834	-1.211714	0.010882	-0.13361	-0.0332	FALSE	2.26E-10
34539_at	-0.0834	-1.211714	0.024884	-0.1982	0.03141	TRUE	1.00E+00
33320_at	-0.08337	-1.21163	0.080824	-0.45626	0.28951	TRUE	1.00E+00
37950_at	-0.08336	-1.211602	0.031358	-0.22803	0.06132	TRUE	1.00E+00
1347_at	-0.08336	-1.211602	0.036221	-0.25046	0.08375	TRUE	1.00E+00
35935_at	-0.08335	-1.211574	0.02564	-0.20165	0.03494	TRUE	1.00E+00
1224_at	-0.08329	-1.211407	0.044527	-0.28871	0.12214	TRUE	1.00E+00
36398_at	-0.08328	-1.211379	0.031703	-0.22954	0.06298	TRUE	1.00E+00
34175_r_at	-0.08321	-1.211184	0.036256	-0.25048	0.08406	TRUE	1.00E+00
39712_at	-0.0832	-1.211156	0.025469	-0.2007	0.03431	TRUE	1.00E+00
34636_at	-0.08316	-1.211044	0.067433	-0.39426	0.22795	TRUE	1.00E+00
38757_at	-0.08312	-1.210933	0.034507	-0.24232	0.07608	TRUE	1.00E+00
41397_at	-0.08311	-1.210905	0.01747	-0.16371	-0.00251	FALSE	2.48E-02
37559_at	-0.08305	-1.210738	0.058224	-0.35168	0.18557	TRUE	1.00E+00
1434_at	-0.08302	-1.210654	0.030373	-0.22314	0.05711	TRUE	1.00E+00
1847_s_at	-0.08294	-1.210431	0.024016	-0.19374	0.02787	TRUE	1.00E+00
40858_at	-0.08293	-1.210403	0.030055	-0.22159	0.05573	TRUE	1.00E+00
39331_at	-0.08292	-1.210375	0.013781	-0.1465	-0.01934	FALSE	2.24E-05
31553_at	-0.08287	-1.210236	0.049982	-0.31346	0.14773	TRUE	1.00E+00

34979_at	-0.08283	-1.210124	0.085057	-0.47525	0.30959	TRUE	1.00E+00
39795_at	-0.0828	-1.210041	0.038463	-0.26025	0.09466	TRUE	1.00E+00
35205_at	-0.08278	-1.209985	0.021044	-0.17987	0.01431	TRUE	1.00E+00
41508_at	-0.08278	-1.209985	0.082191	-0.46197	0.29642	TRUE	1.00E+00
41153_f_at	-0.08274	-1.209874	0.01506	-0.15222	-0.01326	FALSE	4.97E-04
34784_at	-0.08268	-1.209706	0.013473	-0.14484	-0.02052	FALSE	1.06E-05
31406_at	-0.08258	-1.209428	0.017095	-0.16145	-0.00371	FALSE	1.72E-02
36480_at	-0.08254	-1.209317	0.029133	-0.21695	0.05187	TRUE	1.00E+00
1312_at	-0.08243	-1.20901	0.013791	-0.14606	-0.0188	FALSE	2.87E-05
40866_at	-0.0824	-1.208927	0.014727	-0.15035	-0.01446	FALSE	2.78E-04
33519_at	-0.08238	-1.208871	0.053281	-0.3282	0.16344	TRUE	1.00E+00
38585_at	-0.08234	-1.20876	0.056719	-0.34402	0.17934	TRUE	1.00E+00
38134_at	-0.08233	-1.208732	0.03598	-0.24833	0.08367	TRUE	1.00E+00
1997_s_at	-0.08227	-1.208565	0.044585	-0.28797	0.12342	TRUE	1.00E+00
33011_at	-0.08225	-1.208509	0.023222	-0.18939	0.02489	TRUE	1.00E+00
40406_at	-0.08214	-1.208203	0.113062	-0.60376	0.43948	TRUE	1.00E+00
41613_at	-0.08212	-1.208148	0.089767	-0.49626	0.33203	TRUE	1.00E+00
38269_at	-0.08201	-1.207842	0.009118	-0.12408	-0.03995	FALSE	2.98E-15
35230_at	-0.082	-1.207814	0.010793	-0.1318	-0.03221	FALSE	3.81E-10
36539_at	-0.08199	-1.207786	0.096857	-0.52885	0.36486	TRUE	1.00E+00
39934_at	-0.08198	-1.207758	0.034044	-0.23905	0.07509	TRUE	1.00E+00
33341_at	-0.08191	-1.207564	0.013947	-0.14625	-0.01757	FALSE	5.40E-05
1330_at	-0.08185	-1.207397	0.029826	-0.21945	0.05576	TRUE	1.00E+00
34649_at	-0.0818	-1.207258	0.037066	-0.2528	0.08921	TRUE	1.00E+00
33080_s_at	-0.08178	-1.207202	0.071028	-0.40947	0.24592	TRUE	1.00E+00
34305_at	-0.08162	-1.206757	0.019639	-0.17223	0.00899	TRUE	4.09E-01
37420_i_at	-0.08162	-1.206757	0.068967	-0.3998	0.23657	TRUE	1.00E+00
37307_at	-0.08157	-1.206619	0.027304	-0.20754	0.0444	TRUE	1.00E+00
35282_r_at	-0.08156	-1.206591	0.022088	-0.18347	0.02034	TRUE	1.00E+00
35196_at	-0.08151	-1.206452	0.08361	-0.46725	0.30424	TRUE	1.00E+00
35729_at	-0.08146	-1.206313	0.034138	-0.23896	0.07604	TRUE	1.00E+00
31344_at	-0.08144	-1.206257	0.071956	-0.41341	0.25054	TRUE	1.00E+00
33967_at	-0.0814	-1.206146	0.037006	-0.25213	0.08933	TRUE	1.00E+00
38711_at	-0.08135	-1.206007	0.035864	-0.24681	0.08411	TRUE	1.00E+00
37390_at	-0.08128	-1.205813	0.017004	-0.15973	-0.00283	FALSE	2.21E-02
31861_at	-0.08128	-1.205813	0.026415	-0.20315	0.04059	TRUE	1.00E+00
32836_at	-0.08128	-1.205813	0.015561	-0.15307	-0.00949	FALSE	2.22E-03
1501_at	-0.08124	-1.205702	0.129603	-0.67918	0.51669	TRUE	1.00E+00
39285_at	-0.08121	-1.205619	0.038413	-0.25843	0.09602	TRUE	1.00E+00
31947_r_at	-0.08109	-1.205286	0.067723	-0.39354	0.23136	TRUE	1.00E+00
37353_g_at	-0.08109	-1.205286	0.026611	-0.20386	0.04168	TRUE	1.00E+00
39939_at	-0.08107	-1.20523	0.049913	-0.31135	0.14921	TRUE	1.00E+00
39333_at	-0.08106	-1.205202	0.025772	-0.19996	0.03784	TRUE	1.00E+00
32813_s_at	-0.08104	-1.205147	0.085249	-0.47435	0.31226	TRUE	1.00E+00
596_s_at	-0.08101	-1.205064	0.043474	-0.28158	0.11956	TRUE	1.00E+00
39097_at	-0.08101	-1.205064	0.018225	-0.16509	0.00308	TRUE	1.11E-01
39959_at	-0.081	-1.205036	0.019275	-0.16993	0.00792	TRUE	3.33E-01
37603_at	-0.08098	-1.20498	0.097206	-0.52944	0.36749	TRUE	1.00E+00
37544_at	-0.0809	-1.204759	0.019801	-0.17225	0.01045	TRUE	5.55E-01
40090_at	-0.08086	-1.204648	0.077915	-0.44033	0.2786	TRUE	1.00E+00
1020_s_at	-0.08085	-1.20462	0.023008	-0.187	0.0253	TRUE	1.00E+00

31978_at	-0.08084	-1.204592	0.053554	-0.32792	0.16623	TRUE	1.00E+00
35401_s_at	-0.08083	-1.204564	0.03313	-0.23368	0.07202	TRUE	1.00E+00
918_at	-0.0808	-1.204481	0.028466	-0.21213	0.05053	TRUE	1.00E+00
39755_at	-0.08077	-1.204398	0.026037	-0.2009	0.03935	TRUE	1.00E+00
34237_at	-0.08075	-1.204342	0.106784	-0.57341	0.4119	TRUE	1.00E+00
32314_g_at	-0.08074	-1.204315	0.02256	-0.18482	0.02335	TRUE	1.00E+00
31878_at	-0.08068	-1.204148	0.02947	-0.21664	0.05528	TRUE	1.00E+00
37476_at	-0.08065	-1.204065	0.048968	-0.30657	0.14526	TRUE	1.00E+00
36363_at	-0.08058	-1.203871	0.108538	-0.58133	0.42017	TRUE	1.00E+00
36257_at	-0.08057	-1.203843	0.053168	-0.32586	0.16473	TRUE	1.00E+00
38829_r_at	-0.08053	-1.203733	0.049391	-0.3084	0.14734	TRUE	1.00E+00
38746_at	-0.08052	-1.203705	0.022222	-0.18304	0.02201	TRUE	1.00E+00
39836_at	-0.08047	-1.203566	0.083585	-0.4661	0.30515	TRUE	1.00E+00
40911_at	-0.08047	-1.203566	0.041003	-0.26964	0.1087	TRUE	1.00E+00
33002_at	-0.08045	-1.203511	0.043855	-0.28278	0.12188	TRUE	1.00E+00
33977_at	-0.08042	-1.203428	0.106468	-0.57162	0.41078	TRUE	1.00E+00
35515_at	-0.08041	-1.2034	0.031512	-0.2258	0.06497	TRUE	1.00E+00
36816_s_at	-0.08037	-1.203289	0.128021	-0.67101	0.51027	TRUE	1.00E+00
37574_at	-0.08033	-1.203178	0.037462	-0.25317	0.0925	TRUE	1.00E+00
41165_g_at	-0.08033	-1.203178	0.037341	-0.25261	0.09195	TRUE	1.00E+00
40679_at	-0.08023	-1.202901	0.022007	-0.18176	0.0213	TRUE	1.00E+00
34167_s_at	-0.08023	-1.202901	0.019817	-0.17166	0.0112	TRUE	6.51E-01
33018_at	-0.08023	-1.202901	0.105749	-0.56811	0.40766	TRUE	1.00E+00
32438_at	-0.08015	-1.20268	0.019908	-0.172	0.0117	TRUE	7.16E-01
41826_at	-0.08015	-1.20268	0.057556	-0.34569	0.18539	TRUE	1.00E+00
38880_at	-0.08012	-1.202597	0.027248	-0.20583	0.04559	TRUE	1.00E+00
34903_at	-0.08009	-1.202514	0.035344	-0.24315	0.08297	TRUE	1.00E+00
32603_at	-0.08008	-1.202486	0.02203	-0.18172	0.02156	TRUE	1.00E+00
38770_at	-0.08005	-1.202403	0.097087	-0.52797	0.36786	TRUE	1.00E+00
36138_at	-0.08003	-1.202347	0.013749	-0.14346	-0.01659	FALSE	7.41E-05
1513_at	-0.08001	-1.202292	0.025493	-0.19762	0.0376	TRUE	1.00E+00
35956_s_at	-0.08	-1.202264	0.047597	-0.2996	0.13959	TRUE	1.00E+00
32763_r_at	-0.08	-1.202264	0.075921	-0.43027	0.27027	TRUE	1.00E+00
38400_at	-0.07999	-1.202237	0.020681	-0.1754	0.01543	TRUE	1.00E+00
40169_at	-0.07996	-1.202154	0.022939	-0.18579	0.02587	TRUE	1.00E+00
40571_at	-0.07993	-1.202071	0.030902	-0.2225	0.06263	TRUE	1.00E+00
1169_at	-0.07983	-1.201794	0.048046	-0.30149	0.14183	TRUE	1.00E+00
35115_at	-0.07982	-1.201766	0.070548	-0.4053	0.24567	TRUE	1.00E+00
34487_at	-0.07978	-1.201656	0.04283	-0.27738	0.11783	TRUE	1.00E+00
1247_g_at	-0.07977	-1.201628	0.05544	-0.33555	0.17601	TRUE	1.00E+00
1454_at	-0.07974	-1.201545	0.02128	-0.17792	0.01844	TRUE	1.00E+00
35868_at	-0.07973	-1.201517	0.036403	-0.24767	0.08822	TRUE	1.00E+00
2038_g_at	-0.07968	-1.201379	0.035065	-0.24146	0.08209	TRUE	1.00E+00
35517_at	-0.07965	-1.201296	0.102319	-0.5517	0.39241	TRUE	1.00E+00
33667_at	-0.07959	-1.20113	0.015738	-0.1522	-0.00698	FALSE	5.38E-03
33287_at	-0.07951	-1.200909	0.02189	-0.1805	0.02149	TRUE	1.00E+00
2069_s_at	-0.07949	-1.200853	0.018783	-0.16615	0.00716	TRUE	2.92E-01
34626_at	-0.07947	-1.200798	0.034566	-0.23894	0.08001	TRUE	1.00E+00
33903_at	-0.07946	-1.20077	0.021077	-0.1767	0.01778	TRUE	1.00E+00
165_g_at	-0.07946	-1.20077	0.047393	-0.29811	0.13919	TRUE	1.00E+00
180_at	-0.07946	-1.20077	0.033804	-0.23542	0.0765	TRUE	1.00E+00

34256_at	-0.07945	-1.200743	0.036165	-0.2463	0.0874	TRUE	1.00E+00
40138_at	-0.0794	-1.200605	0.028951	-0.21297	0.05417	TRUE	1.00E+00
34724_at	-0.07938	-1.200549	0.048371	-0.30255	0.14378	TRUE	1.00E+00
40954_at	-0.07937	-1.200522	0.050303	-0.31144	0.15271	TRUE	1.00E+00
32307_s_at	-0.07932	-1.200383	0.021268	-0.17744	0.0188	TRUE	1.00E+00
36804_at	-0.07927	-1.200245	0.03691	-0.24956	0.09102	TRUE	1.00E+00
41152_f_at	-0.07926	-1.200218	0.027961	-0.20826	0.04974	TRUE	1.00E+00
36819_at	-0.07924	-1.200162	0.050998	-0.31452	0.15605	TRUE	1.00E+00
34133_at	-0.0792	-1.200052	0.04073	-0.26712	0.10871	TRUE	1.00E+00
38752_r_at	-0.07919	-1.200024	0.043095	-0.27801	0.11963	TRUE	1.00E+00
36534_at	-0.07918	-1.199997	0.042043	-0.27315	0.11479	TRUE	1.00E+00
32638_s_at	-0.07918	-1.199997	0.047411	-0.29791	0.13956	TRUE	1.00E+00
32748_at	-0.07912	-1.199831	0.021964	-0.18045	0.02222	TRUE	1.00E+00
35593_at	-0.07909	-1.199748	0.038219	-0.25542	0.09724	TRUE	1.00E+00
32434_at	-0.07908	-1.19972	0.014622	-0.14654	-0.01162	FALSE	8.03E-04
513_at	-0.07902	-1.199555	0.044357	-0.28366	0.12562	TRUE	1.00E+00
32938_at	-0.07889	-1.199196	0.029312	-0.21412	0.05635	TRUE	1.00E+00
34520_at	-0.07888	-1.199168	0.128058	-0.66968	0.51193	TRUE	1.00E+00
41766_at	-0.07887	-1.19914	0.032522	-0.22891	0.07118	TRUE	1.00E+00
36084_at	-0.07887	-1.19914	0.040717	-0.26672	0.10899	TRUE	1.00E+00
32209_at	-0.07885	-1.199085	0.019073	-0.16685	0.00914	TRUE	4.49E-01
37089_at	-0.07885	-1.199085	0.027652	-0.20642	0.04873	TRUE	1.00E+00
40427_at	-0.07885	-1.199085	0.040765	-0.26692	0.10923	TRUE	1.00E+00
35689_at	-0.07883	-1.19903	0.133079	-0.6928	0.53514	TRUE	1.00E+00
41227_at	-0.07873	-1.198754	0.043058	-0.27738	0.11992	TRUE	1.00E+00
663_at	-0.07872	-1.198726	0.041335	-0.26942	0.11198	TRUE	1.00E+00
945_at	-0.0787	-1.198671	0.012319	-0.13554	-0.02187	FALSE	2.11E-06
38632_at	-0.07869	-1.198643	0.026593	-0.20139	0.044	TRUE	1.00E+00
198_g_at	-0.07868	-1.198616	0.021312	-0.177	0.01965	TRUE	1.00E+00
33250_at	-0.07867	-1.198588	0.00625	-0.1075	-0.04983	FALSE	3.17E-32
724_at	-0.07861	-1.198423	0.157724	-0.80628	0.64906	TRUE	1.00E+00
39099_at	-0.07858	-1.19834	0.01506	-0.14806	-0.0091	FALSE	2.28E-03
656_at	-0.07856	-1.198285	0.026458	-0.20063	0.0435	TRUE	1.00E+00
357_at	-0.07856	-1.198285	0.014609	-0.14596	-0.01116	FALSE	9.54E-04
32378_at	-0.07854	-1.198229	0.019147	-0.16688	0.00979	TRUE	5.17E-01
40534_at	-0.0785	-1.198119	0.047681	-0.29849	0.14148	TRUE	1.00E+00
31981_at	-0.07845	-1.197981	0.0542	-0.3285	0.17161	TRUE	1.00E+00
33316_at	-0.07844	-1.197954	0.151981	-0.77961	0.62274	TRUE	1.00E+00
34126_at	-0.07839	-1.197816	0.037735	-0.25248	0.09571	TRUE	1.00E+00
35680_r_at	-0.07838	-1.197788	0.056065	-0.33704	0.18028	TRUE	1.00E+00
160027_s_at	-0.07827	-1.197485	0.025773	-0.19718	0.04064	TRUE	1.00E+00
41343_at	-0.07827	-1.197485	0.014389	-0.14465	-0.01188	FALSE	6.75E-04
40736_at	-0.07827	-1.197485	0.055549	-0.33455	0.17801	TRUE	1.00E+00
33718_at	-0.07826	-1.197457	0.027544	-0.20533	0.04882	TRUE	1.00E+00
39945_at	-0.07823	-1.197374	0.048503	-0.302	0.14554	TRUE	1.00E+00
38565_at	-0.07822	-1.197347	0.084319	-0.46723	0.3108	TRUE	1.00E+00
31793_at	-0.07821	-1.197319	0.088199	-0.48512	0.3287	TRUE	1.00E+00
37402_at	-0.07818	-1.197237	0.057762	-0.34467	0.18831	TRUE	1.00E+00
33533_at	-0.07817	-1.197209	0.033887	-0.2345	0.07817	TRUE	1.00E+00
37260_at	-0.07815	-1.197154	0.06715	-0.38795	0.23166	TRUE	1.00E+00
39993_at	-0.07812	-1.197071	0.018259	-0.16235	0.00612	TRUE	2.38E-01

34072_s_at	-0.07809	-1.196989	0.045046	-0.28592	0.12973	TRUE	1.00E+00
703_at	-0.078	-1.196741	0.097457	-0.52763	0.37162	TRUE	1.00E+00
affx-biodn-3_at	-0.07799	-1.196713	0.018043	-0.16123	0.00526	TRUE	1.95E-01
35564_at	-0.07798	-1.196685	0.097747	-0.52895	0.37298	TRUE	1.00E+00
34060_g_at	-0.07791	-1.196493	0.080647	-0.44998	0.29416	TRUE	1.00E+00
34343_at	-0.0779	-1.196465	0.087075	-0.47963	0.32383	TRUE	1.00E+00
33104_at	-0.07782	-1.196245	0.11911	-0.62735	0.4717	TRUE	1.00E+00
35915_at	-0.07781	-1.196217	0.092111	-0.50277	0.34715	TRUE	1.00E+00
39773_at	-0.07777	-1.196107	0.030476	-0.21837	0.06283	TRUE	1.00E+00
33161_at	-0.07776	-1.196079	0.02073	-0.1734	0.01788	TRUE	1.00E+00
36053_at	-0.07775	-1.196052	0.045794	-0.28902	0.13353	TRUE	1.00E+00
35796_at	-0.07774	-1.196024	0.026815	-0.20145	0.04598	TRUE	1.00E+00
32818_at	-0.07771	-1.195942	0.189261	-0.95089	0.79546	TRUE	1.00E+00
37411_at	-0.0777	-1.195914	0.025783	-0.19665	0.04125	TRUE	1.00E+00
39386_at	-0.07769	-1.195887	0.02286	-0.18316	0.02778	TRUE	1.00E+00
35810_at	-0.07767	-1.195832	0.018854	-0.16465	0.00931	TRUE	4.79E-01
359_at	-0.07766	-1.195804	0.011294	-0.12977	-0.02555	FALSE	7.76E-08
1841_s_at	-0.07764	-1.195749	0.017186	-0.15693	0.00165	TRUE	7.89E-02
138_at	-0.07763	-1.195721	0.025125	-0.19355	0.03828	TRUE	1.00E+00
41110_at	-0.07763	-1.195721	0.044855	-0.28457	0.12931	TRUE	1.00E+00
32172_at	-0.0776	-1.195639	0.026711	-0.20083	0.04563	TRUE	1.00E+00
35499_at	-0.07755	-1.195501	0.048002	-0.29902	0.14391	TRUE	1.00E+00
32897_at	-0.07755	-1.195501	0.016028	-0.1515	-0.00361	FALSE	1.65E-02
36554_at	-0.07754	-1.195474	0.037444	-0.2503	0.09521	TRUE	1.00E+00
1995_at	-0.07754	-1.195474	0.062471	-0.36576	0.21068	TRUE	1.00E+00
40553_at	-0.07753	-1.195446	0.029032	-0.21148	0.05641	TRUE	1.00E+00
41578_at	-0.0775	-1.195364	0.077037	-0.43292	0.27792	TRUE	1.00E+00
37993_at	-0.07747	-1.195281	0.023747	-0.18703	0.03209	TRUE	1.00E+00
36693_at	-0.07741	-1.195116	0.128921	-0.6722	0.51737	TRUE	1.00E+00
32512_at	-0.07731	-1.194841	0.081669	-0.4541	0.29948	TRUE	1.00E+00
34038_at	-0.07731	-1.194841	0.100513	-0.54104	0.38642	TRUE	1.00E+00
34160_at	-0.07727	-1.194731	0.034563	-0.23673	0.08219	TRUE	1.00E+00
1152_i_at	-0.07724	-1.194648	0.108293	-0.57685	0.42238	TRUE	1.00E+00
126_s_at	-0.07705	-1.194126	0.090628	-0.49517	0.34107	TRUE	1.00E+00
39395_at	-0.07703	-1.194071	0.209303	-1.04267	0.88861	TRUE	1.00E+00
33627_at	-0.07699	-1.193961	0.030534	-0.21786	0.06388	TRUE	1.00E+00
33636_at	-0.07694	-1.193823	0.02301	-0.1831	0.02922	TRUE	1.00E+00
37908_at	-0.0769	-1.193713	0.03084	-0.21919	0.06538	TRUE	1.00E+00
32929_at	-0.07685	-1.193576	0.030687	-0.21843	0.06472	TRUE	1.00E+00
1509_at	-0.07679	-1.193411	0.036334	-0.24442	0.09084	TRUE	1.00E+00
416_s_at	-0.07677	-1.193356	0.039019	-0.25679	0.10325	TRUE	1.00E+00
40659_at	-0.07676	-1.193328	0.052673	-0.31976	0.16625	TRUE	1.00E+00
39675_at	-0.0767	-1.193164	0.055404	-0.3323	0.17891	TRUE	1.00E+00
41453_at	-0.07668	-1.193109	0.031255	-0.22088	0.06752	TRUE	1.00E+00
34525_at	-0.07666	-1.193054	0.029158	-0.21118	0.05786	TRUE	1.00E+00
38617_at	-0.07665	-1.193026	0.034342	-0.23509	0.08179	TRUE	1.00E+00
31790_at	-0.07664	-1.192999	0.017395	-0.1569	0.00361	TRUE	1.33E-01
35516_at	-0.07664	-1.192999	0.035219	-0.23913	0.08584	TRUE	1.00E+00
329_s_at	-0.07662	-1.192944	0.026788	-0.20021	0.04697	TRUE	1.00E+00
920_at	-0.07661	-1.192916	0.037281	-0.24861	0.09539	TRUE	1.00E+00
37034_at	-0.07661	-1.192916	0.039642	-0.2595	0.10628	TRUE	1.00E+00

993_at	-0.07658	-1.192834	0.032828	-0.22803	0.07488	TRUE	1.00E+00
38962_at	-0.07656	-1.192779	0.046354	-0.29042	0.1373	TRUE	1.00E+00
33070_at	-0.07655	-1.192752	0.038771	-0.25542	0.10232	TRUE	1.00E+00
40755_at	-0.07654	-1.192724	0.0463	-0.29015	0.13707	TRUE	1.00E+00
38825_at	-0.07654	-1.192724	0.082068	-0.45516	0.30209	TRUE	1.00E+00
31697_s_at	-0.07647	-1.192532	0.019569	-0.16675	0.01382	TRUE	1.00E+00
272_at	-0.07642	-1.192395	0.031956	-0.22386	0.07101	TRUE	1.00E+00
35740_at	-0.07642	-1.192395	0.057531	-0.34184	0.189	TRUE	1.00E+00
969_s_at	-0.07642	-1.192395	0.014857	-0.14496	-0.00787	FALSE	3.41E-03
41522_at	-0.07636	-1.19223	0.020442	-0.17067	0.01795	TRUE	1.00E+00
35806_at	-0.07632	-1.19212	0.007918	-0.11285	-0.03978	FALSE	6.98E-18
40136_at	-0.0763	-1.192065	0.027075	-0.20121	0.04861	TRUE	1.00E+00
33288_i_at	-0.07616	-1.191681	0.022219	-0.17867	0.02634	TRUE	1.00E+00
35104_r_at	-0.07613	-1.191599	0.062974	-0.36667	0.21441	TRUE	1.00E+00
33187_r_at	-0.07607	-1.191434	0.038143	-0.25204	0.09991	TRUE	1.00E+00
34191_at	-0.07599	-1.191215	0.025917	-0.19556	0.04358	TRUE	1.00E+00
33641_g_at	-0.07595	-1.191105	0.024296	-0.18804	0.03614	TRUE	1.00E+00
32082_at	-0.07593	-1.19105	0.019912	-0.1678	0.01593	TRUE	1.00E+00
32004_s_at	-0.07591	-1.190995	0.023477	-0.18422	0.03241	TRUE	1.00E+00
31673_s_at	-0.07584	-1.190803	0.021683	-0.17588	0.02419	TRUE	1.00E+00
36585_at	-0.07583	-1.190776	0.012711	-0.13448	-0.01719	FALSE	3.07E-05
31534_at	-0.0758	-1.190694	0.038896	-0.25525	0.10365	TRUE	1.00E+00
33964_at	-0.07579	-1.190666	0.079453	-0.44236	0.29077	TRUE	1.00E+00
31802_at	-0.07571	-1.190447	0.025767	-0.19458	0.04317	TRUE	1.00E+00
34655_at	-0.07571	-1.190447	0.029011	-0.20955	0.05814	TRUE	1.00E+00
39360_at	-0.07569	-1.190392	0.021994	-0.17716	0.02578	TRUE	1.00E+00
37631_at	-0.07563	-1.190228	0.02176	-0.17602	0.02477	TRUE	1.00E+00
35535_f_at	-0.07559	-1.190118	0.2191	-1.08643	0.93524	TRUE	1.00E+00
40595_at	-0.07555	-1.190008	0.025672	-0.19399	0.0429	TRUE	1.00E+00
1433_g_at	-0.07551	-1.189899	0.028509	-0.20704	0.05602	TRUE	1.00E+00
36582_g_at	-0.0755	-1.189871	0.026711	-0.19874	0.04774	TRUE	1.00E+00
41546_at	-0.07543	-1.18968	0.023817	-0.18531	0.03445	TRUE	1.00E+00
2071_s_at	-0.07536	-1.189488	0.087412	-0.47865	0.32792	TRUE	1.00E+00
40167_s_at	-0.07535	-1.18946	0.017529	-0.15622	0.00552	TRUE	2.17E-01
37079_at	-0.07533	-1.189406	0.02616	-0.19602	0.04536	TRUE	1.00E+00
33514_at	-0.07527	-1.189241	0.065749	-0.37861	0.22807	TRUE	1.00E+00
35909_at	-0.07527	-1.189241	0.109264	-0.57936	0.42883	TRUE	1.00E+00
40281_at	-0.07525	-1.189187	0.034747	-0.23556	0.08506	TRUE	1.00E+00
39992_at	-0.07523	-1.189132	0.038387	-0.25233	0.10187	TRUE	1.00E+00
427_f_at	-0.07523	-1.189132	0.04638	-0.28921	0.13875	TRUE	1.00E+00
34634_s_at	-0.0752	-1.18905	0.021063	-0.17237	0.02198	TRUE	1.00E+00
33279_s_at	-0.07513	-1.188858	0.03681	-0.24496	0.09469	TRUE	1.00E+00
39908_at	-0.07508	-1.188721	0.027981	-0.20417	0.05401	TRUE	1.00E+00
33414_at	-0.07507	-1.188694	0.011563	-0.12841	-0.02172	FALSE	1.07E-06
40765_at	-0.07506	-1.188666	0.010425	-0.12316	-0.02697	FALSE	7.60E-09
39016_r_at	-0.07506	-1.188666	0.023099	-0.18163	0.03151	TRUE	1.00E+00
33951_at	-0.07504	-1.188612	0.07471	-0.41972	0.26964	TRUE	1.00E+00
34118_at	-0.07498	-1.188447	0.064869	-0.37426	0.2243	TRUE	1.00E+00
41191_at	-0.07498	-1.188447	0.016795	-0.15246	0.0025	TRUE	1.01E-01
1899_s_at	-0.07484	-1.188064	0.116011	-0.61007	0.46038	TRUE	1.00E+00
1166_at	-0.07484	-1.188064	0.01487	-0.14345	-0.00624	FALSE	6.10E-03

32925_at	-0.07481	-1.187982	0.046034	-0.28719	0.13757	TRUE	1.00E+00
33421_s_at	-0.07478	-1.1879	0.04017	-0.26011	0.11055	TRUE	1.00E+00
32536_at	-0.07475	-1.187818	0.035516	-0.23861	0.0891	TRUE	1.00E+00
31745_at	-0.07473	-1.187764	0.026029	-0.19482	0.04536	TRUE	1.00E+00
33913_at	-0.07465	-1.187545	0.024491	-0.18764	0.03834	TRUE	1.00E+00
257_at	-0.07462	-1.187463	0.036931	-0.245	0.09576	TRUE	1.00E+00
34041_at	-0.07462	-1.187463	0.057584	-0.34028	0.19105	TRUE	1.00E+00
771_s_at	-0.0746	-1.187408	0.016947	-0.15279	0.00358	TRUE	1.35E-01
34793_s_at	-0.07457	-1.187326	0.024128	-0.18589	0.03675	TRUE	1.00E+00
35496_at	-0.07448	-1.18708	0.051399	-0.31162	0.16265	TRUE	1.00E+00
36741_at	-0.07448	-1.18708	0.048005	-0.29595	0.147	TRUE	1.00E+00
38787_at	-0.07442	-1.186916	0.101349	-0.542	0.39317	TRUE	1.00E+00
38507_at	-0.0744	-1.186861	0.03142	-0.21936	0.07056	TRUE	1.00E+00
33597_at	-0.07434	-1.186697	0.034572	-0.23384	0.08516	TRUE	1.00E+00
31321_at	-0.07431	-1.186615	0.029068	-0.20841	0.0598	TRUE	1.00E+00
40759_at	-0.07429	-1.186561	0.194333	-0.97086	0.82229	TRUE	1.00E+00
35061_at	-0.07424	-1.186424	0.057147	-0.3379	0.18941	TRUE	1.00E+00
41253_s_at	-0.07417	-1.186233	0.009726	-0.11904	-0.0293	FALSE	3.06E-10
35007_at	-0.0741	-1.186042	0.035938	-0.23991	0.0917	TRUE	1.00E+00
34185_at	-0.07409	-1.186015	0.028474	-0.20546	0.05728	TRUE	1.00E+00
36593_at	-0.07405	-1.185905	0.016931	-0.15216	0.00406	TRUE	1.54E-01
37100_at	-0.07401	-1.185796	0.066273	-0.37977	0.23175	TRUE	1.00E+00
211_at	-0.074	-1.185769	0.02097	-0.17075	0.02275	TRUE	1.00E+00
39487_at	-0.07398	-1.185714	0.115117	-0.60508	0.45713	TRUE	1.00E+00
40959_at	-0.07396	-1.18566	0.016259	-0.14897	0.00105	TRUE	6.81E-02
40696_at	-0.07395	-1.185632	0.013974	-0.13842	-0.00948	FALSE	1.53E-03
38520_r_at	-0.07392	-1.18555	0.063478	-0.36678	0.21894	TRUE	1.00E+00
40932_at	-0.07389	-1.185468	0.022307	-0.17681	0.02903	TRUE	1.00E+00
35936_g_at	-0.07387	-1.185414	0.058015	-0.34153	0.19378	TRUE	1.00E+00
33570_at	-0.07382	-1.185277	0.049916	-0.30411	0.15647	TRUE	1.00E+00
728_at	-0.07373	-1.185032	0.049262	-0.30101	0.15354	TRUE	1.00E+00
1421_at	-0.07368	-1.184895	0.089918	-0.48852	0.34117	TRUE	1.00E+00
32635_at	-0.07356	-1.184568	0.045536	-0.28364	0.13652	TRUE	1.00E+00
36907_at	-0.07354	-1.184513	0.010645	-0.12266	-0.02443	FALSE	6.18E-08
34587_at	-0.07352	-1.184459	0.087844	-0.47879	0.33176	TRUE	1.00E+00
32201_at	-0.07349	-1.184377	0.018945	-0.16089	0.01392	TRUE	1.00E+00
37590_g_at	-0.07346	-1.184295	0.03969	-0.25658	0.10965	TRUE	1.00E+00
34486_at	-0.07342	-1.184186	0.072765	-0.40913	0.26228	TRUE	1.00E+00
41193_at	-0.0734	-1.184132	0.071416	-0.40288	0.25609	TRUE	1.00E+00
39051_at	-0.07338	-1.184077	0.026419	-0.19527	0.0485	TRUE	1.00E+00
38986_at	-0.07336	-1.184023	0.036478	-0.24165	0.09494	TRUE	1.00E+00
34261_at	-0.07335	-1.183995	0.038633	-0.25159	0.10488	TRUE	1.00E+00
594_s_at	-0.07334	-1.183968	0.034361	-0.23186	0.08519	TRUE	1.00E+00
40831_at	-0.07332	-1.183914	0.012431	-0.13068	-0.01597	FALSE	4.64E-05
36380_at	-0.07332	-1.183914	0.128994	-0.66845	0.5218	TRUE	1.00E+00
1931_at	-0.0733	-1.183859	0.113359	-0.59629	0.44969	TRUE	1.00E+00
1793_at	-0.07327	-1.183777	0.019337	-0.16249	0.01594	TRUE	1.00E+00
35114_at	-0.07326	-1.18375	0.03517	-0.23552	0.089	TRUE	1.00E+00
32340_s_at	-0.07325	-1.183723	0.031336	-0.21782	0.07132	TRUE	1.00E+00
1902_at	-0.07322	-1.183641	0.021764	-0.17364	0.02719	TRUE	1.00E+00
37763_at	-0.07322	-1.183641	0.025176	-0.18937	0.04293	TRUE	1.00E+00

1675_at	-0.07318	-1.183532	0.021456	-0.17217	0.02581	TRUE	1.00E+00
32569_at	-0.07317	-1.183505	0.012479	-0.13074	-0.01559	FALSE	5.73E-05
31349_at	-0.07315	-1.18345	0.047609	-0.29279	0.1465	TRUE	1.00E+00
38491_at	-0.07312	-1.183368	0.041575	-0.26492	0.11869	TRUE	1.00E+00
32876_s_at	-0.07306	-1.183205	0.051598	-0.31111	0.165	TRUE	1.00E+00
40657_r_at	-0.07304	-1.183151	0.148605	-0.75865	0.61256	TRUE	1.00E+00
37498_at	-0.07301	-1.183069	0.033004	-0.22528	0.07926	TRUE	1.00E+00
34846_at	-0.07301	-1.183069	0.014029	-0.13773	-0.00828	FALSE	2.46E-03
35519_at	-0.07297	-1.18296	0.036222	-0.24008	0.09415	TRUE	1.00E+00
35366_at	-0.07293	-1.182851	0.027428	-0.19947	0.05361	TRUE	1.00E+00
31662_at	-0.07288	-1.182715	0.040124	-0.25799	0.11224	TRUE	1.00E+00
32701_at	-0.07284	-1.182606	0.01877	-0.15944	0.01376	TRUE	1.00E+00
41376_i_at	-0.07277	-1.182415	0.052318	-0.31414	0.16861	TRUE	1.00E+00
33611_g_at	-0.07275	-1.182361	0.095433	-0.51304	0.36754	TRUE	1.00E+00
33793_at	-0.07273	-1.182306	0.075608	-0.42155	0.2761	TRUE	1.00E+00
37220_at	-0.07272	-1.182279	0.018695	-0.15897	0.01353	TRUE	1.00E+00
1703_g_at	-0.07271	-1.182252	0.01822	-0.15677	0.01135	TRUE	8.32E-01
33377_at	-0.07267	-1.182143	0.058564	-0.34286	0.19752	TRUE	1.00E+00
33422_at	-0.07257	-1.181871	0.017447	-0.15307	0.00793	TRUE	4.03E-01
37157_at	-0.07257	-1.181871	0.03168	-0.21872	0.07359	TRUE	1.00E+00
35076_at	-0.07247	-1.181599	0.057819	-0.33922	0.19428	TRUE	1.00E+00
36737_at	-0.07245	-1.181544	0.019079	-0.16048	0.01557	TRUE	1.00E+00
1129_at	-0.07242	-1.181463	0.018899	-0.15961	0.01477	TRUE	1.00E+00
40247_at	-0.07235	-1.181272	0.054901	-0.32564	0.18094	TRUE	1.00E+00
34868_at	-0.07232	-1.181191	0.029187	-0.20698	0.06233	TRUE	1.00E+00
32961_at	-0.07229	-1.181109	0.020063	-0.16485	0.02027	TRUE	1.00E+00
41255_at	-0.07224	-1.180973	0.065671	-0.37522	0.23074	TRUE	1.00E+00
41034_s_at	-0.07223	-1.180946	0.019278	-0.16117	0.01671	TRUE	1.00E+00
31977_at	-0.07214	-1.180701	0.020544	-0.16692	0.02264	TRUE	1.00E+00
37395_at	-0.07209	-1.180565	0.006799	-0.10345	-0.04072	FALSE	3.66E-22
34250_at	-0.07207	-1.180511	0.02585	-0.19133	0.04719	TRUE	1.00E+00
35138_at	-0.07202	-1.180375	0.022739	-0.17692	0.03289	TRUE	1.00E+00
33464_at	-0.072	-1.180321	0.044339	-0.27656	0.13256	TRUE	1.00E+00
1564_at	-0.07193	-1.18013	0.022477	-0.17563	0.03177	TRUE	1.00E+00
34542_at	-0.07192	-1.180103	0.039114	-0.25238	0.10853	TRUE	1.00E+00
32379_f_at	-0.07192	-1.180103	0.043977	-0.27481	0.13098	TRUE	1.00E+00
36019_at	-0.0719	-1.180049	0.009439	-0.11545	-0.02835	FALSE	3.27E-10
34978_g_at	-0.0719	-1.180049	0.033075	-0.22449	0.0807	TRUE	1.00E+00
37376_at	-0.07185	-1.179913	0.027252	-0.19758	0.05388	TRUE	1.00E+00
40116_at	-0.07177	-1.179696	0.020422	-0.16599	0.02245	TRUE	1.00E+00
265_s_at	-0.07176	-1.179669	0.069489	-0.39236	0.24883	TRUE	1.00E+00
932_i_at	-0.07175	-1.179641	0.024426	-0.18444	0.04094	TRUE	1.00E+00
40712_at	-0.07172	-1.17956	0.015915	-0.14514	0.00171	TRUE	8.33E-02
40101_g_at	-0.07171	-1.179533	0.024902	-0.1866	0.04318	TRUE	1.00E+00
40162_s_at	-0.07169	-1.179478	0.069151	-0.39073	0.24734	TRUE	1.00E+00
1611_s_at	-0.07168	-1.179451	0.130007	-0.67148	0.52812	TRUE	1.00E+00
34358_at	-0.07164	-1.179343	0.013474	-0.1338	-0.00948	FALSE	1.33E-03
41540_at	-0.07159	-1.179207	0.015848	-0.14471	0.00153	TRUE	7.91E-02
1981_s_at	-0.07158	-1.17918	0.033994	-0.22842	0.08525	TRUE	1.00E+00
41000_at	-0.07158	-1.17918	0.037203	-0.24322	0.10006	TRUE	1.00E+00
32225_at	-0.07156	-1.179125	0.029669	-0.20844	0.06532	TRUE	1.00E+00

37299_at	-0.07156	-1.179125	0.011817	-0.12608	-0.01704	FALSE	1.76E-05
34297_at	-0.07136	-1.178583	0.061758	-0.35629	0.21356	TRUE	1.00E+00
39289_at	-0.07136	-1.178583	0.020204	-0.16457	0.02185	TRUE	1.00E+00
34934_at	-0.07124	-1.178257	0.031895	-0.21839	0.07591	TRUE	1.00E+00
41080_at	-0.07122	-1.178203	0.031671	-0.21734	0.07489	TRUE	1.00E+00
38461_at	-0.07122	-1.178203	0.050925	-0.30617	0.16373	TRUE	1.00E+00
33035_at	-0.07119	-1.178121	0.034723	-0.23139	0.089	TRUE	1.00E+00
1666_at	-0.07117	-1.178067	0.039423	-0.25305	0.11071	TRUE	1.00E+00
35920_at	-0.07112	-1.177931	0.021058	-0.16827	0.02603	TRUE	1.00E+00
742_at	-0.07112	-1.177931	0.034476	-0.23017	0.08794	TRUE	1.00E+00
305_g_at	-0.07107	-1.177796	0.025988	-0.19096	0.04883	TRUE	1.00E+00
38545_at	-0.07105	-1.177742	0.111892	-0.58728	0.44517	TRUE	1.00E+00
952_at	-0.07104	-1.177714	0.056384	-0.33118	0.18909	TRUE	1.00E+00
1653_at	-0.07104	-1.177714	0.022062	-0.17282	0.03075	TRUE	1.00E+00
40580_r_at	-0.07099	-1.177579	0.036222	-0.2381	0.09613	TRUE	1.00E+00
affx-crex-3_st	-0.07093	-1.177416	0.172765	-0.86799	0.72614	TRUE	1.00E+00
41029_at	-0.07092	-1.177389	0.020034	-0.16335	0.02151	TRUE	1.00E+00
40999_at	-0.0709	-1.177335	0.041544	-0.26257	0.12077	TRUE	1.00E+00
34177_at	-0.07089	-1.177308	0.015762	-0.14361	0.00183	TRUE	8.69E-02
41178_at	-0.07084	-1.177172	0.02889	-0.20413	0.06244	TRUE	1.00E+00
37047_at	-0.07083	-1.177145	0.01536	-0.14169	0.00004	TRUE	5.05E-02
37837_at	-0.0708	-1.177064	0.031736	-0.21722	0.07562	TRUE	1.00E+00
40276_at	-0.07078	-1.17701	0.011887	-0.12562	-0.01594	FALSE	3.30E-05
1334_s_at	-0.07077	-1.176982	0.028749	-0.20341	0.06186	TRUE	1.00E+00
34301_r_at	-0.07077	-1.176982	0.021476	-0.16985	0.02831	TRUE	1.00E+00
39249_at	-0.07077	-1.176982	0.031358	-0.21544	0.07391	TRUE	1.00E+00
40744_at	-0.07075	-1.176928	0.082921	-0.45331	0.31181	TRUE	1.00E+00
31339_at	-0.07075	-1.176928	0.053009	-0.31531	0.17381	TRUE	1.00E+00
191_at	-0.07073	-1.176874	0.036565	-0.23943	0.09797	TRUE	1.00E+00
41580_at	-0.07071	-1.17682	0.046193	-0.28382	0.14241	TRUE	1.00E+00
664_at	-0.07068	-1.176739	0.035288	-0.23348	0.09212	TRUE	1.00E+00
40149_at	-0.07066	-1.176684	0.014779	-0.13884	-0.00247	FALSE	2.20E-02
32990_at	-0.07063	-1.176603	0.029316	-0.20588	0.06462	TRUE	1.00E+00
32845_at	-0.07063	-1.176603	0.009711	-0.11543	-0.02583	FALSE	4.44E-09
33375_at	-0.07055	-1.176386	0.046566	-0.28539	0.14428	TRUE	1.00E+00
31951_s_at	-0.07053	-1.176332	0.014234	-0.1362	-0.00486	FALSE	9.14E-03
33748_at	-0.07042	-1.176034	0.091341	-0.49183	0.35099	TRUE	1.00E+00
37984_s_at	-0.07041	-1.176007	0.047606	-0.29005	0.14922	TRUE	1.00E+00
368_at	-0.07041	-1.176007	0.025867	-0.18975	0.04893	TRUE	1.00E+00
32384_g_at	-0.07037	-1.175899	0.088069	-0.47668	0.33594	TRUE	1.00E+00
505_at	-0.07031	-1.175736	0.022802	-0.17551	0.03489	TRUE	1.00E+00
40557_at	-0.07028	-1.175655	0.075329	-0.41782	0.27726	TRUE	1.00E+00
40160_at	-0.07027	-1.175628	0.01763	-0.15161	0.01107	TRUE	8.49E-01
41739_s_at	-0.07022	-1.175493	0.029883	-0.20809	0.06764	TRUE	1.00E+00
32460_at	-0.07022	-1.175493	0.054013	-0.31941	0.17897	TRUE	1.00E+00
36935_at	-0.07021	-1.175466	0.018903	-0.15743	0.017	TRUE	1.00E+00
31659_at	-0.07015	-1.175303	0.035838	-0.23549	0.0952	TRUE	1.00E+00
41418_at	-0.07006	-1.17506	0.048329	-0.29302	0.15291	TRUE	1.00E+00
438_at	-0.07003	-1.174979	0.037285	-0.24205	0.10199	TRUE	1.00E+00
189_s_at	-0.06997	-1.174816	0.039516	-0.25228	0.11234	TRUE	1.00E+00
38225_at	-0.06994	-1.174735	0.104567	-0.55236	0.41249	TRUE	1.00E+00

32995_at	-0.06991	-1.174654	0.064918	-0.36941	0.22959	TRUE	1.00E+00
1724_at	-0.0699	-1.174627	0.013035	-0.13004	-0.00977	FALSE	1.03E-03
41041_s_at	-0.0699	-1.174627	0.015467	-0.14126	0.00146	TRUE	7.83E-02
34042_at	-0.06987	-1.174546	0.027017	-0.19451	0.05478	TRUE	1.00E+00
1638_at	-0.06983	-1.174438	0.140299	-0.71711	0.57745	TRUE	1.00E+00
39351_at	-0.0698	-1.174357	0.018891	-0.15696	0.01736	TRUE	1.00E+00
40606_at	-0.06979	-1.17433	0.046675	-0.28513	0.14555	TRUE	1.00E+00
822_s_at	-0.06977	-1.174276	0.013997	-0.13435	-0.0052	FALSE	7.83E-03
32881_at	-0.06974	-1.174194	0.049987	-0.30036	0.16088	TRUE	1.00E+00
36173_r_at	-0.06971	-1.174113	0.016297	-0.1449	0.00547	TRUE	2.38E-01
36183_at	-0.06962	-1.17387	0.013284	-0.13091	-0.00833	FALSE	2.02E-03
38080_at	-0.06962	-1.17387	0.007601	-0.10469	-0.03455	FALSE	6.59E-16
37656_at	-0.06957	-1.173735	0.033992	-0.2264	0.08725	TRUE	1.00E+00
31908_at	-0.06957	-1.173735	0.08029	-0.43999	0.30086	TRUE	1.00E+00
40523_at	-0.06957	-1.173735	0.107692	-0.56641	0.42728	TRUE	1.00E+00
40438_at	-0.06945	-1.173411	0.029034	-0.2034	0.0645	TRUE	1.00E+00
39030_at	-0.06945	-1.173411	0.023463	-0.17769	0.0388	TRUE	1.00E+00
36852_at	-0.06944	-1.173384	0.032975	-0.22158	0.08269	TRUE	1.00E+00
39595_at	-0.06943	-1.173357	0.027794	-0.19766	0.0588	TRUE	1.00E+00
36658_at	-0.06937	-1.173194	0.043334	-0.2693	0.13055	TRUE	1.00E+00
32070_at	-0.06936	-1.173167	0.049207	-0.29638	0.15766	TRUE	1.00E+00
39692_at	-0.06935	-1.17314	0.015184	-0.13941	0.0007	TRUE	6.24E-02
31904_at	-0.06921	-1.172762	0.030819	-0.2114	0.07297	TRUE	1.00E+00
40147_at	-0.06919	-1.172708	0.06206	-0.35551	0.21713	TRUE	1.00E+00
37494_at	-0.06906	-1.172357	0.036005	-0.23517	0.09705	TRUE	1.00E+00
39801_at	-0.06905	-1.17233	0.021541	-0.16843	0.03033	TRUE	1.00E+00
1122_f_at	-0.06904	-1.172303	0.016613	-0.14568	0.0076	TRUE	4.09E-01
37239_r_at	-0.06902	-1.172249	0.057437	-0.33401	0.19597	TRUE	1.00E+00
39537_at	-0.06898	-1.172141	0.033222	-0.22226	0.08429	TRUE	1.00E+00
926_at	-0.06891	-1.171952	0.058392	-0.33831	0.20049	TRUE	1.00E+00
31469_s_at	-0.0689	-1.171925	0.025565	-0.18684	0.04905	TRUE	1.00E+00
41686_s_at	-0.06888	-1.171872	0.029157	-0.2034	0.06564	TRUE	1.00E+00
31888_s_at	-0.06887	-1.171845	0.024056	-0.17985	0.04212	TRUE	1.00E+00
32177_s_at	-0.06882	-1.17171	0.046117	-0.28159	0.14394	TRUE	1.00E+00
38464_at	-0.06881	-1.171683	0.010155	-0.11566	-0.02196	FALSE	1.56E-07
40644_g_at	-0.06878	-1.171602	0.022903	-0.17444	0.03688	TRUE	1.00E+00
1396_at	-0.06877	-1.171575	0.029351	-0.20419	0.06664	TRUE	1.00E+00
1168_at	-0.0687	-1.171386	0.041428	-0.25983	0.12244	TRUE	1.00E+00
36235_at	-0.0687	-1.171386	0.037165	-0.24016	0.10277	TRUE	1.00E+00
37890_at	-0.06868	-1.171332	0.022973	-0.17466	0.03731	TRUE	1.00E+00
40345_at	-0.06866	-1.171278	0.061202	-0.35102	0.2137	TRUE	1.00E+00
617_at	-0.06865	-1.171251	0.051929	-0.30823	0.17092	TRUE	1.00E+00
35635_at	-0.06861	-1.171143	0.044655	-0.27463	0.13741	TRUE	1.00E+00
35788_at	-0.06858	-1.171062	0.018905	-0.15579	0.01864	TRUE	1.00E+00
41308_at	-0.06857	-1.171035	0.075461	-0.41672	0.27958	TRUE	1.00E+00
34007_at	-0.06855	-1.170981	0.088894	-0.47867	0.34157	TRUE	1.00E+00
1506_at	-0.06855	-1.170981	0.099445	-0.52735	0.39025	TRUE	1.00E+00
32509_at	-0.06854	-1.170954	0.032939	-0.22051	0.08343	TRUE	1.00E+00
34861_at	-0.06851	-1.170874	0.015595	-0.14045	0.00344	TRUE	1.41E-01
38210_at	-0.06849	-1.17082	0.027695	-0.19626	0.05928	TRUE	1.00E+00
32665_at	-0.06843	-1.170658	0.019556	-0.15865	0.0218	TRUE	1.00E+00

36753_at	-0.06834	-1.170415	0.05593	-0.32638	0.1897	TRUE	1.00E+00
1033_g_at	-0.06833	-1.170388	0.054969	-0.32193	0.18527	TRUE	1.00E+00
32541_at	-0.0683	-1.170308	0.009036	-0.10999	-0.02662	FALSE	5.13E-10
1241_at	-0.06823	-1.170119	0.019487	-0.15814	0.02167	TRUE	1.00E+00
40022_at	-0.06819	-1.170011	0.039662	-0.25118	0.11479	TRUE	1.00E+00
39182_at	-0.06814	-1.169876	0.018716	-0.15449	0.01821	TRUE	1.00E+00
37042_at	-0.0681	-1.169769	0.027501	-0.19498	0.05878	TRUE	1.00E+00
36058_at	-0.06807	-1.169688	0.013199	-0.12896	-0.00717	FALSE	3.17E-03
40733_f_at	-0.06806	-1.169661	0.070972	-0.3955	0.25937	TRUE	1.00E+00
33093_at	-0.06803	-1.16958	0.047144	-0.28553	0.14947	TRUE	1.00E+00
35697_at	-0.06802	-1.169553	0.025479	-0.18557	0.04953	TRUE	1.00E+00
40025_at	-0.06798	-1.169446	0.032568	-0.21824	0.08227	TRUE	1.00E+00
35341_at	-0.06788	-1.169176	0.022047	-0.1696	0.03383	TRUE	1.00E+00
41724_at	-0.06788	-1.169176	0.014547	-0.13499	-0.00077	FALSE	3.87E-02
35063_at	-0.06783	-1.169042	0.053009	-0.31239	0.17674	TRUE	1.00E+00
38953_at	-0.06782	-1.169015	0.079691	-0.43548	0.29984	TRUE	1.00E+00
688_at	-0.06779	-1.168934	0.01169	-0.12172	-0.01386	FALSE	8.42E-05
34699_at	-0.0677	-1.168692	0.014074	-0.13263	-0.00277	FALSE	1.90E-02
841_at	-0.0677	-1.168692	0.101268	-0.53491	0.39951	TRUE	1.00E+00
39552_at	-0.06767	-1.168611	0.034607	-0.22734	0.09199	TRUE	1.00E+00
37841_at	-0.06766	-1.168584	0.125144	-0.64503	0.5097	TRUE	1.00E+00
41629_at	-0.06766	-1.168584	0.13414	-0.68653	0.55121	TRUE	1.00E+00
34999_at	-0.06766	-1.168584	0.049151	-0.29442	0.1591	TRUE	1.00E+00
335_r_at	-0.06763	-1.168503	0.024067	-0.17866	0.04341	TRUE	1.00E+00
31689_at	-0.06752	-1.168208	0.030892	-0.21004	0.075	TRUE	1.00E+00
38609_at	-0.06751	-1.168181	0.106339	-0.55812	0.42309	TRUE	1.00E+00
38032_at	-0.06747	-1.168073	0.046258	-0.28089	0.14594	TRUE	1.00E+00
36051_s_at	-0.06746	-1.168046	0.03485	-0.22824	0.09332	TRUE	1.00E+00
34642_at	-0.06745	-1.168019	0.022381	-0.1707	0.03581	TRUE	1.00E+00
33745_at	-0.06738	-1.167831	0.025117	-0.18326	0.0485	TRUE	1.00E+00
35951_at	-0.06733	-1.167697	0.044374	-0.27206	0.13739	TRUE	1.00E+00
34406_at	-0.06731	-1.167643	0.013898	-0.13143	-0.00319	FALSE	1.61E-02
34478_at	-0.06727	-1.167535	0.064842	-0.36643	0.23188	TRUE	1.00E+00
2013_at	-0.06726	-1.167508	0.036785	-0.23697	0.10246	TRUE	1.00E+00
36337_at	-0.06721	-1.167374	0.042099	-0.26144	0.12702	TRUE	1.00E+00
36416_g_at	-0.0672	-1.167347	0.038281	-0.24382	0.10941	TRUE	1.00E+00
33109_f_at	-0.0672	-1.167347	0.028291	-0.19772	0.06333	TRUE	1.00E+00
1383_at	-0.06719	-1.16732	0.019041	-0.15504	0.02066	TRUE	1.00E+00
38542_at	-0.06718	-1.167293	0.026783	-0.19075	0.05638	TRUE	1.00E+00
39369_at	-0.06716	-1.16724	0.015234	-0.13745	0.00312	TRUE	1.31E-01
31506_s_at	-0.06716	-1.16724	0.0883	-0.47454	0.34022	TRUE	1.00E+00
37140_s_at	-0.06714	-1.167186	0.051935	-0.30675	0.17246	TRUE	1.00E+00
32038_s_at	-0.06714	-1.167186	0.017028	-0.1457	0.01142	TRUE	1.00E+00
983_at	-0.0669	-1.166541	0.051339	-0.30376	0.16995	TRUE	1.00E+00
118_at	-0.06689	-1.166514	0.02295	-0.17277	0.03899	TRUE	1.00E+00
32870_g_at	-0.06672	-1.166058	0.019848	-0.15829	0.02485	TRUE	1.00E+00
31613_at	-0.06668	-1.16595	0.128964	-0.66166	0.52831	TRUE	1.00E+00
1748_s_at	-0.06664	-1.165843	0.044181	-0.27047	0.13719	TRUE	1.00E+00
1692_s_at	-0.06654	-1.165574	0.035185	-0.22887	0.09578	TRUE	1.00E+00
33909_at	-0.06652	-1.165521	0.037348	-0.23883	0.10579	TRUE	1.00E+00
32787_at	-0.06648	-1.165413	0.055863	-0.32421	0.19124	TRUE	1.00E+00

32872_at	-0.06648	-1.165413	0.047604	-0.2861	0.15315	TRUE	1.00E+00
39416_at	-0.06637	-1.165118	0.035523	-0.23026	0.09752	TRUE	1.00E+00
779_at	-0.06635	-1.165065	0.057359	-0.33098	0.19828	TRUE	1.00E+00
34128_at	-0.06634	-1.165038	0.023969	-0.17693	0.04424	TRUE	1.00E+00
40426_at	-0.06634	-1.165038	0.01365	-0.12931	-0.00337	FALSE	1.48E-02
38812_at	-0.06631	-1.164957	0.027373	-0.1926	0.05997	TRUE	1.00E+00
32446_at	-0.06629	-1.164904	0.082472	-0.44678	0.3142	TRUE	1.00E+00
40628_at	-0.06625	-1.164796	0.038958	-0.24598	0.11349	TRUE	1.00E+00
32339_at	-0.06623	-1.164743	0.098071	-0.51869	0.38623	TRUE	1.00E+00
736_f_at	-0.06621	-1.164689	0.031651	-0.21224	0.07981	TRUE	1.00E+00
32206_at	-0.06618	-1.164609	0.02152	-0.16546	0.03311	TRUE	1.00E+00
35657_at	-0.06614	-1.164501	0.01723	-0.14563	0.01335	TRUE	1.00E+00
40660_at	-0.06602	-1.16418	0.062178	-0.35289	0.22085	TRUE	1.00E+00
affx-humrge/m10	-0.06596	-1.164019	0.048491	-0.28967	0.15776	TRUE	1.00E+00
38424_at	-0.06593	-1.163938	0.014501	-0.13283	0.00097	TRUE	6.88E-02
833_at	-0.06592	-1.163912	0.017107	-0.14484	0.01301	TRUE	1.00E+00
38936_at	-0.06591	-1.163885	0.015836	-0.13897	0.00714	TRUE	3.98E-01
40626_at	-0.0659	-1.163858	0.032593	-0.21627	0.08447	TRUE	1.00E+00
affx-bioc-5_st	-0.06589	-1.163831	0.044017	-0.26896	0.13719	TRUE	1.00E+00
34270_at	-0.06589	-1.163831	0.029804	-0.20339	0.07161	TRUE	1.00E+00
33773_at	-0.06585	-1.163724	0.03349	-0.22036	0.08866	TRUE	1.00E+00
1729_at	-0.06584	-1.163697	0.022016	-0.16742	0.03573	TRUE	1.00E+00
37331_g_at	-0.06583	-1.16367	0.018126	-0.14945	0.0178	TRUE	1.00E+00
1346_at	-0.06579	-1.163563	0.017708	-0.14749	0.0159	TRUE	1.00E+00
33926_at	-0.06578	-1.163536	0.051263	-0.30228	0.17072	TRUE	1.00E+00
37738_g_at	-0.06574	-1.163429	0.020442	-0.16005	0.02857	TRUE	1.00E+00
41450_at	-0.0657	-1.163322	0.067705	-0.37806	0.24666	TRUE	1.00E+00
41558_s_at	-0.06562	-1.163108	0.057848	-0.33251	0.20126	TRUE	1.00E+00
40316_at	-0.06561	-1.163081	0.038908	-0.24512	0.11389	TRUE	1.00E+00
40883_at	-0.06561	-1.163081	0.040962	-0.25459	0.12337	TRUE	1.00E+00
36434_r_at	-0.06559	-1.163028	0.213957	-1.0527	0.92152	TRUE	1.00E+00
33838_at	-0.06557	-1.162974	0.020541	-0.16034	0.0292	TRUE	1.00E+00
33434_at	-0.06554	-1.162894	0.035092	-0.22744	0.09637	TRUE	1.00E+00
36036_at	-0.06546	-1.162679	0.035078	-0.2273	0.09637	TRUE	1.00E+00
34172_s_at	-0.06545	-1.162653	0.042	-0.25922	0.12832	TRUE	1.00E+00
32912_at	-0.06541	-1.162546	0.086481	-0.46439	0.33358	TRUE	1.00E+00
35147_at	-0.06539	-1.162492	0.034771	-0.22581	0.09503	TRUE	1.00E+00
232_at	-0.06529	-1.162224	0.021678	-0.16531	0.03472	TRUE	1.00E+00
38265_at	-0.06529	-1.162224	0.03462	-0.22501	0.09443	TRUE	1.00E+00
1395_at	-0.06527	-1.162171	0.019375	-0.15466	0.02411	TRUE	1.00E+00
40979_at	-0.06523	-1.162064	0.022157	-0.16745	0.037	TRUE	1.00E+00
39109_at	-0.0652	-1.161984	0.06697	-0.37417	0.24378	TRUE	1.00E+00
40228_at	-0.0652	-1.161984	0.015696	-0.13761	0.00722	TRUE	4.13E-01
36054_at	-0.06519	-1.161957	0.031454	-0.21031	0.07993	TRUE	1.00E+00
33463_at	-0.06518	-1.16193	0.05753	-0.3306	0.20024	TRUE	1.00E+00
40200_at	-0.06517	-1.161903	0.020727	-0.1608	0.03046	TRUE	1.00E+00
39657_at	-0.06516	-1.161877	0.025247	-0.18164	0.05132	TRUE	1.00E+00
1694_s_at	-0.06505	-1.161582	0.032223	-0.21371	0.08362	TRUE	1.00E+00
41279_f_at	-0.06502	-1.161502	0.020319	-0.15876	0.02873	TRUE	1.00E+00
35596_at	-0.06497	-1.161368	0.068971	-0.38317	0.25323	TRUE	1.00E+00
36007_at	-0.06497	-1.161368	0.052698	-0.30809	0.17816	TRUE	1.00E+00

33771_at	-0.06492	-1.161235	0.05616	-0.32402	0.19418	TRUE	1.00E+00
963_at	-0.06491	-1.161208	0.018315	-0.14941	0.01959	TRUE	1.00E+00
32622_at	-0.0649	-1.161181	0.015397	-0.13594	0.00613	TRUE	3.15E-01
38998_g_at	-0.0649	-1.161181	0.021654	-0.1648	0.03501	TRUE	1.00E+00
33366_at	-0.06487	-1.161101	0.014577	-0.13213	0.00238	TRUE	1.08E-01
38274_at	-0.06473	-1.160727	0.011437	-0.1175	-0.01197	FALSE	1.91E-04
32609_at	-0.06467	-1.160566	0.065969	-0.36902	0.23968	TRUE	1.00E+00
33010_at	-0.06466	-1.16054	0.078269	-0.42576	0.29644	TRUE	1.00E+00
31353_f_at	-0.06461	-1.160406	0.028958	-0.19821	0.06899	TRUE	1.00E+00
41125_r_at	-0.06459	-1.160353	0.081984	-0.44283	0.31365	TRUE	1.00E+00
36571_at	-0.06457	-1.160299	0.026667	-0.1876	0.05845	TRUE	1.00E+00
31708_at	-0.06456	-1.160273	0.016936	-0.14269	0.01358	TRUE	1.00E+00
38677_at	-0.06456	-1.160273	0.051823	-0.30365	0.17453	TRUE	1.00E+00
34645_at	-0.06455	-1.160246	0.020128	-0.15741	0.02831	TRUE	1.00E+00
32080_at	-0.06452	-1.160166	0.014232	-0.13019	0.00114	TRUE	7.32E-02
959_at	-0.06447	-1.160032	0.033149	-0.21741	0.08847	TRUE	1.00E+00
37549_g_at	-0.06445	-1.159979	0.016888	-0.14236	0.01346	TRUE	1.00E+00
36886_f_at	-0.06438	-1.159792	0.10189	-0.53446	0.4057	TRUE	1.00E+00
33003_at	-0.06435	-1.159712	0.022445	-0.1679	0.0392	TRUE	1.00E+00
39598_at	-0.06435	-1.159712	0.017674	-0.14589	0.01719	TRUE	1.00E+00
32202_at	-0.06435	-1.159712	0.012888	-0.12381	-0.00489	FALSE	7.51E-03
41800_s_at	-0.06428	-1.159525	0.022046	-0.16599	0.03743	TRUE	1.00E+00
36281_at	-0.06417	-1.159231	0.038164	-0.24025	0.11191	TRUE	1.00E+00
39962_at	-0.06414	-1.159151	0.041925	-0.25756	0.12929	TRUE	1.00E+00
38303_at	-0.06414	-1.159151	0.020254	-0.15758	0.02931	TRUE	1.00E+00
35534_at	-0.06408	-1.158991	0.027918	-0.19289	0.06472	TRUE	1.00E+00
541_g_at	-0.06406	-1.158937	0.031026	-0.2072	0.07908	TRUE	1.00E+00
39956_at	-0.06403	-1.158857	0.038863	-0.24333	0.11527	TRUE	1.00E+00
40929_at	-0.06398	-1.158724	0.079582	-0.43114	0.30318	TRUE	1.00E+00
34986_at	-0.06395	-1.158644	0.072859	-0.40009	0.27219	TRUE	1.00E+00
40649_at	-0.06392	-1.158564	0.037042	-0.23482	0.10697	TRUE	1.00E+00
31514_at	-0.06392	-1.158564	0.06264	-0.35291	0.22507	TRUE	1.00E+00
34939_r_at	-0.06382	-1.158297	0.161643	-0.80958	0.68193	TRUE	1.00E+00
31476_g_at	-0.06382	-1.158297	0.101671	-0.53289	0.40525	TRUE	1.00E+00
34154_at	-0.06376	-1.158137	0.050017	-0.29452	0.167	TRUE	1.00E+00
34457_at	-0.06372	-1.158031	0.032217	-0.21235	0.08492	TRUE	1.00E+00
41371_at	-0.06372	-1.158031	0.035612	-0.22801	0.10058	TRUE	1.00E+00
32545_r_at	-0.06371	-1.158004	0.019904	-0.15554	0.02812	TRUE	1.00E+00
1277_at	-0.06365	-1.157844	0.052374	-0.30528	0.17799	TRUE	1.00E+00
37431_at	-0.06363	-1.157791	0.016278	-0.13874	0.01147	TRUE	1.00E+00
35151_at	-0.06355	-1.157577	0.055453	-0.31939	0.19228	TRUE	1.00E+00
35329_at	-0.06354	-1.157551	0.026648	-0.18648	0.05941	TRUE	1.00E+00
895_at	-0.06342	-1.157231	0.020148	-0.15638	0.02953	TRUE	1.00E+00
1379_at	-0.06338	-1.157124	0.033071	-0.21595	0.0892	TRUE	1.00E+00
34148_at	-0.06331	-1.156938	0.062981	-0.35388	0.22726	TRUE	1.00E+00
32400_at	-0.06329	-1.156884	0.044122	-0.26684	0.14027	TRUE	1.00E+00
34511_s_at	-0.06327	-1.156831	0.04295	-0.26142	0.13489	TRUE	1.00E+00
38651_at	-0.06323	-1.156725	0.013114	-0.12373	-0.00272	FALSE	1.80E-02
41036_at	-0.06323	-1.156725	0.040267	-0.249	0.12255	TRUE	1.00E+00
40275_at	-0.06321	-1.156671	0.023185	-0.17017	0.04376	TRUE	1.00E+00
916_at	-0.0632	-1.156645	0.042223	-0.258	0.1316	TRUE	1.00E+00

31988_at	-0.06319	-1.156618	0.069007	-0.38156	0.25518	TRUE	1.00E+00
33706_at	-0.06317	-1.156565	0.023077	-0.16964	0.0433	TRUE	1.00E+00
40051_at	-0.06317	-1.156565	0.024569	-0.17652	0.05018	TRUE	1.00E+00
35384_at	-0.06315	-1.156512	0.058318	-0.3322	0.20591	TRUE	1.00E+00
41156_g_at	-0.06311	-1.156405	0.008745	-0.10346	-0.02277	FALSE	6.72E-09
1988_at	-0.0631	-1.156378	0.01376	-0.12659	0.00038	TRUE	5.71E-02
35187_at	-0.06309	-1.156352	0.022039	-0.16476	0.03859	TRUE	1.00E+00
33887_at	-0.06302	-1.156165	0.010186	-0.11001	-0.01602	FALSE	7.77E-06
41476_at	-0.06299	-1.156086	0.018254	-0.1472	0.02123	TRUE	1.00E+00
36240_at	-0.06299	-1.156086	0.087741	-0.46779	0.34181	TRUE	1.00E+00
1367_f_at	-0.06297	-1.156032	0.018506	-0.14835	0.02241	TRUE	1.00E+00
39440_f_at	-0.06295	-1.155979	0.115372	-0.59522	0.46933	TRUE	1.00E+00
37253_at	-0.06293	-1.155926	0.057308	-0.32733	0.20146	TRUE	1.00E+00
319_g_at	-0.06293	-1.155926	0.013768	-0.12645	0.00059	TRUE	6.13E-02
31539_r_at	-0.06292	-1.155899	0.134853	-0.68508	0.55924	TRUE	1.00E+00
32178_r_at	-0.06291	-1.155873	0.028193	-0.19298	0.06716	TRUE	1.00E+00
33072_at	-0.06284	-1.155686	0.061722	-0.34759	0.22192	TRUE	1.00E+00
39098_at	-0.0628	-1.15558	0.150187	-0.7557	0.6301	TRUE	1.00E+00
39383_at	-0.06275	-1.155447	0.019814	-0.15416	0.02866	TRUE	1.00E+00
37244_at	-0.06275	-1.155447	0.015395	-0.13377	0.00828	TRUE	5.78E-01
39024_at	-0.0627	-1.155314	0.019541	-0.15286	0.02745	TRUE	1.00E+00
41607_at	-0.0627	-1.155314	0.107371	-0.55806	0.43267	TRUE	1.00E+00
32153_s_at	-0.06269	-1.155287	0.045368	-0.272	0.14662	TRUE	1.00E+00
38767_at	-0.06259	-1.155021	0.048328	-0.28556	0.16038	TRUE	1.00E+00
175_s_at	-0.06259	-1.155021	0.031169	-0.20639	0.08121	TRUE	1.00E+00
33095_i_at	-0.06257	-1.154968	0.06221	-0.34958	0.22444	TRUE	1.00E+00
31568_at	-0.06256	-1.154942	0.02363	-0.17158	0.04646	TRUE	1.00E+00
41545_at	-0.06254	-1.154888	0.049842	-0.29249	0.16741	TRUE	1.00E+00
32219_at	-0.06246	-1.154676	0.015696	-0.13487	0.00996	TRUE	8.73E-01
38915_at	-0.06242	-1.154569	0.086374	-0.46091	0.33608	TRUE	1.00E+00
32837_at	-0.06236	-1.15441	0.014623	-0.12983	0.0051	TRUE	2.53E-01
38775_at	-0.06232	-1.154303	0.024288	-0.17437	0.04974	TRUE	1.00E+00
40192_at	-0.06232	-1.154303	0.048884	-0.28785	0.16321	TRUE	1.00E+00
35299_at	-0.06227	-1.154171	0.021905	-0.16333	0.03879	TRUE	1.00E+00
39183_at	-0.06224	-1.154091	0.034901	-0.22326	0.09877	TRUE	1.00E+00
40949_at	-0.06224	-1.154091	0.025763	-0.1811	0.05662	TRUE	1.00E+00
223_at	-0.06224	-1.154091	0.008153	-0.09985	-0.02462	FALSE	2.88E-10
1886_at	-0.06223	-1.154064	0.033735	-0.21788	0.09341	TRUE	1.00E+00
711_at	-0.06223	-1.154064	0.047871	-0.28309	0.15862	TRUE	1.00E+00
40185_at	-0.06221	-1.154011	0.062521	-0.35066	0.22623	TRUE	1.00E+00
1907_at	-0.0622	-1.153985	0.118757	-0.6101	0.48569	TRUE	1.00E+00
33622_at	-0.0622	-1.153985	0.028471	-0.19355	0.06916	TRUE	1.00E+00
32435_at	-0.06218	-1.153931	0.025413	-0.17942	0.05507	TRUE	1.00E+00
33058_at	-0.0621	-1.153719	0.040164	-0.2474	0.1232	TRUE	1.00E+00
36957_at	-0.06209	-1.153692	0.016307	-0.13733	0.01314	TRUE	1.00E+00
34806_at	-0.06208	-1.153666	0.03107	-0.20543	0.08127	TRUE	1.00E+00
41088_at	-0.06207	-1.153639	0.110065	-0.56987	0.44572	TRUE	1.00E+00
39108_at	-0.06207	-1.153639	0.035696	-0.22676	0.10262	TRUE	1.00E+00
38442_at	-0.06206	-1.153613	0.020395	-0.15616	0.03203	TRUE	1.00E+00
39502_at	-0.06204	-1.153559	0.016419	-0.13779	0.01371	TRUE	1.00E+00
39305_at	-0.062	-1.153453	0.035858	-0.22743	0.10344	TRUE	1.00E+00

40622_r_at	-0.06197	-1.153374	0.025434	-0.17931	0.05538	TRUE	1.00E+00
36407_at	-0.06193	-1.153267	0.032144	-0.21023	0.08636	TRUE	1.00E+00
40921_at	-0.06193	-1.153267	0.039156	-0.24258	0.11872	TRUE	1.00E+00
34835_at	-0.0619	-1.153188	0.031022	-0.20502	0.08122	TRUE	1.00E+00
37227_at	-0.06186	-1.153081	0.044249	-0.26601	0.14229	TRUE	1.00E+00
33600_at	-0.06184	-1.153028	0.040183	-0.24723	0.12355	TRUE	1.00E+00
38580_at	-0.06183	-1.153002	0.023908	-0.17214	0.04847	TRUE	1.00E+00
40186_at	-0.06171	-1.152683	0.010855	-0.11179	-0.01163	FALSE	1.65E-04
36310_at	-0.06171	-1.152683	0.081771	-0.43897	0.31555	TRUE	1.00E+00
33290_at	-0.06169	-1.15263	0.032013	-0.20939	0.086	TRUE	1.00E+00
36190_at	-0.06167	-1.152577	0.026695	-0.18483	0.06149	TRUE	1.00E+00
32039_at	-0.06161	-1.152418	0.020879	-0.15793	0.03472	TRUE	1.00E+00
35513_r_at	-0.06159	-1.152365	0.102633	-0.5351	0.41192	TRUE	1.00E+00
36011_at	-0.06158	-1.152338	0.012235	-0.11803	-0.00514	FALSE	6.08E-03
40499_r_at	-0.06158	-1.152338	0.050636	-0.29519	0.17203	TRUE	1.00E+00
37084_at	-0.06153	-1.152206	0.03488	-0.22245	0.09939	TRUE	1.00E+00
33396_at	-0.06152	-1.152179	0.023355	-0.16927	0.04623	TRUE	1.00E+00
32658_at	-0.06152	-1.152179	0.008144	-0.09909	-0.02394	FALSE	5.34E-10
37853_at	-0.06144	-1.151967	0.026847	-0.18531	0.06242	TRUE	1.00E+00
954_s_at	-0.06143	-1.15194	0.026053	-0.18163	0.05877	TRUE	1.00E+00
38352_at	-0.06142	-1.151914	0.038704	-0.23998	0.11715	TRUE	1.00E+00
41121_at	-0.06142	-1.151914	0.102087	-0.5324	0.40957	TRUE	1.00E+00
1268_at	-0.06141	-1.151887	0.020646	-0.15666	0.03384	TRUE	1.00E+00
36748_at	-0.06136	-1.151755	0.086041	-0.45832	0.3356	TRUE	1.00E+00
34594_at	-0.06135	-1.151728	0.049842	-0.2913	0.16859	TRUE	1.00E+00
31823_at	-0.06132	-1.151649	0.033261	-0.21478	0.09213	TRUE	1.00E+00
2019_s_at	-0.0613	-1.151596	0.083591	-0.44695	0.32436	TRUE	1.00E+00
41556_s_at	-0.06128	-1.151543	0.043961	-0.2641	0.14154	TRUE	1.00E+00
964_at	-0.06126	-1.15149	0.046361	-0.27515	0.15263	TRUE	1.00E+00
40902_at	-0.0612	-1.15133	0.030758	-0.20311	0.08071	TRUE	1.00E+00
31835_at	-0.0612	-1.15133	0.030752	-0.20307	0.08068	TRUE	1.00E+00
903_at	-0.06115	-1.151198	0.030592	-0.20229	0.07999	TRUE	1.00E+00
36193_at	-0.06107	-1.150986	0.047824	-0.28171	0.15957	TRUE	1.00E+00
34510_at	-0.06102	-1.150853	0.060558	-0.34041	0.21837	TRUE	1.00E+00
33510_s_at	-0.061	-1.1508	0.032182	-0.20948	0.08747	TRUE	1.00E+00
38866_at	-0.06098	-1.150747	0.194054	-0.95627	0.8343	TRUE	1.00E+00
40235_at	-0.06096	-1.150694	0.024139	-0.17233	0.05041	TRUE	1.00E+00
41731_g_at	-0.06094	-1.150641	0.028517	-0.19251	0.07062	TRUE	1.00E+00
34988_at	-0.06093	-1.150615	0.100886	-0.52638	0.40451	TRUE	1.00E+00
41500_at	-0.0609	-1.150535	0.012834	-0.12011	-0.00169	FALSE	2.63E-02
33682_at	-0.06088	-1.150482	0.038033	-0.23635	0.11459	TRUE	1.00E+00
2092_s_at	-0.06084	-1.150376	0.142157	-0.7167	0.59501	TRUE	1.00E+00
31525_s_at	-0.06083	-1.15035	0.025843	-0.18006	0.0584	TRUE	1.00E+00
34899_at	-0.06078	-1.150218	0.057007	-0.32378	0.20223	TRUE	1.00E+00
31585_at	-0.06069	-1.149979	0.045495	-0.27059	0.1492	TRUE	1.00E+00
32026_s_at	-0.06069	-1.149979	0.019297	-0.14972	0.02834	TRUE	1.00E+00
38284_at	-0.06065	-1.149873	0.027247	-0.18636	0.06506	TRUE	1.00E+00
33105_at	-0.06064	-1.149847	0.032876	-0.21232	0.09103	TRUE	1.00E+00
34836_at	-0.06059	-1.149714	0.024555	-0.17388	0.05269	TRUE	1.00E+00
35898_at	-0.06058	-1.149688	0.071673	-0.39125	0.27009	TRUE	1.00E+00
35971_at	-0.06057	-1.149662	0.07689	-0.41532	0.29417	TRUE	1.00E+00

37666_at	-0.06052	-1.149529	0.015072	-0.13006	0.00901	TRUE	7.48E-01
32831_at	-0.06051	-1.149503	0.048223	-0.28299	0.16197	TRUE	1.00E+00
33267_at	-0.06051	-1.149503	0.101549	-0.52901	0.408	TRUE	1.00E+00
31619_at	-0.0605	-1.149476	0.133624	-0.67698	0.55599	TRUE	1.00E+00
35922_at	-0.06049	-1.14945	0.12171	-0.62201	0.50103	TRUE	1.00E+00
41391_at	-0.06044	-1.149317	0.022591	-0.16467	0.04378	TRUE	1.00E+00
39040_at	-0.06039	-1.149185	0.02299	-0.16646	0.04568	TRUE	1.00E+00
338_at	-0.06039	-1.149185	0.023111	-0.16701	0.04624	TRUE	1.00E+00
35212_at	-0.06036	-1.149106	0.01823	-0.14447	0.02375	TRUE	1.00E+00
1991_s_at	-0.06036	-1.149106	0.023147	-0.16715	0.04643	TRUE	1.00E+00
33412_at	-0.06032	-1.149	0.026004	-0.18029	0.05965	TRUE	1.00E+00
1482_g_at	-0.06023	-1.148762	0.088274	-0.46748	0.34703	TRUE	1.00E+00
41072_at	-0.06017	-1.148603	0.050143	-0.29151	0.17117	TRUE	1.00E+00
1617_at	-0.06017	-1.148603	0.103139	-0.53601	0.41567	TRUE	1.00E+00
37043_at	-0.06012	-1.148471	0.021771	-0.16057	0.04032	TRUE	1.00E+00
34468_at	-0.06007	-1.148339	0.026575	-0.18268	0.06253	TRUE	1.00E+00
37103_at	-0.05993	-1.147969	0.069358	-0.37992	0.26006	TRUE	1.00E+00
31857_r_at	-0.05991	-1.147916	0.067568	-0.37164	0.25182	TRUE	1.00E+00
32793_at	-0.05985	-1.147757	0.026439	-0.18183	0.06213	TRUE	1.00E+00
37587_at	-0.05981	-1.147651	0.090728	-0.47839	0.35877	TRUE	1.00E+00
41102_at	-0.0598	-1.147625	0.023663	-0.16897	0.04937	TRUE	1.00E+00
35107_at	-0.05979	-1.147599	0.046136	-0.27265	0.15306	TRUE	1.00E+00
40326_at	-0.05979	-1.147599	0.027829	-0.18818	0.0686	TRUE	1.00E+00
32463_at	-0.05973	-1.14744	0.022071	-0.16156	0.04209	TRUE	1.00E+00
35630_at	-0.05972	-1.147414	0.024312	-0.17188	0.05244	TRUE	1.00E+00
32275_at	-0.0597	-1.147361	0.077951	-0.41933	0.29994	TRUE	1.00E+00
38176_at	-0.05961	-1.147123	0.02261	-0.16392	0.04471	TRUE	1.00E+00
35824_at	-0.05956	-1.146991	0.034361	-0.21809	0.09897	TRUE	1.00E+00
34447_at	-0.05952	-1.146885	0.024146	-0.17092	0.05188	TRUE	1.00E+00
affx-biob-3_at	-0.05948	-1.14678	0.064222	-0.35578	0.23681	TRUE	1.00E+00
35583_at	-0.05948	-1.14678	0.028866	-0.19266	0.07369	TRUE	1.00E+00
35326_at	-0.05944	-1.146674	0.022674	-0.16405	0.04516	TRUE	1.00E+00
32389_at	-0.05944	-1.146674	0.05969	-0.33482	0.21595	TRUE	1.00E+00
40371_at	-0.05942	-1.146621	0.014711	-0.12729	0.00845	TRUE	6.77E-01
38350_f_at	-0.05934	-1.14641	0.016079	-0.13352	0.01484	TRUE	1.00E+00
38333_at	-0.05929	-1.146278	0.057235	-0.32334	0.20477	TRUE	1.00E+00
41549_s_at	-0.05928	-1.146252	0.038226	-0.23564	0.11708	TRUE	1.00E+00
1621_at	-0.05924	-1.146146	0.044674	-0.26535	0.14686	TRUE	1.00E+00
36556_at	-0.05923	-1.14612	0.029532	-0.19547	0.07702	TRUE	1.00E+00
31594_at	-0.05922	-1.146093	0.018586	-0.14497	0.02653	TRUE	1.00E+00
32269_at	-0.05921	-1.146067	0.028526	-0.19082	0.07239	TRUE	1.00E+00
38925_at	-0.05919	-1.146014	0.114351	-0.58676	0.46837	TRUE	1.00E+00
31524_f_at	-0.05913	-1.145856	0.069245	-0.3786	0.26033	TRUE	1.00E+00
32484_at	-0.05911	-1.145803	0.05823	-0.32776	0.20954	TRUE	1.00E+00
32589_at	-0.0591	-1.145777	0.050248	-0.29092	0.17272	TRUE	1.00E+00
39223_at	-0.059	-1.145513	0.092215	-0.48444	0.36645	TRUE	1.00E+00
34002_at	-0.05896	-1.145407	0.04132	-0.24959	0.13167	TRUE	1.00E+00
38385_at	-0.05892	-1.145302	0.011825	-0.11347	-0.00436	FALSE	7.93E-03
33637_g_at	-0.05892	-1.145302	0.028788	-0.19173	0.0739	TRUE	1.00E+00
40315_at	-0.05886	-1.145144	0.046662	-0.27414	0.15642	TRUE	1.00E+00
36973_at	-0.05886	-1.145144	0.019717	-0.14982	0.03211	TRUE	1.00E+00

41709_at	-0.05881	-1.145012	0.017791	-0.14089	0.02327	TRUE	1.00E+00
36335_at	-0.05873	-1.144801	0.040843	-0.24717	0.1297	TRUE	1.00E+00
34858_at	-0.05871	-1.144748	0.016291	-0.13387	0.01645	TRUE	1.00E+00
40319_at	-0.0587	-1.144722	0.038126	-0.2346	0.11719	TRUE	1.00E+00
38174_at	-0.05861	-1.144485	0.034393	-0.21729	0.10007	TRUE	1.00E+00
33458_r_at	-0.05854	-1.1443	0.028859	-0.19168	0.0746	TRUE	1.00E+00
36305_at	-0.05851	-1.144221	0.049581	-0.28726	0.17024	TRUE	1.00E+00
40594_r_at	-0.0585	-1.144195	0.072245	-0.39181	0.27481	TRUE	1.00E+00
37849_at	-0.05849	-1.144169	0.013715	-0.12177	0.00478	TRUE	2.52E-01
32088_at	-0.05845	-1.144063	0.033549	-0.21323	0.09633	TRUE	1.00E+00
38409_at	-0.05844	-1.144037	0.030941	-0.20119	0.08431	TRUE	1.00E+00
35075_at	-0.05841	-1.143958	0.068072	-0.37247	0.25565	TRUE	1.00E+00
36131_at	-0.05841	-1.143958	0.020089	-0.15109	0.03428	TRUE	1.00E+00
33969_at	-0.0584	-1.143931	0.016356	-0.13386	0.01706	TRUE	1.00E+00
1785_at	-0.05839	-1.143905	0.025643	-0.17669	0.05991	TRUE	1.00E+00
38201_at	-0.05838	-1.143879	0.042333	-0.25369	0.13692	TRUE	1.00E+00
37554_at	-0.05836	-1.143826	0.046324	-0.27208	0.15536	TRUE	1.00E+00
33160_at	-0.05835	-1.1438	0.012891	-0.11783	0.00112	TRUE	7.56E-02
1670_at	-0.05832	-1.143721	0.070598	-0.38403	0.26739	TRUE	1.00E+00
1872_at	-0.05827	-1.143589	0.064906	-0.35772	0.24118	TRUE	1.00E+00
affx-hsac07/x003	-0.05826	-1.143563	0.023953	-0.16877	0.05225	TRUE	1.00E+00
37198_r_at	-0.0582	-1.143405	0.085614	-0.45319	0.33678	TRUE	1.00E+00
37344_at	-0.05818	-1.143352	0.020345	-0.15205	0.03568	TRUE	1.00E+00
38798_s_at	-0.05815	-1.143273	0.049237	-0.28531	0.16901	TRUE	1.00E+00
37386_i_at	-0.05807	-1.143063	0.023366	-0.16587	0.04973	TRUE	1.00E+00
36965_at	-0.05807	-1.143063	0.070188	-0.38189	0.26575	TRUE	1.00E+00
38982_at	-0.05805	-1.14301	0.014099	-0.1231	0.007	TRUE	4.84E-01
37105_at	-0.05801	-1.142905	0.070543	-0.38346	0.26745	TRUE	1.00E+00
35084_at	-0.058	-1.142878	0.058207	-0.32654	0.21055	TRUE	1.00E+00
35933_f_at	-0.05799	-1.142852	0.022214	-0.16048	0.04449	TRUE	1.00E+00
31540_at	-0.05797	-1.142799	0.122557	-0.62339	0.50746	TRUE	1.00E+00
40828_at	-0.05794	-1.14272	0.037031	-0.22879	0.11291	TRUE	1.00E+00
34073_s_at	-0.05794	-1.14272	0.045706	-0.26881	0.15293	TRUE	1.00E+00
793_at	-0.0579	-1.142615	0.102056	-0.52874	0.41295	TRUE	1.00E+00
38324_at	-0.05789	-1.142589	0.135108	-0.68123	0.56544	TRUE	1.00E+00
38377_at	-0.05785	-1.142484	0.048677	-0.28243	0.16673	TRUE	1.00E+00
38064_at	-0.05781	-1.142378	0.031297	-0.2022	0.08658	TRUE	1.00E+00
41738_at	-0.0578	-1.142352	0.021264	-0.15591	0.0403	TRUE	1.00E+00
41338_at	-0.05779	-1.142326	0.017858	-0.14018	0.0246	TRUE	1.00E+00
39087_at	-0.05778	-1.1423	0.120312	-0.61285	0.49728	TRUE	1.00E+00
35032_at	-0.05776	-1.142247	0.025413	-0.17501	0.05949	TRUE	1.00E+00
38708_at	-0.05775	-1.142221	0.02063	-0.15293	0.03742	TRUE	1.00E+00
39786_at	-0.05764	-1.141931	0.021736	-0.15792	0.04264	TRUE	1.00E+00
31719_at	-0.05763	-1.141905	0.01537	-0.12854	0.01328	TRUE	1.00E+00
37012_at	-0.05759	-1.1418	0.013712	-0.12085	0.00567	TRUE	3.37E-01
34691_f_at	-0.05757	-1.141747	0.021651	-0.15746	0.04232	TRUE	1.00E+00
40370_f_at	-0.05754	-1.141668	0.040435	-0.24409	0.12901	TRUE	1.00E+00
36129_at	-0.05752	-1.141616	0.023901	-0.16779	0.05275	TRUE	1.00E+00
34830_at	-0.0575	-1.141563	0.021918	-0.15862	0.04362	TRUE	1.00E+00
39452_s_at	-0.05748	-1.141511	0.042758	-0.25475	0.13978	TRUE	1.00E+00
41155_at	-0.05746	-1.141458	0.015098	-0.12712	0.01219	TRUE	1.00E+00

38894_g_at	-0.05746	-1.141458	0.094761	-0.49465	0.37972	TRUE	1.00E+00
35748_at	-0.05737	-1.141222	0.022808	-0.1626	0.04785	TRUE	1.00E+00
37653_at	-0.05736	-1.141195	0.047457	-0.27631	0.16159	TRUE	1.00E+00
38094_at	-0.05735	-1.141169	0.019558	-0.14758	0.03288	TRUE	1.00E+00
36784_at	-0.05732	-1.14109	0.101126	-0.52387	0.40923	TRUE	1.00E+00
41788_i_at	-0.05723	-1.140854	0.044643	-0.2632	0.14873	TRUE	1.00E+00
41750_at	-0.05723	-1.140854	0.041067	-0.2467	0.13223	TRUE	1.00E+00
32100_r_at	-0.05713	-1.140591	0.019791	-0.14843	0.03418	TRUE	1.00E+00
31559_at	-0.05712	-1.140565	0.032787	-0.20839	0.09414	TRUE	1.00E+00
41521_at	-0.05712	-1.140565	0.054334	-0.30779	0.19356	TRUE	1.00E+00
729_i_at	-0.05709	-1.140486	0.030221	-0.19652	0.08233	TRUE	1.00E+00
40735_at	-0.05709	-1.140486	0.090169	-0.47309	0.35892	TRUE	1.00E+00
34182_at	-0.05707	-1.140434	0.02586	-0.17638	0.06224	TRUE	1.00E+00
41832_s_at	-0.05704	-1.140355	0.061141	-0.33912	0.22505	TRUE	1.00E+00
38599_s_at	-0.05701	-1.140276	0.039071	-0.23726	0.12325	TRUE	1.00E+00
39979_at	-0.057	-1.14025	0.037187	-0.22856	0.11457	TRUE	1.00E+00
31738_at	-0.0569	-1.139987	0.033435	-0.21115	0.09736	TRUE	1.00E+00
38736_at	-0.05677	-1.139646	0.015971	-0.13046	0.01691	TRUE	1.00E+00
38009_at	-0.0567	-1.139462	0.051113	-0.29251	0.17912	TRUE	1.00E+00
41332_at	-0.05669	-1.139436	0.065068	-0.35689	0.24351	TRUE	1.00E+00
455_at	-0.05669	-1.139436	0.035014	-0.21822	0.10485	TRUE	1.00E+00
1584_at	-0.05668	-1.13941	0.050174	-0.28816	0.1748	TRUE	1.00E+00
31684_at	-0.05667	-1.139384	0.023201	-0.16371	0.05037	TRUE	1.00E+00
37119_at	-0.05665	-1.139331	0.021112	-0.15406	0.04075	TRUE	1.00E+00
40784_at	-0.05659	-1.139174	0.0123	-0.11334	0.00016	TRUE	5.32E-02
31627_f_at	-0.05658	-1.139148	0.040872	-0.24514	0.13199	TRUE	1.00E+00
34254_at	-0.05656	-1.139095	0.078311	-0.41785	0.30474	TRUE	1.00E+00
39389_at	-0.05654	-1.139043	0.027958	-0.18553	0.07245	TRUE	1.00E+00
31386_at	-0.05652	-1.13899	0.031523	-0.20196	0.08891	TRUE	1.00E+00
39266_at	-0.05643	-1.138754	0.114858	-0.58634	0.47347	TRUE	1.00E+00
41623_s_at	-0.0564	-1.138676	0.078905	-0.42043	0.30764	TRUE	1.00E+00
33826_at	-0.05636	-1.138571	0.032718	-0.20731	0.09459	TRUE	1.00E+00
35791_at	-0.05635	-1.138544	0.072198	-0.38944	0.27674	TRUE	1.00E+00
1134_at	-0.05635	-1.138544	0.02259	-0.16057	0.04787	TRUE	1.00E+00
32971_at	-0.05627	-1.138335	0.019357	-0.14557	0.03304	TRUE	1.00E+00
39525_at	-0.05617	-1.138073	0.025388	-0.1733	0.06096	TRUE	1.00E+00
39105_at	-0.05616	-1.138046	0.034932	-0.21732	0.10501	TRUE	1.00E+00
39288_at	-0.05615	-1.13802	0.062413	-0.3441	0.23179	TRUE	1.00E+00
32712_at	-0.05612	-1.137942	0.033943	-0.21272	0.10048	TRUE	1.00E+00
38839_at	-0.05611	-1.137915	0.009807	-0.10135	-0.01086	FALSE	1.34E-04
267_at	-0.05602	-1.13768	0.013026	-0.11612	0.00407	TRUE	2.15E-01
32913_i_at	-0.05596	-1.137523	0.033896	-0.21234	0.10042	TRUE	1.00E+00
33453_at	-0.05595	-1.137496	0.021526	-0.15526	0.04336	TRUE	1.00E+00
31886_at	-0.05581	-1.13713	0.073917	-0.39684	0.28521	TRUE	1.00E+00
40879_at	-0.05581	-1.13713	0.010361	-0.10361	-0.00801	FALSE	9.06E-04
34850_at	-0.0558	-1.137104	0.029478	-0.1918	0.0802	TRUE	1.00E+00
888_s_at	-0.0557	-1.136842	0.026062	-0.17594	0.06454	TRUE	1.00E+00
32505_at	-0.05563	-1.136658	0.034235	-0.21358	0.10232	TRUE	1.00E+00
457_s_at	-0.05561	-1.136606	0.008921	-0.09677	-0.01445	FALSE	5.74E-06
35185_at	-0.05561	-1.136606	0.033737	-0.21126	0.10004	TRUE	1.00E+00
1191_s_at	-0.05557	-1.136501	0.024224	-0.16733	0.05619	TRUE	1.00E+00

33678_i_at	-0.05556	-1.136475	0.03029	-0.1953	0.08419	TRUE	1.00E+00
36873_at	-0.0555	-1.136318	0.053126	-0.3006	0.1896	TRUE	1.00E+00
40658_r_at	-0.05549	-1.136292	0.105829	-0.54374	0.43276	TRUE	1.00E+00
32240_at	-0.05548	-1.136266	0.02351	-0.16395	0.05298	TRUE	1.00E+00
1773_at	-0.05546	-1.136214	0.017708	-0.13716	0.02623	TRUE	1.00E+00
39933_at	-0.05542	-1.136109	0.09248	-0.48208	0.37125	TRUE	1.00E+00
41127_at	-0.05534	-1.1359	0.03211	-0.20348	0.0928	TRUE	1.00E+00
34948_at	-0.05533	-1.135874	0.041816	-0.24825	0.13759	TRUE	1.00E+00
32679_at	-0.05531	-1.135821	0.02475	-0.16949	0.05888	TRUE	1.00E+00
32260_at	-0.0553	-1.135795	0.040294	-0.24119	0.1306	TRUE	1.00E+00
affx-bioc-5_at	-0.05528	-1.135743	0.057337	-0.31981	0.20925	TRUE	1.00E+00
33577_at	-0.05527	-1.135717	0.079907	-0.42393	0.31339	TRUE	1.00E+00
41296_s_at	-0.05526	-1.135691	0.036677	-0.22447	0.11395	TRUE	1.00E+00
776_at	-0.05524	-1.135638	0.027302	-0.1812	0.07072	TRUE	1.00E+00
31467_at	-0.05519	-1.135507	0.022497	-0.15898	0.04861	TRUE	1.00E+00
34685_at	-0.05518	-1.135481	0.027909	-0.18394	0.07358	TRUE	1.00E+00
635_s_at	-0.05516	-1.135429	0.02435	-0.1675	0.05718	TRUE	1.00E+00
33134_at	-0.05513	-1.135351	0.02522	-0.17148	0.06123	TRUE	1.00E+00
34306_at	-0.0551	-1.135272	0.015287	-0.12563	0.01542	TRUE	1.00E+00
36773_f_at	-0.0551	-1.135272	0.04985	-0.28509	0.17489	TRUE	1.00E+00
34480_at	-0.0551	-1.135272	0.035635	-0.2195	0.10931	TRUE	1.00E+00
38738_at	-0.05509	-1.135246	0.010799	-0.10491	-0.00527	FALSE	4.25E-03
35421_at	-0.05505	-1.135141	0.072896	-0.39136	0.28126	TRUE	1.00E+00
38624_at	-0.05502	-1.135063	0.010785	-0.10477	-0.00526	FALSE	4.26E-03
41211_at	-0.05498	-1.134959	0.046803	-0.27091	0.16095	TRUE	1.00E+00
34219_at	-0.05492	-1.134802	0.073732	-0.39509	0.28525	TRUE	1.00E+00
32764_at	-0.05486	-1.134645	0.048243	-0.27744	0.16771	TRUE	1.00E+00
33988_at	-0.05478	-1.134436	0.04538	-0.26415	0.15458	TRUE	1.00E+00
41070_r_at	-0.05478	-1.134436	0.040119	-0.23987	0.13031	TRUE	1.00E+00
41777_at	-0.05477	-1.13441	0.18769	-0.9207	0.81115	TRUE	1.00E+00
37415_at	-0.05474	-1.134332	0.031977	-0.20226	0.09279	TRUE	1.00E+00
36728_at	-0.05473	-1.134305	0.07703	-0.41012	0.30065	TRUE	1.00E+00
affx-hum_alu_at	-0.05465	-1.134096	0.021249	-0.15269	0.04338	TRUE	1.00E+00
31788_at	-0.05462	-1.134018	0.037477	-0.22753	0.11828	TRUE	1.00E+00
35981_at	-0.05458	-1.133914	0.045379	-0.26394	0.15478	TRUE	1.00E+00
37048_at	-0.05452	-1.133757	0.050508	-0.28754	0.17851	TRUE	1.00E+00
37192_at	-0.05447	-1.133627	0.020761	-0.15025	0.04132	TRUE	1.00E+00
32696_at	-0.05438	-1.133392	0.027489	-0.1812	0.07244	TRUE	1.00E+00
35152_at	-0.05438	-1.133392	0.016281	-0.12949	0.02074	TRUE	1.00E+00
41001_at	-0.05438	-1.133392	0.041208	-0.24449	0.13574	TRUE	1.00E+00
33277_at	-0.05436	-1.133339	0.020761	-0.15014	0.04142	TRUE	1.00E+00
40752_at	-0.05436	-1.133339	0.02695	-0.1787	0.06998	TRUE	1.00E+00
1678_g_at	-0.05435	-1.133313	0.052563	-0.29685	0.18816	TRUE	1.00E+00
41483_s_at	-0.05433	-1.133261	0.009546	-0.09838	-0.01029	FALSE	1.59E-04
37359_at	-0.05433	-1.133261	0.010241	-0.10158	-0.00709	FALSE	1.42E-03
31402_at	-0.05433	-1.133261	0.036297	-0.22179	0.11313	TRUE	1.00E+00
389_s_at	-0.05422	-1.132974	0.02927	-0.18926	0.08081	TRUE	1.00E+00
38381_at	-0.05422	-1.132974	0.033214	-0.20745	0.09902	TRUE	1.00E+00
2047_s_at	-0.0542	-1.132922	0.057792	-0.32083	0.21243	TRUE	1.00E+00
37543_at	-0.05418	-1.13287	0.033744	-0.20986	0.1015	TRUE	1.00E+00
1869_at	-0.0541	-1.132661	0.024563	-0.16743	0.05922	TRUE	1.00E+00

38588_at	-0.0541	-1.132661	0.0336	-0.20912	0.10092	TRUE	1.00E+00
1870_at	-0.05408	-1.132609	0.030012	-0.19255	0.08438	TRUE	1.00E+00
41423_at	-0.054	-1.1324	0.011984	-0.10929	0.00129	TRUE	8.33E-02
37788_at	-0.054	-1.1324	0.023561	-0.1627	0.0547	TRUE	1.00E+00
32543_at	-0.05399	-1.132374	0.050652	-0.28768	0.1797	TRUE	1.00E+00
32631_at	-0.05399	-1.132374	0.067873	-0.36713	0.25915	TRUE	1.00E+00
1160_at	-0.05398	-1.132348	0.012605	-0.11214	0.00417	TRUE	2.33E-01
37655_at	-0.05396	-1.132296	0.01743	-0.13437	0.02646	TRUE	1.00E+00
41247_at	-0.05394	-1.132244	0.036668	-0.22311	0.11523	TRUE	1.00E+00
632_at	-0.05393	-1.132218	0.01949	-0.14385	0.03599	TRUE	1.00E+00
34995_at	-0.05392	-1.132192	0.039198	-0.23477	0.12692	TRUE	1.00E+00
34866_at	-0.05392	-1.132192	0.028368	-0.1848	0.07696	TRUE	1.00E+00
31808_at	-0.05391	-1.132166	0.01091	-0.10425	-0.00358	FALSE	9.79E-03
40270_at	-0.05385	-1.132009	0.018901	-0.14106	0.03335	TRUE	1.00E+00
41374_at	-0.05385	-1.132009	0.025432	-0.17118	0.06348	TRUE	1.00E+00
40623_at	-0.05384	-1.131983	0.016108	-0.12815	0.02048	TRUE	1.00E+00
40348_s_at	-0.05382	-1.131931	0.042828	-0.25141	0.14377	TRUE	1.00E+00
41482_at	-0.05381	-1.131905	0.060227	-0.33168	0.22405	TRUE	1.00E+00
41632_at	-0.05375	-1.131749	0.018437	-0.13881	0.03131	TRUE	1.00E+00
36186_at	-0.05375	-1.131749	0.030828	-0.19598	0.08848	TRUE	1.00E+00
32874_at	-0.0537	-1.131618	0.022383	-0.15697	0.04956	TRUE	1.00E+00
38173_at	-0.05369	-1.131592	0.029372	-0.18921	0.08182	TRUE	1.00E+00
1700_at	-0.05369	-1.131592	0.030244	-0.19322	0.08585	TRUE	1.00E+00
34091_s_at	-0.05368	-1.131566	0.019702	-0.14458	0.03722	TRUE	1.00E+00
39392_at	-0.05363	-1.131436	0.015372	-0.12455	0.01729	TRUE	1.00E+00
40236_at	-0.05361	-1.131384	0.033923	-0.21011	0.1029	TRUE	1.00E+00
36652_at	-0.0536	-1.131358	0.027947	-0.18254	0.07533	TRUE	1.00E+00
38395_at	-0.05357	-1.13128	0.016269	-0.12863	0.02149	TRUE	1.00E+00
1926_at	-0.05357	-1.13128	0.038015	-0.22896	0.12181	TRUE	1.00E+00
37814_g_at	-0.05357	-1.13128	0.042713	-0.25063	0.14349	TRUE	1.00E+00
37027_at	-0.05357	-1.13128	0.01839	-0.13841	0.03128	TRUE	1.00E+00
39916_r_at	-0.05355	-1.131228	0.032645	-0.20415	0.09706	TRUE	1.00E+00
33978_at	-0.05354	-1.131202	0.103389	-0.53054	0.42345	TRUE	1.00E+00
36516_at	-0.05351	-1.131123	0.010806	-0.10337	-0.00366	FALSE	9.26E-03
34891_at	-0.05351	-1.131123	0.013876	-0.11753	0.01051	TRUE	1.00E+00
32295_at	-0.0535	-1.131097	0.060899	-0.33446	0.22746	TRUE	1.00E+00
410_s_at	-0.0535	-1.131097	0.025997	-0.17344	0.06644	TRUE	1.00E+00
33248_at	-0.05341	-1.130863	0.012333	-0.11031	0.00349	TRUE	1.88E-01
814_s_at	-0.05338	-1.130785	0.060705	-0.33345	0.22669	TRUE	1.00E+00
31502_at	-0.05336	-1.130733	0.02605	-0.17355	0.06682	TRUE	1.00E+00
33986_r_at	-0.05334	-1.130681	0.099047	-0.5103	0.40362	TRUE	1.00E+00
160028_s_at	-0.05331	-1.130603	0.04911	-0.27988	0.17326	TRUE	1.00E+00
40872_at	-0.0533	-1.130577	0.013715	-0.11658	0.00997	TRUE	1.00E+00
35297_at	-0.05329	-1.130551	0.02585	-0.17255	0.06597	TRUE	1.00E+00
38672_at	-0.05327	-1.130499	0.011665	-0.10709	0.00054	TRUE	6.25E-02
31911_at	-0.05326	-1.130472	0.042156	-0.24775	0.14123	TRUE	1.00E+00
40389_at	-0.05325	-1.130446	0.014313	-0.11929	0.01278	TRUE	1.00E+00
36640_at	-0.05315	-1.130186	0.024932	-0.16817	0.06187	TRUE	1.00E+00
33013_at	-0.05314	-1.13016	0.093942	-0.48655	0.38027	TRUE	1.00E+00
33205_at	-0.0531	-1.130056	0.053403	-0.29948	0.19327	TRUE	1.00E+00
34443_at	-0.05309	-1.13003	0.032063	-0.20101	0.09483	TRUE	1.00E+00

31973_at	-0.05308	-1.130004	0.038278	-0.22968	0.12351	TRUE	1.00E+00
36745_at	-0.05306	-1.129952	0.033015	-0.20538	0.09926	TRUE	1.00E+00
31425_g_at	-0.05306	-1.129952	0.080259	-0.42334	0.31722	TRUE	1.00E+00
1047_s_at	-0.05306	-1.129952	0.0348	-0.21361	0.10749	TRUE	1.00E+00
204_at	-0.05303	-1.129874	0.044433	-0.25803	0.15197	TRUE	1.00E+00
32381_at	-0.05303	-1.129874	0.052504	-0.29526	0.1892	TRUE	1.00E+00
35734_at	-0.05303	-1.129874	0.014115	-0.11815	0.01209	TRUE	1.00E+00
39929_at	-0.05302	-1.129848	0.024624	-0.16663	0.06058	TRUE	1.00E+00
35078_at	-0.05302	-1.129848	0.039357	-0.2346	0.12856	TRUE	1.00E+00
32076_at	-0.05297	-1.129718	0.044875	-0.26	0.15406	TRUE	1.00E+00
34409_at	-0.05295	-1.129666	0.014079	-0.11791	0.012	TRUE	1.00E+00
39726_at	-0.05294	-1.12964	0.045785	-0.26418	0.15829	TRUE	1.00E+00
726_f_at	-0.05293	-1.129614	0.020166	-0.14597	0.0401	TRUE	1.00E+00
1565_s_at	-0.05289	-1.12951	0.020952	-0.14955	0.04377	TRUE	1.00E+00
39235_at	-0.05287	-1.129458	0.043299	-0.25264	0.14689	TRUE	1.00E+00
38807_at	-0.05287	-1.129458	0.025775	-0.17178	0.06604	TRUE	1.00E+00
38285_at	-0.05287	-1.129458	0.040619	-0.24027	0.13453	TRUE	1.00E+00
40896_at	-0.05284	-1.12938	0.016932	-0.13096	0.02528	TRUE	1.00E+00
41728_at	-0.05284	-1.12938	0.023899	-0.1631	0.05742	TRUE	1.00E+00
33692_at	-0.05281	-1.129302	0.040869	-0.24136	0.13574	TRUE	1.00E+00
39417_at	-0.05278	-1.129224	0.041698	-0.24516	0.1396	TRUE	1.00E+00
35154_at	-0.05277	-1.129198	0.016965	-0.13104	0.0255	TRUE	1.00E+00
1141_at	-0.05277	-1.129198	0.02264	-0.15721	0.05168	TRUE	1.00E+00
41707_at	-0.05275	-1.129146	0.112277	-0.57075	0.46525	TRUE	1.00E+00
36085_at	-0.05274	-1.12912	0.038174	-0.22886	0.12338	TRUE	1.00E+00
38505_at	-0.0527	-1.129016	0.078368	-0.41426	0.30885	TRUE	1.00E+00
34680_s_at	-0.05264	-1.12886	0.0247	-0.16659	0.06131	TRUE	1.00E+00
35787_at	-0.05264	-1.12886	0.012244	-0.10912	0.00385	TRUE	2.17E-01
37685_at	-0.05261	-1.128782	0.016431	-0.12842	0.0232	TRUE	1.00E+00
327_f_at	-0.0526	-1.128756	0.015563	-0.1244	0.01921	TRUE	1.00E+00
2090_i_at	-0.05259	-1.12873	0.007788	-0.08852	-0.01666	FALSE	1.84E-07
1231_at	-0.05256	-1.128652	0.019022	-0.14032	0.0352	TRUE	1.00E+00
1631_at	-0.05247	-1.128418	0.024557	-0.16577	0.06083	TRUE	1.00E+00
32213_at	-0.05245	-1.128366	0.089217	-0.46406	0.35917	TRUE	1.00E+00
38893_at	-0.0524	-1.128236	0.033621	-0.20751	0.10271	TRUE	1.00E+00
32330_at	-0.05239	-1.12821	0.02721	-0.17793	0.07314	TRUE	1.00E+00
39972_at	-0.05237	-1.128158	0.03481	-0.21297	0.10823	TRUE	1.00E+00
36095_at	-0.05237	-1.128158	0.022343	-0.15545	0.05071	TRUE	1.00E+00
980_at	-0.05232	-1.128028	0.023159	-0.15917	0.05453	TRUE	1.00E+00
33537_at	-0.05228	-1.127924	0.039544	-0.23472	0.13016	TRUE	1.00E+00
33906_at	-0.05228	-1.127924	0.013764	-0.11578	0.01122	TRUE	1.00E+00
40260_g_at	-0.05227	-1.127898	0.037254	-0.22414	0.1196	TRUE	1.00E+00
40166_at	-0.05225	-1.127847	0.037201	-0.22388	0.11938	TRUE	1.00E+00
37195_at	-0.05221	-1.127743	0.012505	-0.10991	0.00548	TRUE	3.76E-01
37383_f_at	-0.0522	-1.127717	0.019359	-0.14151	0.03712	TRUE	1.00E+00
32010_at	-0.05219	-1.127691	0.050216	-0.28386	0.17949	TRUE	1.00E+00
36451_at	-0.05216	-1.127613	0.01889	-0.13931	0.03499	TRUE	1.00E+00
40971_at	-0.05208	-1.127405	0.044283	-0.25638	0.15222	TRUE	1.00E+00
40950_at	-0.05203	-1.127275	0.040888	-0.24067	0.13661	TRUE	1.00E+00
31669_s_at	-0.052	-1.127197	0.032889	-0.20374	0.09974	TRUE	1.00E+00
40947_at	-0.05199	-1.127172	0.035838	-0.21733	0.11335	TRUE	1.00E+00

31465_g_at	-0.05197	-1.12712	0.037955	-0.22708	0.12314	TRUE	1.00E+00
37619_at	-0.05195	-1.127068	0.01645	-0.12785	0.02394	TRUE	1.00E+00
32761_at	-0.05192	-1.12699	0.027767	-0.18002	0.07619	TRUE	1.00E+00
41801_at	-0.05184	-1.126782	0.030668	-0.19333	0.08965	TRUE	1.00E+00
35937_at	-0.05183	-1.126756	0.028802	-0.18471	0.08105	TRUE	1.00E+00
32065_at	-0.05183	-1.126756	0.020207	-0.14505	0.0414	TRUE	1.00E+00
32362_r_at	-0.05182	-1.12673	0.065098	-0.35215	0.24852	TRUE	1.00E+00
31829_r_at	-0.0518	-1.126678	0.028328	-0.18249	0.0789	TRUE	1.00E+00
1725_s_at	-0.05178	-1.126627	0.028493	-0.18324	0.07967	TRUE	1.00E+00
38987_at	-0.05177	-1.126601	0.013207	-0.11271	0.00916	TRUE	1.00E+00
36837_at	-0.05175	-1.126549	0.116804	-0.59064	0.48713	TRUE	1.00E+00
197_at	-0.05175	-1.126549	0.011659	-0.10554	0.00204	TRUE	1.14E-01
634_at	-0.05173	-1.126497	0.035914	-0.21742	0.11396	TRUE	1.00E+00
37385_at	-0.05169	-1.126393	0.027807	-0.17998	0.0766	TRUE	1.00E+00
34370_at	-0.05167	-1.126341	0.019394	-0.14115	0.0378	TRUE	1.00E+00
33610_at	-0.05166	-1.126315	0.093939	-0.48506	0.38173	TRUE	1.00E+00
347_s_at	-0.05162	-1.126212	0.026045	-0.17178	0.06854	TRUE	1.00E+00
35357_at	-0.05148	-1.125849	0.030199	-0.19081	0.08784	TRUE	1.00E+00
31564_at	-0.05144	-1.125745	0.048099	-0.27335	0.17047	TRUE	1.00E+00
41221_at	-0.05135	-1.125512	0.029929	-0.18943	0.08673	TRUE	1.00E+00
34726_at	-0.05132	-1.125434	0.030924	-0.19399	0.09135	TRUE	1.00E+00
35497_at	-0.05129	-1.125356	0.079061	-0.41604	0.31346	TRUE	1.00E+00
41083_at	-0.05121	-1.125149	0.037237	-0.223	0.12059	TRUE	1.00E+00
35661_g_at	-0.05105	-1.124734	0.033157	-0.20403	0.10192	TRUE	1.00E+00
37742_at	-0.05104	-1.124709	0.010734	-0.10057	-0.00152	FALSE	2.50E-02
32557_at	-0.05103	-1.124683	0.024939	-0.16608	0.06403	TRUE	1.00E+00
40613_at	-0.05102	-1.124657	0.024717	-0.16506	0.06301	TRUE	1.00E+00
40789_at	-0.051	-1.124605	0.033127	-0.20383	0.10184	TRUE	1.00E+00
35314_at	-0.05096	-1.124501	0.018742	-0.13743	0.03551	TRUE	1.00E+00
36358_at	-0.05092	-1.124398	0.012778	-0.10987	0.00803	TRUE	8.52E-01
830_at	-0.0509	-1.124346	0.061083	-0.33271	0.23091	TRUE	1.00E+00
41496_at	-0.05082	-1.124139	0.020404	-0.14496	0.04331	TRUE	1.00E+00
41452_at	-0.05079	-1.124061	0.037434	-0.22349	0.12192	TRUE	1.00E+00
39074_at	-0.05075	-1.123958	0.034034	-0.20777	0.10627	TRUE	1.00E+00
38764_at	-0.05074	-1.123932	0.01541	-0.12184	0.02035	TRUE	1.00E+00
32662_at	-0.05073	-1.123906	0.021718	-0.15093	0.04947	TRUE	1.00E+00
36587_at	-0.05068	-1.123777	0.017683	-0.13227	0.0309	TRUE	1.00E+00
39691_at	-0.05068	-1.123777	0.028157	-0.18059	0.07922	TRUE	1.00E+00
38759_at	-0.05064	-1.123673	0.08792	-0.45627	0.35499	TRUE	1.00E+00
39949_at	-0.0506	-1.12357	0.051921	-0.29015	0.18894	TRUE	1.00E+00
1331_s_at	-0.0506	-1.12357	0.071843	-0.38206	0.28085	TRUE	1.00E+00
34458_at	-0.05054	-1.123414	0.098934	-0.50698	0.40591	TRUE	1.00E+00
800_g_at	-0.05053	-1.123389	0.097264	-0.49926	0.39821	TRUE	1.00E+00
39423_f_at	-0.05049	-1.123285	0.038224	-0.22684	0.12586	TRUE	1.00E+00
38935_at	-0.05049	-1.123285	0.052837	-0.29426	0.19328	TRUE	1.00E+00
37323_r_at	-0.05048	-1.123259	0.021985	-0.15191	0.05095	TRUE	1.00E+00
39335_at	-0.05046	-1.123208	0.048293	-0.27327	0.17234	TRUE	1.00E+00
1188_g_at	-0.05046	-1.123208	0.072147	-0.38332	0.2824	TRUE	1.00E+00
35247_at	-0.05042	-1.123104	0.010447	-0.09861	-0.00222	FALSE	1.76E-02
34902_at	-0.05041	-1.123078	0.031163	-0.19419	0.09336	TRUE	1.00E+00
35272_at	-0.05039	-1.123026	0.019987	-0.14261	0.04182	TRUE	1.00E+00

41280_r_at	-0.05038	-1.123001	0.033478	-0.20483	0.10408	TRUE	1.00E+00
1449_at	-0.05036	-1.122949	0.014037	-0.11512	0.0144	TRUE	1.00E+00
31701_r_at	-0.05035	-1.122923	0.037438	-0.22307	0.12238	TRUE	1.00E+00
41617_at	-0.05034	-1.122897	0.035854	-0.21576	0.11508	TRUE	1.00E+00
1660_at	-0.05031	-1.12282	0.024954	-0.16544	0.06481	TRUE	1.00E+00
32865_at	-0.05029	-1.122768	0.112156	-0.56773	0.46716	TRUE	1.00E+00
40064_at	-0.05021	-1.122561	0.031814	-0.19699	0.09656	TRUE	1.00E+00
38495_s_at	-0.05014	-1.12238	0.074902	-0.3957	0.29543	TRUE	1.00E+00
33337_at	-0.05011	-1.122303	0.009389	-0.09343	-0.00679	FALSE	1.19E-03
39187_at	-0.05007	-1.122199	0.076288	-0.40203	0.30189	TRUE	1.00E+00
919_at	-0.05004	-1.122122	0.02815	-0.17991	0.07983	TRUE	1.00E+00
1366_i_at	-0.05003	-1.122096	0.01703	-0.1286	0.02854	TRUE	1.00E+00
32931_at	-0.05001	-1.122044	0.03894	-0.22967	0.12964	TRUE	1.00E+00
31505_at	-0.05001	-1.122044	0.017123	-0.12901	0.02899	TRUE	1.00E+00
36553_at	-0.05	-1.122018	0.032565	-0.20024	0.10024	TRUE	1.00E+00
35580_at	-0.05	-1.122018	0.0399	-0.23408	0.13408	TRUE	1.00E+00
37636_at	-0.04994	-1.121863	0.030237	-0.18944	0.08956	TRUE	1.00E+00
affx-bioc-3_at	-0.04994	-1.121863	0.070109	-0.37339	0.27352	TRUE	1.00E+00
32690_s_at	-0.0499	-1.12176	0.066857	-0.35835	0.25855	TRUE	1.00E+00
34710_r_at	-0.0499	-1.12176	0.167458	-0.82248	0.72268	TRUE	1.00E+00
31655_at	-0.0498	-1.121502	0.026558	-0.17233	0.07273	TRUE	1.00E+00
33858_at	-0.04977	-1.121424	0.047112	-0.26712	0.16759	TRUE	1.00E+00
39273_at	-0.04973	-1.121321	0.090998	-0.46955	0.3701	TRUE	1.00E+00
37977_at	-0.04971	-1.121269	0.027723	-0.17761	0.07819	TRUE	1.00E+00
34697_at	-0.0497	-1.121244	0.025151	-0.16573	0.06634	TRUE	1.00E+00
35646_at	-0.0497	-1.121244	0.036304	-0.21719	0.11779	TRUE	1.00E+00
33518_f_at	-0.04969	-1.121218	0.074572	-0.39374	0.29435	TRUE	1.00E+00
1470_at	-0.04964	-1.121089	0.019522	-0.13971	0.04043	TRUE	1.00E+00
1483_at	-0.04957	-1.120908	0.048972	-0.27551	0.17637	TRUE	1.00E+00
809_at	-0.04953	-1.120805	0.036031	-0.21576	0.1167	TRUE	1.00E+00
31935_s_at	-0.04952	-1.120779	0.020085	-0.14218	0.04315	TRUE	1.00E+00
35293_at	-0.04951	-1.120753	0.040877	-0.23809	0.13908	TRUE	1.00E+00
32535_at	-0.04949	-1.120702	0.028756	-0.18216	0.08318	TRUE	1.00E+00
38465_at	-0.04948	-1.120676	0.044097	-0.25293	0.15396	TRUE	1.00E+00
41108_at	-0.04943	-1.120547	0.04267	-0.2463	0.14743	TRUE	1.00E+00
41094_at	-0.04938	-1.120418	0.104178	-0.53002	0.43125	TRUE	1.00E+00
1069_at	-0.04932	-1.120263	0.106707	-0.54162	0.44298	TRUE	1.00E+00
1140_at	-0.04931	-1.120237	0.016584	-0.12582	0.0272	TRUE	1.00E+00
1026_s_at	-0.04931	-1.120237	0.029774	-0.18667	0.08806	TRUE	1.00E+00
1137_at	-0.04929	-1.120186	0.038389	-0.2264	0.12783	TRUE	1.00E+00
32244_at	-0.04928	-1.12016	0.014052	-0.11411	0.01555	TRUE	1.00E+00
32227_at	-0.04926	-1.120108	0.015191	-0.11934	0.02083	TRUE	1.00E+00
36754_at	-0.04924	-1.120057	0.031967	-0.19672	0.09824	TRUE	1.00E+00
41359_at	-0.04918	-1.119902	0.023732	-0.15866	0.06031	TRUE	1.00E+00
32212_at	-0.04916	-1.11985	0.022807	-0.15438	0.05606	TRUE	1.00E+00
40738_at	-0.04913	-1.119773	0.022997	-0.15523	0.05697	TRUE	1.00E+00
38538_at	-0.04907	-1.119618	0.028425	-0.18022	0.08207	TRUE	1.00E+00
36988_at	-0.04906	-1.119593	0.021396	-0.14777	0.04965	TRUE	1.00E+00
38824_at	-0.04902	-1.119489	0.032535	-0.19913	0.10108	TRUE	1.00E+00
37020_at	-0.04902	-1.119489	0.095942	-0.49166	0.39362	TRUE	1.00E+00
40575_at	-0.04901	-1.119464	0.013692	-0.11218	0.01416	TRUE	1.00E+00

33218_at	-0.04899	-1.119412	0.022519	-0.15288	0.05491	TRUE	1.00E+00
39287_at	-0.04888	-1.119129	0.097431	-0.49839	0.40062	TRUE	1.00E+00
1546_at	-0.04886	-1.119077	0.119935	-0.60219	0.50448	TRUE	1.00E+00
2027_at	-0.04884	-1.119026	0.02609	-0.16921	0.07153	TRUE	1.00E+00
928_at	-0.04884	-1.119026	0.105304	-0.53467	0.43699	TRUE	1.00E+00
38550_at	-0.04879	-1.118897	0.080283	-0.41918	0.3216	TRUE	1.00E+00
1225_g_at	-0.04877	-1.118845	0.060822	-0.32938	0.23184	TRUE	1.00E+00
32329_at	-0.04875	-1.118794	0.025306	-0.1655	0.068	TRUE	1.00E+00
40794_at	-0.04875	-1.118794	0.081017	-0.42253	0.32503	TRUE	1.00E+00
41267_at	-0.04874	-1.118768	0.021407	-0.1475	0.05002	TRUE	1.00E+00
35227_at	-0.04869	-1.118639	0.018377	-0.13348	0.03609	TRUE	1.00E+00
40905_s_at	-0.04869	-1.118639	0.023805	-0.15852	0.06114	TRUE	1.00E+00
263_g_at	-0.04867	-1.118588	0.021524	-0.14797	0.05064	TRUE	1.00E+00
38290_at	-0.04865	-1.118536	0.057363	-0.3133	0.21599	TRUE	1.00E+00
1764_s_at	-0.04863	-1.118485	0.046267	-0.26209	0.16483	TRUE	1.00E+00
34415_at	-0.04858	-1.118356	0.031344	-0.19318	0.09603	TRUE	1.00E+00
41534_at	-0.04856	-1.118304	0.05169	-0.28704	0.18991	TRUE	1.00E+00
33343_at	-0.04855	-1.118279	0.015959	-0.12218	0.02508	TRUE	1.00E+00
1896_s_at	-0.04853	-1.118227	0.016905	-0.12652	0.02946	TRUE	1.00E+00
36004_at	-0.04852	-1.118201	0.031597	-0.1943	0.09725	TRUE	1.00E+00
38726_at	-0.04851	-1.118176	0.018928	-0.13584	0.03881	TRUE	1.00E+00
31336_at	-0.04851	-1.118176	0.125742	-0.62863	0.53162	TRUE	1.00E+00
36433_at	-0.04843	-1.11797	0.065636	-0.35124	0.25439	TRUE	1.00E+00
33216_at	-0.04842	-1.117944	0.017644	-0.12982	0.03299	TRUE	1.00E+00
34746_at	-0.04839	-1.117867	0.041996	-0.24214	0.14536	TRUE	1.00E+00
371_at	-0.04828	-1.117584	0.011397	-0.10086	0.0043	TRUE	2.87E-01
38149_at	-0.04828	-1.117584	0.015918	-0.12172	0.02516	TRUE	1.00E+00
1769_at	-0.04827	-1.117558	0.05427	-0.29865	0.2021	TRUE	1.00E+00
2040_s_at	-0.04821	-1.117403	0.065175	-0.34891	0.25248	TRUE	1.00E+00
39002_at	-0.0482	-1.117378	0.019857	-0.13981	0.04341	TRUE	1.00E+00
41151_at	-0.0482	-1.117378	0.015312	-0.11884	0.02245	TRUE	1.00E+00
41477_at	-0.04817	-1.117301	0.111612	-0.56311	0.46676	TRUE	1.00E+00
32117_at	-0.04817	-1.117301	0.025857	-0.16746	0.07112	TRUE	1.00E+00
36994_at	-0.04816	-1.117275	0.016763	-0.1255	0.02918	TRUE	1.00E+00
37414_at	-0.04815	-1.117249	0.049767	-0.27776	0.18145	TRUE	1.00E+00
1041_at	-0.0481	-1.11712	0.06856	-0.36441	0.2682	TRUE	1.00E+00
35404_at	-0.04807	-1.117043	0.116073	-0.58358	0.48745	TRUE	1.00E+00
390_at	-0.04805	-1.116992	0.049789	-0.27776	0.18165	TRUE	1.00E+00
33973_at	-0.04801	-1.116889	0.05507	-0.30208	0.20606	TRUE	1.00E+00
35907_at	-0.04799	-1.116838	0.055262	-0.30295	0.20696	TRUE	1.00E+00
32442_at	-0.04799	-1.116838	0.045924	-0.25986	0.16388	TRUE	1.00E+00
36102_at	-0.04796	-1.11676	0.022547	-0.15198	0.05606	TRUE	1.00E+00
36566_at	-0.04796	-1.11676	0.031601	-0.19376	0.09783	TRUE	1.00E+00
37769_at	-0.04794	-1.116709	0.020166	-0.14098	0.0451	TRUE	1.00E+00
727_at	-0.04793	-1.116683	0.025429	-0.16525	0.06939	TRUE	1.00E+00
36292_at	-0.04791	-1.116632	0.038994	-0.22782	0.13199	TRUE	1.00E+00
32627_at	-0.04791	-1.116632	0.025624	-0.16613	0.07031	TRUE	1.00E+00
38061_at	-0.04788	-1.116555	0.028321	-0.17854	0.08278	TRUE	1.00E+00
33853_s_at	-0.04787	-1.116529	0.08357	-0.43343	0.33769	TRUE	1.00E+00
33087_s_at	-0.04785	-1.116478	0.039754	-0.23126	0.13556	TRUE	1.00E+00
33245_at	-0.04784	-1.116452	0.108581	-0.54878	0.45311	TRUE	1.00E+00

38418_at	-0.04776	-1.116246	0.027942	-0.17667	0.08116	TRUE	1.00E+00
36750_at	-0.04774	-1.116195	0.040277	-0.23356	0.13809	TRUE	1.00E+00
36169_at	-0.0477	-1.116092	0.013948	-0.11205	0.01665	TRUE	1.00E+00
40793_s_at	-0.0477	-1.116092	0.11771	-0.59077	0.49537	TRUE	1.00E+00
31597_r_at	-0.04756	-1.115732	0.027231	-0.1732	0.07807	TRUE	1.00E+00
34147_g_at	-0.04753	-1.115655	0.022484	-0.15127	0.0562	TRUE	1.00E+00
32595_at	-0.04753	-1.115655	0.02185	-0.14833	0.05328	TRUE	1.00E+00
34277_at	-0.04752	-1.11563	0.016739	-0.12475	0.0297	TRUE	1.00E+00
39570_at	-0.04752	-1.11563	0.010189	-0.09452	-0.00051	FALSE	3.92E-02
37490_at	-0.0475	-1.115578	0.017951	-0.13032	0.03531	TRUE	1.00E+00
35473_at	-0.04742	-1.115373	0.060375	-0.32597	0.23112	TRUE	1.00E+00
40331_at	-0.04739	-1.115296	0.020742	-0.14309	0.0483	TRUE	1.00E+00
294_s_at	-0.04739	-1.115296	0.030882	-0.18987	0.09509	TRUE	1.00E+00
37152_at	-0.04737	-1.115244	0.026571	-0.16996	0.07522	TRUE	1.00E+00
32157_at	-0.04732	-1.115116	0.010256	-0.09464	-0.00001	FALSE	4.98E-02
32835_at	-0.04732	-1.115116	0.012745	-0.10612	0.01148	TRUE	1.00E+00
880_at	-0.04731	-1.11509	0.017745	-0.12918	0.03456	TRUE	1.00E+00
38191_at	-0.04728	-1.115013	0.027889	-0.17594	0.08139	TRUE	1.00E+00
33016_at	-0.04726	-1.114962	0.016978	-0.12559	0.03106	TRUE	1.00E+00
32681_at	-0.04725	-1.114936	0.018582	-0.13298	0.03849	TRUE	1.00E+00
41863_at	-0.04717	-1.114731	0.087945	-0.45291	0.35857	TRUE	1.00E+00
40263_at	-0.04713	-1.114628	0.026562	-0.16967	0.07542	TRUE	1.00E+00
32373_at	-0.04709	-1.114525	0.056352	-0.30708	0.21289	TRUE	1.00E+00
33882_at	-0.04709	-1.114525	0.03399	-0.20391	0.10973	TRUE	1.00E+00
32617_at	-0.04708	-1.1145	0.065712	-0.35025	0.25609	TRUE	1.00E+00
39569_at	-0.04707	-1.114474	0.034569	-0.20656	0.11242	TRUE	1.00E+00
38376_at	-0.04702	-1.114346	0.036328	-0.21462	0.12059	TRUE	1.00E+00
36565_at	-0.04694	-1.114141	0.028006	-0.17615	0.08226	TRUE	1.00E+00
40780_at	-0.04693	-1.114115	0.021273	-0.14508	0.05122	TRUE	1.00E+00
38091_at	-0.04679	-1.113756	0.029496	-0.18287	0.08929	TRUE	1.00E+00
31330_at	-0.04679	-1.113756	0.021509	-0.14602	0.05245	TRUE	1.00E+00
33280_r_at	-0.04678	-1.11373	0.045517	-0.25678	0.16322	TRUE	1.00E+00
31313_at	-0.04674	-1.113628	0.017111	-0.12568	0.03221	TRUE	1.00E+00
40286_r_at	-0.04672	-1.113576	0.019406	-0.13625	0.04281	TRUE	1.00E+00
40811_at	-0.0467	-1.113525	0.033369	-0.20066	0.10725	TRUE	1.00E+00
37602_at	-0.04668	-1.113474	0.081184	-0.42123	0.32787	TRUE	1.00E+00
35179_at	-0.04668	-1.113474	0.03091	-0.18928	0.09593	TRUE	1.00E+00
1209_at	-0.04667	-1.113448	0.028551	-0.17839	0.08505	TRUE	1.00E+00
37620_at	-0.04667	-1.113448	0.039326	-0.2281	0.13477	TRUE	1.00E+00
40886_at	-0.04666	-1.113423	0.01671	-0.12375	0.03044	TRUE	1.00E+00
37118_at	-0.04663	-1.113346	0.08659	-0.44612	0.35286	TRUE	1.00E+00
33425_at	-0.04663	-1.113346	0.0207	-0.14213	0.04887	TRUE	1.00E+00
33406_at	-0.0466	-1.113269	0.02871	-0.17906	0.08585	TRUE	1.00E+00
40815_g_at	-0.04658	-1.113217	0.012654	-0.10496	0.0118	TRUE	1.00E+00
39370_at	-0.04657	-1.113192	0.029222	-0.18139	0.08825	TRUE	1.00E+00
37404_at	-0.04653	-1.113089	0.022775	-0.15161	0.05854	TRUE	1.00E+00
37451_at	-0.04651	-1.113038	0.103406	-0.52358	0.43057	TRUE	1.00E+00
34106_at	-0.04649	-1.112987	0.055617	-0.30309	0.2101	TRUE	1.00E+00
41206_r_at	-0.04644	-1.112859	0.016401	-0.12211	0.02923	TRUE	1.00E+00
32147_at	-0.0464	-1.112756	0.102282	-0.51829	0.42549	TRUE	1.00E+00
34548_at	-0.04638	-1.112705	0.099837	-0.50699	0.41423	TRUE	1.00E+00

37343_at	-0.04637	-1.112679	0.038337	-0.22324	0.1305	TRUE	1.00E+00
35482_at	-0.04637	-1.112679	0.084565	-0.43652	0.34378	TRUE	1.00E+00
31775_at	-0.04636	-1.112654	0.030644	-0.18774	0.09502	TRUE	1.00E+00
40881_at	-0.04629	-1.112474	0.020989	-0.14313	0.05054	TRUE	1.00E+00
41013_at	-0.04621	-1.112269	0.025844	-0.16544	0.07302	TRUE	1.00E+00
40761_at	-0.04619	-1.112218	0.094526	-0.48229	0.38992	TRUE	1.00E+00
551_at	-0.04609	-1.111962	0.022398	-0.14943	0.05724	TRUE	1.00E+00
614_at	-0.04607	-1.111911	0.06045	-0.32496	0.23282	TRUE	1.00E+00
33030_at	-0.04605	-1.11186	0.025537	-0.16387	0.07176	TRUE	1.00E+00
35193_at	-0.04604	-1.111834	0.037636	-0.21967	0.1276	TRUE	1.00E+00
36987_at	-0.04604	-1.111834	0.03223	-0.19473	0.10266	TRUE	1.00E+00
32279_at	-0.04603	-1.111809	0.064416	-0.34322	0.25116	TRUE	1.00E+00
40052_at	-0.04603	-1.111809	0.010905	-0.09634	0.00428	TRUE	3.07E-01
38081_at	-0.046	-1.111732	0.025459	-0.16346	0.07146	TRUE	1.00E+00
33023_at	-0.04599	-1.111706	0.078507	-0.40819	0.3162	TRUE	1.00E+00
33151_s_at	-0.04599	-1.111706	0.046308	-0.25964	0.16765	TRUE	1.00E+00
35490_at	-0.04598	-1.111681	0.089118	-0.45713	0.36517	TRUE	1.00E+00
38263_at	-0.04597	-1.111655	0.183394	-0.89208	0.80013	TRUE	1.00E+00
35198_at	-0.04593	-1.111553	0.025671	-0.16436	0.07251	TRUE	1.00E+00
39005_s_at	-0.04591	-1.111501	0.013946	-0.11025	0.01843	TRUE	1.00E+00
41101_at	-0.0459	-1.111476	0.028246	-0.17622	0.08442	TRUE	1.00E+00
33900_at	-0.04588	-1.111425	0.050258	-0.27775	0.18599	TRUE	1.00E+00
31873_at	-0.04585	-1.111348	0.045428	-0.25544	0.16374	TRUE	1.00E+00
41115_s_at	-0.04581	-1.111245	0.017677	-0.12736	0.03575	TRUE	1.00E+00
32520_at	-0.04577	-1.111143	0.04499	-0.25333	0.1618	TRUE	1.00E+00
39531_at	-0.04574	-1.111066	0.026654	-0.16871	0.07723	TRUE	1.00E+00
41161_at	-0.04565	-1.110836	0.038261	-0.22217	0.13087	TRUE	1.00E+00
1091_at	-0.04563	-1.110785	0.03515	-0.2078	0.11654	TRUE	1.00E+00
36967_g_at	-0.04562	-1.110759	0.12248	-0.61069	0.51945	TRUE	1.00E+00
34678_at	-0.04557	-1.110632	0.01878	-0.13222	0.04107	TRUE	1.00E+00
affx-humrge/m10	-0.04555	-1.11058	0.077782	-0.40441	0.3133	TRUE	1.00E+00
35723_at	-0.04548	-1.110401	0.033163	-0.19848	0.10752	TRUE	1.00E+00
1752_at	-0.04546	-1.11035	0.079047	-0.41015	0.31923	TRUE	1.00E+00
39129_at	-0.04541	-1.110222	0.010789	-0.09519	0.00436	TRUE	3.24E-01
36427_at	-0.04539	-1.110171	0.03715	-0.21678	0.12601	TRUE	1.00E+00
33690_at	-0.04534	-1.110044	0.029774	-0.18271	0.09203	TRUE	1.00E+00
36232_at	-0.04529	-1.109916	0.068107	-0.3595	0.26893	TRUE	1.00E+00
1649_at	-0.04528	-1.10989	0.054694	-0.29762	0.20705	TRUE	1.00E+00
835_at	-0.04523	-1.109762	0.023967	-0.15581	0.06534	TRUE	1.00E+00
274_at	-0.0452	-1.109686	0.029639	-0.18194	0.09154	TRUE	1.00E+00
33915_at	-0.04519	-1.10966	0.010519	-0.09372	0.00334	TRUE	2.20E-01
34613_at	-0.04516	-1.109584	0.012942	-0.10487	0.01455	TRUE	1.00E+00
38776_at	-0.04516	-1.109584	0.040475	-0.23189	0.14158	TRUE	1.00E+00
33480_at	-0.04515	-1.109558	0.062129	-0.33179	0.24149	TRUE	1.00E+00
39573_at	-0.04515	-1.109558	0.057743	-0.31155	0.22125	TRUE	1.00E+00
40655_at	-0.04514	-1.109532	0.131231	-0.65059	0.5603	TRUE	1.00E+00
39029_at	-0.0451	-1.10943	0.013936	-0.10939	0.01919	TRUE	1.00E+00
34803_at	-0.04505	-1.109303	0.015049	-0.11448	0.02438	TRUE	1.00E+00
41758_at	-0.04491	-1.108945	0.011393	-0.09747	0.00766	TRUE	1.00E+00
35823_at	-0.04489	-1.108894	0.023476	-0.1532	0.06342	TRUE	1.00E+00
1979_s_at	-0.04488	-1.108868	0.037415	-0.2175	0.12774	TRUE	1.00E+00

40619_at	-0.04488	-1.108868	0.048958	-0.27075	0.18099	TRUE	1.00E+00
39139_at	-0.04481	-1.10869	0.026485	-0.16701	0.07738	TRUE	1.00E+00
41377_f_at	-0.04479	-1.108639	0.128373	-0.63705	0.54747	TRUE	1.00E+00
32453_at	-0.04478	-1.108613	0.040753	-0.2328	0.14324	TRUE	1.00E+00
32036_i_at	-0.04478	-1.108613	0.022242	-0.14739	0.05784	TRUE	1.00E+00
40506_s_at	-0.04478	-1.108613	0.025522	-0.16253	0.07297	TRUE	1.00E+00
31575_f_at	-0.04477	-1.108588	0.020812	-0.14079	0.05125	TRUE	1.00E+00
32499_at	-0.04477	-1.108588	0.027296	-0.1707	0.08116	TRUE	1.00E+00
39970_at	-0.04477	-1.108588	0.079005	-0.40926	0.31973	TRUE	1.00E+00
38023_at	-0.04474	-1.108511	0.06197	-0.33065	0.24116	TRUE	1.00E+00
36978_at	-0.04472	-1.10846	0.018964	-0.13221	0.04278	TRUE	1.00E+00
38025_r_at	-0.04463	-1.10823	0.087094	-0.44644	0.35719	TRUE	1.00E+00
41833_at	-0.04462	-1.108205	0.0336	-0.19964	0.11039	TRUE	1.00E+00
38205_at	-0.04459	-1.108128	0.03975	-0.22798	0.13881	TRUE	1.00E+00
35793_at	-0.04451	-1.107924	0.022616	-0.14885	0.05984	TRUE	1.00E+00
40233_at	-0.0445	-1.107899	0.023376	-0.15235	0.06334	TRUE	1.00E+00
31498_f_at	-0.0445	-1.107899	0.069174	-0.36363	0.27464	TRUE	1.00E+00
31692_at	-0.04449	-1.107873	0.031303	-0.18891	0.09993	TRUE	1.00E+00
38851_at	-0.04445	-1.107771	0.0186	-0.13026	0.04136	TRUE	1.00E+00
38682_at	-0.04442	-1.107695	0.073293	-0.38256	0.29372	TRUE	1.00E+00
33928_r_at	-0.04442	-1.107695	0.161467	-0.78936	0.70053	TRUE	1.00E+00
33992_at	-0.04439	-1.107618	0.055927	-0.30241	0.21363	TRUE	1.00E+00
34848_at	-0.04432	-1.107439	0.038975	-0.22413	0.1355	TRUE	1.00E+00
36671_at	-0.0443	-1.107388	0.021797	-0.14486	0.05627	TRUE	1.00E+00
41757_at	-0.04428	-1.107337	0.023986	-0.15494	0.06638	TRUE	1.00E+00
38806_at	-0.04426	-1.107286	0.063255	-0.3361	0.24757	TRUE	1.00E+00
31753_at	-0.04419	-1.107108	0.025116	-0.16006	0.07168	TRUE	1.00E+00
41194_at	-0.04415	-1.107006	0.017874	-0.12661	0.03831	TRUE	1.00E+00
33133_at	-0.04413	-1.106955	0.022503	-0.14795	0.05969	TRUE	1.00E+00
297_g_at	-0.04409	-1.106853	0.014999	-0.11329	0.0251	TRUE	1.00E+00
35229_at	-0.04409	-1.106853	0.032863	-0.1957	0.10753	TRUE	1.00E+00
36018_at	-0.04407	-1.106802	0.091224	-0.46494	0.3768	TRUE	1.00E+00
32808_at	-0.04403	-1.1067	0.011057	-0.09504	0.00698	TRUE	8.63E-01
39879_s_at	-0.04397	-1.106547	0.017214	-0.12338	0.03545	TRUE	1.00E+00
33224_at	-0.04395	-1.106496	0.04221	-0.23868	0.15079	TRUE	1.00E+00
36968_s_at	-0.04391	-1.106394	0.024979	-0.15916	0.07133	TRUE	1.00E+00
34400_at	-0.04387	-1.106293	0.011902	-0.09877	0.01104	TRUE	1.00E+00
37932_at	-0.04385	-1.106242	0.010054	-0.09024	0.00254	TRUE	1.63E-01
39412_at	-0.04385	-1.106242	0.021163	-0.14149	0.05379	TRUE	1.00E+00
37618_at	-0.0438	-1.106114	0.039321	-0.22521	0.13761	TRUE	1.00E+00
33763_at	-0.04376	-1.106012	0.04874	-0.26862	0.18111	TRUE	1.00E+00
35954_at	-0.04375	-1.105987	0.065912	-0.34784	0.26034	TRUE	1.00E+00
35753_at	-0.04366	-1.105758	0.017992	-0.12666	0.03935	TRUE	1.00E+00
36972_at	-0.0436	-1.105605	0.024795	-0.158	0.0708	TRUE	1.00E+00
40026_g_at	-0.04359	-1.10558	0.029999	-0.182	0.09481	TRUE	1.00E+00
40861_at	-0.04357	-1.105529	0.010458	-0.09182	0.00467	TRUE	3.90E-01
242_at	-0.04352	-1.105401	0.02972	-0.18064	0.09359	TRUE	1.00E+00
37314_at	-0.04352	-1.105401	0.09426	-0.4784	0.39136	TRUE	1.00E+00
35777_at	-0.04345	-1.105223	0.017469	-0.12405	0.03714	TRUE	1.00E+00
40083_at	-0.04343	-1.105172	0.031966	-0.19091	0.10405	TRUE	1.00E+00
37092_at	-0.04342	-1.105147	0.093461	-0.47461	0.38778	TRUE	1.00E+00

35899_at	-0.04341	-1.105121	0.013046	-0.10359	0.01678	TRUE	1.00E+00
34372_at	-0.0434	-1.105096	0.026691	-0.16654	0.07974	TRUE	1.00E+00
38158_at	-0.04337	-1.10502	0.043518	-0.24414	0.15741	TRUE	1.00E+00
41502_at	-0.04336	-1.104994	0.048849	-0.26873	0.18201	TRUE	1.00E+00
32734_at	-0.04334	-1.104943	0.01647	-0.11933	0.03265	TRUE	1.00E+00
34971_at	-0.04334	-1.104943	0.019502	-0.13331	0.04664	TRUE	1.00E+00
308_f_at	-0.04334	-1.104943	0.083012	-0.42632	0.33965	TRUE	1.00E+00
41008_at	-0.04333	-1.104918	0.094841	-0.48089	0.39423	TRUE	1.00E+00
34572_at	-0.04332	-1.104892	0.053683	-0.29099	0.20436	TRUE	1.00E+00
31323_r_at	-0.0433	-1.104842	0.037789	-0.21764	0.13105	TRUE	1.00E+00
32034_at	-0.0433	-1.104842	0.026271	-0.1645	0.07791	TRUE	1.00E+00
39798_at	-0.04329	-1.104816	0.071672	-0.37396	0.28737	TRUE	1.00E+00
31772_at	-0.04326	-1.10474	0.029847	-0.18096	0.09444	TRUE	1.00E+00
32981_at	-0.04324	-1.104689	0.05889	-0.31493	0.22845	TRUE	1.00E+00
40264_g_at	-0.04323	-1.104663	0.065337	-0.34467	0.25821	TRUE	1.00E+00
37782_at	-0.04323	-1.104663	0.037744	-0.21736	0.1309	TRUE	1.00E+00
32799_at	-0.04316	-1.104485	0.026291	-0.16446	0.07813	TRUE	1.00E+00
1842_at	-0.04314	-1.104435	0.052402	-0.2849	0.19862	TRUE	1.00E+00
34694_at	-0.04314	-1.104435	0.020497	-0.1377	0.05143	TRUE	1.00E+00
40291_r_at	-0.04311	-1.104358	0.059643	-0.31828	0.23206	TRUE	1.00E+00
1240_at	-0.04311	-1.104358	0.043516	-0.24387	0.15766	TRUE	1.00E+00
40253_at	-0.04309	-1.104307	0.082315	-0.42286	0.33668	TRUE	1.00E+00
32702_at	-0.04307	-1.104257	0.058735	-0.31405	0.22792	TRUE	1.00E+00
1332_f_at	-0.04302	-1.104129	0.031516	-0.18843	0.10238	TRUE	1.00E+00
469_at	-0.043	-1.104079	0.027711	-0.17085	0.08484	TRUE	1.00E+00
678_at	-0.043	-1.104079	0.015668	-0.11528	0.02929	TRUE	1.00E+00
38549_at	-0.04299	-1.104053	0.071955	-0.37496	0.28898	TRUE	1.00E+00
37541_at	-0.04298	-1.104028	0.03069	-0.18458	0.09861	TRUE	1.00E+00
33295_at	-0.04298	-1.104028	0.046134	-0.25582	0.16987	TRUE	1.00E+00
37516_at	-0.04295	-1.103952	0.02569	-0.16147	0.07558	TRUE	1.00E+00
1493_r_at	-0.04292	-1.103875	0.072622	-0.37797	0.29213	TRUE	1.00E+00
40764_at	-0.04291	-1.10385	0.015212	-0.11309	0.02727	TRUE	1.00E+00
40448_at	-0.04291	-1.10385	0.056424	-0.30323	0.21741	TRUE	1.00E+00
40549_at	-0.04287	-1.103748	0.017562	-0.12389	0.03815	TRUE	1.00E+00
38742_s_at	-0.04283	-1.103647	0.043508	-0.24356	0.15789	TRUE	1.00E+00
37607_at	-0.04279	-1.103545	0.048462	-0.26638	0.18079	TRUE	1.00E+00
40191_s_at	-0.04279	-1.103545	0.038085	-0.2185	0.13292	TRUE	1.00E+00
32324_at	-0.04279	-1.103545	0.018042	-0.12602	0.04045	TRUE	1.00E+00
1417_at	-0.04274	-1.103418	0.131018	-0.64721	0.56172	TRUE	1.00E+00
37879_r_at	-0.04274	-1.103418	0.055019	-0.29657	0.2111	TRUE	1.00E+00
31515_at	-0.04273	-1.103392	0.028484	-0.17415	0.08868	TRUE	1.00E+00
41763_g_at	-0.0427	-1.103316	0.014805	-0.11101	0.0256	TRUE	1.00E+00
549_at	-0.04267	-1.10324	0.014872	-0.11129	0.02594	TRUE	1.00E+00
36023_at	-0.04266	-1.103215	0.010277	-0.09007	0.00476	TRUE	4.19E-01
36458_at	-0.04261	-1.103088	0.032696	-0.19346	0.10823	TRUE	1.00E+00
1296_at	-0.04253	-1.102884	0.030377	-0.18268	0.09761	TRUE	1.00E+00
41776_at	-0.04249	-1.102783	0.014557	-0.10965	0.02467	TRUE	1.00E+00
345_at	-0.04247	-1.102732	0.033206	-0.19567	0.11073	TRUE	1.00E+00
34395_at	-0.04243	-1.10263	0.022123	-0.14449	0.05964	TRUE	1.00E+00
32233_at	-0.0424	-1.102554	0.020978	-0.13919	0.05438	TRUE	1.00E+00
39242_at	-0.04236	-1.102453	0.024727	-0.15644	0.07172	TRUE	1.00E+00

35309_at	-0.04234	-1.102402	0.017389	-0.12257	0.03788	TRUE	1.00E+00
40591_at	-0.04225	-1.102174	0.024426	-0.15494	0.07044	TRUE	1.00E+00
35891_at	-0.04224	-1.102148	0.078195	-0.40299	0.31852	TRUE	1.00E+00
38933_at	-0.04218	-1.101996	0.096864	-0.48907	0.40471	TRUE	1.00E+00
39660_at	-0.04215	-1.10192	0.021769	-0.14258	0.05828	TRUE	1.00E+00
37912_at	-0.04215	-1.10192	0.014903	-0.1109	0.02661	TRUE	1.00E+00
1187_at	-0.0421	-1.101793	0.026598	-0.16482	0.08061	TRUE	1.00E+00
32440_at	-0.04209	-1.101768	0.026676	-0.16516	0.08099	TRUE	1.00E+00
33001_s_at	-0.04203	-1.101615	0.098948	-0.49854	0.41447	TRUE	1.00E+00
40685_at	-0.04202	-1.10159	0.04077	-0.23012	0.14607	TRUE	1.00E+00
37421_f_at	-0.04201	-1.101565	0.040744	-0.22998	0.14597	TRUE	1.00E+00
813_at	-0.0419	-1.101286	0.094173	-0.47638	0.39257	TRUE	1.00E+00
40088_at	-0.0419	-1.101286	0.016223	-0.11675	0.03295	TRUE	1.00E+00
1008_f_at	-0.04189	-1.10126	0.014407	-0.10835	0.02458	TRUE	1.00E+00
38660_at	-0.04188	-1.101235	0.038884	-0.22128	0.13751	TRUE	1.00E+00
35923_at	-0.04186	-1.101184	0.016256	-0.11686	0.03313	TRUE	1.00E+00
34138_at	-0.04186	-1.101184	0.057119	-0.30538	0.22167	TRUE	1.00E+00
40390_at	-0.04185	-1.101159	0.099107	-0.49909	0.41539	TRUE	1.00E+00
39694_at	-0.04185	-1.101159	0.024208	-0.15353	0.06984	TRUE	1.00E+00
1463_at	-0.04182	-1.101083	0.015938	-0.11535	0.03171	TRUE	1.00E+00
32867_at	-0.04181	-1.101058	0.100144	-0.50383	0.42021	TRUE	1.00E+00
31652_at	-0.04172	-1.100829	0.084798	-0.43294	0.3495	TRUE	1.00E+00
41463_at	-0.04172	-1.100829	0.023898	-0.15197	0.06854	TRUE	1.00E+00
35284_f_at	-0.04172	-1.100829	0.022111	-0.14373	0.0603	TRUE	1.00E+00
32045_at	-0.04166	-1.100677	0.02757	-0.16885	0.08554	TRUE	1.00E+00
36876_at	-0.04159	-1.1005	0.0257	-0.16016	0.07697	TRUE	1.00E+00
35383_at	-0.04158	-1.100475	0.059844	-0.31767	0.23452	TRUE	1.00E+00
37387_r_at	-0.04157	-1.100449	0.024399	-0.15413	0.071	TRUE	1.00E+00
39964_at	-0.04155	-1.100399	0.013069	-0.10184	0.01875	TRUE	1.00E+00
35091_at	-0.04154	-1.100373	0.039265	-0.22269	0.13961	TRUE	1.00E+00
38439_at	-0.04152	-1.100323	0.024596	-0.155	0.07195	TRUE	1.00E+00
40223_r_at	-0.04147	-1.100196	0.036481	-0.20978	0.12684	TRUE	1.00E+00
41390_at	-0.04146	-1.100171	0.022362	-0.14463	0.06171	TRUE	1.00E+00
38199_at	-0.04137	-1.099943	0.091142	-0.46186	0.37913	TRUE	1.00E+00
32344_r_at	-0.04134	-1.099867	0.124128	-0.61402	0.53134	TRUE	1.00E+00
35911_r_at	-0.04131	-1.099791	0.049134	-0.26799	0.18538	TRUE	1.00E+00
34166_at	-0.04128	-1.099715	0.042033	-0.23521	0.15264	TRUE	1.00E+00
40493_at	-0.04128	-1.099715	0.029741	-0.17849	0.09593	TRUE	1.00E+00
36523_at	-0.04124	-1.099613	0.029913	-0.17924	0.09677	TRUE	1.00E+00
41187_at	-0.04123	-1.099588	0.013083	-0.10158	0.01913	TRUE	1.00E+00
37537_at	-0.04121	-1.099537	0.036969	-0.21177	0.12935	TRUE	1.00E+00
37271_at	-0.04119	-1.099487	0.019499	-0.13115	0.04877	TRUE	1.00E+00
441_s_at	-0.04114	-1.09936	0.069452	-0.36156	0.27929	TRUE	1.00E+00
34440_at	-0.04112	-1.09931	0.0261	-0.16153	0.07929	TRUE	1.00E+00
35663_at	-0.0411	-1.099259	0.097809	-0.49235	0.41015	TRUE	1.00E+00
37028_at	-0.04105	-1.099132	0.027179	-0.16645	0.08434	TRUE	1.00E+00
36180_s_at	-0.04105	-1.099132	0.02788	-0.16968	0.08758	TRUE	1.00E+00
40938_at	-0.04096	-1.098905	0.028517	-0.17253	0.0906	TRUE	1.00E+00
35466_at	-0.04095	-1.098879	0.035505	-0.20476	0.12286	TRUE	1.00E+00
39659_at	-0.04093	-1.098829	0.021197	-0.13873	0.05686	TRUE	1.00E+00
33741_at	-0.04093	-1.098829	0.021755	-0.1413	0.05944	TRUE	1.00E+00

40020_at	-0.04091	-1.098778	0.038464	-0.21837	0.13654	TRUE	1.00E+00
33515_at	-0.0409	-1.098753	0.078126	-0.40134	0.31954	TRUE	1.00E+00
36757_at	-0.04089	-1.098728	0.07544	-0.38894	0.30716	TRUE	1.00E+00
41173_at	-0.04087	-1.098677	0.094038	-0.47472	0.39299	TRUE	1.00E+00
32669_at	-0.04085	-1.098626	0.021239	-0.13884	0.05713	TRUE	1.00E+00
35145_at	-0.04085	-1.098626	0.020587	-0.13583	0.05413	TRUE	1.00E+00
897_at	-0.0408	-1.0985	0.026877	-0.1648	0.0832	TRUE	1.00E+00
32220_at	-0.04079	-1.098475	0.023971	-0.15138	0.0698	TRUE	1.00E+00
33810_at	-0.04072	-1.098298	0.034569	-0.20021	0.11876	TRUE	1.00E+00
41861_at	-0.04067	-1.098171	0.013545	-0.10316	0.02182	TRUE	1.00E+00
121_at	-0.04065	-1.098121	0.033922	-0.19716	0.11585	TRUE	1.00E+00
38895_i_at	-0.04064	-1.098095	0.01905	-0.12853	0.04725	TRUE	1.00E+00
31545_at	-0.04064	-1.098095	0.031786	-0.18729	0.10601	TRUE	1.00E+00
37205_at	-0.04063	-1.09807	0.037313	-0.21278	0.13151	TRUE	1.00E+00
229_at	-0.04057	-1.097918	0.019923	-0.13248	0.05135	TRUE	1.00E+00
34807_at	-0.04056	-1.097893	0.074456	-0.38407	0.30295	TRUE	1.00E+00
1627_at	-0.04054	-1.097842	0.044808	-0.24727	0.16618	TRUE	1.00E+00
38183_at	-0.0405	-1.097741	0.079443	-0.40702	0.32602	TRUE	1.00E+00
31577_at	-0.04049	-1.097716	0.10876	-0.54227	0.46128	TRUE	1.00E+00
37016_at	-0.04044	-1.09759	0.027154	-0.16572	0.08484	TRUE	1.00E+00
160042_s_at	-0.04043	-1.097564	0.039384	-0.22213	0.14128	TRUE	1.00E+00
36125_s_at	-0.04041	-1.097514	0.028245	-0.17072	0.0899	TRUE	1.00E+00
41263_at	-0.04041	-1.097514	0.028989	-0.17415	0.09334	TRUE	1.00E+00
40830_at	-0.0404	-1.097489	0.045688	-0.25119	0.17038	TRUE	1.00E+00
31800_at	-0.04036	-1.097387	0.019883	-0.13209	0.05138	TRUE	1.00E+00
36371_at	-0.04034	-1.097337	0.039286	-0.22159	0.14091	TRUE	1.00E+00
40396_at	-0.0403	-1.097236	0.039008	-0.22027	0.13966	TRUE	1.00E+00
34648_at	-0.04025	-1.09711	0.030962	-0.18309	0.1026	TRUE	1.00E+00
32412_at	-0.04024	-1.097084	0.023153	-0.14706	0.06658	TRUE	1.00E+00
33166_at	-0.04022	-1.097034	0.111175	-0.55313	0.4727	TRUE	1.00E+00
39518_at	-0.0402	-1.096983	0.025646	-0.15852	0.07812	TRUE	1.00E+00
33378_at	-0.04019	-1.096958	0.016456	-0.11611	0.03573	TRUE	1.00E+00
40307_at	-0.04014	-1.096832	0.025329	-0.157	0.07672	TRUE	1.00E+00
34878_at	-0.04008	-1.09668	0.060195	-0.31779	0.23764	TRUE	1.00E+00
38214_at	-0.04007	-1.096655	0.105161	-0.52524	0.4451	TRUE	1.00E+00
40087_at	-0.04007	-1.096655	0.039147	-0.22067	0.14054	TRUE	1.00E+00
40404_s_at	-0.04006	-1.09663	0.017009	-0.11853	0.03841	TRUE	1.00E+00
31410_at	-0.04005	-1.096604	0.020403	-0.13418	0.05408	TRUE	1.00E+00
1963_at	-0.03998	-1.096428	0.099059	-0.497	0.41703	TRUE	1.00E+00
1965_s_at	-0.03991	-1.096251	0.057916	-0.30711	0.22729	TRUE	1.00E+00
39017_at	-0.0399	-1.096226	0.028794	-0.17274	0.09294	TRUE	1.00E+00
39963_at	-0.0399	-1.096226	0.046354	-0.25376	0.17396	TRUE	1.00E+00
34689_at	-0.03988	-1.096175	0.028042	-0.16925	0.08949	TRUE	1.00E+00
31658_at	-0.03987	-1.09615	0.038814	-0.21894	0.13921	TRUE	1.00E+00
40864_at	-0.03982	-1.096024	0.014891	-0.10852	0.02888	TRUE	1.00E+00
34837_at	-0.03976	-1.095872	0.014735	-0.10774	0.02822	TRUE	1.00E+00
33993_at	-0.03976	-1.095872	0.04155	-0.23145	0.15194	TRUE	1.00E+00
31825_at	-0.03974	-1.095822	0.033053	-0.19223	0.11275	TRUE	1.00E+00
571_at	-0.03972	-1.095771	0.016163	-0.11429	0.03484	TRUE	1.00E+00
35860_r_at	-0.03968	-1.095671	0.069049	-0.35825	0.27888	TRUE	1.00E+00
35417_at	-0.03966	-1.09562	0.054263	-0.29001	0.21068	TRUE	1.00E+00

40208_at	-0.03964	-1.09557	0.051998	-0.27954	0.20025	TRUE	1.00E+00
41325_at	-0.03963	-1.095544	0.025075	-0.15532	0.07605	TRUE	1.00E+00
41560_at	-0.03962	-1.095519	0.067446	-0.35079	0.27155	TRUE	1.00E+00
41732_at	-0.0396	-1.095469	0.055427	-0.29531	0.21612	TRUE	1.00E+00
39620_at	-0.03959	-1.095444	0.036016	-0.20576	0.12657	TRUE	1.00E+00
39515_s_at	-0.03958	-1.095418	0.030215	-0.17898	0.09982	TRUE	1.00E+00
459_s_at	-0.03956	-1.095368	0.060986	-0.32092	0.2418	TRUE	1.00E+00
38493_at	-0.03955	-1.095343	0.040231	-0.22516	0.14606	TRUE	1.00E+00
36208_at	-0.03951	-1.095242	0.020037	-0.13195	0.05293	TRUE	1.00E+00
32152_at	-0.0395	-1.095217	0.01915	-0.12785	0.04885	TRUE	1.00E+00
237_s_at	-0.03945	-1.09509	0.011003	-0.09021	0.01132	TRUE	1.00E+00
31394_at	-0.03944	-1.095065	0.074157	-0.38157	0.30269	TRUE	1.00E+00
32564_at	-0.03944	-1.095065	0.013781	-0.10302	0.02414	TRUE	1.00E+00
41135_at	-0.03932	-1.094763	0.103184	-0.51537	0.43673	TRUE	1.00E+00
35100_at	-0.0393	-1.094712	0.1408	-0.68889	0.6103	TRUE	1.00E+00
35337_at	-0.03928	-1.094662	0.006876	-0.071	-0.00755	FALSE	1.41E-04
1667_s_at	-0.03927	-1.094637	0.027738	-0.16724	0.0887	TRUE	1.00E+00
35650_at	-0.03926	-1.094611	0.018785	-0.12593	0.04741	TRUE	1.00E+00
38872_at	-0.03918	-1.09441	0.053187	-0.28457	0.2062	TRUE	1.00E+00
38217_at	-0.03918	-1.09441	0.041948	-0.23271	0.15435	TRUE	1.00E+00
33389_at	-0.03915	-1.094334	0.04389	-0.24164	0.16334	TRUE	1.00E+00
36938_at	-0.0391	-1.094208	0.048951	-0.26494	0.18675	TRUE	1.00E+00
2037_s_at	-0.03907	-1.094133	0.033869	-0.19533	0.11719	TRUE	1.00E+00
35321_at	-0.03895	-1.09383	0.012707	-0.09758	0.01967	TRUE	1.00E+00
41684_at	-0.03891	-1.09373	0.031962	-0.18637	0.10854	TRUE	1.00E+00
39347_at	-0.0389	-1.093705	0.039901	-0.22299	0.14518	TRUE	1.00E+00
38440_s_at	-0.03889	-1.093679	0.014111	-0.10399	0.02621	TRUE	1.00E+00
1840_g_at	-0.03887	-1.093629	0.030825	-0.18109	0.10335	TRUE	1.00E+00
41175_at	-0.03882	-1.093503	0.022605	-0.14311	0.06547	TRUE	1.00E+00
452_at	-0.03879	-1.093428	0.026396	-0.16057	0.08299	TRUE	1.00E+00
40109_at	-0.03874	-1.093302	0.027207	-0.16426	0.08678	TRUE	1.00E+00
31816_at	-0.03871	-1.093226	0.033204	-0.1919	0.11448	TRUE	1.00E+00
32000_g_at	-0.03865	-1.093075	0.013201	-0.09955	0.02226	TRUE	1.00E+00
39503_s_at	-0.0386	-1.092949	0.025891	-0.15805	0.08085	TRUE	1.00E+00
160044_g_at	-0.03859	-1.092924	0.038723	-0.21724	0.14007	TRUE	1.00E+00
41406_at	-0.03855	-1.092823	0.013063	-0.09882	0.02172	TRUE	1.00E+00
31779_s_at	-0.03847	-1.092622	0.041216	-0.22862	0.15169	TRUE	1.00E+00
40723_at	-0.03843	-1.092522	0.046439	-0.25268	0.17583	TRUE	1.00E+00
1161_at	-0.03842	-1.092496	0.026338	-0.15993	0.0831	TRUE	1.00E+00
33713_at	-0.0384	-1.092446	0.042329	-0.23368	0.15689	TRUE	1.00E+00
531_at	-0.03839	-1.092421	0.038328	-0.21522	0.13844	TRUE	1.00E+00
38663_at	-0.03831	-1.09222	0.008003	-0.07524	-0.00139	FALSE	2.14E-02
37289_at	-0.03828	-1.092144	0.112532	-0.55745	0.4809	TRUE	1.00E+00
1852_at	-0.03828	-1.092144	0.040447	-0.22488	0.14833	TRUE	1.00E+00
41160_at	-0.03826	-1.092094	0.02041	-0.13242	0.05591	TRUE	1.00E+00
37885_at	-0.03822	-1.091993	0.014632	-0.10573	0.02928	TRUE	1.00E+00
40410_at	-0.0382	-1.091943	0.021646	-0.13807	0.06166	TRUE	1.00E+00
36586_at	-0.0382	-1.091943	0.02309	-0.14473	0.06833	TRUE	1.00E+00
32594_at	-0.0382	-1.091943	0.014174	-0.10359	0.0272	TRUE	1.00E+00
1551_g_at	-0.03816	-1.091843	0.0126	-0.09629	0.01997	TRUE	1.00E+00
33752_at	-0.03815	-1.091817	0.038462	-0.2156	0.1393	TRUE	1.00E+00

32334_f_at	-0.03809	-1.091667	0.019768	-0.12929	0.05311	TRUE	1.00E+00
38221_at	-0.03806	-1.091591	0.020069	-0.13065	0.05453	TRUE	1.00E+00
35939_s_at	-0.03806	-1.091591	0.028617	-0.17008	0.09397	TRUE	1.00E+00
39415_at	-0.03802	-1.091491	0.020881	-0.13436	0.05831	TRUE	1.00E+00
33226_at	-0.038	-1.09144	0.051711	-0.27657	0.20058	TRUE	1.00E+00
31572_at	-0.03795	-1.091315	0.125851	-0.61858	0.54267	TRUE	1.00E+00
36274_at	-0.03795	-1.091315	0.045594	-0.2483	0.1724	TRUE	1.00E+00
34981_at	-0.03795	-1.091315	0.029259	-0.17294	0.09704	TRUE	1.00E+00
162_at	-0.0379	-1.091189	0.023269	-0.14525	0.06946	TRUE	1.00E+00
38765_at	-0.03788	-1.091139	0.032606	-0.18831	0.11255	TRUE	1.00E+00
39402_at	-0.03787	-1.091114	0.045587	-0.24819	0.17245	TRUE	1.00E+00
33073_at	-0.03786	-1.091089	0.100897	-0.50335	0.42764	TRUE	1.00E+00
37953_s_at	-0.03783	-1.091013	0.030556	-0.1788	0.10315	TRUE	1.00E+00
34056_g_at	-0.03782	-1.090988	0.0695	-0.35846	0.28283	TRUE	1.00E+00
36037_g_at	-0.03781	-1.090963	0.029561	-0.17419	0.09857	TRUE	1.00E+00
32921_at	-0.03779	-1.090913	0.163069	-0.79012	0.71454	TRUE	1.00E+00
34224_at	-0.03779	-1.090913	0.035863	-0.20325	0.12767	TRUE	1.00E+00
31399_at	-0.03773	-1.090762	0.024809	-0.15219	0.07673	TRUE	1.00E+00
2083_at	-0.03772	-1.090737	0.159962	-0.77572	0.70027	TRUE	1.00E+00
35113_at	-0.03765	-1.090561	0.052553	-0.28011	0.20481	TRUE	1.00E+00
1259_at	-0.03762	-1.090486	0.0392	-0.21848	0.14323	TRUE	1.00E+00
40134_at	-0.03761	-1.090461	0.016798	-0.11511	0.03989	TRUE	1.00E+00
35527_at	-0.03752	-1.090235	0.051669	-0.2759	0.20086	TRUE	1.00E+00
752_s_at	-0.03751	-1.09021	0.019183	-0.12601	0.05099	TRUE	1.00E+00
41642_at	-0.03749	-1.090159	0.034805	-0.19806	0.12308	TRUE	1.00E+00
33195_at	-0.03748	-1.090134	0.083794	-0.42407	0.34911	TRUE	1.00E+00
196_s_at	-0.03747	-1.090109	0.101902	-0.5076	0.43266	TRUE	1.00E+00
36506_at	-0.03743	-1.090009	0.025913	-0.15698	0.08212	TRUE	1.00E+00
39635_at	-0.03741	-1.089959	0.035632	-0.2018	0.12698	TRUE	1.00E+00
40577_at	-0.03741	-1.089959	0.012948	-0.09714	0.02233	TRUE	1.00E+00
1652_at	-0.0374	-1.089933	0.075168	-0.3842	0.30939	TRUE	1.00E+00
1547_at	-0.03736	-1.089833	0.067016	-0.34654	0.27183	TRUE	1.00E+00
1229_at	-0.03729	-1.089657	0.035443	-0.20081	0.12623	TRUE	1.00E+00
38154_at	-0.03729	-1.089657	0.038484	-0.21483	0.14026	TRUE	1.00E+00
1284_at	-0.03728	-1.089632	0.030127	-0.17628	0.10171	TRUE	1.00E+00
41273_at	-0.03726	-1.089582	0.013152	-0.09793	0.02342	TRUE	1.00E+00
37651_at	-0.03725	-1.089557	0.011201	-0.08893	0.01442	TRUE	1.00E+00
31680_at	-0.03719	-1.089407	0.038814	-0.21626	0.14188	TRUE	1.00E+00
41597_s_at	-0.03714	-1.089281	0.021176	-0.13484	0.06056	TRUE	1.00E+00
31810_g_at	-0.03696	-1.08883	0.030544	-0.17788	0.10396	TRUE	1.00E+00
41478_at	-0.03696	-1.08883	0.018881	-0.12407	0.05015	TRUE	1.00E+00
35821_at	-0.03693	-1.088755	0.023812	-0.14679	0.07293	TRUE	1.00E+00
40206_at	-0.03689	-1.088654	0.013077	-0.09722	0.02344	TRUE	1.00E+00
33159_at	-0.03688	-1.088629	0.036625	-0.20585	0.1321	TRUE	1.00E+00
40668_s_at	-0.03684	-1.088529	0.019413	-0.1264	0.05273	TRUE	1.00E+00
31712_at	-0.03679	-1.088404	0.098189	-0.48979	0.41622	TRUE	1.00E+00
33247_at	-0.03679	-1.088404	0.027823	-0.16515	0.09158	TRUE	1.00E+00
39404_s_at	-0.03679	-1.088404	0.017444	-0.11727	0.04369	TRUE	1.00E+00
35945_at	-0.03677	-1.088354	0.121236	-0.5961	0.52257	TRUE	1.00E+00
1424_s_at	-0.03676	-1.088328	0.033092	-0.18943	0.11591	TRUE	1.00E+00
36955_at	-0.03674	-1.088278	0.031031	-0.1799	0.10643	TRUE	1.00E+00

33712_at	-0.03671	-1.088203	0.027509	-0.16362	0.09021	TRUE	1.00E+00
31420_at	-0.0367	-1.088178	0.03019	-0.17598	0.10259	TRUE	1.00E+00
34392_s_at	-0.03669	-1.088153	0.016239	-0.11162	0.03823	TRUE	1.00E+00
33282_at	-0.03669	-1.088153	0.060596	-0.31625	0.24287	TRUE	1.00E+00
33916_at	-0.03667	-1.088103	0.017098	-0.11555	0.04222	TRUE	1.00E+00
33561_at	-0.03664	-1.088028	0.037856	-0.21129	0.13801	TRUE	1.00E+00
33613_at	-0.03662	-1.087978	0.027712	-0.16447	0.09123	TRUE	1.00E+00
32137_at	-0.03656	-1.087827	0.026846	-0.16041	0.0873	TRUE	1.00E+00
1948_f_at	-0.03655	-1.087802	0.040135	-0.22172	0.14861	TRUE	1.00E+00
36409_f_at	-0.03652	-1.087727	0.075428	-0.38452	0.31147	TRUE	1.00E+00
40212_at	-0.03651	-1.087702	0.041275	-0.22694	0.15392	TRUE	1.00E+00
37413_at	-0.03651	-1.087702	0.042072	-0.23061	0.1576	TRUE	1.00E+00
37212_at	-0.03649	-1.087652	0.021725	-0.13672	0.06374	TRUE	1.00E+00
37267_at	-0.03648	-1.087627	0.018032	-0.11968	0.04671	TRUE	1.00E+00
35768_at	-0.03642	-1.087477	0.020336	-0.13024	0.05741	TRUE	1.00E+00
1647_at	-0.0364	-1.087427	0.077026	-0.39177	0.31896	TRUE	1.00E+00
34472_at	-0.0364	-1.087427	0.020695	-0.13188	0.05908	TRUE	1.00E+00
628_at	-0.03639	-1.087402	0.015348	-0.1072	0.03442	TRUE	1.00E+00
40183_at	-0.03638	-1.087377	0.017855	-0.11876	0.04599	TRUE	1.00E+00
39685_at	-0.03637	-1.087352	0.032932	-0.18831	0.11556	TRUE	1.00E+00
657_at	-0.03634	-1.087276	0.013113	-0.09684	0.02416	TRUE	1.00E+00
1314_at	-0.03629	-1.087151	0.023156	-0.14313	0.07054	TRUE	1.00E+00
41231_f_at	-0.03625	-1.087051	0.02988	-0.1741	0.10161	TRUE	1.00E+00
35020_at	-0.0362	-1.086926	0.031948	-0.18359	0.1112	TRUE	1.00E+00
2077_at	-0.03616	-1.086826	0.045481	-0.24599	0.17367	TRUE	1.00E+00
36564_at	-0.03613	-1.086751	0.037572	-0.20947	0.13721	TRUE	1.00E+00
33659_at	-0.03611	-1.086701	0.022507	-0.13994	0.06773	TRUE	1.00E+00
40515_at	-0.036	-1.086426	0.03114	-0.17966	0.10767	TRUE	1.00E+00
35574_i_at	-0.036	-1.086426	0.044983	-0.24353	0.17154	TRUE	1.00E+00
37201_at	-0.03597	-1.086351	0.046241	-0.24931	0.17737	TRUE	1.00E+00
35374_at	-0.03595	-1.086301	0.060802	-0.31646	0.24457	TRUE	1.00E+00
34425_at	-0.03594	-1.086276	0.016036	-0.10993	0.03804	TRUE	1.00E+00
37370_i_at	-0.03594	-1.086276	0.030579	-0.17702	0.10514	TRUE	1.00E+00
40225_at	-0.03586	-1.086075	0.013319	-0.09731	0.02559	TRUE	1.00E+00
39238_at	-0.03585	-1.08605	0.030855	-0.1782	0.1065	TRUE	1.00E+00
32909_at	-0.03582	-1.085975	0.018009	-0.11891	0.04726	TRUE	1.00E+00
32882_at	-0.03578	-1.085875	0.02869	-0.16815	0.09658	TRUE	1.00E+00
38056_at	-0.03576	-1.085825	0.011388	-0.08831	0.01678	TRUE	1.00E+00
36603_at	-0.03576	-1.085825	0.016471	-0.11175	0.04023	TRUE	1.00E+00
32095_at	-0.03575	-1.0858	0.021907	-0.13682	0.06532	TRUE	1.00E+00
39022_at	-0.03574	-1.085775	0.023912	-0.14606	0.07458	TRUE	1.00E+00
37833_at	-0.03573	-1.08575	0.061255	-0.31834	0.24687	TRUE	1.00E+00
32623_at	-0.03569	-1.08565	0.027925	-0.16452	0.09315	TRUE	1.00E+00
39554_at	-0.03567	-1.0856	0.027891	-0.16434	0.09301	TRUE	1.00E+00
33805_at	-0.03566	-1.085575	0.041906	-0.229	0.15768	TRUE	1.00E+00
41234_at	-0.03554	-1.085276	0.014881	-0.10419	0.03312	TRUE	1.00E+00
40413_at	-0.03551	-1.085201	0.016274	-0.11059	0.03957	TRUE	1.00E+00
35725_at	-0.03543	-1.085001	0.027684	-0.16315	0.09229	TRUE	1.00E+00
38417_at	-0.03543	-1.085001	0.015739	-0.10804	0.03718	TRUE	1.00E+00
2049_s_at	-0.0354	-1.084926	0.063507	-0.3284	0.2576	TRUE	1.00E+00
41834_g_at	-0.03525	-1.084551	0.021327	-0.13364	0.06315	TRUE	1.00E+00

39865_at	-0.03524	-1.084526	0.030618	-0.1765	0.10601	TRUE	1.00E+00
32044_at	-0.03517	-1.084351	0.023079	-0.14165	0.07131	TRUE	1.00E+00
33020_at	-0.03513	-1.084251	0.028057	-0.16458	0.09431	TRUE	1.00E+00
39244_at	-0.03511	-1.084201	0.021176	-0.1328	0.06259	TRUE	1.00E+00
1714_at	-0.03508	-1.084127	0.035768	-0.20009	0.12994	TRUE	1.00E+00
33689_s_at	-0.03506	-1.084077	0.016111	-0.10939	0.03927	TRUE	1.00E+00
34402_at	-0.03503	-1.084002	0.015382	-0.106	0.03593	TRUE	1.00E+00
31495_at	-0.03496	-1.083827	0.04542	-0.24451	0.17459	TRUE	1.00E+00
33754_at	-0.03495	-1.083802	0.047275	-0.25305	0.18316	TRUE	1.00E+00
386_g_at	-0.03488	-1.083627	0.065066	-0.33507	0.26531	TRUE	1.00E+00
33501_r_at	-0.03485	-1.083553	0.02295	-0.14073	0.07103	TRUE	1.00E+00
1569_r_at	-0.03483	-1.083503	0.033194	-0.18797	0.11831	TRUE	1.00E+00
40300_g_at	-0.03483	-1.083503	0.025584	-0.15286	0.08321	TRUE	1.00E+00
34268_at	-0.03481	-1.083453	0.048402	-0.25812	0.18849	TRUE	1.00E+00
32542_at	-0.03479	-1.083403	0.014698	-0.1026	0.03302	TRUE	1.00E+00
31920_at	-0.03478	-1.083378	0.090681	-0.45315	0.38358	TRUE	1.00E+00
32489_at	-0.03475	-1.083303	0.139166	-0.6768	0.6073	TRUE	1.00E+00
40174_at	-0.03461	-1.082954	0.0681	-0.34879	0.27958	TRUE	1.00E+00
39706_at	-0.03458	-1.082879	0.020878	-0.1309	0.06174	TRUE	1.00E+00
41131_f_at	-0.03457	-1.082854	0.031005	-0.17762	0.10847	TRUE	1.00E+00
33329_at	-0.03457	-1.082854	0.069177	-0.35373	0.28458	TRUE	1.00E+00
31573_at	-0.03453	-1.082755	0.021646	-0.1344	0.06534	TRUE	1.00E+00
485_at	-0.03452	-1.08273	0.028126	-0.16428	0.09524	TRUE	1.00E+00
382_at	-0.03447	-1.082605	0.016958	-0.1127	0.04377	TRUE	1.00E+00
33677_at	-0.03446	-1.08258	0.026275	-0.15568	0.08676	TRUE	1.00E+00
40213_at	-0.03442	-1.08248	0.031823	-0.18124	0.1124	TRUE	1.00E+00
32691_s_at	-0.0344	-1.08243	0.024609	-0.14793	0.07914	TRUE	1.00E+00
1716_at	-0.03439	-1.082406	0.057166	-0.29813	0.22935	TRUE	1.00E+00
37899_at	-0.03435	-1.082306	0.075829	-0.3842	0.31549	TRUE	1.00E+00
1992_at	-0.03434	-1.082281	0.047064	-0.25148	0.18279	TRUE	1.00E+00
40562_at	-0.03427	-1.082106	0.015126	-0.10406	0.03551	TRUE	1.00E+00
35684_at	-0.03426	-1.082082	0.036695	-0.20356	0.13503	TRUE	1.00E+00
1494_f_at	-0.03422	-1.081982	0.016155	-0.10875	0.04031	TRUE	1.00E+00
32924_at	-0.0342	-1.081932	0.022737	-0.1391	0.0707	TRUE	1.00E+00
36544_at	-0.0342	-1.081932	0.055764	-0.29148	0.22307	TRUE	1.00E+00
34289_f_at	-0.0342	-1.081932	0.063271	-0.3261	0.25771	TRUE	1.00E+00
35125_at	-0.03418	-1.081882	0.022629	-0.13858	0.07022	TRUE	1.00E+00
41722_at	-0.03418	-1.081882	0.028675	-0.16647	0.09811	TRUE	1.00E+00
41822_at	-0.03417	-1.081857	0.01817	-0.118	0.04967	TRUE	1.00E+00
33504_at	-0.03415	-1.081808	0.050972	-0.26931	0.20101	TRUE	1.00E+00
37255_at	-0.03414	-1.081783	0.044653	-0.24015	0.17187	TRUE	1.00E+00
1437_at	-0.03412	-1.081733	0.041426	-0.22524	0.157	TRUE	1.00E+00
37101_at	-0.03403	-1.081509	0.029787	-0.17146	0.1034	TRUE	1.00E+00
32488_at	-0.034	-1.081434	0.017926	-0.1167	0.04871	TRUE	1.00E+00
37735_at	-0.03381	-1.080961	0.009804	-0.07904	0.01142	TRUE	1.00E+00
1580_f_at	-0.03378	-1.080886	0.039822	-0.21751	0.14994	TRUE	1.00E+00
32549_at	-0.03375	-1.080812	0.050602	-0.26721	0.1997	TRUE	1.00E+00
36353_at	-0.03374	-1.080787	0.030437	-0.17417	0.10668	TRUE	1.00E+00
40443_at	-0.03373	-1.080762	0.03892	-0.21329	0.14583	TRUE	1.00E+00
37200_at	-0.03371	-1.080712	0.077279	-0.39025	0.32282	TRUE	1.00E+00
1696_at	-0.0337	-1.080687	0.020167	-0.12674	0.05934	TRUE	1.00E+00

34436_at	-0.03367	-1.080613	0.035842	-0.19903	0.13169	TRUE	1.00E+00
31923_f_at	-0.03365	-1.080563	0.018684	-0.11985	0.05255	TRUE	1.00E+00
34682_at	-0.03362	-1.080488	0.032648	-0.18425	0.117	TRUE	1.00E+00
1370_at	-0.03362	-1.080488	0.014054	-0.09846	0.03122	TRUE	1.00E+00
35764_at	-0.03355	-1.080314	0.038693	-0.21207	0.14496	TRUE	1.00E+00
36317_at	-0.03352	-1.080239	0.058622	-0.30398	0.23694	TRUE	1.00E+00
34638_r_at	-0.03342	-1.079991	0.03534	-0.19646	0.12963	TRUE	1.00E+00
37449_i_at	-0.03342	-1.079991	0.016544	-0.10975	0.04291	TRUE	1.00E+00
36179_at	-0.03341	-1.079966	0.011605	-0.08695	0.02013	TRUE	1.00E+00
39276_g_at	-0.0334	-1.079941	0.029161	-0.16793	0.10113	TRUE	1.00E+00
36485_at	-0.03336	-1.079841	0.029708	-0.17042	0.1037	TRUE	1.00E+00
37777_at	-0.03336	-1.079841	0.040629	-0.22081	0.15409	TRUE	1.00E+00
33281_at	-0.03334	-1.079792	0.031823	-0.18016	0.11348	TRUE	1.00E+00
39821_s_at	-0.0333	-1.079692	0.021295	-0.13154	0.06495	TRUE	1.00E+00
33311_at	-0.03328	-1.079643	0.041115	-0.22297	0.1564	TRUE	1.00E+00
1523_g_at	-0.03328	-1.079643	0.030065	-0.17199	0.10543	TRUE	1.00E+00
36663_at	-0.03325	-1.079568	0.0269	-0.15736	0.09085	TRUE	1.00E+00
36761_at	-0.03324	-1.079543	0.012999	-0.09321	0.02673	TRUE	1.00E+00
35737_at	-0.03314	-1.079295	0.050968	-0.26829	0.20201	TRUE	1.00E+00
37496_at	-0.03312	-1.079245	0.02634	-0.15464	0.0884	TRUE	1.00E+00
1780_at	-0.03301	-1.078972	0.026425	-0.15492	0.08891	TRUE	1.00E+00
41680_at	-0.033	-1.078947	0.042656	-0.2298	0.16379	TRUE	1.00E+00
40956_at	-0.03299	-1.078922	0.042907	-0.23095	0.16497	TRUE	1.00E+00
34827_at	-0.03298	-1.078897	0.029027	-0.1669	0.10094	TRUE	1.00E+00
36200_at	-0.03297	-1.078872	0.027063	-0.15783	0.09189	TRUE	1.00E+00
1378_g_at	-0.03296	-1.078847	0.028956	-0.16655	0.10063	TRUE	1.00E+00
40437_at	-0.03293	-1.078773	0.023808	-0.14278	0.07691	TRUE	1.00E+00
32129_at	-0.03291	-1.078723	0.016506	-0.10907	0.04324	TRUE	1.00E+00
35770_at	-0.03285	-1.078574	0.013303	-0.09422	0.02852	TRUE	1.00E+00
38204_at	-0.03285	-1.078574	0.022392	-0.13615	0.07046	TRUE	1.00E+00
40637_at	-0.03284	-1.078549	0.044674	-0.23895	0.17326	TRUE	1.00E+00
35866_at	-0.03283	-1.078524	0.102781	-0.50702	0.44136	TRUE	1.00E+00
38841_at	-0.03281	-1.078475	0.040662	-0.22041	0.15478	TRUE	1.00E+00
32333_at	-0.03271	-1.078226	0.054303	-0.28324	0.21782	TRUE	1.00E+00
1435_f_at	-0.03268	-1.078152	0.065593	-0.3353	0.26994	TRUE	1.00E+00
35199_at	-0.03267	-1.078127	0.023252	-0.13994	0.07461	TRUE	1.00E+00
33083_at	-0.03265	-1.078078	0.050563	-0.26592	0.20063	TRUE	1.00E+00
1467_at	-0.03262	-1.078003	0.024457	-0.14545	0.08021	TRUE	1.00E+00
36005_at	-0.03261	-1.077978	0.019608	-0.12307	0.05786	TRUE	1.00E+00
33068_f_at	-0.0326	-1.077953	0.062655	-0.32166	0.25647	TRUE	1.00E+00
35072_at	-0.03259	-1.077929	0.098497	-0.48701	0.42184	TRUE	1.00E+00
38434_at	-0.03256	-1.077854	0.02305	-0.1389	0.07378	TRUE	1.00E+00
36905_at	-0.03255	-1.077829	0.017388	-0.11278	0.04767	TRUE	1.00E+00
1850_at	-0.03255	-1.077829	0.032662	-0.18323	0.11814	TRUE	1.00E+00
41324_g_at	-0.03251	-1.07773	0.057519	-0.29788	0.23287	TRUE	1.00E+00
238_at	-0.03247	-1.077631	0.030894	-0.175	0.11006	TRUE	1.00E+00
34614_at	-0.0324	-1.077457	0.132673	-0.64451	0.5797	TRUE	1.00E+00
38178_at	-0.0324	-1.077457	0.0918	-0.45593	0.39112	TRUE	1.00E+00
39236_s_at	-0.03239	-1.077432	0.074454	-0.37589	0.3111	TRUE	1.00E+00
34888_at	-0.03236	-1.077358	0.056517	-0.29311	0.22838	TRUE	1.00E+00
32458_f_at	-0.03236	-1.077358	0.034501	-0.19153	0.12681	TRUE	1.00E+00

36941_at	-0.03231	-1.077234	0.050793	-0.26665	0.20203	TRUE	1.00E+00
39727_at	-0.0323	-1.077209	0.01439	-0.09869	0.03409	TRUE	1.00E+00
35353_at	-0.03227	-1.077135	0.017223	-0.11174	0.04719	TRUE	1.00E+00
1423_at	-0.03227	-1.077135	0.020813	-0.12829	0.06376	TRUE	1.00E+00
35543_at	-0.03225	-1.077085	0.092165	-0.45746	0.39297	TRUE	1.00E+00
35551_g_at	-0.03222	-1.077011	0.065743	-0.33553	0.27109	TRUE	1.00E+00
36851_g_at	-0.0322	-1.076961	0.027948	-0.16114	0.09674	TRUE	1.00E+00
40924_at	-0.03219	-1.076936	0.037628	-0.20579	0.14141	TRUE	1.00E+00
939_at	-0.03218	-1.076911	0.023842	-0.14218	0.07782	TRUE	1.00E+00
38427_at	-0.03217	-1.076887	0.119392	-0.58299	0.51865	TRUE	1.00E+00
34019_at	-0.03213	-1.076787	0.070228	-0.35613	0.29188	TRUE	1.00E+00
40646_at	-0.03213	-1.076787	0.115975	-0.56718	0.50293	TRUE	1.00E+00
32204_at	-0.0321	-1.076713	0.025132	-0.14805	0.08384	TRUE	1.00E+00
39670_at	-0.03209	-1.076688	0.033232	-0.18541	0.12122	TRUE	1.00E+00
33302_at	-0.03206	-1.076614	0.041272	-0.22248	0.15835	TRUE	1.00E+00
38117_at	-0.03205	-1.076589	0.007721	-0.06767	0.00357	TRUE	4.18E-01
36740_at	-0.03197	-1.076391	0.023633	-0.14101	0.07706	TRUE	1.00E+00
35289_at	-0.03195	-1.076341	0.03215	-0.18028	0.11638	TRUE	1.00E+00
33223_at	-0.03192	-1.076267	0.016936	-0.11005	0.04622	TRUE	1.00E+00
36763_at	-0.03191	-1.076242	0.036417	-0.19993	0.1361	TRUE	1.00E+00
38911_at	-0.03191	-1.076242	0.016115	-0.10626	0.04244	TRUE	1.00E+00
32135_at	-0.0319	-1.076217	0.023306	-0.13943	0.07562	TRUE	1.00E+00
40065_s_at	-0.03182	-1.076019	0.042976	-0.2301	0.16645	TRUE	1.00E+00
39728_at	-0.03182	-1.076019	0.047937	-0.25298	0.18934	TRUE	1.00E+00
33448_at	-0.03179	-1.075945	0.028923	-0.16523	0.10165	TRUE	1.00E+00
41641_at	-0.03179	-1.075945	0.029577	-0.16825	0.10467	TRUE	1.00E+00
32975_g_at	-0.03177	-1.075895	0.0133	-0.09313	0.02959	TRUE	1.00E+00
40739_at	-0.03176	-1.075871	0.028162	-0.16169	0.09816	TRUE	1.00E+00
31595_at	-0.0317	-1.075722	0.058618	-0.30213	0.23874	TRUE	1.00E+00
32171_at	-0.0317	-1.075722	0.018467	-0.11689	0.0535	TRUE	1.00E+00
41226_at	-0.03167	-1.075648	0.037601	-0.20515	0.1418	TRUE	1.00E+00
33783_at	-0.03162	-1.075524	0.033095	-0.18431	0.12106	TRUE	1.00E+00
32374_at	-0.03161	-1.075499	0.025957	-0.15136	0.08815	TRUE	1.00E+00
41140_at	-0.03159	-1.075449	0.041709	-0.22402	0.16083	TRUE	1.00E+00
40420_at	-0.03153	-1.075301	0.006536	-0.06169	-0.00138	FALSE	1.78E-02
33888_at	-0.03152	-1.075276	0.016366	-0.10702	0.04399	TRUE	1.00E+00
35556_at	-0.03146	-1.075128	0.052782	-0.27497	0.21205	TRUE	1.00E+00
41751_at	-0.03146	-1.075128	0.066749	-0.33941	0.27649	TRUE	1.00E+00
37189_at	-0.03144	-1.075078	0.027801	-0.15971	0.09682	TRUE	1.00E+00
33272_at	-0.03144	-1.075078	0.035486	-0.19516	0.13228	TRUE	1.00E+00
39185_at	-0.03144	-1.075078	0.022448	-0.13501	0.07213	TRUE	1.00E+00
36469_at	-0.03143	-1.075053	0.025693	-0.14996	0.08711	TRUE	1.00E+00
143_s_at	-0.03142	-1.075029	0.027152	-0.15669	0.09385	TRUE	1.00E+00
31900_at	-0.03141	-1.075004	0.027836	-0.15983	0.09702	TRUE	1.00E+00
41466_s_at	-0.03139	-1.074954	0.022504	-0.13521	0.07244	TRUE	1.00E+00
33323_r_at	-0.03137	-1.074905	0.023815	-0.14124	0.07851	TRUE	1.00E+00
34401_at	-0.03135	-1.074855	0.029128	-0.16573	0.10304	TRUE	1.00E+00
1447_at	-0.03135	-1.074855	0.006914	-0.06324	0.00055	TRUE	7.31E-02
494_at	-0.03127	-1.074657	0.038665	-0.20965	0.14711	TRUE	1.00E+00
2039_s_at	-0.03125	-1.074608	0.026492	-0.15347	0.09097	TRUE	1.00E+00
33334_at	-0.03122	-1.074534	0.036339	-0.19888	0.13643	TRUE	1.00E+00

33632_g_at	-0.03122	-1.074534	0.030097	-0.17008	0.10763	TRUE	1.00E+00
31583_at	-0.03119	-1.074459	0.025472	-0.1487	0.08633	TRUE	1.00E+00
37434_at	-0.03118	-1.074435	0.032159	-0.17955	0.11719	TRUE	1.00E+00
36951_at	-0.03115	-1.07436	0.01711	-0.11009	0.04779	TRUE	1.00E+00
34864_at	-0.03111	-1.074261	0.025475	-0.14864	0.08642	TRUE	1.00E+00
31536_at	-0.03102	-1.074039	0.016179	-0.10566	0.04362	TRUE	1.00E+00
40745_at	-0.03097	-1.073915	0.01533	-0.1017	0.03976	TRUE	1.00E+00
36057_at	-0.03096	-1.07389	0.022772	-0.13602	0.0741	TRUE	1.00E+00
32486_at	-0.03092	-1.073792	0.020425	-0.12515	0.06332	TRUE	1.00E+00
1488_at	-0.03088	-1.073693	0.022526	-0.13481	0.07304	TRUE	1.00E+00
33540_at	-0.03079	-1.07347	0.0824	-0.41096	0.34937	TRUE	1.00E+00
34103_at	-0.03078	-1.073446	0.165756	-0.79551	0.73395	TRUE	1.00E+00
31696_at	-0.0307	-1.073248	0.062297	-0.31812	0.25671	TRUE	1.00E+00
36147_at	-0.0307	-1.073248	0.017931	-0.11343	0.05202	TRUE	1.00E+00
1009_at	-0.0307	-1.073248	0.025267	-0.14727	0.08587	TRUE	1.00E+00
32439_at	-0.03066	-1.073149	0.074102	-0.37254	0.31122	TRUE	1.00E+00
40845_at	-0.03063	-1.073075	0.051416	-0.26784	0.20658	TRUE	1.00E+00
193_at	-0.0306	-1.073001	0.022981	-0.13662	0.07543	TRUE	1.00E+00
638_at	-0.03059	-1.072976	0.083126	-0.4141	0.35292	TRUE	1.00E+00
35585_at	-0.03057	-1.072927	0.047752	-0.25088	0.18974	TRUE	1.00E+00
1419_g_at	-0.03056	-1.072902	0.037433	-0.20326	0.14214	TRUE	1.00E+00
33346_r_at	-0.03055	-1.072877	0.013928	-0.09481	0.0337	TRUE	1.00E+00
160043_at	-0.03053	-1.072828	0.016508	-0.10669	0.04563	TRUE	1.00E+00
39665_at	-0.0305	-1.072754	0.021327	-0.12889	0.0679	TRUE	1.00E+00
32198_at	-0.03042	-1.072556	0.014069	-0.09533	0.03449	TRUE	1.00E+00
37374_at	-0.03042	-1.072556	0.047751	-0.25073	0.18988	TRUE	1.00E+00
33293_at	-0.03037	-1.072433	0.034539	-0.18972	0.12897	TRUE	1.00E+00
38035_at	-0.03037	-1.072433	0.014054	-0.0952	0.03447	TRUE	1.00E+00
41835_at	-0.03036	-1.072408	0.093547	-0.46195	0.40123	TRUE	1.00E+00
36827_at	-0.03032	-1.072309	0.008862	-0.07121	0.01056	TRUE	1.00E+00
37417_at	-0.03028	-1.07221	0.048808	-0.25546	0.1949	TRUE	1.00E+00
32056_at	-0.03026	-1.072161	0.086497	-0.42932	0.3688	TRUE	1.00E+00
36982_at	-0.03024	-1.072112	0.015552	-0.10199	0.04151	TRUE	1.00E+00
1264_at	-0.03023	-1.072087	0.037859	-0.20489	0.14444	TRUE	1.00E+00
40076_at	-0.03019	-1.071988	0.022313	-0.13313	0.07276	TRUE	1.00E+00
41849_r_at	-0.03007	-1.071692	0.109885	-0.53704	0.47689	TRUE	1.00E+00
35700_at	-0.03007	-1.071692	0.085356	-0.42387	0.36373	TRUE	1.00E+00
1823_g_at	-0.03006	-1.071667	0.070473	-0.35519	0.29507	TRUE	1.00E+00
38659_at	-0.03003	-1.071593	0.013222	-0.09103	0.03097	TRUE	1.00E+00
1115_at	-0.03002	-1.071569	0.128094	-0.62099	0.56096	TRUE	1.00E+00
34767_at	-0.02994	-1.071371	0.021898	-0.13097	0.07109	TRUE	1.00E+00
135_g_at	-0.02993	-1.071347	0.023093	-0.13647	0.07661	TRUE	1.00E+00
33415_at	-0.02992	-1.071322	0.013355	-0.09154	0.03169	TRUE	1.00E+00
38558_at	-0.02992	-1.071322	0.024978	-0.14516	0.08532	TRUE	1.00E+00
32898_at	-0.02992	-1.071322	0.034847	-0.19069	0.13085	TRUE	1.00E+00
34945_at	-0.0299	-1.071273	0.064276	-0.32644	0.26664	TRUE	1.00E+00
33268_at	-0.02988	-1.071223	0.09523	-0.46924	0.40947	TRUE	1.00E+00
37219_at	-0.02988	-1.071223	0.011464	-0.08277	0.02301	TRUE	1.00E+00
35217_at	-0.02988	-1.071223	0.012955	-0.08965	0.02989	TRUE	1.00E+00
41018_at	-0.02986	-1.071174	0.028967	-0.16351	0.10378	TRUE	1.00E+00
38605_at	-0.02984	-1.071125	0.010212	-0.07696	0.01727	TRUE	1.00E+00

31952_at	-0.02984	-1.071125	0.014858	-0.09839	0.03871	TRUE	1.00E+00
33332_at	-0.02983	-1.0711	0.023763	-0.13947	0.0798	TRUE	1.00E+00
37429_g_at	-0.02979	-1.071001	0.023227	-0.13695	0.07737	TRUE	1.00E+00
1784_s_at	-0.02974	-1.070878	0.022078	-0.1316	0.07212	TRUE	1.00E+00
33850_at	-0.02973	-1.070853	0.045563	-0.23994	0.18048	TRUE	1.00E+00
1594_at	-0.02971	-1.070804	0.01853	-0.1152	0.05578	TRUE	1.00E+00
31443_at	-0.02971	-1.070804	0.030191	-0.169	0.10958	TRUE	1.00E+00
39697_at	-0.0297	-1.070779	0.043757	-0.23158	0.17217	TRUE	1.00E+00
38083_at	-0.02967	-1.070705	0.035744	-0.19458	0.13523	TRUE	1.00E+00
33639_g_at	-0.02965	-1.070656	0.076053	-0.38053	0.32122	TRUE	1.00E+00
31337_at	-0.02965	-1.070656	0.066707	-0.3374	0.27811	TRUE	1.00E+00
35244_at	-0.02959	-1.070508	0.039951	-0.21391	0.15472	TRUE	1.00E+00
33390_at	-0.02959	-1.070508	0.023646	-0.13868	0.0795	TRUE	1.00E+00
34131_at	-0.02957	-1.070459	0.019911	-0.12144	0.06229	TRUE	1.00E+00
446_at	-0.02956	-1.070434	0.017508	-0.11033	0.05121	TRUE	1.00E+00
41760_at	-0.02952	-1.070336	0.013434	-0.0915	0.03246	TRUE	1.00E+00
40844_at	-0.02951	-1.070311	0.033784	-0.18538	0.12635	TRUE	1.00E+00
32478_f_at	-0.02945	-1.070163	0.023818	-0.13934	0.08043	TRUE	1.00E+00
34338_at	-0.02945	-1.070163	0.010261	-0.07679	0.01789	TRUE	1.00E+00
36475_at	-0.02942	-1.070089	0.023358	-0.13718	0.07835	TRUE	1.00E+00
36028_at	-0.02939	-1.070015	0.036961	-0.19992	0.14113	TRUE	1.00E+00
40917_at	-0.02938	-1.069991	0.097958	-0.48132	0.42256	TRUE	1.00E+00
992_at	-0.02936	-1.069941	0.039134	-0.2099	0.15119	TRUE	1.00E+00
37232_at	-0.02931	-1.069818	0.034711	-0.18945	0.13083	TRUE	1.00E+00
32989_at	-0.02931	-1.069818	0.028073	-0.15883	0.1002	TRUE	1.00E+00
563_at	-0.02928	-1.069744	0.056858	-0.2916	0.23304	TRUE	1.00E+00
38528_at	-0.02922	-1.069597	0.03143	-0.17423	0.11578	TRUE	1.00E+00
418_at	-0.02918	-1.069498	0.088836	-0.43903	0.38067	TRUE	1.00E+00
38971_r_at	-0.02914	-1.0694	0.116633	-0.56723	0.50896	TRUE	1.00E+00
37191_at	-0.02913	-1.069375	0.024184	-0.14071	0.08245	TRUE	1.00E+00
33984_at	-0.02913	-1.069375	0.007854	-0.06536	0.00711	TRUE	1.00E+00
847_at	-0.02912	-1.06935	0.082425	-0.40939	0.35115	TRUE	1.00E+00
32903_at	-0.02911	-1.069326	0.126474	-0.61261	0.55439	TRUE	1.00E+00
affx-m27830_m_	-0.02903	-1.069129	0.048274	-0.25175	0.19369	TRUE	1.00E+00
33726_at	-0.02902	-1.069104	0.020927	-0.12557	0.06753	TRUE	1.00E+00
37482_at	-0.02901	-1.069079	0.096581	-0.47459	0.41658	TRUE	1.00E+00
41295_at	-0.02898	-1.069006	0.018524	-0.11444	0.05649	TRUE	1.00E+00
40822_at	-0.02896	-1.068956	0.013984	-0.09347	0.03556	TRUE	1.00E+00
41118_at	-0.02895	-1.068932	0.017814	-0.11114	0.05324	TRUE	1.00E+00
719_g_at	-0.02895	-1.068932	0.013145	-0.08959	0.0317	TRUE	1.00E+00
38362_at	-0.02893	-1.068883	0.075305	-0.37636	0.31849	TRUE	1.00E+00
33449_at	-0.02886	-1.06871	0.089378	-0.44121	0.38349	TRUE	1.00E+00
36668_at	-0.02884	-1.068661	0.018356	-0.11353	0.05585	TRUE	1.00E+00
33839_at	-0.02878	-1.068513	0.025936	-0.14844	0.09088	TRUE	1.00E+00
1443_at	-0.02878	-1.068513	0.046042	-0.2412	0.18364	TRUE	1.00E+00
40807_at	-0.02877	-1.068489	0.019579	-0.1191	0.06155	TRUE	1.00E+00
40618_at	-0.02874	-1.068415	0.00802	-0.06575	0.00826	TRUE	1.00E+00
41420_at	-0.02874	-1.068415	0.041246	-0.21903	0.16155	TRUE	1.00E+00
32168_s_at	-0.02868	-1.068267	0.035524	-0.19257	0.13522	TRUE	1.00E+00
39081_at	-0.02866	-1.068218	0.017547	-0.10962	0.05229	TRUE	1.00E+00
affx-hsac07/x003	-0.02866	-1.068218	0.050964	-0.26379	0.20647	TRUE	1.00E+00

38386_r_at	-0.02864	-1.068169	0.021117	-0.12607	0.06878	TRUE	1.00E+00
40804_at	-0.02862	-1.06812	0.043424	-0.22896	0.17172	TRUE	1.00E+00
36645_at	-0.02861	-1.068095	0.012809	-0.0877	0.03049	TRUE	1.00E+00
35948_at	-0.02861	-1.068095	0.064602	-0.32665	0.26944	TRUE	1.00E+00
33949_at	-0.02857	-1.067997	0.019572	-0.11887	0.06173	TRUE	1.00E+00
39437_at	-0.02856	-1.067972	0.013981	-0.09306	0.03595	TRUE	1.00E+00
38305_at	-0.02853	-1.067899	0.048086	-0.25038	0.19332	TRUE	1.00E+00
31960_f_at	-0.02851	-1.067849	0.084006	-0.41608	0.35905	TRUE	1.00E+00
39936_at	-0.02849	-1.0678	0.048886	-0.25402	0.19705	TRUE	1.00E+00
35874_at	-0.02846	-1.067726	0.069473	-0.34898	0.29206	TRUE	1.00E+00
1257_s_at	-0.02842	-1.067628	0.01305	-0.08863	0.03179	TRUE	1.00E+00
41640_at	-0.0284	-1.067579	0.023911	-0.13872	0.08191	TRUE	1.00E+00
41306_at	-0.02838	-1.06753	0.023509	-0.13684	0.08008	TRUE	1.00E+00
37839_at	-0.02834	-1.067431	0.046889	-0.24467	0.18799	TRUE	1.00E+00
31837_at	-0.02832	-1.067382	0.022381	-0.13158	0.07493	TRUE	1.00E+00
40531_at	-0.02826	-1.067235	0.020817	-0.1243	0.06778	TRUE	1.00E+00
2044_s_at	-0.02826	-1.067235	0.020291	-0.12187	0.06536	TRUE	1.00E+00
32708_g_at	-0.02822	-1.067137	0.036196	-0.19522	0.13878	TRUE	1.00E+00
40092_at	-0.02822	-1.067137	0.035182	-0.19053	0.1341	TRUE	1.00E+00
40826_at	-0.02817	-1.067014	0.012586	-0.08624	0.0299	TRUE	1.00E+00
34104_i_at	-0.02816	-1.066989	0.049247	-0.25536	0.19904	TRUE	1.00E+00
41548_at	-0.02812	-1.066891	0.074493	-0.3718	0.31556	TRUE	1.00E+00
1360_at	-0.02812	-1.066891	0.025655	-0.14648	0.09025	TRUE	1.00E+00
34127_at	-0.0281	-1.066842	0.059826	-0.30412	0.24791	TRUE	1.00E+00
2066_at	-0.02806	-1.066743	0.024413	-0.14069	0.08458	TRUE	1.00E+00
41213_at	-0.02806	-1.066743	0.019378	-0.11746	0.06135	TRUE	1.00E+00
38821_at	-0.02805	-1.066719	0.026559	-0.15058	0.09449	TRUE	1.00E+00
36581_at	-0.02802	-1.066645	0.009292	-0.07089	0.01485	TRUE	1.00E+00
31512_at	-0.028	-1.066596	0.101035	-0.49413	0.43813	TRUE	1.00E+00
31393_r_at	-0.02799	-1.066572	0.02986	-0.16575	0.10977	TRUE	1.00E+00
38076_at	-0.02795	-1.066473	0.033352	-0.18182	0.12592	TRUE	1.00E+00
41168_at	-0.02792	-1.0664	0.020909	-0.12438	0.06855	TRUE	1.00E+00
40846_g_at	-0.0279	-1.066351	0.017648	-0.10932	0.05351	TRUE	1.00E+00
40289_at	-0.02788	-1.066301	0.030219	-0.1673	0.11154	TRUE	1.00E+00
34156_i_at	-0.02783	-1.066179	0.063267	-0.31972	0.26406	TRUE	1.00E+00
33528_at	-0.02783	-1.066179	0.092798	-0.45596	0.4003	TRUE	1.00E+00
34199_at	-0.02782	-1.066154	0.017437	-0.10827	0.05263	TRUE	1.00E+00
36709_at	-0.0278	-1.066105	0.047534	-0.2471	0.1915	TRUE	1.00E+00
39534_at	-0.0278	-1.066105	0.030763	-0.16973	0.11413	TRUE	1.00E+00
41752_at	-0.02778	-1.066056	0.024094	-0.13894	0.08338	TRUE	1.00E+00
34418_at	-0.02778	-1.066056	0.112931	-0.5488	0.49324	TRUE	1.00E+00
35695_at	-0.02777	-1.066031	0.018707	-0.11408	0.05853	TRUE	1.00E+00
40115_at	-0.02777	-1.066031	0.013558	-0.09032	0.03478	TRUE	1.00E+00
1878_g_at	-0.02776	-1.066007	0.015447	-0.09903	0.0435	TRUE	1.00E+00
33725_at	-0.02768	-1.065811	0.055719	-0.28474	0.22938	TRUE	1.00E+00
39367_at	-0.02767	-1.065786	0.024638	-0.14135	0.086	TRUE	1.00E+00
40447_at	-0.02765	-1.065737	0.026545	-0.15012	0.09481	TRUE	1.00E+00
35238_at	-0.02764	-1.065712	0.031541	-0.17316	0.11788	TRUE	1.00E+00
41741_at	-0.02763	-1.065688	0.017005	-0.10608	0.05083	TRUE	1.00E+00
35022_at	-0.02761	-1.065639	0.123362	-0.59676	0.54153	TRUE	1.00E+00
32830_g_at	-0.0276	-1.065614	0.01731	-0.10746	0.05226	TRUE	1.00E+00

647_at	-0.02758	-1.065565	0.051434	-0.26488	0.20971	TRUE	1.00E+00
37596_at	-0.02757	-1.065541	0.064253	-0.32401	0.26886	TRUE	1.00E+00
34575_f_at	-0.02752	-1.065418	0.023406	-0.1355	0.08047	TRUE	1.00E+00
36883_at	-0.02752	-1.065418	0.028464	-0.15884	0.1038	TRUE	1.00E+00
33361_at	-0.02751	-1.065393	0.010445	-0.0757	0.02067	TRUE	1.00E+00
39909_g_at	-0.02751	-1.065393	0.028717	-0.16	0.10498	TRUE	1.00E+00
1133_at	-0.0275	-1.065369	0.071797	-0.35874	0.30375	TRUE	1.00E+00
33220_at	-0.02747	-1.065295	0.024787	-0.14183	0.08689	TRUE	1.00E+00
31874_at	-0.02747	-1.065295	0.013433	-0.08945	0.03451	TRUE	1.00E+00
40955_at	-0.02746	-1.065271	0.023322	-0.13506	0.08014	TRUE	1.00E+00
33898_at	-0.02745	-1.065246	0.021575	-0.127	0.07209	TRUE	1.00E+00
1905_s_at	-0.02745	-1.065246	0.024054	-0.13843	0.08352	TRUE	1.00E+00
1801_at	-0.02742	-1.065173	0.043209	-0.22677	0.17193	TRUE	1.00E+00
33398_at	-0.02741	-1.065148	0.04853	-0.2513	0.19649	TRUE	1.00E+00
33148_at	-0.0274	-1.065124	0.039879	-0.21139	0.15658	TRUE	1.00E+00
2005_s_at	-0.02738	-1.065075	0.018282	-0.11173	0.05696	TRUE	1.00E+00
37568_at	-0.02733	-1.064952	0.152087	-0.729	0.67434	TRUE	1.00E+00
35170_at	-0.02733	-1.064952	0.020261	-0.1208	0.06615	TRUE	1.00E+00
40283_at	-0.02732	-1.064927	0.039463	-0.20939	0.15474	TRUE	1.00E+00
38564_at	-0.02732	-1.064927	0.043956	-0.23012	0.17548	TRUE	1.00E+00
40958_at	-0.0273	-1.064878	0.023519	-0.1358	0.08121	TRUE	1.00E+00
40509_at	-0.02729	-1.064854	0.020672	-0.12266	0.06808	TRUE	1.00E+00
303_at	-0.02729	-1.064854	0.045374	-0.23662	0.18205	TRUE	1.00E+00
37128_at	-0.02727	-1.064805	0.040102	-0.21228	0.15775	TRUE	1.00E+00
38878_at	-0.02726	-1.06478	0.038935	-0.20689	0.15236	TRUE	1.00E+00
388_at	-0.02725	-1.064756	0.015823	-0.10025	0.04575	TRUE	1.00E+00
32869_at	-0.02724	-1.064731	0.041949	-0.22078	0.16629	TRUE	1.00E+00
36964_at	-0.02721	-1.064658	0.027535	-0.15425	0.09982	TRUE	1.00E+00
39225_at	-0.02715	-1.064511	0.020721	-0.12275	0.06845	TRUE	1.00E+00
36052_at	-0.02708	-1.064339	0.03333	-0.18085	0.12669	TRUE	1.00E+00
33859_at	-0.02706	-1.06429	0.021742	-0.12736	0.07325	TRUE	1.00E+00
41499_at	-0.02705	-1.064266	0.039494	-0.20926	0.15516	TRUE	1.00E+00
35273_at	-0.02704	-1.064241	0.011626	-0.08068	0.0266	TRUE	1.00E+00
34625_at	-0.02704	-1.064241	0.039089	-0.20738	0.15331	TRUE	1.00E+00
1901_s_at	-0.02701	-1.064168	0.029848	-0.16472	0.1107	TRUE	1.00E+00
40925_at	-0.02699	-1.064119	0.053388	-0.2733	0.21931	TRUE	1.00E+00
1104_s_at	-0.02696	-1.064045	0.029114	-0.16128	0.10735	TRUE	1.00E+00
36211_at	-0.02696	-1.064045	0.029028	-0.16089	0.10696	TRUE	1.00E+00
764_s_at	-0.02693	-1.063972	0.118108	-0.57183	0.51797	TRUE	1.00E+00
36846_s_at	-0.02691	-1.063923	0.009154	-0.06914	0.01533	TRUE	1.00E+00
32888_at	-0.0269	-1.063898	0.037268	-0.19884	0.14504	TRUE	1.00E+00
39354_at	-0.02687	-1.063825	0.032184	-0.17536	0.12161	TRUE	1.00E+00
1200_at	-0.02686	-1.0638	0.044152	-0.23056	0.17684	TRUE	1.00E+00
32276_at	-0.02685	-1.063776	0.012203	-0.08315	0.02945	TRUE	1.00E+00
33004_g_at	-0.02681	-1.063678	0.091516	-0.44902	0.39541	TRUE	1.00E+00
38447_at	-0.02679	-1.063629	0.027907	-0.15554	0.10196	TRUE	1.00E+00
37916_at	-0.02678	-1.063604	0.015784	-0.09959	0.04604	TRUE	1.00E+00
31709_at	-0.02675	-1.063531	0.02468	-0.14062	0.08711	TRUE	1.00E+00
39988_at	-0.02675	-1.063531	0.025598	-0.14485	0.09135	TRUE	1.00E+00
37731_at	-0.02674	-1.063506	0.01833	-0.11131	0.05783	TRUE	1.00E+00
35567_at	-0.02674	-1.063506	0.025676	-0.1452	0.09172	TRUE	1.00E+00

38107_at	-0.02671	-1.063433	0.015803	-0.09962	0.04619	TRUE	1.00E+00
37491_at	-0.02669	-1.063384	0.020384	-0.12073	0.06735	TRUE	1.00E+00
31640_r_at	-0.02667	-1.063335	0.018339	-0.11128	0.05794	TRUE	1.00E+00
38478_at	-0.02666	-1.06331	0.017102	-0.10557	0.05224	TRUE	1.00E+00
38161_at	-0.02663	-1.063237	0.021381	-0.12528	0.07201	TRUE	1.00E+00
35653_at	-0.02662	-1.063212	0.012857	-0.08593	0.0327	TRUE	1.00E+00
598_at	-0.0266	-1.063163	0.030144	-0.16568	0.11247	TRUE	1.00E+00
40094_r_at	-0.02658	-1.063114	0.02566	-0.14496	0.09181	TRUE	1.00E+00
36840_at	-0.02657	-1.06309	0.012546	-0.08445	0.03132	TRUE	1.00E+00
41637_at	-0.02656	-1.063065	0.020419	-0.12077	0.06764	TRUE	1.00E+00
32703_at	-0.02651	-1.062943	0.141407	-0.67891	0.62588	TRUE	1.00E+00
1603_g_at	-0.0265	-1.062919	0.027951	-0.15546	0.10245	TRUE	1.00E+00
37911_at	-0.02646	-1.062821	0.018958	-0.11393	0.061	TRUE	1.00E+00
32806_at	-0.02645	-1.062796	0.077112	-0.38221	0.32931	TRUE	1.00E+00
38643_at	-0.02644	-1.062772	0.039106	-0.20686	0.15398	TRUE	1.00E+00
37362_at	-0.02643	-1.062747	0.031442	-0.17149	0.11863	TRUE	1.00E+00
38747_at	-0.0264	-1.062674	0.022927	-0.13218	0.07938	TRUE	1.00E+00
35173_at	-0.02638	-1.062625	0.047037	-0.24339	0.19063	TRUE	1.00E+00
37134_f_at	-0.02638	-1.062625	0.073459	-0.36529	0.31253	TRUE	1.00E+00
36716_at	-0.02637	-1.0626	0.112696	-0.5463	0.49357	TRUE	1.00E+00
37455_at	-0.02635	-1.062552	0.064245	-0.32276	0.27005	TRUE	1.00E+00
33471_g_at	-0.02634	-1.062527	0.032911	-0.17817	0.1255	TRUE	1.00E+00
36237_at	-0.02631	-1.062454	0.042704	-0.22332	0.17071	TRUE	1.00E+00
1475_s_at	-0.02631	-1.062454	0.018445	-0.1114	0.05879	TRUE	1.00E+00
160041_at	-0.02628	-1.06238	0.053244	-0.27192	0.21937	TRUE	1.00E+00
41807_at	-0.02623	-1.062258	0.014215	-0.09181	0.03935	TRUE	1.00E+00
41612_at	-0.02622	-1.062234	0.016989	-0.1046	0.05216	TRUE	1.00E+00
41584_at	-0.0262	-1.062185	0.012919	-0.0858	0.0334	TRUE	1.00E+00
41655_at	-0.02618	-1.062136	0.025258	-0.14271	0.09035	TRUE	1.00E+00
34462_at	-0.02617	-1.062111	0.032579	-0.17648	0.12413	TRUE	1.00E+00
31593_at	-0.02612	-1.061989	0.011796	-0.08054	0.0283	TRUE	1.00E+00
31866_at	-0.02612	-1.061989	0.01522	-0.09633	0.0441	TRUE	1.00E+00
41473_at	-0.02609	-1.061916	0.067447	-0.33726	0.28508	TRUE	1.00E+00
36290_s_at	-0.02607	-1.061867	0.042576	-0.22249	0.17036	TRUE	1.00E+00
1060_g_at	-0.02605	-1.061818	0.080937	-0.39946	0.34736	TRUE	1.00E+00
39505_at	-0.02601	-1.06172	0.029047	-0.16002	0.108	TRUE	1.00E+00
39802_at	-0.026	-1.061696	0.042696	-0.22298	0.17098	TRUE	1.00E+00
34704_r_at	-0.02597	-1.061622	0.08062	-0.39791	0.34598	TRUE	1.00E+00
1206_at	-0.02596	-1.061598	0.02976	-0.16326	0.11134	TRUE	1.00E+00
35388_at	-0.02596	-1.061598	0.02069	-0.12141	0.06949	TRUE	1.00E+00
37464_at	-0.02594	-1.061549	0.032869	-0.17758	0.12571	TRUE	1.00E+00
34640_at	-0.02591	-1.061476	0.02121	-0.12377	0.07195	TRUE	1.00E+00
33034_at	-0.02587	-1.061378	0.026582	-0.14851	0.09676	TRUE	1.00E+00
31826_at	-0.02587	-1.061378	0.008947	-0.06715	0.01541	TRUE	1.00E+00
32517_at	-0.0258	-1.061207	0.023902	-0.13607	0.08448	TRUE	1.00E+00
35403_at	-0.02578	-1.061158	0.018111	-0.10934	0.05778	TRUE	1.00E+00
32826_at	-0.02575	-1.061085	0.087904	-0.43131	0.3798	TRUE	1.00E+00
1676_s_at	-0.02575	-1.061085	0.021951	-0.12703	0.07552	TRUE	1.00E+00
31632_at	-0.02572	-1.061011	0.097719	-0.47656	0.42511	TRUE	1.00E+00
34159_at	-0.02572	-1.061011	0.084137	-0.41389	0.36246	TRUE	1.00E+00
40195_at	-0.02572	-1.061011	0.064605	-0.32377	0.27234	TRUE	1.00E+00

515_s_at	-0.02571	-1.060987	0.091629	-0.44845	0.39703	TRUE	1.00E+00
41107_at	-0.0257	-1.060962	0.024602	-0.1392	0.0878	TRUE	1.00E+00
33456_at	-0.02569	-1.060938	0.018649	-0.11173	0.06035	TRUE	1.00E+00
36727_at	-0.02568	-1.060914	0.023697	-0.13501	0.08364	TRUE	1.00E+00
36717_at	-0.02564	-1.060816	0.029618	-0.16229	0.111	TRUE	1.00E+00
36778_at	-0.02562	-1.060767	0.111108	-0.53823	0.48698	TRUE	1.00E+00
35600_at	-0.02562	-1.060767	0.055431	-0.28135	0.23012	TRUE	1.00E+00
affx-crex-5_at	-0.02562	-1.060767	0.573407	-2.67108	2.61984	TRUE	1.00E+00
32852_at	-0.02559	-1.060694	0.023363	-0.13338	0.08219	TRUE	1.00E+00
41565_at	-0.02559	-1.060694	0.022366	-0.12878	0.0776	TRUE	1.00E+00
32358_at	-0.02555	-1.060596	0.026923	-0.14976	0.09866	TRUE	1.00E+00
1746_s_at	-0.02549	-1.06045	0.009896	-0.07114	0.02017	TRUE	1.00E+00
37471_at	-0.02546	-1.060376	0.029326	-0.16076	0.10983	TRUE	1.00E+00
36220_at	-0.02546	-1.060376	0.081445	-0.40121	0.3503	TRUE	1.00E+00
31717_at	-0.02543	-1.060303	0.040394	-0.21179	0.16093	TRUE	1.00E+00
31384_at	-0.02542	-1.060279	0.02724	-0.15109	0.10025	TRUE	1.00E+00
660_at	-0.0254	-1.06023	0.092284	-0.45116	0.40036	TRUE	1.00E+00
1940_at	-0.02539	-1.060205	0.02416	-0.13685	0.08607	TRUE	1.00E+00
39184_at	-0.02534	-1.060083	0.016718	-0.10247	0.05179	TRUE	1.00E+00
40768_s_at	-0.02534	-1.060083	0.014659	-0.09297	0.04229	TRUE	1.00E+00
1932_at	-0.02528	-1.059937	0.063551	-0.31848	0.26792	TRUE	1.00E+00
31955_at	-0.02524	-1.059839	0.020757	-0.121	0.07052	TRUE	1.00E+00
37450_r_at	-0.02523	-1.059815	0.022641	-0.12969	0.07922	TRUE	1.00E+00
41792_at	-0.02521	-1.059766	0.021506	-0.12443	0.07401	TRUE	1.00E+00
40262_at	-0.0252	-1.059742	0.022101	-0.12716	0.07676	TRUE	1.00E+00
35359_at	-0.02508	-1.059449	0.020392	-0.11916	0.069	TRUE	1.00E+00
39094_at	-0.02505	-1.059376	0.029188	-0.15972	0.10961	TRUE	1.00E+00
38054_at	-0.02502	-1.059303	0.011803	-0.07948	0.02944	TRUE	1.00E+00
1155_at	-0.02502	-1.059303	0.027461	-0.15171	0.10168	TRUE	1.00E+00
33788_at	-0.02498	-1.059205	0.009652	-0.06951	0.01955	TRUE	1.00E+00
35783_at	-0.02498	-1.059205	0.01274	-0.08375	0.0338	TRUE	1.00E+00
34700_at	-0.02496	-1.059156	0.056254	-0.28449	0.23457	TRUE	1.00E+00
31876_r_at	-0.02493	-1.059083	0.05872	-0.29584	0.24598	TRUE	1.00E+00
37510_at	-0.02489	-1.058985	0.023482	-0.13323	0.08344	TRUE	1.00E+00
36729_g_at	-0.02488	-1.058961	0.025946	-0.14458	0.09482	TRUE	1.00E+00
33625_g_at	-0.02486	-1.058912	0.041104	-0.2145	0.16477	TRUE	1.00E+00
38879_at	-0.02484	-1.058864	0.055764	-0.28211	0.23243	TRUE	1.00E+00
36378_at	-0.02484	-1.058864	0.033047	-0.17731	0.12762	TRUE	1.00E+00
517_at	-0.02483	-1.058839	0.021165	-0.12248	0.07281	TRUE	1.00E+00
37582_at	-0.0248	-1.058766	0.027559	-0.15194	0.10235	TRUE	1.00E+00
890_at	-0.02478	-1.058717	0.013788	-0.08839	0.03883	TRUE	1.00E+00
39615_at	-0.02478	-1.058717	0.018041	-0.10801	0.05846	TRUE	1.00E+00
1974_s_at	-0.02477	-1.058693	0.020999	-0.12165	0.07211	TRUE	1.00E+00
40669_at	-0.02472	-1.058571	0.064328	-0.32151	0.27206	TRUE	1.00E+00
31579_at	-0.02472	-1.058571	0.036943	-0.19516	0.14572	TRUE	1.00E+00
37518_at	-0.02471	-1.058547	0.04501	-0.23237	0.18295	TRUE	1.00E+00
35781_g_at	-0.02471	-1.058547	0.031829	-0.17155	0.12214	TRUE	1.00E+00
35756_at	-0.02471	-1.058547	0.017156	-0.10386	0.05444	TRUE	1.00E+00
40278_at	-0.0247	-1.058522	0.028722	-0.15721	0.10781	TRUE	1.00E+00
38337_at	-0.02462	-1.058327	0.016813	-0.10219	0.05295	TRUE	1.00E+00
35950_at	-0.02461	-1.058303	0.041527	-0.2162	0.16698	TRUE	1.00E+00

37991_at	-0.02459	-1.058254	0.021551	-0.12402	0.07484	TRUE	1.00E+00
39715_at	-0.02445	-1.057913	0.03845	-0.20184	0.15295	TRUE	1.00E+00
1440_s_at	-0.02441	-1.057816	0.058235	-0.29309	0.24426	TRUE	1.00E+00
36479_at	-0.02441	-1.057816	0.020301	-0.11807	0.06926	TRUE	1.00E+00
39861_at	-0.02431	-1.057572	0.022219	-0.12682	0.0782	TRUE	1.00E+00
39789_at	-0.02429	-1.057523	0.036038	-0.19055	0.14198	TRUE	1.00E+00
38320_s_at	-0.02428	-1.057499	0.032312	-0.17336	0.12479	TRUE	1.00E+00
37515_at	-0.02416	-1.057207	0.016777	-0.10156	0.05325	TRUE	1.00E+00
1677_at	-0.02409	-1.057037	0.029595	-0.16063	0.11245	TRUE	1.00E+00
32193_at	-0.024	-1.056818	0.14049	-0.67217	0.62416	TRUE	1.00E+00
31660_at	-0.02399	-1.056793	0.030084	-0.16279	0.1148	TRUE	1.00E+00
34692_r_at	-0.02396	-1.05672	0.015404	-0.09503	0.04711	TRUE	1.00E+00
40800_at	-0.0239	-1.056574	0.057193	-0.28777	0.23996	TRUE	1.00E+00
240_at	-0.02389	-1.05655	0.022218	-0.1264	0.07861	TRUE	1.00E+00
32741_at	-0.02387	-1.056501	0.035612	-0.18817	0.14043	TRUE	1.00E+00
38981_at	-0.02384	-1.056428	0.030892	-0.16636	0.11869	TRUE	1.00E+00
36981_at	-0.02384	-1.056428	0.018052	-0.10712	0.05945	TRUE	1.00E+00
40322_at	-0.02382	-1.05638	0.053094	-0.26878	0.22114	TRUE	1.00E+00
620_at	-0.02378	-1.056282	0.039249	-0.20486	0.1573	TRUE	1.00E+00
36756_at	-0.02378	-1.056282	0.055698	-0.28075	0.23319	TRUE	1.00E+00
1560_g_at	-0.02378	-1.056282	0.058744	-0.2948	0.24724	TRUE	1.00E+00
32611_at	-0.02376	-1.056234	0.044924	-0.23102	0.1835	TRUE	1.00E+00
31528_f_at	-0.02374	-1.056185	0.057541	-0.28922	0.24173	TRUE	1.00E+00
730_r_at	-0.02374	-1.056185	0.025381	-0.14084	0.09336	TRUE	1.00E+00
39152_f_at	-0.02365	-1.055966	0.017564	-0.10468	0.05739	TRUE	1.00E+00
40810_at	-0.02364	-1.055942	0.023691	-0.13294	0.08566	TRUE	1.00E+00
41745_at	-0.02357	-1.055772	0.01741	-0.10389	0.05675	TRUE	1.00E+00
41002_at	-0.02356	-1.055747	0.019852	-0.11515	0.06802	TRUE	1.00E+00
33475_at	-0.02355	-1.055723	0.059213	-0.29673	0.24963	TRUE	1.00E+00
1289_at	-0.02355	-1.055723	0.030192	-0.16284	0.11575	TRUE	1.00E+00
37955_at	-0.02354	-1.055699	0.020947	-0.12018	0.0731	TRUE	1.00E+00
35312_at	-0.02349	-1.055577	0.043286	-0.22319	0.17621	TRUE	1.00E+00
36847_r_at	-0.02342	-1.055407	0.048155	-0.24559	0.19874	TRUE	1.00E+00
36854_s_at	-0.02342	-1.055407	0.048976	-0.24938	0.20254	TRUE	1.00E+00
32848_at	-0.02341	-1.055383	0.028011	-0.15265	0.10582	TRUE	1.00E+00
36357_at	-0.02337	-1.055286	0.049714	-0.25274	0.20599	TRUE	1.00E+00
37270_at	-0.0233	-1.055115	0.039143	-0.20389	0.15729	TRUE	1.00E+00
37677_at	-0.02328	-1.055067	0.014198	-0.08879	0.04222	TRUE	1.00E+00
36913_at	-0.02327	-1.055043	0.0432	-0.22258	0.17603	TRUE	1.00E+00
35231_at	-0.02323	-1.054945	0.019762	-0.11441	0.06794	TRUE	1.00E+00
40688_at	-0.0232	-1.054873	0.062787	-0.31287	0.26648	TRUE	1.00E+00
967_g_at	-0.02318	-1.054824	0.07578	-0.37279	0.32644	TRUE	1.00E+00
1711_at	-0.02318	-1.054824	0.014851	-0.09169	0.04534	TRUE	1.00E+00
37880_at	-0.02317	-1.0548	0.029492	-0.15923	0.11289	TRUE	1.00E+00
1637_at	-0.02315	-1.054751	0.032324	-0.17228	0.12598	TRUE	1.00E+00
38741_at	-0.02315	-1.054751	0.021506	-0.12236	0.07607	TRUE	1.00E+00
35759_at	-0.02308	-1.054581	0.020069	-0.11567	0.06951	TRUE	1.00E+00
32507_at	-0.02303	-1.05446	0.099907	-0.48396	0.4379	TRUE	1.00E+00
41287_s_at	-0.02301	-1.054411	0.040826	-0.21137	0.16534	TRUE	1.00E+00
1541_f_at	-0.02298	-1.054338	0.052404	-0.26475	0.21879	TRUE	1.00E+00
41435_at	-0.02296	-1.05429	0.019053	-0.11086	0.06494	TRUE	1.00E+00

1681_at	-0.02294	-1.054241	0.021975	-0.12433	0.07844	TRUE	1.00E+00
41603_at	-0.02284	-1.053999	0.01391	-0.08702	0.04133	TRUE	1.00E+00
39791_at	-0.02281	-1.053926	0.024868	-0.13754	0.09192	TRUE	1.00E+00
39400_at	-0.02276	-1.053804	0.037426	-0.19543	0.14991	TRUE	1.00E+00
31481_s_at	-0.02276	-1.053804	0.01276	-0.08163	0.03611	TRUE	1.00E+00
35426_at	-0.02275	-1.05378	0.044593	-0.22848	0.18298	TRUE	1.00E+00
34216_at	-0.02273	-1.053732	0.034346	-0.18119	0.13573	TRUE	1.00E+00
34745_at	-0.02272	-1.053707	0.022057	-0.12448	0.07904	TRUE	1.00E+00
39264_at	-0.02271	-1.053683	0.103822	-0.5017	0.45628	TRUE	1.00E+00
37508_f_at	-0.0227	-1.053659	0.038652	-0.20102	0.15563	TRUE	1.00E+00
31354_r_at	-0.02267	-1.053586	0.041133	-0.21244	0.1671	TRUE	1.00E+00
36083_at	-0.02261	-1.05344	0.023819	-0.1325	0.08728	TRUE	1.00E+00
32786_at	-0.02255	-1.053295	0.043811	-0.22467	0.17957	TRUE	1.00E+00
32810_at	-0.02251	-1.053198	0.024213	-0.13422	0.0892	TRUE	1.00E+00
35968_s_at	-0.02248	-1.053125	0.022849	-0.1279	0.08294	TRUE	1.00E+00
34442_at	-0.02238	-1.052883	0.066092	-0.3273	0.28254	TRUE	1.00E+00
38791_at	-0.02237	-1.052858	0.014814	-0.09072	0.04598	TRUE	1.00E+00
33473_at	-0.02237	-1.052858	0.102329	-0.49447	0.44973	TRUE	1.00E+00
32027_at	-0.02236	-1.052834	0.030872	-0.16479	0.12007	TRUE	1.00E+00
35279_at	-0.0223	-1.052689	0.022752	-0.12727	0.08266	TRUE	1.00E+00
37237_at	-0.02229	-1.052665	0.016983	-0.10064	0.05607	TRUE	1.00E+00
31362_at	-0.02228	-1.05264	0.039036	-0.20237	0.15782	TRUE	1.00E+00
36135_at	-0.02227	-1.052616	0.027507	-0.14918	0.10463	TRUE	1.00E+00
38155_at	-0.02225	-1.052568	0.016604	-0.09886	0.05436	TRUE	1.00E+00
34753_at	-0.02223	-1.052519	0.009789	-0.0674	0.02293	TRUE	1.00E+00
37553_at	-0.02223	-1.052519	0.013345	-0.0838	0.03934	TRUE	1.00E+00
affx-biob-m_at	-0.02211	-1.052228	0.060868	-0.30293	0.25871	TRUE	1.00E+00
34161_at	-0.02209	-1.05218	0.028429	-0.15325	0.10907	TRUE	1.00E+00
2011_s_at	-0.02209	-1.05218	0.077535	-0.3798	0.33563	TRUE	1.00E+00
35283_at	-0.02205	-1.052083	0.017371	-0.10219	0.05809	TRUE	1.00E+00
36702_at	-0.02202	-1.05201	0.018493	-0.10733	0.0633	TRUE	1.00E+00
41799_at	-0.02199	-1.051938	0.029124	-0.15635	0.11238	TRUE	1.00E+00
35399_at	-0.02198	-1.051913	0.039243	-0.20303	0.15907	TRUE	1.00E+00
40937_at	-0.02197	-1.051889	0.038281	-0.19858	0.15465	TRUE	1.00E+00
41507_at	-0.02192	-1.051768	0.09317	-0.45177	0.40793	TRUE	1.00E+00
33896_at	-0.0219	-1.05172	0.050284	-0.25389	0.21009	TRUE	1.00E+00
36060_at	-0.02189	-1.051695	0.016072	-0.09604	0.05226	TRUE	1.00E+00
40604_at	-0.02188	-1.051671	0.043209	-0.22123	0.17747	TRUE	1.00E+00
38523_f_at	-0.02182	-1.051526	0.043908	-0.2244	0.18075	TRUE	1.00E+00
32957_g_at	-0.0218	-1.051478	0.058314	-0.29084	0.24723	TRUE	1.00E+00
34274_at	-0.02177	-1.051405	0.0107	-0.07114	0.0276	TRUE	1.00E+00
39059_at	-0.02177	-1.051405	0.03064	-0.16313	0.11959	TRUE	1.00E+00
38622_at	-0.02175	-1.051356	0.021928	-0.12292	0.07942	TRUE	1.00E+00
35395_at	-0.02175	-1.051356	0.074721	-0.36648	0.32299	TRUE	1.00E+00
41233_at	-0.02175	-1.051356	0.016725	-0.09891	0.05541	TRUE	1.00E+00
101_at	-0.02173	-1.051308	0.024976	-0.13696	0.09349	TRUE	1.00E+00
36128_at	-0.02173	-1.051308	0.01649	-0.09781	0.05435	TRUE	1.00E+00
41415_at	-0.02172	-1.051284	0.017408	-0.10203	0.0586	TRUE	1.00E+00
1684_s_at	-0.02171	-1.05126	0.024638	-0.13538	0.09196	TRUE	1.00E+00
33335_at	-0.02171	-1.05126	0.038087	-0.19743	0.15401	TRUE	1.00E+00
40876_at	-0.02169	-1.051211	0.0237	-0.13103	0.08766	TRUE	1.00E+00

504_at	-0.02168	-1.051187	0.022604	-0.12597	0.0826	TRUE	1.00E+00
36891_at	-0.02168	-1.051187	0.029091	-0.15589	0.11254	TRUE	1.00E+00
35996_at	-0.0216	-1.050993	0.03121	-0.16559	0.12239	TRUE	1.00E+00
36164_at	-0.02153	-1.050824	0.010475	-0.06986	0.0268	TRUE	1.00E+00
41314_at	-0.02153	-1.050824	0.021441	-0.12045	0.07739	TRUE	1.00E+00
34740_at	-0.02152	-1.0508	0.020297	-0.11516	0.07212	TRUE	1.00E+00
35413_s_at	-0.02149	-1.050727	0.024523	-0.13463	0.09165	TRUE	1.00E+00
40966_at	-0.02148	-1.050703	0.020023	-0.11385	0.0709	TRUE	1.00E+00
32132_at	-0.0214	-1.05051	0.049042	-0.24766	0.20486	TRUE	1.00E+00
41498_at	-0.02137	-1.050437	0.01988	-0.11309	0.07035	TRUE	1.00E+00
358_at	-0.02129	-1.050243	0.055221	-0.27605	0.23348	TRUE	1.00E+00
37736_at	-0.02128	-1.050219	0.013991	-0.08583	0.04327	TRUE	1.00E+00
36251_at	-0.02128	-1.050219	0.024633	-0.13493	0.09237	TRUE	1.00E+00
41602_at	-0.02127	-1.050195	0.019092	-0.10935	0.06681	TRUE	1.00E+00
31489_at	-0.02125	-1.050147	0.179372	-0.8488	0.8063	TRUE	1.00E+00
213_at	-0.02121	-1.05005	0.052695	-0.26432	0.2219	TRUE	1.00E+00
958_s_at	-0.02121	-1.05005	0.01604	-0.09521	0.0528	TRUE	1.00E+00
32626_at	-0.02118	-1.049978	0.019165	-0.1096	0.06724	TRUE	1.00E+00
39796_at	-0.02118	-1.049978	0.010338	-0.06887	0.02651	TRUE	1.00E+00
31581_at	-0.02118	-1.049978	0.052339	-0.26265	0.22029	TRUE	1.00E+00
39057_at	-0.02117	-1.049953	0.01569	-0.09356	0.05122	TRUE	1.00E+00
34116_at	-0.02116	-1.049929	0.05126	-0.25766	0.21533	TRUE	1.00E+00
affx-crex-5_st	-0.02113	-1.049857	0.114688	-0.55025	0.508	TRUE	1.00E+00
32197_at	-0.02109	-1.04976	0.02875	-0.15372	0.11155	TRUE	1.00E+00
34758_at	-0.02108	-1.049736	0.023465	-0.12934	0.08718	TRUE	1.00E+00
32002_at	-0.02107	-1.049712	0.039579	-0.20367	0.16153	TRUE	1.00E+00
32048_at	-0.02106	-1.049687	0.052639	-0.26392	0.22179	TRUE	1.00E+00
37274_at	-0.02103	-1.049615	0.019642	-0.11165	0.06959	TRUE	1.00E+00
32928_at	-0.02099	-1.049518	0.063995	-0.31624	0.27425	TRUE	1.00E+00
33991_g_at	-0.02099	-1.049518	0.095314	-0.46073	0.41875	TRUE	1.00E+00
35681_r_at	-0.02099	-1.049518	0.032832	-0.17246	0.13049	TRUE	1.00E+00
33940_at	-0.02097	-1.04947	0.089593	-0.43431	0.39238	TRUE	1.00E+00
40259_at	-0.02093	-1.049373	0.025951	-0.14065	0.0988	TRUE	1.00E+00
41793_at	-0.02092	-1.049349	0.011739	-0.07507	0.03324	TRUE	1.00E+00
34374_g_at	-0.02088	-1.049252	0.0284	-0.1519	0.11015	TRUE	1.00E+00
39378_at	-0.02087	-1.049228	0.01102	-0.07171	0.02997	TRUE	1.00E+00
36074_at	-0.02085	-1.04918	0.036958	-0.19136	0.14965	TRUE	1.00E+00
32717_at	-0.02085	-1.04918	0.017742	-0.1027	0.061	TRUE	1.00E+00
39516_at	-0.02079	-1.049035	0.012946	-0.08051	0.03894	TRUE	1.00E+00
34536_g_at	-0.02076	-1.048963	0.040515	-0.20768	0.16616	TRUE	1.00E+00
39838_at	-0.02076	-1.048963	0.037031	-0.19161	0.15008	TRUE	1.00E+00
1381_at	-0.02073	-1.04889	0.068526	-0.33688	0.29542	TRUE	1.00E+00
36501_at	-0.0207	-1.048818	0.023007	-0.12684	0.08544	TRUE	1.00E+00
38712_at	-0.0207	-1.048818	0.021451	-0.11967	0.07827	TRUE	1.00E+00
35881_at	-0.02069	-1.048794	0.077064	-0.37623	0.33485	TRUE	1.00E+00
323_at	-0.02065	-1.048697	0.014662	-0.08829	0.047	TRUE	1.00E+00
33433_at	-0.02062	-1.048624	0.04072	-0.20849	0.16724	TRUE	1.00E+00
40634_at	-0.02059	-1.048552	0.045021	-0.2283	0.18712	TRUE	1.00E+00
38103_at	-0.02059	-1.048552	0.026425	-0.1425	0.10133	TRUE	1.00E+00
37070_at	-0.02054	-1.048431	0.042021	-0.2144	0.17333	TRUE	1.00E+00
38019_at	-0.02053	-1.048407	0.027703	-0.14834	0.10728	TRUE	1.00E+00

34906_g_at	-0.02053	-1.048407	0.014625	-0.08801	0.04694	TRUE	1.00E+00
451_at	-0.02052	-1.048383	0.079519	-0.38739	0.34635	TRUE	1.00E+00
39376_at	-0.02051	-1.048359	0.022684	-0.12517	0.08414	TRUE	1.00E+00
100_g_at	-0.02051	-1.048359	0.021904	-0.12157	0.08054	TRUE	1.00E+00
34069_s_at	-0.02046	-1.048238	0.020947	-0.1171	0.07619	TRUE	1.00E+00
38332_at	-0.02044	-1.04819	0.03341	-0.17458	0.1337	TRUE	1.00E+00
36614_at	-0.02044	-1.04819	0.015263	-0.09086	0.04998	TRUE	1.00E+00
40196_at	-0.02043	-1.048166	0.01368	-0.08355	0.04269	TRUE	1.00E+00
39729_at	-0.02042	-1.048142	0.016216	-0.09524	0.05439	TRUE	1.00E+00
34124_at	-0.02036	-1.047997	0.040796	-0.20858	0.16786	TRUE	1.00E+00
34178_at	-0.0203	-1.047852	0.012831	-0.07949	0.0389	TRUE	1.00E+00
1170_at	-0.02027	-1.04778	0.044519	-0.22566	0.18512	TRUE	1.00E+00
41337_at	-0.02023	-1.047683	0.020906	-0.11669	0.07622	TRUE	1.00E+00
35140_at	-0.02022	-1.047659	0.017522	-0.10106	0.06062	TRUE	1.00E+00
31373_at	-0.02021	-1.047635	0.105365	-0.50632	0.46591	TRUE	1.00E+00
1908_at	-0.0202	-1.047611	0.039654	-0.20315	0.16275	TRUE	1.00E+00
36962_at	-0.0202	-1.047611	0.015305	-0.0908	0.05041	TRUE	1.00E+00
39913_at	-0.02019	-1.047587	0.014498	-0.08707	0.0467	TRUE	1.00E+00
41192_at	-0.02018	-1.047563	0.046443	-0.23445	0.19409	TRUE	1.00E+00
1127_at	-0.02017	-1.047539	0.028894	-0.15348	0.11313	TRUE	1.00E+00
35523_at	-0.02017	-1.047539	0.032372	-0.16952	0.12918	TRUE	1.00E+00
39856_at	-0.02013	-1.047442	0.019136	-0.10842	0.06816	TRUE	1.00E+00
34957_at	-0.02011	-1.047394	0.026269	-0.14131	0.10108	TRUE	1.00E+00
35924_at	-0.0201	-1.04737	0.029096	-0.15433	0.11414	TRUE	1.00E+00
41149_at	-0.02009	-1.047346	0.038858	-0.19937	0.15919	TRUE	1.00E+00
40890_at	-0.02008	-1.047321	0.014484	-0.08691	0.04674	TRUE	1.00E+00
38613_at	-0.02007	-1.047297	0.022493	-0.12385	0.0837	TRUE	1.00E+00
32188_at	-0.02004	-1.047225	0.030008	-0.15849	0.11841	TRUE	1.00E+00
40337_at	-0.02003	-1.047201	0.019293	-0.10904	0.06898	TRUE	1.00E+00
170_at	-0.02001	-1.047153	0.02588	-0.13941	0.09939	TRUE	1.00E+00
799_at	-0.01999	-1.047104	0.02809	-0.14959	0.1096	TRUE	1.00E+00
38118_at	-0.01999	-1.047104	0.015106	-0.08968	0.0497	TRUE	1.00E+00
1399_at	-0.01997	-1.047056	0.022654	-0.12448	0.08455	TRUE	1.00E+00
1142_at	-0.01996	-1.047032	0.022306	-0.12287	0.08295	TRUE	1.00E+00
34233_i_at	-0.01991	-1.046912	0.060332	-0.29825	0.25844	TRUE	1.00E+00
33090_at	-0.0199	-1.046887	0.076878	-0.37458	0.33479	TRUE	1.00E+00
31887_at	-0.01982	-1.046695	0.039238	-0.20085	0.1612	TRUE	1.00E+00
35156_at	-0.0198	-1.046646	0.021994	-0.12127	0.08167	TRUE	1.00E+00
40720_at	-0.01974	-1.046502	0.076064	-0.37067	0.33119	TRUE	1.00E+00
37606_at	-0.01972	-1.046454	0.092	-0.44417	0.40473	TRUE	1.00E+00
34787_at	-0.01972	-1.046454	0.038903	-0.1992	0.15976	TRUE	1.00E+00
33557_at	-0.01965	-1.046285	0.134003	-0.63789	0.59858	TRUE	1.00E+00
36072_at	-0.01963	-1.046237	0.023332	-0.12727	0.08802	TRUE	1.00E+00
33227_at	-0.01962	-1.046213	0.040139	-0.20481	0.16556	TRUE	1.00E+00
38531_at	-0.01945	-1.045803	0.024346	-0.13177	0.09287	TRUE	1.00E+00
32699_s_at	-0.01945	-1.045803	0.025671	-0.13788	0.09899	TRUE	1.00E+00
40086_at	-0.01943	-1.045755	0.019228	-0.10814	0.06928	TRUE	1.00E+00
31847_at	-0.01942	-1.045731	0.032636	-0.16998	0.13115	TRUE	1.00E+00
1699_at	-0.01941	-1.045707	0.113822	-0.54454	0.50572	TRUE	1.00E+00
34232_at	-0.01939	-1.045659	0.12808	-0.6103	0.57152	TRUE	1.00E+00
33568_at	-0.01937	-1.045611	0.018878	-0.10646	0.06773	TRUE	1.00E+00

37443_at	-0.01934	-1.045538	0.041662	-0.21156	0.17287	TRUE	1.00E+00
38973_at	-0.01933	-1.045514	0.0144	-0.08577	0.04711	TRUE	1.00E+00
37392_at	-0.0193	-1.045442	0.033449	-0.17362	0.13502	TRUE	1.00E+00
39286_at	-0.01927	-1.04537	0.02145	-0.11823	0.07969	TRUE	1.00E+00
39490_f_at	-0.01922	-1.04525	0.021839	-0.11998	0.08153	TRUE	1.00E+00
39143_at	-0.01921	-1.045226	0.071029	-0.34691	0.30849	TRUE	1.00E+00
34315_at	-0.01921	-1.045226	0.010803	-0.06904	0.03063	TRUE	1.00E+00
36354_at	-0.01918	-1.045153	0.054429	-0.27029	0.23194	TRUE	1.00E+00
36936_at	-0.01917	-1.045129	0.020277	-0.11272	0.07438	TRUE	1.00E+00
37580_at	-0.01915	-1.045081	0.042265	-0.21415	0.17584	TRUE	1.00E+00
1906_at	-0.01914	-1.045057	0.022481	-0.12286	0.08458	TRUE	1.00E+00
36793_at	-0.01913	-1.045033	0.137342	-0.65277	0.61451	TRUE	1.00E+00
34498_at	-0.01908	-1.044913	0.08448	-0.40883	0.37068	TRUE	1.00E+00
35853_at	-0.01907	-1.044889	0.011239	-0.07092	0.03279	TRUE	1.00E+00
31889_at	-0.01902	-1.044768	0.042568	-0.21541	0.17737	TRUE	1.00E+00
41181_r_at	-0.01897	-1.044648	0.012298	-0.07571	0.03777	TRUE	1.00E+00
affx-humgapdh/m	-0.01893	-1.044552	0.058276	-0.28779	0.24993	TRUE	1.00E+00
666_at	-0.01887	-1.044408	0.018996	-0.10651	0.06877	TRUE	1.00E+00
36956_at	-0.01883	-1.044311	0.037354	-0.19116	0.15351	TRUE	1.00E+00
40000_f_at	-0.01879	-1.044215	0.033181	-0.17187	0.1343	TRUE	1.00E+00
36078_at	-0.01878	-1.044191	0.050129	-0.25006	0.21249	TRUE	1.00E+00
34344_at	-0.01878	-1.044191	0.033261	-0.17223	0.13467	TRUE	1.00E+00
39216_at	-0.01874	-1.044095	0.086237	-0.4166	0.37912	TRUE	1.00E+00
39258_at	-0.01874	-1.044095	0.033628	-0.17389	0.13641	TRUE	1.00E+00
39280_at	-0.01874	-1.044095	0.017947	-0.10154	0.06406	TRUE	1.00E+00
31877_at	-0.01871	-1.044023	0.016823	-0.09632	0.0589	TRUE	1.00E+00
31907_at	-0.01871	-1.044023	0.020532	-0.11343	0.07601	TRUE	1.00E+00
344_s_at	-0.0187	-1.043999	0.023629	-0.12771	0.09031	TRUE	1.00E+00
36276_at	-0.01869	-1.043975	0.017182	-0.09796	0.06058	TRUE	1.00E+00
1338_s_at	-0.01869	-1.043975	0.043839	-0.22094	0.18357	TRUE	1.00E+00
32544_s_at	-0.01864	-1.043855	0.021898	-0.11967	0.08239	TRUE	1.00E+00
39380_at	-0.01863	-1.043831	0.015809	-0.09156	0.05431	TRUE	1.00E+00
1522_at	-0.0186	-1.043758	0.043967	-0.22144	0.18425	TRUE	1.00E+00
32774_at	-0.01859	-1.043734	0.024002	-0.12932	0.09215	TRUE	1.00E+00
31704_at	-0.01857	-1.043686	0.036046	-0.18487	0.14773	TRUE	1.00E+00
31308_at	-0.01856	-1.043662	0.113995	-0.54449	0.50736	TRUE	1.00E+00
41363_at	-0.01855	-1.043638	0.028842	-0.15162	0.11451	TRUE	1.00E+00
34240_s_at	-0.01849	-1.043494	0.041785	-0.21127	0.17429	TRUE	1.00E+00
34790_at	-0.01846	-1.043422	0.055949	-0.27659	0.23967	TRUE	1.00E+00
36799_at	-0.01842	-1.043326	0.028554	-0.15015	0.11332	TRUE	1.00E+00
39873_at	-0.01829	-1.043014	0.026793	-0.14191	0.10532	TRUE	1.00E+00
33546_at	-0.01829	-1.043014	0.024947	-0.13338	0.09681	TRUE	1.00E+00
941_at	-0.01826	-1.042942	0.017552	-0.09924	0.06272	TRUE	1.00E+00
33045_r_at	-0.01824	-1.042894	0.041881	-0.21146	0.17498	TRUE	1.00E+00
37798_at	-0.01823	-1.04287	0.03109	-0.16167	0.1252	TRUE	1.00E+00
32228_at	-0.01823	-1.04287	0.022761	-0.12324	0.08678	TRUE	1.00E+00
1365_at	-0.01814	-1.042653	0.053778	-0.26625	0.22997	TRUE	1.00E+00
36223_at	-0.01811	-1.042581	0.167865	-0.79257	0.75635	TRUE	1.00E+00
37288_g_at	-0.0181	-1.042557	0.087867	-0.42348	0.38728	TRUE	1.00E+00
40991_at	-0.01807	-1.042485	0.03754	-0.19127	0.15512	TRUE	1.00E+00
40641_at	-0.01803	-1.042389	0.022278	-0.12081	0.08475	TRUE	1.00E+00

39586_at	-0.018	-1.042317	0.061184	-0.30028	0.26428	TRUE	1.00E+00
40945_at	-0.01796	-1.042221	0.04704	-0.23498	0.19907	TRUE	1.00E+00
36494_at	-0.01795	-1.042197	0.034146	-0.17548	0.13959	TRUE	1.00E+00
39747_at	-0.01792	-1.042125	0.028043	-0.1473	0.11146	TRUE	1.00E+00
40751_at	-0.01792	-1.042125	0.029484	-0.15394	0.11811	TRUE	1.00E+00
1481_at	-0.01791	-1.042101	0.040155	-0.20317	0.16734	TRUE	1.00E+00
34573_at	-0.01789	-1.042053	0.076194	-0.36942	0.33363	TRUE	1.00E+00
33419_at	-0.01788	-1.042029	0.046681	-0.23325	0.19748	TRUE	1.00E+00
970_r_at	-0.01787	-1.042005	0.037072	-0.18891	0.15316	TRUE	1.00E+00
31510_s_at	-0.01786	-1.041981	0.015334	-0.08861	0.05288	TRUE	1.00E+00
36522_at	-0.01783	-1.04191	0.031701	-0.16409	0.12842	TRUE	1.00E+00
35219_at	-0.01779	-1.041814	0.011709	-0.07181	0.03623	TRUE	1.00E+00
810_at	-0.01778	-1.04179	0.018747	-0.10427	0.06871	TRUE	1.00E+00
33575_at	-0.01776	-1.041742	0.056404	-0.27798	0.24246	TRUE	1.00E+00
35060_at	-0.01775	-1.041718	0.037236	-0.18954	0.15405	TRUE	1.00E+00
38603_at	-0.01775	-1.041718	0.034185	-0.17546	0.13997	TRUE	1.00E+00
911_s_at	-0.01772	-1.041646	0.020781	-0.1136	0.07815	TRUE	1.00E+00
33522_at	-0.01771	-1.041622	0.091754	-0.44103	0.4056	TRUE	1.00E+00
32487_s_at	-0.01771	-1.041622	0.021034	-0.11476	0.07933	TRUE	1.00E+00
41670_at	-0.0177	-1.041598	0.037097	-0.18885	0.15344	TRUE	1.00E+00
36373_at	-0.01769	-1.041574	0.021619	-0.11743	0.08205	TRUE	1.00E+00
37894_at	-0.01769	-1.041574	0.010996	-0.06842	0.03304	TRUE	1.00E+00
37026_at	-0.01766	-1.041502	0.035371	-0.18085	0.14553	TRUE	1.00E+00
40640_at	-0.01764	-1.041454	0.022466	-0.12129	0.08601	TRUE	1.00E+00
33038_at	-0.01764	-1.041454	0.07352	-0.35683	0.32155	TRUE	1.00E+00
1543_at	-0.01763	-1.04143	0.051876	-0.25696	0.22171	TRUE	1.00E+00
33924_at	-0.01756	-1.041262	0.020977	-0.11434	0.07922	TRUE	1.00E+00
35134_at	-0.01752	-1.041166	0.080439	-0.38863	0.35359	TRUE	1.00E+00
41076_at	-0.0175	-1.041118	0.030047	-0.15612	0.12113	TRUE	1.00E+00
31312_at	-0.01748	-1.04107	0.097285	-0.46631	0.43135	TRUE	1.00E+00
35863_g_at	-0.01746	-1.041022	0.099212	-0.47519	0.44026	TRUE	1.00E+00
32853_at	-0.01745	-1.040998	0.01413	-0.08264	0.04775	TRUE	1.00E+00
35012_at	-0.01742	-1.040926	0.069423	-0.33771	0.30287	TRUE	1.00E+00
39980_at	-0.01741	-1.040902	0.052706	-0.26057	0.22576	TRUE	1.00E+00
38450_at	-0.0174	-1.040878	0.026788	-0.14099	0.10619	TRUE	1.00E+00
34816_at	-0.01738	-1.04083	0.031046	-0.16061	0.12585	TRUE	1.00E+00
36372_at	-0.01732	-1.040687	0.028806	-0.15022	0.11558	TRUE	1.00E+00
854_at	-0.01731	-1.040663	0.040806	-0.20557	0.17096	TRUE	1.00E+00
37860_at	-0.0173	-1.040639	0.058304	-0.28629	0.25169	TRUE	1.00E+00
38903_at	-0.0173	-1.040639	0.054109	-0.26694	0.23234	TRUE	1.00E+00
33646_g_at	-0.01729	-1.040615	0.034943	-0.1785	0.14392	TRUE	1.00E+00
40481_r_at	-0.01728	-1.040591	0.089769	-0.43144	0.39687	TRUE	1.00E+00
33386_at	-0.01726	-1.040543	0.034838	-0.17799	0.14347	TRUE	1.00E+00
31754_at	-0.01726	-1.040543	0.069059	-0.33586	0.30135	TRUE	1.00E+00
1394_at	-0.01719	-1.040375	0.053592	-0.26444	0.23006	TRUE	1.00E+00
38272_at	-0.01716	-1.040303	0.019527	-0.10725	0.07293	TRUE	1.00E+00
41262_at	-0.01705	-1.04004	0.029081	-0.15122	0.11712	TRUE	1.00E+00
40946_at	-0.01703	-1.039992	0.026725	-0.14033	0.10627	TRUE	1.00E+00
36428_at	-0.01701	-1.039944	0.033047	-0.16948	0.13545	TRUE	1.00E+00
40647_at	-0.01701	-1.039944	0.036964	-0.18754	0.15353	TRUE	1.00E+00
34742_at	-0.017	-1.03992	0.024473	-0.12991	0.09591	TRUE	1.00E+00

32739_at	-0.01696	-1.039824	0.028656	-0.14917	0.11524	TRUE	1.00E+00
1340_s_at	-0.01695	-1.0398	0.05588	-0.27476	0.24086	TRUE	1.00E+00
34960_g_at	-0.01695	-1.0398	0.017524	-0.09779	0.0639	TRUE	1.00E+00
31511_at	-0.01692	-1.039729	0.031362	-0.16161	0.12777	TRUE	1.00E+00
35025_at	-0.01691	-1.039705	0.048026	-0.23848	0.20467	TRUE	1.00E+00
32392_s_at	-0.01688	-1.039633	0.038424	-0.19415	0.16039	TRUE	1.00E+00
1994_at	-0.01688	-1.039633	0.027493	-0.14372	0.10996	TRUE	1.00E+00
33285_i_at	-0.01687	-1.039609	0.040231	-0.20249	0.16874	TRUE	1.00E+00
33121_g_at	-0.01682	-1.039489	0.046681	-0.23218	0.19855	TRUE	1.00E+00
1148_s_at	-0.01681	-1.039465	0.064696	-0.31529	0.28167	TRUE	1.00E+00
38883_at	-0.0168	-1.039441	0.021549	-0.11621	0.08262	TRUE	1.00E+00
41468_at	-0.01678	-1.039394	0.057265	-0.28098	0.24742	TRUE	1.00E+00
39155_at	-0.01678	-1.039394	0.033942	-0.17337	0.13982	TRUE	1.00E+00
1993_s_at	-0.01677	-1.03937	0.065805	-0.32037	0.28683	TRUE	1.00E+00
35902_at	-0.01676	-1.039346	0.052366	-0.25836	0.22483	TRUE	1.00E+00
35464_at	-0.01676	-1.039346	0.105563	-0.50378	0.47027	TRUE	1.00E+00
40791_at	-0.01675	-1.039322	0.030765	-0.15869	0.12519	TRUE	1.00E+00
31893_at	-0.01673	-1.039274	0.021339	-0.11518	0.08172	TRUE	1.00E+00
39311_at	-0.01673	-1.039274	0.053006	-0.26127	0.22782	TRUE	1.00E+00
40661_at	-0.01672	-1.03925	0.029246	-0.15165	0.11821	TRUE	1.00E+00
39782_at	-0.01671	-1.039226	0.007887	-0.0531	0.01967	TRUE	1.00E+00
40843_at	-0.01669	-1.039178	0.030104	-0.15558	0.1222	TRUE	1.00E+00
40930_at	-0.01668	-1.039154	0.043869	-0.21908	0.18571	TRUE	1.00E+00
39596_at	-0.01657	-1.038891	0.071567	-0.34675	0.31361	TRUE	1.00E+00
33960_s_at	-0.01655	-1.038843	0.066916	-0.32527	0.29217	TRUE	1.00E+00
41626_at	-0.01653	-1.038795	0.044055	-0.21978	0.18673	TRUE	1.00E+00
33994_g_at	-0.01651	-1.038748	0.075493	-0.3648	0.33178	TRUE	1.00E+00
40254_s_at	-0.0165	-1.038724	0.098741	-0.47205	0.43905	TRUE	1.00E+00
806_at	-0.01648	-1.038676	0.024111	-0.12772	0.09476	TRUE	1.00E+00
38988_at	-0.01642	-1.038532	0.024098	-0.1276	0.09476	TRUE	1.00E+00
40241_at	-0.0164	-1.038484	0.038322	-0.19321	0.1604	TRUE	1.00E+00
35148_at	-0.01636	-1.038389	0.095271	-0.4559	0.42318	TRUE	1.00E+00
32104_i_at	-0.01636	-1.038389	0.017809	-0.09853	0.0658	TRUE	1.00E+00
35573_r_at	-0.01636	-1.038389	0.095762	-0.45817	0.42545	TRUE	1.00E+00
33581_at	-0.01634	-1.038341	0.023619	-0.12531	0.09262	TRUE	1.00E+00
36473_at	-0.01632	-1.038293	0.009127	-0.05842	0.02579	TRUE	1.00E+00
39522_at	-0.01626	-1.03815	0.029146	-0.15073	0.1182	TRUE	1.00E+00
32318_s_at	-0.01626	-1.03815	0.022654	-0.12077	0.08826	TRUE	1.00E+00
34546_at	-0.01625	-1.038126	0.040898	-0.20494	0.17244	TRUE	1.00E+00
35715_at	-0.01619	-1.037982	0.017258	-0.09581	0.06343	TRUE	1.00E+00
34345_at	-0.01619	-1.037982	0.016284	-0.09132	0.05894	TRUE	1.00E+00
affx-biob-3_st	-0.01614	-1.037863	0.022136	-0.11827	0.08598	TRUE	1.00E+00
36110_at	-0.01611	-1.037791	0.016973	-0.09442	0.06219	TRUE	1.00E+00
40992_s_at	-0.01606	-1.037672	0.036977	-0.18665	0.15453	TRUE	1.00E+00
38680_at	-0.01605	-1.037648	0.015831	-0.08909	0.05698	TRUE	1.00E+00
39819_at	-0.01605	-1.037648	0.038513	-0.19373	0.16164	TRUE	1.00E+00
37981_at	-0.01604	-1.037624	0.008812	-0.0567	0.02461	TRUE	1.00E+00
40840_at	-0.01602	-1.037576	0.025799	-0.13505	0.103	TRUE	1.00E+00
32572_at	-0.01601	-1.037552	0.020091	-0.1087	0.07669	TRUE	1.00E+00
38662_at	-0.01598	-1.037481	0.029569	-0.1524	0.12044	TRUE	1.00E+00
33082_at	-0.01597	-1.037457	0.117063	-0.55605	0.52412	TRUE	1.00E+00

38716_at	-0.01596	-1.037433	0.025504	-0.13363	0.10171	TRUE	1.00E+00
35070_at	-0.01595	-1.037409	0.061397	-0.29921	0.26731	TRUE	1.00E+00
38924_s_at	-0.01594	-1.037385	0.016342	-0.09134	0.05945	TRUE	1.00E+00
1654_at	-0.01588	-1.037242	0.048459	-0.23945	0.20769	TRUE	1.00E+00
35343_at	-0.01588	-1.037242	0.023889	-0.1261	0.09433	TRUE	1.00E+00
1875_f_at	-0.01586	-1.037194	0.031489	-0.16113	0.12942	TRUE	1.00E+00
32839_at	-0.01585	-1.03717	0.094256	-0.45071	0.419	TRUE	1.00E+00
39721_at	-0.01579	-1.037027	0.030861	-0.15817	0.12659	TRUE	1.00E+00
36172_s_at	-0.01574	-1.036907	0.010556	-0.06444	0.03296	TRUE	1.00E+00
768_at	-0.01567	-1.03674	0.052086	-0.25597	0.22463	TRUE	1.00E+00
37753_at	-0.01563	-1.036645	0.028592	-0.14754	0.11629	TRUE	1.00E+00
36420_at	-0.01558	-1.036526	0.125075	-0.59262	0.56147	TRUE	1.00E+00
41111_at	-0.01557	-1.036502	0.021542	-0.11496	0.08381	TRUE	1.00E+00
41264_at	-0.01557	-1.036502	0.023551	-0.12422	0.09309	TRUE	1.00E+00
38340_at	-0.01555	-1.036454	0.016263	-0.09058	0.05948	TRUE	1.00E+00
38831_f_at	-0.01554	-1.03643	0.010243	-0.0628	0.03172	TRUE	1.00E+00
36601_at	-0.01553	-1.036406	0.03317	-0.16857	0.1375	TRUE	1.00E+00
40130_at	-0.01553	-1.036406	0.00908	-0.05742	0.02636	TRUE	1.00E+00
34135_at	-0.01553	-1.036406	0.022738	-0.12043	0.08938	TRUE	1.00E+00
32584_at	-0.01552	-1.036382	0.015315	-0.08618	0.05513	TRUE	1.00E+00
40941_at	-0.0155	-1.036335	0.028776	-0.14825	0.11726	TRUE	1.00E+00
697_f_at	-0.01549	-1.036311	0.018394	-0.10035	0.06937	TRUE	1.00E+00
36113_s_at	-0.01548	-1.036287	0.040917	-0.20425	0.1733	TRUE	1.00E+00
37586_at	-0.01546	-1.036239	0.022011	-0.11701	0.08608	TRUE	1.00E+00
35955_at	-0.01545	-1.036215	0.024209	-0.12714	0.09624	TRUE	1.00E+00
36285_at	-0.01544	-1.036191	0.019289	-0.10443	0.07355	TRUE	1.00E+00
34665_g_at	-0.01538	-1.036048	0.141554	-0.66845	0.63769	TRUE	1.00E+00
33198_at	-0.01537	-1.036024	0.013264	-0.07657	0.04582	TRUE	1.00E+00
37818_at	-0.0153	-1.035857	0.015041	-0.08469	0.05409	TRUE	1.00E+00
41532_at	-0.01521	-1.035643	0.011355	-0.0676	0.03717	TRUE	1.00E+00
40460_s_at	-0.01521	-1.035643	0.03235	-0.16446	0.13404	TRUE	1.00E+00
731_f_at	-0.0152	-1.035619	0.028632	-0.1473	0.11689	TRUE	1.00E+00
31365_f_at	-0.01519	-1.035595	0.059778	-0.29098	0.26061	TRUE	1.00E+00
38607_at	-0.01515	-1.0355	0.017141	-0.09423	0.06393	TRUE	1.00E+00
39968_at	-0.01512	-1.035428	0.032061	-0.16304	0.13279	TRUE	1.00E+00
158_at	-0.01512	-1.035428	0.043582	-0.21619	0.18596	TRUE	1.00E+00
1320_at	-0.01509	-1.035357	0.030297	-0.15487	0.12468	TRUE	1.00E+00
41053_at	-0.01503	-1.035214	0.116259	-0.5514	0.52134	TRUE	1.00E+00
39160_at	-0.01494	-1.034999	0.020612	-0.11003	0.08016	TRUE	1.00E+00
31931_f_at	-0.01493	-1.034975	0.032328	-0.16407	0.13422	TRUE	1.00E+00
35240_at	-0.01492	-1.034952	0.063227	-0.30662	0.27678	TRUE	1.00E+00
38637_at	-0.01491	-1.034928	0.016914	-0.09294	0.06313	TRUE	1.00E+00
33381_at	-0.01488	-1.034856	0.02738	-0.1412	0.11144	TRUE	1.00E+00
39070_at	-0.01485	-1.034785	0.035086	-0.17673	0.14702	TRUE	1.00E+00
34197_at	-0.01483	-1.034737	0.021816	-0.11548	0.08582	TRUE	1.00E+00
34405_at	-0.01477	-1.034594	0.0423	-0.20993	0.18038	TRUE	1.00E+00
41840_r_at	-0.01475	-1.034546	0.043133	-0.21375	0.18425	TRUE	1.00E+00
32456_s_at	-0.01472	-1.034475	0.081098	-0.38887	0.35943	TRUE	1.00E+00
41025_r_at	-0.01471	-1.034451	0.026964	-0.13911	0.10969	TRUE	1.00E+00
36080_at	-0.01468	-1.03438	0.014133	-0.07989	0.05052	TRUE	1.00E+00
33360_at	-0.01467	-1.034356	0.01362	-0.07751	0.04816	TRUE	1.00E+00

40027_at	-0.01465	-1.034308	0.023394	-0.12258	0.09328	TRUE	1.00E+00
38433_at	-0.01458	-1.034142	0.028146	-0.14444	0.11527	TRUE	1.00E+00
34474_at	-0.01456	-1.034094	0.041481	-0.20594	0.17681	TRUE	1.00E+00
33146_at	-0.01456	-1.034094	0.016301	-0.08977	0.06065	TRUE	1.00E+00
40809_at	-0.01454	-1.034046	0.039577	-0.19713	0.16805	TRUE	1.00E+00
34169_s_at	-0.01451	-1.033975	0.011797	-0.06894	0.03991	TRUE	1.00E+00
33145_at	-0.01451	-1.033975	0.024412	-0.12714	0.09812	TRUE	1.00E+00
398_at	-0.01449	-1.033927	0.018465	-0.09968	0.0707	TRUE	1.00E+00
41606_at	-0.01447	-1.03388	0.018546	-0.10003	0.0711	TRUE	1.00E+00
35360_at	-0.01446	-1.033856	0.022057	-0.11623	0.0873	TRUE	1.00E+00
33417_at	-0.01446	-1.033856	0.017733	-0.09628	0.06735	TRUE	1.00E+00
428_s_at	-0.01441	-1.033737	0.020144	-0.10735	0.07852	TRUE	1.00E+00
36444_s_at	-0.01441	-1.033737	0.082115	-0.39326	0.36444	TRUE	1.00E+00
31681_at	-0.0144	-1.033713	0.029609	-0.15101	0.1222	TRUE	1.00E+00
41395_at	-0.01439	-1.033689	0.020685	-0.10983	0.08104	TRUE	1.00E+00
40486_g_at	-0.01437	-1.033642	0.018833	-0.10126	0.07252	TRUE	1.00E+00
41526_at	-0.01428	-1.033427	0.028677	-0.14658	0.11803	TRUE	1.00E+00
38974_at	-0.01421	-1.033261	0.025476	-0.13175	0.10332	TRUE	1.00E+00
34623_at	-0.01421	-1.033261	0.039665	-0.19721	0.16879	TRUE	1.00E+00
34915_at	-0.01419	-1.033213	0.053863	-0.26269	0.23431	TRUE	1.00E+00
41331_at	-0.01417	-1.033166	0.031535	-0.15966	0.13131	TRUE	1.00E+00
39167_r_at	-0.01412	-1.033047	0.023508	-0.12258	0.09433	TRUE	1.00E+00
affx-yel024w/rip1	-0.01408	-1.032952	0.032955	-0.16612	0.13796	TRUE	1.00E+00
32547_at	-0.01403	-1.032833	0.016315	-0.0893	0.06124	TRUE	1.00E+00
38299_at	-0.01394	-1.032619	0.137223	-0.64703	0.61915	TRUE	1.00E+00
1587_at	-0.01392	-1.032571	0.038937	-0.19355	0.16572	TRUE	1.00E+00
34186_at	-0.01385	-1.032405	0.096134	-0.45737	0.42967	TRUE	1.00E+00
31509_at	-0.01381	-1.03231	0.018546	-0.09937	0.07175	TRUE	1.00E+00
444_g_at	-0.0138	-1.032286	0.025081	-0.12951	0.10191	TRUE	1.00E+00
35815_at	-0.01377	-1.032215	0.023116	-0.12042	0.09288	TRUE	1.00E+00
39748_at	-0.01377	-1.032215	0.024966	-0.12895	0.10142	TRUE	1.00E+00
40121_at	-0.0137	-1.032048	0.018564	-0.09935	0.07194	TRUE	1.00E+00
39835_at	-0.0137	-1.032048	0.024358	-0.12608	0.09867	TRUE	1.00E+00
39046_at	-0.01368	-1.032001	0.025772	-0.13259	0.10522	TRUE	1.00E+00
33893_r_at	-0.01362	-1.031858	0.031063	-0.15693	0.12969	TRUE	1.00E+00
35762_at	-0.01361	-1.031834	0.024947	-0.1287	0.10148	TRUE	1.00E+00
37675_at	-0.01357	-1.031739	0.02015	-0.10653	0.07939	TRUE	1.00E+00
39684_at	-0.01351	-1.031597	0.024899	-0.12838	0.10136	TRUE	1.00E+00
683_at	-0.01351	-1.031597	0.069919	-0.33609	0.30907	TRUE	1.00E+00
33319_at	-0.01351	-1.031597	0.0628	-0.30324	0.27623	TRUE	1.00E+00
38059_g_at	-0.01349	-1.031549	0.082148	-0.39249	0.36551	TRUE	1.00E+00
38626_at	-0.01346	-1.031478	0.013665	-0.0765	0.04959	TRUE	1.00E+00
35530_f_at	-0.01339	-1.031312	0.014213	-0.07897	0.05218	TRUE	1.00E+00
34491_at	-0.01339	-1.031312	0.067451	-0.32458	0.2978	TRUE	1.00E+00
31700_at	-0.01336	-1.031241	0.018489	-0.09866	0.07194	TRUE	1.00E+00
744_at	-0.01331	-1.031122	0.014012	-0.07795	0.05133	TRUE	1.00E+00
33242_at	-0.01331	-1.031122	0.04037	-0.19956	0.17294	TRUE	1.00E+00
31676_at	-0.01329	-1.031074	0.048697	-0.23796	0.21138	TRUE	1.00E+00
32960_at	-0.01327	-1.031027	0.020819	-0.10932	0.08278	TRUE	1.00E+00
32315_at	-0.01325	-1.030979	0.013792	-0.07688	0.05038	TRUE	1.00E+00
31390_at	-0.01325	-1.030979	0.045484	-0.22309	0.1966	TRUE	1.00E+00

1987_at	-0.01323	-1.030932	0.036372	-0.18104	0.15457	TRUE	1.00E+00
32766_at	-0.01315	-1.030742	0.015148	-0.08304	0.05673	TRUE	1.00E+00
33832_at	-0.0131	-1.030623	0.112727	-0.53318	0.50697	TRUE	1.00E+00
34319_at	-0.01309	-1.0306	0.038681	-0.19155	0.16537	TRUE	1.00E+00
934_at	-0.01307	-1.030552	0.056346	-0.27303	0.24688	TRUE	1.00E+00
33778_at	-0.01307	-1.030552	0.017372	-0.09322	0.06708	TRUE	1.00E+00
34826_at	-0.01298	-1.030339	0.016765	-0.09032	0.06437	TRUE	1.00E+00
1900_at	-0.01297	-1.030315	0.060067	-0.2901	0.26415	TRUE	1.00E+00
34064_s_at	-0.01297	-1.030315	0.020233	-0.10632	0.08038	TRUE	1.00E+00
35577_at	-0.01294	-1.030244	0.059712	-0.28843	0.26255	TRUE	1.00E+00
41431_at	-0.01287	-1.030078	0.024406	-0.12547	0.09973	TRUE	1.00E+00
33811_at	-0.01287	-1.030078	0.019612	-0.10335	0.07761	TRUE	1.00E+00
32820_at	-0.01286	-1.030054	0.011185	-0.06447	0.03874	TRUE	1.00E+00
32516_at	-0.01286	-1.030054	0.081205	-0.38751	0.36179	TRUE	1.00E+00
40558_at	-0.01285	-1.03003	0.025584	-0.13088	0.10519	TRUE	1.00E+00
41235_at	-0.01282	-1.029959	0.033987	-0.16962	0.14399	TRUE	1.00E+00
32629_f_at	-0.01281	-1.029935	0.038032	-0.18827	0.16265	TRUE	1.00E+00
33438_at	-0.0128	-1.029912	0.034747	-0.17311	0.14751	TRUE	1.00E+00
32894_at	-0.0128	-1.029912	0.019272	-0.10171	0.07611	TRUE	1.00E+00
33152_at	-0.01277	-1.029841	0.042402	-0.2084	0.18285	TRUE	1.00E+00
32254_at	-0.01272	-1.029722	0.023547	-0.12136	0.09591	TRUE	1.00E+00
40063_at	-0.01269	-1.029651	0.021372	-0.11129	0.08591	TRUE	1.00E+00
35623_at	-0.01269	-1.029651	0.027574	-0.1399	0.11453	TRUE	1.00E+00
39816_g_at	-0.01264	-1.029532	0.049427	-0.24067	0.2154	TRUE	1.00E+00
39485_g_at	-0.01262	-1.029485	0.028142	-0.14246	0.11722	TRUE	1.00E+00
36296_at	-0.0126	-1.029438	0.022488	-0.11635	0.09115	TRUE	1.00E+00
35195_at	-0.01259	-1.029414	0.02571	-0.13121	0.10603	TRUE	1.00E+00
33149_at	-0.01259	-1.029414	0.034821	-0.17323	0.14806	TRUE	1.00E+00
32266_at	-0.01257	-1.029366	0.114528	-0.54095	0.51582	TRUE	1.00E+00
39578_at	-0.01256	-1.029343	0.087841	-0.41782	0.39271	TRUE	1.00E+00
1571_f_at	-0.01256	-1.029343	0.058878	-0.28419	0.25908	TRUE	1.00E+00
908_at	-0.01251	-1.029224	0.028343	-0.14328	0.11825	TRUE	1.00E+00
38705_at	-0.01243	-1.029035	0.013816	-0.07617	0.05131	TRUE	1.00E+00
40960_at	-0.01242	-1.029011	0.018816	-0.09923	0.07439	TRUE	1.00E+00
41382_at	-0.0124	-1.028964	0.022814	-0.11765	0.09286	TRUE	1.00E+00
39826_f_at	-0.01238	-1.028916	0.056237	-0.27184	0.24707	TRUE	1.00E+00
35118_at	-0.01236	-1.028869	0.105276	-0.49806	0.47334	TRUE	1.00E+00
40812_at	-0.01235	-1.028845	0.094172	-0.44682	0.42213	TRUE	1.00E+00
331_at	-0.01235	-1.028845	0.024368	-0.12477	0.10007	TRUE	1.00E+00
41282_s_at	-0.01231	-1.02875	0.03141	-0.15722	0.1326	TRUE	1.00E+00
2046_at	-0.01228	-1.028679	0.018885	-0.09941	0.07485	TRUE	1.00E+00
32272_at	-0.01226	-1.028632	0.021518	-0.11153	0.08702	TRUE	1.00E+00
32251_at	-0.01224	-1.028585	0.016763	-0.08958	0.06509	TRUE	1.00E+00
558_at	-0.01224	-1.028585	0.04191	-0.20559	0.18111	TRUE	1.00E+00
40308_at	-0.01224	-1.028585	0.026682	-0.13533	0.11086	TRUE	1.00E+00
36960_at	-0.01222	-1.028537	0.018049	-0.09549	0.07105	TRUE	1.00E+00
1110_at	-0.01218	-1.028442	0.04482	-0.21896	0.1946	TRUE	1.00E+00
37526_at	-0.01215	-1.028371	0.083028	-0.3952	0.3709	TRUE	1.00E+00
34880_at	-0.01215	-1.028371	0.022755	-0.11713	0.09284	TRUE	1.00E+00
32367_at	-0.01212	-1.0283	0.10825	-0.51154	0.4873	TRUE	1.00E+00
40485_at	-0.01207	-1.028182	0.01777	-0.09405	0.06992	TRUE	1.00E+00

39750_at	-0.01206	-1.028158	0.037802	-0.18646	0.16234	TRUE	1.00E+00
567_s_at	-0.01205	-1.028135	0.022767	-0.11709	0.09299	TRUE	1.00E+00
1702_at	-0.01202	-1.028064	0.110272	-0.52077	0.49673	TRUE	1.00E+00
39000_at	-0.01196	-1.027922	0.015677	-0.08428	0.06037	TRUE	1.00E+00
821_s_at	-0.01196	-1.027922	0.052329	-0.25338	0.22947	TRUE	1.00E+00
41708_at	-0.01192	-1.027827	0.024327	-0.12415	0.10032	TRUE	1.00E+00
387_at	-0.01191	-1.027803	0.016169	-0.0865	0.06269	TRUE	1.00E+00
36862_at	-0.0119	-1.02778	0.033156	-0.16487	0.14106	TRUE	1.00E+00
40697_at	-0.01187	-1.027709	0.069549	-0.33274	0.309	TRUE	1.00E+00
33251_at	-0.01186	-1.027685	0.033298	-0.16549	0.14176	TRUE	1.00E+00
35062_at	-0.01182	-1.02759	0.071611	-0.34221	0.31856	TRUE	1.00E+00
41075_at	-0.01181	-1.027567	0.037246	-0.18364	0.16003	TRUE	1.00E+00
41579_s_at	-0.0118	-1.027543	0.017079	-0.09059	0.06699	TRUE	1.00E+00
37512_at	-0.01179	-1.027519	0.076284	-0.36373	0.34016	TRUE	1.00E+00
32519_at	-0.01178	-1.027496	0.031685	-0.15797	0.1344	TRUE	1.00E+00
1927_s_at	-0.01178	-1.027496	0.115916	-0.54657	0.52301	TRUE	1.00E+00
1138_at	-0.01177	-1.027472	0.053972	-0.26077	0.23724	TRUE	1.00E+00
1661_i_at	-0.01174	-1.027401	0.025628	-0.12998	0.10649	TRUE	1.00E+00
1999_s_at	-0.01171	-1.02733	0.036437	-0.17982	0.15639	TRUE	1.00E+00
37235_g_at	-0.01164	-1.027164	0.145426	-0.68258	0.65929	TRUE	1.00E+00
39500_s_at	-0.01162	-1.027117	0.026169	-0.13235	0.10912	TRUE	1.00E+00
35847_at	-0.01154	-1.026928	0.018037	-0.09475	0.07167	TRUE	1.00E+00
1957_s_at	-0.0115	-1.026833	0.083961	-0.39886	0.37586	TRUE	1.00E+00
593_s_at	-0.0115	-1.026833	0.095357	-0.45144	0.42844	TRUE	1.00E+00
887_at	-0.01147	-1.026763	0.029665	-0.14834	0.12539	TRUE	1.00E+00
40385_at	-0.01146	-1.026739	0.140101	-0.65782	0.63491	TRUE	1.00E+00
32205_at	-0.01145	-1.026715	0.017914	-0.09409	0.0712	TRUE	1.00E+00
38294_at	-0.01141	-1.026621	0.031034	-0.15459	0.13177	TRUE	1.00E+00
924_s_at	-0.01139	-1.026573	0.011143	-0.0628	0.04002	TRUE	1.00E+00
41820_s_at	-0.01137	-1.026526	0.066515	-0.31824	0.29551	TRUE	1.00E+00
41529_g_at	-0.01137	-1.026526	0.011759	-0.06562	0.04289	TRUE	1.00E+00
41509_at	-0.01135	-1.026479	0.046826	-0.22739	0.20469	TRUE	1.00E+00
38889_at	-0.01135	-1.026479	0.039288	-0.19261	0.16991	TRUE	1.00E+00
36675_r_at	-0.01135	-1.026479	0.031454	-0.15647	0.13377	TRUE	1.00E+00
37790_at	-0.01131	-1.026384	0.013251	-0.07245	0.04982	TRUE	1.00E+00
41297_at	-0.01127	-1.02629	0.051391	-0.24837	0.22582	TRUE	1.00E+00
41627_at	-0.01126	-1.026266	0.023778	-0.12097	0.09844	TRUE	1.00E+00
40816_at	-0.01119	-1.026101	0.045573	-0.22144	0.19907	TRUE	1.00E+00
32238_at	-0.01117	-1.026053	0.014755	-0.07925	0.0569	TRUE	1.00E+00
1431_at	-0.01116	-1.02603	0.04028	-0.19699	0.17468	TRUE	1.00E+00
36532_at	-0.01115	-1.026006	0.048558	-0.23518	0.21287	TRUE	1.00E+00
34725_at	-0.01113	-1.025959	0.069381	-0.33123	0.30896	TRUE	1.00E+00
37318_at	-0.01113	-1.025959	0.012832	-0.07033	0.04807	TRUE	1.00E+00
36687_at	-0.01113	-1.025959	0.01185	-0.06579	0.04354	TRUE	1.00E+00
38437_at	-0.01111	-1.025912	0.035761	-0.1761	0.15387	TRUE	1.00E+00
855_at	-0.01108	-1.025841	0.035591	-0.17529	0.15312	TRUE	1.00E+00
37358_at	-0.01108	-1.025841	0.04475	-0.21754	0.19538	TRUE	1.00E+00
32746_at	-0.01106	-1.025794	0.061575	-0.29514	0.27303	TRUE	1.00E+00
40365_at	-0.01105	-1.02577	0.059271	-0.2845	0.2624	TRUE	1.00E+00
32590_at	-0.01103	-1.025723	0.01305	-0.07124	0.04917	TRUE	1.00E+00
34658_at	-0.01101	-1.025676	0.034791	-0.17152	0.1495	TRUE	1.00E+00

33995_at	-0.01101	-1.025676	0.029374	-0.14653	0.12451	TRUE	1.00E+00
1943_at	-0.011	-1.025652	0.086981	-0.41229	0.3903	TRUE	1.00E+00
33283_at	-0.011	-1.025652	0.028004	-0.14019	0.1182	TRUE	1.00E+00
41836_at	-0.01096	-1.025557	0.028921	-0.14439	0.12247	TRUE	1.00E+00
32133_at	-0.01093	-1.025487	0.019628	-0.10149	0.07962	TRUE	1.00E+00
34300_at	-0.01092	-1.025463	0.064318	-0.30766	0.28582	TRUE	1.00E+00
34084_at	-0.01085	-1.025298	0.089361	-0.42312	0.40143	TRUE	1.00E+00
39343_at	-0.01085	-1.025298	0.009214	-0.05335	0.03166	TRUE	1.00E+00
38840_s_at	-0.01083	-1.025251	0.007262	-0.04433	0.02267	TRUE	1.00E+00
36217_at	-0.01077	-1.025109	0.024105	-0.12198	0.10044	TRUE	1.00E+00
35351_at	-0.01076	-1.025085	0.013433	-0.07273	0.05122	TRUE	1.00E+00
38810_at	-0.01076	-1.025085	0.022174	-0.11306	0.09154	TRUE	1.00E+00
39316_at	-0.01075	-1.025062	0.049062	-0.2371	0.2156	TRUE	1.00E+00
41445_at	-0.01071	-1.024967	0.031218	-0.15474	0.13331	TRUE	1.00E+00
32599_at	-0.0107	-1.024944	0.033277	-0.16422	0.14283	TRUE	1.00E+00
36081_s_at	-0.01069	-1.02492	0.025028	-0.12616	0.10478	TRUE	1.00E+00
36044_at	-0.01062	-1.024755	0.013795	-0.07426	0.05303	TRUE	1.00E+00
40424_at	-0.01061	-1.024731	0.030039	-0.1492	0.12798	TRUE	1.00E+00
1004_at	-0.0106	-1.024708	0.084182	-0.39898	0.37778	TRUE	1.00E+00
37029_at	-0.01059	-1.024684	0.020634	-0.10579	0.0846	TRUE	1.00E+00
34387_at	-0.01053	-1.024543	0.028946	-0.14407	0.12301	TRUE	1.00E+00
32415_at	-0.01051	-1.024495	0.062334	-0.29809	0.27708	TRUE	1.00E+00
34369_at	-0.01049	-1.024448	0.013142	-0.07112	0.05015	TRUE	1.00E+00
34058_at	-0.01044	-1.02433	0.029405	-0.14611	0.12522	TRUE	1.00E+00
38029_at	-0.01044	-1.02433	0.017491	-0.09114	0.07026	TRUE	1.00E+00
32075_at	-0.01042	-1.024283	0.04051	-0.19732	0.17647	TRUE	1.00E+00
39682_at	-0.0104	-1.024236	0.056658	-0.2718	0.251	TRUE	1.00E+00
34505_at	-0.01033	-1.024071	0.020844	-0.1065	0.08583	TRUE	1.00E+00
667_at	-0.01032	-1.024047	0.034089	-0.16759	0.14695	TRUE	1.00E+00
35736_at	-0.01032	-1.024047	0.046827	-0.22636	0.20572	TRUE	1.00E+00
40125_at	-0.01029	-1.023977	0.012672	-0.06876	0.04817	TRUE	1.00E+00
38297_at	-0.01029	-1.023977	0.011514	-0.06341	0.04283	TRUE	1.00E+00
31377_r_at	-0.01025	-1.023882	0.048532	-0.23415	0.21366	TRUE	1.00E+00
39482_at	-0.01024	-1.023859	0.122243	-0.57422	0.55374	TRUE	1.00E+00
38792_at	-0.01018	-1.023717	0.018008	-0.09326	0.0729	TRUE	1.00E+00
34429_at	-0.01014	-1.023623	0.024749	-0.12432	0.10404	TRUE	1.00E+00
39488_at	-0.01014	-1.023623	0.080664	-0.38229	0.36201	TRUE	1.00E+00
35818_at	-0.01013	-1.023599	0.026776	-0.13366	0.1134	TRUE	1.00E+00
34856_at	-0.01012	-1.023576	0.049362	-0.23786	0.21762	TRUE	1.00E+00
32032_at	-0.01011	-1.023552	0.046357	-0.22398	0.20376	TRUE	1.00E+00
40767_at	-0.01002	-1.02334	0.069827	-0.33217	0.31214	TRUE	1.00E+00
41199_s_at	-0.01	-1.023293	0.046083	-0.2226	0.20261	TRUE	1.00E+00
38060_at	-0.01	-1.023293	0.023459	-0.11822	0.09823	TRUE	1.00E+00
33946_at	-0.00997	-1.023222	0.099937	-0.47104	0.4511	TRUE	1.00E+00
40934_at	-0.00997	-1.023222	0.049058	-0.2363	0.21636	TRUE	1.00E+00
34502_g_at	-0.00996	-1.023199	0.111023	-0.52217	0.50226	TRUE	1.00E+00
34854_at	-0.00991	-1.023081	0.020624	-0.10507	0.08524	TRUE	1.00E+00
39730_at	-0.00987	-1.022987	0.035119	-0.17189	0.15216	TRUE	1.00E+00
32322_at	-0.00986	-1.022963	0.075628	-0.35878	0.33906	TRUE	1.00E+00
975_at	-0.00985	-1.02294	0.145343	-0.68041	0.6607	TRUE	1.00E+00
33948_at	-0.00983	-1.022893	0.043074	-0.20855	0.1889	TRUE	1.00E+00

268_at	-0.00973	-1.022657	0.086265	-0.40772	0.38826	TRUE	1.00E+00
34357_g_at	-0.00973	-1.022657	0.014185	-0.07518	0.05571	TRUE	1.00E+00
37453_at	-0.0097	-1.022586	0.047869	-0.23054	0.21115	TRUE	1.00E+00
670_s_at	-0.00965	-1.022469	0.1312	-0.61495	0.59565	TRUE	1.00E+00
39996_at	-0.00964	-1.022445	0.02986	-0.1474	0.12812	TRUE	1.00E+00
893_at	-0.00963	-1.022422	0.055021	-0.26347	0.24422	TRUE	1.00E+00
1515_at	-0.00955	-1.022233	0.076569	-0.3628	0.34371	TRUE	1.00E+00
1925_at	-0.00954	-1.02221	0.027672	-0.1372	0.11813	TRUE	1.00E+00
36828_at	-0.00953	-1.022186	0.010837	-0.05953	0.04047	TRUE	1.00E+00
39003_at	-0.00952	-1.022163	0.016478	-0.08554	0.0665	TRUE	1.00E+00
38739_at	-0.00949	-1.022092	0.061579	-0.29359	0.27462	TRUE	1.00E+00
34179_at	-0.00939	-1.021857	0.017947	-0.09219	0.07341	TRUE	1.00E+00
40852_at	-0.00936	-1.021786	0.023087	-0.11587	0.09715	TRUE	1.00E+00
38804_at	-0.00935	-1.021763	0.022476	-0.11305	0.09434	TRUE	1.00E+00
1722_at	-0.00934	-1.021739	0.039091	-0.18969	0.171	TRUE	1.00E+00
41244_f_at	-0.0093	-1.021645	0.026666	-0.13232	0.11373	TRUE	1.00E+00
33527_at	-0.00929	-1.021621	0.138967	-0.65042	0.63185	TRUE	1.00E+00
41735_at	-0.00925	-1.021527	0.03233	-0.15841	0.1399	TRUE	1.00E+00
32964_at	-0.00922	-1.021457	0.053222	-0.25476	0.23633	TRUE	1.00E+00
32354_at	-0.00918	-1.021363	0.08424	-0.39783	0.37946	TRUE	1.00E+00
41198_at	-0.00915	-1.021292	0.022289	-0.11198	0.09368	TRUE	1.00E+00
33321_r_at	-0.00913	-1.021245	0.012691	-0.06768	0.04942	TRUE	1.00E+00
34299_at	-0.00913	-1.021245	0.048595	-0.23333	0.21507	TRUE	1.00E+00
37373_at	-0.00913	-1.021245	0.015562	-0.08093	0.06267	TRUE	1.00E+00
32668_at	-0.00912	-1.021222	0.035089	-0.171	0.15277	TRUE	1.00E+00
38773_at	-0.0091	-1.021175	0.014833	-0.07753	0.05933	TRUE	1.00E+00
41694_at	-0.00906	-1.021081	0.016492	-0.08514	0.06703	TRUE	1.00E+00
33261_at	-0.00905	-1.021057	0.026117	-0.12955	0.11144	TRUE	1.00E+00
40542_at	-0.00904	-1.021034	0.027478	-0.13581	0.11774	TRUE	1.00E+00
1980_s_at	-0.00901	-1.020963	0.014006	-0.07363	0.05561	TRUE	1.00E+00
34802_at	-0.00894	-1.020798	0.014418	-0.07546	0.05758	TRUE	1.00E+00
31987_at	-0.00885	-1.020587	0.063539	-0.30199	0.28429	TRUE	1.00E+00
32278_at	-0.00883	-1.02054	0.056139	-0.26783	0.25018	TRUE	1.00E+00
1094_g_at	-0.0088	-1.020469	0.024926	-0.1238	0.1062	TRUE	1.00E+00
33357_at	-0.00878	-1.020422	0.01875	-0.09529	0.07772	TRUE	1.00E+00
559_s_at	-0.00878	-1.020422	0.064995	-0.30864	0.29108	TRUE	1.00E+00
36242_at	-0.00877	-1.020399	0.032902	-0.16057	0.14303	TRUE	1.00E+00
38109_at	-0.00877	-1.020399	0.034401	-0.16748	0.14995	TRUE	1.00E+00
273_g_at	-0.00875	-1.020352	0.060175	-0.28637	0.26888	TRUE	1.00E+00
32744_at	-0.00874	-1.020328	0.027177	-0.13412	0.11665	TRUE	1.00E+00
37511_at	-0.00868	-1.020188	0.031533	-0.15417	0.1368	TRUE	1.00E+00
41012_r_at	-0.00864	-1.020094	0.127132	-0.59517	0.57789	TRUE	1.00E+00
34900_at	-0.0086	-1.02	0.043535	-0.20946	0.19225	TRUE	1.00E+00
affx-humgapdh/m	-0.0086	-1.02	0.023161	-0.11545	0.09826	TRUE	1.00E+00
41782_g_at	-0.00859	-1.019976	0.020815	-0.10462	0.08744	TRUE	1.00E+00
226_at	-0.00859	-1.019976	0.009847	-0.05402	0.03684	TRUE	1.00E+00
31472_s_at	-0.00859	-1.019976	0.039848	-0.19243	0.17526	TRUE	1.00E+00
34527_r_at	-0.00858	-1.019953	0.066915	-0.3173	0.30014	TRUE	1.00E+00
41479_s_at	-0.00857	-1.019929	0.056845	-0.27082	0.25369	TRUE	1.00E+00
39141_at	-0.00856	-1.019906	0.014879	-0.07721	0.06008	TRUE	1.00E+00
36588_at	-0.00851	-1.019788	0.012867	-0.06788	0.05085	TRUE	1.00E+00

32800_at	-0.00851	-1.019788	0.023846	-0.11852	0.10151	TRUE	1.00E+00
1126_s_at	-0.0085	-1.019765	0.04295	-0.20665	0.18965	TRUE	1.00E+00
36896_s_at	-0.00849	-1.019741	0.044404	-0.21335	0.19638	TRUE	1.00E+00
37350_at	-0.00848	-1.019718	0.017887	-0.09101	0.07404	TRUE	1.00E+00
38796_at	-0.00848	-1.019718	0.100569	-0.47246	0.4555	TRUE	1.00E+00
35537_at	-0.00848	-1.019718	0.013505	-0.07078	0.05383	TRUE	1.00E+00
33445_at	-0.00847	-1.019694	0.077945	-0.36808	0.35113	TRUE	1.00E+00
1857_at	-0.00845	-1.019647	0.045243	-0.21719	0.20028	TRUE	1.00E+00
38809_s_at	-0.00843	-1.0196	0.016794	-0.08591	0.06905	TRUE	1.00E+00
38334_g_at	-0.00841	-1.019553	0.098219	-0.46155	0.44473	TRUE	1.00E+00
221_s_at	-0.00841	-1.019553	0.057009	-0.27143	0.25461	TRUE	1.00E+00
39492_at	-0.00841	-1.019553	0.135427	-0.63321	0.6164	TRUE	1.00E+00
32336_at	-0.0084	-1.01953	0.034226	-0.16631	0.1495	TRUE	1.00E+00
31975_at	-0.00836	-1.019436	0.020525	-0.10305	0.08633	TRUE	1.00E+00
35965_at	-0.00836	-1.019436	0.096582	-0.45395	0.43723	TRUE	1.00E+00
38751_i_at	-0.00835	-1.019413	0.015651	-0.08055	0.06386	TRUE	1.00E+00
38860_at	-0.00834	-1.019389	0.034002	-0.16521	0.14854	TRUE	1.00E+00
38065_at	-0.00827	-1.019225	0.065509	-0.3105	0.29396	TRUE	1.00E+00
1754_at	-0.00825	-1.019178	0.013071	-0.06856	0.05205	TRUE	1.00E+00
35050_at	-0.00823	-1.019131	0.026767	-0.13172	0.11526	TRUE	1.00E+00
32431_at	-0.00821	-1.019084	0.0556	-0.26473	0.24831	TRUE	1.00E+00
34766_r_at	-0.00819	-1.019037	0.014751	-0.07625	0.05986	TRUE	1.00E+00
39462_s_at	-0.00819	-1.019037	0.086043	-0.40516	0.38877	TRUE	1.00E+00
31789_at	-0.00818	-1.019014	0.023565	-0.11691	0.10054	TRUE	1.00E+00
41309_g_at	-0.00815	-1.018943	0.040931	-0.19699	0.18069	TRUE	1.00E+00
39832_at	-0.00813	-1.018896	0.020695	-0.10361	0.08734	TRUE	1.00E+00
39872_at	-0.00812	-1.018873	0.037532	-0.18127	0.16504	TRUE	1.00E+00
34176_at	-0.00808	-1.018779	0.066156	-0.31329	0.29714	TRUE	1.00E+00
34488_i_at	-0.00806	-1.018732	0.03355	-0.16284	0.14673	TRUE	1.00E+00
1426_at	-0.00797	-1.018521	0.038959	-0.18771	0.17177	TRUE	1.00E+00
811_at	-0.00783	-1.018193	0.016338	-0.0832	0.06755	TRUE	1.00E+00
40106_at	-0.00782	-1.018169	0.021154	-0.10542	0.08977	TRUE	1.00E+00
732_f_at	-0.00782	-1.018169	0.028428	-0.13898	0.12334	TRUE	1.00E+00
37345_at	-0.00781	-1.018146	0.006683	-0.03864	0.02302	TRUE	1.00E+00
41367_at	-0.00777	-1.018052	0.143654	-0.67053	0.65498	TRUE	1.00E+00
32091_at	-0.00775	-1.018005	0.038842	-0.18696	0.17145	TRUE	1.00E+00
36100_at	-0.00774	-1.017982	0.043857	-0.21008	0.1946	TRUE	1.00E+00
38276_at	-0.00773	-1.017958	0.043571	-0.20875	0.19329	TRUE	1.00E+00
40545_at	-0.00767	-1.017818	0.029045	-0.14167	0.12633	TRUE	1.00E+00
32580_at	-0.00767	-1.017818	0.057734	-0.27403	0.25869	TRUE	1.00E+00
1655_s_at	-0.00764	-1.017747	0.048043	-0.22929	0.21401	TRUE	1.00E+00
40599_at	-0.00762	-1.017701	0.03109	-0.15105	0.13582	TRUE	1.00E+00
36495_at	-0.00761	-1.017677	0.036188	-0.17456	0.15935	TRUE	1.00E+00
41276_at	-0.00755	-1.017537	0.030279	-0.14724	0.13215	TRUE	1.00E+00
31736_at	-0.00755	-1.017537	0.027176	-0.13293	0.11783	TRUE	1.00E+00
39724_s_at	-0.00755	-1.017537	0.027388	-0.1339	0.11881	TRUE	1.00E+00
33388_at	-0.00751	-1.017443	0.014271	-0.07335	0.05833	TRUE	1.00E+00
32425_at	-0.0075	-1.017419	0.017783	-0.08955	0.07454	TRUE	1.00E+00
31780_f_at	-0.00748	-1.017373	0.03588	-0.17301	0.15805	TRUE	1.00E+00
280_g_at	-0.00744	-1.017279	0.059214	-0.28062	0.26575	TRUE	1.00E+00
32066_g_at	-0.00743	-1.017255	0.030611	-0.14866	0.13379	TRUE	1.00E+00

33215_g_at	-0.00741	-1.017209	0.023023	-0.11363	0.09881	TRUE	1.00E+00
38373_g_at	-0.00741	-1.017209	0.031149	-0.15112	0.1363	TRUE	1.00E+00
718_at	-0.00735	-1.017068	0.020521	-0.10203	0.08733	TRUE	1.00E+00
35323_at	-0.00733	-1.017021	0.02629	-0.12862	0.11397	TRUE	1.00E+00
40537_at	-0.00729	-1.016928	0.009816	-0.05258	0.03799	TRUE	1.00E+00
1309_at	-0.00727	-1.016881	0.020387	-0.10133	0.08678	TRUE	1.00E+00
1162_g_at	-0.00727	-1.016881	0.025024	-0.12272	0.10818	TRUE	1.00E+00
37965_at	-0.00723	-1.016787	0.019503	-0.09721	0.08274	TRUE	1.00E+00
40257_at	-0.00722	-1.016764	0.091102	-0.42753	0.41309	TRUE	1.00E+00
35093_at	-0.0072	-1.016717	0.048398	-0.23049	0.21609	TRUE	1.00E+00
31497_at	-0.00719	-1.016693	0.023237	-0.11439	0.10002	TRUE	1.00E+00
35412_at	-0.00716	-1.016623	0.078589	-0.36974	0.35541	TRUE	1.00E+00
160020_at	-0.00714	-1.016576	0.042273	-0.20217	0.18789	TRUE	1.00E+00
37719_at	-0.00714	-1.016576	0.01933	-0.09632	0.08204	TRUE	1.00E+00
33110_at	-0.00709	-1.016459	0.022936	-0.11291	0.09872	TRUE	1.00E+00
34379_at	-0.00708	-1.016436	0.048077	-0.22889	0.21473	TRUE	1.00E+00
36900_at	-0.00705	-1.016366	0.015898	-0.0804	0.0663	TRUE	1.00E+00
40569_at	-0.00704	-1.016342	0.031146	-0.15073	0.13666	TRUE	1.00E+00
39834_at	-0.00703	-1.016319	0.084195	-0.39547	0.3814	TRUE	1.00E+00
39717_g_at	-0.00696	-1.016155	0.013744	-0.07037	0.05645	TRUE	1.00E+00
39328_at	-0.00693	-1.016085	0.040611	-0.1943	0.18043	TRUE	1.00E+00
38930_at	-0.0069	-1.016015	0.041769	-0.1996	0.18581	TRUE	1.00E+00
35841_at	-0.0069	-1.016015	0.027912	-0.13567	0.12188	TRUE	1.00E+00
34985_at	-0.00689	-1.015991	0.058925	-0.27874	0.26496	TRUE	1.00E+00
41203_at	-0.00688	-1.015968	0.027262	-0.13266	0.1189	TRUE	1.00E+00
34586_s_at	-0.00683	-1.015851	0.047179	-0.22449	0.21084	TRUE	1.00E+00
32735_at	-0.00682	-1.015828	0.037209	-0.17849	0.16485	TRUE	1.00E+00
1912_s_at	-0.0068	-1.015781	0.01014	-0.05358	0.03998	TRUE	1.00E+00
40462_at	-0.00675	-1.015664	0.028739	-0.13934	0.12584	TRUE	1.00E+00
36839_at	-0.0067	-1.015547	0.057041	-0.26986	0.25647	TRUE	1.00E+00
262_at	-0.00667	-1.015477	0.040485	-0.19345	0.18012	TRUE	1.00E+00
34840_at	-0.00663	-1.015383	0.020434	-0.10091	0.08764	TRUE	1.00E+00
39270_at	-0.00652	-1.015126	0.097588	-0.45676	0.44371	TRUE	1.00E+00
35850_at	-0.00652	-1.015126	0.025641	-0.12482	0.11177	TRUE	1.00E+00
39475_at	-0.00641	-1.014869	0.03952	-0.18874	0.17592	TRUE	1.00E+00
39153_r_at	-0.00641	-1.014869	0.027252	-0.13214	0.11932	TRUE	1.00E+00
33665_s_at	-0.00641	-1.014869	0.099897	-0.46729	0.45448	TRUE	1.00E+00
31944_at	-0.0064	-1.014846	0.024696	-0.12034	0.10754	TRUE	1.00E+00
31380_at	-0.0064	-1.014846	0.085892	-0.40267	0.38988	TRUE	1.00E+00
1695_at	-0.00633	-1.014682	0.0144	-0.07277	0.0601	TRUE	1.00E+00
41095_at	-0.00632	-1.014659	0.015066	-0.07583	0.06319	TRUE	1.00E+00
36954_at	-0.00629	-1.014589	0.022958	-0.11221	0.09962	TRUE	1.00E+00
40823_s_at	-0.00628	-1.014565	0.037994	-0.18157	0.16901	TRUE	1.00E+00
41697_at	-0.00626	-1.014519	0.038624	-0.18445	0.17194	TRUE	1.00E+00
41437_at	-0.00625	-1.014495	0.036322	-0.17383	0.16133	TRUE	1.00E+00
36574_at	-0.00624	-1.014472	0.011887	-0.06108	0.04861	TRUE	1.00E+00
36150_at	-0.00623	-1.014448	0.031603	-0.15203	0.13958	TRUE	1.00E+00
1765_at	-0.00622	-1.014425	0.071083	-0.33417	0.32173	TRUE	1.00E+00
1602_at	-0.00622	-1.014425	0.024402	-0.1188	0.10636	TRUE	1.00E+00
568_at	-0.00622	-1.014425	0.051525	-0.24393	0.2315	TRUE	1.00E+00
37903_at	-0.00621	-1.014402	0.02409	-0.11735	0.10493	TRUE	1.00E+00

33114_f_at	-0.00618	-1.014332	0.145992	-0.67973	0.66736	TRUE	1.00E+00
35803_at	-0.00617	-1.014308	0.021013	-0.10312	0.09077	TRUE	1.00E+00
37231_at	-0.00617	-1.014308	0.087589	-0.41027	0.39793	TRUE	1.00E+00
33917_at	-0.00614	-1.014238	0.017898	-0.08872	0.07643	TRUE	1.00E+00
40627_at	-0.00608	-1.014098	0.049048	-0.23237	0.22021	TRUE	1.00E+00
40982_at	-0.00606	-1.014051	0.04761	-0.22571	0.21359	TRUE	1.00E+00
34702_f_at	-0.00605	-1.014028	0.025507	-0.12373	0.11163	TRUE	1.00E+00
31860_at	-0.00604	-1.014005	0.021061	-0.1032	0.09113	TRUE	1.00E+00
34922_at	-0.006	-1.013911	0.119575	-0.55767	0.54567	TRUE	1.00E+00
38027_at	-0.00596	-1.013818	0.018512	-0.09137	0.07944	TRUE	1.00E+00
38193_at	-0.00595	-1.013795	0.032561	-0.15617	0.14427	TRUE	1.00E+00
33762_r_at	-0.00589	-1.013655	0.026672	-0.12894	0.11717	TRUE	1.00E+00
40875_s_at	-0.00584	-1.013538	0.027126	-0.13099	0.11931	TRUE	1.00E+00
37216_at	-0.00581	-1.013468	0.0224	-0.10916	0.09753	TRUE	1.00E+00
37379_at	-0.00576	-1.013351	0.022183	-0.1081	0.09658	TRUE	1.00E+00
1391_s_at	-0.00572	-1.013258	0.022368	-0.10892	0.09748	TRUE	1.00E+00
39517_at	-0.00572	-1.013258	0.011021	-0.05656	0.04513	TRUE	1.00E+00
39863_at	-0.00571	-1.013235	0.029974	-0.144	0.13258	TRUE	1.00E+00
41551_at	-0.0057	-1.013211	0.023509	-0.11416	0.10276	TRUE	1.00E+00
34695_at	-0.00569	-1.013188	0.017658	-0.08716	0.07578	TRUE	1.00E+00
40184_at	-0.00563	-1.013048	0.017845	-0.08797	0.0767	TRUE	1.00E+00
1204_at	-0.00563	-1.013048	0.052838	-0.24941	0.23814	TRUE	1.00E+00
41394_at	-0.0056	-1.012978	0.015885	-0.07889	0.06769	TRUE	1.00E+00
39251_at	-0.00559	-1.012955	0.019419	-0.09518	0.084	TRUE	1.00E+00
34559_at	-0.00559	-1.012955	0.051175	-0.24169	0.23051	TRUE	1.00E+00
36735_f_at	-0.00557	-1.012908	0.052186	-0.24634	0.23519	TRUE	1.00E+00
38517_at	-0.00556	-1.012885	0.039719	-0.1888	0.17769	TRUE	1.00E+00
34101_at	-0.00549	-1.012721	0.036549	-0.17411	0.16313	TRUE	1.00E+00
31749_f_at	-0.0054	-1.012512	0.119847	-0.55832	0.54753	TRUE	1.00E+00
34264_at	-0.00539	-1.012488	0.022021	-0.10698	0.09621	TRUE	1.00E+00
36027_at	-0.00538	-1.012465	0.015129	-0.07517	0.06442	TRUE	1.00E+00
36093_at	-0.00535	-1.012395	0.037763	-0.17958	0.16887	TRUE	1.00E+00
34774_at	-0.00533	-1.012348	0.021641	-0.10517	0.09452	TRUE	1.00E+00
affx-m27830_5_a	-0.0053	-1.012278	0.036673	-0.1745	0.16389	TRUE	1.00E+00
36915_at	-0.00528	-1.012232	0.037784	-0.1796	0.16904	TRUE	1.00E+00
36831_at	-0.00528	-1.012232	0.041375	-0.19617	0.18561	TRUE	1.00E+00
36602_at	-0.00527	-1.012209	0.018038	-0.08849	0.07795	TRUE	1.00E+00
38292_at	-0.00518	-1.011999	0.121928	-0.56771	0.55734	TRUE	1.00E+00
1917_at	-0.00517	-1.011976	0.018468	-0.09038	0.08003	TRUE	1.00E+00
35501_at	-0.00517	-1.011976	0.102629	-0.47865	0.46832	TRUE	1.00E+00
259_s_at	-0.00516	-1.011952	0.029115	-0.13948	0.12917	TRUE	1.00E+00
37086_at	-0.00515	-1.011929	0.047552	-0.22454	0.21423	TRUE	1.00E+00
34322_r_at	-0.00514	-1.011906	0.028928	-0.1386	0.12832	TRUE	1.00E+00
32615_at	-0.00513	-1.011882	0.015675	-0.07745	0.06719	TRUE	1.00E+00
40057_at	-0.00511	-1.011836	0.029107	-0.13939	0.12918	TRUE	1.00E+00
207_at	-0.0051	-1.011812	0.010961	-0.05567	0.04547	TRUE	1.00E+00
39004_at	-0.00509	-1.011789	0.022327	-0.1081	0.09792	TRUE	1.00E+00
40487_at	-0.00508	-1.011766	0.01618	-0.07973	0.06957	TRUE	1.00E+00
35913_at	-0.00506	-1.011719	0.057382	-0.2698	0.25968	TRUE	1.00E+00
35555_r_at	-0.00501	-1.011603	0.053026	-0.24965	0.23963	TRUE	1.00E+00
38043_at	-0.00501	-1.011603	0.048942	-0.23081	0.22079	TRUE	1.00E+00

34351_at	-0.00497	-1.01151	0.014663	-0.07262	0.06268	TRUE	1.00E+00
39851_at	-0.00496	-1.011486	0.131779	-0.61293	0.60301	TRUE	1.00E+00
32296_at	-0.00491	-1.01137	0.051298	-0.24157	0.23176	TRUE	1.00E+00
36167_at	-0.00488	-1.0113	0.013965	-0.06931	0.05954	TRUE	1.00E+00
41621_i_at	-0.00487	-1.011277	0.021336	-0.10331	0.09357	TRUE	1.00E+00
365_at	-0.00487	-1.011277	0.051797	-0.24384	0.2341	TRUE	1.00E+00
463_g_at	-0.00486	-1.011253	0.025313	-0.12165	0.11192	TRUE	1.00E+00
36161_at	-0.00484	-1.011207	0.057567	-0.27043	0.26075	TRUE	1.00E+00
31342_at	-0.00484	-1.011207	0.023958	-0.11537	0.1057	TRUE	1.00E+00
39042_at	-0.00482	-1.01116	0.037789	-0.17917	0.16952	TRUE	1.00E+00
40037_at	-0.00479	-1.01109	0.028223	-0.135	0.12542	TRUE	1.00E+00
1392_at	-0.00477	-1.011044	0.026247	-0.12586	0.11632	TRUE	1.00E+00
41674_at	-0.00469	-1.010858	0.089409	-0.41719	0.40781	TRUE	1.00E+00
35932_at	-0.00464	-1.010741	0.064858	-0.30387	0.29458	TRUE	1.00E+00
34874_at	-0.00463	-1.010718	0.016508	-0.08079	0.07153	TRUE	1.00E+00
38945_at	-0.00462	-1.010695	0.016278	-0.07972	0.07048	TRUE	1.00E+00
36406_at	-0.00462	-1.010695	0.047349	-0.22306	0.21383	TRUE	1.00E+00
39859_r_at	-0.00459	-1.010625	0.046266	-0.21804	0.20887	TRUE	1.00E+00
34119_at	-0.00457	-1.010578	0.073516	-0.34374	0.3346	TRUE	1.00E+00
1767_s_at	-0.00453	-1.010485	0.051842	-0.2437	0.23465	TRUE	1.00E+00
35873_at	-0.0045	-1.010416	0.052395	-0.24623	0.23723	TRUE	1.00E+00
37585_at	-0.00449	-1.010392	0.027788	-0.13269	0.12371	TRUE	1.00E+00
38592_s_at	-0.00447	-1.010346	0.015604	-0.07645	0.06752	TRUE	1.00E+00
34499_at	-0.00446	-1.010322	0.052567	-0.24699	0.23806	TRUE	1.00E+00
1243_at	-0.00445	-1.010299	0.03277	-0.15564	0.14673	TRUE	1.00E+00
38058_at	-0.00445	-1.010299	0.057241	-0.26854	0.25964	TRUE	1.00E+00
32146_s_at	-0.00441	-1.010206	0.032058	-0.15231	0.14349	TRUE	1.00E+00
834_at	-0.00435	-1.010067	0.05685	-0.26663	0.25793	TRUE	1.00E+00
35722_at	-0.00435	-1.010067	0.041656	-0.19653	0.18783	TRUE	1.00E+00
34516_at	-0.00431	-1.009974	0.150244	-0.69747	0.68885	TRUE	1.00E+00
34033_s_at	-0.0043	-1.00995	0.103095	-0.47994	0.47133	TRUE	1.00E+00
39900_at	-0.00427	-1.009881	0.0536	-0.25156	0.24301	TRUE	1.00E+00
39714_at	-0.00426	-1.009857	0.028879	-0.1375	0.12897	TRUE	1.00E+00
31807_at	-0.00422	-1.009764	0.019791	-0.09553	0.08708	TRUE	1.00E+00
40777_at	-0.00421	-1.009741	0.008065	-0.04142	0.033	TRUE	1.00E+00
39372_at	-0.00419	-1.009695	0.051045	-0.23969	0.23131	TRUE	1.00E+00
41214_at	-0.00417	-1.009648	0.349685	-1.61747	1.60913	TRUE	1.00E+00
37924_g_at	-0.00412	-1.009532	0.026584	-0.12677	0.11853	TRUE	1.00E+00
1819_at	-0.00411	-1.009509	0.092594	-0.4313	0.42308	TRUE	1.00E+00
41775_at	-0.00411	-1.009509	0.018264	-0.08837	0.08016	TRUE	1.00E+00
32284_at	-0.00408	-1.009439	0.035082	-0.16593	0.15778	TRUE	1.00E+00
41046_s_at	-0.00408	-1.009439	0.034067	-0.16125	0.1531	TRUE	1.00E+00
38566_at	-0.00406	-1.009392	0.077568	-0.36193	0.3538	TRUE	1.00E+00
112_g_at	-0.00406	-1.009392	0.031589	-0.14979	0.14168	TRUE	1.00E+00
34558_at	-0.00404	-1.009346	0.051162	-0.24008	0.232	TRUE	1.00E+00
38828_s_at	-0.004	-1.009253	0.026137	-0.12458	0.11659	TRUE	1.00E+00
38722_at	-0.00399	-1.00923	0.029932	-0.14209	0.1341	TRUE	1.00E+00
34706_at	-0.00398	-1.009206	0.009707	-0.04876	0.0408	TRUE	1.00E+00
35201_at	-0.00398	-1.009206	0.022909	-0.10967	0.10172	TRUE	1.00E+00
38121_at	-0.00397	-1.009183	0.035521	-0.16785	0.15991	TRUE	1.00E+00
1552_i_at	-0.00395	-1.009137	0.054152	-0.25378	0.24589	TRUE	1.00E+00

39134_at	-0.00393	-1.00909	0.014224	-0.06955	0.0617	TRUE	1.00E+00
39018_at	-0.00393	-1.00909	0.027668	-0.13157	0.12372	TRUE	1.00E+00
34090_at	-0.00391	-1.009044	0.112683	-0.52378	0.51596	TRUE	1.00E+00
37783_at	-0.0039	-1.009021	0.034854	-0.1647	0.1569	TRUE	1.00E+00
38142_at	-0.00388	-1.008974	0.038402	-0.18105	0.17329	TRUE	1.00E+00
39918_at	-0.00382	-1.008835	0.010319	-0.05143	0.04379	TRUE	1.00E+00
37968_at	-0.00378	-1.008742	0.084209	-0.39228	0.38473	TRUE	1.00E+00
41774_at	-0.00374	-1.008649	0.154594	-0.71698	0.70949	TRUE	1.00E+00
40098_at	-0.00372	-1.008602	0.023919	-0.11407	0.10663	TRUE	1.00E+00
39915_at	-0.00371	-1.008579	0.072251	-0.33704	0.32963	TRUE	1.00E+00
31605_at	-0.0037	-1.008556	0.022488	-0.10745	0.10005	TRUE	1.00E+00
977_s_at	-0.00367	-1.008486	0.065209	-0.30452	0.29718	TRUE	1.00E+00
37474_at	-0.00367	-1.008486	0.071398	-0.33307	0.32573	TRUE	1.00E+00
33238_at	-0.00366	-1.008463	0.086157	-0.40115	0.39383	TRUE	1.00E+00
35294_at	-0.00363	-1.008393	0.013933	-0.06791	0.06065	TRUE	1.00E+00
41701_at	-0.00361	-1.008347	0.045315	-0.21268	0.20545	TRUE	1.00E+00
199_s_at	-0.00357	-1.008254	0.03313	-0.15642	0.14928	TRUE	1.00E+00
38398_at	-0.00357	-1.008254	0.03249	-0.15346	0.14632	TRUE	1.00E+00
38691_s_at	-0.00355	-1.008208	0.101855	-0.47347	0.46636	TRUE	1.00E+00
38720_at	-0.00352	-1.008138	0.023218	-0.11064	0.10359	TRUE	1.00E+00
37133_at	-0.00349	-1.008068	0.077596	-0.36149	0.3545	TRUE	1.00E+00
35507_at	-0.00346	-1.007999	0.038553	-0.18133	0.17441	TRUE	1.00E+00
33300_at	-0.00345	-1.007976	0.036191	-0.17042	0.16352	TRUE	1.00E+00
40122_at	-0.00344	-1.007952	0.009796	-0.04863	0.04176	TRUE	1.00E+00
37720_at	-0.00338	-1.007813	0.017929	-0.0861	0.07934	TRUE	1.00E+00
31748_at	-0.00335	-1.007743	0.021767	-0.10377	0.09707	TRUE	1.00E+00
33207_at	-0.00329	-1.007604	0.022053	-0.10503	0.09845	TRUE	1.00E+00
37384_at	-0.00329	-1.007604	0.021861	-0.10415	0.09757	TRUE	1.00E+00
37060_at	-0.00329	-1.007604	0.072272	-0.33672	0.33014	TRUE	1.00E+00
35186_at	-0.00326	-1.007535	0.029146	-0.13773	0.13121	TRUE	1.00E+00
39520_at	-0.00326	-1.007535	0.03007	-0.14199	0.13547	TRUE	1.00E+00
944_s_at	-0.00325	-1.007511	0.028199	-0.13335	0.12684	TRUE	1.00E+00
37766_s_at	-0.00323	-1.007465	0.0073	-0.03691	0.03045	TRUE	1.00E+00
39073_at	-0.00315	-1.00728	0.026067	-0.12341	0.11711	TRUE	1.00E+00
39699_at	-0.00314	-1.007256	0.025231	-0.11954	0.11327	TRUE	1.00E+00
40151_s_at	-0.00313	-1.007233	0.030969	-0.14601	0.13975	TRUE	1.00E+00
39322_at	-0.00308	-1.007117	0.082582	-0.38408	0.37792	TRUE	1.00E+00
38959_s_at	-0.00307	-1.007094	0.034915	-0.16415	0.15801	TRUE	1.00E+00
32069_at	-0.00305	-1.007048	0.023203	-0.1101	0.104	TRUE	1.00E+00
31614_at	-0.00298	-1.006885	0.02607	-0.12325	0.1173	TRUE	1.00E+00
32915_at	-0.00297	-1.006862	0.046079	-0.21557	0.20962	TRUE	1.00E+00
33670_at	-0.00296	-1.006839	0.021483	-0.10207	0.09615	TRUE	1.00E+00
38412_at	-0.0029	-1.0067	0.019516	-0.09294	0.08713	TRUE	1.00E+00
40631_at	-0.00287	-1.00663	0.026283	-0.12413	0.11839	TRUE	1.00E+00
37003_at	-0.00276	-1.006375	0.023268	-0.11011	0.10459	TRUE	1.00E+00
35880_at	-0.00275	-1.006352	0.066345	-0.30884	0.30333	TRUE	1.00E+00
34356_at	-0.00274	-1.006329	0.022135	-0.10485	0.09938	TRUE	1.00E+00
35254_at	-0.00271	-1.00626	0.029968	-0.14097	0.13555	TRUE	1.00E+00
1374_g_at	-0.00271	-1.00626	0.022194	-0.1051	0.09969	TRUE	1.00E+00
32414_at	-0.00265	-1.006121	0.113593	-0.52672	0.52143	TRUE	1.00E+00
32644_at	-0.00264	-1.006097	0.025177	-0.11879	0.11352	TRUE	1.00E+00

34350_at	-0.0026	-1.006005	0.015162	-0.07256	0.06735	TRUE	1.00E+00
35553_at	-0.0026	-1.006005	0.061817	-0.2878	0.28259	TRUE	1.00E+00
38548_at	-0.0026	-1.006005	0.085927	-0.39904	0.39383	TRUE	1.00E+00
534_s_at	-0.0026	-1.006005	0.022988	-0.10865	0.10346	TRUE	1.00E+00
38137_at	-0.00259	-1.005982	0.078971	-0.36693	0.36175	TRUE	1.00E+00
41633_at	-0.00259	-1.005982	0.02121	-0.10044	0.09527	TRUE	1.00E+00
40779_at	-0.00258	-1.005958	0.023656	-0.11172	0.10655	TRUE	1.00E+00
34150_at	-0.00255	-1.005889	0.084986	-0.39464	0.38954	TRUE	1.00E+00
34291_at	-0.00252	-1.005819	0.007202	-0.03574	0.03071	TRUE	1.00E+00
40214_at	-0.00251	-1.005796	0.164621	-0.76201	0.75698	TRUE	1.00E+00
1386_at	-0.00249	-1.00575	0.017396	-0.08274	0.07777	TRUE	1.00E+00
31567_at	-0.00245	-1.005657	0.020963	-0.09917	0.09426	TRUE	1.00E+00
40142_at	-0.00245	-1.005657	0.022663	-0.10701	0.10211	TRUE	1.00E+00
39189_at	-0.00241	-1.005565	0.069308	-0.32217	0.31734	TRUE	1.00E+00
41458_at	-0.0024	-1.005542	0.025288	-0.11907	0.11427	TRUE	1.00E+00
1310_at	-0.00239	-1.005518	0.016801	-0.0799	0.07512	TRUE	1.00E+00
39075_at	-0.00235	-1.005426	0.040794	-0.19056	0.18586	TRUE	1.00E+00
36108_at	-0.0023	-1.00531	0.016726	-0.07946	0.07487	TRUE	1.00E+00
36677_at	-0.00228	-1.005264	0.018902	-0.08949	0.08492	TRUE	1.00E+00
35779_at	-0.00226	-1.005217	0.021638	-0.10209	0.09757	TRUE	1.00E+00
31398_at	-0.00225	-1.005194	0.054851	-0.25531	0.2508	TRUE	1.00E+00
41838_at	-0.00223	-1.005148	0.009265	-0.04498	0.04052	TRUE	1.00E+00
38530_at	-0.00223	-1.005148	0.041546	-0.1939	0.18945	TRUE	1.00E+00
35760_at	-0.00221	-1.005102	0.014035	-0.06696	0.06254	TRUE	1.00E+00
33633_at	-0.0022	-1.005079	0.063179	-0.29368	0.28928	TRUE	1.00E+00
580_at	-0.00213	-1.004917	0.037681	-0.17598	0.17171	TRUE	1.00E+00
33966_at	-0.00212	-1.004893	0.077722	-0.36069	0.35646	TRUE	1.00E+00
38646_s_at	-0.00206	-1.004755	0.067126	-0.31175	0.30763	TRUE	1.00E+00
33729_at	-0.00204	-1.004708	0.039128	-0.18256	0.17849	TRUE	1.00E+00
33922_at	-0.00202	-1.004662	0.027568	-0.12921	0.12517	TRUE	1.00E+00
39983_at	-0.00199	-1.004593	0.04569	-0.21279	0.2088	TRUE	1.00E+00
39839_at	-0.00197	-1.004546	0.022828	-0.10729	0.10335	TRUE	1.00E+00
1719_at	-0.00193	-1.004454	0.03181	-0.14869	0.14483	TRUE	1.00E+00
34733_at	-0.00188	-1.004338	0.024566	-0.11522	0.11145	TRUE	1.00E+00
34427_g_at	-0.00188	-1.004338	0.021723	-0.1021	0.09834	TRUE	1.00E+00
32323_at	-0.00188	-1.004338	0.037683	-0.17573	0.17198	TRUE	1.00E+00
38606_at	-0.00186	-1.004292	0.095761	-0.44367	0.43994	TRUE	1.00E+00
35797_at	-0.00184	-1.004246	0.014156	-0.06715	0.06346	TRUE	1.00E+00
33037_at	-0.00183	-1.004223	0.015306	-0.07244	0.06879	TRUE	1.00E+00
34714_at	-0.00183	-1.004223	0.05064	-0.23546	0.23181	TRUE	1.00E+00
41690_at	-0.00182	-1.004199	0.021591	-0.10143	0.09779	TRUE	1.00E+00
41301_at	-0.00179	-1.00413	0.024991	-0.11709	0.11351	TRUE	1.00E+00
39144_at	-0.00174	-1.004015	0.102951	-0.47672	0.47323	TRUE	1.00E+00
34466_at	-0.00174	-1.004015	0.029196	-0.13644	0.13296	TRUE	1.00E+00
32443_at	-0.00173	-1.003991	0.016087	-0.07595	0.07249	TRUE	1.00E+00
352_at	-0.0017	-1.003922	0.033286	-0.15527	0.15187	TRUE	1.00E+00
35155_at	-0.0017	-1.003922	0.019059	-0.08963	0.08624	TRUE	1.00E+00
33512_at	-0.00169	-1.003899	0.066414	-0.30809	0.30472	TRUE	1.00E+00
38421_at	-0.00166	-1.00383	0.018081	-0.08508	0.08175	TRUE	1.00E+00
38456_s_at	-0.00166	-1.00383	0.02367	-0.11086	0.10754	TRUE	1.00E+00
35405_at	-0.00163	-1.00376	0.152661	-0.70594	0.70269	TRUE	1.00E+00

35808_at	-0.00161	-1.003714	0.023543	-0.11023	0.107	TRUE	1.00E+00
36146_at	-0.00161	-1.003714	0.030496	-0.1423	0.13909	TRUE	1.00E+00
40548_at	-0.00156	-1.003598	0.04944	-0.22965	0.22654	TRUE	1.00E+00
177_at	-0.00152	-1.003506	0.096753	-0.4479	0.44486	TRUE	1.00E+00
affx-humtfrr/m11!	-0.00152	-1.003506	0.019355	-0.09082	0.08778	TRUE	1.00E+00
1975_s_at	-0.0015	-1.00346	0.033281	-0.15505	0.15205	TRUE	1.00E+00
33927_i_at	-0.00148	-1.003414	0.036872	-0.1716	0.16863	TRUE	1.00E+00
38369_at	-0.00147	-1.003391	0.027821	-0.12983	0.12688	TRUE	1.00E+00
38628_at	-0.00146	-1.003367	0.017884	-0.08397	0.08105	TRUE	1.00E+00
31612_at	-0.00146	-1.003367	0.048635	-0.22584	0.22293	TRUE	1.00E+00
33876_at	-0.00144	-1.003321	0.034981	-0.16282	0.15995	TRUE	1.00E+00
39138_g_at	-0.00137	-1.00316	0.043165	-0.20051	0.19778	TRUE	1.00E+00
36599_at	-0.00136	-1.003136	0.031102	-0.14485	0.14214	TRUE	1.00E+00
1477_s_at	-0.00132	-1.003044	0.088145	-0.40798	0.40534	TRUE	1.00E+00
33291_at	-0.00129	-1.002975	0.088503	-0.40961	0.40702	TRUE	1.00E+00
34883_at	-0.00129	-1.002975	0.024574	-0.11466	0.11209	TRUE	1.00E+00
564_at	-0.00126	-1.002905	0.022264	-0.10397	0.10146	TRUE	1.00E+00
41432_at	-0.00123	-1.002836	0.077809	-0.3602	0.35775	TRUE	1.00E+00
37174_at	-0.00123	-1.002836	0.025214	-0.11755	0.1151	TRUE	1.00E+00
37372_at	-0.00117	-1.002698	0.022636	-0.1056	0.10326	TRUE	1.00E+00
41850_s_at	-0.00117	-1.002698	0.018833	-0.08805	0.08572	TRUE	1.00E+00
32047_at	-0.00112	-1.002582	0.013598	-0.06385	0.06162	TRUE	1.00E+00
39348_at	-0.00108	-1.00249	0.019908	-0.09293	0.09077	TRUE	1.00E+00
36604_at	-0.00106	-1.002444	0.021622	-0.10082	0.0987	TRUE	1.00E+00
32430_at	-0.00105	-1.002421	0.048361	-0.22417	0.22206	TRUE	1.00E+00
33228_g_at	-0.00105	-1.002421	0.024987	-0.11633	0.11423	TRUE	1.00E+00
38226_at	-0.00101	-1.002328	0.021351	-0.09952	0.09749	TRUE	1.00E+00
33857_at	-0.00101	-1.002328	0.023491	-0.10939	0.10737	TRUE	1.00E+00
34367_at	-0.00101	-1.002328	0.023337	-0.10867	0.10666	TRUE	1.00E+00
33157_at	-0.00099	-1.002282	0.121543	-0.56174	0.55976	TRUE	1.00E+00
122_at	-0.0009	-1.002074	0.026315	-0.12231	0.1205	TRUE	1.00E+00
1362_s_at	-0.00089	-1.002051	0.029676	-0.1378	0.13602	TRUE	1.00E+00
475_at	-0.00089	-1.002051	0.027905	-0.12963	0.12785	TRUE	1.00E+00
2036_s_at	-0.00088	-1.002028	0.03853	-0.17864	0.17688	TRUE	1.00E+00
532_at	-0.00088	-1.002028	0.07075	-0.32729	0.32553	TRUE	1.00E+00
780_at	-0.00087	-1.002005	0.046134	-0.21372	0.21197	TRUE	1.00E+00
32943_at	-0.00086	-1.001982	0.039619	-0.18365	0.18192	TRUE	1.00E+00
38328_at	-0.00085	-1.001959	0.051281	-0.23744	0.23574	TRUE	1.00E+00
1167_s_at	-0.00085	-1.001959	0.046456	-0.21518	0.21348	TRUE	1.00E+00
33071_at	-0.00085	-1.001959	0.027347	-0.12701	0.12532	TRUE	1.00E+00
40298_at	-0.00082	-1.00189	0.081638	-0.37747	0.37582	TRUE	1.00E+00
35345_at	-0.00082	-1.00189	0.034583	-0.16037	0.15873	TRUE	1.00E+00
36792_at	-0.00074	-1.001705	0.023188	-0.10772	0.10624	TRUE	1.00E+00
38093_at	-0.00073	-1.001682	0.015856	-0.07388	0.07243	TRUE	1.00E+00
1031_at	-0.00072	-1.001659	0.030038	-0.1393	0.13786	TRUE	1.00E+00
33798_at	-0.00071	-1.001636	0.029631	-0.13742	0.136	TRUE	1.00E+00
32738_at	-0.00071	-1.001636	0.024625	-0.11432	0.1129	TRUE	1.00E+00
825_at	-0.00069	-1.00159	0.039405	-0.18248	0.18111	TRUE	1.00E+00
40070_at	-0.00064	-1.001475	0.022891	-0.10625	0.10497	TRUE	1.00E+00
1585_at	-0.00063	-1.001452	0.068856	-0.31831	0.31704	TRUE	1.00E+00
35080_at	-0.00063	-1.001452	0.0115	-0.05369	0.05242	TRUE	1.00E+00

36194_at	-0.00061	-1.001406	0.031156	-0.14435	0.14313	TRUE	1.00E+00
33875_at	-0.0006	-1.001383	0.011742	-0.05477	0.05358	TRUE	1.00E+00
33454_at	-0.00058	-1.001336	0.039051	-0.18074	0.17959	TRUE	1.00E+00
1401_g_at	-0.00056	-1.00129	0.051729	-0.23921	0.2381	TRUE	1.00E+00
36111_s_at	-0.00055	-1.001267	0.023906	-0.11084	0.10975	TRUE	1.00E+00
33462_at	-0.00053	-1.001221	0.142434	-0.65766	0.6566	TRUE	1.00E+00
1271_g_at	-0.00049	-1.001129	0.028881	-0.13373	0.13275	TRUE	1.00E+00
34504_at	-0.00048	-1.001106	0.059425	-0.27464	0.27368	TRUE	1.00E+00
38556_at	-0.00047	-1.001083	0.025808	-0.11954	0.11859	TRUE	1.00E+00
33953_at	-0.00046	-1.00106	0.071114	-0.32855	0.32763	TRUE	1.00E+00
32078_at	-0.00045	-1.001037	0.021083	-0.09772	0.09681	TRUE	1.00E+00
31734_at	-0.00042	-1.000968	0.037978	-0.17563	0.17479	TRUE	1.00E+00
35235_at	-0.00041	-1.000945	0.032515	-0.15042	0.1496	TRUE	1.00E+00
33506_at	-0.00041	-1.000945	0.082213	-0.3797	0.37889	TRUE	1.00E+00
38435_at	-0.0004	-1.000921	0.021524	-0.09971	0.0989	TRUE	1.00E+00
40220_at	-0.0004	-1.000921	0.040184	-0.1858	0.18499	TRUE	1.00E+00
36429_at	-0.00033	-1.00076	0.080592	-0.37215	0.37149	TRUE	1.00E+00
41427_at	-0.00031	-1.000714	0.075584	-0.34903	0.3484	TRUE	1.00E+00
41429_at	-0.00031	-1.000714	0.02299	-0.10637	0.10576	TRUE	1.00E+00
39336_at	-0.0003	-1.000691	0.008906	-0.04139	0.04079	TRUE	1.00E+00
38390_at	-0.00029	-1.000668	0.019764	-0.09147	0.0909	TRUE	1.00E+00
36404_at	-0.00026	-1.000599	0.037563	-0.17356	0.17304	TRUE	1.00E+00
41759_at	-0.00023	-1.00053	0.035974	-0.1662	0.16574	TRUE	1.00E+00
39101_at	-0.00023	-1.00053	0.08914	-0.41148	0.41103	TRUE	1.00E+00
940_g_at	-0.00023	-1.00053	0.032816	-0.15162	0.15117	TRUE	1.00E+00
41154_r_at	-0.00021	-1.000484	0.024991	-0.11551	0.11509	TRUE	1.00E+00
40018_at	-0.0002	-1.000461	0.034054	-0.15731	0.15691	TRUE	1.00E+00
40600_at	-0.00019	-1.000438	0.054378	-0.25106	0.25069	TRUE	1.00E+00
36459_at	-0.00017	-1.000392	0.119391	-0.55099	0.55065	TRUE	1.00E+00
36995_at	-0.00006	-1.000138	0.024392	-0.11259	0.11248	TRUE	1.00E+00
33524_at	-0.00001	-1.000023	0.050811	-0.23443	0.23441	TRUE	1.00E+00
32097_at	0	1	0.121693	-0.56144	0.56145	TRUE	1.00E+00
41162_at	0.00001	1.000023	0.018377	-0.08477	0.0848	TRUE	1.00E+00
40131_at	0.00008	1.000184	0.026708	-0.12314	0.1233	TRUE	1.00E+00
32941_at	0.00009	1.000207	0.086999	-0.40128	0.40147	TRUE	1.00E+00
33051_at	0.00016	1.000368	0.134753	-0.62153	0.62186	TRUE	1.00E+00
34339_at	0.00016	1.000368	0.024971	-0.11505	0.11537	TRUE	1.00E+00
34828_at	0.00018	1.000415	0.018792	-0.08652	0.08688	TRUE	1.00E+00
39050_at	0.00021	1.000484	0.027101	-0.12483	0.12524	TRUE	1.00E+00
38017_at	0.00024	1.000553	0.04378	-0.20175	0.20222	TRUE	1.00E+00
34592_at	0.00026	1.000599	0.02236	-0.1029	0.10342	TRUE	1.00E+00
35056_at	0.00028	1.000645	0.125755	-0.57991	0.58046	TRUE	1.00E+00
37699_at	0.00033	1.00076	0.043742	-0.20148	0.20214	TRUE	1.00E+00
35444_at	0.00035	1.000806	0.041026	-0.18892	0.18963	TRUE	1.00E+00
40161_at	0.00036	1.000829	0.028836	-0.13268	0.13339	TRUE	1.00E+00
40851_r_at	0.00038	1.000875	0.083226	-0.38359	0.38435	TRUE	1.00E+00
1563_s_at	0.00038	1.000875	0.021131	-0.0971	0.09787	TRUE	1.00E+00
34862_at	0.00041	1.000945	0.03099	-0.14257	0.14338	TRUE	1.00E+00
33685_at	0.00042	1.000968	0.025284	-0.11623	0.11706	TRUE	1.00E+00
31416_at	0.00044	1.001014	0.024401	-0.11214	0.11301	TRUE	1.00E+00
34977_at	0.00044	1.001014	0.095907	-0.44203	0.44291	TRUE	1.00E+00

39777_at	0.00046	1.00106	0.028673	-0.13182	0.13275	TRUE	1.00E+00
40411_at	0.00049	1.001129	0.019995	-0.09176	0.09274	TRUE	1.00E+00
31435_at	0.00053	1.001221	0.105922	-0.48815	0.48921	TRUE	1.00E+00
39907_at	0.00053	1.001221	0.052576	-0.24203	0.2431	TRUE	1.00E+00
33942_s_at	0.00056	1.00129	0.012451	-0.05689	0.058	TRUE	1.00E+00
34200_at	0.00056	1.00129	0.039453	-0.18146	0.18258	TRUE	1.00E+00
374_f_at	0.0006	1.001383	0.020846	-0.09557	0.09678	TRUE	1.00E+00
1498_at	0.00061	1.001406	0.032921	-0.15128	0.15249	TRUE	1.00E+00
40711_at	0.00062	1.001429	0.075359	-0.34705	0.3483	TRUE	1.00E+00
356_at	0.00065	1.001498	0.052921	-0.24351	0.2448	TRUE	1.00E+00
32306_g_at	0.00065	1.001498	0.023525	-0.10789	0.10918	TRUE	1.00E+00
37998_at	0.00068	1.001567	0.030619	-0.14058	0.14195	TRUE	1.00E+00
31722_at	0.00071	1.001636	0.016254	-0.07428	0.0757	TRUE	1.00E+00
37337_at	0.00071	1.001636	0.025444	-0.11668	0.1181	TRUE	1.00E+00
39599_at	0.00074	1.001705	0.078236	-0.36021	0.36169	TRUE	1.00E+00
36355_at	0.00076	1.001751	0.024902	-0.11413	0.11564	TRUE	1.00E+00
33918_s_at	0.00076	1.001751	0.029469	-0.1352	0.13672	TRUE	1.00E+00
34621_at	0.00078	1.001798	0.018264	-0.08348	0.08505	TRUE	1.00E+00
39713_at	0.00079	1.001821	0.03522	-0.1617	0.16329	TRUE	1.00E+00
1744_at	0.00083	1.001913	0.039173	-0.1799	0.18156	TRUE	1.00E+00
36330_at	0.00085	1.001959	0.010828	-0.0491	0.05081	TRUE	1.00E+00
36592_at	0.00085	1.001959	0.018564	-0.08479	0.0865	TRUE	1.00E+00
1755_i_at	0.0009	1.002074	0.028513	-0.13065	0.13245	TRUE	1.00E+00
37138_at	0.00092	1.002121	0.083551	-0.38455	0.38639	TRUE	1.00E+00
36696_at	0.00093	1.002144	0.019942	-0.09108	0.09293	TRUE	1.00E+00
41133_at	0.00094	1.002167	0.019375	-0.08845	0.09033	TRUE	1.00E+00
38991_at	0.00095	1.00219	0.037283	-0.17106	0.17296	TRUE	1.00E+00
37728_r_at	0.00095	1.00219	0.014515	-0.06602	0.06792	TRUE	1.00E+00
33181_at	0.00096	1.002213	0.042648	-0.1958	0.19771	TRUE	1.00E+00
34512_at	0.00097	1.002236	0.043487	-0.19966	0.2016	TRUE	1.00E+00
39811_at	0.00099	1.002282	0.015923	-0.07247	0.07445	TRUE	1.00E+00
35448_at	0.00103	1.002374	0.077813	-0.35797	0.36002	TRUE	1.00E+00
37564_at	0.00106	1.002444	0.021165	-0.09658	0.09871	TRUE	1.00E+00
38123_at	0.0011	1.002536	0.017433	-0.07933	0.08152	TRUE	1.00E+00
38838_at	0.00111	1.002559	0.093216	-0.42895	0.43117	TRUE	1.00E+00
35813_at	0.00113	1.002605	0.014176	-0.06427	0.06653	TRUE	1.00E+00
35743_at	0.00115	1.002651	0.011333	-0.05114	0.05344	TRUE	1.00E+00
36119_at	0.00119	1.002744	0.024216	-0.11054	0.11291	TRUE	1.00E+00
32234_at	0.00122	1.002813	0.020005	-0.09107	0.09351	TRUE	1.00E+00
32416_at	0.00122	1.002813	0.060327	-0.2771	0.27955	TRUE	1.00E+00
245_at	0.00123	1.002836	0.087884	-0.40423	0.40669	TRUE	1.00E+00
40740_at	0.00123	1.002836	0.033352	-0.15264	0.15511	TRUE	1.00E+00
1442_at	0.00124	1.002859	0.037585	-0.17216	0.17464	TRUE	1.00E+00
35255_at	0.00126	1.002905	0.016041	-0.07274	0.07527	TRUE	1.00E+00
1853_at	0.00128	1.002952	0.032288	-0.14768	0.15025	TRUE	1.00E+00
1542_at	0.00133	1.003067	0.044301	-0.20306	0.20572	TRUE	1.00E+00
421_at	0.00144	1.003321	0.014033	-0.0633	0.06619	TRUE	1.00E+00
40444_s_at	0.00145	1.003344	0.028823	-0.13152	0.13443	TRUE	1.00E+00
40108_at	0.00147	1.003391	0.013809	-0.06224	0.06518	TRUE	1.00E+00
32282_at	0.00152	1.003506	0.028449	-0.12973	0.13277	TRUE	1.00E+00
34963_at	0.00153	1.003529	0.027748	-0.12649	0.12955	TRUE	1.00E+00

41614_at	0.00158	1.003645	0.031045	-0.14165	0.14481	TRUE	1.00E+00
37011_at	0.00159	1.003668	0.124084	-0.57088	0.57407	TRUE	1.00E+00
39078_at	0.00163	1.00376	0.012684	-0.05689	0.06014	TRUE	1.00E+00
32849_at	0.00163	1.00376	0.028498	-0.12985	0.13311	TRUE	1.00E+00
39990_at	0.00164	1.003783	0.036038	-0.16462	0.16791	TRUE	1.00E+00
33760_at	0.00165	1.003806	0.020651	-0.09362	0.09693	TRUE	1.00E+00
38074_at	0.00166	1.00383	0.006985	-0.03057	0.03389	TRUE	1.00E+00
39337_at	0.00166	1.00383	0.036449	-0.1665	0.16982	TRUE	1.00E+00
31996_at	0.00171	1.003945	0.020251	-0.09172	0.09514	TRUE	1.00E+00
31961_r_at	0.00173	1.003991	0.040082	-0.18319	0.18665	TRUE	1.00E+00
40942_g_at	0.00174	1.004015	0.023223	-0.1054	0.10888	TRUE	1.00E+00
34550_at	0.00174	1.004015	0.106445	-0.48935	0.49283	TRUE	1.00E+00
33046_f_at	0.00176	1.004061	0.042086	-0.19241	0.19592	TRUE	1.00E+00
32366_at	0.00177	1.004084	0.061724	-0.283	0.28654	TRUE	1.00E+00
1935_at	0.00178	1.004107	0.046994	-0.21503	0.21859	TRUE	1.00E+00
34684_at	0.00179	1.00413	0.040528	-0.18519	0.18876	TRUE	1.00E+00
38674_at	0.00181	1.004176	0.030073	-0.13694	0.14056	TRUE	1.00E+00
37613_at	0.00182	1.004199	0.043979	-0.20108	0.20472	TRUE	1.00E+00
32697_at	0.00182	1.004199	0.01577	-0.07093	0.07457	TRUE	1.00E+00
633_s_at	0.00182	1.004199	0.021956	-0.09948	0.10312	TRUE	1.00E+00
37811_at	0.00183	1.004223	0.032565	-0.14841	0.15208	TRUE	1.00E+00
33249_at	0.00184	1.004246	0.039799	-0.18178	0.18546	TRUE	1.00E+00
37272_at	0.00188	1.004338	0.020244	-0.09152	0.09527	TRUE	1.00E+00
40842_at	0.00189	1.004361	0.035783	-0.16321	0.16698	TRUE	1.00E+00
192_at	0.00191	1.004408	0.01764	-0.07948	0.08329	TRUE	1.00E+00
33709_at	0.00197	1.004546	0.059444	-0.27228	0.27622	TRUE	1.00E+00
453_at	0.002	1.004616	0.011325	-0.05025	0.05424	TRUE	1.00E+00
36860_at	0.00202	1.004662	0.030379	-0.13813	0.14218	TRUE	1.00E+00
36722_s_at	0.00203	1.004685	0.059559	-0.27275	0.27681	TRUE	1.00E+00
40612_at	0.00207	1.004778	0.057467	-0.26306	0.2672	TRUE	1.00E+00
1689_at	0.00208	1.004801	0.012504	-0.05561	0.05977	TRUE	1.00E+00
36649_at	0.00208	1.004801	0.031729	-0.1443	0.14847	TRUE	1.00E+00
1064_at	0.0021	1.004847	0.011942	-0.053	0.05719	TRUE	1.00E+00
31405_at	0.00216	1.004986	0.031699	-0.14408	0.14841	TRUE	1.00E+00
39778_at	0.00219	1.005055	0.019197	-0.08637	0.09076	TRUE	1.00E+00
33336_at	0.00221	1.005102	0.087106	-0.39966	0.40409	TRUE	1.00E+00
40714_at	0.00223	1.005148	0.064458	-0.29516	0.29961	TRUE	1.00E+00
34958_at	0.00224	1.005171	0.036426	-0.16582	0.1703	TRUE	1.00E+00
41827_f_at	0.00227	1.005241	0.033876	-0.15402	0.15856	TRUE	1.00E+00
39745_at	0.00232	1.005356	0.025156	-0.11374	0.11838	TRUE	1.00E+00
38343_at	0.00235	1.005426	0.031391	-0.14248	0.14718	TRUE	1.00E+00
33962_at	0.00235	1.005426	0.111907	-0.51394	0.51865	TRUE	1.00E+00
35317_at	0.00242	1.005588	0.025538	-0.1154	0.12024	TRUE	1.00E+00
33961_at	0.00244	1.005634	0.021165	-0.0952	0.10009	TRUE	1.00E+00
37298_at	0.00244	1.005634	0.014561	-0.06474	0.06962	TRUE	1.00E+00
35304_at	0.00245	1.005657	0.022897	-0.10318	0.10809	TRUE	1.00E+00
38073_at	0.00245	1.005657	0.026629	-0.1204	0.12531	TRUE	1.00E+00
40704_at	0.00249	1.00575	0.033142	-0.15041	0.15539	TRUE	1.00E+00
34606_s_at	0.00251	1.005796	0.104808	-0.48103	0.48605	TRUE	1.00E+00
1439_s_at	0.00251	1.005796	0.033129	-0.15033	0.15536	TRUE	1.00E+00
37803_at	0.00253	1.005843	0.018864	-0.0845	0.08957	TRUE	1.00E+00

affx-crex-3_at	0.00254	1.005866	0.385976	-1.77819	1.78327	TRUE	1.00E+00
36320_at	0.00257	1.005935	0.032342	-0.14664	0.15179	TRUE	1.00E+00
38709_at	0.00263	1.006074	0.014067	-0.06227	0.06753	TRUE	1.00E+00
36912_at	0.00265	1.006121	0.112216	-0.51507	0.52037	TRUE	1.00E+00
34731_at	0.00266	1.006144	0.023867	-0.10745	0.11277	TRUE	1.00E+00
35990_at	0.00267	1.006167	0.039029	-0.17739	0.18273	TRUE	1.00E+00
1444_at	0.00268	1.00619	0.01555	-0.06906	0.07442	TRUE	1.00E+00
37907_at	0.00283	1.006538	0.025057	-0.11277	0.11844	TRUE	1.00E+00
40909_at	0.00284	1.006561	0.051484	-0.23469	0.24036	TRUE	1.00E+00
38780_at	0.00288	1.006653	0.012389	-0.05428	0.06004	TRUE	1.00E+00
35741_at	0.00289	1.006677	0.010072	-0.04358	0.04936	TRUE	1.00E+00
32522_f_at	0.0029	1.0067	0.05728	-0.26136	0.26717	TRUE	1.00E+00
33315_at	0.0029	1.0067	0.049261	-0.22436	0.23017	TRUE	1.00E+00
38719_at	0.00292	1.006746	0.018112	-0.08064	0.08648	TRUE	1.00E+00
33930_at	0.00293	1.006769	0.019817	-0.0885	0.09435	TRUE	1.00E+00
38990_at	0.00296	1.006839	0.019337	-0.08625	0.09218	TRUE	1.00E+00
41656_at	0.00298	1.006885	0.020929	-0.09358	0.09954	TRUE	1.00E+00
37432_g_at	0.00299	1.006908	0.024014	-0.1078	0.11379	TRUE	1.00E+00
37757_at	0.00309	1.00714	0.022075	-0.09875	0.10493	TRUE	1.00E+00
817_at	0.0031	1.007164	0.031346	-0.14152	0.14771	TRUE	1.00E+00
33392_at	0.00315	1.00728	0.016563	-0.07327	0.07957	TRUE	1.00E+00
277_at	0.00317	1.007326	0.020656	-0.09213	0.09847	TRUE	1.00E+00
37308_at	0.00318	1.007349	0.040042	-0.18156	0.18792	TRUE	1.00E+00
32041_r_at	0.0032	1.007395	0.024163	-0.10828	0.11467	TRUE	1.00E+00
32573_at	0.0032	1.007395	0.027564	-0.12397	0.13037	TRUE	1.00E+00
41704_at	0.00321	1.007419	0.038676	-0.17523	0.18164	TRUE	1.00E+00
38928_r_at	0.00321	1.007419	0.049949	-0.22724	0.23365	TRUE	1.00E+00
37717_at	0.00322	1.007442	0.018016	-0.0799	0.08634	TRUE	1.00E+00
41225_at	0.00323	1.007465	0.026116	-0.11725	0.12372	TRUE	1.00E+00
40584_at	0.00329	1.007604	0.025175	-0.11285	0.11944	TRUE	1.00E+00
34238_at	0.00334	1.00772	0.095051	-0.43518	0.44187	TRUE	1.00E+00
1390_s_at	0.00341	1.007883	0.023219	-0.10371	0.11054	TRUE	1.00E+00
37672_at	0.00342	1.007906	0.030927	-0.13926	0.14611	TRUE	1.00E+00
40980_at	0.00345	1.007976	0.018124	-0.08016	0.08707	TRUE	1.00E+00
1067_at	0.0035	1.008092	0.02978	-0.13389	0.14089	TRUE	1.00E+00
38983_at	0.00353	1.008161	0.014986	-0.06561	0.07267	TRUE	1.00E+00
38296_at	0.00354	1.008184	0.01246	-0.05395	0.06103	TRUE	1.00E+00
41733_at	0.00357	1.008254	0.026075	-0.11673	0.12387	TRUE	1.00E+00
38164_at	0.00365	1.00844	0.01585	-0.06947	0.07677	TRUE	1.00E+00
36418_at	0.00365	1.00844	0.021868	-0.09724	0.10454	TRUE	1.00E+00
36608_at	0.00366	1.008463	0.009796	-0.04154	0.04885	TRUE	1.00E+00
39749_at	0.00366	1.008463	0.024675	-0.11019	0.1175	TRUE	1.00E+00
33971_f_at	0.00366	1.008463	0.085649	-0.39149	0.39881	TRUE	1.00E+00
38713_at	0.00366	1.008463	0.027508	-0.12325	0.13058	TRUE	1.00E+00
38815_at	0.00369	1.008533	0.024237	-0.10813	0.1155	TRUE	1.00E+00
905_at	0.0037	1.008556	0.031714	-0.14262	0.15001	TRUE	1.00E+00
36022_at	0.00373	1.008626	0.125374	-0.57469	0.58216	TRUE	1.00E+00
1116_at	0.00377	1.008719	0.038143	-0.17221	0.17975	TRUE	1.00E+00
41047_at	0.00387	1.008951	0.018625	-0.08206	0.08979	TRUE	1.00E+00
35685_at	0.00397	1.009183	0.012894	-0.05552	0.06345	TRUE	1.00E+00
1751_g_at	0.00399	1.00923	0.022568	-0.10013	0.10811	TRUE	1.00E+00

41630_at	0.00403	1.009323	0.088055	-0.40222	0.41027	TRUE	1.00E+00
31742_at	0.00405	1.009369	0.047578	-0.21545	0.22356	TRUE	1.00E+00
36375_at	0.00407	1.009416	0.031758	-0.14245	0.15059	TRUE	1.00E+00
35967_at	0.00407	1.009416	0.019674	-0.08669	0.09484	TRUE	1.00E+00
39058_at	0.00413	1.009555	0.020813	-0.09189	0.10015	TRUE	1.00E+00
41519_at	0.00413	1.009555	0.056399	-0.25607	0.26433	TRUE	1.00E+00
39606_at	0.00415	1.009602	0.077702	-0.35434	0.36263	TRUE	1.00E+00
35720_at	0.00416	1.009625	0.031113	-0.13938	0.14771	TRUE	1.00E+00
31993_f_at	0.00417	1.009648	0.027202	-0.12133	0.12967	TRUE	1.00E+00
31971_at	0.00424	1.009811	0.059751	-0.27143	0.2799	TRUE	1.00E+00
31943_g_at	0.00425	1.009834	0.025852	-0.11502	0.12352	TRUE	1.00E+00
33186_i_at	0.00426	1.009857	0.013978	-0.06023	0.06875	TRUE	1.00E+00
147_at	0.00428	1.009904	0.022398	-0.09905	0.10762	TRUE	1.00E+00
31315_at	0.00431	1.009974	0.061971	-0.2816	0.29022	TRUE	1.00E+00
31949_at	0.00432	1.009997	0.068928	-0.31368	0.32233	TRUE	1.00E+00
34469_at	0.00437	1.010113	0.023357	-0.10339	0.11213	TRUE	1.00E+00
39130_at	0.00447	1.010346	0.027999	-0.1247	0.13364	TRUE	1.00E+00
36632_at	0.00447	1.010346	0.029784	-0.13294	0.14189	TRUE	1.00E+00
32856_at	0.00449	1.010392	0.026881	-0.11953	0.12851	TRUE	1.00E+00
37388_at	0.00453	1.010485	0.121855	-0.55766	0.56672	TRUE	1.00E+00
33371_s_at	0.00454	1.010509	0.023734	-0.10496	0.11403	TRUE	1.00E+00
39308_r_at	0.00463	1.010718	0.033918	-0.15186	0.16111	TRUE	1.00E+00
39549_at	0.00466	1.010788	0.059226	-0.26858	0.27791	TRUE	1.00E+00
36557_at	0.00467	1.010811	0.024349	-0.10767	0.11701	TRUE	1.00E+00
33985_s_at	0.00469	1.010858	0.056506	-0.25601	0.26538	TRUE	1.00E+00
1022_f_at	0.00469	1.010858	0.024419	-0.10797	0.11735	TRUE	1.00E+00
1947_g_at	0.0047	1.010881	0.046317	-0.20898	0.21839	TRUE	1.00E+00
41281_s_at	0.00471	1.010904	0.027617	-0.1227	0.13213	TRUE	1.00E+00
36820_r_at	0.00472	1.010927	0.062763	-0.28484	0.29428	TRUE	1.00E+00
38846_at	0.00477	1.011044	0.01589	-0.06854	0.07809	TRUE	1.00E+00
36107_at	0.00479	1.01109	0.017948	-0.07802	0.08759	TRUE	1.00E+00
31855_at	0.0048	1.011114	0.024333	-0.10746	0.11706	TRUE	1.00E+00
37575_at	0.0048	1.011114	0.020117	-0.08801	0.09761	TRUE	1.00E+00
37610_at	0.00484	1.011207	0.023895	-0.1054	0.11508	TRUE	1.00E+00
38240_at	0.00485	1.01123	0.060745	-0.2754	0.2851	TRUE	1.00E+00
832_at	0.00486	1.011253	0.018132	-0.0788	0.08851	TRUE	1.00E+00
32009_at	0.00492	1.011393	0.076413	-0.34762	0.35746	TRUE	1.00E+00
37188_at	0.00493	1.011416	0.02405	-0.10602	0.11589	TRUE	1.00E+00
37165_f_at	0.00496	1.011486	0.077689	-0.35346	0.36339	TRUE	1.00E+00
31917_at	0.00499	1.011556	0.033324	-0.14876	0.15873	TRUE	1.00E+00
34555_at	0.00504	1.011673	0.039102	-0.17536	0.18544	TRUE	1.00E+00
41711_at	0.00505	1.011696	0.072298	-0.3285	0.3386	TRUE	1.00E+00
33150_at	0.00506	1.011719	0.009791	-0.04011	0.05023	TRUE	1.00E+00
37111_g_at	0.00506	1.011719	0.032216	-0.14357	0.15369	TRUE	1.00E+00
36679_at	0.00507	1.011743	0.01443	-0.06151	0.07165	TRUE	1.00E+00
33614_at	0.00507	1.011743	0.023802	-0.10474	0.11488	TRUE	1.00E+00
32878_f_at	0.00507	1.011743	0.031588	-0.14066	0.15081	TRUE	1.00E+00
35677_at	0.00512	1.011859	0.021741	-0.09518	0.10543	TRUE	1.00E+00
38947_at	0.00518	1.011999	0.046084	-0.20744	0.21779	TRUE	1.00E+00
1873_at	0.00521	1.012069	0.017569	-0.07585	0.08626	TRUE	1.00E+00
36875_at	0.00524	1.012139	0.023449	-0.10294	0.11343	TRUE	1.00E+00

33935_at	0.00529	1.012255	0.091257	-0.41573	0.42631	TRUE	1.00E+00
41230_at	0.0053	1.012278	0.036074	-0.16113	0.17174	TRUE	1.00E+00
1352_at	0.00537	1.012442	0.026135	-0.1152	0.12595	TRUE	1.00E+00
32261_at	0.00537	1.012442	0.015487	-0.06608	0.07682	TRUE	1.00E+00
40578_s_at	0.0054	1.012512	0.118433	-0.54101	0.5518	TRUE	1.00E+00
721_g_at	0.00545	1.012628	0.055148	-0.24898	0.25988	TRUE	1.00E+00
35310_at	0.00545	1.012628	0.029547	-0.13087	0.14176	TRUE	1.00E+00
37252_at	0.00546	1.012651	0.018426	-0.07955	0.09047	TRUE	1.00E+00
35545_at	0.00551	1.012768	0.030267	-0.13413	0.14515	TRUE	1.00E+00
33022_at	0.00553	1.012815	0.030341	-0.13445	0.14551	TRUE	1.00E+00
32311_r_at	0.00559	1.012955	0.034156	-0.15199	0.16317	TRUE	1.00E+00
35214_at	0.0056	1.012978	0.028867	-0.12758	0.13878	TRUE	1.00E+00
35769_at	0.00561	1.013001	0.026082	-0.11472	0.12594	TRUE	1.00E+00
41205_at	0.00562	1.013025	0.019695	-0.08525	0.09649	TRUE	1.00E+00
35739_at	0.0057	1.013211	0.029798	-0.13178	0.14317	TRUE	1.00E+00
33820_g_at	0.0057	1.013211	0.021188	-0.09206	0.10345	TRUE	1.00E+00
41298_at	0.0057	1.013211	0.048323	-0.21725	0.22864	TRUE	1.00E+00
34171_at	0.00575	1.013328	0.039182	-0.17503	0.18652	TRUE	1.00E+00
37381_g_at	0.00579	1.013421	0.01695	-0.07241	0.08399	TRUE	1.00E+00
556_s_at	0.00579	1.013421	0.01546	-0.06553	0.07712	TRUE	1.00E+00
39123_s_at	0.00584	1.013538	0.01704	-0.07277	0.08446	TRUE	1.00E+00
1615_at	0.00589	1.013655	0.032797	-0.14542	0.15721	TRUE	1.00E+00
36115_at	0.00597	1.013841	0.018441	-0.07911	0.09104	TRUE	1.00E+00
38490_r_at	0.00603	1.013981	0.06153	-0.27785	0.2899	TRUE	1.00E+00
35166_at	0.00603	1.013981	0.023226	-0.10113	0.11318	TRUE	1.00E+00
38686_at	0.00604	1.014005	0.022089	-0.09587	0.10795	TRUE	1.00E+00
32683_at	0.00606	1.014051	0.076442	-0.34662	0.35873	TRUE	1.00E+00
37785_at	0.00607	1.014075	0.030836	-0.1362	0.14833	TRUE	1.00E+00
40680_at	0.00607	1.014075	0.049672	-0.22309	0.23524	TRUE	1.00E+00
41430_at	0.00611	1.014168	0.025484	-0.11146	0.12369	TRUE	1.00E+00
34747_at	0.00616	1.014285	0.019585	-0.0842	0.09652	TRUE	1.00E+00
41417_at	0.00616	1.014285	0.008953	-0.03514	0.04747	TRUE	1.00E+00
1377_at	0.00617	1.014308	0.030146	-0.13291	0.14526	TRUE	1.00E+00
34933_at	0.00622	1.014425	0.030451	-0.13427	0.14671	TRUE	1.00E+00
33564_at	0.00627	1.014542	0.121272	-0.55323	0.56577	TRUE	1.00E+00
37382_at	0.00627	1.014542	0.023778	-0.10343	0.11597	TRUE	1.00E+00
34298_at	0.00627	1.014542	0.03515	-0.15589	0.16844	TRUE	1.00E+00
36732_at	0.00631	1.014635	0.015786	-0.06653	0.07914	TRUE	1.00E+00
33908_at	0.00635	1.014729	0.021078	-0.09089	0.1036	TRUE	1.00E+00
36622_at	0.00642	1.014892	0.065237	-0.29455	0.3074	TRUE	1.00E+00
37722_s_at	0.00643	1.014916	0.02214	-0.09572	0.10857	TRUE	1.00E+00
31694_at	0.00645	1.014963	0.081439	-0.36928	0.38217	TRUE	1.00E+00
33914_r_at	0.00648	1.015033	0.055677	-0.25039	0.26335	TRUE	1.00E+00
34193_at	0.00651	1.015103	0.109655	-0.49939	0.51241	TRUE	1.00E+00
1518_at	0.00651	1.015103	0.066356	-0.29963	0.31265	TRUE	1.00E+00
40774_at	0.00656	1.01522	0.020204	-0.08665	0.09977	TRUE	1.00E+00
778_s_at	0.00658	1.015266	0.020999	-0.0903	0.10346	TRUE	1.00E+00
41727_at	0.00658	1.015266	0.02665	-0.11637	0.12953	TRUE	1.00E+00
37815_at	0.00672	1.015594	0.11479	-0.52287	0.53631	TRUE	1.00E+00
32883_at	0.00673	1.015617	0.038485	-0.17082	0.18429	TRUE	1.00E+00
34484_at	0.00675	1.015664	0.018392	-0.0781	0.0916	TRUE	1.00E+00

34139_at	0.00679	1.015757	0.038058	-0.16879	0.18237	TRUE	1.00E+00
40490_at	0.0068	1.015781	0.018909	-0.08044	0.09404	TRUE	1.00E+00
32650_at	0.00691	1.016038	0.042454	-0.18896	0.20277	TRUE	1.00E+00
33153_at	0.00691	1.016038	0.039629	-0.17592	0.18974	TRUE	1.00E+00
37254_at	0.00693	1.016085	0.021198	-0.09087	0.10473	TRUE	1.00E+00
34651_at	0.00697	1.016178	0.015577	-0.06489	0.07884	TRUE	1.00E+00
37581_at	0.00697	1.016178	0.009565	-0.03716	0.0511	TRUE	1.00E+00
38392_at	0.00698	1.016202	0.021117	-0.09044	0.1044	TRUE	1.00E+00
32180_s_at	0.00702	1.016295	0.013274	-0.05423	0.06826	TRUE	1.00E+00
39514_s_at	0.00702	1.016295	0.030571	-0.13402	0.14806	TRUE	1.00E+00
36815_at	0.00703	1.016319	0.016687	-0.06996	0.08401	TRUE	1.00E+00
37444_at	0.00707	1.016413	0.018971	-0.08045	0.09459	TRUE	1.00E+00
41315_at	0.00711	1.016506	0.035544	-0.15688	0.17109	TRUE	1.00E+00
2067_f_at	0.00713	1.016553	0.013014	-0.05292	0.06717	TRUE	1.00E+00
36067_at	0.00713	1.016553	0.035672	-0.15744	0.17171	TRUE	1.00E+00
32235_at	0.00715	1.0166	0.025347	-0.10979	0.1241	TRUE	1.00E+00
41099_at	0.00722	1.016764	0.031576	-0.13846	0.1529	TRUE	1.00E+00
40467_at	0.00723	1.016787	0.049091	-0.21925	0.23372	TRUE	1.00E+00
248_at	0.00724	1.01681	0.029732	-0.12993	0.14441	TRUE	1.00E+00
32861_s_at	0.00728	1.016904	0.084574	-0.38291	0.39746	TRUE	1.00E+00
40140_at	0.00728	1.016904	0.017747	-0.0746	0.08916	TRUE	1.00E+00
32815_at	0.0073	1.016951	0.049076	-0.21912	0.23371	TRUE	1.00E+00
37462_i_at	0.00731	1.016974	0.025189	-0.10891	0.12352	TRUE	1.00E+00
41862_at	0.00732	1.016998	0.023423	-0.10074	0.11539	TRUE	1.00E+00
498_at	0.00734	1.017045	0.018103	-0.07618	0.09086	TRUE	1.00E+00
41340_at	0.00734	1.017045	0.028908	-0.12603	0.14072	TRUE	1.00E+00
33912_at	0.00735	1.017068	0.025352	-0.10961	0.12432	TRUE	1.00E+00
32851_at	0.00736	1.017091	0.040177	-0.17799	0.19272	TRUE	1.00E+00
34595_at	0.00741	1.017209	0.063414	-0.28516	0.29997	TRUE	1.00E+00
38635_at	0.00742	1.017232	0.025357	-0.10957	0.12441	TRUE	1.00E+00
34734_at	0.00751	1.017443	0.051044	-0.22799	0.243	TRUE	1.00E+00
37830_at	0.00753	1.01749	0.098225	-0.44564	0.4607	TRUE	1.00E+00
40180_at	0.00756	1.01756	0.051193	-0.22863	0.24374	TRUE	1.00E+00
38034_at	0.00764	1.017747	0.033063	-0.1449	0.16018	TRUE	1.00E+00
38110_at	0.00767	1.017818	0.017419	-0.07269	0.08803	TRUE	1.00E+00
39666_at	0.0077	1.017888	0.056346	-0.25225	0.26766	TRUE	1.00E+00
37268_at	0.00771	1.017911	0.018425	-0.0773	0.09271	TRUE	1.00E+00
34024_at	0.00771	1.017911	0.032235	-0.14101	0.15643	TRUE	1.00E+00
35578_at	0.00771	1.017911	0.098305	-0.44583	0.46125	TRUE	1.00E+00
1045_s_at	0.00771	1.017911	0.032967	-0.14438	0.15981	TRUE	1.00E+00
40827_at	0.00774	1.017982	0.00699	-0.02451	0.03999	TRUE	1.00E+00
38621_at	0.00778	1.018076	0.017478	-0.07286	0.08841	TRUE	1.00E+00
976_s_at	0.00782	1.018169	0.015188	-0.06225	0.07789	TRUE	1.00E+00
1796_s_at	0.00782	1.018169	0.021912	-0.09327	0.10891	TRUE	1.00E+00
39113_at	0.00782	1.018169	0.05055	-0.22539	0.24104	TRUE	1.00E+00
31912_at	0.00786	1.018263	0.047836	-0.21283	0.22855	TRUE	1.00E+00
32335_r_at	0.00788	1.01831	0.014225	-0.05774	0.07351	TRUE	1.00E+00
33989_f_at	0.00789	1.018333	0.019996	-0.08436	0.10015	TRUE	1.00E+00
31492_at	0.00791	1.01838	0.030491	-0.13277	0.14858	TRUE	1.00E+00
32316_s_at	0.00793	1.018427	0.020413	-0.08625	0.10211	TRUE	1.00E+00
1251_g_at	0.00793	1.018427	0.083558	-0.37757	0.39344	TRUE	1.00E+00

1476_s_at	0.00796	1.018498	0.069715	-0.31367	0.3296	TRUE	1.00E+00
33734_at	0.00797	1.018521	0.030967	-0.1349	0.15084	TRUE	1.00E+00
36089_at	0.008	1.018591	0.055012	-0.2458	0.2618	TRUE	1.00E+00
35287_at	0.00801	1.018615	0.081037	-0.36586	0.38188	TRUE	1.00E+00
35264_at	0.00802	1.018638	0.020824	-0.08806	0.10409	TRUE	1.00E+00
1131_at	0.00805	1.018709	0.040681	-0.17964	0.19573	TRUE	1.00E+00
1345_s_at	0.00808	1.018779	0.033965	-0.14862	0.16478	TRUE	1.00E+00
33489_at	0.00817	1.01899	0.042289	-0.18693	0.20327	TRUE	1.00E+00
39885_at	0.00822	1.019108	0.048689	-0.21641	0.23285	TRUE	1.00E+00
341_at	0.00828	1.019248	0.04669	-0.20713	0.22369	TRUE	1.00E+00
36466_at	0.0083	1.019295	0.043628	-0.19299	0.20958	TRUE	1.00E+00
40414_at	0.00831	1.019319	0.01798	-0.07464	0.09127	TRUE	1.00E+00
40055_s_at	0.00831	1.019319	0.050336	-0.22391	0.24054	TRUE	1.00E+00
458_at	0.00835	1.019413	0.074635	-0.33598	0.35269	TRUE	1.00E+00
32854_at	0.00838	1.019483	0.017575	-0.0727	0.08947	TRUE	1.00E+00
829_s_at	0.00843	1.0196	0.027591	-0.11886	0.13572	TRUE	1.00E+00
36021_at	0.00844	1.019624	0.06122	-0.274	0.29089	TRUE	1.00E+00
31999_at	0.00846	1.019671	0.025743	-0.11031	0.12722	TRUE	1.00E+00
37044_at	0.00847	1.019694	0.054658	-0.2437	0.26064	TRUE	1.00E+00
35531_at	0.00853	1.019835	0.07232	-0.32512	0.34219	TRUE	1.00E+00
41132_r_at	0.00854	1.019859	0.020282	-0.08504	0.10211	TRUE	1.00E+00
38909_at	0.0086	1.02	0.090995	-0.41121	0.42841	TRUE	1.00E+00
173_at	0.00861	1.020023	0.056615	-0.25259	0.26981	TRUE	1.00E+00
40974_at	0.00861	1.020023	0.02535	-0.10835	0.12557	TRUE	1.00E+00
31898_at	0.00866	1.020141	0.023196	-0.09836	0.11567	TRUE	1.00E+00
36578_at	0.00866	1.020141	0.020287	-0.08494	0.10226	TRUE	1.00E+00
34037_at	0.0087	1.020234	0.061463	-0.27486	0.29227	TRUE	1.00E+00
36615_at	0.0087	1.020234	0.013886	-0.05536	0.07277	TRUE	1.00E+00
843_at	0.00871	1.020258	0.027989	-0.12042	0.13784	TRUE	1.00E+00
38976_at	0.00872	1.020281	0.020394	-0.08537	0.10281	TRUE	1.00E+00
39140_at	0.00876	1.020375	0.022233	-0.09382	0.11133	TRUE	1.00E+00
35795_at	0.00876	1.020375	0.015418	-0.06237	0.0799	TRUE	1.00E+00
35957_at	0.00877	1.020399	0.07745	-0.34856	0.36609	TRUE	1.00E+00
31882_at	0.00878	1.020422	0.008935	-0.03245	0.05	TRUE	1.00E+00
36264_at	0.00879	1.020446	0.028657	-0.12342	0.141	TRUE	1.00E+00
41451_s_at	0.00879	1.020446	0.047727	-0.2114	0.22898	TRUE	1.00E+00
39121_at	0.00879	1.020446	0.064304	-0.28788	0.30547	TRUE	1.00E+00
41600_at	0.0088	1.020469	0.023273	-0.09857	0.11617	TRUE	1.00E+00
36203_at	0.00884	1.020563	0.027893	-0.11985	0.13753	TRUE	1.00E+00
39124_r_at	0.00898	1.020892	0.027968	-0.12005	0.13801	TRUE	1.00E+00
35846_at	0.00901	1.020963	0.063131	-0.28225	0.30027	TRUE	1.00E+00
37919_at	0.00901	1.020963	0.075229	-0.33806	0.35609	TRUE	1.00E+00
36636_at	0.00905	1.021057	0.016788	-0.0684	0.08651	TRUE	1.00E+00
36387_at	0.00905	1.021057	0.070992	-0.31848	0.33658	TRUE	1.00E+00
35013_at	0.00913	1.021245	0.022715	-0.09567	0.11393	TRUE	1.00E+00
32493_at	0.00914	1.021269	0.033496	-0.1454	0.16368	TRUE	1.00E+00
32192_g_at	0.00915	1.021292	0.017609	-0.07209	0.09039	TRUE	1.00E+00
38799_at	0.00916	1.021316	0.099523	-0.45	0.46832	TRUE	1.00E+00
40408_at	0.0092	1.02141	0.018472	-0.07603	0.09442	TRUE	1.00E+00
32713_at	0.0093	1.021645	0.022465	-0.09434	0.11294	TRUE	1.00E+00
37704_at	0.00931	1.021668	0.040845	-0.17914	0.19775	TRUE	1.00E+00

1706_at	0.00935	1.021763	0.01633	-0.06599	0.08469	TRUE	1.00E+00
34549_g_at	0.00943	1.021951	0.078494	-0.35271	0.37157	TRUE	1.00E+00
38985_at	0.00944	1.021974	0.013367	-0.05223	0.07111	TRUE	1.00E+00
40080_at	0.00947	1.022045	0.021712	-0.0907	0.10964	TRUE	1.00E+00
35774_r_at	0.00948	1.022068	0.016491	-0.06661	0.08556	TRUE	1.00E+00
307_at	0.00948	1.022068	0.050344	-0.22279	0.24175	TRUE	1.00E+00
40218_at	0.00951	1.022139	0.06271	-0.27981	0.29882	TRUE	1.00E+00
39325_at	0.00952	1.022163	0.040734	-0.17841	0.19745	TRUE	1.00E+00
38733_at	0.00952	1.022163	0.023937	-0.10092	0.11996	TRUE	1.00E+00
33785_at	0.00957	1.02228	0.078821	-0.35408	0.37322	TRUE	1.00E+00
40927_at	0.00957	1.02228	0.027684	-0.11815	0.13729	TRUE	1.00E+00
35074_at	0.00961	1.022374	0.018615	-0.07627	0.0955	TRUE	1.00E+00
117_at	0.00961	1.022374	0.079595	-0.35761	0.37683	TRUE	1.00E+00
41664_at	0.00964	1.022445	0.021583	-0.08993	0.10922	TRUE	1.00E+00
38264_at	0.00965	1.022469	0.033023	-0.14271	0.162	TRUE	1.00E+00
38627_at	0.0097	1.022586	0.071249	-0.31902	0.33841	TRUE	1.00E+00
33304_at	0.0097	1.022586	0.046416	-0.20444	0.22385	TRUE	1.00E+00
35202_at	0.00972	1.022633	0.017824	-0.07251	0.09196	TRUE	1.00E+00
34189_at	0.00973	1.022657	0.018259	-0.07451	0.09397	TRUE	1.00E+00
38697_at	0.00974	1.022681	0.009531	-0.03424	0.05371	TRUE	1.00E+00
39461_s_at	0.00976	1.022728	0.03051	-0.131	0.15052	TRUE	1.00E+00
33684_at	0.00978	1.022775	0.044566	-0.19583	0.2154	TRUE	1.00E+00
41591_at	0.00979	1.022798	0.018386	-0.07504	0.09462	TRUE	1.00E+00
34483_at	0.00984	1.022916	0.068127	-0.30446	0.32415	TRUE	1.00E+00
155_s_at	0.00985	1.02294	0.02526	-0.10669	0.12639	TRUE	1.00E+00
39814_s_at	0.00986	1.022963	0.038827	-0.16927	0.18899	TRUE	1.00E+00
35016_at	0.00996	1.023199	0.027119	-0.11516	0.13507	TRUE	1.00E+00
38244_at	0.01	1.023293	0.14842	-0.67475	0.69474	TRUE	1.00E+00
32946_r_at	0.01	1.023293	0.115171	-0.52135	0.54135	TRUE	1.00E+00
39868_at	0.01002	1.02334	0.02165	-0.08987	0.1099	TRUE	1.00E+00
41550_at	0.01002	1.02334	0.01975	-0.0811	0.10114	TRUE	1.00E+00
1184_at	0.01005	1.023411	0.042384	-0.1855	0.20559	TRUE	1.00E+00
1985_s_at	0.01009	1.023505	0.038047	-0.16544	0.18562	TRUE	1.00E+00
34393_r_at	0.01011	1.023552	0.022031	-0.09153	0.11175	TRUE	1.00E+00
34076_at	0.01011	1.023552	0.023948	-0.10037	0.1206	TRUE	1.00E+00
40451_at	0.01013	1.023599	0.079474	-0.35653	0.37679	TRUE	1.00E+00
41667_s_at	0.01014	1.023623	0.013089	-0.05025	0.07052	TRUE	1.00E+00
292_s_at	0.01016	1.02367	0.040146	-0.17506	0.19537	TRUE	1.00E+00
32428_at	0.01018	1.023717	0.07402	-0.33132	0.35167	TRUE	1.00E+00
41789_r_at	0.01018	1.023717	0.050344	-0.22208	0.24245	TRUE	1.00E+00
36550_at	0.01024	1.023859	0.015267	-0.06019	0.08068	TRUE	1.00E+00
35975_at	0.01025	1.023882	0.036442	-0.15788	0.17838	TRUE	1.00E+00
36926_at	0.01025	1.023882	0.016777	-0.06715	0.08765	TRUE	1.00E+00
31428_at	0.01028	1.023953	0.039624	-0.17252	0.19309	TRUE	1.00E+00
40725_at	0.01029	1.023977	0.011566	-0.04307	0.06365	TRUE	1.00E+00
35658_at	0.0103	1.024	0.028499	-0.12118	0.14179	TRUE	1.00E+00
38257_at	0.01031	1.024024	0.033423	-0.14389	0.16451	TRUE	1.00E+00
33844_at	0.01034	1.024094	0.014745	-0.05769	0.07836	TRUE	1.00E+00
34855_at	0.01036	1.024142	0.018638	-0.07563	0.09635	TRUE	1.00E+00
38748_at	0.01039	1.024212	0.04573	-0.20059	0.22137	TRUE	1.00E+00
40329_at	0.01042	1.024283	0.035253	-0.15223	0.17306	TRUE	1.00E+00

33190_g_at	0.01044	1.02433	0.09416	-0.42397	0.44486	TRUE	1.00E+00
37848_at	0.01045	1.024354	0.044465	-0.19469	0.2156	TRUE	1.00E+00
40005_at	0.01047	1.024401	0.017259	-0.06915	0.0901	TRUE	1.00E+00
33170_at	0.01051	1.024495	0.010927	-0.03991	0.06092	TRUE	1.00E+00
35432_at	0.01052	1.024519	0.025142	-0.10548	0.12651	TRUE	1.00E+00
33535_at	0.01054	1.024566	0.089598	-0.40283	0.4239	TRUE	1.00E+00
37904_s_at	0.01057	1.024637	0.026028	-0.10952	0.13065	TRUE	1.00E+00
39643_at	0.01057	1.024637	0.021042	-0.08651	0.10765	TRUE	1.00E+00
32707_at	0.01059	1.024684	0.059019	-0.26169	0.28288	TRUE	1.00E+00
32757_at	0.0106	1.024708	0.011756	-0.04364	0.06483	TRUE	1.00E+00
36431_at	0.0106	1.024708	0.052962	-0.23374	0.25494	TRUE	1.00E+00
41147_at	0.01062	1.024755	0.020975	-0.08615	0.10739	TRUE	1.00E+00
32586_at	0.01062	1.024755	0.014185	-0.05482	0.07607	TRUE	1.00E+00
31769_at	0.01063	1.024778	0.030824	-0.13158	0.15285	TRUE	1.00E+00
33418_at	0.01069	1.02492	0.024387	-0.10182	0.1232	TRUE	1.00E+00
40188_f_at	0.0107	1.024944	0.051195	-0.2255	0.24689	TRUE	1.00E+00
39911_at	0.01073	1.025014	0.032763	-0.14043	0.16188	TRUE	1.00E+00
1561_at	0.01074	1.025038	0.052171	-0.22995	0.25144	TRUE	1.00E+00
35642_at	0.01076	1.025085	0.022506	-0.09308	0.11459	TRUE	1.00E+00
31884_at	0.01087	1.025345	0.01569	-0.06152	0.08326	TRUE	1.00E+00
36667_at	0.01088	1.025369	0.025293	-0.10581	0.12757	TRUE	1.00E+00
39551_at	0.0109	1.025416	0.031453	-0.13421	0.15602	TRUE	1.00E+00
41514_s_at	0.01096	1.025557	0.020608	-0.08412	0.10604	TRUE	1.00E+00
33694_at	0.01098	1.025605	0.021308	-0.08733	0.10928	TRUE	1.00E+00
34225_at	0.01099	1.025628	0.024015	-0.09981	0.12178	TRUE	1.00E+00
33549_at	0.01099	1.025628	0.053632	-0.23645	0.25843	TRUE	1.00E+00
34075_at	0.011	1.025652	0.097959	-0.44094	0.46294	TRUE	1.00E+00
40074_at	0.01103	1.025723	0.024647	-0.10268	0.12474	TRUE	1.00E+00
39468_r_at	0.01104	1.025746	0.036033	-0.1552	0.17729	TRUE	1.00E+00
1375_s_at	0.01105	1.02577	0.037296	-0.16102	0.18312	TRUE	1.00E+00
33275_at	0.01105	1.02577	0.017729	-0.07074	0.09285	TRUE	1.00E+00
33660_at	0.01108	1.025841	0.020756	-0.08468	0.10684	TRUE	1.00E+00
31634_at	0.0111	1.025888	0.036612	-0.15782	0.18001	TRUE	1.00E+00
965_at	0.01114	1.025983	0.036967	-0.15941	0.18169	TRUE	1.00E+00
41223_at	0.01117	1.026053	0.020932	-0.08541	0.10774	TRUE	1.00E+00
38441_s_at	0.01118	1.026077	0.022959	-0.09474	0.1171	TRUE	1.00E+00
39639_s_at	0.01127	1.02629	0.057126	-0.25228	0.27483	TRUE	1.00E+00
662_at	0.01129	1.026337	0.018472	-0.07393	0.09651	TRUE	1.00E+00
772_at	0.01134	1.026455	0.029177	-0.12327	0.14595	TRUE	1.00E+00
40187_at	0.01138	1.02655	0.033887	-0.14496	0.16772	TRUE	1.00E+00
448_s_at	0.0114	1.026597	0.023557	-0.09728	0.12008	TRUE	1.00E+00
1782_s_at	0.01144	1.026692	0.053833	-0.23692	0.2598	TRUE	1.00E+00
33493_at	0.01145	1.026715	0.028227	-0.11877	0.14168	TRUE	1.00E+00
510_g_at	0.01147	1.026763	0.026683	-0.11163	0.13457	TRUE	1.00E+00
41201_at	0.01149	1.02681	0.017335	-0.06849	0.09146	TRUE	1.00E+00
34055_at	0.01149	1.02681	0.045855	-0.20007	0.22305	TRUE	1.00E+00
37036_at	0.0115	1.026833	0.030369	-0.12861	0.15161	TRUE	1.00E+00
32310_f_at	0.01155	1.026952	0.014389	-0.05484	0.07793	TRUE	1.00E+00
38533_s_at	0.01156	1.026975	0.042619	-0.18506	0.20819	TRUE	1.00E+00
40867_at	0.0116	1.02707	0.034691	-0.14845	0.17165	TRUE	1.00E+00
34278_at	0.01168	1.027259	0.032875	-0.13999	0.16335	TRUE	1.00E+00

34120_r_at	0.01171	1.02733	0.115076	-0.51921	0.54262	TRUE	1.00E+00
522_s_at	0.01171	1.02733	0.053292	-0.23415	0.25758	TRUE	1.00E+00
32558_at	0.01172	1.027354	0.012827	-0.04745	0.0709	TRUE	1.00E+00
33806_at	0.01174	1.027401	0.010034	-0.03455	0.05803	TRUE	1.00E+00
38132_at	0.01174	1.027401	0.017318	-0.06816	0.09164	TRUE	1.00E+00
38665_at	0.01175	1.027425	0.055532	-0.24445	0.26795	TRUE	1.00E+00
34347_at	0.01177	1.027472	0.031629	-0.13416	0.15769	TRUE	1.00E+00
499_at	0.01177	1.027472	0.080607	-0.36012	0.38366	TRUE	1.00E+00
37352_at	0.01178	1.027496	0.013429	-0.05018	0.07374	TRUE	1.00E+00
34737_at	0.01183	1.027614	0.024092	-0.09932	0.12298	TRUE	1.00E+00
34824_at	0.01189	1.027756	0.014591	-0.05543	0.07921	TRUE	1.00E+00
34749_at	0.01189	1.027756	0.03609	-0.15461	0.1784	TRUE	1.00E+00
36403_s_at	0.0119	1.02778	0.035571	-0.1522	0.17601	TRUE	1.00E+00
33092_at	0.01194	1.027874	0.034347	-0.14652	0.1704	TRUE	1.00E+00
40031_at	0.01195	1.027898	0.046575	-0.20292	0.22683	TRUE	1.00E+00
39917_at	0.01197	1.027945	0.009256	-0.03073	0.05468	TRUE	1.00E+00
38737_at	0.01199	1.027993	0.154224	-0.69954	0.72351	TRUE	1.00E+00
38534_at	0.012	1.028016	0.019061	-0.07594	0.09994	TRUE	1.00E+00
41586_at	0.01201	1.02804	0.040092	-0.17296	0.19697	TRUE	1.00E+00
39197_s_at	0.01201	1.02804	0.028241	-0.11828	0.1423	TRUE	1.00E+00
36322_at	0.01204	1.028111	0.019197	-0.07653	0.1006	TRUE	1.00E+00
39231_at	0.01209	1.028229	0.019348	-0.07718	0.10135	TRUE	1.00E+00
39272_g_at	0.0121	1.028253	0.016341	-0.06329	0.08749	TRUE	1.00E+00
35887_at	0.0121	1.028253	0.04223	-0.18273	0.20693	TRUE	1.00E+00
38569_at	0.01215	1.028371	0.040502	-0.17471	0.19901	TRUE	1.00E+00
33394_at	0.01217	1.028419	0.015777	-0.06062	0.08495	TRUE	1.00E+00
33873_at	0.0123	1.028727	0.00571	-0.01404	0.03864	TRUE	1.00E+00
41661_at	0.0123	1.028727	0.08188	-0.36546	0.39007	TRUE	1.00E+00
34096_at	0.01234	1.028821	0.119068	-0.53699	0.56167	TRUE	1.00E+00
36439_at	0.01238	1.028916	0.041826	-0.18059	0.20535	TRUE	1.00E+00
41277_at	0.01241	1.028987	0.04403	-0.19073	0.21555	TRUE	1.00E+00
35225_at	0.01242	1.029011	0.032769	-0.13876	0.16361	TRUE	1.00E+00
31592_at	0.01244	1.029058	0.061971	-0.27346	0.29835	TRUE	1.00E+00
35308_at	0.01248	1.029153	0.01918	-0.07601	0.10097	TRUE	1.00E+00
2064_g_at	0.01252	1.029248	0.023171	-0.09438	0.11942	TRUE	1.00E+00
2087_s_at	0.01253	1.029272	0.029498	-0.12356	0.14862	TRUE	1.00E+00
33107_at	0.01254	1.029295	0.034153	-0.14503	0.1701	TRUE	1.00E+00
996_at	0.01254	1.029295	0.074324	-0.33036	0.35545	TRUE	1.00E+00
36577_at	0.01258	1.02939	0.015571	-0.05926	0.08442	TRUE	1.00E+00
1698_g_at	0.01258	1.02939	0.024916	-0.10237	0.12754	TRUE	1.00E+00
38089_at	0.01259	1.029414	0.032795	-0.13871	0.16389	TRUE	1.00E+00
38055_at	0.01265	1.029556	0.024816	-0.10184	0.12714	TRUE	1.00E+00
37438_at	0.01271	1.029698	0.044711	-0.19357	0.21899	TRUE	1.00E+00
37752_at	0.01273	1.029746	0.009904	-0.03296	0.05843	TRUE	1.00E+00
32301_at	0.01277	1.029841	0.034928	-0.14838	0.17391	TRUE	1.00E+00
922_at	0.0129	1.030149	0.017441	-0.06756	0.09337	TRUE	1.00E+00
172_at	0.01293	1.03022	0.01897	-0.07459	0.10045	TRUE	1.00E+00
39858_s_at	0.01296	1.030291	0.049441	-0.21514	0.24106	TRUE	1.00E+00
37240_at	0.01297	1.030315	0.018539	-0.07256	0.0985	TRUE	1.00E+00
37691_at	0.01301	1.03041	0.024667	-0.1008	0.12681	TRUE	1.00E+00
36628_at	0.01304	1.030481	0.007608	-0.02207	0.04814	TRUE	1.00E+00

35521_at	0.01304	1.030481	0.051522	-0.22466	0.25074	TRUE	1.00E+00
34661_at	0.01307	1.030552	0.026078	-0.10725	0.13338	TRUE	1.00E+00
40222_s_at	0.01309	1.0306	0.008803	-0.02752	0.0537	TRUE	1.00E+00
37281_at	0.01309	1.0306	0.019271	-0.07582	0.102	TRUE	1.00E+00
31976_at	0.01318	1.030813	0.113215	-0.50915	0.53551	TRUE	1.00E+00
38813_at	0.01318	1.030813	0.016051	-0.06087	0.08723	TRUE	1.00E+00
1953_at	0.01324	1.030956	0.036439	-0.15488	0.18135	TRUE	1.00E+00
1555_f_at	0.01325	1.030979	0.032452	-0.13647	0.16297	TRUE	1.00E+00
32942_at	0.01327	1.031027	0.036722	-0.15615	0.18269	TRUE	1.00E+00
1789_at	0.01327	1.031027	0.004864	-0.00917	0.03571	TRUE	1.00E+00
283_at	0.01328	1.031051	0.015126	-0.05651	0.08307	TRUE	1.00E+00
39180_at	0.01329	1.031074	0.026387	-0.10845	0.13503	TRUE	1.00E+00
1222_at	0.01332	1.031146	0.017572	-0.06775	0.09439	TRUE	1.00E+00
37843_i_at	0.01334	1.031193	0.06054	-0.26597	0.29265	TRUE	1.00E+00
36813_at	0.01335	1.031217	0.077196	-0.3428	0.3695	TRUE	1.00E+00
35875_at	0.01338	1.031288	0.052246	-0.22766	0.25442	TRUE	1.00E+00
40048_at	0.0134	1.031336	0.016925	-0.06468	0.09149	TRUE	1.00E+00
36953_at	0.01343	1.031407	0.017126	-0.06558	0.09244	TRUE	1.00E+00
34337_s_at	0.01345	1.031454	0.023155	-0.09338	0.12028	TRUE	1.00E+00
35615_at	0.01345	1.031454	0.025703	-0.10513	0.13204	TRUE	1.00E+00
32496_at	0.01345	1.031454	0.072716	-0.32203	0.34894	TRUE	1.00E+00
31865_at	0.01346	1.031478	0.055367	-0.24198	0.2689	TRUE	1.00E+00
1199_at	0.01347	1.031502	0.021224	-0.08445	0.11139	TRUE	1.00E+00
1180_g_at	0.01347	1.031502	0.021266	-0.08464	0.11159	TRUE	1.00E+00
38744_at	0.01352	1.031621	0.033339	-0.14029	0.16734	TRUE	1.00E+00
40384_at	0.01355	1.031692	0.033145	-0.13937	0.16647	TRUE	1.00E+00
37640_at	0.01355	1.031692	0.014	-0.05104	0.07814	TRUE	1.00E+00
36339_at	0.01356	1.031716	0.054226	-0.23662	0.26374	TRUE	1.00E+00
37480_at	0.01357	1.031739	0.076457	-0.33917	0.36631	TRUE	1.00E+00
32352_at	0.01359	1.031787	0.019169	-0.07485	0.10203	TRUE	1.00E+00
41474_at	0.01362	1.031858	0.028572	-0.1182	0.14544	TRUE	1.00E+00
32758_g_at	0.01364	1.031906	0.029985	-0.1247	0.15197	TRUE	1.00E+00
2023_g_at	0.01367	1.031977	0.042953	-0.1845	0.21184	TRUE	1.00E+00
34464_at	0.01369	1.032024	0.102699	-0.46012	0.4875	TRUE	1.00E+00
37339_at	0.01371	1.032072	0.029514	-0.12246	0.14987	TRUE	1.00E+00
1092_at	0.01374	1.032143	0.034885	-0.1472	0.17469	TRUE	1.00E+00
37278_at	0.01376	1.032191	0.020938	-0.08284	0.11036	TRUE	1.00E+00
39831_at	0.01381	1.03231	0.029012	-0.12003	0.14766	TRUE	1.00E+00
33086_at	0.01382	1.032333	0.07589	-0.3363	0.36395	TRUE	1.00E+00
613_at	0.01383	1.032357	0.061152	-0.2683	0.29596	TRUE	1.00E+00
1432_s_at	0.01384	1.032381	0.056589	-0.24724	0.27492	TRUE	1.00E+00
35928_at	0.01386	1.032429	0.036093	-0.15265	0.18038	TRUE	1.00E+00
32769_at	0.01387	1.032452	0.014481	-0.05294	0.08068	TRUE	1.00E+00
34070_s_at	0.01388	1.032476	0.036537	-0.15468	0.18245	TRUE	1.00E+00
698_f_at	0.01394	1.032619	0.021628	-0.08585	0.11372	TRUE	1.00E+00
38387_at	0.01395	1.032643	0.034051	-0.14315	0.17104	TRUE	1.00E+00
35742_at	0.01395	1.032643	0.038057	-0.16162	0.18953	TRUE	1.00E+00
264_at	0.01399	1.032738	0.026576	-0.10862	0.1366	TRUE	1.00E+00
38308_g_at	0.01399	1.032738	0.02685	-0.10988	0.13786	TRUE	1.00E+00
32043_at	0.01401	1.032785	0.028654	-0.11819	0.14621	TRUE	1.00E+00
32776_at	0.01403	1.032833	0.014828	-0.05437	0.08244	TRUE	1.00E+00

36492_at	0.01409	1.032975	0.037277	-0.15789	0.18607	TRUE	1.00E+00
38253_at	0.01411	1.033023	0.022999	-0.092	0.12022	TRUE	1.00E+00
36865_at	0.01411	1.033023	0.029919	-0.12392	0.15215	TRUE	1.00E+00
35628_at	0.01412	1.033047	0.027434	-0.11244	0.14069	TRUE	1.00E+00
33683_at	0.01416	1.033142	0.014008	-0.05046	0.07879	TRUE	1.00E+00
38718_at	0.01417	1.033166	0.015712	-0.05832	0.08666	TRUE	1.00E+00
34099_f_at	0.01417	1.033166	0.01952	-0.07589	0.10423	TRUE	1.00E+00
38229_at	0.01418	1.03319	0.067496	-0.29722	0.32558	TRUE	1.00E+00
35105_at	0.0142	1.033237	0.06249	-0.2741	0.30251	TRUE	1.00E+00
39217_at	0.01423	1.033308	0.053005	-0.23031	0.25878	TRUE	1.00E+00
33008_at	0.01424	1.033332	0.019826	-0.07723	0.10571	TRUE	1.00E+00
38097_at	0.01427	1.033404	0.035987	-0.15176	0.18029	TRUE	1.00E+00
36361_at	0.01432	1.033523	0.022343	-0.08876	0.1174	TRUE	1.00E+00
40790_at	0.01433	1.033546	0.036829	-0.15558	0.18425	TRUE	1.00E+00
36832_at	0.01434	1.03357	0.098129	-0.43839	0.46706	TRUE	1.00E+00
35468_at	0.01434	1.03357	0.033823	-0.1417	0.17039	TRUE	1.00E+00
32910_at	0.01441	1.033737	0.063495	-0.27853	0.30735	TRUE	1.00E+00
40743_at	0.01441	1.033737	0.109302	-0.48986	0.51869	TRUE	1.00E+00
38612_at	0.01445	1.033832	0.033202	-0.13873	0.16762	TRUE	1.00E+00
502_s_at	0.01446	1.033856	0.036974	-0.15613	0.18504	TRUE	1.00E+00
37031_at	0.01448	1.033903	0.018712	-0.07185	0.10081	TRUE	1.00E+00
38480_s_at	0.01448	1.033903	0.017259	-0.06514	0.09411	TRUE	1.00E+00
36351_at	0.0145	1.033951	0.079082	-0.35035	0.37935	TRUE	1.00E+00
33234_at	0.0145	1.033951	0.019425	-0.07512	0.10412	TRUE	1.00E+00
39274_at	0.01456	1.034094	0.022124	-0.08751	0.11663	TRUE	1.00E+00
32169_at	0.0146	1.034189	0.01428	-0.05129	0.08048	TRUE	1.00E+00
39928_at	0.01461	1.034213	0.03338	-0.1394	0.16861	TRUE	1.00E+00
40277_at	0.01463	1.034261	0.06023	-0.26324	0.29251	TRUE	1.00E+00
39790_at	0.01466	1.034332	0.028365	-0.11621	0.14552	TRUE	1.00E+00
38477_at	0.01466	1.034332	0.031241	-0.12947	0.1588	TRUE	1.00E+00
36250_at	0.01468	1.03438	0.019772	-0.07654	0.1059	TRUE	1.00E+00
32994_at	0.0147	1.034427	0.125243	-0.56312	0.59252	TRUE	1.00E+00
32341_f_at	0.01471	1.034451	0.018216	-0.06934	0.09875	TRUE	1.00E+00
39009_at	0.01472	1.034475	0.022152	-0.08747	0.11692	TRUE	1.00E+00
38620_at	0.01477	1.034594	0.015842	-0.05832	0.08786	TRUE	1.00E+00
38814_at	0.0148	1.034666	0.023156	-0.09203	0.12163	TRUE	1.00E+00
39147_g_at	0.01481	1.034689	0.02455	-0.09846	0.12807	TRUE	1.00E+00
39128_r_at	0.01485	1.034785	0.046918	-0.20161	0.23131	TRUE	1.00E+00
737_at	0.01486	1.034809	0.044115	-0.18867	0.21839	TRUE	1.00E+00
37770_at	0.01488	1.034856	0.015023	-0.05443	0.08419	TRUE	1.00E+00
37312_at	0.01489	1.03488	0.024419	-0.09777	0.12755	TRUE	1.00E+00
33939_at	0.01491	1.034928	0.077049	-0.34057	0.37038	TRUE	1.00E+00
36515_at	0.01492	1.034952	0.022964	-0.09103	0.12087	TRUE	1.00E+00
36948_at	0.01493	1.034975	0.027858	-0.1136	0.14345	TRUE	1.00E+00
41806_at	0.01496	1.035047	0.023478	-0.09335	0.12328	TRUE	1.00E+00
41787_at	0.01497	1.035071	0.04657	-0.19988	0.22983	TRUE	1.00E+00
34596_at	0.01498	1.035094	0.078197	-0.34579	0.37574	TRUE	1.00E+00
1245_i_at	0.015	1.035142	0.064956	-0.28468	0.31468	TRUE	1.00E+00
40271_at	0.01501	1.035166	0.015177	-0.05501	0.08503	TRUE	1.00E+00
40261_at	0.01503	1.035214	0.031457	-0.1301	0.16016	TRUE	1.00E+00
543_g_at	0.01513	1.035452	0.08575	-0.38048	0.41074	TRUE	1.00E+00

36426_g_at	0.01518	1.035571	0.032759	-0.13595	0.16632	TRUE	1.00E+00
33941_at	0.01519	1.035595	0.112365	-0.50322	0.5336	TRUE	1.00E+00
35183_at	0.0152	1.035619	0.035197	-0.14718	0.17759	TRUE	1.00E+00
38394_at	0.01521	1.035643	0.025965	-0.10458	0.135	TRUE	1.00E+00
39469_s_at	0.01522	1.035667	0.060327	-0.2631	0.29354	TRUE	1.00E+00
32253_at	0.01534	1.035953	0.023089	-0.09118	0.12186	TRUE	1.00E+00
33891_at	0.01537	1.036024	0.020233	-0.07798	0.10871	TRUE	1.00E+00
827_s_at	0.01539	1.036072	0.093233	-0.41475	0.44553	TRUE	1.00E+00
36155_at	0.01539	1.036072	0.028234	-0.11487	0.14565	TRUE	1.00E+00
41649_at	0.0154	1.036096	0.044077	-0.18796	0.21875	TRUE	1.00E+00
35387_r_at	0.01545	1.036215	0.030955	-0.12737	0.15826	TRUE	1.00E+00
33541_s_at	0.01547	1.036263	0.080052	-0.35386	0.3848	TRUE	1.00E+00
38688_at	0.01558	1.036526	0.018055	-0.06772	0.09888	TRUE	1.00E+00
2007_g_at	0.01558	1.036526	0.046096	-0.19708	0.22825	TRUE	1.00E+00
33819_at	0.01559	1.036549	0.01272	-0.0431	0.07427	TRUE	1.00E+00
34144_at	0.01564	1.036669	0.054853	-0.23743	0.26871	TRUE	1.00E+00
37306_at	0.0157	1.036812	0.008268	-0.02245	0.05384	TRUE	1.00E+00
36535_at	0.0157	1.036812	0.016963	-0.06256	0.09397	TRUE	1.00E+00
40986_s_at	0.01571	1.036836	0.022037	-0.08596	0.11738	TRUE	1.00E+00
37897_s_at	0.01573	1.036884	0.01896	-0.07174	0.10321	TRUE	1.00E+00
41555_at	0.01576	1.036955	0.046347	-0.19807	0.22958	TRUE	1.00E+00
36509_at	0.01576	1.036955	0.027435	-0.11081	0.14233	TRUE	1.00E+00
39381_at	0.01576	1.036955	0.02778	-0.1124	0.14392	TRUE	1.00E+00
40732_at	0.01576	1.036955	0.020242	-0.07763	0.10915	TRUE	1.00E+00
592_at	0.01578	1.037003	0.013735	-0.04758	0.07915	TRUE	1.00E+00
1691_at	0.01579	1.037027	0.072622	-0.31926	0.35084	TRUE	1.00E+00
40419_at	0.01588	1.037242	0.031152	-0.12784	0.1596	TRUE	1.00E+00
38581_at	0.01592	1.037337	0.02945	-0.11995	0.15179	TRUE	1.00E+00
1781_at	0.01596	1.037433	0.029003	-0.11785	0.14977	TRUE	1.00E+00
41618_at	0.01606	1.037672	0.059698	-0.25937	0.29148	TRUE	1.00E+00
35088_at	0.01608	1.03772	0.087982	-0.38983	0.422	TRUE	1.00E+00
34707_at	0.01611	1.037791	0.025793	-0.10288	0.13511	TRUE	1.00E+00
528_at	0.01613	1.037839	0.049093	-0.21037	0.24262	TRUE	1.00E+00
34346_at	0.01616	1.037911	0.02519	-0.10006	0.13237	TRUE	1.00E+00
41130_at	0.01619	1.037982	0.015806	-0.05673	0.08911	TRUE	1.00E+00
37745_s_at	0.0162	1.038006	0.045569	-0.19403	0.22644	TRUE	1.00E+00
171_at	0.01625	1.038126	0.019305	-0.07282	0.10531	TRUE	1.00E+00
160036_at	0.01627	1.038174	0.043728	-0.18547	0.21802	TRUE	1.00E+00
37049_g_at	0.01628	1.038198	0.010921	-0.0341	0.06666	TRUE	1.00E+00
31548_at	0.01629	1.038221	0.100583	-0.44776	0.48034	TRUE	1.00E+00
38008_at	0.01634	1.038341	0.032602	-0.13407	0.16675	TRUE	1.00E+00
40421_at	0.01635	1.038365	0.012998	-0.04362	0.07631	TRUE	1.00E+00
33698_at	0.01642	1.038532	0.049419	-0.21158	0.24442	TRUE	1.00E+00
1528_at	0.01646	1.038628	0.025154	-0.09959	0.13251	TRUE	1.00E+00
35158_at	0.01647	1.038652	0.088284	-0.39084	0.42377	TRUE	1.00E+00
35851_g_at	0.01649	1.0387	0.091575	-0.406	0.43899	TRUE	1.00E+00
1074_at	0.0165	1.038724	0.013129	-0.04408	0.07707	TRUE	1.00E+00
1207_at	0.01651	1.038748	0.035505	-0.14729	0.18032	TRUE	1.00E+00
31558_at	0.01652	1.038771	0.049799	-0.21323	0.24627	TRUE	1.00E+00
32823_at	0.0166	1.038963	0.024508	-0.09647	0.12967	TRUE	1.00E+00
32223_at	0.01664	1.039059	0.011334	-0.03565	0.06894	TRUE	1.00E+00

32953_at	0.01666	1.039106	0.056956	-0.24611	0.27943	TRUE	1.00E+00
40824_at	0.0167	1.039202	0.012069	-0.03899	0.07238	TRUE	1.00E+00
32887_at	0.01672	1.03925	0.066752	-0.29125	0.32469	TRUE	1.00E+00
39722_at	0.01672	1.03925	0.024351	-0.09562	0.12907	TRUE	1.00E+00
40150_at	0.01676	1.039346	0.026852	-0.10713	0.14064	TRUE	1.00E+00
39149_at	0.01682	1.039489	0.024676	-0.09703	0.13066	TRUE	1.00E+00
32054_at	0.01682	1.039489	0.038417	-0.16042	0.19406	TRUE	1.00E+00
33099_at	0.01682	1.039489	0.030422	-0.12353	0.15717	TRUE	1.00E+00
35571_at	0.01684	1.039537	0.032397	-0.13263	0.1663	TRUE	1.00E+00
38979_at	0.01686	1.039585	0.034602	-0.14278	0.1765	TRUE	1.00E+00
34867_at	0.01688	1.039633	0.092066	-0.40787	0.44164	TRUE	1.00E+00
34814_at	0.0169	1.039681	0.016556	-0.05949	0.09328	TRUE	1.00E+00
39126_at	0.01691	1.039705	0.019941	-0.0751	0.10891	TRUE	1.00E+00
36443_at	0.01691	1.039705	0.060949	-0.26428	0.29811	TRUE	1.00E+00
1497_at	0.01701	1.039944	0.019165	-0.07141	0.10543	TRUE	1.00E+00
38317_at	0.01702	1.039968	0.024481	-0.09593	0.12996	TRUE	1.00E+00
41411_at	0.01707	1.040088	0.010956	-0.03348	0.06761	TRUE	1.00E+00
39068_at	0.01707	1.040088	0.014719	-0.05084	0.08497	TRUE	1.00E+00
37046_at	0.01709	1.040136	0.013448	-0.04496	0.07913	TRUE	1.00E+00
37915_at	0.01711	1.040184	0.040771	-0.17099	0.20521	TRUE	1.00E+00
34952_at	0.01713	1.040231	0.029318	-0.11813	0.15239	TRUE	1.00E+00
41465_at	0.01714	1.040255	0.016703	-0.05992	0.09421	TRUE	1.00E+00
32862_at	0.01719	1.040375	0.071491	-0.31264	0.34702	TRUE	1.00E+00
32222_at	0.01723	1.040471	0.007132	-0.01567	0.05014	TRUE	1.00E+00
36910_at	0.01724	1.040495	0.031944	-0.13014	0.16462	TRUE	1.00E+00
33451_s_at	0.01729	1.040615	0.026661	-0.10572	0.14029	TRUE	1.00E+00
33333_at	0.01738	1.04083	0.033522	-0.13728	0.17203	TRUE	1.00E+00
40105_at	0.01738	1.04083	0.02411	-0.09385	0.12862	TRUE	1.00E+00
1636_g_at	0.0174	1.040878	0.028905	-0.11595	0.15076	TRUE	1.00E+00
1969_s_at	0.01742	1.040926	0.017167	-0.06178	0.09662	TRUE	1.00E+00
33688_at	0.01743	1.04095	0.097666	-0.43316	0.46801	TRUE	1.00E+00
36976_at	0.01748	1.04107	0.024897	-0.09738	0.13234	TRUE	1.00E+00
39560_at	0.01749	1.041094	0.02361	-0.09143	0.12642	TRUE	1.00E+00
36841_at	0.01754	1.041214	0.029682	-0.1194	0.15448	TRUE	1.00E+00
35754_at	0.0176	1.041358	0.008823	-0.02311	0.0583	TRUE	1.00E+00
31417_at	0.01761	1.041382	0.017086	-0.06121	0.09644	TRUE	1.00E+00
33579_i_at	0.01764	1.041454	0.033282	-0.13591	0.17119	TRUE	1.00E+00
affx-dapx-5_at	0.01765	1.041478	0.066202	-0.28778	0.32308	TRUE	1.00E+00
40829_at	0.01765	1.041478	0.027088	-0.10732	0.14263	TRUE	1.00E+00
41268_g_at	0.01766	1.041502	0.025846	-0.10159	0.1369	TRUE	1.00E+00
38956_at	0.01768	1.04155	0.074314	-0.32518	0.36053	TRUE	1.00E+00
34554_at	0.01769	1.041574	0.055117	-0.2366	0.27197	TRUE	1.00E+00
41167_at	0.0177	1.041598	0.018306	-0.06675	0.10216	TRUE	1.00E+00
37982_at	0.01772	1.041646	0.019249	-0.07108	0.10653	TRUE	1.00E+00
37597_s_at	0.01774	1.041694	0.02037	-0.07624	0.11172	TRUE	1.00E+00
31413_at	0.01775	1.041718	0.030594	-0.1234	0.15889	TRUE	1.00E+00
38020_at	0.01776	1.041742	0.039732	-0.16555	0.20106	TRUE	1.00E+00
2041_i_at	0.01777	1.041766	0.038322	-0.15903	0.19457	TRUE	1.00E+00
174_s_at	0.01778	1.04179	0.022782	-0.08732	0.12289	TRUE	1.00E+00
1084_at	0.0178	1.041838	0.019006	-0.06988	0.10548	TRUE	1.00E+00
31706_at	0.01787	1.042005	0.035099	-0.14406	0.1798	TRUE	1.00E+00

41113_at	0.0179	1.042077	0.031533	-0.12758	0.16338	TRUE	1.00E+00
36145_at	0.01795	1.042197	0.018636	-0.06803	0.10393	TRUE	1.00E+00
39971_at	0.01797	1.042245	0.035833	-0.14735	0.18329	TRUE	1.00E+00
39342_at	0.01798	1.042269	0.009089	-0.02396	0.05991	TRUE	1.00E+00
41425_at	0.01799	1.042293	0.021318	-0.08036	0.11634	TRUE	1.00E+00
37285_at	0.01801	1.042341	0.028345	-0.11276	0.14878	TRUE	1.00E+00
39064_at	0.01809	1.042533	0.033344	-0.13575	0.17193	TRUE	1.00E+00
39571_at	0.01809	1.042533	0.023468	-0.09018	0.12636	TRUE	1.00E+00
35358_at	0.0181	1.042557	0.090025	-0.39723	0.43344	TRUE	1.00E+00
39095_at	0.01817	1.042726	0.023496	-0.09023	0.12656	TRUE	1.00E+00
36153_at	0.0182	1.042798	0.023182	-0.08876	0.12515	TRUE	1.00E+00
32437_at	0.01821	1.042822	0.015174	-0.0518	0.08822	TRUE	1.00E+00
31715_at	0.01823	1.04287	0.102604	-0.45514	0.4916	TRUE	1.00E+00
39557_at	0.01825	1.042918	0.041232	-0.17197	0.20848	TRUE	1.00E+00
34266_at	0.01827	1.042966	0.081543	-0.35794	0.39448	TRUE	1.00E+00
41441_at	0.0183	1.043038	0.012228	-0.03812	0.07471	TRUE	1.00E+00
34386_at	0.0183	1.043038	0.031108	-0.12522	0.16182	TRUE	1.00E+00
39723_at	0.01832	1.043086	0.02136	-0.08023	0.11686	TRUE	1.00E+00
31905_at	0.01832	1.043086	0.045416	-0.19121	0.22785	TRUE	1.00E+00
491_at	0.01833	1.04311	0.024838	-0.09626	0.13292	TRUE	1.00E+00
39905_i_at	0.01833	1.04311	0.03789	-0.15648	0.19314	TRUE	1.00E+00
38695_at	0.01834	1.043134	0.012431	-0.03901	0.07569	TRUE	1.00E+00
33668_at	0.01836	1.043182	0.025614	-0.09981	0.13653	TRUE	1.00E+00
38877_s_at	0.01838	1.04323	0.056384	-0.24175	0.27851	TRUE	1.00E+00
32540_at	0.01839	1.043254	0.041948	-0.17514	0.21192	TRUE	1.00E+00
1280_i_at	0.01847	1.043446	0.043506	-0.18225	0.21919	TRUE	1.00E+00
31359_at	0.01847	1.043446	0.080949	-0.355	0.39194	TRUE	1.00E+00
32752_at	0.01849	1.043494	0.021484	-0.08063	0.11761	TRUE	1.00E+00
41313_at	0.01852	1.043566	0.026079	-0.10179	0.13884	TRUE	1.00E+00
39540_at	0.01856	1.043662	0.111369	-0.49525	0.53237	TRUE	1.00E+00
36143_at	0.01858	1.04371	0.029314	-0.11666	0.15382	TRUE	1.00E+00
35744_at	0.01858	1.04371	0.020015	-0.07376	0.11092	TRUE	1.00E+00
1865_at	0.01861	1.043782	0.115819	-0.51574	0.55295	TRUE	1.00E+00
1108_s_at	0.01863	1.043831	0.042789	-0.17878	0.21604	TRUE	1.00E+00
41159_at	0.01864	1.043855	0.026013	-0.10137	0.13865	TRUE	1.00E+00
32170_g_at	0.01867	1.043927	0.016581	-0.05782	0.09517	TRUE	1.00E+00
285_g_at	0.01868	1.043951	0.066941	-0.29015	0.32752	TRUE	1.00E+00
32099_at	0.0187	1.043999	0.015313	-0.05195	0.08935	TRUE	1.00E+00
39307_s_at	0.01874	1.044095	0.015356	-0.0521	0.08959	TRUE	1.00E+00
34544_at	0.01876	1.044143	0.021644	-0.0811	0.11862	TRUE	1.00E+00
31590_g_at	0.01878	1.044191	0.02173	-0.08147	0.11904	TRUE	1.00E+00
40611_s_at	0.01879	1.044215	0.088256	-0.38838	0.42597	TRUE	1.00E+00
34687_at	0.0188	1.044239	0.02666	-0.1042	0.1418	TRUE	1.00E+00
33666_at	0.01881	1.044263	0.018846	-0.06813	0.10576	TRUE	1.00E+00
37862_at	0.01889	1.044456	0.0891	-0.39218	0.42996	TRUE	1.00E+00
34833_at	0.01891	1.044504	0.021974	-0.08247	0.12029	TRUE	1.00E+00
36923_at	0.01896	1.044624	0.019741	-0.07211	0.11004	TRUE	1.00E+00
35160_at	0.01898	1.044672	0.022323	-0.08401	0.12197	TRUE	1.00E+00
2052_g_at	0.019	1.04472	0.057817	-0.24774	0.28575	TRUE	1.00E+00
41283_at	0.01901	1.044744	0.016866	-0.0588	0.09683	TRUE	1.00E+00
31439_f_at	0.01902	1.044768	0.023314	-0.08854	0.12659	TRUE	1.00E+00

41510_s_at	0.01905	1.044841	0.016845	-0.05867	0.09676	TRUE	1.00E+00
36510_at	0.01908	1.044913	0.030011	-0.11938	0.15753	TRUE	1.00E+00
39023_at	0.01908	1.044913	0.029713	-0.11801	0.15616	TRUE	1.00E+00
35682_at	0.01911	1.044985	0.033506	-0.13547	0.1737	TRUE	1.00E+00
39032_at	0.01913	1.045033	0.024289	-0.09293	0.13119	TRUE	1.00E+00
34720_at	0.01913	1.045033	0.038771	-0.15974	0.19801	TRUE	1.00E+00
34890_at	0.01916	1.045105	0.012456	-0.03831	0.07663	TRUE	1.00E+00
37007_at	0.01916	1.045105	0.011706	-0.03485	0.07317	TRUE	1.00E+00
40293_at	0.01916	1.045105	0.059399	-0.25488	0.2932	TRUE	1.00E+00
39033_at	0.01917	1.045129	0.014718	-0.04873	0.08708	TRUE	1.00E+00
39830_at	0.01921	1.045226	0.020492	-0.07533	0.11376	TRUE	1.00E+00
32879_at	0.01922	1.04525	0.102363	-0.45304	0.49148	TRUE	1.00E+00
32772_s_at	0.01926	1.045346	0.044229	-0.1848	0.22332	TRUE	1.00E+00
33657_at	0.01928	1.045394	0.021156	-0.07832	0.11688	TRUE	1.00E+00
41422_at	0.01928	1.045394	0.089299	-0.39271	0.43127	TRUE	1.00E+00
33368_at	0.01931	1.045466	0.022815	-0.08594	0.12457	TRUE	1.00E+00
31637_s_at	0.01933	1.045514	0.040663	-0.16827	0.20694	TRUE	1.00E+00
39006_r_at	0.01934	1.045538	0.073457	-0.31956	0.35824	TRUE	1.00E+00
34895_at	0.01941	1.045707	0.01846	-0.06576	0.10458	TRUE	1.00E+00
1212_at	0.01948	1.045876	0.050939	-0.21554	0.25449	TRUE	1.00E+00
1874_at	0.01951	1.045948	0.011395	-0.03306	0.07208	TRUE	1.00E+00
32074_at	0.01954	1.04602	0.032884	-0.13217	0.17125	TRUE	1.00E+00
33952_at	0.01954	1.04602	0.038766	-0.15931	0.19839	TRUE	1.00E+00
37642_at	0.01964	1.046261	0.016651	-0.05718	0.09646	TRUE	1.00E+00
40139_at	0.01965	1.046285	0.021631	-0.08014	0.11945	TRUE	1.00E+00
134_at	0.01966	1.046309	0.029342	-0.11571	0.15504	TRUE	1.00E+00
409_at	0.01967	1.046333	0.010625	-0.02935	0.06869	TRUE	1.00E+00
31739_at	0.01969	1.046381	0.052837	-0.22408	0.26346	TRUE	1.00E+00
35683_at	0.01971	1.04643	0.039271	-0.16147	0.20089	TRUE	1.00E+00
33025_at	0.01971	1.04643	0.021538	-0.07966	0.11908	TRUE	1.00E+00
33740_at	0.01972	1.046454	0.009644	-0.02477	0.06421	TRUE	1.00E+00
35010_at	0.01977	1.046574	0.036494	-0.1486	0.18813	TRUE	1.00E+00
41185_f_at	0.01982	1.046695	0.019963	-0.07228	0.11193	TRUE	1.00E+00
32526_at	0.01983	1.046719	0.041263	-0.17054	0.2102	TRUE	1.00E+00
39626_s_at	0.01984	1.046743	0.087523	-0.38396	0.42363	TRUE	1.00E+00
35369_at	0.01991	1.046912	0.026633	-0.10297	0.14278	TRUE	1.00E+00
34089_at	0.01991	1.046912	0.019272	-0.069	0.10882	TRUE	1.00E+00
1499_at	0.01994	1.046984	0.01945	-0.06979	0.10967	TRUE	1.00E+00
41379_at	0.01995	1.047008	0.024496	-0.09307	0.13296	TRUE	1.00E+00
1255_g_at	0.01995	1.047008	0.048422	-0.20345	0.24335	TRUE	1.00E+00
39867_at	0.01995	1.047008	0.01273	-0.03878	0.07868	TRUE	1.00E+00
38344_at	0.01996	1.047032	0.046576	-0.19492	0.23484	TRUE	1.00E+00
36996_at	0.01998	1.04708	0.00912	-0.0221	0.06205	TRUE	1.00E+00
1573_at	0.02001	1.047153	0.107878	-0.4777	0.51771	TRUE	1.00E+00
34881_at	0.02009	1.047346	0.087551	-0.38383	0.42402	TRUE	1.00E+00
1609_g_at	0.02011	1.047394	0.046184	-0.19296	0.23319	TRUE	1.00E+00
37269_at	0.02012	1.047418	0.030602	-0.12106	0.16131	TRUE	1.00E+00
39568_g_at	0.02014	1.047466	0.020053	-0.07238	0.11265	TRUE	1.00E+00
36822_at	0.02019	1.047587	0.024939	-0.09487	0.13524	TRUE	1.00E+00
1665_s_at	0.02023	1.047683	0.026408	-0.10161	0.14207	TRUE	1.00E+00
32424_at	0.02025	1.047731	0.03579	-0.14487	0.18537	TRUE	1.00E+00

37304_at	0.02026	1.047756	0.013852	-0.04364	0.08417	TRUE	1.00E+00
41443_at	0.0203	1.047852	0.01903	-0.0675	0.10809	TRUE	1.00E+00
34360_s_at	0.0203	1.047852	0.038174	-0.15582	0.19642	TRUE	1.00E+00
35798_at	0.0203	1.047852	0.035838	-0.14504	0.18565	TRUE	1.00E+00
757_at	0.02031	1.047876	0.043162	-0.17882	0.21945	TRUE	1.00E+00
891_at	0.02032	1.0479	0.013994	-0.04425	0.08488	TRUE	1.00E+00
35977_at	0.02033	1.047925	0.052533	-0.22204	0.26269	TRUE	1.00E+00
35603_at	0.02036	1.047997	0.061782	-0.26468	0.30539	TRUE	1.00E+00
39208_i_at	0.02036	1.047997	0.130862	-0.58338	0.6241	TRUE	1.00E+00
41873_at	0.02037	1.048021	0.083719	-0.36588	0.40661	TRUE	1.00E+00
32785_at	0.02038	1.048045	0.012653	-0.038	0.07875	TRUE	1.00E+00
32819_at	0.02038	1.048045	0.060782	-0.26004	0.3008	TRUE	1.00E+00
38242_at	0.02041	1.048118	0.039556	-0.16208	0.2029	TRUE	1.00E+00
1291_s_at	0.02053	1.048407	0.037026	-0.15029	0.19135	TRUE	1.00E+00
35746_r_at	0.02053	1.048407	0.009762	-0.0245	0.06557	TRUE	1.00E+00
40602_at	0.02053	1.048407	0.021614	-0.07919	0.12025	TRUE	1.00E+00
41291_at	0.02056	1.04848	0.047667	-0.19935	0.24048	TRUE	1.00E+00
33052_at	0.02058	1.048528	0.033015	-0.13174	0.1729	TRUE	1.00E+00
37146_at	0.02058	1.048528	0.02362	-0.08839	0.12956	TRUE	1.00E+00
35483_at	0.02064	1.048673	0.05224	-0.22037	0.26165	TRUE	1.00E+00
32548_at	0.02067	1.048745	0.017008	-0.0578	0.09914	TRUE	1.00E+00
35368_at	0.02069	1.048794	0.021398	-0.07803	0.11942	TRUE	1.00E+00
34931_at	0.02078	1.049011	0.022469	-0.08288	0.12444	TRUE	1.00E+00
612_s_at	0.02081	1.049083	0.011493	-0.03221	0.07384	TRUE	1.00E+00
36079_at	0.02082	1.049108	0.03582	-0.14444	0.18608	TRUE	1.00E+00
35918_at	0.02082	1.049108	0.06653	-0.28612	0.32776	TRUE	1.00E+00
31574_i_at	0.02085	1.04918	0.046124	-0.19195	0.23364	TRUE	1.00E+00
34791_at	0.02086	1.049204	0.021624	-0.0789	0.12063	TRUE	1.00E+00
39844_at	0.02088	1.049252	0.021131	-0.07662	0.11837	TRUE	1.00E+00
1910_s_at	0.02088	1.049252	0.036586	-0.14791	0.18968	TRUE	1.00E+00
36277_at	0.02089	1.049277	0.025274	-0.09571	0.1375	TRUE	1.00E+00
35916_s_at	0.02092	1.049349	0.04595	-0.19107	0.23292	TRUE	1.00E+00
40715_at	0.02095	1.049422	0.042383	-0.17459	0.21649	TRUE	1.00E+00
33094_s_at	0.02099	1.049518	0.055149	-0.23344	0.27543	TRUE	1.00E+00
39350_at	0.021	1.049542	0.068793	-0.29638	0.33839	TRUE	1.00E+00
31376_at	0.02105	1.049663	0.085412	-0.373	0.41511	TRUE	1.00E+00
39374_at	0.02111	1.049808	0.100987	-0.44481	0.48702	TRUE	1.00E+00
35305_at	0.02112	1.049832	0.026883	-0.10291	0.14515	TRUE	1.00E+00
32978_g_at	0.02114	1.049881	0.050356	-0.21118	0.25346	TRUE	1.00E+00
38185_at	0.02116	1.049929	0.018352	-0.06351	0.10583	TRUE	1.00E+00
38942_r_at	0.02118	1.049978	0.024671	-0.09264	0.135	TRUE	1.00E+00
37325_at	0.02119	1.050002	0.036821	-0.14869	0.19106	TRUE	1.00E+00
34211_at	0.02119	1.050002	0.03452	-0.13807	0.18045	TRUE	1.00E+00
37001_at	0.02121	1.05005	0.025729	-0.09749	0.13991	TRUE	1.00E+00
36421_at	0.02123	1.050098	0.037638	-0.15242	0.19487	TRUE	1.00E+00
32241_at	0.02123	1.050098	0.01941	-0.06832	0.11078	TRUE	1.00E+00
1400_at	0.02127	1.050195	0.096085	-0.42202	0.46457	TRUE	1.00E+00
35947_at	0.02129	1.050243	0.075525	-0.32715	0.36973	TRUE	1.00E+00
41373_s_at	0.02131	1.050292	0.054832	-0.23166	0.27429	TRUE	1.00E+00
168_at	0.02134	1.050364	0.041664	-0.17088	0.21356	TRUE	1.00E+00
31644_at	0.02138	1.050461	0.050981	-0.21383	0.25658	TRUE	1.00E+00

39112_at	0.0214	1.05051	0.019199	-0.06718	0.10997	TRUE	1.00E+00
40053_at	0.0214	1.05051	0.062643	-0.26761	0.31041	TRUE	1.00E+00
37497_at	0.02141	1.050534	0.043089	-0.17738	0.22021	TRUE	1.00E+00
600_at	0.02147	1.050679	0.029182	-0.11316	0.1561	TRUE	1.00E+00
36980_at	0.02152	1.0508	0.060831	-0.25913	0.30217	TRUE	1.00E+00
34810_at	0.02154	1.050848	0.029424	-0.11421	0.1573	TRUE	1.00E+00
1828_s_at	0.02155	1.050872	0.041863	-0.17159	0.21469	TRUE	1.00E+00
40327_at	0.02157	1.050921	0.021612	-0.07814	0.12128	TRUE	1.00E+00
38642_at	0.02161	1.051018	0.055905	-0.23631	0.27953	TRUE	1.00E+00
34590_at	0.02164	1.05109	0.062621	-0.26727	0.31055	TRUE	1.00E+00
37335_at	0.02165	1.051114	0.016474	-0.05435	0.09765	TRUE	1.00E+00
231_at	0.02169	1.051211	0.07602	-0.32904	0.37242	TRUE	1.00E+00
31852_at	0.02171	1.05126	0.027211	-0.10383	0.14725	TRUE	1.00E+00
37247_at	0.02172	1.051284	0.062336	-0.26587	0.30931	TRUE	1.00E+00
31535_i_at	0.02173	1.051308	0.02948	-0.11428	0.15774	TRUE	1.00E+00
35840_at	0.02174	1.051332	0.045195	-0.18677	0.23025	TRUE	1.00E+00
1106_s_at	0.02174	1.051332	0.050898	-0.21308	0.25656	TRUE	1.00E+00
1495_at	0.02181	1.051502	0.066154	-0.2834	0.32702	TRUE	1.00E+00
34669_at	0.02182	1.051526	0.018775	-0.06481	0.10844	TRUE	1.00E+00
33033_at	0.02184	1.051574	0.127791	-0.56774	0.61142	TRUE	1.00E+00
40469_at	0.02192	1.051768	0.028936	-0.11158	0.15542	TRUE	1.00E+00
603_at	0.02193	1.051792	0.042094	-0.17227	0.21614	TRUE	1.00E+00
39071_at	0.02205	1.052083	0.020937	-0.07455	0.11864	TRUE	1.00E+00
40617_at	0.0221	1.052204	0.014133	-0.0431	0.0873	TRUE	1.00E+00
37674_at	0.02217	1.052374	0.027971	-0.10688	0.15121	TRUE	1.00E+00
802_at	0.02218	1.052398	0.013737	-0.0412	0.08556	TRUE	1.00E+00
36148_at	0.02219	1.052422	0.029126	-0.11218	0.15657	TRUE	1.00E+00
33472_at	0.02224	1.052543	0.032741	-0.12882	0.17329	TRUE	1.00E+00
1608_at	0.02225	1.052568	0.041798	-0.17058	0.21509	TRUE	1.00E+00
36540_at	0.02231	1.052713	0.028012	-0.10692	0.15155	TRUE	1.00E+00
38992_at	0.02232	1.052737	0.012848	-0.03695	0.0816	TRUE	1.00E+00
32863_at	0.02233	1.052762	0.058219	-0.24627	0.29093	TRUE	1.00E+00
38876_at	0.02234	1.052786	0.063192	-0.2692	0.31388	TRUE	1.00E+00
32448_at	0.02245	1.053052	0.047602	-0.19717	0.24206	TRUE	1.00E+00
41705_at	0.02246	1.053077	0.032442	-0.12722	0.17213	TRUE	1.00E+00
37867_at	0.02251	1.053198	0.0637	-0.27137	0.3164	TRUE	1.00E+00
39219_at	0.02252	1.053222	0.017417	-0.05784	0.10287	TRUE	1.00E+00
38830_at	0.02254	1.053271	0.021288	-0.07567	0.12076	TRUE	1.00E+00
34503_at	0.02257	1.053343	0.027047	-0.10221	0.14735	TRUE	1.00E+00
34723_at	0.02262	1.053465	0.02665	-0.10033	0.14557	TRUE	1.00E+00
41444_at	0.02265	1.053538	0.107394	-0.47282	0.51812	TRUE	1.00E+00
35270_at	0.02265	1.053538	0.01004	-0.02367	0.06897	TRUE	1.00E+00
38629_at	0.02267	1.053586	0.032997	-0.12957	0.1749	TRUE	1.00E+00
1848_at	0.02268	1.05361	0.037194	-0.14892	0.19428	TRUE	1.00E+00
35239_at	0.02269	1.053635	0.018847	-0.06427	0.10964	TRUE	1.00E+00
38758_at	0.02269	1.053635	0.013123	-0.03785	0.08324	TRUE	1.00E+00
39019_at	0.02272	1.053707	0.013053	-0.0375	0.08294	TRUE	1.00E+00
36904_at	0.02273	1.053732	0.02943	-0.11305	0.1585	TRUE	1.00E+00
1885_at	0.02274	1.053756	0.01715	-0.05639	0.10186	TRUE	1.00E+00
1157_s_at	0.02274	1.053756	0.02078	-0.07313	0.11861	TRUE	1.00E+00
33587_f_at	0.02277	1.053829	0.069292	-0.29692	0.34245	TRUE	1.00E+00

39245_at	0.02277	1.053829	0.025558	-0.09514	0.14069	TRUE	1.00E+00
1071_at	0.02278	1.053853	0.098148	-0.43003	0.4756	TRUE	1.00E+00
41224_at	0.02278	1.053853	0.009813	-0.02249	0.06806	TRUE	1.00E+00
39986_at	0.02281	1.053926	0.018079	-0.0606	0.10621	TRUE	1.00E+00
35126_at	0.02285	1.054023	0.052476	-0.21925	0.26496	TRUE	1.00E+00
953_g_at	0.02287	1.054071	0.036687	-0.14639	0.19212	TRUE	1.00E+00
41116_at	0.02287	1.054071	0.01123	-0.02894	0.07468	TRUE	1.00E+00
38114_at	0.02288	1.054096	0.019061	-0.06506	0.11082	TRUE	1.00E+00
35673_at	0.02288	1.054096	0.032396	-0.12658	0.17234	TRUE	1.00E+00
34146_at	0.02297	1.054314	0.074988	-0.32299	0.36893	TRUE	1.00E+00
35005_at	0.02298	1.054338	0.032642	-0.12762	0.17358	TRUE	1.00E+00
37321_at	0.023	1.054387	0.012378	-0.03411	0.08011	TRUE	1.00E+00
31483_g_at	0.02305	1.054508	0.040354	-0.16313	0.20922	TRUE	1.00E+00
38869_at	0.02305	1.054508	0.106223	-0.46702	0.51312	TRUE	1.00E+00
39055_at	0.02306	1.054533	0.021178	-0.07465	0.12076	TRUE	1.00E+00
35631_at	0.02306	1.054533	0.024451	-0.08975	0.13586	TRUE	1.00E+00
39385_at	0.02311	1.054654	0.038144	-0.15287	0.19909	TRUE	1.00E+00
33416_at	0.02311	1.054654	0.028399	-0.10791	0.15413	TRUE	1.00E+00
36977_at	0.02311	1.054654	0.025763	-0.09575	0.14197	TRUE	1.00E+00
33233_at	0.02316	1.054775	0.039661	-0.15982	0.20614	TRUE	1.00E+00
33976_at	0.02319	1.054848	0.061902	-0.2624	0.30878	TRUE	1.00E+00
34635_at	0.02322	1.054921	0.012409	-0.03404	0.08047	TRUE	1.00E+00
1500_at	0.02322	1.054921	0.034319	-0.13511	0.18156	TRUE	1.00E+00
31550_at	0.02322	1.054921	0.115969	-0.51181	0.55825	TRUE	1.00E+00
1323_at	0.02324	1.05497	0.025035	-0.09227	0.13874	TRUE	1.00E+00
33815_at	0.02324	1.05497	0.026069	-0.09703	0.14351	TRUE	1.00E+00
1278_at	0.02327	1.055043	0.053194	-0.22214	0.26868	TRUE	1.00E+00
38676_at	0.02329	1.055091	0.029704	-0.11375	0.16033	TRUE	1.00E+00
31867_at	0.0233	1.055115	0.027643	-0.10423	0.15084	TRUE	1.00E+00
1469_at	0.02331	1.05514	0.038907	-0.15619	0.20281	TRUE	1.00E+00
35621_at	0.02334	1.055213	0.024523	-0.0898	0.13648	TRUE	1.00E+00
40585_at	0.02339	1.055334	0.028834	-0.10964	0.15641	TRUE	1.00E+00
32524_s_at	0.0234	1.055358	0.079875	-0.3451	0.39191	TRUE	1.00E+00
36347_f_at	0.02344	1.055456	0.063865	-0.27121	0.31808	TRUE	1.00E+00
34772_at	0.02345	1.05548	0.034978	-0.13792	0.18483	TRUE	1.00E+00
36013_at	0.02346	1.055504	0.013261	-0.03772	0.08464	TRUE	1.00E+00
37696_at	0.02346	1.055504	0.02336	-0.08431	0.13124	TRUE	1.00E+00
41442_at	0.02348	1.055553	0.0397	-0.15968	0.20664	TRUE	1.00E+00
37097_at	0.02354	1.055699	0.066274	-0.28223	0.3293	TRUE	1.00E+00
37238_s_at	0.02355	1.055723	0.012413	-0.03372	0.08082	TRUE	1.00E+00
37667_at	0.02364	1.055942	0.025015	-0.09177	0.13904	TRUE	1.00E+00
36621_at	0.02364	1.055942	0.096594	-0.422	0.46928	TRUE	1.00E+00
32429_f_at	0.02366	1.05599	0.028966	-0.10998	0.15729	TRUE	1.00E+00
36214_at	0.02366	1.05599	0.0333	-0.12997	0.1773	TRUE	1.00E+00
40699_at	0.02368	1.056039	0.039537	-0.15873	0.20608	TRUE	1.00E+00
34563_at	0.02369	1.056063	0.083856	-0.36319	0.41057	TRUE	1.00E+00
1030_s_at	0.02376	1.056234	0.01329	-0.03755	0.08508	TRUE	1.00E+00
38481_at	0.02376	1.056234	0.025749	-0.09503	0.14256	TRUE	1.00E+00
33591_at	0.02379	1.056307	0.065544	-0.2786	0.32619	TRUE	1.00E+00
38031_at	0.02381	1.056355	0.02204	-0.07787	0.12549	TRUE	1.00E+00
33722_at	0.02391	1.056599	0.03788	-0.15086	0.19867	TRUE	1.00E+00

39733_at	0.02394	1.056672	0.02405	-0.08702	0.13489	TRUE	1.00E+00
32166_at	0.02394	1.056672	0.032283	-0.12499	0.17288	TRUE	1.00E+00
37454_at	0.02395	1.056696	0.089392	-0.38846	0.43637	TRUE	1.00E+00
38707_r_at	0.02398	1.056769	0.015674	-0.04834	0.09629	TRUE	1.00E+00
41577_at	0.02403	1.056891	0.037074	-0.14701	0.19508	TRUE	1.00E+00
38833_at	0.02404	1.056915	0.040772	-0.16407	0.21214	TRUE	1.00E+00
38413_at	0.02406	1.056964	0.021228	-0.07388	0.122	TRUE	1.00E+00
37547_at	0.02407	1.056988	0.037405	-0.1485	0.19664	TRUE	1.00E+00
37181_at	0.02409	1.057037	0.013906	-0.04006	0.08824	TRUE	1.00E+00
38689_at	0.02415	1.057183	0.018364	-0.06057	0.10888	TRUE	1.00E+00
39771_at	0.02418	1.057256	0.025396	-0.09299	0.14134	TRUE	1.00E+00
32407_f_at	0.02419	1.05728	0.023087	-0.08232	0.13071	TRUE	1.00E+00
2029_at	0.0242	1.057304	0.03265	-0.12644	0.17483	TRUE	1.00E+00
38449_at	0.0242	1.057304	0.014854	-0.04433	0.09273	TRUE	1.00E+00
33367_s_at	0.02421	1.057329	0.029574	-0.11224	0.16065	TRUE	1.00E+00
35910_f_at	0.02424	1.057402	0.026452	-0.0978	0.14628	TRUE	1.00E+00
31356_at	0.02426	1.05745	0.024128	-0.08706	0.13558	TRUE	1.00E+00
33122_at	0.02427	1.057475	0.065261	-0.27681	0.32536	TRUE	1.00E+00
36117_at	0.02428	1.057499	0.03664	-0.14476	0.19332	TRUE	1.00E+00
38926_at	0.02432	1.057596	0.04168	-0.16798	0.21661	TRUE	1.00E+00
39751_at	0.02439	1.057767	0.035486	-0.13933	0.1881	TRUE	1.00E+00
37703_at	0.0244	1.057791	0.018975	-0.06314	0.11194	TRUE	1.00E+00
36552_at	0.02441	1.057816	0.027141	-0.10081	0.14963	TRUE	1.00E+00
36843_at	0.02443	1.057864	0.065776	-0.27903	0.32789	TRUE	1.00E+00
34917_at	0.02444	1.057889	0.028745	-0.10818	0.15706	TRUE	1.00E+00
35269_at	0.0245	1.058035	0.034682	-0.13551	0.1845	TRUE	1.00E+00
33426_at	0.02452	1.058084	0.129841	-0.57451	0.62356	TRUE	1.00E+00
34788_at	0.02453	1.058108	0.024782	-0.0898	0.13886	TRUE	1.00E+00
40581_at	0.02454	1.058132	0.030389	-0.11566	0.16475	TRUE	1.00E+00
34628_at	0.02456	1.058181	0.105107	-0.46037	0.50948	TRUE	1.00E+00
37061_at	0.02456	1.058181	0.078519	-0.3377	0.38681	TRUE	1.00E+00
35602_at	0.02458	1.05823	0.047449	-0.19433	0.24349	TRUE	1.00E+00
32579_at	0.02459	1.058254	0.023872	-0.08555	0.13473	TRUE	1.00E+00
215_g_at	0.0246	1.058279	0.018685	-0.06161	0.1108	TRUE	1.00E+00
36252_at	0.02467	1.058449	0.02484	-0.08993	0.13927	TRUE	1.00E+00
35484_at	0.02467	1.058449	0.05485	-0.22838	0.27773	TRUE	1.00E+00
36781_at	0.0247	1.058522	0.02246	-0.07892	0.12832	TRUE	1.00E+00
40343_at	0.02471	1.058547	0.020458	-0.06967	0.1191	TRUE	1.00E+00
32530_at	0.02472	1.058571	0.012738	-0.03405	0.08349	TRUE	1.00E+00
37556_at	0.02473	1.058595	0.046526	-0.18993	0.23938	TRUE	1.00E+00
41084_at	0.02474	1.05862	0.022201	-0.07768	0.12717	TRUE	1.00E+00
1308_g_at	0.02475	1.058644	0.05802	-0.24293	0.29243	TRUE	1.00E+00
33225_at	0.02477	1.058693	0.033587	-0.13018	0.17973	TRUE	1.00E+00
39762_at	0.02481	1.05879	0.031734	-0.12159	0.17122	TRUE	1.00E+00
31948_at	0.02486	1.058912	0.033834	-0.13123	0.18096	TRUE	1.00E+00
34622_at	0.02487	1.058937	0.025398	-0.09231	0.14205	TRUE	1.00E+00
36002_at	0.02488	1.058961	0.020823	-0.07118	0.12095	TRUE	1.00E+00
35330_at	0.02489	1.058985	0.053164	-0.22039	0.27016	TRUE	1.00E+00
838_s_at	0.02489	1.058985	0.01568	-0.04745	0.09723	TRUE	1.00E+00
35263_at	0.02494	1.059107	0.026081	-0.09539	0.14527	TRUE	1.00E+00
32993_s_at	0.02495	1.059132	0.033898	-0.13144	0.18134	TRUE	1.00E+00

34785_at	0.02496	1.059156	0.02765	-0.10261	0.15252	TRUE	1.00E+00
38537_at	0.02498	1.059205	0.027641	-0.10255	0.1525	TRUE	1.00E+00
34845_at	0.025	1.059254	0.008315	-0.01336	0.06337	TRUE	1.00E+00
39556_at	0.02502	1.059303	0.032387	-0.1244	0.17444	TRUE	1.00E+00
32577_s_at	0.02503	1.059327	0.071989	-0.3071	0.35716	TRUE	1.00E+00
36701_at	0.02504	1.059351	0.045566	-0.18518	0.23526	TRUE	1.00E+00
36734_at	0.02508	1.059449	0.067381	-0.28579	0.33595	TRUE	1.00E+00
39431_at	0.0251	1.059498	0.018152	-0.05865	0.10884	TRUE	1.00E+00
37670_at	0.02513	1.059571	0.0244	-0.08745	0.1377	TRUE	1.00E+00
36611_at	0.02513	1.059571	0.017934	-0.05761	0.10786	TRUE	1.00E+00
39806_at	0.02513	1.059571	0.025148	-0.09089	0.14116	TRUE	1.00E+00
36607_at	0.02515	1.05962	0.037624	-0.14843	0.19873	TRUE	1.00E+00
32432_f_at	0.02515	1.05962	0.010342	-0.02256	0.07286	TRUE	1.00E+00
36945_at	0.02516	1.059644	0.024339	-0.08713	0.13745	TRUE	1.00E+00
32621_at	0.02518	1.059693	0.0271	-0.09985	0.15021	TRUE	1.00E+00
38063_at	0.02521	1.059766	0.026307	-0.09616	0.14658	TRUE	1.00E+00
36624_at	0.02521	1.059766	0.021704	-0.07492	0.12534	TRUE	1.00E+00
35524_at	0.02521	1.059766	0.032114	-0.12295	0.17338	TRUE	1.00E+00
33069_f_at	0.02526	1.059888	0.107358	-0.47004	0.52056	TRUE	1.00E+00
420_at	0.02526	1.059888	0.025973	-0.09457	0.14509	TRUE	1.00E+00
40369_f_at	0.02526	1.059888	0.036706	-0.14409	0.19461	TRUE	1.00E+00
32305_at	0.02531	1.06001	0.017614	-0.05596	0.10657	TRUE	1.00E+00
31991_at	0.02531	1.06001	0.027947	-0.10363	0.15425	TRUE	1.00E+00
775_at	0.02532	1.060034	0.027882	-0.10332	0.15395	TRUE	1.00E+00
39298_at	0.02536	1.060132	0.052284	-0.21585	0.26658	TRUE	1.00E+00
41567_at	0.02537	1.060157	0.054036	-0.22393	0.27467	TRUE	1.00E+00
39027_at	0.02537	1.060157	0.022856	-0.08008	0.13082	TRUE	1.00E+00
373_at	0.02537	1.060157	0.058214	-0.2432	0.29395	TRUE	1.00E+00
41251_at	0.02539	1.060205	0.019932	-0.06656	0.11735	TRUE	1.00E+00
699_s_at	0.0254	1.06023	0.080369	-0.34539	0.39619	TRUE	1.00E+00
32996_g_at	0.0254	1.06023	0.02535	-0.09155	0.14236	TRUE	1.00E+00
34849_at	0.02541	1.060254	0.00968	-0.01925	0.07007	TRUE	1.00E+00
145_s_at	0.02542	1.060279	0.1153	-0.50653	0.55737	TRUE	1.00E+00
38702_at	0.02542	1.060279	0.025972	-0.0944	0.14525	TRUE	1.00E+00
39313_at	0.02553	1.060547	0.027456	-0.10114	0.1522	TRUE	1.00E+00
32467_at	0.02554	1.060572	0.068923	-0.29245	0.34352	TRUE	1.00E+00
35825_s_at	0.02554	1.060572	0.056878	-0.23687	0.28796	TRUE	1.00E+00
37162_at	0.02556	1.06062	0.021823	-0.07512	0.12625	TRUE	1.00E+00
41052_s_at	0.02557	1.060645	0.094357	-0.40975	0.46089	TRUE	1.00E+00
37947_at	0.02558	1.060669	0.022188	-0.07679	0.12794	TRUE	1.00E+00
34000_r_at	0.02559	1.060694	0.115461	-0.5071	0.55828	TRUE	1.00E+00
32866_at	0.02559	1.060694	0.018912	-0.06166	0.11284	TRUE	1.00E+00
34997_r_at	0.0256	1.060718	0.103536	-0.45207	0.50327	TRUE	1.00E+00
35503_at	0.0256	1.060718	0.030134	-0.11342	0.16463	TRUE	1.00E+00
35838_at	0.02566	1.060865	0.028022	-0.10362	0.15494	TRUE	1.00E+00
32556_at	0.02577	1.061133	0.048768	-0.19922	0.25077	TRUE	1.00E+00
31526_f_at	0.02579	1.061182	0.036719	-0.14362	0.1952	TRUE	1.00E+00
38410_at	0.02581	1.061231	0.041489	-0.1656	0.21722	TRUE	1.00E+00
39942_at	0.02584	1.061304	0.076453	-0.32688	0.37857	TRUE	1.00E+00
41026_f_at	0.0259	1.061451	0.025411	-0.09133	0.14314	TRUE	1.00E+00
41170_at	0.02596	1.061598	0.028023	-0.10332	0.15525	TRUE	1.00E+00

34547_at	0.02599	1.061671	0.048262	-0.19667	0.24866	TRUE	1.00E+00
37881_at	0.02602	1.061744	0.107232	-0.4687	0.52074	TRUE	1.00E+00
35590_s_at	0.02608	1.061891	0.06283	-0.26379	0.31595	TRUE	1.00E+00
34989_at	0.02615	1.062062	0.062917	-0.26412	0.31643	TRUE	1.00E+00
36034_at	0.02616	1.062087	0.028218	-0.10403	0.15635	TRUE	1.00E+00
1858_at	0.02617	1.062111	0.065052	-0.27395	0.3263	TRUE	1.00E+00
36097_at	0.02623	1.062258	0.037404	-0.14634	0.1988	TRUE	1.00E+00
32107_at	0.02623	1.062258	0.033077	-0.12637	0.17884	TRUE	1.00E+00
1430_at	0.02627	1.062356	0.03061	-0.11495	0.16749	TRUE	1.00E+00
39499_s_at	0.02628	1.06238	0.036923	-0.14407	0.19662	TRUE	1.00E+00
34164_at	0.02631	1.062454	0.037988	-0.14895	0.20157	TRUE	1.00E+00
32138_at	0.02632	1.062478	0.031766	-0.12024	0.17287	TRUE	1.00E+00
33239_at	0.02632	1.062478	0.023394	-0.08161	0.13425	TRUE	1.00E+00
39011_at	0.02635	1.062552	0.018644	-0.05967	0.11236	TRUE	1.00E+00
38753_at	0.02636	1.062576	0.011535	-0.02686	0.07958	TRUE	1.00E+00
39150_at	0.02638	1.062625	0.019672	-0.06438	0.11714	TRUE	1.00E+00
2028_s_at	0.02643	1.062747	0.031938	-0.12092	0.17378	TRUE	1.00E+00
33553_r_at	0.02647	1.062845	0.083647	-0.35945	0.41238	TRUE	1.00E+00
35141_at	0.02652	1.062968	0.065868	-0.27737	0.3304	TRUE	1.00E+00
40379_at	0.02659	1.063139	0.053961	-0.22237	0.27554	TRUE	1.00E+00
35378_at	0.0267	1.063408	0.025808	-0.09237	0.14577	TRUE	1.00E+00
35773_i_at	0.02673	1.063482	0.028335	-0.104	0.15746	TRUE	1.00E+00
41365_at	0.02676	1.063555	0.042311	-0.16845	0.22196	TRUE	1.00E+00
31664_at	0.0268	1.063653	0.063851	-0.26778	0.32139	TRUE	1.00E+00
33203_s_at	0.02682	1.063702	0.05309	-0.21812	0.27175	TRUE	1.00E+00
36227_at	0.02686	1.0638	0.061514	-0.25694	0.31066	TRUE	1.00E+00
32337_at	0.02686	1.0638	0.01487	-0.04174	0.09547	TRUE	1.00E+00
440_at	0.02693	1.063972	0.032498	-0.123	0.17687	TRUE	1.00E+00
34690_at	0.02695	1.064021	0.105002	-0.45749	0.51138	TRUE	1.00E+00
41651_at	0.02695	1.064021	0.014663	-0.0407	0.0946	TRUE	1.00E+00
39756_g_at	0.02699	1.064119	0.017643	-0.05441	0.10839	TRUE	1.00E+00
38104_at	0.027	1.064143	0.014101	-0.03806	0.09206	TRUE	1.00E+00
41737_at	0.02704	1.064241	0.010221	-0.02011	0.0742	TRUE	1.00E+00
1978_at	0.02707	1.064315	0.125477	-0.55183	0.60597	TRUE	1.00E+00
39194_at	0.02708	1.064339	0.0607	-0.25297	0.30712	TRUE	1.00E+00
37317_at	0.02712	1.064437	0.045468	-0.18265	0.23689	TRUE	1.00E+00
32181_at	0.02713	1.064462	0.040566	-0.16002	0.21428	TRUE	1.00E+00
40382_at	0.02713	1.064462	0.062639	-0.26186	0.31612	TRUE	1.00E+00
31537_at	0.02713	1.064462	0.042139	-0.16728	0.22155	TRUE	1.00E+00
766_at	0.02714	1.064486	0.025594	-0.09094	0.14522	TRUE	1.00E+00
40853_at	0.02717	1.06456	0.019443	-0.06253	0.11687	TRUE	1.00E+00
33179_at	0.0272	1.064633	0.032896	-0.12457	0.17897	TRUE	1.00E+00
32210_at	0.0272	1.064633	0.039235	-0.15381	0.20821	TRUE	1.00E+00
34206_at	0.02722	1.064682	0.015882	-0.04605	0.10049	TRUE	1.00E+00
38438_at	0.02722	1.064682	0.031706	-0.11906	0.1735	TRUE	1.00E+00
38024_at	0.02725	1.064756	0.021296	-0.071	0.1255	TRUE	1.00E+00
1307_at	0.02732	1.064927	0.015277	-0.04317	0.0978	TRUE	1.00E+00
33048_at	0.02732	1.064927	0.068938	-0.29073	0.34537	TRUE	1.00E+00
36676_at	0.02737	1.06505	0.022002	-0.07414	0.12888	TRUE	1.00E+00
33147_at	0.02738	1.065075	0.077189	-0.32874	0.38349	TRUE	1.00E+00
539_at	0.02738	1.065075	0.017871	-0.05507	0.10983	TRUE	1.00E+00

2014_s_at	0.0274	1.065124	0.074561	-0.3166	0.37139	TRUE	1.00E+00
37751_at	0.0274	1.065124	0.022392	-0.07591	0.1307	TRUE	1.00E+00
1919_at	0.02741	1.065148	0.053764	-0.22064	0.27545	TRUE	1.00E+00
40280_at	0.02741	1.065148	0.014448	-0.03924	0.09407	TRUE	1.00E+00
1952_s_at	0.02745	1.065246	0.03488	-0.13347	0.18837	TRUE	1.00E+00
1511_at	0.02748	1.06532	0.07063	-0.29838	0.35333	TRUE	1.00E+00
38099_r_at	0.02748	1.06532	0.029979	-0.11083	0.16579	TRUE	1.00E+00
38425_at	0.0275	1.065369	0.016552	-0.04886	0.10387	TRUE	1.00E+00
35039_at	0.02751	1.065393	0.02596	-0.09226	0.14728	TRUE	1.00E+00
36314_at	0.02753	1.065442	0.084312	-0.36145	0.41651	TRUE	1.00E+00
1373_at	0.02756	1.065516	0.034403	-0.13116	0.18628	TRUE	1.00E+00
32160_at	0.02761	1.065639	0.024257	-0.0843	0.13952	TRUE	1.00E+00
38453_at	0.02764	1.065712	0.058375	-0.24168	0.29696	TRUE	1.00E+00
33327_at	0.02766	1.065761	0.041147	-0.16218	0.21749	TRUE	1.00E+00
34465_at	0.02768	1.065811	0.057807	-0.23902	0.29438	TRUE	1.00E+00
38948_at	0.02772	1.065909	0.033129	-0.12513	0.18056	TRUE	1.00E+00
34863_s_at	0.02773	1.065933	0.01641	-0.04798	0.10345	TRUE	1.00E+00
40935_at	0.02775	1.065982	0.057632	-0.23814	0.29363	TRUE	1.00E+00
38310_at	0.0278	1.066105	0.05058	-0.20555	0.26116	TRUE	1.00E+00
41868_at	0.02781	1.06613	0.112258	-0.4901	0.54572	TRUE	1.00E+00
38652_at	0.02787	1.066277	0.011662	-0.02593	0.08168	TRUE	1.00E+00
36507_at	0.02789	1.066326	0.026144	-0.09273	0.1485	TRUE	1.00E+00
1939_at	0.02792	1.0664	0.014573	-0.03931	0.09515	TRUE	1.00E+00
34893_at	0.02794	1.066449	0.023363	-0.07985	0.13572	TRUE	1.00E+00
423_at	0.02795	1.066473	0.113567	-0.496	0.5519	TRUE	1.00E+00
33596_at	0.02796	1.066498	0.087235	-0.37451	0.43043	TRUE	1.00E+00
1827_s_at	0.02797	1.066522	0.020397	-0.06613	0.12207	TRUE	1.00E+00
37764_at	0.02802	1.066645	0.032299	-0.121	0.17703	TRUE	1.00E+00
40032_at	0.02803	1.06667	0.045177	-0.18039	0.23646	TRUE	1.00E+00
40933_f_at	0.02805	1.066719	0.044769	-0.1785	0.23459	TRUE	1.00E+00
34192_at	0.02805	1.066719	0.032084	-0.11997	0.17607	TRUE	1.00E+00
109_at	0.02805	1.066719	0.029935	-0.11006	0.16616	TRUE	1.00E+00
34673_r_at	0.02808	1.066793	0.033595	-0.12691	0.18308	TRUE	1.00E+00
36166_at	0.02809	1.066817	0.034226	-0.12982	0.18599	TRUE	1.00E+00
32401_at	0.02809	1.066817	0.023925	-0.08229	0.13847	TRUE	1.00E+00
36791_g_at	0.0281	1.066842	0.04635	-0.18574	0.24194	TRUE	1.00E+00
38479_at	0.02812	1.066891	0.036556	-0.14054	0.19678	TRUE	1.00E+00
40047_at	0.02813	1.066915	0.031749	-0.11835	0.17461	TRUE	1.00E+00
36576_at	0.02813	1.066915	0.009858	-0.01735	0.07361	TRUE	1.00E+00
32258_r_at	0.02814	1.06694	0.039732	-0.15516	0.21145	TRUE	1.00E+00
35699_at	0.02817	1.067014	0.092169	-0.39706	0.4534	TRUE	1.00E+00
32829_at	0.02823	1.067161	0.02094	-0.06838	0.12484	TRUE	1.00E+00
38051_at	0.02823	1.067161	0.042796	-0.16921	0.22567	TRUE	1.00E+00
36888_at	0.02825	1.06721	0.028995	-0.10551	0.16202	TRUE	1.00E+00
38489_at	0.02826	1.067235	0.06612	-0.27679	0.33331	TRUE	1.00E+00
36488_at	0.02829	1.067309	0.038471	-0.1492	0.20578	TRUE	1.00E+00
39465_f_at	0.02834	1.067431	0.090879	-0.39094	0.44762	TRUE	1.00E+00
41659_at	0.02834	1.067431	0.02331	-0.0792	0.13589	TRUE	1.00E+00
37465_at	0.02837	1.067505	0.040583	-0.15886	0.21561	TRUE	1.00E+00
1824_s_at	0.02837	1.067505	0.03666	-0.14076	0.19751	TRUE	1.00E+00
33647_s_at	0.02839	1.067554	0.027228	-0.09723	0.15401	TRUE	1.00E+00

37578_at	0.0284	1.067579	0.02766	-0.09922	0.15601	TRUE	1.00E+00
32871_at	0.02841	1.067604	0.112872	-0.49234	0.54915	TRUE	1.00E+00
33344_at	0.02841	1.067604	0.1769	-0.78773	0.84455	TRUE	1.00E+00
39199_at	0.02842	1.067628	0.033945	-0.12819	0.18503	TRUE	1.00E+00
40104_at	0.02842	1.067628	0.013526	-0.03398	0.09082	TRUE	1.00E+00
1668_s_at	0.02845	1.067702	0.032566	-0.1218	0.17869	TRUE	1.00E+00
35302_at	0.0285	1.067825	0.018068	-0.05485	0.11186	TRUE	1.00E+00
37410_at	0.02854	1.067923	0.022937	-0.07728	0.13436	TRUE	1.00E+00
39399_at	0.02854	1.067923	0.020087	-0.06413	0.12122	TRUE	1.00E+00
38282_at	0.02855	1.067948	0.025024	-0.0869	0.144	TRUE	1.00E+00
34537_at	0.02857	1.067997	0.029345	-0.10682	0.16395	TRUE	1.00E+00
36666_at	0.02861	1.068095	0.022061	-0.07317	0.1304	TRUE	1.00E+00
40998_at	0.02862	1.06812	0.01354	-0.03385	0.09109	TRUE	1.00E+00
37153_at	0.02868	1.068267	0.01969	-0.06216	0.11952	TRUE	1.00E+00
34560_at	0.02869	1.068292	0.040614	-0.15869	0.21606	TRUE	1.00E+00
37293_at	0.02871	1.068341	0.018366	-0.05602	0.11344	TRUE	1.00E+00
32722_at	0.02872	1.068366	0.131502	-0.57798	0.63542	TRUE	1.00E+00
35958_at	0.02875	1.06844	0.116088	-0.50683	0.56433	TRUE	1.00E+00
39752_at	0.02881	1.068587	0.072867	-0.30738	0.36499	TRUE	1.00E+00
1135_at	0.02883	1.068636	0.058685	-0.24192	0.29957	TRUE	1.00E+00
39588_at	0.02884	1.068661	0.034086	-0.12841	0.1861	TRUE	1.00E+00
33322_i_at	0.02888	1.06876	0.009842	-0.01653	0.07429	TRUE	1.00E+00
40249_at	0.02888	1.06876	0.065951	-0.27539	0.33315	TRUE	1.00E+00
1635_at	0.02891	1.068833	0.016766	-0.04845	0.10626	TRUE	1.00E+00
31395_i_at	0.02891	1.068833	0.030615	-0.11233	0.17015	TRUE	1.00E+00
38725_s_at	0.02891	1.068833	0.027706	-0.09891	0.15674	TRUE	1.00E+00
40838_at	0.02893	1.068883	0.046919	-0.18753	0.2454	TRUE	1.00E+00
40459_at	0.02898	1.069006	0.021304	-0.06931	0.12726	TRUE	1.00E+00
33129_at	0.02899	1.06903	0.008312	-0.00936	0.06733	TRUE	1.00E+00
33485_at	0.02899	1.06903	0.011053	-0.022	0.07999	TRUE	1.00E+00
1415_at	0.02906	1.069203	0.022425	-0.0744	0.13252	TRUE	1.00E+00
36445_at	0.02909	1.069276	0.069568	-0.29187	0.35005	TRUE	1.00E+00
301_at	0.02913	1.069375	0.07924	-0.33644	0.39471	TRUE	1.00E+00
40651_s_at	0.02914	1.0694	0.024217	-0.08259	0.14086	TRUE	1.00E+00
32259_at	0.02917	1.069473	0.024726	-0.08491	0.14325	TRUE	1.00E+00
32908_at	0.02918	1.069498	0.071471	-0.30056	0.35892	TRUE	1.00E+00
36878_f_at	0.02919	1.069523	0.098732	-0.42632	0.4847	TRUE	1.00E+00
32386_at	0.0292	1.069547	0.037106	-0.14199	0.20039	TRUE	1.00E+00
33400_r_at	0.02921	1.069572	0.02379	-0.08054	0.13897	TRUE	1.00E+00
1817_at	0.02922	1.069597	0.026639	-0.09368	0.15212	TRUE	1.00E+00
1192_at	0.02923	1.069621	0.026863	-0.0947	0.15317	TRUE	1.00E+00
41535_at	0.02925	1.06967	0.023706	-0.08012	0.13862	TRUE	1.00E+00
422_s_at	0.02928	1.069744	0.050762	-0.20491	0.26348	TRUE	1.00E+00
39041_at	0.0293	1.069794	0.011173	-0.02225	0.08085	TRUE	1.00E+00
36045_at	0.02932	1.069843	0.029514	-0.10685	0.16548	TRUE	1.00E+00
34049_at	0.02938	1.069991	0.103881	-0.44988	0.50865	TRUE	1.00E+00
40874_at	0.0294	1.07004	0.0143	-0.03658	0.09537	TRUE	1.00E+00
36879_at	0.02941	1.070065	0.039683	-0.15367	0.21249	TRUE	1.00E+00
31484_at	0.02949	1.070262	0.074393	-0.31373	0.37271	TRUE	1.00E+00
31842_at	0.02954	1.070385	0.017052	-0.04913	0.10821	TRUE	1.00E+00
32933_r_at	0.02954	1.070385	0.0746	-0.31464	0.37371	TRUE	1.00E+00

39281_at	0.02956	1.070434	0.013801	-0.03412	0.09323	TRUE	1.00E+00
37659_at	0.02958	1.070484	0.018184	-0.05432	0.11347	TRUE	1.00E+00
38616_at	0.02961	1.070558	0.080652	-0.34248	0.4017	TRUE	1.00E+00
1327_s_at	0.02961	1.070558	0.084031	-0.35807	0.4173	TRUE	1.00E+00
1737_s_at	0.02963	1.070607	0.023943	-0.08083	0.1401	TRUE	1.00E+00
39203_at	0.02967	1.070705	0.075198	-0.31726	0.3766	TRUE	1.00E+00
40099_at	0.02967	1.070705	0.017033	-0.04891	0.10826	TRUE	1.00E+00
38102_at	0.02968	1.07073	0.009615	-0.01468	0.07404	TRUE	1.00E+00
33880_at	0.02971	1.070804	0.041421	-0.16139	0.2208	TRUE	1.00E+00
31665_s_at	0.02971	1.070804	0.088497	-0.37857	0.438	TRUE	1.00E+00
40179_at	0.02974	1.070878	0.074512	-0.31403	0.37351	TRUE	1.00E+00
32987_at	0.02975	1.070903	0.043001	-0.16864	0.22814	TRUE	1.00E+00
40465_at	0.02985	1.071149	0.01632	-0.04545	0.10514	TRUE	1.00E+00
39457_r_at	0.03001	1.071544	0.050303	-0.20207	0.26209	TRUE	1.00E+00
39164_at	0.03011	1.071791	0.022409	-0.07328	0.13349	TRUE	1.00E+00
38422_s_at	0.03016	1.071914	0.027967	-0.09887	0.15919	TRUE	1.00E+00
34889_at	0.03018	1.071964	0.02243	-0.0733	0.13367	TRUE	1.00E+00
33043_at	0.03021	1.072038	0.036734	-0.13927	0.19968	TRUE	1.00E+00
589_at	0.03021	1.072038	0.03691	-0.14008	0.2005	TRUE	1.00E+00
1178_at	0.0303	1.07226	0.056049	-0.22829	0.28888	TRUE	1.00E+00
34929_at	0.0303	1.07226	0.028122	-0.09944	0.16004	TRUE	1.00E+00
39020_at	0.03031	1.072284	0.017489	-0.05037	0.111	TRUE	1.00E+00
275_at	0.03032	1.072309	0.042004	-0.16347	0.22411	TRUE	1.00E+00
38443_at	0.03034	1.072359	0.039383	-0.15136	0.21204	TRUE	1.00E+00
36465_at	0.03034	1.072359	0.030691	-0.11125	0.17193	TRUE	1.00E+00
32955_at	0.03036	1.072408	0.037423	-0.14229	0.20301	TRUE	1.00E+00
38473_at	0.03045	1.07263	0.014841	-0.03802	0.09892	TRUE	1.00E+00
36090_at	0.03053	1.072828	0.025836	-0.08867	0.14973	TRUE	1.00E+00
40860_s_at	0.03054	1.072852	0.049176	-0.19634	0.25741	TRUE	1.00E+00
38950_r_at	0.03055	1.072877	0.027546	-0.09653	0.15764	TRUE	1.00E+00
1685_at	0.03056	1.072902	0.027516	-0.09638	0.15751	TRUE	1.00E+00
35069_at	0.03058	1.072951	0.097334	-0.41847	0.47964	TRUE	1.00E+00
39544_at	0.03072	1.073297	0.086118	-0.3666	0.42803	TRUE	1.00E+00
228_at	0.03078	1.073446	0.027092	-0.09421	0.15577	TRUE	1.00E+00
37910_at	0.03079	1.07347	0.030493	-0.10989	0.17147	TRUE	1.00E+00
41853_at	0.03083	1.073569	0.025067	-0.08482	0.14647	TRUE	1.00E+00
37121_at	0.03083	1.073569	0.095783	-0.41107	0.47274	TRUE	1.00E+00
31561_at	0.03084	1.073594	0.047608	-0.18881	0.25048	TRUE	1.00E+00
33520_at	0.03091	1.073767	0.033356	-0.12298	0.1848	TRUE	1.00E+00
31445_at	0.03092	1.073792	0.096945	-0.41634	0.47818	TRUE	1.00E+00
31617_at	0.03096	1.07389	0.100879	-0.43446	0.49637	TRUE	1.00E+00
35142_at	0.03098	1.07394	0.023948	-0.07951	0.14146	TRUE	1.00E+00
34886_at	0.031	1.073989	0.017667	-0.0505	0.11251	TRUE	1.00E+00
32299_at	0.03101	1.074014	0.033425	-0.1232	0.18522	TRUE	1.00E+00
31600_s_at	0.03101	1.074014	0.015882	-0.04226	0.10429	TRUE	1.00E+00
40705_at	0.03102	1.074039	0.05905	-0.24142	0.30345	TRUE	1.00E+00
40473_at	0.03103	1.074064	0.027937	-0.09786	0.15992	TRUE	1.00E+00
227_g_at	0.03105	1.074113	0.004666	0.00953	0.05258	FALSE	3.57E-07
39919_at	0.03107	1.074163	0.03317	-0.12196	0.1841	TRUE	1.00E+00
33702_f_at	0.03109	1.074212	0.070741	-0.29527	0.35746	TRUE	1.00E+00
39025_at	0.0311	1.074237	0.015249	-0.03926	0.10145	TRUE	1.00E+00

31906_at	0.03113	1.074311	0.023812	-0.07872	0.14099	TRUE	1.00E+00
38783_at	0.03114	1.074336	0.066033	-0.27351	0.33578	TRUE	1.00E+00
36775_f_at	0.03116	1.074385	0.044708	-0.17511	0.23742	TRUE	1.00E+00
1172_at	0.0312	1.074484	0.0546	-0.2207	0.2831	TRUE	1.00E+00
41749_at	0.0312	1.074484	0.021089	-0.0661	0.12849	TRUE	1.00E+00
33981_at	0.03121	1.074509	0.060058	-0.24587	0.30829	TRUE	1.00E+00
769_s_at	0.03125	1.074608	0.120047	-0.52259	0.5851	TRUE	1.00E+00
39234_at	0.03128	1.074682	0.039009	-0.14869	0.21126	TRUE	1.00E+00
affx-hsac07/x003	0.03129	1.074707	0.031495	-0.11402	0.17659	TRUE	1.00E+00
41327_at	0.0313	1.074732	0.01699	-0.04709	0.10968	TRUE	1.00E+00
34653_at	0.0313	1.074732	0.038766	-0.14755	0.21015	TRUE	1.00E+00
39268_at	0.03135	1.074855	0.041186	-0.15867	0.22137	TRUE	1.00E+00
35812_at	0.03138	1.07493	0.013401	-0.03044	0.09321	TRUE	1.00E+00
34059_at	0.03139	1.074954	0.019547	-0.05879	0.12158	TRUE	1.00E+00
32731_at	0.03144	1.075078	0.023524	-0.07709	0.13997	TRUE	1.00E+00
38994_at	0.03144	1.075078	0.050221	-0.20026	0.26314	TRUE	1.00E+00
1663_at	0.03144	1.075078	0.026698	-0.09173	0.15462	TRUE	1.00E+00
35237_at	0.03144	1.075078	0.059986	-0.24531	0.30819	TRUE	1.00E+00
36210_g_at	0.03147	1.075152	0.02096	-0.06523	0.12817	TRUE	1.00E+00
40484_g_at	0.03149	1.075202	0.04108	-0.15804	0.22101	TRUE	1.00E+00
33809_at	0.03153	1.075301	0.021584	-0.06805	0.13111	TRUE	1.00E+00
38885_at	0.03153	1.075301	0.045281	-0.17738	0.24044	TRUE	1.00E+00
34644_at	0.03157	1.0754	0.028154	-0.09832	0.16146	TRUE	1.00E+00
33079_at	0.03159	1.075449	0.039037	-0.14851	0.21169	TRUE	1.00E+00
1195_s_at	0.03165	1.075598	0.029112	-0.10266	0.16596	TRUE	1.00E+00
36930_at	0.03165	1.075598	0.015267	-0.03878	0.10209	TRUE	1.00E+00
33532_at	0.03166	1.075623	0.035956	-0.13423	0.19754	TRUE	1.00E+00
37463_r_at	0.0317	1.075722	0.047567	-0.18776	0.25115	TRUE	1.00E+00
39561_at	0.03173	1.075796	0.019841	-0.05981	0.12327	TRUE	1.00E+00
32567_at	0.03173	1.075796	0.030538	-0.10916	0.17262	TRUE	1.00E+00
39509_at	0.03175	1.075846	0.023855	-0.07831	0.14181	TRUE	1.00E+00
32661_s_at	0.0318	1.07597	0.046108	-0.18093	0.24452	TRUE	1.00E+00
35485_at	0.03181	1.075994	0.057604	-0.23395	0.29757	TRUE	1.00E+00
34580_at	0.03183	1.076044	0.037323	-0.14037	0.20402	TRUE	1.00E+00
715_s_at	0.03184	1.076069	0.024662	-0.08194	0.14562	TRUE	1.00E+00
40931_at	0.03186	1.076118	0.013299	-0.0295	0.09321	TRUE	1.00E+00
37706_at	0.03194	1.076317	0.015669	-0.04036	0.10423	TRUE	1.00E+00
34708_at	0.03195	1.076341	0.050131	-0.19933	0.26323	TRUE	1.00E+00
40797_at	0.03198	1.076416	0.042784	-0.16541	0.22937	TRUE	1.00E+00
35177_at	0.03199	1.07644	0.015573	-0.03986	0.10383	TRUE	1.00E+00
32346_at	0.03201	1.07649	0.045282	-0.1769	0.24092	TRUE	1.00E+00
31727_at	0.03208	1.076664	0.047797	-0.18844	0.25259	TRUE	1.00E+00
37715_at	0.03209	1.076688	0.026397	-0.08969	0.15388	TRUE	1.00E+00
37621_at	0.0321	1.076713	0.054073	-0.21737	0.28157	TRUE	1.00E+00
1749_at	0.03211	1.076738	0.023254	-0.07518	0.13939	TRUE	1.00E+00
38907_at	0.03212	1.076763	0.039975	-0.15231	0.21655	TRUE	1.00E+00
35486_at	0.03212	1.076763	0.10438	-0.44944	0.51369	TRUE	1.00E+00
38090_at	0.03214	1.076812	0.032814	-0.11925	0.18353	TRUE	1.00E+00
2074_at	0.03215	1.076837	0.049985	-0.19847	0.26276	TRUE	1.00E+00
32098_at	0.03217	1.076887	0.044265	-0.17205	0.23639	TRUE	1.00E+00
39338_at	0.03222	1.077011	0.03752	-0.14089	0.20532	TRUE	1.00E+00

31518_i_at	0.03225	1.077085	0.056325	-0.22762	0.29211	TRUE	1.00E+00
33064_at	0.03226	1.07711	0.047866	-0.18858	0.25309	TRUE	1.00E+00
857_at	0.03226	1.07711	0.025457	-0.08519	0.14971	TRUE	1.00E+00
34530_at	0.03227	1.077135	0.028281	-0.0982	0.16275	TRUE	1.00E+00
37264_at	0.03236	1.077358	0.021317	-0.06599	0.13071	TRUE	1.00E+00
33855_at	0.03237	1.077383	0.014918	-0.03645	0.1012	TRUE	1.00E+00
39763_at	0.03242	1.077507	0.057775	-0.23413	0.29897	TRUE	1.00E+00
41060_at	0.03246	1.077606	0.086921	-0.36855	0.43348	TRUE	1.00E+00
39368_at	0.03247	1.077631	0.011586	-0.02098	0.08592	TRUE	1.00E+00
37082_at	0.03252	1.077755	0.109715	-0.47366	0.53869	TRUE	1.00E+00
38277_at	0.03254	1.077805	0.032288	-0.11643	0.1815	TRUE	1.00E+00
1818_at	0.03256	1.077854	0.018008	-0.05052	0.11564	TRUE	1.00E+00
39562_at	0.03258	1.077904	0.033573	-0.12231	0.18748	TRUE	1.00E+00
36195_at	0.03259	1.077929	0.029428	-0.10318	0.16836	TRUE	1.00E+00
35750_at	0.03272	1.078251	0.023511	-0.07575	0.14119	TRUE	1.00E+00
31683_at	0.03273	1.078276	0.030448	-0.10775	0.1732	TRUE	1.00E+00
40497_at	0.0328	1.07845	0.012858	-0.02652	0.09212	TRUE	1.00E+00
33507_g_at	0.03281	1.078475	0.039709	-0.15039	0.21601	TRUE	1.00E+00
40757_at	0.03289	1.078673	0.047847	-0.18786	0.25363	TRUE	1.00E+00
40550_at	0.03289	1.078673	0.035121	-0.12914	0.19493	TRUE	1.00E+00
32684_at	0.0329	1.078698	0.09241	-0.39344	0.45925	TRUE	1.00E+00
41576_at	0.03294	1.078798	0.05507	-0.22113	0.28701	TRUE	1.00E+00
38313_at	0.03298	1.078897	0.039139	-0.14759	0.21355	TRUE	1.00E+00
41030_at	0.03299	1.078922	0.088129	-0.3736	0.43958	TRUE	1.00E+00
36400_at	0.03299	1.078922	0.081044	-0.34091	0.4069	TRUE	1.00E+00
37080_at	0.033	1.078947	0.078246	-0.328	0.39399	TRUE	1.00E+00
37918_at	0.033	1.078947	0.093357	-0.39771	0.46371	TRUE	1.00E+00
41035_at	0.03304	1.079046	0.076102	-0.31806	0.38414	TRUE	1.00E+00
39341_at	0.03307	1.079121	0.015218	-0.03714	0.10327	TRUE	1.00E+00
38701_at	0.03308	1.079145	0.040665	-0.15453	0.2207	TRUE	1.00E+00
40033_at	0.03309	1.07917	0.022922	-0.07266	0.13884	TRUE	1.00E+00
1070_at	0.0331	1.079195	0.018507	-0.05229	0.11848	TRUE	1.00E+00
39774_at	0.03312	1.079245	0.013333	-0.02839	0.09464	TRUE	1.00E+00
1861_at	0.03314	1.079295	0.021565	-0.06636	0.13263	TRUE	1.00E+00
40102_at	0.03315	1.079319	0.021937	-0.06806	0.13436	TRUE	1.00E+00
104_at	0.03317	1.079369	0.017869	-0.04927	0.11561	TRUE	1.00E+00
35652_g_at	0.0332	1.079444	0.023303	-0.07431	0.14071	TRUE	1.00E+00
33301_g_at	0.03323	1.079518	0.01286	-0.02611	0.09256	TRUE	1.00E+00
40417_at	0.03325	1.079568	0.029755	-0.10403	0.17053	TRUE	1.00E+00
36772_at	0.03327	1.079618	0.032388	-0.11616	0.18269	TRUE	1.00E+00
31633_g_at	0.03328	1.079643	0.027149	-0.09197	0.15853	TRUE	1.00E+00
627_g_at	0.03328	1.079643	0.033542	-0.12147	0.18803	TRUE	1.00E+00
31555_at	0.03329	1.079667	0.041323	-0.15736	0.22394	TRUE	1.00E+00
39720_g_at	0.03334	1.079792	0.028362	-0.09751	0.1642	TRUE	1.00E+00
39120_at	0.03335	1.079817	0.054198	-0.21669	0.2834	TRUE	1.00E+00
188_at	0.03339	1.079916	0.037518	-0.1397	0.20648	TRUE	1.00E+00
35110_at	0.03339	1.079916	0.092282	-0.39236	0.45915	TRUE	1.00E+00
31767_at	0.03352	1.080239	0.087981	-0.37239	0.43942	TRUE	1.00E+00
33870_at	0.03352	1.080239	0.021207	-0.06432	0.13137	TRUE	1.00E+00
36986_at	0.03353	1.080264	0.035919	-0.13218	0.19925	TRUE	1.00E+00
33266_at	0.03355	1.080314	0.075354	-0.3141	0.3812	TRUE	1.00E+00

39889_at	0.03355	1.080314	0.054554	-0.21814	0.28524	TRUE	1.00E+00
35045_r_at	0.03355	1.080314	0.079109	-0.33142	0.39853	TRUE	1.00E+00
36162_at	0.03358	1.080389	0.021666	-0.06637	0.13354	TRUE	1.00E+00
33188_at	0.03359	1.080413	0.027272	-0.09223	0.15941	TRUE	1.00E+00
41040_at	0.03365	1.080563	0.014842	-0.03483	0.10212	TRUE	1.00E+00
39679_at	0.03367	1.080613	0.031603	-0.11214	0.17947	TRUE	1.00E+00
35538_at	0.03368	1.080637	0.062788	-0.256	0.32336	TRUE	1.00E+00
31984_at	0.03377	1.080861	0.127139	-0.5528	0.62033	TRUE	1.00E+00
923_at	0.03379	1.080911	0.091426	-0.38801	0.45559	TRUE	1.00E+00
35827_at	0.0338	1.080936	0.039798	-0.14981	0.21741	TRUE	1.00E+00
37727_i_at	0.03383	1.081011	0.033086	-0.11881	0.18648	TRUE	1.00E+00
160032_at	0.03388	1.081135	0.049214	-0.19317	0.26094	TRUE	1.00E+00
41078_at	0.03391	1.08121	0.00869	-0.00618	0.074	TRUE	1.00E+00
39944_at	0.03395	1.081309	0.032807	-0.1174	0.18531	TRUE	1.00E+00
31801_at	0.03399	1.081409	0.018782	-0.05266	0.12064	TRUE	1.00E+00
812_at	0.03403	1.081509	0.023929	-0.07637	0.14443	TRUE	1.00E+00
39403_at	0.03409	1.081658	0.016834	-0.04358	0.11176	TRUE	1.00E+00
33655_f_at	0.03412	1.081733	0.082663	-0.34725	0.41549	TRUE	1.00E+00
36189_at	0.03415	1.081808	0.018029	-0.04903	0.11733	TRUE	1.00E+00
32150_at	0.0342	1.081932	0.018751	-0.05231	0.12071	TRUE	1.00E+00
40904_at	0.0342	1.081932	0.071304	-0.29476	0.36317	TRUE	1.00E+00
36185_at	0.03425	1.082057	0.033831	-0.12183	0.19033	TRUE	1.00E+00
33601_at	0.03427	1.082106	0.045024	-0.17345	0.242	TRUE	1.00E+00
33294_at	0.03428	1.082131	0.021676	-0.06572	0.13429	TRUE	1.00E+00
863_g_at	0.03428	1.082131	0.033614	-0.12079	0.18936	TRUE	1.00E+00
40994_at	0.0343	1.082181	0.057228	-0.22973	0.29832	TRUE	1.00E+00
250_at	0.0343	1.082181	0.038443	-0.14306	0.21166	TRUE	1.00E+00
38784_g_at	0.03431	1.082206	0.047979	-0.18704	0.25567	TRUE	1.00E+00
34384_at	0.03434	1.082281	0.052749	-0.20902	0.2777	TRUE	1.00E+00
39265_at	0.03436	1.082331	0.084195	-0.35408	0.4228	TRUE	1.00E+00
1326_at	0.03437	1.082356	0.023201	-0.07267	0.14141	TRUE	1.00E+00
34813_at	0.03443	1.082505	0.050372	-0.19797	0.26682	TRUE	1.00E+00
36940_at	0.03445	1.082555	0.061547	-0.24951	0.3184	TRUE	1.00E+00
41426_at	0.03449	1.082655	0.039094	-0.14587	0.21485	TRUE	1.00E+00
41212_r_at	0.0345	1.08268	0.050856	-0.20013	0.26913	TRUE	1.00E+00
35780_at	0.03456	1.082829	0.028577	-0.09728	0.16641	TRUE	1.00E+00
35135_at	0.03458	1.082879	0.04061	-0.15278	0.22194	TRUE	1.00E+00
38270_at	0.0346	1.082929	0.012757	-0.02425	0.09346	TRUE	1.00E+00
38401_s_at	0.03462	1.082979	0.01539	-0.03639	0.10562	TRUE	1.00E+00
36872_at	0.03466	1.083079	0.023857	-0.0754	0.14473	TRUE	1.00E+00
183_at	0.03468	1.083129	0.058858	-0.23687	0.30623	TRUE	1.00E+00
34352_at	0.03468	1.083129	0.017832	-0.04759	0.11695	TRUE	1.00E+00
38011_at	0.03468	1.083129	0.01465	-0.03291	0.10227	TRUE	1.00E+00
41265_at	0.03471	1.083203	0.027046	-0.09007	0.15948	TRUE	1.00E+00
36207_at	0.03474	1.083278	0.009304	-0.00819	0.07766	TRUE	1.00E+00
41851_at	0.03477	1.083353	0.026675	-0.0883	0.15783	TRUE	1.00E+00
1248_at	0.03477	1.083353	0.024063	-0.07625	0.14578	TRUE	1.00E+00
723_s_at	0.03482	1.083478	0.016363	-0.04068	0.11031	TRUE	1.00E+00
1006_at	0.03484	1.083528	0.089279	-0.37706	0.44674	TRUE	1.00E+00
41042_r_at	0.03484	1.083528	0.05966	-0.24041	0.31009	TRUE	1.00E+00
35339_at	0.03484	1.083528	0.02077	-0.06098	0.13067	TRUE	1.00E+00

41068_at	0.03485	1.083553	0.020103	-0.05789	0.1276	TRUE	1.00E+00
38675_at	0.0349	1.083677	0.050251	-0.19694	0.26674	TRUE	1.00E+00
33139_s_at	0.0349	1.083677	0.040231	-0.15071	0.22051	TRUE	1.00E+00
912_s_at	0.03499	1.083902	0.030897	-0.10755	0.17754	TRUE	1.00E+00
32575_at	0.035	1.083927	0.018445	-0.0501	0.1201	TRUE	1.00E+00
35758_at	0.03501	1.083952	0.027166	-0.09032	0.16035	TRUE	1.00E+00
33865_at	0.03503	1.084002	0.01475	-0.03302	0.10308	TRUE	1.00E+00
33831_at	0.03504	1.084027	0.033936	-0.12153	0.1916	TRUE	1.00E+00
38961_at	0.03504	1.084027	0.097263	-0.41369	0.48377	TRUE	1.00E+00
31744_at	0.03505	1.084052	0.137974	-0.60151	0.6716	TRUE	1.00E+00
41240_at	0.03514	1.084276	0.031553	-0.11043	0.18071	TRUE	1.00E+00
1054_at	0.03518	1.084376	0.037238	-0.13662	0.20698	TRUE	1.00E+00
35547_at	0.03531	1.084701	0.044152	-0.16839	0.239	TRUE	1.00E+00
360_at	0.03531	1.084701	0.121046	-0.52315	0.59377	TRUE	1.00E+00
31925_s_at	0.03537	1.084851	0.078104	-0.32497	0.39571	TRUE	1.00E+00
32525_r_at	0.03543	1.085001	0.055427	-0.22029	0.29114	TRUE	1.00E+00
34247_at	0.03546	1.085076	0.045413	-0.17406	0.24497	TRUE	1.00E+00
35067_at	0.03546	1.085076	0.022042	-0.06624	0.13715	TRUE	1.00E+00
33837_at	0.03551	1.085201	0.024767	-0.07875	0.14978	TRUE	1.00E+00
1613_s_at	0.03552	1.085226	0.042122	-0.15882	0.22985	TRUE	1.00E+00
32980_f_at	0.03553	1.085251	0.039659	-0.14744	0.2185	TRUE	1.00E+00
40255_at	0.03558	1.085375	0.012895	-0.02391	0.09508	TRUE	1.00E+00
39921_at	0.03559	1.0854	0.026168	-0.08514	0.15632	TRUE	1.00E+00
41622_r_at	0.03564	1.085525	0.024683	-0.07823	0.14952	TRUE	1.00E+00
31326_at	0.03566	1.085575	0.046013	-0.17663	0.24794	TRUE	1.00E+00
34943_at	0.03567	1.0856	0.033936	-0.1209	0.19224	TRUE	1.00E+00
35790_at	0.03568	1.085625	0.01703	-0.04289	0.11425	TRUE	1.00E+00
1040_s_at	0.03572	1.085725	0.087349	-0.36727	0.43872	TRUE	1.00E+00
34349_at	0.03575	1.0858	0.0193	-0.05329	0.12479	TRUE	1.00E+00
39396_at	0.03579	1.0859	0.010996	-0.01494	0.08652	TRUE	1.00E+00
36880_at	0.03583	1.086	0.03824	-0.1406	0.21225	TRUE	1.00E+00
37333_at	0.03586	1.086075	0.033954	-0.12079	0.19251	TRUE	1.00E+00
39736_at	0.03589	1.08615	0.077966	-0.32382	0.39559	TRUE	1.00E+00
36336_s_at	0.03594	1.086276	0.047169	-0.18168	0.25356	TRUE	1.00E+00
876_at	0.03598	1.086376	0.048816	-0.18924	0.26119	TRUE	1.00E+00
706_at	0.03599	1.086401	0.028092	-0.09362	0.16559	TRUE	1.00E+00
41329_at	0.03605	1.086551	0.023062	-0.07035	0.14245	TRUE	1.00E+00
36898_r_at	0.0361	1.086676	0.035264	-0.1266	0.1988	TRUE	1.00E+00
31468_f_at	0.0361	1.086676	0.01901	-0.0516	0.12381	TRUE	1.00E+00
37311_at	0.0361	1.086676	0.012335	-0.0208	0.09301	TRUE	1.00E+00
41139_at	0.03611	1.086701	0.024028	-0.07475	0.14696	TRUE	1.00E+00
1252_at	0.03612	1.086726	0.021542	-0.06327	0.13551	TRUE	1.00E+00
35268_at	0.03613	1.086751	0.02243	-0.06735	0.13961	TRUE	1.00E+00
39259_at	0.03614	1.086776	0.025512	-0.08156	0.15384	TRUE	1.00E+00
31922_i_at	0.03619	1.086901	0.046767	-0.17957	0.25196	TRUE	1.00E+00
33096_r_at	0.0362	1.086926	0.107278	-0.45873	0.53114	TRUE	1.00E+00
40836_s_at	0.03629	1.087151	0.019039	-0.05155	0.12412	TRUE	1.00E+00
40358_at	0.03629	1.087151	0.055873	-0.22149	0.29406	TRUE	1.00E+00
873_at	0.03636	1.087327	0.044315	-0.16809	0.24081	TRUE	1.00E+00
40189_at	0.03637	1.087352	0.016241	-0.03856	0.1113	TRUE	1.00E+00
31418_at	0.03638	1.087377	0.043976	-0.16651	0.23927	TRUE	1.00E+00

40172_g_at	0.03643	1.087502	0.043462	-0.16408	0.23695	TRUE	1.00E+00
41232_at	0.03645	1.087552	0.026645	-0.08648	0.15938	TRUE	1.00E+00
35040_at	0.03646	1.087577	0.036152	-0.13034	0.20325	TRUE	1.00E+00
38167_at	0.03646	1.087577	0.078076	-0.32375	0.39667	TRUE	1.00E+00
35960_at	0.03647	1.087602	0.025381	-0.08063	0.15356	TRUE	1.00E+00
41128_at	0.03649	1.087652	0.020749	-0.05923	0.13222	TRUE	1.00E+00
37832_at	0.03649	1.087652	0.026251	-0.08462	0.15761	TRUE	1.00E+00
39092_at	0.03652	1.087727	0.021922	-0.06461	0.13766	TRUE	1.00E+00
40517_at	0.03653	1.087752	0.018944	-0.05086	0.12393	TRUE	1.00E+00
33384_at	0.03658	1.087878	0.12449	-0.53776	0.61093	TRUE	1.00E+00
32628_at	0.03659	1.087903	0.028811	-0.09633	0.16951	TRUE	1.00E+00
38372_at	0.03661	1.087953	0.011203	-0.01508	0.08829	TRUE	1.00E+00
40518_at	0.03662	1.087978	0.083918	-0.35054	0.42378	TRUE	1.00E+00
38778_at	0.03662	1.087978	0.019617	-0.05388	0.12713	TRUE	1.00E+00
1592_at	0.03669	1.088153	0.086863	-0.36406	0.43744	TRUE	1.00E+00
35816_at	0.03672	1.088228	0.020009	-0.0556	0.12903	TRUE	1.00E+00
37222_at	0.03676	1.088328	0.08939	-0.37565	0.44917	TRUE	1.00E+00
34754_at	0.03683	1.088504	0.026141	-0.08378	0.15743	TRUE	1.00E+00
41269_r_at	0.03685	1.088554	0.070603	-0.28889	0.36258	TRUE	1.00E+00
35307_at	0.03687	1.088604	0.017377	-0.0433	0.11704	TRUE	1.00E+00
1775_at	0.0369	1.088679	0.04856	-0.18713	0.26094	TRUE	1.00E+00
1633_g_at	0.03712	1.089231	0.042563	-0.15925	0.23349	TRUE	1.00E+00
38484_at	0.03715	1.089306	0.10376	-0.44156	0.51585	TRUE	1.00E+00
35471_g_at	0.03715	1.089306	0.044523	-0.16826	0.24256	TRUE	1.00E+00
34779_at	0.03716	1.089331	0.045926	-0.17472	0.24904	TRUE	1.00E+00
34839_at	0.03721	1.089457	0.052978	-0.20721	0.28163	TRUE	1.00E+00
41464_at	0.03724	1.089532	0.025815	-0.08186	0.15634	TRUE	1.00E+00
41460_at	0.03725	1.089557	0.012301	-0.0195	0.094	TRUE	1.00E+00
38582_at	0.03728	1.089632	0.066186	-0.26808	0.34263	TRUE	1.00E+00
32877_i_at	0.03729	1.089657	0.079385	-0.32897	0.40354	TRUE	1.00E+00
35207_at	0.03729	1.089657	0.054335	-0.21339	0.28797	TRUE	1.00E+00
33753_at	0.03734	1.089783	0.028006	-0.09187	0.16655	TRUE	1.00E+00
39436_at	0.03735	1.089808	0.025946	-0.08235	0.15706	TRUE	1.00E+00
39263_at	0.03737	1.089858	0.020273	-0.05616	0.1309	TRUE	1.00E+00
32462_s_at	0.03742	1.089984	0.125971	-0.54376	0.61859	TRUE	1.00E+00
38745_at	0.03743	1.090009	0.022992	-0.06865	0.1435	TRUE	1.00E+00
35767_at	0.03754	1.090285	0.017359	-0.04255	0.11763	TRUE	1.00E+00
41258_at	0.03755	1.09031	0.013046	-0.02264	0.09774	TRUE	1.00E+00
39678_at	0.03755	1.09031	0.040293	-0.14834	0.22345	TRUE	1.00E+00
38389_at	0.03762	1.090486	0.076851	-0.31694	0.39218	TRUE	1.00E+00
33054_at	0.03765	1.090561	0.057866	-0.22932	0.30461	TRUE	1.00E+00
32274_r_at	0.03767	1.090611	0.025566	-0.08028	0.15562	TRUE	1.00E+00
40133_s_at	0.03767	1.090611	0.01877	-0.04892	0.12427	TRUE	1.00E+00
31503_at	0.03773	1.090762	0.025705	-0.08087	0.15632	TRUE	1.00E+00
1750_at	0.03774	1.090787	0.011724	-0.01635	0.09183	TRUE	1.00E+00
37161_at	0.03788	1.091139	0.037149	-0.13351	0.20927	TRUE	1.00E+00
35836_at	0.03791	1.091214	0.026887	-0.08614	0.16195	TRUE	1.00E+00
38977_at	0.03795	1.091315	0.014906	-0.03082	0.10672	TRUE	1.00E+00
37302_at	0.03797	1.091365	0.054121	-0.21172	0.28766	TRUE	1.00E+00
31551_at	0.03798	1.09139	0.038376	-0.13907	0.21504	TRUE	1.00E+00
35985_at	0.03799	1.091415	0.0443	-0.16639	0.24237	TRUE	1.00E+00

38462_at	0.03799	1.091415	0.032278	-0.11093	0.18691	TRUE	1.00E+00
32480_at	0.038	1.09144	0.067929	-0.27539	0.3514	TRUE	1.00E+00
927_s_at	0.03807	1.091616	0.045488	-0.17179	0.24794	TRUE	1.00E+00
34053_at	0.03813	1.091767	0.070985	-0.28936	0.36563	TRUE	1.00E+00
36609_at	0.03818	1.091893	0.055444	-0.21762	0.29398	TRUE	1.00E+00
33101_g_at	0.0382	1.091943	0.068468	-0.27769	0.35408	TRUE	1.00E+00
38504_at	0.03821	1.091968	0.076816	-0.31619	0.39261	TRUE	1.00E+00
36031_at	0.03825	1.092069	0.022644	-0.06621	0.14272	TRUE	1.00E+00
32720_at	0.0383	1.092195	0.028262	-0.09209	0.16869	TRUE	1.00E+00
40593_at	0.03838	1.092396	0.018979	-0.04918	0.12595	TRUE	1.00E+00
40670_at	0.03846	1.092597	0.067871	-0.27467	0.35159	TRUE	1.00E+00
35216_at	0.03847	1.092622	0.02699	-0.08605	0.16298	TRUE	1.00E+00
32788_at	0.03848	1.092647	0.03806	-0.13712	0.21407	TRUE	1.00E+00
37365_at	0.03849	1.092672	0.030335	-0.10147	0.17844	TRUE	1.00E+00
40819_at	0.03851	1.092723	0.039962	-0.14586	0.22288	TRUE	1.00E+00
39817_s_at	0.03855	1.092823	0.016607	-0.03807	0.11517	TRUE	1.00E+00
40435_at	0.03857	1.092874	0.034418	-0.12022	0.19736	TRUE	1.00E+00
298_at	0.03861	1.092974	0.028904	-0.09474	0.17196	TRUE	1.00E+00
41673_at	0.03864	1.09305	0.076067	-0.3123	0.38958	TRUE	1.00E+00
361_at	0.03866	1.0931	0.028168	-0.0913	0.16861	TRUE	1.00E+00
653_at	0.03866	1.0931	0.097201	-0.40978	0.48711	TRUE	1.00E+00
40181_f_at	0.03867	1.093125	0.106718	-0.45369	0.53102	TRUE	1.00E+00
38690_at	0.03867	1.093125	0.01967	-0.05208	0.12942	TRUE	1.00E+00
32136_r_at	0.03867	1.093125	0.113892	-0.48678	0.56412	TRUE	1.00E+00
37990_at	0.03869	1.093176	0.078794	-0.32483	0.40221	TRUE	1.00E+00
37913_at	0.03871	1.093226	0.060926	-0.24238	0.31979	TRUE	1.00E+00
1868_g_at	0.03879	1.093428	0.017013	-0.0397	0.11728	TRUE	1.00E+00
41598_at	0.03882	1.093503	0.02291	-0.06688	0.14452	TRUE	1.00E+00
31591_s_at	0.03886	1.093604	0.059477	-0.23555	0.31326	TRUE	1.00E+00
37697_s_at	0.03887	1.093629	0.019621	-0.05165	0.12939	TRUE	1.00E+00
34290_f_at	0.0389	1.093705	0.028214	-0.09126	0.16907	TRUE	1.00E+00
35150_at	0.03894	1.093805	0.016638	-0.03782	0.1157	TRUE	1.00E+00
38335_at	0.03898	1.093906	0.021792	-0.06156	0.13952	TRUE	1.00E+00
39693_at	0.03901	1.093982	0.0256	-0.0791	0.15712	TRUE	1.00E+00
35520_at	0.03902	1.094007	0.040958	-0.14994	0.22798	TRUE	1.00E+00
37730_at	0.03904	1.094057	0.012164	-0.01708	0.09516	TRUE	1.00E+00
39127_f_at	0.03908	1.094158	0.02374	-0.07045	0.1486	TRUE	1.00E+00
31584_at	0.03908	1.094158	0.015813	-0.03388	0.11203	TRUE	1.00E+00
38069_at	0.03913	1.094284	0.026401	-0.08267	0.16093	TRUE	1.00E+00
508_at	0.03913	1.094284	0.021048	-0.05797	0.13624	TRUE	1.00E+00
36012_at	0.0392	1.09446	0.018888	-0.04794	0.12635	TRUE	1.00E+00
209_at	0.0393	1.094712	0.031188	-0.10458	0.18319	TRUE	1.00E+00
41122_at	0.03932	1.094763	0.026942	-0.08498	0.16362	TRUE	1.00E+00
845_at	0.03933	1.094788	0.023303	-0.06818	0.14684	TRUE	1.00E+00
36664_at	0.03936	1.094864	0.029801	-0.09813	0.17685	TRUE	1.00E+00
34052_at	0.03938	1.094914	0.029063	-0.09471	0.17346	TRUE	1.00E+00
33983_at	0.03938	1.094914	0.038809	-0.13967	0.21843	TRUE	1.00E+00
39179_at	0.03939	1.094939	0.073412	-0.2993	0.37809	TRUE	1.00E+00
32061_at	0.03942	1.095015	0.035884	-0.12613	0.20498	TRUE	1.00E+00
39548_at	0.03947	1.095141	0.036604	-0.12941	0.20834	TRUE	1.00E+00
40630_at	0.03951	1.095242	0.03787	-0.13521	0.21423	TRUE	1.00E+00

33081_at	0.03951	1.095242	0.047591	-0.18005	0.25908	TRUE	1.00E+00
32058_at	0.03952	1.095267	0.035721	-0.12528	0.20432	TRUE	1.00E+00
36399_at	0.03957	1.095393	0.035767	-0.12544	0.20458	TRUE	1.00E+00
39394_at	0.03967	1.095645	0.049662	-0.18945	0.26879	TRUE	1.00E+00
40498_g_at	0.0397	1.095721	0.005047	0.01641	0.06298	FALSE	4.65E-11
38347_at	0.03975	1.095847	0.031057	-0.10354	0.18303	TRUE	1.00E+00
39864_at	0.03975	1.095847	0.024015	-0.07105	0.15054	TRUE	1.00E+00
39860_at	0.03975	1.095847	0.027491	-0.08708	0.16658	TRUE	1.00E+00
1316_at	0.0398	1.095973	0.030236	-0.0997	0.1793	TRUE	1.00E+00
1503_at	0.03986	1.096125	0.032702	-0.11102	0.19073	TRUE	1.00E+00
37963_at	0.03987	1.09615	0.017411	-0.04045	0.1202	TRUE	1.00E+00
39137_at	0.0399	1.096226	0.044321	-0.16458	0.24438	TRUE	1.00E+00
32647_at	0.03994	1.096327	0.016989	-0.03844	0.11832	TRUE	1.00E+00
454_at	0.03995	1.096352	0.021939	-0.06127	0.14116	TRUE	1.00E+00
39039_s_at	0.03995	1.096352	0.016889	-0.03797	0.11787	TRUE	1.00E+00
37040_at	0.04	1.096478	0.032665	-0.1107	0.1907	TRUE	1.00E+00
31566_at	0.04002	1.096529	0.04367	-0.16145	0.24149	TRUE	1.00E+00
33180_at	0.0401	1.096731	0.034929	-0.12105	0.20125	TRUE	1.00E+00
33296_at	0.04011	1.096756	0.029396	-0.09552	0.17573	TRUE	1.00E+00
36121_at	0.04011	1.096756	0.017493	-0.0406	0.12081	TRUE	1.00E+00
31641_s_at	0.04012	1.096781	0.048951	-0.18572	0.26595	TRUE	1.00E+00
36024_at	0.04013	1.096806	0.072889	-0.29615	0.37641	TRUE	1.00E+00
35659_at	0.04015	1.096857	0.012818	-0.01899	0.09929	TRUE	1.00E+00
33737_f_at	0.04016	1.096882	0.028845	-0.09292	0.17324	TRUE	1.00E+00
34281_at	0.04017	1.096907	0.047213	-0.17765	0.25799	TRUE	1.00E+00
34326_at	0.0402	1.096983	0.010203	-0.00687	0.08727	TRUE	1.00E+00
41407_at	0.04023	1.097059	0.033597	-0.11477	0.19524	TRUE	1.00E+00
1914_at	0.04024	1.097084	0.05905	-0.2322	0.31267	TRUE	1.00E+00
34781_at	0.04027	1.09716	0.009638	-0.00419	0.08474	TRUE	3.70E-01
32904_at	0.04028	1.097185	0.105095	-0.44458	0.52515	TRUE	1.00E+00
39053_at	0.04029	1.097211	0.014173	-0.0251	0.10568	TRUE	1.00E+00
37876_at	0.04029	1.097211	0.027519	-0.08667	0.16725	TRUE	1.00E+00
38703_at	0.04029	1.097211	0.025646	-0.07803	0.15861	TRUE	1.00E+00
37650_at	0.04034	1.097337	0.034929	-0.12081	0.20149	TRUE	1.00E+00
39429_at	0.04034	1.097337	0.018586	-0.04541	0.12609	TRUE	1.00E+00
32106_at	0.04048	1.097691	0.039831	-0.14329	0.22424	TRUE	1.00E+00
40148_at	0.04048	1.097691	0.02713	-0.08469	0.16564	TRUE	1.00E+00
33447_at	0.04048	1.097691	0.017265	-0.03917	0.12013	TRUE	1.00E+00
34142_at	0.04051	1.097767	0.087706	-0.36413	0.44515	TRUE	1.00E+00
39397_at	0.04052	1.097792	0.022487	-0.06322	0.14426	TRUE	1.00E+00
1659_s_at	0.04054	1.097842	0.020636	-0.05467	0.13575	TRUE	1.00E+00
41380_at	0.04055	1.097868	0.014496	-0.02633	0.10743	TRUE	1.00E+00
33455_at	0.04055	1.097868	0.042284	-0.15453	0.23563	TRUE	1.00E+00
38139_at	0.04064	1.098095	0.044488	-0.16461	0.24588	TRUE	1.00E+00
34654_at	0.04068	1.098196	0.017474	-0.03994	0.12129	TRUE	1.00E+00
39405_at	0.04068	1.098196	0.010698	-0.00868	0.09004	TRUE	1.00E+00
1859_s_at	0.04069	1.098222	0.040089	-0.14426	0.22564	TRUE	1.00E+00
39497_at	0.04077	1.098424	0.041626	-0.15128	0.23282	TRUE	1.00E+00
32816_at	0.04077	1.098424	0.023315	-0.06679	0.14834	TRUE	1.00E+00
33189_at	0.04079	1.098475	0.088753	-0.36868	0.45026	TRUE	1.00E+00
37124_i_at	0.04084	1.098601	0.051126	-0.19504	0.27671	TRUE	1.00E+00

585_at	0.04088	1.098702	0.03054	-0.10002	0.18178	TRUE	1.00E+00
34892_at	0.04091	1.098778	0.050383	-0.19154	0.27335	TRUE	1.00E+00
36901_at	0.04093	1.098829	0.046971	-0.17578	0.25763	TRUE	1.00E+00
35300_at	0.04093	1.098829	0.011521	-0.01222	0.09408	TRUE	1.00E+00
35347_at	0.04095	1.098879	0.036071	-0.12547	0.20736	TRUE	1.00E+00
34938_i_at	0.04095	1.098879	0.081709	-0.33602	0.41792	TRUE	1.00E+00
35319_at	0.04097	1.09893	0.029252	-0.09399	0.17593	TRUE	1.00E+00
38330_at	0.041	1.099006	0.050925	-0.19394	0.27595	TRUE	1.00E+00
35209_at	0.04102	1.099056	0.026107	-0.07942	0.16147	TRUE	1.00E+00
41256_at	0.04106	1.099158	0.018925	-0.04625	0.12837	TRUE	1.00E+00
349_g_at	0.04107	1.099183	0.040248	-0.14462	0.22676	TRUE	1.00E+00
39028_at	0.04108	1.099208	0.02929	-0.09406	0.17621	TRUE	1.00E+00
32807_at	0.04108	1.099208	0.042886	-0.15677	0.23894	TRUE	1.00E+00
31638_at	0.04109	1.099234	0.027764	-0.087	0.16918	TRUE	1.00E+00
41515_at	0.0411	1.099259	0.063918	-0.25379	0.33599	TRUE	1.00E+00
33258_g_at	0.04111	1.099284	0.029547	-0.09521	0.17743	TRUE	1.00E+00
35038_at	0.04112	1.09931	0.128748	-0.55287	0.63511	TRUE	1.00E+00
749_at	0.04113	1.099335	0.022137	-0.061	0.14327	TRUE	1.00E+00
37282_at	0.04116	1.099411	0.135052	-0.58192	0.66423	TRUE	1.00E+00
1105_s_at	0.04118	1.099461	0.041609	-0.15079	0.23314	TRUE	1.00E+00
545_g_at	0.04119	1.099487	0.119694	-0.51103	0.59341	TRUE	1.00E+00
34302_at	0.0412	1.099512	0.031286	-0.10315	0.18554	TRUE	1.00E+00
37418_at	0.0412	1.099512	0.036094	-0.12532	0.20772	TRUE	1.00E+00
36797_at	0.04129	1.09974	0.086021	-0.35557	0.43816	TRUE	1.00E+00
34333_at	0.04131	1.099791	0.023491	-0.06706	0.14969	TRUE	1.00E+00
34385_at	0.04132	1.099816	0.025508	-0.07636	0.159	TRUE	1.00E+00
38785_at	0.04134	1.099867	0.023478	-0.06698	0.14965	TRUE	1.00E+00
35447_s_at	0.04138	1.099968	0.117467	-0.50056	0.58333	TRUE	1.00E+00
40387_at	0.0414	1.100019	0.085333	-0.3523	0.43509	TRUE	1.00E+00
36639_at	0.04143	1.100095	0.033609	-0.11363	0.19648	TRUE	1.00E+00
33393_at	0.04145	1.100145	0.023992	-0.06924	0.15213	TRUE	1.00E+00
36091_at	0.04145	1.100145	0.037228	-0.1303	0.21321	TRUE	1.00E+00
39965_at	0.04157	1.100449	0.02412	-0.06971	0.15285	TRUE	1.00E+00
1221_at	0.04166	1.100677	0.049942	-0.18875	0.27207	TRUE	1.00E+00
37917_at	0.04167	1.100703	0.016325	-0.03364	0.11699	TRUE	1.00E+00
148_at	0.04168	1.100728	0.028941	-0.09184	0.1752	TRUE	1.00E+00
39984_g_at	0.04168	1.100728	0.028537	-0.08998	0.17334	TRUE	1.00E+00
32554_s_at	0.04169	1.100753	0.03397	-0.11504	0.19841	TRUE	1.00E+00
1710_s_at	0.0417	1.100779	0.012832	-0.01751	0.1009	TRUE	1.00E+00
1557_at	0.0417	1.100779	0.097714	-0.40911	0.49251	TRUE	1.00E+00
34221_at	0.04171	1.100804	0.013739	-0.02167	0.1051	TRUE	1.00E+00
33792_at	0.04175	1.100905	0.037434	-0.13096	0.21445	TRUE	1.00E+00
32692_at	0.04175	1.100905	0.097016	-0.40584	0.48934	TRUE	1.00E+00
40453_s_at	0.04176	1.100931	0.03353	-0.11293	0.19646	TRUE	1.00E+00
34145_at	0.04176	1.100931	0.066644	-0.26571	0.34923	TRUE	1.00E+00
35197_at	0.04187	1.10121	0.022306	-0.06104	0.14478	TRUE	1.00E+00
40528_at	0.04188	1.101235	0.044082	-0.1615	0.24525	TRUE	1.00E+00
35494_at	0.04192	1.101336	0.039914	-0.14222	0.22607	TRUE	1.00E+00
35356_at	0.04201	1.101565	0.024864	-0.0727	0.15672	TRUE	1.00E+00
33141_at	0.04202	1.10159	0.036997	-0.12867	0.21271	TRUE	1.00E+00
37509_at	0.04204	1.101641	0.019604	-0.0484	0.13248	TRUE	1.00E+00

36187_at	0.04206	1.101692	0.021746	-0.05827	0.14238	TRUE	1.00E+00
32695_at	0.04207	1.101717	0.030578	-0.09901	0.18315	TRUE	1.00E+00
35311_at	0.0421	1.101793	0.029387	-0.09347	0.17768	TRUE	1.00E+00
34039_at	0.04212	1.101844	0.05766	-0.2239	0.30814	TRUE	1.00E+00
40633_at	0.04219	1.102021	0.052059	-0.19799	0.28236	TRUE	1.00E+00
31833_at	0.04219	1.102021	0.024463	-0.07067	0.15506	TRUE	1.00E+00
35921_at	0.04223	1.102123	0.038627	-0.13598	0.22044	TRUE	1.00E+00
35215_at	0.04227	1.102224	0.03135	-0.10236	0.18691	TRUE	1.00E+00
40436_g_at	0.04229	1.102275	0.034218	-0.11558	0.20015	TRUE	1.00E+00
37801_at	0.04229	1.102275	0.022523	-0.06162	0.1462	TRUE	1.00E+00
40023_at	0.04231	1.102326	0.059439	-0.23192	0.31654	TRUE	1.00E+00
32523_at	0.04231	1.102326	0.01552	-0.02929	0.11392	TRUE	1.00E+00
37834_at	0.04234	1.102402	0.06829	-0.27272	0.35741	TRUE	1.00E+00
40494_at	0.04235	1.102427	0.015496	-0.02914	0.11385	TRUE	1.00E+00
38476_at	0.04241	1.10258	0.016475	-0.0336	0.11842	TRUE	1.00E+00
41773_at	0.04241	1.10258	0.021863	-0.05845	0.14328	TRUE	1.00E+00
34994_at	0.04241	1.10258	0.030978	-0.1005	0.18533	TRUE	1.00E+00
33200_at	0.0425	1.102808	0.028775	-0.09025	0.17526	TRUE	1.00E+00
36561_at	0.04253	1.102884	0.036193	-0.12445	0.20951	TRUE	1.00E+00
32560_s_at	0.04253	1.102884	0.047663	-0.17736	0.26243	TRUE	1.00E+00
37759_at	0.04254	1.10291	0.047913	-0.17851	0.26359	TRUE	1.00E+00
32920_at	0.04257	1.102986	0.065043	-0.25751	0.34265	TRUE	1.00E+00
35492_at	0.04257	1.102986	0.029319	-0.09269	0.17784	TRUE	1.00E+00
32591_at	0.04263	1.103138	0.023328	-0.06499	0.15025	TRUE	1.00E+00
31792_at	0.04264	1.103164	0.055434	-0.21311	0.29839	TRUE	1.00E+00
37962_r_at	0.04267	1.10324	0.025129	-0.07327	0.1586	TRUE	1.00E+00
36983_f_at	0.04267	1.10324	0.097231	-0.40591	0.49125	TRUE	1.00E+00
37676_at	0.04267	1.10324	0.015575	-0.02919	0.11453	TRUE	1.00E+00
41646_at	0.04269	1.103291	0.011651	-0.01106	0.09645	TRUE	1.00E+00
32576_at	0.04276	1.103469	0.021449	-0.05619	0.14172	TRUE	1.00E+00
1642_at	0.04278	1.103519	0.024979	-0.07247	0.15802	TRUE	1.00E+00
36071_at	0.04281	1.103596	0.02673	-0.08051	0.16613	TRUE	1.00E+00
37296_at	0.04281	1.103596	0.023913	-0.06751	0.15314	TRUE	1.00E+00
40605_at	0.04282	1.103621	0.030322	-0.09707	0.18272	TRUE	1.00E+00
31872_at	0.04284	1.103672	0.017749	-0.03904	0.12473	TRUE	1.00E+00
34762_at	0.04286	1.103723	0.084735	-0.34807	0.43379	TRUE	1.00E+00
36774_f_at	0.0429	1.103824	0.034589	-0.11668	0.20248	TRUE	1.00E+00
33399_at	0.04294	1.103926	0.015965	-0.03071	0.1166	TRUE	1.00E+00
41387_r_at	0.04312	1.104384	0.034508	-0.11609	0.20232	TRUE	1.00E+00
32338_at	0.04312	1.104384	0.050804	-0.19127	0.27751	TRUE	1.00E+00
37900_at	0.04313	1.104409	0.008266	0.00499	0.08126	FALSE	2.29E-03
39262_at	0.04314	1.104435	0.014871	-0.02547	0.11175	TRUE	1.00E+00
1686_g_at	0.04316	1.104485	0.013851	-0.02074	0.10706	TRUE	1.00E+00
35008_at	0.04318	1.104536	0.035112	-0.11881	0.20517	TRUE	1.00E+00
2008_s_at	0.04318	1.104536	0.052544	-0.19924	0.2856	TRUE	1.00E+00
32445_at	0.04322	1.104638	0.029058	-0.09084	0.17729	TRUE	1.00E+00
32846_s_at	0.04326	1.10474	0.011034	-0.00764	0.09417	TRUE	1.00E+00
1632_at	0.04328	1.104791	0.062774	-0.24634	0.33289	TRUE	1.00E+00
38657_s_at	0.04331	1.104867	0.034264	-0.11477	0.20139	TRUE	1.00E+00
36446_s_at	0.04336	1.104994	0.007141	0.01041	0.07631	FALSE	1.60E-05
41028_at	0.04339	1.105071	0.032806	-0.10796	0.19474	TRUE	1.00E+00

41117_s_at	0.04344	1.105198	0.052119	-0.19702	0.28389	TRUE	1.00E+00
479_at	0.04348	1.1053	0.021165	-0.05416	0.14113	TRUE	1.00E+00
34686_at	0.04355	1.105478	0.035615	-0.12077	0.20786	TRUE	1.00E+00
41706_at	0.04358	1.105554	0.022887	-0.06201	0.14917	TRUE	1.00E+00
40741_at	0.0436	1.105605	0.049837	-0.18633	0.27353	TRUE	1.00E+00
621_at	0.04362	1.105656	0.036098	-0.12293	0.21016	TRUE	1.00E+00
37690_at	0.04363	1.105681	0.011564	-0.00972	0.09698	TRUE	1.00E+00
31890_s_at	0.04369	1.105834	0.027326	-0.08238	0.16976	TRUE	1.00E+00
921_s_at	0.04371	1.105885	0.099277	-0.41431	0.50173	TRUE	1.00E+00
40572_at	0.04376	1.106012	0.098507	-0.41072	0.49823	TRUE	1.00E+00
31456_at	0.04383	1.106191	0.023109	-0.06278	0.15045	TRUE	1.00E+00
33113_at	0.04384	1.106216	0.021413	-0.05495	0.14262	TRUE	1.00E+00
37517_at	0.04386	1.106267	0.021936	-0.05735	0.14506	TRUE	1.00E+00
32745_at	0.04387	1.106293	0.026048	-0.07631	0.16404	TRUE	1.00E+00
31729_at	0.04391	1.106394	0.077005	-0.31136	0.39918	TRUE	1.00E+00
34782_at	0.04392	1.10642	0.044868	-0.16309	0.25092	TRUE	1.00E+00
39601_at	0.04392	1.10642	0.012051	-0.01168	0.09952	TRUE	1.00E+00
31464_at	0.04393	1.106445	0.029676	-0.09299	0.18084	TRUE	1.00E+00
38844_at	0.04395	1.106496	0.030386	-0.09624	0.18413	TRUE	1.00E+00
1351_at	0.04403	1.1067	0.033041	-0.10841	0.19646	TRUE	1.00E+00
1088_at	0.04404	1.106726	0.052493	-0.19814	0.28622	TRUE	1.00E+00
36998_s_at	0.04405	1.106751	0.031363	-0.10064	0.18875	TRUE	1.00E+00
38399_at	0.0441	1.106879	0.018543	-0.04145	0.12965	TRUE	1.00E+00
34149_at	0.04422	1.107185	0.053735	-0.2037	0.29213	TRUE	1.00E+00
32967_at	0.04422	1.107185	0.028305	-0.08637	0.17481	TRUE	1.00E+00
1037_at	0.04429	1.107363	0.047731	-0.17592	0.2645	TRUE	1.00E+00
36821_at	0.0443	1.107388	0.031213	-0.0997	0.18831	TRUE	1.00E+00
35159_at	0.04432	1.107439	0.025128	-0.07161	0.16025	TRUE	1.00E+00
40006_at	0.04433	1.107465	0.042732	-0.15282	0.24147	TRUE	1.00E+00
31846_at	0.04434	1.10749	0.021149	-0.05323	0.14191	TRUE	1.00E+00
31728_at	0.04436	1.107541	0.011784	-0.01001	0.09872	TRUE	1.00E+00
40501_s_at	0.04436	1.107541	0.035864	-0.12111	0.20982	TRUE	1.00E+00
34722_at	0.0444	1.107643	0.044723	-0.16193	0.25074	TRUE	1.00E+00
33766_at	0.04442	1.107695	0.180782	-0.78964	0.87847	TRUE	1.00E+00
2091_at	0.04442	1.107695	0.012356	-0.01259	0.10143	TRUE	1.00E+00
33202_f_at	0.04446	1.107797	0.01802	-0.03868	0.12759	TRUE	1.00E+00
37320_at	0.04446	1.107797	0.014397	-0.02196	0.11088	TRUE	1.00E+00
33487_at	0.04449	1.107873	0.084692	-0.34624	0.43523	TRUE	1.00E+00
41841_at	0.04456	1.108052	0.015859	-0.0286	0.11773	TRUE	1.00E+00
41650_at	0.04457	1.108077	0.029718	-0.09254	0.18168	TRUE	1.00E+00
255_s_at	0.04457	1.108077	0.043634	-0.15674	0.24588	TRUE	1.00E+00
37113_at	0.0446	1.108154	0.045804	-0.16672	0.25592	TRUE	1.00E+00
41051_at	0.0447	1.108409	0.01033	-0.00296	0.09236	TRUE	1.91E-01
41400_at	0.04472	1.10846	0.109899	-0.46231	0.55175	TRUE	1.00E+00
1671_s_at	0.04474	1.108511	0.034323	-0.11361	0.2031	TRUE	1.00E+00
1062_g_at	0.04477	1.108588	0.078661	-0.31814	0.40768	TRUE	1.00E+00
37927_at	0.04479	1.108639	0.038985	-0.13507	0.22465	TRUE	1.00E+00
1102_s_at	0.0448	1.108664	0.021435	-0.0541	0.14369	TRUE	1.00E+00
840_at	0.04483	1.108741	0.040929	-0.14399	0.23366	TRUE	1.00E+00
1644_at	0.04484	1.108766	0.017929	-0.03788	0.12756	TRUE	1.00E+00
32409_at	0.04487	1.108843	0.0377	-0.12906	0.21881	TRUE	1.00E+00

41335_at	0.04489	1.108894	0.030925	-0.09778	0.18757	TRUE	1.00E+00
32497_s_at	0.04493	1.108996	0.024734	-0.06919	0.15904	TRUE	1.00E+00
1898_at	0.04493	1.108996	0.038579	-0.13306	0.22292	TRUE	1.00E+00
40203_at	0.04497	1.109098	0.015298	-0.02561	0.11555	TRUE	1.00E+00
1154_at	0.04498	1.109124	0.02022	-0.04831	0.13827	TRUE	1.00E+00
34764_at	0.04498	1.109124	0.016079	-0.0292	0.11916	TRUE	1.00E+00
1697_s_at	0.04502	1.109226	0.099458	-0.41384	0.50387	TRUE	1.00E+00
40445_at	0.04503	1.109251	0.036792	-0.12471	0.21478	TRUE	1.00E+00
37943_at	0.04504	1.109277	0.032695	-0.1058	0.19588	TRUE	1.00E+00
38455_at	0.04505	1.109303	0.024432	-0.06767	0.15777	TRUE	1.00E+00
34294_at	0.04508	1.109379	0.035051	-0.11663	0.2068	TRUE	1.00E+00
39079_at	0.04511	1.109456	0.018327	-0.03945	0.12966	TRUE	1.00E+00
41696_at	0.04516	1.109584	0.024073	-0.0659	0.15623	TRUE	1.00E+00
41081_at	0.04516	1.109584	0.084905	-0.34655	0.43688	TRUE	1.00E+00
32918_at	0.04517	1.109609	0.082986	-0.33769	0.42803	TRUE	1.00E+00
41809_at	0.04519	1.10966	0.034128	-0.11227	0.20264	TRUE	1.00E+00
36922_at	0.04521	1.109711	0.076362	-0.30709	0.39751	TRUE	1.00E+00
520_at	0.04524	1.109788	0.017171	-0.03398	0.12445	TRUE	1.00E+00
36230_at	0.04526	1.109839	0.08095	-0.32821	0.41873	TRUE	1.00E+00
40833_r_at	0.04526	1.109839	0.075591	-0.30348	0.394	TRUE	1.00E+00
254_at	0.04531	1.109967	0.013133	-0.01528	0.1059	TRUE	1.00E+00
31933_r_at	0.04535	1.110069	0.030756	-0.09654	0.18724	TRUE	1.00E+00
38743_f_at	0.04543	1.110274	0.013359	-0.01621	0.10706	TRUE	1.00E+00
39866_at	0.04543	1.110274	0.032089	-0.10261	0.19347	TRUE	1.00E+00
33155_at	0.04543	1.110274	0.024732	-0.06867	0.15954	TRUE	1.00E+00
40730_at	0.04547	1.110376	0.071747	-0.28554	0.37648	TRUE	1.00E+00
561_at	0.04548	1.110401	0.026061	-0.07476	0.16571	TRUE	1.00E+00
641_at	0.0455	1.110453	0.019238	-0.04325	0.13426	TRUE	1.00E+00
32559_s_at	0.04556	1.110606	0.01953	-0.04454	0.13567	TRUE	1.00E+00
33031_at	0.04561	1.110734	0.055283	-0.20945	0.30066	TRUE	1.00E+00
38062_at	0.04563	1.110785	0.044761	-0.16088	0.25214	TRUE	1.00E+00
31776_at	0.04564	1.110811	0.041069	-0.14383	0.23512	TRUE	1.00E+00
314_at	0.04569	1.110938	0.015509	-0.02586	0.11724	TRUE	1.00E+00
34242_at	0.0457	1.110964	0.082408	-0.3345	0.42589	TRUE	1.00E+00
1185_at	0.04579	1.111194	0.060265	-0.23225	0.32383	TRUE	1.00E+00
37854_at	0.04581	1.111245	0.022624	-0.05857	0.15018	TRUE	1.00E+00
41089_at	0.04587	1.111399	0.052733	-0.19742	0.28916	TRUE	1.00E+00
37038_at	0.04592	1.111527	0.021428	-0.05294	0.14478	TRUE	1.00E+00
38967_at	0.04599	1.111706	0.023777	-0.06371	0.15569	TRUE	1.00E+00
39897_at	0.04605	1.11186	0.02136	-0.0525	0.1446	TRUE	1.00E+00
31618_at	0.04606	1.111885	0.095613	-0.39506	0.48717	TRUE	1.00E+00
38669_at	0.04606	1.111885	0.025079	-0.06964	0.16176	TRUE	1.00E+00
37855_at	0.04608	1.111937	0.03455	-0.11332	0.20548	TRUE	1.00E+00
33897_at	0.04613	1.112065	0.030815	-0.09603	0.1883	TRUE	1.00E+00
35327_at	0.04614	1.11209	0.015076	-0.02341	0.1157	TRUE	1.00E+00
33443_at	0.04615	1.112116	0.021797	-0.05441	0.14672	TRUE	1.00E+00
38341_at	0.04617	1.112167	0.024815	-0.06832	0.16065	TRUE	1.00E+00
39931_at	0.04619	1.112218	0.035178	-0.1161	0.20849	TRUE	1.00E+00
40324_r_at	0.04622	1.112295	0.013323	-0.01525	0.10768	TRUE	1.00E+00
33868_at	0.04623	1.112321	0.019378	-0.04317	0.13564	TRUE	1.00E+00
1519_at	0.04624	1.112346	0.041812	-0.14667	0.23914	TRUE	1.00E+00

35656_at	0.04631	1.112526	0.013899	-0.01782	0.11043	TRUE	1.00E+00
41767_r_at	0.04634	1.112602	0.071263	-0.28244	0.37512	TRUE	1.00E+00
37829_at	0.04636	1.112654	0.121887	-0.51597	0.6087	TRUE	1.00E+00
33513_at	0.04637	1.112679	0.056763	-0.21552	0.30825	TRUE	1.00E+00
41017_at	0.0464	1.112756	0.085583	-0.34844	0.44125	TRUE	1.00E+00
35809_g_at	0.04645	1.112884	0.038261	-0.13007	0.22297	TRUE	1.00E+00
1712_s_at	0.04645	1.112884	0.062384	-0.24136	0.33426	TRUE	1.00E+00
1933_g_at	0.04649	1.112987	0.022782	-0.05862	0.15159	TRUE	1.00E+00
39430_at	0.04649	1.112987	0.017178	-0.03276	0.12574	TRUE	1.00E+00
31864_at	0.04651	1.113038	0.021508	-0.05272	0.14574	TRUE	1.00E+00
33583_r_at	0.04654	1.113115	0.061926	-0.23916	0.33224	TRUE	1.00E+00
40096_at	0.04657	1.113192	0.014027	-0.01815	0.11128	TRUE	1.00E+00
41657_at	0.04661	1.113294	0.017258	-0.03301	0.12623	TRUE	1.00E+00
590_at	0.04667	1.113448	0.045479	-0.16316	0.25649	TRUE	1.00E+00
34311_at	0.0467	1.113525	0.040125	-0.13842	0.23182	TRUE	1.00E+00
32710_at	0.04674	1.113628	0.028188	-0.08331	0.17679	TRUE	1.00E+00
396_f_at	0.04681	1.113807	0.031312	-0.09765	0.19127	TRUE	1.00E+00
36295_at	0.04684	1.113884	0.036503	-0.12157	0.21525	TRUE	1.00E+00
34441_at	0.04687	1.113961	0.063305	-0.24519	0.33894	TRUE	1.00E+00
39391_at	0.0469	1.114038	0.013366	-0.01476	0.10857	TRUE	1.00E+00
36176_at	0.04691	1.114064	0.021339	-0.05154	0.14536	TRUE	1.00E+00
692_s_at	0.04692	1.114089	0.029777	-0.09046	0.1843	TRUE	1.00E+00
34426_at	0.04696	1.114192	0.033247	-0.10642	0.20035	TRUE	1.00E+00
36219_at	0.04698	1.114243	0.020808	-0.04902	0.14297	TRUE	1.00E+00
31370_at	0.04699	1.114269	0.057373	-0.2177	0.31169	TRUE	1.00E+00
37266_at	0.047	1.114295	0.016978	-0.03133	0.12533	TRUE	1.00E+00
1471_at	0.04702	1.114346	0.082131	-0.3319	0.42594	TRUE	1.00E+00
1211_s_at	0.04704	1.114397	0.031248	-0.09712	0.19121	TRUE	1.00E+00
41197_at	0.04704	1.114397	0.014245	-0.01868	0.11276	TRUE	1.00E+00
33828_at	0.04705	1.114423	0.040545	-0.14001	0.2341	TRUE	1.00E+00
39735_at	0.04706	1.114448	0.020898	-0.04935	0.14348	TRUE	1.00E+00
32798_at	0.0471	1.114551	0.062481	-0.24116	0.33536	TRUE	1.00E+00
40110_at	0.04713	1.114628	0.022115	-0.0549	0.14916	TRUE	1.00E+00
31582_at	0.04714	1.114654	0.079279	-0.31862	0.4129	TRUE	1.00E+00
32248_at	0.04716	1.114705	0.022401	-0.05619	0.15051	TRUE	1.00E+00
36723_at	0.04721	1.114833	0.038743	-0.13153	0.22595	TRUE	1.00E+00
37758_s_at	0.04723	1.114885	0.025178	-0.06893	0.16339	TRUE	1.00E+00
36952_at	0.04727	1.114988	0.010162	0.00038	0.09415	FALSE	4.17E-02
34217_at	0.04729	1.115039	0.026445	-0.07472	0.16929	TRUE	1.00E+00
32057_at	0.04731	1.11509	0.128361	-0.54489	0.63951	TRUE	1.00E+00
39767_at	0.04733	1.115142	0.015445	-0.02393	0.11858	TRUE	1.00E+00
1846_at	0.04735	1.115193	0.02094	-0.04926	0.14396	TRUE	1.00E+00
35514_at	0.0475	1.115578	0.020043	-0.04497	0.13997	TRUE	1.00E+00
32195_at	0.04751	1.115604	0.018919	-0.03977	0.13479	TRUE	1.00E+00
34825_at	0.04751	1.115604	0.006725	0.01649	0.07854	FALSE	2.03E-08
32858_at	0.04757	1.115758	0.020013	-0.04476	0.1399	TRUE	1.00E+00
41828_at	0.04768	1.116041	0.016435	-0.02814	0.12351	TRUE	1.00E+00
33934_at	0.0478	1.116349	0.021023	-0.04919	0.14479	TRUE	1.00E+00
933_f_at	0.0478	1.116349	0.025307	-0.06895	0.16456	TRUE	1.00E+00
1303_at	0.04783	1.116426	0.069229	-0.27156	0.36723	TRUE	1.00E+00
33498_at	0.04786	1.116503	0.0872	-0.35444	0.45017	TRUE	1.00E+00

37545_at	0.04788	1.116555	0.038305	-0.12884	0.2246	TRUE	1.00E+00
32792_at	0.0479	1.116606	0.01638	-0.02767	0.12346	TRUE	1.00E+00
35710_s_at	0.0479	1.116606	0.063168	-0.24354	0.33933	TRUE	1.00E+00
1382_at	0.04792	1.116658	0.030396	-0.09232	0.18815	TRUE	1.00E+00
37681_i_at	0.04794	1.116709	0.019485	-0.04196	0.13784	TRUE	1.00E+00
1487_at	0.04805	1.116992	0.028029	-0.08126	0.17737	TRUE	1.00E+00
40246_at	0.04806	1.117018	0.037231	-0.1237	0.21983	TRUE	1.00E+00
1258_s_at	0.04807	1.117043	0.014343	-0.0181	0.11424	TRUE	1.00E+00
36517_at	0.04814	1.117223	0.025814	-0.07095	0.16724	TRUE	1.00E+00
648_at	0.04817	1.117301	0.027506	-0.07873	0.17508	TRUE	1.00E+00
40056_at	0.04819	1.117352	0.027102	-0.07684	0.17323	TRUE	1.00E+00
33932_at	0.04819	1.117352	0.054892	-0.20506	0.30144	TRUE	1.00E+00
40625_f_at	0.04829	1.117609	0.035539	-0.11568	0.21225	TRUE	1.00E+00
36394_at	0.04829	1.117609	0.038416	-0.12895	0.22553	TRUE	1.00E+00
39519_at	0.04833	1.117712	0.020817	-0.04771	0.14436	TRUE	1.00E+00
37523_at	0.04833	1.117712	0.096305	-0.39598	0.49264	TRUE	1.00E+00
35190_at	0.04834	1.117738	0.032097	-0.09975	0.19642	TRUE	1.00E+00
37072_at	0.04843	1.11797	0.05113	-0.18747	0.28432	TRUE	1.00E+00
32934_i_at	0.04847	1.118073	0.103888	-0.43082	0.52777	TRUE	1.00E+00
37733_at	0.04848	1.118098	0.018655	-0.03758	0.13455	TRUE	1.00E+00
37367_at	0.0485	1.11815	0.026243	-0.07258	0.16957	TRUE	1.00E+00
35844_at	0.04855	1.118279	0.039671	-0.13447	0.23158	TRUE	1.00E+00
38572_at	0.04856	1.118304	0.127847	-0.54128	0.63839	TRUE	1.00E+00
38375_at	0.04856	1.118304	0.016092	-0.02568	0.1228	TRUE	1.00E+00
40258_at	0.04857	1.11833	0.032628	-0.10196	0.1991	TRUE	1.00E+00
40376_at	0.04858	1.118356	0.020511	-0.04605	0.1432	TRUE	1.00E+00
35727_at	0.0486	1.118407	0.036544	-0.11999	0.2172	TRUE	1.00E+00
38522_s_at	0.04864	1.11851	0.118407	-0.49764	0.59492	TRUE	1.00E+00
40328_at	0.04865	1.118536	0.03044	-0.09178	0.18909	TRUE	1.00E+00
35446_at	0.04866	1.118562	0.082463	-0.33179	0.42911	TRUE	1.00E+00
32777_at	0.04866	1.118562	0.017594	-0.03251	0.12983	TRUE	1.00E+00
35934_at	0.0487	1.118665	0.09691	-0.3984	0.4958	TRUE	1.00E+00
39820_at	0.04871	1.118691	0.029165	-0.08584	0.18327	TRUE	1.00E+00
32751_at	0.04874	1.118768	0.019991	-0.04349	0.14098	TRUE	1.00E+00
36486_at	0.04876	1.118819	0.018606	-0.03708	0.1346	TRUE	1.00E+00
41625_at	0.04885	1.119051	0.028027	-0.08046	0.17815	TRUE	1.00E+00
33137_at	0.04886	1.119077	0.027573	-0.07835	0.17606	TRUE	1.00E+00
37081_at	0.04888	1.119129	0.100733	-0.41586	0.51362	TRUE	1.00E+00
870_f_at	0.04895	1.119309	0.054094	-0.20061	0.29852	TRUE	1.00E+00
38863_at	0.04898	1.119386	0.059804	-0.22693	0.32489	TRUE	1.00E+00
40077_at	0.04899	1.119412	0.018779	-0.03765	0.13563	TRUE	1.00E+00
781_at	0.04903	1.119515	0.02133	-0.04938	0.14743	TRUE	1.00E+00
1459_at	0.04903	1.119515	0.053226	-0.19653	0.29459	TRUE	1.00E+00
784_g_at	0.04908	1.119644	0.015965	-0.02457	0.12274	TRUE	1.00E+00
36124_at	0.04909	1.11967	0.015156	-0.02084	0.11901	TRUE	1.00E+00
37130_g_at	0.0491	1.119696	0.029439	-0.08671	0.18492	TRUE	1.00E+00
37147_at	0.04911	1.119721	0.03992	-0.13506	0.23328	TRUE	1.00E+00
35641_g_at	0.04915	1.119825	0.040506	-0.13773	0.23603	TRUE	1.00E+00
40596_at	0.0492	1.119954	0.023826	-0.06073	0.15912	TRUE	1.00E+00
260_at	0.04922	1.120005	0.020743	-0.04648	0.14492	TRUE	1.00E+00
34540_at	0.04923	1.120031	0.116416	-0.48787	0.58632	TRUE	1.00E+00

40984_at	0.04923	1.120031	0.021008	-0.04769	0.14616	TRUE	1.00E+00
34755_at	0.04926	1.120108	0.030276	-0.09042	0.18895	TRUE	1.00E+00
41512_at	0.04931	1.120237	0.035854	-0.1161	0.21473	TRUE	1.00E+00
40407_at	0.04932	1.120263	0.047863	-0.1715	0.27014	TRUE	1.00E+00
40903_at	0.04936	1.120366	0.017891	-0.03318	0.13191	TRUE	1.00E+00
34168_at	0.04938	1.120418	0.040596	-0.13792	0.23667	TRUE	1.00E+00
38756_at	0.04938	1.120418	0.027215	-0.07618	0.17494	TRUE	1.00E+00
33214_at	0.04942	1.120521	0.023727	-0.06005	0.15889	TRUE	1.00E+00
39533_at	0.04943	1.120547	0.024845	-0.06519	0.16405	TRUE	1.00E+00
36046_at	0.04944	1.120573	0.019954	-0.04262	0.1415	TRUE	1.00E+00
36870_at	0.04945	1.120598	0.02063	-0.04573	0.14463	TRUE	1.00E+00
40854_at	0.04954	1.120831	0.037149	-0.12185	0.22093	TRUE	1.00E+00
38721_at	0.04955	1.120856	0.011194	-0.0021	0.10119	TRUE	1.21E-01
34610_at	0.04955	1.120856	0.026502	-0.07272	0.17182	TRUE	1.00E+00
37633_s_at	0.04956	1.120882	0.026339	-0.07196	0.17108	TRUE	1.00E+00
32108_at	0.04966	1.12114	0.031156	-0.09408	0.1934	TRUE	1.00E+00
36884_at	0.04971	1.121269	0.138734	-0.59035	0.68977	TRUE	1.00E+00
39702_at	0.04973	1.121321	0.022084	-0.05216	0.15161	TRUE	1.00E+00
37796_at	0.04976	1.121399	0.031185	-0.09412	0.19363	TRUE	1.00E+00
36537_at	0.04976	1.121399	0.012908	-0.00979	0.10931	TRUE	1.00E+00
38667_at	0.04987	1.121683	0.021643	-0.04998	0.14972	TRUE	1.00E+00
1715_at	0.04994	1.121863	0.127393	-0.5378	0.63768	TRUE	1.00E+00
33184_at	0.04994	1.121863	0.091188	-0.37076	0.47065	TRUE	1.00E+00
36631_at	0.04994	1.121863	0.018505	-0.03543	0.13532	TRUE	1.00E+00
34815_at	0.04996	1.121915	0.033956	-0.1067	0.20662	TRUE	1.00E+00
33673_r_at	0.04998	1.121967	0.159011	-0.68363	0.78359	TRUE	1.00E+00
41428_at	0.04999	1.121993	0.014522	-0.01701	0.11699	TRUE	1.00E+00
37506_at	0.05008	1.122225	0.022994	-0.05601	0.15617	TRUE	1.00E+00
1843_at	0.0501	1.122277	0.024326	-0.06213	0.16233	TRUE	1.00E+00
1013_at	0.05012	1.122329	0.03512	-0.11191	0.21215	TRUE	1.00E+00
35431_g_at	0.05013	1.122354	0.021746	-0.0502	0.15046	TRUE	1.00E+00
40338_at	0.05014	1.12238	0.041192	-0.13991	0.24018	TRUE	1.00E+00
36001_at	0.05014	1.12238	0.024941	-0.06493	0.16521	TRUE	1.00E+00
37975_at	0.05016	1.122432	0.064762	-0.24862	0.34895	TRUE	1.00E+00
949_s_at	0.05017	1.122458	0.028338	-0.08057	0.18091	TRUE	1.00E+00
1504_s_at	0.05024	1.122639	0.038603	-0.12786	0.22834	TRUE	1.00E+00
39800_s_at	0.05024	1.122639	0.017142	-0.02884	0.12933	TRUE	1.00E+00
35608_at	0.05026	1.12269	0.090234	-0.36604	0.46657	TRUE	1.00E+00
32693_at	0.05041	1.123078	0.057306	-0.21397	0.3148	TRUE	1.00E+00
36845_at	0.05042	1.123104	0.016304	-0.0248	0.12564	TRUE	1.00E+00
36048_at	0.05042	1.123104	0.022608	-0.05389	0.15472	TRUE	1.00E+00
38306_at	0.05042	1.123104	0.016883	-0.02747	0.12832	TRUE	1.00E+00
39704_s_at	0.05043	1.12313	0.100153	-0.41164	0.51249	TRUE	1.00E+00
38704_at	0.05043	1.12313	0.024297	-0.06167	0.16253	TRUE	1.00E+00
31921_at	0.05044	1.123156	0.030645	-0.09094	0.19182	TRUE	1.00E+00
36306_at	0.05048	1.123259	0.036589	-0.11833	0.21929	TRUE	1.00E+00
1023_at	0.05052	1.123363	0.016669	-0.02638	0.12743	TRUE	1.00E+00
1450_g_at	0.05053	1.123389	0.009232	0.00794	0.09312	FALSE	5.56E-04
37692_at	0.05056	1.123466	0.039796	-0.13304	0.23416	TRUE	1.00E+00
40446_at	0.05057	1.123492	0.032803	-0.10077	0.20191	TRUE	1.00E+00
34817_s_at	0.05058	1.123518	0.036418	-0.11744	0.2186	TRUE	1.00E+00

34646_at	0.05058	1.123518	0.02248	-0.05313	0.1543	TRUE	1.00E+00
33000_at	0.05066	1.123725	0.055863	-0.20707	0.30839	TRUE	1.00E+00
31587_at	0.05068	1.123777	0.056485	-0.20992	0.31128	TRUE	1.00E+00
37563_at	0.05077	1.12401	0.022814	-0.05448	0.15603	TRUE	1.00E+00
36795_at	0.0508	1.124087	0.018838	-0.03611	0.13771	TRUE	1.00E+00
271_s_at	0.05081	1.124113	0.062594	-0.23797	0.3396	TRUE	1.00E+00
32901_s_at	0.05084	1.124191	0.018419	-0.03414	0.13581	TRUE	1.00E+00
35903_at	0.05085	1.124217	0.046369	-0.16308	0.26478	TRUE	1.00E+00
33770_at	0.05087	1.124268	0.022705	-0.05389	0.15562	TRUE	1.00E+00
40821_at	0.0509	1.124346	0.02027	-0.04261	0.14442	TRUE	1.00E+00
32351_at	0.05092	1.124398	0.022759	-0.05408	0.15592	TRUE	1.00E+00
35184_at	0.05093	1.124424	0.01077	0.00124	0.10062	FALSE	2.85E-02
41569_at	0.051	1.124605	0.052735	-0.1923	0.2943	TRUE	1.00E+00
37521_s_at	0.05103	1.124683	0.030101	-0.08784	0.1899	TRUE	1.00E+00
38611_at	0.05106	1.12476	0.112324	-0.46716	0.56927	TRUE	1.00E+00
206_at	0.05107	1.124786	0.091695	-0.37198	0.47411	TRUE	1.00E+00
32619_at	0.05113	1.124942	0.050378	-0.1813	0.28355	TRUE	1.00E+00
33078_at	0.05114	1.124968	0.024135	-0.06021	0.16249	TRUE	1.00E+00
36814_at	0.05116	1.125019	0.021981	-0.05025	0.15257	TRUE	1.00E+00
36514_at	0.05119	1.125097	0.021067	-0.04601	0.14838	TRUE	1.00E+00
38436_at	0.05119	1.125097	0.01608	-0.02299	0.12538	TRUE	1.00E+00
798_at	0.0512	1.125123	0.060055	-0.22586	0.32827	TRUE	1.00E+00
38327_at	0.05125	1.125253	0.08013	-0.31844	0.42094	TRUE	1.00E+00
37139_at	0.05127	1.125304	0.032837	-0.10023	0.20277	TRUE	1.00E+00
41471_at	0.05129	1.125356	0.07603	-0.29948	0.40206	TRUE	1.00E+00
38576_at	0.05129	1.125356	0.05929	-0.22225	0.32483	TRUE	1.00E+00
33317_at	0.0513	1.125382	0.024794	-0.06309	0.16569	TRUE	1.00E+00
1281_f_at	0.05132	1.125434	0.032133	-0.09693	0.19957	TRUE	1.00E+00
35376_f_at	0.05133	1.12546	0.055053	-0.20267	0.30532	TRUE	1.00E+00
32130_at	0.05133	1.12546	0.042272	-0.1437	0.24635	TRUE	1.00E+00
909_g_at	0.05136	1.125538	0.052195	-0.18945	0.29217	TRUE	1.00E+00
37313_at	0.05138	1.125589	0.06352	-0.24168	0.34443	TRUE	1.00E+00
34926_at	0.05143	1.125719	0.061578	-0.23267	0.33552	TRUE	1.00E+00
39688_at	0.05145	1.125771	0.028834	-0.08158	0.18448	TRUE	1.00E+00
40869_at	0.05146	1.125797	0.01723	-0.02803	0.13096	TRUE	1.00E+00
35042_at	0.05147	1.125823	0.045145	-0.1568	0.25975	TRUE	1.00E+00
34877_at	0.05148	1.125849	0.018664	-0.03463	0.13759	TRUE	1.00E+00
38224_at	0.05153	1.125978	0.018284	-0.03282	0.13589	TRUE	1.00E+00
39779_at	0.05157	1.126082	0.024194	-0.06005	0.16319	TRUE	1.00E+00
35912_at	0.0516	1.12616	0.079065	-0.31317	0.41637	TRUE	1.00E+00
34570_at	0.0516	1.12616	0.020427	-0.04264	0.14585	TRUE	1.00E+00
35540_at	0.05164	1.126263	0.05125	-0.18481	0.28808	TRUE	1.00E+00
36918_at	0.05165	1.126289	0.108742	-0.45004	0.55334	TRUE	1.00E+00
32721_at	0.05169	1.126393	0.019262	-0.03717	0.14056	TRUE	1.00E+00
35626_at	0.0517	1.126419	0.022262	-0.05101	0.15441	TRUE	1.00E+00
35801_at	0.05176	1.126575	0.024933	-0.06327	0.16679	TRUE	1.00E+00
38960_at	0.05181	1.126704	0.032302	-0.09722	0.20084	TRUE	1.00E+00
37107_at	0.05183	1.126756	0.029573	-0.0846	0.18827	TRUE	1.00E+00
32664_at	0.05188	1.126886	0.025268	-0.0647	0.16846	TRUE	1.00E+00
883_s_at	0.05189	1.126912	0.03796	-0.12324	0.22703	TRUE	1.00E+00
36474_at	0.0519	1.126938	0.029864	-0.08588	0.18967	TRUE	1.00E+00

38361_g_at	0.05191	1.126964	0.069631	-0.26934	0.37316	TRUE	1.00E+00
38457_at	0.05192	1.12699	0.03793	-0.12307	0.22692	TRUE	1.00E+00
36810_at	0.05196	1.127094	0.040486	-0.13483	0.23874	TRUE	1.00E+00
31317_r_at	0.05197	1.12712	0.096956	-0.39535	0.49929	TRUE	1.00E+00
37389_at	0.052	1.127197	0.014637	-0.01552	0.11953	TRUE	1.00E+00
37663_at	0.05202	1.127249	0.017013	-0.02647	0.13052	TRUE	1.00E+00
31360_at	0.05209	1.127431	0.075673	-0.29703	0.40122	TRUE	1.00E+00
34109_at	0.05211	1.127483	0.052018	-0.18788	0.2921	TRUE	1.00E+00
31631_f_at	0.05216	1.127613	0.066474	-0.25452	0.35884	TRUE	1.00E+00
1614_s_at	0.05221	1.127743	0.037174	-0.1193	0.22371	TRUE	1.00E+00
462_at	0.05221	1.127743	0.039404	-0.12958	0.23401	TRUE	1.00E+00
31773_at	0.05222	1.127769	0.143917	-0.61175	0.71619	TRUE	1.00E+00
38365_at	0.05232	1.128028	0.023497	-0.05609	0.16073	TRUE	1.00E+00
39172_at	0.05236	1.128132	0.021653	-0.04754	0.15225	TRUE	1.00E+00
32477_at	0.05236	1.128132	0.109124	-0.4511	0.55581	TRUE	1.00E+00
32832_at	0.05237	1.128158	0.01902	-0.03538	0.14012	TRUE	1.00E+00
33211_at	0.05237	1.128158	0.046124	-0.16042	0.26517	TRUE	1.00E+00
41467_at	0.05239	1.12821	0.054335	-0.19829	0.30307	TRUE	1.00E+00
1526_i_at	0.0524	1.128236	0.057356	-0.21222	0.31702	TRUE	1.00E+00
38777_at	0.05241	1.128262	0.079403	-0.31392	0.41874	TRUE	1.00E+00
1262_s_at	0.05241	1.128262	0.052971	-0.19197	0.29679	TRUE	1.00E+00
32780_at	0.05245	1.128366	0.017462	-0.02811	0.13302	TRUE	1.00E+00
36313_at	0.05249	1.12847	0.131977	-0.5564	0.66138	TRUE	1.00E+00
38735_at	0.05252	1.128548	0.02874	-0.08007	0.18511	TRUE	1.00E+00
31415_at	0.05252	1.128548	0.098042	-0.39981	0.50485	TRUE	1.00E+00
38595_r_at	0.05253	1.128574	0.050775	-0.18172	0.28679	TRUE	1.00E+00
40993_r_at	0.05262	1.128808	0.043944	-0.15012	0.25536	TRUE	1.00E+00
971_s_at	0.05267	1.128938	0.039933	-0.13156	0.2369	TRUE	1.00E+00
37882_at	0.05268	1.128964	0.029022	-0.08121	0.18658	TRUE	1.00E+00
39306_at	0.05268	1.128964	0.030601	-0.0885	0.19387	TRUE	1.00E+00
35265_at	0.05272	1.129068	0.033041	-0.09972	0.20516	TRUE	1.00E+00
943_at	0.05288	1.129484	0.038365	-0.12412	0.22988	TRUE	1.00E+00
33538_at	0.05295	1.129666	0.027338	-0.07318	0.17907	TRUE	1.00E+00
1448_at	0.05295	1.129666	0.019999	-0.03931	0.14522	TRUE	1.00E+00
40463_at	0.05297	1.129718	0.022075	-0.04887	0.15482	TRUE	1.00E+00
31643_at	0.05307	1.129978	0.090769	-0.3657	0.47184	TRUE	1.00E+00
1605_g_at	0.05308	1.130004	0.167856	-0.72134	0.8275	TRUE	1.00E+00
38854_at	0.05308	1.130004	0.023981	-0.05755	0.16372	TRUE	1.00E+00
39165_at	0.0531	1.130056	0.021362	-0.04545	0.15166	TRUE	1.00E+00
37948_at	0.05311	1.130082	0.017488	-0.02757	0.1338	TRUE	1.00E+00
763_at	0.05311	1.130082	0.031934	-0.09422	0.20044	TRUE	1.00E+00
39037_at	0.05318	1.130264	0.012126	-0.00276	0.10912	TRUE	1.46E-01
40442_f_at	0.05332	1.130629	0.037764	-0.12091	0.22754	TRUE	1.00E+00
1230_g_at	0.05333	1.130655	0.034468	-0.10569	0.21235	TRUE	1.00E+00
1839_at	0.05333	1.130655	0.01136	0.00092	0.10574	FALSE	3.37E-02
37802_r_at	0.05337	1.130759	0.042847	-0.14431	0.25105	TRUE	1.00E+00
501_g_at	0.05339	1.130811	0.061531	-0.23049	0.33727	TRUE	1.00E+00
41388_at	0.05344	1.130941	0.046654	-0.1618	0.26868	TRUE	1.00E+00
36158_at	0.05344	1.130941	0.022828	-0.05188	0.15876	TRUE	1.00E+00
442_at	0.05352	1.131149	0.035541	-0.11045	0.2175	TRUE	1.00E+00
40028_at	0.05353	1.131176	0.062317	-0.23398	0.34104	TRUE	1.00E+00

34608_at	0.05356	1.131254	0.018074	-0.02983	0.13694	TRUE	1.00E+00
1548_s_at	0.05358	1.131306	0.068404	-0.26201	0.36916	TRUE	1.00E+00
37577_at	0.0536	1.131358	0.070744	-0.27278	0.37998	TRUE	1.00E+00
38311_at	0.05363	1.131436	0.026807	-0.07005	0.1773	TRUE	1.00E+00
37297_at	0.05372	1.131671	0.01311	-0.00676	0.11421	TRUE	5.26E-01
39233_at	0.05374	1.131723	0.016465	-0.02222	0.1297	TRUE	1.00E+00
33886_at	0.05377	1.131801	0.023288	-0.05367	0.16121	TRUE	1.00E+00
36457_at	0.05379	1.131853	0.01779	-0.02829	0.13586	TRUE	1.00E+00
33183_at	0.0538	1.131879	0.023909	-0.05651	0.1641	TRUE	1.00E+00
37987_at	0.05382	1.131931	0.024175	-0.05771	0.16535	TRUE	1.00E+00
38656_s_at	0.05384	1.131983	0.038698	-0.1247	0.23237	TRUE	1.00E+00
38563_at	0.05384	1.131983	0.022543	-0.05016	0.15785	TRUE	1.00E+00
38963_i_at	0.05392	1.132192	0.044772	-0.15264	0.26048	TRUE	1.00E+00
435_g_at	0.05396	1.132296	0.034827	-0.10672	0.21463	TRUE	1.00E+00
40706_at	0.05404	1.132505	0.04304	-0.14453	0.25261	TRUE	1.00E+00
33814_at	0.05404	1.132505	0.020082	-0.03861	0.14669	TRUE	1.00E+00
34768_at	0.05411	1.132687	0.020023	-0.03826	0.14649	TRUE	1.00E+00
1387_at	0.05412	1.132713	0.050041	-0.17675	0.28499	TRUE	1.00E+00
1322_at	0.05415	1.132792	0.028541	-0.07753	0.18583	TRUE	1.00E+00
33135_at	0.05424	1.133026	0.017164	-0.02494	0.13343	TRUE	1.00E+00
38358_at	0.05426	1.133079	0.026595	-0.06844	0.17695	TRUE	1.00E+00
32663_at	0.05431	1.133209	0.081881	-0.32346	0.43207	TRUE	1.00E+00
39116_at	0.05437	1.133366	0.009677	0.00972	0.09901	FALSE	2.43E-04
33884_s_at	0.05443	1.133522	0.01224	-0.00205	0.1109	TRUE	1.10E-01
32031_at	0.05443	1.133522	0.024562	-0.05889	0.16775	TRUE	1.00E+00
39641_at	0.05445	1.133574	0.086589	-0.34504	0.45393	TRUE	1.00E+00
38283_at	0.05446	1.1336	0.025675	-0.064	0.17291	TRUE	1.00E+00
32375_at	0.05449	1.133679	0.011991	-0.00083	0.10981	TRUE	6.96E-02
38039_at	0.05454	1.133809	0.086736	-0.34563	0.4547	TRUE	1.00E+00
31670_s_at	0.05455	1.133835	0.021976	-0.04684	0.15594	TRUE	1.00E+00
34743_at	0.05456	1.133861	0.015539	-0.01713	0.12625	TRUE	1.00E+00
39894_f_at	0.05458	1.133914	0.057243	-0.20952	0.31867	TRUE	1.00E+00
40120_at	0.0546	1.133966	0.025864	-0.06473	0.17393	TRUE	1.00E+00
33539_at	0.0546	1.133966	0.025645	-0.06371	0.17292	TRUE	1.00E+00
41220_at	0.05462	1.134018	0.055628	-0.20203	0.31126	TRUE	1.00E+00
524_at	0.05463	1.134044	0.029955	-0.08357	0.19283	TRUE	1.00E+00
37045_at	0.05464	1.13407	0.025052	-0.06094	0.17022	TRUE	1.00E+00
35883_at	0.05465	1.134096	0.013966	-0.00979	0.11908	TRUE	1.00E+00
35346_at	0.05466	1.134123	0.021604	-0.04501	0.15433	TRUE	1.00E+00
39924_at	0.05467	1.134149	0.04495	-0.15271	0.26205	TRUE	1.00E+00
362_at	0.05469	1.134201	0.026833	-0.0691	0.17849	TRUE	1.00E+00
32218_at	0.0547	1.134227	0.026332	-0.06678	0.17619	TRUE	1.00E+00
33558_at	0.05471	1.134253	0.027302	-0.07125	0.18067	TRUE	1.00E+00
37840_at	0.05474	1.134332	0.030301	-0.08506	0.19453	TRUE	1.00E+00
34430_at	0.05476	1.134384	0.031074	-0.08861	0.19812	TRUE	1.00E+00
41305_at	0.05478	1.134436	0.040056	-0.13002	0.23958	TRUE	1.00E+00
41007_at	0.05479	1.134462	0.036709	-0.11457	0.22414	TRUE	1.00E+00
35858_at	0.05479	1.134462	0.022902	-0.05087	0.16046	TRUE	1.00E+00
38993_r_at	0.05483	1.134567	0.020692	-0.04064	0.15029	TRUE	1.00E+00
38999_s_at	0.05484	1.134593	0.02035	-0.03905	0.14872	TRUE	1.00E+00
391_at	0.05487	1.134671	0.016026	-0.01907	0.1288	TRUE	1.00E+00

36946_at	0.0549	1.134749	0.022491	-0.04886	0.15867	TRUE	1.00E+00
affx-dapx-m_at	0.05496	1.134906	0.057397	-0.20984	0.31976	TRUE	1.00E+00
36562_at	0.05503	1.135089	0.022139	-0.04711	0.15717	TRUE	1.00E+00
40386_r_at	0.05503	1.135089	0.107092	-0.43904	0.54911	TRUE	1.00E+00
41817_g_at	0.05508	1.13522	0.017537	-0.02583	0.13599	TRUE	1.00E+00
37793_r_at	0.05512	1.135324	0.024692	-0.05879	0.16904	TRUE	1.00E+00
981_at	0.05527	1.135717	0.058553	-0.21487	0.32541	TRUE	1.00E+00
584_s_at	0.0553	1.135795	0.022198	-0.04711	0.15771	TRUE	1.00E+00
33265_at	0.05532	1.135847	0.0296	-0.08124	0.19188	TRUE	1.00E+00
34482_at	0.05536	1.135952	0.035265	-0.10734	0.21805	TRUE	1.00E+00
37276_at	0.05539	1.136031	0.10708	-0.43864	0.54941	TRUE	1.00E+00
37836_at	0.0554	1.136057	0.02092	-0.04111	0.15192	TRUE	1.00E+00
36818_at	0.05545	1.136187	0.030627	-0.08585	0.19675	TRUE	1.00E+00
288_s_at	0.05546	1.136214	0.050081	-0.1756	0.28651	TRUE	1.00E+00
1521_at	0.05549	1.136292	0.043918	-0.14713	0.25811	TRUE	1.00E+00
39444_at	0.0555	1.136318	0.022035	-0.04616	0.15716	TRUE	1.00E+00
39420_at	0.05553	1.136397	0.037385	-0.11695	0.22801	TRUE	1.00E+00
1238_at	0.05557	1.136501	0.019921	-0.03634	0.14748	TRUE	1.00E+00
326_i_at	0.05559	1.136554	0.018389	-0.02925	0.14042	TRUE	1.00E+00
35322_at	0.0556	1.13658	0.014793	-0.01264	0.12385	TRUE	1.00E+00
38527_at	0.05561	1.136606	0.021335	-0.04282	0.15404	TRUE	1.00E+00
41381_at	0.05563	1.136658	0.019454	-0.03412	0.14538	TRUE	1.00E+00
35819_at	0.05564	1.136685	0.019759	-0.03552	0.1468	TRUE	1.00E+00
35993_s_at	0.05569	1.136816	0.023044	-0.05063	0.16201	TRUE	1.00E+00
40786_at	0.05572	1.136894	0.037075	-0.11533	0.22677	TRUE	1.00E+00
40898_at	0.05573	1.13692	0.033358	-0.09817	0.20963	TRUE	1.00E+00
820_at	0.05578	1.137051	0.031183	-0.08808	0.19964	TRUE	1.00E+00
31896_at	0.05578	1.137051	0.019987	-0.03643	0.148	TRUE	1.00E+00
35380_at	0.0558	1.137104	0.046394	-0.15825	0.26984	TRUE	1.00E+00
37467_at	0.05582	1.137156	0.038142	-0.12016	0.23179	TRUE	1.00E+00
36224_g_at	0.05584	1.137208	0.030215	-0.08356	0.19524	TRUE	1.00E+00
34260_at	0.05584	1.137208	0.025995	-0.06409	0.17577	TRUE	1.00E+00
40002_r_at	0.05584	1.137208	0.042392	-0.13974	0.25142	TRUE	1.00E+00
34140_at	0.05584	1.137208	0.064445	-0.24148	0.35316	TRUE	1.00E+00
34273_at	0.05591	1.137392	0.040797	-0.13231	0.24414	TRUE	1.00E+00
31841_at	0.05596	1.137523	0.099521	-0.40319	0.51511	TRUE	1.00E+00
1967_f_at	0.05599	1.137601	0.033692	-0.09946	0.21143	TRUE	1.00E+00
38033_at	0.056	1.137627	0.019408	-0.03355	0.14554	TRUE	1.00E+00
34414_at	0.05603	1.137706	0.071423	-0.27349	0.38554	TRUE	1.00E+00
37433_at	0.05604	1.137732	0.031198	-0.08789	0.19998	TRUE	1.00E+00
35970_g_at	0.0561	1.137889	0.031542	-0.08942	0.20162	TRUE	1.00E+00
34323_at	0.05611	1.137915	0.009831	0.01075	0.10147	FALSE	1.45E-04
37747_at	0.05612	1.137942	0.015414	-0.015	0.12723	TRUE	1.00E+00
41438_at	0.05616	1.138046	0.022492	-0.04761	0.15993	TRUE	1.00E+00
38472_at	0.05617	1.138073	0.009633	0.01173	0.10061	FALSE	6.96E-05
38817_at	0.05619	1.138125	0.023055	-0.05018	0.16255	TRUE	1.00E+00
36974_at	0.0562	1.138151	0.030615	-0.08505	0.19744	TRUE	1.00E+00
40457_at	0.05623	1.13823	0.022326	-0.04678	0.15923	TRUE	1.00E+00
496_s_at	0.05625	1.138282	0.036763	-0.11336	0.22586	TRUE	1.00E+00
32809_at	0.05634	1.138518	0.023197	-0.05068	0.16336	TRUE	1.00E+00
34584_at	0.05636	1.138571	0.018504	-0.02901	0.14173	TRUE	1.00E+00

41196_at	0.05637	1.138597	0.049044	-0.1699	0.28263	TRUE	1.00E+00
38325_at	0.05638	1.138623	0.022421	-0.04706	0.15982	TRUE	1.00E+00
32801_at	0.0564	1.138676	0.00995	0.01049	0.1023	FALSE	1.82E-04
40546_s_at	0.0564	1.138676	0.014844	-0.01208	0.12489	TRUE	1.00E+00
40524_at	0.0564	1.138676	0.036815	-0.11345	0.22626	TRUE	1.00E+00
1151_at	0.05642	1.138728	0.019051	-0.03148	0.14431	TRUE	1.00E+00
31576_at	0.05646	1.138833	0.109566	-0.44903	0.56195	TRUE	1.00E+00
39810_at	0.05647	1.138859	0.033585	-0.09848	0.21142	TRUE	1.00E+00
34252_at	0.05649	1.138912	0.072139	-0.27633	0.38931	TRUE	1.00E+00
39895_r_at	0.05653	1.139016	0.085521	-0.33803	0.45109	TRUE	1.00E+00
1014_at	0.05655	1.139069	0.018253	-0.02766	0.14076	TRUE	1.00E+00
32015_at	0.05658	1.139148	0.100145	-0.40545	0.5186	TRUE	1.00E+00
32064_at	0.05658	1.139148	0.01159	0.0031	0.11005	FALSE	1.33E-02
37935_at	0.05662	1.139253	0.018274	-0.02769	0.14092	TRUE	1.00E+00
33664_g_at	0.05665	1.139331	0.064086	-0.23902	0.35231	TRUE	1.00E+00
40054_at	0.05666	1.139357	0.009244	0.01401	0.0993	FALSE	1.12E-05
503_at	0.05675	1.139594	0.03403	-0.10024	0.21375	TRUE	1.00E+00
402_s_at	0.05676	1.13962	0.029795	-0.0807	0.19422	TRUE	1.00E+00
40532_at	0.05677	1.139646	0.055556	-0.19955	0.31308	TRUE	1.00E+00
1272_at	0.0568	1.139725	0.094276	-0.37815	0.49175	TRUE	1.00E+00
1527_s_at	0.05683	1.139804	0.05491	-0.1965	0.31016	TRUE	1.00E+00
34295_at	0.05688	1.139935	0.036326	-0.11071	0.22447	TRUE	1.00E+00
40983_s_at	0.05692	1.14004	0.029173	-0.07767	0.19151	TRUE	1.00E+00
36829_at	0.05692	1.14004	0.022937	-0.0489	0.16274	TRUE	1.00E+00
31599_f_at	0.05692	1.14004	0.088218	-0.35008	0.46393	TRUE	1.00E+00
32267_at	0.05701	1.140276	0.058422	-0.21252	0.32654	TRUE	1.00E+00
37054_at	0.05701	1.140276	0.036232	-0.11015	0.22417	TRUE	1.00E+00
37167_at	0.05702	1.140302	0.04741	-0.16171	0.27576	TRUE	1.00E+00
41492_r_at	0.05704	1.140355	0.048409	-0.1663	0.28037	TRUE	1.00E+00
33340_at	0.05704	1.140355	0.013093	-0.00336	0.11745	TRUE	1.67E-01
33349_at	0.05705	1.140381	0.05443	-0.19406	0.30817	TRUE	1.00E+00
36634_at	0.05715	1.140644	0.044159	-0.14658	0.26088	TRUE	1.00E+00
1581_s_at	0.05716	1.14067	0.039209	-0.12374	0.23805	TRUE	1.00E+00
41241_at	0.05717	1.140696	0.014656	-0.01045	0.12478	TRUE	1.00E+00
38301_at	0.05719	1.140749	0.03221	-0.09141	0.2058	TRUE	1.00E+00
34263_s_at	0.05726	1.140933	0.053854	-0.1912	0.30572	TRUE	1.00E+00
875_g_at	0.05733	1.141117	0.125299	-0.52075	0.63541	TRUE	1.00E+00
35988_i_at	0.05736	1.141195	0.010769	0.00767	0.10704	FALSE	1.27E-03
35286_r_at	0.05737	1.141222	0.027345	-0.06879	0.18353	TRUE	1.00E+00
38416_at	0.05737	1.141222	0.018949	-0.03005	0.1448	TRUE	1.00E+00
1583_at	0.05743	1.141379	0.02047	-0.03701	0.15187	TRUE	1.00E+00
1936_s_at	0.05747	1.141484	0.026077	-0.06284	0.17778	TRUE	1.00E+00
34898_at	0.05752	1.141616	0.096879	-0.38944	0.50448	TRUE	1.00E+00
33029_at	0.05754	1.141668	0.033935	-0.09902	0.21411	TRUE	1.00E+00
36689_at	0.05758	1.141774	0.040249	-0.12812	0.24327	TRUE	1.00E+00
32956_at	0.05758	1.141774	0.036626	-0.11139	0.22656	TRUE	1.00E+00
1673_at	0.05759	1.1418	0.030937	-0.08514	0.20033	TRUE	1.00E+00
33829_at	0.05762	1.141879	0.025544	-0.06023	0.17547	TRUE	1.00E+00
33790_at	0.05762	1.141879	0.023028	-0.04862	0.16387	TRUE	1.00E+00
33551_s_at	0.05773	1.142168	0.091363	-0.36378	0.47924	TRUE	1.00E+00
39803_s_at	0.05777	1.142273	0.036242	-0.10944	0.22498	TRUE	1.00E+00

1492_f_at	0.0578	1.142352	0.032511	-0.09219	0.20779	TRUE	1.00E+00
35817_at	0.05781	1.142378	0.088878	-0.35223	0.46786	TRUE	1.00E+00
32508_at	0.05783	1.142431	0.017751	-0.02407	0.13973	TRUE	1.00E+00
31522_f_at	0.05785	1.142484	0.046703	-0.15762	0.27332	TRUE	1.00E+00
1096_g_at	0.05786	1.14251	0.038413	-0.11936	0.23509	TRUE	1.00E+00
40792_s_at	0.0579	1.142615	0.037114	-0.11333	0.22913	TRUE	1.00E+00
31646_at	0.05793	1.142694	0.091803	-0.36561	0.48147	TRUE	1.00E+00
34844_at	0.05803	1.142957	0.070208	-0.26589	0.38194	TRUE	1.00E+00
38005_at	0.05808	1.143089	0.031452	-0.08703	0.20319	TRUE	1.00E+00
1120_at	0.05819	1.143378	0.044753	-0.14828	0.26466	TRUE	1.00E+00
37679_at	0.05831	1.143694	0.021634	-0.0415	0.15812	TRUE	1.00E+00
35288_at	0.05832	1.143721	0.061674	-0.22622	0.34286	TRUE	1.00E+00
32342_at	0.05834	1.143773	0.101324	-0.40913	0.52581	TRUE	1.00E+00
282_at	0.05835	1.1438	0.039661	-0.12463	0.24133	TRUE	1.00E+00
32518_at	0.05844	1.144037	0.020231	-0.0349	0.15178	TRUE	1.00E+00
33654_at	0.05846	1.14409	0.070657	-0.26752	0.38445	TRUE	1.00E+00
37662_at	0.05848	1.144142	0.02765	-0.06908	0.18605	TRUE	1.00E+00
34897_at	0.05848	1.144142	0.063236	-0.23326	0.35023	TRUE	1.00E+00
39035_at	0.0585	1.144195	0.018399	-0.02639	0.14338	TRUE	1.00E+00
1283_at	0.05853	1.144274	0.062008	-0.22755	0.34461	TRUE	1.00E+00
31320_at	0.05854	1.1443	0.088055	-0.34771	0.46478	TRUE	1.00E+00
36055_at	0.05854	1.1443	0.113284	-0.4641	0.58119	TRUE	1.00E+00
31812_at	0.05858	1.144406	0.023618	-0.05038	0.16755	TRUE	1.00E+00
538_at	0.0586	1.144458	0.034671	-0.10136	0.21855	TRUE	1.00E+00
31695_g_at	0.05862	1.144511	0.040351	-0.12755	0.24478	TRUE	1.00E+00
38932_at	0.05862	1.144511	0.023885	-0.05158	0.16882	TRUE	1.00E+00
39010_at	0.05864	1.144564	0.033127	-0.09419	0.21148	TRUE	1.00E+00
246_at	0.05874	1.144827	0.056382	-0.20138	0.31887	TRUE	1.00E+00
31383_at	0.05876	1.14488	0.140777	-0.59072	0.70825	TRUE	1.00E+00
39451_i_at	0.05877	1.144906	0.034041	-0.09828	0.21582	TRUE	1.00E+00
34394_at	0.05877	1.144906	0.027547	-0.06832	0.18586	TRUE	1.00E+00
33744_at	0.0589	1.145249	0.020458	-0.03549	0.15328	TRUE	1.00E+00
40252_g_at	0.05891	1.145276	0.025525	-0.05886	0.17667	TRUE	1.00E+00
38231_f_at	0.05898	1.14546	0.069681	-0.2625	0.38046	TRUE	1.00E+00
35984_at	0.05899	1.145487	0.032101	-0.08911	0.20709	TRUE	1.00E+00
34712_at	0.05903	1.145592	0.039468	-0.12306	0.24112	TRUE	1.00E+00
37132_at	0.05905	1.145645	0.036331	-0.10857	0.22667	TRUE	1.00E+00
40019_at	0.05907	1.145698	0.059421	-0.21507	0.33322	TRUE	1.00E+00
38143_at	0.05909	1.14575	0.099485	-0.3999	0.51807	TRUE	1.00E+00
36216_at	0.0591	1.145777	0.021725	-0.04113	0.15933	TRUE	1.00E+00
32534_f_at	0.05914	1.145882	0.028796	-0.07371	0.19199	TRUE	1.00E+00
39324_at	0.05922	1.146093	0.022609	-0.04509	0.16353	TRUE	1.00E+00
36856_at	0.05925	1.146173	0.044915	-0.14797	0.26646	TRUE	1.00E+00
702_f_at	0.05931	1.146331	0.063636	-0.23428	0.3529	TRUE	1.00E+00
34778_at	0.05932	1.146357	0.041654	-0.13285	0.25149	TRUE	1.00E+00
32264_at	0.05936	1.146463	0.035692	-0.10531	0.22403	TRUE	1.00E+00
2062_at	0.05937	1.146489	0.020293	-0.03426	0.15299	TRUE	1.00E+00
38834_at	0.05937	1.146489	0.025812	-0.05971	0.17846	TRUE	1.00E+00
38040_at	0.05942	1.146621	0.022583	-0.04476	0.16361	TRUE	1.00E+00
38208_at	0.05946	1.146727	0.020449	-0.03488	0.1538	TRUE	1.00E+00
39687_at	0.0595	1.146833	0.025674	-0.05895	0.17794	TRUE	1.00E+00

36642_at	0.0595	1.146833	0.093025	-0.36968	0.48867	TRUE	1.00E+00
41663_at	0.05952	1.146885	0.026975	-0.06494	0.18397	TRUE	1.00E+00
37039_at	0.05957	1.147017	0.031928	-0.08773	0.20687	TRUE	1.00E+00
37408_at	0.05957	1.147017	0.021825	-0.04112	0.16026	TRUE	1.00E+00
40673_at	0.05957	1.147017	0.026581	-0.06306	0.1822	TRUE	1.00E+00
37150_at	0.05957	1.147017	0.030263	-0.08005	0.19919	TRUE	1.00E+00
32756_at	0.05961	1.147123	0.022725	-0.04523	0.16446	TRUE	1.00E+00
1558_g_at	0.05965	1.147229	0.034657	-0.10025	0.21954	TRUE	1.00E+00
1777_at	0.05965	1.147229	0.020349	-0.03423	0.15353	TRUE	1.00E+00
36266_at	0.05974	1.147466	0.079137	-0.30536	0.42485	TRUE	1.00E+00
38989_at	0.05976	1.147519	0.040762	-0.1283	0.24782	TRUE	1.00E+00
35276_at	0.05977	1.147546	0.038457	-0.11766	0.23719	TRUE	1.00E+00
33950_g_at	0.05979	1.147599	0.023251	-0.04748	0.16706	TRUE	1.00E+00
38485_at	0.05981	1.147651	0.010726	0.01033	0.1093	FALSE	3.10E-04
38596_i_at	0.05982	1.147678	0.191748	-0.82483	0.94447	TRUE	1.00E+00
34279_at	0.05983	1.147704	0.018456	-0.02532	0.14498	TRUE	1.00E+00
41715_at	0.05983	1.147704	0.030273	-0.07984	0.1995	TRUE	1.00E+00
32937_at	0.05984	1.147731	0.09768	-0.39081	0.51049	TRUE	1.00E+00
39191_at	0.05991	1.147916	0.07226	-0.27347	0.39328	TRUE	1.00E+00
40145_at	0.05993	1.147969	0.125328	-0.51828	0.63814	TRUE	1.00E+00
36654_s_at	0.05999	1.148127	0.018568	-0.02568	0.14565	TRUE	1.00E+00
1000_at	0.06	1.148154	0.012528	0.00221	0.1178	FALSE	2.11E-02
31758_at	0.06003	1.148233	0.047634	-0.15973	0.2798	TRUE	1.00E+00
37807_at	0.06003	1.148233	0.048701	-0.16465	0.28472	TRUE	1.00E+00
36315_i_at	0.06004	1.148259	0.082694	-0.32147	0.44156	TRUE	1.00E+00
37729_at	0.06005	1.148286	0.021943	-0.04118	0.16128	TRUE	1.00E+00
39297_at	0.0601	1.148418	0.225646	-0.98093	1.10114	TRUE	1.00E+00
40940_at	0.06012	1.148471	0.0776	-0.2979	0.41814	TRUE	1.00E+00
37740_r_at	0.06012	1.148471	0.035445	-0.10341	0.22365	TRUE	1.00E+00
32498_at	0.06013	1.148497	0.025027	-0.05533	0.1756	TRUE	1.00E+00
946_at	0.06015	1.14855	0.019246	-0.02864	0.14895	TRUE	1.00E+00
34500_at	0.06026	1.148841	0.037199	-0.11136	0.23188	TRUE	1.00E+00
34180_at	0.06027	1.148868	0.031431	-0.08474	0.20528	TRUE	1.00E+00
40089_at	0.06027	1.148868	0.019825	-0.0312	0.15174	TRUE	1.00E+00
40388_at	0.06033	1.149026	0.074052	-0.28131	0.40198	TRUE	1.00E+00
36853_at	0.06034	1.149053	0.071763	-0.27075	0.39142	TRUE	1.00E+00
40182_s_at	0.06036	1.149106	0.058882	-0.21129	0.33202	TRUE	1.00E+00
31950_at	0.06037	1.149132	0.015343	-0.01042	0.13115	TRUE	1.00E+00
35371_at	0.06039	1.149185	0.019468	-0.02943	0.1502	TRUE	1.00E+00
37263_at	0.06039	1.149185	0.037146	-0.11098	0.23177	TRUE	1.00E+00
affx-muril4_at	0.06041	1.149238	0.092246	-0.36518	0.48599	TRUE	1.00E+00
41261_at	0.06042	1.149265	0.025939	-0.05926	0.18009	TRUE	1.00E+00
34063_at	0.06043	1.149291	0.026782	-0.06313	0.18399	TRUE	1.00E+00
38724_at	0.06045	1.149344	0.021501	-0.03875	0.15964	TRUE	1.00E+00
36567_at	0.06045	1.149344	0.135933	-0.56669	0.68759	TRUE	1.00E+00
1139_at	0.06052	1.149529	0.043508	-0.14021	0.26125	TRUE	1.00E+00
33120_at	0.06054	1.149582	0.017427	-0.01986	0.14094	TRUE	1.00E+00
33497_at	0.06058	1.149688	0.061058	-0.22111	0.34228	TRUE	1.00E+00
39277_at	0.06063	1.14982	0.051484	-0.17689	0.29816	TRUE	1.00E+00
35745_f_at	0.06069	1.149979	0.011699	0.00671	0.11466	FALSE	2.69E-03
160033_s_at	0.0607	1.150006	0.018252	-0.02351	0.1449	TRUE	1.00E+00

1295_at	0.06072	1.150059	0.037115	-0.11051	0.23195	TRUE	1.00E+00
37861_at	0.06075	1.150138	0.14961	-0.62949	0.75099	TRUE	1.00E+00
38275_at	0.06079	1.150244	0.015139	-0.00906	0.13063	TRUE	7.49E-01
31419_r_at	0.06079	1.150244	0.173056	-0.73761	0.8592	TRUE	1.00E+00
38941_s_at	0.06084	1.150376	0.110272	-0.44791	0.56959	TRUE	1.00E+00
39511_at	0.06089	1.150509	0.050232	-0.17086	0.29263	TRUE	1.00E+00
1949_at	0.06097	1.150721	0.054549	-0.1907	0.31263	TRUE	1.00E+00
36168_at	0.06099	1.150774	0.03298	-0.09117	0.21315	TRUE	1.00E+00
39539_at	0.06106	1.150959	0.026294	-0.06026	0.18237	TRUE	1.00E+00
40880_r_at	0.06117	1.151251	0.021167	-0.03648	0.15883	TRUE	1.00E+00
32408_s_at	0.06118	1.151277	0.020639	-0.03404	0.1564	TRUE	1.00E+00
37825_at	0.06118	1.151277	0.05224	-0.17983	0.3022	TRUE	1.00E+00
35003_at	0.06122	1.151383	0.018066	-0.02213	0.14457	TRUE	1.00E+00
41595_at	0.06124	1.151437	0.033379	-0.09275	0.21524	TRUE	1.00E+00
34887_at	0.06126	1.15149	0.013803	-0.00243	0.12494	TRUE	1.15E-01
34677_f_at	0.0613	1.151596	0.021009	-0.03563	0.15823	TRUE	1.00E+00
38319_at	0.06134	1.151702	0.092126	-0.36369	0.48637	TRUE	1.00E+00
1733_at	0.06147	1.152046	0.040192	-0.12396	0.2469	TRUE	1.00E+00
41860_at	0.06154	1.152232	0.03474	-0.09874	0.22181	TRUE	1.00E+00
34996_at	0.06154	1.152232	0.090775	-0.35725	0.48034	TRUE	1.00E+00
34201_at	0.06155	1.152259	0.023215	-0.04555	0.16865	TRUE	1.00E+00
34460_at	0.06163	1.152471	0.050275	-0.17032	0.29357	TRUE	1.00E+00
38216_at	0.06168	1.152604	0.033331	-0.09209	0.21546	TRUE	1.00E+00
39759_at	0.0617	1.152657	0.017618	-0.01958	0.14298	TRUE	1.00E+00
32803_at	0.0617	1.152657	0.024237	-0.05012	0.17352	TRUE	1.00E+00
39529_at	0.06172	1.15271	0.029572	-0.07471	0.19816	TRUE	1.00E+00
35236_g_at	0.06175	1.152789	0.044327	-0.14275	0.26626	TRUE	1.00E+00
38797_at	0.06182	1.152975	0.03787	-0.11289	0.23654	TRUE	1.00E+00
37393_at	0.06195	1.15332	0.056348	-0.19801	0.32192	TRUE	1.00E+00
39667_at	0.06197	1.153374	0.043974	-0.14091	0.26484	TRUE	1.00E+00
40972_at	0.06198	1.1534	0.035664	-0.10255	0.22652	TRUE	1.00E+00
34234_f_at	0.062	1.153453	0.026999	-0.06257	0.18656	TRUE	1.00E+00
39711_at	0.06205	1.153586	0.03412	-0.09537	0.21946	TRUE	1.00E+00
35362_at	0.06206	1.153613	0.032666	-0.08864	0.21277	TRUE	1.00E+00
34562_at	0.06209	1.153692	0.036335	-0.10555	0.22972	TRUE	1.00E+00
33982_f_at	0.0621	1.153719	0.049723	-0.1673	0.2915	TRUE	1.00E+00
39793_at	0.06212	1.153772	0.023639	-0.04694	0.17118	TRUE	1.00E+00
37098_at	0.06219	1.153958	0.024331	-0.05006	0.17444	TRUE	1.00E+00
1524_at	0.06222	1.154038	0.024683	-0.05165	0.1761	TRUE	1.00E+00
33175_at	0.06224	1.154091	0.028769	-0.07049	0.19497	TRUE	1.00E+00
36764_at	0.06233	1.15433	0.081459	-0.31349	0.43815	TRUE	1.00E+00
32834_r_at	0.06236	1.15441	0.044879	-0.14469	0.26941	TRUE	1.00E+00
39812_at	0.06238	1.154463	0.034166	-0.09525	0.22001	TRUE	1.00E+00
41491_s_at	0.06239	1.15449	0.039422	-0.11948	0.24427	TRUE	1.00E+00
34340_at	0.0624	1.154516	0.02397	-0.04819	0.17299	TRUE	1.00E+00
36438_at	0.0624	1.154516	0.104755	-0.42089	0.5457	TRUE	1.00E+00
433_at	0.06249	1.154755	0.123286	-0.5063	0.63128	TRUE	1.00E+00
32290_at	0.06251	1.154809	0.085855	-0.33359	0.45861	TRUE	1.00E+00
1721_g_at	0.06262	1.155101	0.091464	-0.35936	0.48459	TRUE	1.00E+00
41679_at	0.06266	1.155208	0.049328	-0.16492	0.29024	TRUE	1.00E+00
31903_at	0.06267	1.155234	0.030538	-0.07822	0.20356	TRUE	1.00E+00

32857_at	0.06268	1.155261	0.019519	-0.02737	0.15273	TRUE	1.00E+00
36417_s_at	0.06278	1.155527	0.021827	-0.03792	0.16348	TRUE	1.00E+00
41718_g_at	0.06278	1.155527	0.044786	-0.14384	0.26941	TRUE	1.00E+00
35670_at	0.0628	1.15558	0.06027	-0.21526	0.34086	TRUE	1.00E+00
41039_at	0.06281	1.155607	0.021572	-0.03671	0.16234	TRUE	1.00E+00
33444_at	0.06282	1.155633	0.016139	-0.01164	0.13728	TRUE	1.00E+00
1680_at	0.06283	1.15566	0.052724	-0.18042	0.30608	TRUE	1.00E+00
31710_at	0.06283	1.15566	0.079084	-0.30202	0.42769	TRUE	1.00E+00
1112_g_at	0.06287	1.155766	0.055093	-0.1913	0.31704	TRUE	1.00E+00
31454_f_at	0.06292	1.155899	0.121459	-0.49745	0.62328	TRUE	1.00E+00
39765_at	0.06297	1.156032	0.023107	-0.04363	0.16958	TRUE	1.00E+00
33126_at	0.06303	1.156192	0.009006	0.02147	0.10458	FALSE	3.28E-08
34043_at	0.06303	1.156192	0.06181	-0.22213	0.3482	TRUE	1.00E+00
37584_at	0.06306	1.156272	0.043578	-0.13799	0.26411	TRUE	1.00E+00
32494_at	0.06306	1.156272	0.057563	-0.20251	0.32863	TRUE	1.00E+00
40351_at	0.0631	1.156378	0.043846	-0.13918	0.26539	TRUE	1.00E+00
39293_at	0.06311	1.156405	0.028161	-0.06682	0.19303	TRUE	1.00E+00
35124_at	0.06314	1.156485	0.045335	-0.14601	0.2723	TRUE	1.00E+00
1959_at	0.06319	1.156618	0.026718	-0.06008	0.18646	TRUE	1.00E+00
40234_at	0.06322	1.156698	0.016777	-0.01418	0.14063	TRUE	1.00E+00
39501_f_at	0.06323	1.156725	0.066292	-0.24262	0.36907	TRUE	1.00E+00
37748_at	0.06326	1.156805	0.013364	0.0016	0.12491	FALSE	2.79E-02
1772_s_at	0.06327	1.156831	0.029271	-0.07177	0.19832	TRUE	1.00E+00
40491_at	0.06335	1.157044	0.012764	0.00447	0.12224	FALSE	8.74E-03
35687_at	0.06338	1.157124	0.053736	-0.18454	0.3113	TRUE	1.00E+00
37870_at	0.06339	1.157151	0.03365	-0.09186	0.21864	TRUE	1.00E+00
33111_at	0.06339	1.157151	0.069849	-0.25886	0.38565	TRUE	1.00E+00
39877_at	0.0634	1.157178	0.059625	-0.21168	0.33848	TRUE	1.00E+00
38053_s_at	0.0634	1.157178	0.031458	-0.08173	0.20854	TRUE	1.00E+00
36646_at	0.06344	1.157284	0.029919	-0.07459	0.20147	TRUE	1.00E+00
35672_at	0.06348	1.157391	0.023332	-0.04417	0.17112	TRUE	1.00E+00
34157_f_at	0.06349	1.157417	0.029242	-0.07142	0.1984	TRUE	1.00E+00
456_at	0.06354	1.157551	0.031406	-0.08135	0.20844	TRUE	1.00E+00
31412_at	0.06355	1.157577	0.075559	-0.28504	0.41215	TRUE	1.00E+00
34670_at	0.06356	1.157604	0.020073	-0.02904	0.15617	TRUE	1.00E+00
40207_g_at	0.06358	1.157657	0.014829	-0.00484	0.13199	TRUE	2.28E-01
40922_at	0.06358	1.157657	0.072172	-0.26939	0.39655	TRUE	1.00E+00
38070_at	0.0636	1.157711	0.029254	-0.07136	0.19857	TRUE	1.00E+00
34255_at	0.06361	1.157737	0.095597	-0.37744	0.50465	TRUE	1.00E+00
315_at	0.06362	1.157764	0.029965	-0.07463	0.20186	TRUE	1.00E+00
37732_at	0.06366	1.157871	0.030466	-0.0769	0.20422	TRUE	1.00E+00
39512_s_at	0.0637	1.157977	0.032341	-0.08551	0.2129	TRUE	1.00E+00
225_at	0.0637	1.157977	0.034126	-0.09374	0.22114	TRUE	1.00E+00
509_at	0.06371	1.158004	0.018956	-0.02374	0.15116	TRUE	1.00E+00
36348_r_at	0.06372	1.158031	0.158604	-0.66802	0.79545	TRUE	1.00E+00
32550_r_at	0.06378	1.158191	0.063022	-0.22697	0.35454	TRUE	1.00E+00
38727_at	0.06381	1.158271	0.02892	-0.06961	0.19724	TRUE	1.00E+00
38508_s_at	0.06386	1.158404	0.050223	-0.16785	0.29557	TRUE	1.00E+00
39456_at	0.0639	1.158511	0.068221	-0.25085	0.37864	TRUE	1.00E+00
37905_r_at	0.06402	1.158831	0.026005	-0.05596	0.18399	TRUE	1.00E+00
1132_s_at	0.06411	1.159071	0.096542	-0.3813	0.50951	TRUE	1.00E+00

40068_at	0.06427	1.159498	0.01802	-0.01886	0.14741	TRUE	1.00E+00
33403_at	0.06428	1.159525	0.008268	0.02613	0.10242	FALSE	9.58E-11
36151_at	0.0643	1.159578	0.037128	-0.10699	0.23559	TRUE	1.00E+00
951_at	0.06432	1.159631	0.014879	-0.00433	0.13296	TRUE	1.95E-01
1344_at	0.06434	1.159685	0.025017	-0.05108	0.17976	TRUE	1.00E+00
39737_at	0.06434	1.159685	0.100277	-0.39829	0.52698	TRUE	1.00E+00
39426_at	0.06436	1.159738	0.012116	0.00846	0.12026	FALSE	1.37E-03
33191_at	0.06436	1.159738	0.035297	-0.09849	0.22721	TRUE	1.00E+00
1626_at	0.06437	1.159765	0.091225	-0.35651	0.48524	TRUE	1.00E+00
32118_at	0.06438	1.159792	0.034359	-0.09414	0.22289	TRUE	1.00E+00
41816_at	0.06442	1.159899	0.035373	-0.09878	0.22762	TRUE	1.00E+00
36126_at	0.0645	1.160112	0.032973	-0.08763	0.21662	TRUE	1.00E+00
1420_s_at	0.06452	1.160166	0.023846	-0.04549	0.17454	TRUE	1.00E+00
35211_at	0.06454	1.160219	0.042296	-0.13059	0.25967	TRUE	1.00E+00
31547_at	0.06456	1.160273	0.079459	-0.30203	0.43115	TRUE	1.00E+00
32727_at	0.06458	1.160326	0.145345	-0.60598	0.73514	TRUE	1.00E+00
1136_at	0.06461	1.160406	0.05581	-0.19288	0.3221	TRUE	1.00E+00
35882_at	0.06461	1.160406	0.077365	-0.29232	0.42154	TRUE	1.00E+00
39823_at	0.06462	1.160433	0.029481	-0.07139	0.20063	TRUE	1.00E+00
36538_at	0.06462	1.160433	0.0688	-0.25279	0.38204	TRUE	1.00E+00
39870_at	0.06463	1.16046	0.028765	-0.06808	0.19734	TRUE	1.00E+00
38638_at	0.06465	1.160513	0.03149	-0.08063	0.20993	TRUE	1.00E+00
37171_at	0.06469	1.16062	0.145876	-0.60832	0.7377	TRUE	1.00E+00
39419_at	0.06475	1.16078	0.032208	-0.08384	0.21334	TRUE	1.00E+00
36283_at	0.06476	1.160807	0.048649	-0.15969	0.2892	TRUE	1.00E+00
36572_r_at	0.06477	1.160834	0.032943	-0.08721	0.21676	TRUE	1.00E+00
1473_s_at	0.06477	1.160834	0.137398	-0.56912	0.69867	TRUE	1.00E+00
40997_at	0.06479	1.160887	0.035851	-0.10062	0.23019	TRUE	1.00E+00
32068_at	0.06482	1.160967	0.100989	-0.4011	0.53074	TRUE	1.00E+00
35581_at	0.06489	1.161154	0.075867	-0.28513	0.41491	TRUE	1.00E+00
37116_at	0.06491	1.161208	0.065258	-0.23617	0.36598	TRUE	1.00E+00
34796_at	0.06496	1.161342	0.024556	-0.04833	0.17825	TRUE	1.00E+00
37196_at	0.06497	1.161368	0.149573	-0.62509	0.75504	TRUE	1.00E+00
39232_at	0.065	1.161449	0.037553	-0.10826	0.23825	TRUE	1.00E+00
32733_at	0.06501	1.161475	0.024215	-0.0467	0.17673	TRUE	1.00E+00
36392_at	0.06503	1.161529	0.038898	-0.11443	0.24448	TRUE	1.00E+00
36932_at	0.06505	1.161582	0.022006	-0.03648	0.16658	TRUE	1.00E+00
40968_at	0.06506	1.161609	0.024636	-0.0486	0.17872	TRUE	1.00E+00
41768_at	0.06506	1.161609	0.018924	-0.02225	0.15237	TRUE	1.00E+00
39725_at	0.0651	1.161716	0.038282	-0.11152	0.24172	TRUE	1.00E+00
1890_at	0.06513	1.161796	0.040557	-0.12198	0.25224	TRUE	1.00E+00
37426_at	0.06517	1.161903	0.096587	-0.38044	0.51078	TRUE	1.00E+00
34942_at	0.06519	1.161957	0.162369	-0.68391	0.8143	TRUE	1.00E+00
2093_s_at	0.06522	1.162037	0.024871	-0.04952	0.17996	TRUE	1.00E+00
34526_s_at	0.06522	1.162037	0.065495	-0.23695	0.36739	TRUE	1.00E+00
36412_s_at	0.06523	1.162064	0.044795	-0.14143	0.27189	TRUE	1.00E+00
38978_at	0.06532	1.162305	0.031284	-0.07901	0.20965	TRUE	1.00E+00
39783_at	0.06533	1.162331	0.020223	-0.02797	0.15863	TRUE	1.00E+00
33711_at	0.06536	1.162412	0.059134	-0.20746	0.33818	TRUE	1.00E+00
38295_at	0.06536	1.162412	0.109022	-0.43762	0.56834	TRUE	1.00E+00
40016_g_at	0.06538	1.162465	0.05044	-0.16733	0.29809	TRUE	1.00E+00

149_at	0.06539	1.162492	0.034719	-0.09479	0.22558	TRUE	1.00E+00
38075_at	0.06554	1.162894	0.009273	0.02276	0.10832	FALSE	1.99E-08
36647_at	0.06562	1.163108	0.025363	-0.0514	0.18263	TRUE	1.00E+00
36109_at	0.06564	1.163161	0.014605	-0.00175	0.13302	TRUE	8.83E-02
41855_at	0.06565	1.163188	0.02887	-0.06755	0.19885	TRUE	1.00E+00
34582_at	0.06566	1.163215	0.080318	-0.3049	0.43621	TRUE	1.00E+00
36613_at	0.06575	1.163456	0.016264	-0.00929	0.14079	TRUE	6.67E-01
36218_g_at	0.06576	1.163483	0.013653	0.00277	0.12875	FALSE	1.84E-02
36038_r_at	0.06579	1.163563	0.081246	-0.30905	0.44063	TRUE	1.00E+00
41055_at	0.0658	1.16359	0.056599	-0.19532	0.32692	TRUE	1.00E+00
34497_at	0.06581	1.163617	0.043075	-0.13292	0.26454	TRUE	1.00E+00
37555_at	0.06581	1.163617	0.043277	-0.13385	0.26547	TRUE	1.00E+00
39681_at	0.06584	1.163697	0.058111	-0.20227	0.33394	TRUE	1.00E+00
37713_at	0.06584	1.163697	0.024466	-0.04703	0.17872	TRUE	1.00E+00
37073_at	0.06585	1.163724	0.070432	-0.25909	0.3908	TRUE	1.00E+00
34705_at	0.06587	1.163778	0.032291	-0.08311	0.21484	TRUE	1.00E+00
35820_at	0.06589	1.163831	0.035241	-0.0967	0.22848	TRUE	1.00E+00
33550_at	0.0659	1.163858	0.062893	-0.22426	0.35606	TRUE	1.00E+00
35171_at	0.0659	1.163858	0.02596	-0.05387	0.18567	TRUE	1.00E+00
38120_at	0.06599	1.164099	0.037701	-0.10795	0.23992	TRUE	1.00E+00
39096_at	0.06599	1.164099	0.035352	-0.09711	0.22909	TRUE	1.00E+00
34865_at	0.066	1.164126	0.02481	-0.04846	0.18047	TRUE	1.00E+00
41189_at	0.06612	1.164448	0.05742	-0.1988	0.33103	TRUE	1.00E+00
36747_at	0.06614	1.164501	0.02984	-0.07152	0.20381	TRUE	1.00E+00
39088_at	0.06626	1.164823	0.014586	-0.00103	0.13355	TRUE	7.01E-02
41304_at	0.06639	1.165172	0.059504	-0.20814	0.34092	TRUE	1.00E+00
36786_at	0.06641	1.165226	0.022385	-0.03686	0.16969	TRUE	1.00E+00
34382_at	0.06644	1.165306	0.062935	-0.22392	0.3568	TRUE	1.00E+00
33602_at	0.06648	1.165413	0.059914	-0.20993	0.3429	TRUE	1.00E+00
782_at	0.06649	1.16544	0.020279	-0.02707	0.16005	TRUE	1.00E+00
38532_at	0.06649	1.16544	0.067964	-0.24706	0.38005	TRUE	1.00E+00
39344_at	0.06651	1.165494	0.013116	0.00599	0.12702	FALSE	5.01E-03
40758_at	0.06651	1.165494	0.047878	-0.15438	0.2874	TRUE	1.00E+00
33119_at	0.06654	1.165574	0.109846	-0.44024	0.57333	TRUE	1.00E+00
36461_at	0.06654	1.165574	0.020643	-0.02869	0.16178	TRUE	1.00E+00
32671_at	0.06657	1.165655	0.043608	-0.13462	0.26776	TRUE	1.00E+00
38802_at	0.06662	1.165789	0.016929	-0.01148	0.14472	TRUE	1.00E+00
34269_at	0.06662	1.165789	0.015543	-0.00509	0.13833	TRUE	2.29E-01
1863_s_at	0.0667	1.166004	0.026461	-0.05538	0.18878	TRUE	1.00E+00
34399_at	0.0667	1.166004	0.075466	-0.28146	0.41487	TRUE	1.00E+00
36824_at	0.0668	1.166272	0.158035	-0.66231	0.79591	TRUE	1.00E+00
33027_at	0.06681	1.166299	0.033788	-0.08907	0.2227	TRUE	1.00E+00
37032_at	0.06684	1.16638	0.046595	-0.14813	0.28181	TRUE	1.00E+00
33324_s_at	0.0669	1.166541	0.095707	-0.37465	0.50846	TRUE	1.00E+00
31466_at	0.06692	1.166595	0.099112	-0.39034	0.52418	TRUE	1.00E+00
40045_g_at	0.06692	1.166595	0.013685	0.00378	0.13006	FALSE	1.27E-02
41662_at	0.06694	1.166648	0.019146	-0.02139	0.15527	TRUE	1.00E+00
31702_at	0.06695	1.166675	0.054588	-0.1849	0.31879	TRUE	1.00E+00
1986_at	0.06695	1.166675	0.030516	-0.07384	0.20774	TRUE	1.00E+00
32361_s_at	0.06696	1.166702	0.032992	-0.08525	0.21918	TRUE	1.00E+00
35342_at	0.067	1.16681	0.051349	-0.16991	0.3039	TRUE	1.00E+00

39379_at	0.06703	1.16689	0.024341	-0.04527	0.17933	TRUE	1.00E+00
37347_at	0.06705	1.166944	0.048069	-0.15471	0.28882	TRUE	1.00E+00
34314_at	0.06708	1.167025	0.04579	-0.14418	0.27834	TRUE	1.00E+00
41399_at	0.06708	1.167025	0.022783	-0.03803	0.17219	TRUE	1.00E+00
477_at	0.06715	1.167213	0.050972	-0.16801	0.30232	TRUE	1.00E+00
36657_at	0.06721	1.167374	0.061596	-0.21697	0.35139	TRUE	1.00E+00
34964_at	0.06723	1.167428	0.085885	-0.32901	0.46347	TRUE	1.00E+00
32767_at	0.06725	1.167481	0.064969	-0.23249	0.36699	TRUE	1.00E+00
37010_at	0.06727	1.167535	0.02589	-0.05218	0.18671	TRUE	1.00E+00
35018_at	0.06736	1.167777	0.054835	-0.18562	0.32035	TRUE	1.00E+00
37527_at	0.06743	1.167965	0.012703	0.00883	0.12604	FALSE	1.40E-03
37942_at	0.06748	1.1681	0.042215	-0.12728	0.26224	TRUE	1.00E+00
33194_at	0.0675	1.168154	0.050459	-0.1653	0.3003	TRUE	1.00E+00
33197_at	0.0675	1.168154	0.040879	-0.1211	0.2561	TRUE	1.00E+00
31838_at	0.06751	1.168181	0.041421	-0.12359	0.2586	TRUE	1.00E+00
32587_at	0.06753	1.168234	0.027521	-0.05944	0.1945	TRUE	1.00E+00
31699_at	0.06755	1.168288	0.023445	-0.04061	0.17572	TRUE	1.00E+00
35398_at	0.06756	1.168315	0.026715	-0.05569	0.19081	TRUE	1.00E+00
37617_at	0.0676	1.168423	0.01195	0.01247	0.12273	FALSE	1.94E-04
37403_at	0.06761	1.16845	0.005421	0.0426	0.09262	FALSE	1.33E-31
37059_at	0.06764	1.16853	0.055052	-0.18635	0.32163	TRUE	1.00E+00
576_at	0.06768	1.168638	0.121601	-0.49333	0.6287	TRUE	1.00E+00
39319_at	0.06768	1.168638	0.104611	-0.41495	0.55032	TRUE	1.00E+00
34481_at	0.0677	1.168692	0.074658	-0.27674	0.41214	TRUE	1.00E+00
852_at	0.0678	1.168961	0.078443	-0.2941	0.42971	TRUE	1.00E+00
37226_at	0.06785	1.169096	0.012953	0.0081	0.12761	FALSE	2.04E-03
1079_g_at	0.06789	1.169203	0.096392	-0.37682	0.51261	TRUE	1.00E+00
34532_at	0.0679	1.16923	0.030841	-0.07439	0.21018	TRUE	1.00E+00
1101_at	0.06791	1.169257	0.026629	-0.05495	0.19076	TRUE	1.00E+00
36746_s_at	0.06799	1.169472	0.019976	-0.02417	0.16015	TRUE	1.00E+00
32646_at	0.068	1.169499	0.020096	-0.02472	0.16071	TRUE	1.00E+00
32495_at	0.06801	1.169526	0.063031	-0.22279	0.3588	TRUE	1.00E+00
1810_s_at	0.06807	1.169688	0.119583	-0.48364	0.61977	TRUE	1.00E+00
2010_at	0.06809	1.169742	0.006515	0.03804	0.09815	FALSE	1.82E-21
40067_at	0.06815	1.169903	0.028593	-0.06377	0.20006	TRUE	1.00E+00
38685_at	0.06828	1.170254	0.049047	-0.158	0.29457	TRUE	1.00E+00
450_g_at	0.06829	1.170281	0.037243	-0.10354	0.24011	TRUE	1.00E+00
36104_at	0.06835	1.170442	0.019509	-0.02166	0.15836	TRUE	1.00E+00
35467_g_at	0.06837	1.170496	0.029831	-0.06925	0.206	TRUE	1.00E+00
449_at	0.06839	1.17055	0.019944	-0.02363	0.1604	TRUE	1.00E+00
35771_at	0.06839	1.17055	0.027875	-0.06021	0.19699	TRUE	1.00E+00
33474_at	0.06839	1.17055	0.080583	-0.30339	0.44016	TRUE	1.00E+00
160023_at	0.0684	1.170577	0.051078	-0.16725	0.30406	TRUE	1.00E+00
36835_at	0.06848	1.170793	0.043455	-0.132	0.26897	TRUE	1.00E+00
816_g_at	0.06854	1.170954	0.05798	-0.19895	0.33603	TRUE	1.00E+00
36196_at	0.06858	1.171062	0.043312	-0.13124	0.26841	TRUE	1.00E+00
710_at	0.06859	1.171089	0.042197	-0.12609	0.26327	TRUE	1.00E+00
33742_f_at	0.06863	1.171197	0.031787	-0.07802	0.21529	TRUE	1.00E+00
39935_at	0.06867	1.171305	0.117635	-0.47405	0.61139	TRUE	1.00E+00
756_at	0.06868	1.171332	0.131625	-0.53858	0.67594	TRUE	1.00E+00
39744_at	0.06873	1.171467	0.016344	-0.00667	0.14414	TRUE	3.29E-01

41249_at	0.06879	1.171629	0.012479	0.01122	0.12637	FALSE	4.46E-04
37531_at	0.06881	1.171683	0.021659	-0.03111	0.16874	TRUE	1.00E+00
39083_at	0.06894	1.172033	0.018634	-0.01702	0.15491	TRUE	1.00E+00
39784_at	0.069	1.172195	0.021977	-0.03239	0.17039	TRUE	1.00E+00
39739_at	0.06901	1.172222	0.011412	0.01637	0.12166	FALSE	1.85E-05
37329_at	0.06904	1.172303	0.015235	-0.00125	0.13932	TRUE	7.39E-02
32405_at	0.06904	1.172303	0.028181	-0.06098	0.19905	TRUE	1.00E+00
521_at	0.06904	1.172303	0.057848	-0.19785	0.33592	TRUE	1.00E+00
642_s_at	0.06905	1.17233	0.02036	-0.02488	0.16298	TRUE	1.00E+00
1911_s_at	0.06907	1.172384	0.030744	-0.07277	0.21091	TRUE	1.00E+00
41005_at	0.06907	1.172384	0.091826	-0.35458	0.49272	TRUE	1.00E+00
1738_at	0.06919	1.172708	0.070284	-0.25508	0.39345	TRUE	1.00E+00
36518_at	0.06926	1.172897	0.121731	-0.49236	0.63087	TRUE	1.00E+00
39401_at	0.06935	1.17314	0.019199	-0.01922	0.15793	TRUE	1.00E+00
37275_at	0.06939	1.173248	0.038164	-0.10669	0.24546	TRUE	1.00E+00
38273_at	0.06944	1.173384	0.021971	-0.03193	0.17081	TRUE	1.00E+00
41375_at	0.06944	1.173384	0.021084	-0.02783	0.16671	TRUE	1.00E+00
32790_at	0.06945	1.173411	0.0282	-0.06065	0.19955	TRUE	1.00E+00
38415_at	0.06948	1.173492	0.018302	-0.01496	0.15391	TRUE	1.00E+00
35400_at	0.06956	1.173708	0.035424	-0.09387	0.23299	TRUE	1.00E+00
40555_at	0.06958	1.173762	0.016324	-0.00573	0.1449	TRUE	2.55E-01
40355_at	0.0696	1.173816	0.084369	-0.31964	0.45885	TRUE	1.00E+00
31997_at	0.06968	1.174032	0.111777	-0.44602	0.58537	TRUE	1.00E+00
36690_at	0.06975	1.174221	0.026949	-0.05458	0.19408	TRUE	1.00E+00
33409_at	0.06976	1.174248	0.025606	-0.04838	0.18789	TRUE	1.00E+00
38251_at	0.06977	1.174276	0.022792	-0.03539	0.17492	TRUE	1.00E+00
31966_at	0.0698	1.174357	0.064922	-0.22972	0.36933	TRUE	1.00E+00
37949_at	0.06985	1.174492	0.075711	-0.27945	0.41915	TRUE	1.00E+00
35340_at	0.06988	1.174573	0.016244	-0.00507	0.14482	TRUE	2.14E-01
34312_at	0.06994	1.174735	0.024318	-0.04225	0.18214	TRUE	1.00E+00
41842_at	0.06997	1.174816	0.051734	-0.16871	0.30865	TRUE	1.00E+00
1792_g_at	0.06997	1.174816	0.02614	-0.05062	0.19057	TRUE	1.00E+00
1003_s_at	0.06999	1.174871	0.050395	-0.16251	0.30248	TRUE	1.00E+00
affx-humrge/m10	0.07002	1.174952	0.041416	-0.12106	0.26109	TRUE	1.00E+00
39580_at	0.07007	1.175087	0.045439	-0.13957	0.2797	TRUE	1.00E+00
38760_f_at	0.07015	1.175303	0.043023	-0.12834	0.26864	TRUE	1.00E+00
37828_at	0.07015	1.175303	0.023673	-0.03906	0.17937	TRUE	1.00E+00
1860_at	0.07019	1.175412	0.033778	-0.08565	0.22603	TRUE	1.00E+00
31941_s_at	0.07023	1.17552	0.072551	-0.26449	0.40495	TRUE	1.00E+00
1358_s_at	0.07027	1.175628	0.028277	-0.06019	0.20073	TRUE	1.00E+00
33775_s_at	0.07029	1.175682	0.106267	-0.41999	0.56056	TRUE	1.00E+00
32370_at	0.07032	1.175764	0.039662	-0.11267	0.2533	TRUE	1.00E+00
278_at	0.07036	1.175872	0.022374	-0.03286	0.17359	TRUE	1.00E+00
affx-dapx-3_at	0.07041	1.176007	0.03979	-0.11316	0.25398	TRUE	1.00E+00
35137_at	0.07047	1.17617	0.057074	-0.19285	0.33378	TRUE	1.00E+00
665_at	0.0705	1.176251	0.031666	-0.0756	0.21659	TRUE	1.00E+00
39809_at	0.07057	1.176441	0.029769	-0.06678	0.20791	TRUE	1.00E+00
34236_at	0.07064	1.17663	0.039494	-0.11157	0.25285	TRUE	1.00E+00
32571_at	0.07072	1.176847	0.022548	-0.03331	0.17475	TRUE	1.00E+00
38046_at	0.07074	1.176901	0.014984	0.00161	0.13987	FALSE	2.96E-02
37888_at	0.07076	1.176955	0.017014	-0.00774	0.14925	TRUE	4.04E-01

38252_s_at	0.07084	1.177172	0.013662	0.00781	0.13387	FALSE	2.72E-03
38171_at	0.07087	1.177254	0.038574	-0.10709	0.24884	TRUE	1.00E+00
31927_s_at	0.07091	1.177362	0.021095	-0.02641	0.16823	TRUE	1.00E+00
32620_at	0.07092	1.177389	0.039254	-0.11018	0.25202	TRUE	1.00E+00
39804_at	0.07093	1.177416	0.068252	-0.24396	0.38582	TRUE	1.00E+00
35802_at	0.07098	1.177552	0.029554	-0.06537	0.20732	TRUE	1.00E+00
1315_at	0.07098	1.177552	0.059024	-0.20133	0.34329	TRUE	1.00E+00
31725_s_at	0.07102	1.17766	0.045051	-0.13683	0.27886	TRUE	1.00E+00
40576_f_at	0.07115	1.178013	0.031765	-0.0754	0.2177	TRUE	1.00E+00
41300_s_at	0.07118	1.178094	0.022618	-0.03317	0.17553	TRUE	1.00E+00
41236_at	0.0712	1.178148	0.02911	-0.0631	0.20551	TRUE	1.00E+00
40423_at	0.07123	1.17823	0.022569	-0.0329	0.17535	TRUE	1.00E+00
34912_at	0.07124	1.178257	0.023782	-0.03848	0.18096	TRUE	1.00E+00
35450_s_at	0.07126	1.178311	0.019971	-0.02087	0.1634	TRUE	1.00E+00
34004_at	0.07126	1.178311	0.058289	-0.19766	0.34018	TRUE	1.00E+00
39195_s_at	0.07138	1.178637	0.11344	-0.45198	0.59475	TRUE	1.00E+00
41033_at	0.07138	1.178637	0.0307	-0.07025	0.21302	TRUE	1.00E+00
32016_at	0.07139	1.178664	0.073025	-0.26552	0.40829	TRUE	1.00E+00
1656_s_at	0.07139	1.178664	0.041807	-0.12149	0.26427	TRUE	1.00E+00
41229_at	0.07141	1.178718	0.035222	-0.09109	0.23391	TRUE	1.00E+00
35178_at	0.07141	1.178718	0.089356	-0.34084	0.48366	TRUE	1.00E+00
31794_at	0.07146	1.178854	0.031633	-0.07448	0.21741	TRUE	1.00E+00
1453_at	0.07153	1.179044	0.01549	0.00007	0.143	FALSE	4.89E-02
34022_at	0.07155	1.179098	0.107893	-0.42623	0.56932	TRUE	1.00E+00
32893_s_at	0.07155	1.179098	0.055744	-0.18563	0.32873	TRUE	1.00E+00
34330_at	0.07156	1.179125	0.021899	-0.02948	0.17259	TRUE	1.00E+00
41837_at	0.07159	1.179207	0.027282	-0.05428	0.19746	TRUE	1.00E+00
38684_at	0.07162	1.179288	0.02084	-0.02453	0.16776	TRUE	1.00E+00
32951_g_at	0.07163	1.179315	0.057284	-0.19265	0.33591	TRUE	1.00E+00
164_at	0.07163	1.179315	0.038691	-0.10687	0.25014	TRUE	1.00E+00
1220_g_at	0.07164	1.179343	0.013802	0.00796	0.13531	FALSE	2.65E-03
32907_at	0.07171	1.179533	0.072051	-0.2607	0.40413	TRUE	1.00E+00
1945_at	0.07175	1.179641	0.094599	-0.36469	0.50819	TRUE	1.00E+00
40526_at	0.07178	1.179723	0.06969	-0.24974	0.3933	TRUE	1.00E+00
1073_at	0.07179	1.17975	0.044118	-0.13175	0.27533	TRUE	1.00E+00
36303_f_at	0.07179	1.17975	0.047468	-0.1472	0.29079	TRUE	1.00E+00
35385_at	0.07179	1.17975	0.028577	-0.06005	0.20364	TRUE	1.00E+00
33749_at	0.0718	1.179777	0.045083	-0.1362	0.27979	TRUE	1.00E+00
37078_at	0.07182	1.179832	0.104941	-0.41234	0.55597	TRUE	1.00E+00
33911_at	0.07182	1.179832	0.126498	-0.51179	0.65543	TRUE	1.00E+00
1046_at	0.07189	1.180022	0.028998	-0.06189	0.20567	TRUE	1.00E+00
31863_at	0.07195	1.180185	0.018418	-0.01302	0.15692	TRUE	1.00E+00
39903_at	0.07195	1.180185	0.025525	-0.04581	0.18972	TRUE	1.00E+00
41278_at	0.07203	1.180402	0.026692	-0.05112	0.19517	TRUE	1.00E+00
40587_s_at	0.07209	1.180565	0.049827	-0.15779	0.30198	TRUE	1.00E+00
33326_at	0.07209	1.180565	0.012582	0.01405	0.13014	FALSE	1.27E-04
31731_at	0.07213	1.180674	0.064846	-0.22704	0.3713	TRUE	1.00E+00
33723_at	0.07214	1.180701	0.030244	-0.06739	0.21167	TRUE	1.00E+00
40452_at	0.07221	1.180892	0.032375	-0.07716	0.22157	TRUE	1.00E+00
41547_at	0.07223	1.180946	0.033835	-0.08387	0.22833	TRUE	1.00E+00
33208_at	0.07227	1.181055	0.032	-0.07536	0.21991	TRUE	1.00E+00

40272_at	0.07228	1.181082	0.068698	-0.24467	0.38922	TRUE	1.00E+00
622_at	0.0723	1.181136	0.01739	-0.00793	0.15253	TRUE	4.06E-01
33817_at	0.0723	1.181136	0.064139	-0.2236	0.36821	TRUE	1.00E+00
31516_f_at	0.07232	1.181191	0.042617	-0.12429	0.26894	TRUE	1.00E+00
32992_at	0.07232	1.181191	0.09897	-0.38429	0.52893	TRUE	1.00E+00
37682_r_at	0.07233	1.181218	0.088759	-0.33717	0.48182	TRUE	1.00E+00
38633_at	0.07235	1.181272	0.025123	-0.04356	0.18826	TRUE	1.00E+00
41596_s_at	0.07235	1.181272	0.024633	-0.0413	0.18599	TRUE	1.00E+00
39630_at	0.07238	1.181354	0.049414	-0.15559	0.30036	TRUE	1.00E+00
37750_at	0.07246	1.181571	0.029022	-0.06144	0.20636	TRUE	1.00E+00
31894_at	0.07246	1.181571	0.018364	-0.01226	0.15719	TRUE	1.00E+00
41470_at	0.07247	1.181599	0.071293	-0.25645	0.40139	TRUE	1.00E+00
38249_at	0.07253	1.181762	0.049729	-0.1569	0.30196	TRUE	1.00E+00
38371_at	0.07262	1.182007	0.02092	-0.02389	0.16914	TRUE	1.00E+00
37925_r_at	0.07271	1.182252	0.091842	-0.35102	0.49643	TRUE	1.00E+00
369_s_at	0.07274	1.182334	0.022087	-0.02916	0.17464	TRUE	1.00E+00
39668_at	0.07277	1.182415	0.120088	-0.48126	0.62681	TRUE	1.00E+00
33106_at	0.0728	1.182497	0.070993	-0.25473	0.40033	TRUE	1.00E+00
507_s_at	0.07281	1.182524	0.034347	-0.08565	0.23127	TRUE	1.00E+00
37810_at	0.07286	1.18266	0.027031	-0.05185	0.19757	TRUE	1.00E+00
34797_at	0.0729	1.182769	0.03292	-0.07897	0.22478	TRUE	1.00E+00
34609_g_at	0.07292	1.182824	0.021458	-0.02608	0.17192	TRUE	1.00E+00
748_s_at	0.07294	1.182878	0.07669	-0.28088	0.42675	TRUE	1.00E+00
38014_at	0.07294	1.182878	0.010048	0.02658	0.1193	FALSE	4.93E-09
1996_s_at	0.07297	1.18296	0.071068	-0.25491	0.40085	TRUE	1.00E+00
40073_at	0.07301	1.183069	0.040474	-0.11371	0.25974	TRUE	1.00E+00
34656_at	0.07303	1.183123	0.120793	-0.48426	0.63032	TRUE	1.00E+00
40538_at	0.07304	1.183151	0.031154	-0.0707	0.21677	TRUE	1.00E+00
38128_at	0.07305	1.183178	0.059699	-0.20238	0.34847	TRUE	1.00E+00
34310_at	0.07305	1.183178	0.054904	-0.18025	0.32636	TRUE	1.00E+00
599_at	0.07315	1.18345	0.026035	-0.04697	0.19327	TRUE	1.00E+00
40975_s_at	0.07316	1.183477	0.036584	-0.09562	0.24194	TRUE	1.00E+00
37172_at	0.07316	1.183477	0.039999	-0.11137	0.2577	TRUE	1.00E+00
39471_at	0.07317	1.183505	0.025805	-0.04589	0.19222	TRUE	1.00E+00
1512_at	0.07321	1.183614	0.022263	-0.0295	0.17592	TRUE	1.00E+00
40802_at	0.07325	1.183723	0.054473	-0.17806	0.32457	TRUE	1.00E+00
33980_at	0.07329	1.183832	0.070821	-0.25345	0.40003	TRUE	1.00E+00
1001_at	0.07331	1.183886	0.10556	-0.4137	0.56032	TRUE	1.00E+00
41421_at	0.07331	1.183886	0.021166	-0.02434	0.17096	TRUE	1.00E+00
37660_at	0.07338	1.184077	0.029639	-0.06337	0.21012	TRUE	1.00E+00
35122_at	0.07338	1.184077	0.058316	-0.19567	0.34242	TRUE	1.00E+00
37223_at	0.0734	1.184132	0.04045	-0.11322	0.26002	TRUE	1.00E+00
35605_at	0.07345	1.184268	0.060003	-0.20337	0.35028	TRUE	1.00E+00
38524_at	0.07348	1.18435	0.098694	-0.38185	0.52881	TRUE	1.00E+00
41037_at	0.07351	1.184432	0.053684	-0.17416	0.32118	TRUE	1.00E+00
1205_at	0.07353	1.184486	0.092974	-0.35541	0.50247	TRUE	1.00E+00
1446_at	0.07355	1.184541	0.012058	0.01792	0.12918	FALSE	1.34E-05
36864_at	0.07358	1.184623	0.030092	-0.06525	0.21241	TRUE	1.00E+00
41333_at	0.07359	1.18465	0.025798	-0.04543	0.19262	TRUE	1.00E+00
651_at	0.07363	1.184759	0.034814	-0.08699	0.23424	TRUE	1.00E+00
1903_at	0.07363	1.184759	0.033289	-0.07995	0.22721	TRUE	1.00E+00

38929_at	0.07366	1.184841	0.077619	-0.28445	0.43176	TRUE	1.00E+00
481_at	0.07376	1.185114	0.06322	-0.21791	0.36543	TRUE	1.00E+00
35266_at	0.07379	1.185196	0.021527	-0.02552	0.17311	TRUE	1.00E+00
38363_at	0.07382	1.185277	0.121869	-0.48844	0.63607	TRUE	1.00E+00
32214_at	0.07384	1.185332	0.013047	0.01364	0.13403	FALSE	1.92E-04
32574_at	0.07385	1.185359	0.030303	-0.06596	0.21365	TRUE	1.00E+00
32067_at	0.07385	1.185359	0.025256	-0.04267	0.19037	TRUE	1.00E+00
41366_at	0.07391	1.185523	0.026916	-0.05026	0.19809	TRUE	1.00E+00
40336_at	0.07408	1.185987	0.033761	-0.08168	0.22984	TRUE	1.00E+00
36209_at	0.07408	1.185987	0.01026	0.02675	0.12142	FALSE	6.53E-09
34245_at	0.07409	1.186015	0.051912	-0.16541	0.31359	TRUE	1.00E+00
38847_at	0.0741	1.186042	0.06318	-0.21739	0.36558	TRUE	1.00E+00
36874_at	0.0741	1.186042	0.062868	-0.21595	0.36415	TRUE	1.00E+00
38661_at	0.0741	1.186042	0.040578	-0.11311	0.26131	TRUE	1.00E+00
39546_s_at	0.07411	1.186069	0.035954	-0.09177	0.23999	TRUE	1.00E+00
32632_g_at	0.07418	1.18626	0.023919	-0.03618	0.18453	TRUE	1.00E+00
33354_at	0.07418	1.18626	0.037852	-0.10045	0.24882	TRUE	1.00E+00
34380_at	0.07427	1.186506	0.019151	-0.01408	0.16263	TRUE	1.00E+00
41022_r_at	0.07428	1.186533	0.026143	-0.04633	0.1949	TRUE	1.00E+00
35629_at	0.0744	1.186861	0.01893	-0.01293	0.16173	TRUE	1.00E+00
1798_at	0.07442	1.186916	0.012289	0.01772	0.13112	FALSE	1.76E-05
40146_at	0.07442	1.186916	0.013162	0.0137	0.13514	FALSE	1.98E-04
33944_at	0.07443	1.186943	0.039595	-0.10825	0.2571	TRUE	1.00E+00
36655_at	0.07443	1.186943	0.038052	-0.10113	0.24999	TRUE	1.00E+00
37823_at	0.07445	1.186998	0.091911	-0.34959	0.4985	TRUE	1.00E+00
34607_at	0.07449	1.187107	0.074232	-0.26799	0.41697	TRUE	1.00E+00
31839_at	0.07455	1.187271	0.031164	-0.06923	0.21833	TRUE	1.00E+00
40416_at	0.07461	1.187435	0.034558	-0.08483	0.23405	TRUE	1.00E+00
35176_at	0.07463	1.18749	0.01932	-0.0145	0.16377	TRUE	1.00E+00
33230_at	0.07465	1.187545	0.046349	-0.13919	0.28848	TRUE	1.00E+00
38356_at	0.07466	1.187572	0.077415	-0.2825	0.43182	TRUE	1.00E+00
40480_s_at	0.0747	1.187682	0.035688	-0.08995	0.23934	TRUE	1.00E+00
38156_at	0.07472	1.187736	0.066346	-0.23138	0.38081	TRUE	1.00E+00
33797_at	0.07475	1.187818	0.021179	-0.02296	0.17246	TRUE	1.00E+00
41103_at	0.07475	1.187818	0.138013	-0.56198	0.71149	TRUE	1.00E+00
35165_at	0.07476	1.187846	0.04543	-0.13484	0.28435	TRUE	1.00E+00
40471_at	0.07481	1.187982	0.023469	-0.03346	0.18308	TRUE	1.00E+00
2033_s_at	0.07481	1.187982	0.02495	-0.0403	0.18992	TRUE	1.00E+00
37872_at	0.07483	1.188037	0.029174	-0.05977	0.20943	TRUE	1.00E+00
36393_at	0.07486	1.188119	0.035293	-0.08796	0.23769	TRUE	1.00E+00
35127_at	0.07488	1.188174	0.023863	-0.03521	0.18497	TRUE	1.00E+00
36610_at	0.07489	1.188201	0.025586	-0.04315	0.19293	TRUE	1.00E+00
1539_at	0.07492	1.188283	0.049532	-0.1536	0.30344	TRUE	1.00E+00
41594_at	0.07501	1.18853	0.014263	0.0092	0.14081	FALSE	1.83E-03
36201_at	0.07501	1.18853	0.011057	0.024	0.12602	FALSE	1.47E-07
32885_f_at	0.07505	1.188639	0.066308	-0.23087	0.38097	TRUE	1.00E+00
39047_at	0.07506	1.188666	0.027745	-0.05294	0.20306	TRUE	1.00E+00
37873_g_at	0.07508	1.188721	0.071191	-0.25337	0.40353	TRUE	1.00E+00
39651_at	0.07508	1.188721	0.045235	-0.13362	0.28378	TRUE	1.00E+00
38970_s_at	0.07512	1.188831	0.047969	-0.14618	0.29643	TRUE	1.00E+00
32940_at	0.07517	1.188968	0.025005	-0.04019	0.19053	TRUE	1.00E+00

40609_at	0.07518	1.188995	0.107068	-0.41878	0.56915	TRUE	1.00E+00
33699_at	0.07518	1.188995	0.022338	-0.02788	0.17825	TRUE	1.00E+00
31761_at	0.07519	1.189022	0.104149	-0.40531	0.55568	TRUE	1.00E+00
35576_f_at	0.0752	1.18905	0.0857	-0.32019	0.47059	TRUE	1.00E+00
41818_at	0.0752	1.18905	0.077144	-0.28071	0.43111	TRUE	1.00E+00
33289_f_at	0.07523	1.189132	0.031674	-0.07091	0.22136	TRUE	1.00E+00
40085_s_at	0.07526	1.189214	0.04848	-0.14841	0.29892	TRUE	1.00E+00
34065_at	0.07528	1.189269	0.039396	-0.10648	0.25703	TRUE	1.00E+00
31881_at	0.07533	1.189406	0.018637	-0.01065	0.16131	TRUE	6.69E-01
35878_at	0.07534	1.189433	0.043717	-0.12635	0.27703	TRUE	1.00E+00
39785_at	0.07534	1.189433	0.014239	0.00965	0.14104	FALSE	1.53E-03
31346_at	0.07535	1.18946	0.055746	-0.18184	0.33254	TRUE	1.00E+00
1812_s_at	0.07545	1.189734	0.043782	-0.12655	0.27744	TRUE	1.00E+00
37864_s_at	0.07547	1.189789	0.05676	-0.1864	0.33734	TRUE	1.00E+00
39532_at	0.07548	1.189817	0.066234	-0.23009	0.38106	TRUE	1.00E+00
36385_at	0.07549	1.189844	0.085539	-0.31916	0.47013	TRUE	1.00E+00
39065_s_at	0.07554	1.189981	0.026409	-0.04629	0.19738	TRUE	1.00E+00
38795_s_at	0.07561	1.190173	0.029977	-0.0627	0.21391	TRUE	1.00E+00
41459_at	0.07564	1.190255	0.025074	-0.04004	0.19132	TRUE	1.00E+00
38202_at	0.07565	1.190282	0.049925	-0.15468	0.30599	TRUE	1.00E+00
41864_at	0.07567	1.190337	0.020804	-0.02031	0.17165	TRUE	1.00E+00
39418_at	0.07571	1.190447	0.017033	-0.00287	0.15429	TRUE	1.11E-01
32979_at	0.07574	1.190529	0.046065	-0.13679	0.28826	TRUE	1.00E+00
1923_at	0.07582	1.190748	0.022628	-0.02858	0.18022	TRUE	1.00E+00
32724_at	0.07584	1.190803	0.02468	-0.03803	0.1897	TRUE	1.00E+00
38266_at	0.07588	1.190913	0.054893	-0.17738	0.32913	TRUE	1.00E+00
34543_at	0.0759	1.190968	0.090503	-0.34164	0.49345	TRUE	1.00E+00
38891_at	0.07594	1.191077	0.060099	-0.20133	0.35322	TRUE	1.00E+00
32952_at	0.07595	1.191105	0.073479	-0.26305	0.41496	TRUE	1.00E+00
32211_at	0.07598	1.191187	0.025152	-0.04006	0.19202	TRUE	1.00E+00
40519_at	0.07599	1.191215	0.081993	-0.30229	0.45427	TRUE	1.00E+00
35257_at	0.0761	1.191516	0.073646	-0.26367	0.41587	TRUE	1.00E+00
38568_at	0.07614	1.191626	0.037941	-0.09891	0.25118	TRUE	1.00E+00
1093_at	0.07615	1.191654	0.024232	-0.03565	0.18795	TRUE	1.00E+00
31616_r_at	0.07616	1.191681	0.125015	-0.5006	0.65293	TRUE	1.00E+00
33743_at	0.07617	1.191708	0.088831	-0.33366	0.486	TRUE	1.00E+00
39036_g_at	0.07619	1.191763	0.02303	-0.03006	0.18244	TRUE	1.00E+00
33751_at	0.07621	1.191818	0.057819	-0.19054	0.34296	TRUE	1.00E+00
569_g_at	0.07627	1.191983	0.050806	-0.15813	0.31066	TRUE	1.00E+00
1574_s_at	0.07629	1.192038	0.044157	-0.12743	0.28001	TRUE	1.00E+00
32648_at	0.07632	1.19212	0.027584	-0.05094	0.20358	TRUE	1.00E+00
38336_at	0.07634	1.192175	0.057104	-0.18711	0.3398	TRUE	1.00E+00
36660_at	0.07636	1.19223	0.022463	-0.02727	0.17999	TRUE	1.00E+00
33063_at	0.07637	1.192257	0.091882	-0.34753	0.50027	TRUE	1.00E+00
39772_at	0.07638	1.192285	0.029546	-0.05993	0.2127	TRUE	1.00E+00
41702_r_at	0.07644	1.19245	0.026978	-0.04803	0.2009	TRUE	1.00E+00
36833_at	0.07645	1.192477	0.016413	0.00073	0.15217	FALSE	4.04E-02
34744_at	0.07655	1.192752	0.008151	0.03894	0.11415	FALSE	7.49E-17
38826_at	0.07669	1.193136	0.055205	-0.17801	0.33138	TRUE	1.00E+00
36992_at	0.07679	1.193411	0.022374	-0.02643	0.18002	TRUE	1.00E+00
32014_at	0.0768	1.193438	0.066034	-0.22785	0.38145	TRUE	1.00E+00

33167_r_at	0.0768	1.193438	0.036735	-0.09268	0.24628	TRUE	1.00E+00
40066_at	0.07683	1.193521	0.013417	0.01493	0.13873	FALSE	1.30E-04
1319_at	0.07687	1.193631	0.017243	-0.00268	0.15642	TRUE	1.04E-01
35696_s_at	0.07687	1.193631	0.119482	-0.47437	0.62811	TRUE	1.00E+00
38728_at	0.07688	1.193658	0.027154	-0.04839	0.20216	TRUE	1.00E+00
39997_at	0.0769	1.193713	0.069822	-0.24523	0.39903	TRUE	1.00E+00
39086_g_at	0.07691	1.193741	0.01797	-0.006	0.15981	TRUE	2.36E-01
35364_at	0.07691	1.193741	0.022838	-0.02846	0.18228	TRUE	1.00E+00
31698_at	0.07693	1.193796	0.104012	-0.40294	0.55679	TRUE	1.00E+00
1984_s_at	0.07696	1.193878	0.072418	-0.25715	0.41106	TRUE	1.00E+00
32802_at	0.07696	1.193878	0.030851	-0.06538	0.21929	TRUE	1.00E+00
1913_at	0.07698	1.193933	0.043723	-0.12474	0.2787	TRUE	1.00E+00
39647_s_at	0.07699	1.193961	0.09721	-0.3715	0.52547	TRUE	1.00E+00
783_at	0.077	1.193988	0.014943	0.00806	0.14595	FALSE	3.24E-03
31913_at	0.07705	1.194126	0.038597	-0.10102	0.25511	TRUE	1.00E+00
796_i_at	0.07708	1.194208	0.037984	-0.09817	0.25232	TRUE	1.00E+00
38262_at	0.07708	1.194208	0.033973	-0.07965	0.23382	TRUE	1.00E+00
32203_at	0.0771	1.194263	0.02348	-0.03122	0.18543	TRUE	1.00E+00
39173_at	0.07711	1.194291	0.019153	-0.01126	0.16547	TRUE	7.17E-01
31507_at	0.07713	1.194346	0.096086	-0.36617	0.52043	TRUE	1.00E+00
34771_at	0.07713	1.194346	0.062088	-0.20931	0.36358	TRUE	1.00E+00
1941_at	0.07714	1.194373	0.119671	-0.47497	0.62926	TRUE	1.00E+00
34112_r_at	0.07717	1.194456	0.035138	-0.08494	0.23928	TRUE	1.00E+00
351_f_at	0.07721	1.194566	0.022295	-0.02565	0.18007	TRUE	1.00E+00
32914_f_at	0.07723	1.194621	0.01426	0.01144	0.14302	FALSE	7.70E-04
39162_at	0.07733	1.194896	0.023535	-0.03125	0.18591	TRUE	1.00E+00
38901_at	0.07734	1.194923	0.021356	-0.02119	0.17586	TRUE	1.00E+00
818_s_at	0.07748	1.195308	0.029186	-0.05717	0.21213	TRUE	1.00E+00
35325_at	0.0775	1.195364	0.011648	0.02376	0.13124	FALSE	3.61E-07
34756_g_at	0.07752	1.195419	0.024058	-0.03348	0.18851	TRUE	1.00E+00
36633_at	0.07753	1.195446	0.016007	0.00368	0.15139	FALSE	1.61E-02
35292_at	0.07757	1.195556	0.027411	-0.0489	0.20403	TRUE	1.00E+00
1356_at	0.0776	1.195639	0.015286	0.00708	0.14812	FALSE	4.85E-03
34832_s_at	0.07762	1.195694	0.02173	-0.02264	0.17787	TRUE	1.00E+00
35009_at	0.07762	1.195694	0.080547	-0.29399	0.44923	TRUE	1.00E+00
37315_f_at	0.07765	1.195776	0.025459	-0.03981	0.19511	TRUE	1.00E+00
447_g_at	0.07767	1.195832	0.031211	-0.06633	0.22166	TRUE	1.00E+00
380_at	0.07775	1.196052	0.043632	-0.12355	0.27905	TRUE	1.00E+00
36408_at	0.07787	1.196382	0.069852	-0.2444	0.40014	TRUE	1.00E+00
41786_at	0.07791	1.196493	0.049035	-0.14831	0.30414	TRUE	1.00E+00
403_s_at	0.07802	1.196796	0.075981	-0.27253	0.42856	TRUE	1.00E+00
34025_at	0.07804	1.196851	0.041846	-0.11502	0.2711	TRUE	1.00E+00
1318_at	0.07805	1.196878	0.017963	-0.00482	0.16093	TRUE	1.76E-01
1164_at	0.07814	1.197126	0.014643	0.01058	0.1457	FALSE	1.20E-03
32504_at	0.07826	1.197457	0.01933	-0.01092	0.16744	TRUE	6.51E-01
38192_at	0.07826	1.197457	0.027311	-0.04774	0.20426	TRUE	1.00E+00
1809_at	0.07835	1.197705	0.033264	-0.07512	0.23181	TRUE	1.00E+00
affx-humtfrr/m11!	0.07835	1.197705	0.037977	-0.09686	0.25356	TRUE	1.00E+00
37775_at	0.07837	1.197761	0.05098	-0.15683	0.31358	TRUE	1.00E+00
379_at	0.07838	1.197788	0.017533	-0.00251	0.15926	TRUE	9.86E-02
41765_at	0.07849	1.198092	0.030855	-0.06386	0.22084	TRUE	1.00E+00

36136_at	0.0785	1.198119	0.047456	-0.14044	0.29744	TRUE	1.00E+00
37500_at	0.07851	1.198147	0.030144	-0.06057	0.21758	TRUE	1.00E+00
36594_s_at	0.07851	1.198147	0.022295	-0.02435	0.18137	TRUE	1.00E+00
35983_at	0.07853	1.198202	0.041376	-0.11236	0.26942	TRUE	1.00E+00
37501_at	0.0786	1.198395	0.033176	-0.07445	0.23166	TRUE	1.00E+00
37999_at	0.07861	1.198423	0.040108	-0.10644	0.26365	TRUE	1.00E+00
38654_at	0.07863	1.198478	0.013946	0.01429	0.14297	FALSE	2.17E-04
41604_at	0.07864	1.198505	0.016851	0.00089	0.15638	FALSE	3.86E-02
36541_at	0.07865	1.198533	0.047388	-0.13997	0.29728	TRUE	1.00E+00
37422_at	0.0787	1.198671	0.037376	-0.09373	0.25114	TRUE	1.00E+00
36436_at	0.07871	1.198699	0.029743	-0.05851	0.21594	TRUE	1.00E+00
39062_at	0.07872	1.198726	0.022452	-0.02486	0.1823	TRUE	1.00E+00
affx-humtfrr/m11!	0.07874	1.198781	0.062427	-0.20927	0.36676	TRUE	1.00E+00
1311_at	0.07874	1.198781	0.008164	0.04108	0.11641	FALSE	6.49E-18
40825_at	0.07878	1.198892	0.03105	-0.06447	0.22203	TRUE	1.00E+00
37726_at	0.07881	1.198975	0.018975	-0.00873	0.16635	TRUE	4.13E-01
40841_at	0.07882	1.199002	0.03327	-0.07468	0.23231	TRUE	1.00E+00
31400_at	0.07889	1.199196	0.142503	-0.57856	0.73634	TRUE	1.00E+00
37507_i_at	0.07891	1.199251	0.015972	0.00522	0.1526	FALSE	9.84E-03
40015_at	0.07895	1.199361	0.094998	-0.35933	0.51723	TRUE	1.00E+00
38614_s_at	0.07904	1.19961	0.018864	-0.008	0.16607	TRUE	3.53E-01
40111_g_at	0.07914	1.199886	0.014311	0.01312	0.14517	FALSE	4.03E-04
38781_at	0.07915	1.199914	0.060864	-0.20165	0.35995	TRUE	1.00E+00
815_at	0.07917	1.199969	0.041216	-0.11098	0.26932	TRUE	1.00E+00
33652_at	0.0792	1.200052	0.088771	-0.33035	0.48875	TRUE	1.00E+00
33795_at	0.07925	1.20019	0.026892	-0.04482	0.20332	TRUE	1.00E+00
41015_at	0.07928	1.200273	0.028941	-0.05424	0.2128	TRUE	1.00E+00
36297_at	0.07935	1.200466	0.032573	-0.07093	0.22963	TRUE	1.00E+00
37169_at	0.07935	1.200466	0.044087	-0.12405	0.28275	TRUE	1.00E+00
41843_r_at	0.07937	1.200522	0.029115	-0.05495	0.21369	TRUE	1.00E+00
36811_at	0.07939	1.200577	0.033447	-0.07491	0.2337	TRUE	1.00E+00
36895_at	0.07945	1.200743	0.019973	-0.0127	0.1716	TRUE	8.78E-01
32475_at	0.07949	1.200853	0.022143	-0.02267	0.18165	TRUE	1.00E+00
33554_at	0.0795	1.200881	0.065664	-0.22345	0.38244	TRUE	1.00E+00
32608_at	0.07951	1.200909	0.089673	-0.3342	0.49322	TRUE	1.00E+00
41699_f_at	0.07956	1.201047	0.026704	-0.04364	0.20276	TRUE	1.00E+00
38820_at	0.07957	1.201075	0.018488	-0.00573	0.16486	TRUE	2.12E-01
1086_at	0.07959	1.20113	0.035639	-0.08484	0.24401	TRUE	1.00E+00
202_at	0.07964	1.201268	0.040819	-0.10868	0.26796	TRUE	1.00E+00
41721_at	0.07966	1.201324	0.075962	-0.2708	0.43011	TRUE	1.00E+00
40820_at	0.07968	1.201379	0.036565	-0.08902	0.24837	TRUE	1.00E+00
36802_at	0.07972	1.20149	0.026741	-0.04365	0.20309	TRUE	1.00E+00
465_at	0.07978	1.201656	0.04528	-0.12912	0.28868	TRUE	1.00E+00
41202_s_at	0.07987	1.201905	0.019678	-0.01092	0.17066	TRUE	6.23E-01
41342_at	0.0799	1.201988	0.028203	-0.05022	0.21002	TRUE	1.00E+00
877_at	0.0799	1.201988	0.042265	-0.11509	0.2749	TRUE	1.00E+00
35435_s_at	0.08	1.202264	0.01902	-0.00775	0.16775	TRUE	3.28E-01
38136_at	0.08001	1.202292	0.044455	-0.12508	0.28511	TRUE	1.00E+00
2065_s_at	0.08003	1.202347	0.016617	0.00336	0.15669	FALSE	1.85E-02
1313_at	0.08006	1.202431	0.018033	-0.00314	0.16326	TRUE	1.14E-01
252_at	0.08011	1.202569	0.031866	-0.06691	0.22712	TRUE	1.00E+00

37838_at	0.08011	1.202569	0.071999	-0.25206	0.41229	TRUE	1.00E+00
1300_at	0.08012	1.202597	0.037949	-0.09496	0.2552	TRUE	1.00E+00
35439_at	0.08012	1.202597	0.044164	-0.12363	0.28388	TRUE	1.00E+00
1531_at	0.08018	1.202763	0.032867	-0.07146	0.23181	TRUE	1.00E+00
40928_at	0.08024	1.202929	0.03841	-0.09697	0.25744	TRUE	1.00E+00
32396_f_at	0.08024	1.202929	0.049171	-0.14662	0.30709	TRUE	1.00E+00
37460_at	0.08025	1.202957	0.038426	-0.09703	0.25753	TRUE	1.00E+00
490_g_at	0.08026	1.202984	0.019348	-0.009	0.16952	TRUE	4.23E-01
32300_s_at	0.08027	1.203012	0.048919	-0.14543	0.30596	TRUE	1.00E+00
1938_at	0.08032	1.203151	0.032735	-0.07071	0.23135	TRUE	1.00E+00
31741_at	0.08038	1.203317	0.093921	-0.35293	0.51369	TRUE	1.00E+00
1179_at	0.08043	1.203455	0.020699	-0.01507	0.17592	TRUE	1.00E+00
38800_at	0.08043	1.203455	0.05837	-0.18886	0.34972	TRUE	1.00E+00
1462_s_at	0.08045	1.203511	0.027141	-0.04477	0.20567	TRUE	1.00E+00
32394_s_at	0.08045	1.203511	0.013134	0.01986	0.14104	FALSE	1.14E-05
466_at	0.0805	1.203649	0.020956	-0.01618	0.17718	TRUE	1.00E+00
38694_at	0.08051	1.203677	0.022354	-0.02262	0.18365	TRUE	1.00E+00
39442_at	0.08057	1.203843	0.019165	-0.00785	0.16899	TRUE	3.31E-01
35296_at	0.08063	1.20401	0.033771	-0.07518	0.23644	TRUE	1.00E+00
1837_at	0.08069	1.204176	0.03265	-0.06995	0.23132	TRUE	1.00E+00
384_at	0.08074	1.204315	0.022231	-0.02182	0.18331	TRUE	1.00E+00
33921_at	0.08075	1.204342	0.0279	-0.04797	0.20947	TRUE	1.00E+00
41326_at	0.08076	1.20437	0.099464	-0.37813	0.53964	TRUE	1.00E+00
33269_at	0.08081	1.204509	0.047461	-0.13815	0.29977	TRUE	1.00E+00
38383_at	0.08081	1.204509	0.024151	-0.03061	0.19224	TRUE	1.00E+00
40885_s_at	0.08087	1.204675	0.030487	-0.05979	0.22152	TRUE	1.00E+00
40795_at	0.08098	1.20498	0.062847	-0.20896	0.37093	TRUE	1.00E+00
38801_at	0.08102	1.205091	0.025637	-0.03726	0.19929	TRUE	1.00E+00
40642_at	0.08102	1.205091	0.059601	-0.19395	0.35599	TRUE	1.00E+00
38762_at	0.08104	1.205147	0.031697	-0.0652	0.22727	TRUE	1.00E+00
36715_at	0.08104	1.205147	0.049757	-0.14852	0.3106	TRUE	1.00E+00
35116_at	0.08107	1.20523	0.041268	-0.10932	0.27147	TRUE	1.00E+00
1727_at	0.08117	1.205508	0.165754	-0.68355	0.84589	TRUE	1.00E+00
39741_at	0.08118	1.205535	0.008635	0.04134	0.12102	FALSE	6.79E-17
497_at	0.0812	1.205591	0.019644	-0.00943	0.17183	TRUE	4.51E-01
34822_at	0.08121	1.205619	0.024557	-0.03208	0.19451	TRUE	1.00E+00
40662_g_at	0.08124	1.205702	0.089201	-0.3303	0.49278	TRUE	1.00E+00
35611_at	0.08128	1.205813	0.030529	-0.05957	0.22213	TRUE	1.00E+00
40919_at	0.08141	1.206174	0.034747	-0.07889	0.24172	TRUE	1.00E+00
32111_at	0.08145	1.206285	0.010035	0.03516	0.12775	FALSE	6.01E-12
41010_at	0.08147	1.206341	0.032015	-0.06624	0.22917	TRUE	1.00E+00
31915_at	0.08148	1.206369	0.039679	-0.10159	0.26454	TRUE	1.00E+00
34152_at	0.0815	1.206424	0.048159	-0.14068	0.30368	TRUE	1.00E+00
1371_s_at	0.08155	1.206563	0.024364	-0.03086	0.19395	TRUE	1.00E+00
35594_at	0.08159	1.206674	0.076318	-0.2705	0.43369	TRUE	1.00E+00
34003_at	0.0816	1.206702	0.030359	-0.05846	0.22166	TRUE	1.00E+00
36944_f_at	0.08163	1.206785	0.01667	0.00472	0.15854	FALSE	1.23E-02
36236_at	0.08164	1.206813	0.039724	-0.10163	0.26491	TRUE	1.00E+00
39239_at	0.08165	1.206841	0.027992	-0.04749	0.2108	TRUE	1.00E+00
37569_at	0.08168	1.206924	0.030942	-0.06108	0.22443	TRUE	1.00E+00
354_s_at	0.08168	1.206924	0.018947	-0.00574	0.16909	TRUE	2.05E-01

34368_at	0.0817	1.20698	0.015738	0.00909	0.1543	FALSE	2.64E-03
40638_at	0.08171	1.207008	0.034588	-0.07786	0.24128	TRUE	1.00E+00
34732_at	0.08174	1.207091	0.023492	-0.02664	0.19013	TRUE	1.00E+00
38693_at	0.0818	1.207258	0.027106	-0.04326	0.20685	TRUE	1.00E+00
33123_at	0.08185	1.207397	0.030771	-0.06012	0.22381	TRUE	1.00E+00
35702_at	0.08186	1.207425	0.080745	-0.29066	0.45439	TRUE	1.00E+00
40245_at	0.08189	1.207508	0.035497	-0.08188	0.24565	TRUE	1.00E+00
34074_s_at	0.0819	1.207536	0.089997	-0.33331	0.4971	TRUE	1.00E+00
39550_at	0.08191	1.207564	0.060481	-0.19712	0.36095	TRUE	1.00E+00
130_s_at	0.082	1.207814	0.025067	-0.03365	0.19765	TRUE	1.00E+00
39776_at	0.08208	1.208036	0.016737	0.00486	0.1593	FALSE	1.19E-02
41821_at	0.08211	1.20812	0.044788	-0.12452	0.28874	TRUE	1.00E+00
31453_s_at	0.08213	1.208175	0.097643	-0.36835	0.53261	TRUE	1.00E+00
34239_at	0.08213	1.208175	0.123239	-0.48644	0.6507	TRUE	1.00E+00
353_at	0.08216	1.208259	0.017626	0.00084	0.16348	FALSE	3.97E-02
37966_at	0.08218	1.208315	0.02503	-0.0333	0.19766	TRUE	1.00E+00
40341_at	0.08228	1.208593	0.017337	0.00229	0.16226	FALSE	2.62E-02
1294_at	0.0823	1.208648	0.028632	-0.0498	0.21439	TRUE	1.00E+00
40724_at	0.08233	1.208732	0.044298	-0.12204	0.2867	TRUE	1.00E+00
39013_at	0.08235	1.208788	0.039202	-0.09851	0.26322	TRUE	1.00E+00
34220_at	0.08236	1.208815	0.040197	-0.10309	0.26781	TRUE	1.00E+00
38772_at	0.08238	1.208871	0.042596	-0.11414	0.2789	TRUE	1.00E+00
34956_at	0.08238	1.208871	0.037664	-0.09138	0.25615	TRUE	1.00E+00
37256_at	0.08239	1.208899	0.032087	-0.06564	0.23043	TRUE	1.00E+00
40418_at	0.08245	1.209066	0.027237	-0.04321	0.20811	TRUE	1.00E+00
40656_at	0.08247	1.209122	0.05072	-0.15153	0.31647	TRUE	1.00E+00
31392_r_at	0.08247	1.209122	0.053659	-0.16509	0.33003	TRUE	1.00E+00
38380_at	0.08249	1.209177	0.022126	-0.01959	0.18456	TRUE	1.00E+00
38658_at	0.08251	1.209233	0.028124	-0.04724	0.21226	TRUE	1.00E+00
33669_at	0.08253	1.209289	0.069744	-0.23924	0.4043	TRUE	1.00E+00
41307_at	0.08253	1.209289	0.052308	-0.1588	0.32386	TRUE	1.00E+00
868_at	0.08257	1.2094	0.036105	-0.084	0.24915	TRUE	1.00E+00
37723_at	0.0826	1.209484	0.050311	-0.14951	0.31471	TRUE	1.00E+00
35849_at	0.08265	1.209623	0.048248	-0.13995	0.30524	TRUE	1.00E+00
41021_s_at	0.08266	1.209651	0.023805	-0.02717	0.19249	TRUE	1.00E+00
36437_s_at	0.08267	1.209679	0.026472	-0.03946	0.2048	TRUE	1.00E+00
1159_at	0.08271	1.20979	0.155903	-0.63657	0.80198	TRUE	1.00E+00
34730_g_at	0.08274	1.209874	0.041939	-0.11075	0.27623	TRUE	1.00E+00
1804_at	0.08275	1.209901	0.028818	-0.0502	0.2157	TRUE	1.00E+00
34871_at	0.0828	1.210041	0.019862	-0.00884	0.17443	TRUE	3.87E-01
36396_at	0.08289	1.210292	0.066242	-0.22272	0.3885	TRUE	1.00E+00
35995_at	0.08294	1.210431	0.093783	-0.34973	0.51562	TRUE	1.00E+00
38241_at	0.08296	1.210487	0.012339	0.02603	0.13988	FALSE	2.25E-07
33075_at	0.08298	1.210542	0.061856	-0.2024	0.36836	TRUE	1.00E+00
40601_at	0.083	1.210598	0.02516	-0.03308	0.19907	TRUE	1.00E+00
34383_at	0.08302	1.210654	0.038021	-0.0924	0.25843	TRUE	1.00E+00
40353_at	0.08304	1.21071	0.019113	-0.00514	0.17122	TRUE	1.76E-01
41434_at	0.08306	1.210765	0.011988	0.02775	0.13837	FALSE	5.38E-08
39957_at	0.08306	1.210765	0.027771	-0.04506	0.21118	TRUE	1.00E+00
32377_at	0.08311	1.210905	0.120063	-0.47081	0.63703	TRUE	1.00E+00
41583_at	0.08314	1.210988	0.077597	-0.27486	0.44115	TRUE	1.00E+00

38529_at	0.08316	1.211044	0.031628	-0.06276	0.22908	TRUE	1.00E+00
41672_at	0.08316	1.211044	0.018782	-0.00349	0.16982	TRUE	1.20E-01
41553_at	0.08324	1.211267	0.063624	-0.21029	0.37678	TRUE	1.00E+00
40508_at	0.0833	1.211435	0.012656	0.02491	0.14169	FALSE	5.88E-07
34717_s_at	0.08339	1.211686	0.031675	-0.06274	0.22952	TRUE	1.00E+00
35086_at	0.0835	1.211993	0.058051	-0.18432	0.35132	TRUE	1.00E+00
39357_at	0.0835	1.211993	0.027721	-0.04439	0.21139	TRUE	1.00E+00
40709_at	0.08351	1.212021	0.005105	0.05996	0.10706	FALSE	4.80E-56
39649_at	0.08351	1.212021	0.057391	-0.18127	0.34829	TRUE	1.00E+00
39906_r_at	0.08352	1.212049	0.043766	-0.1184	0.28544	TRUE	1.00E+00
35301_at	0.08355	1.212132	0.024563	-0.02978	0.19687	TRUE	1.00E+00
34597_at	0.08358	1.212216	0.05811	-0.18451	0.35168	TRUE	1.00E+00
34411_at	0.08359	1.212244	0.018761	-0.00297	0.17014	TRUE	1.06E-01
39603_at	0.08361	1.2123	0.019373	-0.00577	0.17299	TRUE	2.01E-01
1422_g_at	0.08364	1.212383	0.041668	-0.1086	0.27588	TRUE	1.00E+00
39443_s_at	0.08379	1.212802	0.014411	0.0173	0.15027	FALSE	7.70E-05
586_s_at	0.08381	1.212858	0.043291	-0.11591	0.28354	TRUE	1.00E+00
40500_at	0.08384	1.212942	0.086248	-0.31407	0.48175	TRUE	1.00E+00
1893_s_at	0.08385	1.21297	0.097895	-0.3678	0.53549	TRUE	1.00E+00
33578_at	0.08387	1.213026	0.072031	-0.24845	0.4162	TRUE	1.00E+00
40177_at	0.08388	1.213054	0.036799	-0.0859	0.25365	TRUE	1.00E+00
38100_at	0.0839	1.213109	0.023911	-0.02641	0.19421	TRUE	1.00E+00
36368_at	0.08396	1.213277	0.067561	-0.22774	0.39565	TRUE	1.00E+00
1263_at	0.08397	1.213305	0.044396	-0.12086	0.28879	TRUE	1.00E+00
37495_at	0.084	1.213389	0.039675	-0.09905	0.26704	TRUE	1.00E+00
1456_s_at	0.08411	1.213696	0.035712	-0.08065	0.24887	TRUE	1.00E+00
34970_r_at	0.08411	1.213696	0.037638	-0.08954	0.25776	TRUE	1.00E+00
33481_at	0.08412	1.213724	0.075188	-0.26277	0.43101	TRUE	1.00E+00
35940_at	0.08413	1.213752	0.108265	-0.41536	0.58362	TRUE	1.00E+00
affx-yel018w/_at	0.08417	1.213864	0.044763	-0.12235	0.29068	TRUE	1.00E+00
34285_at	0.0842	1.213948	0.024681	-0.02966	0.19807	TRUE	1.00E+00
36688_at	0.08423	1.214032	0.019297	-0.0048	0.17326	TRUE	1.61E-01
33476_at	0.08423	1.214032	0.06938	-0.23586	0.40433	TRUE	1.00E+00
123_at	0.08432	1.214283	0.049327	-0.14326	0.31189	TRUE	1.00E+00
1510_g_at	0.08433	1.214311	0.051493	-0.15324	0.3219	TRUE	1.00E+00
36076_g_at	0.08437	1.214423	0.01713	0.00534	0.16341	FALSE	1.06E-02
35163_at	0.08454	1.214899	0.025632	-0.03371	0.2028	TRUE	1.00E+00
40654_at	0.08456	1.214954	0.040215	-0.10097	0.2701	TRUE	1.00E+00
41302_at	0.08461	1.215094	0.005864	0.05755	0.11166	FALSE	4.38E-43
40995_at	0.08463	1.21515	0.121735	-0.47701	0.64626	TRUE	1.00E+00
470_at	0.08469	1.215318	0.106148	-0.40503	0.57442	TRUE	1.00E+00
41634_at	0.08474	1.215458	0.021492	-0.01441	0.1839	TRUE	1.00E+00
41086_at	0.08474	1.215458	0.037599	-0.08872	0.25821	TRUE	1.00E+00
35784_at	0.08479	1.215598	0.030579	-0.05628	0.22587	TRUE	1.00E+00
33380_at	0.0848	1.215626	0.025068	-0.03085	0.20045	TRUE	1.00E+00
38771_at	0.08485	1.215766	0.023582	-0.02395	0.19364	TRUE	1.00E+00
35423_at	0.08485	1.215766	0.071076	-0.24306	0.41277	TRUE	1.00E+00
39001_at	0.08486	1.215794	0.044728	-0.12149	0.29122	TRUE	1.00E+00
35233_r_at	0.08488	1.21585	0.037409	-0.08771	0.25747	TRUE	1.00E+00
1171_s_at	0.08494	1.216018	0.08886	-0.32503	0.4949	TRUE	1.00E+00
37000_at	0.08498	1.21613	0.028625	-0.04708	0.21705	TRUE	1.00E+00

34391_at	0.08505	1.216326	0.01543	0.01386	0.15624	FALSE	4.48E-04
1630_s_at	0.08511	1.216494	0.030837	-0.05716	0.22738	TRUE	1.00E+00
33782_r_at	0.08512	1.216522	0.035342	-0.07793	0.24817	TRUE	1.00E+00
39290_f_at	0.08515	1.216606	0.035058	-0.07659	0.2469	TRUE	1.00E+00
38858_at	0.08517	1.216662	0.035863	-0.08029	0.25063	TRUE	1.00E+00
36738_at	0.0852	1.216746	0.116315	-0.45143	0.62183	TRUE	1.00E+00
31546_at	0.08541	1.217335	0.012082	0.02967	0.14115	FALSE	1.97E-08
41318_g_at	0.08541	1.217335	0.027416	-0.04108	0.2119	TRUE	1.00E+00
34170_s_at	0.08549	1.217559	0.048	-0.13596	0.30694	TRUE	1.00E+00
39663_at	0.08553	1.217671	0.038196	-0.09069	0.26175	TRUE	1.00E+00
32742_s_at	0.0856	1.217867	0.027295	-0.04033	0.21153	TRUE	1.00E+00
34633_s_at	0.0856	1.217867	0.087008	-0.31581	0.48702	TRUE	1.00E+00
31932_f_at	0.08561	1.217895	0.027406	-0.04083	0.21205	TRUE	1.00E+00
36003_at	0.08562	1.217923	0.028048	-0.04378	0.21502	TRUE	1.00E+00
33297_at	0.08566	1.218036	0.044639	-0.12028	0.29161	TRUE	1.00E+00
36711_at	0.0857	1.218148	0.05696	-0.17709	0.34849	TRUE	1.00E+00
35772_at	0.08574	1.21826	0.027529	-0.04127	0.21274	TRUE	1.00E+00
1177_at	0.08574	1.21826	0.052783	-0.15778	0.32926	TRUE	1.00E+00
1760_s_at	0.08583	1.218513	0.075626	-0.26308	0.43474	TRUE	1.00E+00
41756_at	0.08592	1.218765	0.020948	-0.01072	0.18256	TRUE	5.18E-01
36035_at	0.08599	1.218962	0.0208	-0.00997	0.18195	TRUE	4.50E-01
526_s_at	0.086	1.21899	0.017521	0.00517	0.16683	FALSE	1.16E-02
40748_at	0.08602	1.219046	0.051388	-0.15106	0.32311	TRUE	1.00E+00
38937_at	0.08603	1.219074	0.043677	-0.11548	0.28754	TRUE	1.00E+00
33005_at	0.08606	1.219158	0.100832	-0.37913	0.55126	TRUE	1.00E+00
40176_at	0.08608	1.219214	0.021815	-0.01457	0.18672	TRUE	1.00E+00
40364_at	0.08609	1.219242	0.041155	-0.10378	0.27597	TRUE	1.00E+00
35458_r_at	0.08611	1.219298	0.08568	-0.30919	0.4814	TRUE	1.00E+00
176_at	0.08611	1.219298	0.041774	-0.10662	0.27884	TRUE	1.00E+00
34241_at	0.08617	1.219467	0.077692	-0.27226	0.44461	TRUE	1.00E+00
32194_at	0.08624	1.219663	0.023179	-0.0207	0.19318	TRUE	1.00E+00
40267_s_at	0.08625	1.219692	0.023824	-0.02366	0.19617	TRUE	1.00E+00
33256_at	0.08639	1.220085	0.060765	-0.19395	0.36673	TRUE	1.00E+00
36612_at	0.08639	1.220085	0.049764	-0.14319	0.31598	TRUE	1.00E+00
32659_at	0.08647	1.22031	0.023029	-0.01978	0.19271	TRUE	1.00E+00
38403_at	0.0865	1.220394	0.019532	-0.00361	0.17661	TRUE	1.20E-01
32972_at	0.08653	1.220478	0.146129	-0.58765	0.76071	TRUE	1.00E+00
41436_at	0.08655	1.220534	0.034496	-0.0726	0.24571	TRUE	1.00E+00
37190_at	0.08656	1.220562	0.030366	-0.05353	0.22666	TRUE	1.00E+00
34515_g_at	0.08663	1.220759	0.070958	-0.24074	0.414	TRUE	1.00E+00
37076_at	0.08664	1.220787	0.035721	-0.07817	0.25144	TRUE	1.00E+00
39696_at	0.08675	1.221097	0.075764	-0.26279	0.43629	TRUE	1.00E+00
31494_at	0.08676	1.221125	0.155769	-0.63189	0.80542	TRUE	1.00E+00
40533_at	0.0868	1.221237	0.132162	-0.52294	0.69653	TRUE	1.00E+00
37634_at	0.0868	1.221237	0.030288	-0.05293	0.22654	TRUE	1.00E+00
1593_at	0.08684	1.22135	0.029554	-0.0495	0.22319	TRUE	1.00E+00
37877_at	0.08686	1.221406	0.014325	0.02077	0.15295	FALSE	1.68E-05
35843_at	0.08687	1.221434	0.021992	-0.01459	0.18833	TRUE	9.86E-01
33015_at	0.08688	1.221462	0.167149	-0.68427	0.85804	TRUE	1.00E+00
1125_s_at	0.0869	1.221518	0.024977	-0.02833	0.20214	TRUE	1.00E+00
31757_at	0.08692	1.221575	0.033689	-0.0685	0.24235	TRUE	1.00E+00

34808_at	0.08693	1.221603	0.040625	-0.1005	0.27435	TRUE	1.00E+00
33861_at	0.08698	1.221743	0.008106	0.04958	0.12438	FALSE	9.34E-23
34647_at	0.08703	1.221884	0.020398	-0.00708	0.18113	TRUE	2.51E-01
39896_at	0.08713	1.222165	0.024158	-0.02433	0.19858	TRUE	1.00E+00
32983_at	0.08714	1.222194	0.042524	-0.10904	0.28333	TRUE	1.00E+00
36917_at	0.08717	1.222278	0.066289	-0.21866	0.393	TRUE	1.00E+00
40965_at	0.08729	1.222616	0.047839	-0.13342	0.308	TRUE	1.00E+00
35794_at	0.08731	1.222672	0.037799	-0.08708	0.2617	TRUE	1.00E+00
32672_at	0.08732	1.2227	0.042952	-0.11084	0.28549	TRUE	1.00E+00
547_s_at	0.08735	1.222785	0.084641	-0.30315	0.47784	TRUE	1.00E+00
31668_f_at	0.08741	1.222954	0.064008	-0.2079	0.38271	TRUE	1.00E+00
33154_at	0.08743	1.22301	0.023377	-0.02042	0.19528	TRUE	1.00E+00
1117_at	0.08748	1.223151	0.066565	-0.21963	0.39458	TRUE	1.00E+00
37213_at	0.08748	1.223151	0.07885	-0.2763	0.45126	TRUE	1.00E+00
35089_at	0.08749	1.223179	0.043488	-0.11315	0.28812	TRUE	1.00E+00
35355_at	0.08755	1.223348	0.027891	-0.04113	0.21623	TRUE	1.00E+00
35059_at	0.08756	1.223376	0.026603	-0.03518	0.21029	TRUE	1.00E+00
1505_at	0.08756	1.223376	0.080143	-0.28219	0.45731	TRUE	1.00E+00
35477_at	0.08758	1.223432	0.061829	-0.19767	0.37284	TRUE	1.00E+00
35139_at	0.0876	1.223489	0.028369	-0.04329	0.21848	TRUE	1.00E+00
37179_at	0.08772	1.223827	0.052658	-0.15522	0.33067	TRUE	1.00E+00
31836_at	0.08773	1.223855	0.021673	-0.01226	0.18772	TRUE	6.52E-01
37019_at	0.08777	1.223968	0.056445	-0.17264	0.34818	TRUE	1.00E+00
34879_at	0.08778	1.223996	0.017499	0.00704	0.16852	FALSE	6.66E-03
41495_at	0.08782	1.224109	0.027893	-0.04086	0.21651	TRUE	1.00E+00
34679_at	0.08794	1.224447	0.022856	-0.01751	0.19339	TRUE	1.00E+00
35686_s_at	0.08794	1.224447	0.022281	-0.01485	0.19074	TRUE	9.99E-01
32864_at	0.08795	1.224475	0.052981	-0.15649	0.33238	TRUE	1.00E+00
34272_at	0.08801	1.224644	0.055828	-0.16956	0.34557	TRUE	1.00E+00
350_at	0.08802	1.224673	0.014783	0.01982	0.15622	FALSE	3.29E-05
37485_at	0.08803	1.224701	0.048323	-0.13491	0.31097	TRUE	1.00E+00
40749_at	0.08803	1.224701	0.079433	-0.27844	0.4545	TRUE	1.00E+00
40783_s_at	0.0881	1.224898	0.03116	-0.05566	0.23186	TRUE	1.00E+00
32825_at	0.08811	1.224926	0.019126	-0.00013	0.17635	TRUE	5.17E-02
31879_at	0.08826	1.22535	0.020545	-0.00652	0.18305	TRUE	2.20E-01
40368_r_at	0.08837	1.22566	0.176325	-0.72512	0.90186	TRUE	1.00E+00
36699_at	0.08841	1.225773	0.021463	-0.01061	0.18743	TRUE	4.80E-01
34736_at	0.08846	1.225914	0.083928	-0.29874	0.47567	TRUE	1.00E+00
40839_at	0.08854	1.22614	0.029285	-0.04657	0.22365	TRUE	1.00E+00
35763_at	0.08856	1.226196	0.041323	-0.10209	0.2792	TRUE	1.00E+00
39257_at	0.0886	1.226309	0.034123	-0.06883	0.24603	TRUE	1.00E+00
40754_at	0.08861	1.226337	0.027444	-0.038	0.21523	TRUE	1.00E+00
34444_at	0.08866	1.226479	0.078928	-0.27548	0.4528	TRUE	1.00E+00
370_at	0.08873	1.226676	0.015119	0.01897	0.15848	FALSE	5.55E-05
36975_at	0.08876	1.226761	0.026996	-0.03578	0.21331	TRUE	1.00E+00
34408_at	0.08876	1.226761	0.017478	0.00813	0.1694	FALSE	4.80E-03
853_at	0.08883	1.226959	0.015377	0.01789	0.15977	FALSE	9.61E-05
839_at	0.08885	1.227015	0.185915	-0.76889	0.94658	TRUE	1.00E+00
36463_at	0.08887	1.227072	0.028613	-0.04314	0.22088	TRUE	1.00E+00
31804_f_at	0.08887	1.227072	0.023922	-0.02149	0.19924	TRUE	1.00E+00
36422_s_at	0.08891	1.227185	0.040564	-0.09824	0.27606	TRUE	1.00E+00

33237_at	0.08893	1.227241	0.041145	-0.10089	0.27875	TRUE	1.00E+00
36205_at	0.08896	1.227326	0.013579	0.02631	0.15161	FALSE	7.21E-07
777_at	0.08899	1.227411	0.036847	-0.08101	0.25898	TRUE	1.00E+00
33255_at	0.089	1.227439	0.034111	-0.06837	0.24638	TRUE	1.00E+00
33680_f_at	0.08905	1.227581	0.08518	-0.30393	0.48204	TRUE	1.00E+00
32754_at	0.08916	1.227892	0.062541	-0.19938	0.3777	TRUE	1.00E+00
37131_at	0.08916	1.227892	0.058803	-0.18213	0.36045	TRUE	1.00E+00
31626_i_at	0.08916	1.227892	0.053897	-0.1595	0.33782	TRUE	1.00E+00
1516_g_at	0.08917	1.22792	0.073247	-0.24876	0.42711	TRUE	1.00E+00
32743_at	0.08921	1.228033	0.026574	-0.03338	0.21181	TRUE	1.00E+00
1034_at	0.08931	1.228316	0.029793	-0.04814	0.22676	TRUE	1.00E+00
41372_at	0.08935	1.228429	0.026778	-0.03419	0.21289	TRUE	1.00E+00
33374_at	0.08936	1.228457	0.021697	-0.01074	0.18946	TRUE	4.82E-01
34662_at	0.08939	1.228542	0.034249	-0.06862	0.2474	TRUE	1.00E+00
36942_at	0.08941	1.228599	0.033419	-0.06477	0.24359	TRUE	1.00E+00
32565_at	0.08941	1.228599	0.038103	-0.08638	0.2652	TRUE	1.00E+00
32465_at	0.08948	1.228797	0.037164	-0.08197	0.26094	TRUE	1.00E+00
36188_at	0.08951	1.228881	0.025059	-0.0261	0.20512	TRUE	1.00E+00
41872_at	0.08951	1.228881	0.032925	-0.06239	0.24141	TRUE	1.00E+00
41171_at	0.08954	1.228966	0.02631	-0.03184	0.21092	TRUE	1.00E+00
35408_i_at	0.08959	1.229108	0.032647	-0.06103	0.24021	TRUE	1.00E+00
38589_i_at	0.08962	1.229193	0.064834	-0.2095	0.38874	TRUE	1.00E+00
33567_at	0.08966	1.229306	0.027209	-0.03587	0.21519	TRUE	1.00E+00
40598_at	0.08975	1.229561	0.011699	0.03578	0.14373	FALSE	2.15E-10
32843_s_at	0.08979	1.229674	0.027234	-0.03586	0.21543	TRUE	1.00E+00
738_at	0.0898	1.229702	0.043178	-0.10941	0.28901	TRUE	1.00E+00
40340_at	0.0898	1.229702	0.054511	-0.16169	0.3413	TRUE	1.00E+00
734_at	0.08985	1.229844	0.045489	-0.12001	0.29972	TRUE	1.00E+00
37936_at	0.08991	1.230014	0.017475	0.00929	0.17053	FALSE	3.38E-03
32563_at	0.08994	1.230099	0.025245	-0.02653	0.20641	TRUE	1.00E+00
37856_at	0.08997	1.230184	0.107474	-0.40588	0.58581	TRUE	1.00E+00
492_g_at	0.09002	1.230325	0.034607	-0.06965	0.24968	TRUE	1.00E+00
39441_at	0.09006	1.230439	0.038111	-0.08577	0.26589	TRUE	1.00E+00
40441_g_at	0.09006	1.230439	0.016272	0.01499	0.16513	FALSE	3.94E-04
1728_at	0.09007	1.230467	0.018475	0.00483	0.17531	FALSE	1.37E-02
35613_at	0.0901	1.230552	0.041272	-0.10031	0.28052	TRUE	1.00E+00
38187_at	0.09014	1.230665	0.026116	-0.03035	0.21063	TRUE	1.00E+00
1357_at	0.09015	1.230694	0.016692	0.01314	0.16716	FALSE	8.37E-04
153_f_at	0.09019	1.230807	0.066508	-0.21665	0.39703	TRUE	1.00E+00
39008_at	0.09021	1.230864	0.03857	-0.08773	0.26816	TRUE	1.00E+00
40801_at	0.0903	1.231119	0.046345	-0.12352	0.30412	TRUE	1.00E+00
37424_at	0.09042	1.231459	0.061582	-0.1937	0.37453	TRUE	1.00E+00
747_at	0.09042	1.231459	0.041631	-0.10165	0.28249	TRUE	1.00E+00
35861_at	0.09043	1.231487	0.101351	-0.37716	0.55803	TRUE	1.00E+00
37804_at	0.09046	1.231573	0.024432	-0.02226	0.20318	TRUE	1.00E+00
39427_at	0.09046	1.231573	0.016955	0.01224	0.16869	FALSE	1.20E-03
33904_at	0.09053	1.231771	0.084275	-0.29828	0.47934	TRUE	1.00E+00
673_at	0.0906	1.23197	0.027126	-0.03455	0.21575	TRUE	1.00E+00
34798_at	0.09062	1.232026	0.026683	-0.03249	0.21372	TRUE	1.00E+00
38908_s_at	0.09063	1.232055	0.023396	-0.01731	0.19857	TRUE	1.00E+00
40412_at	0.09067	1.232168	0.081493	-0.2853	0.46665	TRUE	1.00E+00

34719_at	0.09076	1.232424	0.078565	-0.27171	0.45322	TRUE	1.00E+00
35164_at	0.09077	1.232452	0.049848	-0.1392	0.32075	TRUE	1.00E+00
31828_r_at	0.09078	1.23248	0.010752	0.04117	0.14038	FALSE	3.92E-13
38219_at	0.09086	1.232707	0.020832	-0.00525	0.18697	TRUE	1.63E-01
37922_at	0.09088	1.232764	0.015403	0.01982	0.16195	FALSE	4.59E-05
39627_at	0.09089	1.232793	0.051964	-0.14886	0.33063	TRUE	1.00E+00
39132_at	0.0909	1.232821	0.020448	-0.00344	0.18524	TRUE	1.11E-01
41703_r_at	0.09091	1.232849	0.055226	-0.16388	0.3457	TRUE	1.00E+00
38342_at	0.09092	1.232878	0.041821	-0.10202	0.28387	TRUE	1.00E+00
34531_at	0.09096	1.232991	0.037811	-0.08348	0.2654	TRUE	1.00E+00
41290_at	0.09097	1.23302	0.074281	-0.25174	0.43367	TRUE	1.00E+00
38483_at	0.09101	1.233133	0.02403	-0.01985	0.20188	TRUE	1.00E+00
31309_r_at	0.09105	1.233247	0.182179	-0.74945	0.93155	TRUE	1.00E+00
41791_at	0.09106	1.233275	0.033825	-0.065	0.24711	TRUE	1.00E+00
31367_at	0.09106	1.233275	0.04375	-0.11078	0.29291	TRUE	1.00E+00
765_s_at	0.09107	1.233304	0.092077	-0.33374	0.51587	TRUE	1.00E+00
34792_at	0.09107	1.233304	0.009639	0.0466	0.13554	FALSE	4.34E-17
39142_at	0.0911	1.233389	0.026372	-0.03057	0.21277	TRUE	1.00E+00
39953_i_at	0.09123	1.233758	0.030375	-0.0489	0.23137	TRUE	1.00E+00
40610_at	0.09124	1.233786	0.018387	0.00641	0.17607	FALSE	8.80E-03
34226_at	0.09126	1.233843	0.034271	-0.06685	0.24938	TRUE	1.00E+00
2051_at	0.09128	1.2339	0.071833	-0.24012	0.42269	TRUE	1.00E+00
34809_at	0.09128	1.2339	0.03654	-0.0773	0.25987	TRUE	1.00E+00
36573_at	0.09129	1.233929	0.0213	-0.00697	0.18956	TRUE	2.29E-01
39780_at	0.09131	1.233985	0.024087	-0.01982	0.20244	TRUE	1.00E+00
35619_at	0.09136	1.234127	0.055893	-0.1665	0.34923	TRUE	1.00E+00
33221_at	0.09143	1.234326	0.026075	-0.02887	0.21173	TRUE	1.00E+00
39340_at	0.09149	1.234497	0.029497	-0.0446	0.22757	TRUE	1.00E+00
36284_at	0.09151	1.234554	0.032809	-0.05986	0.24287	TRUE	1.00E+00
35786_at	0.09155	1.234667	0.016593	0.015	0.1681	FALSE	4.34E-04
37871_at	0.09156	1.234696	0.067223	-0.21858	0.4017	TRUE	1.00E+00
1121_g_at	0.0916	1.23481	0.084022	-0.29604	0.47924	TRUE	1.00E+00
1683_at	0.09177	1.235293	0.11888	-0.4567	0.64023	TRUE	1.00E+00
37725_at	0.09183	1.235464	0.023254	-0.01545	0.19912	TRUE	9.90E-01
1043_s_at	0.09188	1.235606	0.074339	-0.25109	0.43485	TRUE	1.00E+00
39117_at	0.0919	1.235663	0.023884	-0.01829	0.20209	TRUE	1.00E+00
31661_at	0.09191	1.235691	0.068838	-0.22568	0.4095	TRUE	1.00E+00
38203_at	0.09205	1.23609	0.075085	-0.25436	0.43847	TRUE	1.00E+00
32268_at	0.09213	1.236317	0.082013	-0.28624	0.47051	TRUE	1.00E+00
37616_at	0.09214	1.236346	0.020008	-0.00017	0.18445	TRUE	5.20E-02
986_at	0.09225	1.236659	0.053002	-0.15228	0.33678	TRUE	1.00E+00
1317_at	0.09226	1.236688	0.042948	-0.10589	0.2904	TRUE	1.00E+00
34308_at	0.09226	1.236688	0.06811	-0.22197	0.40649	TRUE	1.00E+00
35261_at	0.09226	1.236688	0.078899	-0.27175	0.45627	TRUE	1.00E+00
40403_at	0.09233	1.236887	0.124439	-0.48178	0.66644	TRUE	1.00E+00
39458_s_at	0.09238	1.237029	0.065822	-0.21129	0.39606	TRUE	1.00E+00
37369_s_at	0.09242	1.237143	0.042924	-0.10561	0.29045	TRUE	1.00E+00
39848_at	0.09248	1.237314	0.067947	-0.221	0.40596	TRUE	1.00E+00
39794_at	0.09254	1.237485	0.027209	-0.03299	0.21807	TRUE	1.00E+00
41740_at	0.09256	1.237542	0.055181	-0.16202	0.34714	TRUE	1.00E+00
34761_r_at	0.09262	1.237713	0.026868	-0.03134	0.21658	TRUE	1.00E+00

33827_at	0.09276	1.238112	0.053733	-0.15514	0.34066	TRUE	1.00E+00
39450_s_at	0.09279	1.238198	0.033054	-0.05971	0.24528	TRUE	1.00E+00
394_at	0.09279	1.238198	0.033857	-0.06341	0.24899	TRUE	1.00E+00
33720_at	0.09281	1.238255	0.016577	0.01633	0.16929	FALSE	2.72E-04
36103_at	0.09282	1.238283	0.041895	-0.10047	0.2861	TRUE	1.00E+00
36032_at	0.09283	1.238312	0.034186	-0.06489	0.25055	TRUE	1.00E+00
35180_at	0.09286	1.238397	0.022708	-0.0119	0.19763	TRUE	5.46E-01
137_at	0.09288	1.238454	0.052327	-0.14853	0.3343	TRUE	1.00E+00
39676_at	0.09289	1.238483	0.05402	-0.15634	0.34211	TRUE	1.00E+00
35941_f_at	0.09289	1.238483	0.047229	-0.125	0.31079	TRUE	1.00E+00
1254_at	0.0929	1.238511	0.053978	-0.15613	0.34193	TRUE	1.00E+00
39294_at	0.09294	1.238625	0.036744	-0.07658	0.26246	TRUE	1.00E+00
1849_s_at	0.09294	1.238625	0.122176	-0.47073	0.65661	TRUE	1.00E+00
37104_at	0.09299	1.238768	0.1159	-0.44172	0.6277	TRUE	1.00E+00
38475_at	0.09302	1.238854	0.015047	0.0236	0.16244	FALSE	7.97E-06
31610_at	0.09306	1.238968	0.118766	-0.45487	0.641	TRUE	1.00E+00
32892_at	0.09314	1.239196	0.052785	-0.15039	0.33666	TRUE	1.00E+00
1568_s_at	0.09315	1.239225	0.033965	-0.06355	0.24985	TRUE	1.00E+00
1236_s_at	0.09319	1.239339	0.116909	-0.44618	0.63256	TRUE	1.00E+00
40884_g_at	0.09322	1.239424	0.034562	-0.06624	0.25267	TRUE	1.00E+00
1726_at	0.09323	1.239453	0.045584	-0.11708	0.30354	TRUE	1.00E+00
216_at	0.09324	1.239481	0.077722	-0.26534	0.45181	TRUE	1.00E+00
38150_at	0.0933	1.239653	0.024845	-0.02132	0.20793	TRUE	1.00E+00
270_at	0.09334	1.239767	0.107533	-0.40277	0.58945	TRUE	1.00E+00
40273_at	0.09336	1.239824	0.023999	-0.01736	0.20408	TRUE	1.00E+00
41396_at	0.09337	1.239852	0.027187	-0.03206	0.21879	TRUE	1.00E+00
36698_at	0.09341	1.239967	0.05819	-0.17506	0.36187	TRUE	1.00E+00
960_g_at	0.09345	1.240081	0.120154	-0.46089	0.64779	TRUE	1.00E+00
33590_at	0.09346	1.240109	0.122302	-0.47079	0.65771	TRUE	1.00E+00
38648_at	0.09348	1.240167	0.016409	0.01777	0.16918	FALSE	1.54E-04
37857_at	0.0935	1.240224	0.055536	-0.16272	0.34972	TRUE	1.00E+00
38115_at	0.09353	1.240309	0.034216	-0.06433	0.25139	TRUE	1.00E+00
31690_at	0.09353	1.240309	0.026663	-0.02948	0.21654	TRUE	1.00E+00
35303_at	0.09353	1.240309	0.099562	-0.36581	0.55287	TRUE	1.00E+00
38761_s_at	0.09356	1.240395	0.052127	-0.14693	0.33405	TRUE	1.00E+00
38044_at	0.09358	1.240452	0.035276	-0.06917	0.25632	TRUE	1.00E+00
39349_at	0.09361	1.240538	0.056656	-0.16778	0.35499	TRUE	1.00E+00
38188_s_at	0.09363	1.240595	0.035108	-0.06835	0.2556	TRUE	1.00E+00
1299_at	0.09364	1.240623	0.032544	-0.0565	0.24379	TRUE	1.00E+00
32051_at	0.09364	1.240623	0.011962	0.03845	0.14883	FALSE	6.26E-11
34811_at	0.0937	1.240795	0.022537	-0.01027	0.19767	TRUE	4.06E-01
39054_at	0.09373	1.240881	0.020607	-0.00134	0.18881	TRUE	6.82E-02
39060_at	0.09374	1.240909	0.024261	-0.01819	0.20567	TRUE	1.00E+00
41571_at	0.09378	1.241023	0.071853	-0.23772	0.42528	TRUE	1.00E+00
39977_at	0.09386	1.241252	0.028	-0.03532	0.22304	TRUE	1.00E+00
35315_at	0.09387	1.241281	0.073933	-0.24722	0.43497	TRUE	1.00E+00
32131_at	0.0939	1.241366	0.03484	-0.06684	0.25464	TRUE	1.00E+00
32230_at	0.09395	1.241509	0.02522	-0.02241	0.2103	TRUE	1.00E+00
39422_at	0.09401	1.241681	0.061734	-0.1908	0.37883	TRUE	1.00E+00
36182_at	0.09407	1.241852	0.058077	-0.17387	0.36201	TRUE	1.00E+00
40474_r_at	0.09409	1.24191	0.062962	-0.19639	0.38457	TRUE	1.00E+00

36794_at	0.0941	1.241938	0.046529	-0.12057	0.30876	TRUE	1.00E+00
37914_at	0.09411	1.241967	0.034864	-0.06674	0.25496	TRUE	1.00E+00
35504_at	0.09412	1.241995	0.037954	-0.08098	0.26922	TRUE	1.00E+00
33313_g_at	0.09413	1.242024	0.07097	-0.2333	0.42156	TRUE	1.00E+00
562_g_at	0.09413	1.242024	0.078113	-0.26625	0.45451	TRUE	1.00E+00
37336_at	0.09416	1.24211	0.018529	0.00868	0.17964	FALSE	4.72E-03
32395_r_at	0.09424	1.242339	0.016969	0.01595	0.17253	FALSE	3.53E-04
1297_at	0.09428	1.242453	0.089529	-0.31877	0.50733	TRUE	1.00E+00
33864_at	0.09429	1.242482	0.029699	-0.04272	0.23131	TRUE	1.00E+00
1930_at	0.09438	1.242739	0.082434	-0.28593	0.4747	TRUE	1.00E+00
37970_at	0.0944	1.242796	0.026923	-0.02981	0.21862	TRUE	1.00E+00
33933_at	0.09441	1.242825	0.083022	-0.28862	0.47743	TRUE	1.00E+00
33653_at	0.09443	1.242882	0.135416	-0.53033	0.71918	TRUE	1.00E+00
39168_at	0.09443	1.242882	0.015688	0.02205	0.16681	FALSE	2.21E-05
40342_at	0.09446	1.242968	0.046838	-0.12163	0.31055	TRUE	1.00E+00
38492_at	0.09448	1.243025	0.035822	-0.07079	0.25975	TRUE	1.00E+00
146_at	0.09449	1.243054	0.011822	0.03995	0.14903	FALSE	1.66E-11
38256_s_at	0.09451	1.243111	0.012116	0.03861	0.15041	FALSE	7.77E-11
41082_at	0.09458	1.243312	0.074714	-0.25013	0.43928	TRUE	1.00E+00
41274_at	0.09459	1.24334	0.025322	-0.02223	0.21142	TRUE	1.00E+00
35751_at	0.09459	1.24334	0.015221	0.02437	0.16482	FALSE	6.48E-06
36648_at	0.09462	1.243426	0.030873	-0.04782	0.23705	TRUE	1.00E+00
2043_s_at	0.09465	1.243512	0.051296	-0.14201	0.33131	TRUE	1.00E+00
32151_at	0.09469	1.243627	0.023344	-0.01301	0.20239	TRUE	6.30E-01
40756_at	0.09471	1.243684	0.026714	-0.02854	0.21796	TRUE	1.00E+00
32974_at	0.09471	1.243684	0.099906	-0.36621	0.55564	TRUE	1.00E+00
33244_at	0.09475	1.243798	0.099529	-0.36444	0.55394	TRUE	1.00E+00
1856_at	0.09476	1.243827	0.045824	-0.11665	0.30618	TRUE	1.00E+00
39650_s_at	0.09482	1.243999	0.035572	-0.06929	0.25894	TRUE	1.00E+00
32804_at	0.09483	1.244028	0.022322	-0.00815	0.19782	TRUE	2.72E-01
40394_at	0.0949	1.244228	0.039467	-0.08718	0.27699	TRUE	1.00E+00
40221_at	0.09491	1.244257	0.028036	-0.03444	0.22425	TRUE	1.00E+00
1059_at	0.09495	1.244371	0.097897	-0.3567	0.54661	TRUE	1.00E+00
190_at	0.09503	1.244601	0.042072	-0.09908	0.28913	TRUE	1.00E+00
41238_s_at	0.09506	1.244687	0.050973	-0.14011	0.33023	TRUE	1.00E+00
35120_at	0.09507	1.244715	0.058597	-0.17527	0.36541	TRUE	1.00E+00
1924_at	0.09517	1.245002	0.015604	0.02318	0.16716	FALSE	1.35E-05
37673_at	0.09522	1.245145	0.023457	-0.013	0.20344	TRUE	6.22E-01
34078_s_at	0.09522	1.245145	0.102379	-0.37712	0.56756	TRUE	1.00E+00
37959_at	0.09524	1.245203	0.030928	-0.04745	0.23793	TRUE	1.00E+00
32304_at	0.09525	1.245231	0.039594	-0.08742	0.27791	TRUE	1.00E+00
32073_at	0.09527	1.245289	0.034089	-0.062	0.25254	TRUE	1.00E+00
41780_at	0.09533	1.245461	0.037249	-0.07652	0.26718	TRUE	1.00E+00
32725_at	0.09536	1.245547	0.024374	-0.01709	0.20781	TRUE	1.00E+00
41186_at	0.09546	1.245833	0.148144	-0.58802	0.77894	TRUE	1.00E+00
32092_at	0.09548	1.245891	0.027751	-0.03255	0.22351	TRUE	1.00E+00
41183_at	0.09548	1.245891	0.021514	-0.00378	0.19474	TRUE	1.15E-01
33840_at	0.09556	1.24612	0.033709	-0.05996	0.25108	TRUE	1.00E+00
37693_at	0.09557	1.246149	0.025549	-0.02231	0.21344	TRUE	1.00E+00
37487_at	0.09561	1.246264	0.031435	-0.04942	0.24063	TRUE	1.00E+00
33385_g_at	0.09562	1.246293	0.017284	0.01587	0.17536	FALSE	4.00E-04

33708_at	0.09563	1.246321	0.043827	-0.10657	0.29783	TRUE	1.00E+00
1895_at	0.0957	1.246522	0.028369	-0.03519	0.22658	TRUE	1.00E+00
31870_at	0.09573	1.246608	0.014917	0.02691	0.16455	FALSE	1.75E-06
38921_at	0.09577	1.246723	0.059315	-0.17788	0.36943	TRUE	1.00E+00
35508_at	0.0958	1.246809	0.103249	-0.38055	0.57214	TRUE	1.00E+00
38175_at	0.0959	1.247096	0.064453	-0.20146	0.39326	TRUE	1.00E+00
31763_at	0.09603	1.24747	0.12801	-0.49455	0.68662	TRUE	1.00E+00
37570_at	0.09612	1.247728	0.034727	-0.0641	0.25633	TRUE	1.00E+00
34404_at	0.09617	1.247872	0.025143	-0.01982	0.21217	TRUE	1.00E+00
169_at	0.09619	1.247929	0.079675	-0.2714	0.46378	TRUE	1.00E+00
40859_at	0.09621	1.247987	0.011153	0.04476	0.14766	FALSE	7.98E-14
34329_at	0.09624	1.248073	0.015291	0.0257	0.16679	FALSE	3.91E-06
40477_r_at	0.09625	1.248102	0.028748	-0.03637	0.22888	TRUE	1.00E+00
644_at	0.09626	1.248131	0.063852	-0.19833	0.39085	TRUE	1.00E+00
41784_at	0.09626	1.248131	0.021972	-0.00511	0.19763	TRUE	1.49E-01
36616_at	0.09629	1.248217	0.015302	0.02569	0.16689	FALSE	3.94E-06
40050_at	0.09635	1.248389	0.020984	-0.00046	0.19316	TRUE	5.55E-02
34184_at	0.09636	1.248418	0.032934	-0.05559	0.2483	TRUE	1.00E+00
41176_at	0.09637	1.248447	0.025302	-0.02036	0.21311	TRUE	1.00E+00
34598_at	0.09643	1.248619	0.088164	-0.31032	0.50318	TRUE	1.00E+00
39637_at	0.09655	1.248964	0.053663	-0.15103	0.34413	TRUE	1.00E+00
39960_at	0.09657	1.249022	0.012878	0.03716	0.15598	FALSE	8.12E-10
39133_at	0.09661	1.249137	0.022376	-0.00662	0.19985	TRUE	1.99E-01
41652_at	0.0967	1.249396	0.068967	-0.22148	0.41489	TRUE	1.00E+00
34911_r_at	0.09675	1.24954	0.180524	-0.73612	0.92961	TRUE	1.00E+00
39882_at	0.09676	1.249568	0.066032	-0.20788	0.40141	TRUE	1.00E+00
2034_s_at	0.09684	1.249799	0.046083	-0.11577	0.30945	TRUE	1.00E+00
35498_at	0.09685	1.249827	0.058452	-0.17282	0.36652	TRUE	1.00E+00
39708_at	0.09686	1.249856	0.029509	-0.03928	0.233	TRUE	1.00E+00
32167_at	0.09694	1.250086	0.010163	0.05006	0.14383	FALSE	1.82E-17
36620_at	0.09696	1.250144	0.0065	0.06697	0.12694	FALSE	3.19E-46
31532_at	0.09707	1.250461	0.062013	-0.18903	0.38318	TRUE	1.00E+00
40287_s_at	0.09713	1.250633	0.038401	-0.08003	0.2743	TRUE	1.00E+00
36191_at	0.09726	1.251008	0.038478	-0.08027	0.27478	TRUE	1.00E+00
801_at	0.09735	1.251267	0.059309	-0.17628	0.37098	TRUE	1.00E+00
39044_s_at	0.09741	1.25144	0.030304	-0.0424	0.23722	TRUE	1.00E+00
37940_f_at	0.09742	1.251469	0.030053	-0.04123	0.23607	TRUE	1.00E+00
184_at	0.09747	1.251613	0.055804	-0.15999	0.35492	TRUE	1.00E+00
39664_at	0.09751	1.251728	0.029963	-0.04072	0.23575	TRUE	1.00E+00
33881_at	0.09761	1.252016	0.046346	-0.11621	0.31143	TRUE	1.00E+00
37023_at	0.09764	1.252103	0.070992	-0.22989	0.42517	TRUE	1.00E+00
1049_g_at	0.09767	1.252189	0.03602	-0.06851	0.26386	TRUE	1.00E+00
40129_at	0.09776	1.252449	0.027011	-0.02686	0.22238	TRUE	1.00E+00
487_g_at	0.09781	1.252593	0.017903	0.01521	0.18041	FALSE	5.90E-04
38250_at	0.09783	1.252651	0.037095	-0.07331	0.26897	TRUE	1.00E+00
39072_at	0.0979	1.252853	0.063058	-0.19302	0.38883	TRUE	1.00E+00
39212_at	0.09793	1.252939	0.063938	-0.19706	0.39291	TRUE	1.00E+00
32162_r_at	0.09796	1.253026	0.040061	-0.08687	0.28278	TRUE	1.00E+00
35973_at	0.09799	1.253112	0.038171	-0.07812	0.27409	TRUE	1.00E+00
544_at	0.098	1.253141	0.042996	-0.10037	0.29636	TRUE	1.00E+00
31936_s_at	0.09801	1.25317	0.033397	-0.05607	0.25209	TRUE	1.00E+00

962_at	0.09801	1.25317	0.063441	-0.19467	0.3907	TRUE	1.00E+00
140_s_at	0.09802	1.253199	0.019478	0.00816	0.18789	FALSE	6.12E-03
1479_g_at	0.09803	1.253228	0.089373	-0.3143	0.51036	TRUE	1.00E+00
35986_at	0.09803	1.253228	0.05199	-0.14183	0.3379	TRUE	1.00E+00
37058_at	0.09807	1.253343	0.145837	-0.57476	0.77091	TRUE	1.00E+00
31520_at	0.09809	1.253401	0.077479	-0.25936	0.45555	TRUE	1.00E+00
41440_at	0.09827	1.25392	0.043098	-0.10057	0.2971	TRUE	1.00E+00
722_at	0.09828	1.253949	0.068554	-0.218	0.41455	TRUE	1.00E+00
39421_at	0.09829	1.253978	0.037973	-0.0769	0.27348	TRUE	1.00E+00
31521_f_at	0.09833	1.254094	0.109958	-0.40897	0.60564	TRUE	1.00E+00
36511_at	0.09834	1.254123	0.018537	0.01282	0.18386	FALSE	1.42E-03
35894_at	0.09838	1.254238	0.061433	-0.18504	0.38181	TRUE	1.00E+00
32444_at	0.09844	1.254411	0.01844	0.01337	0.18352	FALSE	1.18E-03
33365_at	0.09849	1.254556	0.026037	-0.02164	0.21862	TRUE	1.00E+00
37974_at	0.0985	1.254585	0.023566	-0.01023	0.20722	TRUE	3.69E-01
35854_at	0.09856	1.254758	0.044374	-0.10617	0.30328	TRUE	1.00E+00
33845_at	0.09857	1.254787	0.028871	-0.03463	0.23177	TRUE	1.00E+00
1228_s_at	0.0986	1.254874	0.026434	-0.02336	0.22056	TRUE	1.00E+00
34110_g_at	0.09861	1.254903	0.08679	-0.3018	0.49902	TRUE	1.00E+00
37539_at	0.09862	1.254931	0.028311	-0.03199	0.22924	TRUE	1.00E+00
34196_at	0.09865	1.255018	0.025616	-0.01953	0.21683	TRUE	1.00E+00
948_s_at	0.09866	1.255047	0.067047	-0.21067	0.40799	TRUE	1.00E+00
34267_r_at	0.09881	1.255481	0.078635	-0.26398	0.4616	TRUE	1.00E+00
33065_at	0.09885	1.255596	0.10341	-0.37824	0.57594	TRUE	1.00E+00
40551_at	0.09891	1.25577	0.076977	-0.25623	0.45405	TRUE	1.00E+00
41398_at	0.09902	1.256088	0.020213	0.00576	0.19227	FALSE	1.22E-02
32676_at	0.09902	1.256088	0.036805	-0.07078	0.26883	TRUE	1.00E+00
39686_g_at	0.09909	1.25629	0.030795	-0.04298	0.24117	TRUE	1.00E+00
40428_i_at	0.09914	1.256435	0.13249	-0.51211	0.71039	TRUE	1.00E+00
40118_at	0.09919	1.25658	0.056163	-0.15992	0.3583	TRUE	1.00E+00
31444_s_at	0.09921	1.256637	0.070954	-0.22814	0.42657	TRUE	1.00E+00
36863_at	0.09932	1.256956	0.121401	-0.46078	0.65941	TRUE	1.00E+00
1119_at	0.09932	1.256956	0.015898	0.02598	0.17267	FALSE	5.27E-06
37787_at	0.09938	1.257129	0.116906	-0.43998	0.63873	TRUE	1.00E+00
40870_g_at	0.09944	1.257303	0.01942	0.00985	0.18904	FALSE	3.85E-03
32123_at	0.0995	1.257477	0.03373	-0.05612	0.25512	TRUE	1.00E+00
1838_g_at	0.09952	1.257535	0.042305	-0.09566	0.29469	TRUE	1.00E+00
38768_at	0.09953	1.257564	0.034294	-0.05869	0.25775	TRUE	1.00E+00
40464_g_at	0.09963	1.257853	0.01939	0.01017	0.18909	FALSE	3.50E-03
39454_f_at	0.09963	1.257853	0.04684	-0.11647	0.31574	TRUE	1.00E+00
34629_at	0.09964	1.257882	0.057667	-0.16641	0.36569	TRUE	1.00E+00
35320_at	0.09969	1.258027	0.0258	-0.01934	0.21872	TRUE	1.00E+00
36925_at	0.09973	1.258143	0.044561	-0.10586	0.30531	TRUE	1.00E+00
37229_at	0.09977	1.258259	0.021529	0.00044	0.1991	FALSE	4.53E-02
37535_at	0.0998	1.258346	0.016238	0.02489	0.17472	FALSE	1.00E-05
40119_at	0.09982	1.258404	0.034417	-0.05896	0.25861	TRUE	1.00E+00
1778_g_at	0.09984	1.258462	0.049391	-0.12804	0.32771	TRUE	1.00E+00
40449_at	0.09986	1.25852	0.060027	-0.17708	0.3768	TRUE	1.00E+00
31457_at	0.0999	1.258636	0.029902	-0.03806	0.23785	TRUE	1.00E+00
35071_s_at	0.09995	1.25878	0.127304	-0.48737	0.68728	TRUE	1.00E+00
34659_at	0.09997	1.258838	0.02867	-0.0323	0.23225	TRUE	1.00E+00

32889_at	0.10003	1.259012	0.134936	-0.52251	0.72257	TRUE	1.00E+00
41869_at	0.1001	1.259215	0.026486	-0.02209	0.2223	TRUE	1.00E+00
38687_at	0.1001	1.259215	0.033241	-0.05326	0.25346	TRUE	1.00E+00
36682_at	0.10012	1.259273	0.050212	-0.13154	0.33178	TRUE	1.00E+00
671_at	0.10014	1.259331	0.026021	-0.01991	0.22019	TRUE	1.00E+00
33656_at	0.10016	1.259389	0.017879	0.01767	0.18265	FALSE	2.67E-04
40244_s_at	0.10017	1.259418	0.029168	-0.0344	0.23474	TRUE	1.00E+00
41003_at	0.10019	1.259476	0.028754	-0.03247	0.23285	TRUE	1.00E+00
1544_at	0.10023	1.259592	0.069675	-0.22122	0.42168	TRUE	1.00E+00
34928_at	0.10024	1.259621	0.013923	0.036	0.16447	FALSE	7.63E-09
40672_at	0.10026	1.259679	0.036608	-0.06864	0.26916	TRUE	1.00E+00
35121_at	0.10028	1.259737	0.082436	-0.28005	0.48061	TRUE	1.00E+00
32674_at	0.10028	1.259737	0.022706	-0.00447	0.20504	TRUE	1.27E-01
31449_at	0.10037	1.259998	0.109632	-0.40543	0.60616	TRUE	1.00E+00
39707_at	0.1004	1.260085	0.02531	-0.01637	0.21717	TRUE	9.20E-01
33724_at	0.1004	1.260085	0.0242	-0.01125	0.21205	TRUE	4.22E-01
1208_at	0.10044	1.260202	0.033338	-0.05337	0.25424	TRUE	1.00E+00
31355_at	0.10048	1.260318	0.049112	-0.1261	0.32707	TRUE	1.00E+00
35394_at	0.10051	1.260405	0.023467	-0.00776	0.20878	TRUE	2.33E-01
41506_at	0.10053	1.260463	0.024527	-0.01263	0.21369	TRUE	5.25E-01
34829_at	0.10054	1.260492	0.03071	-0.04114	0.24223	TRUE	1.00E+00
32784_at	0.10058	1.260608	0.038168	-0.07551	0.27667	TRUE	1.00E+00
34528_at	0.1006	1.260666	0.102963	-0.37443	0.57563	TRUE	1.00E+00
38198_at	0.10068	1.260898	0.027174	-0.02469	0.22605	TRUE	1.00E+00
745_at	0.10069	1.260927	0.052754	-0.1427	0.34407	TRUE	1.00E+00
803_at	0.10071	1.260985	0.067491	-0.21067	0.41208	TRUE	1.00E+00
33746_at	0.10072	1.261014	0.018984	0.01314	0.1883	FALSE	1.42E-03
40769_r_at	0.10089	1.261508	0.070445	-0.22412	0.42589	TRUE	1.00E+00
37588_s_at	0.1009	1.261537	0.031157	-0.04284	0.24465	TRUE	1.00E+00
affx-hsac07/x003	0.10091	1.261566	0.064294	-0.19571	0.39754	TRUE	1.00E+00
33067_at	0.10094	1.261653	0.081569	-0.27539	0.47726	TRUE	1.00E+00
36077_at	0.10098	1.261769	0.03749	-0.07198	0.27395	TRUE	1.00E+00
41058_g_at	0.10105	1.261973	0.017806	0.01889	0.1832	FALSE	1.75E-04
34974_at	0.10108	1.26206	0.109613	-0.40463	0.60679	TRUE	1.00E+00
36928_at	0.10108	1.26206	0.024925	-0.01391	0.21607	TRUE	6.32E-01
40565_at	0.10108	1.26206	0.026263	-0.02009	0.22225	TRUE	1.00E+00
34336_at	0.10109	1.262089	0.013167	0.04034	0.16183	FALSE	2.05E-10
35845_at	0.10119	1.26238	0.028164	-0.02875	0.23113	TRUE	1.00E+00
36637_at	0.10126	1.262583	0.030659	-0.04019	0.24271	TRUE	1.00E+00
36118_at	0.10127	1.262612	0.019869	0.0096	0.19294	FALSE	4.36E-03
33494_at	0.10127	1.262612	0.02337	-0.00654	0.20909	TRUE	1.85E-01
40615_at	0.10128	1.262641	0.01757	0.02022	0.18234	FALSE	1.03E-04
40770_f_at	0.10132	1.262758	0.032872	-0.05034	0.25298	TRUE	1.00E+00
35224_at	0.10134	1.262816	0.020362	0.0074	0.19529	FALSE	8.15E-03
41754_at	0.10139	1.262961	0.017215	0.02197	0.18082	FALSE	4.89E-05
1954_at	0.10139	1.262961	0.048813	-0.12381	0.3266	TRUE	1.00E+00
32185_at	0.10142	1.263048	0.027806	-0.02687	0.2297	TRUE	1.00E+00
33039_at	0.10146	1.263165	0.159339	-0.63367	0.83658	TRUE	1.00E+00
258_at	0.10147	1.263194	0.039892	-0.08257	0.28552	TRUE	1.00E+00
34132_at	0.10153	1.263368	0.042299	-0.09362	0.29668	TRUE	1.00E+00
35021_at	0.10157	1.263485	0.059451	-0.17272	0.37585	TRUE	1.00E+00

40912_s_at	0.10163	1.263659	0.012003	0.04626	0.15701	FALSE	3.17E-13
39623_at	0.10164	1.263688	0.023686	-0.00764	0.21091	TRUE	2.24E-01
36721_s_at	0.10168	1.263805	0.042306	-0.0935	0.29686	TRUE	1.00E+00
39943_at	0.10172	1.263921	0.029049	-0.0323	0.23574	TRUE	1.00E+00
35999_r_at	0.10178	1.264096	0.019435	0.01211	0.19144	FALSE	2.06E-03
36949_at	0.1018	1.264154	0.010445	0.05361	0.14999	FALSE	2.42E-18
35994_at	0.10186	1.264329	0.017441	0.02139	0.18233	FALSE	6.59E-05
34857_at	0.10192	1.264503	0.048442	-0.12157	0.32541	TRUE	1.00E+00
33556_at	0.10192	1.264503	0.021654	0.00202	0.20183	FALSE	3.18E-02
39237_at	0.10196	1.26462	0.047746	-0.11832	0.32223	TRUE	1.00E+00
32120_at	0.10201	1.264765	0.0905	-0.31551	0.51954	TRUE	1.00E+00
1078_at	0.10208	1.264969	0.107355	-0.39321	0.59738	TRUE	1.00E+00
39353_at	0.10209	1.264998	0.020656	0.00679	0.19738	FALSE	9.75E-03
40981_at	0.10211	1.265057	0.024546	-0.01114	0.21535	TRUE	4.02E-01
36626_at	0.10223	1.265406	0.010311	0.05466	0.1498	FALSE	4.54E-19
35978_at	0.1023	1.26561	0.04135	-0.08847	0.29307	TRUE	1.00E+00
32999_at	0.10234	1.265727	0.103621	-0.37573	0.5804	TRUE	1.00E+00
32655_s_at	0.10234	1.265727	0.025488	-0.01525	0.21993	TRUE	7.50E-01
32958_at	0.10247	1.266106	0.058249	-0.16626	0.37121	TRUE	1.00E+00
1083_s_at	0.10249	1.266164	0.038936	-0.07714	0.28212	TRUE	1.00E+00
38601_at	0.10255	1.266339	0.046442	-0.11171	0.31681	TRUE	1.00E+00
38902_r_at	0.10255	1.266339	0.03787	-0.07217	0.27727	TRUE	1.00E+00
32421_at	0.10256	1.266368	0.051872	-0.13676	0.34188	TRUE	1.00E+00
41678_at	0.10259	1.266456	0.030629	-0.03872	0.24391	TRUE	1.00E+00
346_s_at	0.10265	1.266631	0.034381	-0.05597	0.26127	TRUE	1.00E+00
34875_r_at	0.10277	1.266981	0.056321	-0.15707	0.36261	TRUE	1.00E+00
269_at	0.10279	1.267039	0.067533	-0.20878	0.41436	TRUE	1.00E+00
33605_at	0.1028	1.267068	0.020106	0.01004	0.19556	FALSE	4.00E-03
37739_at	0.10284	1.267185	0.01965	0.01218	0.1935	FALSE	2.10E-03
41059_at	0.10284	1.267185	0.044272	-0.10141	0.30709	TRUE	1.00E+00
39346_at	0.10286	1.267243	0.009691	0.05815	0.14757	FALSE	3.25E-22
40282_s_at	0.10286	1.267243	0.040398	-0.08352	0.28924	TRUE	1.00E+00
560_s_at	0.10286	1.267243	0.098611	-0.35209	0.55781	TRUE	1.00E+00
41250_at	0.10289	1.267331	0.021631	0.00309	0.20269	FALSE	2.49E-02
41803_g_at	0.10291	1.267389	0.078865	-0.26094	0.46676	TRUE	1.00E+00
39926_at	0.10292	1.267418	0.039641	-0.07996	0.28581	TRUE	1.00E+00
32645_at	0.10294	1.267477	0.039106	-0.07748	0.28335	TRUE	1.00E+00
1884_s_at	0.10297	1.267564	0.049827	-0.12692	0.33285	TRUE	1.00E+00
36771_at	0.10305	1.267798	0.032427	-0.04655	0.25265	TRUE	1.00E+00
34852_g_at	0.10306	1.267827	0.10406	-0.37704	0.58315	TRUE	1.00E+00
36524_at	0.10314	1.268061	0.090418	-0.31401	0.52029	TRUE	1.00E+00
34214_at	0.10319	1.268207	0.064561	-0.19467	0.40105	TRUE	1.00E+00
39656_at	0.10327	1.26844	0.034284	-0.0549	0.26144	TRUE	1.00E+00
34473_at	0.10327	1.26844	0.143523	-0.55888	0.76543	TRUE	1.00E+00
33847_s_at	0.10328	1.268469	0.047389	-0.11535	0.32191	TRUE	1.00E+00
38544_at	0.10328	1.268469	0.060563	-0.17613	0.3827	TRUE	1.00E+00
33877_s_at	0.10332	1.268586	0.017467	0.02273	0.1839	FALSE	4.19E-05
32935_at	0.10335	1.268674	0.039247	-0.07772	0.28442	TRUE	1.00E+00
33278_at	0.1034	1.26882	0.071907	-0.22835	0.43515	TRUE	1.00E+00
32660_at	0.10351	1.269141	0.030121	-0.03546	0.24248	TRUE	1.00E+00
40440_at	0.10353	1.2692	0.029899	-0.03441	0.24148	TRUE	1.00E+00

1233_s_at	0.10359	1.269375	0.066002	-0.20092	0.40809	TRUE	1.00E+00
36691_at	0.10361	1.269434	0.05105	-0.13192	0.33913	TRUE	1.00E+00
1648_at	0.10361	1.269434	0.061842	-0.1817	0.38892	TRUE	1.00E+00
32232_at	0.10362	1.269463	0.010817	0.05372	0.15353	FALSE	1.23E-17
33619_at	0.10368	1.269638	0.032439	-0.04598	0.25334	TRUE	1.00E+00
41831_at	0.10374	1.269814	0.031221	-0.0403	0.24778	TRUE	1.00E+00
31813_at	0.10378	1.269931	0.036255	-0.06349	0.27104	TRUE	1.00E+00
34098_f_at	0.1038	1.269989	0.08845	-0.30427	0.51187	TRUE	1.00E+00
41691_at	0.10382	1.270048	0.024595	-0.00965	0.21729	TRUE	3.07E-01
33885_at	0.10389	1.270252	0.025391	-0.01326	0.22103	TRUE	5.41E-01
34676_at	0.10391	1.270311	0.042056	-0.09012	0.29793	TRUE	1.00E+00
36050_at	0.10393	1.270369	0.021244	0.00592	0.20194	FALSE	1.26E-02
37090_at	0.10396	1.270457	0.090148	-0.31195	0.51987	TRUE	1.00E+00
40332_at	0.10397	1.270486	0.023404	-0.004	0.21195	TRUE	1.12E-01
31357_at	0.10404	1.270691	0.087182	-0.29818	0.50626	TRUE	1.00E+00
35800_at	0.10412	1.270925	0.05253	-0.13823	0.34647	TRUE	1.00E+00
37844_at	0.10414	1.270984	0.086197	-0.29354	0.50182	TRUE	1.00E+00
39478_at	0.10419	1.27113	0.049214	-0.12286	0.33125	TRUE	1.00E+00
37041_at	0.10423	1.271247	0.044616	-0.10161	0.31007	TRUE	1.00E+00
34688_at	0.10423	1.271247	0.026745	-0.01916	0.22763	TRUE	1.00E+00
39481_at	0.10427	1.271364	0.035125	-0.05778	0.26632	TRUE	1.00E+00
39565_at	0.10432	1.271511	0.023597	-0.00454	0.21319	TRUE	1.24E-01
32042_at	0.10434	1.271569	0.020066	0.01177	0.19692	FALSE	2.51E-03
40976_at	0.10436	1.271628	0.026153	-0.0163	0.22502	TRUE	8.32E-01
35500_at	0.10438	1.271686	0.026133	-0.01618	0.22495	TRUE	8.19E-01
31533_s_at	0.10442	1.271803	0.074818	-0.24076	0.44959	TRUE	1.00E+00
41182_at	0.10446	1.271921	0.06399	-0.19076	0.39969	TRUE	1.00E+00
38511_at	0.10449	1.272008	0.058646	-0.16608	0.37506	TRUE	1.00E+00
34448_s_at	0.1045	1.272038	0.058329	-0.16461	0.3736	TRUE	1.00E+00
37356_r_at	0.10451	1.272067	0.019772	0.01329	0.19573	FALSE	1.58E-03
36685_at	0.10462	1.272389	0.035341	-0.05843	0.26767	TRUE	1.00E+00
33738_r_at	0.10466	1.272506	0.033066	-0.04789	0.25721	TRUE	1.00E+00
38509_at	0.10467	1.272536	0.035963	-0.06125	0.27059	TRUE	1.00E+00
38291_at	0.10474	1.272741	0.111701	-0.4106	0.62008	TRUE	1.00E+00
41219_at	0.10477	1.272829	0.15686	-0.61892	0.82845	TRUE	1.00E+00
31397_at	0.10478	1.272858	0.10828	-0.39477	0.60434	TRUE	1.00E+00
40814_at	0.10482	1.272975	0.047454	-0.11411	0.32375	TRUE	1.00E+00
37615_at	0.10484	1.273034	0.051756	-0.13394	0.34362	TRUE	1.00E+00
35409_r_at	0.10491	1.273239	0.034584	-0.05464	0.26447	TRUE	1.00E+00
804_s_at	0.10495	1.273356	0.073575	-0.2345	0.44439	TRUE	1.00E+00
32457_f_at	0.10497	1.273415	0.045934	-0.10695	0.31689	TRUE	1.00E+00
41339_at	0.10501	1.273532	0.042733	-0.09215	0.30216	TRUE	1.00E+00
41209_at	0.10516	1.273972	0.031419	-0.03979	0.25011	TRUE	1.00E+00
1791_s_at	0.10517	1.274002	0.114476	-0.42298	0.63331	TRUE	1.00E+00
35582_at	0.1052	1.27409	0.065613	-0.19751	0.40791	TRUE	1.00E+00
40908_r_at	0.10523	1.274178	0.041205	-0.08487	0.29533	TRUE	1.00E+00
41588_at	0.10525	1.274236	0.027553	-0.02187	0.23237	TRUE	1.00E+00
37148_at	0.10526	1.274266	0.058244	-0.16345	0.37398	TRUE	1.00E+00
34020_at	0.10527	1.274295	0.033423	-0.04893	0.25947	TRUE	1.00E+00
31970_r_at	0.10528	1.274324	0.126014	-0.4761	0.68665	TRUE	1.00E+00
35213_at	0.10532	1.274442	0.015913	0.0319	0.17874	FALSE	4.58E-07

34701_at	0.10535	1.27453	0.102139	-0.36587	0.57658	TRUE	1.00E+00
41639_at	0.10544	1.274794	0.041959	-0.08814	0.29902	TRUE	1.00E+00
36047_at	0.10545	1.274823	0.040531	-0.08154	0.29244	TRUE	1.00E+00
1032_at	0.10554	1.275088	0.03963	-0.0773	0.28838	TRUE	1.00E+00
39969_at	0.10554	1.275088	0.058218	-0.16305	0.37413	TRUE	1.00E+00
1805_g_at	0.10557	1.275176	0.097011	-0.342	0.55314	TRUE	1.00E+00
32433_at	0.10558	1.275205	0.014327	0.03948	0.17167	FALSE	2.17E-09
35167_at	0.10564	1.275381	0.032907	-0.04618	0.25746	TRUE	1.00E+00
36914_at	0.10576	1.275734	0.041437	-0.08541	0.29694	TRUE	1.00E+00
32791_at	0.10577	1.275763	0.0818	-0.27162	0.48317	TRUE	1.00E+00
1472_g_at	0.10585	1.275998	0.127283	-0.48138	0.69308	TRUE	1.00E+00
40035_at	0.10586	1.276027	0.031197	-0.03806	0.24979	TRUE	1.00E+00
39414_at	0.10591	1.276174	0.060676	-0.17402	0.38584	TRUE	1.00E+00
37941_at	0.10593	1.276233	0.073897	-0.235	0.44686	TRUE	1.00E+00
35495_at	0.10593	1.276233	0.055295	-0.14918	0.36104	TRUE	1.00E+00
35886_at	0.10595	1.276292	0.057765	-0.16055	0.37246	TRUE	1.00E+00
38920_at	0.10602	1.276498	0.059497	-0.16847	0.38052	TRUE	1.00E+00
39299_at	0.10605	1.276586	0.023751	-0.00352	0.21563	TRUE	1.01E-01
35470_at	0.10608	1.276674	0.0921	-0.31883	0.53099	TRUE	1.00E+00
34451_at	0.10616	1.276909	0.107677	-0.39062	0.60293	TRUE	1.00E+00
33303_at	0.10617	1.276939	0.024807	-0.00827	0.22062	TRUE	2.36E-01
39393_r_at	0.10626	1.277203	0.116097	-0.42936	0.64188	TRUE	1.00E+00
34292_at	0.10626	1.277203	0.045983	-0.10588	0.31841	TRUE	1.00E+00
37371_at	0.1063	1.277321	0.069778	-0.21562	0.42823	TRUE	1.00E+00
36402_at	0.10631	1.27735	0.058567	-0.1639	0.37651	TRUE	1.00E+00
32581_at	0.10635	1.277468	0.049794	-0.12338	0.33608	TRUE	1.00E+00
39163_at	0.10645	1.277762	0.039742	-0.0769	0.2898	TRUE	1.00E+00
41057_at	0.10647	1.277821	0.030169	-0.03272	0.24566	TRUE	1.00E+00
1688_s_at	0.10648	1.27785	0.034378	-0.05212	0.26509	TRUE	1.00E+00
34506_at	0.1065	1.277909	0.021984	0.00508	0.20793	FALSE	1.60E-02
39034_at	0.10653	1.277997	0.017996	0.0235	0.18955	FALSE	4.08E-05
33314_at	0.10655	1.278056	0.02868	-0.02577	0.23886	TRUE	1.00E+00
431_at	0.10659	1.278174	0.089936	-0.30834	0.52151	TRUE	1.00E+00
39523_at	0.10662	1.278262	0.045905	-0.10517	0.31841	TRUE	1.00E+00
36882_at	0.10663	1.278292	0.044033	-0.09652	0.30978	TRUE	1.00E+00
36881_at	0.10665	1.278351	0.06324	-0.18511	0.39841	TRUE	1.00E+00
38194_s_at	0.10665	1.278351	0.057886	-0.16041	0.37371	TRUE	1.00E+00
40726_at	0.10666	1.27838	0.115853	-0.42784	0.64116	TRUE	1.00E+00
38016_at	0.1067	1.278498	0.014215	0.04112	0.17228	FALSE	7.69E-10
38127_at	0.10677	1.278704	0.010348	0.05903	0.15451	FALSE	7.35E-21
306_s_at	0.1068	1.278792	0.028323	-0.02387	0.23747	TRUE	1.00E+00
40556_at	0.10683	1.278881	0.02306	0.00044	0.21322	FALSE	4.56E-02
39295_s_at	0.10685	1.278939	0.053309	-0.1391	0.3528	TRUE	1.00E+00
39211_at	0.10688	1.279028	0.026401	-0.01493	0.22868	TRUE	6.52E-01
35232_f_at	0.10693	1.279175	0.017968	0.02403	0.18983	FALSE	3.36E-05
276_at	0.10696	1.279263	0.008604	0.06726	0.14665	FALSE	2.24E-31
32842_at	0.10702	1.27944	0.046278	-0.10648	0.32053	TRUE	1.00E+00
38068_at	0.10702	1.27944	0.044783	-0.09959	0.31363	TRUE	1.00E+00
37324_at	0.10717	1.279882	0.050109	-0.12401	0.33835	TRUE	1.00E+00
41810_at	0.10738	1.280501	0.055524	-0.14879	0.36354	TRUE	1.00E+00
39999_at	0.10749	1.280826	0.047138	-0.10998	0.32497	TRUE	1.00E+00

32359_at	0.10751	1.280885	0.035439	-0.05599	0.27102	TRUE	1.00E+00
31707_at	0.10774	1.281563	0.106497	-0.3836	0.59907	TRUE	1.00E+00
39987_at	0.10793	1.282124	0.043548	-0.09298	0.30884	TRUE	1.00E+00
33169_at	0.10795	1.282183	0.034617	-0.05176	0.26766	TRUE	1.00E+00
1095_s_at	0.10796	1.282212	0.172221	-0.6866	0.90252	TRUE	1.00E+00
37605_at	0.10806	1.282508	0.078307	-0.25321	0.46934	TRUE	1.00E+00
39590_at	0.10818	1.282862	0.102432	-0.3644	0.58076	TRUE	1.00E+00
37792_s_at	0.10821	1.282951	0.020482	0.01371	0.2027	FALSE	1.60E-03
37199_at	0.10821	1.282951	0.041955	-0.08536	0.30177	TRUE	1.00E+00
38431_at	0.10828	1.283158	0.061451	-0.17523	0.39179	TRUE	1.00E+00
40988_at	0.10834	1.283335	0.015834	0.03528	0.18139	FALSE	9.86E-08
40461_at	0.1084	1.283512	0.030374	-0.03173	0.24853	TRUE	1.00E+00
34819_at	0.10841	1.283542	0.013027	0.04831	0.16851	FALSE	1.09E-12
36171_at	0.10843	1.283601	0.019179	0.01994	0.19692	FALSE	1.99E-04
32436_at	0.10844	1.28363	0.027123	-0.01669	0.23358	TRUE	8.06E-01
37154_at	0.10848	1.283749	0.106778	-0.38416	0.60111	TRUE	1.00E+00
32161_at	0.10854	1.283926	0.047229	-0.10935	0.32644	TRUE	1.00E+00
36830_at	0.1086	1.284103	0.026957	-0.01577	0.23297	TRUE	7.09E-01
205_g_at	0.10865	1.284251	0.131059	-0.496	0.71331	TRUE	1.00E+00
1012_at	0.10869	1.28437	0.073067	-0.22841	0.44579	TRUE	1.00E+00
32578_at	0.10874	1.284517	0.041656	-0.08344	0.30093	TRUE	1.00E+00
1460_g_at	0.10876	1.284577	0.043903	-0.09379	0.31131	TRUE	1.00E+00
41043_at	0.10889	1.284961	0.056646	-0.15245	0.37023	TRUE	1.00E+00
37066_at	0.1089	1.284991	0.054527	-0.14266	0.36047	TRUE	1.00E+00
41403_at	0.10892	1.28505	0.023598	0.00005	0.21779	FALSE	4.95E-02
31853_at	0.10899	1.285257	0.019989	0.01677	0.20121	FALSE	6.27E-04
41174_at	0.10899	1.285257	0.025314	-0.00779	0.22578	TRUE	2.10E-01
34668_at	0.10899	1.285257	0.016342	0.0336	0.18439	FALSE	3.24E-07
35407_at	0.10907	1.285494	0.082879	-0.2733	0.49144	TRUE	1.00E+00
41713_at	0.10911	1.285612	0.018773	0.0225	0.19573	FALSE	7.78E-05
34454_r_at	0.10915	1.285731	0.073921	-0.23189	0.45019	TRUE	1.00E+00
2012_s_at	0.10923	1.285968	0.034072	-0.04797	0.26642	TRUE	1.00E+00
33059_at	0.10927	1.286086	0.116174	-0.42671	0.64525	TRUE	1.00E+00
1270_at	0.1093	1.286175	0.07229	-0.22422	0.44281	TRUE	1.00E+00
302_at	0.10933	1.286264	0.030149	-0.02977	0.24842	TRUE	1.00E+00
36198_at	0.10937	1.286382	0.019035	0.02154	0.19719	FALSE	1.16E-04
1496_at	0.10943	1.28656	0.035648	-0.05503	0.2739	TRUE	1.00E+00
33305_at	0.1095	1.286767	0.035235	-0.05306	0.27206	TRUE	1.00E+00
41665_at	0.10951	1.286797	0.024697	-0.00443	0.22345	TRUE	1.17E-01
41829_at	0.10952	1.286827	0.018294	0.02511	0.19392	FALSE	2.71E-05
32345_at	0.10954	1.286886	0.095856	-0.3327	0.55178	TRUE	1.00E+00
36684_at	0.10955	1.286915	0.01446	0.04284	0.17627	FALSE	4.50E-10
957_at	0.10965	1.287212	0.032798	-0.04167	0.26096	TRUE	1.00E+00
40173_at	0.10967	1.287271	0.063145	-0.18165	0.401	TRUE	1.00E+00
41746_at	0.10968	1.287301	0.021392	0.01098	0.20837	FALSE	3.72E-03
1588_at	0.10968	1.287301	0.089016	-0.301	0.52036	TRUE	1.00E+00
34923_at	0.10971	1.28739	0.025564	-0.00823	0.22765	TRUE	2.24E-01
34657_at	0.10975	1.287508	0.022839	0.00438	0.21512	FALSE	1.95E-02
631_g_at	0.10977	1.287567	0.024144	-0.00162	0.22116	TRUE	6.89E-02
39655_at	0.10985	1.287805	0.080202	-0.26017	0.47987	TRUE	1.00E+00
38922_at	0.10985	1.287805	0.117419	-0.43187	0.65157	TRUE	1.00E+00

41620_at	0.10986	1.287834	0.02397	-0.00073	0.22045	TRUE	5.78E-02
32782_r_at	0.10997	1.288161	0.075012	-0.2361	0.45604	TRUE	1.00E+00
32730_at	0.11002	1.288309	0.040451	-0.0766	0.29665	TRUE	1.00E+00
39967_at	0.11002	1.288309	0.024164	-0.00146	0.22151	TRUE	6.67E-02
36533_at	0.11013	1.288635	0.026052	-0.01006	0.23032	TRUE	2.99E-01
32618_at	0.11017	1.288754	0.033159	-0.04281	0.26315	TRUE	1.00E+00
37794_at	0.11021	1.288873	0.030939	-0.03253	0.25295	TRUE	1.00E+00
32998_at	0.11023	1.288932	0.037362	-0.06215	0.2826	TRUE	1.00E+00
33308_at	0.11024	1.288962	0.013769	0.04672	0.17377	FALSE	1.49E-11
1239_s_at	0.11044	1.289555	0.081001	-0.26326	0.48415	TRUE	1.00E+00
34834_at	0.11051	1.289763	0.039108	-0.06992	0.29093	TRUE	1.00E+00
40882_at	0.11052	1.289793	0.056277	-0.14912	0.37015	TRUE	1.00E+00
35228_at	0.11055	1.289882	0.049206	-0.11647	0.33756	TRUE	1.00E+00
40248_at	0.11062	1.29009	0.090725	-0.30795	0.52919	TRUE	1.00E+00
38141_at	0.11074	1.290446	0.022991	0.00467	0.21682	FALSE	1.84E-02
41589_at	0.11083	1.290714	0.053902	-0.13785	0.35951	TRUE	1.00E+00
34567_at	0.11086	1.290803	0.116729	-0.42768	0.6494	TRUE	1.00E+00
39345_at	0.11099	1.29119	0.021699	0.01088	0.2111	FALSE	3.96E-03
35465_at	0.11102	1.291279	0.046412	-0.10311	0.32514	TRUE	1.00E+00
34348_at	0.11103	1.291308	0.071572	-0.21917	0.44124	TRUE	1.00E+00
32965_f_at	0.11106	1.291398	0.037681	-0.06278	0.2849	TRUE	1.00E+00
38066_at	0.11116	1.291695	0.077487	-0.24634	0.46865	TRUE	1.00E+00
37338_at	0.11123	1.291903	0.015076	0.04168	0.18079	FALSE	2.02E-09
40957_at	0.11124	1.291933	0.017663	0.02975	0.19273	FALSE	3.81E-06
37772_at	0.1113	1.292112	0.055886	-0.14654	0.36913	TRUE	1.00E+00
38639_at	0.11131	1.292141	0.037542	-0.0619	0.28451	TRUE	1.00E+00
35876_s_at	0.11139	1.292379	0.040349	-0.07476	0.29755	TRUE	1.00E+00
35416_at	0.1114	1.292409	0.131895	-0.49711	0.71991	TRUE	1.00E+00
1017_at	0.11148	1.292647	0.029142	-0.02297	0.24593	TRUE	1.00E+00
35665_at	0.1115	1.292707	0.03173	-0.03489	0.25789	TRUE	1.00E+00
36871_at	0.11162	1.293064	0.058528	-0.1584	0.38165	TRUE	1.00E+00
34332_at	0.11162	1.293064	0.021446	0.01268	0.21057	FALSE	2.45E-03
38590_r_at	0.11173	1.293391	0.017018	0.03321	0.19024	FALSE	6.57E-07
106_at	0.11177	1.293511	0.018711	0.02545	0.1981	FALSE	2.93E-05
33599_at	0.11183	1.293689	0.058789	-0.1594	0.38306	TRUE	1.00E+00
36127_g_at	0.11195	1.294047	0.019894	0.02017	0.20373	FALSE	2.31E-04
35095_r_at	0.11195	1.294047	0.081986	-0.2663	0.4902	TRUE	1.00E+00
33430_at	0.11209	1.294464	0.03167	-0.03403	0.2582	TRUE	1.00E+00
41347_at	0.11209	1.294464	0.070959	-0.21529	0.43947	TRUE	1.00E+00
41501_at	0.11227	1.295001	0.063642	-0.18135	0.40589	TRUE	1.00E+00
31569_at	0.11231	1.29512	0.033649	-0.04293	0.26756	TRUE	1.00E+00
32732_at	0.11243	1.295478	0.049473	-0.11582	0.34068	TRUE	1.00E+00
1814_at	0.11245	1.295538	0.036266	-0.05487	0.27977	TRUE	1.00E+00
1836_at	0.11251	1.295717	0.032382	-0.03689	0.26191	TRUE	1.00E+00
37520_at	0.11253	1.295776	0.028848	-0.02056	0.24563	TRUE	1.00E+00
37550_at	0.11258	1.295925	0.033798	-0.04335	0.26851	TRUE	1.00E+00
38041_at	0.11259	1.295955	0.027823	-0.01578	0.24095	TRUE	6.56E-01
34518_at	0.11259	1.295955	0.051736	-0.1261	0.35127	TRUE	1.00E+00
39674_r_at	0.11268	1.296224	0.034385	-0.04596	0.27132	TRUE	1.00E+00
32053_at	0.11273	1.296373	0.06692	-0.19601	0.42147	TRUE	1.00E+00
31880_at	0.11277	1.296492	0.037645	-0.06091	0.28644	TRUE	1.00E+00

37093_at	0.11282	1.296642	0.050528	-0.12029	0.34594	TRUE	1.00E+00
1384_at	0.11287	1.296791	0.129581	-0.48496	0.71071	TRUE	1.00E+00
37242_at	0.11289	1.296851	0.022652	0.00838	0.21739	FALSE	7.88E-03
36423_at	0.11293	1.29697	0.017927	0.03022	0.19564	FALSE	3.78E-06
34423_at	0.11296	1.29706	0.124207	-0.46008	0.686	TRUE	1.00E+00
34967_at	0.11298	1.29712	0.038834	-0.06619	0.29214	TRUE	1.00E+00
35804_at	0.11302	1.297239	0.016334	0.03766	0.18838	FALSE	5.72E-08
33429_at	0.11307	1.297388	0.030129	-0.02593	0.25207	TRUE	1.00E+00
38966_at	0.1131	1.297478	0.029928	-0.02498	0.25117	TRUE	1.00E+00
33307_at	0.1131	1.297478	0.041637	-0.079	0.3052	TRUE	1.00E+00
40701_at	0.11315	1.297627	0.010382	0.06525	0.16105	FALSE	1.49E-23
34262_at	0.11322	1.297837	0.022709	0.00845	0.21799	FALSE	7.80E-03
34325_at	0.11327	1.297986	0.032927	-0.03865	0.26518	TRUE	1.00E+00
32005_at	0.11334	1.298195	0.062283	-0.17401	0.40068	TRUE	1.00E+00
41695_at	0.11337	1.298285	0.028043	-0.01601	0.24275	TRUE	6.67E-01
35929_s_at	0.11348	1.298614	0.142584	-0.54435	0.7713	TRUE	1.00E+00
34611_at	0.11351	1.298703	0.060395	-0.16512	0.39215	TRUE	1.00E+00
39740_g_at	0.11352	1.298733	0.011581	0.06009	0.16695	FALSE	1.38E-18
37700_at	0.11355	1.298823	0.021104	0.01618	0.21092	FALSE	9.38E-04
31519_f_at	0.11356	1.298853	0.028504	-0.01795	0.24507	TRUE	8.56E-01
38892_at	0.1136	1.298973	0.023619	0.00464	0.22257	FALSE	1.91E-02
40152_r_at	0.11369	1.299242	0.085459	-0.28058	0.50796	TRUE	1.00E+00
40514_at	0.11372	1.299332	0.033875	-0.04257	0.27	TRUE	1.00E+00
35354_at	0.11374	1.299391	0.074678	-0.2308	0.45827	TRUE	1.00E+00
38943_at	0.11376	1.299451	0.041467	-0.07755	0.30507	TRUE	1.00E+00
1962_at	0.11377	1.299481	0.06773	-0.19871	0.42624	TRUE	1.00E+00
39200_s_at	0.11382	1.299631	0.05322	-0.13172	0.35935	TRUE	1.00E+00
40722_at	0.11387	1.29978	0.039978	-0.07057	0.29831	TRUE	1.00E+00
37295_at	0.114	1.30017	0.035223	-0.0485	0.27651	TRUE	1.00E+00
34773_at	0.11408	1.300409	0.015551	0.04233	0.18582	FALSE	2.78E-09
1389_at	0.11409	1.300439	0.039609	-0.06865	0.29683	TRUE	1.00E+00
434_at	0.11416	1.300649	0.083106	-0.26925	0.49758	TRUE	1.00E+00
33595_r_at	0.11418	1.300709	0.085067	-0.27828	0.50665	TRUE	1.00E+00
38774_at	0.11421	1.300798	0.031681	-0.03195	0.26037	TRUE	1.00E+00
39466_s_at	0.11425	1.300918	0.050224	-0.11746	0.34596	TRUE	1.00E+00
36225_s_at	0.11428	1.301008	0.042216	-0.08049	0.30905	TRUE	1.00E+00
36472_at	0.11431	1.301098	0.02598	-0.00556	0.23417	TRUE	1.37E-01
1484_at	0.11434	1.301188	0.057542	-0.15114	0.37981	TRUE	1.00E+00
36279_at	0.11434	1.301188	0.027888	-0.01432	0.243	TRUE	5.22E-01
41322_s_at	0.11438	1.301308	0.023401	0.00641	0.22234	FALSE	1.29E-02
36388_at	0.11443	1.301458	0.092789	-0.31366	0.54252	TRUE	1.00E+00
37980_at	0.11444	1.301487	0.044495	-0.09084	0.31973	TRUE	1.00E+00
41858_at	0.1146	1.301967	0.042901	-0.08333	0.31253	TRUE	1.00E+00
38923_at	0.11464	1.302087	0.012271	0.05803	0.17125	FALSE	1.19E-16
37348_s_at	0.11468	1.302207	0.019225	0.02599	0.20338	FALSE	3.08E-05
31686_at	0.11479	1.302537	0.101669	-0.35427	0.58385	TRUE	1.00E+00
33776_at	0.11479	1.302537	0.028715	-0.01769	0.24727	TRUE	8.08E-01
41316_s_at	0.1148	1.302567	0.028677	-0.0175	0.2471	TRUE	7.89E-01
33229_at	0.11483	1.302657	0.026351	-0.00674	0.23641	TRUE	1.66E-01
38048_at	0.11507	1.303377	0.061676	-0.16948	0.39962	TRUE	1.00E+00
35938_at	0.11514	1.303587	0.038062	-0.06047	0.29074	TRUE	1.00E+00

39645_r_at	0.11519	1.303737	0.090305	-0.30144	0.53182	TRUE	1.00E+00
41092_at	0.1152	1.303767	0.085637	-0.27989	0.5103	TRUE	1.00E+00
33479_g_at	0.11522	1.303827	0.033962	-0.04147	0.27191	TRUE	1.00E+00
31851_at	0.11532	1.304127	0.017002	0.03688	0.19376	FALSE	1.49E-07
33919_at	0.11532	1.304127	0.032223	-0.03334	0.26398	TRUE	1.00E+00
468_at	0.11535	1.304217	0.065054	-0.18478	0.41548	TRUE	1.00E+00
861_g_at	0.11552	1.304728	0.024729	0.00143	0.22961	FALSE	3.77E-02
36415_at	0.11564	1.305089	0.04213	-0.07873	0.31001	TRUE	1.00E+00
32593_at	0.11571	1.305299	0.013208	0.05477	0.17664	FALSE	2.46E-14
35914_r_at	0.11573	1.305359	0.064269	-0.18078	0.41223	TRUE	1.00E+00
144_at	0.11574	1.305389	0.02168	0.01572	0.21576	FALSE	1.18E-03
39174_at	0.11577	1.305479	0.014995	0.04659	0.18495	FALSE	1.46E-10
2000_at	0.1158	1.30557	0.027916	-0.013	0.24459	TRUE	4.23E-01
38148_at	0.1158	1.30557	0.020966	0.01907	0.21253	FALSE	4.20E-04
38816_at	0.11582	1.30563	0.037907	-0.05906	0.29071	TRUE	1.00E+00
37445_at	0.11589	1.30584	0.020966	0.01917	0.21262	FALSE	4.09E-04
637_at	0.11599	1.306141	0.09939	-0.34255	0.57454	TRUE	1.00E+00
31487_at	0.11604	1.306291	0.056065	-0.14263	0.3747	TRUE	1.00E+00
37820_at	0.11618	1.306712	0.057127	-0.14738	0.37974	TRUE	1.00E+00
39275_at	0.11628	1.307013	0.037964	-0.05887	0.29143	TRUE	1.00E+00
582_g_at	0.1163	1.307073	0.01506	0.04682	0.18578	FALSE	1.44E-10
34355_at	0.11631	1.307104	0.036996	-0.05437	0.287	TRUE	1.00E+00
37427_at	0.11637	1.307284	0.12798	-0.47408	0.70681	TRUE	1.00E+00
38782_at	0.11645	1.307525	0.024537	0.00325	0.22965	FALSE	2.62E-02
41638_at	0.11646	1.307555	0.024592	0.003	0.22991	FALSE	2.76E-02
37030_at	0.11648	1.307615	0.025915	-0.00308	0.23604	TRUE	8.80E-02
1739_at	0.11664	1.308097	0.12867	-0.477	0.71027	TRUE	1.00E+00
34062_at	0.11669	1.308248	0.035464	-0.04693	0.28031	TRUE	1.00E+00
32173_at	0.11677	1.308489	0.017317	0.03688	0.19666	FALSE	1.96E-07
32327_at	0.11679	1.308549	0.032415	-0.03276	0.26634	TRUE	1.00E+00
39587_at	0.11697	1.309091	0.069385	-0.20314	0.43709	TRUE	1.00E+00
31448_s_at	0.117	1.309182	0.067368	-0.19381	0.42781	TRUE	1.00E+00
40072_at	0.11702	1.309242	0.034497	-0.04214	0.27617	TRUE	1.00E+00
35572_f_at	0.11711	1.309514	0.027672	-0.01056	0.24477	TRUE	2.92E-01
40778_at	0.11718	1.309725	0.023155	0.01035	0.224	FALSE	5.28E-03
34381_at	0.11719	1.309755	0.016272	0.04212	0.19226	FALSE	7.48E-09
40620_at	0.11732	1.310147	0.068184	-0.19725	0.4319	TRUE	1.00E+00
41067_at	0.11732	1.310147	0.03726	-0.05458	0.28923	TRUE	1.00E+00
37776_at	0.11736	1.310268	0.10098	-0.34853	0.58324	TRUE	1.00E+00
33353_at	0.11741	1.310418	0.057999	-0.15017	0.385	TRUE	1.00E+00
39658_at	0.11744	1.310509	0.026877	-0.00656	0.24144	TRUE	1.57E-01
35528_at	0.11754	1.310811	0.084752	-0.27347	0.50855	TRUE	1.00E+00
40607_at	0.11757	1.310901	0.034063	-0.03958	0.27472	TRUE	1.00E+00
38468_at	0.1176	1.310992	0.011885	0.06276	0.17243	FALSE	5.55E-19
1800_g_at	0.11765	1.311143	0.091914	-0.3064	0.5417	TRUE	1.00E+00
37225_at	0.11792	1.311958	0.040321	-0.06811	0.30394	TRUE	1.00E+00
40967_at	0.11799	1.31217	0.055199	-0.13668	0.37265	TRUE	1.00E+00
39563_at	0.11799	1.31217	0.011822	0.06345	0.17253	FALSE	2.34E-19
41177_at	0.11803	1.312291	0.039112	-0.06242	0.29848	TRUE	1.00E+00
37683_at	0.11807	1.312411	0.030512	-0.0227	0.25884	TRUE	1.00E+00
38172_at	0.11808	1.312442	0.061013	-0.16341	0.39957	TRUE	1.00E+00

37002_at	0.11811	1.312532	0.035372	-0.04508	0.2813	TRUE	1.00E+00
40504_at	0.11819	1.312774	0.018685	0.03198	0.20439	FALSE	3.20E-06
40510_at	0.11822	1.312865	0.046038	-0.09419	0.33062	TRUE	1.00E+00
36098_at	0.11822	1.312865	0.012908	0.05867	0.17777	FALSE	6.62E-16
35487_at	0.11825	1.312955	0.055377	-0.13723	0.37374	TRUE	1.00E+00
38881_i_at	0.11838	1.313349	0.019305	0.02932	0.20745	FALSE	1.09E-05
34366_g_at	0.11848	1.313651	0.02559	0.00042	0.23655	FALSE	4.62E-02
40552_s_at	0.11856	1.313893	0.039383	-0.06314	0.30025	TRUE	1.00E+00
33209_at	0.11857	1.313923	0.039854	-0.0653	0.30244	TRUE	1.00E+00
37538_at	0.11861	1.314044	0.112578	-0.40078	0.638	TRUE	1.00E+00
39764_at	0.11861	1.314044	0.028604	-0.01336	0.25058	TRUE	4.26E-01
40393_at	0.11864	1.314135	0.05781	-0.14807	0.38535	TRUE	1.00E+00
37629_at	0.11874	1.314438	0.057816	-0.148	0.38547	TRUE	1.00E+00
38471_r_at	0.11881	1.31465	0.030611	-0.02242	0.26003	TRUE	1.00E+00
39582_at	0.11881	1.31465	0.049591	-0.10998	0.3476	TRUE	1.00E+00
32115_r_at	0.11883	1.31471	0.046142	-0.09405	0.33171	TRUE	1.00E+00
32986_s_at	0.11893	1.315013	0.061791	-0.16615	0.40401	TRUE	1.00E+00
34205_at	0.11908	1.315467	0.096221	-0.32484	0.56301	TRUE	1.00E+00
289_at	0.11916	1.315709	0.035107	-0.04281	0.28113	TRUE	1.00E+00
2003_s_at	0.11923	1.315922	0.02698	-0.00524	0.24371	TRUE	1.25E-01
34361_at	0.11924	1.315952	0.05256	-0.12325	0.36172	TRUE	1.00E+00
33408_at	0.11924	1.315952	0.027224	-0.00636	0.24484	TRUE	1.50E-01
39541_at	0.11926	1.316012	0.035807	-0.04594	0.28446	TRUE	1.00E+00
38664_at	0.11928	1.316073	0.021077	0.02204	0.21652	FALSE	1.92E-04
40143_at	0.11931	1.316164	0.041675	-0.07296	0.31159	TRUE	1.00E+00
950_at	0.1194	1.316437	0.031967	-0.02809	0.26688	TRUE	1.00E+00
38233_at	0.1194	1.316437	0.019699	0.02852	0.21028	FALSE	1.70E-05
37354_at	0.11947	1.316649	0.044242	-0.08465	0.32358	TRUE	1.00E+00
40325_at	0.11951	1.31677	0.031532	-0.02596	0.26499	TRUE	1.00E+00
34675_at	0.11952	1.316801	0.073777	-0.22086	0.4599	TRUE	1.00E+00
39829_at	0.11954	1.316861	0.049266	-0.10776	0.34683	TRUE	1.00E+00
36787_at	0.11975	1.317498	0.030779	-0.02225	0.26175	TRUE	1.00E+00
37364_at	0.11979	1.317619	0.032529	-0.03028	0.26987	TRUE	1.00E+00
39196_i_at	0.11981	1.31768	0.032474	-0.03001	0.26963	TRUE	1.00E+00
34615_at	0.12001	1.318287	0.055459	-0.13585	0.37588	TRUE	1.00E+00
1961_f_at	0.12002	1.318317	0.07221	-0.21313	0.45316	TRUE	1.00E+00
39792_at	0.12014	1.318682	0.030089	-0.01868	0.25896	TRUE	8.25E-01
32902_at	0.12018	1.318803	0.070689	-0.20596	0.44631	TRUE	1.00E+00
31787_at	0.12018	1.318803	0.043701	-0.08144	0.3218	TRUE	1.00E+00
41563_at	0.12019	1.318834	0.079716	-0.24759	0.48797	TRUE	1.00E+00
831_at	0.12021	1.318894	0.029098	-0.01403	0.25446	TRUE	4.55E-01
34097_at	0.12025	1.319016	0.074583	-0.22385	0.46434	TRUE	1.00E+00
669_s_at	0.12027	1.319077	0.055825	-0.13728	0.37783	TRUE	1.00E+00
36304_at	0.12035	1.31932	0.09666	-0.3256	0.5663	TRUE	1.00E+00
37316_r_at	0.12039	1.319441	0.030534	-0.02048	0.26126	TRUE	1.00E+00
41562_at	0.12039	1.319441	0.050151	-0.11098	0.35177	TRUE	1.00E+00
34776_at	0.1204	1.319471	0.031823	-0.02642	0.26722	TRUE	1.00E+00
35651_at	0.12044	1.319593	0.011361	0.06802	0.17285	FALSE	3.74E-22
38811_at	0.12047	1.319684	0.024018	0.00966	0.23128	FALSE	6.67E-03
37705_at	0.12048	1.319715	0.042654	-0.07631	0.31727	TRUE	1.00E+00
39323_at	0.1205	1.319775	0.010744	0.07093	0.17006	FALSE	4.32E-25

35542_at	0.1205	1.319775	0.036087	-0.046	0.28699	TRUE	1.00E+00
654_at	0.12053	1.319866	0.059251	-0.15283	0.39389	TRUE	1.00E+00
38560_at	0.12056	1.319958	0.032706	-0.03033	0.27145	TRUE	1.00E+00
1532_g_at	0.12061	1.32011	0.041944	-0.0729	0.31412	TRUE	1.00E+00
39240_at	0.12068	1.320322	0.082035	-0.25779	0.49916	TRUE	1.00E+00
41077_at	0.12069	1.320353	0.031863	-0.02632	0.26769	TRUE	1.00E+00
33270_i_at	0.12075	1.320535	0.041995	-0.073	0.3145	TRUE	1.00E+00
38454_g_at	0.12077	1.320596	0.048438	-0.1027	0.34425	TRUE	1.00E+00
40891_f_at	0.12083	1.320779	0.020139	0.02791	0.21374	FALSE	2.50E-05
35690_s_at	0.12086	1.32087	0.03886	-0.05842	0.30015	TRUE	1.00E+00
38882_r_at	0.12087	1.3209	0.018186	0.03696	0.20477	FALSE	3.80E-07
33621_at	0.12091	1.321022	0.083465	-0.26417	0.50598	TRUE	1.00E+00
40629_at	0.12096	1.321174	0.029259	-0.01403	0.25594	TRUE	4.50E-01
38129_at	0.12111	1.32163	0.022636	0.01668	0.22554	FALSE	1.11E-03
34667_at	0.12118	1.321843	0.027538	-0.00587	0.24823	TRUE	1.36E-01
38116_at	0.12124	1.322026	0.147145	-0.55763	0.8001	TRUE	1.00E+00
38678_at	0.12126	1.322087	0.023053	0.01491	0.22762	FALSE	1.82E-03
36345_g_at	0.1213	1.322209	0.043047	-0.0773	0.3199	TRUE	1.00E+00
38619_at	0.12134	1.32233	0.036706	-0.048	0.29069	TRUE	1.00E+00
33057_g_at	0.12135	1.322361	0.04278	-0.07602	0.31872	TRUE	1.00E+00
40716_at	0.12136	1.322391	0.084553	-0.26873	0.51146	TRUE	1.00E+00
40521_at	0.12142	1.322574	0.028923	-0.01202	0.25486	TRUE	3.40E-01
38200_at	0.12149	1.322787	0.038226	-0.05487	0.29785	TRUE	1.00E+00
35474_s_at	0.12157	1.323031	0.33447	-1.42154	1.66468	TRUE	1.00E+00
892_at	0.12162	1.323183	0.109206	-0.38221	0.62546	TRUE	1.00E+00
904_s_at	0.12163	1.323214	0.119744	-0.43082	0.67407	TRUE	1.00E+00
31720_s_at	0.12164	1.323244	0.060588	-0.15789	0.40116	TRUE	1.00E+00
34821_at	0.12167	1.323336	0.01148	0.06871	0.17464	FALSE	3.80E-22
32288_r_at	0.12172	1.323488	0.053902	-0.12696	0.3704	TRUE	1.00E+00
40894_at	0.12176	1.32361	0.069415	-0.19849	0.44201	TRUE	1.00E+00
40520_g_at	0.12177	1.32364	0.081843	-0.25582	0.49936	TRUE	1.00E+00
36575_at	0.12178	1.323671	0.053983	-0.12727	0.37083	TRUE	1.00E+00
39705_at	0.12189	1.324006	0.016619	0.04521	0.19856	FALSE	2.82E-09
41811_f_at	0.12199	1.324311	0.109302	-0.38229	0.62626	TRUE	1.00E+00
32597_at	0.12208	1.324586	0.015013	0.05281	0.19134	FALSE	5.35E-12
35094_f_at	0.12219	1.324921	0.108915	-0.3803	0.62467	TRUE	1.00E+00
33791_at	0.12222	1.325013	0.033908	-0.03422	0.27866	TRUE	1.00E+00
36869_at	0.12225	1.325104	0.042297	-0.0729	0.31739	TRUE	1.00E+00
41328_s_at	0.12228	1.325196	0.033262	-0.03117	0.27574	TRUE	1.00E+00
38085_at	0.12229	1.325226	0.034482	-0.03679	0.28138	TRUE	1.00E+00
218_at	0.1223	1.325257	0.011325	0.07005	0.17455	FALSE	4.39E-23
40892_s_at	0.12234	1.325379	0.067812	-0.19052	0.4352	TRUE	1.00E+00
1928_s_at	0.12234	1.325379	0.012495	0.06469	0.17999	FALSE	1.56E-18
36635_at	0.12235	1.325409	0.013691	0.05919	0.18551	FALSE	5.06E-15
33894_at	0.12238	1.325501	0.104034	-0.35759	0.60235	TRUE	1.00E+00
973_at	0.12267	1.326386	0.018129	0.03903	0.20631	FALSE	1.66E-07
392_g_at	0.12268	1.326417	0.021673	0.02269	0.22267	FALSE	1.91E-04
1468_at	0.12268	1.326417	0.023437	0.01455	0.23081	FALSE	2.09E-03
affx-bioc-3_st	0.12271	1.326508	0.071961	-0.20929	0.45471	TRUE	1.00E+00
37197_s_at	0.12285	1.326936	0.026121	0.00233	0.24336	FALSE	3.24E-02
38237_at	0.12286	1.326967	0.040098	-0.06214	0.30786	TRUE	1.00E+00

989_at	0.12288	1.327028	0.009552	0.07881	0.16695	FALSE	9.10E-34
32236_at	0.12299	1.327364	0.024852	0.00833	0.23765	FALSE	9.42E-03
36280_at	0.12302	1.327456	0.067078	-0.18645	0.43249	TRUE	1.00E+00
393_s_at	0.12304	1.327517	0.047616	-0.09664	0.34272	TRUE	1.00E+00
33138_at	0.12313	1.327792	0.070619	-0.20267	0.44894	TRUE	1.00E+00
40608_at	0.12332	1.328373	0.042392	-0.07226	0.3189	TRUE	1.00E+00
37334_at	0.12352	1.328985	0.021124	0.02607	0.22098	FALSE	6.30E-05
33907_at	0.12354	1.329046	0.011254	0.07162	0.17546	FALSE	6.15E-24
40636_at	0.12355	1.329077	0.054094	-0.12602	0.37312	TRUE	1.00E+00
478_g_at	0.12361	1.32926	0.082605	-0.25749	0.50472	TRUE	1.00E+00
40425_at	0.12365	1.329383	0.041819	-0.06929	0.31659	TRUE	1.00E+00
39857_at	0.12367	1.329444	0.065252	-0.17737	0.42472	TRUE	1.00E+00
41049_at	0.1237	1.329536	0.039423	-0.05818	0.30558	TRUE	1.00E+00
38623_at	0.1238	1.329842	0.025589	0.00574	0.24186	FALSE	1.66E-02
33192_g_at	0.12381	1.329872	0.015666	0.05154	0.19609	FALSE	3.43E-11
37025_at	0.12392	1.330209	0.036156	-0.04289	0.29073	TRUE	1.00E+00
37971_at	0.12399	1.330424	0.028961	-0.00962	0.2576	TRUE	2.35E-01
35765_at	0.12404	1.330577	0.088828	-0.28577	0.53386	TRUE	1.00E+00
35442_at	0.12411	1.330791	0.038677	-0.05433	0.30255	TRUE	1.00E+00
1619_g_at	0.12415	1.330914	0.039418	-0.05771	0.30601	TRUE	1.00E+00
40439_at	0.1242	1.331067	0.013819	0.06044	0.18795	FALSE	3.19E-15
37638_at	0.12421	1.331098	0.028555	-0.00753	0.25596	TRUE	1.72E-01
35633_at	0.12432	1.331435	0.028471	-0.00704	0.25567	TRUE	1.60E-01
2078_s_at	0.12434	1.331496	0.054531	-0.12724	0.37593	TRUE	1.00E+00
32510_at	0.12442	1.331742	0.022082	0.02254	0.2263	FALSE	2.22E-04
37341_at	0.12443	1.331772	0.02444	0.01168	0.23719	FALSE	4.49E-03
35026_f_at	0.12445	1.331834	0.05195	-0.11522	0.36413	TRUE	1.00E+00
36674_at	0.12446	1.331864	0.018845	0.03752	0.2114	FALSE	5.03E-07
1002_f_at	0.12447	1.331895	0.039571	-0.05809	0.30703	TRUE	1.00E+00
33103_s_at	0.12455	1.33214	0.02587	0.00519	0.2439	FALSE	1.86E-02
40951_at	0.12468	1.332539	0.058345	-0.1445	0.39386	TRUE	1.00E+00
35441_at	0.12469	1.33257	0.0303	-0.0151	0.26448	TRUE	4.88E-01
39677_at	0.1247	1.332601	0.071136	-0.2035	0.45289	TRUE	1.00E+00
38754_at	0.12473	1.332693	0.028976	-0.00895	0.25842	TRUE	2.11E-01
35974_at	0.12489	1.333184	0.099088	-0.33227	0.58204	TRUE	1.00E+00
32654_g_at	0.12501	1.333552	0.017687	0.04341	0.20662	FALSE	1.98E-08
38368_at	0.1251	1.333829	0.045449	-0.08458	0.33479	TRUE	1.00E+00
37065_f_at	0.12513	1.333921	0.092055	-0.29958	0.54983	TRUE	1.00E+00
40677_at	0.12519	1.334105	0.036243	-0.04201	0.2924	TRUE	1.00E+00
41410_at	0.12527	1.334351	0.016891	0.04735	0.2032	FALSE	1.52E-09
37548_at	0.12535	1.334597	0.101244	-0.34175	0.59244	TRUE	1.00E+00
39799_at	0.12548	1.334996	0.045016	-0.0822	0.33316	TRUE	1.00E+00
34496_at	0.12553	1.33515	0.045287	-0.08341	0.33446	TRUE	1.00E+00
37301_at	0.12557	1.335273	0.075947	-0.22482	0.47596	TRUE	1.00E+00
33164_at	0.12558	1.335304	0.036246	-0.04164	0.29281	TRUE	1.00E+00
36286_at	0.12561	1.335396	0.044991	-0.08196	0.33319	TRUE	1.00E+00
34783_s_at	0.12563	1.335457	0.02661	0.00286	0.2484	FALSE	2.96E-02
39922_at	0.12565	1.335519	0.066038	-0.17902	0.43032	TRUE	1.00E+00
36662_at	0.12575	1.335826	0.060037	-0.15124	0.40274	TRUE	1.00E+00
33693_at	0.12575	1.335826	0.083631	-0.26009	0.51159	TRUE	1.00E+00
1464_at	0.1258	1.33598	0.031529	-0.01966	0.27127	TRUE	8.34E-01

35639_at	0.12583	1.336072	0.147076	-0.55272	0.80438	TRUE	1.00E+00
34602_at	0.12587	1.336195	0.053826	-0.12246	0.3742	TRUE	1.00E+00
1674_at	0.126	1.336596	0.023204	0.01895	0.23306	FALSE	7.11E-04
31341_at	0.12603	1.336688	0.051139	-0.1099	0.36196	TRUE	1.00E+00
38488_s_at	0.12608	1.336842	0.061016	-0.15542	0.40759	TRUE	1.00E+00
41369_at	0.1261	1.336903	0.076859	-0.2285	0.4807	TRUE	1.00E+00
33796_at	0.12611	1.336934	0.04866	-0.09838	0.35061	TRUE	1.00E+00
34715_at	0.12615	1.337057	0.138041	-0.51072	0.76301	TRUE	1.00E+00
36579_at	0.12616	1.337088	0.017483	0.0455	0.20682	FALSE	6.75E-09
33350_s_at	0.12622	1.337273	0.023932	0.01581	0.23663	FALSE	1.69E-03
31604_at	0.12627	1.337427	0.039688	-0.05683	0.30937	TRUE	1.00E+00
37721_at	0.12629	1.337488	0.069926	-0.19632	0.4489	TRUE	1.00E+00
31460_f_at	0.12634	1.337642	0.065335	-0.17509	0.42777	TRUE	1.00E+00
31942_at	0.12639	1.337796	0.052589	-0.11623	0.36902	TRUE	1.00E+00
1525_s_at	0.12642	1.337889	0.067374	-0.18441	0.43726	TRUE	1.00E+00
37938_at	0.12658	1.338382	0.047436	-0.09227	0.34543	TRUE	1.00E+00
41681_at	0.12665	1.338597	0.019782	0.03538	0.21792	FALSE	1.93E-06
38898_at	0.12667	1.338659	0.096164	-0.31699	0.57033	TRUE	1.00E+00
38050_at	0.1268	1.33906	0.023726	0.01734	0.23626	FALSE	1.15E-03
40279_at	0.1268	1.33906	0.030307	-0.01302	0.26663	TRUE	3.62E-01
33435_r_at	0.12685	1.339214	0.031165	-0.01693	0.27064	TRUE	5.93E-01
36504_at	0.12691	1.339399	0.015508	0.05536	0.19845	FALSE	3.49E-12
872_i_at	0.12693	1.339461	0.048494	-0.0968	0.35066	TRUE	1.00E+00
366_s_at	0.12699	1.339646	0.092005	-0.29748	0.55146	TRUE	1.00E+00
36327_at	0.127	1.339677	0.095137	-0.31193	0.56592	TRUE	1.00E+00
41531_at	0.12702	1.339738	0.085546	-0.26765	0.5217	TRUE	1.00E+00
40479_at	0.12709	1.339954	0.039382	-0.0546	0.30879	TRUE	1.00E+00
39559_at	0.12712	1.340047	0.138448	-0.51162	0.76586	TRUE	1.00E+00
34375_at	0.12724	1.340417	0.141167	-0.52405	0.77853	TRUE	1.00E+00
37532_at	0.12731	1.340633	0.025898	0.00783	0.24679	FALSE	1.12E-02
34953_i_at	0.12744	1.341035	0.061155	-0.1547	0.40959	TRUE	1.00E+00
31603_at	0.12749	1.341189	0.067693	-0.18482	0.4398	TRUE	1.00E+00
38236_at	0.1275	1.34122	0.023129	0.02079	0.2342	FALSE	4.47E-04
37668_at	0.12754	1.341343	0.038868	-0.05178	0.30686	TRUE	1.00E+00
31329_at	0.12756	1.341405	0.048564	-0.0965	0.35161	TRUE	1.00E+00
316_g_at	0.12771	1.341869	0.046678	-0.08765	0.34306	TRUE	1.00E+00
32256_r_at	0.12774	1.341961	0.043715	-0.07394	0.32943	TRUE	1.00E+00
156_s_at	0.1278	1.342147	0.098365	-0.32602	0.58161	TRUE	1.00E+00
36088_at	0.1278	1.342147	0.014992	0.05863	0.19697	FALSE	1.93E-13
31403_at	0.12784	1.34227	0.048046	-0.09382	0.3495	TRUE	1.00E+00
39387_at	0.12788	1.342394	0.038858	-0.0514	0.30715	TRUE	1.00E+00
37075_at	0.1279	1.342456	0.079025	-0.23669	0.49248	TRUE	1.00E+00
38496_at	0.1279	1.342456	0.069823	-0.19424	0.45003	TRUE	1.00E+00
32084_at	0.1279	1.342456	0.030517	-0.01289	0.2687	TRUE	3.50E-01
39876_at	0.12798	1.342703	0.029181	-0.00664	0.26261	TRUE	1.46E-01
34018_at	0.12806	1.34295	0.065517	-0.17421	0.43033	TRUE	1.00E+00
630_at	0.1281	1.343074	0.032837	-0.0234	0.2796	TRUE	1.00E+00
34307_at	0.12813	1.343167	0.037261	-0.04378	0.30004	TRUE	1.00E+00
36170_at	0.12829	1.343662	0.021498	0.02911	0.22747	FALSE	3.04E-05
32706_at	0.12852	1.344374	0.014658	0.06089	0.19614	FALSE	2.30E-14
36583_at	0.12853	1.344405	0.015236	0.05824	0.19883	FALSE	4.14E-13

35606_at	0.12859	1.34459	0.042355	-0.06681	0.324	TRUE	1.00E+00
312_s_at	0.12861	1.344652	0.042024	-0.06527	0.32249	TRUE	1.00E+00
35735_at	0.12863	1.344714	0.026519	0.00629	0.25098	FALSE	1.55E-02
34884_at	0.12864	1.344745	0.033694	-0.02681	0.28409	TRUE	1.00E+00
38360_at	0.12869	1.3449	0.079155	-0.2365	0.49388	TRUE	1.00E+00
35982_at	0.12877	1.345148	0.075519	-0.21965	0.47718	TRUE	1.00E+00
35226_at	0.12878	1.345179	0.125189	-0.44879	0.70636	TRUE	1.00E+00
33958_at	0.12881	1.345272	0.086388	-0.26975	0.52736	TRUE	1.00E+00
40893_at	0.12889	1.34552	0.021956	0.0276	0.23019	FALSE	5.49E-05
39536_at	0.12891	1.345581	0.021918	0.02779	0.23003	FALSE	5.14E-05
34998_at	0.12894	1.345674	0.013256	0.06779	0.1901	FALSE	2.91E-18
33219_at	0.12897	1.345767	0.029095	-0.00526	0.26321	TRUE	1.17E-01
856_at	0.12898	1.345798	0.069321	-0.19084	0.44879	TRUE	1.00E+00
36456_at	0.12903	1.345953	0.014764	0.06092	0.19715	FALSE	2.95E-14
41557_at	0.12911	1.346201	0.006691	0.09824	0.15998	FALSE	7.33E-79
1550_at	0.12911	1.346201	0.018366	0.04438	0.21384	FALSE	2.61E-08
32174_at	0.12911	1.346201	0.04162	-0.06291	0.32113	TRUE	1.00E+00
340_at	0.12916	1.346356	0.040881	-0.05945	0.31776	TRUE	1.00E+00
36934_at	0.12918	1.346418	0.021555	0.02973	0.22862	FALSE	2.61E-05
1050_at	0.12922	1.346542	0.171127	-0.66029	0.91873	TRUE	1.00E+00
35666_at	0.12923	1.346573	0.040406	-0.05719	0.31565	TRUE	1.00E+00
36806_at	0.12923	1.346573	0.062918	-0.16104	0.41951	TRUE	1.00E+00
41647_at	0.12928	1.346728	0.031678	-0.01687	0.27542	TRUE	5.66E-01
31791_at	0.12932	1.346852	0.056357	-0.13069	0.38932	TRUE	1.00E+00
40372_at	0.12933	1.346883	0.039971	-0.05508	0.31374	TRUE	1.00E+00
39731_at	0.12936	1.346976	0.021279	0.03118	0.22753	FALSE	1.53E-05
36414_s_at	0.12937	1.347007	0.0115	0.07631	0.18242	FALSE	2.94E-25
35799_at	0.12941	1.347132	0.035865	-0.03605	0.29487	TRUE	1.00E+00
38015_at	0.12942	1.347163	0.052992	-0.11507	0.3739	TRUE	1.00E+00
40737_at	0.12945	1.347256	0.052933	-0.11476	0.37366	TRUE	1.00E+00
1364_at	0.12949	1.34738	0.128817	-0.46482	0.7238	TRUE	1.00E+00
34492_at	0.12953	1.347504	0.028399	-0.00149	0.26055	TRUE	6.42E-02
35259_s_at	0.12959	1.34769	0.030115	-0.00935	0.26853	TRUE	2.13E-01
41762_at	0.1296	1.347721	0.067082	-0.17989	0.43909	TRUE	1.00E+00
35117_at	0.12964	1.347845	0.080678	-0.24258	0.50185	TRUE	1.00E+00
37106_at	0.12966	1.347907	0.026896	0.00557	0.25375	FALSE	1.81E-02
36476_at	0.12978	1.34828	0.032501	-0.02017	0.27973	TRUE	8.24E-01
36525_at	0.12987	1.348559	0.027053	0.00506	0.25468	FALSE	2.00E-02
31832_at	0.12988	1.34859	0.041298	-0.06065	0.32041	TRUE	1.00E+00
39425_at	0.12988	1.34859	0.026964	0.00548	0.25428	FALSE	1.84E-02
39597_at	0.13001	1.348994	0.024163	0.01853	0.24149	FALSE	9.38E-04
37821_at	0.1301	1.349274	0.081713	-0.24689	0.50709	TRUE	1.00E+00
40727_at	0.13011	1.349305	0.034514	-0.02913	0.28934	TRUE	1.00E+00
33644_at	0.13022	1.349646	0.061188	-0.15207	0.41252	TRUE	1.00E+00
40128_at	0.13025	1.34974	0.025874	0.01088	0.24963	FALSE	6.06E-03
31783_at	0.1303	1.349895	0.080579	-0.24146	0.50206	TRUE	1.00E+00
31411_at	0.13032	1.349957	0.09645	-0.31467	0.5753	TRUE	1.00E+00
443_at	0.13034	1.350019	0.10402	-0.34956	0.61025	TRUE	1.00E+00
32411_at	0.13035	1.35005	0.045079	-0.07763	0.33832	TRUE	1.00E+00
33428_s_at	0.13036	1.350082	0.038846	-0.04885	0.30958	TRUE	1.00E+00
31909_at	0.13044	1.35033	0.037705	-0.04352	0.30439	TRUE	1.00E+00

1833_at	0.13052	1.350579	0.05713	-0.13305	0.3941	TRUE	1.00E+00
32705_at	0.13058	1.350766	0.059038	-0.14179	0.40296	TRUE	1.00E+00
35664_at	0.13061	1.350859	0.094979	-0.30759	0.5688	TRUE	1.00E+00
40210_at	0.13074	1.351263	0.012531	0.07293	0.18856	FALSE	2.20E-21
40710_at	0.13076	1.351326	0.069054	-0.18782	0.44935	TRUE	1.00E+00
1024_at	0.13078	1.351388	0.116366	-0.40609	0.66765	TRUE	1.00E+00
37052_at	0.13082	1.351512	0.071098	-0.1972	0.45884	TRUE	1.00E+00
41830_at	0.13096	1.351948	0.019183	0.04246	0.21947	FALSE	1.09E-07
2045_s_at	0.131	1.352073	0.043422	-0.06933	0.33133	TRUE	1.00E+00
37929_at	0.13106	1.352259	0.111362	-0.38272	0.64484	TRUE	1.00E+00
35334_at	0.1311	1.352384	0.069115	-0.18777	0.44997	TRUE	1.00E+00
1834_at	0.13118	1.352633	0.063454	-0.16158	0.42393	TRUE	1.00E+00
37778_at	0.13132	1.353069	0.036797	-0.03845	0.30108	TRUE	1.00E+00
37964_at	0.13135	1.353163	0.035497	-0.03241	0.29512	TRUE	1.00E+00
37565_at	0.13137	1.353225	0.062744	-0.15811	0.42085	TRUE	1.00E+00
178_f_at	0.13149	1.353599	0.070024	-0.19157	0.45455	TRUE	1.00E+00
41712_at	0.13163	1.354035	0.026652	0.00867	0.25459	FALSE	9.93E-03
2084_s_at	0.13173	1.354347	0.078177	-0.22895	0.49241	TRUE	1.00E+00
39154_at	0.13175	1.35441	0.068551	-0.18452	0.44802	TRUE	1.00E+00
35732_at	0.13186	1.354753	0.00571	0.10552	0.15821	FALSE	7.02E-114
32079_at	0.13196	1.355065	0.060798	-0.14854	0.41245	TRUE	1.00E+00
37143_s_at	0.13196	1.355065	0.076387	-0.22046	0.48438	TRUE	1.00E+00
41259_at	0.13196	1.355065	0.05962	-0.1431	0.40702	TRUE	1.00E+00
39743_at	0.13197	1.355096	0.028302	0.0014	0.26255	FALSE	3.93E-02
31785_f_at	0.132	1.355189	0.065204	-0.16883	0.43282	TRUE	1.00E+00
31578_at	0.13207	1.355408	0.086634	-0.26762	0.53177	TRUE	1.00E+00
32297_s_at	0.13214	1.355626	0.075417	-0.2158	0.48008	TRUE	1.00E+00
652_g_at	0.13215	1.355658	0.02519	0.01594	0.24837	FALSE	1.96E-03
2025_s_at	0.13233	1.35622	0.019912	0.04046	0.22419	FALSE	3.81E-07
33312_at	0.13235	1.356282	0.080343	-0.23832	0.50301	TRUE	1.00E+00
41611_at	0.13238	1.356376	0.025905	0.01287	0.2519	FALSE	4.06E-03
32413_at	0.13251	1.356782	0.066676	-0.1751	0.44013	TRUE	1.00E+00
41257_at	0.13266	1.35725	0.020345	0.0388	0.22653	FALSE	8.83E-07
41573_at	0.13269	1.357344	0.033417	-0.02148	0.28686	TRUE	9.05E-01
33136_at	0.13275	1.357532	0.033298	-0.02087	0.28637	TRUE	8.46E-01
1601_s_at	0.13282	1.357751	0.145294	-0.53751	0.80315	TRUE	1.00E+00
39390_at	0.13296	1.358188	0.025449	0.01555	0.25038	FALSE	2.20E-03
37284_at	0.13305	1.35847	0.058497	-0.13683	0.40293	TRUE	1.00E+00
37761_at	0.13307	1.358532	0.03684	-0.03689	0.30304	TRUE	1.00E+00
34459_at	0.13312	1.358689	0.052839	-0.11066	0.37689	TRUE	1.00E+00
36323_at	0.13323	1.359033	0.069018	-0.18519	0.45165	TRUE	1.00E+00
32740_at	0.13323	1.359033	0.023183	0.02627	0.24018	FALSE	1.15E-04
38212_at	0.13343	1.359659	0.029164	-0.00112	0.26798	TRUE	6.01E-02
40568_at	0.13343	1.359659	0.023864	0.02333	0.24353	FALSE	2.85E-04
38794_at	0.13351	1.359909	0.029863	-0.00426	0.27129	TRUE	9.84E-02
36570_at	0.13358	1.360129	0.095095	-0.30515	0.57231	TRUE	1.00E+00
38367_s_at	0.13363	1.360285	0.104174	-0.34698	0.61425	TRUE	1.00E+00
36075_at	0.13373	1.360599	0.09077	-0.28505	0.5525	TRUE	1.00E+00
484_at	0.13374	1.36063	0.041476	-0.05762	0.32509	TRUE	1.00E+00
1097_s_at	0.13374	1.36063	0.105755	-0.35417	0.62165	TRUE	1.00E+00
36520_at	0.1338	1.360818	0.059423	-0.14035	0.40795	TRUE	1.00E+00

36441_at	0.13381	1.360849	0.057757	-0.13265	0.40028	TRUE	1.00E+00
36065_at	0.13386	1.361006	0.048169	-0.08838	0.35609	TRUE	1.00E+00
31429_at	0.13387	1.361037	0.097724	-0.31699	0.58473	TRUE	1.00E+00
36448_at	0.13394	1.361257	0.027496	0.00708	0.26079	FALSE	1.40E-02
34364_at	0.13397	1.361351	0.052762	-0.10946	0.37739	TRUE	1.00E+00
34054_at	0.13399	1.361413	0.084653	-0.25656	0.52454	TRUE	1.00E+00
41581_at	0.13405	1.361601	0.081129	-0.24025	0.50834	TRUE	1.00E+00
37797_at	0.13409	1.361727	0.020473	0.03963	0.22855	FALSE	7.29E-07
41348_at	0.13416	1.361946	0.152405	-0.56898	0.83729	TRUE	1.00E+00
36521_at	0.13418	1.362009	0.020988	0.03735	0.23101	FALSE	2.05E-06
40021_at	0.1343	1.362385	0.028695	0.00191	0.26669	FALSE	3.62E-02
35290_at	0.13432	1.362448	0.022681	0.02968	0.23896	FALSE	4.01E-05
31491_s_at	0.13434	1.362511	0.131651	-0.47304	0.74173	TRUE	1.00E+00
1007_s_at	0.13435	1.362542	0.040279	-0.05148	0.32018	TRUE	1.00E+00
36253_at	0.13437	1.362605	0.077285	-0.22219	0.49094	TRUE	1.00E+00
41243_at	0.1344	1.362699	0.033139	-0.01849	0.28729	TRUE	6.31E-01
38732_at	0.13449	1.362982	0.041901	-0.05882	0.32781	TRUE	1.00E+00
35688_g_at	0.13455	1.36317	0.012241	0.07808	0.19102	FALSE	5.27E-24
36092_at	0.13455	1.36317	0.023157	0.02771	0.24139	FALSE	7.87E-05
35963_at	0.13457	1.363233	0.054452	-0.11665	0.38579	TRUE	1.00E+00
32678_at	0.13461	1.363358	0.080641	-0.23743	0.50665	TRUE	1.00E+00
36783_f_at	0.13469	1.363609	0.037834	-0.03986	0.30924	TRUE	1.00E+00
34508_r_at	0.13472	1.363704	0.033625	-0.02042	0.28985	TRUE	7.78E-01
36947_s_at	0.13473	1.363735	0.067195	-0.17528	0.44474	TRUE	1.00E+00
39269_at	0.13475	1.363798	0.068286	-0.1803	0.44979	TRUE	1.00E+00
38649_at	0.13495	1.364426	0.024961	0.01979	0.25011	FALSE	8.12E-04
36374_at	0.13495	1.364426	0.0739	-0.20599	0.4759	TRUE	1.00E+00
40004_at	0.13506	1.364772	0.044304	-0.06934	0.33946	TRUE	1.00E+00
32393_s_at	0.13511	1.364929	0.057155	-0.12858	0.3988	TRUE	1.00E+00
41808_at	0.13523	1.365306	0.01171	0.0812	0.18925	FALSE	9.56E-27
33351_at	0.13527	1.365432	0.014413	0.06877	0.20176	FALSE	7.92E-17
32596_at	0.13546	1.366029	0.099626	-0.32418	0.59509	TRUE	1.00E+00
37645_at	0.1355	1.366155	0.094994	-0.30276	0.57376	TRUE	1.00E+00
33872_at	0.13551	1.366187	0.064522	-0.16217	0.43319	TRUE	1.00E+00
35778_at	0.13568	1.366721	0.090518	-0.28193	0.55329	TRUE	1.00E+00
35363_at	0.13577	1.367005	0.029643	-0.00099	0.27253	TRUE	5.87E-02
36706_at	0.13603	1.367823	0.032474	-0.01379	0.28585	TRUE	3.54E-01
33543_s_at	0.1361	1.368044	0.015532	0.06444	0.20776	FALSE	2.41E-14
36543_at	0.13611	1.368075	0.069252	-0.18339	0.45562	TRUE	1.00E+00
40857_f_at	0.13616	1.368233	0.116684	-0.40218	0.67449	TRUE	1.00E+00
41384_at	0.13625	1.368516	0.054411	-0.11478	0.38728	TRUE	1.00E+00
35667_at	0.1363	1.368674	0.052053	-0.10385	0.37645	TRUE	1.00E+00
35980_at	0.13639	1.368958	0.039015	-0.04361	0.31639	TRUE	1.00E+00
33661_at	0.13642	1.369052	0.031181	-0.00743	0.28028	TRUE	1.53E-01
41190_at	0.13651	1.369336	0.041769	-0.0562	0.32921	TRUE	1.00E+00
32397_r_at	0.13652	1.369367	0.040962	-0.05246	0.3255	TRUE	1.00E+00
31552_at	0.13657	1.369525	0.103068	-0.33894	0.61208	TRUE	1.00E+00
37895_at	0.13661	1.369651	0.038147	-0.03939	0.3126	TRUE	1.00E+00
36140_at	0.13671	1.369967	0.154742	-0.57721	0.85062	TRUE	1.00E+00
40868_at	0.1369	1.370566	0.027302	0.01093	0.26286	FALSE	6.73E-03
38482_at	0.13691	1.370598	0.063444	-0.15579	0.42962	TRUE	1.00E+00

41370_at	0.13696	1.370756	0.021455	0.03797	0.23594	FALSE	2.19E-06
33019_at	0.13696	1.370756	0.112612	-0.38258	0.6565	TRUE	1.00E+00
39507_at	0.13699	1.37085	0.026822	0.01324	0.26073	FALSE	4.13E-03
208_at	0.13701	1.370913	0.087397	-0.26621	0.54022	TRUE	1.00E+00
38402_at	0.13703	1.370976	0.018213	0.053	0.22105	FALSE	6.72E-10
34249_at	0.13703	1.370976	0.040677	-0.05064	0.3247	TRUE	1.00E+00
1990_g_at	0.13709	1.371166	0.066359	-0.16906	0.44324	TRUE	1.00E+00
34983_at	0.1371	1.371197	0.072223	-0.19611	0.47031	TRUE	1.00E+00
32083_at	0.13712	1.371261	0.048697	-0.08755	0.36179	TRUE	1.00E+00
34961_at	0.13714	1.371324	0.099458	-0.32172	0.596	TRUE	1.00E+00
41341_at	0.13719	1.371482	0.116978	-0.4025	0.67687	TRUE	1.00E+00
31937_at	0.13723	1.371608	0.07253	-0.1974	0.47185	TRUE	1.00E+00
1182_at	0.13737	1.37205	0.068688	-0.17953	0.45426	TRUE	1.00E+00
37707_i_at	0.13752	1.372524	0.013463	0.07541	0.19963	FALSE	2.14E-20
31654_at	0.13759	1.372745	0.082164	-0.24149	0.51666	TRUE	1.00E+00
32427_at	0.13765	1.372935	0.04846	-0.08592	0.36123	TRUE	1.00E+00
34763_at	0.13774	1.37322	0.024772	0.02345	0.25202	FALSE	3.40E-04
37493_at	0.13781	1.373441	0.04956	-0.09084	0.36647	TRUE	1.00E+00
39012_g_at	0.13787	1.373631	0.024465	0.025	0.25074	FALSE	2.21E-04
160037_at	0.13791	1.373757	0.040411	-0.04853	0.32435	TRUE	1.00E+00
1854_at	0.13801	1.374074	0.082733	-0.24368	0.51971	TRUE	1.00E+00
38170_at	0.13803	1.374137	0.040991	-0.05109	0.32715	TRUE	1.00E+00
35833_at	0.13815	1.374517	0.055738	-0.119	0.3953	TRUE	1.00E+00
32653_at	0.13822	1.374738	0.066507	-0.16862	0.44505	TRUE	1.00E+00
36482_s_at	0.13826	1.374865	0.031121	-0.00532	0.28184	TRUE	1.12E-01
1811_at	0.13827	1.374896	0.047283	-0.07987	0.35642	TRUE	1.00E+00
36316_r_at	0.13829	1.37496	0.103523	-0.33932	0.61591	TRUE	1.00E+00
40205_g_at	0.1383	1.374991	0.054219	-0.11185	0.38844	TRUE	1.00E+00
33851_at	0.1383	1.374991	0.099519	-0.32084	0.59744	TRUE	1.00E+00
2094_s_at	0.13839	1.375276	0.08973	-0.27559	0.55237	TRUE	1.00E+00
41272_s_at	0.1385	1.375625	0.033116	-0.01429	0.29128	TRUE	3.64E-01
34034_at	0.13856	1.375815	0.103506	-0.33898	0.6161	TRUE	1.00E+00
33089_s_at	0.13861	1.375973	0.063229	-0.15311	0.43032	TRUE	1.00E+00
40586_at	0.13864	1.376068	0.032601	-0.01177	0.28905	TRUE	2.67E-01
32789_at	0.13868	1.376195	0.017518	0.05786	0.2195	FALSE	3.09E-11
31324_at	0.13868	1.376195	0.038617	-0.03948	0.31685	TRUE	1.00E+00
35557_at	0.1387	1.376258	0.10539	-0.34752	0.62492	TRUE	1.00E+00
33252_at	0.1387	1.376258	0.05135	-0.09821	0.37561	TRUE	1.00E+00
34799_at	0.13871	1.37629	0.035096	-0.02321	0.30063	TRUE	9.78E-01
35738_at	0.13876	1.376449	0.02756	0.01161	0.26591	FALSE	6.03E-03
37756_at	0.13879	1.376544	0.038763	-0.04004	0.31763	TRUE	1.00E+00
1643_g_at	0.13879	1.376544	0.023628	0.02978	0.24781	FALSE	5.37E-05
1713_s_at	0.1388	1.376575	0.068575	-0.17757	0.45518	TRUE	1.00E+00
41357_at	0.13882	1.376639	0.110244	-0.3698	0.64744	TRUE	1.00E+00
38345_at	0.13884	1.376702	0.175394	-0.67035	0.94804	TRUE	1.00E+00
38378_at	0.13888	1.376829	0.094118	-0.29534	0.5731	TRUE	1.00E+00
32121_at	0.13889	1.376861	0.064677	-0.1595	0.43728	TRUE	1.00E+00
514_at	0.1393	1.378161	0.030941	-0.00345	0.28205	TRUE	8.49E-02
1341_at	0.13943	1.378574	0.081499	-0.23657	0.51544	TRUE	1.00E+00
37349_r_at	0.13945	1.378637	0.161543	-0.60585	0.88474	TRUE	1.00E+00
33523_at	0.13964	1.37924	0.046736	-0.07598	0.35526	TRUE	1.00E+00

32781_f_at	0.1397	1.379431	0.027143	0.01448	0.26493	FALSE	3.34E-03
35298_at	0.13995	1.380225	0.012374	0.08286	0.19704	FALSE	1.49E-25
605_at	0.13996	1.380257	0.019807	0.04858	0.23135	FALSE	2.01E-08
40305_r_at	0.14012	1.380766	0.02476	0.02589	0.25435	FALSE	1.92E-04
38910_at	0.14015	1.380861	0.105357	-0.34592	0.62622	TRUE	1.00E+00
38258_at	0.14046	1.381847	0.082547	-0.24037	0.5213	TRUE	1.00E+00
38668_at	0.14049	1.381943	0.039799	-0.04313	0.3241	TRUE	1.00E+00
37734_at	0.14049	1.381943	0.038588	-0.03754	0.31852	TRUE	1.00E+00
36120_at	0.14067	1.382515	0.036684	-0.02857	0.30992	TRUE	1.00E+00
1641_s_at	0.14068	1.382547	0.010371	0.09283	0.18853	FALSE	8.26E-38
32382_at	0.14069	1.382579	0.056886	-0.12176	0.40313	TRUE	1.00E+00
38539_at	0.14079	1.382898	0.111311	-0.37275	0.65434	TRUE	1.00E+00
36825_at	0.14082	1.382993	0.05382	-0.10748	0.38912	TRUE	1.00E+00
41063_g_at	0.14088	1.383184	0.018522	0.05542	0.22633	FALSE	3.58E-10
33931_at	0.14088	1.383184	0.016836	0.06321	0.21856	FALSE	7.41E-13
32719_at	0.14091	1.38328	0.03863	-0.03732	0.31913	TRUE	1.00E+00
39600_at	0.14092	1.383312	0.038582	-0.03708	0.31892	TRUE	1.00E+00
37202_at	0.14097	1.383471	0.066948	-0.1679	0.44984	TRUE	1.00E+00
39742_at	0.14098	1.383503	0.026204	0.02009	0.26188	FALSE	9.39E-04
35834_at	0.14101	1.383598	0.056802	-0.12105	0.40307	TRUE	1.00E+00
35761_at	0.14109	1.383853	0.039734	-0.04223	0.3244	TRUE	1.00E+00
38577_at	0.14117	1.384108	0.05911	-0.13154	0.41388	TRUE	1.00E+00
37896_at	0.14133	1.384618	0.16832	-0.63522	0.91789	TRUE	1.00E+00
34876_at	0.14147	1.385065	0.027448	0.01484	0.2681	FALSE	3.22E-03
36903_at	0.14149	1.385128	0.070712	-0.18475	0.46772	TRUE	1.00E+00
899_at	0.14164	1.385607	0.038677	-0.0368	0.32008	TRUE	1.00E+00
41734_at	0.14167	1.385702	0.050934	-0.09332	0.37666	TRUE	1.00E+00
1766_g_at	0.14169	1.385766	0.08336	-0.2429	0.52628	TRUE	1.00E+00
38079_at	0.14183	1.386213	0.025647	0.02351	0.26016	FALSE	4.04E-04
37083_s_at	0.14196	1.386628	0.146423	-0.53357	0.81749	TRUE	1.00E+00
33902_at	0.14201	1.386788	0.054919	-0.11137	0.39538	TRUE	1.00E+00
735_s_at	0.14213	1.387171	0.053187	-0.10325	0.38752	TRUE	1.00E+00
40889_at	0.14218	1.387331	0.058108	-0.12591	0.41026	TRUE	1.00E+00
41601_at	0.14219	1.387363	0.009573	0.09803	0.18636	FALSE	8.26E-46
32973_s_at	0.14228	1.38765	0.082061	-0.23631	0.52088	TRUE	1.00E+00
32817_at	0.14237	1.387938	0.038138	-0.03359	0.31832	TRUE	1.00E+00
36241_r_at	0.14242	1.388098	0.076553	-0.21076	0.4956	TRUE	1.00E+00
2079_s_at	0.14243	1.38813	0.036309	-0.02508	0.30995	TRUE	1.00E+00
32406_at	0.14247	1.388257	0.038769	-0.03639	0.32133	TRUE	1.00E+00
36112_r_at	0.14262	1.388737	0.068854	-0.17505	0.46028	TRUE	1.00E+00
32144_at	0.14268	1.388929	0.028963	0.00906	0.2763	FALSE	1.06E-02
33026_at	0.14284	1.389441	0.064591	-0.15516	0.44083	TRUE	1.00E+00
40963_at	0.14292	1.389697	0.054843	-0.1101	0.39595	TRUE	1.00E+00
39158_at	0.14293	1.389729	0.017807	0.06078	0.22508	FALSE	1.26E-11
32426_f_at	0.14302	1.390017	0.080019	-0.22615	0.5122	TRUE	1.00E+00
36887_f_at	0.14306	1.390145	0.059885	-0.13323	0.41935	TRUE	1.00E+00
34901_at	0.14314	1.390401	0.058929	-0.12873	0.41502	TRUE	1.00E+00
35462_at	0.1432	1.390593	0.054058	-0.10621	0.3926	TRUE	1.00E+00
32398_s_at	0.14328	1.390849	0.082661	-0.23808	0.52465	TRUE	1.00E+00
40112_at	0.14332	1.390977	0.027281	0.01745	0.26918	FALSE	1.89E-03
34021_at	0.14332	1.390977	0.124913	-0.43298	0.71962	TRUE	1.00E+00

381_s_at	0.14344	1.391362	0.083571	-0.24212	0.529	TRUE	1.00E+00
1103_at	0.14363	1.39197	0.076598	-0.20976	0.49702	TRUE	1.00E+00
41730_at	0.14364	1.392002	0.039458	-0.0384	0.32569	TRUE	1.00E+00
400_at	0.14371	1.392227	0.045592	-0.06664	0.35405	TRUE	1.00E+00
41119_f_at	0.14377	1.392419	0.044722	-0.06256	0.3501	TRUE	1.00E+00
37305_at	0.1438	1.392515	0.026226	0.0228	0.2648	FALSE	5.28E-04
1779_s_at	0.14387	1.39274	0.052556	-0.09861	0.38634	TRUE	1.00E+00
37609_at	0.14389	1.392804	0.021526	0.04457	0.2432	FALSE	2.93E-07
38036_at	0.14391	1.392868	0.028088	0.01432	0.27349	FALSE	3.79E-03
38540_at	0.14391	1.392868	0.030241	0.00439	0.28343	FALSE	2.46E-02
1213_at	0.14393	1.392932	0.016565	0.06751	0.22035	FALSE	4.62E-14
187_at	0.14394	1.392964	0.077196	-0.21221	0.5001	TRUE	1.00E+00
35379_at	0.14403	1.393253	0.073026	-0.19288	0.48094	TRUE	1.00E+00
32403_at	0.14413	1.393574	0.07226	-0.18925	0.47751	TRUE	1.00E+00
39923_at	0.14425	1.393959	0.049253	-0.08298	0.37148	TRUE	1.00E+00
142_at	0.14427	1.394023	0.055245	-0.11061	0.39915	TRUE	1.00E+00
35132_at	0.14428	1.394055	0.052513	-0.09799	0.38656	TRUE	1.00E+00
34882_at	0.14446	1.394633	0.039163	-0.03622	0.32514	TRUE	1.00E+00
33700_at	0.14447	1.394665	0.035682	-0.02016	0.30909	TRUE	6.50E-01
38586_at	0.14447	1.394665	0.088719	-0.26484	0.55378	TRUE	1.00E+00
37826_at	0.14454	1.39489	0.030446	0.00408	0.28501	FALSE	2.60E-02
41846_at	0.14455	1.394922	0.022646	0.04007	0.24902	FALSE	2.19E-06
37056_at	0.14461	1.395115	0.061948	-0.14119	0.43041	TRUE	1.00E+00
31463_s_at	0.14462	1.395147	0.044023	-0.05848	0.34772	TRUE	1.00E+00
40349_at	0.14471	1.395436	0.036315	-0.02283	0.31225	TRUE	8.52E-01
40978_s_at	0.14472	1.395468	0.15009	-0.54773	0.83718	TRUE	1.00E+00
34198_at	0.14475	1.395565	0.025111	0.02889	0.2606	FALSE	1.04E-04
34620_at	0.14476	1.395597	0.08329	-0.23951	0.52902	TRUE	1.00E+00
33848_r_at	0.14479	1.395693	0.023434	0.03667	0.2529	FALSE	8.17E-06
41574_at	0.14492	1.396111	0.026284	0.02365	0.26618	FALSE	4.44E-04
1942_s_at	0.14493	1.396143	0.033484	-0.00955	0.29941	TRUE	1.90E-01
33620_at	0.1451	1.39669	0.037669	-0.02869	0.31889	TRUE	1.00E+00
34713_at	0.14511	1.396722	0.028077	0.01557	0.27464	FALSE	2.98E-03
41062_at	0.14511	1.396722	0.013197	0.08423	0.20599	FALSE	5.05E-24
38287_at	0.14512	1.396754	0.028847	0.01203	0.27821	FALSE	6.17E-03
41360_at	0.14517	1.396915	0.029067	0.01107	0.27928	FALSE	7.45E-03
37251_s_at	0.14526	1.397205	0.060286	-0.13287	0.4234	TRUE	1.00E+00
32677_at	0.14528	1.397269	0.117915	-0.39873	0.68929	TRUE	1.00E+00
40310_at	0.14533	1.39743	0.1044	-0.33633	0.62699	TRUE	1.00E+00
41184_s_at	0.14533	1.39743	0.027163	0.02002	0.27065	FALSE	1.11E-03
1090_f_at	0.14541	1.397687	0.071455	-0.18425	0.47508	TRUE	1.00E+00
33801_at	0.14545	1.397816	0.073154	-0.19206	0.48295	TRUE	1.00E+00
33662_at	0.14551	1.398009	0.081097	-0.22864	0.51966	TRUE	1.00E+00
869_at	0.14572	1.398685	0.032367	-0.00361	0.29504	TRUE	8.50E-02
41336_at	0.14586	1.399136	0.057919	-0.12136	0.41307	TRUE	1.00E+00
32911_s_at	0.14587	1.399168	0.050796	-0.08848	0.38022	TRUE	1.00E+00
34728_g_at	0.14588	1.399201	0.005287	0.12149	0.17027	FALSE	1.70E-163
32454_at	0.14592	1.39933	0.04108	-0.04361	0.33544	TRUE	1.00E+00
39853_at	0.14608	1.399845	0.067276	-0.1643	0.45647	TRUE	1.00E+00
41106_at	0.14608	1.399845	0.106992	-0.34753	0.6397	TRUE	1.00E+00
32966_at	0.14609	1.399877	0.100971	-0.31975	0.61193	TRUE	1.00E+00

41242_at	0.1461	1.39991	0.046844	-0.07001	0.36222	TRUE	1.00E+00
37469_at	0.14618	1.400168	0.067003	-0.16295	0.4553	TRUE	1.00E+00
40561_at	0.14621	1.400264	0.054933	-0.10722	0.39965	TRUE	1.00E+00
32263_at	0.14652	1.401264	0.163268	-0.60673	0.89977	TRUE	1.00E+00
39218_at	0.14652	1.401264	0.053504	-0.10033	0.39337	TRUE	1.00E+00
367_at	0.14656	1.401393	0.113246	-0.37591	0.66903	TRUE	1.00E+00
38318_at	0.14668	1.40178	0.037753	-0.0275	0.32085	TRUE	1.00E+00
32624_at	0.14669	1.401813	0.025382	0.02959	0.26379	FALSE	9.47E-05
1244_at	0.14669	1.401813	0.018503	0.06133	0.23206	FALSE	2.81E-11
33168_at	0.14672	1.40191	0.028477	0.01533	0.2781	FALSE	3.25E-03
32607_at	0.14676	1.402039	0.015404	0.0757	0.21783	FALSE	2.03E-17
39446_s_at	0.14685	1.402329	0.064714	-0.15172	0.44541	TRUE	1.00E+00
32025_at	0.14685	1.402329	0.025216	0.03052	0.26319	FALSE	7.26E-05
40492_at	0.14688	1.402426	0.012583	0.08883	0.20493	FALSE	2.22E-27
31968_at	0.14709	1.403104	0.092222	-0.27839	0.57256	TRUE	1.00E+00
36858_at	0.14716	1.403331	0.02341	0.03916	0.25516	FALSE	4.11E-06
40920_at	0.14724	1.403589	0.031854	0.00027	0.2942	FALSE	4.79E-02
38084_at	0.14725	1.403621	0.039162	-0.03343	0.32792	TRUE	1.00E+00
41142_at	0.14729	1.403751	0.016822	0.06968	0.2249	FALSE	2.56E-14
40678_at	0.14729	1.403751	0.038642	-0.03099	0.32557	TRUE	1.00E+00
38157_at	0.14734	1.403912	0.083573	-0.23823	0.53291	TRUE	1.00E+00
32570_at	0.14746	1.4043	0.088784	-0.26215	0.55708	TRUE	1.00E+00
1520_s_at	0.14751	1.404462	0.070479	-0.17765	0.47267	TRUE	1.00E+00
39014_at	0.14751	1.404462	0.054937	-0.10594	0.40097	TRUE	1.00E+00
33363_at	0.14767	1.40498	0.021475	0.04859	0.24674	FALSE	7.76E-08
31557_at	0.14768	1.405012	0.097292	-0.30119	0.59654	TRUE	1.00E+00
37021_at	0.14779	1.405368	0.047763	-0.07257	0.36815	TRUE	1.00E+00
36697_g_at	0.14787	1.405627	0.05826	-0.12092	0.41666	TRUE	1.00E+00
32308_r_at	0.1479	1.405724	0.050963	-0.08722	0.38302	TRUE	1.00E+00
35306_at	0.14805	1.406209	0.019565	0.05778	0.23832	FALSE	4.82E-10
41798_at	0.14807	1.406274	0.024383	0.03558	0.26057	FALSE	1.59E-05
41729_at	0.14809	1.406339	0.027199	0.0226	0.27357	FALSE	6.55E-04
35129_at	0.14817	1.406598	0.095634	-0.29304	0.58939	TRUE	1.00E+00
40344_at	0.14818	1.40663	0.078411	-0.21358	0.50993	TRUE	1.00E+00
572_at	0.1482	1.406695	0.14361	-0.51435	0.81076	TRUE	1.00E+00
36589_at	0.14831	1.407052	0.026398	0.02652	0.27011	FALSE	2.43E-04
affx-humgapdh/m	0.14832	1.407084	0.061486	-0.13535	0.43199	TRUE	1.00E+00
38571_at	0.14836	1.407214	0.040587	-0.03889	0.33561	TRUE	1.00E+00
34727_at	0.14838	1.407278	0.01999	0.05616	0.24061	FALSE	1.45E-09
35256_at	0.14846	1.407538	0.034216	-0.0094	0.30632	TRUE	1.81E-01
33040_at	0.1485	1.407667	0.106014	-0.34061	0.6376	TRUE	1.00E+00
34671_at	0.1485	1.407667	0.02867	0.01623	0.28078	FALSE	2.81E-03
1035_g_at	0.14882	1.408705	0.049829	-0.08107	0.37871	TRUE	1.00E+00
31963_at	0.14901	1.409321	0.033921	-0.00749	0.30551	TRUE	1.41E-01
33769_at	0.14908	1.409548	0.037088	-0.02203	0.32018	TRUE	7.36E-01
37906_at	0.14925	1.4101	0.061617	-0.13502	0.43352	TRUE	1.00E+00
32765_f_at	0.14926	1.410133	0.01955	0.05907	0.23946	FALSE	2.86E-10
34896_at	0.14934	1.410393	0.094926	-0.28861	0.58728	TRUE	1.00E+00
35926_s_at	0.14936	1.410457	0.117076	-0.39078	0.6895	TRUE	1.00E+00
38593_r_at	0.14951	1.410945	0.062806	-0.14025	0.43927	TRUE	1.00E+00
31432_g_at	0.14953	1.41101	0.024059	0.03853	0.26052	FALSE	6.48E-06

34208_at	0.14958	1.411172	0.122214	-0.41426	0.71343	TRUE	1.00E+00
1005_at	0.14963	1.411335	0.034459	-0.00935	0.30862	TRUE	1.78E-01
38393_at	0.14964	1.411367	0.035067	-0.01215	0.31142	TRUE	2.50E-01
32841_at	0.14964	1.411367	0.018591	0.06387	0.23541	FALSE	1.05E-11
35406_at	0.14969	1.41153	0.189834	-0.72613	1.02551	TRUE	1.00E+00
40126_at	0.14971	1.411595	0.026543	0.02725	0.27216	FALSE	2.15E-04
38165_s_at	0.14971	1.411595	0.094067	-0.28427	0.5837	TRUE	1.00E+00
38679_g_at	0.14984	1.412017	0.010285	0.10238	0.19729	FALSE	5.66E-44
37218_at	0.14985	1.41205	0.026101	0.02943	0.27027	FALSE	1.19E-04
1393_at	0.14996	1.412407	0.077909	-0.20949	0.5094	TRUE	1.00E+00
39883_at	0.14999	1.412505	0.028543	0.0183	0.28168	FALSE	1.87E-03
40832_s_at	0.15001	1.41257	0.027519	0.02305	0.27697	FALSE	6.32E-04
36627_at	0.15004	1.412668	0.111781	-0.36567	0.66576	TRUE	1.00E+00
36260_at	0.15011	1.412895	0.126913	-0.43541	0.73564	TRUE	1.00E+00
40431_at	0.15026	1.413383	0.018747	0.06377	0.23675	FALSE	1.39E-11
41720_r_at	0.15027	1.413416	0.033525	-0.0044	0.30494	TRUE	9.33E-02
37623_at	0.15027	1.413416	0.114135	-0.3763	0.67684	TRUE	1.00E+00
34435_at	0.15037	1.413741	0.045171	-0.05803	0.35877	TRUE	1.00E+00
36970_at	0.15042	1.413904	0.0366	-0.01843	0.31928	TRUE	5.00E-01
34275_s_at	0.15045	1.414002	0.061958	-0.1354	0.4363	TRUE	1.00E+00
1589_s_at	0.15089	1.415435	0.023634	0.04185	0.25993	FALSE	2.17E-06
33757_f_at	0.1509	1.415468	0.042851	-0.04679	0.3486	TRUE	1.00E+00
37994_at	0.15094	1.415598	0.025376	0.03386	0.26801	FALSE	3.42E-05
34195_at	0.151	1.415794	0.01357	0.08839	0.21361	FALSE	1.17E-24
38130_s_at	0.15111	1.416152	0.161798	-0.59536	0.89758	TRUE	1.00E+00
304_at	0.15111	1.416152	0.029932	0.01302	0.28921	FALSE	5.62E-03
34259_at	0.15129	1.416739	0.063029	-0.1395	0.44208	TRUE	1.00E+00
36584_at	0.15133	1.41687	0.140176	-0.49539	0.79804	TRUE	1.00E+00
41778_at	0.15136	1.416968	0.040365	-0.03487	0.33759	TRUE	1.00E+00
41854_at	0.15149	1.417392	0.041042	-0.03786	0.34084	TRUE	1.00E+00
36991_at	0.1515	1.417425	0.018183	0.06762	0.23539	FALSE	1.00E-12
851_s_at	0.15157	1.417653	0.04201	-0.04224	0.34539	TRUE	1.00E+00
35511_at	0.15157	1.417653	0.068417	-0.16407	0.46722	TRUE	1.00E+00
36042_at	0.15158	1.417686	0.108437	-0.34871	0.65186	TRUE	1.00E+00
36529_at	0.15164	1.417882	0.053883	-0.09695	0.40024	TRUE	1.00E+00
1989_at	0.15166	1.417947	0.157386	-0.57445	0.87778	TRUE	1.00E+00
39628_at	0.15179	1.418372	0.0134	0.08996	0.21361	FALSE	1.21E-25
37160_at	0.15197	1.41896	0.072286	-0.18153	0.48547	TRUE	1.00E+00
38867_g_at	0.15197	1.41896	0.071086	-0.17599	0.47993	TRUE	1.00E+00
1156_at	0.152	1.419058	0.029857	0.01425	0.28974	FALSE	4.50E-03
35381_at	0.15206	1.419254	0.085095	-0.24053	0.54466	TRUE	1.00E+00
35267_g_at	0.15213	1.419482	0.025947	0.03243	0.27184	FALSE	5.73E-05
34946_at	0.15224	1.419842	0.166881	-0.61768	0.92216	TRUE	1.00E+00
38919_at	0.15244	1.420496	0.041405	-0.03858	0.34347	TRUE	1.00E+00
34471_at	0.1525	1.420692	0.059487	-0.12195	0.42695	TRUE	1.00E+00
39769_at	0.15251	1.420725	0.04897	-0.07342	0.37843	TRUE	1.00E+00
32927_at	0.15259	1.420987	0.124824	-0.4233	0.72848	TRUE	1.00E+00
36359_at	0.1526	1.421019	0.081863	-0.22509	0.53028	TRUE	1.00E+00
40041_at	0.15267	1.421248	0.139055	-0.48887	0.79422	TRUE	1.00E+00
39373_at	0.15279	1.421641	0.052233	-0.08819	0.39378	TRUE	1.00E+00
33651_at	0.1528	1.421674	0.062085	-0.13364	0.43923	TRUE	1.00E+00

32085_at	0.15311	1.422689	0.016864	0.07531	0.23092	FALSE	1.38E-15
39954_r_at	0.15313	1.422755	0.030365	0.01304	0.29322	FALSE	5.79E-03
35637_at	0.15317	1.422886	0.083577	-0.23242	0.53876	TRUE	1.00E+00
1537_at	0.15321	1.423017	0.031127	0.0096	0.29681	FALSE	1.08E-02
40216_at	0.1533	1.423312	0.048173	-0.06895	0.37555	TRUE	1.00E+00
31672_g_at	0.15333	1.42341	0.031355	0.00867	0.29799	FALSE	1.27E-02
38162_at	0.15341	1.423672	0.030458	0.01289	0.29393	FALSE	5.97E-03
38655_at	0.15346	1.423836	0.023993	0.04276	0.26415	FALSE	2.02E-06
41218_at	0.15359	1.424262	0.055172	-0.10095	0.40813	TRUE	1.00E+00
40614_at	0.15361	1.424328	0.022762	0.0486	0.25862	FALSE	1.89E-07
476_s_at	0.15372	1.424689	0.016127	0.07932	0.22813	FALSE	1.94E-17
32794_g_at	0.15374	1.424754	0.101095	-0.31267	0.62015	TRUE	1.00E+00
35987_g_at	0.15374	1.424754	0.035308	-0.00916	0.31663	TRUE	1.69E-01
1629_s_at	0.15378	1.424886	0.017113	0.07483	0.23273	FALSE	3.23E-15
1533_at	0.15385	1.425115	0.050423	-0.07878	0.38648	TRUE	1.00E+00
40489_at	0.15389	1.425247	0.044521	-0.05151	0.35929	TRUE	1.00E+00
38260_at	0.15392	1.425345	0.102722	-0.31999	0.62784	TRUE	1.00E+00
34434_at	0.15392	1.425345	0.113751	-0.37088	0.67873	TRUE	1.00E+00
36159_s_at	0.15394	1.425411	0.028345	0.02317	0.28472	FALSE	7.07E-04
34031_i_at	0.15402	1.425673	0.017636	0.07266	0.23539	FALSE	3.12E-14
34950_at	0.15415	1.4261	0.096365	-0.29044	0.59874	TRUE	1.00E+00
38419_at	0.15428	1.426527	0.031248	0.01011	0.29844	FALSE	1.00E-02
32353_at	0.15431	1.426626	0.062203	-0.13267	0.44128	TRUE	1.00E+00
39828_at	0.15434	1.426724	0.073004	-0.18247	0.49115	TRUE	1.00E+00
39787_at	0.15442	1.426987	0.025616	0.03624	0.2726	FALSE	2.09E-05
33407_at	0.15443	1.42702	0.061194	-0.12789	0.43676	TRUE	1.00E+00
36643_at	0.15446	1.427118	0.022732	0.04959	0.25933	FALSE	1.37E-07
40664_at	0.15462	1.427644	0.052712	-0.08857	0.39781	TRUE	1.00E+00
1055_g_at	0.15467	1.427809	0.048808	-0.07051	0.37985	TRUE	1.00E+00
1682_s_at	0.15468	1.427841	0.130929	-0.44937	0.75873	TRUE	1.00E+00
33715_r_at	0.15472	1.427973	0.074492	-0.18895	0.49839	TRUE	1.00E+00
34286_at	0.15476	1.428105	0.019268	0.06586	0.24365	FALSE	1.22E-11
35842_at	0.15478	1.42817	0.021747	0.05445	0.25512	FALSE	1.39E-08
41188_at	0.15488	1.428499	0.009944	0.109	0.20076	FALSE	1.35E-50
34696_at	0.15495	1.428729	0.022818	0.04968	0.26023	FALSE	1.41E-07
41669_at	0.15497	1.428795	0.015414	0.08385	0.22608	FALSE	1.12E-19
34321_i_at	0.15507	1.429124	0.040788	-0.03311	0.34325	TRUE	1.00E+00
37600_at	0.1551	1.429223	0.044384	-0.04967	0.35986	TRUE	1.00E+00
35859_f_at	0.15516	1.42942	0.056432	-0.1052	0.41551	TRUE	1.00E+00
37446_at	0.15544	1.430342	0.052499	-0.08676	0.39765	TRUE	1.00E+00
36462_at	0.15547	1.430441	0.033184	0.00238	0.30857	FALSE	3.53E-02
39672_at	0.1557	1.431199	0.070082	-0.16763	0.47903	TRUE	1.00E+00
38405_at	0.15571	1.431232	0.043958	-0.04709	0.35852	TRUE	1.00E+00
34086_at	0.15584	1.43166	0.043416	-0.04446	0.35615	TRUE	1.00E+00
32827_at	0.15589	1.431825	0.02954	0.0196	0.29217	FALSE	1.66E-03
41716_at	0.15596	1.432056	0.022654	0.05144	0.26047	FALSE	7.33E-08
324_f_at	0.15602	1.432254	0.007979	0.11921	0.19283	FALSE	4.86E-81
40144_at	0.15611	1.432551	0.087409	-0.24716	0.55938	TRUE	1.00E+00
34316_at	0.15628	1.433112	0.024612	0.04273	0.26983	FALSE	2.72E-06
39634_at	0.1563	1.433178	0.060188	-0.12138	0.43399	TRUE	1.00E+00
34359_at	0.15632	1.433244	0.031729	0.00994	0.30271	FALSE	1.06E-02

38459_g_at	0.15637	1.433409	0.022975	0.05038	0.26237	FALSE	1.26E-07
680_s_at	0.15644	1.43364	0.076173	-0.19499	0.50786	TRUE	1.00E+00
31774_at	0.15661	1.434201	0.07633	-0.19555	0.50876	TRUE	1.00E+00
38931_at	0.15661	1.434201	0.027834	0.02819	0.28502	FALSE	2.32E-04
41289_at	0.15662	1.434234	0.119848	-0.39631	0.70955	TRUE	1.00E+00
37396_at	0.15695	1.435324	0.034246	-0.00105	0.31495	TRUE	5.79E-02
36899_at	0.15698	1.435423	0.045089	-0.05104	0.365	TRUE	1.00E+00
40877_s_at	0.157	1.435489	0.01791	0.07437	0.23963	FALSE	2.34E-14
36122_at	0.15727	1.436382	0.03349	0.00276	0.31178	FALSE	3.35E-02
38346_at	0.15733	1.436581	0.06131	-0.12553	0.44019	TRUE	1.00E+00
35872_at	0.15746	1.437011	0.106329	-0.33309	0.64802	TRUE	1.00E+00
41795_at	0.15749	1.43711	0.034013	0.00056	0.31441	FALSE	4.61E-02
31310_at	0.15749	1.43711	0.046072	-0.05507	0.37005	TRUE	1.00E+00
33024_at	0.15749	1.43711	0.044453	-0.0476	0.36258	TRUE	1.00E+00
34222_at	0.15749	1.43711	0.054124	-0.09221	0.4072	TRUE	1.00E+00
36961_at	0.15757	1.437375	0.029654	0.02076	0.29439	FALSE	1.36E-03
38474_at	0.15761	1.437507	0.023432	0.0495	0.26571	FALSE	2.20E-07
37901_at	0.15772	1.437871	0.014236	0.09204	0.2234	FALSE	2.00E-24
38286_at	0.15784	1.438269	0.026433	0.03589	0.27979	FALSE	2.97E-05
40367_at	0.15795	1.438633	0.108161	-0.34106	0.65695	TRUE	1.00E+00
786_at	0.15799	1.438765	0.040683	-0.0297	0.34568	TRUE	1.00E+00
38574_at	0.15835	1.439959	0.106556	-0.33326	0.64995	TRUE	1.00E+00
826_at	0.15847	1.440357	0.05129	-0.07816	0.3951	TRUE	1.00E+00
34014_f_at	0.15854	1.440589	0.100423	-0.30477	0.62185	TRUE	1.00E+00
36777_at	0.15867	1.44102	0.085261	-0.23469	0.55203	TRUE	1.00E+00
40719_at	0.15883	1.441551	0.097822	-0.29248	0.61014	TRUE	1.00E+00
38683_s_at	0.15889	1.44175	0.023883	0.04871	0.26907	FALSE	3.63E-07
32723_at	0.15894	1.441916	0.045038	-0.04885	0.36672	TRUE	1.00E+00
32369_at	0.15895	1.441949	0.039685	-0.02414	0.34204	TRUE	7.82E-01
999_at	0.15896	1.441983	0.030423	0.0186	0.29932	FALSE	2.20E-03
34428_at	0.15901	1.442149	0.03985	-0.02484	0.34286	TRUE	8.33E-01
38822_at	0.1591	1.442447	0.020912	0.06262	0.25558	FALSE	3.51E-10
35991_at	0.15913	1.442547	0.031149	0.01542	0.30284	FALSE	4.09E-03
36228_at	0.15915	1.442614	0.108275	-0.34039	0.65868	TRUE	1.00E+00
41048_at	0.15922	1.442846	0.044199	-0.0447	0.36314	TRUE	1.00E+00
36105_at	0.15924	1.442913	0.076446	-0.19345	0.51192	TRUE	1.00E+00
33503_at	0.15936	1.443311	0.064174	-0.13671	0.45544	TRUE	1.00E+00
261_s_at	0.15944	1.443577	0.140008	-0.4865	0.80538	TRUE	1.00E+00
33867_s_at	0.15947	1.443677	0.032725	0.00848	0.31045	FALSE	1.39E-02
39377_at	0.15947	1.443677	0.021533	0.06013	0.25882	FALSE	1.64E-09
33373_at	0.15966	1.444309	0.042022	-0.03421	0.35353	TRUE	1.00E+00
542_at	0.15973	1.444541	0.102904	-0.31503	0.63448	TRUE	1.00E+00
35277_at	0.15973	1.444541	0.079018	-0.20483	0.52429	TRUE	1.00E+00
41014_s_at	0.15976	1.444641	0.104663	-0.32312	0.64263	TRUE	1.00E+00
40141_at	0.15991	1.44514	0.01837	0.07516	0.24466	FALSE	4.01E-14
36605_at	0.16012	1.445839	0.029842	0.02245	0.2978	FALSE	1.02E-03
37937_at	0.16021	1.446139	0.043274	-0.03943	0.35986	TRUE	1.00E+00
37258_at	0.16027	1.446339	0.024833	0.0457	0.27484	FALSE	1.38E-06
35130_at	0.16033	1.446539	0.060425	-0.11845	0.43911	TRUE	1.00E+00
39947_at	0.16036	1.446638	0.087767	-0.24456	0.56528	TRUE	1.00E+00
1556_at	0.16047	1.447005	0.026899	0.03637	0.28458	FALSE	3.08E-05

828_at	0.16051	1.447138	0.079836	-0.20782	0.52884	TRUE	1.00E+00
40295_at	0.16062	1.447505	0.059206	-0.11254	0.43377	TRUE	1.00E+00
35782_at	0.16066	1.447638	0.036767	-0.00897	0.33029	TRUE	1.57E-01
41494_at	0.16079	1.448071	0.084356	-0.22839	0.54998	TRUE	1.00E+00
33182_at	0.16083	1.448205	0.184619	-0.69092	1.01259	TRUE	1.00E+00
39797_at	0.16104	1.448905	0.037876	-0.01371	0.33578	TRUE	2.68E-01
40400_at	0.1612	1.449439	0.07503	-0.18495	0.50736	TRUE	1.00E+00
35459_at	0.1612	1.449439	0.104041	-0.3188	0.64121	TRUE	1.00E+00
37194_at	0.16134	1.449907	0.064977	-0.13844	0.46112	TRUE	1.00E+00
40012_at	0.16146	1.450307	0.103991	-0.31831	0.64123	TRUE	1.00E+00
725_i_at	0.16146	1.450307	0.107527	-0.33463	0.65754	TRUE	1.00E+00
38823_s_at	0.16146	1.450307	0.039184	-0.01932	0.34224	TRUE	4.77E-01
41462_at	0.16146	1.450307	0.035382	-0.00177	0.3247	TRUE	6.35E-02
38248_at	0.16149	1.450407	0.029372	0.02598	0.297	FALSE	4.85E-04
37928_at	0.16169	1.451075	0.025563	0.04375	0.27963	FALSE	3.20E-06
40818_at	0.16169	1.451075	0.009849	0.11625	0.20713	FALSE	1.82E-56
38000_at	0.16171	1.451142	0.032588	0.01136	0.31205	FALSE	8.80E-03
35077_at	0.16175	1.451276	0.055067	-0.0923	0.41581	TRUE	1.00E+00
32183_at	0.16176	1.451309	0.021911	0.06067	0.26285	FALSE	1.96E-09
38189_s_at	0.1618	1.451443	0.074139	-0.18024	0.50385	TRUE	1.00E+00
34080_at	0.16187	1.451677	0.040698	-0.0259	0.34964	TRUE	8.80E-01
38353_at	0.16191	1.451811	0.027513	0.03498	0.28884	FALSE	5.03E-05
37283_at	0.16198	1.452045	0.077208	-0.19422	0.51819	TRUE	1.00E+00
419_at	0.16212	1.452513	0.089139	-0.24913	0.57337	TRUE	1.00E+00
2088_s_at	0.16213	1.452546	0.097645	-0.28837	0.61262	TRUE	1.00E+00
35931_at	0.16214	1.45258	0.13065	-0.44062	0.7649	TRUE	1.00E+00
38098_at	0.1622	1.45278	0.041399	-0.0288	0.35319	TRUE	1.00E+00
39489_g_at	0.16227	1.453015	0.117672	-0.38062	0.70516	TRUE	1.00E+00
33663_at	0.16249	1.453751	0.05884	-0.10897	0.43395	TRUE	1.00E+00
35153_at	0.16259	1.454086	0.012366	0.10554	0.21965	FALSE	2.20E-35
32850_at	0.16271	1.454488	0.019893	0.07093	0.25448	FALSE	3.61E-12
37641_at	0.16282	1.454856	0.034734	0.00258	0.32307	FALSE	3.49E-02
34251_at	0.16284	1.454923	0.09741	-0.28658	0.61225	TRUE	1.00E+00
41488_at	0.16292	1.455191	0.008391	0.12421	0.20163	FALSE	7.15E-80
37566_at	0.16303	1.45556	0.051164	-0.07302	0.39908	TRUE	1.00E+00
41408_at	0.16304	1.455593	0.015089	0.09342	0.23265	FALSE	4.10E-23
32262_at	0.16314	1.455928	0.021291	0.06491	0.26136	FALSE	2.31E-10
35449_at	0.16317	1.456029	0.116146	-0.37268	0.69902	TRUE	1.00E+00
1639_s_at	0.16319	1.456096	0.030514	0.02241	0.30397	FALSE	1.12E-03
41542_at	0.1633	1.456465	0.033111	0.01054	0.31606	FALSE	1.03E-02
35367_at	0.16349	1.457102	0.025826	0.04434	0.28264	FALSE	3.09E-06
31609_s_at	0.16353	1.457236	0.060243	-0.1144	0.44147	TRUE	1.00E+00
1354_at	0.16356	1.457337	0.041785	-0.02922	0.35634	TRUE	1.00E+00
38842_at	0.1636	1.457471	0.033885	0.00726	0.31993	FALSE	1.74E-02
41513_s_at	0.16363	1.457572	0.031958	0.01619	0.31107	FALSE	3.85E-03
37013_at	0.16363	1.457572	0.042321	-0.03162	0.35889	TRUE	1.00E+00
32237_at	0.16372	1.457874	0.021005	0.06681	0.26062	FALSE	8.18E-11
570_at	0.16387	1.458378	0.048184	-0.05843	0.38617	TRUE	1.00E+00
32651_at	0.16413	1.459251	0.066771	-0.14393	0.47218	TRUE	1.00E+00
39204_at	0.16425	1.459654	0.031124	0.02066	0.30784	FALSE	1.66E-03
32455_s_at	0.16426	1.459688	0.088345	-0.24333	0.57184	TRUE	1.00E+00

41454_at	0.16431	1.459856	0.039164	-0.01638	0.34499	TRUE	3.44E-01
1113_at	0.16436	1.460024	0.08422	-0.2242	0.55291	TRUE	1.00E+00
35814_at	0.16446	1.46036	0.030661	0.023	0.30592	FALSE	1.03E-03
34253_at	0.16449	1.460461	0.024266	0.05253	0.27644	FALSE	1.53E-07
38135_at	0.16451	1.460528	0.022465	0.06086	0.26815	FALSE	3.07E-09
33530_at	0.16457	1.46073	0.092259	-0.26107	0.59021	TRUE	1.00E+00
38146_at	0.16461	1.460865	0.080581	-0.20716	0.53637	TRUE	1.00E+00
41216_r_at	0.16462	1.460898	0.102048	-0.30619	0.63542	TRUE	1.00E+00
31868_at	0.16462	1.460898	0.067905	-0.14866	0.47791	TRUE	1.00E+00
32356_at	0.16463	1.460932	0.059065	-0.10788	0.43713	TRUE	1.00E+00
37755_at	0.16474	1.461302	0.025462	0.04727	0.28221	FALSE	1.24E-06
35102_at	0.16479	1.46147	0.02275	0.05983	0.26975	FALSE	5.52E-09
37425_g_at	0.16481	1.461538	0.024924	0.04982	0.2798	FALSE	4.78E-07
34397_at	0.16484	1.461639	0.013065	0.10456	0.22512	FALSE	2.16E-32
32539_at	0.16508	1.462447	0.028298	0.03452	0.29564	FALSE	6.85E-05
472_at	0.1651	1.462514	0.074229	-0.17736	0.50756	TRUE	1.00E+00
38666_at	0.16518	1.462783	0.022055	0.06343	0.26694	FALSE	8.72E-10
35552_at	0.16521	1.462884	0.047812	-0.05537	0.3858	TRUE	1.00E+00
40564_at	0.16522	1.462918	0.024844	0.0506	0.27984	FALSE	3.70E-07
36758_at	0.16525	1.463019	0.022023	0.06365	0.26686	FALSE	7.83E-10
36695_at	0.16528	1.46312	0.060522	-0.11394	0.44451	TRUE	1.00E+00
1508_at	0.16532	1.463255	0.031965	0.01785	0.3128	FALSE	2.93E-03
40124_at	0.1654	1.463525	0.044357	-0.03924	0.37004	TRUE	1.00E+00
37481_at	0.16545	1.463693	0.017133	0.08641	0.2445	FALSE	5.81E-18
40482_s_at	0.16547	1.46376	0.059885	-0.11082	0.44175	TRUE	1.00E+00
31389_at	0.16552	1.463929	0.068169	-0.14898	0.48002	TRUE	1.00E+00
40773_at	0.16579	1.464839	0.055114	-0.08848	0.42006	TRUE	1.00E+00
40563_at	0.16588	1.465143	0.02644	0.0439	0.28787	FALSE	4.45E-06
36033_at	0.16604	1.465683	0.023686	0.05676	0.27531	FALSE	3.01E-08
33102_at	0.16607	1.465784	0.031451	0.02097	0.31117	FALSE	1.63E-03
39555_at	0.16634	1.466696	0.05658	-0.0947	0.42737	TRUE	1.00E+00
40579_at	0.16646	1.467101	0.032439	0.01679	0.31612	FALSE	3.63E-03
32895_f_at	0.16675	1.468081	0.100361	-0.29627	0.62977	TRUE	1.00E+00
33328_at	0.16678	1.468182	0.039705	-0.0164	0.34996	TRUE	3.36E-01
39951_at	0.16691	1.468622	0.024761	0.05268	0.28115	FALSE	1.99E-07
33495_at	0.16707	1.469163	0.062937	-0.1233	0.45743	TRUE	1.00E+00
32363_at	0.16726	1.469806	0.040774	-0.02085	0.35537	TRUE	5.17E-01
41456_at	0.16744	1.470415	0.084252	-0.22127	0.55614	TRUE	1.00E+00
37891_at	0.16751	1.470652	0.021127	0.07004	0.26498	FALSE	2.80E-11
34975_at	0.16757	1.470855	0.081274	-0.20739	0.54254	TRUE	1.00E+00
33955_at	0.16765	1.471126	0.060404	-0.11103	0.44633	TRUE	1.00E+00
36984_f_at	0.16773	1.471397	0.041939	-0.02576	0.36121	TRUE	8.02E-01
31427_at	0.16774	1.471431	0.119645	-0.38425	0.71974	TRUE	1.00E+00
35454_at	0.16775	1.471465	0.043234	-0.03171	0.36722	TRUE	1.00E+00
35676_at	0.16777	1.471533	0.090412	-0.24936	0.58489	TRUE	1.00E+00
35189_at	0.16781	1.471669	0.104636	-0.31494	0.65055	TRUE	1.00E+00
36897_at	0.16785	1.471804	0.041536	-0.02378	0.35948	TRUE	6.72E-01
33747_s_at	0.16789	1.47194	0.052111	-0.07253	0.40831	TRUE	1.00E+00
37458_at	0.16796	1.472177	0.159179	-0.56643	0.90235	TRUE	1.00E+00
37246_at	0.16799	1.472279	0.059728	-0.10756	0.44355	TRUE	1.00E+00
247_s_at	0.16806	1.472516	0.08996	-0.24698	0.5831	TRUE	1.00E+00

1892_s_at	0.1681	1.472652	0.079977	-0.20088	0.53709	TRUE	1.00E+00
36177_at	0.16831	1.473364	0.030694	0.0267	0.30992	FALSE	5.26E-04
40808_at	0.16832	1.473398	0.082504	-0.21232	0.54896	TRUE	1.00E+00
1359_at	0.16845	1.473839	0.103242	-0.30786	0.64477	TRUE	1.00E+00
40422_at	0.1686	1.474348	0.048364	-0.05453	0.39173	TRUE	1.00E+00
39648_at	0.16871	1.474721	0.051589	-0.0693	0.40672	TRUE	1.00E+00
39671_at	0.16881	1.475061	0.06266	-0.12028	0.45789	TRUE	1.00E+00
35149_at	0.16886	1.475231	0.106927	-0.32446	0.66217	TRUE	1.00E+00
40616_at	0.16886	1.475231	0.046057	-0.04363	0.38135	TRUE	1.00E+00
33626_at	0.16887	1.475265	0.053148	-0.07633	0.41408	TRUE	1.00E+00
41006_at	0.169	1.475707	0.049068	-0.05738	0.39538	TRUE	1.00E+00
38890_at	0.16904	1.475842	0.102717	-0.30485	0.64293	TRUE	1.00E+00
41480_at	0.16915	1.476216	0.021133	0.07165	0.26665	FALSE	1.52E-11
32368_at	0.16922	1.476454	0.184134	-0.6803	1.01874	TRUE	1.00E+00
32639_at	0.16934	1.476862	0.027054	0.04452	0.29415	FALSE	4.88E-06
31677_at	0.16949	1.477372	0.074874	-0.17595	0.51493	TRUE	1.00E+00
33253_at	0.16949	1.477372	0.139804	-0.4755	0.81449	TRUE	1.00E+00
41208_at	0.1695	1.477406	0.02185	0.0687	0.27031	FALSE	1.09E-10
37442_at	0.16958	1.477679	0.091377	-0.252	0.59115	TRUE	1.00E+00
34353_at	0.16958	1.477679	0.01312	0.10905	0.23012	FALSE	4.10E-34
1640_at	0.16963	1.477849	0.030921	0.02697	0.31228	FALSE	5.20E-04
39118_at	0.16988	1.4787	0.022096	0.06794	0.27182	FALSE	1.88E-10
39669_at	0.16996	1.478972	0.052897	-0.07408	0.41401	TRUE	1.00E+00
37063_r_at	0.17004	1.479245	0.151782	-0.53022	0.8703	TRUE	1.00E+00
39583_at	0.17006	1.479313	0.028034	0.04072	0.29939	FALSE	1.65E-05
34257_at	0.17007	1.479347	0.023267	0.06273	0.27741	FALSE	3.38E-09
32916_at	0.17016	1.479653	0.083217	-0.21377	0.55408	TRUE	1.00E+00
32747_at	0.17024	1.479926	0.033388	0.0162	0.32428	FALSE	4.32E-03
32501_at	0.17025	1.47996	0.14965	-0.52017	0.86068	TRUE	1.00E+00
40915_r_at	0.17026	1.479994	0.117112	-0.37004	0.71057	TRUE	1.00E+00
36755_s_at	0.17032	1.480199	0.082248	-0.20914	0.54977	TRUE	1.00E+00
33383_f_at	0.17062	1.481221	0.055235	-0.08422	0.42545	TRUE	1.00E+00
38354_at	0.17068	1.481426	0.025747	0.05189	0.28947	FALSE	4.26E-07
33943_at	0.17072	1.481563	0.023833	0.06077	0.28068	FALSE	9.95E-09
39091_at	0.17086	1.48204	0.019326	0.0817	0.26003	FALSE	1.20E-14
34271_at	0.171	1.482518	0.067503	-0.14043	0.48243	TRUE	1.00E+00
35943_s_at	0.17114	1.482996	0.047001	-0.04571	0.38798	TRUE	1.00E+00
38071_at	0.17131	1.483577	0.021977	0.06992	0.27271	FALSE	8.13E-11
1457_at	0.17138	1.483816	0.016366	0.09587	0.24689	FALSE	1.48E-21
39283_at	0.17146	1.484089	0.01297	0.11162	0.2313	FALSE	8.51E-36
37926_at	0.17154	1.484363	0.041719	-0.02093	0.36401	TRUE	4.96E-01
34703_f_at	0.17162	1.484636	0.053384	-0.07467	0.41791	TRUE	1.00E+00
41648_at	0.17174	1.485046	0.019876	0.08004	0.26344	FALSE	7.07E-14
36174_at	0.17187	1.485491	0.028107	0.04219	0.30154	FALSE	1.22E-05
35479_at	0.1719	1.485594	0.125123	-0.40537	0.74916	TRUE	1.00E+00
33531_at	0.17216	1.486483	0.14325	-0.48874	0.83305	TRUE	1.00E+00
36086_at	0.17219	1.486586	0.11296	-0.34896	0.69334	TRUE	1.00E+00
36683_at	0.17233	1.487065	0.046454	-0.04199	0.38665	TRUE	1.00E+00
31743_at	0.17236	1.487168	0.134678	-0.44899	0.7937	TRUE	1.00E+00
40432_at	0.17236	1.487168	0.03569	0.0077	0.33702	FALSE	1.73E-02
36365_at	0.17237	1.487202	0.184021	-0.67662	1.02137	TRUE	1.00E+00

31691_g_at	0.17243	1.487408	0.021546	0.07302	0.27183	FALSE	1.54E-11
33852_at	0.17246	1.48751	0.037001	0.00175	0.34317	FALSE	3.97E-02
35131_at	0.17257	1.487887	0.102561	-0.3006	0.64575	TRUE	1.00E+00
36600_at	0.17259	1.487956	0.030962	0.02974	0.31544	FALSE	3.14E-04
35599_at	0.17276	1.488538	0.079613	-0.19454	0.54006	TRUE	1.00E+00
235_at	0.17278	1.488607	0.081074	-0.20126	0.54682	TRUE	1.00E+00
38195_at	0.17311	1.489738	0.122899	-0.3939	0.74011	TRUE	1.00E+00
1261_i_at	0.17314	1.489841	0.095102	-0.26562	0.6119	TRUE	1.00E+00
32482_at	0.17327	1.490287	0.086522	-0.2259	0.57245	TRUE	1.00E+00
38359_at	0.17339	1.490699	0.052337	-0.06807	0.41485	TRUE	1.00E+00
40639_at	0.1734	1.490733	0.037126	0.00212	0.34469	FALSE	3.79E-02
35390_at	0.17344	1.490871	0.112768	-0.34682	0.69371	TRUE	1.00E+00
35562_at	0.1735	1.491077	0.085154	-0.21936	0.56637	TRUE	1.00E+00
36152_at	0.1735	1.491077	0.051632	-0.0647	0.41171	TRUE	1.00E+00
36263_g_at	0.17351	1.491111	0.022754	0.06853	0.27848	FALSE	3.07E-10
36560_at	0.17351	1.491111	0.043622	-0.02774	0.37477	TRUE	8.79E-01
38470_i_at	0.17352	1.491145	0.031719	0.02718	0.31986	FALSE	5.67E-04
37934_at	0.17356	1.491283	0.053106	-0.07145	0.41857	TRUE	1.00E+00
39302_at	0.17358	1.491351	0.057637	-0.09234	0.43949	TRUE	1.00E+00
32585_at	0.17363	1.491523	0.029099	0.03938	0.30788	FALSE	3.05E-05
40117_at	0.17363	1.491523	0.029999	0.03523	0.31203	FALSE	9.00E-05
31678_at	0.17369	1.491729	0.076562	-0.17954	0.52691	TRUE	1.00E+00
34190_at	0.17369	1.491729	0.15593	-0.54571	0.89309	TRUE	1.00E+00
34579_r_at	0.17396	1.492657	0.132221	-0.43606	0.78397	TRUE	1.00E+00
affx-m27830_3_a	0.17398	1.492726	0.059003	-0.09824	0.44619	TRUE	1.00E+00
37055_at	0.17404	1.492932	0.066249	-0.13161	0.47968	TRUE	1.00E+00
39146_at	0.1741	1.493138	0.054048	-0.07525	0.42346	TRUE	1.00E+00
682_at	0.17412	1.493207	0.061918	-0.11154	0.45979	TRUE	1.00E+00
36099_at	0.17424	1.49362	0.050499	-0.05874	0.40722	TRUE	1.00E+00
37983_at	0.17431	1.49386	0.113036	-0.3472	0.69581	TRUE	1.00E+00
36259_at	0.17435	1.493998	0.110218	-0.33415	0.68285	TRUE	1.00E+00
40375_at	0.17436	1.494032	0.026923	0.05015	0.29858	FALSE	1.19E-06
1693_s_at	0.17441	1.494204	0.071495	-0.15543	0.50426	TRUE	1.00E+00
36069_at	0.17456	1.494721	0.020405	0.08042	0.2687	FALSE	1.49E-13
1418_at	0.17459	1.494824	0.03553	0.01067	0.33851	FALSE	1.13E-02
32588_s_at	0.17462	1.494927	0.029759	0.03732	0.31192	FALSE	5.58E-05
35972_at	0.17472	1.495271	0.066575	-0.13243	0.48187	TRUE	1.00E+00
39989_at	0.17493	1.495995	0.025771	0.05603	0.29383	FALSE	1.44E-07
38082_at	0.17495	1.496063	0.030103	0.03607	0.31383	FALSE	7.80E-05
32086_at	0.175	1.496236	0.034521	0.01574	0.33427	FALSE	5.04E-03
38105_at	0.17503	1.496339	0.020978	0.07825	0.27181	FALSE	9.09E-13
31493_s_at	0.17509	1.496546	0.09268	-0.2525	0.60267	TRUE	1.00E+00
38645_at	0.17521	1.496959	0.034682	0.0152	0.33522	FALSE	5.52E-03
1087_at	0.1753	1.49727	0.115957	-0.35968	0.71027	TRUE	1.00E+00
37694_at	0.17539	1.49758	0.022436	0.07188	0.2789	FALSE	6.81E-11
35925_at	0.17571	1.498684	0.060796	-0.10477	0.4562	TRUE	1.00E+00
38600_r_at	0.17585	1.499167	0.086671	-0.22401	0.57571	TRUE	1.00E+00
436_at	0.17592	1.499409	0.104424	-0.30585	0.65769	TRUE	1.00E+00
1250_at	0.176	1.499685	0.041815	-0.01692	0.36891	TRUE	3.24E-01
37170_at	0.17609	1.499996	0.038929	-0.00351	0.35569	TRUE	7.68E-02
34805_at	0.1761	1.50003	0.066021	-0.12849	0.48069	TRUE	1.00E+00

566_at	0.1761	1.50003	0.063124	-0.11513	0.46733	TRUE	1.00E+00
37123_at	0.17613	1.500134	0.026255	0.055	0.29726	FALSE	2.48E-07
34207_at	0.17616	1.500237	0.060187	-0.10152	0.45384	TRUE	1.00E+00
36527_at	0.17626	1.500583	0.023688	0.06697	0.28554	FALSE	1.26E-09
34420_at	0.17627	1.500617	0.058889	-0.09542	0.44796	TRUE	1.00E+00
35157_at	0.17628	1.500652	0.093914	-0.25701	0.60956	TRUE	1.00E+00
35726_at	0.1764	1.501067	0.047047	-0.04065	0.39346	TRUE	1.00E+00
37050_r_at	0.17656	1.50162	0.047666	-0.04336	0.39647	TRUE	1.00E+00
40334_at	0.17671	1.502139	0.043014	-0.02174	0.37516	TRUE	5.03E-01
36163_at	0.17671	1.502139	0.022505	0.07288	0.28054	FALSE	5.16E-11
1305_s_at	0.17673	1.502208	0.049939	-0.05367	0.40712	TRUE	1.00E+00
38396_at	0.17673	1.502208	0.015518	0.10514	0.24832	FALSE	6.00E-26
39825_at	0.17695	1.502969	0.027223	0.05135	0.30254	FALSE	1.02E-06
1909_at	0.17698	1.503073	0.050434	-0.0557	0.40967	TRUE	1.00E+00
214_at	0.17708	1.503419	0.037577	0.00372	0.35045	FALSE	3.09E-02
31760_at	0.17725	1.504007	0.027409	0.0508	0.30371	FALSE	1.26E-06
33006_at	0.17727	1.504077	0.075365	-0.17043	0.52497	TRUE	1.00E+00
36857_at	0.17739	1.504492	0.024066	0.06635	0.28842	FALSE	2.14E-09
32165_at	0.17744	1.504666	0.052215	-0.06345	0.41834	TRUE	1.00E+00
930_at	0.17757	1.505116	0.027592	0.05027	0.30486	FALSE	1.55E-06
1973_s_at	0.1777	1.505567	0.020036	0.08526	0.27014	FALSE	9.33E-15
33482_at	0.17784	1.506052	0.113241	-0.34461	0.70029	TRUE	1.00E+00
41286_at	0.17797	1.506503	0.095753	-0.2638	0.61973	TRUE	1.00E+00
40391_at	0.17815	1.507128	0.017574	0.09707	0.25922	FALSE	4.79E-20
38095_i_at	0.17821	1.507336	0.023433	0.0701	0.28633	FALSE	3.60E-10
37567_at	0.17832	1.507718	0.033447	0.024	0.33263	FALSE	1.23E-03
32360_s_at	0.17843	1.5081	0.079735	-0.18943	0.5463	TRUE	1.00E+00
33710_at	0.17871	1.509072	0.019045	0.09085	0.26658	FALSE	8.04E-17
36272_r_at	0.17876	1.509246	0.122835	-0.38795	0.74547	TRUE	1.00E+00
38594_i_at	0.1788	1.509385	0.086876	-0.22202	0.57961	TRUE	1.00E+00
38827_at	0.17884	1.509524	0.163411	-0.57507	0.93275	TRUE	1.00E+00
38951_at	0.17886	1.509593	0.082514	-0.20182	0.55955	TRUE	1.00E+00
36229_at	0.17903	1.510184	0.048121	-0.04299	0.40104	TRUE	1.00E+00
31972_at	0.17915	1.510602	0.087613	-0.22506	0.58336	TRUE	1.00E+00
33959_at	0.17933	1.511228	0.091224	-0.24154	0.6002	TRUE	1.00E+00
39912_at	0.17937	1.511367	0.056741	-0.08241	0.44115	TRUE	1.00E+00
40731_at	0.17965	1.512342	0.153396	-0.52806	0.88735	TRUE	1.00E+00
33427_s_at	0.17973	1.512621	0.047441	-0.03915	0.3986	TRUE	1.00E+00
36352_at	0.17983	1.512969	0.125085	-0.39726	0.75692	TRUE	1.00E+00
41120_at	0.17984	1.513004	0.107997	-0.31841	0.67809	TRUE	1.00E+00
31408_at	0.1799	1.513213	0.063237	-0.11185	0.47166	TRUE	1.00E+00
31815_r_at	0.17993	1.513317	0.075214	-0.16707	0.52693	TRUE	1.00E+00
36725_at	0.18001	1.513596	0.054674	-0.07223	0.43226	TRUE	1.00E+00
38144_at	0.18002	1.513631	0.144747	-0.48778	0.84783	TRUE	1.00E+00
35143_at	0.18013	1.514014	0.062337	-0.10746	0.46773	TRUE	1.00E+00
36087_at	0.18018	1.514189	0.047836	-0.04052	0.40088	TRUE	1.00E+00
32125_at	0.18031	1.514642	0.1191	-0.36917	0.72979	TRUE	1.00E+00
35563_at	0.18037	1.514851	0.058731	-0.09059	0.45133	TRUE	1.00E+00
37708_r_at	0.18052	1.515375	0.029707	0.04346	0.31757	FALSE	1.55E-05
31422_at	0.18052	1.515375	0.075543	-0.168	0.52905	TRUE	1.00E+00
38510_at	0.18081	1.516387	0.156251	-0.54007	0.90169	TRUE	1.00E+00

34600_s_at	0.18086	1.516561	0.055438	-0.0749	0.43663	TRUE	1.00E+00
38106_at	0.18102	1.51712	0.021498	0.08183	0.2802	FALSE	4.75E-13
37409_at	0.18104	1.51719	0.026858	0.05713	0.30495	FALSE	1.99E-07
34028_at	0.18111	1.517435	0.098318	-0.27249	0.63471	TRUE	1.00E+00
35161_at	0.18118	1.517679	0.049429	-0.04686	0.40923	TRUE	1.00E+00
988_at	0.18147	1.518693	0.131483	-0.42514	0.78807	TRUE	1.00E+00
33477_at	0.18147	1.518693	0.081289	-0.19357	0.5565	TRUE	1.00E+00
40211_at	0.18151	1.518833	0.034476	0.02245	0.34057	FALSE	1.77E-03
32011_g_at	0.18157	1.519043	0.065758	-0.12181	0.48495	TRUE	1.00E+00
39884_g_at	0.18164	1.519288	0.031113	0.0381	0.32518	FALSE	6.66E-05
33457_at	0.18172	1.519568	0.033028	0.02935	0.3341	FALSE	4.74E-04
34709_r_at	0.18178	1.519777	0.031388	0.03697	0.32659	FALSE	8.81E-05
39738_at	0.18192	1.520267	0.0488	-0.04323	0.40706	TRUE	1.00E+00
37466_at	0.18199	1.520513	0.020883	0.08564	0.27834	FALSE	3.68E-14
41158_at	0.18201	1.520583	0.050758	-0.05217	0.41618	TRUE	1.00E+00
39483_s_at	0.18205	1.520723	0.144748	-0.48576	0.84986	TRUE	1.00E+00
35632_at	0.18212	1.520968	0.022237	0.07953	0.28471	FALSE	3.30E-12
38843_at	0.18213	1.521003	0.019284	0.09316	0.2711	FALSE	4.50E-17
33372_at	0.18227	1.521493	0.040145	-0.00295	0.36748	TRUE	7.10E-02
37774_at	0.1823	1.521598	0.090781	-0.23653	0.60113	TRUE	1.00E+00
759_at	0.18248	1.522229	0.029349	0.04707	0.31788	FALSE	6.38E-06
31849_at	0.18257	1.522545	0.042128	-0.01179	0.37694	TRUE	1.85E-01
32726_g_at	0.18257	1.522545	0.047214	-0.03525	0.4004	TRUE	1.00E+00
41449_at	0.1827	1.523	0.036814	0.01285	0.35254	FALSE	8.78E-03
915_at	0.18275	1.523176	0.028529	0.05113	0.31438	FALSE	1.89E-06
34194_at	0.18286	1.523562	0.099571	-0.27653	0.64224	TRUE	1.00E+00
33862_at	0.18287	1.523597	0.042345	-0.01249	0.37823	TRUE	1.98E-01
37661_at	0.18287	1.523597	0.031471	0.03768	0.32807	FALSE	7.84E-05
32561_at	0.18289	1.523667	0.014018	0.11822	0.24757	FALSE	8.34E-35
37004_at	0.1829	1.523702	0.071683	-0.14782	0.51361	TRUE	1.00E+00
41104_at	0.18294	1.523842	0.090388	-0.23408	0.59995	TRUE	1.00E+00
37976_at	0.18295	1.523877	0.072515	-0.15161	0.5175	TRUE	1.00E+00
31624_at	0.18296	1.523912	0.060877	-0.0979	0.46382	TRUE	1.00E+00
37985_at	0.18296	1.523912	0.141712	-0.47084	0.83676	TRUE	1.00E+00
39110_at	0.18314	1.524544	0.017381	0.10295	0.26332	FALSE	7.39E-22
38535_at	0.18341	1.525492	0.080493	-0.18795	0.55477	TRUE	1.00E+00
34396_at	0.18354	1.525949	0.030121	0.04457	0.3225	FALSE	1.40E-05
40878_f_at	0.18397	1.527461	0.035571	0.01986	0.34808	FALSE	2.93E-03
40415_at	0.1841	1.527918	0.047826	-0.03655	0.40475	TRUE	1.00E+00
33580_r_at	0.18411	1.527953	0.057672	-0.08196	0.45018	TRUE	1.00E+00
1080_s_at	0.18418	1.528199	0.058581	-0.08608	0.45445	TRUE	1.00E+00
33028_at	0.18424	1.52841	0.078212	-0.1766	0.54508	TRUE	1.00E+00
34652_at	0.1843	1.528622	0.130774	-0.41903	0.78764	TRUE	1.00E+00
34328_s_at	0.18454	1.529467	0.031543	0.03902	0.33006	FALSE	6.19E-05
879_at	0.18454	1.529467	0.040744	-0.00344	0.37252	TRUE	7.47E-02
36175_s_at	0.18461	1.529713	0.036002	0.01851	0.35071	FALSE	3.70E-03
37033_s_at	0.18466	1.529889	0.045718	-0.02627	0.39558	TRUE	6.78E-01
36933_at	0.18467	1.529925	0.053699	-0.06307	0.43241	TRUE	1.00E+00
35870_at	0.18472	1.530101	0.058625	-0.08576	0.45519	TRUE	1.00E+00
31679_at	0.18475	1.530206	0.097418	-0.26469	0.6342	TRUE	1.00E+00
39021_at	0.18476	1.530242	0.016819	0.10717	0.26236	FALSE	5.66E-24

33997_at	0.18477	1.530277	0.035711	0.02001	0.34952	FALSE	2.89E-03
33262_at	0.18481	1.530418	0.101925	-0.28543	0.65505	TRUE	1.00E+00
40314_at	0.18486	1.530594	0.069697	-0.13669	0.50641	TRUE	1.00E+00
2056_at	0.18497	1.530982	0.038374	0.00793	0.36202	FALSE	1.81E-02
36015_at	0.18502	1.531158	0.028231	0.05477	0.31526	FALSE	7.09E-07
2063_at	0.18505	1.531264	0.068741	-0.13209	0.50219	TRUE	1.00E+00
1578_g_at	0.1851	1.53144	0.033915	0.02863	0.34157	FALSE	6.08E-04
34741_at	0.18518	1.531722	0.047167	-0.03243	0.40278	TRUE	1.00E+00
424_s_at	0.18518	1.531722	0.02729	0.05927	0.31109	FALSE	1.46E-07
33009_at	0.18523	1.531899	0.121094	-0.37345	0.7439	TRUE	1.00E+00
35888_at	0.18523	1.531899	0.073634	-0.15449	0.52495	TRUE	1.00E+00
35123_at	0.18536	1.532357	0.111644	-0.32972	0.70045	TRUE	1.00E+00
41352_at	0.18549	1.532816	0.034291	0.02729	0.3437	FALSE	7.98E-04
34759_at	0.18551	1.532887	0.030856	0.04315	0.32787	FALSE	2.31E-05
40990_at	0.18567	1.533451	0.032498	0.03574	0.3356	FALSE	1.40E-04
32033_at	0.18587	1.534158	0.096371	-0.25874	0.63049	TRUE	1.00E+00
35649_at	0.18589	1.534228	0.060973	-0.09541	0.46719	TRUE	1.00E+00
33975_at	0.18597	1.534511	0.105575	-0.30111	0.67305	TRUE	1.00E+00
38670_at	0.18613	1.535076	0.019453	0.09638	0.27587	FALSE	1.38E-17
36132_at	0.18632	1.535748	0.031677	0.04017	0.33246	FALSE	5.13E-05
36597_at	0.18636	1.53589	0.018712	0.10003	0.27269	FALSE	2.90E-19
337_at	0.18661	1.536774	0.064276	-0.10993	0.48315	TRUE	1.00E+00
41349_at	0.18669	1.537057	0.047395	-0.03197	0.40535	TRUE	1.00E+00
33517_f_at	0.18673	1.537199	0.11965	-0.36528	0.73875	TRUE	1.00E+00
38207_at	0.18675	1.537269	0.11244	-0.33201	0.7055	TRUE	1.00E+00
37528_at	0.18679	1.537411	0.033205	0.0336	0.33998	FALSE	2.34E-04
39847_at	0.1868	1.537446	0.041109	-0.00286	0.37646	TRUE	6.97E-02
1540_f_at	0.18687	1.537694	0.077734	-0.17176	0.54551	TRUE	1.00E+00
39334_s_at	0.18691	1.537836	0.060548	-0.09243	0.46626	TRUE	1.00E+00
41246_at	0.18696	1.538013	0.037817	0.01249	0.36143	FALSE	9.67E-03
31625_at	0.18706	1.538367	0.129636	-0.41102	0.78515	TRUE	1.00E+00
35807_at	0.18709	1.538473	0.075159	-0.15966	0.53384	TRUE	1.00E+00
39673_i_at	0.18714	1.538651	0.046313	-0.02653	0.40081	TRUE	6.72E-01
36056_g_at	0.18716	1.538721	0.073565	-0.15224	0.52656	TRUE	1.00E+00
40354_at	0.18721	1.538899	0.056066	-0.07145	0.44588	TRUE	1.00E+00
36885_at	0.18724	1.539005	0.0902	-0.2289	0.60339	TRUE	1.00E+00
34006_s_at	0.18746	1.539785	0.077092	-0.16822	0.54313	TRUE	1.00E+00
1604_at	0.18747	1.53982	0.087284	-0.21522	0.59016	TRUE	1.00E+00
33401_at	0.18749	1.539891	0.058601	-0.08287	0.45786	TRUE	1.00E+00
33731_at	0.18752	1.539997	0.08287	-0.19481	0.56985	TRUE	1.00E+00
39100_at	0.18756	1.540139	0.088991	-0.22301	0.59812	TRUE	1.00E+00
32824_at	0.18766	1.540494	0.019991	0.09543	0.27989	FALSE	7.78E-17
34521_at	0.18777	1.540884	0.078122	-0.17265	0.54819	TRUE	1.00E+00
31331_at	0.18782	1.541062	0.049409	-0.04013	0.41577	TRUE	1.00E+00
32601_s_at	0.18786	1.541204	0.072113	-0.14484	0.52056	TRUE	1.00E+00
39888_at	0.18792	1.541416	0.063043	-0.10293	0.47878	TRUE	1.00E+00
39805_at	0.18793	1.541452	0.03563	0.02355	0.35231	FALSE	1.68E-03
1606_at	0.18794	1.541487	0.056475	-0.07262	0.44849	TRUE	1.00E+00
36580_at	0.18798	1.541629	0.034586	0.02841	0.34755	FALSE	6.91E-04
41045_at	0.18819	1.542375	0.067582	-0.1236	0.49999	TRUE	1.00E+00
1736_at	0.18841	1.543157	0.046138	-0.02445	0.40127	TRUE	5.60E-01

39355_at	0.18843	1.543228	0.021628	0.08865	0.28821	FALSE	3.75E-14
37091_g_at	0.18849	1.543441	0.04209	-0.0057	0.38267	TRUE	9.51E-02
38681_at	0.18858	1.543761	0.019883	0.09685	0.28031	FALSE	3.07E-17
31479_f_at	0.18862	1.543903	0.110699	-0.3221	0.69934	TRUE	1.00E+00
36318_at	0.18862	1.543903	0.116193	-0.34744	0.72469	TRUE	1.00E+00
1759_f_at	0.18864	1.543974	0.055764	-0.06863	0.44591	TRUE	1.00E+00
34313_at	0.18875	1.544365	0.019226	0.10005	0.27745	FALSE	1.19E-18
37632_s_at	0.18877	1.544436	0.113741	-0.33598	0.71353	TRUE	1.00E+00
32196_at	0.1889	1.544899	0.019719	0.09792	0.27988	FALSE	1.23E-17
33895_at	0.18893	1.545005	0.018914	0.10167	0.27619	FALSE	2.15E-19
38706_at	0.18944	1.546821	0.076144	-0.16186	0.54074	TRUE	1.00E+00
40036_at	0.18949	1.546999	0.02077	0.09367	0.28532	FALSE	9.20E-16
1625_at	0.18952	1.547106	0.092916	-0.23916	0.61819	TRUE	1.00E+00
1672_f_at	0.18962	1.547462	0.062793	-0.10008	0.47932	TRUE	1.00E+00
32001_s_at	0.18964	1.547533	0.060758	-0.09067	0.46995	TRUE	1.00E+00
36202_at	0.18979	1.548068	0.060546	-0.08954	0.46912	TRUE	1.00E+00
40803_at	0.18999	1.548781	0.015296	0.11942	0.26056	FALSE	2.55E-31
34588_i_at	0.19014	1.549316	0.059017	-0.08214	0.46242	TRUE	1.00E+00
40559_at	0.19029	1.549851	0.083188	-0.1935	0.57409	TRUE	1.00E+00
38723_at	0.19038	1.550172	0.032781	0.03914	0.34162	FALSE	8.00E-05
38304_r_at	0.19049	1.550565	0.176313	-0.62295	1.00393	TRUE	1.00E+00
34936_at	0.19056	1.550815	0.029783	0.05315	0.32796	FALSE	1.99E-06
35586_at	0.19056	1.550815	0.068511	-0.12552	0.50664	TRUE	1.00E+00
1342_g_at	0.19059	1.550922	0.048827	-0.03468	0.41586	TRUE	1.00E+00
40156_at	0.1907	1.551315	0.053373	-0.05554	0.43694	TRUE	1.00E+00
39432_at	0.19076	1.551529	0.034839	0.03003	0.3515	FALSE	5.51E-04
141_s_at	0.19077	1.551565	0.076068	-0.16018	0.54172	TRUE	1.00E+00
334_s_at	0.19111	1.55278	0.112236	-0.3267	0.70892	TRUE	1.00E+00
39640_at	0.19115	1.552923	0.040246	0.00547	0.37683	FALSE	2.57E-02
31475_at	0.19131	1.553496	0.098951	-0.26521	0.64782	TRUE	1.00E+00
35645_at	0.19135	1.553639	0.078257	-0.1697	0.55239	TRUE	1.00E+00
984_g_at	0.19145	1.553996	0.093499	-0.23992	0.62281	TRUE	1.00E+00
41490_at	0.19152	1.554247	0.031162	0.04775	0.33528	FALSE	1.00E-05
40242_at	0.19166	1.554748	0.043517	-0.00911	0.39243	TRUE	1.34E-01
32606_at	0.19168	1.55482	0.026367	0.07003	0.31332	FALSE	4.55E-09
33486_at	0.19188	1.555536	0.092299	-0.23395	0.61772	TRUE	1.00E+00
35136_at	0.19192	1.555679	0.023765	0.08228	0.30157	FALSE	8.45E-12
37303_at	0.19193	1.555715	0.027313	0.06592	0.31794	FALSE	2.66E-08
41353_at	0.19203	1.556073	0.094401	-0.2435	0.62755	TRUE	1.00E+00
40171_at	0.19214	1.556467	0.042547	-0.00415	0.38844	TRUE	7.95E-02
35848_at	0.19224	1.556826	0.021469	0.09319	0.29129	FALSE	4.32E-15
33459_at	0.19226	1.556897	0.051355	-0.04467	0.42919	TRUE	1.00E+00
35927_r_at	0.19236	1.557256	0.147858	-0.4898	0.87451	TRUE	1.00E+00
32350_at	0.19236	1.557256	0.026672	0.06931	0.31541	FALSE	6.96E-09
39181_at	0.1924	1.557399	0.067888	-0.12081	0.50561	TRUE	1.00E+00
32755_at	0.19255	1.557937	0.077896	-0.16683	0.55193	TRUE	1.00E+00
35275_at	0.1926	1.558117	0.084049	-0.19516	0.58037	TRUE	1.00E+00
40320_at	0.19261	1.558153	0.152292	-0.51	0.89523	TRUE	1.00E+00
39818_at	0.19292	1.559265	0.040014	0.00831	0.37753	FALSE	1.80E-02
527_at	0.1931	1.559912	0.119757	-0.35941	0.74561	TRUE	1.00E+00
1325_at	0.19327	1.560522	0.02147	0.09421	0.29232	FALSE	2.81E-15

31361_at	0.19328	1.560558	0.096273	-0.25089	0.63744	TRUE	1.00E+00
38357_at	0.19341	1.561026	0.017834	0.11113	0.27569	FALSE	2.65E-23
37718_at	0.19346	1.561205	0.023922	0.08309	0.30382	FALSE	7.72E-12
38980_at	0.19353	1.561457	0.018426	0.10852	0.27854	FALSE	1.06E-21
35258_f_at	0.19359	1.561673	0.023844	0.08358	0.3036	FALSE	5.93E-12
31477_at	0.19369	1.562032	0.098319	-0.25991	0.64729	TRUE	1.00E+00
38818_at	0.19405	1.563328	0.030086	0.05524	0.33285	FALSE	1.41E-06
39807_at	0.19413	1.563616	0.11364	-0.33016	0.71842	TRUE	1.00E+00
33364_at	0.19416	1.563724	0.060119	-0.0832	0.47153	TRUE	1.00E+00
1066_at	0.19445	1.564768	0.087168	-0.2077	0.59661	TRUE	1.00E+00
333_s_at	0.19481	1.566066	0.020051	0.1023	0.28731	FALSE	3.26E-18
32018_at	0.19501	1.566787	0.071859	-0.13652	0.52654	TRUE	1.00E+00
39166_s_at	0.19528	1.567762	0.032078	0.04728	0.34327	FALSE	1.45E-05
37214_g_at	0.19537	1.568086	0.082337	-0.1845	0.57523	TRUE	1.00E+00
40914_s_at	0.19539	1.568159	0.124903	-0.38086	0.77164	TRUE	1.00E+00
871_s_at	0.19559	1.568881	0.121776	-0.36624	0.75741	TRUE	1.00E+00
33616_at	0.19559	1.568881	0.059355	-0.07825	0.46943	TRUE	1.00E+00
35092_at	0.19565	1.569098	0.073279	-0.14243	0.53373	TRUE	1.00E+00
40796_at	0.19574	1.569423	0.076886	-0.15898	0.55046	TRUE	1.00E+00
1304_at	0.1958	1.56964	0.083772	-0.19069	0.58229	TRUE	1.00E+00
746_at	0.19581	1.569676	0.072153	-0.13708	0.52869	TRUE	1.00E+00
37309_at	0.1962	1.571086	0.028723	0.06368	0.32871	FALSE	1.07E-07
37502_at	0.19635	1.571629	0.10669	-0.29587	0.68857	TRUE	1.00E+00
37096_at	0.1965	1.572172	0.121257	-0.36293	0.75593	TRUE	1.00E+00
36343_at	0.19657	1.572425	0.046794	-0.01932	0.41246	TRUE	3.36E-01
34529_at	0.19687	1.573512	0.06239	-0.09098	0.48471	TRUE	1.00E+00
41312_r_at	0.1969	1.57362	0.062764	-0.09266	0.48647	TRUE	1.00E+00
525_g_at	0.1973	1.575071	0.023407	0.08931	0.30529	FALSE	4.39E-13
39434_at	0.19733	1.575179	0.020323	0.10357	0.29109	FALSE	3.47E-18
39246_at	0.19733	1.575179	0.042835	-0.00029	0.39496	TRUE	5.16E-02
32287_s_at	0.1974	1.575433	0.116974	-0.34226	0.73707	TRUE	1.00E+00
910_at	0.19742	1.575506	0.217124	-0.8043	1.19914	TRUE	1.00E+00
38501_s_at	0.19742	1.575506	0.056216	-0.06193	0.45678	TRUE	1.00E+00
35427_at	0.19751	1.575832	0.059091	-0.07511	0.47014	TRUE	1.00E+00
1266_s_at	0.19753	1.575905	0.079538	-0.16943	0.56449	TRUE	1.00E+00
37954_at	0.19763	1.576268	0.073675	-0.14228	0.53753	TRUE	1.00E+00
1651_at	0.19785	1.577066	0.217009	-0.80334	1.19903	TRUE	1.00E+00
37680_at	0.19787	1.577139	0.042037	0.00393	0.39182	FALSE	3.17E-02
41682_s_at	0.19792	1.577321	0.032346	0.04869	0.34715	FALSE	1.19E-05
34136_s_at	0.19813	1.578084	0.080869	-0.17496	0.57123	TRUE	1.00E+00
35144_at	0.19858	1.57972	0.069844	-0.12365	0.52081	TRUE	1.00E+00
33672_f_at	0.19858	1.57972	0.092454	-0.22796	0.62513	TRUE	1.00E+00
31436_s_at	0.19866	1.580011	0.05959	-0.07626	0.47358	TRUE	1.00E+00
38329_at	0.19869	1.58012	0.080269	-0.17164	0.56902	TRUE	1.00E+00
320_at	0.19884	1.580666	0.044031	-0.00431	0.40198	TRUE	7.96E-02
34130_at	0.19887	1.580775	0.095365	-0.24111	0.63885	TRUE	1.00E+00
32670_at	0.19893	1.580993	0.100381	-0.26419	0.66204	TRUE	1.00E+00
40296_at	0.19904	1.581394	0.06352	-0.09401	0.4921	TRUE	1.00E+00
37978_at	0.19907	1.581503	0.050816	-0.03537	0.43351	TRUE	1.00E+00
41671_at	0.19912	1.581685	0.04563	-0.0114	0.40964	TRUE	1.61E-01
31798_at	0.19915	1.581794	0.084879	-0.19244	0.59075	TRUE	1.00E+00

39761_at	0.19919	1.58194	0.052965	-0.04516	0.44355	TRUE	1.00E+00
35066_g_at	0.19922	1.582049	0.042074	0.00511	0.39333	FALSE	2.77E-02
1051_g_at	0.19931	1.582377	0.088923	-0.21094	0.60956	TRUE	1.00E+00
36360_at	0.19935	1.582523	0.031453	0.05423	0.34446	FALSE	2.94E-06
33387_at	0.19939	1.582669	0.133882	-0.41829	0.81706	TRUE	1.00E+00
39746_at	0.19943	1.582814	0.03138	0.05466	0.34421	FALSE	2.63E-06
34907_at	0.19956	1.583288	0.095944	-0.24309	0.6422	TRUE	1.00E+00
37536_at	0.19963	1.583544	0.047726	-0.02056	0.41982	TRUE	3.63E-01
38602_at	0.19984	1.584309	0.081499	-0.17616	0.57585	TRUE	1.00E+00
41530_at	0.20004	1.585039	0.02439	0.08751	0.31256	FALSE	2.99E-12
35610_at	0.20023	1.585733	0.081874	-0.17751	0.57796	TRUE	1.00E+00
40091_at	0.20057	1.586975	0.012548	0.14268	0.25846	FALSE	2.05E-53
38072_at	0.20065	1.587267	0.03108	0.05727	0.34404	FALSE	1.36E-06
40592_at	0.20072	1.587523	0.03787	0.026	0.37544	FALSE	1.46E-03
39584_at	0.20084	1.587962	0.105629	-0.28649	0.68816	TRUE	1.00E+00
36262_at	0.20095	1.588364	0.01793	0.11823	0.28367	FALSE	4.72E-25
33342_at	0.20098	1.588474	0.039154	0.02034	0.38162	FALSE	3.60E-03
1845_at	0.20102	1.58862	0.069992	-0.12189	0.52393	TRUE	1.00E+00
34303_at	0.20104	1.588693	0.038219	0.02472	0.37737	FALSE	1.82E-03
1329_s_at	0.20118	1.589205	0.034487	0.04207	0.36029	FALSE	6.86E-05
39157_at	0.2012	1.589278	0.087001	-0.20018	0.60259	TRUE	1.00E+00
31901_at	0.20121	1.589315	0.058269	-0.06762	0.47004	TRUE	1.00E+00
39617_at	0.20165	1.590926	0.045648	-0.00895	0.41225	TRUE	1.26E-01
37228_at	0.20177	1.591366	0.112149	-0.31564	0.71918	TRUE	1.00E+00
31440_at	0.2018	1.591476	0.068269	-0.11316	0.51677	TRUE	1.00E+00
40530_at	0.20185	1.591659	0.05175	-0.0369	0.44061	TRUE	1.00E+00
33062_at	0.20199	1.592172	0.067666	-0.11019	0.51417	TRUE	1.00E+00
39464_at	0.20216	1.592795	0.061323	-0.08075	0.48508	TRUE	1.00E+00
33899_at	0.20227	1.593199	0.027721	0.07438	0.33017	FALSE	3.73E-09
31562_at	0.20238	1.593602	0.0435	0.00169	0.40307	FALSE	4.14E-02
33954_at	0.20255	1.594226	0.064475	-0.09491	0.50001	TRUE	1.00E+00
1219_at	0.20262	1.594483	0.039978	0.01818	0.38706	FALSE	5.07E-03
40760_at	0.20267	1.594667	0.076241	-0.14908	0.55441	TRUE	1.00E+00
37391_at	0.20269	1.59474	0.039823	0.01897	0.38642	FALSE	4.52E-03
32797_at	0.20276	1.594997	0.096448	-0.24221	0.64773	TRUE	1.00E+00
33905_at	0.20285	1.595328	0.036884	0.03268	0.37301	FALSE	4.80E-04
38862_at	0.203	1.595879	0.069991	-0.11991	0.52591	TRUE	1.00E+00
36026_at	0.20308	1.596173	0.076567	-0.15017	0.55633	TRUE	1.00E+00
37695_at	0.20328	1.596908	0.113952	-0.32244	0.72901	TRUE	1.00E+00
35046_at	0.20368	1.59838	0.182862	-0.63997	1.04733	TRUE	1.00E+00
40961_at	0.20377	1.598711	0.022404	0.10041	0.30714	FALSE	1.19E-15
39230_at	0.20378	1.598748	0.126452	-0.37962	0.78718	TRUE	1.00E+00
994_at	0.20387	1.599079	0.040224	0.0183	0.38945	FALSE	5.06E-03
37273_at	0.20389	1.599153	0.05393	-0.04492	0.45271	TRUE	1.00E+00
34158_s_at	0.20397	1.599448	0.145124	-0.46557	0.87351	TRUE	1.00E+00
38579_at	0.20398	1.599484	0.089726	-0.20998	0.61794	TRUE	1.00E+00
1293_s_at	0.20412	1.6	0.09214	-0.22098	0.62922	TRUE	1.00E+00
290_s_at	0.20414	1.600074	0.066092	-0.10079	0.50906	TRUE	1.00E+00
39638_at	0.20423	1.600405	0.036754	0.03466	0.3738	FALSE	3.47E-04
39869_at	0.20424	1.600442	0.077224	-0.15204	0.56052	TRUE	1.00E+00
1075_f_at	0.20436	1.600885	0.089088	-0.20666	0.61537	TRUE	1.00E+00

1265_g_at	0.20436	1.600885	0.027891	0.07568	0.33304	FALSE	2.97E-09
36855_r_at	0.20436	1.600885	0.071437	-0.12522	0.53394	TRUE	1.00E+00
1176_at	0.20437	1.600921	0.083397	-0.18039	0.58913	TRUE	1.00E+00
31998_at	0.20442	1.601106	0.109422	-0.30041	0.70924	TRUE	1.00E+00
affx-humgapdh/m	0.20452	1.601474	0.153875	-0.5054	0.91443	TRUE	1.00E+00
37923_at	0.20454	1.601548	0.046962	-0.01212	0.42121	TRUE	1.68E-01
37893_at	0.2046	1.601769	0.019655	0.11392	0.29529	FALSE	2.83E-21
38819_at	0.2048	1.602507	0.027696	0.07703	0.33258	FALSE	1.79E-09
34452_at	0.20488	1.602802	0.146513	-0.47107	0.88083	TRUE	1.00E+00
40597_g_at	0.20488	1.602802	0.042621	0.00825	0.40152	FALSE	1.93E-02
33716_at	0.20516	1.603836	0.037164	0.0337	0.37662	FALSE	4.27E-04
40962_s_at	0.20523	1.604095	0.028774	0.07248	0.33799	FALSE	1.24E-08
35906_at	0.20531	1.60439	0.051986	-0.03453	0.44515	TRUE	9.90E-01
36141_at	0.20549	1.605055	0.054469	-0.04581	0.45678	TRUE	1.00E+00
31964_at	0.20576	1.606053	0.09113	-0.21467	0.6262	TRUE	1.00E+00
40269_at	0.20591	1.606608	0.033827	0.04985	0.36198	FALSE	1.45E-05
31343_at	0.20597	1.60683	0.119936	-0.34737	0.7593	TRUE	1.00E+00
38119_at	0.20603	1.607052	0.043118	0.0071	0.40496	FALSE	2.23E-02
33276_at	0.20607	1.6072	0.079892	-0.16252	0.57466	TRUE	1.00E+00
34569_at	0.20613	1.607422	0.101864	-0.26383	0.67609	TRUE	1.00E+00
2068_s_at	0.20643	1.608533	0.055165	-0.04808	0.46094	TRUE	1.00E+00
37665_at	0.20653	1.608904	0.027136	0.08134	0.33172	FALSE	3.43E-10
41489_at	0.20664	1.609311	0.029549	0.07032	0.34297	FALSE	3.39E-08
36325_at	0.20671	1.60957	0.111922	-0.30965	0.72307	TRUE	1.00E+00
38222_at	0.20677	1.609793	0.038609	0.02864	0.3849	FALSE	1.08E-03
41585_at	0.20692	1.610349	0.049766	-0.02268	0.43652	TRUE	4.05E-01
37649_at	0.20704	1.610794	0.071205	-0.12147	0.53555	TRUE	1.00E+00
41019_at	0.20711	1.611054	0.122405	-0.35762	0.77183	TRUE	1.00E+00
37468_at	0.20718	1.611313	0.03685	0.03717	0.37719	FALSE	2.38E-04
35222_at	0.20742	1.612204	0.02722	0.08184	0.333	FALSE	3.20E-10
40543_at	0.20744	1.612278	0.097739	-0.24348	0.65837	TRUE	1.00E+00
38384_at	0.20747	1.61239	0.032092	0.05941	0.35553	FALSE	1.28E-06
34341_at	0.20749	1.612464	0.077853	-0.1517	0.56667	TRUE	1.00E+00
40588_r_at	0.20756	1.612724	0.041279	0.01711	0.398	FALSE	6.25E-03
39361_f_at	0.20763	1.612984	0.058346	-0.06155	0.47682	TRUE	1.00E+00
39438_at	0.20768	1.613169	0.037143	0.03632	0.37905	FALSE	2.84E-04
31869_at	0.20791	1.614024	0.0236	0.09903	0.31679	FALSE	1.59E-14
34181_at	0.20802	1.614433	0.032022	0.06029	0.35576	FALSE	1.04E-06
41719_i_at	0.20817	1.614991	0.053097	-0.0368	0.45313	TRUE	1.00E+00
38499_s_at	0.20827	1.615363	0.031201	0.06432	0.35222	FALSE	3.12E-07
32657_at	0.20842	1.615921	0.025544	0.09057	0.32626	FALSE	4.26E-12
41317_at	0.20844	1.615995	0.029245	0.07351	0.34336	FALSE	1.29E-08
36545_s_at	0.20858	1.616516	0.062731	-0.08084	0.49799	TRUE	1.00E+00
37456_at	0.20861	1.616628	0.059337	-0.06514	0.48237	TRUE	1.00E+00
31693_f_at	0.20867	1.616851	0.066789	-0.09946	0.51681	TRUE	1.00E+00
36442_g_at	0.20889	1.61767	0.089032	-0.20187	0.61965	TRUE	1.00E+00
39815_at	0.20892	1.617782	0.066784	-0.0992	0.51703	TRUE	1.00E+00
36700_at	0.2093	1.619198	0.030947	0.06652	0.35208	FALSE	1.70E-07
629_at	0.20953	1.620056	0.059784	-0.06629	0.48535	TRUE	1.00E+00
40746_at	0.20964	1.620466	0.077904	-0.14978	0.56905	TRUE	1.00E+00
40008_at	0.20978	1.620989	0.04665	-0.00544	0.42501	TRUE	8.70E-02

995_g_at	0.20987	1.621325	0.042039	0.01592	0.40382	FALSE	7.54E-03
34324_at	0.21044	1.623454	0.042094	0.01624	0.40465	FALSE	7.26E-03
795_s_at	0.21046	1.623529	0.047415	-0.00829	0.42921	TRUE	1.14E-01
33823_at	0.2106	1.624052	0.0145	0.1437	0.2775	FALSE	1.08E-43
33325_at	0.21074	1.624576	0.028558	0.07899	0.3425	FALSE	2.00E-09
33348_at	0.21095	1.625362	0.025462	0.09348	0.32842	FALSE	1.49E-12
1018_at	0.21104	1.625698	0.057176	-0.05274	0.47483	TRUE	1.00E+00
947_at	0.21105	1.625736	0.052433	-0.03086	0.45295	TRUE	7.19E-01
375_at	0.21114	1.626073	0.095525	-0.22957	0.65186	TRUE	1.00E+00
1175_s_at	0.21144	1.627196	0.166194	-0.55531	0.97819	TRUE	1.00E+00
36720_at	0.21152	1.627496	0.063452	-0.08122	0.50426	TRUE	1.00E+00
41815_at	0.21168	1.628096	0.075803	-0.13804	0.5614	TRUE	1.00E+00
37360_at	0.21168	1.628096	0.092014	-0.21284	0.63619	TRUE	1.00E+00
34800_at	0.2118	1.628546	0.056403	-0.04842	0.47202	TRUE	1.00E+00
37430_at	0.21189	1.628883	0.082754	-0.1699	0.59368	TRUE	1.00E+00
41527_f_at	0.212	1.629296	0.115102	-0.31903	0.74304	TRUE	1.00E+00
1816_at	0.21206	1.629521	0.068199	-0.10258	0.52671	TRUE	1.00E+00
35006_at	0.21213	1.629784	0.057436	-0.05286	0.47711	TRUE	1.00E+00
35350_at	0.21221	1.630084	0.039843	0.02839	0.39603	FALSE	1.27E-03
34804_at	0.21221	1.630084	0.062119	-0.07438	0.49881	TRUE	1.00E+00
34831_at	0.21222	1.630122	0.078095	-0.14808	0.57252	TRUE	1.00E+00
34514_at	0.21236	1.630647	0.078941	-0.15185	0.57656	TRUE	1.00E+00
41658_at	0.21239	1.63076	0.029419	0.07666	0.34811	FALSE	6.59E-09
38696_at	0.21243	1.63091	0.076747	-0.14165	0.56651	TRUE	1.00E+00
33821_at	0.21261	1.631586	0.023087	0.1061	0.31913	FALSE	4.15E-16
34561_at	0.21289	1.632638	0.042567	0.0165	0.40927	FALSE	7.19E-03
33376_at	0.21293	1.632789	0.079548	-0.15407	0.57993	TRUE	1.00E+00
39056_at	0.21296	1.632902	0.043344	0.01299	0.41293	FALSE	1.13E-02
31824_at	0.21302	1.633127	0.030647	0.07162	0.35441	FALSE	4.59E-08
39260_at	0.21303	1.633165	0.028898	0.07971	0.34636	FALSE	2.12E-09
32221_at	0.21344	1.634707	0.020344	0.11958	0.3073	FALSE	1.19E-21
37863_at	0.21362	1.635385	0.188145	-0.6544	1.08164	TRUE	1.00E+00
36893_at	0.21369	1.635649	0.046153	0.00076	0.42662	FALSE	4.61E-02
33739_at	0.21373	1.635799	0.104177	-0.2669	0.69436	TRUE	1.00E+00
39222_at	0.21383	1.636176	0.108816	-0.28821	0.71586	TRUE	1.00E+00
34955_at	0.21431	1.637985	0.039316	0.03292	0.3957	FALSE	6.33E-04
37207_at	0.21445	1.638513	0.138945	-0.42659	0.85549	TRUE	1.00E+00
36551_at	0.2145	1.638702	0.030474	0.07391	0.35509	FALSE	2.45E-08
1554_f_at	0.21453	1.638815	0.06748	-0.0968	0.52585	TRUE	1.00E+00
40985_g_at	0.21463	1.639193	0.135266	-0.40943	0.83869	TRUE	1.00E+00
33125_at	0.21472	1.639532	0.035326	0.05174	0.3777	FALSE	1.53E-05
37461_at	0.21474	1.639608	0.051295	-0.02191	0.4514	TRUE	3.58E-01
33516_at	0.21483	1.639948	0.087986	-0.1911	0.62077	TRUE	1.00E+00
31671_at	0.2149	1.640212	0.047441	-0.00398	0.43377	TRUE	7.46E-02
34212_at	0.21521	1.641383	0.106417	-0.27575	0.70618	TRUE	1.00E+00
35612_at	0.21534	1.641875	0.094361	-0.22001	0.65068	TRUE	1.00E+00
39161_at	0.21552	1.642555	0.082742	-0.16622	0.59726	TRUE	1.00E+00
32252_at	0.21558	1.642782	0.06357	-0.0777	0.50886	TRUE	1.00E+00
1036_at	0.21567	1.643123	0.065275	-0.08548	0.51682	TRUE	1.00E+00
604_at	0.21572	1.643312	0.050117	-0.0155	0.44694	TRUE	2.11E-01
37611_at	0.21576	1.643463	0.05473	-0.03674	0.46826	TRUE	1.00E+00

36680_at	0.21578	1.643539	0.112917	-0.30517	0.73674	TRUE	1.00E+00
38631_at	0.21578	1.643539	0.067419	-0.09526	0.52683	TRUE	1.00E+00
40288_r_at	0.21584	1.643766	0.062533	-0.07266	0.50434	TRUE	1.00E+00
36491_at	0.21598	1.644296	0.049399	-0.01193	0.44389	TRUE	1.55E-01
38859_at	0.21603	1.644485	0.053044	-0.02869	0.46075	TRUE	5.87E-01
37245_at	0.21612	1.644826	0.100063	-0.24553	0.67777	TRUE	1.00E+00
41568_at	0.21628	1.645432	0.071514	-0.11365	0.54622	TRUE	1.00E+00
579_at	0.21634	1.64566	0.033469	0.06192	0.37075	FALSE	1.29E-06
837_s_at	0.21638	1.645811	0.037127	0.0451	0.38767	FALSE	7.07E-05
35241_at	0.21649	1.646228	0.054941	-0.03699	0.46996	TRUE	1.00E+00
36849_at	0.21665	1.646835	0.02223	0.11409	0.31921	FALSE	2.43E-18
36178_at	0.21665	1.646835	0.010768	0.16697	0.26633	FALSE	6.27E-86
41717_at	0.21668	1.646948	0.033891	0.06032	0.37303	FALSE	2.05E-06
32087_at	0.21686	1.647631	0.025153	0.10081	0.3329	FALSE	8.35E-14
38122_at	0.21708	1.648466	0.029998	0.07868	0.35548	FALSE	5.81E-09
38852_at	0.21713	1.648656	0.050509	-0.0159	0.45016	TRUE	2.17E-01
40692_at	0.21721	1.64896	0.031607	0.07139	0.36303	FALSE	7.98E-08
35348_at	0.21736	1.649529	0.031318	0.07287	0.36185	FALSE	4.94E-08
1082_at	0.2175	1.650061	0.102167	-0.25385	0.68886	TRUE	1.00E+00
37995_s_at	0.21772	1.650897	0.032975	0.06558	0.36985	FALSE	5.11E-07
40071_at	0.21772	1.650897	0.032318	0.06862	0.36682	FALSE	2.04E-07
37624_at	0.21779	1.651163	0.113417	-0.30547	0.74104	TRUE	1.00E+00
889_at	0.21797	1.651848	0.083712	-0.16825	0.60418	TRUE	1.00E+00
38239_at	0.21799	1.651924	0.058925	-0.05386	0.48985	TRUE	1.00E+00
37024_at	0.218	1.651962	0.049603	-0.01084	0.44685	TRUE	1.40E-01
39952_at	0.21802	1.652038	0.045506	0.00808	0.42797	FALSE	2.09E-02
40377_at	0.2182	1.652723	0.056259	-0.04136	0.47776	TRUE	1.00E+00
35949_at	0.21828	1.653027	0.077304	-0.13836	0.57493	TRUE	1.00E+00
31338_at	0.2183	1.653103	0.03928	0.03708	0.39952	FALSE	3.45E-04
40434_at	0.21846	1.653712	0.09189	-0.20548	0.6424	TRUE	1.00E+00
217_at	0.2186	1.654246	0.045432	0.009	0.4282	FALSE	1.89E-02
39398_s_at	0.21872	1.654703	0.025258	0.10219	0.33525	FALSE	5.99E-14
38857_r_at	0.2188	1.655008	0.140794	-0.43076	0.86837	TRUE	1.00E+00
33131_at	0.21882	1.655084	0.038571	0.04087	0.39677	FALSE	1.77E-04
32976_s_at	0.21882	1.655084	0.142534	-0.43877	0.87642	TRUE	1.00E+00
32265_at	0.219	1.65577	0.109058	-0.28414	0.72215	TRUE	1.00E+00
38734_at	0.21903	1.655884	0.07333	-0.11929	0.55734	TRUE	1.00E+00
35622_at	0.21908	1.656075	0.083588	-0.16656	0.60472	TRUE	1.00E+00
35253_at	0.21933	1.657029	0.024137	0.10797	0.33069	FALSE	1.29E-15
38653_at	0.21954	1.65783	0.030214	0.08015	0.35893	FALSE	4.66E-09
34398_at	0.21961	1.658097	0.025276	0.10299	0.33622	FALSE	4.64E-14
34925_at	0.21974	1.658594	0.094141	-0.21458	0.65407	TRUE	1.00E+00
31892_at	0.21975	1.658632	0.058577	-0.0505	0.49	TRUE	1.00E+00
37163_at	0.21977	1.658708	0.086117	-0.17753	0.61708	TRUE	1.00E+00
40029_at	0.22011	1.660007	0.041885	0.02687	0.41335	FALSE	1.87E-03
36861_at	0.22038	1.66104	0.039838	0.03659	0.40417	FALSE	4.00E-04
40690_at	0.22055	1.66169	0.105707	-0.26713	0.70824	TRUE	1.00E+00
36920_at	0.22064	1.662034	0.026331	0.09916	0.34212	FALSE	6.72E-13
1053_at	0.22073	1.662379	0.026687	0.0976	0.34385	FALSE	1.68E-12
31635_g_at	0.22074	1.662417	0.115464	-0.31196	0.75345	TRUE	1.00E+00
40433_at	0.22096	1.663259	0.022025	0.11934	0.32257	FALSE	1.39E-19

37635_at	0.22119	1.664141	0.140768	-0.42825	0.87064	TRUE	1.00E+00
39734_at	0.22148	1.665252	0.055286	-0.03358	0.47655	TRUE	7.79E-01
33721_at	0.22171	1.666134	0.146702	-0.45511	0.89854	TRUE	1.00E+00
859_at	0.22172	1.666173	0.032075	0.07374	0.3697	FALSE	6.01E-08
39538_at	0.22175	1.666288	0.094341	-0.2135	0.657	TRUE	1.00E+00
40082_at	0.22179	1.666441	0.02614	0.10119	0.34239	FALSE	2.73E-13
33774_at	0.22183	1.666595	0.032179	0.07336	0.37029	FALSE	6.88E-08
31657_at	0.22187	1.666748	0.084873	-0.1697	0.61344	TRUE	1.00E+00
31891_at	0.2219	1.666863	0.081964	-0.15625	0.60005	TRUE	1.00E+00
affx-biob-m_st	0.22209	1.667593	0.038419	0.04484	0.39934	FALSE	9.39E-05
37249_at	0.22234	1.668553	0.087593	-0.18178	0.62646	TRUE	1.00E+00
34765_at	0.22242	1.66886	0.021904	0.12136	0.32347	FALSE	4.00E-20
38591_at	0.22275	1.670129	0.073683	-0.1172	0.56269	TRUE	1.00E+00
39611_at	0.22299	1.671052	0.062302	-0.06444	0.51042	TRUE	1.00E+00
36526_at	0.22325	1.672053	0.04265	0.02649	0.42002	FALSE	2.09E-03
37428_at	0.2233	1.672245	0.117124	-0.31706	0.76366	TRUE	1.00E+00
31322_at	0.22336	1.672476	0.136402	-0.40595	0.85266	TRUE	1.00E+00
38584_at	0.22349	1.672977	0.029637	0.08675	0.36022	FALSE	5.90E-10
32187_at	0.22349	1.672977	0.087608	-0.18069	0.62768	TRUE	1.00E+00
32833_at	0.22349	1.672977	0.032284	0.07455	0.37244	FALSE	5.60E-08
35457_at	0.22352	1.673093	0.056697	-0.03806	0.4851	TRUE	1.00E+00
2057_g_at	0.22358	1.673324	0.053703	-0.02418	0.47134	TRUE	3.96E-01
40114_at	0.2236	1.673401	0.088351	-0.18402	0.63121	TRUE	1.00E+00
41085_at	0.22377	1.674056	0.101929	-0.24649	0.69402	TRUE	1.00E+00
37698_at	0.22425	1.675907	0.022909	0.11855	0.32994	FALSE	1.59E-18
32247_at	0.22425	1.675907	0.050451	-0.00851	0.45701	TRUE	1.11E-01
33560_at	0.22426	1.675946	0.100646	-0.24008	0.6886	TRUE	1.00E+00
789_at	0.22443	1.676602	0.083114	-0.15902	0.60789	TRUE	1.00E+00
35208_at	0.22451	1.676911	0.079447	-0.14203	0.59104	TRUE	1.00E+00
33165_at	0.22456	1.677104	0.034087	0.0673	0.38182	FALSE	5.63E-07
34280_at	0.22459	1.67722	0.040031	0.0399	0.40927	FALSE	2.55E-04
37230_at	0.22466	1.67749	0.032476	0.07483	0.37449	FALSE	5.80E-08
31756_at	0.22472	1.677722	0.129196	-0.37134	0.82077	TRUE	1.00E+00
36312_at	0.22492	1.678495	0.069386	-0.0952	0.54504	TRUE	1.00E+00
39090_at	0.22501	1.678843	0.037782	0.0507	0.39932	FALSE	3.27E-05
41698_at	0.22505	1.678997	0.068796	-0.09234	0.54245	TRUE	1.00E+00
41616_at	0.22515	1.679384	0.09237	-0.20101	0.65131	TRUE	1.00E+00
39424_at	0.22566	1.681357	0.033141	0.07276	0.37856	FALSE	1.24E-07
37647_at	0.22576	1.681744	0.079692	-0.14191	0.59343	TRUE	1.00E+00
32060_at	0.22588	1.682209	0.035066	0.0641	0.38766	FALSE	1.49E-06
31899_at	0.22607	1.682945	0.017231	0.14657	0.30556	FALSE	3.20E-35
1968_g_at	0.22613	1.683178	0.043276	0.02648	0.42579	FALSE	2.19E-03
37357_at	0.22614	1.683217	0.017246	0.14657	0.3057	FALSE	3.52E-35
36226_r_at	0.22618	1.683372	0.017765	0.14421	0.30814	FALSE	4.99E-33
38042_at	0.22632	1.683914	0.029214	0.09154	0.3611	FALSE	1.19E-10
37015_at	0.22642	1.684302	0.115545	-0.30665	0.7595	TRUE	1.00E+00
1044_s_at	0.22683	1.685893	0.037538	0.05364	0.40001	FALSE	1.92E-05
36836_at	0.22686	1.686009	0.075569	-0.12178	0.57551	TRUE	1.00E+00
40837_at	0.22731	1.687757	0.087038	-0.17425	0.62886	TRUE	1.00E+00
41228_r_at	0.22735	1.687913	0.043706	0.02571	0.42899	FALSE	2.49E-03
35440_g_at	0.22737	1.687991	0.106276	-0.26294	0.71768	TRUE	1.00E+00

39202_at	0.22738	1.688029	0.10097	-0.23845	0.69321	TRUE	1.00E+00
35452_at	0.22787	1.689935	0.08026	-0.14242	0.59816	TRUE	1.00E+00
34094_i_at	0.22792	1.69013	0.066372	-0.07829	0.53413	TRUE	1.00E+00
31992_f_at	0.22794	1.690207	0.073104	-0.10934	0.56521	TRUE	1.00E+00
35245_at	0.22801	1.69048	0.105293	-0.25777	0.71379	TRUE	1.00E+00
33890_at	0.22807	1.690713	0.037081	0.057	0.39915	FALSE	9.74E-06
35713_at	0.22822	1.691297	0.07788	-0.13109	0.58753	TRUE	1.00E+00
36939_at	0.22825	1.691414	0.107855	-0.26935	0.72585	TRUE	1.00E+00
41368_at	0.22839	1.69196	0.08147	-0.14748	0.60426	TRUE	1.00E+00
36894_at	0.22846	1.692232	0.056006	-0.02993	0.48685	TRUE	5.71E-01
32063_at	0.22852	1.692466	0.021676	0.12851	0.32852	FALSE	6.96E-22
37887_at	0.22878	1.69348	0.065557	-0.07367	0.53124	TRUE	1.00E+00
40648_at	0.22901	1.694377	0.120322	-0.32611	0.78412	TRUE	1.00E+00
41635_at	0.2293	1.695509	0.036236	0.06212	0.39647	FALSE	3.14E-06
33240_at	0.22939	1.69586	0.086355	-0.16901	0.6278	TRUE	1.00E+00
39632_at	0.22958	1.696602	0.110576	-0.28058	0.73973	TRUE	1.00E+00
40381_at	0.22963	1.696797	0.083284	-0.15461	0.61387	TRUE	1.00E+00
35480_at	0.22985	1.697657	0.157547	-0.49701	0.95671	TRUE	1.00E+00
38913_at	0.22994	1.698009	0.019829	0.13846	0.32142	FALSE	5.43E-27
1815_g_at	0.23018	1.698948	0.020509	0.13556	0.3248	FALSE	3.95E-25
34283_at	0.2302	1.699026	0.068587	-0.08623	0.54664	TRUE	1.00E+00
36842_at	0.23025	1.699222	0.099956	-0.2309	0.6914	TRUE	1.00E+00
affx-biodn-3_st	0.23029	1.699378	0.146149	-0.44398	0.90456	TRUE	1.00E+00
36498_at	0.23033	1.699535	0.118695	-0.31728	0.77794	TRUE	1.00E+00
239_at	0.2308	1.701375	0.039749	0.04742	0.41419	FALSE	8.05E-05
31486_s_at	0.23108	1.702472	0.067757	-0.08152	0.54369	TRUE	1.00E+00
700_s_at	0.23113	1.702668	0.107339	-0.26409	0.72635	TRUE	1.00E+00
40708_at	0.23123	1.70306	0.093963	-0.20228	0.66474	TRUE	1.00E+00
33042_r_at	0.23163	1.70463	0.092286	-0.19414	0.6574	TRUE	1.00E+00
37522_r_at	0.23169	1.704865	0.129702	-0.3667	0.83009	TRUE	1.00E+00
39703_at	0.23191	1.705729	0.059077	-0.04065	0.50447	TRUE	1.00E+00
34048_at	0.23209	1.706436	0.053593	-0.01517	0.47934	TRUE	1.88E-01
1287_at	0.23228	1.707183	0.019586	0.14192	0.32264	FALSE	2.43E-28
35443_at	0.23239	1.707615	0.080697	-0.13991	0.60469	TRUE	1.00E+00
31799_at	0.23268	1.708756	0.022611	0.12836	0.337	FALSE	9.84E-21
38886_i_at	0.23268	1.708756	0.126551	-0.35117	0.81654	TRUE	1.00E+00
37542_at	0.2329	1.709622	0.083852	-0.15396	0.61975	TRUE	1.00E+00
39946_at	0.23303	1.710133	0.06263	-0.05591	0.52198	TRUE	1.00E+00
35252_at	0.23305	1.710212	0.045855	0.02149	0.44461	FALSE	4.71E-03
34751_at	0.23311	1.710448	0.027767	0.105	0.36122	FALSE	5.87E-13
31945_s_at	0.23325	1.711	0.115606	-0.30011	0.76661	TRUE	1.00E+00
35644_at	0.23328	1.711118	0.071748	-0.09773	0.5643	TRUE	1.00E+00
39619_at	0.23349	1.711946	0.033669	0.07815	0.38882	FALSE	5.14E-08
35565_at	0.23379	1.713129	0.096306	-0.21053	0.6781	TRUE	1.00E+00
34578_at	0.2339	1.713563	0.09247	-0.19272	0.66052	TRUE	1.00E+00
1951_at	0.23402	1.714036	0.136437	-0.39544	0.86349	TRUE	1.00E+00
39852_at	0.23404	1.714115	0.033221	0.08077	0.38731	FALSE	2.35E-08
38280_s_at	0.23406	1.714194	0.090326	-0.18267	0.65078	TRUE	1.00E+00
37437_at	0.23413	1.71447	0.038137	0.05818	0.41008	FALSE	1.05E-05
31940_s_at	0.23454	1.71609	0.062965	-0.05596	0.52503	TRUE	1.00E+00
36432_at	0.23456	1.716169	0.041948	0.04104	0.42809	FALSE	2.84E-04

716_at	0.23457	1.716208	0.040116	0.0495	0.41965	FALSE	6.30E-05
35481_at	0.23463	1.716445	0.045884	0.02294	0.44632	FALSE	3.99E-03
39176_f_at	0.23467	1.716604	0.067335	-0.07599	0.54533	TRUE	1.00E+00
41393_at	0.23477	1.716999	0.057953	-0.0326	0.50214	TRUE	6.44E-01
41457_at	0.23483	1.717236	0.026524	0.11246	0.3572	FALSE	1.07E-14
139_at	0.23489	1.717473	0.057357	-0.02974	0.49951	TRUE	5.33E-01
36206_at	0.23506	1.718146	0.04862	0.01075	0.45938	FALSE	1.68E-02
35234_at	0.23533	1.719214	0.043554	0.03439	0.43628	FALSE	8.26E-04
37400_at	0.23544	1.71965	0.118076	-0.30932	0.78019	TRUE	1.00E+00
1701_at	0.23546	1.719729	0.085419	-0.15862	0.62955	TRUE	1.00E+00
32280_at	0.2355	1.719887	0.046528	0.02084	0.45016	FALSE	5.25E-03
36267_at	0.23559	1.720244	0.079073	-0.12922	0.6004	TRUE	1.00E+00
32404_at	0.23563	1.720402	0.067745	-0.07692	0.54818	TRUE	1.00E+00
33705_at	0.23577	1.720957	0.152702	-0.46874	0.94027	TRUE	1.00E+00
40554_at	0.23593	1.721591	0.109612	-0.26977	0.74164	TRUE	1.00E+00
34013_f_at	0.236	1.721869	0.089398	-0.17645	0.64844	TRUE	1.00E+00
34930_at	0.23606	1.722106	0.102702	-0.23777	0.70988	TRUE	1.00E+00
35111_at	0.23633	1.723177	0.089411	-0.17618	0.64883	TRUE	1.00E+00
35831_at	0.23637	1.723336	0.055936	-0.02169	0.49444	TRUE	3.01E-01
1361_at	0.23656	1.72409	0.041243	0.04628	0.42684	FALSE	1.23E-04
38302_at	0.23667	1.724527	0.049131	0.01	0.46334	FALSE	1.84E-02
34564_at	0.23667	1.724527	0.187971	-0.63055	1.10389	TRUE	1.00E+00
41100_at	0.23671	1.724686	0.038314	0.05994	0.41347	FALSE	8.19E-06
35291_at	0.23672	1.724726	0.023948	0.12623	0.3472	FALSE	6.13E-19
325_s_at	0.237	1.725838	0.105259	-0.24862	0.72262	TRUE	1.00E+00
1181_at	0.23702	1.725917	0.072931	-0.09945	0.57349	TRUE	1.00E+00
38245_i_at	0.23717	1.726514	0.080829	-0.13574	0.61008	TRUE	1.00E+00
1186_at	0.23728	1.726951	0.1107	-0.27344	0.74801	TRUE	1.00E+00
32273_at	0.23759	1.728184	0.090844	-0.18152	0.65671	TRUE	1.00E+00
38875_r_at	0.2376	1.728224	0.125376	-0.34083	0.81604	TRUE	1.00E+00
1920_s_at	0.23764	1.728383	0.025716	0.119	0.35628	FALSE	3.08E-16
40522_at	0.23769	1.728582	0.043212	0.03833	0.43706	FALSE	4.78E-04
32376_at	0.23775	1.728821	0.066158	-0.06747	0.54298	TRUE	1.00E+00
2002_s_at	0.23782	1.7291	0.072286	-0.09568	0.57132	TRUE	1.00E+00
850_r_at	0.23818	1.730533	0.060767	-0.04217	0.51853	TRUE	1.00E+00
39252_at	0.2389	1.733405	0.026673	0.11584	0.36196	FALSE	4.23E-15
35318_at	0.23893	1.733525	0.040603	0.05161	0.42626	FALSE	5.04E-05
39718_r_at	0.23898	1.733724	0.031781	0.09235	0.3856	FALSE	6.94E-10
35169_at	0.23916	1.734443	0.049622	0.01022	0.46809	FALSE	1.82E-02
31529_at	0.23929	1.734962	0.080798	-0.13347	0.61206	TRUE	1.00E+00
35053_at	0.23931	1.735042	0.061697	-0.04533	0.52395	TRUE	1.00E+00
36927_at	0.23947	1.735681	0.024047	0.12853	0.35042	FALSE	2.92E-19
40574_at	0.23988	1.737321	0.076525	-0.11318	0.59293	TRUE	1.00E+00
35587_at	0.23996	1.737641	0.085721	-0.15552	0.63544	TRUE	1.00E+00
35811_at	0.24033	1.739122	0.02303	0.13408	0.34658	FALSE	2.16E-21
40570_at	0.24055	1.740003	0.031438	0.09551	0.38559	FALSE	2.51E-10
31542_at	0.24091	1.741446	0.06811	-0.07332	0.55514	TRUE	1.00E+00
34777_at	0.24092	1.741486	0.036135	0.07421	0.40763	FALSE	3.29E-07
36943_r_at	0.24098	1.741727	0.011346	0.18863	0.29332	FALSE	5.23E-96
1820_g_at	0.24117	1.742489	0.036959	0.07065	0.41168	FALSE	8.57E-07
1198_at	0.24126	1.74285	0.173086	-0.55729	1.0398	TRUE	1.00E+00

32583_at	0.24145	1.743613	0.034594	0.08185	0.40105	FALSE	3.74E-08
36859_at	0.24156	1.744054	0.083063	-0.14166	0.62478	TRUE	1.00E+00
646_s_at	0.24158	1.744135	0.035413	0.0782	0.40497	FALSE	1.13E-07
33802_at	0.24162	1.744295	0.115779	-0.29254	0.77577	TRUE	1.00E+00
36549_at	0.24188	1.74534	0.03489	0.08092	0.40285	FALSE	5.21E-08
37819_at	0.24207	1.746104	0.018301	0.15763	0.3265	FALSE	7.76E-36
35560_at	0.24236	1.74727	0.078997	-0.1221	0.60682	TRUE	1.00E+00
37561_at	0.24245	1.747632	0.018395	0.15758	0.32731	FALSE	1.45E-35
1348_s_at	0.24273	1.748759	0.018855	0.15575	0.32972	FALSE	7.98E-34
40475_at	0.24293	1.749565	0.078702	-0.12016	0.60603	TRUE	1.00E+00
33703_f_at	0.24298	1.749766	0.098592	-0.21189	0.69784	TRUE	1.00E+00
34327_at	0.2432	1.750653	0.024108	0.13197	0.35443	FALSE	7.90E-20
37816_at	0.24324	1.750814	0.065865	-0.06063	0.54712	TRUE	1.00E+00
39927_at	0.24349	1.751822	0.039682	0.06041	0.42656	FALSE	1.07E-05
1405_i_at	0.24353	1.751983	0.115933	-0.29134	0.7784	TRUE	1.00E+00
33140_at	0.24355	1.752064	0.030066	0.10484	0.38226	FALSE	6.90E-12
35601_at	0.24362	1.752347	0.060059	-0.03347	0.52071	TRUE	6.29E-01
34873_at	0.24386	1.753315	0.044673	0.03775	0.44996	FALSE	6.06E-04
40197_at	0.24386	1.753315	0.047242	0.02591	0.46182	FALSE	3.08E-03
38526_at	0.24409	1.754244	0.103446	-0.23317	0.72135	TRUE	1.00E+00
41066_at	0.24423	1.75481	0.088223	-0.1628	0.65125	TRUE	1.00E+00
37340_at	0.24433	1.755214	0.128513	-0.34857	0.83724	TRUE	1.00E+00
37865_at	0.24471	1.75675	0.069534	-0.07609	0.5655	TRUE	1.00E+00
39653_at	0.24482	1.757195	0.04245	0.04897	0.44067	FALSE	1.02E-04
2060_at	0.24485	1.757317	0.061892	-0.04069	0.53039	TRUE	9.62E-01
39591_s_at	0.24487	1.757397	0.105117	-0.2401	0.72983	TRUE	1.00E+00
38805_at	0.24501	1.757964	0.025762	0.12615	0.36387	FALSE	2.40E-17
34174_s_at	0.24507	1.758207	0.129363	-0.35175	0.8419	TRUE	1.00E+00
41248_at	0.24513	1.75845	0.0407	0.05735	0.4329	FALSE	2.16E-05
35192_at	0.24517	1.758612	0.084034	-0.14253	0.63287	TRUE	1.00E+00
38180_f_at	0.2452	1.758733	0.073221	-0.09262	0.58301	TRUE	1.00E+00
406_at	0.24543	1.759665	0.088126	-0.16115	0.652	TRUE	1.00E+00
33074_g_at	0.24561	1.760394	0.096252	-0.19846	0.68967	TRUE	1.00E+00
32309_at	0.2459	1.76157	0.072315	-0.08773	0.57953	TRUE	1.00E+00
36909_at	0.24602	1.762057	0.040211	0.0605	0.43154	FALSE	1.19E-05
39296_at	0.24628	1.763112	0.034085	0.08902	0.40353	FALSE	6.31E-09
35097_at	0.24646	1.763843	0.092425	-0.17995	0.67287	TRUE	1.00E+00
33162_at	0.24647	1.763884	0.068031	-0.0674	0.56034	TRUE	1.00E+00
32656_at	0.24657	1.76429	0.041537	0.05494	0.43821	FALSE	3.68E-05
31914_at	0.24666	1.764656	0.078587	-0.11591	0.60922	TRUE	1.00E+00
41362_at	0.24694	1.765794	0.065349	-0.05455	0.54844	TRUE	1.00E+00
36020_at	0.24707	1.766322	0.084453	-0.14256	0.63671	TRUE	1.00E+00
39292_r_at	0.24723	1.766973	0.045436	0.03761	0.45686	FALSE	6.68E-04
33825_at	0.24741	1.767706	0.068622	-0.06918	0.56401	TRUE	1.00E+00
33466_at	0.24747	1.76795	0.037332	0.07524	0.41971	FALSE	4.27E-07
40900_at	0.24749	1.768032	0.037069	0.07646	0.41851	FALSE	3.09E-07
38348_at	0.24759	1.768439	0.055472	-0.00833	0.50352	TRUE	1.02E-01
41544_at	0.24781	1.769335	0.06661	-0.0595	0.55512	TRUE	1.00E+00
36366_at	0.24788	1.76962	0.069932	-0.07476	0.57052	TRUE	1.00E+00
38101_at	0.24803	1.770231	0.036003	0.08193	0.41413	FALSE	7.08E-08
33263_at	0.24805	1.770313	0.030056	0.10938	0.38671	FALSE	1.95E-12

35133_at	0.24808	1.770435	0.056491	-0.01255	0.5087	TRUE	1.42E-01
41725_at	0.24808	1.770435	0.054304	-0.00245	0.49862	TRUE	6.20E-02
674_g_at	0.24814	1.77068	0.034982	0.08675	0.40953	FALSE	1.65E-08
34188_at	0.24814	1.77068	0.036477	0.07985	0.41643	FALSE	1.30E-07
39701_at	0.24815	1.77072	0.067354	-0.0626	0.55889	TRUE	1.00E+00
32700_at	0.24829	1.771291	0.044217	0.0443	0.45229	FALSE	2.48E-04
36039_s_at	0.24855	1.772352	0.084652	-0.14199	0.6391	TRUE	1.00E+00
40505_at	0.24874	1.773128	0.024231	0.13695	0.36053	FALSE	1.27E-20
1474_s_at	0.24894	1.773944	0.026445	0.12694	0.37095	FALSE	6.06E-17
34437_at	0.24925	1.775211	0.02484	0.13465	0.36385	FALSE	1.36E-19
34424_at	0.24933	1.775538	0.088031	-0.15681	0.65547	TRUE	1.00E+00
38630_at	0.24934	1.775579	0.027594	0.12203	0.37665	FALSE	2.05E-15
37524_at	0.24986	1.777706	0.05028	0.01789	0.48183	FALSE	8.48E-03
34932_at	0.25041	1.779959	0.092399	-0.17589	0.6767	TRUE	1.00E+00
1832_at	0.25063	1.780861	0.043667	0.04916	0.45209	FALSE	1.20E-04
34674_at	0.25072	1.78123	0.092703	-0.17697	0.67841	TRUE	1.00E+00
546_at	0.25073	1.781271	0.061444	-0.03275	0.53421	TRUE	5.67E-01
32383_at	0.25079	1.781517	0.076735	-0.10323	0.60482	TRUE	1.00E+00
34113_at	0.25086	1.781804	0.102365	-0.22141	0.72313	TRUE	1.00E+00
581_at	0.25098	1.782297	0.045067	0.04306	0.4589	FALSE	3.23E-04
690_s_at	0.25101	1.78242	0.095341	-0.18886	0.69087	TRUE	1.00E+00
266_s_at	0.25113	1.782912	0.073215	-0.08666	0.58891	TRUE	1.00E+00
33142_at	0.25119	1.783159	0.059052	-0.02125	0.52363	TRUE	2.65E-01
31480_f_at	0.25161	1.784884	0.075973	-0.09889	0.60212	TRUE	1.00E+00
38364_at	0.25197	1.786364	0.039337	0.07048	0.43346	FALSE	1.89E-06
38640_at	0.25201	1.786529	0.075027	-0.09413	0.59815	TRUE	1.00E+00
32613_at	0.25217	1.787187	0.072999	-0.08462	0.58896	TRUE	1.00E+00
32081_at	0.25305	1.790812	0.130382	-0.34848	0.85458	TRUE	1.00E+00
40621_at	0.25338	1.792173	0.026992	0.12885	0.37791	FALSE	7.77E-17
35469_at	0.25348	1.792586	0.059641	-0.02168	0.52864	TRUE	2.70E-01
35549_at	0.25374	1.793659	0.089197	-0.15778	0.66526	TRUE	1.00E+00
35716_at	0.25406	1.794982	0.135967	-0.37323	0.88136	TRUE	1.00E+00
1414_at	0.25407	1.795023	0.039916	0.06991	0.43823	FALSE	2.46E-06
38271_at	0.25416	1.795395	0.013156	0.19346	0.31485	FALSE	4.67E-79
34681_at	0.25457	1.797091	0.115567	-0.27861	0.78775	TRUE	1.00E+00
35058_at	0.25471	1.79767	0.055992	-0.00361	0.51304	TRUE	6.80E-02
36261_at	0.25474	1.797794	0.102248	-0.21699	0.72647	TRUE	1.00E+00
33036_at	0.2549	1.798457	0.132614	-0.35693	0.86673	TRUE	1.00E+00
41364_r_at	0.25493	1.798581	0.048016	0.03341	0.47645	FALSE	1.39E-03
35908_at	0.2551	1.799285	0.062328	-0.03246	0.54265	TRUE	5.38E-01
38411_at	0.25521	1.799741	0.039546	0.07276	0.43766	FALSE	1.38E-06
38615_at	0.25524	1.799865	0.098941	-0.20123	0.71171	TRUE	1.00E+00
937_at	0.25527	1.79999	0.084858	-0.13623	0.64677	TRUE	1.00E+00
40906_at	0.25548	1.80086	0.05863	-0.01502	0.52597	TRUE	1.66E-01
645_at	0.25564	1.801524	0.135501	-0.36951	0.88078	TRUE	1.00E+00
34551_at	0.25572	1.801856	0.085485	-0.13867	0.65011	TRUE	1.00E+00
865_at	0.25574	1.801939	0.027675	0.12806	0.38342	FALSE	3.09E-16
1570_f_at	0.25595	1.80281	0.144713	-0.4117	0.92359	TRUE	1.00E+00
31814_i_at	0.25608	1.80335	0.037491	0.08312	0.42905	FALSE	1.07E-07
1600_at	0.25618	1.803765	0.115281	-0.27568	0.78804	TRUE	1.00E+00
36595_s_at	0.25637	1.804554	0.1401	-0.38999	0.90273	TRUE	1.00E+00

39846_at	0.25642	1.804762	0.023792	0.14665	0.36618	FALSE	5.55E-23
37486_f_at	0.25667	1.805801	0.023242	0.14944	0.3639	FALSE	2.99E-24
595_at	0.25668	1.805843	0.080677	-0.11553	0.6289	TRUE	1.00E+00
33309_at	0.25692	1.806841	0.038093	0.08117	0.43266	FALSE	1.94E-07
40813_at	0.25697	1.807049	0.045525	0.04694	0.46701	FALSE	2.09E-04
38177_at	0.25718	1.807923	0.061425	-0.02621	0.54056	TRUE	3.57E-01
40671_g_at	0.25731	1.808465	0.079767	-0.1107	0.62533	TRUE	1.00E+00
35460_at	0.25759	1.809631	0.132403	-0.35326	0.86845	TRUE	1.00E+00
35609_at	0.25804	1.811507	0.180327	-0.57392	1.08999	TRUE	1.00E+00
32142_at	0.25812	1.811841	0.03329	0.10453	0.4117	FALSE	1.13E-10
37094_at	0.25815	1.811966	0.056713	-0.0035	0.5198	TRUE	6.71E-02
39284_at	0.25855	1.813635	0.060263	-0.01948	0.53658	TRUE	2.25E-01
1599_at	0.25872	1.814346	0.133462	-0.35702	0.87447	TRUE	1.00E+00
32838_at	0.25873	1.814387	0.034779	0.09827	0.41918	FALSE	1.28E-09
36059_at	0.25886	1.814931	0.057833	-0.00796	0.52568	TRUE	9.60E-02
38789_at	0.25918	1.816268	0.042318	0.06394	0.45441	FALSE	1.15E-05
31470_at	0.25922	1.816436	0.093491	-0.17211	0.69055	TRUE	1.00E+00
38246_r_at	0.25939	1.817147	0.123429	-0.31006	0.82884	TRUE	1.00E+00
38837_at	0.2596	1.818026	0.03934	0.0781	0.4411	FALSE	5.23E-07
35081_at	0.25976	1.818696	0.097661	-0.1908	0.71033	TRUE	1.00E+00
32255_i_at	0.26001	1.819743	0.05205	0.01988	0.50015	FALSE	7.41E-03
36844_at	0.26002	1.819785	0.058963	-0.01201	0.53205	TRUE	1.31E-01
626_s_at	0.26018	1.820455	0.044031	0.05704	0.46332	FALSE	4.34E-05
31752_at	0.26036	1.82121	0.05105	0.02483	0.49588	FALSE	4.29E-03
39111_s_at	0.26049	1.821755	0.066425	-0.04597	0.56695	TRUE	1.00E+00
40399_r_at	0.26053	1.821923	0.057226	-0.00349	0.52455	TRUE	6.69E-02
41769_at	0.26103	1.824022	0.092666	-0.1665	0.68855	TRUE	1.00E+00
1915_s_at	0.26106	1.824148	0.065147	-0.0395	0.56162	TRUE	7.76E-01
860_at	0.26127	1.82503	0.029012	0.12743	0.39512	FALSE	2.70E-15
41844_at	0.26174	1.827006	0.094397	-0.17377	0.69725	TRUE	1.00E+00
36029_at	0.26179	1.827216	0.091132	-0.15866	0.68223	TRUE	1.00E+00
38030_at	0.26201	1.828142	0.096345	-0.18249	0.70651	TRUE	1.00E+00
38938_at	0.26235	1.829574	0.08543	-0.13179	0.65649	TRUE	1.00E+00
41856_at	0.26246	1.830038	0.048783	0.0374	0.48753	FALSE	9.40E-04
625_at	0.26248	1.830122	0.031575	0.11681	0.40816	FALSE	1.18E-12
1971_g_at	0.26303	1.832441	0.078311	-0.09827	0.62432	TRUE	1.00E+00
1741_s_at	0.26354	1.834594	0.060035	-0.01343	0.54052	TRUE	1.43E-01
32551_at	0.26387	1.835989	0.085524	-0.1307	0.65844	TRUE	1.00E+00
33318_at	0.2639	1.836116	0.076146	-0.0874	0.61521	TRUE	1.00E+00
741_g_at	0.26397	1.836411	0.060004	-0.01287	0.5408	TRUE	1.37E-01
33436_at	0.26431	1.83785	0.026941	0.14002	0.38861	FALSE	1.28E-18
39621_at	0.26431	1.83785	0.034544	0.10494	0.42368	FALSE	2.51E-10
39243_s_at	0.26447	1.838527	0.053964	0.0155	0.51344	FALSE	1.21E-02
40251_at	0.26483	1.840052	0.105832	-0.22344	0.7531	TRUE	1.00E+00
1011_s_at	0.26489	1.840306	0.06759	-0.04694	0.57672	TRUE	1.00E+00
34660_at	0.26515	1.841408	0.109875	-0.24176	0.77207	TRUE	1.00E+00
33055_at	0.26519	1.841577	0.087554	-0.13875	0.66913	TRUE	1.00E+00
37441_at	0.26525	1.841832	0.032135	0.11699	0.41351	FALSE	1.93E-12
34513_at	0.26545	1.84268	0.064157	-0.03055	0.56144	TRUE	4.43E-01
40478_at	0.26551	1.842935	0.032622	0.11501	0.41601	FALSE	5.03E-12
41865_at	0.26552	1.842977	0.068897	-0.05234	0.58339	TRUE	1.00E+00

34378_at	0.26557	1.84319	0.013465	0.20345	0.32769	FALSE	1.73E-82
35419_g_at	0.26558	1.843232	0.036207	0.09854	0.43262	FALSE	2.80E-09
37479_at	0.26676	1.848247	0.087799	-0.13831	0.67183	TRUE	1.00E+00
332_at	0.26682	1.848502	0.073203	-0.07091	0.60455	TRUE	1.00E+00
1452_at	0.26686	1.848673	0.037005	0.09613	0.43758	FALSE	6.99E-09
37824_at	0.26688	1.848758	0.0444	0.06203	0.47172	FALSE	2.33E-05
31730_at	0.26696	1.849098	0.07505	-0.07929	0.61321	TRUE	1.00E+00
33032_r_at	0.26707	1.849567	0.092672	-0.16049	0.69462	TRUE	1.00E+00
1147_at	0.26714	1.849865	0.039993	0.08263	0.45165	FALSE	3.03E-07
31982_at	0.2674	1.850973	0.047534	0.0481	0.48671	FALSE	2.33E-04
36149_at	0.26763	1.851953	0.046838	0.05154	0.48372	FALSE	1.39E-04
39496_s_at	0.26841	1.855282	0.074945	-0.07736	0.61418	TRUE	1.00E+00
41378_at	0.26845	1.855453	0.063916	-0.02643	0.56333	TRUE	3.37E-01
34445_at	0.2685	1.855667	0.031772	0.12191	0.41508	FALSE	3.66E-13
35614_at	0.26877	1.856821	0.035242	0.10617	0.43136	FALSE	3.05E-10
40527_at	0.2688	1.856949	0.045136	0.06057	0.47704	FALSE	3.27E-05
36502_at	0.26889	1.857334	0.03365	0.11364	0.42414	FALSE	1.69E-11
1350_at	0.26911	1.858275	0.046996	0.05229	0.48593	FALSE	1.30E-04
39572_at	0.26915	1.858446	0.103613	-0.20888	0.74718	TRUE	1.00E+00
1349_at	0.26943	1.859645	0.063868	-0.02524	0.56409	TRUE	3.11E-01
659_g_at	0.26964	1.860544	0.04492	0.0624	0.47688	FALSE	2.45E-05
40352_at	0.26985	1.861444	0.098738	-0.18568	0.72539	TRUE	1.00E+00
37884_f_at	0.26988	1.861573	0.04703	0.0529	0.48686	FALSE	1.21E-04
38952_s_at	0.27011	1.862559	0.105099	-0.21477	0.75499	TRUE	1.00E+00
37599_at	0.27013	1.862645	0.076019	-0.08059	0.62085	TRUE	1.00E+00
38850_at	0.27036	1.863631	0.0823	-0.10934	0.65005	TRUE	1.00E+00
38247_at	0.27038	1.863717	0.121791	-0.29151	0.83228	TRUE	1.00E+00
40274_at	0.27078	1.865434	0.085601	-0.12415	0.66571	TRUE	1.00E+00
33536_at	0.27089	1.865907	0.1072	-0.22369	0.76546	TRUE	1.00E+00
36768_at	0.27116	1.867067	0.114028	-0.25492	0.79724	TRUE	1.00E+00
40285_at	0.2713	1.867669	0.086416	-0.12739	0.66999	TRUE	1.00E+00
35776_at	0.27133	1.867798	0.020223	0.17804	0.36463	FALSE	6.04E-37
37593_at	0.27163	1.869089	0.048687	0.04701	0.49625	FALSE	3.05E-04
39719_at	0.27176	1.869649	0.088177	-0.13505	0.67857	TRUE	1.00E+00
37286_at	0.27188	1.870165	0.111172	-0.24102	0.78478	TRUE	1.00E+00
41523_at	0.27203	1.870811	0.037639	0.09838	0.44568	FALSE	6.22E-09
32714_s_at	0.27214	1.871285	0.066426	-0.03433	0.5786	TRUE	5.29E-01
affx-humgapdh/m	0.27227	1.871846	0.118844	-0.27602	0.82057	TRUE	1.00E+00
41857_r_at	0.27234	1.872147	0.160804	-0.46955	1.01422	TRUE	1.00E+00
35047_at	0.27256	1.873096	0.0828	-0.10944	0.65457	TRUE	1.00E+00
36010_at	0.27261	1.873312	0.057214	0.00865	0.53657	FALSE	2.39E-02
1461_at	0.27279	1.874088	0.032862	0.12117	0.4244	FALSE	1.30E-12
41146_at	0.27286	1.87439	0.026267	0.15168	0.39405	FALSE	3.55E-21
32317_s_at	0.27287	1.874433	0.03405	0.11577	0.42996	FALSE	1.41E-11
39408_at	0.27343	1.876852	0.048398	0.05014	0.49672	FALSE	2.03E-04
36672_at	0.2735	1.877154	0.02107	0.17629	0.3707	FALSE	1.99E-34
41461_at	0.27356	1.877414	0.041091	0.08399	0.46314	FALSE	3.52E-07
321_at	0.27358	1.8775	0.073808	-0.06694	0.6141	TRUE	1.00E+00
35890_at	0.27366	1.877846	0.098464	-0.18061	0.72794	TRUE	1.00E+00
35548_at	0.27376	1.878279	0.143709	-0.38925	0.93677	TRUE	1.00E+00
37005_at	0.27434	1.880789	0.04542	0.06479	0.48389	FALSE	1.94E-05

37724_at	0.27435	1.880832	0.02638	0.15265	0.39606	FALSE	3.13E-21
affx-lysx-m_at	0.27455	1.881698	0.791082	-3.37518	3.92427	TRUE	1.00E+00
31883_at	0.27461	1.881958	0.038515	0.09692	0.4523	FALSE	1.27E-08
40707_at	0.27539	1.885341	0.120269	-0.27948	0.83026	TRUE	1.00E+00
32502_at	0.27589	1.887513	0.037265	0.10396	0.44781	FALSE	1.68E-09
234_s_at	0.276	1.887991	0.097463	-0.17365	0.72566	TRUE	1.00E+00
33461_at	0.27617	1.888731	0.108179	-0.22293	0.77526	TRUE	1.00E+00
1465_s_at	0.27626	1.889122	0.108284	-0.22332	0.77584	TRUE	1.00E+00
32906_at	0.27652	1.890253	0.12119	-0.2826	0.83564	TRUE	1.00E+00
36744_at	0.27675	1.891255	0.059708	0.00129	0.55222	FALSE	4.50E-02
32246_g_at	0.27699	1.8923	0.109997	-0.2305	0.78447	TRUE	1.00E+00
1324_at	0.27709	1.892736	0.084729	-0.11382	0.66799	TRUE	1.00E+00
32945_i_at	0.2771	1.892779	0.171263	-0.51303	1.06724	TRUE	1.00E+00
31916_at	0.27788	1.896182	0.074832	-0.06737	0.62312	TRUE	1.00E+00
36382_at	0.27801	1.89675	0.131445	-0.32842	0.88445	TRUE	1.00E+00
31378_at	0.27803	1.896837	0.073195	-0.05966	0.61573	TRUE	1.00E+00
39766_r_at	0.27807	1.897012	0.047262	0.06002	0.49611	FALSE	5.07E-05
41416_at	0.27816	1.897405	0.106012	-0.21094	0.76725	TRUE	1.00E+00
41145_at	0.27872	1.899853	0.056502	0.01805	0.5394	FALSE	1.02E-02
37215_at	0.27898	1.900991	0.034774	0.11855	0.43942	FALSE	1.31E-11
36770_at	0.27901	1.901122	0.027706	0.15119	0.40684	FALSE	9.43E-20
41179_at	0.27931	1.902436	0.050125	0.04805	0.51056	FALSE	3.17E-04
31541_at	0.27976	1.904408	0.104696	-0.20327	0.76278	TRUE	1.00E+00
34841_at	0.28002	1.905548	0.031047	0.13679	0.42326	FALSE	2.39E-15
33196_at	0.28008	1.905812	0.090483	-0.13737	0.69753	TRUE	1.00E+00
40750_at	0.28014	1.906075	0.065531	-0.02219	0.58248	TRUE	2.41E-01
37009_at	0.28075	1.908754	0.032312	0.13168	0.42983	FALSE	4.62E-14
39015_f_at	0.28093	1.909545	0.05299	0.03645	0.5254	FALSE	1.45E-03
39026_r_at	0.28095	1.909633	0.047439	0.06208	0.49981	FALSE	4.01E-05
38906_at	0.28102	1.909941	0.104946	-0.20316	0.76519	TRUE	1.00E+00
31382_f_at	0.2812	1.910733	0.095102	-0.15756	0.71996	TRUE	1.00E+00
39301_at	0.28129	1.911129	0.069076	-0.0374	0.59997	TRUE	5.88E-01
41543_at	0.28197	1.914124	0.079095	-0.08294	0.64688	TRUE	1.00E+00
40766_at	0.28204	1.914432	0.047441	0.06317	0.50092	FALSE	3.49E-05
40923_at	0.28212	1.914785	0.026638	0.15923	0.40502	FALSE	4.14E-22
974_at	0.28225	1.915358	0.066444	-0.0243	0.5888	TRUE	2.72E-01
40632_at	0.28248	1.916373	0.086034	-0.11445	0.6794	TRUE	1.00E+00
40516_at	0.28271	1.917388	0.029017	0.14884	0.41658	FALSE	2.50E-18
37406_at	0.28293	1.91836	0.046779	0.06711	0.49874	FALSE	1.85E-05
1616_at	0.28332	1.920083	0.059812	0.00737	0.55927	FALSE	2.74E-02
34601_at	0.28348	1.920791	0.072848	-0.05262	0.61957	TRUE	1.00E+00
34125_at	0.28356	1.921144	0.065219	-0.01734	0.58445	TRUE	1.74E-01
40311_at	0.28385	1.922428	0.061058	0.00216	0.56554	FALSE	4.21E-02
31630_at	0.28404	1.923269	0.043313	0.08421	0.48387	FALSE	6.89E-07
1118_at	0.28459	1.925706	0.028427	0.15344	0.41573	FALSE	1.72E-19
31924_at	0.28517	1.92828	0.057574	0.01955	0.5508	FALSE	9.22E-03
34954_r_at	0.28572	1.930723	0.044859	0.07876	0.49268	FALSE	2.40E-06
35905_s_at	0.28596	1.93179	0.071566	-0.04422	0.61613	TRUE	8.14E-01
32555_at	0.28596	1.93179	0.062609	-0.00289	0.57481	TRUE	6.23E-02
35491_at	0.28608	1.932324	0.061683	0.0015	0.57066	FALSE	4.44E-02
40034_r_at	0.2861	1.932413	0.100486	-0.1775	0.7497	TRUE	1.00E+00

37183_at	0.28617	1.932725	0.065912	-0.01792	0.59026	TRUE	1.79E-01
577_at	0.2865	1.934194	0.064009	-0.00881	0.58181	TRUE	9.61E-02
37540_at	0.28668	1.934996	0.107499	-0.20927	0.78264	TRUE	1.00E+00
33246_at	0.2868	1.93553	0.091538	-0.13552	0.70911	TRUE	1.00E+00
37211_at	0.28683	1.935664	0.083055	-0.09635	0.67002	TRUE	1.00E+00
33573_at	0.28695	1.936199	0.101792	-0.18268	0.75658	TRUE	1.00E+00
36275_at	0.28721	1.937359	0.088864	-0.12278	0.69719	TRUE	1.00E+00
35709_at	0.28729	1.937715	0.014996	0.2181	0.35647	FALSE	1.06E-77
34935_at	0.28731	1.937805	0.098419	-0.16675	0.74138	TRUE	1.00E+00
32119_at	0.28786	1.94026	0.029592	0.15134	0.42438	FALSE	2.90E-18
40397_at	0.2879	1.940439	0.129436	-0.30926	0.88507	TRUE	1.00E+00
31770_at	0.28793	1.940573	0.037968	0.11277	0.4631	FALSE	4.24E-10
39941_at	0.28846	1.942943	0.067619	-0.02351	0.60043	TRUE	2.51E-01
40458_at	0.28846	1.942943	0.054116	0.0388	0.53813	FALSE	1.24E-03
39066_at	0.28855	1.943345	0.076831	-0.06592	0.64301	TRUE	1.00E+00
874_at	0.28881	1.944509	0.063683	-0.005	0.58261	TRUE	7.27E-02
41321_s_at	0.28881	1.944509	0.053044	0.04409	0.53353	FALSE	6.55E-04
34750_r_at	0.2893	1.946704	0.085016	-0.10292	0.68153	TRUE	1.00E+00
35461_at	0.28945	1.947377	0.072251	-0.04389	0.62278	TRUE	7.79E-01
2015_s_at	0.28955	1.947825	0.030176	0.15033	0.42877	FALSE	1.05E-17
37398_at	0.28968	1.948408	0.087182	-0.11254	0.6919	TRUE	1.00E+00
40665_at	0.28974	1.948678	0.084626	-0.10069	0.68018	TRUE	1.00E+00
35532_i_at	0.28977	1.948812	0.071887	-0.04189	0.62143	TRUE	7.01E-01
41419_at	0.29025	1.950967	0.028827	0.15725	0.42324	FALSE	9.60E-20
32385_at	0.29063	1.952675	0.146684	-0.38611	0.96737	TRUE	1.00E+00
37646_at	0.29148	1.956501	0.032321	0.14236	0.44059	FALSE	2.41E-15
31854_at	0.29159	1.956996	0.026885	0.16755	0.41562	FALSE	2.64E-23
38211_at	0.29173	1.957627	0.033177	0.13866	0.44479	FALSE	1.84E-14
34001_at	0.29194	1.958574	0.142354	-0.36482	0.9487	TRUE	1.00E+00
300_f_at	0.29265	1.961779	0.040091	0.10768	0.47761	FALSE	3.65E-09
1143_s_at	0.29277	1.962321	0.080616	-0.07916	0.6647	TRUE	1.00E+00
34015_at	0.29278	1.962366	0.075561	-0.05582	0.64139	TRUE	1.00E+00
36736_f_at	0.29355	1.965848	0.044649	0.08755	0.49954	FALSE	6.16E-07
37806_at	0.2936	1.966075	0.090926	-0.12589	0.7131	TRUE	1.00E+00
38636_at	0.29391	1.967479	0.075998	-0.05672	0.64453	TRUE	1.00E+00
38864_at	0.29402	1.967977	0.043494	0.09336	0.49469	FALSE	1.74E-07
39542_at	0.29404	1.968068	0.065531	-0.00829	0.59638	TRUE	9.12E-02
31926_at	0.29434	1.969428	0.118989	-0.25463	0.84331	TRUE	1.00E+00
37716_at	0.2944	1.9697	0.041053	0.105	0.4838	FALSE	9.39E-09
34121_at	0.29474	1.971242	0.089206	-0.11682	0.7063	TRUE	1.00E+00
35331_at	0.29492	1.972059	0.025151	0.17888	0.41095	FALSE	1.18E-27
38868_at	0.29532	1.973877	0.052093	0.05498	0.53565	FALSE	1.81E-04
38575_at	0.29535	1.974013	0.024935	0.18031	0.41039	FALSE	2.89E-28
1290_g_at	0.29552	1.974786	0.103133	-0.1803	0.77133	TRUE	1.00E+00
34716_at	0.29565	1.975377	0.03819	0.11946	0.47185	FALSE	1.24E-10
464_s_at	0.29569	1.975559	0.020513	0.20105	0.39033	FALSE	5.29E-43
1887_g_at	0.29594	1.976697	0.09861	-0.159	0.75089	TRUE	1.00E+00
40017_at	0.29607	1.977288	0.049035	0.06984	0.52229	FALSE	1.97E-05
32814_at	0.29607	1.977288	0.023752	0.18649	0.40565	FALSE	1.46E-31
33849_at	0.2963	1.978336	0.023541	0.18769	0.40491	FALSE	3.16E-32
495_at	0.29639	1.978746	0.052461	0.05436	0.53842	FALSE	2.03E-04

37067_at	0.29644	1.978974	0.042961	0.09823	0.49464	FALSE	6.56E-08
37883_i_at	0.29652	1.979338	0.045167	0.08813	0.5049	FALSE	6.58E-07
39085_at	0.29655	1.979475	0.09356	-0.1351	0.72819	TRUE	1.00E+00
34057_at	0.29671	1.980204	0.087016	-0.10475	0.69816	TRUE	1.00E+00
31388_at	0.29681	1.98066	0.114888	-0.23324	0.82686	TRUE	1.00E+00
684_at	0.29697	1.98139	0.080384	-0.07389	0.66783	TRUE	1.00E+00
33488_at	0.29714	1.982166	0.058375	0.02783	0.56646	FALSE	4.51E-03
1623_s_at	0.29717	1.982303	0.064521	-0.00051	0.59484	TRUE	5.19E-02
34904_at	0.29748	1.983718	0.08247	-0.083	0.67796	TRUE	1.00E+00
38220_at	0.29751	1.983855	0.055929	0.03947	0.55554	FALSE	1.32E-03
41824_at	0.29768	1.984632	0.015163	0.22772	0.36763	FALSE	1.03E-81
31485_at	0.29773	1.984861	0.058779	0.02655	0.56891	FALSE	5.15E-03
40652_at	0.29846	1.9882	0.09189	-0.12548	0.72241	TRUE	1.00E+00
618_at	0.29855	1.988612	0.179723	-0.53062	1.12771	TRUE	1.00E+00
34265_at	0.29883	1.989894	0.04611	0.08609	0.51156	FALSE	1.15E-06
35210_at	0.2991	1.991132	0.095116	-0.13973	0.73793	TRUE	1.00E+00
32768_at	0.29913	1.991269	0.066013	-0.00543	0.60369	TRUE	7.40E-02
33505_at	0.29934	1.992232	0.148505	-0.3858	0.98448	TRUE	1.00E+00
41137_at	0.29936	1.992324	0.05477	0.04667	0.55204	FALSE	5.82E-04
1731_at	0.29947	1.992829	0.055583	0.04304	0.55591	FALSE	9.00E-04
32163_f_at	0.2998	1.994344	0.077236	-0.05654	0.65613	TRUE	1.00E+00
38228_g_at	0.30009	1.995676	0.056362	0.04006	0.56012	FALSE	1.28E-03
36249_at	0.30033	1.996779	0.096014	-0.14264	0.7433	TRUE	1.00E+00
32614_at	0.30159	2.002581	0.062772	0.01199	0.5912	FALSE	1.96E-02
31648_at	0.30197	2.004334	0.067046	-0.00735	0.6113	TRUE	8.42E-02
2086_s_at	0.30205	2.004703	0.045043	0.09424	0.50986	FALSE	2.53E-07
35708_at	0.30229	2.005811	0.116332	-0.23442	0.83899	TRUE	1.00E+00
1425_at	0.30253	2.00692	0.062466	0.01434	0.59073	FALSE	1.61E-02
39775_at	0.30294	2.008815	0.044314	0.0985	0.50739	FALSE	1.03E-07
32096_at	0.30308	2.009463	0.070906	-0.02405	0.63021	TRUE	2.42E-01
39950_at	0.3035	2.011407	0.051483	0.06598	0.54102	FALSE	4.72E-05
39151_at	0.30389	2.013214	0.09989	-0.15696	0.76474	TRUE	1.00E+00
35085_r_at	0.30458	2.016415	0.102964	-0.17045	0.77962	TRUE	1.00E+00
31871_r_at	0.30481	2.017484	0.086268	-0.0932	0.70281	TRUE	1.00E+00
37686_s_at	0.30539	2.02018	0.0361	0.13884	0.47194	FALSE	3.39E-13
39788_at	0.30546	2.020505	0.047548	0.0861	0.52483	FALSE	1.67E-06
31954_f_at	0.30656	2.025629	0.079463	-0.06005	0.67317	TRUE	1.00E+00
1960_at	0.30672	2.026376	0.099901	-0.15418	0.76762	TRUE	1.00E+00
32630_f_at	0.30676	2.026562	0.088887	-0.10333	0.71685	TRUE	1.00E+00
40856_at	0.30695	2.027449	0.035462	0.14334	0.47056	FALSE	6.19E-14
35029_at	0.30731	2.029131	0.114259	-0.21983	0.83446	TRUE	1.00E+00
32348_at	0.30759	2.030439	0.139167	-0.33447	0.94965	TRUE	1.00E+00
36590_at	0.3077	2.030954	0.094272	-0.12723	0.74263	TRUE	1.00E+00
41424_at	0.3078	2.031421	0.066283	0.002	0.6136	FALSE	4.32E-02
1145_g_at	0.30791	2.031936	0.093322	-0.12264	0.73846	TRUE	1.00E+00
35488_at	0.30795	2.032123	0.032009	0.16027	0.45563	FALSE	8.26E-18
37973_at	0.30886	2.036386	0.069652	-0.01249	0.6302	TRUE	1.17E-01
39995_s_at	0.30886	2.036386	0.040179	0.1235	0.49423	FALSE	1.90E-10
1368_at	0.31004	2.041926	0.063846	0.01548	0.60459	FALSE	1.51E-02
35109_at	0.31005	2.041973	0.090446	-0.10723	0.72733	TRUE	1.00E+00
39135_at	0.31042	2.043713	0.030989	0.16745	0.45339	FALSE	1.62E-19

32533_s_at	0.31043	2.04376	0.037856	0.13578	0.48508	FALSE	3.03E-12
36817_at	0.31071	2.045079	0.108525	-0.18998	0.8114	TRUE	1.00E+00
33730_at	0.31082	2.045597	0.037003	0.1401	0.48154	FALSE	5.65E-13
35704_at	0.31153	2.048944	0.03919	0.13072	0.49233	FALSE	2.37E-11
36752_at	0.31161	2.049321	0.081467	-0.06424	0.68747	TRUE	1.00E+00
33719_at	0.31194	2.050879	0.068037	-0.00195	0.62584	TRUE	5.73E-02
35964_at	0.31227	2.052438	0.044225	0.10824	0.51631	FALSE	2.09E-08
36068_at	0.31258	2.053903	0.074139	-0.02946	0.65463	TRUE	3.14E-01
34968_at	0.31293	2.055559	0.034252	0.15491	0.47096	FALSE	8.16E-16
34843_at	0.31305	2.056127	0.067736	0.00054	0.62555	FALSE	4.81E-02
37280_at	0.31332	2.057406	0.026775	0.18979	0.43685	FALSE	1.57E-27
1267_at	0.31339	2.057738	0.127789	-0.27617	0.90296	TRUE	1.00E+00
39362_r_at	0.31344	2.057975	0.075842	-0.03646	0.66334	TRUE	4.52E-01
39205_at	0.31347	2.058117	0.057783	0.04688	0.58005	FALSE	7.32E-04
32775_r_at	0.31396	2.06044	0.025185	0.19776	0.43015	FALSE	1.45E-31
35031_r_at	0.31404	2.06082	0.14481	-0.35406	0.98213	TRUE	1.00E+00
36344_at	0.31405	2.060867	0.055297	0.05893	0.56917	FALSE	1.71E-04
597_at	0.31418	2.061484	0.128176	-0.27717	0.90554	TRUE	1.00E+00
34123_at	0.31437	2.062386	0.081202	-0.06026	0.689	TRUE	1.00E+00
31431_at	0.31462	2.063574	0.032011	0.16694	0.46231	FALSE	1.07E-18
35418_at	0.31488	2.06481	0.08272	-0.06675	0.69652	TRUE	1.00E+00
31517_f_at	0.31496	2.06519	0.057064	0.05169	0.57823	FALSE	4.30E-04
1717_s_at	0.31505	2.065618	0.051483	0.07752	0.55257	FALSE	1.19E-05
34227_i_at	0.31527	2.066665	0.11424	-0.21178	0.84233	TRUE	1.00E+00
41389_s_at	0.31605	2.07038	0.095141	-0.12289	0.75499	TRUE	1.00E+00
35640_at	0.31611	2.070666	0.112693	-0.20381	0.83603	TRUE	1.00E+00
33936_at	0.31625	2.071333	0.025178	0.20009	0.43241	FALSE	4.39E-32
36413_at	0.31635	2.07181	0.090985	-0.10342	0.73612	TRUE	1.00E+00
36866_at	0.31643	2.072192	0.079828	-0.05187	0.68472	TRUE	9.31E-01
37503_at	0.31651	2.072574	0.166481	-0.45156	1.08459	TRUE	1.00E+00
37604_at	0.31719	2.075821	0.128168	-0.27413	0.9085	TRUE	1.00E+00
35785_at	0.31745	2.077065	0.09552	-0.12324	0.75814	TRUE	1.00E+00
36411_s_at	0.31762	2.077878	0.120032	-0.23615	0.8714	TRUE	1.00E+00
41405_at	0.31766	2.078069	0.078014	-0.04227	0.67758	TRUE	5.89E-01
35856_r_at	0.31808	2.08008	0.098694	-0.13726	0.77341	TRUE	1.00E+00
39320_at	0.31838	2.081517	0.086143	-0.07905	0.7158	TRUE	1.00E+00
35246_at	0.31847	2.081949	0.045906	0.10668	0.53026	FALSE	5.04E-08
37265_at	0.31875	2.083291	0.136505	-0.31103	0.94853	TRUE	1.00E+00
34951_at	0.31889	2.083963	0.112407	-0.19971	0.83749	TRUE	1.00E+00
38578_at	0.31935	2.086171	0.063277	0.02741	0.61129	FALSE	5.67E-03
37571_at	0.31995	2.089056	0.046197	0.10682	0.53309	FALSE	5.47E-08
33548_f_at	0.32032	2.090836	0.169939	-0.46371	1.10435	TRUE	1.00E+00
34982_at	0.32093	2.093775	0.113463	-0.20254	0.8444	TRUE	1.00E+00
33598_r_at	0.32155	2.096766	0.10968	-0.18447	0.82757	TRUE	1.00E+00
1410_at	0.32188	2.09836	0.074499	-0.02183	0.66559	TRUE	1.96E-01
36254_at	0.32267	2.10218	0.22698	-0.72453	1.36986	TRUE	1.00E+00
1708_at	0.3227	2.102326	0.060777	0.0423	0.6031	FALSE	1.39E-03
41414_at	0.32334	2.105426	0.088105	-0.08314	0.72982	TRUE	1.00E+00
33813_at	0.32335	2.105475	0.056486	0.06275	0.58395	FALSE	1.31E-04
33618_at	0.32341	2.105765	0.092774	-0.10461	0.75143	TRUE	1.00E+00
39156_at	0.32344	2.105911	0.074708	-0.02124	0.66811	TRUE	1.89E-01

36381_at	0.32366	2.106978	0.061417	0.04031	0.60701	FALSE	1.72E-03
35900_at	0.32376	2.107463	0.113204	-0.19851	0.84604	TRUE	1.00E+00
37322_s_at	0.32378	2.10756	0.159714	-0.41308	1.06063	TRUE	1.00E+00
33440_at	0.32382	2.107754	0.032334	0.17465	0.473	FALSE	1.66E-19
35333_r_at	0.32399	2.10858	0.137213	-0.30905	0.95703	TRUE	1.00E+00
41537_r_at	0.32408	2.109017	0.043037	0.12552	0.52263	FALSE	6.39E-10
37847_at	0.32411	2.109162	0.051044	0.08861	0.5596	FALSE	2.73E-06
41412_at	0.32413	2.109259	0.101673	-0.14495	0.79321	TRUE	1.00E+00
37014_at	0.32475	2.112273	0.049219	0.09767	0.55182	FALSE	5.26E-07
40674_s_at	0.32475	2.112273	0.084819	-0.06656	0.71607	TRUE	1.00E+00
39760_at	0.32481	2.112565	0.03779	0.15046	0.49916	FALSE	1.05E-13
40081_at	0.32549	2.115875	0.03511	0.16351	0.48747	FALSE	2.34E-16
33869_at	0.3255	2.115924	0.02022	0.23221	0.41878	FALSE	3.33E-54
39309_at	0.32552	2.116021	0.075306	-0.02191	0.67295	TRUE	1.95E-01
32245_at	0.32598	2.118264	0.127317	-0.26141	0.91337	TRUE	1.00E+00
33675_at	0.32639	2.120264	0.092131	-0.09866	0.75144	TRUE	1.00E+00
1058_at	0.3269	2.122756	0.076617	-0.02658	0.68038	TRUE	2.50E-01
32527_at	0.32694	2.122951	0.180241	-0.50462	1.1585	TRUE	1.00E+00
31461_at	0.32706	2.123538	0.094555	-0.10918	0.76329	TRUE	1.00E+00
35248_at	0.32735	2.124956	0.090335	-0.08942	0.74412	TRUE	1.00E+00
299_i_at	0.32737	2.125054	0.092814	-0.10084	0.75557	TRUE	1.00E+00
37051_at	0.32771	2.126718	0.09575	-0.11404	0.76946	TRUE	1.00E+00
36800_at	0.32797	2.127992	0.113539	-0.19585	0.85179	TRUE	1.00E+00
32469_at	0.32806	2.128433	0.107363	-0.16727	0.82338	TRUE	1.00E+00
36916_at	0.32819	2.12907	0.072808	-0.00771	0.6641	TRUE	8.27E-02
36769_at	0.32865	2.131327	0.079209	-0.03679	0.69409	TRUE	4.21E-01
39356_at	0.32875	2.131817	0.062129	0.04212	0.61539	FALSE	1.53E-03
38887_r_at	0.32892	2.132652	0.082144	-0.05006	0.70791	TRUE	7.86E-01
33582_s_at	0.32909	2.133487	0.059791	0.05324	0.60494	FALSE	4.69E-04
34276_at	0.32943	2.135158	0.033392	0.17537	0.48349	FALSE	7.43E-19
38366_g_at	0.32965	2.13624	0.084509	-0.06024	0.71953	TRUE	1.00E+00
39279_at	0.32968	2.136387	0.098126	-0.12303	0.78239	TRUE	1.00E+00
39850_at	0.32993	2.137618	0.035453	0.16637	0.4935	FALSE	1.67E-16
36719_r_at	0.32996	2.137765	0.062502	0.0416	0.61832	FALSE	1.64E-03
37628_at	0.33005	2.138208	0.107084	-0.16399	0.8241	TRUE	1.00E+00
34243_i_at	0.33055	2.140671	0.035396	0.16725	0.49385	FALSE	1.23E-16
39585_at	0.33076	2.141707	0.104073	-0.14939	0.81091	TRUE	1.00E+00
35402_at	0.33091	2.142447	0.061208	0.04852	0.6133	FALSE	8.12E-04
41493_at	0.33099	2.142841	0.101749	-0.13844	0.80042	TRUE	1.00E+00
34987_s_at	0.33101	2.14294	0.030615	0.18976	0.47226	FALSE	3.82E-23
31797_at	0.33104	2.143088	0.042431	0.13528	0.5268	FALSE	7.70E-11
39985_r_at	0.33159	2.145804	0.168229	-0.44455	1.10773	TRUE	1.00E+00
38315_at	0.33173	2.146496	0.045035	0.12396	0.53951	FALSE	2.22E-09
37630_at	0.33181	2.146891	0.10355	-0.14593	0.80954	TRUE	1.00E+00
34293_at	0.33196	2.147633	0.061765	0.047	0.61692	FALSE	9.69E-04
34228_r_at	0.33217	2.148671	0.151927	-0.36876	1.0331	TRUE	1.00E+00
34141_at	0.33236	2.149612	0.073543	-0.00694	0.67166	TRUE	7.83E-02
160_at	0.3324	2.14981	0.02811	0.20271	0.46208	FALSE	3.66E-28
32417_at	0.33268	2.151196	0.103131	-0.14312	0.80849	TRUE	1.00E+00
36481_at	0.33317	2.153625	0.117846	-0.21053	0.87686	TRUE	1.00E+00
32059_at	0.33332	2.154369	0.035657	0.16881	0.49783	FALSE	1.13E-16

33974_at	0.3335	2.155262	0.080947	-0.03996	0.70696	TRUE	4.78E-01
38458_at	0.33356	2.155559	0.016519	0.25735	0.40977	FALSE	1.43E-86
38874_s_at	0.3336	2.155758	0.081869	-0.04411	0.71132	TRUE	5.81E-01
33836_at	0.33367	2.156105	0.042751	0.13644	0.53091	FALSE	7.51E-11
376_at	0.33411	2.158291	0.048766	0.10913	0.55909	FALSE	9.24E-08
35998_at	0.33452	2.16033	0.118973	-0.21437	0.88341	TRUE	1.00E+00
39605_at	0.33458	2.160628	0.095587	-0.10642	0.77558	TRUE	1.00E+00
474_at	0.33475	2.161474	0.146585	-0.34153	1.01103	TRUE	1.00E+00
33649_at	0.33495	2.16247	0.085034	-0.05736	0.72726	TRUE	1.00E+00
41027_at	0.33505	2.162968	0.04824	0.11249	0.5576	FALSE	4.76E-08
40158_r_at	0.33526	2.164014	0.109361	-0.16928	0.83981	TRUE	1.00E+00
35662_at	0.33537	2.164562	0.073632	-0.00434	0.67508	TRUE	6.62E-02
37573_at	0.33537	2.164562	0.034317	0.17705	0.4937	FALSE	1.86E-18
32464_at	0.33543	2.164861	0.072828	-0.00057	0.67143	TRUE	5.19E-02
39433_at	0.33586	2.167005	0.07301	-0.00098	0.67269	TRUE	5.33E-02
34739_at	0.33641	2.169752	0.103896	-0.14293	0.81574	TRUE	1.00E+00
1226_at	0.33647	2.170051	0.055309	0.0813	0.59164	FALSE	1.48E-05
39190_s_at	0.33647	2.170051	0.090815	-0.08251	0.75546	TRUE	1.00E+00
39479_g_at	0.33655	2.170451	0.085485	-0.05784	0.73094	TRUE	1.00E+00
1144_at	0.33749	2.175154	0.080423	-0.03355	0.70853	TRUE	3.42E-01
33355_at	0.33782	2.176807	0.035357	0.1747	0.50094	FALSE	1.56E-17
281_s_at	0.33828	2.179114	0.056592	0.07719	0.59938	FALSE	2.86E-05
40590_at	0.33883	2.181876	0.056772	0.07691	0.60076	FALSE	3.03E-05
34589_f_at	0.33916	2.183534	0.054657	0.08699	0.59133	FALSE	6.90E-06
35718_at	0.33945	2.184993	0.099594	-0.12003	0.79894	TRUE	1.00E+00
38160_at	0.33993	2.187409	0.110228	-0.16862	0.84848	TRUE	1.00E+00
32503_at	0.34059	2.190736	0.040685	0.15289	0.5283	FALSE	7.18E-13
41772_at	0.34066	2.191089	0.085702	-0.05474	0.73605	TRUE	8.89E-01
39625_at	0.34119	2.193764	0.083126	-0.04232	0.7247	TRUE	5.12E-01
34729_at	0.34143	2.194977	0.056306	0.08165	0.6012	FALSE	1.68E-05
41475_at	0.34144	2.195028	0.050005	0.11074	0.57215	FALSE	1.09E-07
31918_at	0.34152	2.195432	0.108486	-0.159	0.84203	TRUE	1.00E+00
33163_r_at	0.3419	2.197354	0.047583	0.12237	0.56143	FALSE	8.47E-09
41032_at	0.34235	2.199632	0.110833	-0.16899	0.85369	TRUE	1.00E+00
39695_at	0.34253	2.200544	0.094393	-0.09296	0.77803	TRUE	1.00E+00
33643_at	0.3427	2.201405	0.109561	-0.16276	0.84817	TRUE	1.00E+00
38300_at	0.34278	2.201811	0.027532	0.21576	0.4698	FALSE	1.76E-31
32110_at	0.34279	2.201862	0.063216	0.05114	0.63444	FALSE	7.42E-04
32052_at	0.34302	2.203028	0.063177	0.05155	0.63449	FALSE	7.13E-04
40238_at	0.34317	2.203789	0.033422	0.18897	0.49736	FALSE	1.24E-20
33728_at	0.34333	2.204601	0.124153	-0.22946	0.91612	TRUE	1.00E+00
31340_at	0.34403	2.208157	0.105818	-0.14417	0.83223	TRUE	1.00E+00
31571_at	0.34426	2.209327	0.095975	-0.09853	0.78705	TRUE	1.00E+00
203_at	0.34435	2.209785	0.098348	-0.10939	0.79808	TRUE	1.00E+00
38227_at	0.34446	2.210345	0.035907	0.1788	0.51011	FALSE	1.08E-17
36234_at	0.34473	2.211719	0.074412	0.00142	0.68803	FALSE	4.56E-02
1249_at	0.34489	2.212534	0.038115	0.16905	0.52074	FALSE	1.83E-15
31958_i_at	0.34491	2.212636	0.123235	-0.22364	0.91347	TRUE	1.00E+00
32257_f_at	0.3461	2.218707	0.037706	0.17214	0.52006	FALSE	5.49E-16
33830_at	0.34738	2.225256	0.041093	0.15779	0.53697	FALSE	3.57E-13
33066_at	0.34797	2.228281	0.094625	-0.08859	0.78452	TRUE	1.00E+00

36324_at	0.34835	2.230232	0.084516	-0.04157	0.73827	TRUE	4.75E-01
34992_g_at	0.34864	2.231722	0.054963	0.09506	0.60222	FALSE	2.84E-06
31628_at	0.34931	2.235167	0.126241	-0.23312	0.93173	TRUE	1.00E+00
38914_at	0.34999	2.23867	0.107891	-0.14778	0.84775	TRUE	1.00E+00
35463_at	0.35006	2.23903	0.109446	-0.15488	0.855	TRUE	1.00E+00
40973_at	0.35055	2.241558	0.029408	0.21488	0.48623	FALSE	1.17E-28
33807_at	0.35103	2.244037	0.057897	0.08392	0.61815	FALSE	1.69E-05
808_at	0.35109	2.244347	0.054258	0.10076	0.60141	FALSE	1.23E-06
41254_at	0.35109	2.244347	0.110102	-0.15687	0.85906	TRUE	1.00E+00
35430_at	0.35141	2.246001	0.116014	-0.18383	0.88665	TRUE	1.00E+00
38763_at	0.35144	2.246156	0.017821	0.26922	0.43366	FALSE	1.80E-82
38940_at	0.35158	2.246881	0.197789	-0.56093	1.2641	TRUE	1.00E+00
37626_at	0.35187	2.248381	0.056618	0.09066	0.61308	FALSE	6.49E-06
38506_at	0.35211	2.249624	0.07453	0.00825	0.69596	FALSE	2.91E-02
40297_at	0.35217	2.249935	0.115737	-0.18179	0.88613	TRUE	1.00E+00
32944_at	0.35273	2.252838	0.0774	-0.00437	0.70982	TRUE	6.55E-02
40772_at	0.35294	2.253928	0.090009	-0.06233	0.7682	TRUE	1.00E+00
36326_at	0.35363	2.257512	0.094006	-0.08007	0.78733	TRUE	1.00E+00
1063_s_at	0.35413	2.260112	0.10452	-0.12809	0.83634	TRUE	1.00E+00
506_s_at	0.35474	2.263289	0.092831	-0.07355	0.78302	TRUE	1.00E+00
160031_at	0.35507	2.265009	0.076096	0.00399	0.70615	FALSE	3.88E-02
38502_at	0.35559	2.267723	0.085987	-0.04112	0.7523	TRUE	4.47E-01
41136_s_at	0.35575	2.268559	0.024199	0.24411	0.46739	FALSE	8.00E-45
33534_at	0.35674	2.273736	0.144262	-0.30882	1.02231	TRUE	1.00E+00
31919_at	0.35682	2.274155	0.083071	-0.02643	0.74008	TRUE	2.20E-01
35638_at	0.35689	2.274521	0.046662	0.14161	0.57216	FALSE	2.57E-10
31401_r_at	0.35698	2.274993	0.145474	-0.31418	1.02813	TRUE	1.00E+00
31969_i_at	0.35701	2.27515	0.08371	-0.02919	0.74322	TRUE	2.53E-01
377_g_at	0.35769	2.278715	0.038506	0.18004	0.53534	FALSE	1.96E-16
1227_g_at	0.35805	2.280605	0.141687	-0.29564	1.01173	TRUE	1.00E+00
36066_at	0.35815	2.28113	0.024415	0.24551	0.47079	FALSE	1.28E-44
33609_at	0.35864	2.283705	0.098911	-0.0977	0.81497	TRUE	1.00E+00
41628_at	0.35953	2.28839	0.030541	0.21863	0.50044	FALSE	6.87E-28
34947_at	0.35961	2.288811	0.065367	0.05804	0.66119	FALSE	4.76E-04
40916_at	0.36042	2.293084	0.119231	-0.18967	0.9105	TRUE	1.00E+00
39875_at	0.36066	2.294352	0.091394	-0.061	0.78231	TRUE	1.00E+00
40250_at	0.36188	2.300806	0.149456	-0.32765	1.05141	TRUE	1.00E+00
1883_s_at	0.36243	2.303722	0.102538	-0.11063	0.8355	TRUE	1.00E+00
41354_at	0.36243	2.303722	0.158714	-0.36981	1.09467	TRUE	1.00E+00
36889_at	0.36285	2.305951	0.044705	0.1566	0.5691	FALSE	6.05E-12
160024_at	0.36286	2.306004	0.10404	-0.11713	0.84286	TRUE	1.00E+00
32694_at	0.36287	2.306057	0.120975	-0.19526	0.921	TRUE	1.00E+00
41217_at	0.36296	2.306535	0.069161	0.04388	0.68204	FALSE	1.94E-03
39038_at	0.36311	2.307332	0.027368	0.23685	0.48937	FALSE	4.49E-36
32531_at	0.36329	2.308288	0.019206	0.27469	0.4519	FALSE	1.06E-75
31333_at	0.36416	2.312917	0.085346	-0.02959	0.75792	TRUE	2.50E-01
39382_at	0.36443	2.314355	0.030704	0.22277	0.50609	FALSE	2.17E-28
1881_at	0.36474	2.316008	0.067794	0.05196	0.67751	FALSE	9.40E-04
34407_at	0.36496	2.317181	0.049654	0.13587	0.59404	FALSE	2.50E-09
35168_f_at	0.36537	2.31937	0.067435	0.05425	0.67649	FALSE	7.60E-04
36376_at	0.36562	2.320705	0.062004	0.07955	0.65168	FALSE	4.68E-05

39524_at	0.3657	2.321133	0.107404	-0.12981	0.86122	TRUE	1.00E+00
512_at	0.36581	2.321721	0.123726	-0.20501	0.93663	TRUE	1.00E+00
38790_at	0.36777	2.332223	0.076808	0.01341	0.72214	FALSE	2.12E-02
41615_at	0.36778	2.332276	0.126922	-0.21779	0.95335	TRUE	1.00E+00
31368_at	0.36818	2.334425	0.105175	-0.11705	0.85342	TRUE	1.00E+00
39981_at	0.36933	2.340615	0.131389	-0.23685	0.9755	TRUE	1.00E+00
40292_at	0.36949	2.341478	0.078529	0.00719	0.7318	FALSE	3.20E-02
41819_at	0.36968	2.342502	0.105654	-0.11776	0.85713	TRUE	1.00E+00
32610_at	0.36972	2.342718	0.13252	-0.24167	0.98111	TRUE	1.00E+00
578_at	0.37065	2.34774	0.064113	0.07486	0.66644	FALSE	9.37E-05
33938_g_at	0.37074	2.348227	0.070162	0.04705	0.69444	FALSE	1.59E-03
32155_at	0.37086	2.348876	0.059237	0.09757	0.64415	FALSE	4.84E-06
38769_at	0.37102	2.349741	0.098103	-0.08159	0.82362	TRUE	1.00E+00
1217_g_at	0.37152	2.352448	0.107687	-0.1253	0.86834	TRUE	1.00E+00
32948_at	0.37157	2.352719	0.101079	-0.09477	0.8379	TRUE	1.00E+00
40782_at	0.37181	2.354019	0.033439	0.21754	0.52608	FALSE	1.28E-24
affx-biob-5_st	0.37291	2.359989	0.093107	-0.05665	0.80247	TRUE	7.82E-01
35370_at	0.37326	2.361892	0.068686	0.05638	0.69015	FALSE	6.94E-04
41790_at	0.37347	2.363034	0.042813	0.17595	0.57099	FALSE	3.41E-14
38312_at	0.37354	2.363415	0.104301	-0.10767	0.85474	TRUE	1.00E+00
39955_at	0.37444	2.368318	0.087016	-0.02702	0.77589	TRUE	2.13E-01
37622_r_at	0.3754	2.373559	0.050546	0.1422	0.6086	FALSE	1.40E-09
34284_at	0.37938	2.395411	0.050733	0.14531	0.61344	FALSE	9.54E-10
40013_at	0.37967	2.397011	0.08459	-0.01059	0.76993	TRUE	9.06E-02
34545_at	0.3801	2.399385	0.060424	0.10133	0.65887	FALSE	3.99E-06
34605_at	0.38236	2.411904	0.053039	0.13766	0.62706	FALSE	7.11E-09
32461_f_at	0.38245	2.412404	0.067528	0.0709	0.694	FALSE	1.87E-04
37743_at	0.38319	2.416518	0.042957	0.185	0.58138	FALSE	5.87E-15
38326_at	0.38338	2.417575	0.159629	-0.35308	1.11984	TRUE	1.00E+00
38391_at	0.38353	2.41841	0.044632	0.17762	0.58945	FALSE	1.07E-13
37141_at	0.38358	2.418689	0.122591	-0.182	0.94917	TRUE	1.00E+00
39494_at	0.3837	2.419357	0.119414	-0.16723	0.93462	TRUE	1.00E+00
415_at	0.38381	2.41997	0.155314	-0.33275	1.10036	TRUE	1.00E+00
38717_at	0.38398	2.420918	0.067802	0.07117	0.6968	FALSE	1.87E-04
33585_at	0.3843	2.422702	0.13651	-0.2455	1.0141	TRUE	1.00E+00
32388_at	0.38462	2.424488	0.119561	-0.16698	0.93623	TRUE	1.00E+00
35455_i_at	0.38496	2.426387	0.085324	-0.00869	0.77862	TRUE	8.12E-02
39261_at	0.38572	2.430636	0.037821	0.21123	0.56021	FALSE	2.54E-20
40684_at	0.38734	2.43972	0.061935	0.1016	0.67308	FALSE	5.05E-06
41074_at	0.38762	2.441294	0.067434	0.07651	0.69873	FALSE	1.14E-04
34490_f_at	0.38765	2.441462	0.104609	-0.09497	0.87028	TRUE	1.00E+00
40135_at	0.38777	2.442137	0.051419	0.15055	0.625	FALSE	5.87E-10
32216_r_at	0.38906	2.449402	0.058781	0.11787	0.66025	FALSE	4.57E-07
41693_r_at	0.39002	2.454822	0.095438	-0.0503	0.83033	TRUE	5.53E-01
606_at	0.39172	2.46445	0.079472	0.02506	0.75837	FALSE	1.04E-02
37221_at	0.39209	2.46655	0.053211	0.1466	0.63759	FALSE	2.17E-09
32357_at	0.39247	2.46871	0.07413	0.05046	0.73447	FALSE	1.51E-03
36733_at	0.3928	2.470586	0.084681	0.00212	0.78349	FALSE	4.43E-02
1038_s_at	0.39281	2.470643	0.042869	0.19503	0.59059	FALSE	6.37E-16
37006_at	0.39291	2.471212	0.126955	-0.19281	0.97863	TRUE	1.00E+00
39077_at	0.39362	2.475255	0.04998	0.16304	0.62421	FALSE	4.28E-11

32239_at	0.39374	2.475939	0.04958	0.165	0.62248	FALSE	2.52E-11
1173_g_at	0.39421	2.47862	0.052173	0.15351	0.63491	FALSE	5.25E-10
34419_at	0.39442	2.479819	0.099058	-0.06259	0.85143	TRUE	8.64E-01
2076_s_at	0.39454	2.480504	0.078625	0.03179	0.75728	FALSE	6.59E-03
160030_at	0.39479	2.481933	0.037561	0.2215	0.56808	FALSE	9.74E-22
212_at	0.39567	2.486967	0.101245	-0.07143	0.86277	TRUE	1.00E+00
31934_at	0.39625	2.49029	0.127513	-0.19204	0.98454	TRUE	1.00E+00
37327_at	0.39629	2.49052	0.057564	0.13071	0.66186	FALSE	7.33E-08
34519_at	0.39725	2.496031	0.088346	-0.01034	0.80484	TRUE	8.72E-02
1436_at	0.3989	2.505532	0.069566	0.07796	0.71985	FALSE	1.24E-04
33358_at	0.39901	2.506167	0.032995	0.24679	0.55124	FALSE	1.45E-29
1183_at	0.39912	2.506802	0.086181	0.00152	0.79672	FALSE	4.59E-02
36623_at	0.39913	2.50686	0.06475	0.1004	0.69786	FALSE	8.94E-06
37852_at	0.40082	2.516634	0.05848	0.13102	0.67062	FALSE	9.07E-08
41771_g_at	0.40096	2.517445	0.144273	-0.26466	1.06658	TRUE	1.00E+00
34023_at	0.40122	2.518953	0.087829	-0.00398	0.80643	TRUE	6.21E-02
40970_at	0.40214	2.524294	0.076441	0.04947	0.75481	FALSE	1.81E-03
31796_at	0.40231	2.525283	0.11929	-0.14804	0.95267	TRUE	1.00E+00
41505_r_at	0.40235	2.525515	0.038446	0.22498	0.57973	FALSE	1.57E-21
34940_at	0.4024	2.525806	0.118649	-0.145	0.94979	TRUE	1.00E+00
33284_at	0.4033	2.531046	0.074053	0.06165	0.74496	FALSE	6.50E-04
33732_at	0.40349	2.532153	0.058302	0.13451	0.67247	FALSE	5.68E-08
41504_s_at	0.40392	2.534662	0.038036	0.22844	0.5794	FALSE	3.06E-22
35719_at	0.40445	2.537757	0.055191	0.14982	0.65908	FALSE	2.95E-09
34990_at	0.40464	2.538867	0.041501	0.21317	0.59611	FALSE	2.33E-18
1076_at	0.40584	2.545892	0.107962	-0.09225	0.90394	TRUE	1.00E+00
40333_at	0.40619	2.547945	0.057545	0.1407	0.67168	FALSE	2.12E-08
35493_at	0.40636	2.548942	0.057636	0.14045	0.67227	FALSE	2.25E-08
41448_at	0.40702	2.552819	0.159628	-0.32944	1.14348	TRUE	1.00E+00
36160_s_at	0.40808	2.559057	0.121508	-0.1525	0.96867	TRUE	1.00E+00
38038_at	0.40876	2.563067	0.088445	0.00071	0.81681	FALSE	4.81E-02
1114_at	0.40928	2.566138	0.049775	0.17964	0.63892	FALSE	2.51E-12
529_at	0.40931	2.566315	0.047777	0.18888	0.62973	FALSE	1.34E-13
39553_at	0.40947	2.567261	0.097356	-0.03969	0.85863	TRUE	3.28E-01
31850_at	0.41036	2.572527	0.038368	0.23335	0.58737	FALSE	1.35E-22
36243_at	0.4111	2.576914	0.065003	0.1112	0.711	FALSE	3.21E-06
40303_at	0.41138	2.578576	0.068368	0.09595	0.7268	FALSE	2.24E-05
34129_at	0.41228	2.583926	0.157048	-0.31228	1.13683	TRUE	1.00E+00
38126_at	0.41273	2.586604	0.15057	-0.28193	1.1074	TRUE	1.00E+00
34115_at	0.41326	2.589763	0.119473	-0.13793	0.96446	TRUE	1.00E+00
35174_i_at	0.41352	2.591314	0.089435	0.0009	0.82613	FALSE	4.76E-02
31817_at	0.41368	2.592269	0.104481	-0.06835	0.89571	TRUE	9.49E-01
33118_at	0.41413	2.594956	0.080855	0.0411	0.78716	FALSE	3.82E-03
40079_at	0.41438	2.59645	0.064883	0.11504	0.71373	FALSE	2.14E-06
655_at	0.4145	2.597168	0.105833	-0.07377	0.90277	TRUE	1.00E+00
36496_at	0.41504	2.600399	0.05364	0.16757	0.66251	FALSE	1.28E-10
35867_at	0.41605	2.606454	0.135502	-0.2091	1.0412	TRUE	1.00E+00
37678_at	0.41767	2.616194	0.034255	0.25963	0.57571	FALSE	4.29E-30
1882_g_at	0.418	2.618183	0.103727	-0.06055	0.89656	TRUE	7.05E-01
1891_at	0.4198	2.629057	0.092286	-0.00597	0.84557	TRUE	6.81E-02
39642_at	0.4212	2.637546	0.068275	0.10621	0.7362	FALSE	8.67E-06

36606_at	0.42131	2.638214	0.240717	-0.68926	1.53187	TRUE	1.00E+00
195_s_at	0.42214	2.643261	0.059983	0.1454	0.69887	FALSE	2.47E-08
32277_at	0.42335	2.650635	0.092519	-0.00349	0.85019	TRUE	5.99E-02
33883_at	0.42335	2.650635	0.115428	-0.10919	0.95589	TRUE	1.00E+00
34365_at	0.42379	2.653322	0.101217	-0.04318	0.89077	TRUE	3.57E-01
33572_at	0.42407	2.655033	0.101473	-0.04408	0.89223	TRUE	3.69E-01
40155_at	0.42425	2.656134	0.045195	0.21574	0.63276	FALSE	7.78E-17
279_at	0.42461	2.658337	0.101707	-0.04463	0.89384	TRUE	3.77E-01
35724_at	0.425	2.660725	0.040008	0.24042	0.60958	FALSE	2.94E-22
41859_at	0.42518	2.661828	0.060926	0.14409	0.70627	FALSE	3.76E-08
37684_at	0.42558	2.664281	0.059795	0.14971	0.70145	FALSE	1.39E-08
34908_at	0.42634	2.668947	0.08725	0.02381	0.82888	FALSE	1.30E-02
34631_at	0.42644	2.669562	0.074547	0.08251	0.77036	FALSE	1.34E-04
819_at	0.4269	2.672391	0.122341	-0.13753	0.99133	TRUE	1.00E+00
38077_at	0.4276	2.676702	0.192391	-0.46002	1.31521	TRUE	1.00E+00
39841_at	0.42975	2.689986	0.063932	0.1348	0.72471	FALSE	2.26E-07
41636_at	0.42986	2.690667	0.087123	0.02791	0.83181	FALSE	1.02E-02
34944_at	0.43018	2.692651	0.117276	-0.11088	0.97124	TRUE	1.00E+00
36963_at	0.43018	2.692651	0.033782	0.27433	0.58604	FALSE	4.83E-33
37102_at	0.43024	2.693023	0.048655	0.20576	0.65471	FALSE	1.18E-14
823_at	0.43046	2.694387	0.04175	0.23784	0.62308	FALSE	8.00E-21
35892_at	0.43047	2.694449	0.113017	-0.09095	0.95189	TRUE	1.00E+00
35554_f_at	0.43064	2.695504	0.105289	-0.05512	0.9164	TRUE	5.45E-01
41275_at	0.43072	2.696001	0.108314	-0.06899	0.93044	TRUE	8.83E-01
33299_at	0.43103	2.697926	0.035496	0.26727	0.5948	FALSE	7.88E-30
36269_at	0.43117	2.698796	0.069381	0.11108	0.75127	FALSE	6.50E-06
32640_at	0.43135	2.699914	0.084656	0.04078	0.82192	FALSE	4.40E-03
34389_at	0.43195	2.703647	0.136219	-0.19651	1.06041	TRUE	1.00E+00
31957_r_at	0.4331	2.710816	0.224817	-0.60411	1.47031	TRUE	1.00E+00
36489_at	0.43359	2.713876	0.047129	0.21615	0.65102	FALSE	4.52E-16
32605_r_at	0.43372	2.714688	0.06348	0.14085	0.72659	FALSE	1.05E-07
33565_at	0.43409	2.717002	0.062101	0.14758	0.7206	FALSE	3.47E-08
41839_at	0.43437	2.718755	0.096505	-0.01086	0.87961	TRUE	8.54E-02
37842_at	0.43439	2.71888	0.045602	0.224	0.64478	FALSE	2.07E-17
35705_at	0.43469	2.720759	0.036904	0.26443	0.60495	FALSE	6.32E-28
33478_at	0.4351	2.723328	0.073608	0.0955	0.7747	FALSE	4.29E-05
574_s_at	0.43543	2.725398	0.08023	0.06528	0.80558	FALSE	7.22E-04
32343_at	0.436	2.728978	0.095251	-0.00345	0.87545	TRUE	5.95E-02
36460_at	0.43668	2.733254	0.079174	0.0714	0.80195	FALSE	4.39E-04
34801_at	0.43793	2.741132	0.099548	-0.02134	0.89721	TRUE	1.37E-01
39602_at	0.4385	2.744732	0.107079	-0.05552	0.93252	TRUE	5.33E-01
33998_at	0.43948	2.750933	0.109087	-0.0638	0.94276	TRUE	7.08E-01
35034_at	0.43998	2.754102	0.124546	-0.13462	1.01458	TRUE	1.00E+00
33892_at	0.4409	2.759942	0.209264	-0.52456	1.40636	TRUE	1.00E+00
36868_at	0.44138	2.762994	0.133647	-0.17521	1.05797	TRUE	1.00E+00
36924_r_at	0.44231	2.768917	0.091857	0.01852	0.8661	FALSE	1.86E-02
39506_at	0.44258	2.770639	0.086219	0.04481	0.84036	FALSE	3.60E-03
41533_at	0.44336	2.77562	0.077061	0.08784	0.79889	FALSE	1.10E-04
37960_at	0.44337	2.775684	0.103867	-0.03583	0.92257	TRUE	2.48E-01
40321_at	0.44345	2.776195	0.054437	0.1923	0.6946	FALSE	4.74E-12
39227_at	0.44545	2.78901	0.082546	0.06462	0.82629	FALSE	8.58E-04

40290_f_at	0.44673	2.797242	0.076805	0.09238	0.80108	FALSE	7.59E-05
36287_at	0.44735	2.801238	0.088269	0.04011	0.85458	FALSE	5.08E-03
41271_at	0.44931	2.813909	0.036007	0.28319	0.61544	FALSE	1.24E-31
658_at	0.44985	2.81741	0.042902	0.25192	0.64778	FALSE	1.27E-21
31902_at	0.45082	2.823709	0.146365	-0.22445	1.12608	TRUE	1.00E+00
39654_at	0.45105	2.825205	0.057785	0.18445	0.71765	FALSE	7.48E-11
38557_at	0.45115	2.825856	0.111008	-0.061	0.96329	TRUE	6.09E-01
37595_at	0.45205	2.831718	0.128392	-0.14029	1.0444	TRUE	1.00E+00
32418_at	0.45294	2.837527	0.037971	0.27776	0.62812	FALSE	1.06E-28
37534_at	0.45316	2.838965	0.0653	0.15189	0.75443	FALSE	4.96E-08
33750_at	0.45454	2.848	0.10326	-0.02186	0.93093	TRUE	1.35E-01
36073_at	0.45509	2.851609	0.117741	-0.08812	0.9983	TRUE	1.00E+00
39566_at	0.45612	2.85838	0.096116	0.01268	0.89956	FALSE	2.63E-02
460_at	0.45675	2.86253	0.114809	-0.07294	0.98643	TRUE	8.76E-01
32320_at	0.45772	2.86893	0.07411	0.11581	0.79963	FALSE	8.29E-06
32349_at	0.4583	2.872764	0.097542	0.00828	0.90832	FALSE	3.31E-02
41245_at	0.46015	2.885028	0.158779	-0.27239	1.19269	TRUE	1.00E+00
37902_at	0.46074	2.88895	0.040993	0.27161	0.64986	FALSE	3.30E-25
40046_r_at	0.46127	2.892478	0.068215	0.14655	0.77599	FALSE	1.72E-07
32089_at	0.46136	2.893077	0.120199	-0.09319	1.01591	TRUE	1.00E+00
39493_at	0.46244	2.90028	0.121631	-0.09872	1.0236	TRUE	1.00E+00
41748_at	0.46246	2.900414	0.101024	-0.00362	0.92855	TRUE	5.93E-02
488_at	0.46343	2.906899	0.035219	0.30094	0.62592	FALSE	1.92E-35
41350_at	0.46375	2.909042	0.1304	-0.13786	1.06536	TRUE	1.00E+00
40069_at	0.46382	2.909511	0.058767	0.1927	0.73495	FALSE	3.74E-11
35692_at	0.46438	2.913265	0.069426	0.14408	0.78468	FALSE	2.84E-07
1757_i_at	0.46507	2.917897	0.202974	-0.47136	1.40151	TRUE	1.00E+00
38448_at	0.4654	2.920115	0.088898	0.05527	0.87554	FALSE	2.08E-03
38351_at	0.46729	2.932851	0.073323	0.129	0.80557	FALSE	2.34E-06
34304_s_at	0.46778	2.936162	0.066622	0.16041	0.77515	FALSE	2.77E-08
34282_at	0.46792	2.937109	0.080026	0.09871	0.83712	FALSE	6.32E-05
291_s_at	0.46844	2.940627	0.063784	0.17417	0.76271	FALSE	2.61E-09
615_s_at	0.46878	2.94293	0.122154	-0.09479	1.03235	TRUE	1.00E+00
35391_at	0.4694	2.947135	0.077254	0.11298	0.82581	FALSE	1.56E-05
36718_s_at	0.47036	2.953657	0.159673	-0.26631	1.20702	TRUE	1.00E+00
32250_at	0.47054	2.954881	0.055642	0.21383	0.72725	FALSE	3.48E-13
37944_at	0.47057	2.955085	0.081719	0.09355	0.84758	FALSE	1.07E-04
38912_at	0.47169	2.962716	0.109187	-0.03205	0.97543	TRUE	1.97E-01
32962_at	0.47169	2.962716	0.133417	-0.14384	1.08723	TRUE	1.00E+00
41677_at	0.47393	2.978036	0.101147	0.00728	0.94058	FALSE	3.52E-02
1577_at	0.47491	2.984764	0.10155	0.0064	0.94342	FALSE	3.68E-02
1761_at	0.47544	2.988409	0.086337	0.07712	0.87377	FALSE	4.61E-04
41239_r_at	0.47745	3.002272	0.118542	-0.06946	1.02435	TRUE	7.11E-01
39581_at	0.47822	3.007599	0.11465	-0.05073	1.00716	TRUE	3.83E-01
33446_at	0.47822	3.007599	0.185463	-0.37743	1.33388	TRUE	1.00E+00
36244_at	0.47871	3.010995	0.077226	0.12242	0.835	FALSE	7.18E-06
41303_r_at	0.47909	3.01363	0.071364	0.14985	0.80834	FALSE	2.40E-07
35959_at	0.48019	3.021273	0.088502	0.07187	0.8885	FALSE	7.29E-04
40409_at	0.4813	3.029005	0.052727	0.23804	0.72456	FALSE	8.79E-16
39593_at	0.4818	3.032494	0.148781	-0.20462	1.16821	TRUE	1.00E+00
41692_at	0.48197	3.033682	0.034684	0.32195	0.64198	FALSE	8.46E-40

36749_at	0.48316	3.042006	0.115946	-0.05177	1.01809	TRUE	3.89E-01
608_at	0.48318	3.042146	0.153804	-0.22641	1.19277	TRUE	1.00E+00
38152_at	0.48372	3.045931	0.06983	0.16155	0.80588	FALSE	5.42E-08
41536_at	0.48503	3.055132	0.157513	-0.24167	1.21173	TRUE	1.00E+00
37746_r_at	0.48588	3.061117	0.244877	-0.64389	1.61564	TRUE	1.00E+00
32215_i_at	0.48685	3.067962	0.044545	0.28133	0.69236	FALSE	1.05E-23
33158_at	0.48721	3.070506	0.099714	0.02717	0.94724	FALSE	1.30E-02
38159_at	0.48733	3.071355	0.131935	-0.12137	1.09602	TRUE	1.00E+00
1922_g_at	0.48809	3.076734	0.080636	0.11606	0.86011	FALSE	1.80E-05
1111_at	0.48818	3.077372	0.065023	0.18819	0.78817	FALSE	7.59E-10
40676_at	0.48866	3.080775	0.10556	0.00165	0.97568	FALSE	4.63E-02
41764_at	0.49015	3.091363	0.081856	0.11249	0.8678	FALSE	2.68E-05
33502_at	0.49102	3.097562	0.055195	0.23637	0.74567	FALSE	7.30E-15
129_g_at	0.49131	3.099631	0.035085	0.32944	0.65318	FALSE	1.88E-40
2042_s_at	0.49137	3.100059	0.08535	0.0976	0.88514	FALSE	1.08E-04
33945_at	0.4919	3.103845	0.07336	0.15344	0.83035	FALSE	2.54E-07
41481_at	0.49432	3.121189	0.0926	0.0671	0.92154	FALSE	1.19E-03
40230_at	0.496	3.133286	0.078003	0.13612	0.85587	FALSE	2.57E-06
39449_at	0.4974	3.143403	0.096559	0.05192	0.94288	FALSE	3.27E-03
39470_at	0.49812	3.148618	0.080926	0.12476	0.87148	FALSE	9.47E-06
41871_at	0.49888	3.154133	0.045683	0.28812	0.70964	FALSE	1.16E-23
39495_at	0.5002	3.163734	0.157279	-0.22542	1.22582	TRUE	1.00E+00
32682_at	0.5002	3.163734	0.120076	-0.05378	1.05418	TRUE	3.92E-01
33442_at	0.50095	3.169203	0.132711	-0.11132	1.11323	TRUE	1.00E+00
39310_at	0.50109	3.170224	0.027594	0.37379	0.6284	FALSE	1.36E-69
38927_i_at	0.5017	3.17468	0.14092	-0.14845	1.15184	TRUE	1.00E+00
1455_f_at	0.50174	3.174973	0.102642	0.02819	0.97529	FALSE	1.28E-02
35706_at	0.50322	3.185811	0.049071	0.27683	0.72961	FALSE	1.42E-20
40952_at	0.50351	3.187939	0.072789	0.1677	0.83933	FALSE	5.81E-08
38013_at	0.50524	3.200663	0.04274	0.30805	0.70242	FALSE	3.83E-28
39545_at	0.50554	3.202875	0.027209	0.38	0.63107	FALSE	5.96E-73
40683_at	0.50906	3.22894	0.128131	-0.08209	1.1002	TRUE	8.96E-01
38184_at	0.5097	3.233702	0.073551	0.17037	0.84904	FALSE	5.31E-08
36070_at	0.5101	3.236682	0.052308	0.26877	0.75143	FALSE	2.29E-18
35352_at	0.51011	3.236756	0.048862	0.28468	0.73554	FALSE	2.06E-21
36513_at	0.51127	3.245413	0.070812	0.18457	0.83796	FALSE	6.56E-09
36490_s_at	0.51222	3.25252	0.050782	0.27793	0.7465	FALSE	8.01E-20
33310_at	0.51367	3.263398	0.103786	0.03484	0.9925	FALSE	9.40E-03
1052_s_at	0.51443	3.269114	0.025671	0.396	0.63287	FALSE	3.13E-85
37637_at	0.5151	3.274161	0.085963	0.1185	0.9117	FALSE	2.62E-05
38466_at	0.51514	3.274462	0.045842	0.30365	0.72664	FALSE	3.37E-25
37187_at	0.51519	3.274839	0.112891	-0.00565	1.03602	TRUE	6.35E-02
1586_at	0.51524	3.275216	0.140243	-0.13179	1.16226	TRUE	1.00E+00
40496_at	0.5157	3.278687	0.068268	0.20074	0.83066	FALSE	5.33E-10
34388_at	0.51583	3.279669	0.138075	-0.12119	1.15285	TRUE	1.00E+00
39842_at	0.51784	3.294883	0.120632	-0.03871	1.07438	TRUE	2.23E-01
41666_at	0.51824	3.297919	0.12419	-0.05472	1.0912	TRUE	3.80E-01
37701_at	0.51826	3.298071	0.100453	0.05481	0.98171	FALSE	3.13E-03
37142_at	0.51898	3.303543	0.102964	0.04395	0.99401	FALSE	5.86E-03
38370_at	0.52006	3.311769	0.095531	0.07933	0.9608	FALSE	6.58E-04
37319_at	0.52034	3.313905	0.11979	-0.03232	1.07301	TRUE	1.77E-01

41044_at	0.52137	3.321773	0.095727	0.07973	0.96302	FALSE	6.49E-04
41484_r_at	0.52228	3.328741	0.06891	0.20435	0.8402	FALSE	4.39E-10
37489_s_at	0.52312	3.335186	0.066967	0.21416	0.83208	FALSE	7.13E-11
185_at	0.52359	3.338797	0.067942	0.21014	0.83705	FALSE	1.63E-10
34354_at	0.52643	3.360702	0.114059	0.00021	1.05265	FALSE	4.95E-02
535_s_at	0.52765	3.370156	0.099154	0.07019	0.9851	FALSE	1.30E-03
32419_at	0.52999	3.388364	0.058095	0.26196	0.79801	FALSE	9.24E-16
35757_at	0.53018	3.389846	0.109631	0.02439	1.03597	FALSE	1.67E-02
32685_at	0.53059	3.393048	0.071989	0.19846	0.86272	FALSE	2.15E-09
35579_at	0.53142	3.399539	0.078949	0.16719	0.89566	FALSE	2.12E-07
40383_at	0.53204	3.404395	0.173259	-0.2673	1.33138	TRUE	1.00E+00
40313_at	0.53304	3.412243	0.076368	0.18071	0.88536	FALSE	3.73E-08
38497_at	0.53335	3.41468	0.112564	0.01402	1.05267	FALSE	2.72E-02
40330_at	0.53399	3.419716	0.069094	0.21522	0.85276	FALSE	1.37E-10
36681_at	0.53459	3.424443	0.097032	0.08692	0.98225	FALSE	4.55E-04
40346_at	0.53477	3.425863	0.114987	0.00426	1.06527	FALSE	4.18E-02
661_at	0.53521	3.429336	0.117498	-0.00688	1.07729	TRUE	6.61E-02
40231_at	0.53628	3.437795	0.122645	-0.02955	1.10211	TRUE	1.55E-01
41572_r_at	0.53631	3.438033	0.084015	0.1487	0.92392	FALSE	2.18E-06
33236_at	0.53718	3.444927	0.045415	0.32765	0.7467	FALSE	3.53E-28
37241_at	0.53817	3.452789	0.042945	0.34003	0.7363	FALSE	6.34E-32
34417_at	0.53869	3.456925	0.059659	0.26345	0.81393	FALSE	2.18E-15
154_at	0.53888	3.458438	0.1274	-0.04889	1.12666	TRUE	2.95E-01
675_at	0.54028	3.469605	0.121358	-0.01961	1.10018	TRUE	1.07E-01
1042_at	0.54043	3.470803	0.100384	0.0773	1.00356	FALSE	9.21E-04
36454_at	0.54094	3.474882	0.178922	-0.28453	1.36641	TRUE	1.00E+00
35389_s_at	0.54103	3.475602	0.193791	-0.35304	1.43511	TRUE	1.00E+00
37209_g_at	0.54128	3.477603	0.016231	0.4664	0.61616	FALSE	9.52E-240
35099_at	0.54172	3.481128	0.133728	-0.07525	1.15868	TRUE	6.44E-01
32158_at	0.54217	3.484737	0.054616	0.29019	0.79414	FALSE	4.01E-19
39007_at	0.54253	3.487627	0.059131	0.26972	0.81533	FALSE	5.70E-16
41503_at	0.54301	3.491484	0.027082	0.41806	0.66795	FALSE	2.51E-85
38279_at	0.5434	3.49462	0.048017	0.32187	0.76493	FALSE	1.37E-25
36319_at	0.54372	3.497196	0.139683	-0.10072	1.18816	TRUE	1.00E+00
36049_at	0.54755	3.528174	0.070504	0.22227	0.87282	FALSE	1.02E-10
36453_at	0.54756	3.528255	0.13078	-0.05581	1.15093	TRUE	3.57E-01
31473_s_at	0.54819	3.533377	0.120432	-0.00743	1.10382	TRUE	6.71E-02
1321_s_at	0.55074	3.554185	0.04842	0.32735	0.77412	FALSE	7.10E-26
33687_at	0.55087	3.555249	0.10495	0.06668	1.03507	FALSE	1.93E-03
39930_at	0.55167	3.561804	0.120925	-0.00623	1.10957	TRUE	6.40E-02
36619_r_at	0.55386	3.57981	0.067387	0.24297	0.86476	FALSE	2.59E-12
2001_g_at	0.55494	3.588724	0.073878	0.2141	0.89578	FALSE	7.37E-10
34993_at	0.55502	3.589385	0.061157	0.27287	0.83718	FALSE	1.43E-15
41683_i_at	0.55511	3.590129	0.094343	0.11985	0.99037	FALSE	5.06E-05
35372_r_at	0.55518	3.590707	0.118662	0.00772	1.10264	FALSE	3.65E-02
681_at	0.55829	3.616513	0.065109	0.2579	0.85867	FALSE	1.25E-13
37394_at	0.55865	3.619512	0.095434	0.11836	0.99895	FALSE	6.07E-05
473_g_at	0.55912	3.623431	0.110178	0.05081	1.06744	FALSE	4.90E-03
33701_at	0.55973	3.628524	0.144462	-0.10676	1.22621	TRUE	1.00E+00
37671_at	0.5601	3.631617	0.073215	0.22232	0.89789	FALSE	2.53E-10
38261_at	0.56078	3.637307	0.101844	0.09092	1.03065	FALSE	4.63E-04

36497_at	0.56212	3.648547	0.054906	0.30881	0.81543	FALSE	1.69E-20
36548_at	0.56234	3.650396	0.083602	0.17664	0.94805	FALSE	2.19E-07
38856_at	0.56378	3.66252	0.06498	0.26399	0.86358	FALSE	5.16E-14
1709_g_at	0.56386	3.663195	0.056872	0.30148	0.82625	FALSE	4.54E-19
1016_s_at	0.56437	3.667499	0.061376	0.28121	0.84754	FALSE	4.72E-16
34334_at	0.56726	3.691986	0.082153	0.18824	0.94628	FALSE	6.34E-08
41770_at	0.56839	3.701604	0.127374	-0.01926	1.15604	TRUE	1.02E-01
128_at	0.56874	3.704589	0.041437	0.37757	0.75992	FALSE	9.04E-39
33767_at	0.57124	3.725976	0.095379	0.13121	1.01128	FALSE	2.66E-05
37533_r_at	0.57151	3.728293	0.156922	-0.15246	1.29549	TRUE	1.00E+00
36134_at	0.57507	3.75898	0.068273	0.26009	0.89005	FALSE	4.63E-13
704_at	0.57554	3.76305	0.098278	0.12213	1.02895	FALSE	5.98E-05
35249_at	0.57593	3.766431	0.144724	-0.09177	1.24363	TRUE	8.72E-01
41710_at	0.57741	3.779288	0.13674	-0.05345	1.20827	TRUE	3.05E-01
41660_at	0.57857	3.789396	0.079665	0.21103	0.94611	FALSE	4.80E-09
39613_at	0.58117	3.81215	0.03778	0.40687	0.75548	FALSE	2.69E-49
40001_r_at	0.58323	3.830275	0.10295	0.10827	1.0582	FALSE	1.85E-04
36308_at	0.58444	3.840962	0.228625	-0.47034	1.63922	TRUE	1.00E+00
1372_at	0.58718	3.865271	0.07045	0.26215	0.9122	FALSE	9.82E-13
35752_s_at	0.58818	3.874182	0.063015	0.29746	0.8789	FALSE	1.29E-16
33431_at	0.5889	3.88061	0.180017	-0.24162	1.41942	TRUE	1.00E+00
39409_at	0.59024	3.892602	0.067252	0.27997	0.90051	FALSE	2.13E-14
36890_at	0.5913	3.902114	0.098477	0.13697	1.04564	FALSE	2.42E-05
38836_at	0.59158	3.904631	0.153996	-0.11889	1.30206	TRUE	1.00E+00
36247_f_at	0.59381	3.924732	0.101233	0.12676	1.06085	FALSE	5.65E-05
37405_at	0.59393	3.925817	0.048124	0.3719	0.81595	FALSE	6.83E-31
41554_at	0.59448	3.930791	0.058223	0.32586	0.8631	FALSE	2.25E-20
1388_g_at	0.59502	3.935682	0.04941	0.36707	0.82298	FALSE	2.68E-29
35000_at	0.59934	3.975026	0.089185	0.18787	1.0108	FALSE	2.29E-07
40834_at	0.60552	4.031995	0.103162	0.12957	1.08147	FALSE	5.52E-05
32985_at	0.60829	4.057794	0.094837	0.17075	1.04583	FALSE	1.79E-06
36487_at	0.60853	4.060037	0.151273	-0.08938	1.30644	TRUE	7.26E-01
1664_at	0.61201	4.092701	0.048644	0.38758	0.83643	FALSE	3.38E-32
1916_s_at	0.61318	4.103742	0.102747	0.13915	1.08722	FALSE	3.03E-05
31703_at	0.61502	4.121165	0.077377	0.25804	0.972	FALSE	2.39E-11
41009_at	0.6187	4.156234	0.08876	0.2092	1.0282	FALSE	3.99E-08
34760_at	0.62006	4.16927	0.048351	0.39699	0.84313	FALSE	1.52E-33
34296_at	0.62407	4.207944	0.098339	0.17038	1.07777	FALSE	2.79E-06
36342_r_at	0.62508	4.217742	0.091969	0.20077	1.04939	FALSE	1.35E-07
34820_at	0.63045	4.270218	0.163225	-0.1226	1.38351	TRUE	1.00E+00
2018_at	0.63691	4.334211	0.153216	-0.06996	1.34379	TRUE	4.07E-01
34666_at	0.63734	4.338504	0.093743	0.20485	1.06983	FALSE	1.33E-07
40977_f_at	0.64161	4.381371	0.148357	-0.04285	1.32607	TRUE	1.93E-01
41870_at	0.64194	4.384701	0.031717	0.49561	0.78827	FALSE	5.50E-87
40049_at	0.64351	4.400581	0.06377	0.3493	0.93772	FALSE	7.64E-20
33562_g_at	0.64527	4.418451	0.062494	0.35695	0.93359	FALSE	6.84E-21
36309_at	0.64534	4.419163	0.174711	-0.1607	1.45139	TRUE	1.00E+00
38379_at	0.64726	4.438743	0.053611	0.39993	0.8946	FALSE	1.84E-29
37156_at	0.65709	4.540357	0.078687	0.29406	1.02011	FALSE	8.57E-13
32783_at	0.65742	4.543808	0.145618	-0.0144	1.32924	TRUE	8.01E-02
38793_at	0.65742	4.543808	0.07877	0.29401	1.02084	FALSE	8.91E-13

33772_at	0.65808	4.550719	0.112168	0.14059	1.17558	FALSE	5.60E-05
38968_at	0.65851	4.555227	0.054651	0.40637	0.91065	FALSE	2.47E-29
40239_g_at	0.66123	4.583846	0.039749	0.47785	0.84461	FALSE	4.88E-58
36931_at	0.66852	4.661439	0.064944	0.3689	0.96815	FALSE	9.49E-21
38124_at	0.67028	4.680368	0.10216	0.19896	1.1416	FALSE	6.74E-07
33468_at	0.67048	4.682524	0.081398	0.29494	1.04601	FALSE	2.23E-12
36563_at	0.67205	4.699482	0.062732	0.38263	0.96147	FALSE	1.12E-22
34991_at	0.67292	4.708906	0.052295	0.43165	0.91418	FALSE	8.62E-34
41472_at	0.67405	4.721174	0.103141	0.1982	1.1499	FALSE	8.02E-07
32143_at	0.67431	4.724001	0.11457	0.14573	1.20288	FALSE	5.01E-05
38503_at	0.67504	4.731948	0.105685	0.18746	1.16263	FALSE	2.13E-06
37749_at	0.67805	4.764858	0.183627	-0.16913	1.52523	TRUE	1.00E+00
160021_r_at	0.68132	4.800871	0.117018	0.14145	1.22119	FALSE	7.32E-05
36618_g_at	0.68333	4.823141	0.084609	0.29298	1.07369	FALSE	8.43E-12
34770_at	0.6851	4.842839	0.097515	0.23521	1.13499	FALSE	2.69E-08
38223_at	0.6891	4.887649	0.065232	0.38815	0.99006	FALSE	5.53E-22
31805_at	0.69396	4.942652	0.09592	0.25143	1.1365	FALSE	5.88E-09
40502_r_at	0.69544	4.959524	0.120068	0.1415	1.24939	FALSE	8.78E-05
41031_at	0.69935	5.004377	0.093051	0.27005	1.12865	FALSE	7.14E-10
37168_at	0.69948	5.005875	0.135175	0.07584	1.32312	FALSE	2.88E-03
40541_at	0.70442	5.063141	0.051046	0.46892	0.93993	FALSE	3.23E-39
39061_at	0.70494	5.069207	0.090378	0.28797	1.12191	FALSE	7.82E-11
942_at	0.70811	5.106343	0.116662	0.16988	1.24633	FALSE	1.62E-05
32113_at	0.71246	5.157747	0.108838	0.21032	1.21459	FALSE	7.46E-07
565_at	0.71273	5.160954	0.099095	0.25555	1.16992	FALSE	8.04E-09
33199_at	0.71292	5.163213	0.079584	0.34575	1.08009	FALSE	4.17E-15
40199_at	0.71393	5.175234	0.078907	0.34989	1.07798	FALSE	1.84E-15
37805_at	0.71723	5.214708	0.042456	0.52136	0.91311	FALSE	6.33E-60
36478_at	0.71892	5.23504	0.041067	0.52946	0.90839	FALSE	1.63E-64
33264_at	0.73181	5.392746	0.076467	0.37902	1.0846	FALSE	1.35E-17
38786_at	0.73402	5.420259	0.07092	0.40682	1.06121	FALSE	5.29E-21
41123_s_at	0.73419	5.422381	0.087342	0.33123	1.13715	FALSE	5.36E-13
38855_s_at	0.73604	5.445528	0.045975	0.52393	0.94815	FALSE	1.38E-53
37248_at	0.74062	5.50326	0.051299	0.50395	0.97729	FALSE	3.81E-43
1955_s_at	0.74079	5.505414	0.084386	0.35147	1.13011	FALSE	2.09E-14
34009_at	0.74124	5.511122	0.043951	0.53847	0.94401	FALSE	1.03E-59
37762_at	0.74357	5.540768	0.053442	0.49701	0.99013	FALSE	6.62E-40
32552_at	0.74749	5.591007	0.074013	0.40602	1.08896	FALSE	7.02E-20
38218_at	0.74808	5.598607	0.172313	-0.0469	1.54306	TRUE	1.79E-01
32184_at	0.74917	5.612676	0.247493	-0.39266	1.891	TRUE	1.00E+00
37989_at	0.75196	5.648849	0.113993	0.22604	1.27788	FALSE	5.32E-07
34853_at	0.75318	5.66474	0.146645	0.07662	1.42974	FALSE	3.54E-03
35624_at	0.7626	5.788953	0.09681	0.31596	1.20924	FALSE	4.22E-11
32249_at	0.76613	5.836198	0.081131	0.39183	1.14044	FALSE	4.57E-17
41260_at	0.76746	5.854098	0.037634	0.59384	0.94109	FALSE	2.44E-88
37203_at	0.76763	5.85639	0.099498	0.30859	1.22668	FALSE	1.53E-10
34693_at	0.76869	5.870702	0.098808	0.31283	1.22455	FALSE	9.18E-11
1669_at	0.77084	5.899837	0.104616	0.28819	1.2535	FALSE	2.18E-09
32812_at	0.771	5.902011	0.137319	0.13747	1.40453	FALSE	2.49E-04
1897_at	0.77234	5.920249	0.053177	0.527	1.01768	FALSE	1.08E-43
31862_at	0.77601	5.97049	0.089023	0.3653	1.18672	FALSE	3.61E-14

40401_at	0.77894	6.010907	0.074228	0.43649	1.1214	FALSE	1.16E-21
1970_s_at	0.79279	6.205689	0.079483	0.42609	1.15949	FALSE	2.49E-19
1234_at	0.81504	6.531907	0.102352	0.34283	1.28724	FALSE	2.12E-11
36834_at	0.82018	6.609673	0.109961	0.31286	1.32749	FALSE	1.10E-09
36780_at	0.83011	6.762542	0.052184	0.58936	1.07087	FALSE	7.10E-53
38673_s_at	0.83204	6.792662	0.02467	0.71823	0.94586	FALSE	2.92E-245
36661_s_at	0.83336	6.813339	0.104572	0.3509	1.31581	FALSE	2.02E-11
35073_at	0.83585	6.852515	0.067516	0.52436	1.14734	FALSE	4.24E-31
36867_at	0.8444	6.988758	0.11426	0.31725	1.37154	FALSE	1.85E-09
41385_at	0.84901	7.063338	0.042566	0.65263	1.04539	FALSE	2.06E-84
36617_at	0.85097	7.095288	0.123541	0.28101	1.42094	FALSE	7.13E-08
35822_at	0.85109	7.097248	0.133649	0.23449	1.46769	FALSE	2.42E-06
1888_s_at	0.86211	7.279642	0.039557	0.67961	1.04461	FALSE	3.33E-101
32963_s_at	0.8643	7.316443	0.097165	0.41603	1.31258	FALSE	7.36E-15
33956_at	0.8725	7.455899	0.08787	0.46711	1.2779	FALSE	3.91E-19
34087_at	0.87397	7.481178	0.140138	0.22743	1.5205	FALSE	5.65E-06
36040_at	0.87976	7.581585	0.087327	0.47687	1.28265	FALSE	9.06E-20
38323_at	0.88311	7.640293	0.135153	0.25958	1.50665	FALSE	8.07E-07
38096_f_at	0.88842	7.734282	0.080954	0.51493	1.26191	FALSE	6.41E-24
1380_at	0.88852	7.736063	0.120436	0.33288	1.44416	FALSE	2.04E-09
34213_at	0.89034	7.768551	0.081002	0.51663	1.26405	FALSE	5.29E-24
40698_at	0.89179	7.794531	0.062851	0.60183	1.18176	FALSE	1.35E-41
35561_at	0.89526	7.857059	0.107569	0.39898	1.39154	FALSE	1.09E-12
40398_s_at	0.89747	7.897143	0.119107	0.34796	1.44698	FALSE	6.17E-10
38634_at	0.90024	7.947673	0.08658	0.5008	1.29968	FALSE	3.20E-21
34377_at	0.90454	8.026755	0.130215	0.30378	1.5053	FALSE	4.73E-08
1232_s_at	0.90892	8.108117	0.175401	0.09969	1.71815	FALSE	2.77E-03
1591_s_at	0.91157	8.157743	0.083538	0.52615	1.29698	FALSE	1.28E-23
583_s_at	0.92872	8.486332	0.216943	-0.07217	1.9296	TRUE	2.35E-01
32126_at	0.93027	8.516674	0.141637	0.27681	1.58372	FALSE	6.44E-07
41433_at	0.93392	8.588553	0.219886	-0.08055	1.94838	TRUE	2.73E-01
38972_at	0.93978	8.705225	0.187878	0.07299	1.80658	FALSE	7.16E-03
408_at	0.9431	8.772028	0.112946	0.42201	1.46418	FALSE	8.62E-13
36508_at	0.94641	8.83914	0.198577	0.03026	1.86257	FALSE	2.37E-02
37560_at	0.9511	8.935112	0.073816	0.61055	1.29166	FALSE	6.93E-34
36245_at	0.96365	9.197081	0.108685	0.46222	1.46507	FALSE	9.54E-15
32932_at	0.96491	9.223803	0.115031	0.4342	1.49561	FALSE	6.23E-13
40456_at	0.9669	9.266164	0.133161	0.35255	1.58125	FALSE	4.85E-09
39940_at	0.97959	9.540914	0.066803	0.67139	1.2878	FALSE	1.39E-44
37208_at	0.98701	9.705323	0.03984	0.8032	1.17081	FALSE	2.15E-131
38131_at	0.99573	9.902161	0.109164	0.49209	1.49937	FALSE	9.37E-16
35410_at	0.99578	9.903301	0.175312	0.18696	1.80459	FALSE	1.70E-04
40775_at	0.99959	9.990564	0.102115	0.52848	1.47071	FALSE	1.59E-18
1369_s_at	1.01201	10.2804	0.181932	0.17265	1.85136	FALSE	3.36E-04
36686_at	1.01368	10.320007	0.042495	0.81763	1.20973	FALSE	1.16E-121
40240_at	1.02933	10.698675	0.058527	0.75931	1.29935	FALSE	3.91E-65
32365_at	1.02984	10.711246	0.118232	0.48436	1.57531	FALSE	3.83E-14
36906_at	1.04771	11.161177	0.147425	0.36756	1.72787	FALSE	1.50E-08
41215_s_at	1.0567	11.394624	0.13333	0.44157	1.67183	FALSE	2.87E-11
32521_at	1.06889	11.718985	0.068968	0.7507	1.38708	FALSE	4.50E-50
35730_at	1.06941	11.733025	0.116692	0.53104	1.60778	FALSE	6.29E-16

36782_s_at	1.07109	11.7785	0.1691	0.29094	1.85125	FALSE	3.01E-06
38028_at	1.07441	11.868887	0.091846	0.65067	1.49815	FALSE	1.65E-27
38026_at	1.07567	11.903372	0.088285	0.66836	1.48298	FALSE	4.77E-30
39610_at	1.08215	12.082311	0.108562	0.58129	1.58301	FALSE	2.66E-19
1363_at	1.09137	12.341558	0.071472	0.76163	1.42112	FALSE	1.53E-48
38469_at	1.09555	12.460917	0.062636	0.80658	1.38453	FALSE	2.12E-64
36311_at	1.09612	12.477282	0.121897	0.53374	1.6585	FALSE	3.06E-15
34335_at	1.10208	12.649693	0.090002	0.68685	1.51731	FALSE	2.25E-30
37892_at	1.10809	12.825964	0.23742	0.01273	2.20344	FALSE	3.85E-02
32728_at	1.11074	12.904465	0.058384	0.84138	1.3801	FALSE	1.34E-76
35717_at	1.12591	13.363186	0.153237	0.41894	1.83288	FALSE	2.55E-09
41124_r_at	1.13267	13.572817	0.158499	0.40142	1.86392	FALSE	1.13E-08
34637_f_at	1.21491	16.402498	0.128262	0.62316	1.80666	FALSE	3.46E-17
1787_at	1.22826	16.914533	0.129229	0.63205	1.82447	FALSE	2.54E-17
38957_at	1.24046	17.396425	0.157207	0.51517	1.96574	FALSE	3.80E-11
38151_at	1.24307	17.501288	0.052512	1.0008	1.48534	FALSE	8.81E-120
35648_at	1.24381	17.531134	0.083154	0.86017	1.62745	FALSE	1.75E-46
37399_at	1.25127	17.834872	0.141121	0.6002	1.90235	FALSE	9.51E-15
41073_at	1.27792	18.963566	0.216795	0.27772	2.27812	FALSE	4.74E-05
36197_at	1.28592	19.316125	0.2713	0.03425	2.53759	FALSE	2.70E-02
38086_at	1.31969	20.878053	0.077681	0.96131	1.67808	FALSE	1.26E-60
39119_s_at	1.32038	20.91125	0.108679	0.81898	1.82178	FALSE	7.30E-30
32112_s_at	1.33218	21.487209	0.074349	0.98917	1.6752	FALSE	1.08E-67
38407_r_at	1.42167	26.404017	0.096279	0.97747	1.86586	FALSE	3.06E-45
425_at	1.42731	26.749151	0.083485	1.04215	1.81248	FALSE	1.98E-61
32805_at	1.48142	30.298421	0.125272	0.90347	2.05937	FALSE	3.63E-28
34288_at	1.491	30.974193	0.076155	1.13966	1.84235	FALSE	2.97E-81
1466_s_at	1.56056	36.354653	0.13833	0.92236	2.19876	FALSE	2.05E-25
34363_at	1.64616	44.275146	0.16251	0.89641	2.39591	FALSE	5.15E-20

Supplementary Table 11. Cluster size and corresponding p-value calculation for networks generated from 500 random nodes

		Cluster size						
N	mean	1	2	3	4	5	6	7
50	2.0509	2209	5948	1312	338	108	53	18
75	2.7347	356	5393	2500	979	394	156	90
100	3.4988	35	3276	3230	1582	806	435	216
125	4.506	2	1504	3002	2051	1242	697	453
150	5.6657	0	602	2242	2005	1490	973	717
175	7.1349	0	177	1312	1747	1492	1227	933
200	8.9582	0	46	631	1305	1326	1127	972
225	11.1423	0	14	298	747	1010	993	910
250	13.6823	0	1	91	425	657	809	777
275	16.6524	0	0	49	200	400	547	610
300	20.2156	0	0	9	76	233	352	434
325	24.4903	0	0	1	29	93	197	280
350	29.234	0	0	0	10	41	105	174
375	35.5415	0	0	0	4	18	51	98
400	40.7957	0	0	0	0	5	23	43
425	48.5398	0	0	0	0	3	10	16
450	56.4274	0	0	0	0	0	1	6
475	64.3438	0	0	0	0	0	2	1
500	73.9702	0	0	0	0	0	0	2

	Cluster size +						
N	1	2	3	4	5	6	7
50	1	0.7791	0.1843	0.0531	0.0193	0.0085	0.0032
75	1	0.9644	0.4251	0.1751	0.0772	0.0378	0.0222
100	1	0.9965	0.6689	0.3459	0.1877	0.1071	0.0636
125	1	0.9998	0.8494	0.5492	0.3441	0.2199	0.1502
150	1	1	0.9398	0.7156	0.5151	0.3661	0.2688
175	1	1	0.9823	0.8511	0.6764	0.5272	0.4045
200	1	1	0.9954	0.9323	0.8018	0.6692	0.5565
225	1	1	0.9986	0.9688	0.8941	0.7931	0.6938
250	1	1	0.9999	0.9908	0.9483	0.8826	0.8017
275	1	1	1	0.9951	0.9751	0.9351	0.8804
300	1	1	1	0.9991	0.9915	0.9682	0.933
325	1	1	1	0.9999	0.997	0.9877	0.968
350	1	1	1	1	0.999	0.9949	0.9844
375	1	1	1	1	0.9996	0.9978	0.9927
400	1	1	1	1	1	0.9995	0.9972
425	1	1	1	1	1	0.9997	0.9987
450	1	1	1	1	1	1	0.9999
475	1	1	1	1	1	1	0.9998
500	1	1	1	1	1	1	1

8	9	10	11	12	13	14	15	16
2	7	3	1	1				
52	30	21	9	11	4	4	0	0
136	79	70	39	37	22	11	12	4
301	199	164	79	74	60	45	32	23
527	323	263	185	136	115	83	65	46
672	454	378	280	225	181	166	135	82
839	622	480	387	318	261	258	184	170
846	698	570	477	429	354	343	246	257
773	661	588	533	498	422	384	367	323
649	647	579	534	472	476	378	366	333
475	538	501	482	447	413	398	378	373
399	361	377	369	383	419	361	353	342
218	253	266	293	309	305	310	304	277
131	156	190	189	237	239	218	227	251
71	116	113	152	162	189	173	200	207
31	39	83	91	110	105	122	143	143
21	26	38	67	69	64	70	88	92
8	9	21	30	27	52	55	56	60
3	6	10	18	17	30	20	35	36

8	9	10	11	12	13	14	15	16
0.0014	0.0012	0.0005	0.0002	0.0001	0	0	0	0
0.0132	0.008	0.005	0.0029	0.002	0.0009	0.0005	0.0001	0.0001
0.042	0.0284	0.0205	0.0135	0.0096	0.0059	0.0037	0.0026	0.0014
0.1049	0.0748	0.0549	0.0385	0.0306	0.0232	0.0172	0.0127	0.0095
0.1971	0.1444	0.1121	0.0858	0.0673	0.0537	0.0422	0.0339	0.0274
0.3112	0.244	0.1986	0.1608	0.1328	0.1103	0.0922	0.0756	0.0621
0.4593	0.3754	0.3132	0.2652	0.2265	0.1947	0.1686	0.1428	0.1244
0.6028	0.5182	0.4484	0.3914	0.3437	0.3008	0.2654	0.2311	0.2065
0.724	0.6467	0.5806	0.5218	0.4685	0.4187	0.3765	0.3381	0.3014
0.8194	0.7545	0.6898	0.6319	0.5785	0.5313	0.4837	0.4459	0.4093
0.8896	0.8421	0.7883	0.7382	0.69	0.6453	0.604	0.5642	0.5264
0.94	0.9001	0.864	0.8263	0.7894	0.7511	0.7092	0.6731	0.6378
0.967	0.9452	0.9199	0.8933	0.864	0.8331	0.8026	0.7716	0.7412
0.9829	0.9698	0.9542	0.9352	0.9163	0.8926	0.8687	0.8469	0.8242
0.9929	0.9858	0.9742	0.9629	0.9477	0.9315	0.9126	0.8953	0.8753
0.9971	0.994	0.9901	0.9818	0.9727	0.9617	0.9512	0.939	0.9247
0.9993	0.9972	0.9946	0.9908	0.9841	0.9772	0.9708	0.9638	0.955
0.9997	0.9989	0.998	0.9959	0.9929	0.9902	0.985	0.9795	0.9739
0.9998	0.9995	0.9989	0.9979	0.9961	0.9944	0.9914	0.9894	0.9859

17	18	19	20	21	22	23	24	25
0	1							
6	3	1						
16	16	12	8	3	6	3	2	4
49	43	37	18	21	12	13	10	3
86	67	62	55	54	31	36	29	23
142	135	108	87	91	82	62	50	51
210	171	157	158	119	117	96	105	99
230	236	220	172	167	155	140	125	127
290	285	252	234	209	201	157	158	129
281	262	282	252	258	227	210	199	179
309	293	253	256	252	230	246	197	206
311	283	247	267	248	232	233	209	219
243	237	219	222	217	217	215	210	197
186	198	229	183	200	172	175	194	189
135	132	161	145	139	164	154	170	150
89	90	103	122	117	118	95	110	126
73	69	74	79	82	87	101	75	104
41	54	44	46	58	60	54	71	70

17	18	19	20	21	22	23	24	25
0	0	0	0	0	0	0	0	0
0.0001	0.0001	0	0	0	0	0	0	0
0.001	0.0004	0.0001	0	0	0	0	0	0
0.0072	0.0056	0.004	0.0028	0.002	0.0017	0.0011	0.0008	0.0006
0.0228	0.0179	0.0136	0.0099	0.0081	0.006	0.0048	0.0035	0.0025
0.0539	0.0453	0.0386	0.0324	0.0269	0.0215	0.0184	0.0148	0.0119
0.1074	0.0932	0.0797	0.0689	0.0602	0.0511	0.0429	0.0367	0.0317
0.1808	0.1598	0.1427	0.127	0.1112	0.0993	0.0876	0.078	0.0675
0.2691	0.2461	0.2225	0.2005	0.1833	0.1666	0.1511	0.1371	0.1246
0.376	0.347	0.3185	0.2933	0.2699	0.249	0.2289	0.2132	0.1974
0.4891	0.461	0.4348	0.4066	0.3814	0.3556	0.3329	0.3119	0.292
0.6036	0.5727	0.5434	0.5181	0.4925	0.4673	0.4443	0.4197	0.4
0.7135	0.6824	0.6541	0.6294	0.6027	0.5779	0.5547	0.5314	0.5105
0.7991	0.7748	0.7511	0.7292	0.707	0.6853	0.6636	0.6421	0.6211
0.8546	0.836	0.8162	0.7933	0.775	0.755	0.7378	0.7203	0.7009
0.9104	0.8969	0.8837	0.8676	0.8531	0.8392	0.8228	0.8074	0.7904
0.9458	0.9369	0.9279	0.9176	0.9054	0.8937	0.8819	0.8724	0.8614
0.9679	0.9606	0.9537	0.9463	0.9384	0.9302	0.9215	0.9114	0.9039
0.9823	0.9782	0.9728	0.9684	0.9638	0.958	0.952	0.9466	0.9395

26	27	28	29	30	31	32	33	34
0	0	0	1	0	0	0	0	1
9	2	4	2	1	0	0	3	0
20	17	12	13	8	6	2	4	3
43	34	30	20	19	22	14	11	13
71	72	52	44	29	34	34	35	23
112	83	102	82	100	64	66	63	45
146	128	104	113	133	87	88	95	97
183	174	173	162	137	119	108	107	111
196	192	156	186	150	146	145	157	144
228	192	179	193	183	183	151	153	159
203	180	170	184	143	169	163	148	138
181	182	151	173	171	150	179	148	157
162	142	132	151	148	125	151	129	143
140	130	125	126	123	127	102	102	119
108	97	100	77	109	113	98	112	103
72	72	74	73	85	72	71	80	87

26	27	28	29	30	31	32	33	34
0	0	0	0	0	0	0	0	0
0	0	0	0	0	0	0	0	0
0	0	0	0	0	0	0	0	0
0.0002	0.0002	0.0002	0.0002	0.0001	0.0001	0.0001	0.0001	0.0001
0.0022	0.0013	0.0011	0.0007	0.0005	0.0004	0.0004	0.0004	0.0001
0.0096	0.0076	0.0059	0.0047	0.0034	0.0026	0.002	0.0018	0.0014
0.0266	0.0223	0.0189	0.0159	0.0139	0.012	0.0098	0.0084	0.0073
0.0576	0.0505	0.0433	0.0381	0.0337	0.0308	0.0274	0.024	0.0205
0.1119	0.1007	0.0924	0.0822	0.074	0.064	0.0576	0.051	0.0447
0.1845	0.1699	0.1571	0.1467	0.1354	0.1221	0.1134	0.1046	0.0951
0.2741	0.2558	0.2384	0.2211	0.2049	0.1912	0.1793	0.1685	0.1578
0.3794	0.3598	0.3406	0.325	0.3064	0.2914	0.2768	0.2623	0.2466
0.4886	0.4658	0.4466	0.4287	0.4094	0.3911	0.3728	0.3577	0.3424
0.6014	0.5811	0.5631	0.5461	0.5277	0.5134	0.4965	0.4802	0.4654
0.682	0.6639	0.6457	0.6306	0.6133	0.5962	0.5812	0.5633	0.5485
0.7754	0.7592	0.745	0.7318	0.7167	0.7019	0.6894	0.6743	0.6614
0.8488	0.8348	0.8218	0.8093	0.7967	0.7844	0.7717	0.7615	0.7513
0.8935	0.8827	0.873	0.863	0.8553	0.8444	0.8331	0.8233	0.8121
0.9325	0.9253	0.9181	0.9107	0.9034	0.8949	0.8877	0.8806	0.8726

35	36	37	38	39	40	41	42	43
0	1							
3	1	2	5					
15	9	12	4	4	2	3	2	2
29	21	24	19	12	12	12	9	6
51	41	35	29	33	28	33	20	19
76	63	72	61	52	47	53	44	43
106	92	93	78	89	70	78	71	59
129	118	130	114	118	120	110	99	87
151	147	133	142	129	139	111	157	105
153	134	167	149	143	162	140	136	129
163	125	160	133	145	142	146	127	148
151	126	143	135	125	119	139	125	108
122	121	123	116	112	127	108	129	111
78	94	111	95	99	105	90	93	114
61	104	88	83	67	80	80	70	89

35	36	37	38	39	40	41	42	43
0	0	0	0	0	0	0	0	0
0	0	0	0	0	0	0	0	0
0	0	0	0	0	0	0	0	0
0	0	0	0	0	0	0	0	0
0.0001	0.0001	0	0	0	0	0	0	0
0.0011	0.0008	0.0007	0.0005	0	0	0	0	0
0.006	0.0045	0.0036	0.0024	0.002	0.0016	0.0014	0.0011	0.0009
0.0182	0.0153	0.0132	0.0108	0.0089	0.0077	0.0065	0.0053	0.0044
0.0402	0.0351	0.031	0.0275	0.0246	0.0213	0.0185	0.0152	0.0132
0.0854	0.0778	0.0715	0.0643	0.0582	0.053	0.0483	0.043	0.0386
0.1467	0.1361	0.1269	0.1176	0.1098	0.1009	0.0939	0.0861	0.079
0.2322	0.2193	0.2075	0.1945	0.1831	0.1713	0.1593	0.1483	0.1384
0.3265	0.3114	0.2967	0.2834	0.2692	0.2563	0.2424	0.2313	0.2156
0.4516	0.4363	0.4229	0.4062	0.3913	0.377	0.3608	0.3468	0.3332
0.5328	0.5165	0.504	0.488	0.4747	0.4602	0.446	0.4314	0.4187
0.6471	0.632	0.6194	0.6051	0.5916	0.5791	0.5672	0.5533	0.5408
0.7394	0.7272	0.7151	0.7028	0.6912	0.68	0.6673	0.6565	0.6436
0.8018	0.794	0.7846	0.7735	0.764	0.7541	0.7436	0.7346	0.7253
0.8639	0.8578	0.8474	0.8386	0.8303	0.8236	0.8156	0.8076	0.8006

44	45	46	47	48	49	50	51	52
2	3	0	0	1	0	0	0	0
11	5	1	6	6	2	1	0	2
15	4	21	4	13	11	6	8	7
44	45	32	30	20	16	13	19	17
66	61	65	43	62	44	31	40	30
67	93	81	89	81	62	62	63	58
118	93	118	92	99	103	78	87	66
120	132	110	125	118	103	120	134	96
125	143	120	119	102	118	104	88	123
139	123	121	135	122	119	140	121	116
127	122	145	124	131	111	129	116	121
116	91	94	111	81	119	126	96	105
78	95	92	83	69	89	78	73	99

44	45	46	47	48	49	50	51	52
0	0	0	0	0	0	0	0	0
0	0	0	0	0	0	0	0	0
0	0	0	0	0	0	0	0	0
0	0	0	0	0	0	0	0	0
0	0	0	0	0	0	0	0	0
0	0	0	0	0	0	0	0	0
0.0007	0.0005	0.0002	0.0002	0.0002	0.0001	0.0001	0.0001	0.0001
0.0038	0.0027	0.0022	0.0021	0.0015	0.0009	0.0007	0.0006	0.0006
0.0113	0.0098	0.0094	0.0073	0.0069	0.0056	0.0045	0.0039	0.0031
0.0343	0.0299	0.0254	0.0222	0.0192	0.0172	0.0156	0.0143	0.0124
0.0731	0.0665	0.0604	0.0539	0.0496	0.0434	0.039	0.0359	0.0319
0.1297	0.123	0.1137	0.1056	0.0967	0.0886	0.0824	0.0762	0.0699
0.2051	0.1933	0.184	0.1722	0.163	0.1531	0.1428	0.135	0.1263
0.3203	0.3083	0.2951	0.2841	0.2716	0.2598	0.2495	0.2375	0.2241
0.4039	0.3914	0.3771	0.3651	0.3532	0.343	0.3312	0.3208	0.312
0.53	0.5161	0.5038	0.4917	0.4782	0.466	0.4541	0.4401	0.428
0.6325	0.6198	0.6076	0.5931	0.5807	0.5676	0.5565	0.5436	0.532
0.7139	0.7023	0.6932	0.6838	0.6727	0.6646	0.6527	0.6401	0.6305
0.7917	0.7839	0.7744	0.7652	0.7569	0.75	0.7411	0.7333	0.726

53	54	55	56	57	58	59	60	61
0	0	1						
4								
2	0	3	2	7	3	2	1	1
11	12	17	8	8	8	9	1	7
39	27	28	23	23	16	15	16	15
51	56	44	49	33	33	35	38	31
77	79	71	64	73	71	55	51	55
109	108	92	104	84	94	77	82	74
144	116	98	130	111	108	102	89	88
113	136	132	106	115	117	108	130	122
113	118	103	122	108	104	134	93	127
120	120	112	111	96	112	127	111	90
93	93	80	98	104	97	83	103	85

53	54	55	56	57	58	59	60	61
0	0	0	0	0	0	0	0	0
0	0	0	0	0	0	0	0	0
0	0	0	0	0	0	0	0	0
0	0	0	0	0	0	0	0	0
0	0	0	0	0	0	0	0	0
0	0	0	0	0	0	0	0	0
0.0001	0.0001	0.0001	0	0	0	0	0	0
0.0004	0	0	0	0	0	0	0	0
0.0024	0.0022	0.0022	0.0019	0.0017	0.001	0.0007	0.0005	0.0004
0.0107	0.0096	0.0084	0.0067	0.0059	0.0051	0.0043	0.0034	0.0033
0.0289	0.025	0.0223	0.0195	0.0172	0.0149	0.0133	0.0118	0.0102
0.0641	0.059	0.0534	0.049	0.0441	0.0408	0.0375	0.034	0.0302
0.1197	0.112	0.1041	0.097	0.0906	0.0833	0.0762	0.0707	0.0656
0.2145	0.2036	0.1928	0.1836	0.1732	0.1648	0.1554	0.1477	0.1395
0.2997	0.2853	0.2737	0.2639	0.2509	0.2398	0.229	0.2188	0.2099
0.4164	0.4051	0.3915	0.3783	0.3677	0.3562	0.3445	0.3337	0.3207
0.5199	0.5086	0.4968	0.4865	0.4743	0.4635	0.4531	0.4397	0.4304
0.62	0.608	0.596	0.5848	0.5737	0.5641	0.5529	0.5402	0.5291
0.7161	0.7068	0.6975	0.6895	0.6797	0.6693	0.6596	0.6513	0.641

62	63	64	65	66	67	68	69	70
1	0	0	0	0	0	0	0	2
9	1	3	2	1	2	1	1	2
14	8	9	4	7	5	5	4	5
28	20	26	23	17	17	15	11	9
39	51	41	51	34	35	23	31	22
96	80	69	80	60	54	68	61	57
99	107	100	81	77	75	94	75	71
112	114	103	134	123	82	113	108	97
117	95	127	125	106	119	124	108	114
112	112	100	118	113	100	120	111	121
84	90	107	94	105	113	91	110	98

62	63	64	65	66	67	68	69	70
0	0	0	0	0	0	0	0	0
0	0	0	0	0	0	0	0	0
0	0	0	0	0	0	0	0	0
0	0	0	0	0	0	0	0	0
0	0	0	0	0	0	0	0	0
0	0	0	0	0	0	0	0	0
0	0	0	0	0	0	0	0	0
0	0	0	0	0	0	0	0	0
0.0003	0.0002	0.0002	0.0002	0.0002	0.0002	0.0002	0.0002	0.0002
0.0026	0.0017	0.0016	0.0013	0.0011	0.001	0.0008	0.0007	0.0006
0.0087	0.0073	0.0065	0.0056	0.0052	0.0045	0.004	0.0035	0.0031
0.0271	0.0243	0.0223	0.0197	0.0174	0.0157	0.014	0.0125	0.0114
0.0601	0.0562	0.0511	0.047	0.0419	0.0385	0.035	0.0327	0.0296
0.1321	0.1225	0.1145	0.1076	0.0996	0.0936	0.0882	0.0814	0.0753
0.2011	0.1912	0.1805	0.1705	0.1624	0.1547	0.1472	0.1378	0.1303
0.3085	0.2973	0.2859	0.2756	0.2622	0.2499	0.2417	0.2304	0.2196
0.4177	0.406	0.3965	0.3838	0.3713	0.3607	0.3488	0.3364	0.3256
0.5201	0.5089	0.4977	0.4877	0.4759	0.4646	0.4546	0.4426	0.4315
0.6325	0.6241	0.6151	0.6044	0.595	0.5845	0.5732	0.5641	0.5531

71	72	73	74	75	76	77	78	79
1	2	0	0	1				
5	1	0	1	4	3	2	1	4
11	14	11	3	6	10	3	5	6
26	30	23	23	15	17	17	13	15
52	55	49	35	44	42	42	32	35
79	73	76	64	60	65	59	54	62
102	101	96	90	91	79	91	71	89
116	95	105	108	109	99	100	109	91
104	93	111	121	107	95	120	113	115
118	112	93	122	104	102	97	117	113

71	72	73	74	75	76	77	78	79
0	0	0	0	0	0	0	0	0
0	0	0	0	0	0	0	0	0
0	0	0	0	0	0	0	0	0
0	0	0	0	0	0	0	0	0
0	0	0	0	0	0	0	0	0
0	0	0	0	0	0	0	0	0
0	0	0	0	0	0	0	0	0
0	0	0	0	0	0	0	0	0
0	0	0	0	0	0	0	0	0
0.0004	0.0003	0.0001	0.0001	0.0001	0	0	0	0
0.0026	0.0021	0.002	0.002	0.0019	0.0015	0.0012	0.001	0.0009
0.0105	0.0094	0.008	0.0069	0.0066	0.006	0.005	0.0047	0.0042
0.0274	0.0248	0.0218	0.0195	0.0172	0.0157	0.014	0.0123	0.011
0.0696	0.0644	0.0589	0.054	0.0505	0.0461	0.0419	0.0377	0.0345
0.1232	0.1153	0.108	0.1004	0.094	0.088	0.0815	0.0756	0.0702
0.2099	0.1997	0.1896	0.18	0.171	0.1619	0.154	0.1449	0.1378
0.3142	0.3026	0.2931	0.2826	0.2718	0.2609	0.251	0.241	0.2301
0.4194	0.409	0.3997	0.3886	0.3765	0.3658	0.3563	0.3443	0.333
0.5433	0.5315	0.5203	0.511	0.4988	0.4884	0.4782	0.4685	0.4568

80	81	82	83	84	85	86	87	88
2	0	1	1	0	0	0	0	0
4	5	3	3	8	1	4	2	0
6	11	9	11	6	8	5	6	4
46	28	16	16	21	16	19	13	15
56	39	37	42	27	27	34	25	38
75	63	71	62	73	53	60	64	49
91	74	96	100	87	87	95	70	76
96	111	115	92	108	114	97	95	90
111	103	118	127	120	115	118	126	117

80	81	82	83	84	85	86	87	88
0	0	0	0	0	0	0	0	0
0	0	0	0	0	0	0	0	0
0	0	0	0	0	0	0	0	0
0	0	0	0	0	0	0	0	0
0	0	0	0	0	0	0	0	0
0	0	0	0	0	0	0	0	0
0	0	0	0	0	0	0	0	0
0	0	0	0	0	0	0	0	0
0	0	0	0	0	0	0	0	0
0	0	0	0	0	0	0	0	0
0.0005	0.0003	0.0003	0.0002	0.0001	0.0001	0.0001	0.0001	0.0001
0.0036	0.0032	0.0027	0.0024	0.0021	0.0013	0.0012	0.0008	0.0006
0.0095	0.0089	0.0078	0.0069	0.0058	0.0052	0.0044	0.0039	0.0033
0.031	0.0264	0.0236	0.022	0.0204	0.0183	0.0167	0.0148	0.0135
0.064	0.0584	0.0545	0.0508	0.0466	0.0439	0.0412	0.0378	0.0353
0.1289	0.1214	0.1151	0.108	0.1018	0.0945	0.0892	0.0832	0.0768
0.221	0.2119	0.2045	0.1949	0.1849	0.1762	0.1675	0.158	0.151
0.3215	0.3119	0.3008	0.2893	0.2801	0.2693	0.2579	0.2482	0.2387
0.4455	0.4344	0.4241	0.4123	0.3996	0.3876	0.3761	0.3643	0.3517

89	90	91	92	93	94	95	96	97
1								
2	2	0	2					
4	0	5	3	5	3	1	0	1
14	7	13	6	9	8	7	8	6
33	22	24	25	26	14	18	17	16
53	36	50	35	31	35	31	31	29
74	80	73	69	67	72	60	54	61
91	98	95	89	90	69	81	85	83
88	115	99	102	99	93	109	111	84

89	90	91	92	93	94	95	96	97
0	0	0	0	0	0	0	0	0
0	0	0	0	0	0	0	0	0
0	0	0	0	0	0	0	0	0
0	0	0	0	0	0	0	0	0
0	0	0	0	0	0	0	0	0
0	0	0	0	0	0	0	0	0
0	0	0	0	0	0	0	0	0
0	0	0	0	0	0	0	0	0
0	0	0	0	0	0	0	0	0
0	0	0	0	0	0	0	0	0
0.0001	0	0	0	0	0	0	0	0
0.0006	0.0004	0.0002	0.0002	0	0	0	0	0
0.0029	0.0025	0.0025	0.002	0.0017	0.0012	0.0009	0.0008	0.0008
0.012	0.0106	0.0099	0.0086	0.008	0.0071	0.0063	0.0056	0.0048
0.0315	0.0282	0.026	0.0236	0.0211	0.0185	0.0171	0.0153	0.0136
0.0719	0.0666	0.063	0.058	0.0545	0.0514	0.0479	0.0448	0.0417
0.1434	0.136	0.128	0.1207	0.1138	0.1071	0.0999	0.0939	0.0885
0.2297	0.2206	0.2108	0.2013	0.1924	0.1834	0.1765	0.1684	0.1599
0.34	0.3312	0.3197	0.3098	0.2996	0.2897	0.2804	0.2695	0.2584

98	99	100	101	102	103	104	105	106

1	1	0	0	0	1	1	1	0
5	9	3	3	3	0	1	2	3
6	17	14	6	5	10	11	3	2
27	37	37	18	25	21	11	15	18
59	48	51	53	37	37	39	32	34
68	80	82	77	73	62	66	75	60
101	99	94	83	77	78	85	83	88

98	99	100	101	102	103	104	105	106
0	0	0	0	0	0	0	0	0
0	0	0	0	0	0	0	0	0
0	0	0	0	0	0	0	0	0
0	0	0	0	0	0	0	0	0
0	0	0	0	0	0	0	0	0
0	0	0	0	0	0	0	0	0
0	0	0	0	0	0	0	0	0
0	0	0	0	0	0	0	0	0
0	0	0	0	0	0	0	0	0
0	0	0	0	0	0	0	0	0
0	0	0	0	0	0	0	0	0
0	0	0	0	0	0	0	0	0
0.0007	0.0006	0.0005	0.0005	0.0005	0.0005	0.0004	0.0003	0.0002
0.0042	0.0037	0.0028	0.0025	0.0022	0.0019	0.0019	0.0018	0.0016
0.012	0.0114	0.0097	0.0083	0.0077	0.0072	0.0062	0.0051	0.0048
0.0388	0.0361	0.0324	0.0287	0.0269	0.0244	0.0223	0.0212	0.0197
0.0824	0.0765	0.0717	0.0666	0.0613	0.0576	0.0539	0.05	0.0468
0.1516	0.1448	0.1368	0.1286	0.1209	0.1136	0.1074	0.1008	0.0933
0.25	0.2399	0.23	0.2206	0.2123	0.2046	0.1968	0.1883	0.18

107	108	109	110	111	112	113	114	115
1	1							
4	0	3	1	1	1	2	0	0
5	4	1	8	6	1	3	2	4
16	16	23	12	18	5	6	8	11
37	40	29	25	26	20	25	18	21
54	54	41	39	43	44	42	39	34
78	90	77	91	55	64	67	65	60

107	108	109	110	111	112	113	114	115
0	0	0	0	0	0	0	0	0
0	0	0	0	0	0	0	0	0
0	0	0	0	0	0	0	0	0
0	0	0	0	0	0	0	0	0
0	0	0	0	0	0	0	0	0
0	0	0	0	0	0	0	0	0
0	0	0	0	0	0	0	0	0
0	0	0	0	0	0	0	0	0
0	0	0	0	0	0	0	0	0
0	0	0	0	0	0	0	0	0
0	0	0	0	0	0	0	0	0
0	0	0	0	0	0	0	0	0
0.0002	0.0001	0	0	0	0	0	0	0
0.0013	0.0009	0.0009	0.0006	0.0005	0.0004	0.0003	0.0001	0.0001
0.0046	0.0041	0.0037	0.0036	0.0028	0.0022	0.0021	0.0018	0.0016
0.0179	0.0163	0.0147	0.0124	0.0112	0.0094	0.0089	0.0083	0.0075
0.0434	0.0397	0.0357	0.0328	0.0303	0.0277	0.0257	0.0232	0.0214
0.0873	0.0819	0.0765	0.0724	0.0685	0.0642	0.0598	0.0556	0.0517
0.1712	0.1634	0.1544	0.1467	0.1376	0.1321	0.1257	0.119	0.1125

116	117	118	119	120	121	122	123	124
0	0	0	0	0	0	0	0	0
1	3	1	0	0	1	1	1	2
9	7	3	5	6	3	1	5	1
5	19	12	15	13	11	14	8	13
31	44	31	31	31	19	19	24	22
62	58	48	64	65	58	41	46	52

116	117	118	119	120	121	122	123	124
0	0	0	0	0	0	0	0	0
0	0	0	0	0	0	0	0	0
0	0	0	0	0	0	0	0	0
0	0	0	0	0	0	0	0	0
0	0	0	0	0	0	0	0	0
0	0	0	0	0	0	0	0	0
0	0	0	0	0	0	0	0	0
0	0	0	0	0	0	0	0	0
0	0	0	0	0	0	0	0	0
0	0	0	0	0	0	0	0	0
0	0	0	0	0	0	0	0	0
0	0	0	0	0	0	0	0	0
0	0	0	0	0	0	0	0	0
0.0001	0.0001	0.0001	0.0001	0.0001	0.0001	0.0001	0.0001	0.0001
0.0012	0.0011	0.0008	0.0007	0.0007	0.0007	0.0006	0.0005	0.0004
0.0064	0.0055	0.0048	0.0045	0.004	0.0034	0.0031	0.003	0.0025
0.0193	0.0188	0.0169	0.0157	0.0142	0.0129	0.0118	0.0104	0.0096
0.0483	0.0452	0.0408	0.0377	0.0346	0.0315	0.0296	0.0277	0.0253
0.1065	0.1003	0.0945	0.0897	0.0833	0.0768	0.071	0.0669	0.0623

125	126	127	128	129	130	131	132	133
0	0	1						
0	0	1	0	0	0	0	1	
2	3	6	1	4	3	1	0	1
8	11	9	5	4	7	6	3	1
21	18	17	12	17	13	14	11	11
29	41	33	31	25	33	31	29	20

125	126	127	128	129	130	131	132	133
0	0	0	0	0	0	0	0	0
0	0	0	0	0	0	0	0	0
0	0	0	0	0	0	0	0	0
0	0	0	0	0	0	0	0	0
0	0	0	0	0	0	0	0	0
0	0	0	0	0	0	0	0	0
0	0	0	0	0	0	0	0	0
0	0	0	0	0	0	0	0	0
0	0	0	0	0	0	0	0	0
0	0	0	0	0	0	0	0	0
0	0	0	0	0	0	0	0	0
0	0	0	0	0	0	0	0	0
0	0	0	0	0	0	0	0	0
0.0001	0.0001	0.0001	0	0	0	0	0	0
0.0002	0.0002	0.0002	0.0001	0.0001	0.0001	0.0001	0.0001	0
0.0024	0.0022	0.0019	0.0013	0.0012	0.0008	0.0005	0.0004	0.0004
0.0083	0.0075	0.0064	0.0055	0.005	0.0046	0.0039	0.0033	0.003
0.0231	0.021	0.0192	0.0175	0.0163	0.0146	0.0133	0.0119	0.0108
0.0571	0.0542	0.0501	0.0468	0.0437	0.0412	0.0379	0.0348	0.0319

134	135	136	137	138	139	140	141	142

1	0	1	1					
3	3	2	3	2	4	0	0	2
10	8	1	4	8	12	1	4	5
21	22	25	16	19	16	11	14	16

134	135	136	137	138	139	140	141	142
0	0	0	0	0	0	0	0	0
0	0	0	0	0	0	0	0	0
0	0	0	0	0	0	0	0	0
0	0	0	0	0	0	0	0	0
0	0	0	0	0	0	0	0	0
0	0	0	0	0	0	0	0	0
0	0	0	0	0	0	0	0	0
0	0	0	0	0	0	0	0	0
0	0	0	0	0	0	0	0	0
0	0	0	0	0	0	0	0	0
0	0	0	0	0	0	0	0	0
0	0	0	0	0	0	0	0	0
0	0	0	0	0	0	0	0	0
0	0	0	0	0	0	0	0	0
0	0	0	0	0	0	0	0	0
0.0003	0.0002	0.0002	0.0001	0	0	0	0	0
0.0029	0.0026	0.0023	0.0021	0.0018	0.0016	0.0012	0.0012	0.0012
0.0097	0.0087	0.0079	0.0078	0.0074	0.0066	0.0054	0.0053	0.0049
0.0299	0.0278	0.0256	0.0231	0.0215	0.0196	0.018	0.0169	0.0155

143	144	145	146	147	148	149	150	151

1	2	1	0	2	1	0	0	0
4	4	7	6	1	3	1	2	5
13	13	12	9	7	7	7	4	7

143	144	145	146	147	148	149	150	151
0	0	0	0	0	0	0	0	0
0	0	0	0	0	0	0	0	0
0	0	0	0	0	0	0	0	0
0	0	0	0	0	0	0	0	0
0	0	0	0	0	0	0	0	0
0	0	0	0	0	0	0	0	0
0	0	0	0	0	0	0	0	0
0	0	0	0	0	0	0	0	0
0	0	0	0	0	0	0	0	0
0	0	0	0	0	0	0	0	0
0	0	0	0	0	0	0	0	0
0	0	0	0	0	0	0	0	0
0	0	0	0	0	0	0	0	0
0	0	0	0	0	0	0	0	0
0	0	0	0	0	0	0	0	0
0	0	0	0	0	0	0	0	0
0.001	0.0009	0.0007	0.0006	0.0006	0.0004	0.0003	0.0003	0.0003
0.0044	0.004	0.0036	0.0029	0.0023	0.0022	0.0019	0.0018	0.0016
0.0139	0.0126	0.0113	0.0101	0.0092	0.0085	0.0078	0.0071	0.0067

152	153	154	155	156	157	158	159	160
1	0	0	1	1				
1	1	1	0	2	2	0	1	0
4	6	6	6	3	2	3	3	1

152	153	154	155	156	157	158	159	160
0	0	0	0	0	0	0	0	0
0	0	0	0	0	0	0	0	0
0	0	0	0	0	0	0	0	0
0	0	0	0	0	0	0	0	0
0	0	0	0	0	0	0	0	0
0	0	0	0	0	0	0	0	0
0	0	0	0	0	0	0	0	0
0	0	0	0	0	0	0	0	0
0	0	0	0	0	0	0	0	0
0	0	0	0	0	0	0	0	0
0	0	0	0	0	0	0	0	0
0	0	0	0	0	0	0	0	0
0	0	0	0	0	0	0	0	0
0	0	0	0	0	0	0	0	0
0	0	0	0	0	0	0	0	0
0	0	0	0	0	0	0	0	0
0.0003	0.0002	0.0002	0.0002	0.0001	0	0	0	0
0.0011	0.001	0.0009	0.0008	0.0008	0.0006	0.0004	0.0004	0.0003
0.006	0.0056	0.005	0.0044	0.0038	0.0035	0.0033	0.003	0.0027

161	162	163	164	165	166	167	168	169

0	0	1	0	1	0	0	0	0
3	3	5	4	3	0	0	0	1

161	162	163	164	165	166	167	168	169
0	0	0	0	0	0	0	0	0
0	0	0	0	0	0	0	0	0
0	0	0	0	0	0	0	0	0
0	0	0	0	0	0	0	0	0
0	0	0	0	0	0	0	0	0
0	0	0	0	0	0	0	0	0
0	0	0	0	0	0	0	0	0
0	0	0	0	0	0	0	0	0
0	0	0	0	0	0	0	0	0
0	0	0	0	0	0	0	0	0
0	0	0	0	0	0	0	0	0
0	0	0	0	0	0	0	0	0
0	0	0	0	0	0	0	0	0
0	0	0	0	0	0	0	0	0
0	0	0	0	0	0	0	0	0
0	0	0	0	0	0	0	0	0
0	0	0	0	0	0	0	0	0
0.0003	0.0003	0.0003	0.0002	0.0002	0.0001	0.0001	0.0001	0.0001
0.0026	0.0023	0.002	0.0015	0.0011	0.0008	0.0008	0.0008	0.0008

170	171	172	173	174	175	176	177	178

0	1							
0	1	0	3	0	0	0	1	0

170	171	172	173	174	175	176	177	178
0	0	0	0	0	0	0	0	0
0	0	0	0	0	0	0	0	0
0	0	0	0	0	0	0	0	0
0	0	0	0	0	0	0	0	0
0	0	0	0	0	0	0	0	0
0	0	0	0	0	0	0	0	0
0	0	0	0	0	0	0	0	0
0	0	0	0	0	0	0	0	0
0	0	0	0	0	0	0	0	0
0	0	0	0	0	0	0	0	0
0	0	0	0	0	0	0	0	0
0	0	0	0	0	0	0	0	0
0	0	0	0	0	0	0	0	0
0	0	0	0	0	0	0	0	0
0	0	0	0	0	0	0	0	0
0	0	0	0	0	0	0	0	0
0	0	0	0	0	0	0	0	0
0.0001	0.0001	0	0	0	0	0	0	0
0.0007	0.0007	0.0006	0.0006	0.0003	0.0003	0.0003	0.0003	0.0002

Supplement Table 12. Validation of genes encoding major activated hubs in glaucomatous ONHAs by quantitative RT-PCR.

	Gene	***AFFI gene ID#***	***micro-array FC***	***RT-PCR FC***	***RT-PCR p value***	***PCR primers forward/ reverse (5'-3')***
1	*AKR1C1*	37399_at	30.3	79	0.03	CGCCTGCAGAGGTTCCTAAA ; ACCTGCTCCTCATTATTGTATAAATGAG
2	*AKR1C3*	32805_at	17.8	10	0.01	CACCTCCAGAGGTTCCGAGAA; ACCTGCTCCTCATTATTGTATAAATGAG
3	*COL11A1*	37892_at	11	4.53	0.04	CTGGTATGATGTGTCATCAGGAAGTT; TCATAGCCTTTTCTGGACGTACAAC
4	*CLU*	36780_at	6.8	5.5	0.01	CTTGAGATGATACACGAGGCTCAG; TCATCGTCGCCTTCTCGTATG
5	*GPNMB*	38379_at	4.46	4.33	0.00	TCCGTGAGAATTCAGCATGG; AGCACATCATGAAATCGTTTGG
6	*SOD2*	34666_at	4.3	3.11	0.04	GGGCAGTGTGCGGCA; TCGTAGGGCAGGTCGGG
7	***FOS***	1916_s_at	4.1	5.1	0.01	CGGAGGAGGGAGCTGACTG; TTCTCCTTCAGCAGGTTGGC
8	*APOE*	608_at	3.75	17.90	0.04	CCGCCTCAAGAGCTGGTTC; CCACGGCAGCCTGCAC
9	***RELA***	41572_r_at	3.4	2.3	0.02	GCTCAGTGAGCCCATGGAAT; TGATGCTCTTGAAGGTCTCATATGTC
10	*GJA1*	2018_at	3.04	3.72	0.00	AAAAGAGTGGTGCCCAGGC; CACACCTTCCCTCCAGCAGT
11	***AR***	1577_at	3	4.1	0.03	AAAGAGCCGCTGAAGGGAAA; CCGAAGACGACAAGATGGACA
12	*CAPG*	38391_at	2.58	2.55	0.01	TGGACCTGGGCCAGAACA; TGCCCTGTCGCTCACTGTC
13	*RBP-1*	38634_at	2.3	6.00	0.02	GCCAACTTGCTGAAGCCAGACAAA; TTGCGGTCATCTATGCCTGTCAGA
14	*CALM1*	41143_at	1.7	1.65	0.02	TCACTGGGTCAGAACCCAACAGAA; AAAGACTCGGAATGCCTCACGGAT
15	*MYH10*	32838_at	1.57	1.52	0.37	TACAATGGCGCAGAGAACTGGACT; AGCTTGAGTGGCAGGGTTGTAGAT
16	*CASK*	31854_at	1.31	1.94	0.03	TTGTAGCTGGAGGACGTGTTGGAA; AAAGGCAAACAACCACTGAGCAGG
17	*DMPK*	37996_s_at	-1.62	-1.74	0.07	TTCTTCTTTGGCCTCGACTGGGAT; TCCTCCACCAAGTCGAAGTTGCAT
19	*LOXL2*	33127_at	-1.76	-1.74	0.13	ACTGCAAGCACACGGAGGA; AGGTTGAGAGGATGGCTCGA
19	*AMIGO2*	32919_at	-3.19	-2.10	0.05	AGAACGATTACACCTGTCGCCTGT; GCGCACGAAAGGAACCATTGATGA
20	*BMP1*	39407_at	-3.4	-1.76	0.05	CAGAGGGCAATGATGTGTGCAAGT; GAACTCCACGCGCATGTTGTTGTA
21	***PDGFA***	35703_at	-4.14	-3.18	0.10	
22	*AGC1*	39207_r_at	-26.53	-145	0.11	CCACCACCTACAAACGCAGAC; CTCCTGGGCTCAGCGTCC

Bolded genes represent major hubs on the disease network
