# Supplementary material for: Network analysis of human glaucomatous optic nerve head astrocytes
Source: BMC Med Genomics. 2009 May 9;2:24. doi: 10.1186/1755-8794-2-24 (PMC2705386; doi:10.1186/1755-8794-2-24)
Supplement: Additional file 2 — Original microarray gene expression data, listing AFFY and HUGO IDs for the genes that changed in excess of 2.5 fold in glaucomatous vs. normal donor eyes. [file 1755-8794-2-24-S2.pdf]

Group 1, up-regulated genes, 2.5 fold				
#	Affy tag	Gene Symbol	mean (ratios)	p.value
1	34354_at	FGFR2	3.36070197	0.0495
2	38038_at	LUM	2.56306724	0.0481
3	35174_i_at	EEF1A2	2.59131375	0.0476
4	40676_at	ITGB3BP	3.08077513	0.0463
5	1183_at	CCL17	2.50680181	0.0459
6	40346_at	KIAA0574	3.42586307	0.0418
7	37892_at	COL11A1	12.82596351	0.0385
8	1577_at	AR	2.98476401	0.0368
9	35372_r_at	IL8	3.59070726	0.0365
10	41677_at	IL15RA	2.97803639	0.0352
11	32349_at	ANXA10	2.87276433	0.0331
12	38497_at	ELK4	3.41467991	0.0272
13	36197_at	CHI3L1	19.31612468	0.027
14	39566_at	CHRFAM7A, CHRNA7	2.85838023	0.0263
15	36508_at	GPC4	8.83913974	0.0237
16	36924_r_at	SCG2	2.7689174	0.0186
17	35757_at	CPSF6	3.38984624	0.0167
18	33158_at	KAL1	3.07050635	0.013
19	34908_at	FLJ13946	2.66894731	0.013
20	1455_f_at	CYP2C9	3.17497273	0.0128
21	41636_at	RAB11FIP3	2.6906673	0.0102
22	33310_at	ABHD5	3.26339768	0.0094
23	38972_at	KCTD12	8.70522499	0.00716
24	37142_at	GFRA1	3.30354327	0.00586
25	36287_at	PIK3CG	2.80123794	0.00508
26	473_g_at	STAT5B	3.62343104	0.0049
27	32640_at	ICAM1	2.69991443	0.0044
28	33118_at	SEMA3B	2.59495601	0.00382
29	39506_at		2.77063936	0.0036
30	34853_at	FLRT2	5.66474024	0.00354
31	39449_at	SKP2	3.14340254	0.00327
32	37701_at	RGS2	3.29807099	0.00313
33	37168_at	LAMP3	5.00587499	0.00288
34	1232_s_at	IGFBP1	8.10811687	0.00277
35	38448_at	ACTN2	2.9201153	0.00208
36	33687_at	CG012	3.55524881	0.00193
37	40970_at	RFXAP	2.52429438	0.00181
38	535_s_at	NFKB2	3.37015597	0.0013
39	41481_at	ITGA2	3.12118851	0.00119
40	1042_at	RARRES1	3.47080329	0.000921
41	39227_at	PTPRT	2.78900954	0.000858
42	35959_at	ZNF365	3.02127321	0.000729
43	574_s_at	CASP1	2.72539842	0.000722
44	38370_at	TIAM1	3.31176872	0.000658
45	33284_at	MPO	2.53104578	0.00065
46	41044_at	WDR67	3.32177338	0.000649
47	38261_at	ABCC3	3.63730735	0.000463
48	1761_at	PDGFRL	2.98840875	0.000461
49	36681_at	APOD	3.42444346	0.000455

50	36460_at	POLR1C	2.73325405	0.000439
51	1369_s_at	IL8	10.28039969	0.000336
52	32812_at	DKFZP686A01247	5.9020108	0.000249
53	40001_r_at	VPS13A	3.83027539	0.000185
54	35410_at	CXCL6	9.90330148	0.00017
55	34631_at	EYA4	2.66956193	0.000134
56	1436_at	CYP2D6	2.50553227	0.000124
57	41533_at	C8orf72	2.77561995	0.00011
58	2042_s_at	MYB	3.10005929	0.000108
59	37944_at	GCH1	2.95508515	0.000107
60	40502_r_at	MYBPC1	4.95952404	0.0000878
61	40290_f_at	ST3GAL1	2.79724174	0.0000759
62	160021_r_at		4.8008706	0.0000732
63	34282_at	NFE2L3	2.93710857	0.0000632
64	37394_at	C7	3.61951183	0.0000607
65	704_at		3.7630501	0.0000598
66	36247_f_at	ADH1C	3.92473194	0.0000565
67	33772_at	PTGER4	4.5507188	0.000056
68	40834_at	PDZD2	4.03199514	0.0000552
69	41683_i_at	BCKDHB	3.59012855	0.0000506
70	32143_at	OSR2	4.72400121	0.0000501
71	41073_at	LGR5	18.96356567	0.0000474
72	33478_at	TNP2	2.72332831	0.0000429
73	1916_s_at	FOS	4.10374154	0.0000303
74	41764_at	APOC1	3.09136297	0.0000268
75	33767_at	NEFH	3.72597554	0.0000266
76	37637_at	RGS3	3.27416077	0.0000262
77	36890_at	PPL	3.90211442	0.0000242
78	40303_at	TFAP2C	2.57857638	0.0000224
79	1922_g_at		3.07673435	0.000018
80	942_at	RGN	5.10634319	0.0000162
81	35391_at	ADAM22	2.9471348	0.0000156
82	39470_at		3.14861819	9.47E-06
83	36623_at	ALPL	2.50685953	8.94E-06
84	39642_at	ELOVL2	2.63754574	8.67E-06
85	32320_at	ANXA13	2.86893032	8.29E-06
86	36244_at	ZNF239	3.01099476	7.18E-06
87	36269_at	ADAMTS3	2.69879564	0.0000065
88	34087_at	KCNK2	7.48117821	5.65E-06
89	36243_at	TLR1	2.57691445	3.21E-06
90	36782_s_at	IGF2	11.77850037	3.01E-06
91	34296_at	MID1	4.20794447	2.79E-06
92	40230_at	FRZB	3.13328572	2.57E-06
93	35822_at	CFB	7.09724831	2.42E-06
94	38351_at	KIAA1462	2.932851	2.34E-06
95	41572_r_at	REL	3.43803268	2.18E-06
96	40079_at		2.59645022	2.14E-06
97	38503_at	ALDH1B1	4.7319484	2.13E-06
98	32985_at	SALL1	4.05779404	1.79E-06
99	38323_at	CPVL	7.64029276	8.07E-07
100	41472_at	APOBEC3G	4.72117393	8.02E-07
101	32113_at	AIM1	5.15774658	7.46E-07

102	38124_at	MDK	4.68036799	6.74E-07
103	32126_at	FGF7	8.51667354	6.44E-07
104	37989_at	PTHLH	5.64884945	5.32E-07
105	35692_at	RIS1	2.91326506	2.84E-07
106	33945_at	CD40LG	3.10384482	2.54E-07
107	41303_r_at	C1orf63	3.01363048	2.4E-07
108	35000_at	TNFSF9	3.97502624	2.29E-07
109	39841_at	SLC16A6	2.68998588	2.26E-07
110	36548_at	KIAA0895	3.65039617	2.19E-07
111	35579_at	EXPH5	3.39953878	2.12E-07
112	40046_r_at	C18orf1	2.89247757	1.72E-07
113	36342_r_at	CFHR2	4.2177419	1.35E-07
114	34666_at	SOD2	4.33850398	1.33E-07
115	32605_r_at	RAB1A	2.71468848	1.05E-07
116	37852_at		2.51663365	9.07E-08
117	36617_at	ID1	7.09528754	7.13E-08
118	34334_at	EFNB2	3.69198561	6.34E-08
119	40952_at		3.18793898	5.81E-08
120	33732_at	AP4M1	2.53215333	5.68E-08
121	38152_at	LOH11CR2A	3.04593057	5.42E-08
122	38184_at	PAX2	3.23370204	5.31E-08
123	37534_at	CXADR	2.83896475	4.96E-08
124	34377_at	ATP1A2	8.02675487	4.73E-08
125	41009_at	TMEM16C	4.15623409	3.99E-08
126	41859_at	UST	2.66182807	3.76E-08
127	40313_at	BNC1	3.41224338	3.73E-08
128	33565_at	TSHB	2.71700226	3.47E-08
129	34304_s_at	SAT	2.93616191	2.77E-08
130	34770_at	MAP3K8	4.84283865	2.69E-08
131	195_s_at	CASP4	2.64326071	2.47E-08
132	35493_at	COL4A4	2.54894227	2.25E-08
133	40333_at	BMP4	2.54794471	2.12E-08
134	36906_at	CNR1	11.16117712	1.5E-08
135	37684_at	SLCO2B1	2.66428083	1.39E-08
136	41124_r_at	ENPP2	13.5728172	1.13E-08
137	565_at	SRD5A2	5.16095414	8.04E-09
138	36513_at	MFAP5	3.24541321	6.56E-09
139	31805_at	FGFR3	4.94265161	5.88E-09
140	40456_at	SLC39A8	9.26616437	4.85E-09
141	41660_at	CELSR1	3.78939607	4.8E-09
142	35719_at	PHLPP	2.5377568	2.95E-09
143	291_s_at	TACSTD2	2.9406274	2.61E-09
144	35717_at	ABCA8	13.36318559	2.55E-09
145	1669_at	WNT5A	5.89983682	2.18E-09
146	32685_at	RALGPS1	3.39304797	2.15E-09
147	1380_at	FGF7	7.73606304	2.04E-09
148	36867_at	NFASC	6.98875796	1.85E-09
149	36834_at	MOXD1	6.60967339	1.1E-09
150	1111_at	NCAM1	3.07737201	7.59E-10
151	2001_g_at	ATM	3.58872351	7.37E-10
152	41031_at	OMD	5.00437678	7.14E-10
153	40398_s_at	MEOX2	7.89714296	6.17E-10

154	40496_at	C1S	3.27868731	5.33E-10
155	41484_r_at	JUND	3.32874096	4.39E-10
156	37671_at	LAMA4	3.63161666	2.53E-10
157	185_at	NOVA1	3.33879689	1.63E-10
158	37203_at	CES1	5.85639013	1.53E-10
159	40330_at	PLCE1	3.41971568	1.37E-10
160	36496_at	IMPA2	2.60039906	1.28E-10
161	36049_at	RAB6B	3.52817404	1.02E-10
162	34693_at	ST6GALNAC2	5.87070151	9.18E-11
163	39061_at	BST2	5.0692067	7.82E-11
164	39654_at	ASPA	2.82520522	7.48E-11
165	37489_s_at	SLC4A3	3.33518555	7.13E-11
166	35624_at	ANKRD6	5.78895269	4.22E-11
167	38957_at	DCAMKL1	17.39642464	3.8E-11
168	40069_at	SVIL	2.90951098	3.74E-11
169	41215_s_at	ID2, ID2B	11.39462403	2.87E-11
170	31703_at	CHRNA6	4.12116497	2.39E-11
171	1234_at	EPHA3	6.53190711	2.12E-11
172	36661_s_at	CD14	6.81333903	2.02E-11
173	36618_g_at	ID1	4.82314146	8.43E-12
174	40321_at	IL1RL1	2.77619521	4.74E-12
175	36619_r_at	ID1	3.57981019	2.59E-12
176	1114_at	BMP4	2.56613795	2.51E-12
177	33468_at	DSG2	4.68252387	2.23E-12
178	35561_at	MCC	7.85705874	1.09E-12
179	1372_at	TNFAIP6	3.86527146	9.82E-13
180	38793_at	TNNT2	4.54380829	8.91E-13
181	408_at	CXCL1	8.77202781	8.62E-13
182	37156_at	ETV1	4.54035698	8.57E-13
183	32932_at	RP11-142I17.1	9.2238026	6.23E-13
184	41123_s_at	ENPP2	5.42238063	5.36E-13
185	36134_at	OLFM1	3.75897987	4.63E-13
186	32250_at	CFH	2.95488103	3.48E-13
187	529_at	DUSP5	2.56631522	1.34E-13
188	681_at	MMP8	3.61651275	1.25E-13
189	38856_at	ADAMTSL3	3.66251996	5.16E-14
190	32365_at	LUZP2	10.71124616	3.83E-14
191	31862_at	WNT5A	5.97049034	3.61E-14
192	39409_at	C1R	3.89260199	2.13E-14
193	1955_s_at	SMAD6	5.50541422	2.09E-14
194	37102_at	BRMS1	2.69302261	1.18E-14
195	36245_at	HTR2B	9.19708076	9.54E-15
196	37399_at	AKR1C3	17.83487213	9.51E-15
197	32963_s_at	RRAGD	7.31644311	7.36E-15
198	33502_at		3.09756194	7.3E-15
199	33199_at	ATP8A2	5.16321251	4.17E-15
200	36311_at	PDE1A	12.47728227	3.06E-15
201	34417_at	FLJ36166	3.45692534	2.18E-15
202	40199_at	MSX1	5.1752341	1.84E-15
203	34993_at	SGCD	3.58938464	1.43E-15
204	38131_at	PTGES	9.90216138	9.37E-16
205	32419_at	PDE1C	3.38836354	9.24E-16

206	40409_at	ALDH3A2	3.02900507	8.79E-16
207	35730_at	ADH1B	11.73302511	6.29E-16
208	39007_at	MMP2	3.48762675	5.7E-16
209	1016_s_at	IL13RA2	3.66749897	4.72E-16
210	36489_at	PRPS1	2.713876	4.52E-16
211	35752_s_at	PROS1	3.87418183	1.29E-16
212	40155_at	ABLIM1	2.65613412	7.78E-17
213	32249_at	CFH, CFHR1	5.83619777	4.57E-17
214	34637_f_at	ADH1A	16.40249825	3.46E-17
215	1787_at	CDKN1C	16.91453255	2.54E-17
216	37842_at	MDFIC	2.71887975	2.07E-17
217	33264_at	ENOSF1	5.39274643	1.35E-17
218	34990_at	SETBP1	2.53886729	2.33E-18
219	36070_at	KIAA1199	3.23668176	2.29E-18
220	40775_at	ITM2A	9.99056386	1.59E-18
221	1709_g_at	MAPK10	3.66319468	4.54E-19
222	32158_at	RAD9A	3.48473695	4.01E-19
223	33956_at	LY96	7.45589872	3.91E-19
224	39610_at	HOXB2	12.08231071	2.66E-19
225	1970_s_at	FGFR2	6.2056889	2.49E-19
226	36040_at	SH3BGR	7.58158485	9.06E-20
227	36490_s_at	PRPS1	3.25252018	8.01E-20
228	40049_at	DAPK1	4.4005808	7.64E-20
229	32552_at	RBP4	5.59100654	7.02E-20
230	34363_at	SEPP1	44.2751458	5.15E-20
231	41554_at	C8orf1	3.93079143	2.25E-20
232	36497_at	C14orf78	3.64854746	1.69E-20
233	35706_at	NR1D2	3.18581095	1.42E-20
234	36931_at	TAGLN	4.66143894	9.49E-21
235	823_at	CX3CL1	2.69438716	8E-21
236	33562_g_at	COL4A3	4.41845056	6.84E-21
237	38786_at	SVEP1	5.42025851	5.29E-21
238	38634_at	RBP1	7.94767318	3.2E-21
239	35352_at	ARNT2	3.23675629	2.06E-21
240	41505_r_at	MAF	2.52551528	1.57E-21
241	658_at	THBS2	2.81740966	1.27E-21
242	40401_at	DOK5	6.01090688	1.16E-21
243	38223_at	TBC1D8	4.88764889	5.53E-22
244	41504_s_at	MAF	2.53466169	3.06E-22
245	35724_at	PIR	2.66072506	2.94E-22
246	31850_at	GCLC	2.57252735	1.35E-22
247	36563_at	DKFZP761N09121	4.6994821	1.12E-22
248	1591_s_at	IGF2	8.15774264	1.28E-23
249	41871_at	PDPN	3.15413298	1.16E-23
250	32215_i_at	RHOBTB3	3.06796217	1.05E-23
251	38096_f_at	HLA-DPB1	7.73428195	6.41E-24
252	34213_at	WWC1	7.76855062	5.29E-24
253	38466_at	CTSK	3.27446234	3.37E-25
254	37902_at	CRYZ	2.88894983	3.3E-25
255	1466_s_at	FGF7	36.35465269	2.05E-25
256	38279_at	GNAZ	3.49462034	1.37E-25
257	1321_s_at	EMP1	3.55418476	7.1E-26

258	38028_at	LMO3	11.86888713	1.65E-27
259	35705_at	NR1D2	2.72075853	6.32E-28
260	38013_at	MTUS1	3.20066337	3.83E-28
261	32805_at	AKR1C1, AKR1C2	30.29842129	3.63E-28
262	33236_at	RARRES3	3.44492681	3.53E-28
263	32418_at	PDE1C	2.83752698	1.06E-28
264	1388_g_at	VDR	3.935682	2.68E-29
265	38968_at	SH3BP5	4.55522675	2.47E-29
266	38379_at	GPNMB	4.438743	1.84E-29
267	33358_at	PPM1H	2.50616696	1.45E-29
268	33299_at	GPR125	2.69792579	7.88E-30
269	39119_s_at	IL32	20.91125028	7.3E-30
270	38026_at	FBLN1	11.90337183	4.77E-30
271	37678_at	BAMBI	2.61619433	4.29E-30
272	34335_at	EFNB2	12.64969342	2.25E-30
273	37405_at	SELENBP1	3.92581654	6.83E-31
274	35073_at	SHOX	6.85251508	4.24E-31
275	41271_at	SLC7A8	2.81390869	1.24E-31
276	37241_at	PNMA2	3.45278869	6.34E-32
277	1664_at		4.09270083	3.38E-32
278	36963_at	PGD	2.69265058	4.83E-33
279	34760_at	CD302	4.1692698	1.52E-33
280	34991_at	SGCD	4.70890577	8.62E-34
281	37560_at	SNED1	8.93511199	6.93E-34
282	488_at	ITSN1	2.90689938	1.92E-35
283	128_at	CTSK	3.70458872	9.04E-39
284	40541_at	ASS	5.06314074	3.23E-39
285	41692_at	SYNJ1	3.03368162	8.46E-40
286	37762_at	EMP1	5.54076843	6.62E-40
287	129_g_at	CTSK	3.09963103	1.88E-40
288	40698_at	CLEC2B	7.7945312	1.35E-41
289	37248_at	CPZ	5.5032596	3.81E-43
290	1897_at	TGFBR3	5.92024937	1.08E-43
291	39940_at	RPESP	9.54091441	1.39E-44
292	38407_r_at	PTGDS	26.40401678	3.06E-45
293	35648_at	AUTS2	17.53113362	1.75E-46
294	1363_at	FGFR2	12.34155833	1.53E-48
295	39613_at	MAN1A1	3.81215017	2.69E-49
296	32521_at	SFRP1	11.71898504	4.5E-50
297	36780_at	CLU	6.76254238	7.1E-53
298	38855_s_at	OLFM1	5.44552806	1.38E-53
299	40239_g_at	MGC35048	4.5838458	4.88E-58
300	34009_at	CTAG2	5.51112168	1.03E-59
301	37805_at	SRPX2	5.21470806	6.33E-60
302	38086_at	IGSF3	20.87805321	1.26E-60
303	425_at	IFI27	26.74915085	1.98E-61
304	38469_at	TSPAN8	12.46091691	2.12E-64
305	36478_at	TTF1	5.23503995	1.63E-64
306	40240_at	GPRC5B	10.69867513	3.91E-65
307	32112_s_at	AIM1	21.4872086	1.08E-67
308	39310_at	BDKRB2	3.17022437	1.36E-69
309	39545_at	CDKN1C	3.20287508	5.96E-73

310	32728_at	AMPH	12.90446488	1.34E-76
311	34288_at	CMKOR1	30.97419299	2.97E-81
312	41385_at	EPB41L3	7.06333818	2.06E-84
313	1052_s_at	CEBPD	3.26911351	3.13E-85
314	41503_at	ZHX2	3.49148355	2.51E-85
315	41870_at	PDPN	4.38470117	5.5E-87
316	41260_at	DDX17	5.85409816	2.44E-88
317	1888_s_at	KIT	7.27964163	3.33E-101
318	38151_at	LOH11CR2A	17.50128753	8.81E-120
319	36686_at	ALDH1A3	10.32000719	1.16E-121
320	37208_at	PSPHL	9.70532314	2.15E-131
321	37209_g_at	PSPH	3.47760299	9.52E-240
322	38673_s_at	CDKN1C	6.79266192	2.92E-245

Group 2, up-regulated genes, fold change>2.5				
#	Affy tag	Gene Symbol	Mean (ratios)	P Value
1	40707_at	MPHOSPH9	2.501173185	0.001593543
2	1384_at	PPP2R2B	2.50176543	0.027405691
3	37601_at	SLC22A3	2.507865373	0.000023
4	37215_at	PYGL	2.510713085	0.000000118
5	41837_at	C14orf132	2.510737921	0.0299883
6	38255_at		2.512127334	0.000827581
7	35822_at	CFB	2.513170568	0.00004
8	36722_s_at	HNF4A	2.515800951	0.000000241
9	34995_at	CALCRL	2.521190999	0.000000897
10	31599_f_at	MAGEA3, MAGEA6	2.521846997	6.22E-10
11	36454_at	CA12	2.528124178	2.79E-08
12	32603_at	RPA2	2.530973587	0.000000344
13	38492_at	KYNU	2.531531385	1.05E-13
14	39319_at	LCP2	2.531767485	0.003699768
15	36282_at		2.532236188	0.01304383
16	41352_at	ST6GAL1	2.53254984	0.000000242
17	35237_at	COL21A1	2.534297242	6.43E-09
18	34048_at	GK2	2.534354109	0.0000277
19	35245_at	F5	2.534612008	0.002624751
20	32588_s_at	ZFP36L2	2.537201398	0.000000629
21	39639_s_at	TNP1	2.543797347	0.0000669
22	273_g_at	GRP	2.546622255	0.0000112
23	36680_at	AMY1A, AMY1B, AMY1C, AMY2A, AMY2B	2.548836537	0.001044927
24	902_at	EPHB2	2.550236692	0.000177799
25	35128_at	BRS3	2.563879093	0.009361356
26	37573_at	ANGPTL2	2.564180954	1.48E-16
27	31388_at	EPAG	2.567113426	0.000354808
28	34693_at	ST6GALNAC2	2.567134676	0.048956041
29	33158_at	KAL1	2.572802186	0.000155598
30	37151_at	FLJ43806	2.574096043	0.000124958
31	39144_at	NFATC1	2.577381607	0.001359451
32	38934_at		2.580164722	0.004332003
33	40319_at	DYNC1I1	2.580731638	2.24E-26
34	34993_at	SGCD	2.584400658	0.001998139
35	32359_at	WISP1	2.584639451	0.015206355
36	41857_r_at	ABHD3	2.58821779	0.003273047
37	33298_at	STRN	2.588991407	0.000195835
38	1156_at	SP1	2.59271267	0.0000645
39	38940_at	SPBC25	2.594051739	0.000000945
40	328_at		2.59451895	0.000000205
41	32666_at	CXCL12	2.597006383	0.00000184
42	37513_at	SCD	2.597019389	0.011570593
43	32988_at	CLCNKA	2.605651864	0.00000157
44	1050_at	MLANA	2.609128964	0.007842831
45	35717_at	ABCA8	2.610549701	4.68E-08
46	33056_at		2.613170543	0.001049822
47	37054_at	BPI	2.615133764	0.002727648
48	33958_at	SSH2	2.616439976	0.000915855
49	41085_at	POLE2	2.618214212	0.0000001

50	38220_at	DPYD	2.620176861	5.15E-08
51	35415_at	VIL1	2.620454848	0.004387872
52	37842_at	MDFIC	2.625536753	6.44E-08
53	41748_at	TNNC2	2.627074537	0.000490032
54	31553_at	ZNF272	2.629675366	0.00000157
55	37941_at	MYBPC2	2.637040689	0.000000225
56	37499_at	KIAA0408	2.640044851	0.000135859
57	1546_at	HYAL1	2.649303218	0.00000132
58	33039_at	TRAT1	2.65746941	0.04447695
59	32013_at	ZNF409	2.659986717	0.00000269
60	37887_at	CHEK2	2.660461533	0.000392343
61	37457_at	LRRTM2	2.662078409	0.004756818
62	39732_at	MAP7	2.662087481	0.000113206
63	33658_at	ZNF124	2.662127403	1.26E-09
64	38879_at	S100A12	2.664836634	3.45E-09
65	41071_at	SPINK2	2.667328713	1.5E-11
66	35593_at	AOC2	2.675044537	0.000204894
67	36134_at	OLFM1	2.689291133	0.000157511
68	35376_f_at	LOC284323	2.690052754	0.003400906
69	37032_at	NNMT	2.693468904	0.000486813
70	33073_at	PAK3	2.695036386	0.000208796
71	41730_at	MYL3	2.697266497	0.000161809
72	37116_at	PRKCBP1	2.699860736	0.001846307
73	33355_at	PBX1	2.700539562	4.53E-08
74	41674_at	FGF12	2.705032286	0.000472984
75	41870_at	PDPN	2.705085198	0.004212139
76	38326_at	G0S2	2.706318591	0.0000151
77	31627_f_at	LOC90586	2.708863472	0.00000147
78	36661_s_at	CD14	2.709725043	0.00176309
79	36243_at	TLR1	2.714051661	0.010278526
80	34342_s_at	SPP1	2.718792355	0.0000109
81	38486_at	TNNI1	2.719507388	0.00000368
82	40902_at	WWP1	2.722641118	0.006464572
83	33482_at	MYOD1	2.723693309	2.19E-17
84	32551_at	EFEMP1	2.723719957	7.96E-08
85	31480_f_at	MAGEA12	2.725375698	3.72E-08
86	35644_at	HEPH	2.725827546	3.86E-12
87	33945_at	CD40LG	2.730495751	4.81E-08
88	36042_at	NTRK2	2.731506743	0.000185856
89	35865_at	NR5A2	2.731842996	0.003752936
90	31571_at	POLR3G	2.733781285	0.00570278
91	35497_at	TSPAN2	2.738961193	7.29E-12
92	37081_at	DNAH7	2.739251373	0.000729447
93	33246_at		2.741503957	2.32E-19
94	1585_at	ERBB3	2.743734524	0.04852161
95	34249_at	EPM2A	2.749952903	2.06E-09
96	33236_at	RARRES3	2.751574056	0.00000385
97	38909_at	CYP27B1	2.752302477	0.0000126
98	1057_at	CRABP2	2.755739555	6.79E-15
99	34534_at	OPRM1	2.756661101	4.56E-16
100	34388_at	COL14A1	2.759332207	7.1E-13
101	35817_at	MBP	2.768519693	3.15E-08

102	36750_at	RIMBP2	2.76880747	0.000251503
103	34288_at	CMKOR1	2.77240734	0.014204171
104	33684_at	WNT2B	2.773111175	9.95E-12
105	38315_at	ALDH1A2	2.775590625	0.0000611
106	39559_at	KMO	2.778959529	3.31E-10
107	34002_at	HSD3B2	2.790791888	0.00000131
108	1683_at	WT1	2.795896851	0.000000116
109	34056_g_at	ACVR1B	2.79602655	0.000150155
110	35465_at	MCFP	2.797016846	0.004804249
111	41839_at	GAS1	2.797522241	0.0000336
112	2089_s_at	ERBB3	2.799144277	0.022970044
113	506_s_at	STAT5A	2.799901112	0.000574118
114	34296_at	MID1	2.803549258	0.000002
115	41345_at	PURA	2.807379505	0.00000107
116	35954_at	PDYN	2.80864039	3.95E-10
117	39610_at	HOXB2	2.810391964	0.04880314
118	878_s_at	PRL	2.816136237	1.23E-09
119	38391_at	CAPG	2.817506295	0.0000118
120	2094_s_at	FOS	2.82825273	4.11E-08
121	35867_at	SLC22A2	2.830944029	0.000415544
122	1819_at		2.836267692	5.76E-09
123	33717_at		2.8385498	0.00000926
124	40251_at		2.842183743	0.003591349
125	36808_at	PTPN22	2.848030035	5.33E-11
126	41679_at	C1orf21	2.856049466	0.0000471
127	34022_at	CXCL3	2.85664732	3.53E-52
128	35464_at	IL11	2.858740859	2.91E-12
129	1068_g_at	FLT3LG	2.860318921	0.0000203
130	40496_at	C1S	2.862335639	0.004975585
131	36250_at	ASPHD1	2.869068267	0.00098625
132	32391_g_at	FMO6	2.870093281	0.000000188
133	32997_at	PAGE1	2.871604479	0.00000209
134	33939_at	KCNA1	2.873810194	0.000000619
135	36362_at	SLC12A3	2.874859244	0.000330078
136	31891_at	CHI3L2	2.881084308	0.00000252
137	128_at	CTSK	2.889326036	0.0000348
138	38160_at	LY75	2.912228985	0.022426761
139	33567_at	VIP	2.922817978	0.0000835
140	1143_s_at		2.922990602	0.002797391
141	38302_at	ZNF202	2.930459118	0.009648376
142	39576_at	PDE6B	2.931923753	0.0000193
143	1063_s_at	TYRO3	2.932535114	0.000408015
144	38466_at	CTSK	2.93318291	0.00000229
145	35493_at	COL4A4	2.934094282	0.019445096
146	39850_at	ANK2	2.934354825	4.78E-15
147	37789_at	TADA2L	2.938895433	0.0000902
148	35647_at	XPC	2.941036206	0.000259616
149	33490_at	TXK	2.951071888	0.0000486
150	36385_at	YLPM1	2.951397309	0.0000967
151	39947_at	EFNA4	2.962419802	9.28E-30
152	41660_at	CELSR1	2.963198212	0.01130738
153	34208_at	SLC12A5	2.979444761	0.001344639

154	38914_at	LCMT2	2.984012754	0.00000035
155	37328_at	PLEK	2.984637065	0.000573752
156	40045_g_at	C18orf1	2.993244499	0.027916216
157	31764_at	MASS1	2.994735225	0.005092431
158	31579_at	C1orf46	3.001694172	1.17E-09
159	37187_at	CXCL2	3.005097948	0.00112366
160	33544_at	UNC5C	3.010315342	0.000000116
161	34111_s_at	DISC1	3.012323882	0.002745774
162	32773_at	HLA-DQA1	3.017251473	3.2E-12
163	41405_at	SFRP4	3.025215947	0.0000145
164	38972_at	KCTD12	3.026887752	0.00000057
165	32283_at	IDS	3.034748217	1.95E-10
166	34156_i_at	HIST1H2AL	3.042433035	1.08E-14
167	37482_at	AKR1B10	3.057859036	0.00052445
168	34615_at	KRT12	3.064892977	0.000000475
169	38717_at	METTL7A	3.066876657	0.00000134
170	34377_at	ATP1A2	3.071489641	0.001491955
171	40292_at	DBC1	3.087818217	0.002642052
172	37122_at	PLIN	3.088383461	0.00000168
173	38293_s_at	HOXD3	3.090463872	0.000554761
174	33575_at	CD226	3.092572203	0.000781733
175	35178_at	WIF1	3.097884295	0.0000659
176	35105_at	SCEL	3.106594829	0.00069525
177	40217_s_at	CDS1	3.110659845	0.000215841
178	36319_at	FOXF2	3.11254118	0.002098682
179	32112_s_at	AIM1	3.112692147	0.00069225
180	33894_at	NET1	3.127235813	3.27E-08
181	31679_at		3.130900594	0.000563361
182	38339_at	BBOX1	3.134601478	0.000781241
183	36794_at	ZNF250	3.13707667	0.000311946
184	32320_at	ANXA13	3.142711393	7.66E-11
185	37169_at	ZNF536	3.151465804	4.13E-12
186	39066_at	MFAP4	3.153846579	0.000222423
187	38510_at		3.160005169	0.000144873
188	36157_at	PDGFRA	3.1611152	0.00046774
189	39933_at	DUSP7	3.186987375	1.84E-18
190	38627_at	HLF	3.187550397	0.000205464
191	31360_at		3.189830189	8.74E-15
192	37078_at	CD3Z	3.191865113	0.000000104
193	37773_at	KIAA1005	3.200849753	0.005764397
194	38152_at	LOH11CR2A	3.20619301	0.020210882
195	38169_s_at	SLC7A2	3.214991973	0.001262795
196	31665_s_at	EIF2A	3.226967147	0.00000952
197	661_at	GAS1	3.22882911	0.000392184
198	32277_at	KCNA3	3.237527701	0.0000192
199	40085_s_at	TFCP2	3.252172372	0.0000346
200	34283_at	LOC283824	3.253659189	0.0000169
201	37461_at	ANGPT2	3.266556791	0.00000764
202	32812_at	DKFZP686A01247	3.277837715	0.041070255
203	40231_at	SMAD6	3.283526987	0.000167252
204	34232_at	NKTR	3.293151146	6.15E-21
205	683_at	OTC	3.303463335	0.00000107

206	31578_at	CFTR	3.307768789	0.001178089
207	32411_at	SP4	3.319704728	0.0000396
208	40429_r_at		3.321107887	0.007052489
209	37714_at	GAP43	3.322738075	0.0000303
210	38737_at	IGF1	3.348297781	0.002914553
211	40511_at	GATA3	3.363860653	0.0000481
212	41031_at	OMD	3.365920205	0.000630865
213	37103_at	BEAN	3.366376094	2.72E-08
214	36422_s_at	CHRD	3.373903152	8.89E-09
215	32783_at	FBLN2	3.378537432	0.0000201
216	40624_at	OLIG2	3.382876639	2.71E-10
217	39157_at		3.383313908	0.000117727
218	1145_g_at		3.383734029	2.51E-08
219	1625_at		3.410316545	0.000000465
220	34028_at	GPR19	3.410373877	0.037881319
221	34838_at		3.411727012	9.87E-16
222	32479_at	TNFRSF11A	3.434888192	0.0000206
223	33475_at		3.437588327	7.78E-28
224	921_s_at		3.449887325	3.51E-36
225	1227_g_at	ADAM17	3.465838582	4.5E-09
226	39203_at	C14orf132	3.469382783	1.17E-18
227	35649_at	CDO1	3.470542227	2.54E-17
228	1955_s_at	SMAD6	3.476420826	0.009636884
229	37467_at	IGHD	3.482848407	7.36E-21
230	32927_at	GIMAP5	3.48513193	0.000000401
231	38715_at	GYPB	3.527927746	0.00000137
232	33171_s_at	SEPT11	3.53942385	0.00000102
233	36618_g_at	ID1	3.54216272	0.00000148
234	37196_at	CDH5	3.546088646	1.94E-16
235	36341_s_at	CFHR1, CFHR2	3.547441203	0.000211182
236	34760_at	CD302	3.549276675	0.0000019
237	41385_at	EPB41L3	3.580622324	0.020897894
238	1024_at	CYP1A1	3.603143949	1.42E-11
239	38790_at	EPHX1	3.623925133	0.000268582
240	39310_at	BDKRB2	3.634807956	0.00000372
241	36890_at	PPL	3.645243809	0.0000316
242	41368_at	SLC13A3	3.653796765	1.21E-14
243	37091_g_at	ADAM3A	3.67819419	0.00000948
244	34939_r_at	GPC1	3.686139459	0.001802741
245	37671_at	LAMA4	3.699219374	0.00000043
246	32663_at	RHAG	3.713057126	0.0000113
247	AFFX-BioB-5_at		3.732955502	6.2E-26
248	35350_at	GALNAC4S-6ST	3.743512296	0.011609341
249	38611_at	LIPC	3.756629495	3.89E-27
250	38261_at	ABCC3	3.772544098	0.005853741
251	35638_at	RUNX1T1	3.787087888	0.00000187
252	31329_at		3.794957574	0.003830949
253	37248_at	CPZ	3.804919264	0.000694703
254	37247_at	TCF21	3.814881586	0.000000104
255	41348_at	IRX5	3.815706984	0.000269469
256	675_at	IFITM1	3.824529675	2.98E-09
257	1756_f_at	CYP3A4, CYP3A7	3.841558075	1.38E-10

258	34244_r_at	L3MBTL	3.842870982	0.00135024
259	40368_r_at	CPN2	3.842940656	0.000270598
260	34435_at	AQP9	3.879382586	3.94E-10
261	38874_s_at	RDS	3.95068365	0.000166622
262	35974_at	LRMP	3.961114406	0.000000157
263	36087_at	KIAA0409	3.967797724	1.25E-11
264	39409_at	C1R	3.97547038	0.000153562
265	37688_f_at	FCGR2A	3.976286246	0.005261256
266	708_at	PTH	3.98620929	2.78E-15
267	41245_at	GDF10	3.990352949	1.3E-13
268	39581_at	CSTA	3.991209402	2.31E-09
269	203_at	GATA2	4.000895388	0.00000805
270	39981_at	MSR1	4.012279991	1.74E-08
271	36797_at		4.051017964	0.00000224
272	31964_at	ITSN1	4.061115171	1.75E-19
273	39842_at	CRLF1	4.088166508	2.25E-12
274	1582_at	CEACAM5	4.132809889	0.0000094
275	37068_at	PLA2G7	4.133749258	0.00000689
276	813_at		4.161748877	5.89E-19
277	36508_at	GPC4	4.184355319	0.043046333
278	36617_at	ID1	4.190474691	0.0000131
279	33807_at	PLEKHA6	4.207622889	1.55E-09
280	39449_at	SKP2	4.217179648	0.000527083
281	32896_at		4.279721219	0.00000334
282	35570_at	SLCO1B1	4.279834684	0.000000468
283	38280_s_at	NTRK2	4.291028391	0.000225356
284	33572_at	ZNF165	4.29860935	0.004755043
285	999_at	CYP27A1	4.313969984	1.52E-13
286	38615_at	OLFM4	4.327211205	3.58E-16
287	1921_at		4.33134672	5.82E-10
288	35046_at	KIAA0256	4.334748899	2.96E-14
289	36836_at	NRP1	4.360488104	0.000000106
290	40356_at	CLCA2	4.388617858	3.89E-16
291	41630_at	IL7	4.402080012	0.00000121
292	36311_at	PDE1A	4.409018154	0.01328391
293	32620_at	FETUB	4.411266746	3.3E-11
294	35648_at	AUTS2	4.471286908	0.006107122
295	31352_at	RNASE1	4.476355853	3.85E-16
296	33408_at	DIP2C	4.479373537	0.012044292
297	40375_at	EGR3	4.480654427	2.11E-10
298	1363_at	FGFR2	4.494011061	0.001181005
299	796_i_at	CDKL1	4.552066469	0.000391857
300	34853_at	FLRT2	4.557959155	0.000178176
301	35226_at	EYA2	4.576153703	0.000000451
302	41433_at	VCAM1	4.616872849	0.027652737
303	1787_at	CDKN1C	4.665908673	0.002945638
304	36197_at	CHI3L1	4.705869994	0.007612204
305	33387_at	GAS7	4.716631379	0.00011319
306	1001_at	TIE1	4.73318579	0.000000764
307	34334_at	EFNB2	4.744131254	0.001180544
308	39875_at	WASL	4.754461599	5.29E-118
309	39320_at	CASP1	4.76235919	1E-39

310	33862_at	PPAP2B	4.771202507	4.35E-23
311	37787_at	LOC92973	4.78434502	8.05E-09
312	31430_at	TRAV20	4.83077798	6.61E-14
313	36695_at	OLFML1	4.846747947	0.00000546
314	1584_at	CDC25C	4.969299655	0.000000789
315	38406_f_at	PTGDS	4.97362574	0.001614245
316	38151_at	LOH11CR2A	5.035974229	0.002508805
317	38634_at	RBP1	5.069376322	0.000796312
318	37560_at	SNED1	5.105011785	3.85E-09
319	35541_r_at	KIAA0506	5.145674718	0.00000467
320	37006_at	IGJ	5.179006332	0.009170781
321	38013_at	MTUS1	5.194913027	0.000623068
322	38957_at	DCAMKL1	5.218723397	8.91E-08
323	36309_at	GDF8	5.236745245	0.000107736
324	36059_at	LRP4	5.320432063	9.83E-13
325	39578_at	HR	5.342045786	6.08E-192
326	39265_at	ARTS-1	5.371810602	1.65E-12
327	40929_at	WSB1	5.406161433	1.29E-13
328	31926_at	CYP7A1	5.497456518	0.00000015
329	40013_at	CLIC2	5.550035819	0.0000427
330	1285_at		5.722302114	1.92E-12
331	408_at	CXCL1	5.737793233	2.28E-11
332	36780_at	CLU	5.785159484	0.003672578
333	39395_at	THY1	5.86791393	0.003453026
334	583_s_at	VCAM1	6.068442622	0.032998718
335	32521_at	SFRP1	6.186332991	0.000307299
336	37952_at	ITGB3	6.200893751	5E-34
337	37863_at	EGR2	6.454117713	3.3E-15
338	35462_at	PLA2R1	6.73820347	9.19E-37
339	216_at	PTGDS	6.738846159	0.003484864
340	36906_at	CNR1	6.804416045	0.000732322
341	33182_at	NTRK2	7.106974857	0.00000262
342	32421_at	AKAP5	7.320962108	2.3E-31
343	37399_at	AKR1C3	7.444335027	0.000000479
344	35410_at	CXCL6	7.673198567	4.57E-14
345	35730_at	ADH1B	8.094517308	2.77E-12
346	34637_f_at	ADH1A	8.193313018	9.9E-13
347	32126_at	FGF7	8.287365575	0.00000674
348	38407_r_at	PTGDS	8.36157418	0.003454162
349	36247_f_at	ADH1C	8.672451305	1.08E-26
350	39593_at	FGL2	9.008025211	0.000000123
351	1380_at	FGF7	9.172887707	0.000000134
352	32805_at	AKR1C1, AKR1C2	9.652083137	0.001167111
353	1970_s_at	FGFR2	10.01073746	0.000000886
354	39618_at	HAPLN1	10.21495127	0.000959028
355	34363_at	SEPP1	10.38831029	0.000133288
356	38379_at	GPNMB	11.54970825	0.005712037
357	1466_s_at	FGF7	17.36303277	0.000000458

Group 1 and 2 combined, up-regulated genes, 2.5 fold				
#	Affy tag	Gene Symbol	Mean (ratios)	P Value
1	34770_at	MAP3K8	2.501906803	9.13E-14
2	41472_at	APOBEC3G	2.502119278	2.65E-10
3	36931_at	TAGLN	2.509607364	0.003079
4	39545_at	CDKN1C	2.511103851	1.69E-24
5	35554_f_at	ZIC2	2.523606847	4.14E-10
6	35046_at	KIAA0256	2.533846612	1.14E-07
7	35372_r_at	IL8	2.538474365	1.73E-11
8	33468_at	DSG2	2.538664027	6.19E-10
9	38497_at	ELK4	2.542766733	1.12E-12
10	38370_at	TIAM1	2.549849994	1.47E-09
11	41484_r_at	JUND	2.55526015	2.53E-06
12	36453_at	KBTBD11	2.560602198	3.75E-06
13	33118_at	SEMA3B	2.560633141	6.98E-09
14	704_at		2.562889318	2.7E-18
15	36695_at	OLFML1	2.568409591	2.84E-13
16	33264_at	ENOSF1	2.568762852	2.97E-13
17	33767_at	NEFH	2.56884883	9.08E-08
18	32215_i_at	RHOBTB3	2.570578448	3.93E-14
19	38279_at	GNAZ	2.571363412	6.31E-08
20	38326_at	G0S2	2.57943065	7.15E-10
21	36548_at	KIAA0895	2.582768524	2.92E-15
22	38391_at	CAPG	2.587503137	1.21E-32
23	41692_at	SYNJ1	2.59343385	2.48E-23
24	34666_at	SOD2	2.597617228	1.87E-16
25	1145_g_at		2.598594357	3.9E-19
26	35624_at	ANKRD6	2.60100894	2.18E-09
27	41503_at	ZHX2	2.601374304	8.49E-42
28	2001_g_at	ATM	2.606183087	1.64E-06
29	40398_s_at	MEOX2	2.611755915	1.58E-05
30	36243_at	TLR1	2.614704335	8.38E-14
31	40409_at	ALDH3A2	2.616328831	1.25E-32
32	37533_r_at	TTC9	2.618116695	5.57E-06
33	41260_at	DDX17	2.619020055	1.78E-07
34	33387_at	GAS7	2.621603656	5.94E-08
35	1388_g_at	VDR	2.62372664	1.01E-23
36	1321_s_at	EMP1	2.626738678	3.96E-18
37	33158_at	KAL1	2.639427511	9.55E-11
38	33862_at	PPAP2B	2.651658099	5.41E-29
39	37842_at	MDFIC	2.654122355	1.51E-47
40	41839_at	GAS1	2.654671767	3.62E-15
41	35493_at	COL4A4	2.661139124	4.66E-11
42	35462_at	PLA2R1	2.670409602	1.44E-11
43	37863_at	EGR2	2.674905231	0.000354
44	35752_s_at	PROS1	2.678427572	1.98E-31
45	38615_at	OLFM4	2.680262863	6.47E-19
46	40292_at	DBC1	2.682104279	1.74E-13
47	32985_at	SALL1	2.687405001	5.41E-09
48	38856_at	ADAMTSL3	2.699519786	4.1E-25
49	35638_at	RUNX1T1	2.704915005	5.28E-15

50	31703_at	CHRNA6	2.705661725	3.31E-09
51	32277_at	KCNA3	2.712840554	2.32E-11
52	1372_at	TNFAIP6	2.725025414	1.1E-20
53	38717_at	METTL7A	2.726778318	1.36E-26
54	32320_at	ANXA13	2.728019736	2.61E-11
55	37405_at	SELENBP1	2.749867954	4.02E-67
56	33199_at	ATP8A2	2.764668912	4.44E-11
57	41683_i_at	BCKDHB	2.772774191	3.6E-18
58	36497_at	C14orf78	2.774800621	6.08E-11
59	35000_at	TNFSF9	2.779230768	1.29E-11
60	203_at	GATA2	2.782229935	3.01E-12
61	38351_at	KIAA1462	2.782850956	6.38E-16
62	1052_s_at	CEBPD	2.787757205	1.88E-84
63	37142_at	GFRA1	2.789360252	1.76E-10
64	41123_s_at	ENPP2	2.795814288	2E-09
65	38874_s_at	RDS	2.832516354	6.54E-14
66	36059_at	LRP4	2.840885368	3.86E-18
67	40502_r_at	MYBPC1	2.84263089	1.35E-08
68	41748_at	TNNC2	2.847914875	3.77E-16
69	36496_at	IMPA2	2.858553534	4.46E-08
70	34388_at	COL14A1	2.872397744	1.16E-12
71	40330_at	PLCE1	2.873792262	5.03E-15
72	33945_at	CD40LG	2.883286842	9.7E-34
73	39981_at	MSR1	2.888447837	9.48E-13
74	34213_at	WWC1	2.889517333	5.53E-09
75	31926_at	CYP7A1	2.894958904	6.84E-08
76	33431_at	FMOD	2.899928606	1.57E-09
77	38466_at	CTSK	2.906806348	8.22E-19
78	37187_at	CXCL2	2.910264991	1.63E-09
79	1227_g_at	ADAM17	2.911256807	4.44E-12
80	1232_s_at	IGFBP1	2.913047485	8.33E-08
81	216_at	PTGDS	2.914884521	6.79E-06
82	40049_at	DAPK1	2.91611623	5.87E-15
83	33807_at	PLEKHA6	2.920283677	2.49E-27
84	34354_at	FGFR2	2.920901818	1.34E-16
85	36619_r_at	ID1	2.929346968	9.06E-30
86	41554_at	C8orf1	2.94840891	2.53E-19
87	38124_at	MDK	2.948586467	1.17E-21
88	1916_s_at	FOS	2.972957762	1.65E-18
89	32143_at	OSR2	2.974338406	4.89E-17
90	34993_at	SGCD	2.978937695	4.6E-21
91	37749_at	MEST	2.97935426	2.06E-09
92	36718_s_at	PDK3	2.97996038	1.76E-06
93	34820_at	PTN	2.981152048	1.19E-10
94	38790_at	EPHX1	2.983899275	2.61E-15
95	37156_at	ETV1	2.986963138	1.97E-10
96	37762_at	EMP1	2.987114988	1.35E-14
97	1586_at	IGFBP3	2.993515692	0.000205
98	33182_at	NTRK2	3.009768156	4.03E-07
99	36867_at	NFASC	3.022826793	5.32E-09
100	36563_at	DKFZP761N09121	3.024287114	3.18E-13
101	38223_at	TBC1D8	3.037804254	2.39E-38

102	2018_at	GJA1	3.042801047	2.56E-06
103	1709_g_at	MAPK10	3.05033401	6.6E-28
104	36454_at	CA12	3.074881758	9.64E-11
105	37319_at	IGFBP3	3.086967826	6.74E-05
106	38968_at	SH3BP5	3.091301666	1.49E-38
107	40496_at	C1S	3.141354355	6.11E-19
108	33236_at	RARRES3	3.146400359	1.12E-46
109	39320_at	CASP1	3.166385026	2.62E-29
110	128_at	CTSK	3.186830295	1.07E-32
111	31805_at	FGFR3	3.191608485	2.1E-12
112	32365_at	LUZP2	3.199926316	4.12E-09
113	38152_at	LOH11CR2A	3.201572891	1.31E-12
114	38836_at	NPTXR	3.20183832	2.18E-07
115	1897_at	TGFBR3	3.203799144	2.64E-20
116	36134_at	OLFM1	3.219100511	1.27E-31
117	32421_at	AKAP5	3.228717912	1.57E-09
118	661_at	GAS1	3.240590742	3.47E-15
119	38855_s_at	OLFM1	3.256074297	4.13E-17
120	1664_at		3.262254163	4.6E-14
121	36319_at	FOXF2	3.267534585	1.94E-12
122	32113_at	AIM1	3.276177226	3.46E-14
123	40698_at	CLEC2B	3.288794027	4.52E-15
124	38793_at	TNNT2	3.299807627	5.19E-08
125	39875_at	WASL	3.307917556	2.65E-36
126	41245_at	GDF10	3.340515721	8.76E-16
127	33572_at	ZNF165	3.343844623	2.48E-11
128	36478_at	TTF1	3.351040132	4.91E-18
129	40231_at	SMAD6	3.366307968	6.09E-17
130	40013_at	CLIC2	3.378018946	5.03E-14
131	41710_at	LOC54103	3.385342044	1.83E-09
132	35561_at	MCC	3.411705503	2.89E-20
133	39310_at	BDKRB2	3.457642765	5.14E-42
134	38503_at	ALDH1B1	3.459127022	9.91E-15
135	38218_at	GCNT1	3.472566962	8.97E-12
136	41870_at	PDPN	3.486397881	1.19E-29
137	37006_at	IGJ	3.519771432	2.07E-08
138	40199_at	MSX1	3.524272816	3.74E-13
139	38786_at	SVEP1	3.55107994	1.86E-15
140	41660_at	CELSR1	3.562604286	2.84E-15
141	39449_at	SKP2	3.577265639	3.9E-17
142	40401_at	DOK5	3.592257656	1.16E-25
143	34296_at	MID1	3.59395049	1.35E-27
144	40456_at	SLC39A8	3.599164421	1.62E-14
145	35073_at	SHOX	3.603869489	5.9E-12
146	39581_at	CSTA	3.615100107	1.53E-19
147	942_at	RGN	3.619464597	1.42E-12
148	32249_at	CFH, CFHR1	3.631821339	3.56E-21
149	37671_at	LAMA4	3.643974398	1.88E-37
150	33562_g_at	COL4A3	3.696331318	2.63E-14
151	38673_s_at	CDKN1C	3.71267685	1.23E-16
152	36890_at	PPL	3.715753684	1.87E-24
153	38013_at	MTUS1	3.751749886	1.37E-14

154	39061_at	BST2	3.753336308	2.63E-09
155	1669_at	WNT5A	3.774846037	7.1E-14
156	1369_s_at	IL8	3.783236901	4.46E-08
157	37208_at	PSPHL	3.811948872	5.03E-07
158	40240_at	GPRC5B	3.829035634	4.54E-13
159	39842_at	CRLF1	3.854121451	3.53E-20
160	34760_at	CD302	3.855783708	1.36E-51
161	675_at	IFITM1	3.865873128	1.55E-18
162	40541_at	ASS	3.881711391	5.58E-16
163	39409_at	C1R	3.885226533	6.53E-28
164	38261_at	ABCC3	3.980345114	1.42E-12
165	32783_at	FBLN2	3.984433244	1.69E-18
166	38026_at	FBLN1	3.986015811	5.6E-14
167	38096_f_at	HLA-DPB1	3.986311284	3.15E-13
168	36245_at	HTR2B	4.019862044	6.26E-19
169	36834_at	MOXD1	4.028874442	1.24E-18
170	41031_at	OMD	4.042922544	8.45E-23
171	31862_at	WNT5A	4.064475968	7.44E-15
172	34334_at	EFNB2	4.09472746	1.53E-18
173	39940_at	RPESP	4.228345982	7.51E-16
174	40775_at	ITM2A	4.25095979	8.48E-15
175	34693_at	ST6GALNAC2	4.321782205	2.27E-13
176	34087_at	KCNK2	4.367185436	7.24E-23
177	35822_at	CFB	4.445343097	4.4E-24
178	36618_g_at	ID1	4.446736979	1.23E-27
179	33956_at	LY96	4.447226788	1.61E-27
180	37892_at	COL11A1	4.462640762	6.42E-08
181	38323_at	CPVL	4.471284828	1.78E-20
182	1955_s_at	SMAD6	4.548542448	2.64E-20
183	32812_at	DKFZP686A01247	4.581094304	1.22E-12
184	32728_at	AMPH	4.584982559	3.63E-14
185	36661_s_at	CD14	4.714448501	1.47E-22
186	36040_at	SH3BGR	4.728811364	3.59E-17
187	37248_at	CPZ	4.779974481	2.12E-32
188	1591_s_at	IGF2	4.785073444	3.04E-19
189	41124_r_at	ENPP2	4.804082081	2.15E-10
190	38131_at	PTGES	4.825624413	5.06E-19
191	38469_at	TSPAN8	4.847265991	2.48E-17
192	41385_at	EPB41L3	4.929711399	7.09E-18
193	34853_at	FLRT2	4.977740521	1.76E-18
194	36309_at	GDF8	5.056058102	1.78E-14
195	39593_at	FGL2	5.089283042	1.24E-17
196	39119_s_at	IL32	5.098498584	8.76E-14
197	36686_at	ALDH1A3	5.130092572	2.61E-25
198	34335_at	EFNB2	5.309828241	1.21E-20
199	37989_at	PTHLH	5.311275094	2.37E-09
200	34377_at	ATP1A2	5.360618317	3.25E-22
201	36247_f_at	ADH1C	5.554507685	4.4E-49
202	38972_at	KCTD12	5.574577993	4.29E-18
203	425_at	IFI27	5.603594689	5.23E-08
204	36617_at	ID1	5.707591848	1.44E-29
205	38028_at	LMO3	5.749636767	5.71E-19

206	41073_at	LGR5	6.007704598	6.43E-10
207	41215_s_at	ID2, ID2B	6.008015494	1.21E-16
208	39610_at	HOXB2	6.112362956	1.95E-20
209	41433_at	VCAM1	6.317853352	1.41E-10
210	38634_at	RBP1	6.432416096	5.21E-30
211	36782_s_at	IGF2	6.448947835	1.49E-12
212	35717_at	ABCA8	6.479159272	3.74E-27
213	38379_at	GPNMB	6.528589332	1.67E-11
214	36780_at	CLU	6.625388141	4.65E-22
215	408_at	CXCL1	6.761795079	7.33E-36
216	36508_at	GPC4	6.986157397	6.07E-10
217	37560_at	SNED1	7.032827723	5.01E-70
218	583_s_at	VCAM1	7.183521137	5.15E-10
219	1363_at	FGFR2	7.845589969	3.99E-38
220	36311_at	PDE1A	8.026918344	3.31E-21
221	38086_at	IGSF3	8.02987286	1.51E-22
222	1970_s_at	FGFR2	8.085935412	1.51E-36
223	36906_at	CNR1	8.371836884	2.22E-20
224	35410_at	CXCL6	8.494052247	3.29E-33
225	32521_at	SFRP1	8.694766612	3.02E-39
226	32126_at	FGF7	8.971453449	1.11E-26
227	1380_at	FGF7	9.058093891	2.5E-30
228	1787_at	CDKN1C	9.351761255	1.52E-28
229	32112_s_at	AIM1	9.423139382	5.76E-36
230	38957_at	DCAMKL1	9.62150343	1.22E-34
231	35730_at	ADH1B	9.717170186	7.61E-60
232	35648_at	AUTS2	9.758627147	2.33E-30
233	36197_at	CHI3L1	9.826160509	2.08E-14
234	38151_at	LOH11CR2A	10.51311885	9.01E-33
235	34288_at	CMKOR1	11.01744342	3.02E-29
236	34637_f_at	ADH1A	11.71961419	2.21E-60
237	37399_at	AKR1C3	12.26801027	1.01E-42
238	38407_r_at	PTGDS	17.14256039	2.47E-25
239	32805_at	AKR1C1, AKR1C2	18.77231647	7.76E-32
240	34363_at	SEPP1	24.49503549	1.64E-33
241	1466_s_at	FGF7	29.88026611	8.54E-33

Group 1, up-regulated genes (HUGO nomenclature), fold change > 2.5					
#	Gene Symbol	Protein	Protein name	Signal	P_value
1	ABCA8	ABCA8	ATP-binding cassette, sub-family A (ABC1), member 8	13.36	2.6E-09
2	ABCC3	MRP3_HUMAN	Canalicular multispecific organic anion transporter 2	3.637	0.00046
3	ABHD5	ABHD5_HUMAN	Abhydrolase domain-containing protein 5	3.263	0.0094
4	ABLIM1	ABLM1_HUMAN	Actin-binding LIM protein 1	2.656	7.8E-17
5	ACTN2	ACTN2_HUMAN	Alpha-actinin-2	2.92	0.00208
6	ADAM22	ADA22_HUMAN	ADAM 22 precursor	2.947	1.6E-05
7	ADAMTS3	ATS3_HUMAN	ADAMTS-3 precursor	2.699	6.5E-06
8	ADAMTSL3			3.663	5.2E-14
9	ADH1A	ADH1A_HUMAN	Alcohol dehydrogenase 1A	16.4	3.5E-17
10	ADH1B	ADH1B_HUMAN	Alcohol dehydrogenase 1B	11.73	6.3E-16
11	ADH1C	ADH1G_HUMAN	Alcohol dehydrogenase 1C	3.925	5.7E-05
12	AIM1	AIM1_HUMAN	Absent in melanoma 1 protein	21.49	1.1E-67
13	AKR1C1	AK1C1_HUMAN	Aldo-keto reductase family 1 member C1	30.3	3.6E-28
14	AKR1C2	AK1C2_HUMAN	Aldo-keto reductase family 1 member C2	30.3	3.6E-28
15	AKR1C3	AK1C3_HUMAN	Aldo-keto reductase family 1 member C3	17.83	9.5E-15
16	ALDH1A3	AL1A3_HUMAN	Aldehyde dehydrogenase 1A3	10.32	1E-121
17	ALDH1B1	AL1B1_HUMAN	Aldehyde dehydrogenase X, mitochondrial precursor	4.732	2.1E-06
18	ALDH3A2	AL3A2_HUMAN	Fatty aldehyde dehydrogenase	3.029	8.8E-16
19	ALPL	PPBT_HUMAN	Alkaline phosphatase, tissue-nonspecific isozyme precursor(EC 3.1.3.1	2.507	8.9E-06
20	AMPH	AMPH_HUMAN	Amphiphysin	12.9	1.3E-76
21	ANKRD6	ANKR6_HUMAN	Ankyrin repeat domain-containing protein 6	5.789	4.2E-11
22	ANXA10	ANX10_HUMAN	Annexin A10	2.873	0.0331
23	ANXA13	ANX13_HUMAN	Annexin A13	2.869	8.3E-06
24	AP4M1	AP4M1_HUMAN	AP-4 complex subunit mu-1	2.532	5.7E-08
25	APOBEC3G	ABC3G_HUMAN	DNA dC->dU-editing enzyme APOBEC-3G	4.721	8E-07
26	APOC1	APOC1_HUMAN	Apolipoprotein C-I precursor	3.091	2.7E-05
27	APOD	APOD_HUMAN	Apolipoprotein D precursor	3.424	0.00046
28	AR	ANDR_HUMAN	Androgen receptor	2.985	0.0368
29	ARNT2	ARNT2_HUMAN	Aryl hydrocarbon receptor nuclear translocator 2	3.237	2.1E-21
30	ASPA	ACY2_HUMAN	Aspartoacylase	2.825	7.5E-11
31	ASS	ASSY_HUMAN	Argininosuccinate synthase	5.063	3.2E-39
32	ATM	ATM_HUMAN	Serine-protein kinase ATM	3.589	7.4E-10
33	ATP1A2	AT1A2_HUMAN	Sodium/potassium-transporting ATPase alpha-2 chain precursor(EC 3.6.3.9	8.027	4.7E-08
34	ATP8A2	AT8A2_HUMAN	Probable phospholipid-transporting ATPase IB	5.163	4.2E-15
35	AUTS2	AUTS2_HUMAN	Autism susceptibility gene 2 protein	17.53	1.8E-46
36	BAMBI	BAMBI_HUMAN	BMP and activin membrane-bound inhibitor homolog precursor	2.616	4.3E-30
37	BCKDHB	ODBB_HUMAN	2-oxoisovalerate dehydrogenase subunit beta, mitochondrial precursor(EC 1.2.4.4	3.59	5.1E-05
38	BDKRB2	BKRB2_HUMAN	B2 bradykinin receptor	3.17	1.4E-69
39	BMP4	BMP4_HUMAN	Bone morphogenetic protein 4 precursor	2.566	2.5E-12
40	BNC1	BNC1_HUMAN	Zinc finger protein basonuclin-1	3.412	3.7E-08
41	BRMS1	BRMS1_HUMAN	Breast cancer metastasis-suppressor 1	2.693	1.2E-14
42	BST2	BST2_HUMAN	Bone marrow stromal antigen 2	5.069	7.8E-11

43	C14orf78	C14orf78	chromosome 14 open reading frame 78	3.649	1.7E-20
44	C18orf1	CR001_HUMAN	Protein C18orf1	2.892	1.7E-07
45	C1R	C1R_HUMAN	Complement C1r subcomponent precursor	3.893	2.1E-14
46	C1S	PRP5_HUMAN	Basic proline-rich peptide IB-1	3.279	5.3E-10
		C1S_HUMAN	Complement C1s subcomponent precursor	3.279	5.3E-10
47	C1orf63	C1orf63		3.014	2.4E-07
48	C7	CO7_HUMAN	Complement component C7 precursor	3.62	6.1E-05
49	C8orf1	CH001_HUMAN	Protein C8orf1	3.931	2.3E-20
50	C8orf72			2.776	0.00011
51	CASP1	CASP1_HUMAN	Caspase-1 precursor	2.725	0.00072
52	CASP4	CASP4_HUMAN	Caspase-4 precursor	2.643	2.5E-08
53	CCL17	CCL17_HUMAN	Small inducible cytokine A17 precursor	2.507	0.0459
54	CD14	CD14_HUMAN	Monocyte differentiation antigen CD14 precursor	6.813	2E-11
55	CD302	CD302	CD302 antigen	4.169	1.5E-33
56	CD40LG	CD40L_HUMAN	CD40 ligand	3.104	2.5E-07
57	CDKN1C	CDN1C_HUMAN	Cyclin-dependent kinase inhibitor 1C	16.91	2.5E-17
58	CEBPD	CEBPD_HUMAN	CCAAT/enhancer-binding protein delta	3.269	3.1E-85
59	CELSR1	CELR1_HUMAN	Cadherin EGF LAG seven-pass G-type receptor 1 precursor	3.789	4.8E-09
60	CES1	EST1_HUMAN	Liver carboxylesterase 1 precursor	5.856	1.5E-10
61	CFB	CFAB_HUMAN	Complement factor B precursor	7.097	2.4E-06
62	CFH	CFAH_HUMAN	Complement factor H precursor	5.836	4.6E-17
63	CFHR1	FHR1_HUMAN	Complement factor H-related protein 1 precursor	5.836	4.6E-17
64	CFHR2	FHR2_HUMAN	Complement factor H-related protein 2 precursor	4.218	1.4E-07
65	CG012			3.555	0.00193
66	CHI3L1	CH3L1_HUMAN	Chitinase-3-like protein 1 precursor	19.32	0.027
67	CHRFAM7A	CHRFAM7A	CHRNA7 (cholinergic receptor, nicotinic, alpha polypeptide 7, exons 5-10) and FAM7A (family with sequence similarity 7A, exons A-E) fusion	2.858	0.0263
68	CHRNA6	ACHA6_HUMAN	Neuronal acetylcholine receptor protein subunit alpha-6 precursor	4.121	2.4E-11
69	CHRNA7	ACHA7_HUMAN	Neuronal acetylcholine receptor protein subunit alpha-7 precursor	2.858	0.0263
70	CLEC2B	CLC2B_HUMAN	C-type lectin domain family 2 member B	7.795	1.4E-41
71	CLU	CLUS_HUMAN	Clusterin precursor	6.763	7.1E-53
72	CMKOR1	RDC1_HUMAN	G-protein coupled receptor RDC1 homolog	30.97	3E-81
73	CNR1	CNR1_HUMAN	Cannabinoid receptor 1	11.16	1.5E-08
74	COL11A1	COBA1_HUMAN	Collagen alpha-1(XI	12.83	0.0385
75	COL4A3	CO4A3_HUMAN	Collagen alpha-3(IV	4.418	6.8E-21
76	COL4A4	CO4A4_HUMAN	Collagen alpha-4(IV	2.549	2.3E-08
77	CPSF6	CPSF6_HUMAN	Cleavage and polyadenylation specificity factor 6	3.39	0.0167
78	CPVL	CPVL_HUMAN	Probable serine carboxypeptidase CPVL precursor	7.64	8.1E-07
79	CPZ	CPZ	carboxypeptidase Z	5.503	3.8E-43
80	CRYZ	QOR_HUMAN	Quinone oxidoreductase	2.889	3.3E-25
81	CTAG1A			5.511	1E-59
82	CTAG1B	CTG1B_HUMAN	Cancer/testis antigen 1B	5.511	1E-59
83	CTAG2	CTAG2_HUMAN	Cancer/testis antigen 2	5.511	1E-59
84	CTSK	CATK_HUMAN	Cathepsin K precursor	3.705	9E-39
85	CX3CL1	X3CL1_HUMAN	Fractalkine precursor	2.694	8E-21

86	CXADR	CXAR_HUMAN	Coxsackievirus and adenovirus receptor precursor	2.839	5E-08
87	CXCL1	GROA_HUMAN	Growth-regulated protein alpha precursor	8.772	8.6E-13
88	CXCL6	SCYB6_HUMAN	Small inducible cytokine B6 precursor	9.903	0.00017
89	CYP2C9	CP2C9_HUMAN	Cytochrome P450 2C9	3.175	0.0128
90	CYP2D6	CP2D6_HUMAN	Cytochrome P450 2D6	2.506	0.00012
91	DAPK1	DAPK1_HUMAN	Death-associated protein kinase 1	4.401	7.6E-20
92	DCAMKL1	DCAK1_HUMAN	Serine/threonine-protein kinase DCAMKL1	17.4	3.8E-11
93	DDX17	DDX17_HUMAN	Probable ATP-dependent RNA helicase DDX17	5.854	2.4E-88
94	DKFZP686A01 247			5.902	0.00025
95	DKFZP761N09 121			4.699	1.1E-22
96	DOK5	DOK5_HUMAN	Docking protein 5	6.011	1.2E-21
97	DSG2	DSG2_HUMAN	Desmoglein-2 precursor	4.683	2.2E-12
98	DUSP5	DUS5_HUMAN	Dual specificity protein phosphatase 5	2.566	1.3E-13
99	EEF1A2	EF1A2_HUMAN	Elongation factor 1-alpha 2	2.591	0.0476
100	EFNB2	EFNB2_HUMAN	Ephrin-B2 precursor	12.65	2.3E-30
101	ELK4	ELK4_HUMAN	ETS domain-containing protein Elk-4	3.415	0.0272
102	ELOVL2	ELOV2_HUMAN	Elongation of very long chain fatty acids protein 2	2.638	8.7E-06
103	EMP1	EMP1_HUMAN	Epithelial membrane protein 1	5.541	6.6E-40
104	ENOSF1			5.393	1.4E-17
105	ENPP2	ENPP2_HUMAN	Ectonucleotide pyrophosphatase/phosphodiesterase 2	13.57	1.1E-08
106	EPB41L3	E41L3_HUMAN	Band 4.1-like protein 3	7.063	2.1E-84
107	EPHA3	EPHA3_HUMAN	Ephrin type-A receptor 3 precursor	6.532	2.1E-11
108	ETV1	ETV1_HUMAN	ETS translocation variant 1	4.54	8.6E-13
109	EXPH5	SLC2B_HUMAN	Slp homolog lacking C2 domains b	3.4	2.1E-07
110	EYA4	EYA4_HUMAN	Eyes absent homolog 4	2.67	0.00013
111	FBLN1	FBLN1_HUMAN	Fibulin-1 precursor	11.9	4.8E-30
112	FGF7	FGF7_HUMAN	Keratinocyte growth factor precursor	36.35	2.1E-25
113	FGFR2	FGFR2_HUMAN	Fibroblast growth factor receptor 2 precursor	12.34	1.5E-48
114	FGFR3	FGFR3_HUMAN	Fibroblast growth factor receptor 3 precursor	4.943	5.9E-09
115	FLJ13946	FLJ13946	hypothetical protein LOC92104	2.669	0.013
116	FLJ36166			3.457	2.2E-15
117	FLRT2	FLRT2_HUMAN	Leucine-rich repeat transmembrane protein FLRT2 precursor(Fibronectin-like domain-containing leucine-rich transmembrane protein2	5.665	0.00354
118	FOS	FOS_HUMAN	Proto-oncogene protein c-fos	4.104	3E-05
119	FRZB	SFRP3_HUMAN	Secreted frizzled-related protein 3 precursor	3.133	2.6E-06
120	GCH1	GCH1_HUMAN	GTP cyclohydrolase I	2.955	0.00011
121	GCLC	GSH1_HUMAN	Glutamate--cysteine ligase catalytic subunit	2.573	1.4E-22
122	GFRA1	GFRA1_HUMAN	GDNF family receptor alpha-1 precursor	3.304	0.00586
123	GNAZ	GNAZ_HUMAN	Guanine nucleotide-binding protein G(z	3.495	1.4E-25
124	GPC4	GPC4_HUMAN	Glypican-4 precursor	8.839	0.0237
125	GPNMB	GPNMB_HUMAN	Transmembrane glycoprotein NMB precursor	4.439	1.8E-29
126	GPR125	GP125_HUMAN	Probable G-protein coupled receptor 125 precursor	2.698	7.9E-30

127	GPRC5B	GPC5B_HUMAN	G-protein coupled receptor family C group 5 member B precursor(Retinoic acid-induced gene 2 protein	10.7	3.9E-65
128	HLA-DPB1	HB2T_HUMAN	HLA class II histocompatibility antigen, SB beta chain	7.734	6.4E-24
		HB2Q_HUMAN	HLA class II histocompatibility antigen, DP(W2	7.734	6.4E-24
		HB2P_HUMAN	HLA class II histocompatibility antigen, DP(W4	7.734	6.4E-24
		HB2S_HUMAN	HLA class II histocompatibility antigen, SB beta chain	7.734	6.4E-24
129	HOXB2	HXB2_HUMAN	Homeobox protein Hox-B2	12.08	2.7E-19
130	HTR2B	5HT2B_HUMAN	5-hydroxytryptamine 2B receptor	9.197	9.5E-15
131	ICAM1	ICAM1_HUMAN	Intercellular adhesion molecule 1 precursor	2.7	0.0044
132	ID1	ID1_HUMAN	DNA-binding protein inhibitor ID-1	7.095	7.1E-08
133	ID2	ID2_HUMAN	DNA-binding protein inhibitor ID-2	11.39	2.9E-11
134	ID2B			11.39	2.9E-11
135	IFI27	INI7_HUMAN	Interferon-alpha-induced 11.5 kDa protein	26.75	2E-61
136	IGF2	IG2R_HUMAN	Putative insulin-like growth factor 2-associated protein	11.78	3E-06
		IGF2_HUMAN	Insulin-like growth factor II precursor	11.78	3E-06
137	IGFBP1	IBP1_HUMAN	Insulin-like growth factor-binding protein 1 precursor	8.108	0.00277
138	IGSF3	IGSF3	immunoglobulin superfamily, member 3	20.88	1.3E-60
139	IL13RA2	I13R2_HUMAN	Interleukin-13 receptor alpha-2 chain precursor	3.667	4.7E-16
140	IL15RA	I15RA_HUMAN	Interleukin-15 receptor alpha chain precursor	2.978	0.0352
141	IL1RL1	ILRL1_HUMAN	Interleukin-1 receptor-like 1 precursor	2.776	4.7E-12
142	IL32	IL32_HUMAN	Interleukin-32 precursor	20.91	7.3E-30
143	IL8	IL8_HUMAN	Interleukin-8 precursor	10.28	0.00034
144	IMPA2	IMPA2_HUMAN	Inositol monophosphatase 2	2.6	1.3E-10
145	ITGA2	ITA2_HUMAN	Integrin alpha-2 precursor	3.121	0.00119
146	ITGB3BP	NRIF3_HUMAN	Nuclear receptor-interacting factor 3	3.081	0.0463
147	ITM2A	ITM2A_HUMAN	Integral membrane protein 2A	9.991	1.6E-18
148	ITSN1	ITSN1_HUMAN	Intersectin-1	2.907	1.9E-35
149	JUND	JUND_HUMAN	Transcription factor jun-D	3.329	4.4E-10
150	KAL1	KALM_HUMAN	Anosmin-1 precursor	3.071	0.013
151	KCNK2	KCNK2_HUMAN	Potassium channel subfamily K member 2	7.481	5.7E-06
152	KCTD12	KCD12_HUMAN	BTB/POZ domain-containing protein KCTD12	8.705	0.00716
153	KIAA0574	K0574_HUMAN	Protein KIAA0574	3.426	0.0418
154	KIAA0895			3.65	2.2E-07
155	KIAA1199	K1199_HUMAN	Protein KIAA1199 precursor	3.237	2.3E-18
156	KIAA1462			2.933	2.3E-06
157	KIT	KIT_HUMAN	Mast/stem cell growth factor receptor precursor	7.28	3E-101
158	LAMA4	LAMA4_HUMAN	Laminin alpha-4 chain precursor	3.632	2.5E-10
159	LAMP3	LAMP3_HUMAN	Lysosome-associated membrane glycoprotein 3 precursor	5.006	0.00288
160	LGR5	LGR5_HUMAN	Leucine-rich repeat-containing G-protein coupled receptor 5 precursor(Orphan G-protein coupled receptor HG38	18.96	4.7E-05
161	LMO3	LMO3_HUMAN	LIM-only protein 3	11.87	1.7E-27
162	LOC440352			3.188	5.8E-08
163	LOC643676			3.893	2.1E-14
164	LOC652740			2.858	0.0263
165	LOC653387			5.511	1E-59

166	LOC654153			7.734	6.4E-24
167	LOH11CR2A	LHR2A_HUMAN	Loss of heterozygosity 11 chromosomal region 2 gene A protein	17.5	9E-120
168	LUM	LUM_HUMAN	Lumican precursor	2.563	0.0481
169	LUZP2			10.71	3.8E-14
170	LY96	LY96_HUMAN	Lymphocyte antigen 96 precursor	7.456	3.9E-19
171	MAF	MAF_HUMAN	Transcription factor Maf	2.535	3.1E-22
172	MAN1A1	MA1A1_HUMAN	Mannosyl-oligosaccharide 1,2-alpha-mannosidase IA	3.812	2.7E-49
173	MAP3K8	M3K8_HUMAN	Mitogen-activated protein kinase kinase kinase 8	4.843	2.7E-08
174	MAPK10	MK10_HUMAN	Mitogen-activated protein kinase 10	3.663	4.5E-19
175	MCC	CRCM_HUMAN	Colorectal mutant cancer protein	7.857	1.1E-12
176	MDFIC	MDFIC	MyoD family inhibitor domain containing	2.719	2.1E-17
177	MDK	MK_HUMAN	Midkine precursor	4.68	6.7E-07
178	MEOX2	MEOX2_HUMAN	Homeobox protein MOX-2	7.897	6.2E-10
179	MFAP5	MFAP5_HUMAN	Microfibrillar-associated protein 5 precursor	3.245	6.6E-09
180	MGC35048			4.584	4.9E-58
181	MID1	TRI18_HUMAN	Midline-1	4.208	2.8E-06
182	MMP2	MMP2_HUMAN	72 kDa type IV collagenase precursor	3.488	5.7E-16
183	MMP8	MMP8_HUMAN	Neutrophil collagenase precursor	3.617	1.3E-13
184	MOXD1	MOXD1	monooxygenase, DBH-like 1	6.61	1.1E-09
185	MPO	PERM_HUMAN	Myeloperoxidase precursor	2.531	0.00065
186	MSX1	MSX1_HUMAN	Homeobox protein MSX-1	5.175	1.8E-15
187	MTUS1	MTUS1	mitochondrial tumor suppressor 1	3.201	3.8E-28
188	MYB	MYB_HUMAN	Myb proto-oncogene protein	3.1	0.00011
189	MYBPC1	MYPC1_HUMAN	Myosin-binding protein C, slow-type	4.96	8.8E-05
190	NCAM1	NCA12_HUMAN	Neural cell adhesion molecule 1, 120 kDa isoform precursor	3.077	7.6E-10
		NCA11_HUMAN	Neural cell adhesion molecule 1, 140 kDa isoform precursor	3.077	7.6E-10
191	NEFH	NFH_HUMAN	Neurofilament triplet H protein	3.726	2.7E-05
192	NFASC	NFASC_HUMAN	Neurofascin precursor	6.989	1.9E-09
193	NFE2L3	NF2L3_HUMAN	Nuclear factor erythroid 2-related factor 3	2.937	6.3E-05
194	NFKB2	NFKB2_HUMAN	Nuclear factor NF-kappa-B p100 subunit	3.37	0.0013
195	NOVA1	NOVA1_HUMAN	RNA-binding protein Nova-1	3.339	1.6E-10
196	NR1D2	NR1D2_HUMAN	Orphan nuclear receptor NR1D2	3.186	1.4E-20
197	OLFM1	NOE1_HUMAN	Noelin precursor	5.446	1.4E-53
198	OMD	OMD_HUMAN	Osteomodulin precursor	5.004	7.1E-10
199	OSR2	OSR2_HUMAN	Protein odd-skipped-related 2	4.724	5E-05
200	PAX2	PAX2_HUMAN	Paired box protein Pax-2	3.234	5.3E-08
201	PDE1A	PDE1A_HUMAN	Calcium/calmodulin-dependent 3',5'-cyclic nucleotide phosphodiesterase1A	12.48	3.1E-15
202	PDE1C	PDE1C_HUMAN	Calcium/calmodulin-dependent 3',5'-cyclic nucleotide phosphodiesterase1C	3.388	9.2E-16
203	PDGFRL	PGFRL_HUMAN	Platelet-derived growth factor receptor-like protein precursor	2.988	0.00046
204	PDPN	PDPN_HUMAN	Podoplanin precursor	4.385	5.5E-87
205	PDZD2	PDZD2_HUMAN	PDZ domain-containing protein 2	4.032	5.5E-05
206	PGD	6PGD_HUMAN	6-phosphogluconate dehydrogenase, decarboxylating	2.693	4.8E-33
207	PGR	PRGR_HUMAN	Progesterone receptor	4.801	7.3E-05

208	PHLPP	PHLPP_HUMAN	PH domain leucine-rich repeat-containing protein phosphatase(EC 3.1.3.16	2.538	3E-09
209	PIK3CG	PK3CG_HUMAN	Phosphatidylinositol-4,5-bisphosphate 3-kinase catalytic subunit gammaisoform	2.801	0.00508
210	PIR	PIR_HUMAN	Pirin	2.661	2.9E-22
211	PLCE1	PLCE1	phospholipase C, epsilon 1	3.42	1.4E-10
212	PNMA2	PNMA2_HUMAN	Paraneoplastic antigen Ma2	3.453	6.3E-32
213	POLR1C	RPA5_HUMAN	DNA-directed RNA polymerase I 40 kDa polypeptide	2.733	0.00044
214	PPL	PEPL_HUMAN	Periplakin	3.902	2.4E-05
215	PPM1H			2.506	1.5E-29
216	PROS1	PROS_HUMAN	Vitamin K-dependent protein S precursor	3.874	1.3E-16
217	PRPS1	PRPS1_HUMAN	Ribose-phosphate pyrophosphokinase I	3.253	8E-20
218	PSPH	SERB_HUMAN	Phosphoserine phosphatase	3.478	0
219	PSPHL	PSPHL	phosphoserine phosphatase-like	9.705	0
220	PTGDS	PTGDS_HUMAN	Prostaglandin-H2 D-isomerase precursor	26.4	3.1E-45
221	PTGER4	PE2R4_HUMAN	Prostaglandin E2 receptor, EP4 subtype	4.551	5.6E-05
222	PTGES	PTGES_HUMAN	Prostaglandin E synthase	9.902	9.4E-16
223	PTHLH	PTHR_HUMAN	Parathyroid hormone-related protein precursor	5.649	5.3E-07
224	PTPN20A			9.224	6.2E-13
225	PTPRT	PTPRT_HUMAN	Receptor-type tyrosine-protein phosphatase T precursor	2.789	0.00086
226	RAB11FIP3	RFIP3_HUMAN	Rab11 family-interacting protein 3	2.691	0.0102
227	RAB1A	RAB1A_HUMAN	Ras-related protein Rab-1A	2.715	1.1E-07
228	RAB6B	RAB6B_HUMAN	Ras-related protein Rab-6B	3.528	1E-10
229	RAD9A	RAD9A_HUMAN	Cell cycle checkpoint control protein RAD9A	3.485	4E-19
230	RALGPS1	RALGPS1	Ral GEF with PH domain and SH3 binding motif 1	3.393	2.2E-09
231	RARRES1	TIG1_HUMAN	Retinoic acid receptor responder protein 1	3.471	0.00092
232	RARRES3	TIG3_HUMAN	Retinoic acid receptor responder protein 3	3.445	3.5E-28
233	RBP1	RET1_HUMAN	Retinol-binding protein I, cellular	7.948	3.2E-21
234	RBP4	RETBP_HUMAN	Plasma retinol-binding protein precursor	5.591	7E-20
235	REL	REL_HUMAN	C-Rel proto-oncogene protein	3.438	2.2E-06
236	RFXAP	RFXAP_HUMAN	Regulatory factor X-associated protein	2.524	0.00181
237	RGN	RGN_HUMAN	Regucalcin	5.106	1.6E-05
238	RGS2	RGS2_HUMAN	Regulator of G-protein signaling 2	3.298	0.00313
239	RGS3	RGS3_HUMAN	Regulator of G-protein signaling 3	3.274	2.6E-05
240	RHOBTB3	RHBT3_HUMAN	Rho-related BTB domain-containing protein 3	3.068	1.1E-23
241	RIS1	RIS1	BINP receptor	2.913	2.8E-07
242	RP11-142I17.1	PTPN20	OTTHUMP00000059234	9.224	6.2E-13
243	RPESP			9.541	1.4E-44
244	RRAGD	RRAGD_HUMAN	Ras-related GTP-binding protein D	7.316	7.4E-15
245	SALL1	SALL1_HUMAN	Sal-like protein 1	4.058	1.8E-06
246	SAT	SAT1_HUMAN	Diamine acetyltransferase 1	2.936	2.8E-08
247	SCG2	SCG2_HUMAN	Secretogranin-2 precursor	2.769	0.0186
248	SELENBP1	SBP1_HUMAN	Selenium-binding protein 1	3.926	6.8E-31
249	SEMA3B	SEM3B_HUMAN	Semaphorin-3B precursor	2.595	0.00382
250	SEPP1	SEPP1_HUMAN	Selenoprotein P precursor	44.28	5.2E-20
251	SETBP1	SETBP_HUMAN	SET-binding protein	2.539	2.3E-18
252	SFRP1	SFRP1_HUMAN	Secreted frizzled-related protein 1 precursor	11.72	4.5E-50
253	SGCD	SGCD_HUMAN	Delta-sarcoglycan	4.709	8.6E-34

254	SH3BGR	SH3BG_HUMAN	SH3 domain-binding glutamic acid-rich protein	7.582	9.1E-20
255	SH3BP5	3BP5_HUMAN	SH3 domain-binding protein 5	4.555	2.5E-29
256	SHOX	SHOX_HUMAN	Short stature homeobox protein	6.853	4.2E-31
257	SKP2	SKP2_HUMAN	S-phase kinase-associated protein 2	3.143	0.00327
258	SLC16A6	MOT7_HUMAN	Monocarboxylate transporter 7	2.69	2.3E-07
259	SLC39A8	SLC39A8__64116	solute carrier family 39 (zinc transporter), member 8	9.266	4.9E-09
		SLC39A8	solute carrier family 39 (zinc transporter), member 8	9.266	4.9E-09
260	SLC4A3	B3A3_HUMAN	Anion exchange protein 3	3.335	7.1E-11
261	SLC7A8	LAT2_HUMAN	Large neutral amino acids transporter small subunit 2	2.814	1.2E-31
262	SLCO2B1	SO2B1_HUMAN	Solute carrier organic anion transporter family member 2B1	2.664	1.4E-08
263	SMAD6	SMAD6_HUMAN	Mothers against decapentaplegic homolog 6	5.505	2.1E-14
264	SNED1			8.935	6.9E-34
265	SOD2	SODM_HUMAN	Superoxide dismutase [Mn], mitochondrial precursor	4.339	1.3E-07
266	SRD5A2	S5A2_HUMAN	3-oxo-5-alpha-steroid 4-dehydrogenase 2	5.161	8E-09
267	SRPX2			5.215	6.3E-60
268	ST3GAL1	SIA4A_HUMAN	CMP-N-acetylneuraminate-beta-galactosamide-alpha-2,3-sialyltransferase(EC 2.4.99.4	2.797	7.6E-05
269	ST6GALNAC2	SIA7B_HUMAN	Alpha-N-acetylgalactosaminide alpha-2,6-sialyltransferase 2(EC 2.4.99.-	5.871	9.2E-11
270	STAT5B	STA5B_HUMAN	Signal transducer and activator of transcription 5B	3.623	0.0049
271	SVEP1	SVEP1	sushi, von Willebrand factor type A, EGF and pentraxin domain containing 1	5.42	5.3E-21
272	SVIL	SVIL_HUMAN	Supervillin	2.91	3.7E-11
273	SYNJ1	SYNJ1_HUMAN	Synaptojanin-1	3.034	8.5E-40
274	TACSTD2	TACD2_HUMAN	Tumor-associated calcium signal transducer 2 precursor	2.941	2.6E-09
275	TAGLN	TAGL_HUMAN	Transgelin	4.661	9.5E-21
276	TBC1D8	TBCD8_HUMAN	TBC1 domain family member 8	4.888	5.5E-22
277	TFAP2C	AP2C_HUMAN	Transcription factor AP-2 gamma	2.579	2.2E-05
278	TGFBR3	TGBR3_HUMAN	TGF-beta receptor type III precursor	5.92	1.1E-43
279	THBS2	TSP2_HUMAN	Thrombospondin-2 precursor	2.817	1.3E-21
280	TIAM1	TIAM1_HUMAN	T-lymphoma invasion and metastasis-inducing protein 1	3.312	0.00066
281	TLR1	TLR1_HUMAN	Toll-like receptor 1 precursor	2.577	3.2E-06
282	TMEM16C	TM16C_HUMAN	Transmembrane protein 16C	4.156	4E-08
283	TNFAIP6	TSG6_HUMAN	Tumor necrosis factor-inducible protein TSG-6 precursor	3.865	9.8E-13
284	TNFSF9	TNFL9_HUMAN	Tumor necrosis factor ligand superfamily member 9	3.975	2.3E-07
285	TNNT2	TNNT2_HUMAN	Troponin T, cardiac muscle	4.544	8.9E-13
286	TNP2	STP2_HUMAN	Nuclear transition protein 2	2.723	4.3E-05
287	TSHB	TSHB_HUMAN	Thyrotropin beta chain precursor	2.717	3.5E-08
288	TSPAN8	TSN8_HUMAN	Tetraspanin-8	12.46	2.1E-64
289	TTF1	TTF1	transcription termination factor, RNA polymerase I	5.235	1.6E-64
290	UST	UST_HUMAN	Uronyl 2-sulfotransferase	2.662	3.8E-08

291	VDR	VDR_HUMAN	Vitamin D3 receptor	3.936	2.7E-29
292	VPS13A	VP13A_HUMAN	Vacuolar protein sorting 13A	3.83	0.00019
293	WDR67	WDR67_HUMAN	WD repeat protein 67	3.322	0.00065
294	WNT5A	WNT5A_HUMAN	Protein Wnt-5a precursor	5.97	3.6E-14
295	WWC1	WWC1_HUMAN	WW domain-containing protein 1	7.769	5.3E-24
296	ZHX2	ZHX2_HUMAN	Zinc fingers and homeoboxes protein 2	3.491	2.5E-85
297	ZNF239	ZN239_HUMAN	Zinc finger protein 239	3.011	7.2E-06
298	ZNF365	ZN365_HUMAN	Protein ZNF365	3.021	0.00073
		TALAN_HUMAN	Talanin	3.021	0.00073

Group 2, up-regulated genes (HUGO nomenclature), fold change > 2.5					
#	Gene Symbol	Protein	Protein name	Signal	P_value
1	ABCA8	ABCA8	ATP-binding cassette, sub-family A (ABC1), member 8	2.6105	4.68E-08
2	ABCC3	MRP3_HUMAN	Canalicular multispecific organic anion transporter 2	3.7725	0.005853741
3	ABHD3			2.5882	0.003273047
4	ACVR1B	ACV1B_HUMAN	Activin receptor type 1B precursor	2.796	0.000150155
5	ADAM17	ADA17_HUMAN	ADAM 17 precursor	3.4658	4.5E-09
6	ADAM3A	ADAM3A	ADAM metallopeptidase domain 3a (cyritestin 1)	3.6782	0.00000948
7	ADH1A	ADH1A_HUMAN	Alcohol dehydrogenase 1A	8.1933	9.9E-13
8	ADH1B	ADH1B_HUMAN	Alcohol dehydrogenase 1B	8.0945	2.77E-12
9	ADH1C	ADH1G_HUMAN	Alcohol dehydrogenase 1C	8.6725	1.08E-26
10	AIM1	AIM1_HUMAN	Absent in melanoma 1 protein	3.1127	0.00069225
11	AKAP5	AKAP5_HUMAN	A-kinase anchor protein 5	7.321	2.3E-31
12	AKR1B10	AK1BA_HUMAN	Aldo-keto reductase family 1 member B10	3.0579	0.00052445
13	AKR1C1	AK1C1_HUMAN	Aldo-keto reductase family 1 member C1	9.6521	0.001167111
14	AKR1C2	AK1C2_HUMAN	Aldo-keto reductase family 1 member C2	9.6521	0.001167111
15	AKR1C3	AK1C3_HUMAN	Aldo-keto reductase family 1 member C3	7.4443	0.000000479
16	ALDH1A2	AL1A2_HUMAN	Retinal dehydrogenase 2	2.7756	0.0000611
17	AMY1A	AMYS_HUMAN	Salivary alpha-amylase precursor	2.5488	0.001044927
18	AMY1B			2.5488	0.001044927
19	AMY1C			2.5488	0.001044927
20	AMY2A	AMYP_HUMAN	Pancreatic alpha-amylase precursor	2.5488	0.001044927
21	AMY2B	AMYC_HUMAN	Alpha-amylase 2B precursor	2.5488	0.001044927
22	ANGPT2	ANGP2_HUMAN	Angiopoietin-2 precursor	3.2666	0.00000764
23	ANGPTL2	ANGL2_HUMAN	Angiopoietin-related protein 2 precursor	2.5642	1.48E-16
24	ANK2	ANK2_HUMAN	Ankyrin-2	2.9344	4.78E-15
25	ANXA13	ANX13_HUMAN	Annexin A13	3.1427	7.66E-11
26	AOC2	AOC2_HUMAN	Retina-specific copper amine oxidase precursor	2.675	0.000204894
27	AQP9	AQP9_HUMAN	Aquaporin-9	3.8794	3.94E-10
28	ARTS-1	ARTS1_HUMAN	Adipocyte-derived leucine aminopeptidase precursor	5.3718	1.65E-12
29	ASPHD1			2.8691	0.00098625
30	ATP1A2	AT1A2_HUMAN	Sodium/potassium-transporting ATPase alpha-2 chain precursor(EC 3.6.3.9	3.0715	0.001491955
31	AUTS2	AUTS2_HUMAN	Autism susceptibility gene 2 protein	4.4713	0.006107122
32	BBOX1	BODG_HUMAN	Gamma-butyrobetaine dioxygenase	3.1346	0.000781241
33	BDKRB2	BKRB2_HUMAN	B2 bradykinin receptor	3.6348	0.00000372
34	BEAN			3.3664	2.72E-08
35	BPI	BPI_HUMAN	Bactericidal permeability-increasing protein precursor	2.6151	0.002727648
36	BRS3	BRS3_HUMAN	Bombesin receptor subtype-3	2.5639	0.009361356
37	C14orf120	CN120_HUMAN	Protein C14orf120	2.5121	0.000827581
38	C14orf132	CN132_HUMAN	Protein C14orf132	3.4694	1.17E-18
39	C18orf1	CR001_HUMAN	Protein C18orf1	2.9932	0.02791622
40	C1R	C1R_HUMAN	Complement C1r subcomponent precursor	3.9755	0.000153562
41	C1S	PRP5_HUMAN	Basic proline-rich peptide IB-1	2.8623	0.004975585
		C1S_HUMAN	Complement C1s subcomponent precursor	2.8623	0.004975585

42	C1orf21	CA021_HUMAN	Protein C1orf21	2.856	0.0000471
43	C1orf46			3.0017	1.17E-09
44	C8orf70			4.4021	0.00000121
45	CA12	CAH12_HUMAN	Carbonic anhydrase 12 precursor	2.5281	2.79E-08
46	CALCRL	CALRL_HUMAN	Calcitonin gene-related peptide type 1 receptor precursor	2.5212	0.000000897
47	CAPG	CAPG_HUMAN	Macrophage capping protein	2.8175	0.0000118
48	CASP1	CASP1_HUMAN	Caspase-1 precursor	4.7624	1E-39
49	CD14	CD14_HUMAN	Monocyte differentiation antigen CD14 precursor	2.7097	0.00176309
50	CD226	CD226_HUMAN	CD226 antigen precursor	3.0926	0.000781733
51	CD302	CD302	CD302 antigen	3.5493	0.0000019
52	CD3Z	CD3Z_HUMAN	T-cell surface glycoprotein CD3 zeta chain precursor	3.1919	0.000000104
53	CD40LG	CD40L_HUMAN	CD40 ligand	2.7305	4.81E-08
54	CDC25C	MPIP3_HUMAN	M-phase inducer phosphatase 3	4.9693	0.000000789
55	CDH5	CADH5_HUMAN	Cadherin-5 precursor	3.5461	1.94E-16
56	CDKL1	CDKL1_HUMAN	Cyclin-dependent kinase-like 1	4.5521	0.000391857
57	CDKN1C	CDN1C_HUMAN	Cyclin-dependent kinase inhibitor 1C	4.6659	0.002945638
58	CDO1	CDO1_HUMAN	Cysteine dioxygenase type 1	3.4705	2.54E-17
59	CDS1	CDS1_HUMAN	Phosphatidate cytidylyltransferase 1	3.1107	0.000215841
60	CEACAM5	CEAM5_HUMAN	Carcinoembryonic antigen-related cell adhesion molecule 5 precursor(Carcinoembryonic antigen	4.1328	0.0000094
61	CELSR1	CELR1_HUMAN	Cadherin EGF LAG seven-pass G-type receptor 1 precursor	2.9632	0.01130738
62	CFB	CFAB_HUMAN	Complement factor B precursor	2.5132	0.00004
63	CFHR1	FHR1_HUMAN	Complement factor H-related protein 1 precursor	3.5474	0.000211182
64	CFHR2	FHR2_HUMAN	Complement factor H-related protein 2 precursor	3.5474	0.000211182
65	CFTR	CFTR_HUMAN	Cystic fibrosis transmembrane conductance regulator	3.3078	0.001178089
66	CHEK2	CHK2_HUMAN	Serine/threonine-protein kinase Chk2	2.6605	0.000392343
67	CHI3L1	CH3L1_HUMAN	Chitinase-3-like protein 1 precursor	4.7059	0.007612204
68	CHI3L2	CH3L2_HUMAN	Chitinase 3-like protein 2 precursor	2.8811	0.00000252
69	CHRD	CHRD_HUMAN	Chordin precursor	3.3739	8.89E-09
70	CLCA2	CLCA2	chloride channel, calcium activated, family member 2	4.3886	3.89E-16
71	CLCNKA	CLCKA_HUMAN	Chloride channel protein ClC-Ka	2.6057	0.00000157
72	CLIC2	CLIC2_HUMAN	Chloride intracellular channel protein 2	5.55	0.0000427
73	CLU	CLUS_HUMAN	Clusterin precursor	5.7852	0.003672578
74	CMKOR1	RDC1_HUMAN	G-protein coupled receptor RDC1 homolog	2.7724	0.01420417
75	CNR1	CNR1_HUMAN	Cannabinoid receptor 1	6.8044	0.000732322
76	COL14A1	COEA1_HUMAN	Collagen alpha-1(XIV	2.7593	7.1E-13
77	COL21A1	COL21A1	collagen, type XXI, alpha 1	2.5343	6.43E-09
78	COL4A4	CO4A4_HUMAN	Collagen alpha-4(IV	2.9341	0.0194451
79	CPN2	CPN2_HUMAN	Carboxypeptidase N subunit 2 precursor	3.8429	0.000270598
80	CPZ	CPZ	carboxypeptidase Z	3.8049	0.000694703
81	CRABP2	RABP2_HUMAN	Cellular retinoic acid-binding protein 2	2.7557	6.79E-15
82	CRLF1	CRLF1_HUMAN	Cytokine receptor-like factor 1 precursor	4.0882	2.25E-12
83	CSTA	CYTA_HUMAN	Cystatin A	3.9912	2.31E-09

84	CTSK	CATK_HUMAN	Cathepsin K precursor	2.9332	0.00000229
85	CXCL1	GROA_HUMAN	Growth-regulated protein alpha precursor	5.7378	2.28E-11
86	CXCL12	SDF1_HUMAN	Stromal cell-derived factor 1 precursor	2.597	0.00000184
87	CXCL2	MIP2A_HUMAN	Macrophage inflammatory protein 2-alpha precursor	3.0051	0.00112366
88	CXCL3	MIP2B_HUMAN	Macrophage inflammatory protein 2-beta precursor	2.8566	3.53E-52
89	CXCL6	SCYB6_HUMAN	Small inducible cytokine B6 precursor	7.6732	4.57E-14
90	CYP1A1	CP1A1_HUMAN	Cytochrome P450 1A1	3.6031	1.42E-11
91	CYP27A1	CP27A_HUMAN	Cytochrome P450 27, mitochondrial precursor	4.314	1.52E-13
92	CYP27B1	CP27B_HUMAN	25-hydroxyvitamin D-1 alpha hydroxylase, mitochondrial precursor(EC 1.14.13.13	2.7523	0.0000126
93	CYP3A4	CP3A3_HUMAN	Cytochrome P450 3A3	3.8416	1.38E-10
		CP3A4_HUMAN	Cytochrome P450 3A4	3.8416	1.38E-10
94	CYP3A7	CP3A7_HUMAN	Cytochrome P450 3A7	3.8416	1.38E-10
95	CYP7A1	CP7A1_HUMAN	Cytochrome P450 7A1	5.4975	0.00000015
96	DBC1	FAM5A_HUMAN	Protein FAM5A precursor	3.0878	0.002642052
97	DCAMKL1	DCAK1_HUMAN	Serine/threonine-protein kinase DCAMKL1	5.2187	8.91E-08
98	DIP2C	DIP2C_HUMAN	Disco-interacting protein 2 homolog C	4.4794	0.01204429
99	DISC1	DISC1_HUMAN	Disrupted in schizophrenia 1 protein	3.0123	0.002745774
100	DKFZP686A01247			3.2778	0.04107026
101	DNAH7	DNAH7	dynein, axonemal, heavy polypeptide 7	2.7393	0.000729447
102	DPYD	DPYD_HUMAN	Dihydropyrimidine dehydrogenase [NADP+] precursor	2.6202	5.15E-08
103	DUSP7	DUS7_HUMAN	Dual specificity protein phosphatase 7	3.187	1.84E-18
104	DYNC1I1	DC1I1_HUMAN	Cytoplasmic dynein 1 intermediate chain 1	2.5807	2.24E-26
105	EFEMP1	FBLN3_HUMAN	EGF-containing fibulin-like extracellular matrix protein 1 precursor(Fibulin-3	2.7237	7.96E-08
106	EFNA4	EFNA4_HUMAN	Ephrin-A4 precursor	2.9624	9.28E-30
107	EFNB2	EFNB2_HUMAN	Ephrin-B2 precursor	4.7441	0.001180544
108	EGR2	EGR2_HUMAN	Early growth response protein 2	6.4541	3.3E-15
109	EGR3	EGR3_HUMAN	Early growth response protein 3	4.4807	2.11E-10
110	EIF2A	eIF2A	CDA02 protein	3.227	0.00000952
111	EPAG			2.5671	0.000354808
112	EPB41L3	E41L3_HUMAN	Band 4.1-like protein 3	3.5806	0.02089789
113	EPHB2	EPHB2_HUMAN	Ephrin type-B receptor 2 precursor	2.5502	0.000177799
114	EPHX1	HYEP_HUMAN	Epoxide hydrolase 1	3.6239	0.000268582
115	EPM2A	EPM2A_HUMAN	Laforin	2.75	2.06E-09
116	ERBB3	ERBB3_HUMAN	Receptor tyrosine-protein kinase erbB-3 precursor	2.7991	0.02297004
117	EYA2	EYA2_HUMAN	Eyes absent homolog 2	4.5762	0.000000451
118	F5	FA5_HUMAN	Coagulation factor V precursor	2.5346	0.002624751
119	FBLN2	FBLN2_HUMAN	Fibulin-2 precursor	3.3785	0.0000201
120	FCGR2A	FCG2A_HUMAN	Low affinity immunoglobulin gamma Fc region receptor II-a precursor(Fc-gamma RII-a	3.9763	0.005261256
121	FETUB	FETUB_HUMAN	Fetuin-B precursor	4.4113	3.3E-11
122	FGF12	FGF12_HUMAN	Fibroblast growth factor 12	2.705	0.000472984
123	FGF7	FGF7_HUMAN	Keratinocyte growth factor precursor	17.363	0.000000458
124	FGFR2	FGFR2_HUMAN	Fibroblast growth factor receptor 2 precursor	10.011	0.000000886
125	FGL2	FGL2_HUMAN	Fibroleukin precursor	9.008	0.000000123
126	FLJ43806			2.5741	0.000124958

127	FLRT2	FLRT2_HUMAN	Leucine-rich repeat transmembrane protein FLRT2 precursor(Fibronectin-like domain-containing leucine-rich transmembrane protein2	4.558	0.000178176
128	FLT3LG	FLT3L_HUMAN	SL cytokine precursor	2.8603	0.0000203
129	FMO6	FMO6_HUMAN	Putative dimethylaniline monooxygenase [N-oxide-forming] 6(EC 1.14.13.8	2.8701	0.000000188
130	FOS	FOS_HUMAN	Proto-oncogene protein c-fos	2.8283	4.11E-08
131	FOXF2	FOXF2_HUMAN	Forkhead box protein F2	3.1125	0.002098682
132	G0S2	G0S2_HUMAN	Putative lymphocyte G0/G1 switch protein 2	2.7063	0.0000151
133	GALNAC4S-6ST	ST4S6_HUMAN	N-acetylgalactosamine 4-sulfate 6-O-sulfotransferase	3.7435	0.01160934
134	GAP43	NEUM_HUMAN	Neuromodulin	3.3227	0.0000303
135	GAS1	GAS1_HUMAN	Growth-arrest-specific protein 1 precursor	3.2288	0.000392184
136	GAS7	GAS7_HUMAN	Growth-arrest-specific protein 7	4.7166	0.00011319
137	GATA2	GATA2_HUMAN	Endothelial transcription factor GATA-2	4.0009	0.00000805
138	GATA3	GATA3_HUMAN	Trans-acting T-cell-specific transcription factor GATA-3	3.3639	0.0000481
139	GDF10	BMP3B_HUMAN	Bone morphogenetic protein 3b precursor	3.9904	1.3E-13
140	GDF8	GDF8_HUMAN	Growth/differentiation factor 8 precursor	5.2367	0.000107736
141	GIMAP5	GIMA5_HUMAN	GTPase, IMAP family member 5	3.4851	0.000000401
142	GK2	GLPK2_HUMAN	Glycerol kinase, testis specific 2	2.5344	0.0000277
143	GPC1	GPC1_HUMAN	Glypican-1 precursor	3.6861	0.001802741
144	GPC4	GPC4_HUMAN	Glypican-4 precursor	4.1844	0.04304633
145	GPNMB	GPNMB_HUMAN	Transmembrane glycoprotein NMB precursor	11.55	0.005712037
146	GPR19	GPR19_HUMAN	Probable G-protein coupled receptor 19	3.4104	0.03788132
147	GRP	GRP_HUMAN	Gastrin-releasing peptide precursor	2.5466	0.0000112
148	GYPB	GLPB_HUMAN	Glycophorin B precursor	3.5279	0.00000137
149	HAPLN1	HPLN1_HUMAN	Hyaluronan and proteoglycan link protein 1 precursor	10.215	0.000959028
150	HEPH	HEPH_HUMAN	Hephaestin precursor	2.7258	3.86E-12
151	HIST1H2AL	H2A1_HUMAN	Histone H2A type 1	3.0424	1.08E-14
152	HLA-DQA1	HA25_HUMAN	HLA class II histocompatibility antigen, DQ(5	3.0173	3.2E-12
		HA22_HUMAN	HLA class II histocompatibility antigen, DQ(2	3.0173	3.2E-12
		HA24_HUMAN	HLA class II histocompatibility antigen, DQ(4	3.0173	3.2E-12
		HA27_HUMAN	HLA class II histocompatibility antigen, DQ(W3	3.0173	3.2E-12
		HA23_HUMAN	HLA class II histocompatibility antigen, DQ(3	3.0173	3.2E-12
153	HLF	HLF_HUMAN	Hepatic leukemia factor	3.1876	0.000205464
154	HNF4A	HNF4A_HUMAN	Hepatocyte nuclear factor 4-alpha	2.5158	0.000000241
155	HOXB2	HXB2_HUMAN	Homeobox protein Hox-B2	2.8104	0.04880314
156	HOXD3	HXD3_HUMAN	Homeobox protein Hox-D3	3.0905	0.000554761
157	HR	HAIR_HUMAN	Protein hairless	5.342	0
158	HSD3B2	3BHS2_HUMAN	3 beta-hydroxysteroid dehydrogenase/delta 5-->4-isomerase type II(3Beta-HSD II	2.7908	0.00000131
159	HSDL2			3.4117	9.87E-16
160	HYAL1	HYAL1_HUMAN	Hyaluronidase-1 precursor	2.6493	0.00000132
161	ID1	ID1_HUMAN	DNA-binding protein inhibitor ID-1	4.1905	0.0000131
162	IDS	IDS_HUMAN	Iduronate 2-sulfatase precursor	3.0347	1.95E-10
163	IFITM1	IFM1_HUMAN	Interferon-induced transmembrane protein 1	3.8245	2.98E-09
164	IGF1	IGF1A_HUMAN	Insulin-like growth factor IA precursor	3.3483	0.002914553
		IGF1B_HUMAN	Insulin-like growth factor IB precursor	3.3483	0.002914553

165	IGHD	IGHD_HUMAN	Ig delta chain C region	3.4828	7.36E-21
166	IGJ	IGJ_HUMAN	Immunoglobulin J chain	5.179	0.009170781
167	IL11	IL11_HUMAN	Interleukin-11 precursor	2.8587	2.91E-12
168	IL7	IL7_HUMAN	Interleukin-7 precursor	4.4021	0.00000121
169	IRX5	IRX5_HUMAN	Iroquois-class homeodomain protein IRX-5	3.8157	0.000269469
170	ITGB3	ITB3_HUMAN	Integrin beta-3 precursor	6.2009	5E-34
171	ITSN1	ITSN1_HUMAN	Intersectin-1	4.0611	1.75E-19
172	KAL1	KALM_HUMAN	Anosmin-1 precursor	2.5728	0.000155598
173	KCNA1	KCNA1_HUMAN	Potassium voltage-gated channel subfamily A member 1	2.8738	0.000000619
174	KCNA3	KCNA3_HUMAN	Potassium voltage-gated channel subfamily A member 3	3.2375	0.0000192
175	KCTD12	KCD12_HUMAN	BTB/POZ domain-containing protein KCTD12	3.0269	0.00000057
176	KIAA0256	K0256_HUMAN	Protein KIAA0256	4.3347	2.96E-14
177	KIAA0408	K0408_HUMAN	Protein KIAA0408	2.64	0.000135859
178	KIAA0409	HUCE1_HUMAN	Cerebral protein 1	3.9678	1.25E-11
179	KIAA0506			5.1457	0.00000467
180	KIAA1005			3.2008	0.005764397
181	KMO	KMO_HUMAN	Kynurenine 3-monooxygenase	2.779	3.31E-10
182	KRT12	K1C12_HUMAN	Keratin, type I cytoskeletal 12	3.0649	0.000000475
183	KYNU	KYNU_HUMAN	Kynureninase	2.5315	1.05E-13
184	L3MBTL	LMBTL_HUMAN	Lethal(3)malignant brain tumor-like protein	3.8429	0.00135024
185	LAMA4	LAMA4_HUMAN	Laminin alpha-4 chain precursor	3.6992	0.00000043
186	LCMT2	LCMT2_HUMAN	Leucine carboxyl methyltransferase 2	2.984	0.00000035
187	LCP2	LCP2_HUMAN	Lymphocyte cytosolic protein 2	2.5318	0.003699768
188	LIPC	LIPH_HUMAN	Hepatic triacylglycerol lipase precursor	3.7566	3.89E-27
189	LOC283824			3.2537	0.0000169
190	LOC284323			2.6901	0.003400906
191	LOC441282	LOC441282	similar to aldo-keto reductase family 1, member B10; aldose reductase-like 1; aldo-keto reductase family 1, member B11 (aldose reductase-like); aldose reductase-like peptide; aldose reductase-related protein; small intestine reductase ...	3.0579	0.00052445
192	LOC643676			3.9755	0.000153562
193	LOC646621			3.3833	0.000117727
194	LOC647537			2.5488	0.001044927
195	LOC648759			2.5488	0.001044927
196	LOC650946			3.0173	3.2E-12
197	LOC652331			3.5474	0.000211182
198	LOC90586			2.7089	0.00000147
199	LOC92973			4.7843	8.05E-09
200	LOH11CR2A	LHR2A_HUMAN	Loss of heterozygosity 11 chromosomal region 2 gene A protein	5.036	0.002508805
201	LRMP	LRMP_HUMAN	Lymphoid-restricted membrane protein	3.9611	0.000000157
202	LRP4	LRP4_HUMAN	Low-density lipoprotein receptor-related protein 4 precursor	5.3204	9.83E-13
203	LRRTM2	LRTM2_HUMAN	Leucine-rich repeat transmembrane neuronal protein 2 precursor(Leucine-rich repeat neuronal 2 protein	2.6621	0.004756818
204	LY75	LY75_HUMAN	Lymphocyte antigen 75 precursor	2.9122	0.02242676
205	MAGEA12	MAGAC_HUMAN	Melanoma-associated antigen 12	2.7254	3.72E-08

206	MAGEA3	MAGA3_HUMAN	Melanoma-associated antigen 3	2.5218	6.22E-10
207	MAGEA6	MAGA6_HUMAN	Melanoma-associated antigen 6	2.5218	6.22E-10
208	MAP7	MAP7	microtubule-associated protein 7	2.6621	0.000113206
209	MASS1	MASS1_HUMAN	Monogenic audiogenic seizure susceptibility protein 1 homologprecursor	2.9947	0.005092431
210	MBP	MBP_HUMAN	Myelin basic protein	2.7685	3.15E-08
211	MCFP	MCFP	mitochondrial carrier family protein	2.797	0.004804249
212	MDFIC	MDFIC	MyoD family inhibitor domain containing	2.6255	6.44E-08
213	METTL7A	METTL7A	methyltransferase like 7A	3.0669	0.00000134
214	MFAP4	MFAP4_HUMAN	Microfibril-associated glycoprotein 4 precursor	3.1538	0.000222423
215	MID1	TRI18_HUMAN	Midline-1	2.8035	0.000002
216	MLANA	MAR1_HUMAN	Melanoma antigen recognized by T-cells 1	2.6091	0.007842831
217	MPHOSPH9	MPP9_HUMAN	M-phase phosphoprotein 9	2.5012	0.001593543
218	MSR1	MSRE_HUMAN	Macrophage scavenger receptor types I and II	4.0123	1.74E-08
219	MTUS1	MTUS1	mitochondrial tumor suppressor 1	5.1949	0.000623068
220	MYBPC2	MYPC2_HUMAN	Myosin-binding protein C, fast-type	2.637	0.000000225
221	MYL3	MYL3_HUMAN	Myosin light polypeptide 3	2.6973	0.000161809
222	MYOD1	MYOD1_HUMAN	Myoblast determination protein 1	2.7237	2.19E-17
223	NET1	ARHG8_HUMAN	Neuroepithelial cell-transforming gene 1 protein	3.1272	3.27E-08
224	NFATC1	NFAC1_HUMAN	Nuclear factor of activated T-cells, cytoplasmic 1	2.5774	0.001359451
225	NKTR	NKTR_HUMAN	NK-tumor recognition protein	3.2932	6.15E-21
226	NNMT	NNMT_HUMAN	Nicotinamide N-methyltransferase	2.6935	0.000486813
227	NR5A2	NR5A2_HUMAN	Orphan nuclear receptor NR5A2	2.7318	0.003752936
228	NRP1	NRP1_HUMAN	Neuropilin-1 precursor	4.3605	0.000000106
229	NTRK2	NTRK2_HUMAN	BDNF/NT-3 growth factors receptor precursor	7.107	0.00000262
230	OLFM1	NOE1_HUMAN	Noelin precursor	2.6893	0.000157511
231	OLFM4	OLFM4	olfactomedin 4	4.3272	3.58E-16
232	OLFML1	OLFL1_HUMAN	Olfactomedin-like protein 1 precursor	4.8467	0.00000546
233	OLIG2	OLIG2_HUMAN	Oligodendrocyte transcription factor 2	3.3829	2.71E-10
234	OMD	OMD_HUMAN	Osteomodulin precursor	3.3659	0.000630865
235	OPRM1	OPRM_HUMAN	Mu-type opioid receptor	2.7567	4.56E-16
236	OTC	OTC_HUMAN	Ornithine carbamoyltransferase, mitochondrial precursor	3.3035	0.00000107
237	PAGE1	GAGB1_HUMAN	G antigen family B member 1	2.8716	0.00000209
238	PAK3	PAK3_HUMAN	Serine/threonine-protein kinase PAK 3	2.695	0.000208796
239	PBX1	PBX1_HUMAN	Pre-B-cell leukemia transcription factor 1	2.7005	4.53E-08
240	PDE1A	PDE1A_HUMAN	Calcium/calmodulin-dependent 3',5'-cyclic nucleotide phosphodiesterase1A	4.409	0.01328391
241	PDE6B	PDE6B_HUMAN	Rod cGMP-specific 3',5'-cyclic phosphodiesterase beta-subunitprecursor	2.9319	0.0000193
242	PDGFRA	PGFRA_HUMAN	Alpha platelet-derived growth factor receptor precursor	3.1611	0.00046774
243	PDPN	PDPN_HUMAN	Podoplanin precursor	2.7051	0.004212139
244	PDYN	PDYN_HUMAN	Beta-neoendorphin-dynorphin precursor	2.8086	3.95E-10
245	PLA2G7	PAFA_HUMAN	Platelet-activating factor acetylhydrolase precursor	4.1337	0.00000689
246	PLA2R1	PLA2R1	phospholipase A2 receptor 1, 180kDa	6.7382	9.19E-37
247	PLEK	PLEK_HUMAN	Pleckstrin	2.9846	0.000573752
248	PLEKHA6	PKHA6_HUMAN	Pleckstrin homology domain-containing family A member 6(Phosphoinositol 3-phosphate-binding protein 3	4.2076	1.55E-09

249	PLIN	PLIN_HUMAN	Perilipin	3.0884	0.00000168
250	POLE2	DPOE2_HUMAN	DNA polymerase epsilon subunit 2	2.6182	0.0000001
251	POLR3G	RPO3G_HUMAN	DNA-directed RNA polymerase III subunit G	2.7338	0.00570278
252	PPAP2B	LPP3_HUMAN	Lipid phosphate phosphohydrolase 3	4.7712	4.35E-23
253	PPL	PEPL_HUMAN	Periplakin	3.6452	0.0000316
254	PPP2R2B	2ABB_HUMAN	Serine/threonine-protein phosphatase 2A 55 kDa regulatory subunit Bbeta isoform	2.5018	0.02740569
255	PRKCBP1	PKCB1_HUMAN	Protein kinase C-binding protein 1	2.6999	0.001846307
256	PRL	PRL_HUMAN	Prolactin precursor	2.8161	1.23E-09
257	PTGDS	PTGDS_HUMAN	Prostaglandin-H2 D-isomerase precursor	8.3616	0.003454162
258	PTH	PTHY_HUMAN	Parathyroid hormone precursor	3.9862	2.78E-15
259	PTPN22	PTN22_HUMAN	Tyrosine-protein phosphatase non-receptor type 22	2.848	5.33E-11
260	PURA	PURA_HUMAN	Transcriptional activator protein Pur-alpha	2.8074	0.00000107
261	PYGL	PYGL_HUMAN	Glycogen phosphorylase, liver form	2.5107	0.000000118
262	RARRES3	TIG3_HUMAN	Retinoic acid receptor responder protein 3	2.7516	0.00000385
263	RBP1	RET1_HUMAN	Retinol-binding protein I, cellular	5.0694	0.000796312
264	RDS	RDS_HUMAN	Peripherin	3.9507	0.000166622
265	RHAG	RHAG_HUMAN	Rhesus blood group-associated glycoprotein	3.7131	0.0000113
266	RIMBP2	RIMB2_HUMAN	RIM-binding protein 2	2.7688	0.000251503
267	RNASE1	RNAS1_HUMAN	Ribonuclease pancreatic precursor	4.4764	3.85E-16
268	RPA2	RFA2_HUMAN	Replication protein A 32 kDa subunit	2.531	0.000000344
269	RUNX1T1	MTG8_HUMAN	Protein CBFA2T1	3.7871	0.00000187
270	S100A12	S10AC_HUMAN	Protein S100-A12	2.6648	3.45E-09
271	SCD	ACOD_HUMAN	Acyl-CoA desaturase	2.597	0.01157059
272	SCEL	SCEL_HUMAN	Sciellin	3.1066	0.00069525
273	SEPP1	SEPP1_HUMAN	Selenoprotein P precursor	10.388	0.000133288
274	SEPT11	SEP11_HUMAN	Septin-11	3.5394	0.00000102
275	SFRP1	SFRP1_HUMAN	Secreted frizzled-related protein 1 precursor	6.1863	0.000307299
276	SFRP4	SFRP4_HUMAN	Secreted frizzled-related protein 4 precursor	3.0252	0.0000145
277	SGCD	SGCD_HUMAN	Delta-sarcoglycan	2.5844	0.001998139
278	SKP2	SKP2_HUMAN	S-phase kinase-associated protein 2	4.2172	0.000527083
279	SLC12A3	S12A3_HUMAN	Solute carrier family 12 member 3	2.8749	0.000330078
280	SLC12A5	S12A5_HUMAN	Solute carrier family 12 member 5	2.9794	0.001344639
281	SLC13A3	S13A3_HUMAN	Solute carrier family 13 member 3	3.6538	1.21E-14
282	SLC22A2	SLC22A2	solute carrier family 22 (organic cation transporter), member 2	2.8309	0.000415544
283	SLC22A3	S22A3_HUMAN	Organic cation transporter 3	2.5079	0.000023
284	SLC30A10	SLC30A10	solute carrier family 30, member 10	2.7415	2.32E-19
285	SLC7A2	CTR2_HUMAN	Low-affinity cationic amino acid transporter 2	3.215	0.001262795
286	SLCO1B1	SO1B1_HUMAN	Solute carrier organic anion transporter family member 1B1	4.2798	0.000000468
287	SMAD6	SMAD6_HUMAN	Mothers against decapentaplegic homolog 6	3.4764	0.009636884
288	SNED1			5.105	3.85E-09
289	SP1	SP1_HUMAN	Transcription factor Sp1	2.5927	0.0000645
290	SP4	SP4_HUMAN	Transcription factor Sp4	3.3197	0.0000396
291	SPBC25	SPBC25	spindle pole body component 25 homolog (S. cerevisiae)	2.5941	0.000000945
292	SPINK2	IPK2_HUMAN	Serine protease inhibitor Kazal-type 2 precursor	2.6673	1.5E-11
293	SPP1	OSTP_HUMAN	Osteopontin precursor	2.7188	0.0000109
294	SSH2	SSH2_HUMAN	Protein phosphatase Slingshot homolog 2	2.6164	0.000915855

295	ST6GAL1	SIAT1_HUMAN	CMP-N-acetylneuraminate-beta-galactosamide-alpha-2,6-sialyltransferase(EC 2.4.99.1	2.5325	0.000000242
296	ST6GALNAC2	SIA7B_HUMAN	Alpha-N-acetylgalactosaminide alpha-2,6-sialyltransferase 2(EC 2.4.99.-	2.5671	0.04895604
297	STAT5A	STA5A_HUMAN	Signal transducer and activator of transcription 5A	2.7999	0.000574118
298	STRN	STRN_HUMAN	Striatin	2.589	0.000195835
299	TADA2L	TAD2L_HUMAN	Transcriptional adapter 2-like	2.9389	0.0000902
300	TCF21	TCF21_HUMAN	Transcription factor 21	3.8149	0.000000104
301	TFCP2	TFCP2_HUMAN	Alpha-globin transcription factor CP2	3.2522	0.0000346
302	THY1	THY1_HUMAN	Thy-1 membrane glycoprotein precursor	5.8679	0.003453026
303	TIE1	TIE1_HUMAN	Tyrosine-protein kinase receptor Tie-1 precursor	4.7332	0.000000764
304	TLR1	TLR1_HUMAN	Toll-like receptor 1 precursor	2.7141	0.01027853
305	TNFRSF11A	TNR11_HUMAN	Tumor necrosis factor receptor superfamily member 11A precursor(Receptor activator of NF-KB	3.4349	0.0000206
306	TNNC2	TNNC2_HUMAN	Troponin C, skeletal muscle	2.6271	0.000490032
307	TNNI1	TNNI1_HUMAN	Troponin I, slow skeletal muscle	2.7195	0.00000368
308	TNP1	STP1_HUMAN	Spermatid nuclear transition protein 1	2.5438	0.0000669
309	TRAT1	TRAT1_HUMAN	T-cell receptor-associated transmembrane adapter 1	2.6575	0.04447695
310	TRAV20			4.8308	6.61E-14
311	TSPAN2	TSN2_HUMAN	Tetraspanin-2	2.739	7.29E-12
312	TXK	TXK_HUMAN	Tyrosine-protein kinase TXK	2.9511	0.0000486
313	TYRO3	TYRO3_HUMAN	Tyrosine-protein kinase receptor TYRO3 precursor	2.9325	0.000408015
314	UNC5C	UNC5C_HUMAN	Netrin receptor UNC5C precursor	3.0103	0.000000116
315	VCAM1	VCAM1_HUMAN	Vascular cell adhesion protein 1 precursor	6.0684	0.03299872
316	VIL1	VILI_HUMAN	Villin-1	2.6205	0.004387872
317	VIP	VIP_HUMAN	VIP peptides precursor [Contains: Intestinal peptide PHV-42	2.9228	0.0000835
318	WASL	WASL_HUMAN	Neural Wiskott-Aldrich syndrome protein	4.7545	5.29E-118
319	WIF1	WIF1_HUMAN	Wnt inhibitory factor 1 precursor	3.0979	0.0000659
320	WISP1	WISP1_HUMAN	WNT1-inducible signaling pathway protein 1 precursor	2.5846	0.01520636
321	WIT1	WIT1_HUMAN	Wilms' tumor-associated protein	2.7959	0.000000116
322	WNT2B	WNT2B_HUMAN	Protein Wnt-2b precursor	2.7731	9.95E-12
323	WSB1	WSB1_HUMAN	WD repeat and SOCS box-containing protein 1	5.4062	1.29E-13
324	WT1	WT1_HUMAN	Wilms' tumor protein	2.7959	0.000000116
325	WWP1	WWP1_HUMAN	NEDD4-like E3 ubiquitin-protein ligase WWP1	2.7226	0.006464572
326	XPC	XPC_HUMAN	DNA-repair protein complementing XP-C cells	2.941	0.000259616
327	YLPM1	YLPM1_HUMAN	YLP motif-containing protein 1	2.9514	0.0000967
328	ZFP36L2	TISD_HUMAN	Butyrate response factor 2	2.5372	0.000000629
329	ZNF124	ZN124_HUMAN	Zinc finger protein 124	2.6621	1.26E-09
330	ZNF165	ZN165_HUMAN	Zinc finger protein 165	4.2986	0.004755043
331	ZNF202	ZN202_HUMAN	Zinc finger protein 202	2.9305	0.009648376
332	ZNF250	ZN250_HUMAN	Zinc finger protein 250	3.1371	0.000311946
333	ZNF272	ZN460_HUMAN	Zinc finger protein 460	2.6297	0.00000157
334	ZNF409	ZN409_HUMAN	Zinc finger protein 409	2.66	0.00000269
335	ZNF536	ZNF536	zinc finger protein 536	3.1515	4.13E-12

Group 1 and 2 combined, up-regulated AFFI gene IDs, fold change > 2.5

#	Affi Gene ID	Gene NetworkSymbol	Encoding protein name
1	39981_at	08p22/MSR1	MSR1("macrophage scavenger receptor 1")
2	36040_at	21-GARP	SH3BGR("SH3 domain binding glutamic acid-rich protein")
3	36963_at	6PGD	PGD("phosphogluconate dehydrogenase")
4	36808_at	70Z-PEP	PTPN22("protein tyrosine phosphatase, non-receptor type 22
5	31578_at	ABCC7	CFTR("cystic fibrosis transmembrane conductance regulator, ATP-
6	39654_at	ACY2	ASPA("aspartoacylase (Canavan disease)")
7	37789_at	ADA2	TADA2L("transcriptional adaptor 2 (ADA2 homolog, yeast)-like")
8	36269_at	ADAM-TS 3	ADAMTS3("ADAM metallopeptidase with thrombospondin type 1
9	1227_g_at	ADAM17	ADAM17("ADAM metallopeptidase domain 17 (tumor necrosis factor,
10	35391_at	ADAM22	ADAM22("ADAM metallopeptidase domain 22")
11	37091_g_at	ADAM3A	ADAM3A("ADAM metallopeptidase domain 3a (cyritestin 1)")
12	34637_f_at	ADHA	ADH1A("alcohol dehydrogenase 1A (class I), alpha polypeptide")
13	35730_at	ADHB	ADH1B("alcohol dehydrogenase IB (class I), beta polypeptide")
14	34637_f_at,	ADHG	ADH1C("alcohol dehydrogenase 1C (class I), gamma polypeptide")
15	36497_at	AHNAK2	C14orf78("chromosome 14 open reading frame 78")
16	32112_s_at	AIM1	AIM1("absent in melanoma 1")
17	37482_at	AK1BA	AKR1B10("aldo-keto reductase family 1, member B10 (aldose
18	32421_at	AKAP5	AKAP5("A kinase (PRKA) anchor protein 5")
19	32805_at	AKR1C2	AKR1C2("aldo-keto reductase family 1, member C2 (dihydrodiol
20	37399_at	AKR1C3	AKR1C3("aldo-keto reductase family 1, member C3 (3-alpha
21	38503_at	AL1B1	ALDH1B1("aldehyde dehydrogenase 1 family, member B1")
22	40409_at	AL3A2	ALDH3A2("aldehyde dehydrogenase 3 family, member A2")
23	36686_at,	ALDH 1/3 *	ALDH1A2("aldehyde dehydrogenase 1 family, member A2"),
24	34056_g_at	ALK-4	ACVR1B("activin A receptor, type IB")
25	36623_at	ALPL	ALPL("alkaline phosphatase, liver/bone/kidney")
26	35638_at	AML1/MDS1/EVI1 fusion	MDS1("myelodysplasia syndrome 1")
27	36680_at	AMYS	AMY1A("amylase, alpha 1A; salivary")
28	35624_at	ANKRD6	ANKRD6("ankyrin repeat domain 6")
29	35593_at	AOC2	AOC2("amine oxidase, copper containing 2 (retina-specific)")
30	40303_at	AP-2C	TFAP2C("transcription factor AP-2 gamma (activating enhancer
31	33732_at	AP4M1	AP4M1("adaptor-related protein complex 4, mu 1 subunit")
32	41472_at	APOBEC3G	APOBEC3G("apolipoprotein B mRNA editing enzyme, catalytic
33	36681_at	APOD	APOD("apolipoprotein D")
34	35352_at	ARNT2	ARNT2("aryl-hydrocarbon receptor nuclear translocator 2")
35	40541_at	ASSY	ASS("argininosuccinate synthetase")
36	38013_at	ATIP1	MTUS1("mitochondrial tumor suppressor 1")
37	2001_g_at	ATM	ATM("ataxia telangiectasia mutated (includes complementation
38	34377_at	ATP1A2	ATP1A2("ATPase, Na+/K+ transporting, alpha 2 (+) polypeptide")
39	38448_at	Alpha-actinin 2	ACTN2("actinin, alpha 2")
40	32728_at	Amphiphysin I	AMPH("amphiphysin (Stiff-Man syndrome with breast cancer 128kDa
41	1577_at	Androgen receptor	AR("androgen receptor (dihydrotestosterone receptor; testicular
42	37461_at	Angiopoietin 2	ANGPT2("angiopoietin 2")
43	39850_at	Ankyrin-B	ANK2("ankyrin 2, neuronal")
44	32320_at	Annexin A13	ANXA13("annexin A13")
45	34435_at	Aquaporin 9	AQP9("aquaporin 9")
46	39310_at	B2 bradykinin receptor	BDKRB2("bradykinin receptor B2")
47	37678_at	BAMBI	BAMBI("BMP and activin membrane-bound inhibitor homolog
48	38339_at	BBOX1	BBOX1("butyrobetaine (gamma), 2-oxoglutarate dioxygenase
49	41683_i_at	BCKD-E1beta	BCKDHB("branched chain keto acid dehydrogenase E1, beta
50	41245_at	BMP3B	GDF10("growth differentiation factor 10")
51	1114_at,	BMP4	BMP4("bone morphogenetic protein 4")
52	37054_at	BPI	BPI("bactericidal/permeability-increasing protein")
53	37102_at	BRMS1	BRMS1("breast cancer metastasis suppressor 1")
54	39061_at	BST2	BST2("bone marrow stromal cell antigen 2")
55	40313_at	Basonuclin	BNC1("basonuclin 1")

56	35128_at	Bombesin receptor	BRS3("bombesin-like receptor 3")
57	1052_s_at	C/EBPdelta	CEBPD("CCAAT/enhancer binding protein (C/EBP), delta")
58	39409_at	C1r	C1R("complement component 1, r subcomponent")
59	40496_at	C1s	C1S("complement component 1, s subcomponent")
60	35822_at	Complement Factor B	CFB("complement factor B")
61	37394_at	C7	C7("complement component 7")
62	38169_s_at	CAT-2	SLC7A2("solute carrier family 7 (cationic amino acid transporter, y+
63	1183_at	CCL17	CCL17("chemokine (C-C motif) ligand 17")
64	35000_at	CD137 ligand(TNFSF9)	TNFSF9("tumor necrosis factor (ligand) superfamily, member 9")
65	36661_s_at	CD14	CD14("CD14 antigen")
66	37078_at	CD3 zeta	CD3Z("CD3Z antigen, zeta polypeptide (TiT3 complex)")
67	34760_at	CD302	CD302("CD302 antigen")
68	33945_at	CD40L(TNFSF5)	CD40LG("CD40 ligand (TNF superfamily, member 5, hyper-IgM
69	36797_at	CD43	SPN("sialophorin (gpL115, leukosialin, CD43)")
70	1584_at	CDC25C	CDC25C("cell division cycle 25C")
71	33299_at	CDC2L2	CDC2L2("cell division cycle 2-like 2 (PITSLRE proteins)")
72	35649_at	CDO1	CDO1("cysteine dioxygenase, type I")
73	40217_s_at	CDS1	CDS1("CDP-diacylglycerol synthase (phosphatidate
74	1582_at	CEACAM5	CEACAM5("carcinoembryonic antigen-related cell adhesion molecule
75	37203_at	CES1	CES1("carboxylesterase 1 (monocyte/macrophage serine esterase
76	33310_at	CGI58	ABHD5("abhydrolase domain containing 5")
77	36197_at	CHI3L1	CHI3L1("chitinase 3-like 1 (cartilage glycoprotein-39)")
78	32988_at	CLCN voltage-gated *	CLCNKA("chloride channel Ka")
79	39842_at	CLF-1	CRLF1("cytokine receptor-like factor 1")
80	40013_at	CLIC2	CLIC2("chloride intracellular channel 2")
81	36906_at	CNR1	CNR1("cannabinoid receptor 1 (brain)")
82	38469_at	CO-029	TSPAN8("tetraspanin 8")
83	33562_g_at	COL4A3	COL4A3("collagen, type IV, alpha 3 (Goodpasture antigen)")
84	35493_at	COL4A4	COL4A4("collagen, type IV, alpha 4")
85	40368_r_at	CPN2	CPN2("carboxypeptidase N, polypeptide 2, 83kD")
86	35757_at	CPSF6	CPSF6("cleavage and polyadenylation specific factor 6, 68kDa")
87	37248_at	CPZ	CPZ("carboxypeptidase Z")
88	1057_at	CRABP2	CRABP2("cellular retinoic acid binding protein 2")
89	35561_at	CRCM	MCC("mutated in colorectal cancers")
90	34995_at	CRLR	CALCRL("calcitonin receptor-like")
91	39618_at	CRTL1	HAPLN1("hyaluronan and proteoglycan link protein 1")
92	823_at	CX3CL1	CX3CL1("chemokine (C-X3-C motif) ligand 1")
93	37534_at	CXADR	CXADR("coxsackie virus and adenovirus receptor")
94	1024_at	CYP1A1	CYP1A1("cytochrome P450, family 1, subfamily A, polypeptide 1")
95	999_at	CYP27A1	CYP27A1("cytochrome P450, family 27, subfamily A, polypeptide 1")
96	38909_at	CYP27B1	CYP27B1("cytochrome P450, family 27, subfamily B, polypeptide 1")
97	1455_f_at	CYP2C9	CYP2C9("cytochrome P450, family 2, subfamily C, polypeptide 9")
98	1436_at	CYP2D6	CYP2D6("cytochrome P450, family 2, subfamily D, polypeptide 6")
99	1756_f_at	CYP3A3	CYP3A4("cytochrome P450, family 3, subfamily A, polypeptide 4")
100	31926_at	CYP7A1	CYP7A1("cytochrome P450, family 7, subfamily A, polypeptide 1")
101	40356_at	CLCA2	CLCA2("chloride channel, calcium activated, family member 2")
102	38879_at	Calgranulin C	S100A12("S100 calcium binding protein A12 (calgranulin C)")
103	38391_at	CapG	CAPG("capping protein (actin filament), gelsolin-like")
104	36454_at	Carbonic anhydrase XII	CA12("carbonic anhydrase XII")
105	39320_at,	Caspase-1	CASP1("caspase 1, apoptosis-related cysteine peptidase (interleukin
106	195_s_at	Caspase-4	CASP4("caspase 4, apoptosis-related cysteine peptidase")
107	128_at,	Cathepsin K	CTSK("cathepsin K (pycnodysostosis)")
108	37887_at	Chk2	CHEK2("CHK2 checkpoint homolog (S. pombe)")
109	36422_s_at	Chordin	CHRD("chordin")
110	41764_at	APOC1	APOC1("apolipoprotein C-I")
111	36780_at	Clusterin	CLU("clusterin")
112	35245_at	Coagulation factor V	F5("coagulation factor V (proaccelerin, labile factor)")
113	33562_g_at,	Collagen IV *	COL4A3("collagen, type IV, alpha 3 (Goodpasture antigen)"),
114	37892_at	COL11A1	COL11A1("collagen, type XI, alpha 1")

115 34388_at	Collagen XIV	COL14A1("collagen, type XIV, alpha 1 (undulin)")
116 39581_at	Cystatin A	CSTA("cystatin A (stefin A)")
117 41385_at	DAL1	EPB41L3("erythrocyte membrane protein band 4.1-like 3")
118 40049_at	DAPK1	DAPK1("death-associated protein kinase 1")
119 40292_at	DBCCR1	DBC1("deleted in bladder cancer 1")
120 38957_at	DCAMKL1	DCAMKL1("doublecortin and CaM kinase-like 1")
121 41260_at	DDX17	DDX17("DEAD (Asp-Glu-Ala-Asp) box polypeptide 17")
122 38315_at	DHA2	ALDH1A2("aldehyde dehydrogenase 1 family, member A2")
123 36686_at	DHA6	ALDH1A3("aldehyde dehydrogenase 1 family, member A3")
124 34111_s_at	DISC1	DISC1("disrupted in schizophrenia 1")
125 33575_at	DNAM1	CD226("CD226 antigen")
126 38220_at	DPYD	DPYD("dihydropyrimidine dehydrogenase")
127 529_at	DUSP5	DUSP5("dual specificity phosphatase 5")
128 34991_at,	Delta-sarcoglycan	SGCD("sarcoglycan, delta (35kDa dystrophin-associated
129 33468_at	Desmoglein 2	DSG2("desmoglein 2")
130 38028_at	Dopamine transporter	SLC6A3("solute carrier family 6 (neurotransmitter transporter,
131 37081_at	Dynein, axonemal, heavy	DNAH7("dynein, axonemal, heavy polypeptide 7")
132 40319_at	Dynein, cytoplasmic,	DYNC1I1("dynein, cytoplasmic 1, intermediate chain 1")
133 35954_at	Dynorphin	PDYN("prodynorphin")
134 33355_at	E2A	TCF3("transcription factor 3 (E2A immunoglobulin enhancer binding
135 37863_at	EGR2 (Krox20)	EGR2("early growth response 2 (Krox-20 homolog, Drosophila)")
136 40375_at	EGR3	EGR3("early growth response 3")
137 31665_s_at	EIF2A	EIF2A("eukaryotic translation initiation factor 2A, 65kDa")
138 39642_at	ELOVL2	ELOVL2("elongation of very long chain fatty acids (FEN1/Elo2,
139 1321_s_at,	EMP1 (Tmp)	EMP1("epithelial membrane protein 1")
140 41123_s_at,	ENPP2	ENPP2("ectonucleotide pyrophosphatase/phosphodiesterase 2
141 1683_at	EWS/WT1 fusion protein *	WT1("Wilms tumor 1")
142 35226_at	EYA2	EYA2("eyes absent homolog 2 (Drosophila)")
143 34631_at	EYA4	EYA4("eyes absent homolog 4 (Drosophila)")
144 38497_at	Elk-4	ELK4("ELK4, ETS-domain protein (SRF accessory protein 1)")
145 39947_at	Ephrin-A4	EFNA4("ephrin-A4")
146 1234_at	Ephrin-A receptor 3	EPHA3("EPH receptor A3")
147 902_at	Ephrin-B receptor 2	EPHB2("EPH receptor B2")
148 34334_at,	Ephrin-B2	EFNB2("ephrin-B2")
149 1585_at,	ErbB3	ERBB3("v-erb-b2 erythroblastic leukemia viral oncogene homolog 3
150 41674_at	FGF12	FGF12("fibroblast growth factor 12")
151 1380_at,	FGF7	FGF7("fibroblast growth factor 7 (keratinocyte growth factor)")
152 1363_at,	FGFR2	FGFR2("fibroblast growth factor receptor 2 (bacteria-expressed
153 31805_at	FGFR3	FGFR3("fibroblast growth factor receptor 3 (achondroplasia,
154 39593_at	FGL2	FGL2("fibrinogen-like 2")
155 32249_at,	FHR-1	CFHR1("complement factor H-related 1")
156 36342_r_at	FHR-2	CFHR2("complement factor H-related 2")
157 1068_g_at	FLT3 ligand	FLT3LG("fms-related tyrosine kinase 3 ligand")
158 36319_at	FOXF2	FOXF2("forkhead box F2")
159 40230_at	FRZB1	FRZB("frizzled-related protein")
160 32250_at	Factor H	CFH("complement factor H")
161 37688_f_at	Fc gamma RII alpha	FCGR2A("Fc fragment of IgG, low affinity IIa, receptor (CD32)")
162 38026_at	Fibulin-1	FBLN1("fibulin 1")
163 32783_at	Fibulin-2	FBLN2("fibulin 2")
164 32551_at	Fibulin-3	EFEMP1("EGF-containing fibulin-like extracellular matrix protein 1")
165 38279_at	G-protein alpha-z	GNAZ("guanine nucleotide binding protein (G protein), alpha z
166 38326_at	G0/G1switch 2	G0S2("G0/G1switch 2")
167 41839_at,	GAS1	GAS1("growth arrest-specific 1")
168 33387_at	GAS7	GAS7("growth arrest-specific 7")
169 203_at	GATA-2	GATA2("GATA binding protein 2")
170 40511_at	GATA-3	GATA3("GATA binding protein 3")
171 37944_at	GCH1	GCH1("GTP cyclohydrolase 1 (dopa-responsive dystonia)")
172 35410_at	GCP2	CXCL6("chemokine (C-X-C motif) ligand 6 (granulocyte chemotactic
173 36309_at	GDF8	GDF8("growth differentiation factor 8")

174 37142_at	GFRalpha1	GFRA1("GDNF family receptor alpha 1")
175 34048_at	GKP2	GK2("glycerol kinase 2")
176 38379_at	GPNMB	GPNMB("glycoprotein (transmembrane) nmb")
177 34028_at	GPR19_HUMAN	GPR19("G protein-coupled receptor 19")
178 31764_at	GPR98	MASS1("monogenic, audiogenic seizure susceptibility 1 homolog
179 408_at	GRO-1	CXCL1("chemokine (C-X-C motif) ligand 1 (melanoma growth
180 37187_at,	GRO-beta	CXCL2("chemokine (C-X-C motif) ligand 2")
181 34022_at	GRO-gamma	CXCL3("chemokine (C-X-C motif) ligand 3")
182 273_g_at	GRP	GRP("gastrin-releasing peptide")
183 31850_at	GSH1	GCLC("glutamate-cysteine ligase, catalytic subunit")
184 38615_at	GW112	OLFM4("olfactomedin 4")
185 31329_at,	Galpha(q)-specific peptide	BDKRB2("bradykinin receptor B2"), BRS3("bombesin-like receptor
186 37215_at	Glycogen phosphorylase *	PYGL("phosphorylase, glycogen; liver (Hers disease, glycogen
187 38715_at	Glycophorin B	GYPB("glycophorin B (MNS blood group)")
188 36508_at	Glypican-4	GPC4("glypican 4")
189 35644_at	HEPH	HEPH("hephaestin")
190 37842_at	HIC	MDFIC("MyoD family inhibitor domain containing")
191 32773_at	HLA-DQA1 *	HLA-DQA2("major histocompatibility complex, class II, DQ alpha 2")
192 38627_at	HLF	HLF("hepatic leukemia factor")
193 36722_s_at	HNF4-alpha	HNF4A("hepatocyte nuclear factor 4, alpha")
194 39610_at	HOXB2	HOXB2("homeobox B2")
195 38293_s_at	HOXD3	HOXD3("homeobox D3")
196 39578_at	HR	HR("hairless homolog (mouse)")
197 34002_at	HSD3B2	HSD3B2("hydroxy-delta-5-steroid dehydrogenase, 3 beta- and
198 31360_at	GRP78	HSPA5("heat shock 70kDa protein 5 (glucose-regulated protein,
199 38790_at	HYEP	EPHX1("epoxide hydrolase 1, microsomal (xenobiotic)")
200 1232_s_at	IBP1	IGFBP1("insulin-like growth factor binding protein 1")
201 32640_at	ICAM1	ICAM1("intercellular adhesion molecule 1 (CD54), human rhinovirus
202 36617_at,	ID1	ID1("inhibitor of DNA binding 1, dominant negative helix-loop-helix
203 41215_s_at	ID2	ID2("inhibitor of DNA binding 2, dominant negative helix-loop-helix
204 32283_at	IDS	IDS("iduronate 2-sulfatase (Hunter syndrome)")
205 675_at	IFI17	IFITM1("interferon induced transmembrane protein 1 (9-27)")
206 425_at	IFI27	IFI27("interferon, alpha-inducible protein 27")
207 33118_at	IFRD2	IFRD2("interferon-related developmental regulator 2")
208 38737_at	IGF-1	IGF1("insulin-like growth factor 1 (somatomedin C)")
209 1591_s_at,	IGF-2	IGF2("insulin-like growth factor 2 (somatomedin A)")
210 37467_at	IGHM	IGHM("immunoglobulin heavy constant mu")
211 35464_at	IL-11	IL11("interleukin 11")
212 41630_at	IL-7	IL7("interleukin 7")
213 1369_s_at,	IL-8	IL8("interleukin 8")
214 1016_s_at	IL13RA2	IL13RA2("interleukin 13 receptor, alpha 2")
215 41677_at	IL15RA	IL15RA("interleukin 15 receptor, alpha")
216 36496_at	IMPA2	IMPA2("inositol(myo)-1(or 4)-monophosphatase 2")
217 40401_at	IRS6(DOK5)	DOK5("docking protein 5")
218 41348_at	IRX5	IRX5("iroquois homeobox protein 5")
219 41481_at	ITGA2	ITGA2("integrin, alpha 2 (CD49B, alpha 2 subunit of VLA-2
220 37952_at	ITGB3	ITGB3("integrin, beta 3 (platelet glycoprotein IIIa, antigen CD61)")
221 40775_at	ITM2A	ITM2A("integral membrane protein 2A")
222 37006_at	IgJ	IGJ("immunoglobulin J polypeptide, linker protein for
223 37952_at,	Integrin *	ITGA2("integrin, alpha 2 (CD49B, alpha 2 subunit of VLA-2
224 488_at	Intersectin	ITSN1("intersectin 1 (SH3 domain protein)")
225 1709_g_at	JNK(MAPK8-10) *	MAPK10("mitogen-activated protein kinase 10")
226 41484_r_at	JunD	JUND("jun D proto-oncogene")
227 1916_s_at,	JunD/c-Fos *	FOS("v-fos FBJ murine osteosarcoma viral oncogene homolog"),
228 33158_at	KAL1	KAL1("Kallmann syndrome 1 sequence")
229 34208_at	KCC2	SLC12A5("solute carrier family 12, (potassium-chloride transporter)
230 34087_at	KCNK2	KCNK2("potassium channel, subfamily K, member 2")
231 38972_at	KCTD12	KCTD12("potassium channel tetramerisation domain containing 12")
232 34213_at	KIBRA	WWC1("WW, C2 and coiled-coil domain containing 1")

233 39559_at	KMO	KMO("kynurenine 3-monooxygenase (kynurenine 3-hydroxylase)")
234 36489_at,	PRPS1	PRPS1("phosphoribosyl pyrophosphate synthetase 1")
235 38492_at	KYNU	KYNU("kynureninase (L-kynurenine hydrolase)")
236 34615_at	Keratin 12	KRT12("keratin 12 (Meesmann corneal dystrophy)")
237 32277_at,	Kv1 alpha subunits *	KCNA1("potassium voltage-gated channel, shaker-related subfamily,
238 33939_at	Kv1.1	KCNA1("potassium voltage-gated channel, shaker-related subfamily,
239 32277_at	Kv1.3	KCNA3("potassium voltage-gated channel, shaker-related subfamily,
240 34244_r_at	L3MBTL	L3MBTL("l(3)mbt-like (Drosophila)")
241 37671_at	LAMA4	LAMA4("laminin, alpha 4")
242 38914_at	LCMT2	LCMT2("leucine carboxyl methyltransferase 2")
243 38611_at	LIPC (HL)	LIPC("lipase, hepatic")
244 33862_at	PPAP2 *	PPAP2B("phosphatidic acid phosphatase type 2B")
245 35865_at	LRH1	NR5A2("nuclear receptor subfamily 5, group A, member 2")
246 36059_at	LRP4	LRP4("low density lipoprotein receptor-related protein 4")
247 34249_at	Laforin	EPM2A("epilepsy, progressive myoclonus type 2A, Lafora disease
248 38038_at	Lumican	LUM("lumican")
249 39613_at	MA1A1	MAN1A1("mannosidase, alpha, class 1A, member 1")
250 31480_f_at,	MAGE-6	MAGEA6("melanoma antigen family A, 6")
251 36513_at	MAGP (1,2) *	MFAP5("microfibrillar associated protein 5")
252 39732_at	MAP7(EMAP115)	MAP7("microtubule-associated protein 7")
253 35465_at	MCFP	MCFP("mitochondrial carrier family protein")
254 39841_at	MCT *	SLC16A6("solute carrier family 16 (monocarboxylic acid
255 33956_at	MD-2	LY96("lymphocyte antigen 96")
256 41730_at	MYL3	MYL3("myosin, light polypeptide 3, alkali; ventricular, skeletal,
257 38717_at	METTL7A	METTL7A("methyltransferase like 7A")
258 39066_at	MFAP4	MFAP4("microfibrillar-associated protein 4")
259 34296_at	MID1	MID1("midline 1 (Opitz/BBB syndrome)")
260 39933_at	MKP-X	DUSP7("dual specificity phosphatase 7")
261 1050_at	MLANA	MLANA("melan-A")
262 39007_at	MMP-2	MMP2("matrix metallopeptidase 2 (gelatinase A, 72kDa gelatinase,
263 681_at	MMP-8	MMP8("matrix metallopeptidase 8 (neutrophil collagenase)")
264 40398_s_at	MOX2	MEOX2("mesenchyme homeobox 2")
265 36834_at	MOXD1	MOXD1("monooxygenase, DBH-like 1")
266 38261_at	MRP3	ABCC3("ATP-binding cassette, sub-family C (CFTR/MRP), member
267 40199_at	MSX1	MSX1("msh homeobox homolog 1 (Drosophila)")
268 37941_at	MYBPC2	MYBPC2("myosin binding protein C, fast type")
269 33482_at	MYOD	MYOD1("myogenic differentiation 1")
270 33355_at,	MYOD/E47 *	MYOD1("myogenic differentiation 1"), TCF3("transcription factor 3
271 40502_r_at	MYPS	MYBPC1("myosin binding protein C, slow type")
272 38124_at	Midkine	MDK("midkine (neurite growth-promoting factor 2)")
273 34534_at	Mu-type opioid receptor	OPRM1("opioid receptor, mu 1")
274 35817_at	Myelin basic protein	MBP("myelin basic protein")
275 39875_at	N-WASP	WASL("Wiskott-Aldrich syndrome-like")
276 1111_at	NCAM1	NCAM1("neural cell adhesion molecule 1")
277 33767_at	NEFH	NEFH("neurofilament, heavy polypeptide 200kDa")
278 33894_at	NET1	NET1("neuroepithelial cell transforming gene 1")
279 39144_at	NF-AT2(NFATC1)	NFATC1("nuclear factor of activated T-cells, cytoplasmic, calcineurin-
280 41572_r_at,	NF-kB heterodimers *	NFKB2("nuclear factor of kappa light polypeptide gene enhancer in B-
535_s_at	NF-kB *	cells 2 (p49/p100)"), REL("v-rel reticuloendotheliosis viral oncogene
281 535_s_at	NF-kB2 (p100)	NFKB2("nuclear factor of kappa light polypeptide gene enhancer in B-
282 39119_s_at	NK4	IL32("interleukin 32")
283 37032_at	NNMT	NNMT("nicotinamide N-methyltransferase")
284 34282_at	NRF3	NFE2L3("nuclear factor (erythroid-derived 2)-like 3")
285 40676_at	NRIF3	ITGB3BP("integrin beta 3 binding protein (beta3-endonexin)")
286 37714_at	Neuromodulin	GAP43("growth associated protein 43")
287 36836_at	Neuropilin-1	NRP1("neuropilin 1")
288 41554_at	Nibrin	NBN("nibrin")
289 31329_at	Nk3R	TACR3("tachykinin receptor 3")
290 36134_at,	Noelin	OLFM1("olfactomedin 1")

291 185_at	Nova1	NOVA1("neuro-oncological ventral antigen 1")
292 37684_at	OATP-B	SLCO2B1("solute carrier organic anion transporter family, member
293 35570_at	OATP-C	SLCO1B1("solute carrier organic anion transporter family, member
294 35867_at	OCTN2 *	SLC22A2("solute carrier family 22 (organic cation transporter),
295 40624_at	OLIG2	OLIG2("oligodendrocyte lineage transcription factor 2")
296 32143_at	OSR2	OSR2("odd-skipped related 2 (Drosophila)")
297 683_at	OTC	OTC("ornithine carbamoyltransferase")
298 41031_at	Osteomodulin	OMD("osteomodulin")
299 34342_s_at	Osteopontin	SPP1("secreted phosphoprotein 1 (osteopontin, bone sialoprotein I,
300 33073_at	PAK3	PAK3("p21 (CDKN1A)-activated kinase 3")
301 38184_at	PAX2	PAX2("paired box gene 2")
302 32418_at,	PDE *	PDE1A("phosphodiesterase 1A, calmodulin-dependent"),
303 32418_at,	PDE1 *	PDE1A("phosphodiesterase 1A, calmodulin-dependent"),
304 36311_at	PDE1A	PDE1A("phosphodiesterase 1A, calmodulin-dependent")
305 32418_at,	PDE1C	PDE1C("phosphodiesterase 1C, calmodulin-dependent 70kDa")
306 39576_at	PDE6B	PDE6B("phosphodiesterase 6B, cGMP-specific, rod, beta (congenital
307 36157_at	PDGF receptor *	PDGFRA("platelet-derived growth factor receptor, alpha
308 33284_at	PERM	MPO("myeloperoxidase")
309 33772_at	PGE2R4	PTGER4("prostaglandin E receptor 4 (subtype EP4)")
310 38131_at	PGES	PTGES("prostaglandin E synthase")
311 216_at,	PGHD	PTGDS("prostaglandin D2 synthase 21kDa (brain)")
312 36287_at	PI3K cat class IB (p110-	PIK3CG("phosphoinositide-3-kinase, catalytic, gamma polypeptide")
313 37068_at	PLA2G7	PLA2G7("phospholipase A2, group VII (platelet-activating factor
314 35462_at	PLA2R1	PLA2R1("phospholipase A2 receptor 1, 180kDa")
315 40330_at	PLC-epsilon	PLCE1("phospholipase C, epsilon 1")
316 33807_at	PLEKHA6	PLEKHA6("pleckstrin homology domain containing, family A member
317 41085_at	POLE2	POLE2("polymerase (DNA directed), epsilon 2 (p59 subunit)")
318 31571_at	POLR3G	POLR3G("polymerase (RNA) III (DNA directed) polypeptide G
319 36890_at	PPL(periplakin)	PPL("periplakin")
320 1384_at	PPP2R2B	PPP2R2B("protein phosphatase 2 (formerly 2A), regulatory subunit
321 37116_at	PRKCBP1	PRKCBP1("protein kinase C binding protein 1")
322 37989_at	PTHrP	PTHLH("parathyroid hormone-like hormone")
323 39227_at	PTPRT	PTPRT("protein tyrosine phosphatase, receptor type, T")
324 41345_at	PUR-alpha	PURA("purine-rich element binding protein A")
325 708_at	Parathyroid hormone	PTH("parathyroid hormone")
326 37122_at	Perilipin	PLIN("perilipin")
327 40834_at	Pin1	PIN1("protein (peptidylprolyl cis/trans isomerase) NIMA-interacting
328 35724_at	Pirin	PIR("pirin (iron-binding nuclear protein)")
329 33475_at	Plakophilin 4	PKP4("plakophilin 4")
330 37328_at	Pleckstrin	PLEK("pleckstrin")
331 160021_r_at	Progesterone receptor	PGR("progesterone receptor")
332 878_s_at	Prolactin	PRL("prolactin")
333 35752_s_at	Protein S	PROS1("protein S (alpha)")
334 37902_at	Quinone oxidoreductase	CRYZ("crystallin, zeta (quinone reductase)")
335 32158_at	RAD9 *	RAD9A("RAD9 homolog A (S. pombe)")
336 40079_at	RAIG1	GPRC5A("G protein-coupled receptor, family C, group 5, member
337 32479_at	RANK(TNFRSF11A)	TNFRSF11A("tumor necrosis factor receptor superfamily, member
338 38634_at	RBP1	RBP1("retinol binding protein 1, cellular")
339 32552_at	RBP4	RBP4("retinol binding protein 4, plasma")
340 34288_at	RDC1	CMKOR1("chemokine orphan receptor 1")
341 38874_s_at	RDS	RDS("retinal degeneration, slow")
342 35705_at,	REV-ERB-BETA	NR1D2("nuclear receptor subfamily 1, group D, member 2")
343 40970_at	RFXAP	RFXAP("regulatory factor X-associated protein")
344 37701_at	RGS2	RGS2("regulator of G-protein signalling 2, 24kDa")
345 37637_at	RGS3	RGS3("regulator of G-protein signalling 3")
346 32663_at	RHAG	RHAG("Rh-associated glycoprotein")
347 36750_at	RIM-bp2	RIMBP2("RIMS binding protein 2")
348 31352_at	RNase 1	RNASE1("ribonuclease, RNase A family, 1 (pancreatic)")
	RPA2	

350	36460_at	RPA39	POLR1C("polymerase (RNA) I polypeptide C, 30kDa")
351	813_at	RPLP0	RPLP0("ribosomal protein, large, P0")
352	33717_at	Rab-22A	RAB22A("RAB22A, member RAS oncogene family")
353	41636_at	Rab11-FIP3	RAB11FIP3("RAB11 family interacting protein 3 (class II)")
354	32963_s_at	RagD	RRAGD("Ras-related GTP binding D")
355	32685_at	RalGEF2	RALGPS1("Ral GEF with PH domain and SH3 binding motif 1")
356	32552_at,	Retinol-binding protein *	RBP1("retinol binding protein 1, cellular"), RBP4("retinol binding
357	565_at	S5AR2	SRD5A2("steroid-5-alpha-reductase, alpha polypeptide 2 (3-oxo-5
358	38968_at	SAB	SH3BP5("SH3-domain binding protein 5 (BTK-associated)")
359	32985_at	SALL-1	SALL1("sal-like 1 (Drosophila)")
360	37513_at	SCD	SCD("stearoyl-CoA desaturase (delta-9-desaturase)")
361	35719_at	SCOP	PHLPP("PH domain and leucine rich repeat protein phosphatase")
362	32666_at	SDF-1	CXCL12("chemokine (C-X-C motif) ligand 12 (stromal cell-derived
363	34990_at	SETBP1	SETBP1("SET binding protein 1")
364	32521_at	SFRP1	SFRP1("secreted frizzled-related protein 1")
365	41405_at	SFRP4	SFRP4("secreted frizzled-related protein 4")
366	41352_at	SIAT1	ST6GAL1("ST6 beta-galactosamide alpha-2,6-sialyltranferase 1")
367	40290_f_at	SIAT4A	ST3GAL1("ST3 beta-galactoside alpha-2,3-sialyltransferase 1")
368	35579_at	SLAC2-B	EXPH5("exophilin 5")
369	36362_at	SLC12A3	SLC12A3("solute carrier family 12 (sodium/chloride transporters),
370	41368_at	SLC13A3	SLC13A3("solute carrier family 13 (sodium-dependent dicarboxylate
371	37601_at	SLC22A3	SLC22A3("solute carrier family 22 (extraneuronal monoamine
372	40456_at	SLC39A8	SLC39A8("solute carrier family 39 (zinc transporter), member 8")
373	37489_s_at	SLC4A3	SLC4A3("solute carrier family 4, anion exchanger, member 3")
374	41271_at	SLC7A8	SLC7A8("solute carrier family 7 (cationic amino acid transporter, y+
375	942_at	SM30	RGN("regucalcin (senescence marker protein-30)")
376	1955_s_at,	SMAD6	SMAD6("SMAD, mothers against DPP homolog 6 (Drosophila)")
377	40952_at	SMAR1	BANP("BTG3 associated nuclear protein")
378	34666_at	SOD2	SOD2("superoxide dismutase 2, mitochondrial")
379	1156_at	SP1	SP1("Sp1 transcription factor")
380	32411_at	SP4	SP4("Sp4 transcription factor")
381	38940_at	SPBC25	SPBC25("spindle pole body component 25 homolog (S. cerevisiae)")
382	34304_s_at	SSAT	SAT("spermidine/spermine N1-acetyltransferase")
383	40321_at	ST2(L)	IL1RL1("interleukin 1 receptor-like 1")
384	35350_at	ST4S6	GALNAC4S-6ST("B cell RAG associated protein")
385	473_g_at,	STAT5 *	STAT5A("signal transducer and activator of transcription 5A"),
386	506_s_at	STAT5A	STAT5A("signal transducer and activator of transcription 5A")
387	473_g_at	STAT5B	STAT5B("signal transducer and activator of transcription 5B")
388	40069_at	SVIL	SVIL("supervillin")
389	41692_at	SYNJ1	SYNJ1("synaptojanin 1")
390	35105_at	Sciellin	SCEL("sciellin")
391	36924_r_at	Secretogranin II	SCG2("secretogranin II (chromogranin C)")
392	34363_at	Selenoprotein P	SEPP1("selenoprotein P, plasma, 1")
393	33171_s_at	Septin 11	SEPT11("septin 11")
394	36245_at	HTR2B	HTR2B("5-hydroxytryptamine (serotonin) receptor 2B")
395	39449_at	Skp2/TrCP/FBXW7 *	SKP2("S-phase kinase-associated protein 2 (p45)")
396	39319_at	Slp76	LCP2("lymphocyte cytosolic protein 2 (SH2 domain containing
397	33298_at	Striatin	STRN("striatin, calmodulin binding protein")
398	41870_at,	T1A-2	PDPN("podoplanin")
399	291_s_at	TACSTD2 (TROP2)	TACSTD2("tumor-associated calcium signal transducer 2")
400	31430_at	TCR alpha/beta *	TRAV10("T cell receptor alpha variable 10
401	40085_s_at	TFCP2	TFCP2("transcription factor CP2")
402	1897_at	TGF-beta receptor type III	TGFBR3("transforming growth factor, beta receptor III (betaglycan,
403	1001_at	TIE	TIE1("tyrosine kinase with immunoglobulin-like and EGF-like
404	1042_at	TIG1	RARRES1("retinoic acid receptor responder (tazarotene induced) 1")
405	33236_at	TIG3	RARRES3("retinoic acid receptor responder (tazarotene induced) 3")
406	36243_at	TLR1	TLR1("toll-like receptor 1")
407	36070_at	TMEM2L	KIAA1199("KIAA1199")
		TPL2(MAP3K8)	

409 33039_at	TRIM	TRAT1("T cell receptor associated transmembrane adaptor 1")
410 1372_at	TSG-6	TNFAIP6("tumor necrosis factor, alpha-induced protein 6")
411 33565_at	TSH-beta	TSHB("thyroid stimulating hormone, beta")
412 36478_at	TTF1	TTF1("transcription termination factor, RNA polymerase I")
413 33490_at	TXK	TXK("TXK tyrosine kinase")
414 35497_at	Tetraspanin-2	TSPAN2("tetraspanin 2")
415 658_at	Thrombospondin 2	THBS2("thrombospondin 2")
416 39395_at	Thy-1	THY1("Thy-1 cell surface antigen")
417 38370_at	Tiam 1	TIAM1("T-cell lymphoma invasion and metastasis 1")
418 39639_s_at	Tnp1	TNP1("transition protein 1 (during histone to protamine
419 33478_at	Tnp2	TNP2("transition protein 2 (during histone to protamine
420 37247_at	Transcription factor 21	TCF21("transcription factor 21")
421 36931_at	Transgelin	TAGLN("transgelin")
422 33182_at,	TrkB	NTRK2("neurotrophic tyrosine kinase, receptor, type 2")
423 41748_at	Troponin C, skeletal muscle	TNNC2("troponin C type 2 (fast)")
424 38486_at	Troponin I, slow skeletal	TNNI1("troponin I type 1 (skeletal, slow)")
425 38793_at	Troponin T, cardiac	TNNT2("troponin T type 2 (cardiac)")
426 1063_s_at	Tyro3	TYRO3("TYRO3 protein tyrosine kinase")
427 33544_at	UNC5C	UNC5C("unc-5 homolog C (C. elegans)")
428 41859_at	Uronyl-2-sulfotransferase	UST("uronyl-2-sulfotransferase")
429 41433_at,	VCAM1	VCAM1("vascular cell adhesion molecule 1")
430 1388_g_at	VDR	VDR("vitamin D (1,25- dihydroxyvitamin D3) receptor")
431 37196_at	VE-cadherin	CDH5("cadherin 5, type 2, VE-cadherin (vascular epithelium)")
432 33567_at	VIP	VIP("vasoactive intestinal peptide")
433 35415_at	Villin 1	VIL1("villin 1")
434 35178_at	WIF1	WIF1("WNT inhibitory factor 1")
435 32359_at	WISP1	WISP1("WNT1 inducible signaling pathway protein 1")
436 1669_at,	WNT *	WNT2B("wingless-type MMTV integration site family, member 2B"),
437 1669_at,	WNT5A	WNT5A("wingless-type MMTV integration site family, member 5A")
438 40929_at	WSB1	WSB1("WD repeat and SOCS box-containing 1")
439 40902_at	WWP1	WWP1("WW domain containing E3 ubiquitin protein ligase 1")
440 35647_at	XPC	XPC("xeroderma pigmentosum, complementation group C")
441 32588_s_at	ZFP36L2	ZFP36L2("zinc finger protein 36, C3H type-like 2")
442 41503_at	ZHX2	ZHX2("zinc fingers and homeoboxes 2")
443 33658_at	ZK7	ZNF124("zinc finger protein 124 (HZF-16)")
444 38302_at	ZNF202	ZNF202("zinc finger protein 202")
445 36244_at	ZNF239	ZNF239("zinc finger protein 239")
446 35959_at	ZNF365	ZNF365("zinc finger protein 365")
447 40155_at	abLIM	ABLIM1("actin binding LIM protein 1")
448 1916_s_at,	c-Fos	FOS("v-fos FBJ murine osteosarcoma viral oncogene homolog")
449 1888_s_at	c-Kit	KIT("v-kit Hardy-Zuckerman 4 feline sarcoma viral oncogene
450 41504_s_at,	c-Maf	MAF("v-maf musculoaponeurotic fibrosarcoma oncogene homolog
451 2042_s_at	c-Myb	MYB("v-myb myeloblastosis viral oncogene homolog (avian)")
452 41572_r_at	c-Rel	REL("v-rel reticuloendotheliosis viral oncogene homolog (avian)")
453 35237_at	collagen XXI, alpha 1	COL21A1("collagen, type XXI, alpha 1")
454 35174_i_at	eEF1A2	EEF1A2("eukaryotic translation elongation factor 1 alpha 2")
455 32927_at	hIAN5	GIMAP5("GTPase, IMAP family member 5")
456 31703_at,	nAChR alpha *	CHRNA6("cholinergic receptor, nicotinic, alpha 6"),
457 31703_at	nAChR alpha-6	CHRNA6("cholinergic receptor, nicotinic, alpha 6")
458 39566_at	nAChR alpha-7	CHRNA7("cholinergic receptor, nicotinic, alpha 7")
459 33246_at	p38delta (MAPK13)	MAPK13("mitogen-activated protein kinase 13")
460 35692_at	p40BBP	RIS1("BINP receptor")
461 1787_at,	p57	CDKN1C("cyclin-dependent kinase inhibitor 1C (p57, Kip2)")

Group 1, down-regulated Affymetrix gene IDs, fold change >2.5				
**#**	**Affy tag**	**Gene Symbol**	**mean (ratios)**	**p.value**
1	35871_s_at	SLC4A4	-2.550821	0.0387
2	35324_at		-7.463628	0.037500019
3	31766_s_at	DOCK10	-2.519939	0.0346
4	1404_r_at	CCL5	-4.679075	0.0331
5	40691_at	ZNF274	-2.840142	0.0308
6	36370_at	FOXE1	-7.075547	0.0288
7	35041_at	NTF3	-2.564602	0.0242
8	36157_at	PDGFRA	-2.994677	0.022500019
9	39680_at	STATH	-2.74606	0.019
10	31656_at	SLC1A3	-3.580635	0.0128
11	39575_at	AJAP1	-3.374815	0.0124
12	31843_at	ESRRG	-4.690833	0.0123
13	31666_f_at		-2.700661	0.00813
14	41294_at	KRT7	-2.577033	0.00659
15	37279_at	GEM	-2.74006	0.00457
16	931_at	EBI2	-3.179655	0.00309
17	34050_at	ACSM1	-2.589047	0.00252
18	34870_at	LDB3	-2.761849	0.00186
19	34203_at	CNN1	-3.267157	0.000978
20	1098_at	THPO	-3.224482	0.000921
21	1732_at	FGF5	-3.395393	0.000684
22	34972_s_at	DNAH17	-2.578695	0.000595
23	34905_at	GRIK5	-2.67196	0.000561
24	1866_g_at	SKIL	-3.064714	0.000492
25	36650_at	CCND2	-5.79789	0.000467
26	38562_g_at	UTP20	-3.221291	0.000453
27	901_g_at	PLCB4	-3.495103	0.000352
28	36966_at		-5.096594	0.000342
29	34040_s_at	NCR1	-3.050563	0.000307
30	1806_at	MCF2	-5.165472	0.000274
31	40899_at	KRT19	-7.196809	0.000238
32	679_at	CTSG	-2.654728	0.000232
33	1679_at	PMS2L1	-3.110928	0.000217
34	1826_at	RHOB	-2.528657	0.000157
35	32711_g_at	KCNAB1	-4.850651	0.000141
36	1128_s_at	CCR1	-2.806209	0.000117
37	36410_f_at	ELAVL2	-3.614348	0.000104
38	31682_s_at	CSPG2	-2.577805	0.0000796
39	34650_at		-3.094568	0.0000367
40	35616_at	CIITA	-4.066212	0.0000359
41	31714_at	MSH4	-3.001719	0.0000352
42	31848_at	CADPS	-3.589963	0.0000301
43	34517_at	HMGCS1	-4.182154	0.0000293
44	31314_at	BMP3	-2.588511	0.0000242
45	36246_at	ADCY8	-2.977488	0.0000211
46	194_at	CATR1	-4.246685	0.000019
47	31318_at	KITLG	-2.661889	0.0000137
48	1279_s_at		-2.871111	0.0000116
49	343_s_at	ENPP1	-2.646671	0.00000619

50	2061_at	ITGA4	-7.259555	0.00000474
51	31636_s_at	SLC18A3	-3.265427	0.00000395
52	32328_at	KRTHB5	-4.700889	0.00000316
53	40168_at	CAV2	-2.993574	0.00000306
54	1687_s_at	BAK1	-2.603574	0.00000135
55	34479_at	PIK3R3	-3.295187	0.00000087
56	35889_at	SEMA3D	-5.614486	0.000000808
57	1286_s_at		-3.191832	0.000000485
58	36877_at	HLA-DQB1	-3.746709	0.000000279
59	902_at	EPHB2	-4.69959	9.82E-08
60	34718_at	IGF1R	-2.583093	0.000000084
61	37185_at	SERPINB2	-3.676207	0.000000027
62	41299_f_at	GTL3	-3.416095	6.48E-09
63	36258_at	PRKG1	-6.034621	4.68E-09
64	40525_at	DOT1L	-2.8044	1.56E-09
65	41654_at	ADA	-2.870186	1.1E-09
66	40062_s_at	MYL4	-3.011272	1.08E-09
67	39206_s_at	AGC1	-14.766243	9.59E-10
68	1808_s_at	FAS	-2.831392	7.77E-10
69	40560_at	TBX2	-2.755117	5.28E-10
70	1490_at	MYCL1	-2.921326	4.05E-10
71	35558_at	UGP2	-4.530958	3.39E-10
72	36425_at	NEBL	-3.111	3.36E-10
73	38112_g_at	CSPG2	-2.50761	3E-10
74	35832_at	SULF1	-3.776331	2.89E-10
75	36331_at	TMEM1	-2.953589	2.62E-10
76	38267_at	SLC1A1	-2.775045	1.93E-10
77	35433_s_at	APC	-3.316347	1.2E-10
78	1099_s_at	GSTT2	-4.068647	7.13E-11
79	40488_at	DMD	-4.207266	3.26E-11
80	914_g_at	ERG	-10.354282	1.73E-11
81	1596_g_at	TEK	-3.061822	1.27E-11
82	483_g_at	CDH13	-3.863581	7.87E-12
83	32847_at	MYLK	-4.314992	2.87E-12
84	33232_at	CRIP1	-10.256992	2.74E-12
85	907_at	ADA	-2.679045	1.33E-12
86	1776_at	RRAD	-6.624605	1.1E-12
87	39528_at	RRAD	-4.885399	8.6E-13
88	159_at	VEGFC	-7.157807	5.21E-13
89	31733_at	P2RX3	-3.81057	4.52E-13
90	110_at	CSPG4	-4.077744	3.77E-13
91	33206_at	LOC92154	-3.125072	2.48E-13
92	35083_at	FTL	-5.153117	1.04E-13
93	1934_s_at	VEGFC	-3.012659	9.82E-14
94	40895_g_at	SIKE	-2.789652	5.44E-14
95	32242_at	CRYAB	-2.789202	1.53E-14
96	758_at	PTGIR	-3.34503	2.29E-15
97	1545_g_at	FLT1	-6.353895	1.05E-15
98	32919_at	AMIGO2	-3.194699	6.46E-16
99	38004_at	CSPG4	-7.672025	5.13E-16
100	37562_at	PCDH1	-2.854763	7.29E-17
101	41401_at	CSRP2	-3.919765	5.4E-17

102	2073_s_at	CDH13	-5.406174	5.09E-17
103	36009_at	GPX7	-2.714063	4.84E-17
104	35172_at	TPST2	-3.461705	1.04E-17
105	34235_at	GPR116	-7.277966	6.85E-18
106	33410_at	ITGA6	-5.393243	5.36E-18
107	39901_at	EDIL3	-3.913362	2.79E-18
108	36156_at	AQP1	-3.701093	1.48E-18
109	39207_r_at	AGC1	-26.53751953	6.6E-19
110	39690_at	PDLIM3	-4.291311	5.94E-19
111	342_at	ENPP1	-2.532095	3.21E-19
112	1597_at	GAS6	-4.330121	1.2E-19
113	38965_at	AGC1	-39.839144	1.04E-19
114	33143_s_at	SLC16A3	-5.871107	9.95E-20
115	35712_at	LRRN3	-12.481593	7.47E-20
116	885_g_at	ITGA3	-2.901416	4.29E-20
117	41355_at	BCL11A	-3.411065	8.75E-21
118	37765_at	LMOD1	-2.548414	1.06E-21
119	39069_at	AEBP1	-2.550469	9.57E-22
120	33411_g_at	ITGA6	-9.245704	2.49E-22
121	33359_at	LPHN3	-4.072584	1.92E-22
122	236_at	GNAO1	-3.705442	6.68E-23
123	affx-humisgf3a/m97	STAT1	-2.655889	2.23E-23
124	40350_at	ADAM23	-6.92309	1.1E-23
125	38289_r_at	NF1, NF1P1	-5.403063	1.36E-24
126	707_s_at		-2.557349	1.55E-25
127	35679_s_at	DPP6	-4.352312	9.18E-26
128	31311_at		-3.289652	1.83E-26
129	32582_at	MYH11	-27.207585	7.39E-27
130	767_at	MYH11	-20.100645	6.15E-27
131	34476_r_at	EREG	-14.501402	1.99E-27
132	41714_at	LPPR4	-7.169023	6.75E-28
133	32859_at	STAT1	-2.664465	4.11E-28
134	38749_at	LYPD1	-15.043538	2.84E-31
135	31856_at	LRRC32	-2.803238	9.39E-33
136	35731_at	ITGA4	-13.360724	2.19E-35
137	41266_at	ITGA6	-4.336407	1.99E-35
138	906_at	STAT4	-3.735167	1.12E-37
139	35285_at	SLC4A4	-2.805692	1.77E-39
140	39407_at	BMP1	-3.404395	8.01E-40
141	40075_at		-4.274546	1.41E-41
142	37459_at	COL8A1	-3.247955	6.94E-43
143	884_at	ITGA3	-2.623977	1.38E-44
144	39710_at	C5orf13, LOC389473	-4.915979	2.23E-46
145	affx-humisgf3a/m97	STAT1	-2.877862	4.38E-58
146	37407_s_at	MYH11	-36.442654	6.1E-61
147	34390_at	P4HA2	-2.906899	2.56E-62
148	774_g_at	MYH11	-23.365227	4.52E-64
149	672_at	SERPINE1	-2.753658	1.39E-66
150	35703_at	PDGFA	-4.147916	4.3E-76
151	36043_at	OPCML	-7.50447	1.16E-91
152	33338_at	STAT1	-2.960602	2.51E-138

Group 2, down-regulated Affymetrix gene IDs, fold change >2.5				
#	Affy tag	Gene Symbol	Mean (ratios)	P Value
1	37185_at	SERPINB2	-13.2160512	0.01205
2	1364_at	PTPRZ1	-11.8204226	2.2E-09
3	40995_at	NEFL	-10.2594587	0.00068
4	34190_at	COCH	-8.80295955	6.7E-07
5	38428_at	MMP1	-8.45140249	0.01608
6	37251_s_at	GPM6B	-8.41264175	0.00066
7	36400_at	PRIM1	-7.07011394	4.1E-35
8	34235_at	GPR116	-6.98150999	0.04046
9	34476_r_at	EREG	-6.9570901	0.0007
10	32582_at	MYH11	-6.9492357	0.01488
11	35989_at	MYOZ2	-6.7980453	7.6E-06
12	35406_at	KALRN	-6.51884157	1.5E-07
13	37407_s_at	MYH11	-6.51307287	0.02903
14	37483_at	HDAC9	-6.24522542	3.1E-05
15	32299_at	C4orf6	-6.12121531	1.1E-09
16	32050_r_at		-5.97449332	1.4E-14
17	39898_at	FAM13C1	-5.7395479	1.3E-21
18	41148_at	CGNL1, HTN3	-5.67209742	5.6E-09
19	35031_r_at	FRMPD4	-5.66697523	9.1E-06
20	39384_at	ELAVL1	-5.626709	4.6E-12
21	37290_at	CDH8	-5.49335048	1.9E-06
22	41608_at	CRHBP	-5.41740419	0.0339
23	35885_at	USP9Y	-5.32408827	1.4E-08
24	31763_at	OR7A5	-5.30796992	2.4E-11
25	32948_at	USH2A	-5.29696187	2.7E-12
26	31327_at		-5.14480247	4.3E-13
27	34012_at	KRTHA4	-5.12894587	0.00431
28	33051_at	GABRR1	-4.9973333	5.9E-20
29	36254_at	TAC1	-4.96390166	1.6E-17
30	208_at	CTNNA2	-4.9054746	1.7E-14
31	774_g_at	MYH11	-4.87223877	0.02556
32	33462_at	P2RY14	-4.84346469	2.7E-09
33	1203_at	INHBA	-4.81213704	6.8E-11
34	34441_at	NNT	-4.78672149	2.4E-14
35	32090_at	NMNAT2	-4.78002861	6E-05
36	34665_g_at	FCGR2A, FCGR2B, FCGR2C	-4.74006072	1.3E-05
37	37530_s_at	RELN	-4.71804475	0.00106
38	375_at	GSTT1	-4.61210276	0.00072
39	37583_at	SMCY	-4.59411153	2.1E-06
40	35857_at	GRIK1	-4.48419172	5.7E-13
41	31732_at	RLN2	-4.47774734	1.5E-10
42	40834_at	PDZD2	-4.47075592	0.0003
43	1473_s_at	MYB	-4.43602472	0.00122
44	34551_at	POU4F2	-4.41902244	5.8E-06
45	40158_r_at	MYL1	-4.40376293	3.4E-20
46	39321_at		-4.40112454	6.8E-19
47	33483_at	NMU	-4.37301685	0.00024
48	33669_at		-4.35301148	2.8E-08
49	41157_at	LOC131185, RAD23B	-4.32174789	0.00032

50	37479_at	CD72	-4.31741648	4.2E-13
51	395_at		-4.27416082	1.1E-06
52	40702_at	IFNG	-4.26463688	8.4E-14
53	931_at	EBI2	-4.24244671	0.00321
54	40687_at	GJA4	-4.17622235	5.6E-05
55	35929_s_at	TSPY1, TSPY2	-4.16348547	4.7E-11
56	1174_at		-4.12001011	0.00022
57	36877_at	HLA-DQB1	-4.11369651	3.3E-08
58	34964_at	HIST1H3D	-4.10692229	0.00173
59	34911_r_at	PHTF2	-4.10568276	1.9E-05
60	40024_at	STAC	-3.99122372	5.1E-14
61	38582_at	SPINK1	-3.97052803	1.6E-12
62	35158_at	MYCN	-3.95081688	4.4E-13
63	35877_at	SOX21	-3.91452145	4.1E-10
64	33970_at	MAGEB1	-3.89411671	0.00192
65	37363_at	MTSS1	-3.86004118	0.01623
66	773_at	MYH11	-3.84426714	0.00052
67	1069_at	PTGS2	-3.82836278	0.00678
68	35174_i_at	EEF1A2	-3.81704419	9.9E-14
69	959_at		-3.77867862	2.3E-32
70	36854_s_at	GRIA1	-3.77265811	2.6E-06
71	32291_at	UNC13A	-3.76595402	1.4E-05
72	38920_at	CHEK1	-3.75937554	1E-05
73	36370_at	FOXE1	-3.74207874	0.02091
74	36239_at	POU2AF1	-3.739764	0.00071
75	35731_at	ITGA4	-3.73107007	0.00159
76	35064_at	TRIM31	-3.7299881	0.00086
77	33596_at	SPOCK3	-3.70205421	2E-10
78	35491_at	MAGEA10	-3.68716303	3.3E-05
79	31885_at	PTPN3	-3.68555729	2.2E-05
80	35405_at	TAT	-3.66361947	2.6E-06
81	977_s_at	CDH1	-3.66301562	0.00365
82	200_at	BMP5	-3.66248569	8.2E-32
83	32759_at		-3.65137127	1.8E-07
84	34650_at		-3.6376211	0.01686
85	32821_at	LCN2	-3.6313823	8.8E-08
86	39453_at		-3.58705874	9.1E-19
87	32027_at	PDZK1	-3.56826233	4.5E-05
88	37222_at	GSTT1	-3.56788486	0.00076
89	269_at	OASL	-3.56035733	1.3E-45
90	38259_at	STXBP2	-3.54671449	0.00015
91	33728_at	BSN	-3.53676541	2E-06
92	670_s_at	CREB5	-3.53233237	1.2E-12
93	32703_at	PLK4	-3.531899	2.1E-05
94	750_at	TRHR	-3.52714627	2.4E-05
95	1561_at	DUSP8	-3.48699845	4.5E-08
96	33598_r_at	CIAS1	-3.4757166	2.3E-07
97	39214_at	PLXNB3, STK23	-3.4447623	1.5E-15
98	31565_at		-3.44380574	3.8E-16
99	35049_g_at	GRIA3	-3.43383358	1.4E-07
100	32477_at	CAPN9	-3.41033079	1.1E-05
101	40942_g_at	VAPB	-3.40723031	9.9E-06

102	35674_at	PADI2	-3.39795372	0.02168
103	32763_r_at	IARS2	-3.37104431	0.00059
104	36326_at	NHLH2	-3.36297224	2.4E-07
105	38933_at	KIFC1	-3.3618638	1.7E-20
106	36567_at	SLC17A7	-3.35907616	3E-09
107	1021_at	IFNG	-3.35802791	1.6E-09
108	32331_at	AK3L1	-3.34683401	7.6E-17
109	117_at	HSPA6	-3.33324304	4.7E-22
110	36816_s_at	CFTR	-3.32068762	4.1E-06
111	37233_at	OLR1	-3.31982989	0.00016
112	32266_at	CDH12	-3.3193992	6.1E-11
113	38848_at	ZG16	-3.30781862	1.7E-29
114	32932_at	RP11-142I17.1	-3.28696842	0.00748
115	31747_g_at	LOC441135	-3.28584697	9.6E-12
116	419_at	MKI67	-3.28333229	0.00029
117	34014_f_at	POU1F1	-3.27450675	0.0022
118	38355_at	DDX3Y	-3.27288824	0.03645
119	38583_at	B3GALT2	-3.26789092	0.0016
120	33529_at	ADH7	-3.25261966	1.8E-16
121	38140_at	EXTL1	-3.25117975	3.1E-05
122	32301_at	GUCY1A2	-3.24071479	8.2E-14
123	31394_at	SERPINI2	-3.22951224	1.9E-10
124	32537_at	ACSBG1	-3.22186848	0.00019
125	35051_at	CA6	-3.2195162	6.4E-11
126	38557_at	NCAM2	-3.21951549	0.00055
127	33991_g_at	ALB	-3.20794509	5.5E-11
128	35863_g_at	SLC15A2	-3.20350975	7.2E-13
129	32404_at	CNGA3	-3.1999388	0.00027
130	34559_at	KRTAP26-1	-3.19490878	4.1E-05
131	502_s_at	HOXA1	-3.18857013	0.00253
132	33766_at	VIPR1	-3.18563823	3.2E-17
133	1918_at	SKI	-3.18302478	0.00184
134	33157_at	INSM1	-3.18168707	9.7E-13
135	39584_at	CAMTA1	-3.15488277	9.4E-07
136	37851_at	HIPK2	-3.14604831	9.6E-17
137	266_s_at	CD24	-3.14043627	2.2E-05
138	39407_at	BMP1	-3.12477332	0.00011
139	1282_s_at		-3.11929968	5E-11
140	31450_s_at	RIT2	-3.11637151	0.00053
141	31316_at		-3.1016272	0.00021
142	346_s_at	AGTR1	-3.0907466	0.00012
143	37558_at	IGF2BP3	-3.08715678	0.00053
144	1610_s_at	DHFR	-3.08537238	3.7E-09
145	38875_r_at	GREB1	-3.084368	0.00112
146	40193_at	ENO2	-3.07624713	5.9E-93
147	39457_r_at		-3.07380253	0.00054
148	40653_at	RGS7	-3.06290136	1.1E-23
149	37841_at	BCHE	-3.0625211	0.00701
150	35565_at	LOC401131	-3.05705593	1.6E-05
151	2026_at	RUNX1	-3.05560595	6.2E-06
152	39491_s_at		-3.05371757	1.9E-13
153	40573_s_at	MAP3K9	-3.031511	0.00394

154	34578_at	SGCG	-3.02761991	0.00711
155	1197_at	ACTG2	-3.02376052	0.01417
156	41355_at	BCL11A	-3.02136465	0.00139
157	569_g_at	PRKACA	-3.01828223	5.9E-39
158	33092_at	FPRL2	-3.01755637	0.00015
159	40378_at	SH3GL2	-3.00813044	5.5E-05
160	37149_s_at	LTF	-3.00544443	1.5E-05
161	1349_at	DCC	-3.00307056	2.2E-06
162	36393_at	DPF1	-2.9992495	0.00058
163	33990_at	ALB	-2.98645729	0.00015
164	37279_at	GEM	-2.98610084	2.4E-11
165	36627_at	SPARCL1	-2.95892375	8.6E-06
166	39255_at	PROC	-2.95759279	1.2E-15
167	33502_at		-2.9357824	0.00022
168	41290_at	NCAM1	-2.93464599	6.8E-43
169	474_at	DGKE	-2.92064861	0.00042
170	34973_at	ATP8A1	-2.91990647	1.8E-06
171	32368_at	PCDH8	-2.90793986	0.01448
172	33442_at	KIAA0367	-2.90706883	9.2E-06
173	37702_at	CUL3	-2.90380724	4.5E-06
174	38167_at	PPFIA4	-2.90333688	2.5E-06
175	33803_at	THBD	-2.89207447	2.6E-09
176	37844_at	IL27RA	-2.88088932	0.01276
177	35912_at	MUC4	-2.87973761	5.5E-05
178	1553_r_at	CYP2A13	-2.87583252	0.00203
179	36878_f_at	HLA-DQB1	-2.8709859	7.5E-08
180	32709_at	KCNAB1	-2.86936857	4E-08
181	31621_s_at	ELN	-2.86553853	6.9E-05
182	36716_at	ADRA1A	-2.85515569	3.3E-06
183	1934_s_at	VEGFC	-2.84387608	0.03021
184	40157_s_at	MYL1	-2.84116458	5.1E-06
185	34898_at	AREG	-2.83396989	0.0018
186	32128_at	CCL18	-2.83361374	5.4E-06
187	34616_at	F13B	-2.82102957	0.02307
188	36329_at	SCGB2A2	-2.82099022	0.00151
189	31909_at	KIAA0754	-2.81816681	9.2E-07
190	34045_at	LOC196993	-2.81524877	3.3E-09
191	37579_at	CYFIP2	-2.81455737	0.00048
192	40135_at		-2.81094616	8.6E-07
193	40320_at	CDC14A	-2.80930525	0.01596
194	34623_at	DEFA5	-2.80817311	1.3E-14
195	38037_at	HBEGF	-2.80564213	0.00389
196	37089_at	ADAM3A	-2.80250625	0.00196
197	1016_s_at	IL13RA2	-2.80142414	0.01093
198	35982_at	HSPB3	-2.79258889	7.3E-05
199	31490_at	SCN5A	-2.78687154	3.5E-05
200	35592_at	SLC8A2	-2.78585638	1.2E-07
201	40899_at	KRT19	-2.77778517	0.01922
202	33531_at	KLRD1	-2.77644172	1.4E-05
203	979_g_at	BCL6	-2.77177049	0.00023
204	33359_at	LPHN3	-2.75753122	0.00915
205	33940_at	HCG4	-2.75185338	0.0089

206	34984_at	TRPC3	-2.75147217	0.00378
207	161_at	RAB9P1	-2.74964553	0.00025
208	37289_at	CDH8	-2.74337982	0.0292
209	37930_at	ATP7B	-2.74063929	0.00013
210	31570_at	SIM1	-2.74013124	2.7E-08
211	35077_at	TACR1	-2.73774126	1.3E-07
212	610_at	ADRB2	-2.73410788	1.8E-06
213	38322_at	PAGE4	-2.73136067	0.00669
214	33979_at	RNASE3	-2.72988129	0.02023
215	34507_s_at	LMTK2	-2.725746	2E-06
216	862_at	SERPINB5	-2.72324742	0.00159
217	32496_at	KCNE1	-2.7178668	0.00764
218	36609_at	SLC1A3	-2.71557283	2.1E-05
219	35041_at	NTF3	-2.71293831	0.03066
220	31754_at	ABCA12	-2.69007803	3.2E-14
221	31698_at	FOXC2	-2.6892335	1.1E-05
222	40680_at	SPIN2	-2.68587927	0.00019
223	35669_at	COBL	-2.68533789	0.00021
224	1478_at	ITK	-2.68461275	0.00208
225	32946_r_at	MBL2	-2.68073867	0.01066
226	40812_at	MRPS6	-2.67287357	0.00098
227	36227_at	IL7R	-2.6720914	7.2E-06
228	39696_at	PEG10	-2.67198175	0.00593
229	728_at		-2.66781379	0.00001
230	37257_at	PRUNE	-2.6668691	5.7E-06
231	36308_at	ZIC1	-2.66201244	0.00037
232	33284_at	MPO	-2.65891225	9.9E-05
233	37317_at	PAFAH1B1	-2.64187802	1.3E-05
234	41055_at	KIAA0363	-2.64057411	0.00043
235	39440_f_at	C10orf95	-2.63949968	0.00418
236	33672_f_at	UGT2B15, UGT2B17	-2.63902356	8.7E-13
237	172_at	INPP5D	-2.63459612	6.1E-15
238	1474_s_at	MYB	-2.62986867	2.3E-12
239	39682_at	SCNN1B	-2.62208443	4.4E-05
240	33977_at	GFI1	-2.62171439	1.3E-07
241	37137_at	GZMB	-2.60905335	0.00343
242	1739_at	FOLH1	-2.60535924	0.00141
243	36374_at		-2.60094439	0.00191
244	34132_at	SV2C	-2.60047979	0.00024
245	41578_at	NHLH2	-2.59872301	0.00791
246	31336_at	FLJ16360	-2.59340406	0.00239
247	863_g_at	SERPINB5	-2.59227028	0.00057
248	1175_s_at		-2.58928335	0.03005
249	38195_at	PHF14	-2.58867067	0.01599
250	34664_at	FCGR2B, FCGR2C	-2.58774741	0.00181
251	36543_at	F3	-2.58525857	1.8E-06
252	34158_s_at	SLC1A3	-2.58267096	3E-07
253	33159_at	NRL	-2.57629592	1.1E-05
254	41323_at		-2.57625875	0.00061
255	33491_at	SI	-2.57570412	0.02948
256	38861_at	PON1	-2.56509048	0.00965
257	37186_s_at	ABP1	-2.56059303	9.4E-06

258	37086_at	LYZL6	-2.55084105	0.01107
259	34897_at	TNK2	-2.53296915	5E-07
260	34590_at	CNTF	-2.53232787	0.00013
261	31714_at	MSH4	-2.53026892	0.00102
262	36824_at	ASTN	-2.52832491	0.04134
263	40978_s_at	CDR1	-2.52578818	0.00069
264	32865_at		-2.52572434	0.00099
265	2019_s_at	ITGB7	-2.52469955	3.2E-05
266	33516_at	HBD	-2.51655931	2.4E-05
267	32917_at	LCT	-2.51367594	6.3E-28
268	37712_g_at	MEF2C	-2.5130584	0.00058
269	36788_at	GPC5	-2.51064808	0.00019
270	41486_at	RASSF8	-2.50939199	0.00058
271	33001_s_at	TGM5	-2.50393515	0.00022

Group 1, down-regulated genes (HUGO nomenclature), fold change > 2.5					
#	Gene Symbc	Protein	Protein name	Signal	P_value
1	ACSM1			-2.589	0.00252
2	ADA	ADA_HUMAN	Adenosine deaminase	-2.8702	1.1E-09
3	ADAM23	ADA23_HUMAN	ADAM 23 precursor	-6.9231	1.1E-23
4	ADCY8	ADCY8_HUMAN	Adenylate cyclase type 8	-2.9775	2.11E-05
5	AEBP1	AEBP1	AE binding protein 1	-2.5505	9.57E-22
6	AGC1	PGCA_HUMAN	Aggrecan core protein precursor	-39.839	1.04E-19
7	AJAP1	AJAP1	adherens junction associated protein 1	-3.3748	0.0124
8	AMIGO2	AMGO2_HUMAN	Amphoterin-induced protein 2 precursor	-3.1947	6.46E-16
9	APC	APC_HUMAN	Adenomatous polyposis coli protein	-3.3163	1.2E-10
10	AQP1	AQP1_HUMAN	Aquaporin-1	-3.7011	1.48E-18
11	BAK1	BAK_HUMAN	Bcl-2 homologous antagonist/killer	-2.6036	1.35E-06
12	BCL11A	BC11A_HUMAN	B-cell lymphoma/leukemia 11A	-3.4111	8.75E-21
13	BMP1	BMP1_HUMAN	Bone morphogenetic protein 1 precursor	-3.4044	8.01E-40
14	BMP3	BMP3_HUMAN	Bone morphogenetic protein 3 precursor	-2.5885	2.42E-05
15	C5orf13	NP311_HUMAN	Neuronal protein 3.1	-4.916	2.23E-46
16	CADPS	CAPS1_HUMAN	Calcium-dependent secretion activator 1	-3.59	3.01E-05
17	CATR1	CATR1_HUMAN	CATR tumorigenic conversion 1 protein	-4.2467	0.000019
18	CAV2	CAV2_HUMAN	Caveolin-2	-2.9936	3.06E-06
19	CCL5	CCL5_HUMAN	Small inducible cytokine A5 precursor	-4.6791	0.0331
20	CCND2	CCND2_HUMAN	G1/S-specific cyclin-D2	-5.7979	0.000467
21	CCR1	CCR1_HUMAN	C-C chemokine receptor type 1	-2.8062	0.000117
22	CDH13	CAD13_HUMAN	Cadherin-13 precursor	-5.4062	5.09E-17
23	CIITA	C2TA_HUMAN	MHC class II transactivator	-4.0662	3.59E-05
24	CNN1	CNN1_HUMAN	Calponin-1	-3.2672	0.000978
25	COL8A1	CO8A1_HUMAN	Collagen alpha-1(VIII	-3.248	6.94E-43
26	CRIP1	CRIP1_HUMAN	Cysteine-rich protein 1	-10.257	2.74E-12
27	CRYAB	CRYAB_HUMAN	Alpha crystallin B chain	-2.7892	1.53E-14
28	CSPG2	CSPG2_HUMAN	Versican core protein precursor	-2.5778	7.96E-05
29	CSPG4	CSPG4_HUMAN	Chondroitin sulfate proteoglycan 4 precursor	-7.672	5.13E-16
30	CSRP2	CSRP2_HUMAN	Cysteine and glycine-rich protein 2	-3.9198	5.4E-17
31	CTSG	CATG_HUMAN	Cathepsin G precursor	-2.6547	0.000232
32	DMD	DMD_HUMAN	Dystrophin	-4.2073	3.26E-11
33	DNAH17	DNAH17	dynein, axonemal, heavy polypeptide 17	-2.5787	0.000595
34	DOCK10	DOC10_HUMAN	Dedicator of cytokinesis protein 10	-2.5199	0.0346
35	DOT1L	DOT1L_HUMAN	Histone-lysine N-methyltransferase, H3 lysine-79 specific(EC 2.1.1.43	-2.8044	1.56E-09
36	DPP6	DPP6_HUMAN	Dipeptidyl aminopeptidase-like protein 6	-4.3523	9.18E-26
37	EBI2	EBI2_HUMAN	EBV-induced G-protein coupled receptor 2	-3.1797	0.00309
38	EDIL3	EDIL3_HUMAN	EGF-like repeat and discoidin I-like domain-containing protein 3precursor	-3.9134	2.79E-18
39	ELAVL2	ELAV2_HUMAN	ELAV-like protein 2	-3.6143	0.000104
40	ENPP1	ENPP1_HUMAN	Ectonucleotide pyrophosphatase/phosphodiesterase 1	-2.6467	6.19E-06
41	EPHB2	EPHB2_HUMAN	Ephrin type-B receptor 2 precursor	-4.6996	9.82E-08
42	EREG	EREG_HUMAN	Epiregulin precursor	-14.501	1.99E-27
43	ERG	ERG_HUMAN	Transcriptional regulator ERG	-10.354	1.73E-11
44	ESRRG	ERR3_HUMAN	Estrogen-related receptor gamma	-4.6908	0.0123
45	FAS	TNR6_HUMAN	Tumor necrosis factor receptor superfamily member 6 precursor	-2.8314	7.77E-10

46	FGF5	FGF5_HUMAN	Fibroblast growth factor 5 precursor	-3.3954	0.000684
47	FLT1	VGFR1_HUMAN	Vascular endothelial growth factor receptor 1 precursor	-6.3539	1.05E-15
48	FOXE1	FOXE1_HUMAN	Forkhead box protein E1	-7.0755	0.0288
		FOXE2_HUMAN	Forkhead box protein E2	-7.0755	0.0288
49	FTL	FRIL_HUMAN	Ferritin light chain	-5.1531	1.04E-13
50	GAS6	GAS6_HUMAN	Growth-arrest-specific protein 6 precursor	-4.3301	1.2E-19
51	GEM	GEM_HUMAN	GTP-binding protein GEM	-2.7401	0.00457
52	GNAO1	GNAO1_HUMAN	Guanine nucleotide-binding protein G(o	-3.7054	6.68E-23
		GNAO2_HUMAN	Guanine nucleotide-binding protein G(o	-3.7054	6.68E-23
53	GPR116	GP116_HUMAN	Probable G-protein coupled receptor 116 precursor	-7.278	6.85E-18
54	GPX7	YZX3_HUMAN	6.2 kDa protein from clone 683	-2.7141	4.84E-17
		GPX7_HUMAN	Glutathione peroxidase 7 precursor	-2.7141	4.84E-17
55	GRIK5	GRIK5_HUMAN	Glutamate receptor, ionotropic kainate 5 precursor	-2.672	0.000561
56	GSTT2	GSTT2_HUMAN	Glutathione S-transferase theta-2	-4.0686	7.13E-11
57	GTL3	GTL3	transcription factor IIB	-3.4161	6.48E-09
58	HLA-DQB1	HB24_HUMAN	HLA class II histocompatibility antigen, DQ(3	-3.7467	2.79E-07
		HB23_HUMAN	HLA class II histocompatibility antigen, DQ(W3	-3.7467	2.79E-07
		HB25_HUMAN	HLA class II histocompatibility antigen, DQB1*0602 beta chainprecursor	-3.7467	2.79E-07
		HB22_HUMAN	HLA class II histocompatibility antigen, DQ(W1.1	-3.7467	2.79E-07
		HB21_HUMAN	HLA class II histocompatibility antigen, DQ(1	-3.7467	2.79E-07
59	HMGCS1	HMCS1_HUMAN	Hydroxymethylglutaryl-CoA synthase, cytoplasmic	-4.1822	2.93E-05
60	IGF1R	IGF1R_HUMAN	Insulin-like growth factor 1 receptor precursor	-2.5831	8.4E-08
61	ITGA3	ITA3_HUMAN	Integrin alpha-3 precursor	-2.9014	4.29E-20
62	ITGA4	ITA4_HUMAN	Integrin alpha-4 precursor	-13.361	2.19E-35
63	ITGA6	ITA6_HUMAN	Integrin alpha-6 precursor	-9.2457	2.49E-22
64	KCNAB1	KCAB1_HUMAN	Voltage-gated potassium channel subunit beta-1	-4.8507	0.000141
65	KITLG	SCF_HUMAN	Kit ligand precursor	-2.6619	1.37E-05
66	KRT19	K1C19_HUMAN	Keratin, type I cytoskeletal 19	-7.1968	0.000238
67	KRT7	K2C7_HUMAN	Keratin, type II cytoskeletal 7	-2.577	0.00659
68	KRTHB5	KRHB5_HUMAN	Keratin, type II cuticular Hb5	-4.7009	3.16E-06
69	LDB3	LDB3_HUMAN	LIM domain-binding protein 3	-2.7618	0.00186
70	LMOD1	LMD1_HUMAN	Leiomodin-1	-2.5484	1.06E-21
71	LOC389473			-4.916	2.23E-46
72	LOC646793			-5.4031	1.36E-24
73	LOC649366			-39.839	1.04E-19
74	LOC652330			-5.4031	1.36E-24
75	LOC653689			-4.0686	7.13E-11
76	LOC92154			-3.1251	2.48E-13

77	LPHN3	LPHN3_HUMAN	Latrophilin-3 precursor	-4.0726	1.92E-22
78	LPPR4			-7.169	6.75E-28
79	LRRC32	LRC32_HUMAN	Leucine-rich repeat-containing protein 32 precursor	-2.8032	9.39E-33
80	LRRN3	LRRN3_HUMAN	Leucine-rich repeats neuronal protein 3 precursor	-12.482	7.47E-20
81	LYPD1	LYPD1_HUMAN	Ly6/PLAUR domain-containing protein 1 precursor	-15.044	2.84E-31
82	MCF2	MCF2_HUMAN	Proto-oncogene DBL	-5.1655	0.000274
83	MSH4	MSH4_HUMAN	MutS protein homolog 4	-3.0017	3.52E-05
84	MYCL1	MYCL1_HUMAN	L-myc-1 proto-oncogene protein	-2.9213	4.05E-10
85	MYH11	MYH11_HUMAN	Myosin-11	-36.443	6.1E-61
86	MYL4	MYL4_HUMAN	Myosin light polypeptide 4	-3.0113	1.08E-09
87	MYLK	MYLK_HUMAN	Myosin light chain kinase, smooth muscle	-4.315	2.87E-12
88	NCR1	NCTR1_HUMAN	Natural cytotoxicity triggering receptor 1 precursor	-3.0506	0.000307
89	NEBL	NEBL_HUMAN	Nebulette	-3.111	3.36E-10
90	NF1	NF1_HUMAN	Neurofibromin	-5.4031	1.36E-24
91	NF1P1			-5.4031	1.36E-24
92	NTF3	NT3_HUMAN	Neurotrophin-3 precursor	-2.5646	0.0242
93	OPCML	OPCM_HUMAN	Opioid-binding protein/cell adhesion molecule precursor	-7.5045	1.16E-91
94	P2RX3	P2RX3_HUMAN	P2X purinoceptor 3	-3.8106	4.52E-13
95	P4HA2	P4HA2_HUMAN	Prolyl 4-hydroxylase alpha-2 subunit precursor	-2.9069	2.56E-62
96	PCDH1	PCDH1_HUMAN	Protocadherin-1 precursor	-2.8548	7.29E-17
97	PDGFA	PDGFA_HUMAN	Platelet-derived growth factor A chain precursor	-4.1479	4.3E-76
98	PDGFRA	PGFRA_HUMAN	Alpha platelet-derived growth factor receptor precursor	-2.9947	0.0225
99	PDLIM3	PDLI3_HUMAN	PDZ and LIM domain protein 3	-4.2913	5.94E-19
100	PIK3R3	P55G_HUMAN	Phosphatidylinositol 3-kinase regulatory subunit gamma	-3.2952	8.7E-07
101	PLCB4	PLCB4_HUMAN	1-phosphatidylinositol-4,5-bisphosphate phosphodiesterase beta 4(EC 3.1.4.11	-3.4951	0.000352
102	PMS2L1	PMS2L1	postmeiotic segregation increased 2-like 1	-3.1109	0.000217
103	PRKG1	KGP1A_HUMAN	cGMP-dependent protein kinase 1, alpha isozyme	-6.0346	4.68E-09
		KGP1B_HUMAN	cGMP-dependent protein kinase 1, beta isozyme	-6.0346	4.68E-09
104	PTGIR	PI2R_HUMAN	Prostacyclin receptor	-3.345	2.29E-15
105	RASSF2	RASF2_HUMAN	Ras association domain-containing protein 2	-2.7007	0.00813
106	RHOB	RHOB_HUMAN	Rho-related GTP-binding protein RhoB precursor	-2.5287	0.000157
107	RRAD	RAD_HUMAN	GTP-binding protein RAD	-6.6246	1.1E-12
108	SEMA3D	SEM3D_HUMAN	Semaphorin-3D precursor	-5.6145	8.08E-07
109	SERPINB2	PAI2_HUMAN	Plasminogen activator inhibitor 2 precursor	-3.6762	2.7E-08
110	SERPINE1	PAI1_HUMAN	Plasminogen activator inhibitor 1 precursor	-2.7537	1.39E-66
111	SIKE	FLJ21168	hypothetical protein LOC80143	-2.7897	5.44E-14
112	SKIL	SKIL_HUMAN	Ski-like protein	-3.0647	0.000492
113	SLC16A3	MOT4_HUMAN	Monocarboxylate transporter 4	-5.8711	9.95E-20

114	SLC18A3	VACHT_HUMAN	Vesicular acetylcholine transporter	-3.2654	3.95E-06
115	SLC1A1	EAA3_HUMAN	Excitatory amino acid transporter 3	-2.775	1.93E-10
116	SLC1A3	EAA1_HUMAN	Excitatory amino acid transporter 1	-3.5806	0.0128
117	SLC4A4	S4A4_HUMAN	Electrogenic sodium bicarbonate cotransporter 1	-2.8057	1.77E-39
118	STAT1	STAT1_HUMAN	Signal transducer and activator of transcription 1-alpha/beta(Transcription factor ISGF-3 components p91/p84	-2.9606	0
119	STAT4	STAT4_HUMAN	Signal transducer and activator of transcription 4	-3.7352	1.12E-37
120	STATH	STAT_HUMAN	Statherin precursor	-2.7461	0.019
121	SULF1	SULF1_HUMAN	Extracellular sulfatase Sulf-1 precursor	-3.7763	2.89E-10
122	TBX2	TBX2_HUMAN	T-box transcription factor TBX2	-2.7551	5.28E-10
123	TEK	TIE2_HUMAN	Angiopoietin-1 receptor precursor	-3.0618	1.27E-11
124	THPO	TPO_HUMAN	Thrombopoietin precursor	-3.2245	0.000921
125	TMEM1	TMEM1_HUMAN	Transmembrane protein 1	-2.9536	2.62E-10
126	TPST2	TPS2_HUMAN	Protein-tyrosine sulfotransferase 2	-3.4617	1.04E-17
127	UGP2	UGPA2_HUMAN	UTP--glucose-1-phosphate uridylyltransferase 2	-4.531	3.39E-10
128	UTP20	UTP20_HUMAN	Small subunit processome component 20 homolog	-3.2213	0.000453
129	VEGFC	VEGFC_HUMAN	Vascular endothelial growth factor C precursor	-7.1578	5.21E-13
130	ZNF274	ZN274_HUMAN	Zinc finger protein 274	-2.8401	0.0308

Group 2, down-regulated genes (HUGO nomenclature), fold change> 2.5				
#	Gene Syml	Protein	Protein name	Signal
1	ABCA12	ABCAC_HUMAN	ATP-binding cassette sub-family A member 12	-2.690078
2	ABP1	ABP1_HUMAN	Amiloride-sensitive amine oxidase [copper-containing]	-2.560593
3	ACSBG1	ACSBG1	acyl-CoA synthetase bubblegum family member 1	-3.221868
4	ACTG2	ACTH_HUMAN	Actin, gamma-enteric smooth muscle	-3.023761
5	ADAM3A	ADAM3A	ADAM metallopeptidase domain 3a (cyritestin 1)	-2.802506
6	ADH7	ADH7_HUMAN	Alcohol dehydrogenase class 4 mu/sigma chain	-3.25262
7	ADRA1A	ADA1A_HUMAN	Alpha-1A adrenergic receptor	-2.855156
8	ADRB2	ADRB2_HUMAN	Beta-2 adrenergic receptor	-2.734108
9	AGTR1	AG2S_HUMAN	Type-1B angiotensin II receptor	-3.090747
10	AK3L1	KAD4_HUMAN	Adenylate kinase isoenzyme 4, mitochondrial	-3.346834
11	AK3L2			-3.346834
12	ALB	ALBU_HUMAN	Serum albumin precursor	-3.207945
13	AREG	AREG_HUMAN	Amphiregulin precursor	-2.83397
14	ASTN	ASTN_HUMAN	Astrotactin-1 precursor	-2.528325
15	ATP7B	ATP7B_HUMAN	Copper-transporting ATPase 2	-2.740639
16	ATP8A1	AT8A1_HUMAN	Probable phospholipid-transporting ATPase IA	-2.919906
17	B3GALT2	B3GT2_HUMAN	Beta-1,3-galactosyltransferase 2	-3.267891
18	BCHE	CHLE_HUMAN	Cholinesterase precursor	-3.062521
19	BCL11A	BC11A_HUMAN	B-cell lymphoma/leukemia 11A	-3.021365
20	BCL6	BCL6_HUMAN	B-cell lymphoma 6 protein	-2.77177
21	BMP1	BMP1_HUMAN	Bone morphogenetic protein 1 precursor	-3.124773
22	BMP5	BMP5_HUMAN	Bone morphogenetic protein 5 precursor	-3.662486
23	BSN	BSN_HUMAN	Protein bassoon	-3.536765
24	C10orf95	CJ095_HUMAN	Protein C10orf95	-2.6395
25	C4orf6	CD006_HUMAN	Protein C4orf6	-6.121215
26	CA6	CAH6_HUMAN	Carbonic anhydrase 6 precursor	-3.219516
27	CAMTA1	CMTA1_HUMAN	Calmodulin-binding transcription activator 1	-3.154883
28	CAPN9	CAN9_HUMAN	Calpain-9	-3.410331
29	CCL18	CCL18_HUMAN	Small inducible cytokine A18 precursor	-2.833614
30	CD24	CD24_HUMAN	Signal transducer CD24 precursor	-3.140436
31	CD72	CD72_HUMAN	B-cell differentiation antigen CD72	-4.317416
32	CDC14A	CC14A_HUMAN	Dual specificity protein phosphatase CDC14A	-2.809305
33	CDH1	CADH1_HUMAN	Epithelial-cadherin precursor	-3.663016
34	CDH12	CAD12_HUMAN	Cadherin-12 precursor	-3.319399
35	CDH8	CADH8_HUMAN	Cadherin-8 precursor	-5.49335
36	CDR1	CDR1_HUMAN	Cerebellar degeneration-related antigen 1	-2.525788
37	CFTR	CFTR_HUMAN	Cystic fibrosis transmembrane conductance regulator	-3.320688
38	CGNL1	CGNL1	cingulin-like 1	-5.672097
39	CHEK1	CHK1_HUMAN	Serine/threonine-protein kinase Chk1	-3.759376
40	CIAS1	CIAS1_HUMAN	Cold autoinflammatory syndrome 1 protein	-3.475717
41	CNGA3	CNGA3_HUMAN	Cyclic nucleotide-gated cation channel alpha 3	-3.199939
42	CNTF	CNTF_HUMAN	Ciliary neurotrophic factor	-2.532328
43	COBL			-2.685338
44	COCH	COCH_HUMAN	Cochlin precursor	-8.80296
45	CREB5	CREB5_HUMAN	cAMP response element-binding protein 5	-3.532332
46	CRHBP	CRHBP_HUMAN	Corticotropin-releasing factor-binding protein precursor	-5.417404
47	CTNNA2	CTN2_HUMAN	Alpha-2 catenin	-4.905475
48	CUL3	CUL3_HUMAN	Cullin-3	-2.903807

49	CYFIP2	CYFIP2	cytoplasmic FMR1 interacting protein 2	-2.814557
50	CYP2A13	CP2AD_HUMAN	Cytochrome P450 2A13	-2.875833
51	DCC	DCC_HUMAN	Netrin receptor DCC precursor	-3.003071
52	DDX3Y	DDX3Y_HUMAN	ATP-dependent RNA helicase DDX3Y	-3.272888
53	DEFA5	DEF5_HUMAN	Defensin 5 precursor	-2.808173
54	DGKE	DGKE_HUMAN	Diacylglycerol kinase epsilon	-2.920649
55	DHFR	DYR_HUMAN	Dihydrofolate reductase	-3.085372
56	DPF1	DPF1_HUMAN	Zinc-finger protein neuro-d4	-2.999249
57	DUSP8	DUS8_HUMAN	Dual specificity protein phosphatase 8	-3.486998
58	EBI2	EBI2_HUMAN	EBV-induced G-protein coupled receptor 2	-4.242447
59	EEF1A2	EF1A2_HUMAN	Elongation factor 1-alpha 2	-3.817044
60	ELAVL1	ELAV1_HUMAN	ELAV-like protein 1	-5.626709
61	ELN	ELN_HUMAN	Elastin precursor	-2.865539
62	ENO2	ENOG_HUMAN	Gamma-enolase	-3.076247
63	EREG	EREG_HUMAN	Epiregulin precursor	-6.95709
64	EXTL1	EXTL1_HUMAN	Exostosin-like 1	-3.25118
65	F13B	F13B_HUMAN	Coagulation factor XIII B chain precursor	-2.82103
66	F3	TF_HUMAN	Tissue factor precursor	-2.585259
67	FAM13C1	FA13C_HUMAN	Protein FAM13C1	-5.739548
68	FCGR2A	FCG2A_HUMAN	Low affinity immunoglobulin gamma Fc region receptor II-a precursor(Fc-gamma RII-a	-4.740061
69	FCGR2B	FCG2B_HUMAN	Low affinity immunoglobulin gamma Fc region receptor II-b precursor(Fc-gamma RII-b	-4.740061
70	FCGR2C	FCG2C_HUMAN	Low affinity immunoglobulin gamma Fc region receptor II-c precursor(Fc-gamma RII-c	-4.740061
71	FLJ16360			-2.593404
72	FOLH1	FOLH1_HUMAN	Glutamate carboxypeptidase 2	-2.605359
73	FOXC2	FOXC2_HUMAN	Forkhead box protein C2	-2.689234
74	FOXE1	FOXE1_HUMAN	Forkhead box protein E1	-3.742079
		FOXE2_HUMAN	Forkhead box protein E2	-3.742079
75	FPRL2	FPRL2_HUMAN	FMLP-related receptor II	-3.017556
76	FRMPD4	PDZK10	PDZ domain containing 10	-5.666975
77	GABRA5	GBRA5_HUMAN	Gamma-aminobutyric-acid receptor alpha-5 subunit	-4.401125
78	GABRR1	GBRR1_HUMAN	Gamma-aminobutyric-acid receptor rho-1 subunit	-4.997333
79	GEM	GEM_HUMAN	GTP-binding protein GEM	-2.986101
80	GFI1	GFI1_HUMAN	Zinc finger protein Gfi-1	-2.621714
81	GJA4	CXA4_HUMAN	Gap junction alpha-4 protein	-4.176222
82	GPC5	GPC5_HUMAN	Glypican-5 precursor	-2.510648
83	GPM6B	GPM6B_HUMAN	Neuronal membrane glycoprotein M6-b	-8.412642
84	GPR116	GP116_HUMAN	Probable G-protein coupled receptor 116 precursor	-6.98151
85	GREB1	GREB1	GREB1 protein	-3.084368
86	GRIA1	GRIA1_HUMAN	Glutamate receptor 1 precursor	-3.772658
87	GRIA3	GRIA3_HUMAN	Glutamate receptor 3 precursor	-3.433834
88	GRIK1	GRIK1_HUMAN	Glutamate receptor, ionotropic kainate 1 precursor	-4.484192
89	GSTT1	GSTT1_HUMAN	Glutathione S-transferase theta-1	-4.612103
90	GUCY1A2	GCYA2_HUMAN	Guanylate cyclase soluble subunit alpha-2	-3.240715
91	GZMB	GRAB_HUMAN	Granzyme B precursor	-2.609053
92	HBD	HBD_HUMAN	Hemoglobin subunit delta	-2.516559
93	HBEGF	HBEGF_HUMAN	Heparin-binding EGF-like growth factor precursor	-2.805642
94	HCG4			-2.751853
95	HDAC9	HDAC9_HUMAN	Histone deacetylase 9	-6.245225

96	HIPK2	HIPK2_HUMAN	Homeodomain-interacting protein kinase 2	-3.146048
97	HIST1H3D			-4.106922
98	HLA-DQB1	HB24_HUMAN	HLA class II histocompatibility antigen, DQ(3	-4.113697
		HB23_HUMAN	HLA class II histocompatibility antigen, DQ(W3	-4.113697
		HB25_HUMAN	HLA class II histocompatibility antigen, DQB1*0602 beta chainprecursor	-4.113697
		HB22_HUMAN	HLA class II histocompatibility antigen, DQ(W1.1	-4.113697
		HB21_HUMAN	HLA class II histocompatibility antigen, DQ(1	-4.113697
99	HOXA1	HXA1_HUMAN	Homeobox protein Hox-A1	-3.18857
100	HSPA6	HSP76_HUMAN	Heat shock 70 kDa protein 6	-3.333243
101	HSPB3	HSPB3_HUMAN	Heat-shock protein beta-3	-2.792589
102	HTN3	HIS3_HUMAN	Histatin-3 precursor	-5.672097
103	IARS2	SYIM_HUMAN	Isoleucyl-tRNA synthetase, mitochondrial precursor	-3.371044
104	IFNG	IFNG_HUMAN	Interferon gamma precursor	-4.264637
105	IGF2BP3	IMP-3	IGF-II mRNA-binding protein 3	-3.087157
106	IL13RA2	I13R2_HUMAN	Interleukin-13 receptor alpha-2 chain precursor	-2.801424
107	IL27RA	I27RA_HUMAN	Interleukin-27 receptor alpha chain precursor	-2.880889
108	IL7R	IL7RA_HUMAN	Interleukin-7 receptor alpha chain precursor	-2.672091
109	INHBA	INHBA_HUMAN	Inhibin beta A chain precursor	-4.812137
110	INPP5D	INPP5D	inositol polyphosphate-5-phosphatase, 145kDa	-2.634596
111	INSM1	INSM1_HUMAN	Insulinoma-associated protein 1	-3.181687
112	ITGA4	ITA4_HUMAN	Integrin alpha-4 precursor	-3.73107
113	ITGB7	ITB7_HUMAN	Integrin beta-7 precursor	-2.5247
114	ITK	ITK_HUMAN	Tyrosine-protein kinase ITK/TSK	-2.684613
115	KALRN	DUET_HUMAN	Serine/threonine-protein kinase Duet	-6.518842
		HAPIP_HUMAN	Huntingtin-associated protein-interacting protein	-6.518842
116	KCNAB1	KCAB1_HUMAN	Voltage-gated potassium channel subunit beta-1	-2.869369
117	KCNE1	KCNE1_HUMAN	Potassium voltage-gated channel subfamily E member 1	-2.717867
118	KIAA0363			-2.640574
119	KIAA0367			-2.907069
120	KIFC1	KIFC1_HUMAN	Kinesin-like protein KIFC1	-3.361864
121	KLRD1	KLRD1_HUMAN	Natural killer cells antigen CD94	-2.776442
122	KRT19	K1C19_HUMAN	Keratin, type I cytoskeletal 19	-2.777785
123	KRTAP26-	KR261_HUMAN	Keratin-associated protein 26-1	-3.194909
124	KRTHA4	K1H4_HUMAN	Keratin, type I cuticular Ha4	-5.128946
125	LCN2	NGAL_HUMAN	Neutrophil gelatinase-associated lipocalin precursor	-3.631382
126	LCT	LPH_HUMAN	Lactase-phlorizin hydrolase precursor	-2.513676
127	LMTK2	LMTK2	lemur tyrosine kinase 2	-2.725746
128	LOC13118			-4.321748
129	LOC19699			-2.815249
130	LOC40113			-3.057056
131	LOC44113			-3.285847
132	LOC64331			-2.818167
133	LOC64334			-3.005444
134	LOC64350			-3.085372
135	LOC64445			-3.057056
136	LOC64561			-3.346834
137	LOC65287			-3.333243
138	LOC65316			-4.163485

139	LOC65317			-4.163485
140	LOC65318			-2.639024
141	LOC65319			-2.83397
142	LOC65327			-4.163485
143	LOC65328			-4.163485
144	LOC65329			-4.163485
145	LOC65380			-3.307819
146	LOC65387			-3.085372
147	LPHN3	LPHN3_HUMAN	Latrophilin-3 precursor	-2.757531
148	LTF	TRFL_HUMAN	Lactotransferrin precursor	-3.005444
149	LYZL6	LYZL6_HUMAN	Lysozyme-like protein 6 precursor	-2.550841
150	MAGEA10	MAGAA_HUMAN	Melanoma-associated antigen 10	-3.687163
151	MAGEB1	MAGB1_HUMAN	Melanoma-associated antigen B1	-3.894117
152	MAP3K9	M3K9_HUMAN	Mitogen-activated protein kinase kinase kinase 9	-3.031511
153	MBL2	MBL2_HUMAN	Mannose-binding protein C precursor	-2.680739
154	MEF2C	MEF2C_HUMAN	Myocyte-specific enhancer factor 2C	-2.513058
155	MKI67	KI67_HUMAN	Antigen KI-67	-3.283332
156	MMP1	MMP1_HUMAN	Interstitial collagenase precursor	-8.451402
157	MPO	PERM_HUMAN	Myeloperoxidase precursor	-2.658912
158	MRPS6	RT06_HUMAN	Mitochondrial 28S ribosomal protein S6	-2.672874
159	MSH4	MSH4_HUMAN	MutS protein homolog 4	-2.530269
160	MTSS1	MTSS1_HUMAN	Metastasis suppressor protein 1	-3.860041
161	MUC4	MUC4_HUMAN	Mucin-4	-2.879738
162	MYB	MYB_HUMAN	Myb proto-oncogene protein	-4.436025
163	MYCN	MYCN_HUMAN	N-myc proto-oncogene protein	-3.950817
164	MYH11	MYH11_HUMAN	Myosin-11	-6.949236
165	MYL1	MLE1_HUMAN	Myosin light chain 1, skeletal muscle isoform	-4.403763
		MLE3_HUMAN	Myosin light chain 3, skeletal muscle isoform	-4.403763
166	MYOZ2	MYOZ2_HUMAN	Myozenin-2	-6.798045
167	NCAM1	NCA12_HUMAN	Neural cell adhesion molecule 1, 120 kDa isoform precursor	-2.934646
		NCA11_HUMAN	Neural cell adhesion molecule 1, 140 kDa isoform precursor	-2.934646
168	NCAM2	NCAM2_HUMAN	Neural cell adhesion molecule 2 precursor	-3.219515
169	NEFL	NFL_HUMAN	Neurofilament triplet L protein	-10.25946
170	NHLH2	HEN2_HUMAN	Helix-loop-helix protein 2	-3.362972
171	NMNAT2	NMNA2_HUMAN	Nicotinamide mononucleotide adenylyltransferase 2	-4.780029
172	NMU	NEUU_HUMAN	Neuromedin U-25 precursor	-4.373017
173	NNT	NNTM_HUMAN	NAD(P	-4.786721
174	NRL	NRL_HUMAN	Neural retina-specific leucine zipper protein	-2.576296
175	NTF3	NT3_HUMAN	Neurotrophin-3 precursor	-2.712938
176	OASL	OASL_HUMAN	59 kDa 2'-5'-oligoadenylate synthetase-like protein	-3.560357
177	OLR1	OLR1_HUMAN	Oxidized low-density lipoprotein receptor 1	-3.31983
178	OR7A5	OR7A5_HUMAN	Olfactory receptor 7A5	-5.30797
		OR7C1_HUMAN	Olfactory receptor 7C1	-5.30797
179	P2RY14	P2Y14_HUMAN	P2Y purinoceptor 14	-4.843465
180	PADI2	PADI2_HUMAN	Protein-arginine deiminase type-2	-3.397954
181	PAFAH1B1	LIS1_HUMAN	Platelet-activating factor acetylhydrolase IB subunit alpha	-2.641878
182	PAGE4	GAGC1_HUMAN	G antigen family C member 1	-2.731361

183	PCDH8	PCDH8_HUMAN	Protocadherin-8 precursor	-2.90794
184	PDZD2	PDZD2_HUMAN	PDZ domain-containing protein 2	-4.470756
185	PDZK1	PDZD1_HUMAN	PDZ domain-containing protein 1	-3.568262
186	PEG10	PEG10	paternally expressed 10	-2.671982
187	PHF14	PHF14_HUMAN	PHD finger protein 14	-2.588671
188	PHTF2			-4.105683
189	PLK4	PLK4_HUMAN	Serine/threonine-protein kinase PLK4	-3.531899
190	PLXNB3	PLXB3_HUMAN	Plexin-B3 precursor	-3.444762
191	POM121L			-3.285847
192	PON1	PON1_HUMAN	Serum paraoxonase/arylesterase 1	-2.56509
193	POU1F1	PIT1_HUMAN	Pituitary-specific positive transcription factor 1	-3.274507
194	POU2AF1	OBF1_HUMAN	POU domain class 2-associating factor 1	-3.739764
195	POU4F2	PO4F2_HUMAN	POU domain, class 4, transcription factor 2	-4.419022
196	PPFIA4	LIPA4_HUMAN	Liprin-alpha-4	-2.903337
197	PRIM1	PRI1_HUMAN	DNA primase small subunit	-7.070114
198	PRKACA	KAPCA_HUMAN	cAMP-dependent protein kinase, alpha-catalytic subunit	-3.018282
199	PROC	PROC_HUMAN	Vitamin K-dependent protein C precursor	-2.957593
200	PRUNE	PRUNE	prune homolog (Drosophila)	-2.666869
201	PTGS2	PGH2_HUMAN	Prostaglandin G/H synthase 2 precursor	-3.828363
202	PTPN20A			-3.286968
203	PTPN3	PTN3_HUMAN	Tyrosine-protein phosphatase non-receptor type 3	-3.685557
204	PTPRZ1	PTPRZ_HUMAN	Receptor-type tyrosine-protein phosphatase zeta precursor(EC 3.1.3.48	-11.82042
205	RAB9P1			-2.749646
206	RAD23B	RD23B_HUMAN	UV excision repair protein RAD23 homolog B	-4.321748
207	RASSF8	RASF8_HUMAN	Ras association domain-containing protein 8	-2.509392
208	RELN	RELN_HUMAN	Reelin precursor	-4.718045
209	RGS7	RGS7_HUMAN	Regulator of G-protein signaling 7	-3.062901
210	RIT2	RIT2_HUMAN	GTP-binding protein Rit2	-3.116372
211	RLN2	REL2_HUMAN	Prorelaxin H2 precursor [Contains: Relaxin B chain	-4.477747
212	RNASE3	ECP_HUMAN	Eosinophil cationic protein precursor	-2.729881
213	RP11-	PTPN20	OTTHUMP00000059234	-3.286968
214	RUNX1	RUNX1_HUMAN	Runt-related transcription factor 1	-3.055606
215	SCGB2A2	SG2A2_HUMAN	Mammaglobin-A precursor	-2.82099
216	SCN5A	SCN5A_HUMAN	Sodium channel protein type 5 subunit alpha	-2.786872
217	SCNN1B	SCNNB_HUMAN	Amiloride-sensitive sodium channel subunit beta	-2.622084
218	SERPINB2	PAI2_HUMAN	Plasminogen activator inhibitor 2 precursor	-13.21605
219	SERPINB5	SPB5_HUMAN	Serpin B5 precursor	-2.723247
220	SERPINI2	SPI2_HUMAN	Serpin I2 precursor	-3.229512
221	SGCG	SGCG_HUMAN	Gamma-sarcoglycan	-3.02762
222	SH3GL2	SH3G2_HUMAN	SH3-containing GRB2-like protein 2	-3.00813
223	SI	SUIS_HUMAN	Sucrase-isomaltase, intestinal [Contains: Sucrase	-2.575704
224	SIM1	SIM1_HUMAN	Single-minded homolog 1	-2.740131
225	SKI	SKI_HUMAN	Ski oncogene	-3.183025
226	SLC15A2	S15A2_HUMAN	Oligopeptide transporter, kidney isoform	-3.20351
227	SLC17A7	SLC17A7	solute carrier family 17 (sodium-dependent inorganic phosphate cotransporter), member 7	-3.359076
228	SLC1A3	EAA1_HUMAN	Excitatory amino acid transporter 1	-2.715573
229	SLC8A2	NAC2_HUMAN	Sodium/calcium exchanger 2 precursor	-2.785856
230	SMCY	JAD1D_HUMAN	Jumonji/ARID domain-containing protein 1D	-4.594112
231	SOX21	SOX21_HUMAN	Transcription factor SOX-21	-3.914521

232	SPARCL1	SPRL1_HUMAN	SPARC-like protein 1 precursor	-2.958924
233	SPIN-2			-2.685879
234	SPIN2	SPIN3_HUMAN	Spindlin-3	-2.685879
		SPIN2_HUMAN	Spindlin-2	-2.685879
235	SPINK1	IPK1_HUMAN	Pancreatic secretory trypsin inhibitor precursor	-3.970528
236	SPOCK3	TICN3_HUMAN	Testican-3 precursor	-3.702054
237	STAC	STAC_HUMAN	SH3 and cysteine-rich domain-containing protein	-3.991224
238	STK23	STK23_HUMAN	Serine/threonine-protein kinase 23	-3.444762
239	STXBP2	STXB2_HUMAN	Syntaxin-binding protein 2	-3.546714
240	SV2C	SV2C_HUMAN	Synaptic vesicle glycoprotein 2C	-2.60048
241	TAC1	TKN1_HUMAN	Protachykinin 1 precursor	-4.963902
242	TACR1	NK1R_HUMAN	Substance-P receptor	-2.737741
243	TAT	ATTY_HUMAN	Tyrosine aminotransferase	-3.663619
244	TGM5	TGM5_HUMAN	Protein-glutamine gamma-glutamyltransferase 5	-2.503935
245	THBD	TRBM_HUMAN	Thrombomodulin precursor	-2.892074
246	TNK2	ACK1_HUMAN	Activated CDC42 kinase 1	-2.532969
247	TRHR	TRFR_HUMAN	Thyrotropin-releasing hormone receptor	-3.527146
248	TRIM31	TRI31_HUMAN	Tripartite motif-containing protein 31	-3.729988
249	TRPC3	TRPC3_HUMAN	Short transient receptor potential channel 3	-2.751472
250	TSPY1	TSPY1_HUMAN	Testis-specific Y-encoded protein 1	-4.163485
251	TSPY2	TSPY2	testis specific protein, Y-linked 2	-4.163485
252	UGT2B15	UDB15_HUMAN	UDP-glucuronosyltransferase 2B15 precursor	-2.639024
253	UGT2B17	UDB17_HUMAN	UDP-glucuronosyltransferase 2B17 precursor	-2.639024
254	UNC13A	UN13A_HUMAN	Unc-13 homolog A	-3.765954
255	USH2A	USH2A_HUMAN	Usherin precursor	-5.296962
256	USP9Y	USP9Y_HUMAN	Probable ubiquitin carboxyl-terminal hydrolase FAF-Y	-5.324088
257	VAPB	VAPB_HUMAN	Vesicle-associated membrane protein-associated protein B/C	-3.40723
258	VEGFC	VEGFC_HUMAN	Vascular endothelial growth factor C precursor	-2.843876
259	VIPR1	VIPR1_HUMAN	Vasoactive intestinal polypeptide receptor 1 precursor	-3.185638
260	ZG16	ZG16_HUMAN	Zymogen granule membrane protein 16 precursor	-3.307819
261	ZIC1	ZIC1_HUMAN	Zinc finger protein ZIC 1	-2.662012

Group 1 and 2 combined, down-regulated AFFI gene IDs, fold change > 2.5

#	Affi Gene ID	Gene Network Symbol	Encoding protein name
1	33516_at	11p155/HBD	HBD("hemoglobin, delta")
2	31754_at	ABCA12	ABCA12("ATP-binding cassette, sub-family A (ABC1), member 12")
3	36816_s_at	ABCC7	CFTR("cystic fibrosis transmembrane conductance regulator, ATP-binding cassette (sub-family C, member 7)")
4	37186_s_at	ABP1	ABP1("amiloride binding protein 1 (amine oxidase (copper-containing))")
5	32537_at	ACSBG1	ACSBG1("acyl-CoA synthetase bubblegum family member 1")
6	41654_at, 907_at	ADA	ADA("adenosine deaminase")
7	40350_at	ADAM23	ADAM23("ADAM metallopeptidase domain 23")
8	37089_at	ADAM3A	ADAM3A("ADAM metallopeptidase domain 3a (cyritestin 1)")
9	33529_at	ADH7	ADH7("alcohol dehydrogenase 7 (class IV), mu or sigma polypeptide")
10	39069_at	AEBP1	AEBP1("AE binding protein 1")
11	32331_at	AK3	AK3L1("adenylate kinase 3-like 1")
12	32919_at	AMIGO2	AMIGO2("adhesion molecule with Ig-like domain 2")
13	2026_at	AML1/MDS1/EVI1 fusion protein *	MDS1("myelodysplasia syndrome 1")
14	35049_g_at, 36854_s_at	AMPA receptor *	GRIA1("glutamate receptor, ionotropic, AMPA 1"), GRIA3("glutamate receptor, ionotrophic, AMPA 3")
15	35433_s_at	APC protein	APC("adenomatosis polyposis coli")
16	40135_at	ATPK	ATP5J2("ATP synthase, H+ transporting, mitochondrial F0 complex, subunit F2")
17	1197_at	ACTG2	ACTG2("actin, gamma 2, smooth muscle, enteric")
18	1203_at	Activin beta A	INHBA("inhibin, beta A (activin A, activin AB alpha polypeptide)")
19	36246_at	Adenylate cyclase type VIII	ADCY8("adenylate cyclase 8 (brain)")
20	38965_at, 39206_s_at,	Aggrecan	AGC1("aggrecan 1 (chondroitin sulfate proteoglycan 1, large aggregating proteoglycan, antigen identified by monoclonal antibody A0122)")
21	33990_at,	Albumin	ALB("albumin")
22	32242_at	Alpha crystallin B	CRYAB("crystallin, alpha B")
23	208_at	Alpha-catenin *	CTNNA2("catenin (cadherin-associated protein), alpha 2")
24	34898_at	Amphiregulin	AREG("amphiregulin (schwannoma-derived growth factor)")
25	346_s_at	Angiotensin II receptor, type-1	AGTR1("angiotensin II receptor, type 1")
26	36966_at	Ankyrin-G	ANK3("ankyrin 3, node of Ranvier (ankyrin G)")
27	419_at	Antigen KI-67	MKI67("antigen identified by monoclonal antibody Ki-67")
28	36156_at	Aquaporin 1	AQP1("aquaporin 1 (Colton blood group)")
29	36824_at	Astrotactin-1	ASTN("astrotactin")
30	38583_at	B3GT2	B3GALT2("UDP-Gal:betaGlcNAc beta 1,3-galactosyltransferase, polypeptide 2")
31	37841_at	BChE	BCHE("butyrylcholinesterase")
32	39407_at	BMP1	BMP1("bone morphogenetic protein 1")
33	31314_at	BMP3	BMP3("bone morphogenetic protein 3 (osteogenic)")
34	200_at	BMP5	BMP5("bone morphogenetic protein 5")
35	36239_at	BOB1	POU2AF1("POU domain, class 2, associating factor 1")
36	1687_s_at	Bak	BAK1("BCL2-antagonist/killer 1")
37	33728_at	Bassoon	BSN("bassoon (presynaptic cytomatrix protein)")
38	979_g_at	Bcl-6	BCL6("B-cell CLL/lymphoma 6 (zinc finger protein 51)")
39	31848_at	CADPS	CADPS("Ca2+-dependent secretion activator")
40	32128_at	CCL18	CCL18("chemokine (C-C motif) ligand 18 (pulmonary and activation- [illegible]
41	1404_r_at	CCL5	CCL5("chemokine (C-C motif) ligand 5")
42	1128_s_at	CCR1	CCR1("chemokine (C-C motif) receptor 1")
43	266_s_at	CD24	CD24("CD24 antigen (small cell lung carcinoma cluster 4 antigen)")

44	37479_at	CD72	CD72("CD72 antigen")
45	33531_at	CD94	KLRD1("killer cell lectin-like receptor subfamily D, member 1")
46	40320_at	CDC14a	CDC14A("CDC14 cell division cycle 14 homolog A (S. cerevisiae)")
47	35616_at	CIITA	CIITA("class II, major histocompatibility complex, transactivator")
48	32404_at	CNGA3	CNGA3("cyclic nucleotide gated channel alpha 3")
49	34590_at	CNTF	CNTF("ciliary neurotrophic factor")
50	37459_at	COL8A1	COL8A1("collagen, type VIII, alpha 1")
51	1069_at	COX-2	PTGS2("prostaglandin-endoperoxide synthase 2 (prostaglandin G/H synthase
52	670_s_at	CREB5	CREB5("cAMP responsive element binding protein 5")
53	41608_at	CRF-BP	CRHBP("corticotropin releasing hormone binding protein")
54	33232_at	CRIP1	CRIP1("cysteine-rich protein 1 (intestinal)")
55	41401_at	CRP2	CSRP2("cysteine and glycine-rich protein 2")
56	110_at,	CSPG4 (NG2)	CSPG4("chondroitin sulfate proteoglycan 4 (melanoma-associated)")
57	41355_at	CTIP1	BCL11A("B-cell CLL/lymphoma 11A (zinc finger protein)")
58	37579_at	CYFIP2	CYFIP2("cytoplasmic FMR1 interacting protein 2")
59	1553_r_at	CYP2A6	CYP2A6("cytochrome P450, family 2, subfamily A, polypeptide 6")
60	32266_at	Cadherin 12	CDH12("cadherin 12, type 2 (N-cadherin 2)")
61	37289_at, 37290_at	Cadherin 8	CDH8("cadherin 8, type 2")
62	34203_at	Calponin H1	CNN1("calponin 1, basic, smooth muscle")
64	679_at	Cathepsin G	CTSG("cathepsin G")
65	40168_at	Caveolin-2	CAV2("caveolin 2")
66	38920_at	Chk1	CHEK1("CHK1 checkpoint homolog (S. pombe)")
67	34616_at	Coagulation factor XIII *	F13B("coagulation factor XIII, B polypeptide")
68	40687_at	Connexin 37	GJA4("gap junction protein, alpha 4, 37kDa (connexin 37)")
69	37930_at	ATP7B	ATP7B("ATPase, Cu++ transporting, beta polypeptide")
70	37702_at	Cullin 3	CUL3("cullin 3")
71	36650_at	Cyclin D2	CCND2("cyclin D2")
72	34870_at	Cypher	LDB3("LIM domain binding 3")
73	474_at	DGKE_HUMAN	DGKE("diacylglycerol kinase, epsilon 64kDa")
74	1806_at	DBL	MCF2("MCF.2 cell line derived transforming sequence")
75	1610_s_at	DHFR	DHFR("dihydrofolate reductase")
76	40525_at	DOT1L	DOT1L("DOT1-like, histone H3 methyltransferase (S. cerevisiae)")
77	35679_s_at	DPP6	DPP6("dipeptidyl-peptidase 6")
78	38562_g_at	DRIM	UTP20("UTP20, small subunit (SSU) processome component, homolog (yeast)")
79	1561_at	DUSP8	DUSP8("dual specificity phosphatase 8")
80	34972_s_at	Dynein, axonemal, heavy [illegible]	DNAH17("dynein, axonemal, heavy polypeptide 17")
81	40488_at	Dystrophin	DMD("dystrophin (muscular dystrophy, Duchenne and Becker types)")
82	977_s_at	E-cadherin	CDH1("cadherin 1, type 1, E-cadherin (epithelial)")
83	33979_at	ECP (RNase 3)	RNASE3("ribonuclease, RNase A family, 3 (eosinophil cationic protein)")
84	39901_at	EDIL3	EDIL3("EGF-like repeats and discoidin I-like domains 3")
85	40378_at	EEN-B1<br>Endophilin A *	SH3GL2("SH3-domain GRB2-like 2")
86	39384_at	ELAVL1 (HuR)	ELAVL1("ELAV (embryonic lethal, abnormal vision, Drosophila)-like 1 (Hu antigen R)")
87	36410_f_at	ELAVL2	ELAVL2("ELAV (embryonic lethal, abnormal vision, Drosophila)-like 2 (Hu antigen B)")
88	40193_at	ENO2	ENO2("enolase 2 (gamma, neuronal)")
89	342_at,	ENPP1	ENPP1("ectonucleotide pyrophosphatase/phosphodiesterase 1")
90	31843_at	ERR3	ESRRG("estrogen-related receptor gamma")
91	33669_at	ESR1	ESR1("estrogen receptor 1")

92	914_g_at	ERG	ERG("v-ets erythroblastosis virus E26 oncogene like (avian)")
93	38140_at	EXTL1	EXTL1("exostoses (multiple)-like 1")
94	31621_s_at	Elastin	ELN("elastin (supravalvular aortic stenosis, Williams-Beuren syndrome)")
95	32050_r_at	Elongin A	TCEB3("transcription elongation factor B (SIII), polypeptide 3 (110kDa, elongin
96	902_at	Ephrin-B receptors *	EPHB2("EPH receptor B2")
97	34476_r_at	Epiregulin	EREG("epiregulin")
98	36543_at	F3	F3("coagulation factor III (thromboplastin, tissue factor)")
99	1732_at	FGF5	FGF5("fibroblast growth factor 5")
100	1739_at	FOLH1 (GCP2)	FOLH1("folate hydrolase (prostate-specific membrane antigen) 1")
101	31698_at	FOXC2	FOXC2("forkhead box C2 (MFH-1, mesenchyme forkhead 1)")
102	36370_at	FOXE1	FOXE1("forkhead box E1 (thyroid transcription factor 2)")
103	41323_at	FOXM1B	FOXM1("forkhead box M1")
104	33092_at	FPRL2	FPRL2("formyl peptide receptor-like 2")
105	1808_s_at	FasR(CD95)	FAS("Fas (TNF receptor superfamily, member 6)")
106	34664_at,	Fc gamma RII beta	FCGR2B("Fc fragment of IgG, low affinity IIb, receptor (CD32)")
107	236_at	G-protein alpha-O 2	GNAO1("guanine nucleotide binding protein (G protein), alpha activating activity
108	39321_at	GABA-A receptor alpha-5	GABRA5("gamma-aminobutyric acid (GABA) A receptor, alpha 5")
109	33051_at	GABA receptor rho-1	GABRR1("gamma-aminobutyric acid (GABA) receptor, rho 1")
110	37279_at	GEM	GEM("GTP binding protein overexpressed in skeletal muscle")
111	33977_at	GFI-1	GFI1("growth factor independent 1")
112	34158_s_at, 36609_at	GLAST1/EAAT1	SLC1A3("solute carrier family 1 (glial high affinity glutamate transporter), member 3")
113	36009_at	GPX6	GPX6("glutathione peroxidase 6 (olfactory)")
114	38875_r_at	GREB1	GREB1("GREB1 protein")
115	34905_at	GRIK5	GRIK5("glutamate receptor, ionotropic, kainate 5")
116	37222_at,	GSTT1	GSTT1("glutathione S-transferase theta 1")
117	1099_s_at	GSTT2	GSTT2("glutathione S-transferase theta 2")
118	41299_f_at	GTL3	GTL3("transcription factor IIB")
119	610_at	Beta-2 adrenergic receptor	ADRB2("adrenergic, beta-2-, receptor, surface")
120	33462_at	Galpha(i)-specific class A orphan/other GPCRs *	P2RY14("purinergic receptor P2Y, G-protein coupled, 14")
121	36716_at	Galpha(q)-specific amine [illegible]	ADRA1A("adrenergic, alpha-1A-, receptor")
122	1128_s_at, 33092_at,	Galpha(q)-specific peptide GPCRs *	AGTR1("angiotensin II receptor, type 1"), CCR1("chemokine (C-C motif) receptor 1"), FPRL2("formyl peptide receptor-like 2"), TACR1("tachykinin
123	38749_at	GPR39	GPR39("G protein-coupled receptor 39")
124	33766_at	VIP receptor 1	VIPR1("vasoactive intestinal peptide receptor 1")
125	34578_at	Gamma-sarcoglycan	SGCG("sarcoglycan, gamma (35kDa dystrophin-associated glycoprotein)")
126	1597_at	Gas6	GAS6("growth arrest-specific 6")
127	36854_s_at	GluR1	GRIA1("glutamate receptor, ionotropic, AMPA 1")
128	35049_g_at	GluR3	GRIA3("glutamate receptor, ionotrophic, AMPA 3")
129	35857_at	GluR5	GRIK1("glutamate receptor, ionotropic, kainate 1")
130	37137_at	Granzyme B	GZMB("granzyme B (granzyme 2, cytotoxic T-lymphocyte-associated serine esterase 1)")
131	32301_at	GUCY1A2	GUCY1A2("guanylate cyclase 1, soluble, alpha 2")
132	2073_s_at, 483_g_at	H-cadherin	CDH13("cadherin 13, H-cadherin (heart)")
133	38037_at	HB-EGF	HBEGF("heparin-binding EGF-like growth factor")
134	36877_at,	HLA-DQB1	HLA-DQB1("major histocompatibility complex, class II, DQ beta 1")
135	34517_at	HMGCS1	HMGCS1("3-hydroxy-3-methylglutaryl-Coenzyme A synthase 1 (soluble)")
136	41486_at	HOJ-1	RASSF8("Ras association (RalGDS/AF-6) domain family 8")
137	502_s_at	HOXA1	HOXA1("homeobox A1")
138	35982_at	HSBP3	HSPB3("heat shock 27kDa protein 3")
139	117_at	HSPA6	HSPA6("heat shock 70kDa protein 6 (HSP70B')")

140	34158_s_at, 36609_at,	High-affinity glutamate transporters *	SLC1A1("solute carrier family 1 (neuronal/epithelial high affinity glutamate transporter, system Xag), member 1"), SLC1A3("solute carrier family 1 (glial
141	41148_at	Histatin 3(5)	HTN3("histatin 3")
142	37483_at	HDAC9	HDAC9("histone deacetylase 9")
143	33157_at	IA-1	INSM1("insulinoma-associated 1")
144	34718_at	IGF-1 receptor	IGF1R("insulin-like growth factor 1 receptor")
145	37558_at	IGF2BP3	IGF2BP3("insulin-like growth factor 2 mRNA binding protein 3")
146	31327_at	IGHG1	IGHG1("immunoglobulin heavy constant gamma 1 (G1m marker)")
147	1016_s_at	IL13RA2	IL13RA2("interleukin 13 receptor, alpha 2")
148	36227_at	IL7RA	IL7R("interleukin 7 receptor")
149	884_at,	ITGA3	ITGA3("integrin, alpha 3 (antigen CD49C, alpha 3 subunit of VLA-3 receptor)")
150	2061_at,	ITGA4	ITGA4("integrin, alpha 4 (antigen CD49D, alpha 4 subunit of VLA-4 receptor)")
151	33410_at, [illegible]	ITGA6	ITGA6("integrin, alpha 6")
152	2019_s_at	ITGB7	ITGB7("integrin, beta 7")
153	1478_at	ITK	ITK("IL2-inducible T-cell kinase")
154	2019_s_at,	Integrin *	ITGA3("integrin, alpha 3 (antigen CD49C, alpha 3 subunit of VLA-3 receptor)"),
155	1021_at, 40702_at	Interferon * IFN-gamma	IFNG("interferon, gamma")
156	34905_at,	Ionotropic glutamate	GRIA1("glutamate receptor, ionotropic, AMPA 1"), GRIA3("glutamate receptor,
157	32709_at,	KCNAB1	KCNAB1("potassium voltage-gated channel, shaker-related subfamily, beta
158	34905_at,	Kainate receptor *	GRIK1("glutamate receptor, ionotropic, kainate 1"), GRIK5("glutamate receptor,
159	40899_at	Keratin 19	KRT19("keratin 19")
160	41294_at	Keratin 7	KRT7("keratin 7")
161	34012_at	Keratin HA4	KRTHA4("keratin, hair, acidic, 4")
162	32328_at	Keratin HB5	KRTHB5("keratin, hair, basic, 5")
163	34507_s_at	LMTK2	LMTK2("lemur tyrosine kinase 2")
164	32917_at	LPH	LCT("lactase")
165	35712_at	LRRN3	LRRN3("leucine rich repeat neuronal 3")
166	37149_s_at	Lactoferrin	LTF("lactotransferrin")
167	37765_at	Leiomodin	LMOD1("leiomodin 1 (smooth muscle)")
168	38167_at	Liprin-alpha4	PPFIA4("protein tyrosine phosphatase, receptor type, f polypeptide (PTPRF), [illegible]
169	37251_s_at	M6B	GPM6B("glycoprotein M6B")
170	32946_r_at	MBL	MBL2("mannose-binding lectin (protein C) 2, soluble (opsonic defect)")
171	33143_s_at	MCT *	SLC16A3("solute carrier family 16 (monocarboxylic acid transporters), member
172	37712_g_at	MEF2C	MEF2C("MADS box transcription enhancer factor 2, polypeptide C (myocyte
173	40157_s_at,	MELC *	MYL1("myosin, light polypeptide 1, alkali; skeletal, fast")
174	38933_at	MKLP2	KIFC1("kinesin family member C1")
175	32847_at	MYLK1	MYLK("myosin, light polypeptide kinase")
176	38428_at	MMP-1	MMP1("matrix metallopeptidase 1 (interstitial collagenase)")
177	31714_at	MSH4	MSH4("mutS homolog 4 (E. coli)")
178	37363_at	MTSS1	MTSS1("metastasis suppressor 1")
179	35912_at	MUC4	MUC4("mucin 4, tracheobronchial")
180	32291_at	MUNC13-1	UNC13A("unc-13 homolog A (C. elegans)")
181	38259_at	MUNC18-2	STXBP2("syntaxin binding protein 2")
182	1490_at	MYCL1	MYCL1("v-myc myelocytomatosis viral oncogene homolog 1, lung carcinoma [illegible]
183	32582_at,	MYH11	MYH11("myosin, heavy polypeptide 11, smooth muscle")
184	40062_s_at	MYL4	MYL4("myosin, light polypeptide 4, alkali; atrial, embryonic")
185	35989_at	MYOZ2	MYOZ2("myozenin 2")
186	862_at,	Maspin	SERPINB5("serpin peptidase inhibitor, clade B (ovalbumin), member 5")
187	32496_at	MinK	KCNE1("potassium voltage-gated channel, Isk-related family, member 1")
188	35158_at	N-Myc	MYCN("v-myc myelocytomatosis viral related oncogene, neuroblastoma derived [illegible]

189	33598_r_at	NALP3	CIAS1("cold autoinflammatory syndrome 1")
190	41290_at	NCAM1	NCAM1("neural cell adhesion molecule 1")
191	35592_at	NCX2	SLC8A2("solute carrier family 8 (sodium-calcium exchanger), member 2")
192	36425_at	NEBL	NEBL("nebulette")
193	40995_at	NEFL	NEFL("neurofilament, light polypeptide 68kDa")
194	32821_at	NGAL	LCN2("lipocalin 2 (oncogene 24p3)")
195	36326_at,	NHLH2	NHLH2("nescient helix loop helix 2")
196	34040_s_at	NKp46	NCR1("natural cytotoxicity triggering receptor 1")
197	32090_at	NMNA2	NMNAT2("nicotinamide nucleotide adenylyltransferase 2")
198	34441_at	NNTM	NNT("nicotinamide nucleotide transhydrogenase")
199	40691_at	NRIF	ZNF274("zinc finger protein 274")
200	33159_at	NRL	NRL("neural retina leucine zipper")
201	35041_at	NT-3	NTF3("neurotrophin 3")
202	38289_r_at	Neurofibromin	NF1("neurofibromin 1 (neurofibromatosis, von Recklinghausen disease, Watson
203	33483_at	Neuromedin U	NMU("neuromedin U")
204	32759_at	Nuclear pore complex	RAE1("RAE1 RNA export 1 homolog (S. pombe)")
205	34964_at	Histone H3.1 *	HIST1H3F("histone 1, H3f")
206	269_at	OASL	OASL("2'-5'-oligoadenylate synthetase-like")
207	36043_at	OBCAM	OPCML("opioid binding protein/cell adhesion molecule-like")
208	37233_at	OLR1	OLR1("oxidised low density lipoprotein (lectin-like) receptor 1")
209	31733_at	P2X3	P2RX3("purinergic receptor P2X, ligand-gated ion channel, 3")
210	39710_at	P311	C5orf13("chromosome 5 open reading frame 13")
211	34390_at	P4HA2	P4HA2("procollagen-proline, 2-oxoglutarate 4-dioxygenase (proline 4-
212	37317_at	PAFAH alpha (LIS1)	PAFAH1B1("platelet-activating factor acetylhydrolase, isoform Ib, alpha subunit
213	38322_at	PAGE4	PAGE4("P antigen family, member 4 (prostate associated)")
214	672_at	PAI1	SERPINE1("serpin peptidase inhibitor, clade E (nexin, plasminogen activator
215	37185_at	PAI2	SERPINB2("serpin peptidase inhibitor, clade B (ovalbumin), member 2")
216	32368_at	PCDH8	PCDH8("protocadherin 8")
217	34650_at	PDE3A	PDE3A("phosphodiesterase 3A, cGMP-inhibited")
218	36157_at	PDGF-R-alpha	PDGFRA("platelet-derived growth factor receptor, alpha polypeptide")
219	35703_at	PDGF-A	PDGFA("platelet-derived growth factor alpha polypeptide")
220	39690_at	PDLIM3	PDLIM3("PDZ and LIM domain 3")
221	32027_at	PDZK1	PDZK1("PDZ domain containing 1")
222	35031_r_at	PDZK10	FRMPD4("FERM and PDZ domain containing 4")
223	40834_at	PDZK3	PDZD2("PDZ domain containing 2")
224	35863_g_at	PEPT2	SLC15A2("solute carrier family 15 (H+/peptide transporter), member 2")
225	33284_at	PERM	MPO("myeloperoxidase")
226	38195_at	PHF14	PHF14("PHD finger protein 14")
227	34479_at	PI3K reg class IA (p55-	PIK3R3("phosphoinositide-3-kinase, regulatory subunit 3 (p55, gamma)")
228	901_g_at	PIB4	PLCB4("phospholipase C, beta 4")
229	34014_f_at	PIT1	POU1F1("POU domain, class 1, transcription factor 1 (Pit1, growth hormone factor 1)")
230	569_g_at	PKA-cat (cAMP-dependent) *	PRKACA("protein kinase, cAMP-dependent, catalytic, alpha")
231	38861_at	PON1	PON1("paraoxonase 1")
232	34551_at	POU4F2	POU4F2("POU domain, class 4, transcription factor 2")
233	34897_at	PPP4R2	PPP4R2("protein phosphatase 4, regulatory subunit 2")
234	37257_at	PRUNE	PRUNE("prune homolog (Drosophila)")
235	31885_at	PTPH1	PTPN3("protein tyrosine phosphatase, non-receptor type 3")
237	1364_at	PTPR-zeta	PTPRZ1("protein tyrosine phosphatase, receptor-type, Z polypeptide 1")
	39696_at	Peg10	PEG10("paternally expressed 10")

238	39214_at	Plexin-B3	PLXNB3("plexin B3")
239	758_at	Prostacyclin receptor	PTGIR("prostaglandin I2 (prostacyclin) receptor (IP)")
240	39255_at	Protein C	PROC("protein C (inactivator of coagulation factors Va and VIIIa)")
241	36258_at	Protein kinase G 1	PRKG1("protein kinase, cGMP-dependent, type I")
242	32763_r_at	RAB3-GAP150	RAB3GAP2("RAB3 GTPase activating protein subunit 2 (non-catalytic)")
243	41157_at	RAD23B	RAD23B("RAD23 homolog B (S. cerevisiae)")
244	31666_f_at	RASSF2	RASSF2("Ras association (RalGDS/AF-6) domain family 2")
245	40653_at	RGS7	RGS7("regulator of G-protein signalling 7")
246	31450_s_at	RIN	RIT2("Ras-like without CAAX 2")
247	35083_at	RNF24	RNF24("ring finger protein 24")
248	1776_at,	RRAD	RRAD("Ras-related associated with diabetes")
249	37530_s_at	Reelin	RELN("reelin")
250	31732_at	Relaxin 2	RLN2("relaxin 2")
251	1826_at	RhoB	RHOB("ras homolog gene family, member B")
252	36329_at	SCGB2A2	SCGB2A2("secretoglobin, family 2A, member 2")
253	172_at	SHIP	INPP5D("inositol polyphosphate-5-phosphatase, 145kDa")
254	39575_at	SHREW1	AJAP1("adherens junction associated protein 1")
255	40895_g_at	SIKE	SIKE("hypothetical protein LOC80143")
256	31570_at	SIM1	SIM1("single-minded homolog 1 (Drosophila)")
257	36567_at	SLC17A7	SLC17A7("solute carrier family 17 (sodium-dependent inorganic phosphate
258	31636_s_at	SLC18A3	SLC18A3("solute carrier family 18 (vesicular acetylcholine), member 3")
259	38267_at	SLC1A1/EAAT3	SLC1A1("solute carrier family 1 (neuronal/epithelial high affinity glutamate [illegible]
260	35285_at,	SLC4A4	SLC4A4("solute carrier family 4, sodium bicarbonate cotransporter, member 4")
261	35324_at	SLIT3	SLIT3("slit homolog 3 (Drosophila)")
262	40812_at	SMIT	SLC5A3("solute carrier family 5 (inositol transporters), member 3")
263	39457_r_at	SNX4	SNX4("sorting nexin 4")
264	36627_at	SPARCL	SPARCL1("SPARC-like 1 (mast9, hevin)")
265	40680_at	SPIN2	SPIN2("spindlin family, member 2")
266	33596_at	SPOCK3	SPOCK3("sparc/osteonectin, cwcv and kazal-like domains proteoglycan
267	32859_at,	STAT1	STAT1("signal transducer and activator of transcription 1, 91kDa")
268	906_at	STAT4	STAT4("signal transducer and activator of transcription 4")
269	32703_at	STK18	PLK4("polo-like kinase 4 (Drosophila)")
270	33491_at	SUIS	SI("sucrase-isomaltase (alpha-glucosidase)")
271	35832_at	SULF1	SULF1("sulfatase 1")
272	34132_at	SV2C	SV2C("synaptic vesicle glycoprotein 2C")
273	31394_at	Serpin MEPI	SERPINI2("serpin peptidase inhibitor, clade I (pancpin), member 2")
274	34984_at	TRPC3	TRPC3("transient receptor potential cation channel, subfamily C, member 3")
275	1918_at	Ski	SKI("v-ski sarcoma viral oncogene homolog (avian)")
276	1866_g_at	Sno-N	SKIL("SKI-like")
277	39680_at	Statherin	STATH("statherin")
278	36254_at	Substance P	TAC1("tachykinin, precursor 1 (substance K, substance P, neurokinin 1,
279	35077_at	Substance P receptor	TACR1("tachykinin receptor 1")
280	40075_at	Synaptotagmin I [illegible]	SYT1("synaptotagmin I")
281	35405_at	TAT	TAT("tyrosine aminotransferase")
282	38582_at	TATI	SPINK1("serine peptidase inhibitor, Kazal type 1")
283	40560_at	TBX2	TBX2("T-box 2")
284	1596_g_at	TIE2	TEK("TEK tyrosine kinase, endothelial (venous malformations, multiple
285	750_at	TRH receptor	TRHR("thyrotropin-releasing hormone receptor")
286	35929_s_at	TSPY1	TSPY1("testis specific protein, Y-linked 1")
287	33803_at	Thrombomodulin	THBD("thrombomodulin")

288	1098_at	Thrombopoietin	THPO("thrombopoietin (myeloproliferative leukemia virus oncogene ligand,
289	35558_at	UGPA2	UGP2("UDP-glucose pyrophosphorylase 2")
290	33672_f_at	UGT2B17	UGT2B17("UDP glucuronosyltransferase 2 family, polypeptide B17")
291	32948_at	USH2A	USH2A("Usher syndrome 2A (autosomal recessive, mild)")
292	40942_g_at	VAPB	VAPB("VAMP (vesicle-associated membrane protein)-associated protein B and
293	159_at, [illegible]	VEGF-C	VEGFC("vascular endothelial growth factor C")
294	1545_g_at	VEGFR-1	FLT1("fms-related tyrosine kinase 1 (vascular endothelial growth factor/vascular
295	31682_s_at,	Versican	CSPG2("chondroitin sulfate proteoglycan 2 (versican)")
296	37844_at	WSX1	IL27RA("interleukin 27 receptor, alpha")
297	36308_at	ZIC1	ZIC1("Zic family member 1 (odd-paired homolog, Drosophila)")
298	2019_s_at,	alpha-4/beta-7 integrin *	ITGA4("integrin, alpha 4 (antigen CD49D, alpha 4 subunit of VLA-4 receptor)"),
299	39682_at	beta-ENaC	SCNN1B("sodium channel, nonvoltage-gated 1, beta (Liddle syndrome)")
300	1473_s_at, [illegible]	c-Myb	MYB("v-myb myeloblastosis viral oncogene homolog (avian)")
301	35174_i_at	eEF1A2	EEF1A2("eukaryotic translation elongation factor 1 alpha 2")
