# Supplementary material for: Network analysis of human glaucomatous optic nerve head astrocytes
Source: BMC Med Genomics. 2009 May 9;2:24. doi: 10.1186/1755-8794-2-24 (PMC2705386; doi:10.1186/1755-8794-2-24)
Supplement: Additional file 3 — Supplement Figure S1. The shortest paths (SP) network built using up-regulated genes from GO process "complement activation" with mapped experimental data. Supplement Figure S2. The shortest paths (SP) network built from the gene list derived from the up-regulated GO process "nervous system development". Supplement Figure S3. Canonical map for "ephrin signaling" that scored the highest for differential activation during the experimental data mapping and analysis. Supplement Figure S4. Canonical map of VDR signaling with mapped experimental data. Supplement Figure S5. The highest scored map "Bile acids biosynthesis", a part of the process for cholesterol metabolism, with mapped experimental data. Supplement Figure S6. PDGF signaling was the highest scored (lowest p-values) pathway map among down-regulated pathways. Supplement Figure S7. A DI network built from the subset of downregulated genes extracted from the top 12 scored pathway maps. Supplement Figure S8. A DI network for the combined differential gene expression data set in glaucoma. Supplement Figure S9. A DI network for the downregulated genes from the group 1 and group 2 combined data set. Supplement Figure S10. The highest scored AN network for the Androgen receptor (AR) cluster built using the combined data set as an input list. Supplement Figure S11. The DI network for the combined transcriptomic and proteomic data set. Supplement Figure S12. The DI network built using 70 "literature" genes (G-set). Supplement Figure S13. A network illustrating the proximity analysis using the AN algorithm. Supplement Figure S14a. The "disease" DI network for the combined G/DE proximity data set implicated in glaucoma. Supplement Figure S14b. The "disease" DI network with the mapped gene expression data. [file 1755-8794-2-24-S3.pdf]

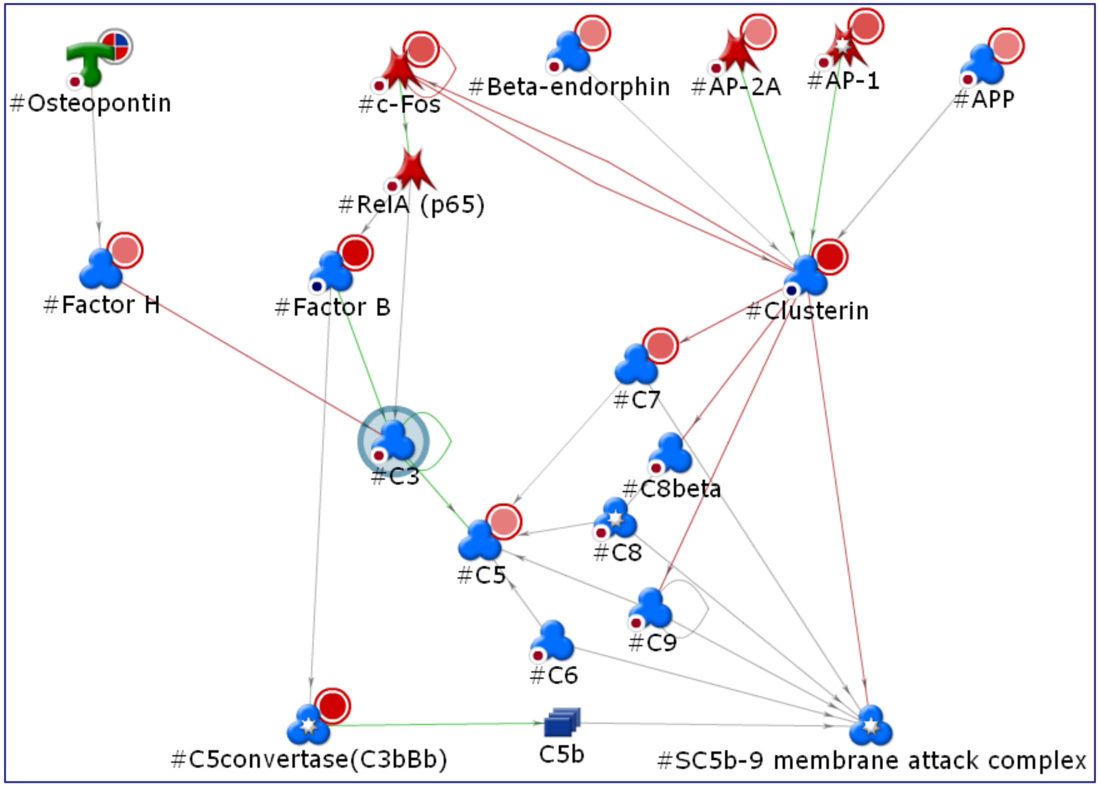

Supplement Figure 1. The shortest paths (SP) network built using up-regulated genes from GO process “complement activation” as the input list. Red frames indicate expression change in the combined file; blue – in the donor Group 1; green – in the donor Group 2. transcriptional activation of Factor H, Factor B, Clusterin and C3 was also confirmed at the level of protein accumulation.

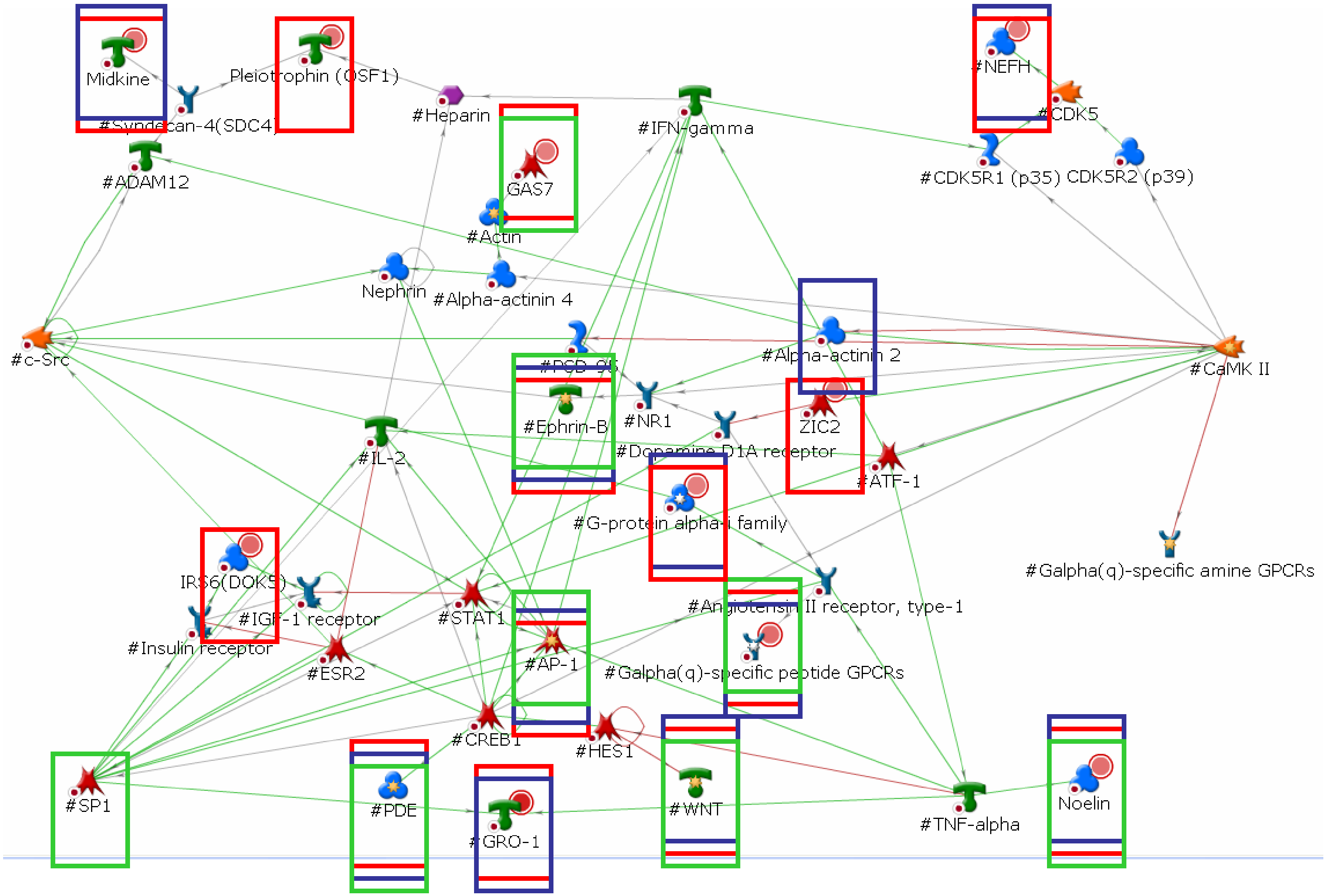

Supplement Figure 2. The shortest paths (SP) network built using the up-regulated genes from the “nervous system development” GO process as the input list. Red frames indicate expression change in the combined file; blue – in the donor Group 1; green – in the donor Group 2.

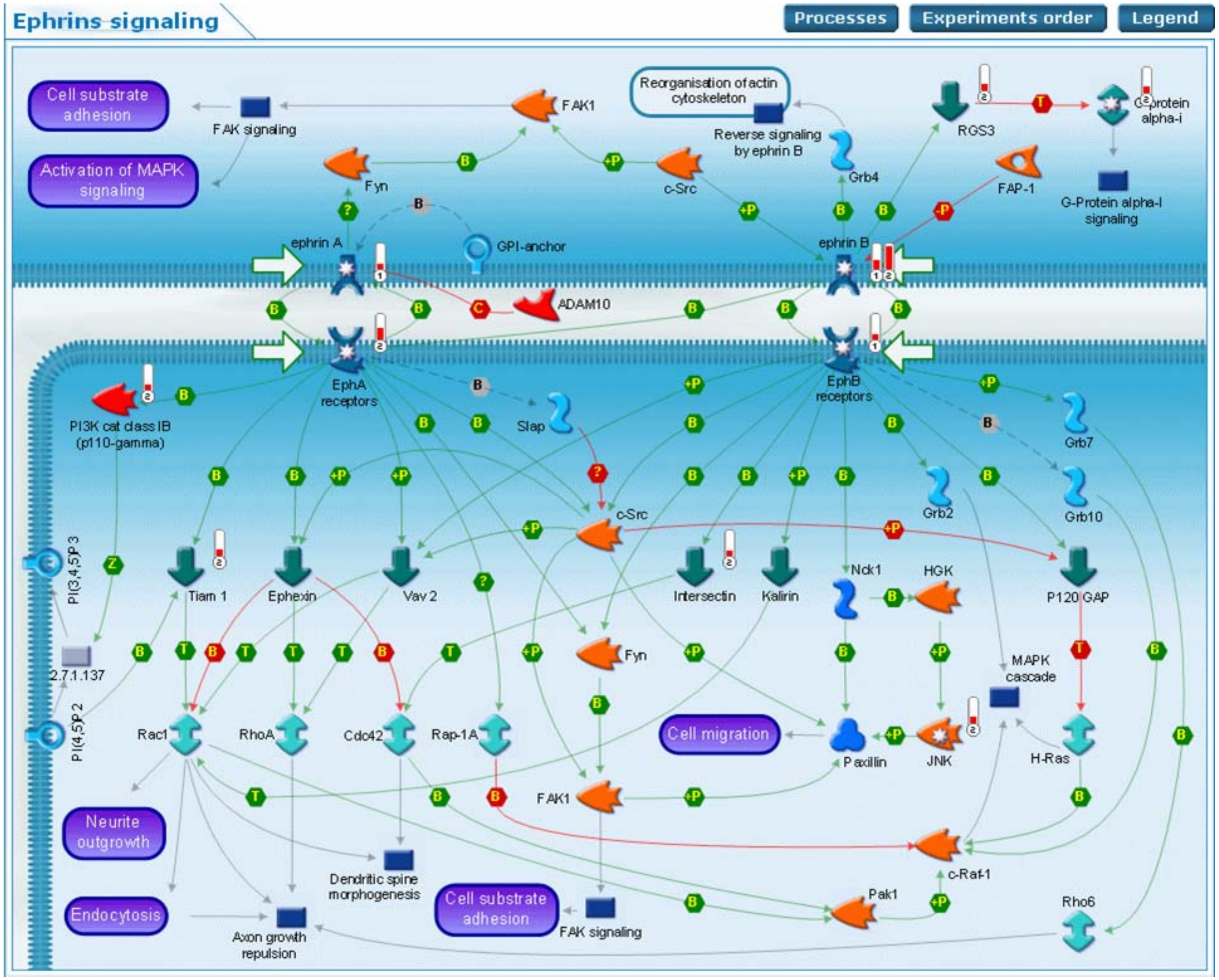

Supplement Figure 3. The highest scored signaling map "ephrin signaling" with the activated gene expression data for donor Group 1 (left indicator) and 2 (right indicator). The blue ribbons represent plasma membrane. The complete legend of symbols for the map objects is shown in Supplement Table II.

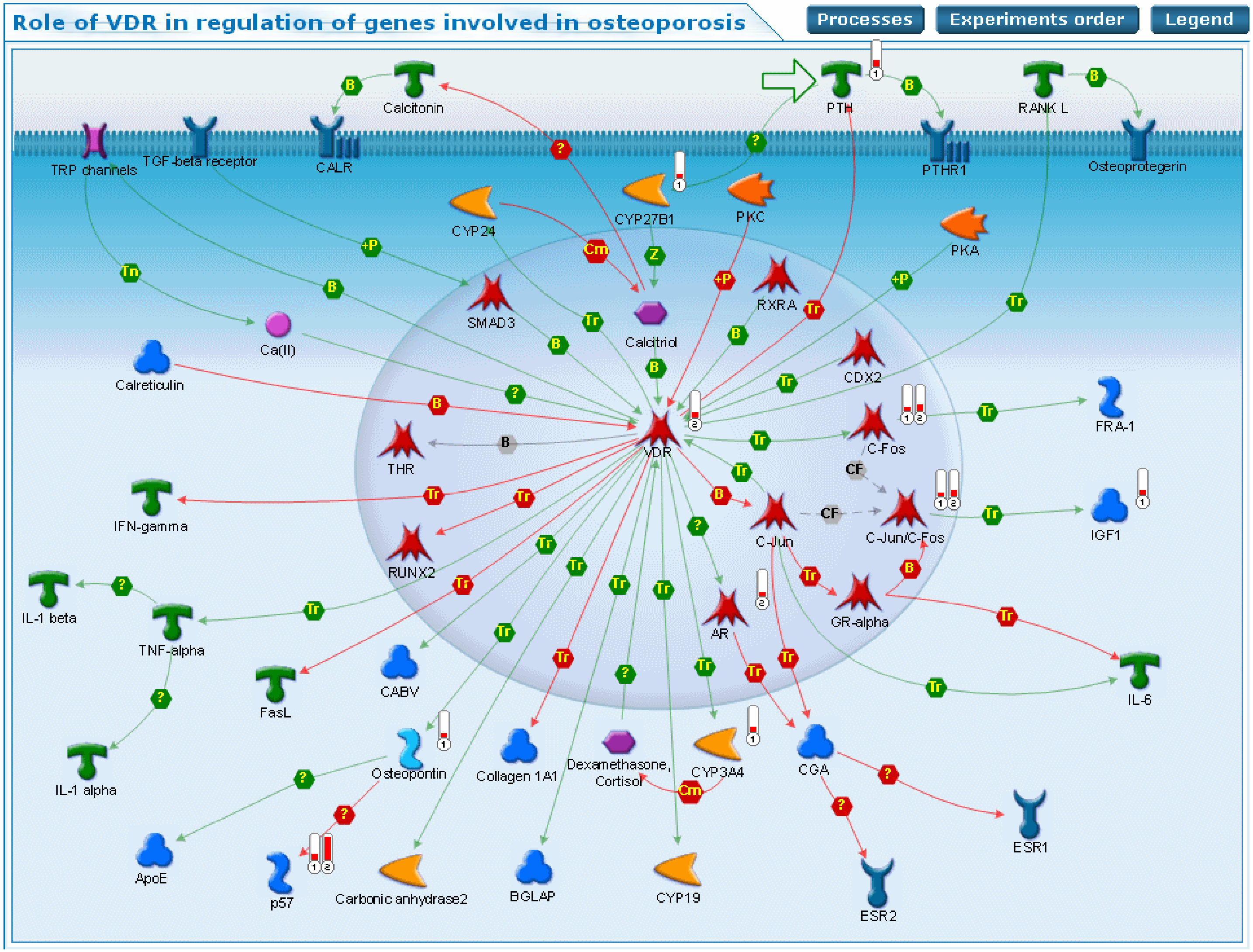

Supplement Figure 4. Activated VDR signaling shown on a standard signaling pathway map from MetaCore. The major participants are c-Fos (up-regulated in both groups), AR (donor group 2), p57 (both donor groups), osteoporin (group 1) and cytochromes CYP27B1 and CYP3A4. Gene expression level indicators: 1, donor Group1; 2, donor Group 2

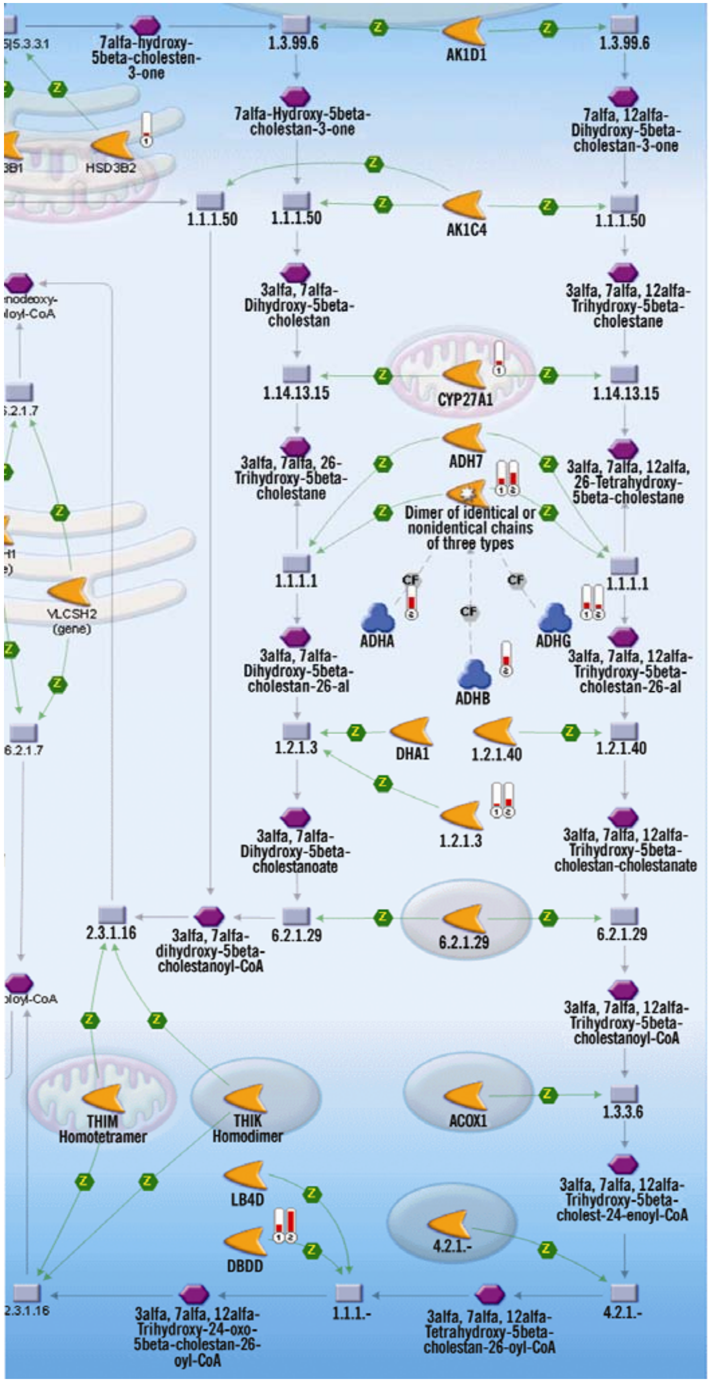

Supplement Figure 5. The fragment of the highest scored metabolic map “Bile acids biosynthesis”, which is a part of cholesterol metabolism. The activated gene expression data shown for donor Group 1 (left indicator) and donor Group 2 (right indicator). The complete legend of symbols for the map objects is shown in Supplementary Table 2.

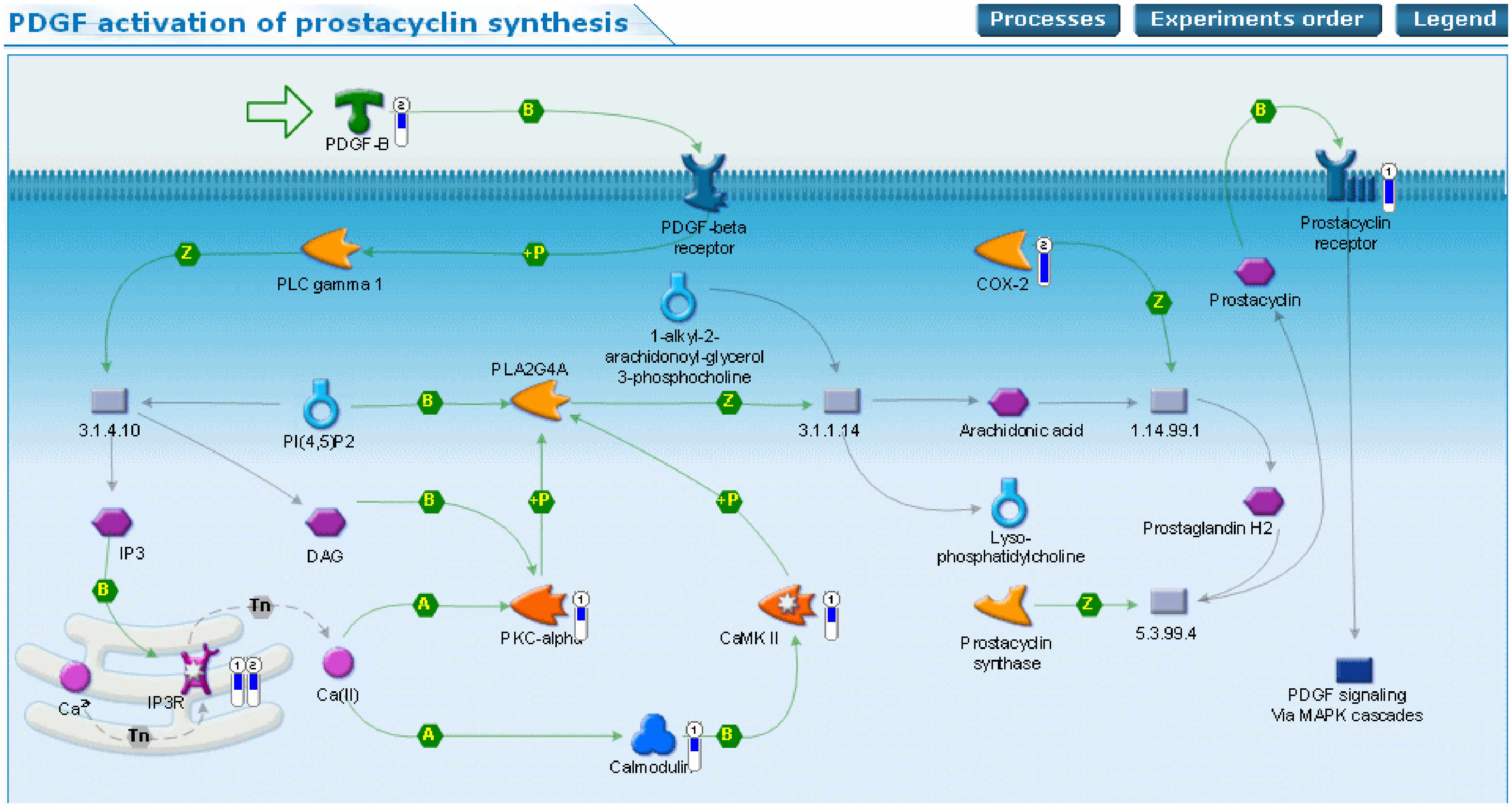

Supplement Figure 6. PDGF signaling was the highest scored (lowest p-values) pathway maps for down-regulated genes. Gene expression level indicators: 1, donor Group1; 2, donor Group 2

A

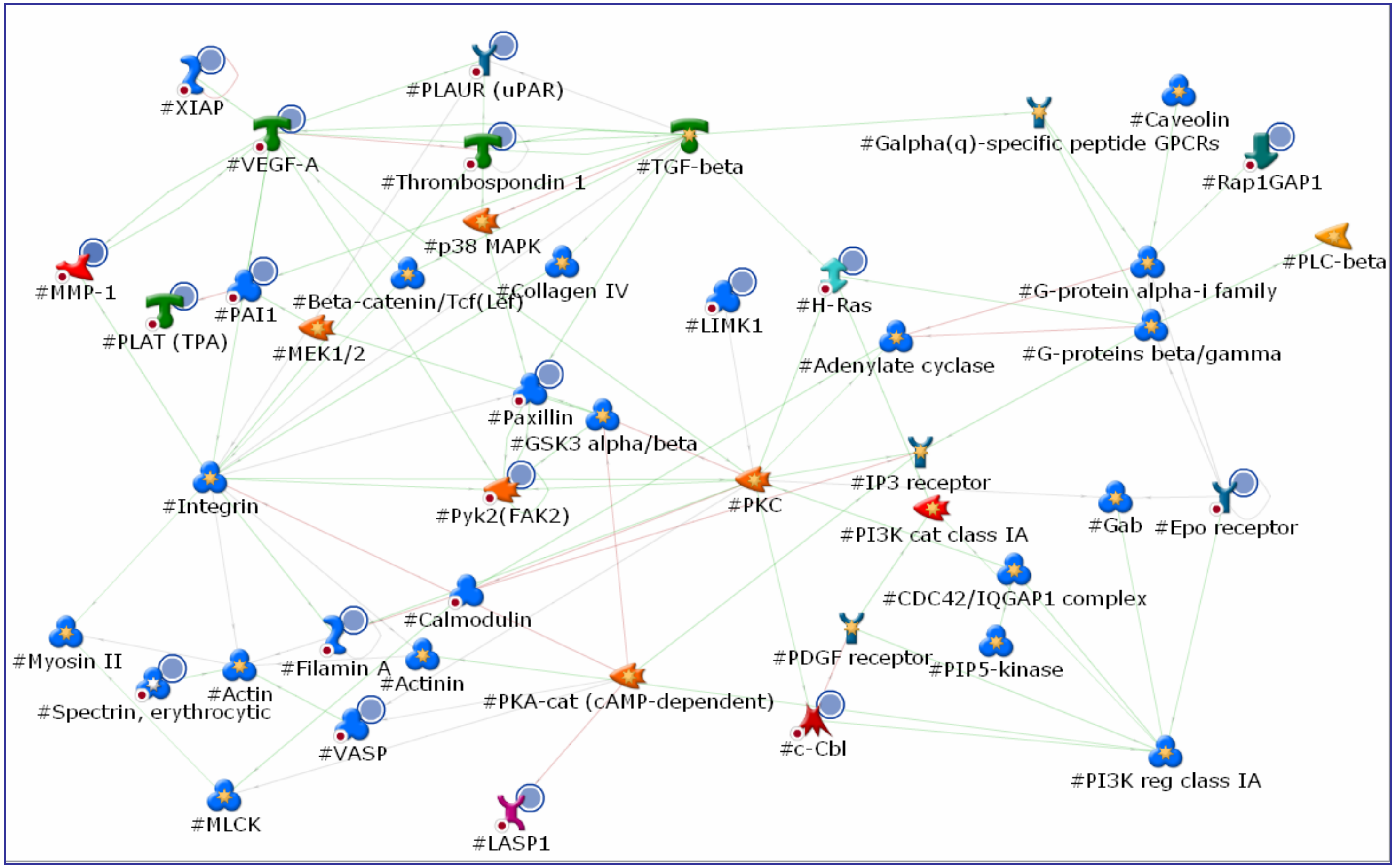

B

1	☐	signal transduction	35.29	5.853e-16
2	☐	cell adhesion	32.77	4.825e-30
3	☐	integrin-mediated signaling pathway	26.89	1.703e-44
4	☐	cell-matrix adhesion	25.21	2.078e-42
5	☐	development	24.37	5.754e-15
6	☐	regulation of progression through cell cycle	21.01	2.237e-17
7	☐	protein amino acid phosphorylation	20.17	1.692e-13
8	☐	cell migration	13.45	2.460e-21
9	☐	blood coagulation	10.92	8.332e-14
10	☐	negative regulation of cell differentiation	10.08	1.552e-18
11	☐	blood vessel development	9.24	4.650e-16
12	☐	cell-substrate junction assembly	6.72	3.792e-14

Supplement Figure 7. A. DI network for the negatively regulated genes retrieved from the top 12 scored pathway maps (fold change > 1.5). The color density of the blue indicator circles reflects the degree of downregulation; the expression data is shown from the donor groups 1 and 2 combined data set . ***B.*** Top 12 GO processes for the gene content of the network.

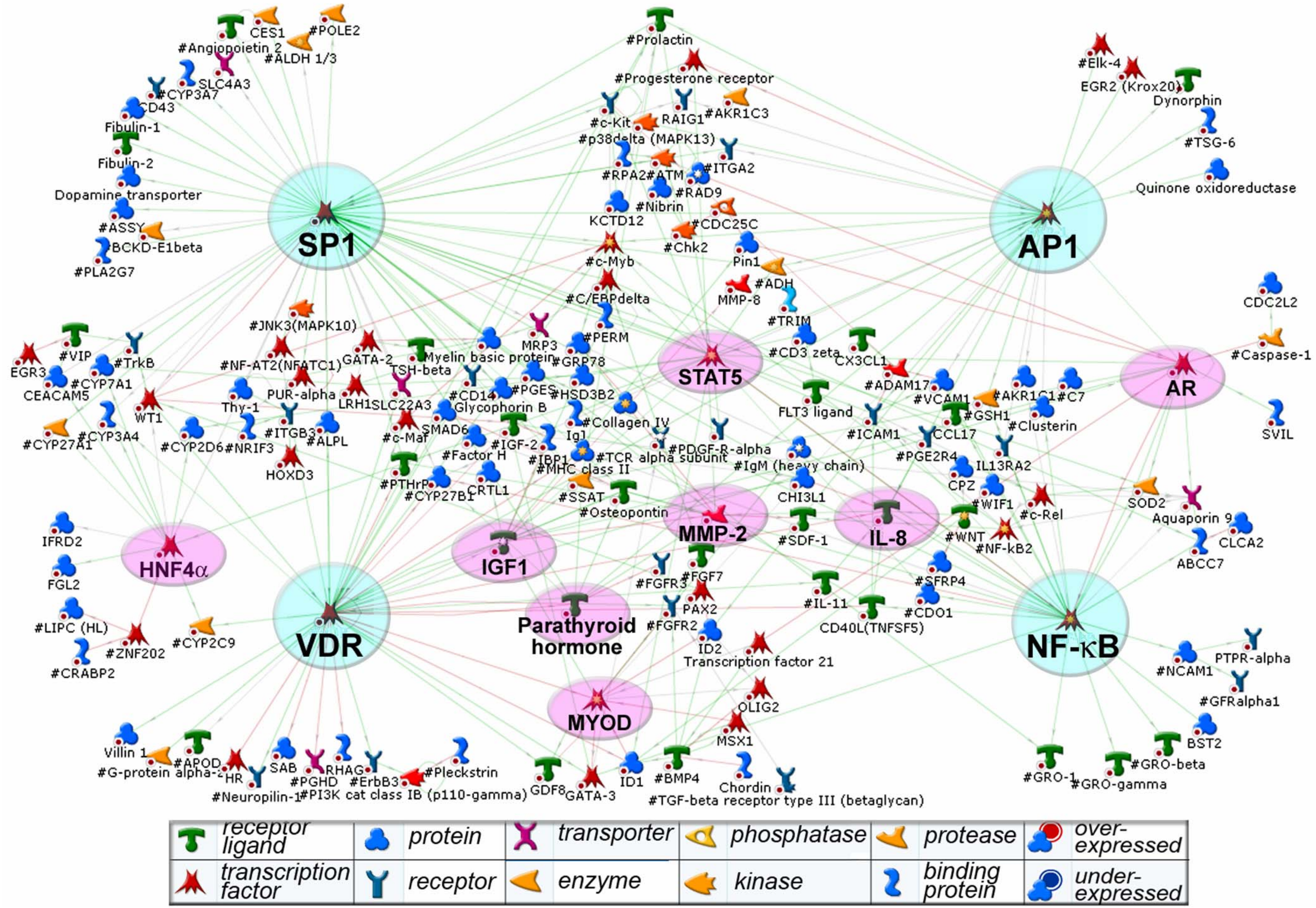

Supplement Figure 8. A DI network for the combined differential gene expression data set in glaucoma. The color density of the red and blue indicator circles reflects the degree of up- and downregulation, correspondingly. Cyan circles, highly connected hubs (>20 edges); pink ovals, the lower order hubs with <20 edges

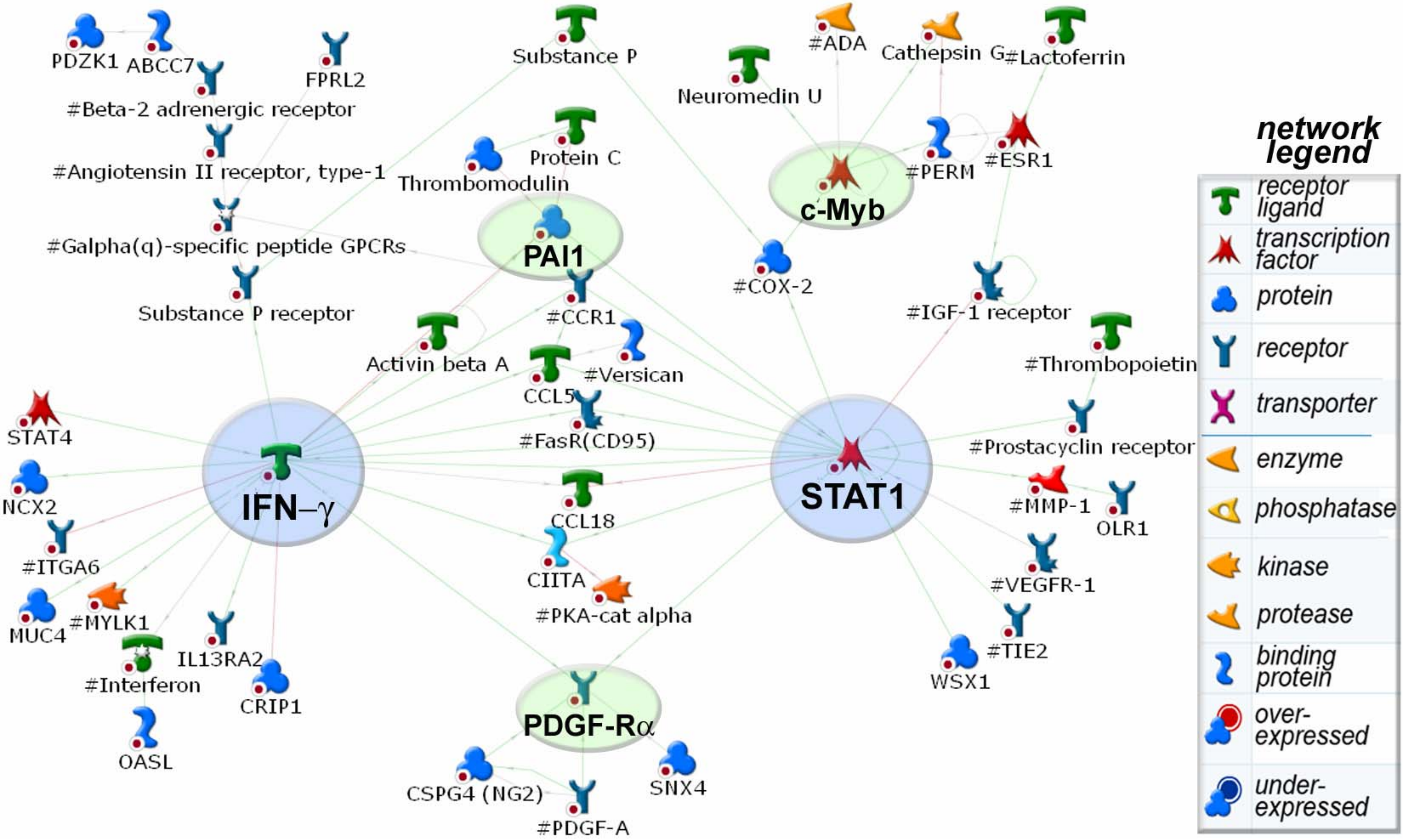

Supplement Figure 9. A direct interactions (DI) network for the downregulated genes from the combined (donor groups 1 and 2) data set . The highly connected hubs are marked with blue circles, four less connected ones with green oval. The full legend of symbols used as network and map objects is shown in Supplement Table II.

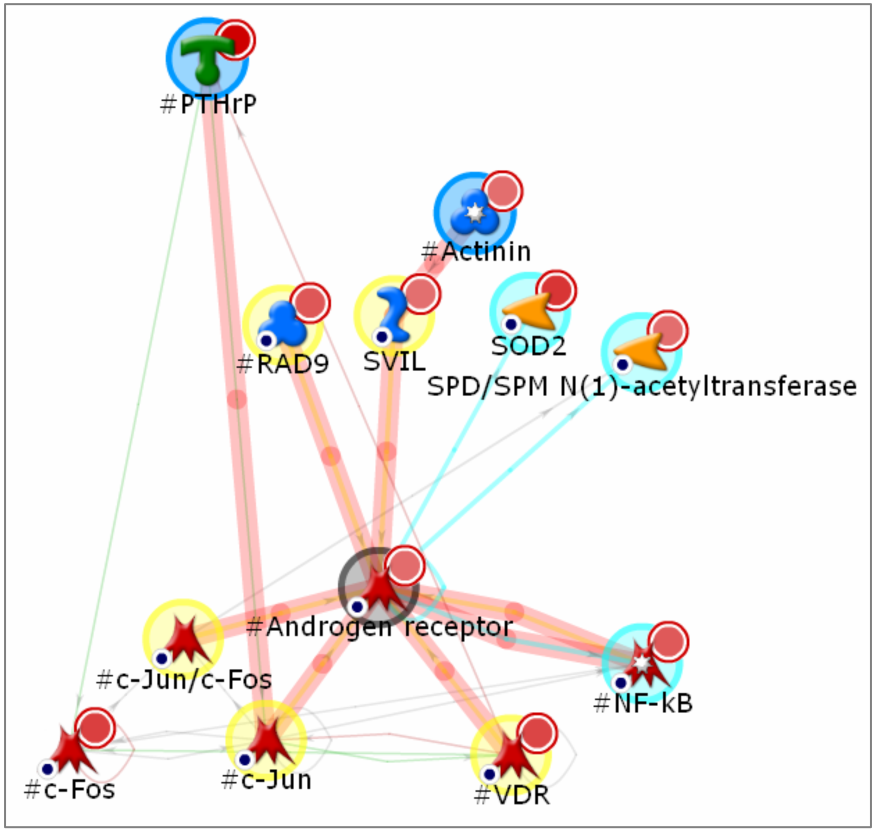

Supplement Figure 10. The highest scored AN network for the Androgen receptor (AR) cluster built using the combined data set as an input list. The color density of red and blue indicator circles reflects the degree of up- and downregulation, correspondingly. The links belonging to canonic AR activation pathways of are highlighted with red

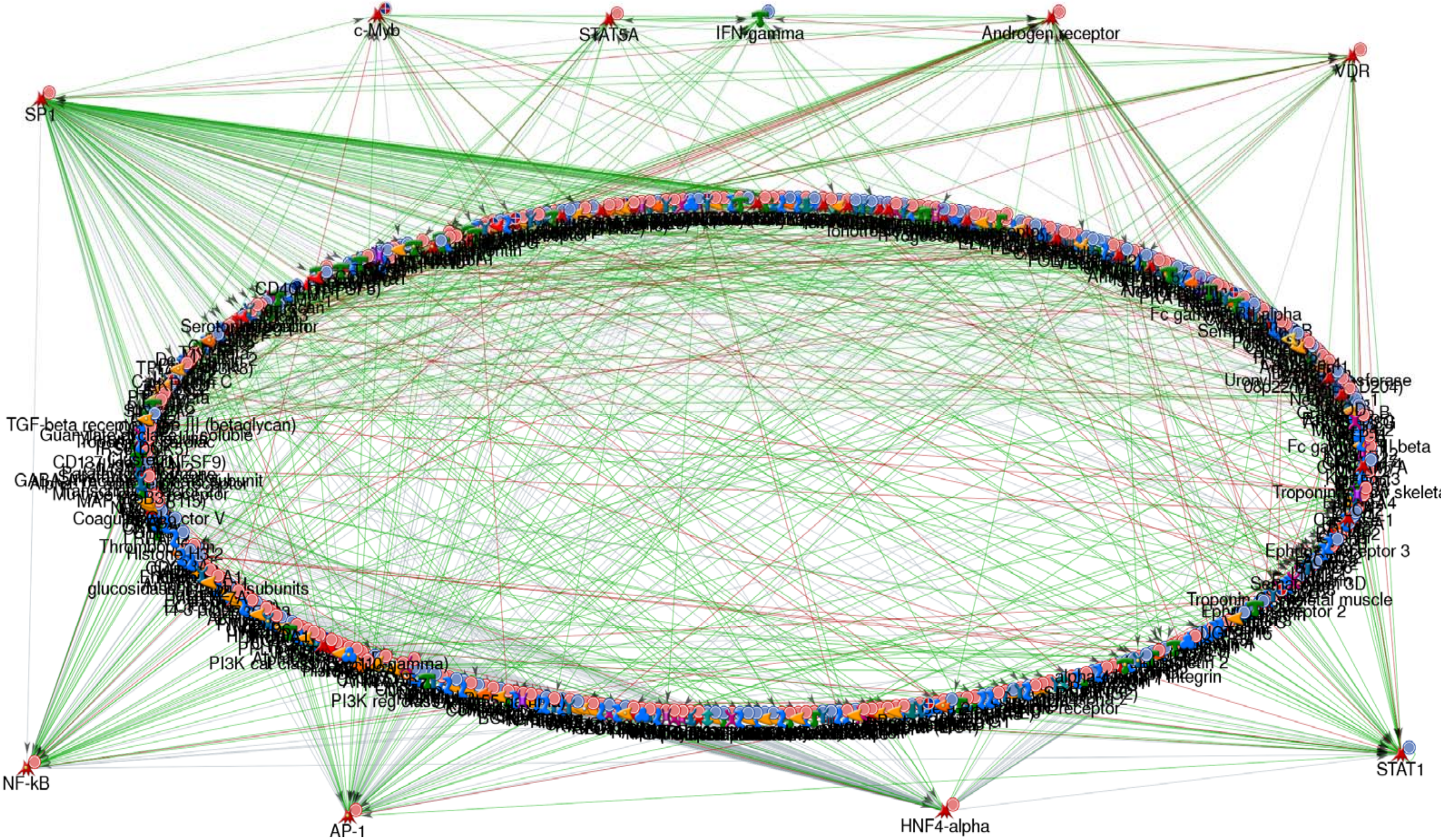

Supplement Figure 11. The DI network for the combined transcriptomic and proteomic data set interconnected 488 nodes that changed (>2.5 fold, both up- and down-regulated) in glaucomatous ONHAs. Top 12 major hubs are shown at the periphery. Subunits of NF-kB (c-Rel and NF-kB1) and AP-1 (c-Fos, c-Jun, JunD) are grouped together.

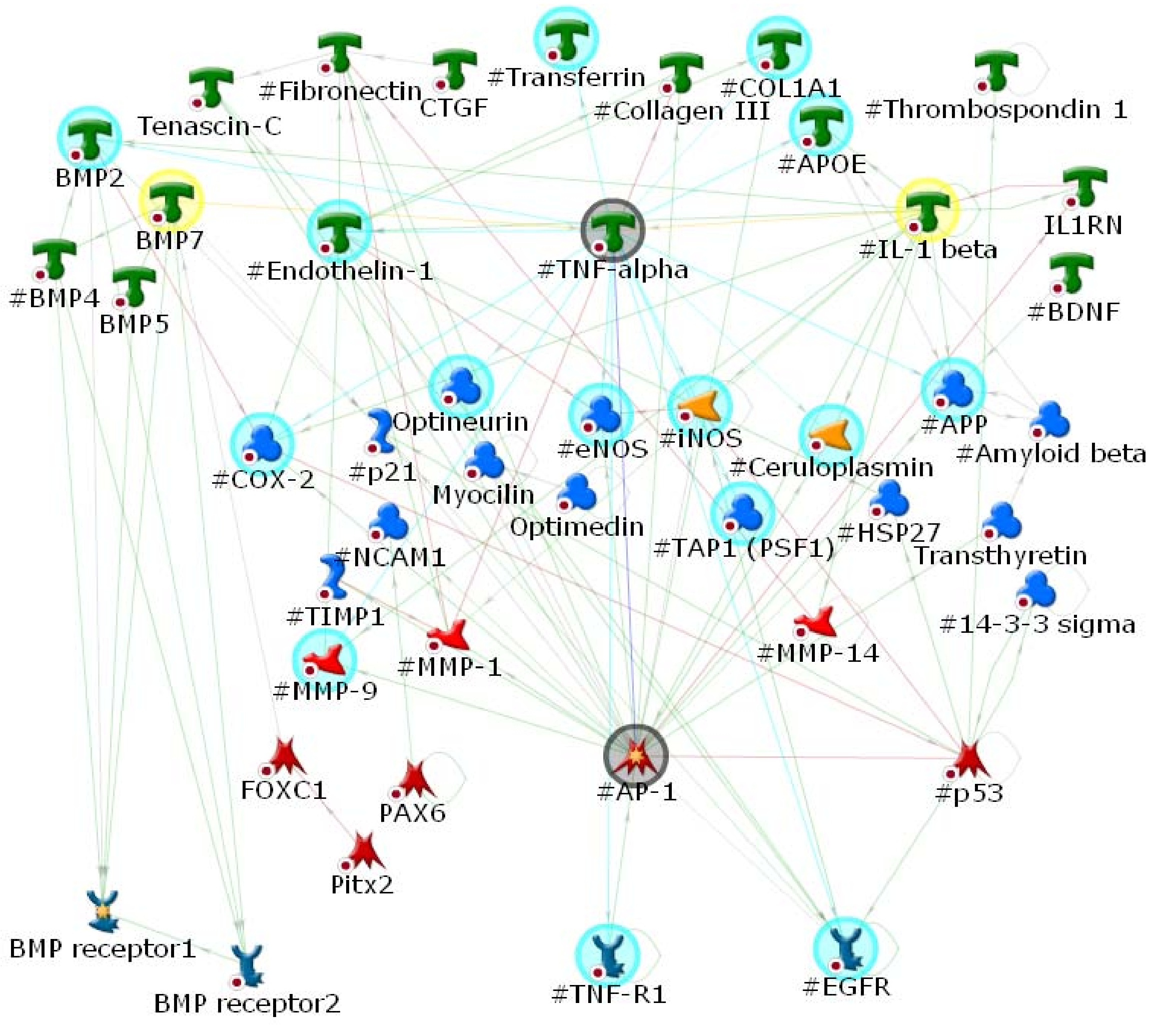

Supplement Figure 12. The DI network built using 70 “literature” genes (G-set) as the input file has TNF-alpha, Endothelin-1, IL-1 as the top interconnected hubs and AP-1 as the major transcription factor. pstream (yellow) and downstream (cyan) interactions are highlighted for TNF-alpha only.

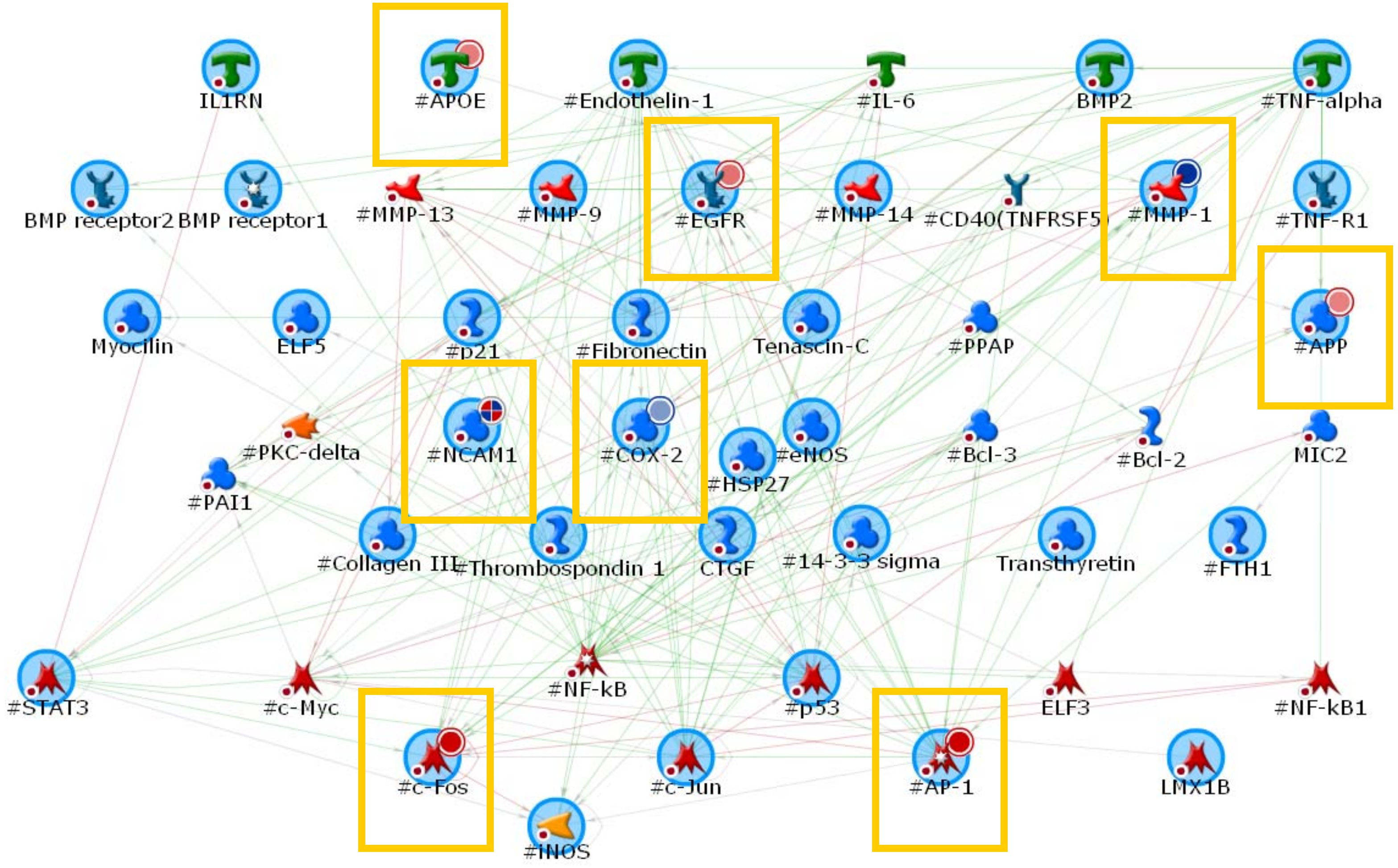

Supplement Figure 13. Proximity analysis using the AN algorithm revealed the DE (framed nodes with red and blue indicator circles) genes among close neighbors of the genes from the G-set (labeled with light blue circles)

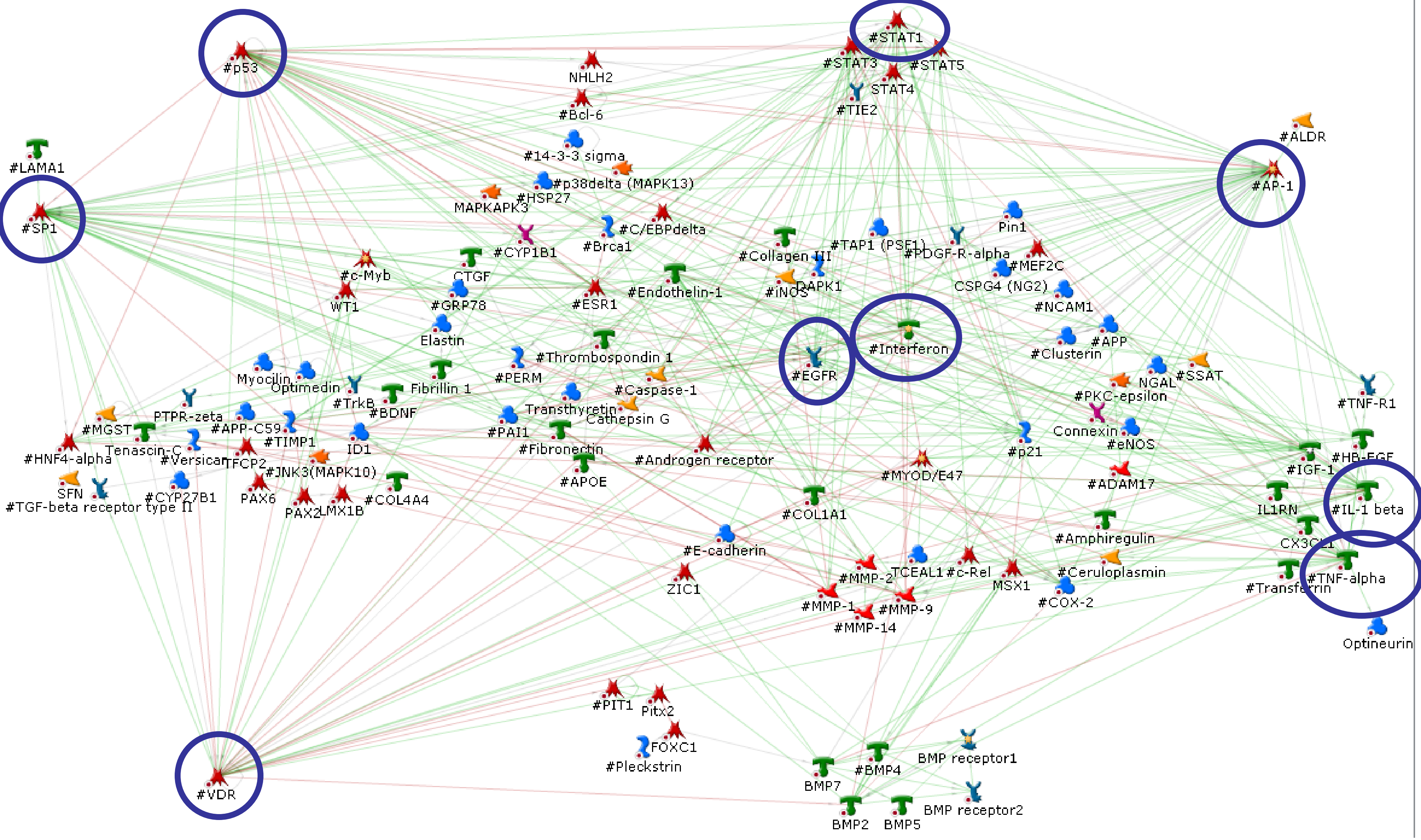

Supplement Figure 14a. The "disease" DI network for the combined G/DE proximity data set connected 102 out of 119 edges implicated in glaucoma. The major hubs are highlighted by blue circles. Experimental data is omitted for better visualization of the network topology. The gene expression data is shown on Supplement Figure 14b.

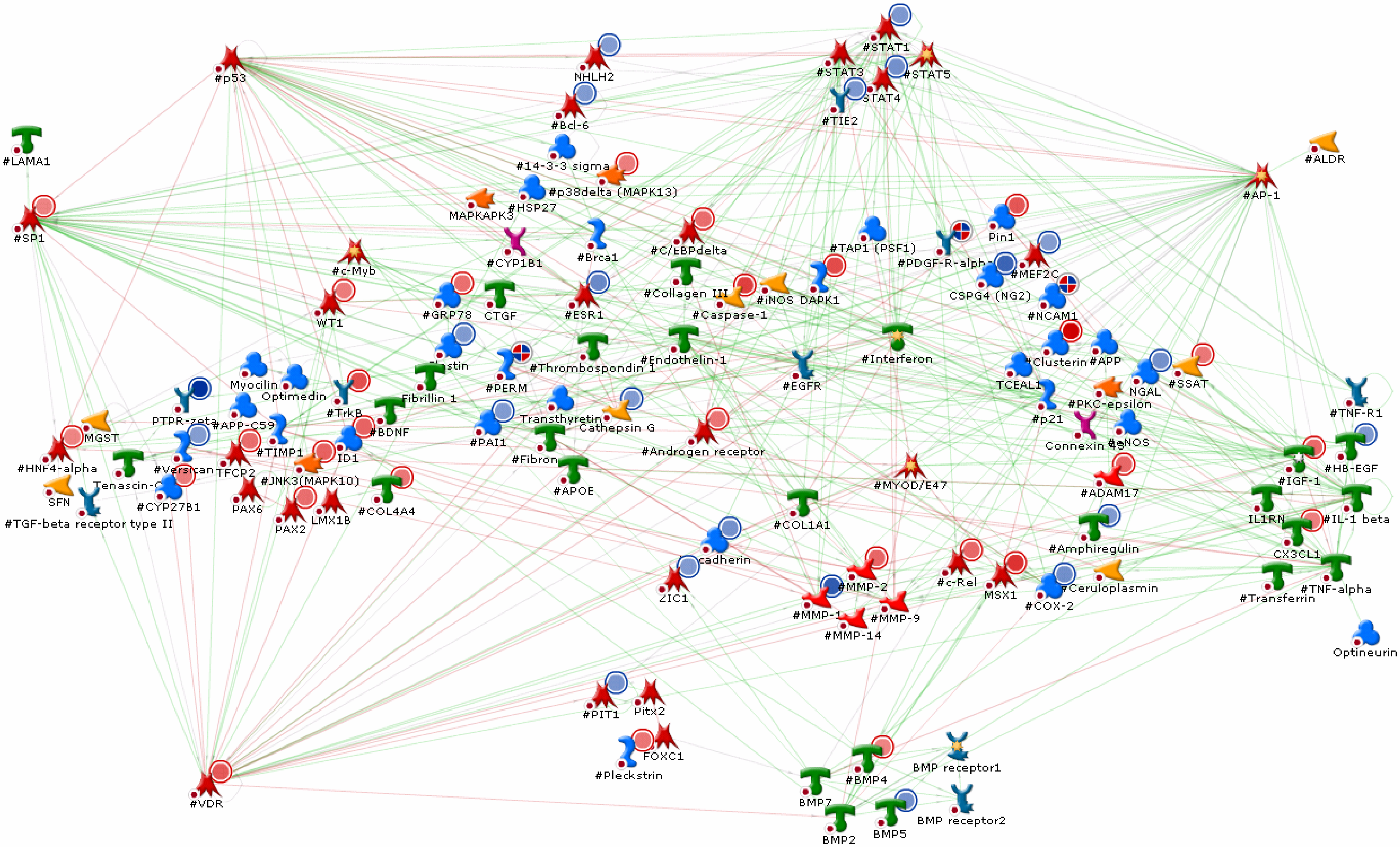

Supplement Figure 14b. The "disease" DI network connecting 102 nodes from the combined G/DE proximity data set implicated in glaucoma with experimental and literature-derived data mapped. Red and blue circles indicate up- and downregulated genes correspondingly. The genes from the "G-set" are not marked for expression.
